# Comparison of gene expression profiles among caste differentiations in the termite *Reticulitermes speratus*

**DOI:** 10.1038/s41598-022-15984-z

**Published:** 2022-07-13

**Authors:** Ryota Saiki, Yoshinobu Hayashi, Kouhei Toga, Hajime Yaguchi, Yudai Masuoka, Ryutaro Suzuki, Kokuto Fujiwara, Shuji Shigenobu, Kiyoto Maekawa

**Affiliations:** 1 Graduate School of Science and Engineering, University of Toyama, Toyama, 930-8555 Japan; 2 Laboratory of Ecological Genetics, Graduate School of Environmental Science, Hokkaido University, Sapporo, 060-0810 Japan; 3 NIBB Core Research Facilities, National Institute for Basic Biology, Okazaki, 444-8585 Japan; 4 Ishikawa Insect Museum, Hakusan, Ishikawa 920-2113 Japan; 5 Department of Biology, Keio University, Yokohama, Kanagawa 223-8521 Japan; 6 Department of Biosciences, College of Humanities and Sciences, Nihon University, Tokyo, 156-8550 Japan; 7 Department of Bioscience, School of Science and Technology, Kwansei Gakuin University, Sanda, Hyogo 669-1337 Japan; 8 Institute of Agrobiological Sciences, National Agriculture and Food Research Organization, Tsukuba, 305-8634 Japan; 9 Faculty of Science, Academic Assembly, University of Toyama, 3190 Gofuku, Toyama, 930-8555 Japan

**Keywords:** Social evolution, Evolutionary biology

## Abstract

Termite castes express specialized phenotypes for their own tasks and are a good example of insect polyphenism. To understand the comprehensive gene expression profiles during caste differentiation, RNA-seq analysis based on the genome data was performed during the worker, presoldier, and nymphoid molts in *Reticulitermes speratus*. In this species, artificial induction methods for each molt have already been established, and the time scale has been clarified. Three different periods (before the gut purge (GP), during the GP, and after the molt) were discriminated in each molt, and two body parts (head and other body regions) were separately sampled. The results revealed that many differentially expressed genes (head: 2884, body: 2579) were identified in each molt. Based on the independent real-time quantitative PCR analysis, we confirmed the different expression patterns of seven out of eight genes in the presoldier molt. Based on the GO and KEGG enrichment analyses, the expressions of genes related to juvenile hormone titer changes (e.g., *JH acid methyltransferase*), nutrition status (e.g., *Acyl-CoA Delta desaturase*), and cell proliferation (e.g., *insulin receptor*), were shown to specifically fluctuate in each molt. These differences may have a crucial impact on caste differentiation. These data are important resources for future termite sociogenomics.

## Introduction

Termites, ants and bees constitute the major social insect groups. The division of labor, including reproduction, maintains the social insect colony and increases overall colony productivity^1^. Castes are morphologically and behaviorally specialized for specific tasks, and they are a good example of insect polyphenism in response to various environmental factors^2,3^. Termite castes are classified as workers, soldiers, or reproductives^4–6^. Workers feed and care for the brood and have highly developed digestive organs whereas soldiers are differentiated from workers by an intermediate stage (presoldier) and are specialized for colony defense with sclerotized heads and weapons (e.g., enlarged mandibles). Primary reproductives, who establish new colonies, and neotenic reproductives, who take over reproduction in their natal nests, make up the reproductive caste^6^. Both have functional gonads for reproduction. Caste differentiation to form different phenotypes is accomplished through the expression of different gene sets in response to environmental stimuli^7^.

The candidate gene approach has primarily been used in molecular analyses of termite caste differentiation^8,9^. These analyses were particularly focused on soldier differentiation because, in many species, juvenile hormone (or JH analog) treatments could be used to induce soldiers from workers^10,11^. Some important genes, such as *hexamerin*^8^, *insulin receptor*^12^, *distal-less*^9^ and *deformed*^13,14^ have been found to be specifically expressed at a certain point during the molting period (i.e., worker-presoldier molt). Furthermore, some specifically expressed genes and cascades have been identified during the differentiation of the first soldier in an incipient colony^15–17^, based on the genome sequences of the primitive termite *Zootermopsis nevadensis*^18^. However, even in *Z. nevadensis,* there are no artificial induction methods of caste differentiation other than those for soldiers, therefore, comprehensive gene expression patterns and comparative transcriptome profiles among caste differentiations are not fully understood.

The genome sequence of the most common subterranean termite in Japan, *Reticulitermes speratus*, has been identified (gene model OGS1.0)^19^. This species has a bifurcated developmental pathway, where larvae molt into workers (apterous line) or nymphs (imaginal line) at the early developmental stage^20^. Soldiers are differentiated from 2 to 5th stage workers (W2–W5), and nymphoids are differentiated from 3 to 6th stage nymphs (N3–N6)^20–22^. Importantly, in this species, artificial induction methods for the worker-worker molt^23^, the worker-presoldier molt^24,25^ and the nymph-nymphoid molt^26,27^ have been established. Moreover, the time scale of each molt was clarified by observing the initiation of the gut purge (GP), and all molts essentially took the same period of time for completion (approximately 11 days after the treatment)^23,26^. As a result, this species has several advantages in terms of comparing gene expression profiles across all caste differentiations.

RNA-seq analysis was used to identify the gene expression profiles in *R. speratus* at several time periods of the molting process. The process was divided into four periods in each molt, and 72 cDNA libraries were constructed [3 molts (worker-worker, worker-presoldier, nymph-nymphoid) × 4 periods (worker or nymph, pre-GP, GP and molt individuals) × 2 body parts (head and other body parts) × 3 colonies]. All libraries were sequenced using an Illumina HiSeq2500. After mapping to the genome sequence and acquiring the gene expression data, differentially expressed genes (DEGs) among the four periods in each molt were identified. To verify the DEGs list obtained, we performed real-time quantitative PCR analysis of some genes highly expressed in the worker-presoldier molt. Gene Ontology (GO) and Kyoto Encyclopedia of Genes and Genomes (KEGG) enrichment analyses were carried out to determine the caste-specific gene expression profiles during each molting process. The roles of DEGs for each molt are discussed using information from previously described homologs.

## Materials and methods

### Termites used for RNA-seq analysis

Termites were collected from five colonies (#A, B, C, D, and E) for transcriptome sequencing. All colonies were collected in Furudo, Toyama Prefecture, Japan, between June and September 2013. All colonies were maintained in plastic cases at 25 °C in constant darkness until the induction of the worker-worker, worker-presoldier, and nymph-nymphoid molts (see below). The 5th stage nymphs (N5 nymphs) and the 4th-5th stage workers (old-age workers) were collected from the colonies based on the shape of wing buds, body size, and antennal segments. Nymphs were distinct from workers by the possession of wing puds, and N5 nymphs and old-age workers had 16 and 15–17 antennal segments, respectively^20,28^.

### Induction of worker-worker molt by 20E application

**Figure 1:**
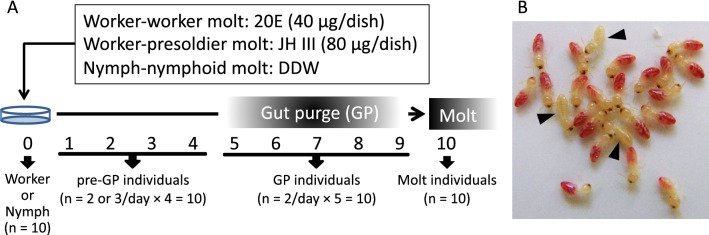
(**A**) Artificial induction method of each molt and time schedule for sampling of individuals examined. Each molt required a similar period of time. Ten individuals were collected from 4 different periods (worker or nymph, pre-GP, GP and molt stages) in each molt. To collect individuals before the gut purge (pre-GP), 2 or 3 individuals were sampled daily and mixed during the same period. Gut-purged individuals (GP-0, 1, 2, 3 and 4) were equally mixed for the GP individuals. Molt individuals were sampled within 24 h after each molt. (**B**) Examples of gut-purged workers (GP workers; arrowheads) with yellowish-white abdomens. Non-GP workers were used for the artificial induction treatment described in (**A**).

To obtain individuals at multiple times during the worker-worker molt, we used the 20-hydroxyecdysone (20E) application method, as described previously^23^ (Fig. 1A). Old-age workers were collected from each of three different colonies (#A, B, and C) and kept overnight amidst moist red-colored thin papers (Goshikizuru No.14, Goukaseishi Co., Ltd., Aichi, Japan). Gut-purged workers (GP workers) were identified as having yellowish-white abdomens (Fig. 1B). Non-GP workers were used for the following analyses. Red colored thin paper was treated with 40 µg 20E dissolved in 400 µL acetone and placed in a 65 mm Petri dish with 20 non-GP workers. Ten Petri dishes (containing 200 workers) from each colony were used to collect individuals for RNA extraction. 20E and control-acetone-treated Petri dishes were also used to measure the induction rate (n = 1–3 dishes, 20 individuals per dish). In addition, to obtain natural GP workers, 200 workers were kept in a 90 mm Petri dish with no 20E or acetone treatments. Because of low frequency of natural molting events and limitation of the space in the incubator, we used a 90 mm Petri dish for this analysis. All Petri dishes were kept in an incubator at 25 °C. Over a 2-week period, all dishes were checked every 24 h for dead individuals. According to previous research^23^, GP workers appeared approximately 5 days after 20E treatments. During the worker-worker molt, four main periods were identified: (1) before the 20E treatment (worker), (2) just before the gut purge (pre-GP worker), (3) during the gut purge (GP worker), and (4) just after the molt (molt worker). For the pre-GP workers, we randomly collected 10 individuals from multiple dishes each day following the 20E treatment (total 4 days). GP workers, including natural GP workers, that emerged on the same day were transferred to a new 40 mm dish (up to 10 individuals). These workers (referred to as GP-0 workers) were kept in an incubator at 25 °C. Because the period for gut purging was approximately 5 days^23^, GP-0, -1, -2, -3, and -4 workers (i.e., GP workers 0, 1, 2, 3 and 4 days after the emergence, respectively) were collected, respectively (n = 10 per day). For the molt workers, individuals were collected within 24 h of the worker molt. For RNA extraction, 10 individuals were used per period. For the pre-GP workers, 2, 3, 3, and 2 individuals were randomly selected at 1–4 days after 20E treatment, respectively, and mixed for the same period. For the GP workers, GP-0, -1, -2, -3, and -4 workers were equally mixed (Fig. 1A). Each individual was dissected on ice, with the head and other parts of the body (including thorax and abdomen with guts; hereafter body) separated, immediately frozen in liquid nitrogen, and stored at -80 °C until RNA extraction.

### Induction of presoldier differentiation by JH III application

Induction of presoldier differentiation was performed as previously described^23–25^. Old-age workers collected from each of the three different colonies (#A, B, C) were kept overnight with wetted red-colored thin paper. Non-GP workers were used for the following analyses (Fig. 1). Red colored thin paper was treated with 80 µg JH III (Santa Cruz Biotechnology Inc., California, USA) dissolved in 400 µL acetone and placed in a 65 mm Petri dish with 20 non-GP workers. Ten Petri dishes (containing 200 workers) from each colony were used to collect individuals for RNA extraction. We also prepared JH and control-acetone-treated dishes to measure the induction rate (n = 1–3 dishes, 20 individuals per dish). All Petri dishes were kept in an incubator at 25 °C. Over a 2-week period, all dishes were checked every 24 h for dead individuals. As the method described for worker-worker molt (“Induction of worker-worker molt by 20E application” section), pre-GP workers (individuals collected at 1–4 days after JH treatments), GP workers (GP-0, -1, -2, -3, and -4 workers), and molt presoldiers (individuals collected within 24 h of the presoldier molt) were sampled. For RNA extraction, 10 individuals were used of each period. Each sample was dissected to separate the head and body on ice, immediately frozen in liquid nitrogen, and stored at -80 °C until RNA extraction.

### Induction of nymphoid differentiation by isolation from the natal nest

Nymphoid differentiation was induced by isolation from the natal nest, according to the method of Saiki and Maekawa (2011)^26^. The 5th stage nymphs (N5 nymphs) were collected from two different colonies (#D, E) and kept overnight with wetted red-colored thin paper. Non-GP nymphs were used for subsequent analyses (Fig. 1). Red thin paper was placed in a 65 mm Petri dish with 20 non-GP nymphs prepared from colony #D. We prepared > 11 dishes (> 220 nymphs) to collect individuals for RNA extraction. Since a large number of nymphs (> 600) were collected from colony #E, red-colored paper (90 mm) was placed in a 90 mm Petri dish with approximately 200 non-GP nymphs from this colony. We prepared three dishes (> 600 nymphs) to collect individuals for RNA extraction. Because of the sampling difficulties and large number of nymphs in colony #E, we doubled the number of Petri dishes for biological replications using nymphs from colony #E. All Petri dishes were kept in an incubator at 25 °C. Over a 2-week period, all dishes were checked every 24 h for dead individuals. As the method described for worker-worker molt (“Induction of worker-worker molt by 20E application” section), the N5 nymphs, the pre-GP nymphs (individuals collected at 1–4 days after isolation), GP nymphs (GP-0, -1, -2, -3, and -4 nymphs), and molt nymphoids (individuals collected within 24 h of the nymphoid molt) were used. For RNA extraction, 10 individuals were used in each period. Each sample was dissected on ice to separate the head and body, immediately frozen in liquid nitrogen, and stored at 80 °C until RNA extraction.

### Total RNA extraction

We prepared 72 categories: 3 molts (worker-worker, worker-presoldier, nymph-nymphoid) × 4 periods (workers or nymphs, pre-GP, GP, and molt individuals), × two body parts (head and body), × three colonies (in the case of nymphoid differentiation, triplicates were prepared from two colonies; see “Induction of nymphoid differentiation by isolation from the natal nest” section) (10 individuals were used per category). Total RNA was isolated from each category using the SV Total RNA Isolation System (Promega, Madison, WI, USA). DNA was digested with RNase-free DNase I for 20 min at 37 °C. The quantity and quality of the extracted RNA were checked using a NanoVue spectrophotometer (GE Healthcare Bio-Sciences, Tokyo, Japan), Qubit 2.0, fluorometer (Life Technologies, Darmstadt, Germany), and Agilent 2100 bioanalyzer (Agilent Technologies, Palo Alto, CA).

### cDNA synthesis for HiSeq 2500 and RNA-seq analysis

cDNA libraries for RNA-seq were prepared using the Truseq Stranded mRNA LT kit (Illumina, San Diego, CA, USA) according to the manufacturer's instructions. First- and second-strand cDNA synthesis, adaptor ligation, and amplification were carried out. After cDNA preparation, the quantity and quality of cDNA were checked using the KAPA qPCR SYBR green PCR kit (Geneworks, Thebarton, Australia) and Agilent 2100 bioanalyzer (Agilent Technologies, Palo Alto, CA). Twelve cDNA libraries were pooled in equal quantities, and six tubes from 72 libraries were prepared. All libraries were subjected to single-end sequencing of 150 bp fragments on Hiseq2500 (Illumina, San Diego, CA). Sequence read quality was determined using FastQC^29^. The adaptor sequences, low-quality sequences (< 20 quality scores), and too short reads (< 50 bp) were removed from the sequence data using Trimmomatic v0.35^30^. Trimmed sequence data were mapped to the *R. speratus* genome data (gene prediction model RspeOGS1.0^19^) using Tophat v2.1.0 with Bowtie2 v2.2.3^31^. Counting reads were performed using featureCounts^32^.

### DEG analysis

Gene expression levels were compared using the generalized linear model (GLM) approach implemented in the edgeR Bioconductor package^33^. For the DEG analysis, we kept only genes with at least one count per million (CPM) in at least three samples. Then, the DEG analysis was carried out using the read count data normalized with the trimmed mean of M-values (TMM) method^34^. We compared gene expression levels (head or body) during each molting process at four different periods (worker/nymph, pre-GP, GP, and molt individuals). We selected DEGs with an FDR cut-off of 0.01 and log2 fold change cut-off of 1. To test the effect of artificial 20E treatment, the number of DEGs was compared between 20E-induced GP workers and natural GP workers using edgeR.

### Real-time quantitative PCR (qPCR) analysis

To validate the DEGs list obtained in “DEG analysis” section, we focused on the genes that were highly expressed during the worker-presoldier molt (total eight; Fig. S1 and Table S1). We especially focused on those genes; some of which could be involved in the soldier-specific morphogenesis, mainly occurred in the head parts during the worker-presoldier molt^11^. Independent real-time qPCR analysis was used to compare gene expression levels in heads (pre-GP period) between worker-presoldier and worker-worker molts. In October 2020, three new colonies were found in Himi and Yatsuo, Toyama Prefecture. Following the methods described above, each colony received both 20E and JH treatments, and workers were collected at three time points: early (day 1–2), middle (day 2–3) and late (day 3–4). Four heads were placed into one tube, and four tubes were prepared for each category. RNA extraction was carried out using the ReliaPrep RNA Tissue Miniprep System (Promega, Madison, USA). RNA and DNA were quantified using a Qubit fluorometer, and RNA purity and quantity were measured using a NanoVue spectrophotometer. DNase-treated RNA was used for cDNA synthesis using the High-Capacity cDNA Reverse Transcription Kit (Thermo Fisher). Quantitative real-time PCR was performed using a Thunderbird SYBR qPCR Mix (TOYOBO, Japan) and a QuantStudio 3 Real-Time PCR System (Thermo Fisher). To determine an internal control gene, the suitability of six reference genes, *EF1-alfa* (accession no. AB602838)^35^, *NADH-dh* (AB602837)^35^, *beta-actin* (AB520714)^36^, *glutathione-S-transferase 1* (*GstD1*, gene ID: RS001168^19^), *eukaryotic initiation factor 1A* (*EIF-1,* RS005199^19^), and *ribosomal protein S18* (*RPS18*, RS015150^19^) was evaluated using GeNorm^37^ and NormFinder^38^ software. Specific primers were designed against each gene sequence using Primer3Plus^39^ (Table S2). We performed the Levene’s test on the variance equality using R ver. 3.3.3 (available at https://cran.r-project.org/). Statistical analysis (two-way analysis of variance (two-way ANOVA)) was performed using the Mac Statistical Analysis ver. 2.0 (Esumi, Tokyo, Japan).

### Gene ontology (GO) enrichment analysis

Translated protein sequences of the *R. speratus* genome data (gene model RspeOGS1.0^19^) were subjected to BlastP searches against the 'nr' database of NCBI with an e-value threshold of 1e-4. In addition, protein domain searches using InterProScan version 5.17^40^ with the default settings were performed for those proteins. The results of BlastP and InterProScan searches were analyzed using the Blast2GO v2.8 software^41^ to provide functional annotations including GO terms. GO terms with at least 30 genes were retained for the enrichment analysis. Enrichment of DEGs in each of the GO terms were examined using Fisher’s exact test implemented with the ‘fisher.exact’ function of the ‘Exact2 × 2’ package^42^ in R.

### KEGG enrichment analysis

To assign the RspeOGS1.0 genes to KEGG pathways, we performed the BlastKOALA analysis^43^. Enrichment of DEGs in each of the GO terms was examined using Fisher’s exact test, as described above (see “Gene ontology (GO) enrichment analysis” section).

## Results and discussion

### Induction of each molt

In all colonies, neither worker nor presoldier molts occurred during the control-acetone treatments within 2 weeks (Table S3). The induction rates of the worker and presoldier molts were 73.3–85% and 66.7–90.0% (average), respectively. Because sufficient numbers of the N5 nymphs could not be obtained from multiple colonies (see “Induction of nymphoid differentiation by isolation from the natal nest” section), induction rates of nymphoids were not measured in this study. A previous study^26^ reported that the average nymphoid induction rate was approximately 35%.

### Sequencing and mapping

The minimum and maximum read counts in all 72 RNA-seq libraries were 12,592,720 and 19,447,093, respectively. Low quality and short sequences (quality score < 20, < 50 bp sequences) were removed, and the remaining reads were counted. The minimum and maximum counts of trimmed sequences were 11,886,368 and 18,346,386, respectively. These RNA-seq reads were used to map the *R. speratus* genome data (gene model OGS1.0)^19^. The minimum and maximum mapping rates were 57.50 ± 1.57% and 86.23 ± 1.46% (average ± S.D. of triplicates), respectively (Table S4). The rates observed in this study were slightly lower than but comparable with those published of the damp-wood termite *Z. nevadensis* (65.0–98.58%)^18^. Low mapping rates (< 70%) were observed of the bodies of workers (57.5%) and pre-GP workers (60.6–62.9%) (Table S4), most likely due to the presence of symbiotic protists in their hind guts^44–46^. Even in the worker bodies, however, high mapping rates were observed after the gut purge (82.2–85.5%), owing to the loss of symbionts from their hind guts. We excluded the data probably derived from symbiotic protists and other microorganisms in the following analysis, because these data might not map to the genome sequence, as previously shown in *Z. nevadensis*^18^.

### DEGs between natural and 20E-treated workers

No DEGs were observed between the 20E-induced GP workers and the natural GP workers (FDR < 0.01), when all genes were analyzed (11,884 and 12,481 genes for head and body, respectively). Because presoldier differentiation is usually quite rare in mature colonies under natural conditions, most natural GP worker castes are composed of individuals that are at the stage before the worker molt. These results suggest that artificial 20E treatment could be used to induce more developmentally synchronized individuals during and after the worker molt. In the mature colony, the timing of worker molts is usually different from each individual, and we can never collect the pre-GP workers without the 20E treatment.

### Numbers of DEGs during each molt

**Figure 2:**
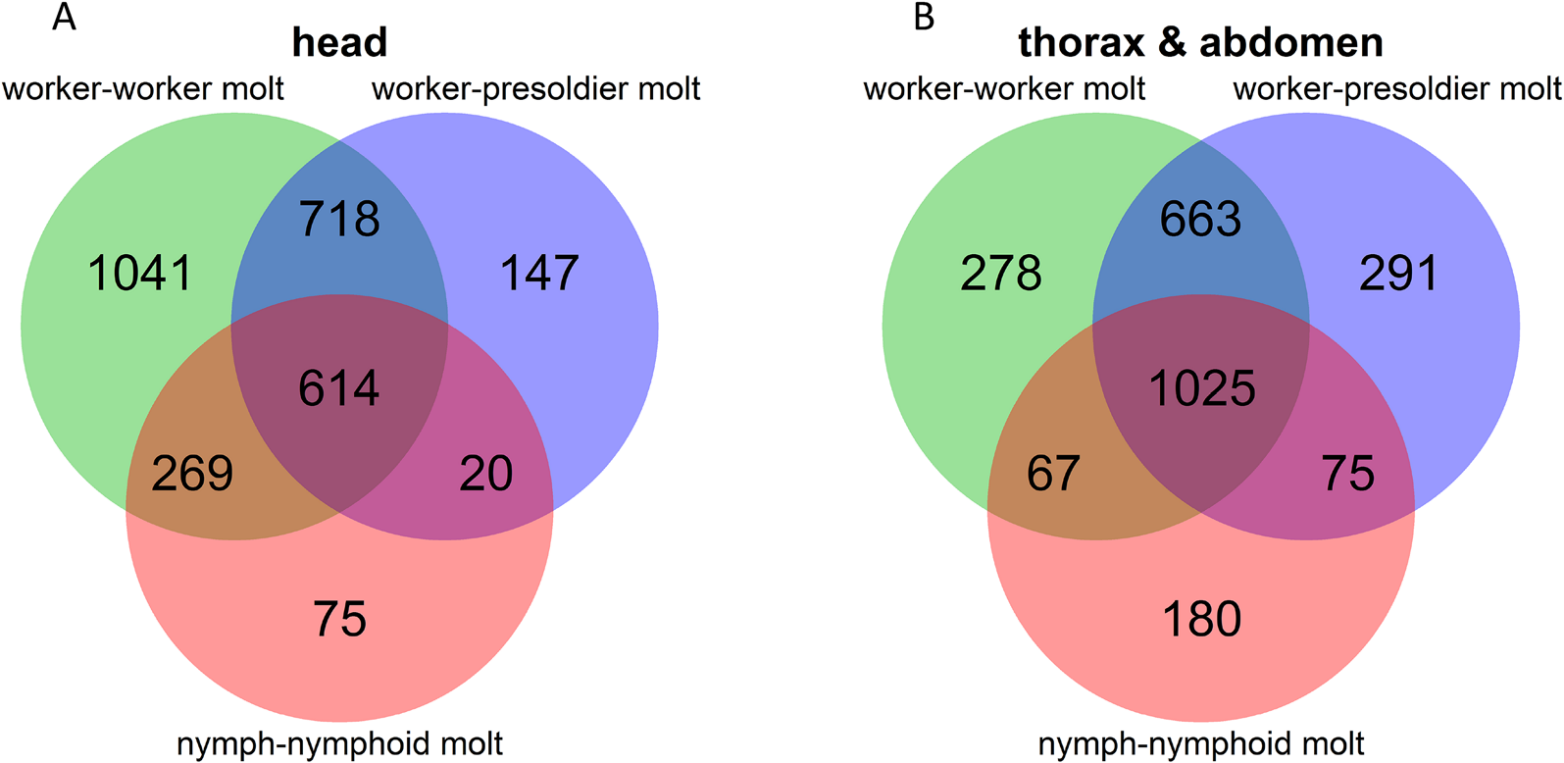
Venn diagram showing the numbers of differentially expressed and co-expressed genes among worker-worker molt, worker-presoldier molt and nymph-nymphoid molt in head (**A**) and body (thorax and abdomen) (**B**).

The numbers of differentially expressed and co-expressed genes in each molt are shown in the Venn diagram (Fig. 2). This is the first evidence to show the DEGs among all termite caste differentiations, and many co-expressed genes (614 and 1025 for heads and bodies, respectively) responsible for three completely different molts in *R. speratus*. The number of overlapping genes between the worker and presoldier molts (head: 614 + 718, body: 1025 + 663) were larger than those between the worker and nymphoid molts (head: 614 + 269, body: 1025 + 67) and between the presoldier and nymphoid molts (head: 614 + 20, body: 1025 + 75). This may be due to the different initial stages (N5 nymphs) of the nymphoid molt, compared to those of the other molts (old-age workers).

### Verification of DEGs in worker-presoldier molt

**Figure 3:**
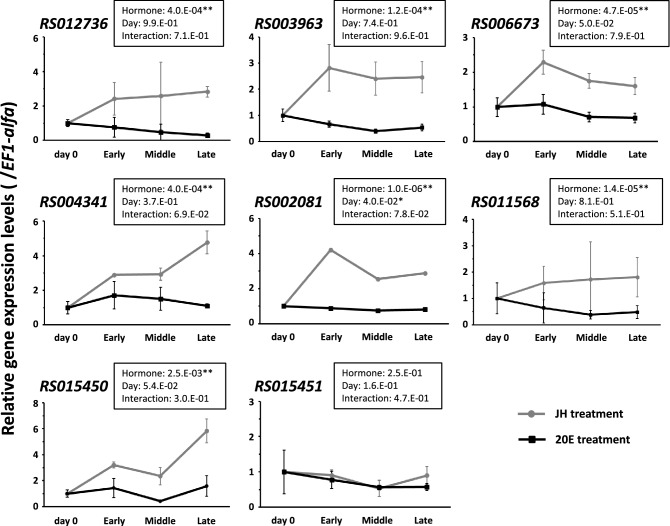
Expression patterns of eight genes identified as DEGs in heads of the worker-presoldier molt (Table S1 and Fig. S1). Gene expression levels (mean ± S.E., n = 4) were compared between worker-presoldier molt (gray line) and worker-worker molt (black line). JH- and 20E-treated individuals collected at three time points [early (day 1–2), middle (day 2–3) and late (day 3–4)] during pre-GP period, and workers in the colony (day 0), were used for the analysis. All data are consistent with the use of parametric statistics by the Levene’s test (*p* values are shown in the “Verification of DEGs in worker-presoldier molt” section). Statistical results of two-way ANOVA are described in each box (*p < 0.05, **p < 0.01). Significant differences among time points were only observed in *RS002081*, and no interaction was detected between treatments and time points in all genes.

We concentrated on some genes that were highly expressed in the heads of JH-treated workers during the pre-GP period (Table S1). Based on the stability values obtained by GeNorm and NormFinder software (Table S5), we selected *EF-1 alfa* as the internal control gene. All data are consistent with the use of parametric statistics by the Levene’s test (*RS012736*: *p* = 0.1995; *RS003963*: *p* = 0.409; *RS006673*: *p* = 0.918; *RS004341*: *p* = 0.556; *RS002081*: *p* = 0.876; *RS011568*: *p* = 0.452; *RS015450*: *p* = 0.67; *RS015451*: *p* = 0.784). Relatively higher gene expression levels were observed in JH-treated than in 20E-treated workers, and significant differences were detected in seven out of eight genes examined (Fig. 3; two-way ANOVA, *P* < 0.05). These results validated the DEGs list derived from the RNA-seq analysis. Specific morphogenesis of (pre)soldiers (e.g., frontal gland formation in the head part^11^) is probably initiated during the pre-GP period^23^, and thus the function of the identified genes should be studied in-depth to facilitate a full understanding of the proximate mechanism of soldier differentiation.

### Gene ontology (GO) terms specifically observed during each molt

The number of GO terms specifically observed during the worker-worker molt (odds ratio > 1) was much larger than that of the other two molts (Tables 1, 2, 3). In particular, the expression levels of genes involved in chemical transport and transporter activity specifically fluctuated during the worker-worker molt (16 out of 32 specific GO terms; Table 1 and S6). In contrast, the expression of genes involved in the chemical compound biosynthetic or metabolic processes specifically fluctuated during the worker-presoldier molt (12 out of 17 specific GO terms; Table 2). For example, in the isoprenoid metabolic processes (GO: 0006720), expression levels of *juvenile hormone acid methyltransferase* (*JHAMT*) (gene ID RS007861), and predicted genes of *cytochrome P450 305a1* (RS000980, RS000985, and RS013788) fluctuated significantly (Table S7). Although this GO term was not found in the head (including the corpora allata), expression changes in these genes may be related to JH titer changes during the presoldier molt. In the same GO term, expression levels of predicted genes of *geranylgeranyl pyrophosphate synthase* (*GGPPS*) (RS007482 and 100014) were also shown to be significantly changed. A previous study found that several *GGPPS* paralogs were specifically expressed in the frontal gland cells of *R. speratus* soldier heads^35^. Since *Reticulitermes* soldiers have a frontal gland that produces chemical compounds, including several types of terpenes^47^, these findings may be related to the production of chemicals involved in both defensive (a toxic secretion to the enemies) and non-defensive (regulation of soldier differentiation and antimicrobial activity) roles^48–50^. Moreover, in the amine metabolic processes (GO: 0009308, 0044106 and 0006576), expression levels of *N-b-alanyldopamine* (*NBAD*) *synthase* (= *ebony*, RS003967) and predicted genes of *Aromatic-L-amino-acid decarboxylase* (= *DDC*, RS006642) and *henna* (RS010135) were significantly fluctuating (Table S7). Henna is an enzyme that catalyzes the reaction from phenylalanine to tyrosine in *Locusta migratoria*^51^ while DDC and ebony are important enzymes in the tyrosine metabolic (TM) pathway required for the cuticular tanning of insects^52,53^. Changes in the expression of these genes were consistent with the results obtained during soldier differentiation in *Z. nevadensis*^54^. However, the expression levels of genes related to chitin-based cuticle development (GO ID 0040003), including *laccase2* (= *Lac2*, RS002050) and predicted genes of some cuticle proteins (RS004549, RS004555, RS004558, RS004559, and RS013966), fluctuated significantly during the nymph-nymphoid molt (Table 3 and S8). In *Z. nevadensis*, high *Lac2* expression was observed not only during the presoldier-soldier molt, but also during the nymph-alate molt^54^. Although alates have well-developed black cuticles, nymphoids also have dark pigmentation when compared to nymphs, particularly in the head region^55,56^. Thus, some genes involved in cuticular formation may be highly expressed during nymph-nymphoid molt.

**Table 1:** Gene Ontology (GO) terms specifically observed during the worker-worker molt.

Term	Category	Accession number	Body parts examined	No. DEGs	No. none DEGs	Odds ratio	Corrected *p*-value
Glutathione metabolic process	Biological process	GO:0006749	Body	15	19	4.055244573	0.000588672
Hydrolase activity, acting on carbon–nitrogen (but not peptide) bonds, in linear amides	Molecular function	GO:0016811	Head	17	17	3.792480637	0.000652214
Amino acid transmembrane transporter activity	Molecular function	GO:0015171	Head	15	16	3.552104298	0.002234252
Organic anion transmembrane transporter activity	Molecular function	GO:0008514	Head	21	23	3.468133044	0.000421117
Organic acid transmembrane transporter activity	Molecular function	GO:0005342	Head	20	22	3.45089148	0.00062807
Carboxylic acid transmembrane transporter activity	Molecular function	GO:0046943	Head	19	22	3.276583717	0.001171744
Secondary active transmembrane transporter activity	Molecular function	GO:0015291	Head	36	44	3.125251096	8.11E-06
Anion transmembrane transport	Biological process	GO:0098656	Head	22	27	2.844059561	0.002221008
Solute:cation symporter activity	Molecular function	GO:0015294	Head	19	26	2.770878864	0.004087267
Symporter activity	Molecular function	GO:0015293	Head	19	26	2.770878864	0.004087267
Anion transmembrane transporter activity	Molecular function	GO:0008509	Head	33	46	2.73453831	0.000155818
Organic acid transport	Biological process	GO:0015849	Head	21	27	2.712799635	0.005816385
Metalloendopeptidase activity	Molecular function	GO:0004222	Body	21	43	2.710134939	0.002125256
Pyridoxal phosphate binding	Molecular function	GO:0030170	Head	18	26	2.623693627	0.007317148
Carboxylic acid transport	Biological process	GO:0046942	Head	20	27	2.582104247	0.008623207
Hydrolase activity, acting on carbon–nitrogen (but not peptide) bonds	Molecular function	GO:0016810	Head	25	39	2.434489667	0.003291214
Response to oxidative stress	Biological process	GO:0006979	Body	20	43	2.390138252	0.009153678
Organic anion transport	Biological process	GO:0015711	Head	25	39	2.236969338	0.009015542
Coenzyme binding	Molecular function	GO:0050662	Body	38	100	2.12042096	0.000717479
Anion transport	Biological process	GO:0006820	Head	38	63	2.113772355	0.003034655
Transferase activity, transferring hexosyl groups	Molecular function	GO:0016758	Head	38	71	2.038383378	0.00277491
Fatty acid metabolic process	Biological process	GO:0006631	Head	35	62	1.974699191	0.007959462
Cofactor binding	Molecular function	GO:0048037	Head	62	126	1.884929979	0.000519646
Coenzyme binding	Molecular function	GO:0050662	Head	44	94	1.782666039	0.008329613
Active transmembrane transporter activity	Molecular function	GO:0022804	Head	54	118	1.746781003	0.003921257
Calcium ion binding	Molecular function	GO:0005509	Head	62	136	1.743630536	0.001981543
Transmembrane transporter activity	Molecular function	GO:0022857	Head	148	354	1.629121597	1.93E-05
Transmembrane transport	Biological process	GO:0055085	Head	160	367	1.568189968	9.87E-05
Carbohydrate metabolic process	Biological process	GO:0005975	Head	95	222	1.512152181	0.006260291
Substrate-specific transmembrane transporter activity	Molecular function	GO:0022891	Head	120	310	1.492371085	0.001908885
Substrate-specific transporter activity	Molecular function	GO:0022892	Head	131	351	1.439215118	0.002775891
Single-organism process	Biological process	GO:0044699	Head	984	3026	1.363537172	4.24E-06

**Table 2:** Gene Ontology (GO) terms specifically observed during the worker-presoldier molt.

Term	Category	Accession number	Body parts examined	No. DEGs	No. none DEGs	Odds ratio	Corrected *p*-value
Oxidoreductase activity, acting on the aldehyde or oxo group of donors, NAD or NADP as acceptor	Molecular function	GO:0016620	Head	14	22	4.792387095	0.000153701
Oxidoreductase activity, acting on the aldehyde or oxo group of donors	Molecular function	GO:0016903	Head	15	30	3.765333354	0.000597631
Cellular response to xenobiotic stimulus	Biological process	GO:0071466	Body	15	25	3.042030041	0.005932687
Response to xenobiotic stimulus	Biological process	GO:0009410	Body	15	25	3.042030041	0.005932687
Xenobiotic metabolic process	Biological process	GO:0006805	Body	15	25	3.042030041	0.005932687
Isoprenoid metabolic process	Biological process	GO:0006720	Body	20	34	2.990991386	0.00120716
Monocarboxylic acid biosynthetic process	Biological process	GO:0072330	Head	22	59	2.676947168	0.001537407
Indole-containing compound metabolic process	Biological process	GO:0042430	Body	18	36	2.536867738	0.007511795
Indolalkylamine metabolic process	Biological process	GO:0006586	Body	17	34	2.535509399	0.009978944
Tryptophan metabolic process	Biological process	GO:0006568	Body	17	34	2.535509399	0.009978944
Amine metabolic process	Biological process	GO:0009308	Body	21	47	2.268869914	0.008310391
Cellular amine metabolic process	Biological process	GO:0044106	Body	21	47	2.268869914	0.008310391
Cellular biogenic amine metabolic process	Biological process	GO:0006576	Body	21	47	2.268869914	0.008310391
Carboxylic acid biosynthetic process	Biological process	GO:0046394	Body	46	115	2.052840719	0.000681819
Organic acid biosynthetic process	Biological process	GO:0016053	Body	46	115	2.052840719	0.000681819
Monocarboxylic acid metabolic process	Biological process	GO:0032787	Body	48	137	1.794352777	0.004170597
Single-organism process	Biological process	GO:0044699	Body	746	3358	1.337965479	9.93E-05

**Table 3:** Gene Ontology (GO) terms specifically observed during the nymph-nymphoid molt.

Term	Category	Accession number	Body parts examined	No. DEGs	No. none DEGs	Odds ratio	Corrected *p*-value
Chitin-based cuticle development	Biological process	GO:0040003	Head	16	14	14.63034311	9.08E−10
Transferase activity, transferring sulfur-containing groups	Molecular function	GO:0016782	Head	9	22	5.371889084	0.00118848
Aminoglycan catabolic process	Biological process	GO:0006026	Head	10	26	4.856176597	0.001752514
Carbohydrate transport	Biological process	GO:0008643	Head	11	33	4.213021547	0.002265424
UDP-glycosyltransferase activity	Molecular function	GO:0008194	Body	14	44	2.978165227	0.004832037
Proteolysis	Biological process	GO:0006508	Head	58	414	1.83936313	0.001061719
Hydrolase activity	Molecular function	GO:0016787	Head	153	1407	1.558537962	8.73E−05

### Kyoto encyclopedia of genes and genomes (KEGG) terms specifically observed during each molt

Nine specific KEGG terms (odds ratio > 1) were observed during the worker-worker molt (Table 4). From the head, more than half of the genes in the insect hormone (JH and 20E) biosynthesis pathway (KEGG: ko00981) were identified as DEGs. These genes were predicted to be *juvenile hormone epoxide hydrolase* (*JHEH*) *1-like* (RS011542), *JHAMT* (RS007861), and predicted genes of *cytochrome P450* (two mitochndrial (*P450 302a1* (RS012246) and *315a1* (RS010451)) and three nuclear genes (*P450 18a1* (RS002863), *306a1* (RS002862) and *307a1* (RS010514)) (Table S9). JHEH is an enzyme that inactivates JH. In a previous study, JHEH hydrolyzed JH III to JH III-diol in the cat flea *Ctenocephalides felis*^57^. Thus, changes in the expression of these JH-related genes in the head may be related to changes in JH titer during the worker-worker molt. Moreover, in the body region, the expression levels of many genes involved in the fatty acid biosynthesis pathway (ko00061) fluctuated (Table 4). In the biosynthesis of unsaturated fatty acids and PPAR signaling pathways (ko01040 and ko03320), the expression levels of 14 predicted *Acyl-CoA Delta (11) desaturase*, which is an important gene for lepidopteran sex pheromone synthesis (derived from fatty acids)^58^, significantly fluctuated in heads (Table S9). Thus, Acyl-CoA Delta (11) desaturase could be involved in the synthesis of some releaser pheromones or related substances derived from worker heads. Alternatively, expression patterns of genes related to biosynthesis and/or metabolism of fatty acids may reflect the changes of nutrition status during a molt. A biochemical analysis of workers prior to the molt should be performed to clarify these possibilities.

**Table 4:** Kyoto Encyclopedia of Genes and Genomes (KEGG) terms specifically observed during each molt.

Term	Category	Accession number	Body parts examined	No. DEGs	No. none DEGs	Odds ratio	Corrected *p*-value
Worker	Caffeine metabolism	ko00232	Head	7	1	32.86011576	0.000793442
	Fatty acid biosynthesis	ko00061	Body	8	7	8.936293912	0.001121242
	Renin-angiotensin system	ko04614	Head	10	7	6.727925435	0.002796424
	Insect hormone biosynthesis	ko00981	Head	9	7	6.044562799	0.00833359
	Biosynthesis of unsaturated fatty acids	ko01040	Head	10	9	5.229584499	0.00833359
	PPAR signaling pathway	ko03320	Head	18	18	4.759532998	0.000223277
	Glutathione metabolism	ko00480	Body	12	27	3.492009557	0.009576391
	Neuroactive ligand-receptor interaction	ko04080	Body	16	37	3.423786174	0.00216174
	Pancreatic secretion	ko04972	Body	15	35	3.386231649	0.003589559
Presoldier	Phenylalanine metabolism	ko00360	Body	10	8	9.860043766	0.000129709
	Histidine metabolism	ko00340	Head	10	13	8.637521899	0.000190487
	Tryptophan metabolism	ko00380	Head	12	20	6.775630086	0.00015598
	Histidine metabolism	ko00340	Body	10	13	6.061097295	0.001193997
	Protein digestion and absorption	ko04974	Head	10	21	5.334293645	0.003210678
	Terpenoid backbone biosynthesis	ko00900	Head	10	22	5.09067243	0.003818987
	Tryptophan metabolism	ko00380	Body	12	19	4.994117456	0.000982212
	Cocaine addiction	ko05030	Body	10	18	4.370871418	0.007324307
	Longevity regulating pathway—worm	ko04212	Head	15	52	3.261587476	0.005954121
Nymphoid	Hypertrophic cardiomyopathy (HCM)	ko05410	Head	7	25	6.80282238	0.006687023

During the worker-presoldier molt, a total of nine specific KEGG terms (odds ratio > 1) were observed (Table 4), most of which were metabolic pathways of amino acids (histidine, phenylalanine, and tryptophan) (ko00340, ko00360, and ko00380). These results suggest that nutrition status of individuals is remarkably changed during the molting event. The other remarkable KEGG terms were the terpenoid backbone biosynthesis (ko00900) and longevity regulating pathway (ko04212), both of which were specifically observed in the head. In the former pathway, expression levels of 10 genes fluctuated significantly, including a total of eight predicted *geranylgeranyl pyrophosphate synthase* (*GGPPS*) genes (Table S10). These results were not contradictory to the GO analysis (“Verification of DEGs in worker-presoldier molt” section). These findings also suggest that there are numerous *GGPPS* paralogs in the *R. speratus* genome that are specifically expressed in soldier frontal gland cells, as shown in the previous study^35^. Recent genome analysis of *R. speratus* clarified this possibility. Total 13 *GGPPS* paralogs were observed in the same scaffold and some of these were highly expressed in soldier heads^19^. Further research is required to determine whether they play a role in the biosynthesis of soldier defensive chemicals during the presoldier molt. In the longevity regulating pathway, expression levels of the predicted *insulin-like receptor* (RS007018) were found to fluctuate in heads during the presoldier molt (Table S10). A previous study found that *insulin receptor* was strongly expressed in mandibular epithelial tissues, and that RNAi treatment caused soldier-specific morphogenesis, including mandibular elongation, in *Hodotermopsis sjostedti*^12^. The present results also suggest that insulin/insulin-like signaling activity and related specific cell proliferation may activate drastic morphological reorganization during presoldier differentiation.

Only one specific KEGG term was observed in the heads during nymph-nymphoid molt (Table 4). Five out of seven DEGs were predicted to be *angiotensin-converting enzyme* (*ACE*) (RS009620, RS009621, RS009623, RS009070, and RS009071) (Table S11). Several insects’ brains showed immune responses to ACE, suggesting that insect ACE is involved in the activation and/or inactivation of peptide hormones in the nervous system^59^. Furthermore, several ACE paralogs have been identified in the genome of *Bombyx mori*, and highly expressed paralogs may be involved in growth and reproduction^60^. Further research is needed to confirm the function of these genes during nymphoid differentiation.

## Conclusion

This study aimed to develop a comprehensive list of the molecular mechanisms underlying termite caste differentiation. We performed both GO and KEGG enrichment analyses using the differential gene expression profiles of each molt (worker, presoldier, and nymphoid molts) of *R. speratus*. Based on the timing of initiation of the gut purge (GP), we identified three developmental stages during each molt (pre-GP, GP, and after the molt). The results revealed that a large number of genes were differentially expressed during each molt and/or were differentially expressed in each body part. In each molt, the expression of genes, related to JH titer changes, nutrition status, and cell proliferation, specifically fluctuated. These differences may have a crucial impact on caste differentiation. To understand the regulatory mechanisms of caste differentiation in termites, more functional and detailed expression analyses of the genes listed here are required. It should be noted that some gene expressions, especially observed in the worker-presoldier molt, may be affected by the artificial hormone treatment. Further comparative transcriptome analysis should be performed between artificial conditions described in this paper and natural conditions, the latter of which has been reported in other species^17,61^.

## Supplementary Information

Supplementary Information.

## Data Availability

DDBJ accession numbers for RNA-seq data is DRA013156.
