# Supplementary material for: Comparison of gene expression profiles among caste differentiations in the termite Reticulitermes speratus
Source: Sci Rep. 2022 Jul 13;12:11947. doi: 10.1038/s41598-022-15984-z (PMC9279399; doi:10.1038/s41598-022-15984-z)
Supplement: Supplementary file 1 — Supplementary Information. [file 41598_2022_15984_MOESM1_ESM.pdf]

Supplementary Information for:

Comparison of gene expression profiles among caste differentiations in the termite *Reticulitermes speratus*

Ryota Saiki[1,4], Yoshinobu Hayashi[2,5], Kouhei Toga[1,6], Hajime Yaguchi[1,7], Yudai Masuoka[1,8], Ryutaro Suzuki[1,4], Kokuto Fujiwara[1], Shuji Shigenobu[3] and Kiyoto Maekawa[9]

[1]Graduate School of Science and Engineering, University of Toyama, Toyama 930-8555 Japan
[2]Laboratory of Ecological Genetics, Graduate School of Environmental Science, Hokkaido University, Sapporo 060–0810, Japan
[3]NIBB Core Research Facilities, National Institute for Basic Biology, Okazaki 444-8585, Japan
[4]Ishikawa Insect Museum, Hakusan, Ishikawa 920-2113, Japan
[5]Department of Biology, Keio University, Yokohama, Kanagawa 223-8521, Japan
[6]Department of Biosciences, College of Humanities and Sciences, Nihon University, Tokyo 156-8550, Japan
[7]Department of Bioscience, School of Science and Technology, Kwansei Gakuin University, Sanda, Hyogo 669-1337, Japan
[8]Institute of Agrobiological Sciences, National Agriculture and Food Research Organization, Tsukuba 305-8634, Japan
[9]Faculty of Science, Academic Assembly, University of Toyama, 3190 Gofuku, Toyama 930-8555, Japan

Table of Contents:

Figure S1. Gene expression patterns of eight genes identified as DEGs in worker-presoldier molt (head).	Page 001
Table S1. Genes selected for verification of DEGs in worker-presoldier molt.	Page 002
Table S2. Primer sequences used in this study.	Page 003
Table S3. Induced molt and mortality rates (%) of workers and presoldiers in each colony (average ± S.D.).	Page 004
Table S4. Mapping rates (%) of each categories (n = 3, average ± S.D.).	Page 005
Table S5. Stability values of internal control genes using GeNorm and NormFinder.	Page 006
Table S6. Genes belonging to the significant GO terms specifically observed during the worker-worker molt.	Page 007
Table S7. Genes belonging to the significant GO terms specifically observed during the worker-presoldier molt.	Page 082
Table S8. Genes belonging to the significant GO terms specifically observed during the nymph-nymphoid molt.	Page 130

Table S9. Genes belonging to the significant KEGG terms specifically observed during the worker-worker molt.	Page 151
Table S10. Genes belonging to the significant KEGG terms specifically observed during the worker-presoldier molt.	Page 155
Table S11. Genes belonging to the significant KEGG terms specifically observed during the nymph-nymphoid molt.	Page 159

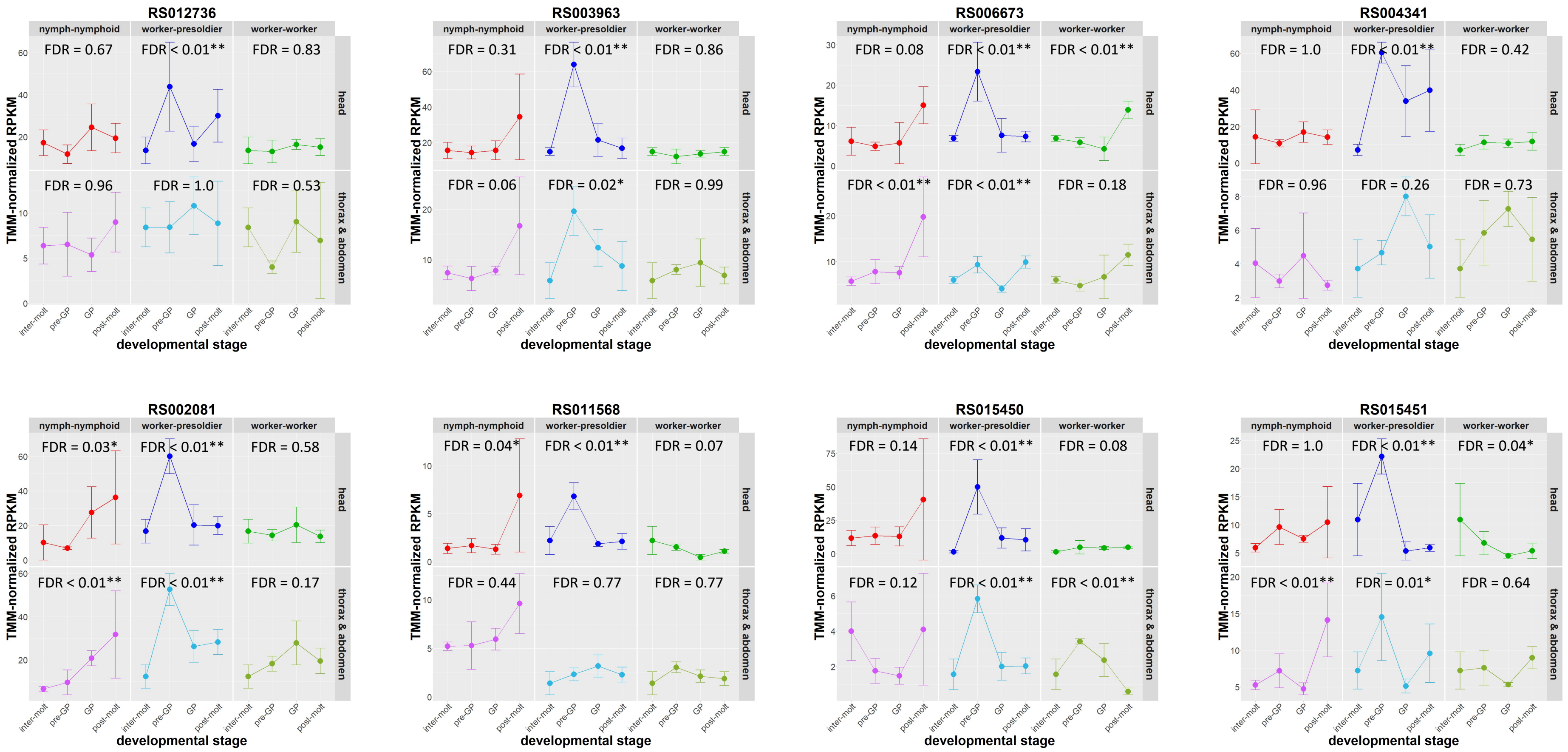

Fig. S1

Gene expression patterns of eight genes identified as DEGs in worker-presoldier molt (head). See Table S1 for details. Expression levels are indicated as RPKM (reads per kilobase million) values normalized with the TMM (trimmed mean of M-values) method calculated from RNA-seq analysis. Statistical results of GLM analysis with edgeR are indicated in each graph (*FDR < 0.05, **FDR < 0.01).

Table S1. Genes selected for verification of DEGs in worker-presoldier molt.
LogFC.Stage means the log fold-change in expression between each developmental stage and worker.
LR means the likelihood ratio of the full model and the null model that assumes no differences in the expression levels among developmental stages.

seq ID	logFC.Pre-GP	logFC.GP	logFC.Molt	logCPM	LR	PValue	FDR	Annotation
RS015450	5.056853423	3.008390928	2.84679032	4.777376403	68.91864819	7.27E-15	1.03E-12	PREDICTED: growth/differentiation factor 11
RS003963	2.123502355	0.575652995	0.238107852	4.850579245	41.60596964	4.86E-09	0.000000169	PREDICTED: protein hairy
RS004341	3.081282603	2.285990042	2.515177471	5.495647985	33.64881052	0.000000235	0.00000571	PREDICTED: fork head domain-containing protein crocodile
RS006673	1.792710733	0.201278723	0.148825807	5.596223357	33.21239508	0.00000029	0.00000686	PREDICTED: SUN domain-containing ossification factor
RS015451	1.034757745	-0.98691223	-0.825283638	4.602925156	31.85849723	0.000000561	0.000012	PREDICTED: zinc finger protein jing homolog
RS002081	1.854953873	0.318936438	0.302135384	5.750568406	26.67821789	0.00000688	0.000114979	PREDICTED: Krueppel homolog 1-like
RS011568	1.621155039	-0.216238813	-0.022238118	2.020960193	20.66462098	0.000123582	0.00144156	PREDICTED: protein vestigial
RS012736	1.709532012	0.34960297	1.202520287	5.250036073	16.32728374	0.000971565	0.008420907	PREDICTED: sine oculis-binding protein homolog

Table S2. Primer sequences used in this study.

Gene name	Primer name	Sequence (5' to 3')
beta-actin	Rspe_beta-actin qPCR-F	5'- AGCGGGAAATCGTCCGTGA -3'
	Rspe_beta-actin qPCR-R	5'- CAATGGTGATGACCTGCCCAT -3'
EF1-alpha	Rspe_EF1-alpha qPCR-F	5'- GGTGATGCGGCTATTGTTAACC -3'
	Rspe_EF1-alpha qPCR-R	5'- GTGGTGGGAATTCTGAGAAAGATT -3'
NADH-dh	Rspe_NADH-dh qPCR-F	5'- GCTGGGGGGGTTATTCATTCCAT -3'
	Rspe_NADH-dh qPCR-R	5'- GGCATACCACAAAGGGCAAAA -3'
GstD1	Rspe_GstD1 qPCR-F	5'- GCTGTTGGTGTGGATTTGAA -3'
	Rspe_GstD1 qPCR-R	5'- GTATGCTGCGGGTTCATCTT -3'
EIF-1	Rspe_EIF-1 qPCR-F	5'- ATGGTAGGCTTGAAGCGATG -3'
	Rspe_EIF-1 qPCR-R	5'- TTTGCATCCTGGTAGTCACG -3'
RPS18	Rspe_RPS18 qPCR-F	5'- ACTCTCAGCTCACATCCAGT -3'
	Rspe_RPS18 qPCR-R	5'- CCTCAGGCCCCAATAATGTC -3'
RS002081	RS002081_F_qPCR	5'- TCGAACACAGCGGTAAACTG -3'
	RS002081_R_qPCR	5'- TGGATAAACGTCTTGGCACA -3'
RS003963	RS003963_F_qPCR	5'- TGCAAGGATCAACCAGTCCC -3'
	RS003963_R_qPCR	5'- AGGATGTCCGCCTTTTCCAG -3'
RS004341	RS004341_F_qPCR	5'- GGGTTGTGGGTACGTTCAGT -3'
	RS004341_R_qPCR	5'- CCCCGTGTAGCTGAGATGTT -3'
RS006673	RS006673_F_qPCR	5'- GAACTGTGTGAGGCCATTCA -3'
	RS006673_R_qPCR	5'- ACTCAGCCACACGGAGAAGT -3'
RS011568	RS011568_qPCR_F	5'- CCAGGACAACCCCATTTCCA -3'
	RS011568_qPCR_R	5'- GTTGGCAGAGACGAACCAGA -3'
RS012736	RS012736_F_qPCR	5'- TCACTGAAGACACCGACTGG -3'
	RS012736_R_qPCR	5'- CGTTTTGTTCCGCTTGAAGT -3'
RS015450	RS015450_F_qPCR	5'- ATTACGCCATGGACTCAAGG -3'
	RS015450_R_qPCR	5'- CCCTGAGGTGTATCCAGAGC -3'
RS015451	RS015451_F_qPCR	5'- CTTGTGAAACCGCATCTGGC -3'
	RS015451_R_qPCR	5'- CTTGCTTTGGACAGTGGCAC -3'

Table S3. Induced molt and mortality rates (%) of workers and presoldiers in each colony (average ± S.D.).

Colony A			
Treatment	Acetone control (n = 1)	20E for worker-worker molt (n = 2)	JH III for worker-presoldier molt (n = 3)
Molt	0	75.0 ± 21.2	66.7 ± 7.6
Mortality	5	17.5 ± 10.6	10.0 ± 8.7
Colony B			
Treatment	Acetone control (n = 1)	20E for worker-worker molt (n = 3)	JH III for worker-presoldier molt (n = 3)
Molt	0	73.3 ± 12.6	73.3 ± 20.8
Mortality	0	8.3 ± 2.9	11.7 ± 16.1
Colony C			
Treatment	Acetone control (n = 1)	20E for worker-worker molt (n = 1)	JH III for worker-presoldier molt (n = 3)
Molt	0	85	90.0 ± 0.0
Mortality	10	0	8.3 ± 2.9

Table S4. Mapping rates (%) of each categories (n = 3, average ± S.D.).

A. Worker - worker molt					
	workers	Pre-GP workers	GP workers	Molt workers	Natural GP workers
Head	83.97 ± 0.71	85.60 ± 0.66	81.30 ± 7.71	86.23 ± 1.46	83.27 ± 1.81
Body	57.50 ± 1.57	60.60 ± 5.02	82.23 ± 6.55	84.83 ± 2.25	85.00 ± 2.35
B. Worker - presoldier molt					
		Pre-GP workers	GP workers	Molt presoldiers	
Head		83.60 ± 2.01	84.60 ± 2.10	85.90 ± 0.44	
Body		62.90 ± 9.88	85.47 ± 1.64	84.67 ± 2.66	
C. Nymph - nymphoid molt					
	Nymphs	Pre-GP nymphs	GP nymphs	Molt nymphoids	
Head	85.57 ± 1.14	83.20 ± 1.04	83.10 ± 1.06	82.97 ± 3.57	
Body	76.47 ± 7.11	83.63 ± 4.33	83.93 ± 3.70	83.47 ± 0.71	

Table S5. Stability values of internal control genes using GeNorm and NormFinder.

Gene name	Accession no/Seq ID	Stability value (GeNorm)	Stability value (NormFinder)
*EF1-alfa**	AB602838	0.425147601	0.105355386
NADH-dh	AB602837	0.503490174	0.202707439
beta-actin	AB520714	0.776113561	0.506199366
GstD1	RS001168	0.633917593	0.388007161
*EIF-1**	RS005199	0.439554553	0.075944942
RPS18	RS015150	0.468854556	0.198810353

*Candidate internal control genes due to the low stability values.

Table S6. Genes belonging to the significant GO terms specifically observed during the worker-worker molt.
LogFC.Stage means the log fold-change in expression between each developmental stage and worker/nymph. LR means the likelihood ratio of the full model and the null model that assumes no differences in the expression levels among developmental stages.

seq ID	GO ID	GO Name	GO category	logFC.Pre-GP	logFC.GP	logFC.Molt	logCPM	LR	P Value	FDR	Annotation
RS009320	GO:0006749	glutathione metabolic process	BP	-2.884845028	-0.020862095	5.230683542	4.703088105	253.4010396	1.20E-54	1.41E-52	PREDICTED: superoxide dismutase [Cu-Zn]-like
RS007455	GO:0006749	glutathione metabolic process	BP	-0.77532182	-0.627371757	1.27736906	4.392216644	37.83412897	3.06E-08	3.93E-07	PREDICTED: glutathione peroxidase 6
RS003134	GO:0006749	glutathione metabolic process	BP	2.062228103	0.951620284	-0.406153315	6.753303233	34.81508325	1.33E-07	1.56E-06	PREDICTED: glutathione S-transferase-like
RS009342	GO:0006749	glutathione metabolic process	BP	-1.217914674	-2.30744558	-0.307894532	9.683899353	28.21324829	3.28E-06	3.21E-05	PREDICTED: pyrimidodiazepine synthase-like
RS001401	GO:0006749	glutathione metabolic process	BP	-0.977743475	-1.429648234	0.392313729	5.961713183	27.81212111	3.98E-06	3.85E-05	PREDICTED: isocitrate dehydrogenase [NADP] cytoplasmic-like
RS009343	GO:0006749	glutathione metabolic process	BP	-1.193613349	-1.438254177	0.667042255	7.32338802	25.38032447	1.29E-05	0.000116334	PREDICTED: pyrimidodiazepine synthase-like
RS007454	GO:0006749	glutathione metabolic process	BP	-0.535903331	-0.95556464	1.260192704	2.793782052	24.87535072	1.64E-05	0.00014493	Glutathione peroxidase 6
RS002014	GO:0006749	glutathione metabolic process	BP	-0.194117663	-1.487488373	-1.249022222	8.229465708	24.56515913	1.90E-05	0.000166007	PREDICTED: cystathionine gamma-lyase
RS001168	GO:0006749	glutathione metabolic process	BP	1.345278897	0.253868771	-0.144089257	8.804984824	22.84570418	4.35E-05	0.000362027	glutathione S-transferase D1
RS006683	GO:0006749	glutathione metabolic process	BP	-2.138846935	0.841419709	0.351318347	3.204951472	20.97931603	0.000106323	0.000836439	PREDICTED: peroxiredoxin-6-like
RS003511	GO:0006749	glutathione metabolic process	BP	-1.696496143	-1.520785043	-0.820708746	4.992839142	20.54485306	0.000130861	0.001011739	PREDICTED: glutamate--cysteine ligase regulatory subunit
RS009344	GO:0006749	glutathione metabolic process	BP	-0.162677384	-0.63146939	0.659352401	5.715074271	18.03543958	0.000432509	0.003072627	PREDICTED: pyrimidodiazepine synthase-like
RS014190	GO:0006749	glutathione metabolic process	BP	-0.578216815	-1.519924339	-0.99177885	9.035509083	17.24592229	0.000629022	0.004332894	PREDICTED: probable phospholipid hydroperoxide glutathione peroxidase
RS007489	GO:0006749	glutathione metabolic process	BP	-1.189290174	-1.675727474	-0.216839483	8.546801564	17.11205004	0.000670213	0.004585075	glutathione S-transferase D1
RS000475	GO:0006749	glutathione metabolic process	BP	-1.383152416	-1.258743408	-0.724737132	5.345183519	15.6305472	0.001349908	0.008751873	PREDICTED: glutamate--cysteine ligase catalytic subunit
RS009467	GO:0006749	glutathione metabolic process	BP	0.333872237	1.127003035	0.245511411	6.694953435	15.02921718	0.00179185	0.011297899	PREDICTED: gamma-glutamyltransferase 7
RS006807	GO:0006749	glutathione metabolic process	BP	-0.12837582	-0.716605346	-0.991282865	7.447282818	9.8430759	0.019948245	0.095218842	PREDICTED: peroxiredoxin-6-like
RS000237	GO:0006749	glutathione metabolic process	BP	-0.067312161	0.973025543	0.136881421	3.262838683	7.28194536	0.063434019	0.249216021	PREDICTED: gamma-glutamyltranspeptidase 1-like
RS000508	GO:0006749	glutathione metabolic process	BP	-0.502317595	-1.067438271	-0.543592665	7.670787121	6.773929239	0.079463352	0.296608472	PREDICTED: microsomal glutathione S-transferase 3
RS010039	GO:0006749	glutathione metabolic process	BP	0.174677068	-0.252986067	-0.563499023	5.29308911	6.074124651	0.108059066	0.374451284	PREDICTED: pyrimidodiazepine synthase-like
RS003811	GO:0006749	glutathione metabolic process	BP	-0.397698274	-1.083470426	-0.048671229	7.61164351	6.066050341	1.08E-01	0.375074118	CG12628 protein
RS000244	GO:0006749	glutathione metabolic process	BP	-0.706197281	-0.422825102	0.249542668	6.465405563	4.965407191	0.174347796	0.523324918	PREDICTED: gamma-glutamyltranspeptidase 1-like
RS013874	GO:0006749	glutathione metabolic process	BP	0.013903141	-0.479297983	-0.464220577	6.451119994	3.838149087	0.279479827	0.695200058	PREDICTED: peroxiredoxin-6
RS007160	GO:0006749	glutathione metabolic process	BP	-0.266965371	-0.958259852	-0.038280786	7.986560693	3.306724355	0.346707836	0.783086679	cytoplasmic NADP+-dependent isocitrate dehydrogenase
RS014031	GO:0006749	glutathione metabolic process	BP	0.396127843	0.065477186	-0.226533202	3.415047413	2.485740827	0.477873548	0.920516744	PREDICTED: glutathione S-transferase-like
RS015157	GO:0006749	glutathione metabolic process	BP	-0.028100926	-0.209013634	-0.551958882	10.38252737	2.178881635	0.53612	0.960307014	PREDICTED: elongation factor 1-gamma
RS001190	GO:0006749	glutathione metabolic process	BP	-0.158211249	-0.190763361	-0.510494858	10.03945401	1.352757705	0.716644967	0.99990777	PREDICTED: 40S ribosomal protein S3
RS002313	GO:0006749	glutathione metabolic process	BP	-0.183433173	-0.563018765	-0.068752215	6.442015484	1.562874363	0.667836792	0.99990777	PREDICTED: glucose-6-phosphate 1-dehydrogenase
RS005799	GO:0006749	glutathione metabolic process	BP	0.037095004	0.152230836	-0.050642901	4.07489719	0.242690299	0.970420029	0.99990777	PREDICTED: probable maleylacetoacetate isomerase 2
RS011379	GO:0006749	glutathione metabolic process	BP	-0.241741309	0.199481332	0.0435458	6.927940186	0.907609555	0.823591071	0.99990777	PREDICTED: chloride intracellular channel exc-4
RS001212	GO:0006749	glutathione metabolic process	BP	-0.114686241	-0.104566831	-0.270351611	5.402498611	0.563256331	9.05E-01	1.00E+00	PREDICTED: gamma-glutamylcyclotransferase-like
RS001533	GO:0006749	glutathione metabolic process	BP	0.009651977	0.069512623	0.128771412	6.733243276	0.105607563	0.991156107	0.99990777	PREDICTED: glutathione synthetase-like
RS010009	GO:0006749	glutathione metabolic process	BP	-0.143533883	-0.210296096	-0.327043261	6.035778544	1.259262346	0.738828601	0.99990777	PREDICTED: hydroxyacylglutathione hydrolase, mitochondrial
RS015346	GO:0006749	glutathione metabolic process	BP	-0.311128354	-0.266651951	-0.078377411	7.250386727	1.259847488	0.738689037	0.99990777	PREDICTED: cystathionine gamma-lyase
RS014580	GO:0016811	hydrolase activity, acting on carbon-nitrogen (but not peptide) bonds, in linear amides	MF	-2.976523608	-6.808866752	-1.942701116	5.214122185	156.927034	8.43836E-34	4.16756E-32	PREDICTED: kynurenine formamidase-like
RS012361	GO:0016811	hydrolase activity, acting on carbon-nitrogen (but not peptide) bonds, in linear amides	MF	-0.272408132	-0.554085634	1.369334256	6.34819019	93.14482576	4.62346E-20	1.18109E-18	PREDICTED: vanin-like protein 1
RS008819	GO:0016811	hydrolase activity, acting on carbon-nitrogen (but not peptide) bonds, in linear amides	MF	-1.032692902	-2.222642533	-1.657320465	5.534608709	75.39402177	2.98301E-16	5.70943E-15	PREDICTED: glutaminase liver isoform, mitochondrial
RS010041	GO:0016811	hydrolase activity, acting on carbon-nitrogen (but not peptide) bonds, in linear amides	MF	-0.841304888	-1.311133394	1.016447212	5.056278579	65.67682339	3.59396E-14	5.85541E-13	PREDICTED: peptidoglycan-recognition protein LC-like
RS013013	GO:0016811	hydrolase activity, acting on carbon-nitrogen (but not peptide) bonds, in linear amides	MF	-1.540094428	-1.189185544	1.548167541	3.519055593	65.31696378	4.29099E-14	6.95504E-13	PREDICTED: peptidoglycan-recognition protein LB-like
RS001234	GO:0016811	hydrolase activity, acting on carbon-nitrogen (but not peptide) bonds, in linear amides	MF	-3.049313173	1.196358565	1.454889377	7.193983845	38.01690606	2.80328E-08	2.73043E-07	chitin deacetylase 4
RS013012	GO:0016811	hydrolase activity, acting on carbon-nitrogen (but not peptide) bonds, in linear amides	MF	-1.470953782	-1.559057804	-1.338853711	3.899322122	34.27053677	1.73703E-07	1.56482E-06	PREDICTED: peptidoglycan-recognition protein LB-like
RS003758	GO:0016811	hydrolase activity, acting on carbon-nitrogen (but not peptide) bonds, in linear amides	MF	-0.559608852	2.208187894	1.597392533	6.933108648	31.62779144	6.2693E-07	5.36383E-06	chitin deacetylase 3
RS015186	GO:0016811	hydrolase activity, acting on carbon-nitrogen (but not peptide) bonds, in linear amides	MF	-1.21066846	0.888184276	1.336908068	10.2190989	26.71453317	6.75703E-06	5.00577E-05	chitin deacetylase 1
RS004692	GO:0016811	hydrolase activity, acting on carbon-nitrogen (but not peptide) bonds, in linear amides	MF	-1.533477303	-1.581672673	-0.440490513	3.98590693	25.83868371	1.03087E-05	7.43996E-05	PREDICTED: histone deacetylase 4
RS005123	GO:0016811	hydrolase activity, acting on carbon-nitrogen (but not peptide) bonds, in linear amides	MF	0.781802156	0.484030121	-1.078810264	3.903771807	24.55032113	1.91713E-05	0.00013237	PREDICTED: kynurenine formamidase-like
RS000970	GO:0016811	hydrolase activity, acting on carbon-nitrogen (but not peptide) bonds, in linear amides	MF	-1.826169573	-1.767109952	-3.471752553	2.26650892	22.95978906	4.11699E-05	0.000268372	PREDICTED: alkaline ceramidase
RS005544	GO:0016811	hydrolase activity, acting on carbon-nitrogen (but not peptide) bonds, in linear amides	MF	-0.308180157	0.689248167	-0.738011841	4.466023101	22.07108314	6.30469E-05	0.000399001	PREDICTED: beta-ureidopropionase
RS005296	GO:0016811	hydrolase activity, acting on carbon-nitrogen (but not peptide) bonds, in linear amides	MF	-0.666577041	-0.650920673	0.350048094	4.593415922	21.99923013	6.52552E-05	0.000411529	PREDICTED: protein kinase C, brain isozyme
RS015187	GO:0016811	hydrolase activity, acting on carbon-nitrogen (but not peptide) bonds, in linear amides	MF	0.013748187	1.340727689	1.795231312	10.35011632	20.46140263	0.000136183	0.000809002	chitin deacetylase 1 precursor
RS014120	GO:0016811	hydrolase activity, acting on carbon-nitrogen (but not peptide) bonds, in linear amides	MF	-0.922827123	-1.289252361	-1.238323899	2.633512953	15.71916541	0.001294661	0.006118183	PREDICTED: nicotinamidase
RS006022	GO:0016811	hydrolase activity, acting on carbon-nitrogen (but not peptide) bonds, in linear amides	MF	-1.813369421	-1.750081698	-0.062538905	2.776091628	15.04967814	0.001774684	0.008054187	PREDICTED: beta-ureidopropionase
RS015068	GO:0016811	hydrolase activity, acting on carbon-nitrogen (but not peptide) bonds, in linear amides	MF	-1.101346708	-1.40826429	-0.609149078	2.183952585	13.86326014	0.003097313	0.013028576	PREDICTED: vanin-like protein 1
RS009242	GO:0016811	hydrolase activity, acting on carbon-nitrogen (but not peptide) bonds, in linear amides	MF	0.682073862	0.906384828	-0.119195863	3.436386452	13.55373124	0.003580051	0.014826428	PREDICTED: putative N-acetylglucosamine-6-phosphate deacetylase
RS015067	GO:0016811	hydrolase activity, acting on carbon-nitrogen (but not peptide) bonds, in linear amides	MF	-1.545741566	-1.369518026	-0.217080276	1.014262688	9.345369419	0.025034565	0.076284856	PREDICTED: vanin-like protein 1
RS006460	GO:0016811	hydrolase activity, acting on carbon-nitrogen (but not peptide) bonds, in linear amides	MF	-1.355855211	-0.229188099	0.081221757	1.62049498	7.749984171	0.051471499	0.135585036	CG9380
RS003177	GO:0016811	hydrolase activity, acting on carbon-nitrogen (but not peptide) bonds, in linear amides	MF	0.737615696	0.712418457	0.180131171	3.012166732	6.812877803	0.078107285	0.186268348	PREDICTED: protein N-terminal glutamine amidohydrolase
RS008195	GO:0016811	hydrolase activity, acting on carbon-nitrogen (but not peptide) bonds, in linear amides	MF	0.533863066	0.426337346	0.621528576	6.350472863	6.759324797	0.079977665	0.189622234	PREDICTED: NAD-dependent protein deacylase sirtuin-5, mitochondrial
RS008683	GO:0016811	hydrolase activity, acting on carbon-nitrogen (but not peptide) bonds, in linear amides	MF	-0.685747102	-0.486113188	-0.249052476	4.253825687	5.276867736	0.152610471	0.305512044	PREDICTED: aminoacylase-1-like
RS008767	GO:0016811	hydrolase activity, acting on carbon-nitrogen (but not peptide) bonds, in linear amides	MF	-0.071185743	-0.285368576	0.183550764	4.880406744	3.670316078	0.299335164	0.481890203	PREDICTED: histone deacetylase 3
RS011390	GO:0016811	hydrolase activity, acting on carbon-nitrogen (but not peptide) bonds, in linear amides	MF	0.252324079	0.550359201	0.410388975	3.310648883	3.424796226	0.330647576	0.512513312	PREDICTED: peptide deformylase, mitochondrial-like
RS010779	GO:0016811	hydrolase activity, acting on carbon-nitrogen (but not peptide) bonds, in linear amides	MF	0.212439189	-0.035024314	-0.595729899	2.244190775	3.227818371	0.357815991	0.538894287	PREDICTED: histone deacetylase 8
RS012446	GO:0016811	hydrolase activity, acting on carbon-nitrogen (but not peptide) bonds, in linear amides	MF	-0.33520903	-0.326059321	0.041645679	5.694017303	2.740242498	0.433431766	0.607177439	PREDICTED: NAD-dependent protein deacetylase sirtuin-3, mitochondrial
RS010270	GO:0016811	hydrolase activity, acting on carbon-nitrogen (but not peptide) bonds, in linear amides	MF	-0.499816687	-0.179901857	-0.776722233	3.403430526	2.21634321	0.528736447	0.688401407	PREDICTED: protein N-terminal asparagine amidohydrolase
RS009146	GO:0016811	hydrolase activity, acting on carbon-nitrogen (but not peptide) bonds, in linear amides	MF	0.273209024	0.243574118	0.076497435	5.63647883	2.001763487	0.572040767	0.721944225	PREDICTED: breast cancer metastasis-suppressor 1-like protein
RS012536	GO:0016811	hydrolase activity, acting on carbon-nitrogen (but not peptide) bonds, in linear amides	MF	0.047139577	-0.258366711	-0.192122824	5.403598014	1.660854375	0.645671538	0.77814233	PREDICTED: histone deacetylase Rpd3
RS013047	GO:0016811	hydrolase activity, acting on carbon-nitrogen (but not peptide) bonds, in linear amides	MF	0.231685496	0.106278735	0.095579367	5.899186066	1.241080553	0.743169287	0.848450098	PREDICTED: NAD-dependent protein deacetylase sirtuin-2
RS015233	GO:0016811	hydrolase activity, acting on carbon-nitrogen (but not peptide) bonds, in linear amides	MF	-0.028168318	0.124444095	-0.146279858	3.77098604	0.6887061	0.875857119	0.933052322	PREDICTED: kynurenine formamidase
RS005405	GO:0016811	hydrolase activity, acting on carbon-nitrogen (but not peptide) bonds, in linear amides	MF	-0.387669428	-0.565118451	0.210226756	1.222213754	0.652216457	0.884381294	0.938003319	PREDICTED: peptidoglycan-recognition protein LC-like
RS003228	GO:0015171	amino acid transmembrane transporter activity	MF	-0.561103954	2.12048168	1.83683218	5.406987214	114.6440514	1.09829E-24	3.71824E-23	PREDICTED: b(0,+)-type amino acid transporter 1-like
RS013974	GO:0015171	amino acid transmembrane transporter activity	MF	1.308057058	2.315023189	0.876327879	6.127768048	111.7248777	4.66769E-24	1.53887E-22	PREDICTED: monocarboxylate transporter 10
RS006315	GO:0015171	amino acid transmembrane transporter activity	MF	-2.445857294	-4.975199992	0.147442902	3.069628072	71.7469443	1.80352E-15	3.24017E-14	PREDICTED: bumetanide-sensitive sodium-(potassium)-chloride cotransporter
RS008617	GO:0015171	amino acid transmembrane transporter activity	MF	-1.758654905	-1.933395291	0.119722157	4.240860888	52.46640405	2.38231E-11	3.09308E-10	PREDICTED: proton-coupled amino acid transporter 1
RS006021	GO:0015171	amino acid transmembrane transporter activity	MF	-1.978389488	-0.987147201	0.23457041	5.767022167	52.1310105	2.80858E-11	3.63527E-10	PREDICTED: cationic amino acid transporter 3
RS009380	GO:0015171	amino acid transmembrane transporter activity	MF	1.181487238	0.230316163	-1.011984835	4.090504935	45.23692145	8.23976E-10	9.50289E-09	PREDICTED: b(0,+)-type amino acid transporter 1-like
RS001007	GO:0015171	amino acid transmembrane transporter activity	MF	-0.057453873	2.550863666	4.593751253	0.34449975	41.36132174	5.4813E-09	5.84023E-08	PREDICTED: sodium-dependent nutrient amino acid transporter 1-like
RS005851	GO:0015171	amino acid transmembrane transporter activity	MF	0.526397294	1.41413322	1.673681755	7.829535198	40.74316203	7.413E-09	7.77347E-08	PREDICTED: proton-coupled amino acid transporter 4
RS006814	GO:0015171	amino acid transmembrane transporter activity	MF	-0.514796382	1.014347124	0.975336891	4.041760153	30.97725487	8.59474E-07	7.23543E-06	PREDICTED: large neutral amino acids transporter small subunit 2
RS006320	GO:0015171	amino acid transmembrane transporter activity	MF	1.668458777	1.549661536	0.932473469	6.183206129	30.51118393	1.07731E-06	8.96741E-06	PREDICTED: bumetanide-sensitive sodium-(potassium)-chloride cotransporter
RS000193	GO:0015171	amino acid transmembrane transporter activity	MF	-0.612906575	0.552707825	0.864925878	6.395375097	30.19397061	1.25629E-06	1.03772E-05	PREDICTED: b(0,+)-type amino acid transporter 1
RS000927	GO:0015171	amino acid transmembrane transporter activity	MF	-2.570527298	-2.804707147	-0.755163456	0.667619183	16.35029216	0.000961058	0.004715063	PREDICTED: b(0,+)-type amino acid transporter 1-like
RS013870	GO:0015171	amino acid transmembrane transporter activity	MF	-1.185159015	-0.789079424	-0.942978819	4.024142004	15.86729932	0.001207277	0.005765813	PREDICTED: sodium- and chloride-dependent glycine transporter 1-like
RS000928	GO:0015171	amino acid transmembrane transporter activity	MF	-2.818387407	-2.387215456	-1.195316578	0.324906115	15.29049888	0.001584497	0.007309578	B(0,+)-type amino acid transporter 1
RS005708	GO:0015171	amino acid transmembrane transporter activity	MF	-2.57707626	-0.767810406	-0.121337824	1.208165742	14.61680361	0.002175202	0.009598631	PREDICTED: sodium- and chloride-dependent glycine transporter 1-like
RS005241	GO:0015171	amino acid transmembrane transporter activity	MF	-1.24648029	-0.946331544	-0.692073388	4.41212653	12.5745733	0.005653054	0.021933014	PREDICTED: high-affinity choline transporter 1
RS002985	GO:0015171	amino acid transmembrane transporter activity	MF	-0.571114377	-0.476136182	-0.859409917	5.548946497	12.07709757	0.007123669	0.026732864	PREDICTED: Y+L amino acid transporter 2
RS003950	GO:0015171	amino acid transmembrane transporter activity	MF	-0.587671115	-0.868990799	-0.714405964	4.844171479	10.0941278	0.017782729	0.057765203	PREDICTED: cationic amino acid transporter 2
RS009139	GO:0015171	amino acid transmembrane transporter activity	MF	0.649351007	1.292845053	0.336142374	2.039377777	9.031613663	0.028873505	0.085540561	PREDICTED: Y+L amino acid transporter 2
RS007859	GO:0015171	amino acid transmembrane transporter activity	MF	-1.037355681	-0.953164057	-1.137934678	2.631254112	8.895408464	0.0307143	0.08987358	PREDICTED: proton-coupled amino acid transporter 4
RS006017	GO:0015171	amino acid transmembrane transporter activity	MF	-0.226333875	0.829010789	0.173010283	3.457042927	8.42538268	0.037991671	0.106302401	PREDICTED: cationic amino acid transporter 2
RS006810	GO:0015171	amino acid transmembrane transporter activity	MF	-0.692918241	-0.709642301	-0.469276201	5.552917252	7.276110601	0.063598968	0.159523084	PREDICTED: Y+L amino acid transporter 2
RS009471	GO:0015171	amino acid transmembrane transporter activity	MF	0.454440582	0.068570398	0.92902968	1.975531925	6.680170813	0.082821497	0.194672252	PREDICTED: potassium voltage-gated channel protein Shal
RS006076	GO:0015171	amino acid transmembrane transporter activity	MF	-0.399500112	-0.234112198	-0.791572834	3.218719557	5.135126595	0.162165734	0.319411206	PREDICTED: cationic amino acid transporter 4
RS007790	GO:0015171	amino acid transmembrane transporter activity	MF	0.13034852	0.225712601	0.386180211	4.625198528	2.606288996	0.456388071	0.628856824	PREDICTED: mitochondrial glutamate carrier 1-like
RS004471	GO:0015171	amino acid transmembrane transporter activity	MF	0.302505199	0.1016203	-0.272900349	3.077100879	2.396738775	0.494241988	0.660492741	PREDICTED: Y+L amino acid transporter 2
RS002882	GO:0015171	amino acid transmembrane transporter activity	MF	0.362043543	0.12287268	0.14951259	3.567153224	1.277605364	0.734457649	0.842342193	PREDICTED: putative sodium-coupled neutral amino acid transporter 7
RS013172	GO:0015171	amino acid transmembrane transporter activity	MF	0.064707769	0.050177661	0.256276698	4.54520608	0.870549691	0.832528393	0.905630734	PREDICTED: N-acetyl-D-glucosamine kinase
RS006019	GO:0015171	amino acid transmembrane transporter activity	MF	0.016800687	-0.569398535	-0.206623396	0.539126613	0.775635116	0.855285471	0.920559373	PREDICTED: cationic amino acid transporter 2
RS014940	GO:0015171	amino acid transmembrane transporter activity	MF	0.00473671	-0.350880149	-0.354295712	0.736422598	0.582482205	0.900430699	0.946555742	PREDICTED: potassium voltage-gated channel protein Shal
RS004110	GO:0015171	amino acid transmembrane transporter activity	MF	-0.109326082	-0.245764423	-0.393853677	2.856144774	0.536964028	0.910698682	0.95276119	PREDICTED: phospholipase A2 inhibitor and Ly6/PLAUR domain-containing protein
RS003228	GO:0008514	organic anion transmembrane transporter activity	MF	-0.561103954	2.12048168	1.83683218	5.406987214	114.6440514	1.09829E-24	3.71824E-23	PREDICTED: b(0,+)-type amino acid transporter 1-like
RS013974	GO:0008514	organic anion transmembrane transporter activity	MF	1.308057058	2.315023189	0.876327879	6.127768048	111.7248777	4.66769E-24	1.53887E-22	PREDICTED: monocarboxylate transporter 10
RS012045	GO:0008514	organic anion transmembrane transporter activity	MF	-2.417547613	-3.846942398	-2.948472949	3.138267013	95.12499238	1.73561E-20	4.58246E-19	PREDICTED: sodium-independent sulfate anion transporter
RS014732	GO:0008514	organic anion transmembrane transporter activity	MF	-2.171879817	-1.24728548	0.09932889	5.367265273	82.18961662	1.04062E-17	2.21004E-16	PREDICTED: solute carrier organic anion transporter family member 2A1
RS006315	GO:0008514	organic anion transmembrane transporter activity	MF	-2.445857294	-4.975199992	0.147442902	3.069628072	71.7469443	1.80352E-15	3.24017E-14	PREDICTED: bumetanide-sensitive sodium-(potassium)-chloride cotransporter
RS008617	GO:0008514	organic anion transmembrane transporter activity	MF	-1.758654905	-1.933395291	0.119722157	4.240860888	52.46640405	2.38231E-11	3.09308E-10	PREDICTED: proton-coupled amino acid transporter 1
RS006021	GO:0008514	organic anion transmembrane transporter activity	MF	-1.978389488	-0.987147201	0.23457041	5.767022167	52.1310105	2.80858E-11	3.63527E-10	PREDICTED: cationic amino acid transporter 3
RS009380	GO:0008514	organic anion transmembrane transporter activity	MF	1.181487238	0.230316163	-1.011984835	4.090504935	45.23692145	8.23976E-10	9.50289E-09	PREDICTED: b(0,+)-type amino acid transporter 1-like
RS001007	GO:0008514	organic anion transmembrane transporter activity	MF	-0.057453873	2.550863666	4.593751253	0.34449975	41.36132174	5.4813E-09	5.84023E-08	PREDICTED: sodium-dependent nutrient amino acid transporter 1-like

RS005851	GO:0008514	organic anion transmembrane transporter activity	MF	0.526397294	1.41413322	1.673681755	7.829535198	40.74316203	7.413E-09	7.77347E-08	PREDICTED: proton-coupled amino acid transporter 4
RS006814	GO:0008514	organic anion transmembrane transporter activity	MF	-0.514796382	1.014347124	0.975336891	4.041760153	30.97725487	8.59474E-07	7.23543E-06	PREDICTED: large neutral amino acids transporter small subunit 2
RS006320	GO:0008514	organic anion transmembrane transporter activity	MF	1.668458777	1.549661536	0.932473469	6.183206129	30.51118393	1.07731E-06	8.96741E-06	PREDICTED: bumetanide-sensitive sodium-(potassium)-chloride cotransporter
RS000193	GO:0008514	organic anion transmembrane transporter activity	MF	-0.612906575	0.552707825	0.864925878	6.395375097	30.19397061	1.25629E-06	1.03772E-05	PREDICTED: b(0,+)-type amino acid transporter 1
RS012462	GO:0008514	organic anion transmembrane transporter activity	MF	-1.887439376	-1.068323927	-1.822700455	2.935725927	21.16526065	9.72757E-05	0.000593839	PREDICTED: synaptic vesicle glycoprotein 2B
RS004393	GO:0008514	organic anion transmembrane transporter activity	MF	-1.227790861	-2.264108903	-0.817189459	2.289519889	20.23673107	0.000151606	0.000890546	PREDICTED: putative tricarboxylate transport protein, mitochondrial
RS000837	GO:0008514	organic anion transmembrane transporter activity	MF	-1.260815808	-0.582381283	0.014148923	5.012164404	17.85146996	0.000471988	0.002476758	PREDICTED: excitatory amino acid transporter 1-like
RS000849	GO:0008514	organic anion transmembrane transporter activity	MF	-0.753175709	-1.571391853	-1.058453667	2.708513766	17.06922787	0.000683946	0.003464852	PREDICTED: monocarboxylate transporter 9-like
RS000927	GO:0008514	organic anion transmembrane transporter activity	MF	-2.570527298	-2.804707147	-0.755163456	0.667619183	16.35029216	0.000961058	0.004715063	PREDICTED: b(0,+)-type amino acid transporter 1-like
RS013870	GO:0008514	organic anion transmembrane transporter activity	MF	-1.185159015	-0.789079424	-0.942978819	4.024142004	15.86729932	0.001207277	0.005765813	PREDICTED: sodium- and chloride-dependent glycine transporter 1-like
RS000928	GO:0008514	organic anion transmembrane transporter activity	MF	-2.818387407	-2.387215456	-1.195316578	0.324906115	15.29049888	0.001584497	0.007309578	B(0,+)-type amino acid transporter 1
RS005708	GO:0008514	organic anion transmembrane transporter activity	MF	-2.57707626	-0.767810406	-0.121337824	1.208165742	14.61680361	0.002175202	0.009598631	PREDICTED: sodium- and chloride-dependent glycine transporter 1-like
RS008723	GO:0008514	organic anion transmembrane transporter activity	MF	0.765724899	0.492155106	-0.726953396	2.604372289	14.25219643	0.002581214	0.011140444	PREDICTED: excitatory amino acid transporter
RS005241	GO:0008514	organic anion transmembrane transporter activity	MF	-1.24648029	-0.946331544	-0.692073388	4.41212653	12.5745733	0.005653054	0.021933014	PREDICTED: high-affinity choline transporter 1
RS002985	GO:0008514	organic anion transmembrane transporter activity	MF	-0.571114377	-0.476136182	-0.859409917	5.548946497	12.07709757	0.007123669	0.026732864	PREDICTED: Y+L amino acid transporter 2
RS003950	GO:0008514	organic anion transmembrane transporter activity	MF	-0.587671115	-0.868990799	-0.714405964	4.844171479	10.0941278	0.017782729	0.057765203	PREDICTED: cationic amino acid transporter 2
RS009139	GO:0008514	organic anion transmembrane transporter activity	MF	0.649351007	1.292845053	0.336142374	2.039377777	9.031613663	0.028873505	0.085540561	PREDICTED: Y+L amino acid transporter 2
RS007859	GO:0008514	organic anion transmembrane transporter activity	MF	-1.037355681	-0.953164057	-1.137934678	2.631254112	8.895408464	0.0307143	0.08987358	PREDICTED: proton-coupled amino acid transporter 4
RS006017	GO:0008514	organic anion transmembrane transporter activity	MF	-0.226333875	0.829010789	0.173010283	3.457042927	8.42538268	0.037991671	0.106302401	PREDICTED: cationic amino acid transporter 2
RS012784	GO:0008514	organic anion transmembrane transporter activity	MF	-0.964188065	-1.053670411	-1.487092291	6.693074429	8.119987423	0.043595935	0.118762068	PREDICTED: excitatory amino acid transporter-like
RS006810	GO:0008514	organic anion transmembrane transporter activity	MF	-0.692918241	-0.709642301	-0.469276201	5.552917252	7.276110601	0.063598968	0.159523084	PREDICTED: Y+L amino acid transporter 2
RS015105	GO:0008514	organic anion transmembrane transporter activity	MF	-0.678666411	-2.224992259	-0.562865466	0.559101899	7.185153044	0.066224707	0.164438873	PREDICTED: organic cation transporter 1
RS009471	GO:0008514	organic anion transmembrane transporter activity	MF	0.454440582	0.068570398	0.92902968	1.975531925	6.680170813	0.082821497	0.194672252	PREDICTED: potassium voltage-gated channel protein Shal
RS012532	GO:0008514	organic anion transmembrane transporter activity	MF	0.52422059	0.27169055	0.170626759	5.950381512	5.527522768	0.137001687	0.282351902	PREDICTED: adenosine 3'-phospho 5'-phosphosulfate transporter 2
RS002769	GO:0008514	organic anion transmembrane transporter activity	MF	-0.180596666	-0.510025269	-0.494314256	5.266272897	5.147923967	0.161280396	0.318264698	PREDICTED: mitochondrial 2-oxoglutarate/malate carrier protein
RS006076	GO:0008514	organic anion transmembrane transporter activity	MF	-0.399500112	-0.234112198	-0.791572834	3.218719557	5.135126595	0.162165734	0.319411206	PREDICTED: cationic amino acid transporter 4
RS000049	GO:0008514	organic anion transmembrane transporter activity	MF	-0.167016261	-0.078808593	0.315739518	4.19490186	3.918242209	0.270428905	0.452114912	PREDICTED: protein I'm not dead yet-like
RS007790	GO:0008514	organic anion transmembrane transporter activity	MF	0.13034852	0.225712601	0.386180211	4.625198528	2.606288996	0.456388071	0.628856824	PREDICTED: mitochondrial glutamate carrier 1-like
RS004471	GO:0008514	organic anion transmembrane transporter activity	MF	0.302505199	0.1016203	-0.272900349	3.077100879	2.396738775	0.494241988	0.660492741	PREDICTED: Y+L amino acid transporter 2
RS002882	GO:0008514	organic anion transmembrane transporter activity	MF	0.362043543	0.12287268	0.14951259	3.567153224	1.277605364	0.734457649	0.842342193	PREDICTED: putative sodium-coupled neutral amino acid transporter 7
RS013172	GO:0008514	organic anion transmembrane transporter activity	MF	0.064707769	0.050177661	0.256276698	4.54520608	0.870549691	0.832528393	0.905630734	PREDICTED: N-acetyl-D-glucosamine kinase
RS006019	GO:0008514	organic anion transmembrane transporter activity	MF	0.016800687	-0.569398535	-0.206623396	0.539126613	0.775635116	0.855285471	0.920559373	PREDICTED: cationic amino acid transporter 2
RS014940	GO:0008514	organic anion transmembrane transporter activity	MF	0.00473671	-0.350880149	-0.354295712	0.736422598	0.582482205	0.900430699	0.946555742	PREDICTED: potassium voltage-gated channel protein Shal
RS004110	GO:0008514	organic anion transmembrane transporter activity	MF	-0.109326082	-0.245764423	-0.393853677	2.856144774	0.536964028	0.910698682	0.95276119	PREDICTED: phospholipase A2 inhibitor and Ly6/PLAUR domain-containing protein
RS002585	GO:0008514	organic anion transmembrane transporter activity	MF	0.061337763	0.015683494	0.119579633	4.655608643	0.205533408	0.976691112	0.987602807	PREDICTED: mitochondrial pyruvate carrier 1
RS003228	GO:0005342	organic acid transmembrane transporter activity	MF	-0.561103954	2.12048168	1.83683218	5.406987214	114.6440514	1.09829E-24	3.71824E-23	PREDICTED: b(0,+)-type amino acid transporter 1-like
RS013974	GO:0005342	organic acid transmembrane transporter activity	MF	1.308057058	2.315023189	0.876327879	6.127768048	111.7248777	4.66769E-24	1.53887E-22	PREDICTED: monocarboxylate transporter 10
RS012045	GO:0005342	organic acid transmembrane transporter activity	MF	-2.417547613	-3.846942398	-2.948472949	3.138267013	95.12499238	1.73561E-20	4.58246E-19	PREDICTED: sodium-independent sulfate anion transporter
RS006315	GO:0005342	organic acid transmembrane transporter activity	MF	-2.445857294	-4.975199992	0.147442902	3.069628072	71.7469443	1.80352E-15	3.24017E-14	PREDICTED: bumetanide-sensitive sodium-(potassium)-chloride cotransporter
RS008617	GO:0005342	organic acid transmembrane transporter activity	MF	-1.758654905	-1.933395291	0.119722157	4.240860888	52.46640405	2.38231E-11	3.09308E-10	PREDICTED: proton-coupled amino acid transporter 1
RS006021	GO:0005342	organic acid transmembrane transporter activity	MF	-1.978389488	-0.987147201	0.23457041	5.767022167	52.1310105	2.80858E-11	3.63527E-10	PREDICTED: cationic amino acid transporter 3
RS009380	GO:0005342	organic acid transmembrane transporter activity	MF	1.181487238	0.230316163	-1.011984835	4.090504935	45.23692145	8.23976E-10	9.50289E-09	PREDICTED: b(0,+)-type amino acid transporter 1-like
RS001007	GO:0005342	organic acid transmembrane transporter activity	MF	-0.057453873	2.550863666	4.593751253	0.34449975	41.36132174	5.4813E-09	5.84023E-08	PREDICTED: sodium-dependent nutrient amino acid transporter 1-like
RS005851	GO:0005342	organic acid transmembrane transporter activity	MF	0.526397294	1.41413322	1.673681755	7.829535198	40.74316203	7.413E-09	7.77347E-08	PREDICTED: proton-coupled amino acid transporter 4
RS006814	GO:0005342	organic acid transmembrane transporter activity	MF	-0.514796382	1.014347124	0.975336891	4.041760153	30.97725487	8.59474E-07	7.23543E-06	PREDICTED: large neutral amino acids transporter small subunit 2
RS006320	GO:0005342	organic acid transmembrane transporter activity	MF	1.668458777	1.549661536	0.932473469	6.183206129	30.51118393	1.07731E-06	8.96741E-06	PREDICTED: bumetanide-sensitive sodium-(potassium)-chloride cotransporter
RS000193	GO:0005342	organic acid transmembrane transporter activity	MF	-0.612906575	0.552707825	0.864925878	6.395375097	30.19397061	1.25629E-06	1.03772E-05	PREDICTED: b(0,+)-type amino acid transporter 1
RS014340	GO:0005342	organic acid transmembrane transporter activity	MF	-0.92887959	-0.092715464	0.219765138	5.620832256	22.82606695	4.38983E-05	0.00028483	PREDICTED: ileal sodium/bile acid cotransporter-like
RS004393	GO:0005342	organic acid transmembrane transporter activity	MF	-1.227790861	-2.264108903	-0.817189459	2.289519889	20.23673107	0.000151606	0.000890546	PREDICTED: putative tricarboxylate transport protein, mitochondrial
RS000837	GO:0005342	organic acid transmembrane transporter activity	MF	-1.260815808	-0.582381283	0.014148923	5.012164404	17.85146996	0.000471988	0.002476758	PREDICTED: excitatory amino acid transporter 1-like
RS000849	GO:0005342	organic acid transmembrane transporter activity	MF	-0.753175709	-1.571391853	-1.058453667	2.708513766	17.06922787	0.000683946	0.003464852	PREDICTED: monocarboxylate transporter 9-like
RS000927	GO:0005342	organic acid transmembrane transporter activity	MF	-2.570527298	-2.804707147	-0.755163456	0.667619183	16.35029216	0.000961058	0.004715063	PREDICTED: b(0,+)-type amino acid transporter 1-like
RS013870	GO:0005342	organic acid transmembrane transporter activity	MF	-1.185159015	-0.789079424	-0.942978819	4.024142004	15.86729932	0.001207277	0.005765813	PREDICTED: sodium- and chloride-dependent glycine transporter 1-like
RS000928	GO:0005342	organic acid transmembrane transporter activity	MF	-2.818387407	-2.387215456	-1.195316578	0.324906115	15.29049888	0.001584497	0.007309578	B(0,+)-type amino acid transporter 1
RS005708	GO:0005342	organic acid transmembrane transporter activity	MF	-2.57707626	-0.767810406	-0.121337824	1.208165742	14.61680361	0.002175202	0.009598631	PREDICTED: sodium- and chloride-dependent glycine transporter 1-like
RS008723	GO:0005342	organic acid transmembrane transporter activity	MF	0.765724899	0.492155106	-0.726953396	2.604372289	14.25219643	0.002581214	0.011140444	PREDICTED: excitatory amino acid transporter
RS005241	GO:0005342	organic acid transmembrane transporter activity	MF	-1.24648029	-0.946331544	-0.692073388	4.41212653	12.5745733	0.005653054	0.021933014	PREDICTED: high-affinity choline transporter 1
RS002985	GO:0005342	organic acid transmembrane transporter activity	MF	-0.571114377	-0.476136182	-0.859409917	5.548946497	12.07709757	0.007123669	0.026732864	PREDICTED: Y+L amino acid transporter 2
RS003950	GO:0005342	organic acid transmembrane transporter activity	MF	-0.587671115	-0.868990799	-0.714405964	4.844171479	10.0941278	0.017782729	0.057765203	PREDICTED: cationic amino acid transporter 2
RS009139	GO:0005342	organic acid transmembrane transporter activity	MF	0.649351007	1.292845053	0.336142374	2.039377777	9.031613663	0.028873505	0.085540561	PREDICTED: Y+L amino acid transporter 2
RS007859	GO:0005342	organic acid transmembrane transporter activity	MF	-1.037355681	-0.953164057	-1.137934678	2.631254112	8.895408464	0.0307143	0.08987358	PREDICTED: proton-coupled amino acid transporter 4
RS006017	GO:0005342	organic acid transmembrane transporter activity	MF	-0.226333875	0.829010789	0.173010283	3.457042927	8.42538268	0.037991671	0.106302401	PREDICTED: cationic amino acid transporter 2
RS012784	GO:0005342	organic acid transmembrane transporter activity	MF	-0.964188065	-1.053670411	-1.487092291	6.693074429	8.119987423	0.043595935	0.118762068	PREDICTED: excitatory amino acid transporter-like
RS006810	GO:0005342	organic acid transmembrane transporter activity	MF	-0.692918241	-0.709642301	-0.469276201	5.552917252	7.276110601	0.063598968	0.159523084	PREDICTED: Y+L amino acid transporter 2
RS015105	GO:0005342	organic acid transmembrane transporter activity	MF	-0.678666411	-2.224992259	-0.562865466	0.559101899	7.185153044	0.066224707	0.164438873	PREDICTED: organic cation transporter 1
RS009471	GO:0005342	organic acid transmembrane transporter activity	MF	0.454440582	0.068570398	0.92902968	1.975531925	6.680170813	0.082821497	0.194672252	PREDICTED: potassium voltage-gated channel protein Shal
RS002769	GO:0005342	organic acid transmembrane transporter activity	MF	-0.180596666	-0.510025269	-0.494314256	5.266272897	5.147923967	0.161280396	0.318264698	PREDICTED: mitochondrial 2-oxoglutarate/malate carrier protein
RS006076	GO:0005342	organic acid transmembrane transporter activity	MF	-0.399500112	-0.234112198	-0.791572834	3.218719557	5.135126595	0.162165734	0.319411206	PREDICTED: cationic amino acid transporter 4
RS000049	GO:0005342	organic acid transmembrane transporter activity	MF	-0.167016261	-0.078808593	0.315739518	4.19490186	3.918242209	0.270428905	0.452114912	PREDICTED: protein I'm not dead yet-like
RS007790	GO:0005342	organic acid transmembrane transporter activity	MF	0.13034852	0.225712601	0.386180211	4.625198528	2.606288996	0.456388071	0.628856824	PREDICTED: mitochondrial glutamate carrier 1-like
RS004471	GO:0005342	organic acid transmembrane transporter activity	MF	0.302505199	0.1016203	-0.272900349	3.077100879	2.396738775	0.494241988	0.660492741	PREDICTED: Y+L amino acid transporter 2
RS002882	GO:0005342	organic acid transmembrane transporter activity	MF	0.362043543	0.12287268	0.14951259	3.567153224	1.277605364	0.734457649	0.842342193	PREDICTED: putative sodium-coupled neutral amino acid transporter 7
RS013172	GO:0005342	organic acid transmembrane transporter activity	MF	0.064707769	0.050177661	0.256276698	4.54520608	0.870549691	0.832528393	0.905630734	PREDICTED: N-acetyl-D-glucosamine kinase
RS006019	GO:0005342	organic acid transmembrane transporter activity	MF	0.016800687	-0.569398535	-0.206623396	0.539126613	0.775635116	0.855285471	0.920559373	PREDICTED: cationic amino acid transporter 2
RS014940	GO:0005342	organic acid transmembrane transporter activity	MF	0.00473671	-0.350880149	-0.354295712	0.736422598	0.582482205	0.900430699	0.946555742	PREDICTED: potassium voltage-gated channel protein Shal
RS004110	GO:0005342	organic acid transmembrane transporter activity	MF	-0.109326082	-0.245764423	-0.393853677	2.856144774	0.536964028	0.910698682	0.95276119	PREDICTED: phospholipase A2 inhibitor and Ly6/PLAUR domain-containing protein
RS002585	GO:0005342	organic acid transmembrane transporter activity	MF	0.061337763	0.015683494	0.119579633	4.655608643	0.205533408	0.976691112	0.987602807	PREDICTED: mitochondrial pyruvate carrier 1
RS003228	GO:0046943	carboxylic acid transmembrane transporter activity	MF	-0.561103954	2.12048168	1.83683218	5.406987214	114.6440514	1.09829E-24	3.71824E-23	PREDICTED: b(0,+)-type amino acid transporter 1-like
RS013974	GO:0046943	carboxylic acid transmembrane transporter activity	MF	1.308057058	2.315023189	0.876327879	6.127768048	111.7248777	4.66769E-24	1.53887E-22	PREDICTED: monocarboxylate transporter 10
RS012045	GO:0046943	carboxylic acid transmembrane transporter activity	MF	-2.417547613	-3.846942398	-2.948472949	3.138267013	95.12499238	1.73561E-20	4.58246E-19	PREDICTED: sodium-independent sulfate anion transporter
RS006315	GO:0046943	carboxylic acid transmembrane transporter activity	MF	-2.445857294	-4.975199992	0.147442902	3.069628072	71.7469443	1.80352E-15	3.24017E-14	PREDICTED: bumetanide-sensitive sodium-(potassium)-chloride cotransporter
RS008617	GO:0046943	carboxylic acid transmembrane transporter activity	MF	-1.758654905	-1.933395291	0.119722157	4.240860888	52.46640405	2.38231E-11	3.09308E-10	PREDICTED: proton-coupled amino acid transporter 1
RS006021	GO:0046943	carboxylic acid transmembrane transporter activity	MF	-1.978389488	-0.987147201	0.23457041	5.767022167	52.1310105	2.80858E-11	3.63527E-10	PREDICTED: cationic amino acid transporter 3
RS009380	GO:0046943	carboxylic acid transmembrane transporter activity	MF	1.181487238	0.230316163	-1.011984835	4.090504935	45.23692145	8.23976E-10	9.50289E-09	PREDICTED: b(0,+)-type amino acid transporter 1-like
RS001007	GO:0046943	carboxylic acid transmembrane transporter activity	MF	-0.057453873	2.550863666	4.593751253	0.34449975	41.36132174	5.4813E-09	5.84023E-08	PREDICTED: sodium-dependent nutrient amino acid transporter 1-like
RS005851	GO:0046943	carboxylic acid transmembrane transporter activity	MF	0.526397294	1.41413322	1.673681755	7.829535198	40.74316203	7.413E-09	7.77347E-08	PREDICTED: proton-coupled amino acid transporter 4
RS006814	GO:0046943	carboxylic acid transmembrane transporter activity	MF	-0.514796382	1.014347124	0.975336891	4.041760153	30.97725487	8.59474E-07	7.23543E-06	PREDICTED: large neutral amino acids transporter small subunit 2
RS006320	GO:0046943	carboxylic acid transmembrane transporter activity	MF	1.668458777	1.549661536	0.932473469	6.183206129	30.51118393	1.07731E-06	8.96741E-06	PREDICTED: bumetanide-sensitive sodium-(potassium)-chloride cotransporter
RS000193	GO:0046943	carboxylic acid transmembrane transporter activity	MF	-0.612906575	0.552707825	0.864925878	6.395375097	30.19397061	1.25629E-06	1.03772E-05	PREDICTED: b(0,+)-type amino acid transporter 1
RS004393	GO:0046943	carboxylic acid transmembrane transporter activity	MF	-1.227790861	-2.264108903	-0.817189459	2.289519889	20.23673107	0.000151606	0.000890546	PREDICTED: putative tricarboxylate transport protein, mitochondrial
RS000837	GO:0046943	carboxylic acid transmembrane transporter activity	MF	-1.260815808	-0.582381283	0.014148923	5.012164404	17.85146996	0.000471988	0.002476758	PREDICTED: excitatory amino acid transporter 1-like
RS000849	GO:0046943	carboxylic acid transmembrane transporter activity	MF	-0.753175709	-1.571391853	-1.058453667	2.708513766	17.06922787	0.000683946	0.003464852	PREDICTED: monocarboxylate transporter 9-like
RS000927	GO:0046943	carboxylic acid transmembrane transporter activity	MF	-2.570527298	-2.804707147	-0.755163456	0.667619183	16.35029216	0.000961058	0.004715063	PREDICTED: b(0,+)-type amino acid transporter 1-like
RS013870	GO:0046943	carboxylic acid transmembrane transporter activity	MF	-1.185159015	-0.789079424	-0.942978819	4.024142004	15.86729932	0.001207277	0.005765813	PREDICTED: sodium- and chloride-dependent glycine transporter 1-like
RS000928	GO:0046943	carboxylic acid transmembrane transporter activity	MF	-2.818387407	-2.387215456	-1.195316578	0.324906115	15.29049888	0.001584497	0.007309578	B(0,+)-type amino acid transporter 1
RS005708	GO:0046943	carboxylic acid transmembrane transporter activity	MF	-2.57707626	-0.767810406	-0.121337824	1.208165742	14.61680361	0.002175202	0.009598631	PREDICTED: sodium- and chloride-dependent glycine transporter 1-like
RS008723	GO:0046943	carboxylic acid transmembrane transporter activity	MF	0.765724899	0.492155106	-0.726953396	2.604372289	14.25219643	0.002581214	0.011140444	PREDICTED: excitatory amino acid transporter
RS005241	GO:0046943	carboxylic acid transmembrane transporter activity	MF	-1.24648029	-0.946331544	-0.692073388	4.41212653	12.5745733	0.005653054	0.021933014	PREDICTED: high-affinity choline transporter 1
RS002985	GO:0046943	carboxylic acid transmembrane transporter activity	MF	-0.571114377	-0.476136182	-0.859409917	5.548946497	12.07709757	0.007123669	0.026732864	PREDICTED: Y+L amino acid transporter 2
RS003950	GO:0046943	carboxylic acid transmembrane transporter activity	MF	-0.587671115	-0.868990799	-0.714405964	4.844171479	10.0941278	0.017782729	0.057765203	PREDICTED: cationic amino acid transporter 2
RS009139	GO:0046943	carboxylic acid transmembrane transporter activity	MF	0.649351007	1.292845053	0.336142374	2.039377777	9.031613663	0.028873505	0.085540561	PREDICTED: Y+L amino acid transporter 2
RS007859	GO:0046943	carboxylic acid transmembrane transporter activity	MF	-1.037355681	-0.953164057	-1.137934678	2.631254112	8.895408464	0.0307143	0.08987358	PREDICTED: proton-coupled amino acid transporter 4
RS006017	GO:0046943	carboxylic acid transmembrane transporter activity	MF	-0.226333875	0.829010789	0.173010283	3.457042927	8.42538268	0.037991671	0.106302401	PREDICTED: cationic amino acid transporter 2
RS012784	GO:0046943	carboxylic acid transmembrane transporter activity	MF	-0.964188065	-1.053670411	-1.487092291	6.693074429	8.119987423	0.043595935	0.118762068	PREDICTED: excitatory amino acid transporter-like
RS006810	GO:0046943	carboxylic acid transmembrane transporter activity	MF	-0.692918241	-0.709642301	-0.469276201	5.552917252	7.276110601	0.063598968	0.159523084	PREDICTED: Y+L amino acid transporter 2
RS015105	GO:0046943	carboxylic acid transmembrane transporter activity	MF	-0.678666411	-2.224992259	-0.562865466	0.559101899	7.185153044	0.066224707	0.164438873	PREDICTED: organic cation transporter 1
RS009471	GO:0046943	carboxylic acid transmembrane transporter activity	MF	0.454440582	0.068570398	0.92902968	1.975531925	6.680170813	0.082821497	0.194672252	PREDICTED: potassium voltage-gated channel protein Shal
RS002769	GO:0046943	carboxylic acid transmembrane transporter activity	MF	-0.180596666	-0.510025269	-0.494314256	5.266272897	5.147923967	0.161280396	0.318264698	PREDICTED: mitochondrial 2-oxoglutarate/malate carrier protein
RS006076	GO:0046943	carboxylic acid transmembrane transporter activity	MF	-0.399500112	-0.234112198	-0.791572834	3.218719557	5.135126595	0.162165734	0.319411206	PREDICTED: cationic amino acid transporter 4
RS000049	GO:0046943	carboxylic acid transmembrane transporter activity	MF	-0.167016261	-0.078808593	0.315739518	4.19490186	3.918242209	0.270428905	0.452114912	PREDICTED: protein I'm not dead yet-like
RS007790	GO:0046943	carboxylic acid transmembrane transporter activity	MF	0.13034852	0.225712601	0.386180211	4.625198528	2.606288996	0.456388071	0.628856824	PREDICTED: mitochondrial glutamate carrier 1-like

RS004471	GO:0046943	carboxylic acid transmembrane transporter activity	MF	0.302505199	0.1016203	-0.272900349	3.077100879	2.396738775	0.494241988	0.660492741	PREDICTED: Y+L amino acid transporter 2
RS002882	GO:0046943	carboxylic acid transmembrane transporter activity	MF	0.362043543	0.12287268	0.14951259	3.567153224	1.277605364	0.734457649	0.842342193	PREDICTED: putative sodium-coupled neutral amino acid transporter 7
RS013172	GO:0046943	carboxylic acid transmembrane transporter activity	MF	0.064707769	0.050177661	0.256276698	4.54520608	0.870549691	0.832528393	0.905630734	PREDICTED: N-acetyl-D-glucosamine kinase
RS006019	GO:0046943	carboxylic acid transmembrane transporter activity	MF	0.016800687	-0.569398535	-0.206623396	0.539126613	0.775635116	0.855285471	0.920559373	PREDICTED: cationic amino acid transporter 2
RS014940	GO:0046943	carboxylic acid transmembrane transporter activity	MF	0.00473671	-0.350880149	-0.354295712	0.736422598	0.582482205	0.900430699	0.946555742	PREDICTED: potassium voltage-gated channel protein Shal
RS004110	GO:0046943	carboxylic acid transmembrane transporter activity	MF	-0.109326082	-0.245764423	-0.393853677	2.856144774	0.536964028	0.910698682	0.95276119	PREDICTED: phospholipase A2 inhibitor and Ly6/PLAUR domain-containing protein
RS002585	GO:0046943	carboxylic acid transmembrane transporter activity	MF	0.061337763	0.015683494	0.119579633	4.655608643	0.205533408	0.976691112	0.987602807	PREDICTED: mitochondrial pyruvate carrier 1
RS007652	GO:0015291	secondary active transmembrane transporter activity	MF	0.585883623	0.528505008	4.851177054	4.584889963	291.2117195	7.93792E-63	8.92591E-61	PREDICTED: solute carrier family 26 member 10-like
RS004606	GO:0015291	secondary active transmembrane transporter activity	MF	-0.152775887	3.199032258	1.582974114	7.379011967	144.4003621	4.25168E-31	1.83376E-29	PREDICTED: peptide transporter family 1
RS003228	GO:0015291	secondary active transmembrane transporter activity	MF	-0.561103954	2.12048168	1.83683218	5.406987214	114.6440514	1.09829E-24	3.71824E-23	PREDICTED: b(0,+)-type amino acid transporter 1-like
RS013974	GO:0015291	secondary active transmembrane transporter activity	MF	1.308057058	2.315023189	0.876327879	6.127768048	111.7248777	4.66769E-24	1.53887E-22	PREDICTED: monocarboxylate transporter 10
RS012045	GO:0015291	secondary active transmembrane transporter activity	MF	-2.417547613	-3.846942398	-2.948472949	3.138267013	95.12499238	1.73561E-20	4.58246E-19	PREDICTED: sodium-independent sulfate anion transporter
RS014732	GO:0015291	secondary active transmembrane transporter activity	MF	-2.171879817	-1.24728548	0.09932889	5.367265273	82.18961662	1.04062E-17	2.21004E-16	PREDICTED: solute carrier organic anion transporter family member 2A1
RS015390	GO:0015291	secondary active transmembrane transporter activity	MF	-1.260588417	-0.88728058	1.174441311	5.643186003	81.54774944	1.42892E-17	3.01438E-16	PREDICTED: sodium-independent sulfate anion transporter
RS002366	GO:0015291	secondary active transmembrane transporter activity	MF	-2.013469227	2.285528889	0.940362283	2.009763796	76.37983445	1.83374E-16	3.57494E-15	PREDICTED: sodium-dependent serotonin transporter
RS006315	GO:0015291	secondary active transmembrane transporter activity	MF	-2.445857294	-4.975199992	0.147442902	3.069628072	71.7469443	1.80352E-15	3.24017E-14	PREDICTED: bumetanide-sensitive sodium-(potassium)-chloride cotransporter
RS001006	GO:0015291	secondary active transmembrane transporter activity	MF	-1.308055843	0.748595123	2.011362686	7.151837524	64.31436805	7.03086E-14	1.12512E-12	PREDICTED: sodium-dependent nutrient amino acid transporter 1-like
RS007831	GO:0015291	secondary active transmembrane transporter activity	MF	-0.285689079	2.23184291	-0.54998832	3.347851609	60.46667692	4.67238E-13	7.00523E-12	PREDICTED: sodium- and chloride-dependent GABA transporter ine-like
RS010878	GO:0015291	secondary active transmembrane transporter activity	MF	-0.202147251	0.219397642	1.259334773	5.8954575	54.42606082	9.10217E-12	1.23527E-10	PREDICTED: band 3 anion transport protein
RS009380	GO:0015291	secondary active transmembrane transporter activity	MF	1.181487238	0.230316163	-1.011984835	4.090504935	45.23692145	8.23976E-10	9.50289E-09	PREDICTED: b(0,+)-type amino acid transporter 1-like
RS009578	GO:0015291	secondary active transmembrane transporter activity	MF	-2.77838182	-2.247321578	-2.454512864	4.019688809	42.39569844	3.30671E-09	3.59937E-08	PREDICTED: solute carrier family 28 member 3
RS001007	GO:0015291	secondary active transmembrane transporter activity	MF	-0.057453873	2.550863666	4.593751253	0.34449975	41.36132174	5.4813E-09	5.84023E-08	PREDICTED: sodium-dependent nutrient amino acid transporter 1-like
RS009711	GO:0015291	secondary active transmembrane transporter activity	MF	0.862276886	1.272132135	1.421293308	6.056371062	36.7986326	5.07562E-08	4.85732E-07	PREDICTED: tropinone reductase 2-like
RS006320	GO:0015291	secondary active transmembrane transporter activity	MF	1.668458777	1.549661536	0.932473469	6.183206129	30.51118393	1.07731E-06	8.96741E-06	PREDICTED: bumetanide-sensitive sodium-(potassium)-chloride cotransporter
RS000193	GO:0015291	secondary active transmembrane transporter activity	MF	-0.612906575	0.552707825	0.864925878	6.395375097	30.19397061	1.25629E-06	1.03772E-05	PREDICTED: b(0,+)-type amino acid transporter 1
RS009579	GO:0015291	secondary active transmembrane transporter activity	MF	-0.361377028	0.937501921	0.004604885	5.160969196	28.85056945	2.40732E-06	1.91039E-05	PREDICTED: solute carrier family 28 member 3-like
RS013310	GO:0015291	secondary active transmembrane transporter activity	MF	-1.594173173	0.30942411	0.502900611	3.784418211	25.32704004	1.31907E-05	9.38549E-05	PREDICTED: sodium- and chloride-dependent transporter XTRP3
RS006319	GO:0015291	secondary active transmembrane transporter activity	MF	-1.264071762	-1.378688832	-0.296664377	4.499770473	23.99784591	2.50059E-05	0.000169587	PREDICTED: bumetanide-sensitive sodium-(potassium)-chloride cotransporter
RS011507	GO:0015291	secondary active transmembrane transporter activity	MF	0.081672207	0.439418944	1.152509484	5.150989282	22.16598652	6.02438E-05	0.000382801	PREDICTED: sialin
RS012046	GO:0015291	secondary active transmembrane transporter activity	MF	-0.044612109	1.318321788	-0.182662099	3.873303092	21.0021542	0.000105168	0.000636465	PREDICTED: sodium-independent sulfate anion transporter-like
RS007340	GO:0015291	secondary active transmembrane transporter activity	MF	1.72289566	2.228109689	-0.488523344	0.853368064	20.24210443	0.000151218	0.000888679	PREDICTED: mitochondrial sodium/hydrogen exchanger 9B2
RS004393	GO:0015291	secondary active transmembrane transporter activity	MF	-1.227790861	-2.264108903	-0.817189459	2.289519889	20.23673107	0.000151606	0.000890546	PREDICTED: putative tricarboxylate transport protein, mitochondrial
RS007614	GO:0015291	secondary active transmembrane transporter activity	MF	0.699165628	-0.052782673	-0.817546641	5.472681929	18.94140065	0.000281133	0.001554478	PREDICTED: facilitated trehalose transporter Tret1-like
RS007694	GO:0015291	secondary active transmembrane transporter activity	MF	-1.402205119	-1.429007484	-0.154037672	3.235518975	18.72862507	0.000311092	0.001703432	PREDICTED: proton myo-inositol cotransporter-like
RS009272	GO:0015291	secondary active transmembrane transporter activity	MF	-0.152985688	-1.205701524	-0.53386679	4.590610282	18.71946093	0.000312451	0.001710132	PREDICTED: mitochondrial sodium/hydrogen exchanger 9B2
RS009604	GO:0015291	secondary active transmembrane transporter activity	MF	-1.648345631	0.550771769	0.526514629	2.368500525	18.41833332	0.000360561	0.001945804	PREDICTED: sodium-dependent noradrenaline transporter
RS014410	GO:0015291	secondary active transmembrane transporter activity	MF	-0.538555444	-0.069436224	0.424307658	5.574699261	17.86202393	0.00046963	0.002466436	PREDICTED: sodium- and chloride-dependent neutral and basic amino acid transporter B(0+)
RS000837	GO:0015291	secondary active transmembrane transporter activity	MF	-1.260815808	-0.582381283	0.014148923	5.012164404	17.85146996	0.000471988	0.002476758	PREDICTED: excitatory amino acid transporter 1-like
RS012047	GO:0015291	secondary active transmembrane transporter activity	MF	-1.033691566	-1.204387105	-0.573210977	3.717195265	16.53395031	0.000881135	0.004370625	PREDICTED: sodium-independent sulfate anion transporter-like
RS013870	GO:0015291	secondary active transmembrane transporter activity	MF	-1.185159015	-0.789079424	-0.942978819	4.024142004	15.86729932	0.001207277	0.005765813	PREDICTED: sodium- and chloride-dependent glycine transporter 1-like
RS013164	GO:0015291	secondary active transmembrane transporter activity	MF	-0.720894807	-1.573394185	-0.567112816	2.35012097	15.85215695	0.001215934	0.005800558	PREDICTED: sodium/hydrogen exchanger 3
RS000928	GO:0015291	secondary active transmembrane transporter activity	MF	-2.818387407	-2.387215456	-1.195316578	0.324906115	15.29049888	0.001584497	0.007309578	B(0,+)-type amino acid transporter 1
RS006318	GO:0015291	secondary active transmembrane transporter activity	MF	-3.618949223	-2.702749807	-1.072386578	0.467850565	14.73641507	0.002056334	0.009147816	PREDICTED: bumetanide-sensitive sodium-(potassium)-chloride cotransporter
RS005708	GO:0015291	secondary active transmembrane transporter activity	MF	-2.57707626	-0.767810406	-0.121337824	1.208165742	14.61680361	0.002175202	0.009598631	PREDICTED: sodium- and chloride-dependent glycine transporter 1-like
RS008723	GO:0015291	secondary active transmembrane transporter activity	MF	0.765724899	0.492155106	-0.726953396	2.604372289	14.25219643	0.002581214	0.011140444	PREDICTED: excitatory amino acid transporter
RS012010	GO:0015291	secondary active transmembrane transporter activity	MF	-0.658055692	-0.163236513	0.25753802	6.662027841	13.45566221	0.003748014	0.01543574	PREDICTED: sodium-dependent phosphate transporter 1-B-like
RS009863	GO:0015291	secondary active transmembrane transporter activity	MF	0.247967949	1.994626146	0.840783905	0.741237487	12.85234652	0.004967042	0.019634316	PREDICTED: sodium- and chloride-dependent glycine transporter 1-like
RS005241	GO:0015291	secondary active transmembrane transporter activity	MF	-1.24648029	-0.946331544	-0.692073388	4.41212653	12.5745733	0.005653054	0.021933014	PREDICTED: high-affinity choline transporter 1
RS000227	GO:0015291	secondary active transmembrane transporter activity	MF	-1.32952285	-0.75353739	-1.12184367	1.981758767	12.38833226	0.00616466	0.023605269	PREDICTED: facilitated trehalose transporter Tret1-2 homolog
RS003288	GO:0015291	secondary active transmembrane transporter activity	MF	-0.468141457	-0.663116971	0.331112622	4.523147937	12.02307867	0.007304519	0.027292266	PREDICTED: sodium- and chloride-dependent GABA transporter ine
RS011468	GO:0015291	secondary active transmembrane transporter activity	MF	-0.318737056	-0.110014844	-1.568608212	1.963272184	11.9167036	0.007674014	0.028433815	PREDICTED: solute carrier family 35 member F6
RS009360	GO:0015291	secondary active transmembrane transporter activity	MF	-0.052778092	-0.516301747	0.304429459	6.031942967	11.69868265	0.008489957	0.031037017	PREDICTED: sodium-dependent nutrient amino acid transporter 1
RS002295	GO:0015291	secondary active transmembrane transporter activity	MF	-0.760632417	-0.881718429	-1.336627404	7.31207469	11.50237509	0.009297576	0.033436228	PREDICTED: sodium- and chloride-dependent GABA transporter 1-like
RS012007	GO:0015291	secondary active transmembrane transporter activity	MF	-0.8805953	0.446042174	-0.616249485	2.603532973	10.7020781	0.013450915	0.045771636	PREDICTED: sodium-dependent phosphate transporter 2
RS002046	GO:0015291	secondary active transmembrane transporter activity	MF	-1.040945999	-1.141609481	-1.378037143	3.132734296	9.81352734	0.020219576	0.06404561	PREDICTED: sodium- and chloride-dependent GABA transporter 1-like
RS006809	GO:0015291	secondary active transmembrane transporter activity	MF	-0.069599294	0.161390813	-0.669189997	5.521140821	9.427681054	0.02411329	0.074118459	PREDICTED: sodium bicarbonate cotransporter 3
RS005942	GO:0015291	secondary active transmembrane transporter activity	MF	0.222003058	0.357500804	-0.707071584	4.173704825	9.375192754	0.024696847	0.075474905	PREDICTED: mitochondrial sodium/hydrogen exchanger 9B2-like
RS009928	GO:0015291	secondary active transmembrane transporter activity	MF	-0.835456878	-2.347674561	-0.849585016	1.053053669	9.23428246	0.02633282	0.079385129	PREDICTED: sodium-dependent noradrenaline transporter-like
RS011130	GO:0015291	secondary active transmembrane transporter activity	MF	0.62266454	0.316671327	-0.198111492	8.646709884	8.691352167	0.03368886	0.096889131	PREDICTED: 60 kDa heat shock protein, mitochondrial-like
RS006017	GO:0015291	secondary active transmembrane transporter activity	MF	-0.226333875	0.829010789	0.173010283	3.457042927	8.42538268	0.037991671	0.106302401	PREDICTED: cationic amino acid transporter 2
RS012784	GO:0015291	secondary active transmembrane transporter activity	MF	-0.964188065	-1.053670411	-1.487092291	6.693074429	8.119987423	0.043595935	0.118762068	PREDICTED: excitatory amino acid transporter-like
RS010293	GO:0015291	secondary active transmembrane transporter activity	MF	-1.323536549	-0.537537624	-0.40616185	2.969834016	7.797939139	0.050377598	0.133149101	PREDICTED: sodium/hydrogen exchanger 3
RS012051	GO:0015291	secondary active transmembrane transporter activity	MF	-1.206457775	-0.320139517	-0.400442703	3.93903891	7.774642457	0.050906164	0.13434875	PREDICTED: sodium-independent sulfate anion transporter-like
RS005264	GO:0015291	secondary active transmembrane transporter activity	MF	-1.488549143	-1.308665565	-1.379377385	1.600885014	7.17732657	0.066455483	0.164816926	PREDICTED: sodium-dependent neutral amino acid transporter B(0)AT3
RS012048	GO:0015291	secondary active transmembrane transporter activity	MF	-0.674894361	-1.155560739	-0.486644875	2.649535097	6.691851744	0.082395778	0.193852499	PREDICTED: sodium-independent sulfate anion transporter-like
RS009361	GO:0015291	secondary active transmembrane transporter activity	MF	-0.469548252	-1.030089652	-0.272125463	2.379260128	5.571170404	0.134443268	0.278300217	PREDICTED: sodium- and chloride-dependent glycine transporter 2-like
RS012521	GO:0015291	secondary active transmembrane transporter activity	MF	-0.27911124	0.207963234	0.176143396	5.682101261	5.464162095	0.140797903	0.287847294	PREDICTED: sodium/hydrogen exchanger 8
RS002769	GO:0015291	secondary active transmembrane transporter activity	MF	-0.180596666	-0.510025269	-0.494314256	5.266272897	5.147923967	0.161280396	0.318264698	PREDICTED: mitochondrial 2-oxoglutarate/malate carrier protein
RS006076	GO:0015291	secondary active transmembrane transporter activity	MF	-0.399500112	-0.234112198	-0.791572834	3.218719557	5.135126595	0.162165734	0.319411206	PREDICTED: cationic amino acid transporter 4
RS001012	GO:0015291	secondary active transmembrane transporter activity	MF	-0.050637771	0.65837124	0.220417241	5.028311487	5.034189195	0.169310694	0.329872366	PREDICTED: sodium/calcium exchanger 2
RS007406	GO:0015291	secondary active transmembrane transporter activity	MF	1.53218285	1.416234104	0.234676903	0.509107572	4.915967777	0.17805483	0.341676447	PREDICTED: sodium-independent sulfate anion transporter-like
RS009362	GO:0015291	secondary active transmembrane transporter activity	MF	-0.151734251	-1.379851033	-0.853593219	0.577299208	4.353138426	0.225770076	0.399764452	PREDICTED: sodium- and chloride-dependent glycine transporter 2-like
RS015079	GO:0015291	secondary active transmembrane transporter activity	MF	-1.376295246	0.661290456	0.618943801	0.209343525	4.174647883	0.243212401	0.420568148	PREDICTED: facilitated trehalose transporter Tret1-like
RS000693	GO:0015291	secondary active transmembrane transporter activity	MF	-0.697277451	-0.63859341	-1.105155719	1.668516432	3.761841056	0.288355965	0.47128419	PREDICTED: solute carrier family 12 member 4
RS015453	GO:0015291	secondary active transmembrane transporter activity	MF	-0.09263959	0.415348464	0.277714417	5.686672294	3.355959113	0.33992981	0.521573591	PREDICTED: sodium-dependent phosphate transporter 2
RS007790	GO:0015291	secondary active transmembrane transporter activity	MF	0.13034852	0.225712601	0.386180211	4.625198528	2.606288996	0.456388071	0.628856824	PREDICTED: mitochondrial glutamate carrier 1-like
RS007514	GO:0015291	secondary active transmembrane transporter activity	MF	-0.393644456	0.206862252	-0.369998274	2.851975701	2.440356048	0.48616657	0.653933758	PREDICTED: solute carrier family 26 member 10-like
RS002215	GO:0015291	secondary active transmembrane transporter activity	MF	-0.459893543	-0.373663745	-0.327616751	3.245153068	2.258109459	0.520593488	0.682313913	PREDICTED: CMP-sialic acid transporter 1
RS003602	GO:0015291	secondary active transmembrane transporter activity	MF	-0.963739329	-0.484991816	-0.758608967	0.476696176	1.634124817	0.651677236	0.782683875	PREDICTED: solute carrier family 28 member 3
RS003990	GO:0015291	secondary active transmembrane transporter activity	MF	-0.055368209	0.238070575	0.293320892	4.25914611	1.454595738	0.692788544	0.811066898	PREDICTED: UDP-galactose translocator
RS015498	GO:0015291	secondary active transmembrane transporter activity	MF	0.184679301	0.472371696	0.26432428	2.413896137	1.414695214	0.702093736	0.818113296	PREDICTED: calcium uniporter protein, mitochondrial
RS004705	GO:0015291	secondary active transmembrane transporter activity	MF	-0.430701847	-0.465359916	-0.484712083	3.064343052	1.370281204	0.712515526	0.826890945	PREDICTED: sodium/hydrogen exchanger 7
RS008405	GO:0015291	secondary active transmembrane transporter activity	MF	-0.507380646	-0.391727947	-0.160990784	2.501471485	1.183658987	0.75692617	0.857267824	PREDICTED: solute carrier family 12 member 9
RS004706	GO:0015291	secondary active transmembrane transporter activity	MF	-0.281248638	-0.101038833	-0.415774939	3.347688564	1.047260677	0.789818336	0.878882504	PREDICTED: sodium/hydrogen exchanger 7
RS006075	GO:0015291	secondary active transmembrane transporter activity	MF	-0.226394382	-0.065362369	0.088572196	3.008430896	0.898142308	0.82587609	0.901150881	PREDICTED: acetyl-coenzyme A transporter 1
RS013172	GO:0015291	secondary active transmembrane transporter activity	MF	0.064707769	0.050177661	0.256276698	4.54520608	0.870549691	0.832528393	0.905630734	PREDICTED: N-acetyl-D-glucosamine kinase
RS007404	GO:0015291	secondary active transmembrane transporter activity	MF	0.341548622	0.423696565	0.486833568	3.079113058	0.267214539	0.9660718	0.982215891	PREDICTED: sodium-independent sulfate anion transporter-like
RS002366	GO:0015294	solute:cation symporter activity	MF	-2.013469227	2.285528889	0.940362283	2.009763796	76.37983445	1.83374E-16	3.57494E-15	PREDICTED: sodium-dependent serotonin transporter
RS006315	GO:0015294	solute:cation symporter activity	MF	-2.445857294	-4.975199992	0.147442902	3.069628072	71.7469443	1.80352E-15	3.24017E-14	PREDICTED: bumetanide-sensitive sodium-(potassium)-chloride cotransporter
RS001006	GO:0015294	solute:cation symporter activity	MF	-1.308055843	0.748595123	2.011362686	7.151837524	64.31436805	7.03086E-14	1.12512E-12	PREDICTED: sodium-dependent nutrient amino acid transporter 1-like
RS007831	GO:0015294	solute:cation symporter activity	MF	-0.285689079	2.23184291	-0.54998832	3.347851609	60.46667692	4.67238E-13	7.00523E-12	PREDICTED: sodium- and chloride-dependent GABA transporter ine-like
RS009578	GO:0015294	solute:cation symporter activity	MF	-2.77838182	-2.247321578	-2.454512864	4.019688809	42.39569844	3.30671E-09	3.59937E-08	PREDICTED: solute carrier family 28 member 3
RS001007	GO:0015294	solute:cation symporter activity	MF	-0.057453873	2.550863666	4.593751253	0.34449975	41.36132174	5.4813E-09	5.84023E-08	PREDICTED: sodium-dependent nutrient amino acid transporter 1-like
RS009711	GO:0015294	solute:cation symporter activity	MF	0.862276886	1.272132135	1.421293308	6.056371062	36.7986326	5.07562E-08	4.85732E-07	PREDICTED: tropinone reductase 2-like
RS006320	GO:0015294	solute:cation symporter activity	MF	1.668458777	1.549661536	0.932473469	6.183206129	30.51118393	1.07731E-06	8.96741E-06	PREDICTED: bumetanide-sensitive sodium-(potassium)-chloride cotransporter
RS009579	GO:0015294	solute:cation symporter activity	MF	-0.361377028	0.937501921	0.004604885	5.160969196	28.85056945	2.40732E-06	1.91039E-05	PREDICTED: solute carrier family 28 member 3-like
RS013310	GO:0015294	solute:cation symporter activity	MF	-1.594173173	0.30942411	0.502900611	3.784418211	25.32704004	1.31907E-05	9.38549E-05	PREDICTED: sodium- and chloride-dependent transporter XTRP3
RS006319	GO:0015294	solute:cation symporter activity	MF	-1.264071762	-1.378688832	-0.296664377	4.499770473	23.99784591	2.50059E-05	0.000169587	PREDICTED: bumetanide-sensitive sodium-(potassium)-chloride cotransporter
RS011507	GO:0015294	solute:cation symporter activity	MF	0.081672207	0.439418944	1.152509484	5.150989282	22.16598652	6.02438E-05	0.000382801	PREDICTED: sialin
RS007614	GO:0015294	solute:cation symporter activity	MF	0.699165628	-0.052782673	-0.817546641	5.472681929	18.94140065	0.000281133	0.001554478	PREDICTED: facilitated trehalose transporter Tret1-like
RS007694	GO:0015294	solute:cation symporter activity	MF	-1.402205119	-1.429007484	-0.154037672	3.235518975	18.72862507	0.000311092	0.001703432	PREDICTED: proton myo-inositol cotransporter-like
RS009604	GO:0015294	solute:cation symporter activity	MF	-1.648345631	0.550771769	0.526514629	2.368500525	18.41833332	0.000360561	0.001945804	PREDICTED: sodium-dependent noradrenaline transporter
RS014410	GO:0015294	solute:cation symporter activity	MF	-0.538555444	-0.069436224	0.424307658	5.574699261	17.86202393	0.00046963	0.002466436	PREDICTED: sodium- and chloride-dependent neutral and basic amino acid transporter B(0+)
RS000837	GO:0015294	solute:cation symporter activity	MF	-1.260815808	-0.582381283	0.014148923	5.012164404	17.85146996	0.000471988	0.002476758	PREDICTED: excitatory amino acid transporter 1-like
RS013870	GO:0015294	solute:cation symporter activity	MF	-1.185159015	-0.789079424	-0.942978819	4.024142004	15.86729932	0.001207277	0.005765813	PREDICTED: sodium- and chloride-dependent glycine transporter 1-like
RS006318	GO:0015294	solute:cation symporter activity	MF	-3.618949223	-2.702749807	-1.072386578	0.467850565	14.73641507	0.002056334	0.009147816	PREDICTED: bumetanide-sensitive sodium-(potassium)-chloride cotransporter
RS005708	GO:0015294	solute:cation symporter activity	MF	-2.57707626	-0.767810406	-0.121337824	1.208165742	14.61680361	0.002175202	0.009598631	PREDICTED: sodium- and chloride-dependent glycine transporter 1-like
RS008723	GO:0015294	solute:cation symporter activity	MF	0.765724899	0.492155106	-0.726953396	2.604372289	14.25219643	0.002581214	0.011140444	PREDICTED: excitatory amino acid transporter
RS009863	GO:0015294	solute:cation symporter activity	MF	0.247967949	1.994626146	0.840783905	0.741237487	12.85234652	0.004967042	0.019634316	PREDICTED: sodium- and chloride-dependent glycine transporter 1-like
RS005241	GO:0015294	solute:cation symporter activity	MF	-1.24648029	-0.946331544	-0.692073388	4.41212653	12.5745733	0.005653054	0.021933014	PREDICTED: high-affinity choline transporter 1
RS000227	GO:0015294	solute:cation symporter activity	MF	-1.32952285	-0.75353739	-1.12184367	1.981758767	12.38833226	0.00616466	0.023605269	PREDICTED: facilitated trehalose transporter Tret1-2 homolog

RS003288	GO:0015294	solute:cation symporter activity	MF	-0.468141457	-0.663116971	0.331112622	4.523147937	12.02307867	0.007304519	0.027292266	PREDICTED: sodium- and chloride-dependent GABA transporter ine
RS011468	GO:0015294	solute:cation symporter activity	MF	-0.318737056	-0.110014844	-1.568608212	1.963272184	11.9167036	0.007674014	0.028433815	PREDICTED: solute carrier family 35 member F6
RS009360	GO:0015294	solute:cation symporter activity	MF	-0.052778092	-0.516301747	0.304429459	6.031942967	11.69868265	0.008489957	0.031037017	PREDICTED: sodium-dependent nutrient amino acid transporter 1
RS002295	GO:0015294	solute:cation symporter activity	MF	-0.760632417	-0.881718429	-1.336627404	7.31207469	11.50237509	0.009297576	0.033436228	PREDICTED: sodium- and chloride-dependent GABA transporter 1-like
RS012007	GO:0015294	solute:cation symporter activity	MF	-0.8805953	0.446042174	-0.616249485	2.603532973	10.7020781	0.013450915	0.045771636	PREDICTED: sodium-dependent phosphate transporter 2
RS002046	GO:0015294	solute:cation symporter activity	MF	-1.040945999	-1.141609481	-1.378037143	3.132734296	9.81352734	0.020219576	0.06404561	PREDICTED: sodium- and chloride-dependent GABA transporter 1-like
RS009928	GO:0015294	solute:cation symporter activity	MF	-0.835456878	-2.347674561	-0.849585016	1.053053669	9.23428246	0.02633282	0.079385129	PREDICTED: sodium-dependent noradrenaline transporter-like
RS012784	GO:0015294	solute:cation symporter activity	MF	-0.964188065	-1.053670411	-1.487092291	6.693074429	8.119987423	0.043595935	0.118762068	PREDICTED: excitatory amino acid transporter-like
RS005264	GO:0015294	solute:cation symporter activity	MF	-1.488549143	-1.308665565	-1.379377385	1.600885014	7.17732657	0.066455483	0.164816926	PREDICTED: sodium-dependent neutral amino acid transporter B(0)AT3
RS009361	GO:0015294	solute:cation symporter activity	MF	-0.469548252	-1.030089652	-0.272125463	2.379260128	5.571170404	0.134443268	0.278300217	PREDICTED: sodium- and chloride-dependent glycine transporter 2-like
RS009362	GO:0015294	solute:cation symporter activity	MF	-0.151734251	-1.379851033	-0.853593219	0.577299208	4.353138426	0.225770076	0.399764452	PREDICTED: sodium- and chloride-dependent glycine transporter 2-like
RS015079	GO:0015294	solute:cation symporter activity	MF	-1.376295246	0.661290456	0.618943801	0.209343525	4.174647883	0.243212401	0.420568148	PREDICTED: facilitated trehalose transporter Tret1-like
RS000693	GO:0015294	solute:cation symporter activity	MF	-0.697277451	-0.63859341	-1.105155719	1.668516432	3.761841056	0.288355965	0.47128419	PREDICTED: solute carrier family 12 member 4
RS015453	GO:0015294	solute:cation symporter activity	MF	-0.09263959	0.415348464	0.277714417	5.686672294	3.355959113	0.33992981	0.521573591	PREDICTED: sodium-dependent phosphate transporter 2
RS007790	GO:0015294	solute:cation symporter activity	MF	0.13034852	0.225712601	0.386180211	4.625198528	2.606288996	0.456388071	0.628856824	PREDICTED: mitochondrial glutamate carrier 1-like
RS002215	GO:0015294	solute:cation symporter activity	MF	-0.459893543	-0.373663745	-0.327616751	3.245153068	2.258109459	0.520593488	0.682313913	PREDICTED: CMP-sialic acid transporter 1
RS003602	GO:0015294	solute:cation symporter activity	MF	-0.963739329	-0.484991816	-0.758608967	0.476696176	1.634124817	0.651677236	0.782683875	PREDICTED: solute carrier family 28 member 3
RS003990	GO:0015294	solute:cation symporter activity	MF	-0.055368209	0.238070575	0.293320892	4.25914611	1.454595738	0.692788544	0.811066898	PREDICTED: UDP-galactose translocator
RS008405	GO:0015294	solute:cation symporter activity	MF	-0.507380646	-0.391727947	-0.160990784	2.501471485	1.183658987	0.75692617	0.857267824	PREDICTED: solute carrier family 12 member 9
RS006075	GO:0015294	solute:cation symporter activity	MF	-0.226394382	-0.065362369	0.088572196	3.008430896	0.898142308	0.82587609	0.901150881	PREDICTED: acetyl-coenzyme A transporter 1
RS013172	GO:0015294	solute:cation symporter activity	MF	0.064707769	0.050177661	0.256276698	4.54520608	0.870549691	0.832528393	0.905630734	PREDICTED: N-acetyl-D-glucosamine kinase
RS002366	GO:0015293	symporter activity	MF	-2.013469227	2.285528889	0.940362283	2.009763796	76.37983445	1.83374E-16	3.57494E-15	PREDICTED: sodium-dependent serotonin transporter
RS006315	GO:0015293	symporter activity	MF	-2.445857294	-4.975199992	0.147442902	3.069628072	71.7469443	1.80352E-15	3.24017E-14	PREDICTED: bumetanide-sensitive sodium-(potassium)-chloride cotransporter
RS001006	GO:0015293	symporter activity	MF	-1.308055843	0.748595123	2.011362686	7.151837524	64.31436805	7.03086E-14	1.12512E-12	PREDICTED: sodium-dependent nutrient amino acid transporter 1-like
RS007831	GO:0015293	symporter activity	MF	-0.285689079	2.23184291	-0.54998832	3.347851609	60.46667692	4.67238E-13	7.00523E-12	PREDICTED: sodium- and chloride-dependent GABA transporter ine-like
RS009578	GO:0015293	symporter activity	MF	-2.77838182	-2.247321578	-2.454512864	4.019688809	42.39569844	3.30671E-09	3.59937E-08	PREDICTED: solute carrier family 28 member 3
RS001007	GO:0015293	symporter activity	MF	-0.057453873	2.550863666	4.593751253	0.34449975	41.36132174	5.4813E-09	5.84023E-08	PREDICTED: sodium-dependent nutrient amino acid transporter 1-like
RS009711	GO:0015293	symporter activity	MF	0.862276886	1.272132135	1.421293308	6.056371062	36.7986326	5.07562E-08	4.85732E-07	PREDICTED: tropinone reductase 2-like
RS006320	GO:0015293	symporter activity	MF	1.668458777	1.549661536	0.932473469	6.183206129	30.51118393	1.07731E-06	8.96741E-06	PREDICTED: bumetanide-sensitive sodium-(potassium)-chloride cotransporter
RS009579	GO:0015293	symporter activity	MF	-0.361377028	0.937501921	0.004604885	5.160969196	28.85056945	2.40732E-06	1.91039E-05	PREDICTED: solute carrier family 28 member 3-like
RS013310	GO:0015293	symporter activity	MF	-1.594173173	0.30942411	0.502900611	3.784418211	25.32704004	1.31907E-05	9.38549E-05	PREDICTED: sodium- and chloride-dependent transporter XTRP3
RS006319	GO:0015293	symporter activity	MF	-1.264071762	-1.378688832	-0.296664377	4.499770473	23.99784591	2.50059E-05	0.000169587	PREDICTED: bumetanide-sensitive sodium-(potassium)-chloride cotransporter
RS011507	GO:0015293	symporter activity	MF	0.081672207	0.439418944	1.152509484	5.150989282	22.16598652	6.02438E-05	0.000382801	PREDICTED: sialin
RS007614	GO:0015293	symporter activity	MF	0.699165628	-0.052782673	-0.817546641	5.472681929	18.94140065	0.000281133	0.001554478	PREDICTED: facilitated trehalose transporter Tret1-like
RS007694	GO:0015293	symporter activity	MF	-1.402205119	-1.429007484	-0.154037672	3.235518975	18.72862507	0.000311092	0.001703432	PREDICTED: proton myo-inositol cotransporter-like
RS009604	GO:0015293	symporter activity	MF	-1.648345631	0.550771769	0.526514629	2.368500525	18.41833332	0.000360561	0.001945804	PREDICTED: sodium-dependent noradrenaline transporter
RS014410	GO:0015293	symporter activity	MF	-0.538555444	-0.069436224	0.424307658	5.574699261	17.86202393	0.00046963	0.002466436	PREDICTED: sodium- and chloride-dependent neutral and basic amino acid transporter B(0+)
RS000837	GO:0015293	symporter activity	MF	-1.260815808	-0.582381283	0.014148923	5.012164404	17.85146996	0.000471988	0.002476758	PREDICTED: excitatory amino acid transporter 1-like
RS013870	GO:0015293	symporter activity	MF	-1.185159015	-0.789079424	-0.942978819	4.024142004	15.86729932	0.001207277	0.005765813	PREDICTED: sodium- and chloride-dependent glycine transporter 1-like
RS006318	GO:0015293	symporter activity	MF	-3.618949223	-2.702749807	-1.072386578	0.467850565	14.73641507	0.002056334	0.009147816	PREDICTED: bumetanide-sensitive sodium-(potassium)-chloride cotransporter
RS005708	GO:0015293	symporter activity	MF	-2.57707626	-0.767810406	-0.121337824	1.208165742	14.61680361	0.002175202	0.009598631	PREDICTED: sodium- and chloride-dependent glycine transporter 1-like
RS008723	GO:0015293	symporter activity	MF	0.765724899	0.492155106	-0.726953396	2.604372289	14.25219643	0.002581214	0.011140444	PREDICTED: excitatory amino acid transporter
RS009863	GO:0015293	symporter activity	MF	0.247967949	1.994626146	0.840783905	0.741237487	12.85234652	0.004967042	0.019634316	PREDICTED: sodium- and chloride-dependent glycine transporter 1-like
RS005241	GO:0015293	symporter activity	MF	-1.24648029	-0.946331544	-0.692073388	4.41212653	12.5745733	0.005653054	0.021933014	PREDICTED: high-affinity choline transporter 1
RS000227	GO:0015293	symporter activity	MF	-1.32952285	-0.75353739	-1.12184367	1.981758767	12.38833226	0.00616466	0.023605269	PREDICTED: facilitated trehalose transporter Tret1-2 homolog
RS003288	GO:0015293	symporter activity	MF	-0.468141457	-0.663116971	0.331112622	4.523147937	12.02307867	0.007304519	0.027292266	PREDICTED: sodium- and chloride-dependent GABA transporter ine
RS011468	GO:0015293	symporter activity	MF	-0.318737056	-0.110014844	-1.568608212	1.963272184	11.9167036	0.007674014	0.028433815	PREDICTED: solute carrier family 35 member F6
RS009360	GO:0015293	symporter activity	MF	-0.052778092	-0.516301747	0.304429459	6.031942967	11.69868265	0.008489957	0.031037017	PREDICTED: sodium-dependent nutrient amino acid transporter 1
RS002295	GO:0015293	symporter activity	MF	-0.760632417	-0.881718429	-1.336627404	7.31207469	11.50237509	0.009297576	0.033436228	PREDICTED: sodium- and chloride-dependent GABA transporter 1-like
RS012007	GO:0015293	symporter activity	MF	-0.8805953	0.446042174	-0.616249485	2.603532973	10.7020781	0.013450915	0.045771636	PREDICTED: sodium-dependent phosphate transporter 2
RS002046	GO:0015293	symporter activity	MF	-1.040945999	-1.141609481	-1.378037143	3.132734296	9.81352734	0.020219576	0.06404561	PREDICTED: sodium- and chloride-dependent GABA transporter 1-like
RS009928	GO:0015293	symporter activity	MF	-0.835456878	-2.347674561	-0.849585016	1.053053669	9.23428246	0.02633282	0.079385129	PREDICTED: sodium-dependent noradrenaline transporter-like
RS012784	GO:0015293	symporter activity	MF	-0.964188065	-1.053670411	-1.487092291	6.693074429	8.119987423	0.043595935	0.118762068	PREDICTED: excitatory amino acid transporter-like
RS005264	GO:0015293	symporter activity	MF	-1.488549143	-1.308665565	-1.379377385	1.600885014	7.17732657	0.066455483	0.164816926	PREDICTED: sodium-dependent neutral amino acid transporter B(0)AT3
RS009361	GO:0015293	symporter activity	MF	-0.469548252	-1.030089652	-0.272125463	2.379260128	5.571170404	0.134443268	0.278300217	PREDICTED: sodium- and chloride-dependent glycine transporter 2-like
RS009362	GO:0015293	symporter activity	MF	-0.151734251	-1.379851033	-0.853593219	0.577299208	4.353138426	0.225770076	0.399764452	PREDICTED: sodium- and chloride-dependent glycine transporter 2-like
RS015079	GO:0015293	symporter activity	MF	-1.376295246	0.661290456	0.618943801	0.209343525	4.174647883	0.243212401	0.420568148	PREDICTED: facilitated trehalose transporter Tret1-like
RS000693	GO:0015293	symporter activity	MF	-0.697277451	-0.63859341	-1.105155719	1.668516432	3.761841056	0.288355965	0.47128419	PREDICTED: solute carrier family 12 member 4
RS015453	GO:0015293	symporter activity	MF	-0.09263959	0.415348464	0.277714417	5.686672294	3.355959113	0.33992981	0.521573591	PREDICTED: sodium-dependent phosphate transporter 2
RS007790	GO:0015293	symporter activity	MF	0.13034852	0.225712601	0.386180211	4.625198528	2.606288996	0.456388071	0.628856824	PREDICTED: mitochondrial glutamate carrier 1-like
RS002215	GO:0015293	symporter activity	MF	-0.459893543	-0.373663745	-0.327616751	3.245153068	2.258109459	0.520593488	0.682313913	PREDICTED: CMP-sialic acid transporter 1
RS003602	GO:0015293	symporter activity	MF	-0.963739329	-0.484991816	-0.758608967	0.476696176	1.634124817	0.651677236	0.782683875	PREDICTED: solute carrier family 28 member 3
RS003990	GO:0015293	symporter activity	MF	-0.055368209	0.238070575	0.293320892	4.25914611	1.454595738	0.692788544	0.811066898	PREDICTED: UDP-galactose translocator
RS008405	GO:0015293	symporter activity	MF	-0.507380646	-0.391727947	-0.160990784	2.501471485	1.183658987	0.75692617	0.857267824	PREDICTED: solute carrier family 12 member 9
RS006075	GO:0015293	symporter activity	MF	-0.226394382	-0.065362369	0.088572196	3.008430896	0.898142308	0.82587609	0.901150881	PREDICTED: acetyl-coenzyme A transporter 1
RS013172	GO:0015293	symporter activity	MF	0.064707769	0.050177661	0.256276698	4.54520608	0.870549691	0.832528393	0.905630734	PREDICTED: N-acetyl-D-glucosamine kinase
RS007652	GO:0008509	anion transmembrane transporter activity	MF	0.585883623	0.528505008	4.851177054	4.584889963	291.2117195	7.93792E-63	8.92591E-61	PREDICTED: solute carrier family 26 member 10-like
RS004125	GO:0008509	anion transmembrane transporter activity	MF	1.407227463	2.012417871	-0.423642507	5.906215214	131.7530272	2.26607E-28	8.8083E-27	PREDICTED: ABC transporter G family member 9
RS003228	GO:0008509	anion transmembrane transporter activity	MF	-0.561103954	2.12048168	1.83683218	5.406987214	114.6440514	1.09829E-24	3.71824E-23	PREDICTED: b(0,+)-type amino acid transporter 1-like
RS013974	GO:0008509	anion transmembrane transporter activity	MF	1.308057058	2.315023189	0.876327879	6.127768048	111.7248777	4.66769E-24	1.53887E-22	PREDICTED: monocarboxylate transporter 10
RS012045	GO:0008509	anion transmembrane transporter activity	MF	-2.417547613	-3.846942398	-2.948472949	3.138267013	95.12499238	1.73561E-20	4.58246E-19	PREDICTED: sodium-independent sulfate anion transporter
RS002200	GO:0008509	anion transmembrane transporter activity	MF	-2.534854462	-2.965618885	0.446701011	4.187537984	94.94596227	1.89639E-20	4.97566E-19	PREDICTED: ATP-binding cassette sub-family G member 4
RS014732	GO:0008509	anion transmembrane transporter activity	MF	-2.171879817	-1.24728548	0.09932889	5.367265273	82.18961662	1.04062E-17	2.21004E-16	PREDICTED: solute carrier organic anion transporter family member 2A1
RS015390	GO:0008509	anion transmembrane transporter activity	MF	-1.260588417	-0.88728058	1.174441311	5.643186003	81.54774944	1.42892E-17	3.01438E-16	PREDICTED: sodium-independent sulfate anion transporter
RS006315	GO:0008509	anion transmembrane transporter activity	MF	-2.445857294	-4.975199992	0.147442902	3.069628072	71.7469443	1.80352E-15	3.24017E-14	PREDICTED: bumetanide-sensitive sodium-(potassium)-chloride cotransporter
RS010878	GO:0008509	anion transmembrane transporter activity	MF	-0.202147251	0.219397642	1.259334773	5.8954575	54.42606082	9.10217E-12	1.23527E-10	PREDICTED: band 3 anion transport protein
RS008617	GO:0008509	anion transmembrane transporter activity	MF	-1.758654905	-1.933395291	0.119722157	4.240860888	52.46640405	2.38231E-11	3.09308E-10	PREDICTED: proton-coupled amino acid transporter 1
RS006021	GO:0008509	anion transmembrane transporter activity	MF	-1.978389488	-0.987147201	0.23457041	5.767022167	52.1310105	2.80858E-11	3.63527E-10	PREDICTED: cationic amino acid transporter 3
RS009380	GO:0008509	anion transmembrane transporter activity	MF	1.181487238	0.230316163	-1.011984835	4.090504935	45.23692145	8.23976E-10	9.50289E-09	PREDICTED: b(0,+)-type amino acid transporter 1-like
RS005640	GO:0008509	anion transmembrane transporter activity	MF	0.969003673	1.718434974	-0.876644168	4.401662752	43.66119041	1.78117E-09	1.9764E-08	PREDICTED: glycine receptor subunit alpha-4
RS001007	GO:0008509	anion transmembrane transporter activity	MF	-0.057453873	2.550863666	4.593751253	0.34449975	41.36132174	5.4813E-09	5.84023E-08	PREDICTED: sodium-dependent nutrient amino acid transporter 1-like
RS005851	GO:0008509	anion transmembrane transporter activity	MF	0.526397294	1.41413322	1.673681755	7.829535198	40.74316203	7.413E-09	7.77347E-08	PREDICTED: proton-coupled amino acid transporter 4
RS006814	GO:0008509	anion transmembrane transporter activity	MF	-0.514796382	1.014347124	0.975336891	4.041760153	30.97725487	8.59474E-07	7.23543E-06	PREDICTED: large neutral amino acids transporter small subunit 2
RS006320	GO:0008509	anion transmembrane transporter activity	MF	1.668458777	1.549661536	0.932473469	6.183206129	30.51118393	1.07731E-06	8.96741E-06	PREDICTED: bumetanide-sensitive sodium-(potassium)-chloride cotransporter
RS000193	GO:0008509	anion transmembrane transporter activity	MF	-0.612906575	0.552707825	0.864925878	6.395375097	30.19397061	1.25629E-06	1.03772E-05	PREDICTED: b(0,+)-type amino acid transporter 1
RS006319	GO:0008509	anion transmembrane transporter activity	MF	-1.264071762	-1.378688832	-0.296664377	4.499770473	23.99784591	2.50059E-05	0.000169587	PREDICTED: bumetanide-sensitive sodium-(potassium)-chloride cotransporter
RS011507	GO:0008509	anion transmembrane transporter activity	MF	0.081672207	0.439418944	1.152509484	5.150989282	22.16598652	6.02438E-05	0.000382801	PREDICTED: sialin
RS012462	GO:0008509	anion transmembrane transporter activity	MF	-1.887439376	-1.068323927	-1.822700455	2.935725927	21.16526065	9.72757E-05	0.000593839	PREDICTED: synaptic vesicle glycoprotein 2B
RS012046	GO:0008509	anion transmembrane transporter activity	MF	-0.044612109	1.318321788	-0.182662099	3.873303092	21.0021542	0.000105168	0.000636465	PREDICTED: sodium-independent sulfate anion transporter-like
RS014287	GO:0008509	anion transmembrane transporter activity	MF	-0.825809928	1.486619083	1.592270521	1.389357496	20.39684624	0.000140447	0.000831501	PREDICTED: sialin
RS004393	GO:0008509	anion transmembrane transporter activity	MF	-1.227790861	-2.264108903	-0.817189459	2.289519889	20.23673107	0.000151606	0.000890546	PREDICTED: putative tricarboxylate transport protein, mitochondrial
RS000837	GO:0008509	anion transmembrane transporter activity	MF	-1.260815808	-0.582381283	0.014148923	5.012164404	17.85146996	0.000471988	0.002476758	PREDICTED: excitatory amino acid transporter 1-like
RS000849	GO:0008509	anion transmembrane transporter activity	MF	-0.753175709	-1.571391853	-1.058453667	2.708513766	17.06922787	0.000683946	0.003464852	PREDICTED: monocarboxylate transporter 9-like
RS012047	GO:0008509	anion transmembrane transporter activity	MF	-1.033691566	-1.204387105	-0.573210977	3.717195265	16.53395031	0.000881135	0.004370625	PREDICTED: sodium-independent sulfate anion transporter-like
RS000927	GO:0008509	anion transmembrane transporter activity	MF	-2.570527298	-2.804707147	-0.755163456	0.667619183	16.35029216	0.000961058	0.004715063	PREDICTED: b(0,+)-type amino acid transporter 1-like
RS013870	GO:0008509	anion transmembrane transporter activity	MF	-1.185159015	-0.789079424	-0.942978819	4.024142004	15.86729932	0.001207277	0.005765813	PREDICTED: sodium- and chloride-dependent glycine transporter 1-like
RS000928	GO:0008509	anion transmembrane transporter activity	MF	-2.818387407	-2.387215456	-1.195316578	0.324906115	15.29049888	0.001584497	0.007309578	B(0,+)-type amino acid transporter 1
RS006318	GO:0008509	anion transmembrane transporter activity	MF	-3.618949223	-2.702749807	-1.072386578	0.467850565	14.73641507	0.002056334	0.009147816	PREDICTED: bumetanide-sensitive sodium-(potassium)-chloride cotransporter
RS005708	GO:0008509	anion transmembrane transporter activity	MF	-2.57707626	-0.767810406	-0.121337824	1.208165742	14.61680361	0.002175202	0.009598631	PREDICTED: sodium- and chloride-dependent glycine transporter 1-like
RS008723	GO:0008509	anion transmembrane transporter activity	MF	0.765724899	0.492155106	-0.726953396	2.604372289	14.25219643	0.002581214	0.011140444	PREDICTED: excitatory amino acid transporter
RS011379	GO:0008509	anion transmembrane transporter activity	MF	-0.945895165	-0.137739549	0.02413553	6.197611827	14.11352967	0.002754622	0.01177587	PREDICTED: chloride intracellular channel exc-4
RS012010	GO:0008509	anion transmembrane transporter activity	MF	-0.658055692	-0.163236513	0.25753802	6.662027841	13.45566221	0.003748014	0.01543574	PREDICTED: sodium-dependent phosphate transporter 1-B-like
RS005241	GO:0008509	anion transmembrane transporter activity	MF	-1.24648029	-0.946331544	-0.692073388	4.41212653	12.5745733	0.005653054	0.021933014	PREDICTED: high-affinity choline transporter 1
RS002985	GO:0008509	anion transmembrane transporter activity	MF	-0.571114377	-0.476136182	-0.859409917	5.548946497	12.07709757	0.007123669	0.026732864	PREDICTED: Y+L amino acid transporter 2
RS006740	GO:0008509	anion transmembrane transporter activity	MF	-0.43064001	-0.612335737	-0.738849827	6.295225865	10.98541247	0.011805018	0.040967869	PREDICTED: glutamate-gated chloride channel
RS007836	GO:0008509	anion transmembrane transporter activity	MF	-0.670404831	1.05713246	-0.759263575	1.495067002	10.91840834	0.012175354	0.042079146	PREDICTED: bestrophin-4
RS000123	GO:0008509	anion transmembrane transporter activity	MF	0.692109916	0.810423229	0.325162475	7.0801236	10.8417935	0.012612853	0.04340062	PREDICTED: ATP-binding cassette sub-family F member 3
RS012007	GO:0008509	anion transmembrane transporter activity	MF	-0.8805953	0.446042174	-0.616249485	2.603532973	10.7020781	0.013450915	0.045771636	PREDICTED: sodium-dependent phosphate transporter 2
RS003950	GO:0008509	anion transmembrane transporter activity	MF	-0.587671115	-0.868990799	-0.714405964	4.844171479	10.0941278	0.017782729	0.057765203	PREDICTED: cationic amino acid transporter 2
RS006809	GO:0008509	anion transmembrane transporter activity	MF	-0.069599294	0.161390813	-0.669189997	5.521140821	9.427681054	0.02411329	0.074118459	PREDICTED: sodium bicarbonate cotransporter 3
RS012808	GO:0008509	anion transmembrane transporter activity	MF	0.436531101	0.768358177	0.047570539	8.601906761	9.12311113	0.027698056	0.082719781	PREDICTED: ATP-binding cassette sub-family E member 1

RS009139	GO:0008509	anion transmembrane transporter activity	MF	0.649351007	1.292845053	0.336142374	2.039377777	9.031613663	0.028873505	0.085540561	PREDICTED: Y+L amino acid transporter 2
RS007859	GO:0008509	anion transmembrane transporter activity	MF	-1.037355681	-0.953164057	-1.137934678	2.631254112	8.895408464	0.0307143	0.08987358	PREDICTED: proton-coupled amino acid transporter 4
RS006017	GO:0008509	anion transmembrane transporter activity	MF	-0.226333875	0.829010789	0.173010283	3.457042927	8.42538268	0.037991671	0.106302401	PREDICTED: cationic amino acid transporter 2
RS012784	GO:0008509	anion transmembrane transporter activity	MF	-0.964188065	-1.053670411	-1.487092291	6.693074429	8.119987423	0.043595935	0.118762068	PREDICTED: excitatory amino acid transporter-like
RS008614	GO:0008509	anion transmembrane transporter activity	MF	-0.377384484	0.178151297	0.139169662	6.960985845	7.911936368	0.047867212	0.127882831	PREDICTED: H(+)/Cl(-) exchange transporter 5
RS012051	GO:0008509	anion transmembrane transporter activity	MF	-1.206457775	-0.320139517	-0.400442703	3.93903891	7.774642457	0.050906164	0.13434875	PREDICTED: sodium-independent sulfate anion transporter-like
RS006810	GO:0008509	anion transmembrane transporter activity	MF	-0.692918241	-0.709642301	-0.469276201	5.552917252	7.276110601	0.063598968	0.159523084	PREDICTED: Y+L amino acid transporter 2
RS015105	GO:0008509	anion transmembrane transporter activity	MF	-0.678666411	-2.224992259	-0.562865466	0.559101899	7.185153044	0.066224707	0.164438873	PREDICTED: organic cation transporter 1
RS003825	GO:0008509	anion transmembrane transporter activity	MF	-0.116552603	-0.74841501	-0.195189608	7.935069367	6.915883201	0.07462774	0.18005155	PREDICTED: H(+)/Cl(-) exchange transporter 7
RS012048	GO:0008509	anion transmembrane transporter activity	MF	-0.674894361	-1.155560739	-0.486644875	2.649535097	6.691851744	0.082395778	0.193852499	PREDICTED: sodium-independent sulfate anion transporter-like
RS009471	GO:0008509	anion transmembrane transporter activity	MF	0.454440582	0.068570398	0.92902968	1.975531925	6.680170813	0.082821497	0.194672252	PREDICTED: potassium voltage-gated channel protein Shal
RS011409	GO:0008509	anion transmembrane transporter activity	MF	-0.278990895	0.058673971	0.653521491	10.28933054	5.992909669	0.111955695	0.243939451	PREDICTED: voltage-dependent anion-selective channel protein 2
RS012532	GO:0008509	anion transmembrane transporter activity	MF	0.52422059	0.27169055	0.170626759	5.950381512	5.527522768	0.137001687	0.282351902	PREDICTED: adenosine 3'-phospho 5'-phosphosulfate transporter 2
RS012098	GO:0008509	anion transmembrane transporter activity	MF	-0.722635213	-0.863193595	-0.599560773	3.5591329	5.486483871	0.13944927	0.285797251	PREDICTED: protein tweety
RS002769	GO:0008509	anion transmembrane transporter activity	MF	-0.180596666	-0.510025269	-0.494314256	5.266272897	5.147923967	0.161280396	0.318264698	PREDICTED: mitochondrial 2-oxoglutarate/malate carrier protein
RS006076	GO:0008509	anion transmembrane transporter activity	MF	-0.399500112	-0.234112198	-0.791572834	3.218719557	5.135126595	0.162165734	0.319411206	PREDICTED: cationic amino acid transporter 4
RS007406	GO:0008509	anion transmembrane transporter activity	MF	1.53218285	1.416234104	0.234676903	0.509107572	4.915967777	0.17805483	0.341676447	PREDICTED: sodium-independent sulfate anion transporter-like
RS000049	GO:0008509	anion transmembrane transporter activity	MF	-0.167016261	-0.078808593	0.315739518	4.19490186	3.918242209	0.270428905	0.452114912	PREDICTED: protein I'm not dead yet-like
RS000693	GO:0008509	anion transmembrane transporter activity	MF	-0.697277451	-0.63859341	-1.105155719	1.668516432	3.761841056	0.288355965	0.47128419	PREDICTED: solute carrier family 12 member 4
RS008419	GO:0008509	anion transmembrane transporter activity	MF	-0.579270886	-0.22684126	-0.07023873	5.06982487	3.507414936	0.319801701	0.502353125	PREDICTED: chloride channel protein 2
RS015453	GO:0008509	anion transmembrane transporter activity	MF	-0.09263959	0.415348464	0.277714417	5.686672294	3.355959113	0.33992981	0.521573591	PREDICTED: sodium-dependent phosphate transporter 2
RS007790	GO:0008509	anion transmembrane transporter activity	MF	0.13034852	0.225712601	0.386180211	4.625198528	2.606288996	0.456388071	0.628856824	PREDICTED: mitochondrial glutamate carrier 1-like
RS007514	GO:0008509	anion transmembrane transporter activity	MF	-0.393644456	0.206862252	-0.369998274	2.851975701	2.440356048	0.48616657	0.653933758	PREDICTED: solute carrier family 26 member 10-like
RS004471	GO:0008509	anion transmembrane transporter activity	MF	0.302505199	0.1016203	-0.272900349	3.077100879	2.396738775	0.494241988	0.660492741	PREDICTED: Y+L amino acid transporter 2
RS005021	GO:0008509	anion transmembrane transporter activity	MF	-0.06781788	-0.474899669	-0.350734329	3.155367473	2.352458366	0.50254583	0.667200314	PREDICTED: molybdate-anion transporter-like
RS002882	GO:0008509	anion transmembrane transporter activity	MF	0.362043543	0.12287268	0.14951259	3.567153224	1.277605364	0.734457649	0.842342193	PREDICTED: putative sodium-coupled neutral amino acid transporter 7
RS008405	GO:0008509	anion transmembrane transporter activity	MF	-0.507380646	-0.391727947	-0.160990784	2.501471485	1.183658987	0.75692617	0.857267824	PREDICTED: solute carrier family 12 member 9
RS013172	GO:0008509	anion transmembrane transporter activity	MF	0.064707769	0.050177661	0.256276698	4.54520608	0.870549691	0.832528393	0.905630734	PREDICTED: N-acetyl-D-glucosamine kinase
RS006019	GO:0008509	anion transmembrane transporter activity	MF	0.016800687	-0.569398535	-0.206623396	0.539126613	0.775635116	0.855285471	0.920559373	PREDICTED: cationic amino acid transporter 2
RS014940	GO:0008509	anion transmembrane transporter activity	MF	0.00473671	-0.350880149	-0.354295712	0.736422598	0.582482205	0.900430699	0.946555742	PREDICTED: potassium voltage-gated channel protein Shal
RS004110	GO:0008509	anion transmembrane transporter activity	MF	-0.109326082	-0.245764423	-0.393853677	2.856144774	0.536964028	0.910698682	0.95276119	PREDICTED: phospholipase A2 inhibitor and Ly6/PLAUR domain-containing protein
RS007404	GO:0008509	anion transmembrane transporter activity	MF	0.341548622	0.423696565	0.486833568	3.079113058	0.267214539	0.9660718	0.982215891	PREDICTED: sodium-independent sulfate anion transporter-like
RS007304	GO:0008509	anion transmembrane transporter activity	MF	-0.03874979	-0.117709759	-0.024621974	4.815387852	0.237457293	0.971327222	0.985126901	PREDICTED: solute carrier family 17 member 9
RS002585	GO:0008509	anion transmembrane transporter activity	MF	0.061337763	0.015683494	0.119579633	4.655608643	0.205533408	0.976691112	0.987602807	PREDICTED: mitochondrial pyruvate carrier 1
RS003228	GO:0015849	organic acid transport	BP	-0.561103954	2.12048168	1.83683218	5.406987214	114.6440514	1.09829E-24	3.71824E-23	PREDICTED: b(0,+)-type amino acid transporter 1-like
RS013974	GO:0015849	organic acid transport	BP	1.308057058	2.315023189	0.876327879	6.127768048	111.7248777	4.66769E-24	1.53887E-22	PREDICTED: monocarboxylate transporter 10
RS012045	GO:0015849	organic acid transport	BP	-2.417547613	-3.846942398	-2.948472949	3.138267013	95.12499238	1.73561E-20	4.58246E-19	PREDICTED: sodium-independent sulfate anion transporter
RS006315	GO:0015849	organic acid transport	BP	-2.445857294	-4.975199992	0.147442902	3.069628072	71.7469443	1.80352E-15	3.24017E-14	PREDICTED: bumetanide-sensitive sodium-(potassium)-chloride cotransporter
RS008617	GO:0015849	organic acid transport	BP	-1.758654905	-1.933395291	0.119722157	4.240860888	52.46640405	2.38231E-11	3.09308E-10	PREDICTED: proton-coupled amino acid transporter 1
RS006021	GO:0015849	organic acid transport	BP	-1.978389488	-0.987147201	0.23457041	5.767022167	52.1310105	2.80858E-11	3.63527E-10	PREDICTED: cationic amino acid transporter 3
RS009380	GO:0015849	organic acid transport	BP	1.181487238	0.230316163	-1.011984835	4.090504935	45.23692145	8.23976E-10	9.50289E-09	PREDICTED: b(0,+)-type amino acid transporter 1-like
RS001007	GO:0015849	organic acid transport	BP	-0.057453873	2.550863666	4.593751253	0.34449975	41.36132174	5.4813E-09	5.84023E-08	PREDICTED: sodium-dependent nutrient amino acid transporter 1-like
RS005851	GO:0015849	organic acid transport	BP	0.526397294	1.41413322	1.673681755	7.829535198	40.74316203	7.413E-09	7.77347E-08	PREDICTED: proton-coupled amino acid transporter 4
RS006814	GO:0015849	organic acid transport	BP	-0.514796382	1.014347124	0.975336891	4.041760153	30.97725487	8.59474E-07	7.23543E-06	PREDICTED: large neutral amino acids transporter small subunit 2
RS006320	GO:0015849	organic acid transport	BP	1.668458777	1.549661536	0.932473469	6.183206129	30.51118393	1.07731E-06	8.96741E-06	PREDICTED: bumetanide-sensitive sodium-(potassium)-chloride cotransporter
RS007713	GO:0015849	organic acid transport	BP	-1.593472957	-0.392083561	-0.180235411	5.938924251	30.40817878	1.13245E-06	9.40771E-06	PREDICTED: ATP-binding cassette sub-family D member 2
RS000193	GO:0015849	organic acid transport	BP	-0.612906575	0.552707825	0.864925878	6.395375097	30.19397061	1.25629E-06	1.03772E-05	PREDICTED: b(0,+)-type amino acid transporter 1
RS014340	GO:0015849	organic acid transport	BP	-0.92887959	-0.092715464	0.219765138	5.620832256	22.82606695	4.38983E-05	0.00028483	PREDICTED: ileal sodium/bile acid cotransporter-like
RS004393	GO:0015849	organic acid transport	BP	-1.227790861	-2.264108903	-0.817189459	2.289519889	20.23673107	0.000151606	0.000890546	PREDICTED: putative tricarboxylate transport protein, mitochondrial
RS000837	GO:0015849	organic acid transport	BP	-1.260815808	-0.582381283	0.014148923	5.012164404	17.85146996	0.000471988	0.002476758	PREDICTED: excitatory amino acid transporter 1-like
RS011194	GO:0015849	organic acid transport	BP	-0.043178438	0.101493976	0.864859407	6.406781775	17.21343829	0.00063878	0.003251735	PREDICTED: mitochondrial pyruvate carrier 2-like
RS000849	GO:0015849	organic acid transport	BP	-0.753175709	-1.571391853	-1.058453667	2.708513766	17.06922787	0.000683946	0.003464852	PREDICTED: monocarboxylate transporter 9-like
RS000927	GO:0015849	organic acid transport	BP	-2.570527298	-2.804707147	-0.755163456	0.667619183	16.35029216	0.000961058	0.004715063	PREDICTED: b(0,+)-type amino acid transporter 1-like
RS013870	GO:0015849	organic acid transport	BP	-1.185159015	-0.789079424	-0.942978819	4.024142004	15.86729932	0.001207277	0.005765813	PREDICTED: sodium- and chloride-dependent glycine transporter 1-like
RS000928	GO:0015849	organic acid transport	BP	-2.818387407	-2.387215456	-1.195316578	0.324906115	15.29049888	0.001584497	0.007309578	B(0,+)-type amino acid transporter 1
RS005708	GO:0015849	organic acid transport	BP	-2.57707626	-0.767810406	-0.121337824	1.208165742	14.61680361	0.002175202	0.009598631	PREDICTED: sodium- and chloride-dependent glycine transporter 1-like
RS008723	GO:0015849	organic acid transport	BP	0.765724899	0.492155106	-0.726953396	2.604372289	14.25219643	0.002581214	0.011140444	PREDICTED: excitatory amino acid transporter
RS005241	GO:0015849	organic acid transport	BP	-1.24648029	-0.946331544	-0.692073388	4.41212653	12.5745733	0.005653054	0.021933014	PREDICTED: high-affinity choline transporter 1
RS002985	GO:0015849	organic acid transport	BP	-0.571114377	-0.476136182	-0.859409917	5.548946497	12.07709757	0.007123669	0.026732864	PREDICTED: Y+L amino acid transporter 2
RS003950	GO:0015849	organic acid transport	BP	-0.587671115	-0.868990799	-0.714405964	4.844171479	10.0941278	0.017782729	0.057765203	PREDICTED: cationic amino acid transporter 2
RS009139	GO:0015849	organic acid transport	BP	0.649351007	1.292845053	0.336142374	2.039377777	9.031613663	0.028873505	0.085540561	PREDICTED: Y+L amino acid transporter 2
RS007859	GO:0015849	organic acid transport	BP	-1.037355681	-0.953164057	-1.137934678	2.631254112	8.895408464	0.0307143	0.08987358	PREDICTED: proton-coupled amino acid transporter 4
RS006017	GO:0015849	organic acid transport	BP	-0.226333875	0.829010789	0.173010283	3.457042927	8.42538268	0.037991671	0.106302401	PREDICTED: cationic amino acid transporter 2
RS002298	GO:0015849	organic acid transport	BP	0.444685109	0.888871791	0.46931162	5.877833769	8.218233291	0.041710358	0.114594295	PREDICTED: battenin
RS012784	GO:0015849	organic acid transport	BP	-0.964188065	-1.053670411	-1.487092291	6.693074429	8.119987423	0.043595935	0.118762068	PREDICTED: excitatory amino acid transporter-like
RS006810	GO:0015849	organic acid transport	BP	-0.692918241	-0.709642301	-0.469276201	5.552917252	7.276110601	0.063598968	0.159523084	PREDICTED: Y+L amino acid transporter 2
RS015105	GO:0015849	organic acid transport	BP	-0.678666411	-2.224992259	-0.562865466	0.559101899	7.185153044	0.066224707	0.164438873	PREDICTED: organic cation transporter 1
RS009471	GO:0015849	organic acid transport	BP	0.454440582	0.068570398	0.92902968	1.975531925	6.680170813	0.082821497	0.194672252	PREDICTED: potassium voltage-gated channel protein Shal
RS002769	GO:0015849	organic acid transport	BP	-0.180596666	-0.510025269	-0.494314256	5.266272897	5.147923967	0.161280396	0.318264698	PREDICTED: mitochondrial 2-oxoglutarate/malate carrier protein
RS006076	GO:0015849	organic acid transport	BP	-0.399500112	-0.234112198	-0.791572834	3.218719557	5.135126595	0.162165734	0.319411206	PREDICTED: cationic amino acid transporter 4
RS002889	GO:0015849	organic acid transport	BP	-0.128703142	0.3529612	0.286577104	4.360050122	4.605415706	0.203077987	0.373149135	PREDICTED: bifunctional arginine demethylase and lysyl-hydroxylase PSR
RS000049	GO:0015849	organic acid transport	BP	-0.167016261	-0.078808593	0.315739518	4.19490186	3.918242209	0.270428905	0.452114912	PREDICTED: protein I'm not dead yet-like
RS007790	GO:0015849	organic acid transport	BP	0.13034852	0.225712601	0.386180211	4.625198528	2.606288996	0.456388071	0.628856824	PREDICTED: mitochondrial glutamate carrier 1-like
RS004471	GO:0015849	organic acid transport	BP	0.302505199	0.1016203	-0.272900349	3.077100879	2.396738775	0.494241988	0.660492741	PREDICTED: Y+L amino acid transporter 2
RS002882	GO:0015849	organic acid transport	BP	0.362043543	0.12287268	0.14951259	3.567153224	1.277605364	0.734457649	0.842342193	PREDICTED: putative sodium-coupled neutral amino acid transporter 7
RS013172	GO:0015849	organic acid transport	BP	0.064707769	0.050177661	0.256276698	4.54520608	0.870549691	0.832528393	0.905630734	PREDICTED: N-acetyl-D-glucosamine kinase
RS006019	GO:0015849	organic acid transport	BP	0.016800687	-0.569398535	-0.206623396	0.539126613	0.775635116	0.855285471	0.920559373	PREDICTED: cationic amino acid transporter 2
RS014940	GO:0015849	organic acid transport	BP	0.00473671	-0.350880149	-0.354295712	0.736422598	0.582482205	0.900430699	0.946555742	PREDICTED: potassium voltage-gated channel protein Shal
RS004110	GO:0015849	organic acid transport	BP	-0.109326082	-0.245764423	-0.393853677	2.856144774	0.536964028	0.910698682	0.95276119	PREDICTED: phospholipase A2 inhibitor and Ly6/PLAUR domain-containing protein
RS002585	GO:0015849	organic acid transport	BP	0.061337763	0.015683494	0.119579633	4.655608643	0.205533408	0.976691112	0.987602807	PREDICTED: mitochondrial pyruvate carrier 1
RS008706	GO:0015849	organic acid transport	BP	0.063443874	0.058144534	-0.01579188	5.633533699	0.196321115	0.978180999	0.988383465	PREDICTED: ATP-binding cassette sub-family D member 3
RS005614	GO:0015849	organic acid transport	BP	-0.026120006	0.069729297	0.030053445	4.472662817	0.146084989	0.985784124	0.992693667	PREDICTED: mitochondrial folate transporter/carrier
RS006078	GO:0004222	metalloendopeptidase activity	MF	1.058700106	4.318785292	0.61657367	5.835263341	191.7148064	2.6012E-41	1.85972E-39	PREDICTED: membrane metallo-endopeptidase-like 1
RS003189	GO:0004222	metalloendopeptidase activity	MF	1.421133507	7.114465875	1.711486225	2.707691835	180.9071544	5.61771E-39	3.76792E-37	PREDICTED: zinc metalloproteinase nas-13
RS007383	GO:0004222	metalloendopeptidase activity	MF	3.140849899	1.953394017	-0.173133765	5.560811886	113.7013705	1.75249E-24	6.16307E-23	PREDICTED: A disintegrin and metalloproteinase with thrombospondin motifs 2
RS007886	GO:0004222	metalloendopeptidase activity	MF	2.694380781	3.033223869	-0.604166269	2.768332908	78.04950717	8.04176E-17	1.96322E-15	PREDICTED: high choriolytic enzyme 1
RS003677	GO:0004222	metalloendopeptidase activity	MF	0	7.198437613	4.2500184	1.019458099	75.40696016	2.96402E-16	6.98693E-15	PREDICTED: zinc metalloproteinase nas-4-like
RS006399	GO:0004222	metalloendopeptidase activity	MF	0.72248749	2.652725153	-0.926248399	3.569216287	68.19752105	1.0379E-14	2.26596E-13	PREDICTED: zinc metalloproteinase nas-13-like
RS007302	GO:0004222	metalloendopeptidase activity	MF	3.592624209	1.332691686	-0.178641399	4.991456973	61.90227609	2.30536E-13	4.57276E-12	Zinc metalloproteinase nas-4
RS003932	GO:0004222	metalloendopeptidase activity	MF	0.926854931	3.003472024	0.942694689	8.150548831	57.65602571	1.86149E-12	3.44059E-11	PREDICTED: membrane metallo-endopeptidase-like 1
RS003509	GO:0004222	metalloendopeptidase activity	MF	0.89051121	2.217933238	2.805968503	6.287950549	51.47633143	3.87261E-11	6.411E-10	PREDICTED: venom metalloproteinase 3
RS003347	GO:0004222	metalloendopeptidase activity	MF	1.800907388	1.911544861	3.083438647	4.715227846	46.64160152	4.14248E-10	6.29696E-09	PREDICTED: A disintegrin and metalloproteinase with thrombospondin motifs 18-like
RS001995	GO:0004222	metalloendopeptidase activity	MF	1.823183595	5.681933258	3.751988813	4.01519423	46.09032342	5.42615E-10	8.16244E-09	PREDICTED: endothelin-converting enzyme 1-like
RS006934	GO:0004222	metalloendopeptidase activity	MF	0.749688438	0.341267485	2.399126912	5.017245847	44.89414693	9.74461E-10	1.42629E-08	PREDICTED: A disintegrin and metalloproteinase with thrombospondin motifs 9
RS014350	GO:0004222	metalloendopeptidase activity	MF	0.668526397	2.620672	2.38029958	2.978004963	30.12660914	1.29797E-06	1.35656E-05	Matrix metalloproteinase-16
RS007612	GO:0004222	metalloendopeptidase activity	MF	-1.515691305	-1.272810596	0.134745682	5.700207293	27.871131	3.86542E-06	3.74511E-05	PREDICTED: endothelin-converting enzyme 1-like
RS001994	GO:0004222	metalloendopeptidase activity	MF	2.080190019	2.170868651	-0.068481762	2.794763337	25.37592917	1.28837E-05	0.000116418	PREDICTED: neprilysin-11-like
RS004383	GO:0004222	metalloendopeptidase activity	MF	1.189523129	0.632207527	-0.245245255	6.77526985	24.46696536	1.99557E-05	0.000173572	PREDICTED: insulin-degrading enzyme
RS011631	GO:0004222	metalloendopeptidase activity	MF	-0.899517087	-3.488934247	-1.233320527	6.863876195	23.4516059	3.25112E-05	0.000275414	PREDICTED: zinc metalloproteinase nas-13
RS008393	GO:0004222	metalloendopeptidase activity	MF	-0.717809628	-2.220050675	-0.474940999	5.76534016	22.32685011	5.57733E-05	0.000458448	PREDICTED: membrane metallo-endopeptidase-like 1
RS008394	GO:0004222	metalloendopeptidase activity	MF	-0.720154246	-1.769242461	-0.428915292	7.142277948	20.52081285	0.000132372	0.001022211	PREDICTED: membrane metallo-endopeptidase-like 1
RS005374	GO:0004222	metalloendopeptidase activity	MF	1.001965489	1.612517988	1.627277473	4.057646904	18.23852093	0.000392732	0.002817102	PREDICTED: disintegrin and metalloproteinase domain-containing protein 10
RS011155	GO:0004222	metalloendopeptidase activity	MF	0.093206252	0.990194392	1.040461707	5.730305223	17.6130615	0.000528525	0.0036974	PREDICTED: disintegrin and metalloproteinase domain-containing protein 12
RS007103	GO:0004222	metalloendopeptidase activity	MF	0.966099555	1.623485325	2.240799608	5.854619692	14.51507578	0.002281642	0.014023962	PREDICTED: matrix metalloproteinase-14
RS001042	GO:0004222	metalloendopeptidase activity	MF	0.779135261	1.233045874	0.8952081	5.885498217	13.85999182	0.003102057	0.018439452	PREDICTED: leishmanolysin-like peptidase
RS011280	GO:0004222	metalloendopeptidase activity	MF	1.591307394	0.887559236	1.029215512	7.50047051	13.80643337	0.003180828	0.018864599	PREDICTED: bone morphogenetic protein 1
RS010809	GO:0004222	metalloendopeptidase activity	MF	-0.610912196	-1.435211632	-0.384889955	4.840533802	12.17081443	0.006820351	0.037319766	PREDICTED: matrix metalloproteinase-14
RS004382	GO:0004222	metalloendopeptidase activity	MF	1.179241261	0.87052315	0.083821459	7.357470516	10.82800359	0.012693227	0.064424632	PREDICTED: insulin-degrading enzyme
RS007435	GO:0004222	metalloendopeptidase activity	MF	-0.518093958	-0.176124158	0.718890778	4.068398857	10.80380773	0.012835473	0.065062398	PREDICTED: A disintegrin and metalloproteinase with thrombospondin motifs 3-like
RS015456	GO:0004222	metalloendopeptidase activity	MF	0.479643427	1.099675167	0.588039909	4.57911676	9.105350178	0.0279225	0.127483356	PREDICTED: endothelin-converting enzyme 1-like
RS006149	GO:0004222	metalloendopeptidase activity	MF	0.425385295	1.017385435	0.424609281	6.263579981	8.521504533	0.036378024	0.159219991	PREDICTED: endothelin-converting enzyme 1

RS005515	GO:0004222	metalloendopeptidase activity	MF	-0.623441041	0.371284604	0.662974301	5.867603782	7.649361012	0.053842407	0.218526948	PREDICTED: A disintegrin and metalloproteinase with thrombospondin motifs 7-like
RS004277	GO:0004222	metalloendopeptidase activity	MF	0.603281186	0.947912248	0.514805483	4.881857333	6.614940428	0.085238102	0.312163855	PREDICTED: disintegrin and metalloproteinase domain-containing protein 10
RS010808	GO:0004222	metalloendopeptidase activity	MF	-1.066748432	-0.439692405	0.057366011	5.04170549	6.447268334	0.09176547	0.330945758	PREDICTED: matrix metalloproteinase-14
RS001471	GO:0004222	metalloendopeptidase activity	MF	0.368482727	1.192697613	0.606853095	7.577851262	6.30814262	0.097543855	0.347832459	PREDICTED: papilin
RS001569	GO:0004222	metalloendopeptidase activity	MF	0.308045084	0.73622758	0.459542662	6.524801282	6.07477001	0.108028629	0.374451284	PREDICTED: matrix metalloproteinase-14-like
RS011300	GO:0004222	metalloendopeptidase activity	MF	-0.32700634	1.716647487	1.330848991	0.15000857	5.104455226	0.164306274	0.502220633	PREDICTED: zinc metalloproteinase nas-4-like
RS007616	GO:0004222	metalloendopeptidase activity	MF	-0.822681397	-0.71677253	-0.150257381	3.526121311	4.669530589	0.197657918	0.566752936	PREDICTED: endothelin-converting enzyme 1
RS010807	GO:0004222	metalloendopeptidase activity	MF	-0.282269785	0.32880258	-0.535727775	4.698881667	4.626114682	0.201313245	0.573192112	PREDICTED: matrix metalloproteinase-14
RS002186	GO:0004222	metalloendopeptidase activity	MF	-0.260314957	-0.630647657	0.187422375	5.695464141	4.300685431	0.230772707	0.621360359	PREDICTED: ATP-dependent Clp protease ATP-binding subunit clpX-like, mitochondrial
RS003736	GO:0004222	metalloendopeptidase activity	MF	-1.194589937	-0.431798891	-0.813061389	1.314065193	4.085799201	0.252347169	0.657102535	PREDICTED: endothelin-converting enzyme 1-like
RS005091	GO:0004222	metalloendopeptidase activity	MF	0.701227209	0.89406611	0.441949617	4.553996031	2.693723858	0.441294913	0.886076274	PREDICTED: neprilysin-2-like
RS014397	GO:0004222	metalloendopeptidase activity	MF	0.480369465	0.178435372	0.485940868	4.480855991	2.581675778	0.46071108	0.90528127	PREDICTED: thrombospondin type-1 domain-containing protein 4-like
RS006152	GO:0004222	metalloendopeptidase activity	MF	-0.865654659	0.027245092	0.136994069	1.274235337	2.277091275	0.516923811	0.949359581	PREDICTED: neprilysin-2-like
RS010810	GO:0004222	metalloendopeptidase activity	MF	-0.358762751	-0.077644664	0.155340023	4.065551791	2.125940135	0.54668275	0.964781197	PREDICTED: matrix metalloproteinase-14
RS004741	GO:0004222	metalloendopeptidase activity	MF	-0.076595862	-0.194458268	-0.411891367	5.989768743	2.034663599	0.565243406	0.975457188	PREDICTED: probable cytosolic oligopeptidase A
RS011539	GO:0004222	metalloendopeptidase activity	MF	-0.021072226	-0.388755115	-0.171976696	7.115971996	1.969118185	0.578841018	0.980968915	PREDICTED: AFG3-like protein 2
RS001312	GO:0004222	metalloendopeptidase activity	MF	-0.033986629	-0.254964437	-0.336290402	6.54144214	1.225506456	0.746893534	0.99990777	PREDICTED: membrane-bound transcription factor site-2 protease
RS001810	GO:0004222	metalloendopeptidase activity	MF	0.530782923	0.288478504	0.277891774	2.30206142	1.17347453	0.759372989	0.99990777	PREDICTED: meprin A subunit beta-like
RS001938	GO:0004222	metalloendopeptidase activity	MF	0.029239481	-0.118435749	0.044561105	6.491620321	0.326634061	0.954943918	0.99990777	PREDICTED: mitochondrial intermediate peptidase
RS002797	GO:0004222	metalloendopeptidase activity	MF	-0.019768802	-0.202919516	0.157663977	5.838793507	0.823537964	0.843829173	0.99990777	PREDICTED: ADAM 17-like protease
RS002854	GO:0004222	metalloendopeptidase activity	MF	-0.153356328	-0.305186094	-0.255241961	6.45761334	0.749399973	0.861527552	0.99990777	PREDICTED: mitochondrial inner membrane protease ATP23 homolog
RS003965	GO:0004222	metalloendopeptidase activity	MF	0.117551191	0.260664426	0.315597492	4.177967166	0.964801322	0.809768112	0.99990777	PREDICTED: probable tRNA N6-adenosine threonylcarbamoyltransferase, mitochondrial
RS004342	GO:0004222	metalloendopeptidase activity	MF	-0.154828796	-0.017405825	0.082814369	7.569803176	0.423150773	0.935419641	0.99990777	PREDICTED: paraplegin
RS004860	GO:0004222	metalloendopeptidase activity	MF	-0.123413772	-0.277651068	-0.065629128	5.614653015	0.80260109	0.848844786	0.99990777	PREDICTED: presequence protease, mitochondrial
RS005808	GO:0004222	metalloendopeptidase activity	MF	-0.464347763	-0.040805565	0.434058336	1.115720122	1.530081826	0.675345065	0.99990777	PREDICTED: disintegrin and metalloproteinase domain-containing protein 11
RS006927	GO:0004222	metalloendopeptidase activity	MF	-0.132144206	-0.389608962	-0.208277049	8.726756552	1.108871596	0.774932888	0.99990777	PREDICTED: mitochondrial-processing peptidase subunit beta
RS007399	GO:0004222	metalloendopeptidase activity	MF	-0.364699857	-0.908865172	-0.047056795	0.456572134	1.39097591	0.707651385	0.99990777	PREDICTED: endothelin-converting enzyme 1
RS007948	GO:0004222	metalloendopeptidase activity	MF	-0.203912889	-0.245046606	-0.26251925	7.988518544	0.686641778	0.876341349	0.99990777	PREDICTED: mitochondrial-processing peptidase subunit alpha
RS009310	GO:0004222	metalloendopeptidase activity	MF	0.246866188	0.035611425	-0.077417597	5.349995055	0.572893781	0.902608701	0.99990777	PREDICTED: CAAX prenyl protease 1 homolog
RS010021	GO:0004222	metalloendopeptidase activity	MF	0.277402108	0.237443318	0.057915945	7.543610555	1.01009521	0.798809232	0.99990777	PREDICTED: nardilysin-like
RS010806	GO:0004222	metalloendopeptidase activity	MF	0.08462838	0.368730395	0.029422425	4.602970791	1.641432436	0.650032204	0.99990777	PREDICTED: matrix metalloproteinase-14
RS012218	GO:0004222	metalloendopeptidase activity	MF	0.320776601	0.11036328	0.175227228	5.628810729	0.889944388	0.827853757	0.99990777	PREDICTED: ATP-dependent zinc metalloprotease YME1 homolog
RS013301	GO:0004222	metalloendopeptidase activity	MF	-0.010887464	0.399638577	0.327067467	6.377556594	1.10065881	0.776915047	0.99990777	PREDICTED: metalloendopeptidase OMA1, mitochondrial
RS014193	GO:0004222	metalloendopeptidase activity	MF	0.027574156	0.204578753	-0.001994962	1.103472076	0.167271434	0.982691383	0.99990777	PREDICTED: neprilysin-1
RS015105	GO:0004222	metalloendopeptidase activity	MF	0.23373343	-0.096225565	0.443664791	0.927284795	0.438946496	0.932084597	0.99990777	PREDICTED: organic cation transporter 1
RS011049	GO:0030170	pyridoxal phosphate binding	MF	-2.29908087	3.299439657	8.25278355	7.490502686	408.7578628	2.80601E-88	5.12158E-86	PREDICTED: cysteine sulfinic acid decarboxylase
RS006639	GO:0030170	pyridoxal phosphate binding	MF	-3.083868425	-7.637695989	-8.133777043	4.628300865	186.0851837	4.27786E-40	2.64095E-38	PREDICTED: aromatic-L-amino-acid decarboxylase-like
RS004822	GO:0030170	pyridoxal phosphate binding	MF	-1.15913694	1.950800853	0.602046639	4.964662266	91.76587262	9.14602E-20	2.3037E-18	PREDICTED: mitochondrial amidoxime-reducing component 1-like
RS006640	GO:0030170	pyridoxal phosphate binding	MF	-1.791513421	5.598946144	0.991323579	0.785411688	73.73810476	6.75358E-16	1.25635E-14	PREDICTED: aromatic-L-amino-acid decarboxylase-like
RS006641	GO:0030170	pyridoxal phosphate binding	MF	0	7.485546798	0	0.803640671	72.21979448	1.42834E-15	2.58085E-14	PREDICTED: aromatic-L-amino-acid decarboxylase-like
RS008573	GO:0030170	pyridoxal phosphate binding	MF	-2.260713301	-3.030774322	-1.815246754	3.622901311	46.35220856	4.77314E-10	5.60233E-09	PREDICTED: probable aspartate aminotransferase, cytoplasmic
RS011857	GO:0030170	pyridoxal phosphate binding	MF	-2.520329028	-3.351704626	0.098895726	4.019687658	45.14359349	8.62483E-10	9.90887E-09	PREDICTED: 2-amino-3-ketobutyrate coenzyme A ligase, mitochondrial
RS001674	GO:0030170	pyridoxal phosphate binding	MF	-0.725177387	-0.370012813	0.932400504	6.657705976	30.72741593	9.70131E-07	8.11276E-06	PREDICTED: cystathionine beta-synthase
RS006642	GO:0030170	pyridoxal phosphate binding	MF	-2.419823612	0.944025566	1.645528914	4.053624516	29.85374943	1.48135E-06	1.21538E-05	PREDICTED: aromatic-L-amino-acid decarboxylase
RS010957	GO:0030170	pyridoxal phosphate binding	MF	0.104578349	-0.713610498	-0.868724544	6.502009154	28.08418135	3.48723E-06	2.69933E-05	PREDICTED: ornithine aminotransferase, mitochondrial
RS007624	GO:0030170	pyridoxal phosphate binding	MF	-1.33998228	-3.692172416	0.752205035	1.953523445	27.98010021	3.66714E-06	2.82816E-05	PREDICTED: tyrosine aminotransferase
RS011800	GO:0030170	pyridoxal phosphate binding	MF	-1.018570964	-2.603412306	-0.804726968	3.250067261	27.61409107	4.37655E-06	3.33848E-05	PREDICTED: serine dehydratase-like
RS000627	GO:0030170	pyridoxal phosphate binding	MF	-1.23214239	-1.256196129	-0.507713368	5.68807099	26.33446348	8.11684E-06	5.93977E-05	PREDICTED: 5-aminolevulinate synthase, erythroid-specific, mitochondrial
RS006448	GO:0030170	pyridoxal phosphate binding	MF	-0.438550285	-0.842818516	-1.111661943	7.029395929	20.1090662	0.000161133	0.000940802	PREDICTED: glycogen phosphorylase
RS014437	GO:0030170	pyridoxal phosphate binding	MF	-0.988357721	-0.015003324	-0.20706366	6.1139878	20.07392409	0.000163859	0.000955386	PREDICTED: alanine--glyoxylate aminotransferase 2-like
RS004020	GO:0030170	pyridoxal phosphate binding	MF	0.760635188	0.220093961	-0.261771613	5.591518584	19.6288157	0.00020262	0.001156755	PREDICTED: nicalin
RS012059	GO:0030170	pyridoxal phosphate binding	MF	-0.487537417	-1.155775031	-1.008744238	4.503721781	19.20044033	0.000248509	0.001392227	PREDICTED: serine hydroxymethyltransferase, cytosolic
RS005416	GO:0030170	pyridoxal phosphate binding	MF	-0.412284439	-1.050899627	-2.077424575	2.957561508	17.37798325	0.000590855	0.003035999	PREDICTED: threo-3-hydroxyaspartate ammonia-lyase
RS010509	GO:0030170	pyridoxal phosphate binding	MF	-1.527063033	-1.135110705	-1.09559939	2.940762741	17.22393729	0.00063561	0.003239526	PREDICTED: cysteine sulfinic acid decarboxylase
RS008575	GO:0030170	pyridoxal phosphate binding	MF	-0.895887835	-1.094241053	-0.498680763	6.153628076	14.59558766	0.002196986	0.009674422	PREDICTED: probable aspartate aminotransferase, cytoplasmic
RS008574	GO:0030170	pyridoxal phosphate binding	MF	-1.398204707	-1.439633479	-0.772632276	3.648197129	13.9848864	0.002925797	0.012394044	PREDICTED: probable aspartate aminotransferase, cytoplasmic
RS010970	GO:0030170	pyridoxal phosphate binding	MF	-0.654166537	0.492469445	-0.220477149	4.881203789	13.84299924	0.003126837	0.013126461	PREDICTED: alanine aminotransferase 1
RS010694	GO:0030170	pyridoxal phosphate binding	MF	-1.376784469	-1.151365045	-1.019735694	3.615734954	11.25799445	0.010409554	0.036761977	PREDICTED: glutamate decarboxylase
RS000146	GO:0030170	pyridoxal phosphate binding	MF	0.201922556	-0.781716042	-0.183638845	5.004160987	9.356926226	0.024903162	0.075921187	PREDICTED: serine hydroxymethyltransferase, mitochondrial
RS014200	GO:0030170	pyridoxal phosphate binding	MF	0.368512158	-0.23900377	-0.315539871	4.740713374	8.620559497	0.034785227	0.09929734	PREDICTED: sphingosine-1-phosphate lyase
RS015346	GO:0030170	pyridoxal phosphate binding	MF	-0.035897868	0.497726136	0.291117237	6.601582051	7.299752842	0.062933161	0.158389335	PREDICTED: cystathionine gamma-lyase
RS003797	GO:0030170	pyridoxal phosphate binding	MF	-0.779191865	-0.909904321	-0.8699466	6.927393112	7.246172474	0.064451878	0.1612129	PREDICTED: 4-aminobutyrate aminotransferase, mitochondrial
RS000758	GO:0030170	pyridoxal phosphate binding	MF	0.436016025	0.658391235	0.438858138	7.421745581	7.173549798	0.066567125	0.164996334	PREDICTED: kynurenine--oxoglutarate transaminase 3
RS006445	GO:0030170	pyridoxal phosphate binding	MF	-0.639463442	-0.446387769	0.056703064	3.503852672	5.903016491	0.11642521	0.251028779	PREDICTED: serine palmitoyltransferase 2
RS003690	GO:0030170	pyridoxal phosphate binding	MF	-0.232113403	-0.608329994	-0.182240398	4.819621893	5.891630852	0.117003316	0.251844089	PREDICTED: 2-aminoadipate transaminase-like
RS002083	GO:0030170	pyridoxal phosphate binding	MF	-0.323137174	-0.49516432	0.148595856	8.605719851	5.666822715	0.128994345	0.270039027	PREDICTED: aspartate aminotransferase, mitochondrial
RS013885	GO:0030170	pyridoxal phosphate binding	MF	-0.653620991	-1.203365067	-0.496719101	1.563650614	4.985052582	0.172894945	0.334270639	PREDICTED: tyrosine decarboxylase
RS013883	GO:0030170	pyridoxal phosphate binding	MF	-1.119764452	-0.852715943	0.034262468	6.463866364	4.838736295	0.183993753	0.349694714	PREDICTED: aromatic-L-amino-acid decarboxylase-like
RS002014	GO:0030170	pyridoxal phosphate binding	MF	0.036539526	-0.332351867	0.222033102	8.334994989	3.995693418	0.261929539	0.442664335	PREDICTED: cystathionine gamma-lyase
RS013884	GO:0030170	pyridoxal phosphate binding	MF	-1.483581091	-0.28373006	-0.000921218	2.848064172	2.784924475	0.425987633	0.600838644	PREDICTED: aromatic-L-amino-acid decarboxylase-like
RS004819	GO:0030170	pyridoxal phosphate binding	MF	-0.296041288	-0.224467726	-0.522676724	4.879480613	2.747178566	0.432269238	0.606100955	PREDICTED: mitochondrial amidoxime-reducing component 1-like
RS007076	GO:0030170	pyridoxal phosphate binding	MF	-0.333532028	0.014299236	-0.014087004	5.372273562	2.600146824	0.457463807	0.629994444	PREDICTED: serine palmitoyltransferase 1
RS004039	GO:0030170	pyridoxal phosphate binding	MF	-0.217625409	-0.338436678	0.119231972	2.598033638	1.520372268	0.677576466	0.800421353	PREDICTED: molybdenum cofactor sulfurase 1
RS012079	GO:0030170	pyridoxal phosphate binding	MF	0.239219771	0.02432342	0.187017072	5.848565127	1.497472914	0.682853703	0.803950597	PREDICTED: pyridoxal-dependent decarboxylase domain-containing protein 1
RS014513	GO:0030170	pyridoxal phosphate binding	MF	0.245831721	0.239105658	0.17414925	5.204247819	1.344995027	0.718477293	0.831181996	PREDICTED: ras-related protein Rab-6.2
RS014187	GO:0030170	pyridoxal phosphate binding	MF	0.158474751	0.095400176	0.272547424	5.44957084	0.920998054	0.820357765	0.897490049	PREDICTED: 1-aminocyclopropane-1-carboxylate synthase-like protein 1
RS008879	GO:0030170	pyridoxal phosphate binding	MF	0.109355227	0.022608977	-0.119419284	4.359906481	0.776449349	0.855091344	0.920507768	PREDICTED: proline synthase co-transcribed bacterial homolog protein
RS006226	GO:0030170	pyridoxal phosphate binding	MF	-0.000697599	-0.093453208	-0.154614222	4.887176637	0.380294869	0.944282348	0.970591928	PREDICTED: alanine--glyoxylate aminotransferase 2, mitochondrial
RS014512	GO:0030170	pyridoxal phosphate binding	MF	-0.029889403	-0.146982534	-0.072958369	7.559121721	0.32842394	0.954596993	0.97588441	PREDICTED: cysteine desulfurase, mitochondrial
RS003228	GO:0046942	carboxylic acid transport	BP	-0.561103954	2.12048168	1.83683218	5.406987214	114.6440514	1.09829E-24	3.71824E-23	PREDICTED: b(0,+)-type amino acid transporter 1-like
RS013974	GO:0046942	carboxylic acid transport	BP	1.308057058	2.315023189	0.876327879	6.127768048	111.7248777	4.66769E-24	1.53887E-22	PREDICTED: monocarboxylate transporter 10
RS012045	GO:0046942	carboxylic acid transport	BP	-2.417547613	-3.846942398	-2.948472949	3.138267013	95.12499238	1.73561E-20	4.58246E-19	PREDICTED: sodium-independent sulfate anion transporter
RS006315	GO:0046942	carboxylic acid transport	BP	-2.445857294	-4.975199992	0.147442902	3.069628072	71.7469443	1.80352E-15	3.24017E-14	PREDICTED: bumetanide-sensitive sodium-(potassium)-chloride cotransporter
RS008617	GO:0046942	carboxylic acid transport	BP	-1.758654905	-1.933395291	0.119722157	4.240860888	52.46640405	2.38231E-11	3.09308E-10	PREDICTED: proton-coupled amino acid transporter 1
RS006021	GO:0046942	carboxylic acid transport	BP	-1.978389488	-0.987147201	0.23457041	5.767022167	52.1310105	2.80858E-11	3.63527E-10	PREDICTED: cationic amino acid transporter 3
RS009380	GO:0046942	carboxylic acid transport	BP	1.181487238	0.230316163	-1.011984835	4.090504935	45.23692145	8.23976E-10	9.50289E-09	PREDICTED: b(0,+)-type amino acid transporter 1-like
RS001007	GO:0046942	carboxylic acid transport	BP	-0.057453873	2.550863666	4.593751253	0.34449975	41.36132174	5.4813E-09	5.84023E-08	PREDICTED: sodium-dependent nutrient amino acid transporter 1-like
RS005851	GO:0046942	carboxylic acid transport	BP	0.526397294	1.41413322	1.673681755	7.829535198	40.74316203	7.413E-09	7.77347E-08	PREDICTED: proton-coupled amino acid transporter 4
RS006814	GO:0046942	carboxylic acid transport	BP	-0.514796382	1.014347124	0.975336891	4.041760153	30.97725487	8.59474E-07	7.23543E-06	PREDICTED: large neutral amino acids transporter small subunit 2
RS006320	GO:0046942	carboxylic acid transport	BP	1.668458777	1.549661536	0.932473469	6.183206129	30.51118393	1.07731E-06	8.96741E-06	PREDICTED: bumetanide-sensitive sodium-(potassium)-chloride cotransporter
RS007713	GO:0046942	carboxylic acid transport	BP	-1.593472957	-0.392083561	-0.180235411	5.938924251	30.40817878	1.13245E-06	9.40771E-06	PREDICTED: ATP-binding cassette sub-family D member 2
RS000193	GO:0046942	carboxylic acid transport	BP	-0.612906575	0.552707825	0.864925878	6.395375097	30.19397061	1.25629E-06	1.03772E-05	PREDICTED: b(0,+)-type amino acid transporter 1
RS004393	GO:0046942	carboxylic acid transport	BP	-1.227790861	-2.264108903	-0.817189459	2.289519889	20.23673107	0.000151606	0.000890546	PREDICTED: putative tricarboxylate transport protein, mitochondrial
RS000837	GO:0046942	carboxylic acid transport	BP	-1.260815808	-0.582381283	0.014148923	5.012164404	17.85146996	0.000471988	0.002476758	PREDICTED: excitatory amino acid transporter 1-like
RS011194	GO:0046942	carboxylic acid transport	BP	-0.043178438	0.101493976	0.864859407	6.406781775	17.21343829	0.00063878	0.003251735	PREDICTED: mitochondrial pyruvate carrier 2-like
RS000849	GO:0046942	carboxylic acid transport	BP	-0.753175709	-1.571391853	-1.058453667	2.708513766	17.06922787	0.000683946	0.003464852	PREDICTED: monocarboxylate transporter 9-like
RS000927	GO:0046942	carboxylic acid transport	BP	-2.570527298	-2.804707147	-0.755163456	0.667619183	16.35029216	0.000961058	0.004715063	PREDICTED: b(0,+)-type amino acid transporter 1-like
RS013870	GO:0046942	carboxylic acid transport	BP	-1.185159015	-0.789079424	-0.942978819	4.024142004	15.86729932	0.001207277	0.005765813	PREDICTED: sodium- and chloride-dependent glycine transporter 1-like
RS000928	GO:0046942	carboxylic acid transport	BP	-2.818387407	-2.387215456	-1.195316578	0.324906115	15.29049888	0.001584497	0.007309578	B(0,+)-type amino acid transporter 1
RS005708	GO:0046942	carboxylic acid transport	BP	-2.57707626	-0.767810406	-0.121337824	1.208165742	14.61680361	0.002175202	0.009598631	PREDICTED: sodium- and chloride-dependent glycine transporter 1-like
RS008723	GO:0046942	carboxylic acid transport	BP	0.765724899	0.492155106	-0.726953396	2.604372289	14.25219643	0.002581214	0.011140444	PREDICTED: excitatory amino acid transporter
RS005241	GO:0046942	carboxylic acid transport	BP	-1.24648029	-0.946331544	-0.692073388	4.41212653	12.5745733	0.005653054	0.021933014	PREDICTED: high-affinity choline transporter 1
RS002985	GO:0046942	carboxylic acid transport	BP	-0.571114377	-0.476136182	-0.859409917	5.548946497	12.07709757	0.007123669	0.026732864	PREDICTED: Y+L amino acid transporter 2
RS003950	GO:0046942	carboxylic acid transport	BP	-0.587671115	-0.868990799	-0.714405964	4.844171479	10.0941278	0.017782729	0.057765203	PREDICTED: cationic amino acid transporter 2
RS009139	GO:0046942	carboxylic acid transport	BP	0.649351007	1.292845053	0.336142374	2.039377777	9.031613663	0.028873505	0.085540561	PREDICTED: Y+L amino acid transporter 2
RS007859	GO:0046942	carboxylic acid transport	BP	-1.037355681	-0.953164057	-1.137934678	2.631254112	8.895408464	0.0307143	0.08987358	PREDICTED: proton-coupled amino acid transporter 4
RS006017	GO:0046942	carboxylic acid transport	BP	-0.226333875	0.829010789	0.173010283	3.457042927	8.42538268	0.037991671	0.106302401	PREDICTED: cationic amino acid transporter 2
RS002298	GO:0046942	carboxylic acid transport	BP	0.444685109	0.888871791	0.46931162	5.877833769	8.218233291	0.041710358	0.114594295	PREDICTED: battenin
RS012784	GO:0046942	carboxylic acid transport	BP	-0.964188065	-1.053670411	-1.487092291	6.693074429	8.119987423	0.043595935	0.118762068	PREDICTED: excitatory amino acid transporter-like
RS006810	GO:0046942	carboxylic acid transport	BP	-0.692918241	-0.709642301	-0.469276201	5.552917252	7.276110601	0.063598968	0.159523084	PREDICTED: Y+L amino acid transporter 2
RS015105	GO:0046942	carboxylic acid transport	BP	-0.678666411	-2.224992259	-0.562865466	0.559101899	7.185153044	0.066224707	0.164438873	PREDICTED: organic cation transporter 1

RS009471	GO:0046942	carboxylic acid transport	BP	0.454440582	0.068570398	0.92902968	1.975531925	6.680170813	0.082821497	0.194672252	PREDICTED: potassium voltage-gated channel protein Shal
RS002769	GO:0046942	carboxylic acid transport	BP	-0.180596666	-0.510025269	-0.494314256	5.266272897	5.147923967	0.161280396	0.318264698	PREDICTED: mitochondrial 2-oxoglutarate/malate carrier protein
RS006076	GO:0046942	carboxylic acid transport	BP	-0.399500112	-0.234112198	-0.791572834	3.218719557	5.135126595	0.162165734	0.319411206	PREDICTED: cationic amino acid transporter 4
RS002889	GO:0046942	carboxylic acid transport	BP	-0.128703142	0.3529612	0.286577104	4.360050122	4.605415706	0.203077987	0.373149135	PREDICTED: bifunctional arginine demethylase and lysyl-hydroxylase PSR
RS000049	GO:0046942	carboxylic acid transport	BP	-0.167016261	-0.078808593	0.315739518	4.19490186	3.918242209	0.270428905	0.452114912	PREDICTED: protein I'm not dead yet-like
RS007790	GO:0046942	carboxylic acid transport	BP	0.13034852	0.225712601	0.386180211	4.625198528	2.606288996	0.456388071	0.628856824	PREDICTED: mitochondrial glutamate carrier 1-like
RS004471	GO:0046942	carboxylic acid transport	BP	0.302505199	0.1016203	-0.272900349	3.077100879	2.396738775	0.494241988	0.660492741	PREDICTED: Y+L amino acid transporter 2
RS002882	GO:0046942	carboxylic acid transport	BP	0.362043543	0.12287268	0.14951259	3.567153224	1.277605364	0.734457649	0.842342193	PREDICTED: putative sodium-coupled neutral amino acid transporter 7
RS013172	GO:0046942	carboxylic acid transport	BP	0.064707769	0.050177661	0.256276698	4.54520608	0.870549691	0.832528393	0.905630734	PREDICTED: N-acetyl-D-glucosamine kinase
RS006019	GO:0046942	carboxylic acid transport	BP	0.016800687	-0.569398535	-0.206623396	0.539126613	0.775635116	0.855285471	0.920559373	PREDICTED: cationic amino acid transporter 2
RS014940	GO:0046942	carboxylic acid transport	BP	0.00473671	-0.350880149	-0.354295712	0.736422598	0.582482205	0.900430699	0.946555742	PREDICTED: potassium voltage-gated channel protein Shal
RS004110	GO:0046942	carboxylic acid transport	BP	-0.109326082	-0.245764423	-0.393853677	2.856144774	0.536964028	0.910698682	0.95276119	PREDICTED: phospholipase A2 inhibitor and Ly6/PLAUR domain-containing protein
RS002585	GO:0046942	carboxylic acid transport	BP	0.061337763	0.015683494	0.119579633	4.655608643	0.205533408	0.976691112	0.987602807	PREDICTED: mitochondrial pyruvate carrier 1
RS008706	GO:0046942	carboxylic acid transport	BP	0.063443874	0.058144534	-0.01579188	5.633533699	0.196321115	0.978180999	0.988383465	PREDICTED: ATP-binding cassette sub-family D member 3
RS005614	GO:0046942	carboxylic acid transport	BP	-0.026120006	0.069729297	0.030053445	4.472662817	0.146084989	0.985784124	0.992693667	PREDICTED: mitochondrial folate transporter/carrier
RS000375	GO:0016810	hydrolase activity, acting on carbon-nitrogen (but not peptide) bonds	MF	-0.34491686	5.884963026	2.082677396	7.234288024	328.8906103	5.55E-71	7.76E-69	putative allantoinase 1
RS014580	GO:0016810	hydrolase activity, acting on carbon-nitrogen (but not peptide) bonds	MF	-2.976523608	-6.808866752	-1.942701116	5.214122185	156.927034	8.44E-34	4.17E-32	PREDICTED: kynurenine formamidase-like
RS012361	GO:0016810	hydrolase activity, acting on carbon-nitrogen (but not peptide) bonds	MF	-0.272408132	-0.554085634	1.369334256	6.34819019	93.14482576	4.62E-20	1.18E-18	PREDICTED: vanin-like protein 1
RS004205	GO:0016810	hydrolase activity, acting on carbon-nitrogen (but not peptide) bonds	MF	1.324987867	5.035657228	-0.333819443	2.580237392	81.31706065	1.60E-17	3.36E-16	AAEL005685-PA
RS004535	GO:0016810	hydrolase activity, acting on carbon-nitrogen (but not peptide) bonds	MF	-0.802770311	1.915930027	-0.207346837	3.634266563	78.09649306	7.86E-17	1.57E-15	PREDICTED: C->U-editing enzyme APOBEC-1
RS008819	GO:0016810	hydrolase activity, acting on carbon-nitrogen (but not peptide) bonds	MF	-1.032692902	-2.222642533	-1.657320465	5.534608709	75.39402177	2.98E-16	5.71E-15	PREDICTED: glutaminase liver isoform, mitochondrial
RS008510	GO:0016810	hydrolase activity, acting on carbon-nitrogen (but not peptide) bonds	MF	-1.907443864	0.513471025	2.887314324	3.573724567	74.77986444	4.04E-16	7.62E-15	PREDICTED: GTP cyclohydrolase 1
RS010041	GO:0016810	hydrolase activity, acting on carbon-nitrogen (but not peptide) bonds	MF	-0.841304888	-1.311133394	1.016447212	5.056278579	65.67682339	3.59E-14	5.86E-13	PREDICTED: peptidoglycan-recognition protein LC-like
RS013013	GO:0016810	hydrolase activity, acting on carbon-nitrogen (but not peptide) bonds	MF	-1.540094428	-1.189185544	1.548167541	3.519055593	65.31696378	4.29E-14	6.96E-13	PREDICTED: peptidoglycan-recognition protein LB-like
RS011394	GO:0016810	hydrolase activity, acting on carbon-nitrogen (but not peptide) bonds	MF	-1.130512888	-4.311859926	-0.867929923	2.581686454	42.35833137	3.37E-09	3.66E-08	PREDICTED: hydroxyacyl-coenzyme A dehydrogenase, mitochondrial-like
RS005087	GO:0016810	hydrolase activity, acting on carbon-nitrogen (but not peptide) bonds	MF	-1.968225029	0.898040261	2.102745618	2.388969678	39.77259646	1.19E-08	1.22E-07	PREDICTED: GTP cyclohydrolase 1
RS001234	GO:0016810	hydrolase activity, acting on carbon-nitrogen (but not peptide) bonds	MF	-3.049313173	1.196358565	1.454889377	7.193983845	38.01690606	2.80E-08	2.73E-07	chitin deacetylase 4
RS013012	GO:0016810	hydrolase activity, acting on carbon-nitrogen (but not peptide) bonds	MF	-1.470953782	-1.559057804	-1.338853711	3.899322122	34.27053677	1.74E-07	1.56E-06	PREDICTED: peptidoglycan-recognition protein LB-like
RS003758	GO:0016810	hydrolase activity, acting on carbon-nitrogen (but not peptide) bonds	MF	-0.559608852	2.208187894	1.597392533	6.933108648	31.62779144	6.27E-07	5.36E-06	chitin deacetylase 3
RS014930	GO:0016810	hydrolase activity, acting on carbon-nitrogen (but not peptide) bonds	MF	0.323746001	-0.667582543	-0.725755425	6.121124277	29.05530974	2.18E-06	1.74E-05	PREDICTED: bifunctional purine biosynthesis protein PURH
RS015186	GO:0016810	hydrolase activity, acting on carbon-nitrogen (but not peptide) bonds	MF	-1.21066846	0.888184276	1.336908068	10.2190989	26.71453317	6.76E-06	5.01E-05	chitin deacetylase 1
RS004692	GO:0016810	hydrolase activity, acting on carbon-nitrogen (but not peptide) bonds	MF	-1.533477303	-1.581672673	-0.440490513	3.98590693	25.83868371	1.03E-05	7.44E-05	PREDICTED: histone deacetylase 4
RS005123	GO:0016810	hydrolase activity, acting on carbon-nitrogen (but not peptide) bonds	MF	0.781802156	0.484030121	-1.078810264	3.903771807	24.55032113	1.92E-05	0.00013237	PREDICTED: kynurenine formamidase-like
RS000970	GO:0016810	hydrolase activity, acting on carbon-nitrogen (but not peptide) bonds	MF	-1.826169573	-1.767109952	-3.471752553	2.26650892	22.95978906	4.12E-05	0.000268372	PREDICTED: alkaline ceramidase
RS005544	GO:0016810	hydrolase activity, acting on carbon-nitrogen (but not peptide) bonds	MF	-0.308180157	0.689248167	-0.738011841	4.466023101	22.07108314	6.30E-05	0.000399001	PREDICTED: beta-ureidopropionase
RS005296	GO:0016810	hydrolase activity, acting on carbon-nitrogen (but not peptide) bonds	MF	-0.666577041	-0.650920673	0.350048094	4.593415922	21.99923013	6.53E-05	0.000411529	PREDICTED: protein kinase C, brain isozyme
RS015187	GO:0016810	hydrolase activity, acting on carbon-nitrogen (but not peptide) bonds	MF	0.013748187	1.340727689	1.795231312	10.35011632	20.46140263	0.000136183	0.000809002	chitin deacetylase 1 precursor
RS014120	GO:0016810	hydrolase activity, acting on carbon-nitrogen (but not peptide) bonds	MF	-0.922827123	-1.289252361	-1.238323899	2.633512953	15.71916541	0.001294661	0.006118183	PREDICTED: nicotinamidase
RS006044	GO:0016810	hydrolase activity, acting on carbon-nitrogen (but not peptide) bonds	MF	-0.676228211	-0.513648981	0.462911231	3.973647365	15.33152104	0.001554179	0.007182874	PREDICTED: allantoinase, mitochondrial-like
RS006022	GO:0016810	hydrolase activity, acting on carbon-nitrogen (but not peptide) bonds	MF	-1.813369421	-1.750081698	-0.062538905	2.776091628	15.04967814	0.001774684	0.008054187	PREDICTED: beta-ureidopropionase
RS015068	GO:0016810	hydrolase activity, acting on carbon-nitrogen (but not peptide) bonds	MF	-1.101346708	-1.40826429	-0.609149078	2.183952585	13.86326014	0.003097313	0.013028576	PREDICTED: vanin-like protein 1
RS009242	GO:0016810	hydrolase activity, acting on carbon-nitrogen (but not peptide) bonds	MF	0.682073862	0.906384828	-0.119195863	3.436386452	13.55373124	0.003580051	0.014826428	PREDICTED: putative N-acetylglucosamine-6-phosphate deacetylase
RS011903	GO:0016810	hydrolase activity, acting on carbon-nitrogen (but not peptide) bonds	MF	0.432407009	-0.458087969	-0.635284859	4.879343122	10.81435958	0.012773247	0.043832773	PREDICTED: CAD protein
RS004919	GO:0016810	hydrolase activity, acting on carbon-nitrogen (but not peptide) bonds	MF	-0.084781076	0.421100466	0.603685917	4.476790205	9.839099022	0.019984553	0.063503274	PREDICTED: nitrilase homolog 1-like
RS015067	GO:0016810	hydrolase activity, acting on carbon-nitrogen (but not peptide) bonds	MF	-1.545741566	-1.369518026	-0.217080276	1.014262688	9.345369419	0.025034565	0.076284856	PREDICTED: vanin-like protein 1
RS007025	GO:0016810	hydrolase activity, acting on carbon-nitrogen (but not peptide) bonds	MF	-0.646756609	-0.209658007	0.290941208	7.803114963	9.184468997	0.026936199	0.08082785	PREDICTED: fumarate hydratase, mitochondrial-like
RS007024	GO:0016810	hydrolase activity, acting on carbon-nitrogen (but not peptide) bonds	MF	-0.128844159	0.682616829	-0.186562737	3.98321222	8.695142083	0.033631128	0.09676729	PREDICTED: arginase, hepatic
RS006460	GO:0016810	hydrolase activity, acting on carbon-nitrogen (but not peptide) bonds	MF	-1.355855211	-0.229188099	0.081221757	1.62049498	7.749984171	0.051471499	0.135585036	CG9380
RS003177	GO:0016810	hydrolase activity, acting on carbon-nitrogen (but not peptide) bonds	MF	0.737615696	0.712418457	0.180131171	3.012166732	6.812877803	0.078107285	0.186268348	PREDICTED: protein N-terminal glutamine amidohydrolase
RS007649	GO:0016810	hydrolase activity, acting on carbon-nitrogen (but not peptide) bonds	MF	0.846027187	0.412689701	0.198143485	7.278084726	6.794919147	0.078729752	0.18730164	PREDICTED: dihydropyrimidinase
RS008195	GO:0016810	hydrolase activity, acting on carbon-nitrogen (but not peptide) bonds	MF	0.533863066	0.426337346	0.621528576	6.350472863	6.759324797	0.079977665	0.189622234	PREDICTED: NAD-dependent protein deacylase sirtuin-5, mitochondrial
RS015377	GO:0016810	hydrolase activity, acting on carbon-nitrogen (but not peptide) bonds	MF	0.064687264	-0.135391647	-0.540226237	5.050775931	6.503445798	0.089526709	0.207299021	PREDICTED: probable glutamine-dependent NAD(+) synthetase
RS003658	GO:0016810	hydrolase activity, acting on carbon-nitrogen (but not peptide) bonds	MF	-0.041216828	-0.331006227	-0.650832232	8.423732837	6.322317544	0.096939502	0.219534001	PREDICTED: AMP deaminase 2
RS008683	GO:0016810	hydrolase activity, acting on carbon-nitrogen (but not peptide) bonds	MF	-0.685747102	-0.486113188	-0.249052476	4.253825687	5.276867736	0.152610471	0.305512044	PREDICTED: aminoacylase-1-like
RS002645	GO:0016810	hydrolase activity, acting on carbon-nitrogen (but not peptide) bonds	MF	0.149218139	-0.965306677	-0.005280552	1.52336568	5.253108871	0.154174188	0.307637209	PREDICTED: homeobox protein B-H1-like
RS014621	GO:0016810	hydrolase activity, acting on carbon-nitrogen (but not peptide) bonds	MF	0.323635837	0.220273567	0.59271354	5.78002931	4.621341853	0.201718896	0.371769495	PREDICTED: tRNA-specific adenosine deaminase 1
RS003665	GO:0016810	hydrolase activity, acting on carbon-nitrogen (but not peptide) bonds	MF	0.159558316	0.432738932	0.516913502	4.027816739	4.425603816	0.219023029	0.392595507	PREDICTED: tRNA-specific adenosine deaminase 2
RS015378	GO:0016810	hydrolase activity, acting on carbon-nitrogen (but not peptide) bonds	MF	-0.11464752	-0.47318258	-0.790743101	2.363076796	4.098614325	0.251010572	0.429864991	PREDICTED: probable glutamine-dependent NAD(+) synthetase
RS006163	GO:0016810	hydrolase activity, acting on carbon-nitrogen (but not peptide) bonds	MF	-0.188001786	-0.186486274	0.219195614	6.678644856	3.786521228	0.285457836	0.46836187	PREDICTED: nitrilase homolog 1-like
RS011308	GO:0016810	hydrolase activity, acting on carbon-nitrogen (but not peptide) bonds	MF	0.354601056	-0.027381385	0.181923114	6.868671023	3.772230039	0.287132809	0.470118382	PREDICTED: guanine deaminase
RS008767	GO:0016810	hydrolase activity, acting on carbon-nitrogen (but not peptide) bonds	MF	-0.071185743	-0.285368576	0.183550764	4.880406744	3.670316078	0.299335164	0.481890203	PREDICTED: histone deacetylase 3
RS011215	GO:0016810	hydrolase activity, acting on carbon-nitrogen (but not peptide) bonds	MF	0.144642587	-0.292630102	-0.045170464	4.999931895	3.644097085	0.302548453	0.485078958	PREDICTED: nucleolin-like
RS011390	GO:0016810	hydrolase activity, acting on carbon-nitrogen (but not peptide) bonds	MF	0.252324079	0.550359201	0.410388975	3.310648883	3.424796226	0.330647576	0.512513312	PREDICTED: peptide deformylase, mitochondrial-like
RS010779	GO:0016810	hydrolase activity, acting on carbon-nitrogen (but not peptide) bonds	MF	0.212439189	-0.035024314	-0.595729899	2.244190775	3.227818371	0.357815991	0.538894287	PREDICTED: histone deacetylase 8
RS012446	GO:0016810	hydrolase activity, acting on carbon-nitrogen (but not peptide) bonds	MF	-0.33520903	-0.326059321	0.041645679	5.694017303	2.740242498	0.433431766	0.607177439	PREDICTED: NAD-dependent protein deacetylase sirtuin-3, mitochondrial
RS002759	GO:0016810	hydrolase activity, acting on carbon-nitrogen (but not peptide) bonds	MF	0.028967615	0.090840031	0.328940162	5.233792198	2.466480112	0.481379347	0.650551721	PREDICTED: bifunctional methylenetetrahydrofolate dehydrogenase/cyclohydrolase, mitochondrial
RS007677	GO:0016810	hydrolase activity, acting on carbon-nitrogen (but not peptide) bonds	MF	-0.031540972	0.139528929	0.298765141	5.192452988	2.297748506	0.512952371	0.67624015	PREDICTED: omega-amidase NIT2
RS010270	GO:0016810	hydrolase activity, acting on carbon-nitrogen (but not peptide) bonds	MF	-0.499816687	-0.179901857	-0.776722233	3.403430526	2.21634321	0.528736447	0.688401407	PREDICTED: protein N-terminal asparagine amidohydrolase
RS002260	GO:0016810	hydrolase activity, acting on carbon-nitrogen (but not peptide) bonds	MF	-0.844003423	-0.819264903	-0.423327512	1.50342137	2.175127996	0.536863981	0.694443515	PREDICTED: probable imidazolonepropionase
RS011393	GO:0016810	hydrolase activity, acting on carbon-nitrogen (but not peptide) bonds	MF	-0.041730957	-0.230300536	0.07476639	6.804211163	2.01329809	0.569651229	0.720350873	PREDICTED: hydroxyacyl-coenzyme A dehydrogenase, mitochondrial-like
RS009146	GO:0016810	hydrolase activity, acting on carbon-nitrogen (but not peptide) bonds	MF	0.273209024	0.243574118	0.076497435	5.63647883	2.001763487	0.572040767	0.721944225	PREDICTED: breast cancer metastasis-suppressor 1-like protein
RS002596	GO:0016810	hydrolase activity, acting on carbon-nitrogen (but not peptide) bonds	MF	0.185252017	0.028392534	0.351365092	3.868060209	1.711535085	0.634372335	0.771080465	PREDICTED: cytidine deaminase-like
RS012536	GO:0016810	hydrolase activity, acting on carbon-nitrogen (but not peptide) bonds	MF	0.047139577	-0.258366711	-0.192122824	5.403598014	1.660854375	0.645671538	0.77814233	PREDICTED: histone deacetylase Rpd3
RS007077	GO:0016810	hydrolase activity, acting on carbon-nitrogen (but not peptide) bonds	MF	-0.04909779	-0.016114303	-0.376194193	4.092885597	1.559472702	0.668613615	0.793714758	PREDICTED: double-stranded RNA-specific editase 1
RS013047	GO:0016810	hydrolase activity, acting on carbon-nitrogen (but not peptide) bonds	MF	0.231685496	0.106278735	0.095579367	5.899186066	1.241080553	0.743169287	0.848450098	PREDICTED: NAD-dependent protein deacetylase sirtuin-2
RS001074	GO:0016810	hydrolase activity, acting on carbon-nitrogen (but not peptide) bonds	MF	0.209133952	0.10331734	-0.085594621	2.799301808	0.800021453	0.849461902	0.916575594	5-hydroxyisourate hydrolase
RS015233	GO:0016810	hydrolase activity, acting on carbon-nitrogen (but not peptide) bonds	MF	-0.028168318	0.124444095	-0.146279858	3.77098604	0.6887061	0.875857119	0.933052322	PREDICTED: kynurenine formamidase
RS005405	GO:0016810	hydrolase activity, acting on carbon-nitrogen (but not peptide) bonds	MF	-0.387669428	-0.565118451	0.210226756	1.222213754	0.652216457	0.884381294	0.938003319	PREDICTED: peptidoglycan-recognition protein LC-like
RS003714	GO:0016810	hydrolase activity, acting on carbon-nitrogen (but not peptide) bonds	MF	0.073593867	0.045834412	0.154226165	4.6890794	0.411422784	0.937872837	0.967685606	PREDICTED: deoxycytidylate deaminase
RS002966	GO:0006979	response to oxidative stress	BP	0.807999528	6.460241315	5.118879977	6.206668189	256.2899719	2.85319E-55	3.43756E-53	PREDICTED: peroxidase
RS009320	GO:0006979	response to oxidative stress	BP	-2.884845028	-0.020862095	5.230683542	4.703088105	253.4010396	1.20285E-54	1.41004E-52	PREDICTED: superoxide dismutase [Cu-Zn]-like
RS012299	GO:0006979	response to oxidative stress	BP	0.162985364	4.509300165	1.798722556	5.221412597	187.5825383	2.03143E-40	1.43658E-38	Peroxidasin-like protein
RS012128	GO:0006979	response to oxidative stress	BP	1.523930405	6.662919141	2.066133643	3.584635144	166.9023589	5.93422E-36	3.44715E-34	Chorion peroxidase
RS009222	GO:0006979	response to oxidative stress	BP	0.466049588	4.033329864	2.253088186	5.271113036	100.0319955	1.52973E-21	4.77336E-20	Peroxidasin-like
RS010587	GO:0006979	response to oxidative stress	BP	-0.073093498	2.36964901	2.374680605	6.180432942	96.27626382	9.81787E-21	2.95718E-19	PREDICTED: dual oxidase maturation factor 1
RS004290	GO:0006979	response to oxidative stress	BP	0.667850135	1.323655158	3.117631032	4.286424881	54.31478538	9.61345E-12	1.67681E-10	PREDICTED: peroxidase-like
RS001654	GO:0006979	response to oxidative stress	BP	-3.194844089	-4.83623054	-0.727136042	3.630636751	46.95079738	3.56038E-10	5.43752E-09	PREDICTED: catalase
RS014619	GO:0006979	response to oxidative stress	BP	0.353947555	2.007224757	0.385418818	6.238045445	41.12141437	6.16273E-09	8.44099E-08	PREDICTED: dual oxidase
RS007455	GO:0006979	response to oxidative stress	BP	-0.77532182	-0.627371757	1.27736906	4.392216644	37.83412897	3.0645E-08	3.93248E-07	PREDICTED: glutathione peroxidase 6
RS001672	GO:0006979	response to oxidative stress	BP	1.208071687	1.027429099	-0.580522305	5.007937909	27.17087702	5.42117E-06	5.16023E-05	PREDICTED: catalase
RS004201	GO:0006979	response to oxidative stress	BP	-1.155218486	3.110521082	2.680638544	0.84721977	25.12159031	1.45625E-05	0.000130053	PREDICTED: peroxidase
RS007454	GO:0006979	response to oxidative stress	BP	-0.535903331	-0.95556464	1.260192704	2.793782052	24.87535072	1.63954E-05	0.00014493	Glutathione peroxidase 6
RS013770	GO:0006979	response to oxidative stress	BP	-1.247308681	0.14432905	1.096599722	5.766279891	24.12093326	2.35691E-05	0.000202697	PREDICTED: peroxidase-like
RS006683	GO:0006979	response to oxidative stress	BP	-2.138846935	0.841419709	0.351318347	3.204951472	20.97931603	0.000106323	0.000836439	PREDICTED: peroxiredoxin-6-like
RS013365	GO:0006979	response to oxidative stress	BP	-0.032426315	-1.189999976	-2.078019007	7.216223147	19.82050069	0.000184919	0.001387642	PREDICTED: peptide methionine sulfoxide reductase
RS004203	GO:0006979	response to oxidative stress	BP	-0.283218866	3.300380803	2.915469187	0.463856894	18.67883599	0.000318549	0.00230917	PREDICTED: peroxidase
RS006166	GO:0006979	response to oxidative stress	BP	0.459896408	1.361902973	0.294940041	4.84256352	17.91948131	0.000456992	0.003231729	PREDICTED: peroxidasin homolog
RS014190	GO:0006979	response to oxidative stress	BP	-0.578216815	-1.519924339	-0.99177885	9.035509083	17.24592229	0.000629022	0.004332894	PREDICTED: probable phospholipid hydroperoxide glutathione peroxidase
RS006581	GO:0006979	response to oxidative stress	BP	-0.677014247	-1.267505539	-0.937057338	5.838897263	16.12849272	0.00106723	0.007056301	PREDICTED: copper chaperone for superoxide dismutase
RS006807	GO:0006979	response to oxidative stress	BP	-0.12837582	-0.716605346	-0.991282865	7.447282818	9.8430759	0.019948245	0.095218842	PREDICTED: peroxiredoxin-6-like
RS001584	GO:0006979	response to oxidative stress	BP	0.895336184	1.270102176	0.129810273	6.410473584	9.812854042	0.020225801	0.096402243	PREDICTED: superoxide dismutase [Cu-Zn], chloroplastic-like
RS007655	GO:0006979	response to oxidative stress	BP	-0.177145336	-1.153797445	-0.750870964	8.617236745	8.632668942	0.034595247	0.152852063	PREDICTED: peroxiredoxin 1
RS002298	GO:0006979	response to oxidative stress	BP	0.546327906	0.941506695	-0.112778408	6.616725781	8.56891699	0.035606938	0.156471002	PREDICTED: battenin
RS013005	GO:0006979	response to oxidative stress	BP	-0.216007548	-0.956243103	-0.794335656	9.592037722	8.34821377	0.039337533	0.169658596	PREDICTED: superoxide dismutase [Cu-Zn]
RS001583	GO:0006979	response to oxidative stress	BP	-1.161566801	-2.497842489	-0.147212796	4.345535541	7.026554096	0.071056202	0.271776396	PREDICTED: superoxide dismutase [Cu-Zn]
RS000508	GO:0006979	response to oxidative stress	BP	-0.502317595	-1.067438271	-0.543592665	7.670787121	6.773929239	0.079463352	0.296608472	PREDICTED: microsomal glutathione S-transferase 3
RS002951	GO:0006979	response to oxidative stress	BP	0.52326563	0.689732975	-0.263965767	8.903142044	5.694396	0.127463013	0.423416924	PREDICTED: dual oxidase 2-like
RS013725	GO:0006979	response to oxidative stress	BP	-0.646328106	-0.68580797	0.619918472	1.397708696	5.61919647	0.131680643	0.432902609	PREDICTED: forkhead box protein O
RS004133	GO:0006979	response to oxidative stress	BP	-0.783712089	-0.638892552	-0.334755882	7.385032478	5.17848178	0.159184744	0.491297886	superoxide dismutase
RS013590	GO:0006979	response to oxidative stress	BP	1.259105005	1.788910613	1.491782581	1.28874968	5.082205733	0.16587566	0.505003762	PREDICTED: zinc finger and SCAN domain-containing protein 12
RS002757	GO:0006979	response to oxidative stress	BP	-0.348780035	-0.784728642	-0.550000388	7.383764883	4.950578976	0.175451971	0.525306269	mitochondrial manganese superoxide dismutase

RS004289	GO:0006979	response to oxidative stress	BP	0.581844762	0.613291671	-0.135700525	8.593804498	4.318543449	0.229058187	0.617849323 PREDICTED: peroxidase-like
RS013721	GO:0006979	response to oxidative stress	BP	-0.136156837	-0.739541345	-0.438621181	6.945958821	4.223395584	0.238329957	0.633888515 PREDICTED: peroxiredoxin-2
RS013874	GO:0006979	response to oxidative stress	BP	0.013903141	-0.479297983	-0.464220577	6.451119994	3.838149087	0.279479827	0.695200058 PREDICTED: peroxiredoxin-6
RS005301	GO:0006979	response to oxidative stress	BP	-0.401903067	0.065978471	0.291378926	6.906019939	3.686868507	0.297322289	0.719233616 PREDICTED: apolipoprotein D
RS006464	GO:0006979	response to oxidative stress	BP	-0.213186283	-0.683313237	-0.460996572	7.168303692	3.608960601	0.306902822	0.732065907 PREDICTED: peroxiredoxin-like
RS007161	GO:0006979	response to oxidative stress	BP	0.159770842	0.500563073	0.54212058	5.079589487	3.582069882	0.310272885	0.735732735 PREDICTED: methionine-R-sulfoxide reductase B1
RS010551	GO:0006979	response to oxidative stress	BP	-0.489875704	-0.385811384	-0.050184616	6.411759735	3.352201822	0.340442958	0.774042245 PREDICTED: nicotinate phosphoribosyltransferase
RS001412	GO:0006979	response to oxidative stress	BP	0.007466485	0.016448123	-0.624237018	10.73951032	3.088891446	0.378121887	0.824573365 catalase
RS000915	GO:0006979	response to oxidative stress	BP	0.160494101	-0.352038111	-0.30984918	6.450375555	3.009447118	0.390170909	0.837152396 PREDICTED: ubiquitin-conjugating enzyme E2 N
RS007832	GO:0006979	response to oxidative stress	BP	0.018904578	-0.272094856	-0.67756934	6.103828283	2.327520822	0.50726916	0.942212253 PREDICTED: copper transport protein ATOX1
RS004313	GO:0006979	response to oxidative stress	BP	-0.203387624	-0.515381364	-0.514283324	3.765517594	2.273658661	0.517585982	0.949779252 PREDICTED: succinate dehydrogenase assembly factor 3, mitochondrial
RS000181	GO:0006979	response to oxidative stress	BP	0.009146447	-0.001731797	0.43034961	4.82008936	2.164519946	0.538970602	0.960931086 PREDICTED: probable ATP-dependent RNA helicase DHX34
RS011742	GO:0006979	response to oxidative stress	BP	-0.375470265	-0.28252387	-0.080356064	6.155061975	2.139998825	0.543863207	0.963479091 PREDICTED: NADH dehydrogenase [ubiquinone] iron-sulfur protein 8, mitochondrial-like
RS002399	GO:0006979	response to oxidative stress	BP	-1.263069443	-0.004389656	-0.373016841	0.178370386	1.845362455	0.605113656	0.990294198 beat-VI
RS001904	GO:0006979	response to oxidative stress	BP	-0.465716194	-0.256832961	-0.25365829	9.109283159	1.277489504	0.73448523	0.99990777 PREDICTED: ATP synthase subunit d, mitochondrial
RS001941	GO:0006979	response to oxidative stress	BP	0.475615952	0.242555597	0.179826629	4.81610899	1.662554235	0.645290678	0.99990777 PREDICTED: methionine-R-sulfoxide reductase B1
RS002771	GO:0006979	response to oxidative stress	BP	0.11429921	0.057260947	-0.260086354	4.621633405	1.601399058	0.659072634	0.99990777 PREDICTED: transcription factor A, mitochondrial
RS007072	GO:0006979	response to oxidative stress	BP	-0.160606204	-0.006774872	-0.067528379	6.130790005	0.217884661	0.974651735	0.99990777 PREDICTED: U4/U6 small nuclear ribonucleoprotein Prp3
RS009427	GO:0006979	response to oxidative stress	BP	0.517285933	0.503047992	-0.250621855	0.828342903	1.378393533	0.710607131	0.99990777 PREDICTED: peroxidase
RS011379	GO:0006979	response to oxidative stress	BP	-0.241741309	0.199481332	0.0435458	6.927940186	0.907609555	0.823591071	0.99990777 PREDICTED: chloride intracellular channel exc-4
RS013366	GO:0006979	response to oxidative stress	BP	-0.040780434	0.02657982	-0.325158759	6.325234921	1.512995267	0.679274294	0.99990777 PREDICTED: peptide methionine sulfoxide reductase
RS001413	GO:0006979	response to oxidative stress	BP	-0.235474314	-0.249539517	-0.060596629	7.120899059	0.685689763	0.876564588	0.99990777 PREDICTED: cytochrome b-c1 complex subunit 7-like
RS009585	GO:0006979	response to oxidative stress	BP	-0.060253257	-0.282174073	-0.27361297	7.480437595	1.149655769	0.765102488	0.99990777 PREDICTED: NADH dehydrogenase [ubiquinone] 1 beta subcomplex subunit 5, mitochondrial
RS002425	GO:0006979	response to oxidative stress	BP	-0.427136756	-0.441192835	-0.23678134	2.361125962	1.185263902	0.756540763	0.99990777 PREDICTED: DNA repair protein RAD52 homolog
RS005007	GO:0006979	response to oxidative stress	BP	-0.126969964	-0.36580846	-0.224030125	7.522517394	0.44949536	0.929838391	0.99990777 PREDICTED: thioredoxin-2
RS010700	GO:0006979	response to oxidative stress	BP	-0.109829087	-0.11088491	-0.268923902	5.5809346	0.743390315	0.862953705	0.99990777 PREDICTED: thioredoxin-like protein 1
RS012029	GO:0006979	response to oxidative stress	BP	0.211733487	-0.057949473	-0.074157086	3.616879517	0.659578906	0.882667641	0.99990777 PREDICTED: thioredoxin, mitochondrial
RS000130	GO:0006979	response to oxidative stress	BP	0.142168336	0.008524439	0.042519685	5.217251692	0.230642699	0.972497194	0.99990777 PREDICTED: ubiquitin-like modifier-activating enzyme ATG7
RS011766	GO:0006979	response to oxidative stress	BP	0.511550546	0.165329659	-0.190457257	2.37336811	1.464205365	0.690556007	0.99990777 PREDICTED: protein Wnt-16
RS001190	GO:0006979	response to oxidative stress	BP	-0.158211249	-0.190763361	-0.510494858	10.03945401	1.352757705	0.716644967	0.99990777 PREDICTED: 40S ribosomal protein S3
RS008466	GO:0006979	response to oxidative stress	BP	0.036773914	0.04518977	-0.200013924	6.041327146	0.724363171	0.867459071	0.99990777 PREDICTED: protein deglycase DJ-1-like
RS003228	GO:0015711	organic anion transport	BP	-0.561103954	2.12048168	1.83683218	5.406987214	114.6440514	1.09829E-24	3.71824E-23 PREDICTED: b(0,+)-type amino acid transporter 1-like
RS013974	GO:0015711	organic anion transport	BP	1.308057058	2.315023189	0.876327879	6.127768048	111.7248777	4.66769E-24	1.53887E-22 PREDICTED: monocarboxylate transporter 10
RS012045	GO:0015711	organic anion transport	BP	-2.417547613	-3.846942398	-2.948472949	3.138267013	95.12499238	1.73561E-20	4.58246E-19 PREDICTED: sodium-independent sulfate anion transporter
RS014732	GO:0015711	organic anion transport	BP	-2.171879817	-1.24728548	0.09932889	5.367265273	82.18961662	1.04062E-17	2.21004E-16 PREDICTED: solute carrier organic anion transporter family member 2A1
RS006315	GO:0015711	organic anion transport	BP	-2.445857294	-4.975199992	0.147442902	3.069628072	71.7469443	1.80352E-15	3.24017E-14 PREDICTED: bumetanide-sensitive sodium-(potassium)-chloride cotransporter
RS010878	GO:0015711	organic anion transport	BP	-0.202147251	0.219397642	1.259334773	5.8954575	54.42606082	9.10217E-12	1.23527E-10 PREDICTED: band 3 anion transport protein
RS008617	GO:0015711	organic anion transport	BP	-1.758654905	-1.933395291	0.119722157	4.240860888	52.46640405	2.38231E-11	3.09308E-10 PREDICTED: proton-coupled amino acid transporter 1
RS006021	GO:0015711	organic anion transport	BP	-1.978389488	-0.987147201	0.23457041	5.767022167	52.1310105	2.80858E-11	3.63527E-10 PREDICTED: cationic amino acid transporter 3
RS009380	GO:0015711	organic anion transport	BP	1.181487238	0.230316163	-1.011984835	4.090504935	45.23692145	8.23976E-10	9.50289E-09 PREDICTED: b(0,+)-type amino acid transporter 1-like
RS007743	GO:0015711	organic anion transport	BP	-1.804323011	-1.669176003	-0.470211701	5.80929799	43.79813802	1.66579E-09	1.85163E-08 PREDICTED: probable phospholipid-transporting ATPase IF
RS001007	GO:0015711	organic anion transport	BP	-0.057453873	2.550863666	4.593751253	0.34449975	41.36132174	5.4813E-09	5.84023E-08 PREDICTED: sodium-dependent nutrient amino acid transporter 1-like
RS005851	GO:0015711	organic anion transport	BP	0.526397294	1.41413322	1.673681755	7.829535198	40.74316203	7.413E-09	7.77347E-08 PREDICTED: proton-coupled amino acid transporter 4
RS006814	GO:0015711	organic anion transport	BP	-0.514796382	1.014347124	0.975336891	4.041760153	30.97725487	8.59474E-07	7.23543E-06 PREDICTED: large neutral amino acids transporter small subunit 2
RS006320	GO:0015711	organic anion transport	BP	1.668458777	1.549661536	0.932473469	6.183206129	30.51118393	1.07731E-06	8.96741E-06 PREDICTED: bumetanide-sensitive sodium-(potassium)-chloride cotransporter
RS007713	GO:0015711	organic anion transport	BP	-1.593472957	-0.392083561	-0.180235411	5.938924251	30.40817878	1.13245E-06	9.40771E-06 PREDICTED: ATP-binding cassette sub-family D member 2
RS000193	GO:0015711	organic anion transport	BP	-0.612906575	0.552707825	0.864925878	6.395375097	30.19397061	1.25629E-06	1.03772E-05 PREDICTED: b(0,+)-type amino acid transporter 1
RS007526	GO:0015711	organic anion transport	BP	-1.223662153	-0.387211114	-0.507976241	5.27455292	24.05874887	2.42843E-05	0.00016505 PREDICTED: phospholipid-transporting ATPase ID
RS012462	GO:0015711	organic anion transport	BP	-1.887439376	-1.068323927	-1.822700455	2.935725927	21.16526065	9.72757E-05	0.000593839 PREDICTED: synaptic vesicle glycoprotein 2B
RS004393	GO:0015711	organic anion transport	BP	-1.227790861	-2.264108903	-0.817189459	2.289519889	20.23673107	0.000151606	0.000890546 PREDICTED: putative tricarboxylate transport protein, mitochondrial
RS000837	GO:0015711	organic anion transport	BP	-1.260815808	-0.582381283	0.014148923	5.012164404	17.85146996	0.000471988	0.002476758 PREDICTED: excitatory amino acid transporter 1-like
RS011194	GO:0015711	organic anion transport	BP	-0.043178438	0.101493976	0.864859407	6.406781775	17.21343829	0.00063878	0.003251735 PREDICTED: mitochondrial pyruvate carrier 2-like
RS000849	GO:0015711	organic anion transport	BP	-0.753175709	-1.571391853	-1.058453667	2.708513766	17.06922787	0.000683946	0.003464852 PREDICTED: monocarboxylate transporter 9-like
RS000927	GO:0015711	organic anion transport	BP	-2.570527298	-2.804707147	-0.755163456	0.667619183	16.35029216	0.000961058	0.004715063 PREDICTED: b(0,+)-type amino acid transporter 1-like
RS013870	GO:0015711	organic anion transport	BP	-1.185159015	-0.789079424	-0.942978819	4.024142004	15.86729932	0.001207277	0.005765813 PREDICTED: sodium- and chloride-dependent glycine transporter 1-like
RS000928	GO:0015711	organic anion transport	BP	-2.818387407	-2.387215456	-1.195316578	0.324906115	15.29049888	0.001584497	0.007309578 B(0,+)-type amino acid transporter 1
RS005708	GO:0015711	organic anion transport	BP	-2.57707626	-0.767810406	-0.121337824	1.208165742	14.61680361	0.002175202	0.009598631 PREDICTED: sodium- and chloride-dependent glycine transporter 1-like
RS008723	GO:0015711	organic anion transport	BP	0.765724899	0.492155106	-0.726953396	2.604372289	14.25219643	0.002581214	0.011140444 PREDICTED: excitatory amino acid transporter
RS005241	GO:0015711	organic anion transport	BP	-1.24648029	-0.946331544	-0.692073388	4.41212653	12.5745733	0.005653054	0.021933014 PREDICTED: high-affinity choline transporter 1
RS002985	GO:0015711	organic anion transport	BP	-0.571114377	-0.476136182	-0.859409917	5.548946497	12.07709757	0.007123669	0.026732864 PREDICTED: Y+L amino acid transporter 2
RS010641	GO:0015711	organic anion transport	BP	-0.807148767	-0.226247423	-0.169065413	4.942005241	11.48155079	0.009387573	0.033673341 PREDICTED: probable phospholipid-transporting ATPase VD
RS003950	GO:0015711	organic anion transport	BP	-0.587671115	-0.868990799	-0.714405964	4.844171479	10.0941278	0.017782729	0.057765203 PREDICTED: cationic amino acid transporter 2
RS006809	GO:0015711	organic anion transport	BP	-0.069599294	0.161390813	-0.669189997	5.521140821	9.427681054	0.02411329	0.074118459 PREDICTED: sodium bicarbonate cotransporter 3
RS009139	GO:0015711	organic anion transport	BP	0.649351007	1.292845053	0.336142374	2.039377777	9.031613663	0.028873505	0.085540561 PREDICTED: Y+L amino acid transporter 2
RS007859	GO:0015711	organic anion transport	BP	-1.037355681	-0.953164057	-1.137934678	2.631254112	8.895408464	0.0307143	0.08987358 PREDICTED: proton-coupled amino acid transporter 4
RS006017	GO:0015711	organic anion transport	BP	-0.226333875	0.829010789	0.173010283	3.457042927	8.42538268	0.037991671	0.106302401 PREDICTED: cationic amino acid transporter 2
RS002298	GO:0015711	organic anion transport	BP	0.444685109	0.888871791	0.46931162	5.877833769	8.218233291	0.041710358	0.114594295 PREDICTED: battenin
RS012784	GO:0015711	organic anion transport	BP	-0.964188065	-1.053670411	-1.487092291	6.693074429	8.119987423	0.043595935	0.118762068 PREDICTED: excitatory amino acid transporter-like
RS003841	GO:0015711	organic anion transport	BP	-0.018830809	0.667905762	-0.171179406	4.919354176	7.849821458	0.04921958	0.130930145 PREDICTED: major facilitator superfamily domain-containing protein 10
RS006810	GO:0015711	organic anion transport	BP	-0.692918241	-0.709642301	-0.469276201	5.552917252	7.276110601	0.063598968	0.159523084 PREDICTED: Y+L amino acid transporter 2
RS015105	GO:0015711	organic anion transport	BP	-0.678666411	-2.224992259	-0.562865466	0.559101899	7.185153044	0.066224707	0.164438873 PREDICTED: organic cation transporter 1
RS009471	GO:0015711	organic anion transport	BP	0.454440582	0.068570398	0.92902968	1.975531925	6.680170813	0.082821497	0.194672252 PREDICTED: potassium voltage-gated channel protein Shal
RS012532	GO:0015711	organic anion transport	BP	0.52422059	0.27169055	0.170626759	5.950381512	5.527522768	0.137001687	0.282351902 PREDICTED: adenosine 3'-phospho 5'-phosphosulfate transporter 2
RS002769	GO:0015711	organic anion transport	BP	-0.180596666	-0.510025269	-0.494314256	5.266272897	5.147923967	0.161280396	0.318264698 PREDICTED: mitochondrial 2-oxoglutarate/malate carrier protein
RS006076	GO:0015711	organic anion transport	BP	-0.399500112	-0.234112198	-0.791572834	3.218719557	5.135126595	0.162165734	0.319411206 PREDICTED: cationic amino acid transporter 4
RS002889	GO:0015711	organic anion transport	BP	-0.128703142	0.3529612	0.286577104	4.360050122	4.605415706	0.203077987	0.373149135 PREDICTED: bifunctional arginine demethylase and lysyl-hydroxylase PSR
RS000049	GO:0015711	organic anion transport	BP	-0.167016261	-0.078808593	0.315739518	4.19490186	3.918242209	0.270428905	0.452114912 PREDICTED: protein I'm not dead yet-like
RS013707	GO:0015711	organic anion transport	BP	-0.500388832	-0.162680031	-0.110632879	6.260634623	3.457673335	0.326293381	0.508267019 PREDICTED: phospholipid scramblase 2
RS002254	GO:0015711	organic anion transport	BP	0.026779126	-0.240562604	-0.407019639	8.0023415	2.926808228	0.403048115	0.579800873 PREDICTED: non-specific lipid-transfer protein
RS007790	GO:0015711	organic anion transport	BP	0.13034852	0.225712601	0.386180211	4.625198528	2.606288996	0.456388071	0.628856824 PREDICTED: mitochondrial glutamate carrier 1-like
RS004471	GO:0015711	organic anion transport	BP	0.302505199	0.1016203	-0.272900349	3.077100879	2.396738775	0.494241988	0.660492741 PREDICTED: Y+L amino acid transporter 2
RS002215	GO:0015711	organic anion transport	BP	-0.459893543	-0.373663745	-0.327616751	3.245153068	2.258109459	0.520593488	0.682313913 PREDICTED: CMP-sialic acid transporter 1
RS010646	GO:0015711	organic anion transport	BP	-0.387797302	-0.153467445	-0.201059514	5.65127642	2.191739529	0.533577259	0.692056848 PREDICTED: probable phospholipid-transporting ATPase IA
RS010044	GO:0015711	organic anion transport	BP	-0.032740719	-0.492484393	-0.25544283	2.787037183	2.091316821	0.553671426	0.707481528 PREDICTED: solute carrier family 35 member C2
RS015410	GO:0015711	organic anion transport	BP	-0.258061334	-0.246025988	-0.260658269	5.743258539	1.692882375	0.638517364	0.773443079 PREDICTED: protein slowmo
RS012406	GO:0015711	organic anion transport	BP	-0.118916496	-0.22943197	0.145617021	8.722210769	1.650202623	0.648061031	0.779972717 PREDICTED: probable phospholipid-transporting ATPase IIB
RS002882	GO:0015711	organic anion transport	BP	0.362043543	0.12287268	0.14951259	3.567153224	1.277605364	0.734457649	0.842342193 PREDICTED: putative sodium-coupled neutral amino acid transporter 7
RS006075	GO:0015711	organic anion transport	BP	-0.226394382	-0.065362369	0.088572196	3.008430896	0.898142308	0.82587609	0.901150881 PREDICTED: acetyl-coenzyme A transporter 1
RS013172	GO:0015711	organic anion transport	BP	0.064707769	0.050177661	0.256276698	4.54520608	0.870549691	0.832528393	0.905630734 PREDICTED: N-acetyl-D-glucosamine kinase
RS006019	GO:0015711	organic anion transport	BP	0.016800687	-0.569398535	-0.206623396	0.539126613	0.775635116	0.855285471	0.920559373 PREDICTED: cationic amino acid transporter 2
RS014940	GO:0015711	organic anion transport	BP	0.00473671	-0.350880149	-0.354295712	0.736422598	0.582482205	0.900430699	0.946555742 PREDICTED: potassium voltage-gated channel protein Shal
RS004110	GO:0015711	organic anion transport	BP	-0.109326082	-0.245764423	-0.393853677	2.856144774	0.536964028	0.910698682	0.95276119 PREDICTED: phospholipase A2 inhibitor and Ly6/PLAUR domain-containing protein
RS002585	GO:0015711	organic anion transport	BP	0.061337763	0.015683494	0.119579633	4.655608643	0.205533408	0.976691112	0.987602807 PREDICTED: mitochondrial pyruvate carrier 1
RS008706	GO:0015711	organic anion transport	BP	0.063443874	0.058144534	-0.01579188	5.633533699	0.196321115	0.978180999	0.988383465 PREDICTED: ATP-binding cassette sub-family D member 3
RS005614	GO:0015711	organic anion transport	BP	-0.026120006	0.069729297	0.030053445	4.472662817	0.146084989	0.985784124	0.992693667 PREDICTED: mitochondrial folate transporter/carrier
RS011718	GO:0050662	coenzyme binding	MF	-1.677134346	7.355612033	1.42823741	4.226265198	269.3606101	4.24421E-58	5.81323E-56 PREDICTED: glucose dehydrogenase [FAD, quinone]
RS011720	GO:0050662	coenzyme binding	MF	-0.878405082	6.450933872	3.89893986	5.100146776	253.7209487	1.02569E-54	1.22443E-52 PREDICTED: glucose dehydrogenase [FAD, quinone]
RS011712	GO:0050662	coenzyme binding	MF	-0.691837377	5.47244167	0.194934181	4.073579827	240.7750285	6.47001E-52	6.84454E-50 PREDICTED: glucose dehydrogenase [FAD, quinone]
RS008170	GO:0050662	coenzyme binding	MF	0.464417083	3.756902162	1.942742291	5.537004482	172.6178007	3.46336E-37	2.10585E-35 PREDICTED: glucose dehydrogenase [FAD, quinone]
RS011719	GO:0050662	coenzyme binding	MF	-0.201289446	3.815855918	0.979940861	5.376871056	132.0706959	1.93557E-28	8.25757E-27 PREDICTED: glucose dehydrogenase [FAD, quinone]
RS011717	GO:0050662	coenzyme binding	MF	-1.782578311	2.295553581	1.745379872	4.155616337	94.85427561	1.98442E-20	5.82872E-19 PREDICTED: glucose dehydrogenase [FAD, quinone]-like
RS006397	GO:0050662	coenzyme binding	MF	3.837844051	4.841195518	3.140033232	5.091996475	80.54891857	2.3404E-17	5.879E-16 PREDICTED: glucose dehydrogenase [FAD, quinone]
RS003016	GO:0050662	coenzyme binding	MF	1.211947769	-1.549948573	-1.990414638	7.001580375	74.51831663	4.59557E-16	1.07938E-14 PREDICTED: glucose dehydrogenase [FAD, quinone]
RS003580	GO:0050662	coenzyme binding	MF	-0.349887763	1.980932562	-0.316713103	4.990271951	64.48253049	6.47206E-14	1.32413E-12 PREDICTED: uncharacterized aarF domain-containing protein kinase 1
RS013606	GO:0050662	coenzyme binding	MF	4.363630256	4.336884662	2.51959187	5.502134837	58.93121738	9.94379E-13	1.87792E-11 PREDICTED: glucose dehydrogenase [FAD, quinone]-like
RS011394	GO:0050662	coenzyme binding	MF	-1.310535646	-3.096294509	-0.517909132	5.808602068	53.32498951	1.563E-11	2.65505E-10 PREDICTED: hydroxyacyl-coenzyme A dehydrogenase, mitochondrial-like
RS003648	GO:0050662	coenzyme binding	MF	2.2339965	2.712820849	0.055008188	6.310513776	53.06980816	1.7716E-11	2.99377E-10 PREDICTED: protein singed
RS011716	GO:0050662	coenzyme binding	MF	0.661835892	2.374573374	5.099888992	3.499987369	50.73259443	5.57778E-11	9.10641E-10 PREDICTED: glucose dehydrogenase [FAD, quinone]-like
RS011734	GO:0050662	coenzyme binding	MF	-2.381150947	1.53441958	-0.155193175	3.200718541	50.09797192	7.61442E-11	1.23386E-09 PREDICTED: glucose dehydrogenase [FAD, quinone]-like
RS013157	GO:0050662	coenzyme binding	MF	3.109085667	3.794642952	1.821329985	2.612997621	45.59706242	6.90815E-10	1.02613E-08 Rm62
RS013033	GO:0050662	coenzyme binding	MF	-2.067865792	-3.155771773	-1.509585787	4.376849268	39.67142748	1.25083E-08	1.6659E-07 PREDICTED: glucose dehydrogenase [FAD, quinone]

RS014962	GO:0050662	coenzyme binding	MF	-0.723414571	-3.283728393	-0.977439939	8.602717144	35.08484269	1.16899E-07	1.37655E-06 PREDICTED: delta(24)-sterol reductase
RS007667	GO:0050662	coenzyme binding	MF	-2.271726545	-2.772423044	-1.508064761	3.931848614	34.15601591	1.83649E-07	2.11099E-06 PREDICTED: xanthine dehydrogenase/oxidase-like
RS003551	GO:0050662	coenzyme binding	MF	-2.320704019	-2.211406868	-0.33940574	3.30376148	32.1872846	4.77877E-07	5.24737E-06 PREDICTED: xanthine dehydrogenase/oxidase-like
RS003553	GO:0050662	coenzyme binding	MF	-1.792475202	-2.788631843	-0.988191431	7.24571982	32.11857982	4.9408E-07	5.41615E-06 PREDICTED: xanthine dehydrogenase/oxidase-like
RS013610	GO:0050662	coenzyme binding	MF	-1.414646775	0.986031468	0.512338668	4.087496549	29.21105762	2.02201E-06	2.04909E-05 PREDICTED: glucose dehydrogenase [FAD, quinone]-like
RS007610	GO:0050662	coenzyme binding	MF	-0.860794812	-1.494346941	0.030222932	6.595353721	29.04342528	2.19287E-06	2.20848E-05 PREDICTED: short-chain specific acyl-CoA dehydrogenase, mitochondrial
RS001401	GO:0050662	coenzyme binding	MF	-0.977743475	-1.429648234	0.392313729	5.961713183	27.81212111	3.97723E-06	3.85057E-05 PREDICTED: isocitrate dehydrogenase [NADP] cytoplasmic-like
RS013848	GO:0050662	coenzyme binding	MF	-0.484403671	-1.643873838	-1.299488794	6.666651214	27.10251482	5.60309E-06	5.32559E-05 PREDICTED: protein CREG1
RS010228	GO:0050662	coenzyme binding	MF	-0.4477261	-3.856305519	-0.79793105	4.076468118	25.71180482	1.09587E-05	0.000100419 PREDICTED: xanthine dehydrogenase/oxidase-like
RS003552	GO:0050662	coenzyme binding	MF	-2.499522175	-1.892065472	-0.945108894	4.060088254	25.29890711	1.33706E-05	0.000120151 PREDICTED: xanthine dehydrogenase/oxidase-like
RS000919	GO:0050662	coenzyme binding	MF	0.265455328	1.209618201	-0.138864047	7.571941316	23.36746642	3.38518E-05	0.000286026 PREDICTED: 3-hydroxy-3-methylglutaryl-coenzyme A reductase
RS013098	GO:0050662	coenzyme binding	MF	-2.627761832	-2.171547526	-1.375349886	5.010664496	20.25921267	0.000149988	0.001148663 PREDICTED: xanthine dehydrogenase/oxidase-like
RS010386	GO:0050662	coenzyme binding	MF	1.273208137	1.042150088	0.31368786	6.102481464	20.20213961	0.000154131	0.001176967 PREDICTED: ketimine reductase mu-crystallin
RS010272	GO:0050662	coenzyme binding	MF	-2.310395071	-3.420644367	-0.829924791	7.520837969	20.07413896	0.000163842	0.001245274 PREDICTED: glucose dehydrogenase [FAD, quinone]-like
RS007102	GO:0050662	coenzyme binding	MF	-1.158824091	-1.04699828	-0.00307914	6.070446705	19.83328236	0.000183795	0.001381598 PREDICTED: glycerol-3-phosphate dehydrogenase [NAD(+)], cytoplasmic
RS006415	GO:0050662	coenzyme binding	MF	-1.651997012	-2.962727725	-0.457633844	2.720032924	18.60239614	0.000330345	0.002392012 PREDICTED: glucose dehydrogenase [FAD, quinone]-like
RS007147	GO:0050662	coenzyme binding	MF	-0.546304096	-1.231390285	-0.07000755	6.347679657	17.95585171	0.000449168	0.003179856 PREDICTED: hydroxyacid oxidase 1
RS010161	GO:0050662	coenzyme binding	MF	-1.408904056	-1.039903857	0.304797042	7.254219051	17.56197202	0.00054149	0.003773896 PREDICTED: glucose dehydrogenase [FAD, quinone]
RS009449	GO:0050662	coenzyme binding	MF	-0.210212423	0.045351531	-1.352961977	6.598685537	17.4890606	0.000560542	0.003892087 PREDICTED: very-long-chain 3-oxoacyl-CoA reductase
RS007239	GO:0050662	coenzyme binding	MF	-1.690194917	-1.006335231	-2.601370143	9.362292558	17.21752904	0.000637543	0.004384623 PREDICTED: D-3-phosphoglycerate dehydrogenase
RS011713	GO:0050662	coenzyme binding	MF	-1.399610201	-1.047967269	-4.489105762	0.579082779	15.66566934	0.001327738	0.008633943 PREDICTED: glucose dehydrogenase [FAD, quinone]
RS006985	GO:0050662	coenzyme binding	MF	-0.889685267	0.399745441	-0.087279987	5.320884465	15.6629973	0.001329411	0.00864051 PREDICTED: probable 2-oxoglutarate dehydrogenase E1 component DHKTD1 homolog, mitochondrial
RS015505	GO:0050662	coenzyme binding	MF	-0.540587544	-1.35503344	-0.323706554	7.227672234	14.8094815	0.001986915	0.012393929 PREDICTED: probable methylmalonate-semialdehyde dehydrogenase [acylating], mitochondrial
RS008782	GO:0050662	coenzyme binding	MF	-0.290790891	-0.956056388	-1.111908007	7.99510642	14.34941892	0.002466131	0.015002009 PREDICTED: putative acyl-CoA-binding protein
RS010160	GO:0050662	coenzyme binding	MF	-1.886521476	-0.946496063	0.327392983	6.148416556	14.06047071	0.002823987	0.016956952 PREDICTED: glucose dehydrogenase [FAD, quinone]
RS006300	GO:0050662	coenzyme binding	MF	-0.553963926	-1.182294641	-0.863267173	5.022348343	11.6104729	0.00884391	0.046798272 PREDICTED: pyridoxine-5'-phosphate oxidase
RS003251	GO:0050662	coenzyme binding	MF	-0.160022359	-0.107480119	1.077028476	5.90623249	11.49415125	0.009333015	0.049018733 PREDICTED: alkyldihydroxyacetonephosphate synthase
RS012259	GO:0050662	coenzyme binding	MF	-1.037569088	-0.237504992	0.194085759	8.791699835	10.59122231	0.014154682	0.070648532 PREDICTED: putative glutamate synthase [NADPH]
RS008249	GO:0050662	coenzyme binding	MF	-0.050973023	2.07922754	0.061887416	2.374448226	10.44247032	0.015156276	0.075000623 PREDICTED: glucose dehydrogenase [FAD, quinone]-like
RS011402	GO:0050662	coenzyme binding	MF	-0.709898376	-0.945582049	-0.09296649	4.879493434	9.483329034	0.023509374	0.109958296 PREDICTED: glyoxylate reductase/hydroxypyruvate reductase-like
RS011967	GO:0050662	coenzyme binding	MF	-1.529709032	-0.324687056	-0.637465432	6.575025044	9.155271706	0.027296135	0.124930462 PREDICTED: flavin-containing monooxygenase FMO GS-OX-like 4
RS002576	GO:0050662	coenzyme binding	MF	-0.812888282	-1.248557048	-0.322378291	7.349885041	9.079229379	0.028255826	0.128734177 PREDICTED: NADP-dependent malic enzyme
RS010080	GO:0050662	coenzyme binding	MF	-0.90809991	-0.983654565	-0.3068705	8.748492406	9.044142511	0.028709709	0.130429943 PREDICTED: 2-oxoglutarate dehydrogenase, mitochondrial
RS000419	GO:0050662	coenzyme binding	MF	-0.173058161	-0.438003632	-0.895708169	4.822231756	8.846365	0.031404848	0.140764688 PREDICTED: peroxisomal acyl-coenzyme A oxidase 3
RS010472	GO:0050662	coenzyme binding	MF	-1.026707823	-1.197899702	-0.915014401	7.802419524	7.929782959	0.047485391	0.197676545 PREDICTED: isovaleryl-CoA dehydrogenase, mitochondrial
RS002770	GO:0050662	coenzyme binding	MF	0.328166032	-0.205639527	-0.764873783	9.960070132	7.863327496	0.048922398	0.20228098 glyceraldehyde-3-phosphate dehydrogenase
RS003700	GO:0050662	coenzyme binding	MF	0.027952133	0.095675547	1.160946536	6.244525396	7.713409905	0.052321287	0.213340319 PREDICTED: UDP-glucose 6-dehydrogenase
RS014702	GO:0050662	coenzyme binding	MF	0.370816027	-0.107832607	-0.531915299	6.989121648	7.662533178	0.053526106	0.217492468 PREDICTED: glutaryl-CoA dehydrogenase, mitochondrial
RS005467	GO:0050662	coenzyme binding	MF	1.202807461	0.825481754	0.381694479	2.532198433	7.308681743	0.062683469	0.24686359 PREDICTED: lysine-specific histone demethylase 1A
RS007101	GO:0050662	coenzyme binding	MF	0.672064541	-0.079902458	0.630684144	3.567156404	6.95376732	0.073386056	0.278640022 FAD-linked oxidoreductase
RS007857	GO:0050662	coenzyme binding	MF	0.286523988	0.752318655	0.863885212	8.395901468	6.859924259	0.076499041	0.287772628 PREDICTED: xanthine dehydrogenase
RS014330	GO:0050662	coenzyme binding	MF	-0.547619455	-0.984090693	0.102137177	7.940897528	6.327677389	0.09671192	0.345498825 PREDICTED: NADPH--cytochrome P450 reductase
RS011383	GO:0050662	coenzyme binding	MF	-0.562053873	-0.830815635	-0.239507137	5.935011736	6.21672363	0.101529263	0.357982134 PREDICTED: 2-hydroxyacyl-CoA lyase 1
RS011032	GO:0050662	coenzyme binding	MF	-0.268958926	-0.726796129	-0.525522169	5.764106106	6.197243518	0.10239845	0.360305202 PREDICTED: D-aspartate oxidase
RS015148	GO:0050662	coenzyme binding	MF	0.362114549	1.211992883	-0.098398662	2.069285428	5.979173869	0.11262787	0.386373277 PREDICTED: L-threonine 3-dehydrogenase, mitochondrial
RS000370	GO:0050662	coenzyme binding	MF	0.163380228	0.672081111	-0.68984363	5.788067827	5.907309219	0.116207955	0.396076987 PREDICTED: nitric oxide synthase, salivary gland
RS003219	GO:0050662	coenzyme binding	MF	-0.60805082	-0.758994211	-0.077735532	6.577607798	5.904551523	0.116347478	0.396416177 PREDICTED: probable peroxisomal acyl-coenzyme A oxidase 1
RS002509	GO:0050662	coenzyme binding	MF	0.400384812	-0.298727669	-0.16771026	6.495095546	5.693841077	0.12749366	0.423416924 PREDICTED: poly [ADP-ribose] polymerase
RS007471	GO:0050662	coenzyme binding	MF	0.177649745	-0.017075131	-0.63575638	8.685126312	5.53198665	0.13673793	0.442595508 PREDICTED: lambda-crystallin homolog
RS011393	GO:0050662	coenzyme binding	MF	-0.092851264	-0.771839308	0.112119668	7.379663604	5.51803093	0.137564137	0.444275142 PREDICTED: hydroxyacyl-coenzyme A dehydrogenase, mitochondrial-like
RS000486	GO:0050662	coenzyme binding	MF	0.149264968	-0.476649943	-0.298886689	6.825954701	5.383686168	0.145763371	0.462490365 PREDICTED: apoptosis-inducing factor 1, mitochondrial
RS001628	GO:0050662	coenzyme binding	MF	-0.862731725	-0.758883814	-0.128775174	5.394799439	5.374118119	0.146364637	0.463043195 PREDICTED: apoptosis-inducing factor 3
RS015504	GO:0050662	coenzyme binding	MF	-0.22383399	-0.869880626	0.078160009	6.737485668	4.602155027	0.20335729	0.577362389 NAD(P) transhydrogenase, mitochondrial
RS001108	GO:0050662	coenzyme binding	MF	-0.111828215	-0.628399916	0.120485142	5.025645681	4.448850234	0.216898359	0.598702047 PREDICTED: short-chain specific acyl-CoA dehydrogenase, mitochondrial
RS012274	GO:0050662	coenzyme binding	MF	-0.157151321	-0.445345448	-0.585280604	6.166687992	4.392931818	0.222041667	0.607742149 PREDICTED: probable GDP-L-fucose synthase
RS003220	GO:0050662	coenzyme binding	MF	-0.290428325	-0.962495354	-0.261658733	6.502916295	4.20729073	0.239932958	0.636640928 PREDICTED: probable peroxisomal acyl-coenzyme A oxidase 1
RS006951	GO:0050662	coenzyme binding	MF	0.391598781	0.164990919	-0.20676917	6.46543475	4.182029974	0.242467195	0.641126426 PREDICTED: lysine-specific histone demethylase 1A
RS000629	GO:0050662	coenzyme binding	MF	-0.593328197	-0.467055593	-0.154522039	6.966413176	4.138091025	0.246933701	0.649242537 PREDICTED: thioredoxin reductase 3
RS012446	GO:0050662	coenzyme binding	MF	-0.758377616	-0.563761583	-0.392830087	5.458637643	4.132864133	0.247470002	0.650032302 PREDICTED: NAD-dependent protein deacetylase sirtuin-3, mitochondrial
RS000974	GO:0050662	coenzyme binding	MF	-0.060643312	-0.536072666	-0.495255374	7.659231763	4.011761531	0.260196895	0.667787375 PREDICTED: electron transfer flavoprotein subunit alpha, mitochondrial
RS015527	GO:0050662	coenzyme binding	MF	0.227446765	-0.168375725	-0.421383123	5.579762775	3.739885757	0.290956281	0.711773479 PREDICTED: acyl-CoA dehydrogenase family member 9, mitochondrial
RS009254	GO:0050662	coenzyme binding	MF	0.329363236	-0.1679262	-0.236103203	6.931272759	3.72855853	0.292306046	0.712824029 PREDICTED: histidine triad nucleotide-binding protein 1
RS009326	GO:0050662	coenzyme binding	MF	0.271233216	-0.52054431	0.281230551	7.156265409	3.639210319	0.303150743	0.727160014 phosphoenolpyruvate decarboxylase
RS011722	GO:0050662	coenzyme binding	MF	-0.683768484	-0.260694548	-0.543176829	3.956302	3.562420306	0.312756197	0.738715491 PREDICTED: NAD-dependent protein deacetylase Sirt4-like
RS010695	GO:0050662	coenzyme binding	MF	0.080660918	-0.269991958	-0.511528881	4.057423162	3.508763189	0.319627342	0.747930145 PREDICTED: dihydrofolate reductase
RS002439	GO:0050662	coenzyme binding	MF	-0.190892983	-0.30270329	0.333270898	7.04111046	3.452194077	0.327015523	0.757845857 PREDICTED: UDP-glucose 4-epimerase-like
RS007152	GO:0050662	coenzyme binding	MF	-1.661119036	0.054831494	-0.726096348	0.38835773	3.410514582	0.332554873	0.764929116 PREDICTED: neuroligin-4, Y-linked-like
RS007160	GO:0050662	coenzyme binding	MF	-0.266965371	-0.958259852	-0.038280786	7.986560693	3.306724355	0.346707836	0.783086679 cytoplasmic NADP+-dependent isocitrate dehydrogenase
RS012147	GO:0050662	coenzyme binding	MF	-0.003844476	0.534728412	0.648097732	2.95113613	3.100241495	0.376426647	0.822650911 PREDICTED: NAD-dependent protein deacetylase sirtuin-7
RS011714	GO:0050662	coenzyme binding	MF	-0.680616416	-1.21502682	-0.069453686	4.965240929	2.977488993	0.395108969	0.842536529 PREDICTED: glucose dehydrogenase [FAD, quinone]-like
RS007459	GO:0050662	coenzyme binding	MF	-0.418427547	-0.067453359	0.26238096	4.488682328	2.86793239	0.412438777	0.857460404 PREDICTED: NAD-dependent protein deacetylase sirtuin-1
RS002545	GO:0050662	coenzyme binding	MF	-0.300074026	-0.525515743	-0.456727527	8.241268035	2.812688476	0.421415425	0.866401882 PREDICTED: NADH-ubiquinone oxidoreductase 49 kDa subunit
RS002591	GO:0050662	coenzyme binding	MF	-0.281796043	-0.496060318	-0.016455478	7.554385005	2.71789938	0.437194048	0.883333673 PREDICTED: succinate dehydrogenase [ubiquinone] flavoprotein subunit, mitochondrial
RS010959	GO:0050662	coenzyme binding	MF	-0.219964887	-0.474866529	-0.016714397	5.074522056	2.644483397	0.449744347	0.895711868 PREDICTED: probable sulfite oxidase, mitochondrial
RS013491	GO:0050662	coenzyme binding	MF	-0.860941075	-0.802547543	-0.148621609	5.005651027	2.586240397	0.459906891	0.90528127 PREDICTED: amine oxidase [flavin-containing] B
RS010692	GO:0050662	coenzyme binding	MF	0.353391735	0.58755906	0.383472499	6.319056823	2.45754997	0.483011624	0.92452016 PREDICTED: acyl-CoA-binding domain-containing protein 4
RS001900	GO:0050662	coenzyme binding	MF	-0.32899135	-0.469609787	-0.30935471	7.59668921	2.362862316	0.500585227	0.937593606 PREDICTED: ATP-dependent RNA helicase p62-like
RS009084	GO:0050662	coenzyme binding	MF	-0.167814955	-0.235734666	0.247572872	4.131260134	2.284989925	0.515402536	0.948161941 dTDP-glucose 4,6-dehydratase
RS007877	GO:0050662	coenzyme binding	MF	0.231261259	-0.301141191	-0.159101912	6.11514377	2.195042161	0.532925571	0.957947831 PREDICTED: D-aspartate oxidase
RS009408	GO:0050662	coenzyme binding	MF	0.332406588	0.064876657	-0.119432113	7.56140999	2.149137594	0.54203603	0.962731753 PREDICTED: 26S protease regulatory subunit 8
RS006389	GO:0050662	coenzyme binding	MF	0.0839095	0.473419928	0.279876216	6.06692309	2.130381677	0.545790838	0.964745796 PREDICTED: epimerase family protein SDR39U1
RS010824	GO:0050662	coenzyme binding	MF	0.11638411	0.006340801	-0.312086561	4.911327057	2.057601311	0.560537811	0.973863574 PREDICTED: NAD-dependent protein deacetylase Sirt6
RS002905	GO:0050662	coenzyme binding	MF	-0.208807259	-0.485681741	-0.271449041	4.212062169	2.042408049	0.563651573	0.974393273 PREDICTED: elongation factor Tu, mitochondrial-like
RS005517	GO:0050662	coenzyme binding	MF	-0.211559073	0.508167407	0.398875873	9.62409344	2.011791203	0.569963007	0.977881438 PREDICTED: ATP-dependent RNA helicase p62-like
RS011715	GO:0050662	coenzyme binding	MF	0.232398683	-0.32467993	-0.497749239	9.255753823	1.995956647	0.573246342	0.979513468 PREDICTED: glucose dehydrogenase [FAD, quinone]-like
RS005051	GO:0050662	coenzyme binding	MF	0.132564164	-0.184946277	0.268487178	6.10889186	1.925639688	0.58798298	0.982261462 PREDICTED: DNA repair and recombination protein RAD54-like
RS007230	GO:0050662	coenzyme binding	MF	-0.321815599	-0.145177091	0.111851666	7.452678053	1.888909626	0.595780867	0.986039957 PREDICTED: isocitrate dehydrogenase [NAD] subunit beta, mitochondrial
RS000848	GO:0050662	coenzyme binding	MF	-0.155177077	-0.054380213	-0.106039556	6.035599641	0.203432361	0.977033304	0.99990777 PREDICTED: NADH dehydrogenase [ubiquinone] 1 alpha subcomplex subunit 9, mitochondrial
RS011007	GO:0050662	coenzyme binding	MF	0.370491714	0.145766581	0.132592935	4.054493476	0.670485363	0.880123138	0.99990777 PREDICTED: E3 ubiquitin-protein ligase znrf1
RS000296	GO:0050662	coenzyme binding	MF	-0.154498762	-0.37652032	0.030675109	6.715589582	1.23285073	0.745136608	0.99990777 PREDICTED: peroxisomal acyl-coenzyme A oxidase 3
RS002455	GO:0050662	coenzyme binding	MF	-0.201227463	-0.103527722	-0.061657793	8.817941177	0.221257741	0.974086741	0.99990777 PREDICTED: very long-chain specific acyl-CoA dehydrogenase, mitochondrial
RS005907	GO:0050662	coenzyme binding	MF	-0.028571701	-0.250665994	-0.167826705	6.338717792	1.008817914	0.799118294	0.99990777 PREDICTED: Golgi resident protein GCP60
RS010621	GO:0050662	coenzyme binding	MF	-0.235228566	-0.151341716	-0.182369329	7.593265225	0.605581061	0.895153537	0.99990777 PREDICTED: probable medium-chain specific acyl-CoA dehydrogenase, mitochondrial
RS010926	GO:0050662	coenzyme binding	MF	-0.110904215	-0.046201239	-0.056471766	6.641446402	0.114050507	0.990099578	0.99990777 PREDICTED: short/branched chain specific acyl-CoA dehydrogenase, mitochondrial
RS012144	GO:0050662	coenzyme binding	MF	0.004213152	-0.168666595	0.018926112	7.627539159	0.203994393	0.976941905	0.99990777 PREDICTED: acyl-CoA-binding domain-containing protein 6
RS006776	GO:0050662	coenzyme binding	MF	0.082636477	0.19479615	0.026218657	5.988644963	0.230633352	0.97249879	0.99990777 PREDICTED: NADPH-dependent diflavin oxidoreductase 1
RS008221	GO:0050662	coenzyme binding	MF	-0.296242233	-0.292877171	-0.167499981	6.949734867	1.110334936	0.774579793	0.99990777 PREDICTED: S-adenosyl-L-methionine-dependent tRNA 4-demethylwyosine synthase
RS015204	GO:0050662	coenzyme binding	MF	-0.340734028	-0.280871593	-0.166570298	7.528821968	1.296010127	0.730080728	0.99990777 PREDICTED: NADH dehydrogenase [ubiquinone] flavoprotein 1, mitochondrial
RS000379	GO:0050662	coenzyme binding	MF	-0.095818015	-0.187323344	-0.106469915	4.871558625	0.25459218	0.968329263	0.99990777 PREDICTED: protein MTO1 homolog, mitochondrial
RS002730	GO:0050662	coenzyme binding	MF	0.112442444	0.097728046	-0.073103862	5.750991436	0.389850138	0.942331145	0.99990777 PREDICTED: tRNA-dihydrouridine(20a/20b) synthase [NAD(P)+]-like
RS003259	GO:0050662	coenzyme binding	MF	-0.068769809	-0.278245674	0.018362693	5.590298551	0.893915693	0.826895839	0.99990777 PREDICTED: tRNA-dihydrouridine(20) synthase [NAD(P)+]-like
RS003678	GO:0050662	coenzyme binding	MF	0.08116747	0.083723646	0.072102225	3.720696088	0.057284426	0.996415551	0.99990777 PREDICTED: tRNA-dihydrouridine(16/17) synthase [NAD(P)(+)]-like
RS004672	GO:0050662	coenzyme binding	MF	-0.045339079	-0.03791758	-0.379156562	7.248991538	1.700430071	0.636838185	0.99990777 PREDICTED: coatomer subunit epsilon
RS005140	GO:0050662	coenzyme binding	MF	-0.326725695	-0.4292927	-0.1926739	7.010512515	1.74011872	0.628051626	0.99990777 PREDICTED: D-2-hydroxyglutarate dehydrogenase, mitochondrial
RS005465	GO:0050662	coenzyme binding	MF	-0.415280802	-0.3559176	-0.083424036	2.668264203	1.320767587	0.724207614	0.99990777 PREDICTED: lysine-specific histone demethylase 1A
RS005636	GO:0050662	coenzyme binding	MF	-0.126431053	-0.256799359	-0.112814183	7.340931587	0.431193327	0.933725913	0.99990777 PREDICTED: dihydrolipoyl dehydrogenase, mitochondrial
RS006120	GO:0050662	coenzyme binding	MF	-0.114381587	-0.285076445	0.242614973	4.648817041	1.563018554	0.667803875	0.99990777 PREDICTED: tRNA-dihydrouridine(47) synthase [NAD(P)(+)]-like
RS013432	GO:0050662	coenzyme binding	MF	0.039392391	-0.094394556	0.187505343	4.745243893	0.476344021	0.924056928	0.99990777 PREDICTED: glucose dehydrogenase [FAD, quinone]-like
RS014351	GO:0050662	coenzyme binding	MF	-0.103914379	-0.267122842	-0.255874543	7.288624768	0.39826778	0.940600098	0.99990777 PREDICTED: thioredoxin reductase 1, mitochondrial
RS002313	GO:0050662	coenzyme binding	MF	-0.183433173	-0.563018765	-0.068752215	6.442015484	1.562874363	0.667836792	0.99990777 PREDICTED: glucose-6-phosphate 1-dehydrogenase
RS004318	GO:0050662	coenzyme binding	MF	0.10562588	0.605786034	0.169589424	4.752752149	1.31125912	0.726461239	0.99990777 PREDICTED: glyoxylate/hydroxypyruvate reductase A-like

RS004876	GO:0050662	coenzyme binding	MF	0.297503543	0.028799372	-0.129035056	4.711878997	1.739005436	0.628297094	0.99990777	PREDICTED: 3-hydroxyisobutyrate dehydrogenase, mitochondrial
RS006771	GO:0050662	coenzyme binding	MF	0.537812339	0.475649282	0.367948482	5.687855747	1.5523842	0.670233909	0.99990777	PREDICTED: C-terminal-binding protein
RS007359	GO:0050662	coenzyme binding	MF	0.116162047	-0.123530607	-0.24472837	5.799156213	1.648400339	0.648465832	0.99990777	PREDICTED: putative oxidoreductase GLYR1 homolog
RS007419	GO:0050662	coenzyme binding	MF	-0.313414214	-0.11767338	0.040747486	6.996312811	1.295199387	0.730273344	0.99990777	PREDICTED: probable isocitrate dehydrogenase [NAD] subunit alpha, mitochondrial
RS007491	GO:0050662	coenzyme binding	MF	-0.315441425	-0.392926326	-0.025968449	6.310611193	1.590381033	0.661572818	0.99990777	PREDICTED: NADP-dependent malic enzyme
RS009629	GO:0050662	coenzyme binding	MF	-0.092190417	0.072967098	-0.005828954	7.578009556	0.218541343	0.974542006	0.99990777	PREDICTED: isocitrate dehydrogenase [NAD] subunit gamma, mitochondrial
RS005332	GO:0050662	coenzyme binding	MF	0.148105349	0.097256194	0.310691274	5.826726314	0.75930586	0.859173677	0.99990777	PREDICTED: proline dehydrogenase 1, mitochondrial
RS013061	GO:0050662	coenzyme binding	MF	0.394661732	0.077504241	0.334692141	3.375176155	1.525593969	0.676375978	0.99990777	PREDICTED: ubiquinone biosynthesis monooxygenase COQ6
RS004746	GO:0050662	coenzyme binding	MF	0.148840628	0.011239069	0.06904351	6.195621592	0.316944162	0.95681087	0.99990777	PREDICTED: puff-specific protein Bx42
RS008195	GO:0050662	coenzyme binding	MF	-0.053173536	0.131663412	0.234277038	6.329598459	0.454958078	0.928669441	0.99990777	PREDICTED: NAD-dependent protein deacylase sirtuin-5, mitochondrial
RS013047	GO:0050662	coenzyme binding	MF	0.114475553	-0.105384237	-0.076892357	5.837491413	0.670667218	0.880080652	0.99990777	PREDICTED: NAD-dependent protein deacetylase sirtuin-2
RS007652	GO:0006820	anion transport	BP	0.585883623	0.528505008	4.851177054	4.584889963	291.2117195	7.93792E-63	8.92591E-61	PREDICTED: solute carrier family 26 member 10-like
RS004125	GO:0006820	anion transport	BP	1.407227463	2.012417871	-0.423642507	5.906215214	131.7530272	2.26607E-28	8.8083E-27	PREDICTED: ABC transporter G family member 9
RS003228	GO:0006820	anion transport	BP	-0.561103954	2.12048168	1.83683218	5.406987214	114.6440514	1.09829E-24	3.71824E-23	PREDICTED: b(0,+)-type amino acid transporter 1-like
RS013974	GO:0006820	anion transport	BP	1.308057058	2.315023189	0.876327879	6.127768048	111.7248777	4.66769E-24	1.53887E-22	PREDICTED: monocarboxylate transporter 10
RS012045	GO:0006820	anion transport	BP	-2.417547613	-3.846942398	-2.948472949	3.138267013	95.12499238	1.73561E-20	4.58246E-19	PREDICTED: sodium-independent sulfate anion transporter
RS014732	GO:0006820	anion transport	BP	-2.171879817	-1.24728548	0.09932889	5.367265273	82.18961662	1.04062E-17	2.21004E-16	PREDICTED: solute carrier organic anion transporter family member 2A1
RS015390	GO:0006820	anion transport	BP	-1.260588417	-0.88728058	1.174441311	5.643186003	81.54774944	1.42892E-17	3.01438E-16	PREDICTED: sodium-independent sulfate anion transporter
RS006315	GO:0006820	anion transport	BP	-2.445857294	-4.975199992	0.147442902	3.069628072	71.7469443	1.80352E-15	3.24017E-14	PREDICTED: bumetanide-sensitive sodium-(potassium)-chloride cotransporter
RS006994	GO:0006820	anion transport	BP	-0.66398235	-0.594278945	1.697528097	4.919565	60.59841537	4.37914E-13	6.597E-12	PREDICTED: putative inorganic phosphate cotransporter
RS010878	GO:0006820	anion transport	BP	-0.202147251	0.219397642	1.259334773	5.8954575	54.42606082	9.10217E-12	1.23527E-10	PREDICTED: band 3 anion transport protein
RS008617	GO:0006820	anion transport	BP	-1.758654905	-1.933395291	0.119722157	4.240860888	52.46640405	2.38231E-11	3.09308E-10	PREDICTED: proton-coupled amino acid transporter 1
RS006021	GO:0006820	anion transport	BP	-1.978389488	-0.987147201	0.23457041	5.767022167	52.1310105	2.80858E-11	3.63527E-10	PREDICTED: cationic amino acid transporter 3
RS009380	GO:0006820	anion transport	BP	1.181487238	0.230316163	-1.011984835	4.090504935	45.23692145	8.23976E-10	9.50289E-09	PREDICTED: b(0,+)-type amino acid transporter 1-like
RS007743	GO:0006820	anion transport	BP	-1.804323011	-1.669176003	-0.470211701	5.80929799	43.79813802	1.66579E-09	1.85163E-08	PREDICTED: probable phospholipid-transporting ATPase IF
RS005640	GO:0006820	anion transport	BP	0.969003673	1.718434974	-0.876644168	4.401662752	43.66119041	1.78117E-09	1.9764E-08	PREDICTED: glycine receptor subunit alpha-4
RS001007	GO:0006820	anion transport	BP	-0.057453873	2.550863666	4.593751253	0.34449975	41.36132174	5.4813E-09	5.84023E-08	PREDICTED: sodium-dependent nutrient amino acid transporter 1-like
RS005851	GO:0006820	anion transport	BP	0.526397294	1.41413322	1.673681755	7.829535198	40.74316203	7.413E-09	7.77347E-08	PREDICTED: proton-coupled amino acid transporter 4
RS011831	GO:0006820	anion transport	BP	-1.435266558	0.383547956	3.056810998	0.766698698	36.38839562	6.19812E-08	5.87352E-07	PREDICTED: P protein-like
RS006814	GO:0006820	anion transport	BP	-0.514796382	1.014347124	0.975336891	4.041760153	30.97725487	8.59474E-07	7.23543E-06	PREDICTED: large neutral amino acids transporter small subunit 2
RS006320	GO:0006820	anion transport	BP	1.668458777	1.549661536	0.932473469	6.183206129	30.51118393	1.07731E-06	8.96741E-06	PREDICTED: bumetanide-sensitive sodium-(potassium)-chloride cotransporter
RS007713	GO:0006820	anion transport	BP	-1.593472957	-0.392083561	-0.180235411	5.938924251	30.40817878	1.13245E-06	9.40771E-06	PREDICTED: ATP-binding cassette sub-family D member 2
RS000193	GO:0006820	anion transport	BP	-0.612906575	0.552707825	0.864925878	6.395375097	30.19397061	1.25629E-06	1.03772E-05	PREDICTED: b(0,+)-type amino acid transporter 1
RS007526	GO:0006820	anion transport	BP	-1.223662153	-0.387211114	-0.507976241	5.27455292	24.05874887	2.42843E-05	0.00016505	PREDICTED: phospholipid-transporting ATPase ID
RS006319	GO:0006820	anion transport	BP	-1.264071762	-1.378688832	-0.296664377	4.499770473	23.99784591	2.50059E-05	0.000169587	PREDICTED: bumetanide-sensitive sodium-(potassium)-chloride cotransporter
RS011507	GO:0006820	anion transport	BP	0.081672207	0.439418944	1.152509484	5.150989282	22.16598652	6.02438E-05	0.000382801	PREDICTED: sialin
RS012462	GO:0006820	anion transport	BP	-1.887439376	-1.068323927	-1.822700455	2.935725927	21.16526065	9.72757E-05	0.000593839	PREDICTED: synaptic vesicle glycoprotein 2B
RS012046	GO:0006820	anion transport	BP	-0.044612109	1.318321788	-0.182662099	3.873303092	21.0021542	0.000105168	0.000636465	PREDICTED: sodium-independent sulfate anion transporter-like
RS014287	GO:0006820	anion transport	BP	-0.825809928	1.486619083	1.592270521	1.389357496	20.39684624	0.000140447	0.000831501	PREDICTED: sialin
RS004393	GO:0006820	anion transport	BP	-1.227790861	-2.264108903	-0.817189459	2.289519889	20.23673107	0.000151606	0.000890546	PREDICTED: putative tricarboxylate transport protein, mitochondrial
RS000837	GO:0006820	anion transport	BP	-1.260815808	-0.582381283	0.014148923	5.012164404	17.85146996	0.000471988	0.002476758	PREDICTED: excitatory amino acid transporter 1-like
RS011194	GO:0006820	anion transport	BP	-0.043178438	0.101493976	0.864859407	6.406781775	17.21343829	0.00063878	0.003251735	PREDICTED: mitochondrial pyruvate carrier 2-like
RS000849	GO:0006820	anion transport	BP	-0.753175709	-1.571391853	-1.058453667	2.708513766	17.06922787	0.000683946	0.003464852	PREDICTED: monocarboxylate transporter 9-like
RS012047	GO:0006820	anion transport	BP	-1.033691566	-1.204387105	-0.573210977	3.717195265	16.53395031	0.000881135	0.004370625	PREDICTED: sodium-independent sulfate anion transporter-like
RS000927	GO:0006820	anion transport	BP	-2.570527298	-2.804707147	-0.755163456	0.667619183	16.35029216	0.000961058	0.004715063	PREDICTED: b(0,+)-type amino acid transporter 1-like
RS013870	GO:0006820	anion transport	BP	-1.185159015	-0.789079424	-0.942978819	4.024142004	15.86729932	0.001207277	0.005765813	PREDICTED: sodium- and chloride-dependent glycine transporter 1-like
RS007810	GO:0006820	anion transport	BP	-0.820216184	-1.018356117	-0.958242629	3.620004773	15.51103249	0.001428141	0.006659019	PREDICTED: vesicular glutamate transporter 1
RS000928	GO:0006820	anion transport	BP	-2.818387407	-2.387215456	-1.195316578	0.324906115	15.29049888	0.001584497	0.007309578	B(0,+)-type amino acid transporter 1
RS006318	GO:0006820	anion transport	BP	-3.618949223	-2.702749807	-1.072386578	0.467850565	14.73641507	0.002056334	0.009147816	PREDICTED: bumetanide-sensitive sodium-(potassium)-chloride cotransporter
RS005708	GO:0006820	anion transport	BP	-2.57707626	-0.767810406	-0.121337824	1.208165742	14.61680361	0.002175202	0.009598631	PREDICTED: sodium- and chloride-dependent glycine transporter 1-like
RS008723	GO:0006820	anion transport	BP	0.765724899	0.492155106	-0.726953396	2.604372289	14.25219643	0.002581214	0.011140444	PREDICTED: excitatory amino acid transporter
RS011379	GO:0006820	anion transport	BP	-0.945895165	-0.137739549	0.02413553	6.197611827	14.11352967	0.002754622	0.01177587	PREDICTED: chloride intracellular channel exc-4
RS012010	GO:0006820	anion transport	BP	-0.658055692	-0.163236513	0.25753802	6.662027841	13.45566221	0.003748014	0.01543574	PREDICTED: sodium-dependent phosphate transporter 1-B-like
RS010905	GO:0006820	anion transport	BP	-0.573248945	-0.768712677	-0.837001645	4.71486708	13.23543162	0.00415417	0.016871208	PREDICTED: gamma-aminobutyric acid receptor subunit beta
RS005241	GO:0006820	anion transport	BP	-1.24648029	-0.946331544	-0.692073388	4.41212653	12.5745733	0.005653054	0.021933014	PREDICTED: high-affinity choline transporter 1
RS002985	GO:0006820	anion transport	BP	-0.571114377	-0.476136182	-0.859409917	5.548946497	12.07709757	0.007123669	0.026732864	PREDICTED: Y+L amino acid transporter 2
RS010641	GO:0006820	anion transport	BP	-0.807148767	-0.226247423	-0.169065413	4.942005241	11.48155079	0.009387573	0.033673341	PREDICTED: probable phospholipid-transporting ATPase VD
RS006740	GO:0006820	anion transport	BP	-0.43064001	-0.612335737	-0.738849827	6.295225865	10.98541247	0.011805018	0.040967869	PREDICTED: glutamate-gated chloride channel
RS007836	GO:0006820	anion transport	BP	-0.670404831	1.05713246	-0.759263575	1.495067002	10.91840834	0.012175354	0.042079146	PREDICTED: bestrophin-4
RS012007	GO:0006820	anion transport	BP	-0.8805953	0.446042174	-0.616249485	2.603532973	10.7020781	0.013450915	0.045771636	PREDICTED: sodium-dependent phosphate transporter 2
RS003950	GO:0006820	anion transport	BP	-0.587671115	-0.868990799	-0.714405964	4.844171479	10.0941278	0.017782729	0.057765203	PREDICTED: cationic amino acid transporter 2
RS006809	GO:0006820	anion transport	BP	-0.069599294	0.161390813	-0.669189997	5.521140821	9.427681054	0.02411329	0.074118459	PREDICTED: sodium bicarbonate cotransporter 3
RS009139	GO:0006820	anion transport	BP	0.649351007	1.292845053	0.336142374	2.039377777	9.031613663	0.028873505	0.085540561	PREDICTED: Y+L amino acid transporter 2
RS007859	GO:0006820	anion transport	BP	-1.037355681	-0.953164057	-1.137934678	2.631254112	8.895408464	0.0307143	0.08987358	PREDICTED: proton-coupled amino acid transporter 4
RS002100	GO:0006820	anion transport	BP	0.521250686	0.500182617	0.580767602	6.787528007	8.558799736	0.035770127	0.101347352	PREDICTED: methylosome subunit pICln
RS006017	GO:0006820	anion transport	BP	-0.226333875	0.829010789	0.173010283	3.457042927	8.42538268	0.037991671	0.106302401	PREDICTED: cationic amino acid transporter 2
RS002298	GO:0006820	anion transport	BP	0.444685109	0.888871791	0.46931162	5.877833769	8.218233291	0.041710358	0.114594295	PREDICTED: battenin
RS012784	GO:0006820	anion transport	BP	-0.964188065	-1.053670411	-1.487092291	6.693074429	8.119987423	0.043595935	0.118762068	PREDICTED: excitatory amino acid transporter-like
RS008614	GO:0006820	anion transport	BP	-0.377384484	0.178151297	0.139169662	6.960985845	7.911936368	0.047867212	0.127882831	PREDICTED: H(+)/Cl(-) exchange transporter 5
RS003841	GO:0006820	anion transport	BP	-0.018830809	0.667905762	-0.171179406	4.919354176	7.849821458	0.04921958	0.130930145	PREDICTED: major facilitator superfamily domain-containing protein 10
RS012051	GO:0006820	anion transport	BP	-1.206457775	-0.320139517	-0.400442703	3.93903891	7.774642457	0.050906164	0.13434875	PREDICTED: sodium-independent sulfate anion transporter-like
RS006810	GO:0006820	anion transport	BP	-0.692918241	-0.709642301	-0.469276201	5.552917252	7.276110601	0.063598968	0.159523084	PREDICTED: Y+L amino acid transporter 2
RS015105	GO:0006820	anion transport	BP	-0.678666411	-2.224992259	-0.562865466	0.559101899	7.185153044	0.066224707	0.164438873	PREDICTED: organic cation transporter 1
RS003825	GO:0006820	anion transport	BP	-0.116552603	-0.74841501	-0.195189608	7.935069367	6.915883201	0.07462774	0.18005155	PREDICTED: H(+)/Cl(-) exchange transporter 7
RS012048	GO:0006820	anion transport	BP	-0.674894361	-1.155560739	-0.486644875	2.649535097	6.691851744	0.082395778	0.193852499	PREDICTED: sodium-independent sulfate anion transporter-like
RS009471	GO:0006820	anion transport	BP	0.454440582	0.068570398	0.92902968	1.975531925	6.680170813	0.082821497	0.194672252	PREDICTED: potassium voltage-gated channel protein Shal
RS012532	GO:0006820	anion transport	BP	0.52422059	0.27169055	0.170626759	5.950381512	5.527522768	0.137001687	0.282351902	PREDICTED: adenosine 3'-phospho 5'-phosphosulfate transporter 2
RS012098	GO:0006820	anion transport	BP	-0.722635213	-0.863193595	-0.599560773	3.5591329	5.486483871	0.13944927	0.285797251	PREDICTED: protein tweety
RS011419	GO:0006820	anion transport	BP	0.142670051	0.566918683	0.76286631	3.172442667	5.332525292	0.149005743	0.30010848	PREDICTED: putative inorganic phosphate cotransporter
RS002769	GO:0006820	anion transport	BP	-0.180596666	-0.510025269	-0.494314256	5.266272897	5.147923967	0.161280396	0.318264698	PREDICTED: mitochondrial 2-oxoglutarate/malate carrier protein
RS006076	GO:0006820	anion transport	BP	-0.399500112	-0.234112198	-0.791572834	3.218719557	5.135126595	0.162165734	0.319411206	PREDICTED: cationic amino acid transporter 4
RS007406	GO:0006820	anion transport	BP	1.53218285	1.416234104	0.234676903	0.509107572	4.915967777	0.17805483	0.341676447	PREDICTED: sodium-independent sulfate anion transporter-like
RS013193	GO:0006820	anion transport	BP	-0.571318105	-0.638002435	-0.492911161	3.125045391	4.612135042	0.202503545	0.372595481	PREDICTED: gamma-aminobutyric acid receptor subunit beta-like
RS002889	GO:0006820	anion transport	BP	-0.128703142	0.3529612	0.286577104	4.360050122	4.605415706	0.203077987	0.373149135	PREDICTED: bifunctional arginine demethylase and lysyl-hydroxylase PSR
RS000049	GO:0006820	anion transport	BP	-0.167016261	-0.078808593	0.315739518	4.19490186	3.918242209	0.270428905	0.452114912	PREDICTED: protein I'm not dead yet-like
RS000693	GO:0006820	anion transport	BP	-0.697277451	-0.63859341	-1.105155719	1.668516432	3.761841056	0.288355965	0.47128419	PREDICTED: solute carrier family 12 member 4
RS008419	GO:0006820	anion transport	BP	-0.579270886	-0.22684126	-0.07023873	5.06982487	3.507414936	0.319801701	0.502353125	PREDICTED: chloride channel protein 2
RS013707	GO:0006820	anion transport	BP	-0.500388832	-0.162680031	-0.110632879	6.260634623	3.457673335	0.326293381	0.508267019	PREDICTED: phospholipid scramblase 2
RS015453	GO:0006820	anion transport	BP	-0.09263959	0.415348464	0.277714417	5.686672294	3.355959113	0.33992981	0.521573591	PREDICTED: sodium-dependent phosphate transporter 2
RS002254	GO:0006820	anion transport	BP	0.026779126	-0.240562604	-0.407019639	8.0023415	2.926808228	0.403048115	0.579800873	PREDICTED: non-specific lipid-transfer protein
RS007790	GO:0006820	anion transport	BP	0.13034852	0.225712601	0.386180211	4.625198528	2.606288996	0.456388071	0.628856824	PREDICTED: mitochondrial glutamate carrier 1-like
RS007514	GO:0006820	anion transport	BP	-0.393644456	0.206862252	-0.369998274	2.851975701	2.440356048	0.48616657	0.653933758	PREDICTED: solute carrier family 26 member 10-like
RS004471	GO:0006820	anion transport	BP	0.302505199	0.1016203	-0.272900349	3.077100879	2.396738775	0.494241988	0.660492741	PREDICTED: Y+L amino acid transporter 2
RS005021	GO:0006820	anion transport	BP	-0.06781788	-0.474899669	-0.350734329	3.155367473	2.352458366	0.50254583	0.667200314	PREDICTED: molybdate-anion transporter-like
RS002215	GO:0006820	anion transport	BP	-0.459893543	-0.373663745	-0.327616751	3.245153068	2.258109459	0.520593488	0.682313913	PREDICTED: CMP-sialic acid transporter 1
RS010646	GO:0006820	anion transport	BP	-0.387797302	-0.153467445	-0.201059514	5.65127642	2.191739529	0.533577259	0.692056848	PREDICTED: probable phospholipid-transporting ATPase IA
RS010044	GO:0006820	anion transport	BP	-0.032740719	-0.492484393	-0.25544283	2.787037183	2.091316821	0.553671426	0.707481528	PREDICTED: solute carrier family 35 member C2
RS015410	GO:0006820	anion transport	BP	-0.258061334	-0.246025988	-0.260658269	5.743258539	1.692882375	0.638517364	0.773443079	PREDICTED: protein slowmo
RS012406	GO:0006820	anion transport	BP	-0.118916496	-0.22943197	0.145617021	8.722210769	1.650202623	0.648061031	0.779972717	PREDICTED: probable phospholipid-transporting ATPase IIB
RS009390	GO:0006820	anion transport	BP	-0.711993959	-0.803385471	-0.355292865	0.366452897	1.289104566	0.731721917	0.839984739	PREDICTED: gamma-aminobutyric acid receptor alpha-like
RS002882	GO:0006820	anion transport	BP	0.362043543	0.12287268	0.14951259	3.567153224	1.277605364	0.734457649	0.842342193	PREDICTED: putative sodium-coupled neutral amino acid transporter 7
RS008405	GO:0006820	anion transport	BP	-0.507380646	-0.391727947	-0.160990784	2.501471485	1.183658987	0.75692617	0.857267824	PREDICTED: solute carrier family 12 member 9
RS006075	GO:0006820	anion transport	BP	-0.226394382	-0.065362369	0.088572196	3.008430896	0.898142308	0.82587609	0.901150881	PREDICTED: acetyl-coenzyme A transporter 1
RS013172	GO:0006820	anion transport	BP	0.064707769	0.050177661	0.256276698	4.54520608	0.870549691	0.832528393	0.905630734	PREDICTED: N-acetyl-D-glucosamine kinase
RS006019	GO:0006820	anion transport	BP	0.016800687	-0.569398535	-0.206623396	0.539126613	0.775635116	0.855285471	0.920559373	PREDICTED: cationic amino acid transporter 2
RS014940	GO:0006820	anion transport	BP	0.00473671	-0.350880149	-0.354295712	0.736422598	0.582482205	0.900430699	0.946555742	PREDICTED: potassium voltage-gated channel protein Shal
RS004110	GO:0006820	anion transport	BP	-0.109326082	-0.245764423	-0.393853677	2.856144774	0.536964028	0.910698682	0.95276119	PREDICTED: phospholipase A2 inhibitor and Ly6/PLAUR domain-containing protein
RS007404	GO:0006820	anion transport	BP	0.341548622	0.423696565	0.486833568	3.079113058	0.267214539	0.9660718	0.982215891	PREDICTED: sodium-independent sulfate anion transporter-like
RS007304	GO:0006820	anion transport	BP	-0.03874979	-0.117709759	-0.024621974	4.815387852	0.237457293	0.971327222	0.985126901	PREDICTED: solute carrier family 17 member 9
RS002585	GO:0006820	anion transport	BP	0.061337763	0.015683494	0.119579633	4.655608643	0.205533408	0.976691112	0.987602807	PREDICTED: mitochondrial pyruvate carrier 1
RS008706	GO:0006820	anion transport	BP	0.063443874	0.058144534	-0.01579188	5.633533699	0.196321115	0.978180999	0.988383465	PREDICTED: ATP-binding cassette sub-family D member 3

RS005614	GO:0006820	anion transport	BP	-0.026120006	0.069729297	0.030053445	4.472662817	0.146084989	0.985784124	0.992693667	PREDICTED: mitochondrial folate transporter/carrier
RS003373	GO:0016758	transferase activity, transferring hexosyl groups	MF	2.394141754	0.518855653	-1.476597425	5.561080032	174.1882806	1.58642E-37	8.84041E-36	PREDICTED: UDP-glucuronosyltransferase 2C1-like
RS011942	GO:0016758	transferase activity, transferring hexosyl groups	MF	-0.498390242	3.974481158	0.858281392	5.803436912	146.0821711	1.84434E-31	8.15005E-30	PREDICTED: 2-hydroxyacylsphingosine 1-beta-galactosyltransferase
RS008883	GO:0016758	transferase activity, transferring hexosyl groups	MF	-2.635704858	-2.991775763	-1.126686458	4.895920198	91.7066491	9.41792E-20	2.36274E-18	UDP-glucuronosyltransferase 2B31
RS014377	GO:0016758	transferase activity, transferring hexosyl groups	MF	-2.879618381	-2.202364727	0.22442043	5.12611155	82.5458203	8.72689E-18	1.86282E-16	PREDICTED: UDP-glucuronosyltransferase 1-9-like
RS008158	GO:0016758	transferase activity, transferring hexosyl groups	MF	-2.795604811	-2.090262321	-0.775069233	3.968954702	80.55398436	2.33455E-17	4.81988E-16	PREDICTED: UDP-glucuronosyltransferase 2B23-like
RS009152	GO:0016758	transferase activity, transferring hexosyl groups	MF	-0.076188961	2.318086608	-0.327763265	4.849879578	67.57555546	1.4102E-14	2.35857E-13	PREDICTED: alpha-(1,6)-fucosyltransferase
RS007005	GO:0016758	transferase activity, transferring hexosyl groups	MF	-1.957650605	-3.444698739	-1.402146743	3.791829129	65.05107475	4.89142E-14	7.89775E-13	UDP-glucuronosyltransferase 2C1
RS007007	GO:0016758	transferase activity, transferring hexosyl groups	MF	-0.721518244	-2.957994238	-1.525438971	5.459856141	63.77955032	9.1492E-14	1.4512E-12	UDP-glucuronosyltransferase 2C1
RS002511	GO:0016758	transferase activity, transferring hexosyl groups	MF	-0.69900862	-1.454715384	-1.592361101	6.212273362	61.91143998	2.29498E-13	3.52906E-12	PREDICTED: glycogen [starch] synthase
RS007006	GO:0016758	transferase activity, transferring hexosyl groups	MF	-1.668917347	-3.810763551	-1.549924403	5.684810206	60.5350737	4.51777E-13	6.78959E-12	UDP-glucuronosyltransferase 2C1
RS003633	GO:0016758	transferase activity, transferring hexosyl groups	MF	2.406817281	4.495780859	0.555172218	1.526066816	53.73260343	1.2795E-11	1.70699E-10	PREDICTED: lipase 3-like
RS007002	GO:0016758	transferase activity, transferring hexosyl groups	MF	-2.468593161	-5.0492494	-2.627899474	3.73092382	48.053052	2.07484E-10	2.50773E-09	UDP-glucuronosyltransferase 2C1
RS014374	GO:0016758	transferase activity, transferring hexosyl groups	MF	-2.757401794	-1.293839431	0.61208896	5.598754173	44.48882029	1.18821E-09	1.3445E-08	PREDICTED: UDP-glucuronosyltransferase 1-9-like
RS003365	GO:0016758	transferase activity, transferring hexosyl groups	MF	0.856897098	0.731950102	-0.93990766	5.568310002	42.69422487	2.85778E-09	3.12692E-08	PREDICTED: UDP-glucuronosyltransferase 1-7C-like
RS003374	GO:0016758	transferase activity, transferring hexosyl groups	MF	-2.112223946	-2.113156246	1.013788856	5.111927965	39.82788732	1.15888E-08	1.19142E-07	PREDICTED: UDP-glucuronosyltransferase 2C1-like
RS001802	GO:0016758	transferase activity, transferring hexosyl groups	MF	-1.504319905	-3.880991983	-1.493255907	2.87429931	39.50810122	1.3546E-08	1.38248E-07	UDP-glucuronosyltransferase 2C1
RS003366	GO:0016758	transferase activity, transferring hexosyl groups	MF	-2.213981747	-4.422449764	-1.127783247	3.308359091	38.76633937	1.94519E-08	1.93505E-07	PREDICTED: UDP-glucuronosyltransferase 2C1-like
RS006626	GO:0016758	transferase activity, transferring hexosyl groups	MF	-2.700806188	0.747961514	2.346199654	7.636078588	38.74712028	1.96351E-08	1.95173E-07	chitin synthase
RS014375	GO:0016758	transferase activity, transferring hexosyl groups	MF	-2.786211871	-1.430864034	0.9193075	4.949678243	38.53374047	2.17885E-08	2.14882E-07	PREDICTED: UDP-glucuronosyltransferase 2B31-like
RS008155	GO:0016758	transferase activity, transferring hexosyl groups	MF	-2.182264988	-2.377110203	-0.172911265	6.890910628	38.26508249	2.48381E-08	2.44193E-07	PREDICTED: UDP-glucuronosyltransferase 2B23-like
RS005966	GO:0016758	transferase activity, transferring hexosyl groups	MF	0.813382742	1.201323264	-0.273628464	6.163653891	36.410532	6.13166E-08	5.8193E-07	PREDICTED: protein O-mannosyltransferase 1
RS002163	GO:0016758	transferase activity, transferring hexosyl groups	MF	-0.458782111	1.077749636	0.903170703	4.233761172	36.05963192	7.27411E-08	6.84679E-07	PREDICTED: beta-1,3-galactosyltransferase 5
RS012629	GO:0016758	transferase activity, transferring hexosyl groups	MF	-1.664809796	-2.643705194	-0.289606186	5.568377112	34.74408644	1.37974E-07	1.25643E-06	PREDICTED: UDP-glucuronosyltransferase 2B23-like
RS007000	GO:0016758	transferase activity, transferring hexosyl groups	MF	0.34917013	-0.237458639	-2.707698005	5.363500758	32.81100863	3.53027E-07	3.1026E-06	PREDICTED: UDP-glucuronosyltransferase 2C1-like
RS007008	GO:0016758	transferase activity, transferring hexosyl groups	MF	-2.246581332	-4.732977392	-4.18045202	1.765316562	32.00256756	5.22695E-07	4.52428E-06	UDP-glucuronosyltransferase 2C1
RS001804	GO:0016758	transferase activity, transferring hexosyl groups	MF	-1.140184699	-2.043399778	-0.266753479	3.969583194	31.59405457	6.37275E-07	5.44494E-06	UDP-glucuronosyltransferase 2C1
RS001803	GO:0016758	transferase activity, transferring hexosyl groups	MF	-2.556565135	-5.115278842	-2.022389619	2.829005306	31.37957173	7.07144E-07	6.00524E-06	glucosyl/glucuronosyl transferase
RS003370	GO:0016758	transferase activity, transferring hexosyl groups	MF	1.148136507	-0.965311261	-0.4811304	3.625502469	30.18481425	1.26188E-06	1.04142E-05	PREDICTED: UDP-glucuronosyltransferase 1-7C-like
RS008055	GO:0016758	transferase activity, transferring hexosyl groups	MF	-0.825323661	-2.247239237	-0.017353512	3.705997202	26.6008784	7.13793E-06	5.25395E-05	PREDICTED: UDP-glucuronosyltransferase 2B15-like
RS008160	GO:0016758	transferase activity, transferring hexosyl groups	MF	-1.86019723	-1.119619791	1.214513641	3.193269805	24.5107924	1.95394E-05	0.000134543	PREDICTED: UDP-glucuronosyltransferase 2C1-like
RS012932	GO:0016758	transferase activity, transferring hexosyl groups	MF	-1.049335672	-1.635763072	-1.382307762	3.469160191	21.88763407	6.88387E-05	0.000432396	PREDICTED: beta-1,4-mannosyltransferase egh
RS004481	GO:0016758	transferase activity, transferring hexosyl groups	MF	1.158258886	1.648956931	2.316008472	2.609786787	20.65713141	0.000124025	0.000742383	PREDICTED: beta-1,3-galactosyltransferase 5
RS006448	GO:0016758	transferase activity, transferring hexosyl groups	MF	-0.438550285	-0.842818516	-1.111661943	7.029395929	20.1090662	0.000161133	0.000940802	PREDICTED: glycogen phosphorylase
RS009017	GO:0016758	transferase activity, transferring hexosyl groups	MF	-0.045189261	0.043390885	0.916173056	6.536550332	19.702086	0.000195664	0.001120594	PREDICTED: beta-1,3-galactosyltransferase 5-like
RS001711	GO:0016758	transferase activity, transferring hexosyl groups	MF	0.690769604	1.181462709	0.955368065	6.847403504	19.20530543	0.000247934	0.001389623	PREDICTED: probable dolichol-phosphate mannosyltransferase
RS007003	GO:0016758	transferase activity, transferring hexosyl groups	MF	-1.579708317	-3.271522625	-2.234795788	1.587150287	18.17783497	0.000404221	0.00215163	PREDICTED: 2-hydroxyacylsphingosine 1-beta-galactosyltransferase-like
RS008159	GO:0016758	transferase activity, transferring hexosyl groups	MF	-2.198687	-0.932718964	1.058559857	2.638901427	17.68068324	0.000511838	0.002670292	PREDICTED: UDP-glucuronosyltransferase 2B23-like
RS011385	GO:0016758	transferase activity, transferring hexosyl groups	MF	-1.067362169	-1.417870026	-0.982647581	7.67666387	17.57511164	0.000538125	0.00279355	PREDICTED: Ig-like and fibronectin type-III domain-containing protein T04A11.3
RS014418	GO:0016758	transferase activity, transferring hexosyl groups	MF	-0.478113679	-0.820374615	0.805474325	2.345331841	14.9793906	0.001834346	0.008283168	PREDICTED: alpha-1,6-mannosyl-glycoprotein 2-beta-N-acetylglucosaminyltransferase
RS010291	GO:0016758	transferase activity, transferring hexosyl groups	MF	-1.148046164	-1.387251265	0.083086546	1.759013597	13.03205805	0.004567788	0.018268253	PREDICTED: galactoside 2-alpha-L-fucosyltransferase 3-like
RS014435	GO:0016758	transferase activity, transferring hexosyl groups	MF	-0.984407799	-0.946476751	-0.953386041	3.329331458	13.0094291	0.004616259	0.018438682	PREDICTED: chondroitin sulfate N-acetylgalactosaminyltransferase 1
RS006766	GO:0016758	transferase activity, transferring hexosyl groups	MF	-0.318612109	-0.764780567	0.962282512	4.270639859	12.4793297	0.005909213	0.022737743	PREDICTED: alpha-(1,3)-fucosyltransferase 7-like
RS001133	GO:0016758	transferase activity, transferring hexosyl groups	MF	1.00904016	0.474949441	0.334976687	4.645037343	12.45381435	0.005979765	0.022963196	PREDICTED: bolA-like protein 2
RS003371	GO:0016758	transferase activity, transferring hexosyl groups	MF	0.472707825	-0.12338578	-0.39256252	5.540350268	12.16672405	0.006833319	0.025827977	PREDICTED: UDP-glucuronosyltransferase 2B15-like
RS006443	GO:0016758	transferase activity, transferring hexosyl groups	MF	0.143296238	-0.770738062	-0.789685541	3.548052193	12.1322361	0.006943634	0.026189915	PREDICTED: alpha,alpha-trehalose-phosphate synthase [UDP-forming]
RS004813	GO:0016758	transferase activity, transferring hexosyl groups	MF	0.328719298	0.63887773	0.7101367	6.230566513	12.08586277	0.007094745	0.026640194	PREDICTED: dolichyl-phosphate beta-glucosyltransferase
RS005798	GO:0016758	transferase activity, transferring hexosyl groups	MF	0.42141247	0.736875665	0.248473058	5.783054674	10.99667203	0.011743885	0.040797551	PREDICTED: protein O-mannosyl-transferase 2
RS014419	GO:0016758	transferase activity, transferring hexosyl groups	MF	-0.812097852	-0.035393336	0.555610255	3.206085641	10.88864079	0.012343532	0.042590258	PREDICTED: alpha-1,6-mannosyl-glycoprotein 2-beta-N-acetylglucosaminyltransferase
RS008660	GO:0016758	transferase activity, transferring hexosyl groups	MF	-0.610580371	-0.844004978	-0.632634161	7.281264035	10.5950421	0.01412984	0.047746499	PREDICTED: glycogen debranching enzyme
RS003249	GO:0016758	transferase activity, transferring hexosyl groups	MF	-0.207283219	-0.369134294	-0.971045776	4.452945458	10.45254875	0.015086265	0.05039693	PREDICTED: protein O-linked-mannose beta-1,2-N-acetylglucosaminyltransferase 1
RS012938	GO:0016758	transferase activity, transferring hexosyl groups	MF	-1.846939656	-0.327189547	0.081992913	1.097980344	9.391278764	0.024516548	0.075087891	PREDICTED: beta-1,3-galactosyltransferase 5
RS011064	GO:0016758	transferase activity, transferring hexosyl groups	MF	-1.082972026	-1.027491454	-0.806343821	3.290957675	9.019949401	0.029026821	0.085833243	PREDICTED: glycoprotein 3-alpha-L-fucosyltransferase A
RS004501	GO:0016758	transferase activity, transferring hexosyl groups	MF	-0.612352886	-1.4810284	-0.556824138	2.311605203	8.894183325	0.030731368	0.089881756	PREDICTED: basement membrane-specific heparan sulfate proteoglycan core protein-like
RS005796	GO:0016758	transferase activity, transferring hexosyl groups	MF	0.276557201	0.51395375	0.608133832	6.545528814	8.82830223	0.031662987	0.092263689	PREDICTED: 15 kDa selenoprotein
RS014200	GO:0016758	transferase activity, transferring hexosyl groups	MF	0.368512158	-0.23900377	-0.315539871	4.740713374	8.620559497	0.034785227	0.09929734	PREDICTED: sphingosine-1-phosphate lyase
RS015009	GO:0016758	transferase activity, transferring hexosyl groups	MF	0.452819	0.56796968	0.343760656	6.19776174	8.407300491	0.038302942	0.107030675	PREDICTED: dolichyl-diphosphooligosaccharide--protein glycosyltransferase subunit DAD1
RS012691	GO:0016758	transferase activity, transferring hexosyl groups	MF	-0.172595861	-0.578904307	-0.031671583	6.933895552	8.088105422	0.044225497	0.120245231	PREDICTED: 1,4-alpha-glucan-branching enzyme
RS013516	GO:0016758	transferase activity, transferring hexosyl groups	MF	-0.071257629	-0.443466359	-0.469509849	6.036825379	7.367035791	0.061075118	0.15485807	PREDICTED: exostosin-3
RS005354	GO:0016758	transferase activity, transferring hexosyl groups	MF	-1.00471418	-0.664056052	-0.408294102	2.883865853	7.337282876	0.061890101	0.156389668	PREDICTED: alpha-1,3-mannosyl-glycoprotein 4-beta-N-acetylglucosaminyltransferase B
RS012830	GO:0016758	transferase activity, transferring hexosyl groups	MF	0.512471195	0.69308213	0.478993909	7.410547394	7.303674851	0.062823366	0.158207852	PREDICTED: glycosyltransferase-like protein LARGE1
RS003372	GO:0016758	transferase activity, transferring hexosyl groups	MF	0.279193296	-0.310394924	-1.009526263	2.057829716	7.289239778	0.063228389	0.158878358	PREDICTED: UDP-glucuronosyltransferase 1-7C-like
RS014164	GO:0016758	transferase activity, transferring hexosyl groups	MF	-0.323373255	-0.480954149	-0.674381743	7.10845976	7.054385301	0.070184382	0.171965391	PREDICTED: exostosin-2
RS013092	GO:0016758	transferase activity, transferring hexosyl groups	MF	-1.21991486	-0.311211194	-1.526773013	0.464605072	5.999428186	0.111638048	0.243540502	PREDICTED: polypeptide N-acetylgalactosaminyltransferase 2
RS002734	GO:0016758	transferase activity, transferring hexosyl groups	MF	0.299931428	-0.05370325	-0.483067767	7.336391207	5.907492348	0.116198696	0.250669128	PREDICTED: dolichyl-diphosphooligosaccharide--protein glycosyltransferase subunit 1
RS013091	GO:0016758	transferase activity, transferring hexosyl groups	MF	-0.634795047	0.023977642	-0.224683025	3.598027978	5.731282535	0.125441482	0.265335912	PREDICTED: polypeptide N-acetylgalactosaminyltransferase 14
RS010624	GO:0016758	transferase activity, transferring hexosyl groups	MF	-1.888144745	-0.350620517	-1.036675402	0.280983459	5.728136435	0.125612703	0.265452684	PREDICTED: exostosin-1
RS005181	GO:0016758	transferase activity, transferring hexosyl groups	MF	-0.168717926	0.322198012	0.688998249	2.903388389	5.420247571	0.143487277	0.292076736	PREDICTED: beta-1,3-galactosyltransferase 1-like
RS007245	GO:0016758	transferase activity, transferring hexosyl groups	MF	-0.109691638	-0.538704272	-0.521010048	4.600455628	5.356909305	0.147451966	0.297788657	PREDICTED: beta-1,3-galactosyltransferase 6
RS011885	GO:0016758	transferase activity, transferring hexosyl groups	MF	0.560958428	0.381144006	0.226682277	7.447775785	5.334338605	0.148889666	0.299945651	PREDICTED: dolichyl-diphosphooligosaccharide--protein glycosyltransferase subunit STT3B
RS008006	GO:0016758	transferase activity, transferring hexosyl groups	MF	-1.537762826	-0.128930159	-0.462894442	1.415657251	5.194123574	0.158121985	0.31385158	PREDICTED: beta-1,3-galactosyltransferase 1-like
RS011641	GO:0016758	transferase activity, transferring hexosyl groups	MF	-2.074551338	-0.763334889	-1.763867752	0.058250841	4.989449686	0.172571322	0.333696182	PREDICTED: exostosin-1
RS012633	GO:0016758	transferase activity, transferring hexosyl groups	MF	0.3400712	-0.042971241	-0.128072376	5.95450125	4.828059073	0.184829233	0.350404841	PREDICTED: probable C-mannosyltransferase DPY19L1
RS011065	GO:0016758	transferase activity, transferring hexosyl groups	MF	-0.875027536	-0.699860567	-0.617836046	1.97912169	4.744920077	0.191457	0.358704174	PREDICTED: glycoprotein 3-alpha-L-fucosyltransferase A
RS001682	GO:0016758	transferase activity, transferring hexosyl groups	MF	0.427496794	0.162370784	-0.166794293	4.20654832	4.689803666	0.195972291	0.364345296	PREDICTED: putative Dol-P-Glc:Glc(2)Man(9)GlcNAc(2)-PP-Dol alpha-1,2-glucosyltransferase
RS003712	GO:0016758	transferase activity, transferring hexosyl groups	MF	0.03100834	-0.555218793	-0.842108264	3.063114051	4.678534462	0.196907622	0.365525714	PREDICTED: probable dolichyl pyrophosphate Glc1Man9GlcNAc2 alpha-1,3-glucosyltransferase
RS007095	GO:0016758	transferase activity, transferring hexosyl groups	MF	0.192687609	0.009365646	-0.454674091	8.985348469	4.560229052	0.206980499	0.378277813	PREDICTED: UDP-glucose:glycoprotein glucosyltransferase
RS010338	GO:0016758	transferase activity, transferring hexosyl groups	MF	-0.000753394	0.421834083	0.188139531	4.933078321	4.386735521	0.222618469	0.396500777	PREDICTED: putative bifunctional UDP-N-acetylglucosamine transferase and deubiquitinase ALG13
RS005227	GO:0016758	transferase activity, transferring hexosyl groups	MF	0.455821833	0.259038003	0.102179355	5.644742258	4.314230826	0.229471159	0.403821294	PREDICTED: DNA polymerase beta-like
RS014997	GO:0016758	transferase activity, transferring hexosyl groups	MF	-0.068392036	-0.306113841	0.205901561	4.99125296	4.308069794	0.23006232	0.404608974	PREDICTED: lethal(2)neighbour of Tid protein
RS014750	GO:0016758	transferase activity, transferring hexosyl groups	MF	0.175545865	0.023639013	-0.262438133	5.756277047	4.136485535	0.247098318	0.42536307	PREDICTED: GDP-D-glucose phosphorylase 1
RS012431	GO:0016758	transferase activity, transferring hexosyl groups	MF	0.101037027	-0.018627158	-0.475372339	3.924987682	3.858618333	0.277141065	0.459554256	PREDICTED: UDP-GalNAc:beta-1,3-N-acetylgalactosaminyltransferase 2
RS001134	GO:0016758	transferase activity, transferring hexosyl groups	MF	-0.472202252	-0.066367351	-0.014278242	4.816744369	3.847768291	0.278378555	0.46099928	PREDICTED: dolichyl pyrophosphate Man9GlcNAc2 alpha-1,3-glucosyltransferase
RS007814	GO:0016758	transferase activity, transferring hexosyl groups	MF	-0.022934896	0.013283399	0.724460106	4.088570218	3.831671389	0.280223647	0.463019761	PREDICTED: fringe glycosyltransferase
RS009912	GO:0016758	transferase activity, transferring hexosyl groups	MF	-0.007471245	-0.317003797	-0.486745701	4.012886914	3.810008462	0.282724123	0.465502367	PREDICTED: phosphatidylinositol N-acetylglucosaminyltransferase subunit P
RS000878	GO:0016758	transferase activity, transferring hexosyl groups	MF	-0.536410077	-0.620333003	-0.480574696	3.333423954	3.791921767	0.284827164	0.467988351	PREDICTED: protein O-linked-mannose beta-1,2-N-acetylglucosaminyltransferase 1
RS001681	GO:0016758	transferase activity, transferring hexosyl groups	MF	-0.413729733	-0.679395811	-0.380973277	3.503026248	3.786679393	0.285439347	0.46836187	PREDICTED: galactosylgalactosylxylosylprotein 3-beta-glucuronosyltransferase P-like
RS014614	GO:0016758	transferase activity, transferring hexosyl groups	MF	0.17118901	-0.326282215	-0.090631117	4.420046044	3.544195221	0.315075223	0.497775097	PREDICTED: phosphatidylinositol N-acetylglucosaminyltransferase subunit Q
RS010149	GO:0016758	transferase activity, transferring hexosyl groups	MF	-0.154098234	-0.466690935	-0.860655645	2.124840163	3.506315269	0.319943975	0.502353125	PREDICTED: O-glucosyltransferase rumi homolog
RS006323	GO:0016758	transferase activity, transferring hexosyl groups	MF	-0.317230354	0.003056746	0.337317135	4.743049644	3.318471262	0.345080052	0.525982127	PREDICTED: chondroitin sulfate synthase 1
RS002950	GO:0016758	transferase activity, transferring hexosyl groups	MF	0.051940014	0.251144097	-0.140848098	6.508547037	3.237096775	0.356493993	0.537429417	PREDICTED: GPI mannosyltransferase 2
RS013077	GO:0016758	transferase activity, transferring hexosyl groups	MF	0.320745853	-0.047417166	-0.242885267	3.632774111	3.029654065	0.387075662	0.566379794	PREDICTED: phosphatidylinositol N-acetylglucosaminyltransferase subunit H
RS004857	GO:0016758	transferase activity, transferring hexosyl groups	MF	0.001882822	-0.133955239	-0.647468617	2.077593454	2.936643799	0.40149696	0.578409142	PREDICTED: alpha-1,3-mannosyl-glycoprotein 2-beta-N-acetylglucosaminyltransferase
RS000021	GO:0016758	transferase activity, transferring hexosyl groups	MF	0.352698076	0.223428782	0.088380723	6.096238225	2.849526925	0.415411664	0.59115192	PREDICTED: KDEL motif-containing protein 1-like
RS006685	GO:0016758	transferase activity, transferring hexosyl groups	MF	0.007325471	-0.293223436	-0.432311597	3.533055228	2.809344423	0.421963968	0.597572721	PREDICTED: GDP-fucose protein O-fucosyltransferase 1
RS009609	GO:0016758	transferase activity, transferring hexosyl groups	MF	0.237545237	0.05526396	-0.190935826	4.176700496	2.498126856	0.475629697	0.645682607	PREDICTED: S-methyl-5'-thioadenosine phosphorylase
RS012264	GO:0016758	transferase activity, transferring hexosyl groups	MF	0.056631713	0.03848964	-0.253035463	6.466737478	2.48425876	0.478142596	0.647358402	PREDICTED: galactosylgalactosylxylosylprotein 3-beta-glucuronosyltransferase I
RS007162	GO:0016758	transferase activity, transferring hexosyl groups	MF	0.352804678	-0.213394026	-0.027296829	4.583000343	2.241629936	0.523795185	0.684160606	PREDICTED: dolichyl-diphosphooligosaccharide--protein glycosyltransferase subunit STT3A
RS009549	GO:0016758	transferase activity, transferring hexosyl groups	MF	-0.121708317	-0.303976597	-0.279583953	5.648786218	1.907941736	0.591731796	0.737708359	PREDICTED: chondroitin sulfate synthase 2
RS009803	GO:0016758	transferase activity, transferring hexosyl groups	MF	0.213911252	0.218935271	0.288323068	5.18116662	1.76239907	0.623151298	0.762368939	PREDICTED: stromal cell-derived factor 2
RS003681	GO:0016758	transferase activity, transferring hexosyl groups	MF	0.085803928	-0.429040718	-0.172407369	2.598676023	1.749886099	0.625900512	0.764462367	PREDICTED: phosphatidylinositol N-acetylglucosaminyltransferase subunit C
RS015538	GO:0016758	transferase activity, transferring hexosyl groups	MF	0.225640179	-0.10699019	0.104179018	4.908035582	1.70244913	0.636389435	0.77242565	PREDICTED: chitobiosyldiphosphodolichol beta-mannosyltransferase
RS005984	GO:0016758	transferase activity, transferring hexosyl groups	MF	0.152379143	-0.112920669	-0.068279543	4.98672439	1.116242857	0.773154511	0.868067033	PREDICTED: beta-1,3-galactosyltransferase brn
RS002699	GO:0016758	transferase activity, transferring hexosyl groups	MF	0.18641679	-0.113157012	0.19259875	3.645600491	1.098441167	0.777450405	0.870639375	PREDICTED: galactosylgalactosylxylosylprotein 3-beta-glucuronosyltransferase S
RS011344	GO:0016758	transferase activity, transferring hexosyl groups	MF	-0.159583809	-0.219183185	-0.241957742	3.786489846	0.684898687	0.876750051	0.933750515	PREDICTED: ceramide glucosyltransferase
RS005270	GO:0016758	transferase activity, transferring hexosyl groups	MF	-0.004544488	-0.216158524	-0.097291995	3.745309896	0.56443291	0.904524136	0.94929808	PREDICTED: xylosyltransferase oxt
RS011838	GO:0016758	transferase activity, transferring hexosyl groups	MF	0.075328587	0.16154379	0.135711958	4.870803793	0.532227388	0.911756246	0.953313256	PREDICTED: GPI mannosyltransferase 1
RS013735	GO:0016758	transferase activity, transferring hexosyl groups	MF	0.104677793	0.022858359	-0.09563073	2.80861291	0.320642672	0.956100531	0.976888698	PREDICTED: alpha-(1,3)-fucosyltransferase 10
RS004720	GO:0016758	transferase activity, transferring hexosyl groups	MF	-0.146782098	-0.172357517	-0.158017647	3.893542108	0.308665541	0.958390572	0.977721415	PREDICTED: protein O-linked-mannose beta-1,2-N-acetylglucosaminyltransferase 1
RS012220	GO:0016758	transferase activity, transferring hexosyl groups	MF	-0.029335723	0.050689391	0.128078453	3.427049826	0.305819403	0.958930318	0.977774901	PREDICTED: galactoside 2-alpha-L-fucosyltransferase 3-like
RS010208	GO:0006631	fatty acid metabolic process	BP	-0.084960135	7.892665833	1.075100468	4.896692372	313.5860255	1.14066E-67	1.42233E-65	PREDICTED: acyl-CoA Delta(11) desaturase-like

RS001262	GO:0006631	fatty acid metabolic process	BP	-2.399000696	4.824243006	1.184902522	2.908556297	139.6588544	4.4773E-30	1.86096E-28	PREDICTED: elongation of very long chain fatty acids protein AAEL008004
RS005642	GO:0006631	fatty acid metabolic process	BP	-0.158087639	3.50903035	0.033669894	7.188992405	105.9789213	8.04565E-23	2.4299E-21	PREDICTED: elongation of very long chain fatty acids protein 4-like
RS011319	GO:0006631	fatty acid metabolic process	BP	-2.200812301	2.311506339	4.542956934	3.965152586	105.9687966	8.0861E-23	2.43628E-21	PREDICTED: elongation of very long chain fatty acids protein AAEL008004-like
RS011853	GO:0006631	fatty acid metabolic process	BP	-1.313648639	-2.478519389	-2.71556709	4.85148492	76.50416751	1.72458E-16	3.38308E-15	PREDICTED: aldehyde dehydrogenase, dimeric NADP-preferring
RS010209	GO:0006631	fatty acid metabolic process	BP	-1.199116022	0.810918744	-0.247836435	5.677637623	69.45798048	5.5759E-15	9.71271E-14	PREDICTED: acyl-CoA Delta(11) desaturase
RS013244	GO:0006631	fatty acid metabolic process	BP	-1.611287022	-4.550784717	-2.084168564	3.087514358	63.42642333	1.08867E-13	1.71169E-12	PREDICTED: acyl-CoA Delta(11) desaturase-like
RS006876	GO:0006631	fatty acid metabolic process	BP	-1.906405126	-4.198252583	-2.68639647	6.979759297	60.5192047	4.55318E-13	6.83465E-12	PREDICTED: acyl-CoA Delta(11) desaturase-like
RS002789	GO:0006631	fatty acid metabolic process	BP	-1.372012942	-1.490274038	-0.050279956	6.171605621	58.36997718	1.31043E-12	1.89478E-11	PREDICTED: long-chain-fatty-acid--CoA ligase 5
RS001261	GO:0006631	fatty acid metabolic process	BP	-0.735144857	-2.614482301	-2.129153596	5.833143755	54.62533616	8.25351E-12	1.12616E-10	PREDICTED: elongation of very long chain fatty acids protein 7
RS001805	GO:0006631	fatty acid metabolic process	BP	-1.712509312	0.709000933	-0.641501281	4.340777854	48.29287055	1.84479E-10	2.24693E-09	PREDICTED: elongation of very long chain fatty acids protein AAEL008004-like
RS010737	GO:0006631	fatty acid metabolic process	BP	-0.645730611	1.009936317	1.210261513	4.679785325	45.64187507	6.75826E-10	7.83014E-09	PREDICTED: elongation of very long chain fatty acids protein AAEL008004
RS011394	GO:0006631	fatty acid metabolic process	BP	-1.130512888	-4.311859926	-0.867929923	2.581686454	42.35833137	3.36765E-09	3.65938E-08	PREDICTED: hydroxyacyl-coenzyme A dehydrogenase, mitochondrial-like
RS002535	GO:0006631	fatty acid metabolic process	BP	-0.91575853	-1.304494659	-0.314668602	6.514376839	41.85373159	4.30936E-09	4.64261E-08	PREDICTED: acetyl-CoA carboxylase
RS015505	GO:0006631	fatty acid metabolic process	BP	-0.873277592	-1.775991461	-0.249713989	6.67190604	41.13584257	6.11945E-09	6.48178E-08	PREDICTED: probable methylmalonate-semialdehyde dehydrogenase [acylating], mitochondrial
RS000244	GO:0006631	fatty acid metabolic process	BP	-2.077293385	0.322822201	0.471009763	5.277306481	39.6052201	1.2919E-08	1.32063E-07	PREDICTED: gamma-glutamyltranspeptidase 1-like
RS010472	GO:0006631	fatty acid metabolic process	BP	-0.707846719	-1.359223275	-1.145612806	6.054168603	37.56822123	3.48855E-08	3.377E-07	PREDICTED: isovaleryl-CoA dehydrogenase, mitochondrial
RS003648	GO:0006631	fatty acid metabolic process	BP	0.799121324	0.883024374	-0.745381312	6.113323662	35.82944046	8.13666E-08	7.62449E-07	PREDICTED: protein singed
RS010978	GO:0006631	fatty acid metabolic process	BP	-1.244943903	-1.839221985	-1.263048219	7.621355649	35.10486661	1.15766E-07	1.0681E-06	PREDICTED: fatty acid synthase
RS001808	GO:0006631	fatty acid metabolic process	BP	-1.457307761	-1.90386158	0.642631365	1.825702934	33.50863405	2.51557E-07	2.24211E-06	PREDICTED: elongation of very long chain fatty acids protein AAEL008004-like
RS013686	GO:0006631	fatty acid metabolic process	BP	0.294389451	-2.065909015	-0.50139354	4.039478596	33.22682692	2.88467E-07	2.55303E-06	PREDICTED: acyl-CoA Delta(11) desaturase-like
RS009061	GO:0006631	fatty acid metabolic process	BP	-0.381717049	0.872229307	1.423987272	3.354243538	31.77457362	5.83839E-07	5.01903E-06	PREDICTED: elongation of very long chain fatty acids protein AAEL008004
RS007469	GO:0006631	fatty acid metabolic process	BP	-0.888748458	-2.227463608	-1.919844509	7.982695057	31.31984973	7.27926E-07	6.17756E-06	PREDICTED: retinal dehydrogenase 1
RS007713	GO:0006631	fatty acid metabolic process	BP	-1.593472957	-0.392083561	-0.180235411	5.938924251	30.40817878	1.13245E-06	9.40771E-06	PREDICTED: ATP-binding cassette sub-family D member 2
RS002190	GO:0006631	fatty acid metabolic process	BP	-1.585867798	-2.762372308	0.098229096	2.73757327	29.10799577	2.12541E-06	1.69629E-05	PREDICTED: fatty acid 2-hydroxylase
RS000419	GO:0006631	fatty acid metabolic process	BP	0.040399421	-0.493996515	-1.164051968	4.981441137	24.64016581	1.83602E-05	0.000127118	PREDICTED: peroxisomal acyl-coenzyme A oxidase 3
RS007610	GO:0006631	fatty acid metabolic process	BP	-0.937977299	-0.674413913	0.09856611	5.486082389	23.09307983	3.86186E-05	0.000253578	PREDICTED: short-chain specific acyl-CoA dehydrogenase, mitochondrial
RS000681	GO:0006631	fatty acid metabolic process	BP	-1.508540822	0.325287212	0.756664918	5.674807397	22.14032278	6.09894E-05	0.000387147	PREDICTED: elongation of very long chain fatty acids protein AAEL008004-like
RS011502	GO:0006631	fatty acid metabolic process	BP	0.997139085	1.110937602	0.643258028	7.455027006	21.29469842	9.14347E-05	0.000561174	PREDICTED: 3-hydroxyacyl-CoA dehydrogenase type-2
RS013245	GO:0006631	fatty acid metabolic process	BP	-0.919192882	-3.212762255	-1.960378095	3.150082705	20.75615985	0.000118292	0.000710093	PREDICTED: acyl-CoA Delta(11) desaturase-like
RS013197	GO:0006631	fatty acid metabolic process	BP	-1.613311304	-1.711943527	-0.937859313	5.506175009	19.92371087	0.000176035	0.001019304	PREDICTED: fatty acid hydroxylase domain-containing protein 2
RS013198	GO:0006631	fatty acid metabolic process	BP	-0.170174414	-2.032426041	-0.722029511	3.416328583	19.90072697	0.000177976	0.001029596	PREDICTED: fatty acid hydroxylase domain-containing protein 2
RS010210	GO:0006631	fatty acid metabolic process	BP	-0.994802094	1.493392713	-0.157135873	2.117526785	19.31894942	0.000234868	0.001324644	PREDICTED: acyl-CoA Delta(11) desaturase-like
RS007875	GO:0006631	fatty acid metabolic process	BP	0.743606352	-0.046039924	0.105888728	5.909432159	18.82016803	0.000297832	0.001640095	PREDICTED: splicing factor 3B subunit 6-like protein
RS003219	GO:0006631	fatty acid metabolic process	BP	0.030734368	-1.119228443	-0.51621698	3.887749858	18.36336163	0.000370108	0.001991939	PREDICTED: probable peroxisomal acyl-coenzyme A oxidase 1
RS013246	GO:0006631	fatty acid metabolic process	BP	-1.373852886	-4.090748051	-2.164995168	2.336943256	17.88036325	0.000465559	0.002448119	PREDICTED: acyl-CoA Delta(11) desaturase-like
RS000435	GO:0006631	fatty acid metabolic process	BP	0.515479598	0.900602724	0.726672321	5.243663251	17.3682661	0.000593584	0.00304753	PREDICTED: 39S ribosomal protein L13, mitochondrial
RS011383	GO:0006631	fatty acid metabolic process	BP	-0.592908391	-0.975532691	-0.419225628	5.386382038	17.30604756	0.000611351	0.003123469	PREDICTED: 2-hydroxyacyl-CoA lyase 1
RS013812	GO:0006631	fatty acid metabolic process	BP	-0.90352517	-0.658147253	-0.537176094	4.464856555	15.24382038	0.001619709	0.007447467	PREDICTED: propionyl-CoA carboxylase beta chain, mitochondrial
RS013687	GO:0006631	fatty acid metabolic process	BP	-0.15225672	-2.860275429	-1.652752871	2.658895556	14.35252769	0.002462536	0.010708972	PREDICTED: acyl-CoA Delta(11) desaturase-like
RS015477	GO:0006631	fatty acid metabolic process	BP	-1.101064815	-0.393684254	-0.930550173	3.969326348	14.3061796	0.002516672	0.010910488	PREDICTED: pyruvate carboxylase, mitochondrial
RS003220	GO:0006631	fatty acid metabolic process	BP	-0.919595261	-1.193174547	-0.639458968	5.160771178	13.94596359	0.002979636	0.012596688	PREDICTED: probable peroxisomal acyl-coenzyme A oxidase 1
RS011854	GO:0006631	fatty acid metabolic process	BP	-1.112002956	0.070911347	-0.516191421	6.87646831	13.85956875	0.003102671	0.013038052	PREDICTED: aldehyde dehydrogenase, dimeric NADP-preferring
RS014031	GO:0006631	fatty acid metabolic process	BP	-0.521995584	-0.786620499	-1.18332395	3.371095243	12.38651585	0.006169869	0.023610856	PREDICTED: glutathione S-transferase-like
RS009864	GO:0006631	fatty acid metabolic process	BP	-0.647431205	-0.951712197	-0.350535749	4.647846858	12.11603837	0.00699605	0.026348164	PREDICTED: acyl-CoA synthetase family member 2, mitochondrial-like
RS007097	GO:0006631	fatty acid metabolic process	BP	0.340367486	-0.138491072	-0.70896225	4.143803222	11.99504412	0.007400157	0.027589571	PREDICTED: methylcrotonoyl-CoA carboxylase subunit alpha, mitochondrial
RS009060	GO:0006631	fatty acid metabolic process	BP	-0.445440747	0.052931611	1.34094544	4.027025825	11.53172946	0.009172158	0.033098611	PREDICTED: elongation of very long chain fatty acids protein 4-like
RS006548	GO:0006631	fatty acid metabolic process	BP	0.059898012	0.416718771	0.839565831	7.671508238	11.47459596	0.009417822	0.033753001	PREDICTED: acyl carrier protein, mitochondrial
RS013389	GO:0006631	fatty acid metabolic process	BP	-0.224647569	-0.44483051	0.622610016	5.896195007	10.82662015	0.012701318	0.043633497	PREDICTED: propionyl-CoA carboxylase alpha chain, mitochondrial
RS004124	GO:0006631	fatty acid metabolic process	BP	-0.61907943	-0.690409053	-0.007043237	5.012696694	9.991139429	0.018641604	0.060074811	PREDICTED: probable trans-2-enoyl-CoA reductase, mitochondrial
RS010105	GO:0006631	fatty acid metabolic process	BP	-0.567206255	-1.412112129	-0.886187691	5.676367558	9.069724408	0.028378083	0.084254422	PREDICTED: elongation of very long chain fatty acids protein 6
RS013655	GO:0006631	fatty acid metabolic process	BP	-0.201023512	0.012381247	-1.007472533	9.301050197	8.410677765	0.038244617	0.106938877	PREDICTED: transforming growth factor-beta-induced protein ig-h3-like
RS007468	GO:0006631	fatty acid metabolic process	BP	0.457030598	-0.368844241	-0.214826608	7.85084108	8.327687594	0.039703285	0.110210089	PREDICTED: retinal dehydrogenase 1
RS013199	GO:0006631	fatty acid metabolic process	BP	-0.830868079	-0.654963754	-0.904770904	4.065881842	7.990715751	0.046203973	0.124522328	PREDICTED: fatty acid hydroxylase domain-containing protein 2
RS002101	GO:0006631	fatty acid metabolic process	BP	-0.144951689	-0.554820667	-0.459503979	7.127596683	7.614008776	0.054700317	0.14227505	PREDICTED: long-chain-fatty-acid--CoA ligase 4
RS000570	GO:0006631	fatty acid metabolic process	BP	0.518702229	0.116476368	-0.226939285	5.273182682	7.320315563	0.062359576	0.157266002	PREDICTED: peroxisomal membrane protein PEX16
RS002642	GO:0006631	fatty acid metabolic process	BP	0.38648925	0.622840374	0.048143591	4.971006951	6.995050777	0.072055688	0.175356394	PREDICTED: acetyl-CoA acetyltransferase, cytosolic
RS007471	GO:0006631	fatty acid metabolic process	BP	-0.166455797	0.50637605	-0.01116969	8.121929428	6.835928055	0.077315296	0.184975082	PREDICTED: lambda-crystallin homolog
RS012825	GO:0006631	fatty acid metabolic process	BP	0.519876278	0.380407444	0.607764994	5.674018807	6.701163938	0.082057901	0.193238072	PREDICTED: lipoyl synthase, mitochondrial
RS011039	GO:0006631	fatty acid metabolic process	BP	-0.244819279	-0.634405128	-0.5346659	4.962293715	6.690040947	0.082461635	0.193971205	PREDICTED: enoyl-CoA delta isomerase 2, mitochondrial
RS003393	GO:0006631	fatty acid metabolic process	BP	0.415730181	0.169253589	-0.163673807	6.750170107	6.428868474	0.092510364	0.212416519	PREDICTED: leukotriene A-4 hydrolase
RS000296	GO:0006631	fatty acid metabolic process	BP	-0.154575641	-0.731686684	-0.647867681	5.62740596	6.283265117	0.09861327	0.222250451	PREDICTED: peroxisomal acyl-coenzyme A oxidase 3
RS007309	GO:0006631	fatty acid metabolic process	BP	-0.215890885	-0.384966284	0.175524897	6.831394536	6.173301657	0.103476489	0.230244329	PREDICTED: acyl-CoA synthetase family member 3, mitochondrial
RS006877	GO:0006631	fatty acid metabolic process	BP	-1.565839401	-1.137352864	-1.254175337	0.6979186	5.839978249	0.119660554	0.255813437	PREDICTED: acyl-CoA Delta(11) desaturase
RS009467	GO:0006631	fatty acid metabolic process	BP	0.105282131	0.575829771	0.630071704	6.50153031	5.708395503	0.12669216	0.267269998	PREDICTED: gamma-glutamyltransferase 7
RS000237	GO:0006631	fatty acid metabolic process	BP	-0.122122545	0.34733073	-0.422707365	3.88004886	5.545752767	0.135927562	0.28068072	PREDICTED: gamma-glutamyltranspeptidase 1-like
RS001719	GO:0006631	fatty acid metabolic process	BP	0.056240993	0.353605259	0.507377244	7.900530978	5.074050324	0.16645443	0.325769556	PREDICTED: trifunctional enzyme subunit beta, mitochondrial
RS002678	GO:0006631	fatty acid metabolic process	BP	-0.444950422	-1.104137516	-0.306756919	3.80292744	4.750004591	0.191045384	0.358244364	PREDICTED: fatty acid synthase-like
RS009359	GO:0006631	fatty acid metabolic process	BP	0.28026819	-0.408253169	0.031587479	4.49329609	4.682466065	0.196580833	0.365311231	PREDICTED: probable malonyl-CoA-acyl carrier protein transacylase, mitochondrial
RS001183	GO:0006631	fatty acid metabolic process	BP	0.381173523	0.452466843	0.551870902	6.511626778	4.513584138	0.211081809	0.382773838	PREDICTED: golgi-specific brefeldin A-resistance guanine nucleotide exchange factor 1
RS010926	GO:0006631	fatty acid metabolic process	BP	0.254824514	0.395156365	0.422047001	6.39620994	3.972494832	0.264449549	0.445815046	PREDICTED: short/branched chain specific acyl-CoA dehydrogenase, mitochondrial
RS002455	GO:0006631	fatty acid metabolic process	BP	-0.194242358	0.195237976	0.397980266	8.707600168	3.965482633	0.265215586	0.446421423	PREDICTED: very long-chain specific acyl-CoA dehydrogenase, mitochondrial
RS000240	GO:0006631	fatty acid metabolic process	BP	-0.600573278	-0.607829638	-0.158787656	4.031758924	3.682980404	0.297794016	0.480716988	PREDICTED: phytanoyl-CoA dioxygenase, peroxisomal
RS002269	GO:0006631	fatty acid metabolic process	BP	-0.261710878	-0.14856721	0.173629907	6.885308754	3.554684137	0.313738721	0.496137057	PREDICTED: succinate-semialdehyde dehydrogenase, mitochondrial
RS014514	GO:0006631	fatty acid metabolic process	BP	0.458849962	0.461206587	0.309540175	8.341540804	3.513253966	0.31904719	0.50157038	PREDICTED: trifunctional enzyme subunit alpha, mitochondrial
RS015110	GO:0006631	fatty acid metabolic process	BP	-0.075114035	0.056180732	0.466052101	5.236733039	3.378954547	0.33680395	0.518546327	PREDICTED: dehydrogenase/reductase SDR family member 4
RS010621	GO:0006631	fatty acid metabolic process	BP	0.319373764	0.387129475	0.372445429	7.228363378	3.353578394	0.340254876	0.521596292	PREDICTED: probable medium-chain specific acyl-CoA dehydrogenase, mitochondrial
RS015449	GO:0006631	fatty acid metabolic process	BP	0.241533289	-0.182689412	0.023986423	5.25590239	3.209399612	0.360452886	0.541776304	PREDICTED: peroxisome biogenesis factor 10
RS012396	GO:0006631	fatty acid metabolic process	BP	0.327124195	0.012418108	0.069947446	5.465353023	2.691227933	0.441720074	0.615449672	PREDICTED: peroxisome biogenesis factor 13
RS012653	GO:0006631	fatty acid metabolic process	BP	0.056734656	-0.372769409	-0.163659265	4.115912994	2.527360648	0.470366688	0.641826581	PREDICTED: 3-oxoacyl-[acyl-carrier-protein] synthase, mitochondrial
RS013452	GO:0006631	fatty acid metabolic process	BP	0.469213761	0.184190107	-0.021150747	3.385565932	2.381445454	0.497097891	0.663327623	PREDICTED: putative lipoyltransferase 2, mitochondrial
RS011651	GO:0006631	fatty acid metabolic process	BP	-0.351250373	-0.35252654	-0.287462621	4.967773394	2.18354716	0.535196334	0.693184484	PREDICTED: fatty acid synthase-like
RS007897	GO:0006631	fatty acid metabolic process	BP	0.062440003	-0.169160121	-0.302568052	5.867401431	2.164590839	0.538956504	0.696023196	PREDICTED: enoyl-CoA delta isomerase 1, mitochondrial-like
RS001108	GO:0006631	fatty acid metabolic process	BP	-0.351307875	-0.364078375	-0.192697335	4.467218055	2.150105206	0.54184283	0.698032794	PREDICTED: short-chain specific acyl-CoA dehydrogenase, mitochondrial
RS011393	GO:0006631	fatty acid metabolic process	BP	-0.041730957	-0.230300536	0.07476639	6.804211163	2.01329809	0.569651229	0.720350873	PREDICTED: hydroxyacyl-coenzyme A dehydrogenase, mitochondrial-like
RS006279	GO:0006631	fatty acid metabolic process	BP	0.731956001	0.312619525	-0.157127015	1.511408191	1.970524751	0.578546886	0.727453039	PREDICTED: fatty acid synthase-like
RS003278	GO:0006631	fatty acid metabolic process	BP	-0.229798233	-0.363111867	-0.210643548	4.730975205	1.917947367	0.589610433	0.735931991	PREDICTED: malonyl-CoA decarboxylase, mitochondrial-like
RS009535	GO:0006631	fatty acid metabolic process	BP	-0.371380403	-0.243526213	-0.109065039	6.875240596	1.879978368	0.597687259	0.742847463	PREDICTED: delta-1-pyrroline-5-carboxylate dehydrogenase, mitochondrial
RS007596	GO:0006631	fatty acid metabolic process	BP	-0.43536767	-0.490752696	-0.330090188	3.448549998	1.761913314	0.623257885	0.762368939	PREDICTED: fatty acid synthase-like
RS014553	GO:0006631	fatty acid metabolic process	BP	-0.735733025	0.024315289	-0.582972029	0.379469694	1.581739377	0.663537345	0.791044048	PREDICTED: fatty acid synthase-like
RS013397	GO:0006631	fatty acid metabolic process	BP	-0.201757507	-0.230383602	-0.008206272	6.648735594	1.554620765	0.669722452	0.79473142	PREDICTED: delta(3,5)-Delta(2,4)-dienoyl-CoA isomerase, mitochondrial
RS014702	GO:0006631	fatty acid metabolic process	BP	0.048428453	-0.238349601	-0.12992294	6.529352259	1.523281632	0.67690746	0.800220491	PREDICTED: glutaryl-CoA dehydrogenase, mitochondrial
RS003105	GO:0006631	fatty acid metabolic process	BP	0.209748722	0.196596402	0.069569969	4.899802605	0.955619911	0.811988837	0.892181767	PREDICTED: alkylglycerol monooxygenase-like
RS002932	GO:0006631	fatty acid metabolic process	BP	0.055970556	-0.003033501	0.220016606	7.070781008	0.921709314	0.820185942	0.897430213	PREDICTED: aldehyde dehydrogenase, mitochondrial
RS015527	GO:0006631	fatty acid metabolic process	BP	0.095932391	-0.126719206	0.030481933	5.507190068	0.897198446	0.826103835	0.901321295	PREDICTED: acyl-CoA dehydrogenase family member 9, mitochondrial
RS007595	GO:0006631	fatty acid metabolic process	BP	-0.043630471	-0.222354057	0.080312883	5.057929375	0.63376465	0.888661066	0.940408157	PREDICTED: fatty acid synthase-like
RS007594	GO:0006631	fatty acid metabolic process	BP	-0.043954148	-0.103763802	0.083984987	7.697619471	0.311465254	0.957857949	0.977604853	PREDICTED: fatty acid synthase
RS011720	GO:0048037	cofactor binding	MF	-2.78993926	6.666162583	5.530650553	4.728455414	504.2844383	5.6255E-109	1.3892E-106	PREDICTED: glucose dehydrogenase [FAD, quinone]
RS011718	GO:0048037	cofactor binding	MF	-0.059496373	8.716087878	3.96160781	4.311804415	501.7385592	2.004E-108	4.762E-106	PREDICTED: glucose dehydrogenase [FAD, quinone]
RS011049	GO:0048037	cofactor binding	MF	-2.29908087	3.299439657	8.25278355	7.490502686	408.7578628	2.80601E-88	5.12158E-86	PREDICTED: cysteine sulfinic acid decarboxylase
RS006639	GO:0048037	cofactor binding	MF	-3.083868425	-7.637695989	-8.133777043	4.628300865	186.0851837	4.27786E-40	2.64095E-38	PREDICTED: aromatic-L-amino-acid decarboxylase-like
RS011712	GO:0048037	cofactor binding	MF	-1.230020342	4.072791347	0.764053697	2.678048388	161.0891676	1.06678E-34	5.35262E-33	PREDICTED: glucose dehydrogenase [FAD, quinone]
RS011719	GO:0048037	cofactor binding	MF	-1.396212466	3.56799454	1.196979804	5.060980959	110.7270473	7.65358E-24	2.47152E-22	PREDICTED: glucose dehydrogenase [FAD, quinone]
RS004822	GO:0048037	cofactor binding	MF	-1.15913694	1.950800853	0.602046639	4.964662266	91.76587262	9.14602E-20	2.3037E-18	PREDICTED: mitochondrial amidoxime-reducing component 1-like
RS003553	GO:0048037	cofactor binding	MF	-1.896615492	-3.604139345	-1.858803184	8.22403702	88.21801298	5.28765E-19	1.24472E-17	PREDICTED: xanthine dehydrogenase/oxidase-like
RS011716	GO:0048037	cofactor binding	MF	-6.01820819	3.489449018	2.81410635	3.308115746	83.10682532	6.61417E-18	1.42635E-16	PREDICTED: glucose dehydrogenase [FAD, quinone]-like
RS006415	GO:0048037	cofactor binding	MF	-3.059456516	-3.953414729	-1.888715959	4.231267415	80.90022593	1.96755E-17	4.11617E-16	PREDICTED: glucose dehydrogenase [FAD, quinone]-like
RS006640	GO:0048037	cofactor binding	MF	-1.791513421	5.598946144	0.991323579	0.785411688	73.73810476	6.75358E-16	1.25635E-14	PREDICTED: aromatic-L-amino-acid decarboxylase-like
RS006641	GO:0048037	cofactor binding	MF	0	7.485546798	0	0.803640671	72.21979448	1.42834E-15	2.58085E-14	PREDICTED: aromatic-L-amino-acid decarboxylase-like
RS013098	GO:0048037	cofactor binding	MF	-2.126898304	-3.4336079	-2.804919227	4.337624443	68.06603945	1.10738E-14	1.87704E-13	PREDICTED: xanthine dehydrogenase/oxidase-like
RS013033	GO:0048037	cofactor binding	MF	-2.44596251	-3.881013688	-4.124427842	5.607467213	67.36154464	1.56705E-14	2.61743E-13	PREDICTED: glucose dehydrogenase [FAD, quinone]
RS003016	GO:0048037	cofactor binding	MF	0.355128266	-1.63524154	-2.121597333	3.87230946	64.30058562	7.07874E-14	1.13134E-12	PREDICTED: glucose dehydrogenase [FAD, quinone]

RS010386	GO:0048037	cofactor binding	MF	1.385615233	2.177722429	1.08390832	5.303798446	64.21218649	7.3937E-14	1.18018E-12	PREDICTED: ketimine reductase mu-crystallin
RS014962	GO:0048037	cofactor binding	MF	-0.778249315	-3.477526571	-0.968757046	7.419767245	62.95916817	1.37025E-13	2.14106E-12	PREDICTED: delta(24)-sterol reductase
RS013848	GO:0048037	cofactor binding	MF	0.385280105	-0.451376677	-1.360242751	6.103207414	62.73512141	1.53003E-13	2.38481E-12	PREDICTED: protein CREG1
RS000919	GO:0048037	cofactor binding	MF	0.070629326	1.524717298	0.182975292	7.336844113	61.36491303	3.00324E-13	4.57904E-12	PREDICTED: 3-hydroxy-3-methylglutaryl-coenzyme A reductase
RS011713	GO:0048037	cofactor binding	MF	-2.342114325	-5.146606544	-5.648708234	2.431264584	60.21279143	5.294E-13	7.91837E-12	PREDICTED: glucose dehydrogenase [FAD, quinone]
RS011717	GO:0048037	cofactor binding	MF	-5.032597315	2.326479602	1.338171742	5.261254656	58.26088503	1.38264E-12	1.99461E-11	PREDICTED: glucose dehydrogenase [FAD, quinone]-like
RS010228	GO:0048037	cofactor binding	MF	-0.603654192	-3.938345019	-0.506568246	3.3634338	56.06539856	4.06808E-12	5.66115E-11	PREDICTED: xanthine dehydrogenase/oxidase-like
RS010272	GO:0048037	cofactor binding	MF	-3.250896666	-4.41990282	-0.187787053	3.271423774	55.38395874	5.68588E-12	7.81746E-11	PREDICTED: glucose dehydrogenase [FAD, quinone]-like
RS008170	GO:0048037	cofactor binding	MF	0.253513984	1.733052836	0.806515481	5.816940129	54.81424338	7.52215E-12	1.02972E-10	PREDICTED: glucose dehydrogenase [FAD, quinone]
RS009449	GO:0048037	cofactor binding	MF	0.217859906	0.894808756	-2.254717648	4.298914796	53.88906176	1.18488E-11	1.59257E-10	PREDICTED: very-long-chain 3-oxoacyl-CoA reductase
RS006397	GO:0048037	cofactor binding	MF	2.677889399	3.536925796	2.975240508	5.435489187	53.28452354	1.59436E-11	2.10696E-10	PREDICTED: glucose dehydrogenase [FAD, quinone]
RS007667	GO:0048037	cofactor binding	MF	-2.905496692	-3.771454356	-1.959403868	2.847931386	46.70215936	4.02143E-10	4.75549E-09	PREDICTED: xanthine dehydrogenase/oxidase-like
RS008573	GO:0048037	cofactor binding	MF	-2.260713301	-3.030774322	-1.815246754	3.622901311	46.35220856	4.77314E-10	5.60233E-09	PREDICTED: probable aspartate aminotransferase, cytoplasmic
RS011857	GO:0048037	cofactor binding	MF	-2.520329028	-3.351704626	0.098895726	4.019687658	45.14359349	8.62483E-10	9.90887E-09	PREDICTED: 2-amino-3-ketobutyrate coenzyme A ligase, mitochondrial
RS011734	GO:0048037	cofactor binding	MF	-2.971337729	0.243659454	-0.177326697	3.7415488	42.72791998	2.81109E-09	3.08119E-08	PREDICTED: glucose dehydrogenase [FAD, quinone]-like
RS011394	GO:0048037	cofactor binding	MF	-1.130512888	-4.311859926	-0.867929923	2.581686454	42.35833137	3.36765E-09	3.65938E-08	PREDICTED: hydroxyacyl-coenzyme A dehydrogenase, mitochondrial-like
RS015505	GO:0048037	cofactor binding	MF	-0.873277592	-1.775991461	-0.249713989	6.67190604	41.13584257	6.11945E-09	6.48178E-08	PREDICTED: probable methylmalonate-semialdehyde dehydrogenase [acylating], mitochondrial
RS003552	GO:0048037	cofactor binding	MF	-2.156474624	-2.867477226	-2.328741761	3.097576061	38.13053428	2.65221E-08	2.59131E-07	PREDICTED: xanthine dehydrogenase/oxidase-like
RS013610	GO:0048037	cofactor binding	MF	-1.884472907	-1.020334627	-0.783788058	5.426462338	37.74288835	3.20387E-08	3.1062E-07	PREDICTED: glucose dehydrogenase [FAD, quinone]-like
RS010472	GO:0048037	cofactor binding	MF	-0.707846719	-1.359223275	-1.145612806	6.054168603	37.56822123	3.48855E-08	3.377E-07	PREDICTED: isovaleryl-CoA dehydrogenase, mitochondrial
RS003648	GO:0048037	cofactor binding	MF	0.799121324	0.883024374	-0.745381312	6.113323662	35.82944046	8.13666E-08	7.62449E-07	PREDICTED: protein singed
RS010161	GO:0048037	cofactor binding	MF	-2.438095508	-2.352258281	-0.643986362	7.162442842	33.12541031	3.03034E-07	2.67631E-06	PREDICTED: glucose dehydrogenase [FAD, quinone]
RS013606	GO:0048037	cofactor binding	MF	2.703077388	2.005591113	-0.078311465	4.987158317	32.7122742	3.70365E-07	3.24592E-06	PREDICTED: glucose dehydrogenase [FAD, quinone]-like
RS001674	GO:0048037	cofactor binding	MF	-0.725177387	-0.370012813	0.932400504	6.657705976	30.72741593	9.70131E-07	8.11276E-06	PREDICTED: cystathionine beta-synthase
RS006642	GO:0048037	cofactor binding	MF	-2.419823612	0.944025566	1.645528914	4.053624516	29.85374943	1.48135E-06	1.21538E-05	PREDICTED: aromatic-L-amino-acid decarboxylase
RS010957	GO:0048037	cofactor binding	MF	0.104578349	-0.713610498	-0.868724544	6.502009154	28.08418135	3.48723E-06	2.69933E-05	PREDICTED: ornithine aminotransferase, mitochondrial
RS007102	GO:0048037	cofactor binding	MF	-0.78190331	-0.653917209	0.250142998	5.561485709	27.98775305	3.6536E-06	2.81945E-05	PREDICTED: glycerol-3-phosphate dehydrogenase [NAD(+)], cytoplasmic
RS007624	GO:0048037	cofactor binding	MF	-1.33998228	-3.692172416	0.752205035	1.953523445	27.98010021	3.66714E-06	2.82816E-05	PREDICTED: tyrosine aminotransferase
RS011800	GO:0048037	cofactor binding	MF	-1.018570964	-2.603412306	-0.804726968	3.250067261	27.61409107	4.37655E-06	3.33848E-05	PREDICTED: serine dehydratase-like
RS000627	GO:0048037	cofactor binding	MF	-1.23214239	-1.256196129	-0.507713368	5.68807099	26.33446348	8.11684E-06	5.93977E-05	PREDICTED: 5-aminolevulinate synthase, erythroid-specific, mitochondrial
RS010160	GO:0048037	cofactor binding	MF	-2.894349966	-2.510383916	-0.914696982	6.182434793	24.85038105	1.65936E-05	0.000115714	PREDICTED: glucose dehydrogenase [FAD, quinone]
RS000419	GO:0048037	cofactor binding	MF	0.040399421	-0.493996515	-1.164051968	4.981441137	24.64016581	1.83602E-05	0.000127118	PREDICTED: peroxisomal acyl-coenzyme A oxidase 3
RS003551	GO:0048037	cofactor binding	MF	-1.777164237	-2.890195639	-1.40533891	2.518693384	24.2447302	2.2207E-05	0.000151915	PREDICTED: xanthine dehydrogenase/oxidase-like
RS007610	GO:0048037	cofactor binding	MF	-0.937977299	-0.674413913	0.09856611	5.486082389	23.09307983	3.86186E-05	0.000253578	PREDICTED: short-chain specific acyl-CoA dehydrogenase, mitochondrial
RS007239	GO:0048037	cofactor binding	MF	-0.751585402	-1.063608644	-2.049559478	8.039978062	22.36996591	5.46324E-05	0.000349259	PREDICTED: D-3-phosphoglycerate dehydrogenase
RS003580	GO:0048037	cofactor binding	MF	-0.349903781	0.97880878	-0.297171717	4.29862202	21.28103667	9.20343E-05	0.000564029	PREDICTED: uncharacterized aarF domain-containing protein kinase 1
RS006389	GO:0048037	cofactor binding	MF	0.239929193	0.823284331	1.063454236	6.934960336	21.26037652	9.29486E-05	0.000569078	PREDICTED: epimerase family protein SDR39U1
RS009408	GO:0048037	cofactor binding	MF	1.126351349	0.929006696	0.370540152	7.960119173	20.64642983	0.000124661	0.000745759	PREDICTED: 26S protease regulatory subunit 8
RS006448	GO:0048037	cofactor binding	MF	-0.438550285	-0.842818516	-1.111661943	7.029395929	20.1090662	0.000161133	0.000940802	PREDICTED: glycogen phosphorylase
RS014437	GO:0048037	cofactor binding	MF	-0.988357721	-0.015003324	-0.20706366	6.1139878	20.07392409	0.000163859	0.000955386	PREDICTED: alanine--glyoxylate aminotransferase 2-like
RS004020	GO:0048037	cofactor binding	MF	0.760635188	0.220093961	-0.261771613	5.591518584	19.6288157	0.00020262	0.001156755	PREDICTED: nicalin
RS012059	GO:0048037	cofactor binding	MF	-0.487537417	-1.155775031	-1.008744238	4.503721781	19.20044033	0.000248509	0.001392227	PREDICTED: serine hydroxymethyltransferase, cytosolic
RS011715	GO:0048037	cofactor binding	MF	-1.02776739	-1.798297028	-1.766096501	7.764040057	18.73944327	0.000309495	0.001695424	PREDICTED: glucose dehydrogenase [FAD, quinone]-like
RS002509	GO:0048037	cofactor binding	MF	0.07347544	-0.720252922	-0.547625565	6.183077943	18.51719795	0.000344005	0.001866607	PREDICTED: poly [ADP-ribose] polymerase
RS003219	GO:0048037	cofactor binding	MF	0.030734368	-1.119228443	-0.51621698	3.887749858	18.36336163	0.000370108	0.001991939	PREDICTED: probable peroxisomal acyl-coenzyme A oxidase 1
RS010080	GO:0048037	cofactor binding	MF	-1.225591648	-1.424043287	-0.670731436	9.443830356	18.26507346	0.000387807	0.002070388	PREDICTED: 2-oxoglutarate dehydrogenase, mitochondrial
RS005416	GO:0048037	cofactor binding	MF	-0.412284439	-1.050899627	-2.077424575	2.957561508	17.37798325	0.000590855	0.003035999	PREDICTED: threo-3-hydroxyaspartate ammonia-lyase
RS011383	GO:0048037	cofactor binding	MF	-0.592908391	-0.975532691	-0.419225628	5.386382038	17.30604756	0.000611351	0.003123469	PREDICTED: 2-hydroxyacyl-CoA lyase 1
RS010509	GO:0048037	cofactor binding	MF	-1.527063033	-1.135110705	-1.09559939	2.940762741	17.22393729	0.00063561	0.003239526	PREDICTED: cysteine sulfinic acid decarboxylase
RS001900	GO:0048037	cofactor binding	MF	0.920032231	0.681311079	0.164965465	7.432319705	16.37741042	0.000948819	0.004665093	PREDICTED: ATP-dependent RNA helicase p62-like
RS014330	GO:0048037	cofactor binding	MF	-0.904307343	-1.006861252	-0.448014081	6.451669659	15.83661629	0.001224883	0.005834407	PREDICTED: NADPH--cytochrome P450 reductase
RS001401	GO:0048037	cofactor binding	MF	-0.75955343	-0.918377897	-0.01602954	4.379386426	15.3535465	0.001538139	0.007115841	PREDICTED: isocitrate dehydrogenase [NADP] cytoplasmic-like
RS001628	GO:0048037	cofactor binding	MF	-0.066987655	0.88088736	0.583161669	4.399335423	14.93361565	0.001874267	0.008442244	PREDICTED: apoptosis-inducing factor 3
RS009254	GO:0048037	cofactor binding	MF	0.276763099	0.307712871	0.901515863	7.284745885	14.831862	0.00196612	0.008811857	PREDICTED: histidine triad nucleotide-binding protein 1
RS008575	GO:0048037	cofactor binding	MF	-0.895887835	-1.094241053	-0.498680763	6.153628076	14.59558766	0.002196986	0.009674422	PREDICTED: probable aspartate aminotransferase, cytoplasmic
RS008574	GO:0048037	cofactor binding	MF	-1.398204707	-1.439633479	-0.772632276	3.648197129	13.9848864	0.002925797	0.012394044	PREDICTED: probable aspartate aminotransferase, cytoplasmic
RS003220	GO:0048037	cofactor binding	MF	-0.919595261	-1.193174547	-0.639458968	5.160771178	13.94596359	0.002979636	0.012596688	PREDICTED: probable peroxisomal acyl-coenzyme A oxidase 1
RS010970	GO:0048037	cofactor binding	MF	-0.654166537	0.492469445	-0.220477149	4.881203789	13.84299924	0.003126837	0.013126461	PREDICTED: alanine aminotransferase 1
RS011402	GO:0048037	cofactor binding	MF	-0.518766755	-1.355522075	-0.785081892	3.459941294	13.73057373	0.0032958	0.013776073	PREDICTED: glyoxylate reductase/hydroxypyruvate reductase-like
RS000848	GO:0048037	cofactor binding	MF	-0.491509622	-0.039197542	0.539051459	7.220480495	13.7028662	0.003338812	0.013932736	PREDICTED: NADH dehydrogenase [ubiquinone] 1 alpha subcomplex subunit 9, mitochondrial
RS007147	GO:0048037	cofactor binding	MF	-0.183438953	-1.074134638	-0.371314656	3.810955234	13.11351454	0.004397445	0.017702537	PREDICTED: hydroxyacid oxidase 1
RS002313	GO:0048037	cofactor binding	MF	-0.451678341	0.180904234	-0.854553222	5.296298932	13.08468271	0.004457007	0.017899092	PREDICTED: glucose-6-phosphate 1-dehydrogenase
RS011032	GO:0048037	cofactor binding	MF	-0.526292146	-0.829736458	-0.646061965	4.781042562	12.38343437	0.006178715	0.023623174	PREDICTED: D-aspartate oxidase
RS010694	GO:0048037	cofactor binding	MF	-1.376784469	-1.151365045	-1.019735694	3.615734954	11.25799445	0.010409554	0.036761977	PREDICTED: glutamate decarboxylase
RS006300	GO:0048037	cofactor binding	MF	-0.511538755	-0.90580939	-0.346492661	4.099933328	10.62089422	0.013962833	0.04732183	PREDICTED: pyridoxine-5'-phosphate oxidase
RS015148	GO:0048037	cofactor binding	MF	-1.963276978	0.635742593	-0.155791267	2.122363384	10.45700384	0.01505542	0.050360678	PREDICTED: L-threonine 3-dehydrogenase, mitochondrial
RS012239	GO:0048037	cofactor binding	MF	0.482459701	0.838142166	1.02225669	9.155423628	10.25350664	0.01652971	0.054453354	PREDICTED: succinyl-CoA ligase [ADP/GDP-forming] subunit alpha, mitochondrial
RS003251	GO:0048037	cofactor binding	MF	-0.926007043	-0.972699255	-0.243788129	4.618803471	10.07731546	0.01792023	0.058121911	PREDICTED: alkyldihydroxyacetonephosphate synthase
RS002591	GO:0048037	cofactor binding	MF	-0.862816032	-0.632941949	0.029415913	8.699755638	9.964280052	0.018872221	0.060647296	PREDICTED: succinate dehydrogenase [ubiquinone] flavoprotein subunit, mitochondrial
RS005636	GO:0048037	cofactor binding	MF	-0.453071987	-0.038123733	0.527166131	7.855258218	9.838636626	0.019988779	0.063503274	PREDICTED: dihydrolipoyl dehydrogenase, mitochondrial
RS010692	GO:0048037	cofactor binding	MF	-0.309120066	0.465179547	0.256485608	4.965103348	9.720155556	0.021101013	0.066370169	PREDICTED: acyl-CoA-binding domain-containing protein 4
RS007419	GO:0048037	cofactor binding	MF	-0.810509574	-0.496169472	0.271583652	8.964283994	9.715009245	0.021150677	0.066476571	PREDICTED: probable isocitrate dehydrogenase [NAD] subunit alpha, mitochondrial
RS002956	GO:0048037	cofactor binding	MF	-2.928056308	-0.897843684	-0.292887679	1.412347179	9.602623126	0.022264315	0.069370802	PREDICTED: glucose dehydrogenase [FAD, quinone]-like
RS000146	GO:0048037	cofactor binding	MF	0.201922556	-0.781716042	-0.183638845	5.004160987	9.356926226	0.024903162	0.075921187	PREDICTED: serine hydroxymethyltransferase, mitochondrial
RS007160	GO:0048037	cofactor binding	MF	-0.549555066	-1.005296558	-0.449020655	6.369743433	9.315822987	0.025373612	0.077090385	cytoplasmic NADP+-dependent isocitrate dehydrogenase
RS014200	GO:0048037	cofactor binding	MF	0.368512158	-0.23900377	-0.315539871	4.740713374	8.620559497	0.034785227	0.09929734	PREDICTED: sphingosine-1-phosphate lyase
RS006776	GO:0048037	cofactor binding	MF	0.71638764	0.579864743	0.458766163	5.992561337	8.584837864	0.035351611	0.10061428	PREDICTED: NADPH-dependent diflavin oxidoreductase 1
RS015504	GO:0048037	cofactor binding	MF	-0.346792054	-0.919939272	-0.688384249	5.423422413	7.961040774	0.046823775	0.125842642	NAD(P) transhydrogenase, mitochondrial
RS008221	GO:0048037	cofactor binding	MF	0.494584107	0.577949482	0.596443217	6.228276146	7.64532803	0.053939612	0.140674151	PREDICTED: S-adenosyl-L-methionine-dependent tRNA 4-demethylwyosine synthase
RS004672	GO:0048037	cofactor binding	MF	0.353656262	0.61047509	0.327590838	7.102946281	7.619168949	0.05457427	0.142005858	PREDICTED: coatomer subunit epsilon
RS004318	GO:0048037	cofactor binding	MF	0.491549724	0.757087987	0.844223327	5.176959997	7.614963704	0.05467697	0.142243701	PREDICTED: glyoxylate/hydroxypyruvate reductase A-like
RS003700	GO:0048037	cofactor binding	MF	0.445500208	0.547805665	0.90099913	5.029149185	7.491054113	0.057788756	0.148619889	PREDICTED: UDP-glucose 6-dehydrogenase
RS015346	GO:0048037	cofactor binding	MF	-0.035897868	0.497726136	0.291117237	6.601582051	7.299752842	0.062933161	0.158389335	PREDICTED: cystathionine gamma-lyase
RS003797	GO:0048037	cofactor binding	MF	-0.779191865	-0.909904321	-0.8699466	6.927393112	7.246172474	0.064451878	0.1612129	PREDICTED: 4-aminobutyrate aminotransferase, mitochondrial
RS000758	GO:0048037	cofactor binding	MF	0.436016025	0.658391235	0.438858138	7.421745581	7.173549798	0.066567125	0.164996334	PREDICTED: kynurenine--oxoglutarate transaminase 3
RS007230	GO:0048037	cofactor binding	MF	-0.595941154	-0.25795038	0.362903371	9.093119676	7.171002242	0.066642534	0.165140653	PREDICTED: isocitrate dehydrogenase [NAD] subunit beta, mitochondrial
RS010959	GO:0048037	cofactor binding	MF	-0.01139964	0.540038099	0.153812167	4.863464778	6.854286188	0.076690074	0.183723568	PREDICTED: probable sulfite oxidase, mitochondrial
RS007471	GO:0048037	cofactor binding	MF	-0.166455797	0.50637605	-0.01116969	8.121929428	6.835928055	0.077315296	0.184975082	PREDICTED: lambda-crystallin homolog
RS008195	GO:0048037	cofactor binding	MF	0.533863066	0.426337346	0.621528576	6.350472863	6.759324797	0.079977665	0.189622234	PREDICTED: NAD-dependent protein deacylase sirtuin-5, mitochondrial
RS002576	GO:0048037	cofactor binding	MF	-0.620693831	-0.682180231	-0.612309734	6.887876847	6.631270757	0.084626811	0.198360331	PREDICTED: NADP-dependent malic enzyme
RS012259	GO:0048037	cofactor binding	MF	-0.47689811	-0.550626227	-0.829212685	8.367846941	6.487657393	0.090150539	0.2083745	PREDICTED: putative glutamate synthase [NADPH]
RS012144	GO:0048037	cofactor binding	MF	0.492953747	0.364658232	0.724065803	7.642199443	6.330771461	0.096580778	0.218844606	PREDICTED: acyl-CoA-binding domain-containing protein 6
RS000296	GO:0048037	cofactor binding	MF	-0.154575641	-0.731686684	-0.647867681	5.62740596	6.283265117	0.09861327	0.222250451	PREDICTED: peroxisomal acyl-coenzyme A oxidase 3
RS011722	GO:0048037	cofactor binding	MF	0.237984518	0.417339262	0.681790752	4.012277886	6.150741526	0.104502256	0.231807939	PREDICTED: NAD-dependent protein deacetylase Sirt4-like
RS006445	GO:0048037	cofactor binding	MF	-0.639463442	-0.446387769	0.056703064	3.503852672	5.903016491	0.11642521	0.251028779	PREDICTED: serine palmitoyltransferase 2
RS003690	GO:0048037	cofactor binding	MF	-0.232113403	-0.608329994	-0.182240398	4.819621893	5.891630852	0.117003316	0.251844089	PREDICTED: 2-aminoadipate transaminase-like
RS006951	GO:0048037	cofactor binding	MF	0.362871559	0.199149505	-0.106154987	6.457125486	5.692470091	0.127569407	0.268446696	PREDICTED: lysine-specific histone demethylase 1A
RS009629	GO:0048037	cofactor binding	MF	-0.691618563	-0.413021374	0.205371865	9.600427322	5.679143987	0.128307911	0.269372103	PREDICTED: isocitrate dehydrogenase [NAD] subunit gamma, mitochondrial
RS002083	GO:0048037	cofactor binding	MF	-0.323137174	-0.49516432	0.148595856	8.605719851	5.666822715	0.128994345	0.270039027	PREDICTED: aspartate aminotransferase, mitochondrial
RS007152	GO:0048037	cofactor binding	MF	-0.79033743	-0.938189121	-1.446925399	0.97652372	5.151356345	0.161043715	0.317897264	PREDICTED: neuroligin-4, Y-linked-like
RS013885	GO:0048037	cofactor binding	MF	-0.653620991	-1.203365067	-0.496719101	1.563650614	4.985052582	0.172894945	0.334270639	PREDICTED: tyrosine decarboxylase
RS009326	GO:0048037	cofactor binding	MF	0.162898799	-0.39930567	0.293003033	5.545630119	4.932236884	0.176826874	0.339837883	phosphoenolpyruvate decarboxylase
RS005738	GO:0048037	cofactor binding	MF	-0.378294814	-0.071697406	0.312780479	7.87711044	4.907000397	0.178735077	0.342512106	PREDICTED: putative succinate dehydrogenase [ubiquinone] cytochrome b small subunit, mitochondrial
RS013883	GO:0048037	cofactor binding	MF	-1.119764452	-0.852715943	0.034262468	6.463866364	4.838736295	0.183993753	0.349694714	PREDICTED: aromatic-L-amino-acid decarboxylase-like
RS010695	GO:0048037	cofactor binding	MF	0.518417779	0.062491693	-0.045494596	3.878001861	4.706009864	0.194634444	0.362661072	PREDICTED: dihydrofolate reductase
RS015204	GO:0048037	cofactor binding	MF	-0.40385631	-0.267684874	0.256413441	8.673377735	4.675269768	0.197179364	0.365763575	PREDICTED: NADH dehydrogenase [ubiquinone] flavoprotein 1, mitochondrial
RS005140	GO:0048037	cofactor binding	MF	-0.416469633	-0.263839851	-0.01550935	6.34712109	4.659101598	0.198530293	0.36774386	PREDICTED: D-2-hydroxyglutarate dehydrogenase, mitochondrial
RS000111	GO:0048037	cofactor binding	MF	-0.493551406	-0.558539536	0.006439579	9.004522239	4.55700476	0.207261605	0.378541045	PREDICTED: succinate dehydrogenase [ubiquinone] iron-sulfur subunit, mitochondrial
RS007359	GO:0048037	cofactor binding	MF	-0.033186439	-0.296037869	-0.421053457	5.816316114	4.527960563	0.209809787	0.381376047	PREDICTED: putative oxidoreductase GLYR1 homolog
RS007491	GO:0048037	cofactor binding	MF	-0.446508158	-0.228275875	-0.036390422	6.67592503	4.497785152	0.212487932	0.384471824	PREDICTED: NADP-dependent malic enzyme
RS007608	GO:0048037	cofactor binding	MF	-0.313919656	-0.221843514	-0.598786917	6.860573137	4.416671791	0.219844495	0.393527242	PREDICTED: ATP-citrate synthase

RS005051	GO:0048037	cofactor binding	MF	0.153997919	-0.221089516	0.305477893	5.583582093	4.392675483	0.222065502	0.395922591	PREDICTED: DNA repair and recombination protein RAD54-like
RS002014	GO:0048037	cofactor binding	MF	0.036539526	-0.332351867	0.222033102	8.334994989	3.995693418	0.261929539	0.442664335	PREDICTED: cystathionine gamma-lyase
RS000326	GO:0048037	cofactor binding	MF	-0.22680592	0.003848613	0.324328015	8.109325185	3.972399064	0.264459998	0.445815046	PREDICTED: NADH-ubiquinone oxidoreductase 20 kDa subunit-like
RS010926	GO:0048037	cofactor binding	MF	0.254824514	0.395156365	0.422047001	6.39620994	3.972494832	0.264449549	0.445815046	PREDICTED: short/branched chain specific acyl-CoA dehydrogenase, mitochondrial
RS002455	GO:0048037	cofactor binding	MF	-0.194242358	0.195237976	0.397980266	8.707600168	3.965482633	0.265215586	0.446421423	PREDICTED: very long-chain specific acyl-CoA dehydrogenase, mitochondrial
RS007857	GO:0048037	cofactor binding	MF	0.019057041	0.160899051	0.363191345	6.304461316	3.810263323	0.282694589	0.465502367	PREDICTED: xanthine dehydrogenase
RS008249	GO:0048037	cofactor binding	MF	-0.391953891	1.193441996	1.153073924	1.121001183	3.791832584	0.284837569	0.467988351	PREDICTED: glucose dehydrogenase [FAD, quinone]-like
RS014351	GO:0048037	cofactor binding	MF	0.156227108	0.422293573	0.344826693	6.658243688	3.776584112	0.286621565	0.469477085	PREDICTED: thioredoxin reductase 1, mitochondrial
RS007459	GO:0048037	cofactor binding	MF	-0.532660554	-0.028004958	-0.266996105	3.941775201	3.748025441	0.2899898	0.472829045	PREDICTED: NAD-dependent protein deacetylase sirtuin-1
RS000240	GO:0048037	cofactor binding	MF	-0.600573278	-0.607829638	-0.158787656	4.031758924	3.682980404	0.297794016	0.480716988	PREDICTED: phytanoyl-CoA dioxygenase, peroxisomal
RS005465	GO:0048037	cofactor binding	MF	-0.255589849	-0.533680261	-0.609552513	2.6851131	3.620655703	0.305447325	0.488173047	PREDICTED: lysine-specific histone demethylase 1A
RS005467	GO:0048037	cofactor binding	MF	0.676741581	0.380751093	0.512853019	2.637868892	3.538021229	0.315864272	0.498396739	PREDICTED: lysine-specific histone demethylase 1A
RS012147	GO:0048037	cofactor binding	MF	-0.558038474	-0.077034269	0.32175258	2.735746395	3.396752893	0.33440186	0.516363048	PREDICTED: NAD-dependent protein deacetylase sirtuin-7
RS010621	GO:0048037	cofactor binding	MF	0.319373764	0.387129475	0.372445429	7.228363378	3.353578394	0.340254876	0.521596292	PREDICTED: probable medium-chain specific acyl-CoA dehydrogenase, mitochondrial
RS002545	GO:0048037	cofactor binding	MF	-0.486437272	-0.387085875	0.043929873	9.369782347	3.274374469	0.351225196	0.531905979	PREDICTED: NADH-ubiquinone oxidoreductase 49 kDa subunit
RS000974	GO:0048037	cofactor binding	MF	0.063768524	0.231555619	0.399568336	7.698083467	3.143071865	0.370087987	0.551062675	PREDICTED: electron transfer flavoprotein subunit alpha, mitochondrial
RS010824	GO:0048037	cofactor binding	MF	0.346918779	0.141745704	-0.020641719	5.248339231	3.076469603	0.379984682	0.560022359	PREDICTED: NAD-dependent protein deacetylase Sirt6
RS013884	GO:0048037	cofactor binding	MF	-1.483581091	-0.28373006	-0.000921218	2.848064172	2.784924475	0.425987633	0.600838644	PREDICTED: aromatic-L-amino-acid decarboxylase-like
RS009084	GO:0048037	cofactor binding	MF	-0.462615302	-0.281297931	-0.024727583	3.250454039	2.773936031	0.427808502	0.602394932	dTDP-glucose 4,6-dehydratase
RS004819	GO:0048037	cofactor binding	MF	-0.296041288	-0.224467726	-0.522676724	4.879480613	2.747178566	0.432269238	0.606100955	PREDICTED: mitochondrial amidoxime-reducing component 1-like
RS012446	GO:0048037	cofactor binding	MF	-0.33520903	-0.326059321	0.041645679	5.694017303	2.740242498	0.433431766	0.607177439	PREDICTED: NAD-dependent protein deacetylase sirtuin-3, mitochondrial
RS000379	GO:0048037	cofactor binding	MF	0.238639889	0.002576451	-0.1801865	4.995979504	2.646519158	0.44939244	0.622623585	PREDICTED: protein MTO1 homolog, mitochondrial
RS006771	GO:0048037	cofactor binding	MF	0.475535507	0.066379896	0.054545274	5.13346181	2.640006509	0.450519017	0.623635579	PREDICTED: C-terminal-binding protein
RS007076	GO:0048037	cofactor binding	MF	-0.333532028	0.014299236	-0.014087004	5.372273562	2.600146824	0.457463807	0.629994444	PREDICTED: serine palmitoyltransferase 1
RS012274	GO:0048037	cofactor binding	MF	0.250868348	0.130831382	-0.091269758	5.86768545	2.371175161	0.499022911	0.66490632	PREDICTED: probable GDP-L-fucose synthase
RS011967	GO:0048037	cofactor binding	MF	-0.487444254	-0.165820874	-0.461490275	5.204715649	2.301911612	0.512154792	0.67540078	PREDICTED: flavin-containing monooxygenase FMO GS-OX-like 4
RS013061	GO:0048037	cofactor binding	MF	0.055667037	0.39151864	0.322142146	3.784294836	2.253027649	0.521579236	0.683037215	PREDICTED: ubiquinone biosynthesis monooxygenase COQ6
RS001108	GO:0048037	cofactor binding	MF	-0.351307875	-0.364078375	-0.192697335	4.467218055	2.150105206	0.54184283	0.698032794	PREDICTED: short-chain specific acyl-CoA dehydrogenase, mitochondrial
RS005332	GO:0048037	cofactor binding	MF	0.03211789	-0.20554948	-0.243739032	6.261746697	2.15056386	0.541751269	0.698032794	PREDICTED: proline dehydrogenase 1, mitochondrial
RS011007	GO:0048037	cofactor binding	MF	0.164182996	-0.125816708	-0.353987059	3.604506135	2.107128644	0.550471937	0.704536949	PREDICTED: E3 ubiquitin-protein ligase znrf1
RS000486	GO:0048037	cofactor binding	MF	0.232389203	-0.011565709	-0.040844754	6.74387093	2.012991222	0.569714711	0.720350873	PREDICTED: apoptosis-inducing factor 1, mitochondrial
RS011393	GO:0048037	cofactor binding	MF	-0.041730957	-0.230300536	0.07476639	6.804211163	2.01329809	0.569651229	0.720350873	PREDICTED: hydroxyacyl-coenzyme A dehydrogenase, mitochondrial-like
RS004746	GO:0048037	cofactor binding	MF	-0.053765857	-0.290830416	-0.105459059	6.353586052	1.929510254	0.587165226	0.733679815	PREDICTED: puff-specific protein Bx42
RS005517	GO:0048037	cofactor binding	MF	-0.040617769	0.277956167	0.454102457	9.66049094	1.870199645	0.599779119	0.744859307	PREDICTED: ATP-dependent RNA helicase p62-like
RS002439	GO:0048037	cofactor binding	MF	0.322813085	0.111104145	-0.161839796	6.005037147	1.846999235	0.604761168	0.748757585	PREDICTED: UDP-glucose 4-epimerase-like
RS003259	GO:0048037	cofactor binding	MF	0.345067689	0.214790695	0.161470789	5.364845303	1.826626715	0.609157877	0.751688644	PREDICTED: tRNA-dihydrouridine(20) synthase [NAD(P)+]-like
RS006120	GO:0048037	cofactor binding	MF	0.282310094	0.26654938	0.010128256	4.191105626	1.744965367	0.626983667	0.765138788	PREDICTED: tRNA-dihydrouridine(47) synthase [NAD(P)(+)]-like
RS006985	GO:0048037	cofactor binding	MF	0.006933679	0.269486901	-0.078587833	5.040871731	1.689258661	0.639324476	0.774122916	PREDICTED: probable 2-oxoglutarate dehydrogenase E1 component DHKTD1 homolog, mitochondrial
RS007101	GO:0048037	cofactor binding	MF	0.302041092	0.019148607	0.249776245	3.7895307	1.606596267	0.657895087	0.786999499	FAD-linked oxidoreductase
RS002730	GO:0048037	cofactor binding	MF	0.273731849	0.243679505	0.179335078	5.847607944	1.567473925	0.666787166	0.792742148	PREDICTED: tRNA-dihydrouridine(20a/20b) synthase [NAD(P)+]-like
RS014702	GO:0048037	cofactor binding	MF	0.048428453	-0.238349601	-0.12992294	6.529352259	1.523281632	0.67690746	0.800220491	PREDICTED: glutaryl-CoA dehydrogenase, mitochondrial
RS004039	GO:0048037	cofactor binding	MF	-0.217625409	-0.338436678	0.119231972	2.598033638	1.520372268	0.677576466	0.800421353	PREDICTED: molybdenum cofactor sulfurase 1
RS012079	GO:0048037	cofactor binding	MF	0.239219771	0.02432342	0.187017072	5.848565127	1.497472914	0.682853703	0.803950597	PREDICTED: pyridoxal-dependent decarboxylase domain-containing protein 1
RS003678	GO:0048037	cofactor binding	MF	-0.334465949	-0.114289915	-0.203522905	4.076361414	1.425503068	0.69956771	0.815865352	PREDICTED: tRNA-dihydrouridine(16/17) synthase [NAD(P)(+)]-like
RS014513	GO:0048037	cofactor binding	MF	0.245831721	0.239105658	0.17414925	5.204247819	1.344995027	0.718477293	0.831181996	PREDICTED: ras-related protein Rab-6.2
RS013491	GO:0048037	cofactor binding	MF	0.50959189	0.011563311	0.381662714	3.356752204	1.246647485	0.741839413	0.847577485	PREDICTED: amine oxidase [flavin-containing] B
RS013047	GO:0048037	cofactor binding	MF	0.231685496	0.106278735	0.095579367	5.899186066	1.241080553	0.743169287	0.848450098	PREDICTED: NAD-dependent protein deacetylase sirtuin-2
RS004876	GO:0048037	cofactor binding	MF	0.254514777	-0.013585511	0.016602412	4.233428366	1.08122198	0.781608997	0.873820125	PREDICTED: 3-hydroxyisobutyrate dehydrogenase, mitochondrial
RS002770	GO:0048037	cofactor binding	MF	-0.119360703	-0.359384597	-0.31109241	10.31331347	0.993680302	0.802781134	0.886207234	glyceraldehyde-3-phosphate dehydrogenase
RS014187	GO:0048037	cofactor binding	MF	0.158474751	0.095400176	0.272547424	5.44957084	0.920998054	0.820357765	0.897490049	PREDICTED: 1-aminocyclopropane-1-carboxylate synthase-like protein 1
RS015527	GO:0048037	cofactor binding	MF	0.095932391	-0.126719206	0.030481933	5.507190068	0.897198446	0.826103835	0.901321295	PREDICTED: acyl-CoA dehydrogenase family member 9, mitochondrial
RS008879	GO:0048037	cofactor binding	MF	0.109355227	0.022608977	-0.119419284	4.359906481	0.776449349	0.855091344	0.920507768	PREDICTED: proline synthase co-transcribed bacterial homolog protein
RS002905	GO:0048037	cofactor binding	MF	0.088090998	0.253655374	0.156881191	4.076313798	0.723831965	0.867584626	0.928559597	PREDICTED: elongation factor Tu, mitochondrial-like
RS008782	GO:0048037	cofactor binding	MF	0.087717049	0.093210778	0.206874064	7.105662688	0.684144487	0.876926837	0.93376185	PREDICTED: putative acyl-CoA-binding protein
RS013157	GO:0048037	cofactor binding	MF	0.115195703	0.398542992	-0.12346977	0.94470257	0.569142202	0.903458769	0.9486709	Rm62
RS007877	GO:0048037	cofactor binding	MF	-0.04006425	0.125386876	0.039173322	5.581612122	0.5433599	0.909267233	0.952321145	PREDICTED: D-aspartate oxidase
RS000629	GO:0048037	cofactor binding	MF	0.00837018	0.116143531	-0.015561228	6.338092866	0.458808573	0.927843179	0.962389796	PREDICTED: thioredoxin reductase 3
RS000370	GO:0048037	cofactor binding	MF	-0.324119489	-0.104677593	0.006917945	5.382292536	0.439925008	0.931876866	0.964422487	PREDICTED: nitric oxide synthase, salivary gland
RS013432	GO:0048037	cofactor binding	MF	0.186161353	0.083115952	0.073559077	4.349613188	0.400756431	0.94008619	0.96877878	PREDICTED: glucose dehydrogenase [FAD, quinone]-like
RS006226	GO:0048037	cofactor binding	MF	-0.000697599	-0.093453208	-0.154614222	4.887176637	0.380294869	0.944282348	0.970591928	PREDICTED: alanine--glyoxylate aminotransferase 2, mitochondrial
RS014512	GO:0048037	cofactor binding	MF	-0.029889403	-0.146982534	-0.072958369	7.559121721	0.32842394	0.954596993	0.97588441	PREDICTED: cysteine desulfurase, mitochondrial
RS005907	GO:0048037	cofactor binding	MF	0.095461109	0.014842875	0.085453782	6.336643908	0.318112256	0.956586828	0.976930029	PREDICTED: Golgi resident protein GCP60
RS011720	GO:0050662	coenzyme binding	MF	-2.78993926	6.666162583	5.530650553	4.728455414	504.2844383	5.6255E-109	1.3892E-106	PREDICTED: glucose dehydrogenase [FAD, quinone]
RS011718	GO:0050662	coenzyme binding	MF	-0.059496373	8.716087878	3.96160781	4.311804415	501.7385592	2.004E-108	4.762E-106	PREDICTED: glucose dehydrogenase [FAD, quinone]
RS011712	GO:0050662	coenzyme binding	MF	-1.230020342	4.072791347	0.764053697	2.678048388	161.0891676	1.06678E-34	5.35262E-33	PREDICTED: glucose dehydrogenase [FAD, quinone]
RS011719	GO:0050662	coenzyme binding	MF	-1.396212466	3.56799454	1.196979804	5.060980959	110.7270473	7.65358E-24	2.47152E-22	PREDICTED: glucose dehydrogenase [FAD, quinone]
RS003553	GO:0050662	coenzyme binding	MF	-1.896615492	-3.604139345	-1.858803184	8.22403702	88.21801298	5.28765E-19	1.24472E-17	PREDICTED: xanthine dehydrogenase/oxidase-like
RS011716	GO:0050662	coenzyme binding	MF	-6.01820819	3.489449018	2.81410635	3.308115746	83.10682532	6.61417E-18	1.42635E-16	PREDICTED: glucose dehydrogenase [FAD, quinone]-like
RS006415	GO:0050662	coenzyme binding	MF	-3.059456516	-3.953414729	-1.888715959	4.231267415	80.90022593	1.96755E-17	4.11617E-16	PREDICTED: glucose dehydrogenase [FAD, quinone]-like
RS013098	GO:0050662	coenzyme binding	MF	-2.126898304	-3.4336079	-2.804919227	4.337624443	68.06603945	1.10738E-14	1.87704E-13	PREDICTED: xanthine dehydrogenase/oxidase-like
RS013033	GO:0050662	coenzyme binding	MF	-2.44596251	-3.881013688	-4.124427842	5.607467213	67.36154464	1.56705E-14	2.61743E-13	PREDICTED: glucose dehydrogenase [FAD, quinone]
RS003016	GO:0050662	coenzyme binding	MF	0.355128266	-1.63524154	-2.121597333	3.87230946	64.30058562	7.07874E-14	1.13134E-12	PREDICTED: glucose dehydrogenase [FAD, quinone]
RS010386	GO:0050662	coenzyme binding	MF	1.385615233	2.177722429	1.08390832	5.303798446	64.21218649	7.3937E-14	1.18018E-12	PREDICTED: ketimine reductase mu-crystallin
RS014962	GO:0050662	coenzyme binding	MF	-0.778249315	-3.477526571	-0.968757046	7.419767245	62.95916817	1.37025E-13	2.14106E-12	PREDICTED: delta(24)-sterol reductase
RS013848	GO:0050662	coenzyme binding	MF	0.385280105	-0.451376677	-1.360242751	6.103207414	62.73512141	1.53003E-13	2.38481E-12	PREDICTED: protein CREG1
RS000919	GO:0050662	coenzyme binding	MF	0.070629326	1.524717298	0.182975292	7.336844113	61.36491303	3.00324E-13	4.57904E-12	PREDICTED: 3-hydroxy-3-methylglutaryl-coenzyme A reductase
RS011713	GO:0050662	coenzyme binding	MF	-2.342114325	-5.146606544	-5.648708234	2.431264584	60.21279143	5.294E-13	7.91837E-12	PREDICTED: glucose dehydrogenase [FAD, quinone]
RS011717	GO:0050662	coenzyme binding	MF	-5.032597315	2.326479602	1.338171742	5.261254656	58.26088503	1.38264E-12	1.99461E-11	PREDICTED: glucose dehydrogenase [FAD, quinone]-like
RS010228	GO:0050662	coenzyme binding	MF	-0.603654192	-3.938345019	-0.506568246	3.3634338	56.06539856	4.06808E-12	5.66115E-11	PREDICTED: xanthine dehydrogenase/oxidase-like
RS010272	GO:0050662	coenzyme binding	MF	-3.250896666	-4.41990282	-0.187787053	3.271423774	55.38395874	5.68588E-12	7.81746E-11	PREDICTED: glucose dehydrogenase [FAD, quinone]-like
RS008170	GO:0050662	coenzyme binding	MF	0.253513984	1.733052836	0.806515481	5.816940129	54.81424338	7.52215E-12	1.02972E-10	PREDICTED: glucose dehydrogenase [FAD, quinone]
RS009449	GO:0050662	coenzyme binding	MF	0.217859906	0.894808756	-2.254717648	4.298914796	53.88906176	1.18488E-11	1.59257E-10	PREDICTED: very-long-chain 3-oxoacyl-CoA reductase
RS006397	GO:0050662	coenzyme binding	MF	2.677889399	3.536925796	2.975240508	5.435489187	53.28452354	1.59436E-11	2.10696E-10	PREDICTED: glucose dehydrogenase [FAD, quinone]
RS007667	GO:0050662	coenzyme binding	MF	-2.905496692	-3.771454356	-1.959403868	2.847931386	46.70215936	4.02143E-10	4.75549E-09	PREDICTED: xanthine dehydrogenase/oxidase-like
RS011734	GO:0050662	coenzyme binding	MF	-2.971337729	0.243659454	-0.177326697	3.7415488	42.72791998	2.81109E-09	3.08119E-08	PREDICTED: glucose dehydrogenase [FAD, quinone]-like
RS011394	GO:0050662	coenzyme binding	MF	-1.130512888	-4.311859926	-0.867929923	2.581686454	42.35833137	3.36765E-09	3.65938E-08	PREDICTED: hydroxyacyl-coenzyme A dehydrogenase, mitochondrial-like
RS015505	GO:0050662	coenzyme binding	MF	-0.873277592	-1.775991461	-0.249713989	6.67190604	41.13584257	6.11945E-09	6.48178E-08	PREDICTED: probable methylmalonate-semialdehyde dehydrogenase [acylating], mitochondrial
RS003552	GO:0050662	coenzyme binding	MF	-2.156474624	-2.867477226	-2.328741761	3.097576061	38.13053428	2.65221E-08	2.59131E-07	PREDICTED: xanthine dehydrogenase/oxidase-like
RS013610	GO:0050662	coenzyme binding	MF	-1.884472907	-1.020334627	-0.783788058	5.426462338	37.74288835	3.20387E-08	3.1062E-07	PREDICTED: glucose dehydrogenase [FAD, quinone]-like
RS010472	GO:0050662	coenzyme binding	MF	-0.707846719	-1.359223275	-1.145612806	6.054168603	37.56822123	3.48855E-08	3.377E-07	PREDICTED: isovaleryl-CoA dehydrogenase, mitochondrial
RS003648	GO:0050662	coenzyme binding	MF	0.799121324	0.883024374	-0.745381312	6.113323662	35.82944046	8.13666E-08	7.62449E-07	PREDICTED: protein singed
RS010161	GO:0050662	coenzyme binding	MF	-2.438095508	-2.352258281	-0.643986362	7.162442842	33.12541031	3.03034E-07	2.67631E-06	PREDICTED: glucose dehydrogenase [FAD, quinone]
RS013606	GO:0050662	coenzyme binding	MF	2.703077388	2.005591113	-0.078311465	4.987158317	32.7122742	3.70365E-07	3.24592E-06	PREDICTED: glucose dehydrogenase [FAD, quinone]-like
RS007102	GO:0050662	coenzyme binding	MF	-0.78190331	-0.653917209	0.250142998	5.561485709	27.98775305	3.6536E-06	2.81945E-05	PREDICTED: glycerol-3-phosphate dehydrogenase [NAD(+)], cytoplasmic
RS010160	GO:0050662	coenzyme binding	MF	-2.894349966	-2.510383916	-0.914696982	6.182434793	24.85038105	1.65936E-05	0.000115714	PREDICTED: glucose dehydrogenase [FAD, quinone]
RS000419	GO:0050662	coenzyme binding	MF	0.040399421	-0.493996515	-1.164051968	4.981441137	24.64016581	1.83602E-05	0.000127118	PREDICTED: peroxisomal acyl-coenzyme A oxidase 3
RS003551	GO:0050662	coenzyme binding	MF	-1.777164237	-2.890195639	-1.40533891	2.518693384	24.2447302	2.2207E-05	0.000151915	PREDICTED: xanthine dehydrogenase/oxidase-like
RS007610	GO:0050662	coenzyme binding	MF	-0.937977299	-0.674413913	0.09856611	5.486082389	23.09307983	3.86186E-05	0.000253578	PREDICTED: short-chain specific acyl-CoA dehydrogenase, mitochondrial
RS007239	GO:0050662	coenzyme binding	MF	-0.751585402	-1.063608644	-2.049559478	8.039978062	22.36996591	5.46324E-05	0.000349259	PREDICTED: D-3-phosphoglycerate dehydrogenase
RS003580	GO:0050662	coenzyme binding	MF	-0.349903781	0.97880878	-0.297171717	4.29862202	21.28103667	9.20343E-05	0.000564029	PREDICTED: uncharacterized aarF domain-containing protein kinase 1
RS006389	GO:0050662	coenzyme binding	MF	0.239929193	0.823284331	1.063454236	6.934960336	21.26037652	9.29486E-05	0.000569078	PREDICTED: epimerase family protein SDR39U1
RS009408	GO:0050662	coenzyme binding	MF	1.126351349	0.929006696	0.370540152	7.960119173	20.64642983	0.000124661	0.000745759	PREDICTED: 26S protease regulatory subunit 8
RS011715	GO:0050662	coenzyme binding	MF	-1.02776739	-1.798297028	-1.766096501	7.764040057	18.73944327	0.000309495	0.001695424	PREDICTED: glucose dehydrogenase [FAD, quinone]-like
RS002509	GO:0050662	coenzyme binding	MF	0.07347544	-0.720252922	-0.547625565	6.183077943	18.51719795	0.000344005	0.001866607	PREDICTED: poly [ADP-ribose] polymerase
RS003219	GO:0050662	coenzyme binding	MF	0.030734368	-1.119228443	-0.51621698	3.887749858	18.36336163	0.000370108	0.001991939	PREDICTED: probable peroxisomal acyl-coenzyme A oxidase 1
RS010080	GO:0050662	coenzyme binding	MF	-1.225591648	-1.424043287	-0.670731436	9.443830356	18.26507346	0.000387807	0.002070388	PREDICTED: 2-oxoglutarate dehydrogenase, mitochondrial
RS011383	GO:0050662	coenzyme binding	MF	-0.592908391	-0.975532691	-0.419225628	5.386382038	17.30604756	0.000611351	0.003123469	PREDICTED: 2-hydroxyacyl-CoA lyase 1
RS001900	GO:0050662	coenzyme binding	MF	0.920032231	0.681311079	0.164965465	7.432319705	16.37741042	0.000948819	0.004665093	PREDICTED: ATP-dependent RNA helicase p62-like
RS014330	GO:0050662	coenzyme binding	MF	-0.904307343	-1.006861252	-0.448014081	6.451669659	15.83661629	0.001224883	0.005834407	PREDICTED: NADPH--cytochrome P450 reductase
RS001401	GO:0050662	coenzyme binding	MF	-0.75955343	-0.918377897	-0.01602954	4.379386426	15.3535465	0.001538139	0.007115841	PREDICTED: isocitrate dehydrogenase [NADP] cytoplasmic-like
RS001628	GO:0050662	coenzyme binding	MF	-0.066987655	0.88088736	0.583161669	4.399335423	14.93361565	0.001874267	0.008442244	PREDICTED: apoptosis-inducing factor 3

RS009254	GO:0050662	coenzyme binding	MF	0.276763099	0.307712871	0.901515863	7.284745885	14.831862	0.00196612	0.008811857	PREDICTED: histidine triad nucleotide-binding protein 1
RS003220	GO:0050662	coenzyme binding	MF	-0.919595261	-1.193174547	-0.639458968	5.160771178	13.94596359	0.002979636	0.012596688	PREDICTED: probable peroxisomal acyl-coenzyme A oxidase 1
RS011402	GO:0050662	coenzyme binding	MF	-0.518766755	-1.355522075	-0.785081892	3.459941294	13.73057373	0.0032958	0.013776073	PREDICTED: glyoxylate reductase/hydroxypyruvate reductase-like
RS000848	GO:0050662	coenzyme binding	MF	-0.491509622	-0.039197542	0.539051459	7.220480495	13.7028662	0.003338812	0.013932736	PREDICTED: NADH dehydrogenase [ubiquinone] 1 alpha subcomplex subunit 9, mitochondrial
RS007147	GO:0050662	coenzyme binding	MF	-0.183438953	-1.074134638	-0.371314656	3.810955234	13.11351454	0.004397445	0.017702537	PREDICTED: hydroxyacid oxidase 1
RS002313	GO:0050662	coenzyme binding	MF	-0.451678341	0.180904234	-0.854553222	5.296298932	13.08468271	0.004457007	0.017899092	PREDICTED: glucose-6-phosphate 1-dehydrogenase
RS011032	GO:0050662	coenzyme binding	MF	-0.526292146	-0.829736458	-0.646061965	4.781042562	12.38343437	0.006178715	0.023623174	PREDICTED: D-aspartate oxidase
RS006300	GO:0050662	coenzyme binding	MF	-0.511538755	-0.90580939	-0.346492661	4.099933328	10.62089422	0.013962833	0.04732183	PREDICTED: pyridoxine-5'-phosphate oxidase
RS015148	GO:0050662	coenzyme binding	MF	-1.963276978	0.635742593	-0.155791267	2.122363384	10.45700384	0.01505542	0.050360678	PREDICTED: L-threonine 3-dehydrogenase, mitochondrial
RS003251	GO:0050662	coenzyme binding	MF	-0.926007043	-0.972699255	-0.243788129	4.618803471	10.07731546	0.01792023	0.058121911	PREDICTED: alkyldihydroxyacetonephosphate synthase
RS002591	GO:0050662	coenzyme binding	MF	-0.862816032	-0.632941949	0.029415913	8.699755638	9.964280052	0.018872221	0.060647296	PREDICTED: succinate dehydrogenase [ubiquinone] flavoprotein subunit, mitochondrial
RS005636	GO:0050662	coenzyme binding	MF	-0.453071987	-0.038123733	0.527166131	7.855258218	9.838636626	0.019988779	0.063503274	PREDICTED: dihydrolipoyl dehydrogenase, mitochondrial
RS010692	GO:0050662	coenzyme binding	MF	-0.309120066	0.465179547	0.256485608	4.965103348	9.720155556	0.021101013	0.066370169	PREDICTED: acyl-CoA-binding domain-containing protein 4
RS007419	GO:0050662	coenzyme binding	MF	-0.810509574	-0.496169472	0.271583652	8.964283994	9.715009245	0.021150677	0.066476571	PREDICTED: probable isocitrate dehydrogenase [NAD] subunit alpha, mitochondrial
RS002956	GO:0050662	coenzyme binding	MF	-2.928056308	-0.897843684	-0.292887679	1.412347179	9.602623126	0.022264315	0.069370802	PREDICTED: glucose dehydrogenase [FAD, quinone]-like
RS007160	GO:0050662	coenzyme binding	MF	-0.549555066	-1.005296558	-0.449020655	6.369743433	9.315822987	0.025373612	0.077090385	cytoplasmic NADP+-dependent isocitrate dehydrogenase
RS006776	GO:0050662	coenzyme binding	MF	0.71638764	0.579864743	0.458766163	5.992561337	8.584837864	0.035351611	0.10061428	PREDICTED: NADPH-dependent diflavin oxidoreductase 1
RS015504	GO:0050662	coenzyme binding	MF	-0.346792054	-0.919939272	-0.688384249	5.423422413	7.961040774	0.046823775	0.125842642	NAD(P) transhydrogenase, mitochondrial
RS008221	GO:0050662	coenzyme binding	MF	0.494584107	0.577949482	0.596443217	6.228276146	7.64532803	0.053939612	0.140674151	PREDICTED: S-adenosyl-L-methionine-dependent tRNA 4-demethylwyosine synthase
RS004672	GO:0050662	coenzyme binding	MF	0.353656262	0.61047509	0.327590838	7.102946281	7.619168949	0.05457427	0.142005858	PREDICTED: coatomer subunit epsilon
RS004318	GO:0050662	coenzyme binding	MF	0.491549724	0.757087987	0.844223327	5.176959997	7.614963704	0.05467697	0.142243701	PREDICTED: glyoxylate/hydroxypyruvate reductase A-like
RS003700	GO:0050662	coenzyme binding	MF	0.445500208	0.547805665	0.90099913	5.029149185	7.491054113	0.057788756	0.148619889	PREDICTED: UDP-glucose 6-dehydrogenase
RS007230	GO:0050662	coenzyme binding	MF	-0.595941154	-0.25795038	0.362903371	9.093119676	7.171002242	0.066642534	0.165140653	PREDICTED: isocitrate dehydrogenase [NAD] subunit beta, mitochondrial
RS010959	GO:0050662	coenzyme binding	MF	-0.01139964	0.540038099	0.153812167	4.863464778	6.854286188	0.076690074	0.183723568	PREDICTED: probable sulfite oxidase, mitochondrial
RS007471	GO:0050662	coenzyme binding	MF	-0.166455797	0.50637605	-0.01116969	8.121929428	6.835928055	0.077315296	0.184975082	PREDICTED: lambda-crystallin homolog
RS008195	GO:0050662	coenzyme binding	MF	0.533863066	0.426337346	0.621528576	6.350472863	6.759324797	0.079977665	0.189622234	PREDICTED: NAD-dependent protein deacylase sirtuin-5, mitochondrial
RS002576	GO:0050662	coenzyme binding	MF	-0.620693831	-0.682180231	-0.612309734	6.887876847	6.631270757	0.084626811	0.198360331	PREDICTED: NADP-dependent malic enzyme
RS012259	GO:0050662	coenzyme binding	MF	-0.47689811	-0.550626227	-0.829212685	8.367846941	6.487657393	0.090150539	0.2083745	PREDICTED: putative glutamate synthase [NADPH]
RS012144	GO:0050662	coenzyme binding	MF	0.492953747	0.364658232	0.724065803	7.642199443	6.330771461	0.096580778	0.218844606	PREDICTED: acyl-CoA-binding domain-containing protein 6
RS000296	GO:0050662	coenzyme binding	MF	-0.154575641	-0.731686684	-0.647867681	5.62740596	6.283265117	0.09861327	0.222250451	PREDICTED: peroxisomal acyl-coenzyme A oxidase 3
RS011722	GO:0050662	coenzyme binding	MF	0.237984518	0.417339262	0.681790752	4.012277886	6.150741526	0.104502256	0.231807939	PREDICTED: NAD-dependent protein deacetylase Sirt4-like
RS006951	GO:0050662	coenzyme binding	MF	0.362871559	0.199149505	-0.106154987	6.457125486	5.692470091	0.127569407	0.268446696	PREDICTED: lysine-specific histone demethylase 1A
RS009629	GO:0050662	coenzyme binding	MF	-0.691618563	-0.413021374	0.205371865	9.600427322	5.679143987	0.128307911	0.269372103	PREDICTED: isocitrate dehydrogenase [NAD] subunit gamma, mitochondrial
RS007152	GO:0050662	coenzyme binding	MF	-0.79033743	-0.938189121	-1.446925399	0.97652372	5.151356345	0.161043715	0.317897264	PREDICTED: neuroligin-4, Y-linked-like
RS009326	GO:0050662	coenzyme binding	MF	0.162898799	-0.39930567	0.293003033	5.545630119	4.932236884	0.176826874	0.339837883	phosphoenolpyruvate decarboxylase
RS010695	GO:0050662	coenzyme binding	MF	0.518417779	0.062491693	-0.045494596	3.878001861	4.706009864	0.194634444	0.362661072	PREDICTED: dihydrofolate reductase
RS015204	GO:0050662	coenzyme binding	MF	-0.40385631	-0.267684874	0.256413441	8.673377735	4.675269768	0.197179364	0.365763575	PREDICTED: NADH dehydrogenase [ubiquinone] flavoprotein 1, mitochondrial
RS005140	GO:0050662	coenzyme binding	MF	-0.416469633	-0.263839851	-0.01550935	6.34712109	4.659101598	0.198530293	0.36774386	PREDICTED: D-2-hydroxyglutarate dehydrogenase, mitochondrial
RS007359	GO:0050662	coenzyme binding	MF	-0.033186439	-0.296037869	-0.421053457	5.816316114	4.527960563	0.209809787	0.381376047	PREDICTED: putative oxidoreductase GLYR1 homolog
RS007491	GO:0050662	coenzyme binding	MF	-0.446508158	-0.228275875	-0.036390422	6.67592503	4.497785152	0.212487932	0.384471824	PREDICTED: NADP-dependent malic enzyme
RS005051	GO:0050662	coenzyme binding	MF	0.153997919	-0.221089516	0.305477893	5.583582093	4.392675483	0.222065502	0.395922591	PREDICTED: DNA repair and recombination protein RAD54-like
RS010926	GO:0050662	coenzyme binding	MF	0.254824514	0.395156365	0.422047001	6.39620994	3.972494832	0.264449549	0.445815046	PREDICTED: short/branched chain specific acyl-CoA dehydrogenase, mitochondrial
RS002455	GO:0050662	coenzyme binding	MF	-0.194242358	0.195237976	0.397980266	8.707600168	3.965482633	0.265215586	0.446421423	PREDICTED: very long-chain specific acyl-CoA dehydrogenase, mitochondrial
RS007857	GO:0050662	coenzyme binding	MF	0.019057041	0.160899051	0.363191345	6.304461316	3.810263323	0.282694589	0.465502367	PREDICTED: xanthine dehydrogenase
RS008249	GO:0050662	coenzyme binding	MF	-0.391953891	1.193441996	1.153073924	1.121001183	3.791832584	0.284837569	0.467988351	PREDICTED: glucose dehydrogenase [FAD, quinone]-like
RS014351	GO:0050662	coenzyme binding	MF	0.156227108	0.422293573	0.344826693	6.658243688	3.776584112	0.286621565	0.469477085	PREDICTED: thioredoxin reductase 1, mitochondrial
RS007459	GO:0050662	coenzyme binding	MF	-0.532660554	-0.028004958	-0.266996105	3.941775201	3.748025441	0.2899898	0.472829045	PREDICTED: NAD-dependent protein deacetylase sirtuin-1
RS005465	GO:0050662	coenzyme binding	MF	-0.255589849	-0.533680261	-0.609552513	2.6851131	3.620655703	0.305447325	0.488173047	PREDICTED: lysine-specific histone demethylase 1A
RS005467	GO:0050662	coenzyme binding	MF	0.676741581	0.380751093	0.512853019	2.637868892	3.538021229	0.315864272	0.498396739	PREDICTED: lysine-specific histone demethylase 1A
RS012147	GO:0050662	coenzyme binding	MF	-0.558038474	-0.077034269	0.32175258	2.735746395	3.396752893	0.33440186	0.516363048	PREDICTED: NAD-dependent protein deacetylase sirtuin-7
RS010621	GO:0050662	coenzyme binding	MF	0.319373764	0.387129475	0.372445429	7.228363378	3.353578394	0.340254876	0.521596292	PREDICTED: probable medium-chain specific acyl-CoA dehydrogenase, mitochondrial
RS002545	GO:0050662	coenzyme binding	MF	-0.486437272	-0.387085875	0.043929873	9.369782347	3.274374469	0.351225196	0.531905979	PREDICTED: NADH-ubiquinone oxidoreductase 49 kDa subunit
RS000974	GO:0050662	coenzyme binding	MF	0.063768524	0.231555619	0.399568336	7.698083467	3.143071865	0.370087987	0.551062675	PREDICTED: electron transfer flavoprotein subunit alpha, mitochondrial
RS010824	GO:0050662	coenzyme binding	MF	0.346918779	0.141745704	-0.020641719	5.248339231	3.076469603	0.379984682	0.560022359	PREDICTED: NAD-dependent protein deacetylase Sirt6
RS009084	GO:0050662	coenzyme binding	MF	-0.462615302	-0.281297931	-0.024727583	3.250454039	2.773936031	0.427808502	0.602394932	dTDP-glucose 4,6-dehydratase
RS012446	GO:0050662	coenzyme binding	MF	-0.33520903	-0.326059321	0.041645679	5.694017303	2.740242498	0.433431766	0.607177439	PREDICTED: NAD-dependent protein deacetylase sirtuin-3, mitochondrial
RS000379	GO:0050662	coenzyme binding	MF	0.238639889	0.002576451	-0.1801865	4.995979504	2.646519158	0.44939244	0.622623585	PREDICTED: protein MTO1 homolog, mitochondrial
RS006771	GO:0050662	coenzyme binding	MF	0.475535507	0.066379896	0.054545274	5.13346181	2.640006509	0.450519017	0.623635579	PREDICTED: C-terminal-binding protein
RS012274	GO:0050662	coenzyme binding	MF	0.250868348	0.130831382	-0.091269758	5.86768545	2.371175161	0.499022911	0.66490632	PREDICTED: probable GDP-L-fucose synthase
RS011967	GO:0050662	coenzyme binding	MF	-0.487444254	-0.165820874	-0.461490275	5.204715649	2.301911612	0.512154792	0.67540078	PREDICTED: flavin-containing monooxygenase FMO GS-OX-like 4
RS013061	GO:0050662	coenzyme binding	MF	0.055667037	0.39151864	0.322142146	3.784294836	2.253027649	0.521579236	0.683037215	PREDICTED: ubiquinone biosynthesis monooxygenase COQ6
RS001108	GO:0050662	coenzyme binding	MF	-0.351307875	-0.364078375	-0.192697335	4.467218055	2.150105206	0.54184283	0.698032794	PREDICTED: short-chain specific acyl-CoA dehydrogenase, mitochondrial
RS005332	GO:0050662	coenzyme binding	MF	0.03211789	-0.20554948	-0.243739032	6.261746697	2.15056386	0.541751269	0.698032794	PREDICTED: proline dehydrogenase 1, mitochondrial
RS011007	GO:0050662	coenzyme binding	MF	0.164182996	-0.125816708	-0.353987059	3.604506135	2.107128644	0.550471937	0.704536949	PREDICTED: E3 ubiquitin-protein ligase znrf1
RS000486	GO:0050662	coenzyme binding	MF	0.232389203	-0.011565709	-0.040844754	6.74387093	2.012991222	0.569714711	0.720350873	PREDICTED: apoptosis-inducing factor 1, mitochondrial
RS011393	GO:0050662	coenzyme binding	MF	-0.041730957	-0.230300536	0.07476639	6.804211163	2.01329809	0.569651229	0.720350873	PREDICTED: hydroxyacyl-coenzyme A dehydrogenase, mitochondrial-like
RS004746	GO:0050662	coenzyme binding	MF	-0.053765857	-0.290830416	-0.105459059	6.353586052	1.929510254	0.587165226	0.733679815	PREDICTED: puff-specific protein Bx42
RS005517	GO:0050662	coenzyme binding	MF	-0.040617769	0.277956167	0.454102457	9.66049094	1.870199645	0.599779119	0.744859307	PREDICTED: ATP-dependent RNA helicase p62-like
RS002439	GO:0050662	coenzyme binding	MF	0.322813085	0.111104145	-0.161839796	6.005037147	1.846999235	0.604761168	0.748757585	PREDICTED: UDP-glucose 4-epimerase-like
RS003259	GO:0050662	coenzyme binding	MF	0.345067689	0.214790695	0.161470789	5.364845303	1.826626715	0.609157877	0.751688644	PREDICTED: tRNA-dihydrouridine(20) synthase [NAD(P)+]-like
RS006120	GO:0050662	coenzyme binding	MF	0.282310094	0.26654938	0.010128256	4.191105626	1.744965367	0.626983667	0.765138788	PREDICTED: tRNA-dihydrouridine(47) synthase [NAD(P)(+)]-like
RS006985	GO:0050662	coenzyme binding	MF	0.006933679	0.269486901	-0.078587833	5.040871731	1.689258661	0.639324476	0.774122916	PREDICTED: probable 2-oxoglutarate dehydrogenase E1 component DHKTD1 homolog, mitochondrial
RS007101	GO:0050662	coenzyme binding	MF	0.302041092	0.019148607	0.249776245	3.7895307	1.606596267	0.657895087	0.786999499	FAD-linked oxidoreductase
RS002730	GO:0050662	coenzyme binding	MF	0.273731849	0.243679505	0.179335078	5.847607944	1.567473925	0.666787166	0.792742148	PREDICTED: tRNA-dihydrouridine(20a/20b) synthase [NAD(P)+]-like
RS014702	GO:0050662	coenzyme binding	MF	0.048428453	-0.238349601	-0.12992294	6.529352259	1.523281632	0.67690746	0.800220491	PREDICTED: glutaryl-CoA dehydrogenase, mitochondrial
RS003678	GO:0050662	coenzyme binding	MF	-0.334465949	-0.114289915	-0.203522905	4.076361414	1.425503068	0.69956771	0.815865352	PREDICTED: tRNA-dihydrouridine(16/17) synthase [NAD(P)(+)]-like
RS013491	GO:0050662	coenzyme binding	MF	0.50959189	0.011563311	0.381662714	3.356752204	1.246647485	0.741839413	0.847577485	PREDICTED: amine oxidase [flavin-containing] B
RS013047	GO:0050662	coenzyme binding	MF	0.231685496	0.106278735	0.095579367	5.899186066	1.241080553	0.743169287	0.848450098	PREDICTED: NAD-dependent protein deacetylase sirtuin-2
RS004876	GO:0050662	coenzyme binding	MF	0.254514777	-0.013585511	0.016602412	4.233428366	1.08122198	0.781608997	0.873820125	PREDICTED: 3-hydroxyisobutyrate dehydrogenase, mitochondrial
RS002770	GO:0050662	coenzyme binding	MF	-0.119360703	-0.359384597	-0.31109241	10.31331347	0.993680302	0.802781134	0.886207234	glyceraldehyde-3-phosphate dehydrogenase
RS015527	GO:0050662	coenzyme binding	MF	0.095932391	-0.126719206	0.030481933	5.507190068	0.897198446	0.826103835	0.901321295	PREDICTED: acyl-CoA dehydrogenase family member 9, mitochondrial
RS002905	GO:0050662	coenzyme binding	MF	0.088090998	0.253655374	0.156881191	4.076313798	0.723831965	0.867584626	0.928559597	PREDICTED: elongation factor Tu, mitochondrial-like
RS008782	GO:0050662	coenzyme binding	MF	0.087717049	0.093210778	0.206874064	7.105662688	0.684144487	0.876926837	0.93376185	PREDICTED: putative acyl-CoA-binding protein
RS013157	GO:0050662	coenzyme binding	MF	0.115195703	0.398542992	-0.12346977	0.94470257	0.569142202	0.903458769	0.9486709	Rm62
RS007877	GO:0050662	coenzyme binding	MF	-0.04006425	0.125386876	0.039173322	5.581612122	0.5433599	0.909267233	0.952321145	PREDICTED: D-aspartate oxidase
RS000629	GO:0050662	coenzyme binding	MF	0.00837018	0.116143531	-0.015561228	6.338092866	0.458808573	0.927843179	0.962389796	PREDICTED: thioredoxin reductase 3
RS000370	GO:0050662	coenzyme binding	MF	-0.324119489	-0.104677593	0.006917945	5.382292536	0.439925008	0.931876866	0.964422487	PREDICTED: nitric oxide synthase, salivary gland
RS013432	GO:0050662	coenzyme binding	MF	0.186161353	0.083115952	0.073559077	4.349613188	0.400756431	0.94008619	0.96877878	PREDICTED: glucose dehydrogenase [FAD, quinone]-like
RS005907	GO:0050662	coenzyme binding	MF	0.095461109	0.014842875	0.085453782	6.336643908	0.318112256	0.956586828	0.976930029	PREDICTED: Golgi resident protein GCP60
RS011718	GO:0022804	active transmembrane transporter activity	MF	-0.059496373	8.716087878	3.96160781	4.311804415	501.7385592	2.004E-108	4.762E-106	PREDICTED: glucose dehydrogenase [FAD, quinone]
RS007652	GO:0022804	active transmembrane transporter activity	MF	0.585883623	0.528505008	4.851177054	4.584889963	291.2117195	7.93792E-63	8.92591E-61	PREDICTED: solute carrier family 26 member 10-like
RS009243	GO:0022804	active transmembrane transporter activity	MF	-0.589487895	2.064901326	2.91752274	4.715025796	173.1275538	2.68806E-37	1.47832E-35	PREDICTED: multidrug resistance protein homolog 49
RS004606	GO:0022804	active transmembrane transporter activity	MF	-0.152775887	3.199032258	1.582974114	7.379011967	144.4003621	4.25168E-31	1.83376E-29	PREDICTED: peptide transporter family 1
RS004125	GO:0022804	active transmembrane transporter activity	MF	1.407227463	2.012417871	-0.423642507	5.906215214	131.7530272	2.26607E-28	8.8083E-27	PREDICTED: ABC transporter G family member 9
RS003228	GO:0022804	active transmembrane transporter activity	MF	-0.561103954	2.12048168	1.83683218	5.406987214	114.6440514	1.09829E-24	3.71824E-23	PREDICTED: b(0,+)-type amino acid transporter 1-like
RS013974	GO:0022804	active transmembrane transporter activity	MF	1.308057058	2.315023189	0.876327879	6.127768048	111.7248777	4.66769E-24	1.53887E-22	PREDICTED: monocarboxylate transporter 10
RS012045	GO:0022804	active transmembrane transporter activity	MF	-2.417547613	-3.846942398	-2.948472949	3.138267013	95.12499238	1.73561E-20	4.58246E-19	PREDICTED: sodium-independent sulfate anion transporter
RS002200	GO:0022804	active transmembrane transporter activity	MF	-2.534854462	-2.965618885	0.446701011	4.187537984	94.94596227	1.89639E-20	4.97566E-19	PREDICTED: ATP-binding cassette sub-family G member 4
RS014732	GO:0022804	active transmembrane transporter activity	MF	-2.171879817	-1.24728548	0.09932889	5.367265273	82.18961662	1.04062E-17	2.21004E-16	PREDICTED: solute carrier organic anion transporter family member 2A1
RS015390	GO:0022804	active transmembrane transporter activity	MF	-1.260588417	-0.88728058	1.174441311	5.643186003	81.54774944	1.42892E-17	3.01438E-16	PREDICTED: sodium-independent sulfate anion transporter
RS002366	GO:0022804	active transmembrane transporter activity	MF	-2.013469227	2.285528889	0.940362283	2.009763796	76.37983445	1.83374E-16	3.57494E-15	PREDICTED: sodium-dependent serotonin transporter
RS005453	GO:0022804	active transmembrane transporter activity	MF	0.668735883	1.879757994	0.412329477	5.54953181	75.34668517	3.05352E-16	5.83551E-15	PREDICTED: ATP-binding cassette sub-family G member 1
RS006315	GO:0022804	active transmembrane transporter activity	MF	-2.445857294	-4.975199992	0.147442902	3.069628072	71.7469443	1.80352E-15	3.24017E-14	PREDICTED: bumetanide-sensitive sodium-(potassium)-chloride cotransporter
RS001006	GO:0022804	active transmembrane transporter activity	MF	-1.308055843	0.748595123	2.011362686	7.151837524	64.31436805	7.03086E-14	1.12512E-12	PREDICTED: sodium-dependent nutrient amino acid transporter 1-like
RS007831	GO:0022804	active transmembrane transporter activity	MF	-0.285689079	2.23184291	-0.54998832	3.347851609	60.46667692	4.67238E-13	7.00523E-12	PREDICTED: sodium- and chloride-dependent GABA transporter ine-like
RS010878	GO:0022804	active transmembrane transporter activity	MF	-0.202147251	0.219397642	1.259334773	5.8954575	54.42606082	9.10217E-12	1.23527E-10	PREDICTED: band 3 anion transport protein
RS012141	GO:0022804	active transmembrane transporter activity	MF	-0.795199967	0.272005658	1.157168	4.936082578	49.75842476	8.99389E-11	1.12594E-09	PREDICTED: UNC93-like protein MFSD11
RS009380	GO:0022804	active transmembrane transporter activity	MF	1.181487238	0.230316163	-1.011984835	4.090504935	45.23692145	8.23976E-10	9.50289E-09	PREDICTED: b(0,+)-type amino acid transporter 1-like
RS001492	GO:0022804	active transmembrane transporter activity	MF	-1.047661717	-2.014014601	-0.725945308	5.708393329	45.11153039	8.76123E-10	1.00308E-08	PREDICTED: multidrug resistance-associated protein 4-like
RS009578	GO:0022804	active transmembrane transporter activity	MF	-2.77838182	-2.247321578	-2.454512864	4.019688809	42.39569844	3.30671E-09	3.59937E-08	PREDICTED: solute carrier family 28 member 3
RS001007	GO:0022804	active transmembrane transporter activity	MF	-0.057453873	2.550863666	4.593751253	0.34449975	41.36132174	5.4813E-09	5.84023E-08	PREDICTED: sodium-dependent nutrient amino acid transporter 1-like

RS015310	GO:0022804	active transmembrane transporter activity	MF	-1.26553277	0.940311337	0.163445694	5.405928146	41.08193487	6.2827E-09	6.64911E-08	PREDICTED: ATP-binding cassette sub-family G member 1
RS009711	GO:0022804	active transmembrane transporter activity	MF	0.862276886	1.272132135	1.421293308	6.056371062	36.7986326	5.07562E-08	4.85732E-07	PREDICTED: tropinone reductase 2-like
RS014465	GO:0022804	active transmembrane transporter activity	MF	-2.203563111	-3.568610423	-0.279641755	5.849726949	35.76065503	8.4137E-08	7.87237E-07	PREDICTED: probable multidrug resistance-associated protein lethal(2)03659
RS000365	GO:0022804	active transmembrane transporter activity	MF	-2.043121889	-2.26896537	0.189661525	11.00673959	34.00204383	1.97922E-07	1.77664E-06	PREDICTED: calcium-transporting ATPase sarcoplasmic/endoplasmic reticulum type
RS006320	GO:0022804	active transmembrane transporter activity	MF	1.668458777	1.549661536	0.932473469	6.183206129	30.51118393	1.07731E-06	8.96741E-06	PREDICTED: bumetanide-sensitive sodium-(potassium)-chloride cotransporter
RS007713	GO:0022804	active transmembrane transporter activity	MF	-1.593472957	-0.392083561	-0.180235411	5.938924251	30.40817878	1.13245E-06	9.40771E-06	PREDICTED: ATP-binding cassette sub-family D member 2
RS000193	GO:0022804	active transmembrane transporter activity	MF	-0.612906575	0.552707825	0.864925878	6.395375097	30.19397061	1.25629E-06	1.03772E-05	PREDICTED: b(0,+)-type amino acid transporter 1
RS010500	GO:0022804	active transmembrane transporter activity	MF	1.19026545	2.873457875	1.841995752	12.00045446	30.1935168	1.25657E-06	1.03772E-05	PREDICTED: tubulin alpha-1 chain
RS009579	GO:0022804	active transmembrane transporter activity	MF	-0.361377028	0.937501921	0.004604885	5.160969196	28.85056945	2.40732E-06	1.91039E-05	PREDICTED: solute carrier family 28 member 3-like
RS013310	GO:0022804	active transmembrane transporter activity	MF	-1.594173173	0.30942411	0.502900611	3.784418211	25.32704004	1.31907E-05	9.38549E-05	PREDICTED: sodium- and chloride-dependent transporter XTRP3
RS003638	GO:0022804	active transmembrane transporter activity	MF	-0.69625377	-0.738048152	0.351114728	5.7591544	24.53074045	1.93528E-05	0.00013355	PREDICTED: ATP-binding cassette sub-family G member 4
RS006319	GO:0022804	active transmembrane transporter activity	MF	-1.264071762	-1.378688832	-0.296664377	4.499770473	23.99784591	2.50059E-05	0.000169587	PREDICTED: bumetanide-sensitive sodium-(potassium)-chloride cotransporter
RS007091	GO:0022804	active transmembrane transporter activity	MF	-0.260344997	-0.000107201	0.982798512	6.405504865	23.00178887	4.03484E-05	0.000263836	PREDICTED: probable multidrug resistance-associated protein lethal(2)03659
RS011507	GO:0022804	active transmembrane transporter activity	MF	0.081672207	0.439418944	1.152509484	5.150989282	22.16598652	6.02438E-05	0.000382801	PREDICTED: sialin
RS012046	GO:0022804	active transmembrane transporter activity	MF	-0.044612109	1.318321788	-0.182662099	3.873303092	21.0021542	0.000105168	0.000636465	PREDICTED: sodium-independent sulfate anion transporter-like
RS015154	GO:0022804	active transmembrane transporter activity	MF	0.77118859	1.275597924	0.795023023	8.092063536	20.820501	0.00011471	0.000690232	PREDICTED: protein transport protein Sec61 subunit gamma
RS010854	GO:0022804	active transmembrane transporter activity	MF	-0.464601417	0.421962906	0.417617417	6.7210824	20.51691341	0.000132619	0.000789321	PREDICTED: V-type proton ATPase subunit e 2-like
RS007340	GO:0022804	active transmembrane transporter activity	MF	1.72289566	2.228109689	-0.488523344	0.853368064	20.24210443	0.000151218	0.000888679	PREDICTED: mitochondrial sodium/hydrogen exchanger 9B2
RS004393	GO:0022804	active transmembrane transporter activity	MF	-1.227790861	-2.264108903	-0.817189459	2.289519889	20.23673107	0.000151606	0.000890546	PREDICTED: putative tricarboxylate transport protein, mitochondrial
RS007614	GO:0022804	active transmembrane transporter activity	MF	0.699165628	-0.052782673	-0.817546641	5.472681929	18.94140065	0.000281133	0.001554478	PREDICTED: facilitated trehalose transporter Tret1-like
RS007694	GO:0022804	active transmembrane transporter activity	MF	-1.402205119	-1.429007484	-0.154037672	3.235518975	18.72862507	0.000311092	0.001703432	PREDICTED: proton myo-inositol cotransporter-like
RS009272	GO:0022804	active transmembrane transporter activity	MF	-0.152985688	-1.205701524	-0.53386679	4.590610282	18.71946093	0.000312451	0.001710132	PREDICTED: mitochondrial sodium/hydrogen exchanger 9B2
RS011525	GO:0022804	active transmembrane transporter activity	MF	-0.806138244	0.427082827	0.274818707	7.810153084	18.69931455	0.000315461	0.001724354	PREDICTED: sodium/potassium-transporting ATPase subunit beta-2
RS009604	GO:0022804	active transmembrane transporter activity	MF	-1.648345631	0.550771769	0.526514629	2.368500525	18.41833332	0.000360561	0.001945804	PREDICTED: sodium-dependent noradrenaline transporter
RS014410	GO:0022804	active transmembrane transporter activity	MF	-0.538555444	-0.069436224	0.424307658	5.574699261	17.86202393	0.00046963	0.002466436	PREDICTED: sodium- and chloride-dependent neutral and basic amino acid transporter B(0+)
RS000837	GO:0022804	active transmembrane transporter activity	MF	-1.260815808	-0.582381283	0.014148923	5.012164404	17.85146996	0.000471988	0.002476758	PREDICTED: excitatory amino acid transporter 1-like
RS003799	GO:0022804	active transmembrane transporter activity	MF	-0.143761693	0.372761559	-0.639546518	5.875204513	17.46636866	0.000566606	0.002928125	PREDICTED: ATP-binding cassette sub-family C member Sur
RS012047	GO:0022804	active transmembrane transporter activity	MF	-1.033691566	-1.204387105	-0.573210977	3.717195265	16.53395031	0.000881135	0.004370625	PREDICTED: sodium-independent sulfate anion transporter-like
RS009094	GO:0022804	active transmembrane transporter activity	MF	0.810509918	0.868482563	0.707370764	6.315762678	16.22341499	0.001020437	0.004986955	PREDICTED: mitochondrial import inner membrane translocase subunit Tim17-B-like
RS013870	GO:0022804	active transmembrane transporter activity	MF	-1.185159015	-0.789079424	-0.942978819	4.024142004	15.86729932	0.001207277	0.005765813	PREDICTED: sodium- and chloride-dependent glycine transporter 1-like
RS013164	GO:0022804	active transmembrane transporter activity	MF	-0.720894807	-1.573394185	-0.567112816	2.35012097	15.85215695	0.001215934	0.005800558	PREDICTED: sodium/hydrogen exchanger 3
RS012306	GO:0022804	active transmembrane transporter activity	MF	-1.171605666	-1.08070317	-0.099574832	5.667246046	15.66500563	0.001328153	0.006252994	PREDICTED: ABC transporter G family member 23
RS000928	GO:0022804	active transmembrane transporter activity	MF	-2.818387407	-2.387215456	-1.195316578	0.324906115	15.29049888	0.001584497	0.007309578	B(0,+)-type amino acid transporter 1
RS009126	GO:0022804	active transmembrane transporter activity	MF	0.004334845	0.550905396	0.691970165	7.068681097	14.88906784	0.001913944	0.008599435	PREDICTED: V-type proton ATPase subunit F
RS006318	GO:0022804	active transmembrane transporter activity	MF	-3.618949223	-2.702749807	-1.072386578	0.467850565	14.73641507	0.002056334	0.009147816	PREDICTED: bumetanide-sensitive sodium-(potassium)-chloride cotransporter
RS005708	GO:0022804	active transmembrane transporter activity	MF	-2.57707626	-0.767810406	-0.121337824	1.208165742	14.61680361	0.002175202	0.009598631	PREDICTED: sodium- and chloride-dependent glycine transporter 1-like
RS008723	GO:0022804	active transmembrane transporter activity	MF	0.765724899	0.492155106	-0.726953396	2.604372289	14.25219643	0.002581214	0.011140444	PREDICTED: excitatory amino acid transporter
RS013212	GO:0022804	active transmembrane transporter activity	MF	-0.878244991	-0.110123051	0.018139925	7.487946573	14.16068921	0.002694389	0.011553674	PREDICTED: V-type proton ATPase subunit B
RS004592	GO:0022804	active transmembrane transporter activity	MF	-0.294046429	0.156068735	0.564893198	6.831343324	13.651508	0.003420014	0.014243271	PREDICTED: ATP-binding cassette sub-family B member 7, mitochondrial
RS006255	GO:0022804	active transmembrane transporter activity	MF	-0.565848028	-0.409978879	0.214379631	6.839035823	13.48918712	0.003689736	0.015225603	PREDICTED: copper-transporting ATPase 1
RS012010	GO:0022804	active transmembrane transporter activity	MF	-0.658055692	-0.163236513	0.25753802	6.662027841	13.45566221	0.003748014	0.01543574	PREDICTED: sodium-dependent phosphate transporter 1-B-like
RS010731	GO:0022804	active transmembrane transporter activity	MF	-0.395182325	-0.639006133	-0.675231861	6.20607259	13.34648856	0.003944186	0.016153848	PREDICTED: V-type proton ATPase 116 kDa subunit a
RS015097	GO:0022804	active transmembrane transporter activity	MF	0.838222973	0.450337326	-0.261713518	6.777317829	13.04891082	0.004532018	0.018148247	PREDICTED: tubulin alpha-1 chain
RS009863	GO:0022804	active transmembrane transporter activity	MF	0.247967949	1.994626146	0.840783905	0.741237487	12.85234652	0.004967042	0.019634316	PREDICTED: sodium- and chloride-dependent glycine transporter 1-like
RS005166	GO:0022804	active transmembrane transporter activity	MF	0.797745292	0.959053152	1.137980998	8.88871257	12.83598972	0.005005051	0.019744379	PREDICTED: nascent polypeptide-associated complex subunit alpha
RS005241	GO:0022804	active transmembrane transporter activity	MF	-1.24648029	-0.946331544	-0.692073388	4.41212653	12.5745733	0.005653054	0.021933014	PREDICTED: high-affinity choline transporter 1
RS000227	GO:0022804	active transmembrane transporter activity	MF	-1.32952285	-0.75353739	-1.12184367	1.981758767	12.38833226	0.00616466	0.023605269	PREDICTED: facilitated trehalose transporter Tret1-2 homolog
RS008215	GO:0022804	active transmembrane transporter activity	MF	0.162922308	0.46909669	0.671411093	6.515514341	12.06788131	0.007154208	0.026831478	PREDICTED: mitochondrial import receptor subunit TOM22 homolog
RS003288	GO:0022804	active transmembrane transporter activity	MF	-0.468141457	-0.663116971	0.331112622	4.523147937	12.02307867	0.007304519	0.027292266	PREDICTED: sodium- and chloride-dependent GABA transporter ine
RS011468	GO:0022804	active transmembrane transporter activity	MF	-0.318737056	-0.110014844	-1.568608212	1.963272184	11.9167036	0.007674014	0.028433815	PREDICTED: solute carrier family 35 member F6
RS000866	GO:0022804	active transmembrane transporter activity	MF	-0.851954648	-0.389874807	-0.092110043	7.989972168	11.90615845	0.007711634	0.028547895	PREDICTED: sodium/potassium-transporting ATPase subunit alpha
RS003756	GO:0022804	active transmembrane transporter activity	MF	-0.408342321	-0.364800511	-0.786353931	7.1095919	11.79402076	0.00812312	0.029860645	PREDICTED: multidrug resistance-associated protein 1
RS015096	GO:0022804	active transmembrane transporter activity	MF	-0.857980934	-0.446788274	-0.194471442	5.697782265	11.71197794	0.008437836	0.030900291	PREDICTED: V-type proton ATPase subunit D
RS009360	GO:0022804	active transmembrane transporter activity	MF	-0.052778092	-0.516301747	0.304429459	6.031942967	11.69868265	0.008489957	0.031037017	PREDICTED: sodium-dependent nutrient amino acid transporter 1
RS008458	GO:0022804	active transmembrane transporter activity	MF	-1.182823715	-0.196910535	-0.390818396	8.429616967	11.6884148	0.008530428	0.031131399	PREDICTED: probable cation-transporting ATPase 13A3
RS002363	GO:0022804	active transmembrane transporter activity	MF	-0.329275879	-0.729371826	0.101654401	6.167217704	11.55948198	0.009055119	0.032732541	PREDICTED: multidrug resistance-associated protein 5
RS002295	GO:0022804	active transmembrane transporter activity	MF	-0.760632417	-0.881718429	-1.336627404	7.31207469	11.50237509	0.009297576	0.033436228	PREDICTED: sodium- and chloride-dependent GABA transporter 1-like
RS007389	GO:0022804	active transmembrane transporter activity	MF	-0.71821859	-0.059688714	0.178211598	8.141021926	11.17756615	0.010803454	0.037931058	PREDICTED: V-type proton ATPase catalytic subunit A
RS007388	GO:0022804	active transmembrane transporter activity	MF	-1.012601041	-0.502641916	-0.211964145	6.238906794	11.11536226	0.011118147	0.038884183	PREDICTED: V-type proton ATPase catalytic subunit A
RS000123	GO:0022804	active transmembrane transporter activity	MF	0.692109916	0.810423229	0.325162475	7.0801236	10.8417935	0.012612853	0.04340062	PREDICTED: ATP-binding cassette sub-family F member 3
RS011127	GO:0022804	active transmembrane transporter activity	MF	0.510801033	0.668255039	0.469000295	5.728845819	10.81526531	0.01276792	0.043826433	PREDICTED: ATP-binding cassette sub-family F member 2
RS002816	GO:0022804	active transmembrane transporter activity	MF	-0.748548229	0.030574886	-0.160819942	5.861566925	10.7303973	0.013276729	0.045264516	PREDICTED: multidrug resistance protein 1-like
RS012007	GO:0022804	active transmembrane transporter activity	MF	-0.8805953	0.446042174	-0.616249485	2.603532973	10.7020781	0.013450915	0.045771636	PREDICTED: sodium-dependent phosphate transporter 2
RS010501	GO:0022804	active transmembrane transporter activity	MF	-0.249581193	0.164542324	0.830736506	8.23731792	10.57427827	0.014265398	0.048114201	PREDICTED: ATP synthase subunit e, mitochondrial
RS012348	GO:0022804	active transmembrane transporter activity	MF	-1.171136152	-0.623448827	-0.241561177	4.399276235	10.47489738	0.014932153	0.050028076	V-type proton ATPase subunit S1
RS004574	GO:0022804	active transmembrane transporter activity	MF	-0.256414135	0.160648707	0.765161971	8.179741228	10.46991438	0.01496638	0.050122195	PREDICTED: ATP synthase subunit delta, mitochondrial
RS005209	GO:0022804	active transmembrane transporter activity	MF	-0.863563851	-1.023827568	-0.616859801	5.79782502	10.40323263	0.015431901	0.051374401	PREDICTED: multidrug resistance protein homolog 49
RS000868	GO:0022804	active transmembrane transporter activity	MF	-0.929392451	-0.475108864	-0.289097862	8.324170965	10.36013171	0.01574035	0.052235565	PREDICTED: sodium/potassium-transporting ATPase subunit alpha
RS009153	GO:0022804	active transmembrane transporter activity	MF	0.613293967	0.998084897	0.966219795	3.330543492	10.10124836	0.017724806	0.057621634	PREDICTED: mitochondrial import inner membrane translocase subunit Tim9
RS005883	GO:0022804	active transmembrane transporter activity	MF	0.579677196	0.662723942	0.941061143	6.190916775	10.02257363	0.018375223	0.059324417	PREDICTED: mitochondrial import inner membrane translocase subunit Tim10
RS002046	GO:0022804	active transmembrane transporter activity	MF	-1.040945999	-1.141609481	-1.378037143	3.132734296	9.81352734	0.020219576	0.06404561	PREDICTED: sodium- and chloride-dependent GABA transporter 1-like
RS006809	GO:0022804	active transmembrane transporter activity	MF	-0.069599294	0.161390813	-0.669189997	5.521140821	9.427681054	0.02411329	0.074118459	PREDICTED: sodium bicarbonate cotransporter 3
RS003636	GO:0022804	active transmembrane transporter activity	MF	0.658899301	-0.027122995	0.775979951	5.812404377	9.412838955	0.024276916	0.074486992	PREDICTED: ATP-binding cassette sub-family G member 4
RS007719	GO:0022804	active transmembrane transporter activity	MF	-0.363554514	0.201823484	0.241238455	6.872179235	9.390306687	0.024527407	0.075102884	PREDICTED: V-type proton ATPase 21 kDa proteolipid subunit
RS005942	GO:0022804	active transmembrane transporter activity	MF	0.222003058	0.357500804	-0.707071584	4.173704825	9.375192754	0.024696847	0.075474905	PREDICTED: mitochondrial sodium/hydrogen exchanger 9B2-like
RS003802	GO:0022804	active transmembrane transporter activity	MF	0.811082921	0.486372202	-0.08894724	2.933919036	9.26719754	0.025941417	0.078459703	PREDICTED: cGMP-dependent protein kinase, isozyme 1
RS009928	GO:0022804	active transmembrane transporter activity	MF	-0.835456878	-2.347674561	-0.849585016	1.053053669	9.23428246	0.02633282	0.079385129	PREDICTED: sodium-dependent noradrenaline transporter-like
RS003234	GO:0022804	active transmembrane transporter activity	MF	-0.402942012	0.351806881	0.253791792	7.730694199	9.198914238	0.026759846	0.080432815	PREDICTED: V-type proton ATPase 16 kDa proteolipid subunit
RS012808	GO:0022804	active transmembrane transporter activity	MF	0.436531101	0.768358177	0.047570539	8.601906761	9.12311113	0.027698056	0.082719781	PREDICTED: ATP-binding cassette sub-family E member 1
RS011130	GO:0022804	active transmembrane transporter activity	MF	0.62266454	0.316671327	-0.198111492	8.646709884	8.691352167	0.03368886	0.096889131	PREDICTED: 60 kDa heat shock protein, mitochondrial-like
RS006017	GO:0022804	active transmembrane transporter activity	MF	-0.226333875	0.829010789	0.173010283	3.457042927	8.42538268	0.037991671	0.106302401	PREDICTED: cationic amino acid transporter 2
RS003858	GO:0022804	active transmembrane transporter activity	MF	-0.359815662	-0.446595841	0.382189615	3.498410245	8.385315512	0.038684752	0.10786673	PREDICTED: sodium/potassium-transporting ATPase subunit beta-2-like
RS012784	GO:0022804	active transmembrane transporter activity	MF	-0.964188065	-1.053670411	-1.487092291	6.693074429	8.119987423	0.043595935	0.118762068	PREDICTED: excitatory amino acid transporter-like
RS005210	GO:0022804	active transmembrane transporter activity	MF	-0.986436868	-1.240920184	-1.092022056	3.917975026	7.935991254	0.047353263	0.126994675	PREDICTED: multidrug resistance protein homolog 49
RS007665	GO:0022804	active transmembrane transporter activity	MF	-0.631881952	-0.428365421	0.007983181	8.098194771	7.871009593	0.048754144	0.129949141	PREDICTED: plasma membrane calcium-transporting ATPase 2
RS010293	GO:0022804	active transmembrane transporter activity	MF	-1.323536549	-0.537537624	-0.40616185	2.969834016	7.797939139	0.050377598	0.133149101	PREDICTED: sodium/hydrogen exchanger 3
RS012051	GO:0022804	active transmembrane transporter activity	MF	-1.206457775	-0.320139517	-0.400442703	3.93903891	7.774642457	0.050906164	0.13434875	PREDICTED: sodium-independent sulfate anion transporter-like
RS012317	GO:0022804	active transmembrane transporter activity	MF	-0.648427381	0.001131192	-0.007541087	6.300999393	7.771586789	0.050975892	0.13447641	PREDICTED: calcium-transporting ATPase type 2C member 1
RS015407	GO:0022804	active transmembrane transporter activity	MF	-0.53343531	-0.824506724	0.003613607	6.554411162	7.589715422	0.055297539	0.143502413	PREDICTED: sodium/potassium-transporting ATPase subunit beta-2
RS005847	GO:0022804	active transmembrane transporter activity	MF	-0.009134422	0.240630937	0.785370217	8.208365721	7.589016609	0.055314812	0.143517665	PREDICTED: ATP synthase subunit epsilon, mitochondrial-like
RS002799	GO:0022804	active transmembrane transporter activity	MF	-0.659046602	-0.211273757	-0.16274335	6.419710777	7.409924332	0.059918615	0.152787009	PREDICTED: V-type proton ATPase subunit H
RS002292	GO:0022804	active transmembrane transporter activity	MF	-0.849226044	-0.724590211	-0.752733052	3.999828127	7.339934983	0.06181703	0.156330055	V-type proton ATPase subunit S1
RS005264	GO:0022804	active transmembrane transporter activity	MF	-1.488549143	-1.308665565	-1.379377385	1.600885014	7.17732657	0.066455483	0.164816926	PREDICTED: sodium-dependent neutral amino acid transporter B(0)AT3
RS015404	GO:0022804	active transmembrane transporter activity	MF	-0.033173942	0.491254687	-0.13268681	6.364272838	7.035330626	0.07078015	0.172954058	PREDICTED: sodium/potassium-transporting ATPase subunit beta-2
RS012048	GO:0022804	active transmembrane transporter activity	MF	-0.674894361	-1.155560739	-0.486644875	2.649535097	6.691851744	0.082395778	0.193852499	PREDICTED: sodium-independent sulfate anion transporter-like
RS002982	GO:0022804	active transmembrane transporter activity	MF	0.437159386	0.690352357	0.482073212	5.918755174	6.64375724	0.084162258	0.197455193	PREDICTED: multidrug resistance-associated protein 5-like
RS010561	GO:0022804	active transmembrane transporter activity	MF	0.077383384	1.01828952	0.325351919	10.99254901	6.38788389	0.094190516	0.215171867	PREDICTED: tubulin alpha-1 chain
RS010275	GO:0022804	active transmembrane transporter activity	MF	-0.327809837	0.151537852	-0.881426827	3.129420115	6.335110353	0.096397162	0.218625222	PREDICTED: equilibrative nucleoside transporter 4
RS007852	GO:0022804	active transmembrane transporter activity	MF	-0.4463895	-0.500436907	-0.353366809	5.359879642	5.946281355	0.114253205	0.24733912	PREDICTED: multidrug resistance-associated protein 7
RS009361	GO:0022804	active transmembrane transporter activity	MF	-0.469548252	-1.030089652	-0.272125463	2.379260128	5.571170404	0.134443268	0.278300217	PREDICTED: sodium- and chloride-dependent glycine transporter 2-like
RS002972	GO:0022804	active transmembrane transporter activity	MF	-0.311172151	-0.506842424	-0.237806006	6.495567074	5.534163734	0.136609467	0.281857737	PREDICTED: sodium leak channel non-selective protein
RS012521	GO:0022804	active transmembrane transporter activity	MF	-0.27911124	0.207963234	0.176143396	5.682101261	5.464162095	0.140797903	0.287847294	PREDICTED: sodium/hydrogen exchanger 8
RS007787	GO:0022804	active transmembrane transporter activity	MF	-0.305912555	0.11337882	0.601620189	9.43857381	5.186627171	0.15863048	0.314655166	PREDICTED: ATP synthase subunit b, mitochondrial
RS002769	GO:0022804	active transmembrane transporter activity	MF	-0.180596666	-0.510025269	-0.494314256	5.266272897	5.147923967	0.161280396	0.318264698	PREDICTED: mitochondrial 2-oxoglutarate/malate carrier protein
RS000666	GO:0022804	active transmembrane transporter activity	MF	-0.080539122	0.013403368	0.652885093	9.136399161	5.147116783	0.161336103	0.31832475	PREDICTED: ATP synthase subunit g, mitochondrial
RS006076	GO:0022804	active transmembrane transporter activity	MF	-0.399500112	-0.234112198	-0.791572834	3.218719557	5.135126595	0.162165734	0.319411206	PREDICTED: cationic amino acid transporter 4
RS015132	GO:0022804	active transmembrane transporter activity	MF	-0.311288053	0.021217655	0.441252337	8.852842665	5.035692944	0.169202112	0.329814486	PREDICTED: ATP synthase subunit O, mitochondrial
RS001012	GO:0022804	active transmembrane transporter activity	MF	-0.050637771	0.65837124	0.220417241	5.028311487	5.034189195	0.169310694	0.329872366	PREDICTED: sodium/calcium exchanger 2
RS007406	GO:0022804	active transmembrane transporter activity	MF	1.53218285	1.416234104	0.234676903	0.509107572	4.915967777	0.17805483	0.341676447	PREDICTED: sodium-independent sulfate anion transporter-like
RS012974	GO:0022804	active transmembrane transporter activity	MF	0.386362631	0.350475987	0.590578301	4.958398051	4.857275707	0.182551447	0.347919632	PREDICTED: mitochondrial import inner membrane translocase subunit Tim16
RS015406	GO:0022804	active transmembrane transporter activity	MF	-0.548835798	-0.34994381	-0.083936965	5.176471396	4.839807661	0.183910115	0.349694714	PREDICTED: sodium/potassium-transporting ATPase subunit beta-2

RS002889	GO:0022804	active transmembrane transporter activity	MF	-0.128703142	0.3529612	0.286577104	4.360050122	4.605415706	0.203077987	0.373149135 PREDICTED: bifunctional arginine demethylase and lysyl-hydroxylase PSR
RS013960	GO:0022804	active transmembrane transporter activity	MF	-0.472338012	-0.452157922	0.021607712	3.915657005	4.540751566	0.208684017	0.380067463 PREDICTED: multidrug resistance-associated protein 5-like
RS009362	GO:0022804	active transmembrane transporter activity	MF	-0.151734251	-1.379851033	-0.853593219	0.577299208	4.353138426	0.225770076	0.399764452 PREDICTED: sodium- and chloride-dependent glycine transporter 2-like
RS012560	GO:0022804	active transmembrane transporter activity	MF	-0.041301573	-0.515464379	-0.426603809	4.255404315	4.307057209	0.230159613	0.404677505 PREDICTED: ATP-binding cassette sub-family B member 6, mitochondrial
RS007791	GO:0022804	active transmembrane transporter activity	MF	-0.379787464	0.049869261	0.105041138	7.48004628	4.225689753	0.238102407	0.41486742 PREDICTED: V-type proton ATPase subunit E
RS015079	GO:0022804	active transmembrane transporter activity	MF	-1.376295246	0.661290456	0.618943801	0.209343525	4.174647883	0.243212401	0.420568148 PREDICTED: facilitated trehalose transporter Tret1-like
RS011747	GO:0022804	active transmembrane transporter activity	MF	-0.380454491	0.007681606	0.358897081	9.615965078	3.849118736	0.278224261	0.460864966 PREDICTED: ATP synthase subunit gamma, mitochondrial
RS000693	GO:0022804	active transmembrane transporter activity	MF	-0.697277451	-0.63859341	-1.105155719	1.668516432	3.761841056	0.288355965	0.47128419 PREDICTED: solute carrier family 12 member 4
RS004593	GO:0022804	active transmembrane transporter activity	MF	0.262368668	-0.314833663	-0.606891149	2.686998086	3.735102132	0.29152562	0.474414272 PREDICTED: SET domain-containing protein 4
RS014392	GO:0022804	active transmembrane transporter activity	MF	-0.97591621	-0.707349255	0.232142575	0.530622375	3.721584252	0.293139895	0.476361786 PREDICTED: probable multidrug resistance-associated protein lethal(2)03659
RS014617	GO:0022804	active transmembrane transporter activity	MF	-0.072631645	0.084385518	0.526454273	9.138635249	3.625994331	0.304784959	0.487596216 PREDICTED: putative ATP synthase subunit f, mitochondrial
RS015453	GO:0022804	active transmembrane transporter activity	MF	-0.09263959	0.415348464	0.277714417	5.686672294	3.355959113	0.33992981	0.521573591 PREDICTED: sodium-dependent phosphate transporter 2
RS001904	GO:0022804	active transmembrane transporter activity	MF	-0.27687136	-0.081401122	0.3505686	9.687890931	2.842286187	0.416586082	0.592334615 PREDICTED: ATP synthase subunit d, mitochondrial
RS007790	GO:0022804	active transmembrane transporter activity	MF	0.13034852	0.225712601	0.386180211	4.625198528	2.606288996	0.456388071	0.628856824 PREDICTED: mitochondrial glutamate carrier 1-like
RS007514	GO:0022804	active transmembrane transporter activity	MF	-0.393644456	0.206862252	-0.369998274	2.851975701	2.440356048	0.48616657	0.653933758 PREDICTED: solute carrier family 26 member 10-like
RS002215	GO:0022804	active transmembrane transporter activity	MF	-0.459893543	-0.373663745	-0.327616751	3.245153068	2.258109459	0.520593488	0.682313913 PREDICTED: CMP-sialic acid transporter 1
RS010646	GO:0022804	active transmembrane transporter activity	MF	-0.387797302	-0.153467445	-0.201059514	5.65127642	2.191739529	0.533577259	0.692056848 PREDICTED: probable phospholipid-transporting ATPase IA
RS009552	GO:0022804	active transmembrane transporter activity	MF	-0.317174334	0.102956036	0.402195532	11.51695741	2.083221242	0.555314671	0.708862049 PREDICTED: ATP synthase subunit beta, mitochondrial
RS002902	GO:0022804	active transmembrane transporter activity	MF	-0.415647108	-0.152738152	0.265175951	11.47381982	2.020686865	0.56812419	0.719524944 PREDICTED: ATP synthase subunit alpha, mitochondrial
RS010787	GO:0022804	active transmembrane transporter activity	MF	0.111909178	-0.194537134	-0.164571213	5.38282424	1.963969035	0.579918644	0.728455555 PREDICTED: ATP-binding cassette sub-family F member 1
RS003602	GO:0022804	active transmembrane transporter activity	MF	-0.963739329	-0.484991816	-0.758608967	0.476696176	1.634124817	0.651677236	0.782683875 PREDICTED: solute carrier family 28 member 3
RS000107	GO:0022804	active transmembrane transporter activity	MF	0.215541555	0.120252028	-0.217617098	3.595365183	1.517996661	0.678122983	0.800549125 PREDICTED: multidrug resistance protein 1A
RS003990	GO:0022804	active transmembrane transporter activity	MF	-0.055368209	0.238070575	0.293320892	4.25914611	1.454595738	0.692788544	0.811066898 PREDICTED: UDP-galactose translocator
RS015498	GO:0022804	active transmembrane transporter activity	MF	0.184679301	0.472371696	0.26432428	2.413896137	1.414695214	0.702093736	0.818113296 PREDICTED: calcium uniporter protein, mitochondrial
RS004705	GO:0022804	active transmembrane transporter activity	MF	-0.430701847	-0.465359916	-0.484712083	3.064343052	1.370281204	0.712515526	0.826890945 PREDICTED: sodium/hydrogen exchanger 7
RS005898	GO:0022804	active transmembrane transporter activity	MF	-0.109533007	-0.24419355	-0.181501684	5.469138988	1.210081204	0.750587452	0.852649778 PREDICTED: ATP-binding cassette sub-family B member 10, mitochondrial
RS008405	GO:0022804	active transmembrane transporter activity	MF	-0.507380646	-0.391727947	-0.160990784	2.501471485	1.183658987	0.75692617	0.857267824 PREDICTED: solute carrier family 12 member 9
RS013461	GO:0022804	active transmembrane transporter activity	MF	0.139158606	-0.101013347	0.199986874	6.071677902	1.15813645	0.763061416	0.861033552 PREDICTED: ATP-binding cassette sub-family G member 5
RS001035	GO:0022804	active transmembrane transporter activity	MF	0.124094061	0.276516429	0.187901485	8.14568359	1.113792392	0.773745632	0.868231611 PREDICTED: tubulin alpha-1 chain
RS014288	GO:0022804	active transmembrane transporter activity	MF	-0.267562999	-0.21719274	-0.273627167	4.00532462	1.061069676	0.786479339	0.876852054 PREDICTED: ATP-binding cassette sub-family B member 8, mitochondrial
RS004706	GO:0022804	active transmembrane transporter activity	MF	-0.281248638	-0.101038833	-0.415774939	3.347688564	1.047260677	0.789818336	0.878882504 PREDICTED: sodium/hydrogen exchanger 7
RS007150	GO:0022804	active transmembrane transporter activity	MF	0.246217518	0.212666126	0.29750827	5.406495568	0.963851814	0.80999779	0.890926827 PREDICTED: probable multidrug resistance-associated protein lethal(2)03659
RS007717	GO:0022804	active transmembrane transporter activity	MF	0.066529061	0.463299961	0.014408782	1.808915589	0.956769727	0.811710752	0.892106596 PREDICTED: V-type proton ATPase 21 kDa proteolipid subunit
RS006075	GO:0022804	active transmembrane transporter activity	MF	-0.226394382	-0.065362369	0.088572196	3.008430896	0.898142308	0.82587609	0.901150881 PREDICTED: acetyl-coenzyme A transporter 1
RS013172	GO:0022804	active transmembrane transporter activity	MF	0.064707769	0.050177661	0.256276698	4.54520608	0.870549691	0.832528393	0.905630734 PREDICTED: N-acetyl-D-glucosamine kinase
RS010583	GO:0022804	active transmembrane transporter activity	MF	-0.204132095	-0.051330039	0.096105151	3.162457527	0.486088584	0.921936886	0.959149956 PREDICTED: partitioning defective protein 6
RS007128	GO:0022804	active transmembrane transporter activity	MF	0.030616457	-0.137872106	-0.048843691	6.138938535	0.365078568	0.947358098	0.972178127 PREDICTED: probable multidrug resistance-associated protein lethal(2)03659
RS007404	GO:0022804	active transmembrane transporter activity	MF	0.341548622	0.423696565	0.486833568	3.079113058	0.267214539	0.9660718	0.982215891 PREDICTED: sodium-independent sulfate anion transporter-like
RS008706	GO:0022804	active transmembrane transporter activity	MF	0.063443874	0.058144534	-0.01579188	5.633533699	0.196321115	0.978180999	0.988383465 PREDICTED: ATP-binding cassette sub-family D member 3
RS002038	GO:0005509	calcium ion binding	MF	-0.135176655	0.16006179	3.844560167	8.604326986	176.5862288	4.81554E-38	2.71959E-36 PREDICTED: fibrillin-2
RS013327	GO:0005509	calcium ion binding	MF	4.160581503	4.46067403	1.925626339	4.485054186	152.472056	7.71738E-33	3.62659E-31 PREDICTED: latent-transforming growth factor beta-binding protein 4
RS001172	GO:0005509	calcium ion binding	MF	0.36961837	4.03338728	-3.985714045	3.196112011	144.9618676	3.21709E-31	1.3971E-29 PREDICTED: cadherin-89D
RS006854	GO:0005509	calcium ion binding	MF	6.283120461	6.552275357	-0.537889305	5.75451878	138.70125	7.20252E-30	2.98383E-28 AAEL007579-PA
RS002686	GO:0005509	calcium ion binding	MF	2.763811404	2.611957119	-0.723707642	4.513758678	135.3970454	3.71383E-29	1.48013E-27 PREDICTED: micronuclear linker histone polyprotein
RS011123	GO:0005509	calcium ion binding	MF	-0.064367361	6.039195805	1.496286249	1.119543417	111.0087632	6.6563E-24	2.16613E-22 PREDICTED: phospholipase A2
RS014350	GO:0005509	calcium ion binding	MF	0.394221017	3.916656328	2.472104182	3.250648745	110.2945537	9.48297E-24	3.05444E-22 Matrix metalloproteinase-16
RS014619	GO:0005509	calcium ion binding	MF	0.135810517	1.948291035	0.695319866	6.46161189	96.1170343	1.06229E-20	2.83441E-19 PREDICTED: dual oxidase
RS002765	GO:0005509	calcium ion binding	MF	-0.765336453	0.724847601	1.654824096	4.938944897	88.44014686	4.73763E-19	1.12154E-17 PREDICTED: EF-hand domain-containing protein D2 homolog
RS012649	GO:0005509	calcium ion binding	MF	3.182916055	4.392257166	2.420456386	9.066174684	75.07046164	3.49949E-16	6.63788E-15 PREDICTED: troponin C
RS014237	GO:0005509	calcium ion binding	MF	-1.358203588	-0.858052145	0.808855596	6.87948729	73.79033318	6.58177E-16	1.22983E-14 PREDICTED: calcium-binding mitochondrial carrier protein Aralar1
RS009152	GO:0005509	calcium ion binding	MF	-0.076188961	2.318086608	-0.327763265	4.849879578	67.57555546	1.4102E-14	2.35857E-13 PREDICTED: alpha-(1,6)-fucosyltransferase
RS002823	GO:0005509	calcium ion binding	MF	-1.548255239	-2.66012337	-0.792696835	5.627365329	66.20568156	2.76964E-14	4.55959E-13 PREDICTED: regucalcin-like
RS002252	GO:0005509	calcium ion binding	MF	-0.958466976	0.563257376	2.066826911	6.610726046	61.18665646	3.27858E-13	4.9928E-12 PREDICTED: plastin-2
RS004283	GO:0005509	calcium ion binding	MF	-2.440750595	1.709249571	1.880477321	8.115587586	59.35367847	8.07824E-13	1.18991E-11 PREDICTED: uncharacterized threonine-rich GPI-anchored glycoprotein PJ4664.02
RS013624	GO:0005509	calcium ion binding	MF	0.161051998	0.020855506	1.593432636	5.172488776	58.39327821	1.2955E-12	1.87535E-11 PREDICTED: troponin C-like
RS001616	GO:0005509	calcium ion binding	MF	1.443216192	2.20981117	1.84746894	6.073310642	54.13960898	1.04771E-11	1.41122E-10 PREDICTED: cadherin-23
RS007103	GO:0005509	calcium ion binding	MF	-0.382427879	1.993835631	2.235797012	4.361167724	50.16865111	7.35502E-11	9.24442E-10 PREDICTED: matrix metalloproteinase-14
RS005920	GO:0005509	calcium ion binding	MF	-0.313607163	-0.086575041	1.086721692	6.422782192	49.20345088	1.18064E-10	1.45917E-09 PREDICTED: calpain-A
RS009824	GO:0005509	calcium ion binding	MF	-0.817741214	-2.218066115	-1.013010224	7.221999497	47.2744302	3.03845E-10	3.62006E-09 PREDICTED: calcyphosin-like protein
RS010869	GO:0005509	calcium ion binding	MF	-0.29920756	1.897410696	2.275660728	9.102894538	45.56697794	7.01063E-10	8.11506E-09 PREDICTED: FK506-binding protein 2
RS002764	GO:0005509	calcium ion binding	MF	-1.247613913	0.37235859	1.685412582	2.524591073	44.41494333	1.23194E-09	1.38899E-08 PREDICTED: EF-hand domain-containing protein D2 homolog
RS008843	GO:0005509	calcium ion binding	MF	-0.556368473	1.303284851	1.28365863	7.598206543	43.52398372	1.90479E-09	2.10799E-08 PREDICTED: sushi, von Willebrand factor type A, EGF and pentraxin domain-containing protein 1
RS003050	GO:0005509	calcium ion binding	MF	-0.547628467	1.078731713	0.376015467	5.671667378	43.14241527	2.29548E-09	2.52925E-08 PREDICTED: NADPH oxidase 5
RS009124	GO:0005509	calcium ion binding	MF	-1.567723088	2.440604203	1.276918035	3.164152789	42.50261207	3.13836E-09	3.42918E-08 PREDICTED: regucalcin-like
RS009947	GO:0005509	calcium ion binding	MF	-0.133867435	1.065636208	0.708920051	6.792021792	39.18991216	1.58209E-08	1.60039E-07 PREDICTED: calcium-binding mitochondrial carrier protein SCaMC-2
RS005268	GO:0005509	calcium ion binding	MF	-0.288027268	1.114580813	0.085981016	4.939687807	36.38002468	6.22343E-08	5.89308E-07 PREDICTED: 1-acylglycerophosphocholine O-acyltransferase 1
RS003180	GO:0005509	calcium ion binding	MF	1.000477947	2.77733509	1.561957152	5.407064332	36.37762793	6.2307E-08	5.89553E-07 63 kDa sperm flagellar membrane protein
RS004294	GO:0005509	calcium ion binding	MF	0.312111868	1.509915339	1.465815481	8.689460038	36.20284866	6.78418E-08	6.4E-07 PREDICTED: myosin-2 essential light chain
RS013651	GO:0005509	calcium ion binding	MF	0.446310293	1.960840406	0.988148147	3.363050399	35.82012059	8.17366E-08	7.65346E-07 PREDICTED: protocadherin Fat 4
RS012862	GO:0005509	calcium ion binding	MF	-0.607441674	-0.230065514	1.014673933	5.194678981	35.47494967	9.66887E-08	8.9867E-07 PREDICTED: fibrillin-2-like
RS006848	GO:0005509	calcium ion binding	MF	-0.66754309	0.9581648	1.223015946	5.733467437	35.19938959	1.10563E-07	1.02159E-06 PREDICTED: protocadherin-15
RS008217	GO:0005509	calcium ion binding	MF	-0.058063532	0.427477589	-1.164768064	4.782352072	34.91230534	1.27134E-07	1.16531E-06 PREDICTED: calcium-binding protein E63-1
RS008323	GO:0005509	calcium ion binding	MF	0.172300495	1.684288353	1.434517397	6.128767325	33.74577634	2.24176E-07	2.00374E-06 PREDICTED: ras and EF-hand domain-containing protein
RS014192	GO:0005509	calcium ion binding	MF	0.688964938	1.567107088	0.870440333	7.888499222	31.51414601	6.62462E-07	5.64482E-06 PREDICTED: myosin regulatory light chain sqh
RS002051	GO:0005509	calcium ion binding	MF	-1.109884166	-1.180423566	0.174832998	4.532750487	30.95485985	8.68856E-07	7.30953E-06 PREDICTED: troponin C
RS002822	GO:0005509	calcium ion binding	MF	-1.231023642	-1.360014482	-0.623882637	5.860607647	29.29477914	1.94172E-06	1.56056E-05 PREDICTED: regucalcin-like
RS002843	GO:0005509	calcium ion binding	MF	-0.892147693	0.161552464	1.23538029	5.890487207	29.08355836	2.15069E-06	1.71321E-05 hypothetical protein
RS007069	GO:0005509	calcium ion binding	MF	-1.090693094	-0.787452036	0.487574628	8.688494739	28.79647484	2.47115E-06	1.95721E-05 PREDICTED: gelsolin
RS011280	GO:0005509	calcium ion binding	MF	1.947610095	1.207768892	1.049882235	7.584136496	28.26208982	3.19986E-06	2.49684E-05 PREDICTED: bone morphogenetic protein 1
RS011754	GO:0005509	calcium ion binding	MF	-1.514433262	-1.507265295	0.844105975	11.28921924	26.6976021	6.81246E-06	5.0409E-05 PREDICTED: troponin C
RS007951	GO:0005509	calcium ion binding	MF	-0.94620471	-0.331607953	0.404848952	7.299643225	25.87913236	1.01096E-05	7.31573E-05 PREDICTED: very low-density lipoprotein receptor-like
RS008831	GO:0005509	calcium ion binding	MF	-0.967601819	-0.861587646	-0.143784827	6.787953208	25.4758244	1.22784E-05	8.786E-05 PREDICTED: calcineurin B homologous protein 1
RS012861	GO:0005509	calcium ion binding	MF	-0.283853037	0.348133884	1.245676432	7.267077838	25.19074564	1.40856E-05	9.94358E-05 PREDICTED: fibrillin-2-like
RS003289	GO:0005509	calcium ion binding	MF	-0.057033187	1.109096523	1.821536007	10.18934077	24.08579597	2.39706E-05	0.000163182 Fibrillin-2
RS011207	GO:0005509	calcium ion binding	MF	0.526909114	0.672637237	1.070322125	5.664986164	23.1408246	3.77435E-05	0.000248475 PREDICTED: group XIIA secretory phospholipase A2
RS005522	GO:0005509	calcium ion binding	MF	-0.838298435	-1.309181794	0.022916046	7.425604668	21.35374419	8.88876E-05	0.000547141 PREDICTED: cartilage oligomeric matrix protein
RS002951	GO:0005509	calcium ion binding	MF	-0.713484452	-1.159017956	-0.981253249	5.089094554	19.9574752	0.000173222	0.001005326 PREDICTED: dual oxidase 2-like
RS011299	GO:0005509	calcium ion binding	MF	-0.764016582	-0.593212665	0.49722129	4.442221584	18.32142123	0.00037756	0.002024261 PREDICTED: cadherin-86C
RS012501	GO:0005509	calcium ion binding	MF	-1.250093155	-1.758275813	-0.217147236	3.060515632	18.24989505	0.000390615	0.002083609 PREDICTED: collagen and calcium-binding EGF domain-containing protein 1
RS013623	GO:0005509	calcium ion binding	MF	-0.572979083	-1.747015153	-0.730223745	3.430703168	18.10154502	0.00041914	0.002223523 PREDICTED: neo-calmodulin-like
RS012992	GO:0005509	calcium ion binding	MF	0.136275224	-0.863373278	-0.62196395	5.387271514	17.98229805	0.000443563	0.0023442 PREDICTED: hemicentin-1
RS009810	GO:0005509	calcium ion binding	MF	-1.393669765	-0.799726174	-0.408687427	4.462416695	17.62535716	0.000525451	0.002735646 PREDICTED: 1-phosphatidylinositol 4,5-bisphosphate phosphodiesterase delta-4
RS014628	GO:0005509	calcium ion binding	MF	-0.742367417	-0.611313702	0.354118192	3.683582866	17.44515899	0.000572334	0.002954086 PREDICTED: calcium/calmodulin-dependent protein kinase kinase 1-like
RS008703	GO:0005509	calcium ion binding	MF	0.891413178	0.410697322	-0.681554837	9.477370831	17.33816384	0.000602115	0.003082533 PREDICTED: calreticulin
RS013191	GO:0005509	calcium ion binding	MF	0.105641406	-0.473422065	-1.100786682	4.568604849	17.21352583	0.000638753	0.003251735 PREDICTED: low-density lipoprotein receptor-related protein 4
RS014615	GO:0005509	calcium ion binding	MF	0.884888219	1.632601496	1.287263146	2.513002334	16.71847579	0.000807487	0.004037115 PREDICTED: protein rhomboid
RS007321	GO:0005509	calcium ion binding	MF	-0.380491989	-0.68257877	0.228975511	6.841823405	16.64682652	0.000835328	0.004164735 PREDICTED: annexin B10
RS003625	GO:0005509	calcium ion binding	MF	-0.919867298	-0.750704213	-0.704835111	6.933958906	16.40972231	0.000934438	0.004607796 PREDICTED: synaptotagmin 1
RS013853	GO:0005509	calcium ion binding	MF	0.846391908	0.326861642	-0.16506872	5.581154157	16.3966953	0.00094021	0.004632629 PREDICTED: mesencephalic astrocyte-derived neurotrophic factor homolog
RS013858	GO:0005509	calcium ion binding	MF	-0.798801991	0.078731809	1.485884039	12.69321155	15.89635178	0.001190839	0.005698111 PREDICTED: myosin light chain alkali
RS012953	GO:0005509	calcium ion binding	MF	0.589515386	1.345194903	0.529815275	3.658878845	15.85311415	0.001215385	0.005800136 PREDICTED: neuronal calcium sensor 2
RS014120	GO:0005509	calcium ion binding	MF	-0.922827123	-1.289252361	-1.238323899	2.633512953	15.71916541	0.001294661	0.006118183 PREDICTED: nicotinamidase
RS012386	GO:0005509	calcium ion binding	MF	-1.113494458	-1.408132111	-1.035003478	4.276380866	15.3721214	0.00152474	0.007059771 PREDICTED: calbindin-32
RS011576	GO:0005509	calcium ion binding	MF	-0.975652066	0.542681057	0.262513159	6.233797955	14.98369804	0.001830633	0.008272334 PREDICTED: cadherin-23
RS002071	GO:0005509	calcium ion binding	MF	-0.71762685	-0.888387207	-0.958293408	7.066000838	14.81436821	0.001982356	0.008871994 PREDICTED: neural-cadherin
RS008874	GO:0005509	calcium ion binding	MF	-1.206434787	-0.289083994	1.260310342	13.62975944	14.75148622	0.002041821	0.009096105 PREDICTED: myosin regulatory light chain 2
RS002824	GO:0005509	calcium ion binding	MF	-1.122471703	-0.764017572	-0.34587481	3.692264967	14.42695662	0.002378007	0.01039161 PREDICTED: regucalcin-like
RS011379	GO:0005509	calcium ion binding	MF	-0.945895165	-0.137739549	0.02413553	6.197611827	14.11352967	0.002754622	0.01177587 PREDICTED: chloride intracellular channel exc-4
RS011122	GO:0005509	calcium ion binding	MF	0.082189494	1.196958457	0.74482172	3.346133987	14.0443711	0.002845374	0.012090866 PREDICTED: phospholipase A2
RS000190	GO:0005509	calcium ion binding	MF	-0.155501564	-0.563676059	-0.693571659	6.889722774	13.74883694	0.00326775	0.01368153 PREDICTED: nuclear pore complex protein Nup214
RS013446	GO:0005509	calcium ion binding	MF	-0.668960475	-0.754766011	-0.82958789	5.394285007	13.6909099	0.003357544	0.014006262 PREDICTED: calexcitin-2

RS014066	GO:0005509	calcium ion binding	MF	-0.926645409	-0.838433517	-0.530800089	6.139995747	12.72909668	0.005260626	0.020581647	hypothetical protein
RS010671	GO:0005509	calcium ion binding	MF	-0.365955325	-0.969670493	-0.732597595	7.955985544	12.52155768	0.005794254	0.022377442	PREDICTED: prolow-density lipoprotein receptor-related protein 1
RS010964	GO:0005509	calcium ion binding	MF	-1.004995812	-0.588637144	-0.424145768	8.038347207	11.94194578	0.007584699	0.028169182	PREDICTED: low-density lipoprotein receptor-related protein 2
RS001952	GO:0005509	calcium ion binding	MF	-0.796459757	-0.904276224	-1.349830192	3.620084555	11.65393196	0.008667741	0.03154046	PREDICTED: delta and Notch-like epidermal growth factor-related receptor
RS015567	GO:0005509	calcium ion binding	MF	0.376413076	-0.071504162	-0.448105985	7.311814141	11.60916555	0.008849263	0.032126729	PREDICTED: glucosidase 2 subunit beta
RS001244	GO:0005509	calcium ion binding	MF	-1.333168723	-2.553229897	-1.478513338	1.009183264	11.58665291	0.008941962	0.032404011	PREDICTED: EF-hand calcium-binding domain-containing protein 1
RS002072	GO:0005509	calcium ion binding	MF	-0.870937442	-1.078603815	-1.323351854	4.112820184	11.44381857	0.009552838	0.034126805	PREDICTED: neural-cadherin
RS003739	GO:0005509	calcium ion binding	MF	-0.712942094	-0.798417023	-1.059321241	4.256625193	11.39223286	0.009783434	0.034864904	PREDICTED: follistatin-related protein 5-like
RS002180	GO:0005509	calcium ion binding	MF	-1.072077009	-0.499365966	-1.04699579	3.109457407	11.32512516	0.010091652	0.035781042	PREDICTED: serine/threonine-protein phosphatase rdgC
RS005914	GO:0005509	calcium ion binding	MF	0.479962664	-0.053327376	-0.164381233	6.215797354	10.63133139	0.01389596	0.047107866	PREDICTED: cleavage and polyadenylation specificity factor subunit 1
RS009608	GO:0005509	calcium ion binding	MF	-0.650681402	-0.131877234	-0.002301287	6.339415917	9.831393766	0.020055087	0.063636624	PREDICTED: V-type proton ATPase subunit C
RS003051	GO:0005509	calcium ion binding	MF	-0.064961478	1.836288981	0.63869261	0.362805235	9.79580548	0.020384044	0.064428604	PREDICTED: NADPH oxidase 5
RS009463	GO:0005509	calcium ion binding	MF	0.695529538	0.361666046	0.249123277	7.193603156	9.33435209	0.025160467	0.076631419	PREDICTED: cysteine-rich with EGF-like domain protein 2
RS008567	GO:0005509	calcium ion binding	MF	-0.395964157	0.055050505	0.329555756	6.356343245	9.231251867	0.026369147	0.079448096	PREDICTED: glycerol-3-phosphate dehydrogenase, mitochondrial
RS004600	GO:0005509	calcium ion binding	MF	0.255072008	0.08235856	-1.653097913	0.906918497	9.114929342	0.027801228	0.082909938	Cadherin-related tumor suppressor
RS008214	GO:0005509	calcium ion binding	MF	-0.591657749	-0.60801793	-0.342781866	5.622477097	8.89592842	0.030707059	0.089873274	PREDICTED: sodium channel protein paralytic
RS011670	GO:0005509	calcium ion binding	MF	-0.500688314	-0.906644814	-0.001083981	3.985350954	8.857204244	0.031250932	0.091231859	PREDICTED: protein slit
RS009065	GO:0005509	calcium ion binding	MF	-0.795865105	-0.041756702	-0.175358405	3.718429062	8.855541505	0.031274495	0.091271489	PREDICTED: calcyphosin-like protein
RS015046	GO:0005509	calcium ion binding	MF	-0.280790113	-0.813988496	-1.105079288	4.0892248	8.854403082	0.031290638	0.091284292	PREDICTED: pikachurin
RS006943	GO:0005509	calcium ion binding	MF	-0.745844928	-0.858790002	-0.719751741	4.133949221	8.813227185	0.031880016	0.092788751	PREDICTED: diacylglycerol kinase 1
RS000001	GO:0005509	calcium ion binding	MF	-0.923753843	-0.774146522	-0.309728113	8.964042611	8.801674733	0.032047317	0.093060619	PREDICTED: ryanodine receptor 44F
RS005260	GO:0005509	calcium ion binding	MF	-0.401439177	0.192974821	0.345669705	7.152876139	8.707442589	0.03344442	0.096409171	PREDICTED: cadherin-87A
RS007992	GO:0005509	calcium ion binding	MF	-0.683085858	-0.393016463	-0.296418872	5.853329436	8.335060107	0.039571535	0.109892815	PREDICTED: fat-like cadherin-related tumor suppressor homolog
RS014206	GO:0005509	calcium ion binding	MF	-0.331060594	-0.319039029	0.770529033	2.915958338	8.315762111	0.039917305	0.110682196	PREDICTED: mannosyl-oligosaccharide 1,2-alpha-mannosidase IA
RS009278	GO:0005509	calcium ion binding	MF	0.57242159	0.753774941	0.32276576	5.958966852	8.263206677	0.040873974	0.112739123	PREDICTED: multiple coagulation factor deficiency protein 2 homolog
RS002073	GO:0005509	calcium ion binding	MF	-0.730372331	-0.653911276	-1.441098065	2.248321382	8.194678957	0.042155042	0.115463374	PREDICTED: neural-cadherin-like
RS011310	GO:0005509	calcium ion binding	MF	0.512463955	0.266450018	-0.590116921	4.721320355	7.903303067	0.048052989	0.128308919	PREDICTED: protocadherin-like wing polarity protein stan
RS012827	GO:0005509	calcium ion binding	MF	0.270811737	0.00479393	-0.664455338	6.443908707	7.879526255	0.04856827	0.129535957	PREDICTED: calnexin
RS012798	GO:0005509	calcium ion binding	MF	-0.604233235	-0.423742906	-0.721708768	4.952598734	7.599872966	0.055047062	0.143088276	PREDICTED: annexin B9
RS003764	GO:0005509	calcium ion binding	MF	-1.154969853	-0.552489744	-0.181870194	2.19921009	7.540167043	0.056535321	0.146030227	PREDICTED: 1-phosphatidylinositol 4,5-bisphosphate phosphodiesterase classes I and II
RS005737	GO:0005509	calcium ion binding	MF	0.302243019	-0.40559564	-0.905264064	4.115692831	7.398482299	0.060225059	0.153382081	PREDICTED: protein jagged-1
RS008996	GO:0005509	calcium ion binding	MF	-0.818894069	-0.144607229	0.175883673	2.981815036	7.237819263	0.064691824	0.161620478	PREDICTED: cytochrome c oxidase assembly factor 6 homolog
RS003427	GO:0005509	calcium ion binding	MF	-0.513964722	-0.550228674	-0.09809219	5.664872118	6.801016027	0.078517892	0.186983778	PREDICTED: voltage-dependent calcium channel type A subunit alpha-1
RS002039	GO:0005509	calcium ion binding	MF	0.248080734	0.662785639	0.647591418	4.279003846	6.545725772	0.087876531	0.204304418	PREDICTED: fibrillin-2
RS011010	GO:0005509	calcium ion binding	MF	-0.339378177	0.263861661	0.201713494	6.807371148	6.414441659	0.093098476	0.213489113	PREDICTED: rab11 family-interacting protein 4
RS007947	GO:0005509	calcium ion binding	MF	-0.758574283	-0.215476913	-0.300311328	5.94500423	6.345524122	0.095957831	0.217838484	PREDICTED: protein crumbs
RS013850	GO:0005509	calcium ion binding	MF	0.226155429	-0.16034169	-0.376830354	4.968715242	6.079013068	0.107828712	0.237100249	PREDICTED: cubilin-like
RS004797	GO:0005509	calcium ion binding	MF	0.459004562	-0.000986695	-0.496189284	4.718991441	5.504275332	0.138383129	0.284445426	PREDICTED: dystroglycan-like
RS014794	GO:0005509	calcium ion binding	MF	0.684288907	0.57343907	0.452752305	7.409120301	5.463876404	0.140815244	0.287847294	PREDICTED: serine/threonine-protein phosphatase 2A regulatory subunit B'' subunit gamma-like
RS010809	GO:0005509	calcium ion binding	MF	-0.207521915	0.236579594	0.549695454	4.02997305	5.457083026	0.14122818	0.288504088	PREDICTED: matrix metalloproteinase-14
RS007980	GO:0005509	calcium ion binding	MF	0.197066924	0.488438041	0.322953674	6.235166424	5.432059662	0.142759147	0.290923737	PREDICTED: centrin-2
RS006238	GO:0005509	calcium ion binding	MF	0.238584558	-0.04397443	-0.231491456	5.794637148	5.284717858	0.152097106	0.304823512	PREDICTED: mitochondrial Rho GTPase
RS006643	GO:0005509	calcium ion binding	MF	-0.474901931	-0.524926376	0.235758919	3.614518101	5.066753162	0.166973901	0.326431126	PREDICTED: multiple epidermal growth factor-like domains protein 8
RS014216	GO:0005509	calcium ion binding	MF	-0.410521539	-0.222608491	-0.785892482	7.084822935	5.060121692	0.167447305	0.327255021	PREDICTED: testican-1
RS014862	GO:0005509	calcium ion binding	MF	-0.717021268	-0.337097413	0.432780493	11.96886137	5.0593915	0.167499508	0.327306254	PREDICTED: alpha-actinin, sarcomeric
RS006240	GO:0005509	calcium ion binding	MF	-0.456558706	-0.421545107	-0.731005218	5.012200364	4.908981407	0.178584593	0.342327908	PREDICTED: annexin B9-like
RS010810	GO:0005509	calcium ion binding	MF	-0.110840312	0.018908635	0.437248026	4.07693735	4.870375311	0.181538723	0.34667152	PREDICTED: matrix metalloproteinase-14
RS007991	GO:0005509	calcium ion binding	MF	-0.359355689	0.17088743	0.136788825	4.569710428	4.81489702	0.185864026	0.351625588	PREDICTED: fat-like cadherin-related tumor suppressor homolog
RS001535	GO:0005509	calcium ion binding	MF	0.062265296	-0.265689108	-0.507984727	4.762472418	4.780932374	0.188559358	0.355219028	PREDICTED: vitellogenin receptor
RS005058	GO:0005509	calcium ion binding	MF	0.353228534	0.485228455	0.31705476	5.309859263	4.617278076	0.202064882	0.372151441	PREDICTED: soluble calcium-activated nucleotidase 1
RS011922	GO:0005509	calcium ion binding	MF	-0.649385456	-0.169072995	-1.344964409	1.159816594	4.617457825	0.202049567	0.372151441	PREDICTED: protein eyes shut
RS005244	GO:0005509	calcium ion binding	MF	-0.065249759	0.38909471	-0.560413614	7.914524942	4.557440342	0.207223609	0.378541045	PREDICTED: SPARC
RS001215	GO:0005509	calcium ion binding	MF	-0.248316136	0.178767481	0.203779096	5.031686558	4.377029249	0.223524784	0.397426505	PREDICTED: protein naked cuticle
RS010861	GO:0005509	calcium ion binding	MF	-0.201147579	-0.03157159	0.468079894	3.756400194	4.352990241	0.225784067	0.399764452	PREDICTED: Kv channel-interacting protein 1
RS009584	GO:0005509	calcium ion binding	MF	-0.627940175	-0.676833081	-0.684356289	7.202114236	4.300158609	0.230823465	0.40549459	PREDICTED: calsyntenin-1
RS015158	GO:0005509	calcium ion binding	MF	-0.221959913	0.017696906	0.233149674	6.687630933	4.297117886	0.231116631	0.405839774	PREDICTED: calcineurin subunit B type 2
RS005592	GO:0005509	calcium ion binding	MF	-0.408049599	-0.607376572	-0.512216376	6.303486981	4.102236304	0.250633977	0.429713316	PREDICTED: SPARC-related modular calcium-binding protein 2
RS008636	GO:0005509	calcium ion binding	MF	-0.316881817	-0.44388272	-0.528476252	4.300550223	3.877802288	0.274965196	0.457040085	PREDICTED: protein kinase C-binding protein NELL1-like
RS009555	GO:0005509	calcium ion binding	MF	-0.103052035	-0.259879899	-0.587944698	3.90503452	3.786490644	0.285461411	0.46836187	PREDICTED: protein FAM188A homolog
RS002065	GO:0005509	calcium ion binding	MF	-0.252868211	-0.310629676	-0.472782655	4.95188401	3.691650518	0.296743023	0.479802495	PREDICTED: reticulocalbin-2
RS014057	GO:0005509	calcium ion binding	MF	-0.39000049	-0.477575822	0.129544323	3.466363476	3.631517015	0.304101102	0.486886509	PREDICTED: EF-hand calcium-binding domain-containing protein 4A
RS014483	GO:0005509	calcium ion binding	MF	-0.307179688	-0.359551596	-0.333262969	5.784863588	3.609903734	0.306785217	0.489379738	PREDICTED: TBC1 domain family member 9
RS007993	GO:0005509	calcium ion binding	MF	-0.386644495	-0.062883345	0.393404769	2.263885577	3.458009684	0.326249098	0.508260903	PREDICTED: fat-like cadherin-related tumor suppressor homolog
RS006561	GO:0005509	calcium ion binding	MF	0.353995304	0.237572917	0.419480704	5.635873753	3.395244005	0.334604917	0.516387054	PREDICTED: EH domain-containing protein 3
RS004489	GO:0005509	calcium ion binding	MF	-0.49287906	-0.290129923	-0.514718357	3.478984221	3.356184306	0.339899076	0.521573591	PREDICTED: 1-phosphatidylinositol 4,5-bisphosphate phosphodiesterase gamma-1
RS001866	GO:0005509	calcium ion binding	MF	0.040123917	-0.623017482	-0.214094236	5.514447489	3.354957946	0.340066477	0.521596292	PREDICTED: cadherin-related tumor suppressor
RS009407	GO:0005509	calcium ion binding	MF	-0.055373153	-0.327300962	-0.377162054	4.676573478	3.267656302	0.352169714	0.532695818	PREDICTED: ras guanyl-releasing protein 3
RS014840	GO:0005509	calcium ion binding	MF	-0.175383987	-0.325833617	-0.325229019	5.697533573	3.167201054	0.36655751	0.547897912	PREDICTED: 1-phosphatidylinositol 4,5-bisphosphate phosphodiesterase-like
RS010211	GO:0005509	calcium ion binding	MF	1.071391991	0.905933246	0.557883471	0.690909896	3.137344567	0.370930257	0.551469208	PREDICTED: frequenin-1
RS000548	GO:0005509	calcium ion binding	MF	0.187638816	0.214437515	-0.148122314	6.348047805	3.071771925	0.380691189	0.560358769	PREDICTED: calumenin
RS015048	GO:0005509	calcium ion binding	MF	-0.740243311	-0.374333487	-0.470195995	10.45326798	3.0714738	0.380736064	0.560358769	PREDICTED: microtubule-actin cross-linking factor 1
RS014834	GO:0005509	calcium ion binding	MF	-0.392314146	-0.205624105	-0.156066244	5.709038925	2.984948528	0.393951643	0.571926215	PREDICTED: alpha-sarcoglycan
RS000892	GO:0005509	calcium ion binding	MF	0.338138191	0.424482879	0.167447565	4.601167068	2.953865432	0.398793051	0.576108366	PREDICTED: calcium and integrin-binding family member 2
RS005563	GO:0005509	calcium ion binding	MF	-0.287333382	-0.127875056	0.182185235	4.579647806	2.875984629	0.411143752	0.587334893	PREDICTED: E3 ubiquitin-protein ligase CBL-B
RS006563	GO:0005509	calcium ion binding	MF	-0.489174516	-0.417725507	-0.674213578	3.687949172	2.85405574	0.41467851	0.590575728	PREDICTED: EH domain-containing protein 3
RS005488	GO:0005509	calcium ion binding	MF	-0.358914371	0.284662883	-0.146435242	7.242662048	2.822318182	0.419839113	0.595232893	PREDICTED: basement membrane-specific heparan sulfate proteoglycan core protein
RS013398	GO:0005509	calcium ion binding	MF	0.846055402	1.118274394	0.139393517	0.96384649	2.762996912	0.429627563	0.604010887	PREDICTED: EF-hand calcium-binding domain-containing protein 2
RS013346	GO:0005509	calcium ion binding	MF	-0.095021443	-0.55837102	-0.473417441	6.796225014	2.749473682	0.431885126	0.605764704	PREDICTED: neurogenic locus Notch protein
RS010806	GO:0005509	calcium ion binding	MF	0.417731943	-0.214862196	0.112210512	3.575135255	2.731397887	0.434917896	0.608527495	PREDICTED: matrix metalloproteinase-14
RS011707	GO:0005509	calcium ion binding	MF	0.074263254	-0.295263017	-0.262038316	6.555914748	2.679327961	0.443751729	0.617492952	PREDICTED: calmodulin
RS003728	GO:0005509	calcium ion binding	MF	0.114317509	0.329043383	0.441655586	4.430613617	2.492072946	0.476725383	0.646533906	PREDICTED: calmodulin-like protein 4
RS013488	GO:0005509	calcium ion binding	MF	0.08417961	0.284993346	0.287565764	6.694944952	2.46777472	0.481143073	0.650302196	Serine/threonine-protein phosphatase 2A regulatory subunit B'' subunit alpha
RS007283	GO:0005509	calcium ion binding	MF	0.385879704	-0.017402138	-0.066148604	6.650502341	2.456985058	0.483115026	0.65184815	PREDICTED: nidogen-2
RS009282	GO:0005509	calcium ion binding	MF	-0.523115983	-0.205799461	-0.290286585	9.304493683	2.454208505	0.4836235	0.652349219	PREDICTED: spectrin alpha chain
RS014448	GO:0005509	calcium ion binding	MF	0.063218695	0.360518634	0.2332296	4.966069177	2.442711039	0.485733499	0.653560651	PREDICTED: neurogenic locus protein delta
RS009954	GO:0005509	calcium ion binding	MF	-0.366071957	-0.447842359	-0.294150693	4.51045727	2.422804257	0.489403735	0.65688486	PREDICTED: ubiquitin carboxyl-terminal hydrolase 32
RS006756	GO:0005509	calcium ion binding	MF	0.137422387	0.134324258	-0.234788686	7.596886249	2.378954156	0.497564323	0.663524469	PREDICTED: nucleobindin-2
RS009234	GO:0005509	calcium ion binding	MF	0.126988243	0.342457159	0.395519048	7.837797102	2.3304594	0.506710822	0.67032732	PREDICTED: putative RNA-binding protein Luc7-like 2
RS001131	GO:0005509	calcium ion binding	MF	-0.46672796	-0.438731687	-0.485486742	3.00696323	2.28306329	0.515773294	0.678325905	PREDICTED: two pore calcium channel protein 1-like
RS004896	GO:0005509	calcium ion binding	MF	0.258725126	0.153243084	0.316020819	6.888961678	2.281927063	0.515992041	0.678432466	PREDICTED: ER degradation-enhancing alpha-mannosidase-like protein 3
RS007841	GO:0005509	calcium ion binding	MF	0.208183302	0.20903907	-0.039573047	6.104963809	2.229226125	0.526214701	0.686324352	PREDICTED: ninein-like protein
RS003240	GO:0005509	calcium ion binding	MF	0.255011601	0.2449423	0.024189273	7.260073137	2.220353778	0.527950447	0.687888957	PREDICTED: ER degradation-enhancing alpha-mannosidase-like protein 2
RS006678	GO:0005509	calcium ion binding	MF	0.139354119	0.346144069	0.216283711	4.801909094	2.111453968	0.549599025	0.704135313	PREDICTED: calcium and integrin-binding protein 1-like
RS011544	GO:0005509	calcium ion binding	MF	0.377175568	-0.064832165	0.03084746	3.098145541	1.902260953	0.592938469	0.738768014	PREDICTED: endoplasmic reticulum mannosyl-oligosaccharide 1,2-alpha-mannosidase
RS012954	GO:0005509	calcium ion binding	MF	-0.176644427	-0.317583234	-0.156924207	5.388438607	1.862359781	0.601459661	0.746080227	PREDICTED: neurocalcin homolog
RS010807	GO:0005509	calcium ion binding	MF	0.001269449	-0.509367404	-0.252962804	2.656086521	1.8581118	0.602371528	0.746826839	PREDICTED: matrix metalloproteinase-14
RS009143	GO:0005509	calcium ion binding	MF	-0.592281409	-0.46079711	-0.228264881	2.777417387	1.856987004	0.602613126	0.746979302	PREDICTED: colorectal mutant cancer protein
RS007865	GO:0005509	calcium ion binding	MF	0.189614824	-0.107146276	0.466934837	3.905162157	1.731854836	0.629875113	0.767457844	PREDICTED: protein dachsous
RS010808	GO:0005509	calcium ion binding	MF	-0.222515765	0.181347069	-0.09862163	2.934325809	1.601644393	0.659017022	0.787657085	PREDICTED: matrix metalloproteinase-14
RS003212	GO:0005509	calcium ion binding	MF	0.052326316	0.082884624	-0.229696609	5.678727517	1.556360078	0.669324848	0.794334445	PREDICTED: peflin
RS008803	GO:0005509	calcium ion binding	MF	-0.077683173	-0.04308921	0.151856068	6.090281047	1.319131764	0.724595141	0.835214278	PREDICTED: LETM1 and EF-hand domain-containing protein anon-60Da, mitochondrial
RS011746	GO:0005509	calcium ion binding	MF	-0.39884548	-0.351698614	-0.321292158	2.434881592	1.267908853	0.736767171	0.843838282	PREDICTED: rabphilin-3A
RS013220	GO:0005509	calcium ion binding	MF	0.718575153	0.746584402	0.46567104	3.967403819	1.178460107	0.758174967	0.85813451	PREDICTED: polycystic kidney disease protein 1-like 2
RS003510	GO:0005509	calcium ion binding	MF	0.244621151	0.10160928	0.152536394	6.915704488	1.137607604	0.768004082	0.864905965	PREDICTED: transmembrane protein 14C
RS005015	GO:0005509	calcium ion binding	MF	0.218507771	0.083225809	0.27778338	5.630853004	1.117763329	0.772787768	0.86781	PREDICTED: sushi, von Willebrand factor type A, EGF and pentraxin domain-containing protein 1-like
RS006804	GO:0005509	calcium ion binding	MF	-0.106727555	-0.311113867	0.044117663	3.55950746	1.080563363	0.781768115	0.873920437	PREDICTED: Kv channel-interacting protein 4
RS003339	GO:0005509	calcium ion binding	MF	-0.157029432	-0.06369688	0.166726836	3.703953529	1.06484554	0.78556656	0.876299845	PREDICTED: epidermal growth factor receptor substrate 15-like 1
RS002160	GO:0005509	calcium ion binding	MF	-0.16847688	0.007004528	-0.250212606	5.629359875	1.037015493	0.792296295	0.88037582	PREDICTED: DE-cadherin
RS007512	GO:0005509	calcium ion binding	MF	-0.043134924	-0.166066137	0.132661348	3.705982437	1.035010932	0.792781187	0.880681509	PREDICTED: 45 kDa calcium-binding protein
RS007702	GO:0005509	calcium ion binding	MF	-0.207976473	0.196473721	0.085101443	3.379751314	1.033521878	0.793141391	0.880709569	PREDICTED: ER degradation-enhancing alpha-mannosidase-like protein 1
RS010187	GO:0005509	calcium ion binding	MF	0.109541571	0.279119563	0.217881347	4.191743664	1.014648008	0.797707631	0.883615901	PREDICTED: rhomboid-related protein 2

RS014637	GO:0005509	calcium ion binding	MF	0.066840247	0.600909401	0.396436471	2.051509931	1.008937941	0.799089251	0.884723519	PREDICTED: ER degradation-enhancing alpha-mannosidase-like protein 1
RS005232	GO:0005509	calcium ion binding	MF	-0.155378497	-0.177681158	-0.17511301	5.949209397	0.998838089	0.801533105	0.885725087	PREDICTED: intersectin-2
RS002685	GO:0005509	calcium ion binding	MF	-0.106623202	0.129132903	-0.002060714	5.277398632	0.946440961	0.814208493	0.893217924	PREDICTED: calcium uptake protein 3, mitochondrial
RS008931	GO:0005509	calcium ion binding	MF	-0.187945581	0.0019551	0.189486468	5.343501087	0.912902362	0.822313108	0.898722287	PREDICTED: annexin B9-like
RS006306	GO:0005509	calcium ion binding	MF	-0.149310168	-0.081536306	0.103685003	4.142573342	0.892516551	0.827233352	0.902241001	PREDICTED: agrin-like
RS005778	GO:0005509	calcium ion binding	MF	-0.021233673	-0.136204615	0.132446444	3.744812426	0.702271019	0.872669653	0.931784791	PREDICTED: ralBP1-associated Eps domain-containing protein 1
RS004375	GO:0005509	calcium ion binding	MF	-0.439923774	-0.123751868	-0.173141227	1.274835456	0.678853987	0.878166071	0.934326561	PREDICTED: Kv channel-interacting protein 1
RS014130	GO:0005509	calcium ion binding	MF	0.393138432	0.253437286	0.451794066	1.035306574	0.652814694	0.884242175	0.938003319	PREDICTED: ras guanyl-releasing protein 3
RS010462	GO:0005509	calcium ion binding	MF	-0.138850056	-0.003492612	0.016667127	6.764082567	0.6500062	0.884895093	0.938087548	PREDICTED: calcium uptake protein 1 homolog, mitochondrial
RS001706	GO:0005509	calcium ion binding	MF	0.033602523	0.158734434	-0.006749049	5.288609056	0.590995424	0.898490636	0.945803333	PREDICTED: programmed cell death protein 6
RS001867	GO:0005509	calcium ion binding	MF	-0.219015309	-0.312481311	-0.195553489	2.164090749	0.435081401	0.932903856	0.964850234	PREDICTED: cadherin-related tumor suppressor
RS014134	GO:0005509	calcium ion binding	MF	0.202412564	0.022912512	0.074608072	10.02891467	0.337859024	0.952757814	0.975354966	PREDICTED: calmodulin
RS001622	GO:0005509	calcium ion binding	MF	0.355796693	0.107830408	0.261374858	0.550161694	0.328136821	0.954652687	0.97588441	cadherin
RS007866	GO:0005509	calcium ion binding	MF	-0.123239502	0.339316842	0.191117175	0.229582542	0.305854213	0.958923727	0.977774901	PREDICTED: protein dachsous-like
RS000823	GO:0005509	calcium ion binding	MF	0.152634503	-0.141914787	0.094256861	4.451047718	0.281885013	0.963398621	0.980685599	PREDICTED: fibrillin-1-like
RS011718	GO:0022857	transmembrane transporter activity	MF	-0.059496373	8.716087878	3.96160781	4.311804415	501.7385592	2.004E-108	4.762E-106	PREDICTED: glucose dehydrogenase [FAD, quinone]
RS007652	GO:0022857	transmembrane transporter activity	MF	0.585883623	0.528505008	4.851177054	4.584889963	291.2117195	7.93792E-63	8.92591E-61	PREDICTED: solute carrier family 26 member 10-like
RS009299	GO:0022857	transmembrane transporter activity	MF	-0.833536193	5.147671256	1.07098716	3.418953047	224.4882423	2.15009E-48	1.67149E-46	PREDICTED: organic cation transporter protein-like
RS003905	GO:0022857	transmembrane transporter activity	MF	-1.535395853	-3.809880637	1.439384598	5.164045266	219.9356219	2.07317E-47	1.56345E-45	PREDICTED: facilitated trehalose transporter Tret1-like
RS009243	GO:0022857	transmembrane transporter activity	MF	-0.589487895	2.064901326	2.91752274	4.715025796	173.1275538	2.68806E-37	1.47832E-35	PREDICTED: multidrug resistance protein homolog 49
RS003129	GO:0022857	transmembrane transporter activity	MF	-1.935857211	0.013218622	3.698610157	4.556804627	145.8197805	2.10104E-31	9.2418E-30	PREDICTED: organic cation transporter protein
RS004606	GO:0022857	transmembrane transporter activity	MF	-0.152775887	3.199032258	1.582974114	7.379011967	144.4003621	4.25168E-31	1.83376E-29	PREDICTED: peptide transporter family 1
RS004125	GO:0022857	transmembrane transporter activity	MF	1.407227463	2.012417871	-0.423642507	5.906215214	131.7530272	2.26607E-28	8.8083E-27	PREDICTED: ABC transporter G family member 9
RS003228	GO:0022857	transmembrane transporter activity	MF	-0.561103954	2.12048168	1.83683218	5.406987214	114.6440514	1.09829E-24	3.71824E-23	PREDICTED: b(0,+)-type amino acid transporter 1-like
RS013974	GO:0022857	transmembrane transporter activity	MF	1.308057058	2.315023189	0.876327879	6.127768048	111.7248777	4.66769E-24	1.53887E-22	PREDICTED: monocarboxylate transporter 10
RS011780	GO:0022857	transmembrane transporter activity	MF	-1.685309235	-4.03663067	-0.682423211	4.573186577	105.6851221	9.30608E-23	2.78387E-21	PREDICTED: facilitated trehalose transporter Tret1-2 homolog
RS000720	GO:0022857	transmembrane transporter activity	MF	-1.414180497	1.484024302	-1.819002501	5.701350612	100.1058089	1.47484E-21	4.12758E-20	aquaporin
RS012045	GO:0022857	transmembrane transporter activity	MF	-2.417547613	-3.846942398	-2.948472949	3.138267013	95.12499238	1.73561E-20	4.58246E-19	PREDICTED: sodium-independent sulfate anion transporter
RS002200	GO:0022857	transmembrane transporter activity	MF	-2.534854462	-2.965618885	0.446701011	4.187537984	94.94596227	1.89639E-20	4.97566E-19	PREDICTED: ATP-binding cassette sub-family G member 4
RS009298	GO:0022857	transmembrane transporter activity	MF	-2.524665679	0.320369244	1.997080797	2.729083611	85.53693786	1.99021E-18	4.50803E-17	PREDICTED: organic cation transporter protein-like
RS009300	GO:0022857	transmembrane transporter activity	MF	-0.334428722	2.777656488	1.996146097	5.84981916	85.14092159	2.42051E-18	5.44355E-17	PREDICTED: organic cation transporter protein-like
RS014732	GO:0022857	transmembrane transporter activity	MF	-2.171879817	-1.24728548	0.09932889	5.367265273	82.18961662	1.04062E-17	2.21004E-16	PREDICTED: solute carrier organic anion transporter family member 2A1
RS015390	GO:0022857	transmembrane transporter activity	MF	-1.260588417	-0.88728058	1.174441311	5.643186003	81.54774944	1.42892E-17	3.01438E-16	PREDICTED: sodium-independent sulfate anion transporter
RS014579	GO:0022857	transmembrane transporter activity	MF	-1.833927302	-1.637670215	-2.03241566	4.946947748	80.73878787	2.13088E-17	4.42843E-16	PREDICTED: transient receptor potential cation channel protein painless
RS011973	GO:0022857	transmembrane transporter activity	MF	-0.861945869	1.222753935	-0.568825813	4.359430432	80.33905671	2.59602E-17	5.34219E-16	PREDICTED: zinc transporter 1
RS004424	GO:0022857	transmembrane transporter activity	MF	-0.154114067	2.332271731	0.36051487	5.646491566	77.24068683	1.19889E-16	2.38151E-15	PREDICTED: TWiK family of potassium channels protein 7
RS002366	GO:0022857	transmembrane transporter activity	MF	-2.013469227	2.285528889	0.940362283	2.009763796	76.37983445	1.83374E-16	3.57494E-15	PREDICTED: sodium-dependent serotonin transporter
RS005453	GO:0022857	transmembrane transporter activity	MF	0.668735883	1.879757994	0.412329477	5.54953181	75.34668517	3.05352E-16	5.83551E-15	PREDICTED: ATP-binding cassette sub-family G member 1
RS006074	GO:0022857	transmembrane transporter activity	MF	0.061476322	1.293176276	1.550346639	6.752584934	73.22602795	8.69474E-16	1.6009E-14	PREDICTED: viral IAP-associated factor homolog
RS011988	GO:0022857	transmembrane transporter activity	MF	-1.045428446	0.08149937	1.776943069	7.507925055	72.44172426	1.28023E-15	2.31656E-14	PREDICTED: aquaporin-12
RS006315	GO:0022857	transmembrane transporter activity	MF	-2.445857294	-4.975199992	0.147442902	3.069628072	71.7469443	1.80352E-15	3.24017E-14	PREDICTED: bumetanide-sensitive sodium-(potassium)-chloride cotransporter
RS008278	GO:0022857	transmembrane transporter activity	MF	0.642268679	2.750452439	1.283258456	3.961355993	71.14168969	2.43087E-15	4.34246E-14	PREDICTED: potassium voltage-gated channel protein Shaw-like
RS000228	GO:0022857	transmembrane transporter activity	MF	-1.198401056	0.748426917	1.708285335	5.232498935	70.64756833	3.1016E-15	5.48618E-14	PREDICTED: facilitated trehalose transporter Tret1-like
RS006271	GO:0022857	transmembrane transporter activity	MF	1.985922002	1.711587011	-1.594562947	3.353974541	68.37067995	9.52991E-15	1.62849E-13	PREDICTED: organic cation transporter 1
RS008443	GO:0022857	transmembrane transporter activity	MF	-1.979883882	-8.057075206	-1.206380805	2.046366836	67.84965365	1.23201E-14	2.07155E-13	PREDICTED: facilitated trehalose transporter Tret1-2 homolog
RS014695	GO:0022857	transmembrane transporter activity	MF	-3.182123286	-4.605585624	-2.434488323	1.709736198	64.954873	5.12876E-14	8.27038E-13	PREDICTED: organic cation transporter protein
RS001006	GO:0022857	transmembrane transporter activity	MF	-1.308055843	0.748595123	2.011362686	7.151837524	64.31436805	7.03086E-14	1.12512E-12	PREDICTED: sodium-dependent nutrient amino acid transporter 1-like
RS006994	GO:0022857	transmembrane transporter activity	MF	-0.66398235	-0.594278945	1.697528097	4.919565	60.59841537	4.37914E-13	6.597E-12	PREDICTED: putative inorganic phosphate cotransporter
RS007831	GO:0022857	transmembrane transporter activity	MF	-0.285689079	2.23184291	-0.54998832	3.347851609	60.46667692	4.67238E-13	7.00523E-12	PREDICTED: sodium- and chloride-dependent GABA transporter ine-like
RS010878	GO:0022857	transmembrane transporter activity	MF	-0.202147251	0.219397642	1.259334773	5.8954575	54.42606082	9.10217E-12	1.23527E-10	PREDICTED: band 3 anion transport protein
RS008617	GO:0022857	transmembrane transporter activity	MF	-1.758654905	-1.933395291	0.119722157	4.240860888	52.46640405	2.38231E-11	3.09308E-10	PREDICTED: proton-coupled amino acid transporter 1
RS006021	GO:0022857	transmembrane transporter activity	MF	-1.978389488	-0.987147201	0.23457041	5.767022167	52.1310105	2.80858E-11	3.63527E-10	PREDICTED: cationic amino acid transporter 3
RS005693	GO:0022857	transmembrane transporter activity	MF	-1.530868766	-3.126670283	-3.45883824	4.672689231	51.54252942	3.74885E-11	4.81275E-10	PREDICTED: facilitated trehalose transporter Tret1-2 homolog
RS000134	GO:0022857	transmembrane transporter activity	MF	-2.137136524	0.447963208	0.262936854	3.43282157	50.22811762	7.14362E-11	8.98769E-10	PREDICTED: putative ammonium transporter 1
RS012141	GO:0022857	transmembrane transporter activity	MF	-0.795199967	0.272005658	1.157168	4.936082578	49.75842476	8.99389E-11	1.12594E-09	PREDICTED: UNC93-like protein MFSD11
RS008254	GO:0022857	transmembrane transporter activity	MF	-1.84400551	0.769806662	1.172103168	3.667348911	48.7792472	1.45353E-10	1.78592E-09	PREDICTED: facilitated trehalose transporter Tret1-like
RS009380	GO:0022857	transmembrane transporter activity	MF	1.181487238	0.230316163	-1.011984835	4.090504935	45.23692145	8.23976E-10	9.50289E-09	PREDICTED: b(0,+)-type amino acid transporter 1-like
RS001492	GO:0022857	transmembrane transporter activity	MF	-1.047661717	-2.014014601	-0.725945308	5.708393329	45.11153039	8.76123E-10	1.00308E-08	PREDICTED: multidrug resistance-associated protein 4-like
RS006648	GO:0022857	transmembrane transporter activity	MF	-1.472287059	-1.627468546	0.001448714	3.845832603	45.04057983	9.07077E-10	1.03664E-08	PREDICTED: voltage-dependent L-type calcium channel subunit beta-2
RS006270	GO:0022857	transmembrane transporter activity	MF	-0.625556145	-0.022131593	2.665286326	2.775447041	44.95816183	9.44409E-10	1.07832E-08	PREDICTED: organic cation transporter 1
RS011778	GO:0022857	transmembrane transporter activity	MF	-1.482690204	-0.163981472	-1.627972092	5.097898358	44.03120475	1.48632E-09	1.66834E-08	PREDICTED: facilitated trehalose transporter Tret1-2 homolog
RS005640	GO:0022857	transmembrane transporter activity	MF	0.969003673	1.718434974	-0.876644168	4.401662752	43.66119041	1.78117E-09	1.9764E-08	PREDICTED: glycine receptor subunit alpha-4
RS009578	GO:0022857	transmembrane transporter activity	MF	-2.77838182	-2.247321578	-2.454512864	4.019688809	42.39569844	3.30671E-09	3.59937E-08	PREDICTED: solute carrier family 28 member 3
RS001007	GO:0022857	transmembrane transporter activity	MF	-0.057453873	2.550863666	4.593751253	0.34449975	41.36132174	5.4813E-09	5.84023E-08	PREDICTED: sodium-dependent nutrient amino acid transporter 1-like
RS015310	GO:0022857	transmembrane transporter activity	MF	-1.26553277	0.940311337	0.163445694	5.405928146	41.08193487	6.2827E-09	6.64911E-08	PREDICTED: ATP-binding cassette sub-family G member 1
RS010452	GO:0022857	transmembrane transporter activity	MF	-1.264493454	-3.411377807	-0.656775569	3.802175748	40.96006363	6.66799E-09	7.05094E-08	PREDICTED: transient receptor potential cation channel protein painless
RS014047	GO:0022857	transmembrane transporter activity	MF	-0.592092988	1.748032789	5.355436168	1.817327481	40.93864928	6.73809E-09	7.11311E-08	PREDICTED: zinc transporter ZIP1-like
RS005851	GO:0022857	transmembrane transporter activity	MF	0.526397294	1.41413322	1.673681755	7.829535198	40.74316203	7.413E-09	7.77347E-08	PREDICTED: proton-coupled amino acid transporter 4
RS012191	GO:0022857	transmembrane transporter activity	MF	-0.827958312	0.960594434	-0.015691014	7.391207833	39.39645508	1.43044E-08	1.45634E-07	PREDICTED: V-type proton ATPase 116 kDa subunit a
RS009711	GO:0022857	transmembrane transporter activity	MF	0.862276886	1.272132135	1.421293308	6.056371062	36.7986326	5.07562E-08	4.85732E-07	PREDICTED: tropinone reductase 2-like
RS014465	GO:0022857	transmembrane transporter activity	MF	-2.203563111	-3.568610423	-0.279641755	5.849726949	35.76065503	8.4137E-08	7.87237E-07	PREDICTED: probable multidrug resistance-associated protein lethal(2)03659
RS011779	GO:0022857	transmembrane transporter activity	MF	-2.22356949	-3.571952783	-1.230326148	2.104606825	35.6579628	8.84493E-08	8.24523E-07	PREDICTED: facilitated trehalose transporter Tret1-2 homolog
RS013235	GO:0022857	transmembrane transporter activity	MF	-2.068035818	-1.64402774	1.426351822	0.894091033	34.98409736	1.22771E-07	1.1286E-06	PREDICTED: glutamate receptor 1-like
RS012786	GO:0022857	transmembrane transporter activity	MF	-1.725772589	-2.922030511	-0.315113572	3.189291251	34.971992	1.23496E-07	1.13444E-06	PREDICTED: facilitated trehalose transporter Tret1-2 homolog
RS004248	GO:0022857	transmembrane transporter activity	MF	-2.357800174	-1.138542703	0.799750155	3.47254249	34.60029849	1.47968E-07	1.34356E-06	PREDICTED: facilitated trehalose transporter Tret1-like
RS009116	GO:0022857	transmembrane transporter activity	MF	-2.774708917	-1.965598127	1.306984186	1.044379091	34.54483465	1.52014E-07	1.3783E-06	PREDICTED: urea transporter 1-like
RS000365	GO:0022857	transmembrane transporter activity	MF	-2.043121889	-2.26896537	0.189661525	11.00673959	34.00204383	1.97922E-07	1.77664E-06	PREDICTED: calcium-transporting ATPase sarcoplasmic/endoplasmic reticulum type
RS007032	GO:0022857	transmembrane transporter activity	MF	-1.972036202	-0.832866298	0.156731051	5.195017285	33.86656984	2.11394E-07	1.89487E-06	PREDICTED: ammonium transporter Rh type A
RS001722	GO:0022857	transmembrane transporter activity	MF	-0.334283753	-2.198611554	-0.278419111	3.485639997	32.69185314	3.74056E-07	3.27598E-06	PREDICTED: solute carrier family 41 member 1-like
RS007693	GO:0022857	transmembrane transporter activity	MF	-0.242378739	-0.621851837	1.493665703	5.076496585	32.00947491	5.20946E-07	4.51534E-06	PREDICTED: zinc transporter ZIP14
RS004347	GO:0022857	transmembrane transporter activity	MF	-0.916171765	-2.042094379	0.238772477	3.454218425	31.9850429	5.27159E-07	4.55665E-06	PREDICTED: organic cation transporter protein-like
RS006814	GO:0022857	transmembrane transporter activity	MF	-0.514796382	1.014347124	0.975336891	4.041760153	30.97725487	8.59474E-07	7.23543E-06	PREDICTED: large neutral amino acids transporter small subunit 2
RS006320	GO:0022857	transmembrane transporter activity	MF	1.668458777	1.549661536	0.932473469	6.183206129	30.51118393	1.07731E-06	8.96741E-06	PREDICTED: bumetanide-sensitive sodium-(potassium)-chloride cotransporter
RS007713	GO:0022857	transmembrane transporter activity	MF	-1.593472957	-0.392083561	-0.180235411	5.938924251	30.40817878	1.13245E-06	9.40771E-06	PREDICTED: ATP-binding cassette sub-family D member 2
RS003904	GO:0022857	transmembrane transporter activity	MF	-2.706690593	-2.534495324	0.490780854	4.216110096	30.29657835	1.19537E-06	9.89777E-06	PREDICTED: facilitated trehalose transporter Tret1-like
RS010500	GO:0022857	transmembrane transporter activity	MF	1.19026545	2.873457875	1.841995752	12.00045446	30.1935168	1.25657E-06	1.03772E-05	PREDICTED: tubulin alpha-1 chain
RS000193	GO:0022857	transmembrane transporter activity	MF	-0.612906575	0.552707825	0.864925878	6.395375097	30.19397061	1.25629E-06	1.03772E-05	PREDICTED: b(0,+)-type amino acid transporter 1
RS000996	GO:0022857	transmembrane transporter activity	MF	-1.508231266	-0.156640833	0.876498273	2.831732428	29.09435446	2.13949E-06	1.70644E-05	PREDICTED: zinc transporter ZIP3-like
RS005491	GO:0022857	transmembrane transporter activity	MF	-0.937465516	-1.630388798	-0.209059704	4.107611972	28.88192352	2.37108E-06	1.88281E-05	PREDICTED: voltage-gated potassium channel subunit beta-2
RS009579	GO:0022857	transmembrane transporter activity	MF	-0.361377028	0.937501921	0.004604885	5.160969196	28.85056945	2.40732E-06	1.91039E-05	PREDICTED: solute carrier family 28 member 3-like
RS011850	GO:0022857	transmembrane transporter activity	MF	-0.767278606	-0.313949384	0.713484854	8.083358391	28.67013092	2.62688E-06	2.07027E-05	PREDICTED: trimeric intracellular cation channel type B
RS008885	GO:0022857	transmembrane transporter activity	MF	-1.592550754	-2.358375405	-0.949039702	4.983900477	28.41800515	2.96751E-06	2.32998E-05	PREDICTED: facilitated trehalose transporter Tret1-2 homolog
RS010090	GO:0022857	transmembrane transporter activity	MF	0.323234855	1.546465547	-0.132352818	2.938456182	28.16134763	3.35955E-06	2.60531E-05	PREDICTED: cyclic nucleotide-gated cation channel subunit A-like
RS008631	GO:0022857	transmembrane transporter activity	MF	-0.45372484	0.018443887	2.172529191	1.173123173	28.08258513	3.48992E-06	2.69976E-05	PREDICTED: glutamate receptor ionotropic, delta-1-like
RS008887	GO:0022857	transmembrane transporter activity	MF	-0.760329305	-4.339267866	-1.430676324	3.320036492	27.63642804	4.32958E-06	3.3078E-05	PREDICTED: facilitated trehalose transporter Tret1-like
RS006269	GO:0022857	transmembrane transporter activity	MF	-1.78180142	-1.191554952	-0.830372469	4.550094908	27.39894348	4.85584E-06	3.68178E-05	PREDICTED: organic cation transporter 1
RS012590	GO:0022857	transmembrane transporter activity	MF	-0.22021637	0.861437679	0.641734274	6.761709937	27.36974453	4.9248E-06	3.72958E-05	PREDICTED: high affinity copper uptake protein 1
RS012741	GO:0022857	transmembrane transporter activity	MF	-1.060191661	0.585439948	0.026171952	4.628484614	26.6687965	6.90781E-06	5.10845E-05	PREDICTED: organic cation transporter protein
RS000895	GO:0022857	transmembrane transporter activity	MF	1.014348253	1.385977981	0.68384576	7.930053973	26.65746374	6.94569E-06	5.13345E-05	PREDICTED: translocon-associated protein subunit beta
RS000719	GO:0022857	transmembrane transporter activity	MF	-0.394780773	-1.247672358	-1.502851389	6.140116665	25.60119706	1.15586E-05	8.29929E-05	PREDICTED: aquaporin AQPAn.G
RS003902	GO:0022857	transmembrane transporter activity	MF	0.212010943	1.72144821	0.98329227	4.242513254	25.33598899	1.31339E-05	9.3504E-05	PREDICTED: facilitated trehalose transporter Tret1
RS013310	GO:0022857	transmembrane transporter activity	MF	-1.594173173	0.30942411	0.502900611	3.784418211	25.32704004	1.31907E-05	9.38549E-05	PREDICTED: sodium- and chloride-dependent transporter XTRP3
RS003638	GO:0022857	transmembrane transporter activity	MF	-0.69625377	-0.738048152	0.351114728	5.7591544	24.53074045	1.93528E-05	0.00013355	PREDICTED: ATP-binding cassette sub-family G member 4
RS005461	GO:0022857	transmembrane transporter activity	MF	-1.570191565	-0.758659476	-0.541009138	7.57624513	24.31985122	2.1419E-05	0.000146843	PREDICTED: solute carrier family 22 member 21-like
RS003139	GO:0022857	transmembrane transporter activity	MF	-0.462942241	-0.484276182	0.670801183	4.994154039	24.0689115	2.4166E-05	0.000164334	PREDICTED: zinc transporter 1
RS006319	GO:0022857	transmembrane transporter activity	MF	-1.264071762	-1.378688832	-0.296664377	4.499770473	23.99784591	2.50059E-05	0.000169587	PREDICTED: bumetanide-sensitive sodium-(potassium)-chloride cotransporter
RS008934	GO:0022857	transmembrane transporter activity	MF	-2.249974024	-1.610242152	-0.986553809	2.596021099	23.90344612	2.61667E-05	0.000177078	ionotropic receptor 25a
RS010443	GO:0022857	transmembrane transporter activity	MF	-2.227753564	-2.234532899	-0.56368196	2.617137088	23.82549146	2.71656E-05	0.000183248	PREDICTED: pickpocket protein 28-like
RS014152	GO:0022857	transmembrane transporter activity	MF	-1.430499389	-1.354570713	-0.671668718	4.505777329	23.40073256	3.33153E-05	0.000221644	PREDICTED: potassium channel subfamily K member 1-like
RS012504	GO:0022857	transmembrane transporter activity	MF	1.701019285	0.103598753	-0.952358557	2.60795793	23.14266371	3.77102E-05	0.000248475	PREDICTED: zinc transporter ZIP3
RS007091	GO:0022857	transmembrane transporter activity	MF	-0.260344997	-0.000107201	0.982798512	6.405504865	23.00178887	4.03484E-05	0.000263836	PREDICTED: probable multidrug resistance-associated protein lethal(2)03659

RS014340	GO:0022857	transmembrane transporter activity	MF	-0.92887959	-0.092715464	0.219765138	5.620832256	22.82606695	4.38983E-05	0.00028483	PREDICTED: ileal sodium/bile acid cotransporter-like
RS011507	GO:0022857	transmembrane transporter activity	MF	0.081672207	0.439418944	1.152509484	5.150989282	22.16598652	6.02438E-05	0.000382801	PREDICTED: sialin
RS005054	GO:0022857	transmembrane transporter activity	MF	-0.882564902	-1.772840386	-2.09465887	2.914764324	21.56658479	8.02786E-05	0.000498781	PREDICTED: ETS domain-containing protein Elk-3-like
RS012462	GO:0022857	transmembrane transporter activity	MF	-1.887439376	-1.068323927	-1.822700455	2.935725927	21.16526065	9.72757E-05	0.000593839	PREDICTED: synaptic vesicle glycoprotein 2B
RS012046	GO:0022857	transmembrane transporter activity	MF	-0.044612109	1.318321788	-0.182662099	3.873303092	21.0021542	0.000105168	0.000636465	PREDICTED: sodium-independent sulfate anion transporter-like
RS015154	GO:0022857	transmembrane transporter activity	MF	0.77118859	1.275597924	0.795023023	8.092063536	20.820501	0.00011471	0.000690232	PREDICTED: protein transport protein Sec61 subunit gamma
RS015395	GO:0022857	transmembrane transporter activity	MF	-1.227128143	-0.999149743	-0.38673491	5.789022217	20.66182653	0.000123747	0.000741733	PREDICTED: ryanodine receptor 44F
RS010854	GO:0022857	transmembrane transporter activity	MF	-0.464601417	0.421962906	0.417617417	6.7210824	20.51691341	0.000132619	0.000789321	PREDICTED: V-type proton ATPase subunit e 2-like
RS006273	GO:0022857	transmembrane transporter activity	MF	-0.841668922	-0.780206767	0.412687786	4.250127088	20.41178555	0.000139449	0.000826843	PREDICTED: organic cation transporter 1
RS014287	GO:0022857	transmembrane transporter activity	MF	-0.825809928	1.486619083	1.592270521	1.389357496	20.39684624	0.000140447	0.000831501	PREDICTED: sialin
RS007340	GO:0022857	transmembrane transporter activity	MF	1.72289566	2.228109689	-0.488523344	0.853368064	20.24210443	0.000151218	0.000888679	PREDICTED: mitochondrial sodium/hydrogen exchanger 9B2
RS004393	GO:0022857	transmembrane transporter activity	MF	-1.227790861	-2.264108903	-0.817189459	2.289519889	20.23673107	0.000151606	0.000890546	PREDICTED: putative tricarboxylate transport protein, mitochondrial
RS015388	GO:0022857	transmembrane transporter activity	MF	-2.602050655	-3.074067005	-0.457367858	0.547921075	20.16611512	0.000156804	0.000918937	PREDICTED: glutamate receptor ionotropic, delta-1
RS000135	GO:0022857	transmembrane transporter activity	MF	-0.631745801	-0.940404648	0.237026994	4.32458574	20.11793543	0.000160452	0.000937697	PREDICTED: putative ammonium transporter 1
RS011787	GO:0022857	transmembrane transporter activity	MF	-0.438940613	0.619762966	1.077554296	6.634288702	19.98955295	0.000170591	0.000992342	Zinc finger protein 782
RS006499	GO:0022857	transmembrane transporter activity	MF	-1.047637087	0.301924919	0.728056025	3.319116729	19.94751042	0.000174047	0.001009187	PREDICTED: transient receptor potential cation channel subfamily V member 5
RS015396	GO:0022857	transmembrane transporter activity	MF	-1.256182982	-1.010805264	-0.293857127	6.048626436	19.73517564	0.0001926	0.00110506	PREDICTED: ryanodine receptor 44F
RS008662	GO:0022857	transmembrane transporter activity	MF	-0.323694319	-0.887991823	-0.04413226	5.702043181	19.66407708	0.000199242	0.001138501	PREDICTED: transient receptor potential cation channel subfamily V member 5
RS005651	GO:0022857	transmembrane transporter activity	MF	-0.219646792	-1.283329628	-0.275521678	5.060327004	19.22158667	0.000246019	0.001380116	PREDICTED: transient-receptor-potential-like protein
RS012441	GO:0022857	transmembrane transporter activity	MF	-0.795629624	0.983276815	1.634723389	1.23435714	19.18509754	0.000250332	0.001401285	AGAP004474
RS006140	GO:0022857	transmembrane transporter activity	MF	0.033292599	1.273516036	1.00013418	2.720807394	19.17927372	0.000251027	0.00140446	PREDICTED: potassium channel subfamily K member 18
RS005838	GO:0022857	transmembrane transporter activity	MF	-1.272361428	-3.114064861	-3.237130599	0.429125999	19.0443607	0.000267685	0.001488399	PREDICTED: glutamate receptor U1-like
RS003726	GO:0022857	transmembrane transporter activity	MF	-0.35022698	0.054528944	0.659374478	4.976150075	18.98906357	0.000274826	0.001524592	PREDICTED: mitochondrial coenzyme A transporter SLC25A42
RS001336	GO:0022857	transmembrane transporter activity	MF	0.412169289	1.116247727	1.639275177	2.741362032	18.96980298	0.000277358	0.001536754	PREDICTED: glutamate receptor U1-like
RS007614	GO:0022857	transmembrane transporter activity	MF	0.699165628	-0.052782673	-0.817546641	5.472681929	18.94140065	0.000281133	0.001554478	PREDICTED: facilitated trehalose transporter Tret1-like
RS007694	GO:0022857	transmembrane transporter activity	MF	-1.402205119	-1.429007484	-0.154037672	3.235518975	18.72862507	0.000311092	0.001703432	PREDICTED: proton myo-inositol cotransporter-like
RS009272	GO:0022857	transmembrane transporter activity	MF	-0.152985688	-1.205701524	-0.53386679	4.590610282	18.71946093	0.000312451	0.001710132	PREDICTED: mitochondrial sodium/hydrogen exchanger 9B2
RS011525	GO:0022857	transmembrane transporter activity	MF	-0.806138244	0.427082827	0.274818707	7.810153084	18.69931455	0.000315461	0.001724354	PREDICTED: sodium/potassium-transporting ATPase subunit beta-2
RS009604	GO:0022857	transmembrane transporter activity	MF	-1.648345631	0.550771769	0.526514629	2.368500525	18.41833332	0.000360561	0.001945804	PREDICTED: sodium-dependent noradrenaline transporter
RS009304	GO:0022857	transmembrane transporter activity	MF	-2.056162023	-1.492683824	-1.148437663	1.440266038	18.0307206	0.000433479	0.002294761	PREDICTED: glutamate receptor ionotropic, delta-1-like
RS014410	GO:0022857	transmembrane transporter activity	MF	-0.538555444	-0.069436224	0.424307658	5.574699261	17.86202393	0.00046963	0.002466436	PREDICTED: sodium- and chloride-dependent neutral and basic amino acid transporter B(0+)
RS000837	GO:0022857	transmembrane transporter activity	MF	-1.260815808	-0.582381283	0.014148923	5.012164404	17.85146996	0.000471988	0.002476758	PREDICTED: excitatory amino acid transporter 1-like
RS003799	GO:0022857	transmembrane transporter activity	MF	-0.143761693	0.372761559	-0.639546518	5.875204513	17.46636866	0.000566606	0.002928125	PREDICTED: ATP-binding cassette sub-family C member Sur
RS007085	GO:0022857	transmembrane transporter activity	MF	-2.123520843	-1.442400071	-1.654436852	1.159522622	17.26753428	0.000622612	0.00317714	PREDICTED: cyclic nucleotide-gated cation channel alpha-3
RS000849	GO:0022857	transmembrane transporter activity	MF	-0.753175709	-1.571391853	-1.058453667	2.708513766	17.06922787	0.000683946	0.003464852	PREDICTED: monocarboxylate transporter 9-like
RS012432	GO:0022857	transmembrane transporter activity	MF	-1.318193047	-1.41640057	-1.093486391	3.821570882	17.02394884	0.000698772	0.003535692	PREDICTED: acetylcholine receptor subunit alpha-type acr-16-like
RS013332	GO:0022857	transmembrane transporter activity	MF	-1.915754341	-0.761067399	-0.092972296	1.96508851	16.98236489	0.000712669	0.003598779	PREDICTED: potassium channel subfamily T member 2
RS004044	GO:0022857	transmembrane transporter activity	MF	-0.216859326	0.637026253	1.004892576	2.871056733	16.9549321	0.000721987	0.003638536	PREDICTED: glutamate-gated chloride channel
RS012047	GO:0022857	transmembrane transporter activity	MF	-1.033691566	-1.204387105	-0.573210977	3.717195265	16.53395031	0.000881135	0.004370625	PREDICTED: sodium-independent sulfate anion transporter-like
RS010364	GO:0022857	transmembrane transporter activity	MF	-0.052944401	0.405459181	0.815688442	7.091949691	16.43172938	0.000924767	0.004565472	PREDICTED: cytochrome c oxidase subunit 7A2, mitochondrial
RS002855	GO:0022857	transmembrane transporter activity	MF	-1.74839573	-1.535258414	-0.16366084	1.79539144	16.39328726	0.000941725	0.00463647	PREDICTED: glycine receptor subunit alpha-3
RS000927	GO:0022857	transmembrane transporter activity	MF	-2.570527298	-2.804707147	-0.755163456	0.667619183	16.35029216	0.000961058	0.004715063	PREDICTED: b(0,+)-type amino acid transporter 1-like
RS009094	GO:0022857	transmembrane transporter activity	MF	0.810509918	0.868482563	0.707370764	6.315762678	16.22341499	0.001020437	0.004986955	PREDICTED: mitochondrial import inner membrane translocase subunit Tim17-B-like
RS003544	GO:0022857	transmembrane transporter activity	MF	-0.410771227	-0.348918681	1.010423069	3.516674428	16.0029682	0.001132396	0.005455777	PREDICTED: glutamate receptor ionotropic, kainate 2
RS006141	GO:0022857	transmembrane transporter activity	MF	0.186642093	0.509454722	0.888482209	5.536921472	15.98603197	0.001141486	0.005494665	Potassium channel subfamily K member 9
RS003914	GO:0022857	transmembrane transporter activity	MF	-0.408304954	-1.308021582	-1.113950825	3.842131166	15.96402321	0.001153407	0.005540047	PREDICTED: facilitated trehalose transporter Tret1-like
RS013870	GO:0022857	transmembrane transporter activity	MF	-1.185159015	-0.789079424	-0.942978819	4.024142004	15.86729932	0.001207277	0.005765813	PREDICTED: sodium- and chloride-dependent glycine transporter 1-like
RS013164	GO:0022857	transmembrane transporter activity	MF	-0.720894807	-1.573394185	-0.567112816	2.35012097	15.85215695	0.001215934	0.005800558	PREDICTED: sodium/hydrogen exchanger 3
RS014761	GO:0022857	transmembrane transporter activity	MF	0.850386459	0.827647699	0.741742978	6.600879234	15.79201097	0.001250932	0.005936034	PREDICTED: mitochondrial import receptor subunit TOM40 homolog 1
RS012306	GO:0022857	transmembrane transporter activity	MF	-1.171605666	-1.08070317	-0.099574832	5.667246046	15.66500563	0.001328153	0.006252994	PREDICTED: ABC transporter G family member 23
RS012806	GO:0022857	transmembrane transporter activity	MF	-0.671548237	-0.771532863	-0.831977584	6.320380877	15.5512679	0.001401314	0.006555774	PREDICTED: glutamate [NMDA] receptor subunit 1
RS004534	GO:0022857	transmembrane transporter activity	MF	-0.580382142	2.035273319	0.497901415	1.025113205	15.51468926	0.001425682	0.006654944	PREDICTED: sodium channel protein Nach-like
RS007810	GO:0022857	transmembrane transporter activity	MF	-0.820216184	-1.018356117	-0.958242629	3.620004773	15.51103249	0.001428141	0.006659019	PREDICTED: vesicular glutamate transporter 1
RS000928	GO:0022857	transmembrane transporter activity	MF	-2.818387407	-2.387215456	-1.195316578	0.324906115	15.29049888	0.001584497	0.007309578	B(0,+)-type amino acid transporter 1
RS006610	GO:0022857	transmembrane transporter activity	MF	0.2664942	0.92285009	0.741592285	5.324734931	15.2782684	0.001593649	0.007342643	PREDICTED: zinc transporter 7
RS008896	GO:0022857	transmembrane transporter activity	MF	-1.274733506	-1.006034401	-0.767037953	2.62762816	15.13459455	0.001705167	0.007799012	PREDICTED: mitoferrin-1
RS011193	GO:0022857	transmembrane transporter activity	MF	0.23153241	-0.655301507	0.610210215	6.072153027	15.07588643	0.001752934	0.007978478	PREDICTED: organic cation transporter protein
RS006757	GO:0022857	transmembrane transporter activity	MF	-0.233100515	0.411940029	0.749443033	4.26814335	14.98825356	0.001826715	0.008260555	PREDICTED: cell death-inducing p53-target protein 1
RS009126	GO:0022857	transmembrane transporter activity	MF	0.004334845	0.550905396	0.691970165	7.068681097	14.88906784	0.001913944	0.008599435	PREDICTED: V-type proton ATPase subunit F
RS001526	GO:0022857	transmembrane transporter activity	MF	-0.188683928	0.519017421	0.650345185	7.644232128	14.87803596	0.001923898	0.008634916	PREDICTED: V-type proton ATPase subunit G
RS006318	GO:0022857	transmembrane transporter activity	MF	-3.618949223	-2.702749807	-1.072386578	0.467850565	14.73641507	0.002056334	0.009147816	PREDICTED: bumetanide-sensitive sodium-(potassium)-chloride cotransporter
RS005708	GO:0022857	transmembrane transporter activity	MF	-2.57707626	-0.767810406	-0.121337824	1.208165742	14.61680361	0.002175202	0.009598631	PREDICTED: sodium- and chloride-dependent glycine transporter 1-like
RS013194	GO:0022857	transmembrane transporter activity	MF	-0.402235939	1.468418978	0.620702468	1.800456379	14.57025873	0.002223278	0.009779937	PREDICTED: gamma-aminobutyric acid receptor alpha-like
RS008446	GO:0022857	transmembrane transporter activity	MF	0.363470434	-0.27703378	-1.408383647	4.990252537	14.55183725	0.002242595	0.009858024	PREDICTED: facilitated trehalose transporter Tret1-2 homolog
RS008491	GO:0022857	transmembrane transporter activity	MF	-0.83498967	-1.675590127	-1.132486269	2.878684988	14.45091871	0.00235141	0.010286093	PREDICTED: callisulfakinin
RS012689	GO:0022857	transmembrane transporter activity	MF	-0.335162446	-0.922302748	-1.067051914	4.164281375	14.43562454	0.002368352	0.010353914	PREDICTED: solute carrier family 41 member 1-like
RS008723	GO:0022857	transmembrane transporter activity	MF	0.765724899	0.492155106	-0.726953396	2.604372289	14.25219643	0.002581214	0.011140444	PREDICTED: excitatory amino acid transporter
RS005190	GO:0022857	transmembrane transporter activity	MF	-0.561761317	0.20318587	0.563166122	6.506172496	14.19948267	0.002645822	0.011376401	PREDICTED: calcium-activated potassium channel slowpoke
RS013212	GO:0022857	transmembrane transporter activity	MF	-0.878244991	-0.110123051	0.018139925	7.487946573	14.16068921	0.002694389	0.011553674	PREDICTED: V-type proton ATPase subunit B
RS011379	GO:0022857	transmembrane transporter activity	MF	-0.945895165	-0.137739549	0.02413553	6.197611827	14.11352967	0.002754622	0.01177587	PREDICTED: chloride intracellular channel exc-4
RS014046	GO:0022857	transmembrane transporter activity	MF	-0.827652517	0.123541367	0.39500677	4.371003694	13.78924417	0.003206526	0.0134386	PREDICTED: zinc transporter ZIP1-like
RS004592	GO:0022857	transmembrane transporter activity	MF	-0.294046429	0.156068735	0.564893198	6.831343324	13.651508	0.003420014	0.014243271	PREDICTED: ATP-binding cassette sub-family B member 7, mitochondrial
RS006255	GO:0022857	transmembrane transporter activity	MF	-0.565848028	-0.409978879	0.214379631	6.839035823	13.48918712	0.003689736	0.015225603	PREDICTED: copper-transporting ATPase 1
RS012010	GO:0022857	transmembrane transporter activity	MF	-0.658055692	-0.163236513	0.25753802	6.662027841	13.45566221	0.003748014	0.01543574	PREDICTED: sodium-dependent phosphate transporter 1-B-like
RS010731	GO:0022857	transmembrane transporter activity	MF	-0.395182325	-0.639006133	-0.675231861	6.20607259	13.34648856	0.003944186	0.016153848	PREDICTED: V-type proton ATPase 116 kDa subunit a
RS006343	GO:0022857	transmembrane transporter activity	MF	0.700005517	0.678494058	0.385858572	5.661988764	13.33113205	0.003972583	0.016264862	PREDICTED: zinc transporter 6
RS010905	GO:0022857	transmembrane transporter activity	MF	-0.573248945	-0.768712677	-0.837001645	4.71486708	13.23543162	0.00415417	0.016871208	PREDICTED: gamma-aminobutyric acid receptor subunit beta
RS015097	GO:0022857	transmembrane transporter activity	MF	0.838222973	0.450337326	-0.261713518	6.777317829	13.04891082	0.004532018	0.018148247	PREDICTED: tubulin alpha-1 chain
RS014000	GO:0022857	transmembrane transporter activity	MF	0.486986842	0.493065666	-0.511287156	7.326747337	12.99389808	0.00464982	0.018525729	TWiK family of potassium channels protein 18
RS007615	GO:0022857	transmembrane transporter activity	MF	0.842199769	0.08001472	-0.354074907	7.382223287	12.96671251	0.004709147	0.018738389	PREDICTED: facilitated trehalose transporter Tret1-like
RS009863	GO:0022857	transmembrane transporter activity	MF	0.247967949	1.994626146	0.840783905	0.741237487	12.85234652	0.004967042	0.019634316	PREDICTED: sodium- and chloride-dependent glycine transporter 1-like
RS005166	GO:0022857	transmembrane transporter activity	MF	0.797745292	0.959053152	1.137980998	8.88871257	12.83598972	0.005005051	0.019744379	PREDICTED: nascent polypeptide-associated complex subunit alpha
RS005460	GO:0022857	transmembrane transporter activity	MF	-0.996149896	-0.953790558	-0.537751487	3.769830749	12.77993102	0.005137514	0.020193827	PREDICTED: solute carrier family 22 member 21-like
RS005241	GO:0022857	transmembrane transporter activity	MF	-1.24648029	-0.946331544	-0.692073388	4.41212653	12.5745733	0.005653054	0.021933014	PREDICTED: high-affinity choline transporter 1
RS006299	GO:0022857	transmembrane transporter activity	MF	0.929966764	0.90845916	-0.169322471	3.276482954	12.53656321	0.005753939	0.022276393	PREDICTED: facilitated trehalose transporter Tret1-like
RS008051	GO:0022857	transmembrane transporter activity	MF	-0.405673123	-1.320265794	-1.066020494	4.63196021	12.49923358	0.00585475	0.022555742	PREDICTED: transient receptor potential channel pyrexia
RS000227	GO:0022857	transmembrane transporter activity	MF	-1.32952285	-0.75353739	-1.12184367	1.981758767	12.38833226	0.00616466	0.023605269	PREDICTED: facilitated trehalose transporter Tret1-2 homolog
RS012250	GO:0022857	transmembrane transporter activity	MF	-1.126562781	-0.381786444	0.539034757	1.985904525	12.14501042	0.00690257	0.026042829	PREDICTED: organic cation transporter protein-like
RS002985	GO:0022857	transmembrane transporter activity	MF	-0.571114377	-0.476136182	-0.859409917	5.548946497	12.07709757	0.007123669	0.026732864	PREDICTED: Y+L amino acid transporter 2
RS008215	GO:0022857	transmembrane transporter activity	MF	0.162922308	0.46909669	0.671411093	6.515514341	12.06788131	0.007154208	0.026831478	PREDICTED: mitochondrial import receptor subunit TOM22 homolog
RS003288	GO:0022857	transmembrane transporter activity	MF	-0.468141457	-0.663116971	0.331112622	4.523147937	12.02307867	0.007304519	0.027292266	PREDICTED: sodium- and chloride-dependent GABA transporter ine
RS003997	GO:0022857	transmembrane transporter activity	MF	-1.222850277	-0.635965438	-0.411407393	6.247970682	11.97127476	0.007482212	0.027852345	PREDICTED: ABC transporter G family member 23
RS011468	GO:0022857	transmembrane transporter activity	MF	-0.318737056	-0.110014844	-1.568608212	1.963272184	11.9167036	0.007674014	0.028433815	PREDICTED: solute carrier family 35 member F6
RS000866	GO:0022857	transmembrane transporter activity	MF	-0.851954648	-0.389874807	-0.092110043	7.989972168	11.90615845	0.007711634	0.028547895	PREDICTED: sodium/potassium-transporting ATPase subunit alpha
RS003756	GO:0022857	transmembrane transporter activity	MF	-0.408342321	-0.364800511	-0.786353931	7.1095919	11.79402076	0.00812312	0.029860645	PREDICTED: multidrug resistance-associated protein 1
RS015096	GO:0022857	transmembrane transporter activity	MF	-0.857980934	-0.446788274	-0.194471442	5.697782265	11.71197794	0.008437836	0.030900291	PREDICTED: V-type proton ATPase subunit D
RS009360	GO:0022857	transmembrane transporter activity	MF	-0.052778092	-0.516301747	0.304429459	6.031942967	11.69868265	0.008489957	0.031037017	PREDICTED: sodium-dependent nutrient amino acid transporter 1
RS008458	GO:0022857	transmembrane transporter activity	MF	-1.182823715	-0.196910535	-0.390818396	8.429616967	11.6884148	0.008530428	0.031131399	PREDICTED: probable cation-transporting ATPase 13A3
RS011196	GO:0022857	transmembrane transporter activity	MF	0.675575742	0.753360958	0.761120595	5.457606567	11.66397815	0.008627513	0.031412228	PREDICTED: 39S ribosomal protein L19, mitochondrial
RS004997	GO:0022857	transmembrane transporter activity	MF	-0.851696275	-1.530670443	-0.345032758	2.982640462	11.64463017	0.008705154	0.031658304	PREDICTED: kinesin-like protein KIF17
RS002363	GO:0022857	transmembrane transporter activity	MF	-0.329275879	-0.729371826	0.101654401	6.167217704	11.55948198	0.009055119	0.032732541	PREDICTED: multidrug resistance-associated protein 5
RS008898	GO:0022857	transmembrane transporter activity	MF	-0.172960297	-0.478698026	-0.774628888	4.936093792	11.53878925	0.009142244	0.033009582	PREDICTED: sideroflexin-1
RS002295	GO:0022857	transmembrane transporter activity	MF	-0.760632417	-0.881718429	-1.336627404	7.31207469	11.50237509	0.009297576	0.033436228	PREDICTED: sodium- and chloride-dependent GABA transporter 1-like
RS012787	GO:0022857	transmembrane transporter activity	MF	-1.042887095	-1.14108662	-0.848878197	2.917253142	11.38739346	0.009805347	0.03491082	PREDICTED: facilitated trehalose transporter Tret1-2 homolog
RS000219	GO:0022857	transmembrane transporter activity	MF	0.217052434	-0.673773561	-1.028588766	6.485154642	11.32601418	0.010087507	0.035776418	PREDICTED: innexin inx2
RS007389	GO:0022857	transmembrane transporter activity	MF	-0.71821859	-0.059688714	0.178211598	8.141021926	11.17756615	0.010803454	0.037931058	PREDICTED: V-type proton ATPase catalytic subunit A
RS007388	GO:0022857	transmembrane transporter activity	MF	-1.012601041	-0.502641916	-0.211964145	6.238906794	11.11536226	0.011118147	0.038884183	PREDICTED: V-type proton ATPase catalytic subunit A
RS006740	GO:0022857	transmembrane transporter activity	MF	-0.43064001	-0.612335737	-0.738849827	6.295225865	10.98541247	0.011805018	0.040967869	PREDICTED: glutamate-gated chloride channel
RS007836	GO:0022857	transmembrane transporter activity	MF	-0.670404831	1.05713246	-0.759263575	1.495067002	10.91840834	0.012175354	0.042079146	PREDICTED: bestrophin-4
RS000123	GO:0022857	transmembrane transporter activity	MF	0.692109916	0.810423229	0.325162475	7.0801236	10.8417935	0.012612853	0.04340062	PREDICTED: ATP-binding cassette sub-family F member 3
RS011127	GO:0022857	transmembrane transporter activity	MF	0.510801033	0.668255039	0.469000295	5.728845819	10.81526531	0.01276792	0.043826433	PREDICTED: ATP-binding cassette sub-family F member 2

RS002816	GO:0022857	transmembrane transporter activity	MF	-0.748548229	0.030574886	-0.160819942	5.861566925	10.7303973	0.013276729	0.045264516	PREDICTED: multidrug resistance protein 1-like
RS012007	GO:0022857	transmembrane transporter activity	MF	-0.8805953	0.446042174	-0.616249485	2.603532973	10.7020781	0.013450915	0.045771636	PREDICTED: sodium-dependent phosphate transporter 2
RS002237	GO:0022857	transmembrane transporter activity	MF	-0.77443485	-0.782975074	-0.864409769	3.087700691	10.69522479	0.013493406	0.045891426	PREDICTED: transient receptor potential cation channel protein painless
RS010501	GO:0022857	transmembrane transporter activity	MF	-0.249581193	0.164542324	0.830736506	8.23731792	10.57427827	0.014265398	0.048114201	PREDICTED: ATP synthase subunit e, mitochondrial
RS013211	GO:0022857	transmembrane transporter activity	MF	-0.46592983	-1.425136997	0.127678346	1.648820506	10.50692227	0.014714013	0.049415541	PREDICTED: pickpocket protein 19-like
RS005829	GO:0022857	transmembrane transporter activity	MF	-0.565901365	-0.398114437	0.075675202	5.880326068	10.47839461	0.014908177	0.04996104	PREDICTED: facilitated trehalose transporter Tret1-like
RS012348	GO:0022857	transmembrane transporter activity	MF	-1.171136152	-0.623448827	-0.241561177	4.399276235	10.47489738	0.014932153	0.050028076	V-type proton ATPase subunit S1
RS004574	GO:0022857	transmembrane transporter activity	MF	-0.256414135	0.160648707	0.765161971	8.179741228	10.46991438	0.01496638	0.050122195	PREDICTED: ATP synthase subunit delta, mitochondrial
RS005209	GO:0022857	transmembrane transporter activity	MF	-0.863563851	-1.023827568	-0.616859801	5.79782502	10.40323263	0.015431901	0.051374401	PREDICTED: multidrug resistance protein homolog 49
RS012105	GO:0022857	transmembrane transporter activity	MF	-0.587037702	-0.604268783	0.220956679	3.910659327	10.37503836	0.015632991	0.051906642	Inward rectifier potassium channel 2
RS000868	GO:0022857	transmembrane transporter activity	MF	-0.929392451	-0.475108864	-0.289097862	8.324170965	10.36013171	0.01574035	0.052235565	PREDICTED: sodium/potassium-transporting ATPase subunit alpha
RS005239	GO:0022857	transmembrane transporter activity	MF	-0.442689839	-0.720832595	-0.092125906	4.79919063	10.32683206	0.015982804	0.05290077	PREDICTED: vesicular acetylcholine transporter
RS008445	GO:0022857	transmembrane transporter activity	MF	-0.794376386	-2.696738816	-1.11925367	1.261432481	10.25254337	0.016537016	0.054463175	PREDICTED: facilitated trehalose transporter Tret1-2 homolog
RS008079	GO:0022857	transmembrane transporter activity	MF	-1.109710923	-0.938014135	-0.296800028	2.900944855	10.13930135	0.017418391	0.056816166	PREDICTED: potassium voltage-gated channel protein Shaker
RS009153	GO:0022857	transmembrane transporter activity	MF	0.613293967	0.998084897	0.966219795	3.330543492	10.10124836	0.017724806	0.057621634	PREDICTED: mitochondrial import inner membrane translocase subunit Tim9
RS003950	GO:0022857	transmembrane transporter activity	MF	-0.587671115	-0.868990799	-0.714405964	4.844171479	10.0941278	0.017782729	0.057765203	PREDICTED: cationic amino acid transporter 2
RS005883	GO:0022857	transmembrane transporter activity	MF	0.579677196	0.662723942	0.941061143	6.190916775	10.02257363	0.018375223	0.059324417	PREDICTED: mitochondrial import inner membrane translocase subunit Tim10
RS009608	GO:0022857	transmembrane transporter activity	MF	-0.650681402	-0.131877234	-0.002301287	6.339415917	9.831393766	0.020055087	0.063636624	PREDICTED: V-type proton ATPase subunit C
RS002046	GO:0022857	transmembrane transporter activity	MF	-1.040945999	-1.141609481	-1.378037143	3.132734296	9.81352734	0.020219576	0.06404561	PREDICTED: sodium- and chloride-dependent GABA transporter 1-like
RS001413	GO:0022857	transmembrane transporter activity	MF	-0.248212965	0.06615521	0.706495165	7.98044758	9.766834252	0.020655746	0.065132313	PREDICTED: cytochrome b-c1 complex subunit 7-like
RS015433	GO:0022857	transmembrane transporter activity	MF	-1.023491195	-0.871650733	-0.547044844	4.320060789	9.694765434	0.021347153	0.067027185	PREDICTED: glutamate receptor ionotropic, kainate 2
RS012325	GO:0022857	transmembrane transporter activity	MF	-0.610322555	-0.73163567	0.587302153	2.890049632	9.633930261	0.021948434	0.068590218	PREDICTED: serine/threonine-protein phosphatase 6 regulatory ankyrin repeat subunit A
RS001773	GO:0022857	transmembrane transporter activity	MF	-1.179645502	-1.391479741	-1.203064835	2.188248107	9.497351264	0.023359555	0.072211644	PREDICTED: acetylcholine receptor subunit alpha-like
RS006809	GO:0022857	transmembrane transporter activity	MF	-0.069599294	0.161390813	-0.669189997	5.521140821	9.427681054	0.02411329	0.074118459	PREDICTED: sodium bicarbonate cotransporter 3
RS003636	GO:0022857	transmembrane transporter activity	MF	0.658899301	-0.027122995	0.775979951	5.812404377	9.412838955	0.024276916	0.074486992	PREDICTED: ATP-binding cassette sub-family G member 4
RS007719	GO:0022857	transmembrane transporter activity	MF	-0.363554514	0.201823484	0.241238455	6.872179235	9.390306687	0.024527407	0.075102884	PREDICTED: V-type proton ATPase 21 kDa proteolipid subunit
RS005942	GO:0022857	transmembrane transporter activity	MF	0.222003058	0.357500804	-0.707071584	4.173704825	9.375192754	0.024696847	0.075474905	PREDICTED: mitochondrial sodium/hydrogen exchanger 9B2-like
RS003802	GO:0022857	transmembrane transporter activity	MF	0.811082921	0.486372202	-0.08894724	2.933919036	9.26719754	0.025941417	0.078459703	PREDICTED: cGMP-dependent protein kinase, isozyme 1
RS009928	GO:0022857	transmembrane transporter activity	MF	-0.835456878	-2.347674561	-0.849585016	1.053053669	9.23428246	0.02633282	0.079385129	PREDICTED: sodium-dependent noradrenaline transporter-like
RS003234	GO:0022857	transmembrane transporter activity	MF	-0.402942012	0.351806881	0.253791792	7.730694199	9.198914238	0.026759846	0.080432815	PREDICTED: V-type proton ATPase 16 kDa proteolipid subunit
RS012808	GO:0022857	transmembrane transporter activity	MF	0.436531101	0.768358177	0.047570539	8.601906761	9.12311113	0.027698056	0.082719781	PREDICTED: ATP-binding cassette sub-family E member 1
RS007617	GO:0022857	transmembrane transporter activity	MF	-1.32510407	-1.458516586	-0.870134155	3.353068646	9.039095435	0.028775582	0.085310659	PREDICTED: zinc transporter ZIP10
RS009139	GO:0022857	transmembrane transporter activity	MF	0.649351007	1.292845053	0.336142374	2.039377777	9.031613663	0.028873505	0.085540561	PREDICTED: Y+L amino acid transporter 2
RS002268	GO:0022857	transmembrane transporter activity	MF	-0.663213265	-0.543123609	-0.277002394	5.289469542	8.984030819	0.029503961	0.086978671	PREDICTED: inositol 1,4,5-trisphosphate receptor
RS013576	GO:0022857	transmembrane transporter activity	MF	-0.152692106	0.186422925	0.739608273	8.524782718	8.933399856	0.030189603	0.088564608	PREDICTED: cytochrome c oxidase subunit 7A2, mitochondrial-like
RS008214	GO:0022857	transmembrane transporter activity	MF	-0.591657749	-0.60801793	-0.342781866	5.622477097	8.89592842	0.030707059	0.089873274	PREDICTED: sodium channel protein paralytic
RS007859	GO:0022857	transmembrane transporter activity	MF	-1.037355681	-0.953164057	-1.137934678	2.631254112	8.895408464	0.0307143	0.08987358	PREDICTED: proton-coupled amino acid transporter 4
RS003404	GO:0022857	transmembrane transporter activity	MF	-0.282230962	0.285723529	0.434899732	4.438872301	8.825502366	0.031703186	0.092359456	PREDICTED: DET1- and DDB1-associated protein 1
RS013668	GO:0022857	transmembrane transporter activity	MF	-1.170027407	-0.729178035	0.872714223	0.383894951	8.820674505	0.031772619	0.092540325	PREDICTED: glutamate receptor 3.4-like
RS000001	GO:0022857	transmembrane transporter activity	MF	-0.923753843	-0.774146522	-0.309728113	8.964042611	8.801674733	0.032047317	0.093060619	PREDICTED: ryanodine receptor 44F
RS009135	GO:0022857	transmembrane transporter activity	MF	-2.013397474	0.050668944	0.558887993	0.476879882	8.748046843	0.032835258	0.094920773	PREDICTED: sodium channel protein Nach-like
RS011130	GO:0022857	transmembrane transporter activity	MF	0.62266454	0.316671327	-0.198111492	8.646709884	8.691352167	0.03368886	0.096889131	PREDICTED: 60 kDa heat shock protein, mitochondrial-like
RS014303	GO:0022857	transmembrane transporter activity	MF	1.015519516	-0.140449015	-1.617687421	1.096446077	8.671576225	0.033991683	0.097559539	PREDICTED: sodium channel protein Nach-like
RS001434	GO:0022857	transmembrane transporter activity	MF	-2.10642209	-0.940504046	-0.189980266	0.468874389	8.555806562	0.035818546	0.101461711	PREDICTED: sodium channel protein Nach-like
RS012852	GO:0022857	transmembrane transporter activity	MF	0.095937018	0.366584098	-0.497735083	4.298329529	8.535728142	0.036145003	0.102133761	PREDICTED: glutamate receptor ionotropic, kainate 2-like
RS005358	GO:0022857	transmembrane transporter activity	MF	0.260075089	0.471390925	0.694718137	6.608164203	8.461122113	0.037383693	0.104938933	PREDICTED: surfeit locus protein 1
RS006017	GO:0022857	transmembrane transporter activity	MF	-0.226333875	0.829010789	0.173010283	3.457042927	8.42538268	0.037991671	0.106302401	PREDICTED: cationic amino acid transporter 2
RS003858	GO:0022857	transmembrane transporter activity	MF	-0.359815662	-0.446595841	0.382189615	3.498410245	8.385315512	0.038684752	0.10786673	PREDICTED: sodium/potassium-transporting ATPase subunit beta-2-like
RS012784	GO:0022857	transmembrane transporter activity	MF	-0.964188065	-1.053670411	-1.487092291	6.693074429	8.119987423	0.043595935	0.118762068	PREDICTED: excitatory amino acid transporter-like
RS005143	GO:0022857	transmembrane transporter activity	MF	0.352665656	0.523610265	0.606871417	5.289665213	8.000205041	0.046007468	0.124178752	PREDICTED: protein SCO1 homolog, mitochondrial
RS002239	GO:0022857	transmembrane transporter activity	MF	-0.518558054	-0.887931947	-1.328774582	2.771875246	7.996103171	0.04609231	0.124359208	PREDICTED: transient receptor potential cation channel protein painless
RS005210	GO:0022857	transmembrane transporter activity	MF	-0.986436868	-1.240920184	-1.092022056	3.917975026	7.935991254	0.047353263	0.126994675	PREDICTED: multidrug resistance protein homolog 49
RS008614	GO:0022857	transmembrane transporter activity	MF	-0.377384484	0.178151297	0.139169662	6.960985845	7.911936368	0.047867212	0.127882831	PREDICTED: H(+)/Cl(-) exchange transporter 5
RS007665	GO:0022857	transmembrane transporter activity	MF	-0.631881952	-0.428365421	0.007983181	8.098194771	7.871009593	0.048754144	0.129949141	PREDICTED: plasma membrane calcium-transporting ATPase 2
RS003841	GO:0022857	transmembrane transporter activity	MF	-0.018830809	0.667905762	-0.171179406	4.919354176	7.849821458	0.04921958	0.130930145	PREDICTED: major facilitator superfamily domain-containing protein 10
RS008434	GO:0022857	transmembrane transporter activity	MF	-0.152418883	-0.335351871	-0.836822092	4.94412872	7.808586514	0.050137806	0.132702402	PREDICTED: potassium voltage-gated channel subfamily H member 2
RS010293	GO:0022857	transmembrane transporter activity	MF	-1.323536549	-0.537537624	-0.40616185	2.969834016	7.797939139	0.050377598	0.133149101	PREDICTED: sodium/hydrogen exchanger 3
RS002931	GO:0022857	transmembrane transporter activity	MF	-0.784498789	-0.645948105	-0.625581434	3.345246421	7.781766391	0.050743963	0.133976828	PREDICTED: acetylcholine receptor subunit beta-like 2
RS012051	GO:0022857	transmembrane transporter activity	MF	-1.206457775	-0.320139517	-0.400442703	3.93903891	7.774642457	0.050906164	0.13434875	PREDICTED: sodium-independent sulfate anion transporter-like
RS012317	GO:0022857	transmembrane transporter activity	MF	-0.648427381	0.001131192	-0.007541087	6.300999393	7.771586789	0.050975892	0.13447641	PREDICTED: calcium-transporting ATPase type 2C member 1
RS003265	GO:0022857	transmembrane transporter activity	MF	0.67065611	0.570992468	0.622458253	5.669387556	7.641310173	0.054036623	0.140868812	PREDICTED: 28S ribosomal protein S9, mitochondrial
RS015407	GO:0022857	transmembrane transporter activity	MF	-0.53343531	-0.824506724	0.003613607	6.554411162	7.589715422	0.055297539	0.143502413	PREDICTED: sodium/potassium-transporting ATPase subunit beta-2
RS005847	GO:0022857	transmembrane transporter activity	MF	-0.009134422	0.240630937	0.785370217	8.208365721	7.589016609	0.055314812	0.143517665	PREDICTED: ATP synthase subunit epsilon, mitochondrial-like
RS014918	GO:0022857	transmembrane transporter activity	MF	-0.265116474	0.084232844	0.568475259	7.79189049	7.503165544	0.057477171	0.147999897	PREDICTED: cytochrome c1, heme protein, mitochondrial
RS007235	GO:0022857	transmembrane transporter activity	MF	-0.411884685	-0.555436113	-0.88679334	5.718214674	7.500689334	0.057540743	0.148133302	PREDICTED: melanotransferrin
RS009082	GO:0022857	transmembrane transporter activity	MF	-1.344763505	-0.953478298	-1.303298962	1.512508029	7.470773642	0.058314177	0.149663762	PREDICTED: glycine receptor subunit alpha-2-like
RS008448	GO:0022857	transmembrane transporter activity	MF	-0.874319748	-1.179805365	-0.159545862	2.543187294	7.449190097	0.058878449	0.150714469	PREDICTED: facilitated trehalose transporter Tret1-2 homolog
RS002799	GO:0022857	transmembrane transporter activity	MF	-0.659046602	-0.211273757	-0.16274335	6.419710777	7.409924332	0.059918615	0.152787009	PREDICTED: V-type proton ATPase subunit H
RS002292	GO:0022857	transmembrane transporter activity	MF	-0.849226044	-0.724590211	-0.752733052	3.999828127	7.339934983	0.06181703	0.156330055	V-type proton ATPase subunit S1
RS004018	GO:0022857	transmembrane transporter activity	MF	-0.854655712	-0.687918747	0.356622885	2.251863917	7.340140368	0.061811374	0.156330055	PREDICTED: potassium/sodium hyperpolarization-activated cyclic nucleotide-gated channel 2
RS010902	GO:0022857	transmembrane transporter activity	MF	-0.413608261	-0.854721106	-0.933757255	3.754238005	7.337994128	0.061870497	0.156389668	PREDICTED: glutamate receptor 1
RS006810	GO:0022857	transmembrane transporter activity	MF	-0.692918241	-0.709642301	-0.469276201	5.552917252	7.276110601	0.063598968	0.159523084	PREDICTED: Y+L amino acid transporter 2
RS006920	GO:0022857	transmembrane transporter activity	MF	-0.632866598	-0.974633547	-0.967627708	3.248752993	7.241310635	0.064591429	0.161433709	PREDICTED: glutamate receptor ionotropic, NMDA 2B
RS008996	GO:0022857	transmembrane transporter activity	MF	-0.818894069	-0.144607229	0.175883673	2.981815036	7.237819263	0.064691824	0.161620478	PREDICTED: cytochrome c oxidase assembly factor 6 homolog
RS002605	GO:0022857	transmembrane transporter activity	MF	-0.518735488	0.392463203	-0.08725497	4.03235098	7.22499608	0.065061855	0.162287383	PREDICTED: solute carrier family 40 member 1
RS000609	GO:0022857	transmembrane transporter activity	MF	0.612008555	0.732502903	0.190053663	8.31658549	7.219606678	0.065217984	0.162580225	PREDICTED: muscle calcium channel subunit alpha-1
RS006503	GO:0022857	transmembrane transporter activity	MF	-0.789357073	-0.069673576	-0.042357301	4.655019811	7.200877419	0.065763394	0.163551378	PREDICTED: transient receptor potential cation channel trpm
RS015105	GO:0022857	transmembrane transporter activity	MF	-0.678666411	-2.224992259	-0.562865466	0.559101899	7.185153044	0.066224707	0.164438873	PREDICTED: organic cation transporter 1
RS005264	GO:0022857	transmembrane transporter activity	MF	-1.488549143	-1.308665565	-1.379377385	1.600885014	7.17732657	0.066455483	0.164816926	PREDICTED: sodium-dependent neutral amino acid transporter B(0)AT3
RS006739	GO:0022857	transmembrane transporter activity	MF	-0.215562063	-0.194338635	-0.661480143	5.23328973	7.126492884	0.067973454	0.167558754	PREDICTED: potassium voltage-gated channel protein Shab
RS000133	GO:0022857	transmembrane transporter activity	MF	-0.045454902	0.182331435	0.994722318	2.687399163	7.116976504	0.068261333	0.168071011	PREDICTED: putative ammonium transporter 1
RS015404	GO:0022857	transmembrane transporter activity	MF	-0.033173942	0.491254687	-0.13268681	6.364272838	7.035330626	0.07078015	0.172954058	PREDICTED: sodium/potassium-transporting ATPase subunit beta-2
RS003449	GO:0022857	transmembrane transporter activity	MF	-0.271604031	-0.623910119	-0.38398901	6.1655336	7.019104607	0.07129133	0.173865804	PREDICTED: glutamate receptor ionotropic, kainate 2
RS014295	GO:0022857	transmembrane transporter activity	MF	0.412981653	0.489533068	0.464743455	5.949145628	7.0080526	0.071641551	0.174618482	PREDICTED: zinc transporter 5
RS003825	GO:0022857	transmembrane transporter activity	MF	-0.116552603	-0.74841501	-0.195189608	7.935069367	6.915883201	0.07462774	0.18005155	PREDICTED: H(+)/Cl(-) exchange transporter 7
RS004291	GO:0022857	transmembrane transporter activity	MF	0.781190319	-1.037280119	-0.352171624	0.672722301	6.843951183	0.07704145	0.184460079	PREDICTED: synaptic vesicle glycoprotein 2B-like
RS003427	GO:0022857	transmembrane transporter activity	MF	-0.513964722	-0.550228674	-0.09809219	5.664872118	6.801016027	0.078517892	0.186983778	PREDICTED: voltage-dependent calcium channel type A subunit alpha-1
RS002296	GO:0022857	transmembrane transporter activity	MF	0.050929101	-1.155041098	-1.453067074	0.872761787	6.743534644	0.080537346	0.190763087	PREDICTED: glycine receptor subunit alpha-4
RS003145	GO:0022857	transmembrane transporter activity	MF	-1.309880541	-0.628756059	-0.991127574	1.553035926	6.713260678	0.081620986	0.19245815	PREDICTED: neuronal acetylcholine receptor subunit alpha-7-like
RS012048	GO:0022857	transmembrane transporter activity	MF	-0.674894361	-1.155560739	-0.486644875	2.649535097	6.691851744	0.082395778	0.193852499	PREDICTED: sodium-independent sulfate anion transporter-like
RS009471	GO:0022857	transmembrane transporter activity	MF	0.454440582	0.068570398	0.92902968	1.975531925	6.680170813	0.082821497	0.194672252	PREDICTED: potassium voltage-gated channel protein Shal
RS002982	GO:0022857	transmembrane transporter activity	MF	0.437159386	0.690352357	0.482073212	5.918755174	6.64375724	0.084162258	0.197455193	PREDICTED: multidrug resistance-associated protein 5-like
RS000035	GO:0022857	transmembrane transporter activity	MF	-0.59529335	-0.304820032	0.347972721	8.866264808	6.597065948	0.085912068	0.200794204	PREDICTED: cytochrome b-c1 complex subunit Rieske, mitochondrial-like
RS000318	GO:0022857	transmembrane transporter activity	MF	-0.409411201	-0.696964023	-0.524046279	5.146315283	6.540394841	0.088082972	0.204696733	PREDICTED: alpha-aminoadipic semialdehyde dehydrogenase
RS001821	GO:0022857	transmembrane transporter activity	MF	-0.416559115	-0.243117375	0.420607418	9.114591662	6.45061119	0.091630759	0.210852874	PREDICTED: cytochrome c oxidase subunit 4 isoform 1, mitochondrial
RS003508	GO:0022857	transmembrane transporter activity	MF	-0.169271487	0.401452192	-0.239447825	4.192461457	6.422099786	0.092785845	0.212888492	PREDICTED: organic cation transporter 1-like
RS010561	GO:0022857	transmembrane transporter activity	MF	0.077383384	1.01828952	0.325351919	10.99254901	6.38788389	0.094190516	0.215171867	PREDICTED: tubulin alpha-1 chain
RS007738	GO:0022857	transmembrane transporter activity	MF	-2.236721279	-1.816342735	0.104485909	0.00738012	6.381695156	0.094446755	0.215638584	PREDICTED: dynein heavy chain 10, axonemal
RS006697	GO:0022857	transmembrane transporter activity	MF	-0.137394972	-0.578930188	0.149350385	8.454117513	6.353830787	0.095608773	0.21750305	PREDICTED: zinc transporter ZIP13 homolog
RS007190	GO:0022857	transmembrane transporter activity	MF	-0.878979816	-0.595509543	0.227085088	2.704210207	6.34629855	0.095925237	0.217838484	PREDICTED: sodium channel protein Nach-like
RS010275	GO:0022857	transmembrane transporter activity	MF	-0.327809837	0.151537852	-0.881426827	3.129420115	6.335110353	0.096397162	0.218625222	PREDICTED: equilibrative nucleoside transporter 4
RS015389	GO:0022857	transmembrane transporter activity	MF	-1.014604708	-1.708407111	-0.480189784	0.854123779	6.332870963	0.096491888	0.218718663	PREDICTED: glutamate receptor ionotropic, delta-2
RS009966	GO:0022857	transmembrane transporter activity	MF	-0.243614202	-0.508062646	-0.78761656	3.789270855	6.228722713	0.100997397	0.225925617	PREDICTED: transient receptor potential channel pyrexia-like
RS005351	GO:0022857	transmembrane transporter activity	MF	-0.397867942	-0.039889938	0.245645216	7.41412513	6.082183312	0.107679573	0.237000444	PREDICTED: glucose transporter type 1
RS011409	GO:0022857	transmembrane transporter activity	MF	-0.278990895	0.058673971	0.653521491	10.28933054	5.992909669	0.111955695	0.243939451	PREDICTED: voltage-dependent anion-selective channel protein 2
RS007852	GO:0022857	transmembrane transporter activity	MF	-0.4463895	-0.500436907	-0.353366809	5.359879642	5.946281355	0.114253205	0.24733912	PREDICTED: multidrug resistance-associated protein 7
RS000168	GO:0022857	transmembrane transporter activity	MF	-0.555511687	-0.887978015	-1.234124807	1.652741901	5.837936606	0.119766759	0.255954957	PREDICTED: potassium voltage-gated channel protein eag
RS000398	GO:0022857	transmembrane transporter activity	MF	0.277198819	0.379861004	-0.066027388	5.894739848	5.761774521	0.123793477	0.26277685	PREDICTED: RNA polymerase I-specific transcription initiation factor RRN3
RS001145	GO:0022857	transmembrane transporter activity	MF	-0.53262649	-0.12107554	0.117285332	6.762749099	5.723923744	0.125842321	0.265692906	PREDICTED: solute carrier family 2, facilitated glucose transporter member 1-like
RS003358	GO:0022857	transmembrane transporter activity	MF	-0.353225898	-0.121840444	0.371616686	8.291426996	5.633056759	0.130893435	0.272925814	PREDICTED: ATP synthase-coupling factor 6, mitochondrial
RS008279	GO:0022857	transmembrane transporter activity	MF	-0.484679453	-0.433954641	0.086422434	3.880506245	5.606020043	0.132433181	0.27531586	PREDICTED: potassium voltage-gated channel protein Shaw-like

RS006497	GO:0022857	transmembrane transporter activity	MF	-0.499541328	-1.800371012	-1.244184902	0.270751657	5.580972087	0.133875009	0.277534463	PREDICTED: facilitated trehalose transporter Tret1-2 homolog
RS009361	GO:0022857	transmembrane transporter activity	MF	-0.469548252	-1.030089652	-0.272125463	2.379260128	5.571170404	0.134443268	0.278300217	PREDICTED: sodium- and chloride-dependent glycine transporter 2-like
RS002972	GO:0022857	transmembrane transporter activity	MF	-0.311172151	-0.506842424	-0.237806006	6.495567074	5.534163734	0.136609467	0.281857737	PREDICTED: sodium leak channel non-selective protein
RS012532	GO:0022857	transmembrane transporter activity	MF	0.52422059	0.27169055	0.170626759	5.950381512	5.527522768	0.137001687	0.282351902	PREDICTED: adenosine 3'-phospho 5'-phosphosulfate transporter 2
RS007587	GO:0022857	transmembrane transporter activity	MF	0.548015678	0.376775275	0.454348959	4.86046888	5.504926438	0.138344259	0.284411948	PREDICTED: magnesium transporter NIPA2
RS012098	GO:0022857	transmembrane transporter activity	MF	-0.722635213	-0.863193595	-0.599560773	3.5591329	5.486483871	0.13944927	0.285797251	PREDICTED: protein tweety
RS009262	GO:0022857	transmembrane transporter activity	MF	-0.25857572	0.038639212	0.546361074	8.794488927	5.469506202	0.140473903	0.28738277	PREDICTED: cytochrome c oxidase subunit 7C, mitochondrial-like
RS012521	GO:0022857	transmembrane transporter activity	MF	-0.27911124	0.207963234	0.176143396	5.682101261	5.464162095	0.140797903	0.287847294	PREDICTED: sodium/hydrogen exchanger 8
RS011419	GO:0022857	transmembrane transporter activity	MF	0.142670051	0.566918683	0.76286631	3.172442667	5.332525292	0.149005743	0.30010848	PREDICTED: putative inorganic phosphate cotransporter
RS000402	GO:0022857	transmembrane transporter activity	MF	-0.26718069	0.008072239	0.51581876	8.673900207	5.301862053	0.150981611	0.302828859	PREDICTED: cytochrome c oxidase subunit 6A, mitochondrial
RS006927	GO:0022857	transmembrane transporter activity	MF	-0.523545819	-0.19238077	0.403170712	9.665847911	5.252945184	0.154185014	0.307637209	PREDICTED: mitochondrial-processing peptidase subunit beta
RS014310	GO:0022857	transmembrane transporter activity	MF	-0.394503871	-0.23364982	-0.939036808	2.647212913	5.221628248	0.156269422	0.310811292	PREDICTED: neuronal acetylcholine receptor subunit alpha-7
RS004262	GO:0022857	transmembrane transporter activity	MF	-0.304556625	0.103631535	0.676510563	10.41026235	5.204184057	0.157441978	0.312748307	PREDICTED: ATP synthase lipid-binding protein, mitochondrial
RS007787	GO:0022857	transmembrane transporter activity	MF	-0.305912555	0.11337882	0.601620189	9.43857381	5.186627171	0.15863048	0.314655166	PREDICTED: ATP synthase subunit b, mitochondrial
RS008202	GO:0022857	transmembrane transporter activity	MF	0.24252685	0.342844048	-0.965951499	1.493557986	5.185516154	0.158705973	0.314677881	PREDICTED: voltage-dependent calcium channel gamma-5 subunit
RS002769	GO:0022857	transmembrane transporter activity	MF	-0.180596666	-0.510025269	-0.494314256	5.266272897	5.147923967	0.161280396	0.318264698	PREDICTED: mitochondrial 2-oxoglutarate/malate carrier protein
RS000666	GO:0022857	transmembrane transporter activity	MF	-0.080539122	0.013403368	0.652885093	9.136399161	5.147116783	0.161336103	0.31832475	PREDICTED: ATP synthase subunit g, mitochondrial
RS006076	GO:0022857	transmembrane transporter activity	MF	-0.399500112	-0.234112198	-0.791572834	3.218719557	5.135126595	0.162165734	0.319411206	PREDICTED: cationic amino acid transporter 4
RS015132	GO:0022857	transmembrane transporter activity	MF	-0.311288053	0.021217655	0.441252337	8.852842665	5.035692944	0.169202112	0.329814486	PREDICTED: ATP synthase subunit O, mitochondrial
RS001012	GO:0022857	transmembrane transporter activity	MF	-0.050637771	0.65837124	0.220417241	5.028311487	5.034189195	0.169310694	0.329872366	PREDICTED: sodium/calcium exchanger 2
RS013006	GO:0022857	transmembrane transporter activity	MF	-0.538272733	-0.817432042	-1.008287916	2.921270255	4.979088813	0.173334784	0.334904704	PREDICTED: exportin-4-like
RS007406	GO:0022857	transmembrane transporter activity	MF	1.53218285	1.416234104	0.234676903	0.509107572	4.915967777	0.17805483	0.341676447	PREDICTED: sodium-independent sulfate anion transporter-like
RS007390	GO:0022857	transmembrane transporter activity	MF	-0.350929593	-1.171722101	-0.259139814	1.654126139	4.900550276	0.179225874	0.343191525	PREDICTED: open rectifier potassium channel protein 1
RS012974	GO:0022857	transmembrane transporter activity	MF	0.386362631	0.350475987	0.590578301	4.958398051	4.857275707	0.182551447	0.347919632	PREDICTED: mitochondrial import inner membrane translocase subunit Tim16
RS004430	GO:0022857	transmembrane transporter activity	MF	-0.579491375	-0.057917442	0.425475066	2.309902759	4.852857272	0.182894224	0.348520179	Glutamate [NMDA] receptor subunit 3A
RS015406	GO:0022857	transmembrane transporter activity	MF	-0.548835798	-0.34994381	-0.083936965	5.176471396	4.839807661	0.183910115	0.349694714	PREDICTED: sodium/potassium-transporting ATPase subunit beta-2
RS002766	GO:0022857	transmembrane transporter activity	MF	-0.496155234	-0.78863534	-0.632950682	4.168210328	4.786567039	0.188109698	0.354852096	PREDICTED: solute carrier family 25 member 36-A
RS015035	GO:0022857	transmembrane transporter activity	MF	-0.486032243	-0.438869682	-0.112800307	5.262898327	4.77200592	0.189273765	0.356097071	PREDICTED: solute carrier family 2, facilitated glucose transporter member 10-like
RS011272	GO:0022857	transmembrane transporter activity	MF	-0.449550793	-0.842262029	0.317704025	1.68576224	4.742178986	0.191679247	0.358960362	PREDICTED: transient receptor potential-gamma protein
RS013062	GO:0022857	transmembrane transporter activity	MF	-0.00162788	-0.815422885	-0.945361754	1.375980209	4.702121991	0.194954616	0.363203911	PREDICTED: transient receptor potential channel pyrexia
RS008733	GO:0022857	transmembrane transporter activity	MF	-0.069301153	-0.696190609	0.565661049	1.222507914	4.700705743	0.195071368	0.363260212	PREDICTED: pickpocket protein 28-like
RS005980	GO:0022857	transmembrane transporter activity	MF	-0.519964769	-0.115820264	0.014177289	6.649793844	4.674091701	0.197277507	0.365853766	PREDICTED: V-type proton ATPase subunit d
RS013193	GO:0022857	transmembrane transporter activity	MF	-0.571318105	-0.638002435	-0.492911161	3.125045391	4.612135042	0.202503545	0.372595481	PREDICTED: gamma-aminobutyric acid receptor subunit beta-like
RS002889	GO:0022857	transmembrane transporter activity	MF	-0.128703142	0.3529612	0.286577104	4.360050122	4.605415706	0.203077987	0.373149135	PREDICTED: bifunctional arginine demethylase and lysyl-hydroxylase PSR
RS012106	GO:0022857	transmembrane transporter activity	MF	-0.818692691	-0.45460686	0.366734327	1.141769569	4.599841297	0.203555696	0.373808755	Inward rectifier potassium channel 2
RS013960	GO:0022857	transmembrane transporter activity	MF	-0.472338012	-0.452157922	0.021607712	3.915657005	4.540751566	0.208684017	0.380067463	PREDICTED: multidrug resistance-associated protein 5-like
RS009973	GO:0022857	transmembrane transporter activity	MF	0.1676973	0.302067179	0.694181931	3.324934717	4.531641986	0.2094852	0.381030706	PREDICTED: sugar transporter SWEET1
RS002890	GO:0022857	transmembrane transporter activity	MF	-0.213107922	-0.035236467	0.494651933	9.00551627	4.518839864	0.210615958	0.382259313	PREDICTED: cytochrome c oxidase subunit 5A, mitochondrial
RS000217	GO:0022857	transmembrane transporter activity	MF	-0.750015472	-0.258912471	-0.859143521	4.771460381	4.479971735	0.214083735	0.38649234	PREDICTED: innexin inx1
RS012324	GO:0022857	transmembrane transporter activity	MF	-0.639112315	-1.234689077	-0.08776879	1.305640976	4.37485531	0.22372824	0.397633848	PREDICTED: serine/threonine-protein phosphatase 6 regulatory ankyrin repeat subunit A
RS009362	GO:0022857	transmembrane transporter activity	MF	-0.151734251	-1.379851033	-0.853593219	0.577299208	4.353138426	0.225770076	0.399764452	PREDICTED: sodium- and chloride-dependent glycine transporter 2-like
RS001539	GO:0022857	transmembrane transporter activity	MF	-0.380736825	-1.0292407	-0.36481089	2.349632725	4.316399244	0.229263428	0.403767811	PREDICTED: transient receptor potential cation channel protein painless
RS012560	GO:0022857	transmembrane transporter activity	MF	-0.041301573	-0.515464379	-0.426603809	4.255404315	4.307057209	0.230159613	0.404677505	PREDICTED: ATP-binding cassette sub-family B member 6, mitochondrial
RS005280	GO:0022857	transmembrane transporter activity	MF	0.572920685	0.052379455	0.938447567	3.032392488	4.273149565	0.233439508	0.40894939	PREDICTED: acid-sensing ion channel 4
RS001911	GO:0022857	transmembrane transporter activity	MF	-0.354858321	-0.511067736	-0.530111822	4.46637499	4.265443861	0.234190845	0.409944281	PREDICTED: palmitoyltransferase ZDHHC17
RS007791	GO:0022857	transmembrane transporter activity	MF	-0.379787464	0.049869261	0.105041138	7.48004628	4.225689753	0.238102407	0.41486742	PREDICTED: V-type proton ATPase subunit E
RS015079	GO:0022857	transmembrane transporter activity	MF	-1.376295246	0.661290456	0.618943801	0.209343525	4.174647883	0.243212401	0.420568148	PREDICTED: facilitated trehalose transporter Tret1-like
RS012398	GO:0022857	transmembrane transporter activity	MF	-0.181909214	0.045983675	0.559515665	9.326609998	4.173573583	0.243321024	0.420643099	PREDICTED: cytochrome c oxidase subunit 5B, mitochondrial-like
RS011634	GO:0022857	transmembrane transporter activity	MF	-1.482237858	-0.869085331	-1.279144023	0.748490273	4.162431432	0.24445023	0.422083595	PREDICTED: glutamate receptor ionotropic, delta-2-like
RS001774	GO:0022857	transmembrane transporter activity	MF	-0.556967124	-0.686693785	0.064399051	2.246327006	4.014186998	0.25993626	0.440531132	PREDICTED: acetylcholine receptor subunit alpha-like
RS012212	GO:0022857	transmembrane transporter activity	MF	1.298461473	1.241884362	0.487723216	0.415207106	3.920422645	0.270186252	0.451829193	PREDICTED: sodium channel protein 60E
RS000049	GO:0022857	transmembrane transporter activity	MF	-0.167016261	-0.078808593	0.315739518	4.19490186	3.918242209	0.270428905	0.452114912	PREDICTED: protein I'm not dead yet-like
RS004419	GO:0022857	transmembrane transporter activity	MF	-0.39398529	0.162481593	-1.185227216	0.809494392	3.887905498	0.273825486	0.455851742	PREDICTED: transient receptor potential-gamma protein
RS003691	GO:0022857	transmembrane transporter activity	MF	-0.396884624	-0.571293958	0.253637659	1.856747806	3.871872414	0.275636118	0.457912129	PREDICTED: transient receptor potential cation channel subfamily A member 1
RS010418	GO:0022857	transmembrane transporter activity	MF	-0.19455437	0.000394051	0.481391778	9.395600975	3.855673322	0.277476464	0.459791431	PREDICTED: cytochrome c oxidase subunit 6B1-like
RS011747	GO:0022857	transmembrane transporter activity	MF	-0.380454491	0.007681606	0.358897081	9.615965078	3.849118736	0.278224261	0.460864966	PREDICTED: ATP synthase subunit gamma, mitochondrial
RS000693	GO:0022857	transmembrane transporter activity	MF	-0.697277451	-0.63859341	-1.105155719	1.668516432	3.761841056	0.288355965	0.47128419	PREDICTED: solute carrier family 12 member 4
RS006724	GO:0022857	transmembrane transporter activity	MF	1.072023102	0.112416307	0.609701895	0.789605252	3.761098346	0.288443587	0.47128419	PREDICTED: glutamate receptor ionotropic, delta-1-like
RS004593	GO:0022857	transmembrane transporter activity	MF	0.262368668	-0.314833663	-0.606891149	2.686998086	3.735102132	0.29152562	0.474414272	PREDICTED: SET domain-containing protein 4
RS014392	GO:0022857	transmembrane transporter activity	MF	-0.97591621	-0.707349255	0.232142575	0.530622375	3.721584252	0.293139895	0.476361786	PREDICTED: probable multidrug resistance-associated protein lethal(2)03659
RS002930	GO:0022857	transmembrane transporter activity	MF	-0.451673532	-0.71437108	-0.718954078	3.306419228	3.701408177	0.295564164	0.478695356	PREDICTED: acetylcholine receptor subunit alpha-L1
RS005072	GO:0022857	transmembrane transporter activity	MF	-0.654987192	-0.4219481	-0.18940484	3.26003723	3.685352455	0.297506145	0.480656437	PREDICTED: voltage-dependent T-type calcium channel subunit alpha-1G
RS006990	GO:0022857	transmembrane transporter activity	MF	-0.310134595	-0.4355977	-0.507024072	4.194119455	3.681544826	0.297968358	0.480765048	PREDICTED: BTB/POZ domain-containing protein KCTD3
RS008197	GO:0022857	transmembrane transporter activity	MF	-0.307158998	-0.373647818	-0.769382915	3.163133414	3.663806527	0.300130093	0.48236835	PREDICTED: lysophospholipid acyltransferase 7
RS003147	GO:0022857	transmembrane transporter activity	MF	-0.505550451	-0.027606287	-0.18120612	3.786984288	3.627252198	0.30462908	0.487532937	PREDICTED: acetylcholine receptor subunit beta-like 1
RS014617	GO:0022857	transmembrane transporter activity	MF	-0.072631645	0.084385518	0.526454273	9.138635249	3.625994331	0.304784959	0.487596216	PREDICTED: putative ATP synthase subunit f, mitochondrial
RS012817	GO:0022857	transmembrane transporter activity	MF	-0.287960684	-0.628111107	-0.66343646	3.635326683	3.625082508	0.304897998	0.48766642	PREDICTED: facilitated trehalose transporter Tret1-like
RS015381	GO:0022857	transmembrane transporter activity	MF	-0.14122205	0.494615068	-0.457720223	1.269481569	3.572397731	0.311493056	0.493953414	PREDICTED: potassium voltage-gated channel subfamily KQT member 1
RS008419	GO:0022857	transmembrane transporter activity	MF	-0.579270886	-0.22684126	-0.07023873	5.06982487	3.507414936	0.319801701	0.502353125	PREDICTED: chloride channel protein 2
RS013677	GO:0022857	transmembrane transporter activity	MF	0.034211804	0.280550659	0.595537596	9.222031987	3.437461301	0.328964218	0.510658864	PREDICTED: cytochrome c oxidase subunit 6C
RS009119	GO:0022857	transmembrane transporter activity	MF	-0.633398641	0.027582619	-0.254763808	2.374824839	3.400802845	0.333857375	0.51571198	PREDICTED: lateral signaling target protein 2 homolog
RS006561	GO:0022857	transmembrane transporter activity	MF	0.353995304	0.237572917	0.419480704	5.635873753	3.395244005	0.334604917	0.516387054	PREDICTED: EH domain-containing protein 3
RS015453	GO:0022857	transmembrane transporter activity	MF	-0.09263959	0.415348464	0.277714417	5.686672294	3.355959113	0.33992981	0.521573591	PREDICTED: sodium-dependent phosphate transporter 2
RS008482	GO:0022857	transmembrane transporter activity	MF	0.456620351	0.231742272	0.664992756	4.899508409	3.329273867	0.343589006	0.524567819	PREDICTED: organic cation transporter protein
RS008880	GO:0022857	transmembrane transporter activity	MF	0.14769946	0.553054828	0.336408558	3.369176491	3.28851771	0.349243966	0.529733651	PREDICTED: pickpocket protein 19-like
RS008726	GO:0022857	transmembrane transporter activity	MF	-0.239198332	0.278689624	0.146315272	4.384525019	3.151643572	0.368830479	0.550035649	PREDICTED: equilibrative nucleoside transporter 3
RS008888	GO:0022857	transmembrane transporter activity	MF	-0.294502555	-0.567523121	-0.206094758	5.180319559	3.112412951	0.374615949	0.554657096	PREDICTED: facilitated trehalose transporter Tret1-2 homolog
RS003956	GO:0022857	transmembrane transporter activity	MF	-0.323676554	0.116745023	-0.07691896	6.422899031	3.056887659	0.382937112	0.562262205	PREDICTED: solute carrier family 2, facilitated glucose transporter member 1-like
RS003609	GO:0022857	transmembrane transporter activity	MF	-0.051627285	0.355758402	0.153478437	5.36998255	3.050524042	0.383900772	0.56337058	PREDICTED: zinc transporter ZIP1
RS000591	GO:0022857	transmembrane transporter activity	MF	-0.210745642	-0.711938748	-0.062273638	2.249015287	2.914724059	0.404960781	0.581735608	PREDICTED: solute carrier family 41 member 1-like
RS003109	GO:0022857	transmembrane transporter activity	MF	-0.563508854	-0.456515106	-0.489126671	3.799327004	2.910695341	0.405600131	0.582321939	PREDICTED: organic cation transporter protein-like
RS005802	GO:0022857	transmembrane transporter activity	MF	-0.201437577	-0.069914946	-0.593279778	3.766942431	2.895793271	0.407972418	0.584129676	PREDICTED: two pore potassium channel protein sup-9
RS001904	GO:0022857	transmembrane transporter activity	MF	-0.27687136	-0.081401122	0.3505686	9.687890931	2.842286187	0.416586082	0.592334615	PREDICTED: ATP synthase subunit d, mitochondrial
RS011686	GO:0022857	transmembrane transporter activity	MF	-0.652816077	-0.692313812	-0.834928913	1.23665669	2.748017073	0.432128872	0.606039089	PREDICTED: TWiK family of potassium channels protein 18-like
RS012107	GO:0022857	transmembrane transporter activity	MF	-0.368799719	0.188292375	-0.013404979	5.272470317	2.653348424	0.448213543	0.621757305	PREDICTED: G protein-activated inward rectifier potassium channel 3-like
RS014879	GO:0022857	transmembrane transporter activity	MF	-0.246283404	-0.362594032	0.41505142	1.337328694	2.627851228	0.452627775	0.625660651	PREDICTED: glycine receptor subunit alphaZ1-like
RS007790	GO:0022857	transmembrane transporter activity	MF	0.13034852	0.225712601	0.386180211	4.625198528	2.606288996	0.456388071	0.628856824	PREDICTED: mitochondrial glutamate carrier 1-like
RS010426	GO:0022857	transmembrane transporter activity	MF	-0.0320805	-0.19528696	-0.535965982	2.761673686	2.581268642	0.460782863	0.632865926	PREDICTED: glutamate receptor ionotropic, NMDA 2B
RS013293	GO:0022857	transmembrane transporter activity	MF	0.198558902	1.083035633	0.686996214	0.580149697	2.56014753	0.464519072	0.636353747	PREDICTED: synaptic vesicle glycoprotein 2B-like
RS007973	GO:0022857	transmembrane transporter activity	MF	0.255068646	0.370011576	0.284397768	6.620720548	2.53491853	0.469013562	0.640437689	PREDICTED: zinc transporter 9
RS009279	GO:0022857	transmembrane transporter activity	MF	0.294401775	0.208728083	0.372173078	5.384293591	2.517191015	0.472192292	0.643035221	PREDICTED: probable low affinity copper uptake protein 2
RS003198	GO:0022857	transmembrane transporter activity	MF	-1.073241752	-1.028205771	-0.905668183	0.370380606	2.506990701	0.474029032	0.644352037	PREDICTED: glutamate receptor ionotropic, delta-1-like
RS003214	GO:0022857	transmembrane transporter activity	MF	0.817513479	0.952415195	0.900489695	0.817958486	2.496776884	0.475873854	0.64588375	PREDICTED: potassium voltage-gated channel subfamily KQT member 4
RS006138	GO:0022857	transmembrane transporter activity	MF	0.077942232	0.584419518	-0.370544993	1.33370078	2.490635188	0.476985892	0.646552279	PREDICTED: potassium channel subfamily K member 18-like
RS000905	GO:0022857	transmembrane transporter activity	MF	0.022025399	0.042858662	0.42533997	8.298571127	2.483368496	0.478304268	0.647507681	PREDICTED: cytochrome b-c1 complex subunit 10-like
RS012899	GO:0022857	transmembrane transporter activity	MF	0.204967532	0.199038052	0.483016212	5.559314101	2.452259782	0.483980622	0.652426585	PREDICTED: SID1 transmembrane family member 1-like
RS009087	GO:0022857	transmembrane transporter activity	MF	-0.753493655	-0.466320961	-0.51762636	2.340610515	2.452889414	0.483865213	0.652426585	PREDICTED: probable glutamate receptor
RS007514	GO:0022857	transmembrane transporter activity	MF	-0.393644456	0.206862252	-0.369998274	2.851975701	2.440356048	0.48616657	0.653933758	PREDICTED: solute carrier family 26 member 10-like
RS002311	GO:0022857	transmembrane transporter activity	MF	0.21167088	0.356479307	0.364913574	7.124016358	2.415178378	0.490815427	0.65773111	PREDICTED: putative GTP-binding protein 6
RS004471	GO:0022857	transmembrane transporter activity	MF	0.302505199	0.1016203	-0.272900349	3.077100879	2.396738775	0.494241988	0.660492741	PREDICTED: Y+L amino acid transporter 2
RS003266	GO:0022857	transmembrane transporter activity	MF	-0.36926559	-0.039795183	-0.401685153	5.103564049	2.37990733	0.497385826	0.663356676	PREDICTED: transient receptor potential channel pyrexia
RS006811	GO:0022857	transmembrane transporter activity	MF	-0.091072902	-0.032205255	0.237958262	6.439935586	2.353693428	0.502312778	0.667124828	PREDICTED: angio-associated migratory cell protein
RS005021	GO:0022857	transmembrane transporter activity	MF	-0.06781788	-0.474899669	-0.350734329	3.155367473	2.352458366	0.50254583	0.667200314	PREDICTED: molybdate-anion transporter-like
RS001365	GO:0022857	transmembrane transporter activity	MF	0.237278489	0.141297916	0.554160331	3.551638791	2.315424336	0.509572456	0.67338898	PREDICTED: glutamate receptor ionotropic, kainate 2
RS014502	GO:0022857	transmembrane transporter activity	MF	-0.219647182	0.224994314	0.605975653	1.297553296	2.311846878	0.51025516	0.673954219	PREDICTED: small conductance calcium-activated potassium channel protein
RS001131	GO:0022857	transmembrane transporter activity	MF	-0.46672796	-0.438731687	-0.485486742	3.00696323	2.28306329	0.515773294	0.678325905	PREDICTED: two pore calcium channel protein 1-like
RS005763	GO:0022857	transmembrane transporter activity	MF	-0.049864591	0.331988566	0.418799139	2.785560138	2.265395088	0.519182685	0.681106738	PREDICTED: TWiK family of potassium channels protein 7
RS002215	GO:0022857	transmembrane transporter activity	MF	-0.459893543	-0.373663745	-0.327616751	3.245153068	2.258109459	0.520593488	0.682313913	PREDICTED: CMP-sialic acid transporter 1
RS010646	GO:0022857	transmembrane transporter activity	MF	-0.387797302	-0.153467445	-0.201059514	5.65127642	2.191739529	0.533577259	0.692056848	PREDICTED: probable phospholipid-transporting ATPase IA
RS002929	GO:0022857	transmembrane transporter activity	MF	-0.39492811	-0.310803863	-0.368352585	3.630774923	2.110337646	0.54982422	0.704208911	PREDICTED: acetylcholine receptor subunit alpha-like 1
RS009552	GO:0022857	transmembrane transporter activity	MF	-0.317174334	0.102956036	0.402195532	11.51695741	2.083221242	0.555314671	0.708862049	PREDICTED: ATP synthase subunit beta, mitochondrial

RS002902	GO:0022857	transmembrane transporter activity	MF	-0.415647108	-0.152738152	0.265175951	11.47381982	2.020686865	0.56812419	0.719524944	PREDICTED: ATP synthase subunit alpha, mitochondrial
RS010798	GO:0022857	transmembrane transporter activity	MF	0.021270494	0.09209859	-1.197682465	0.555972403	2.01223563	0.569871042	0.720350873	PREDICTED: zinc transporter ZIP11
RS010787	GO:0022857	transmembrane transporter activity	MF	0.111909178	-0.194537134	-0.164571213	5.38282424	1.963969035	0.579918644	0.728455555	PREDICTED: ATP-binding cassette sub-family F member 1
RS005231	GO:0022857	transmembrane transporter activity	MF	-0.391467446	-0.412384168	-0.179305009	4.071451755	1.941339648	0.584670698	0.731588315	PREDICTED: organic cation transporter protein
RS014789	GO:0022857	transmembrane transporter activity	MF	-0.384309417	-0.340794963	-0.701560358	2.91880799	1.866727447	0.600523037	0.745342183	PREDICTED: zinc transporter ZIP9
RS010157	GO:0022857	transmembrane transporter activity	MF	0.252451639	0.308689007	0.216067222	5.404322605	1.826234541	0.609242714	0.751719677	PREDICTED: exportin-T
RS005281	GO:0022857	transmembrane transporter activity	MF	0.147692306	-0.427946659	0.130849224	2.285946009	1.819237088	0.610757704	0.752790867	PREDICTED: acid-sensing ion channel 4
RS008886	GO:0022857	transmembrane transporter activity	MF	-0.289536803	-0.51024615	-0.479741606	2.079554922	1.757477457	0.624231737	0.763037416	PREDICTED: facilitated trehalose transporter Tret1
RS014536	GO:0022857	transmembrane transporter activity	MF	-0.137268948	-0.07750242	-0.358531119	4.210868086	1.71200184	0.634268817	0.771080465	PREDICTED: cyclic nucleotide-gated cation channel beta-3
RS015165	GO:0022857	transmembrane transporter activity	MF	0.203835116	0.086955701	-0.092770826	6.68983845	1.645463293	0.649125814	0.780587272	PREDICTED: sodium channel protein 60E
RS003602	GO:0022857	transmembrane transporter activity	MF	-0.963739329	-0.484991816	-0.758608967	0.476696176	1.634124817	0.651677236	0.782683875	PREDICTED: solute carrier family 28 member 3
RS010427	GO:0022857	transmembrane transporter activity	MF	-0.256640259	-0.293251267	-0.831731644	1.070368751	1.607645315	0.657657541	0.78679007	PREDICTED: glutamate receptor ionotropic, NMDA 2B
RS000107	GO:0022857	transmembrane transporter activity	MF	0.215541555	0.120252028	-0.217617098	3.595365183	1.517996661	0.678122983	0.800549125	PREDICTED: multidrug resistance protein 1A
RS004131	GO:0022857	transmembrane transporter activity	MF	0.244077702	0.428184836	0.166004547	3.05086288	1.47403705	0.688275398	0.807916894	PREDICTED: synaptic vesicle 2-related protein
RS003990	GO:0022857	transmembrane transporter activity	MF	-0.055368209	0.238070575	0.293320892	4.25914611	1.454595738	0.692788544	0.811066898	PREDICTED: UDP-galactose translocator
RS009066	GO:0022857	transmembrane transporter activity	MF	-0.284333504	-0.421360118	-0.423891407	2.706723454	1.434128224	0.697554745	0.814482149	PREDICTED: solute carrier family 35 member E3-like
RS015498	GO:0022857	transmembrane transporter activity	MF	0.184679301	0.472371696	0.26432428	2.413896137	1.414695214	0.702093736	0.818113296	PREDICTED: calcium uniporter protein, mitochondrial
RS014968	GO:0022857	transmembrane transporter activity	MF	0.070753676	0.338611082	0.008973976	4.167384444	1.401868016	0.705096916	0.82085326	PREDICTED: solute carrier family 41 member 1-like
RS011446	GO:0022857	transmembrane transporter activity	MF	-0.226602437	0.017489836	-0.173735734	4.50551615	1.392641248	0.707260567	0.822459795	PREDICTED: stress-activated protein kinase JNK
RS005975	GO:0022857	transmembrane transporter activity	MF	0.184305527	-0.340054217	-0.375631512	2.198485768	1.388734626	0.708177509	0.823222037	PREDICTED: piezo-type mechanosensitive ion channel component
RS004705	GO:0022857	transmembrane transporter activity	MF	-0.430701847	-0.465359916	-0.484712083	3.064343052	1.370281204	0.712515526	0.826890945	PREDICTED: sodium/hydrogen exchanger 7
RS001366	GO:0022857	transmembrane transporter activity	MF	-0.202685308	-0.234557446	-0.316543451	6.418554265	1.353303228	0.71651627	0.829698961	PREDICTED: glutamate receptor ionotropic, kainate 2
RS010673	GO:0022857	transmembrane transporter activity	MF	0.194686816	0.144745443	0.237663354	5.4673327	1.348021968	0.717762586	0.830648431	PREDICTED: zinc transporter foi-like
RS005974	GO:0022857	transmembrane transporter activity	MF	-0.266864024	-0.195937093	-0.171697422	7.122329353	1.335637313	0.720688539	0.832387331	PREDICTED: piezo-type mechanosensitive ion channel component
RS009390	GO:0022857	transmembrane transporter activity	MF	-0.711993959	-0.803385471	-0.355292865	0.366452897	1.289104566	0.731721917	0.839984739	PREDICTED: gamma-aminobutyric acid receptor alpha-like
RS002882	GO:0022857	transmembrane transporter activity	MF	0.362043543	0.12287268	0.14951259	3.567153224	1.277605364	0.734457649	0.842342193	PREDICTED: putative sodium-coupled neutral amino acid transporter 7
RS015540	GO:0022857	transmembrane transporter activity	MF	-0.463597962	-0.808832157	-0.297537157	1.427647784	1.257020479	0.73936339	0.845811839	PREDICTED: zinc transporter ZIP11
RS013953	GO:0022857	transmembrane transporter activity	MF	-0.420067551	-0.07959435	-0.044577109	2.09881014	1.242877248	0.742739999	0.848223421	PREDICTED: two pore potassium channel protein sup-9
RS000297	GO:0022857	transmembrane transporter activity	MF	-0.078397003	0.556951886	0.159163891	1.385608421	1.213828293	0.749689662	0.85220612	PREDICTED: probable glutamate receptor
RS005062	GO:0022857	transmembrane transporter activity	MF	0.042915661	-0.171071911	-0.235081974	3.733684216	1.211821777	0.75017038	0.852564992	PREDICTED: mitochondrial uncoupling protein 4
RS005898	GO:0022857	transmembrane transporter activity	MF	-0.109533007	-0.24419355	-0.181501684	5.469138988	1.210081204	0.750587452	0.852649778	PREDICTED: ATP-binding cassette sub-family B member 10, mitochondrial
RS008405	GO:0022857	transmembrane transporter activity	MF	-0.507380646	-0.391727947	-0.160990784	2.501471485	1.183658987	0.75692617	0.857267824	PREDICTED: solute carrier family 12 member 9
RS013461	GO:0022857	transmembrane transporter activity	MF	0.139158606	-0.101013347	0.199986874	6.071677902	1.15813645	0.763061416	0.861033552	PREDICTED: ATP-binding cassette sub-family G member 5
RS001035	GO:0022857	transmembrane transporter activity	MF	0.124094061	0.276516429	0.187901485	8.14568359	1.113792392	0.773745632	0.868231611	PREDICTED: tubulin alpha-1 chain
RS010672	GO:0022857	transmembrane transporter activity	MF	-0.302029346	0.22206596	0.191043022	1.595359022	1.095814464	0.778084584	0.871039755	Zinc transporter 2
RS003408	GO:0022857	transmembrane transporter activity	MF	0.14875062	-0.250114218	0.329780306	2.915781806	1.09604514	0.778028887	0.871039755	PREDICTED: probable glutamate receptor
RS011943	GO:0022857	transmembrane transporter activity	MF	0.336788752	0.302655858	-0.329953803	0.718404792	1.062395748	0.786158762	0.876649854	PREDICTED: glutamate receptor ionotropic, delta-1-like
RS014288	GO:0022857	transmembrane transporter activity	MF	-0.267562999	-0.21719274	-0.273627167	4.00532462	1.061069676	0.786479339	0.876852054	PREDICTED: ATP-binding cassette sub-family B member 8, mitochondrial
RS001346	GO:0022857	transmembrane transporter activity	MF	-0.305712719	-0.459994451	-0.225727855	2.010578526	1.048797397	0.789446703	0.878744111	PREDICTED: glutamate receptor ionotropic, delta-1-like
RS004706	GO:0022857	transmembrane transporter activity	MF	-0.281248638	-0.101038833	-0.415774939	3.347688564	1.047260677	0.789818336	0.878882504	PREDICTED: sodium/hydrogen exchanger 7
RS007150	GO:0022857	transmembrane transporter activity	MF	0.246217518	0.212666126	0.29750827	5.406495568	0.963851814	0.80999779	0.890926827	PREDICTED: probable multidrug resistance-associated protein lethal(2)03659
RS007717	GO:0022857	transmembrane transporter activity	MF	0.066529061	0.463299961	0.014408782	1.808915589	0.956769727	0.811710752	0.892106596	PREDICTED: V-type proton ATPase 21 kDa proteolipid subunit
RS006075	GO:0022857	transmembrane transporter activity	MF	-0.226394382	-0.065362369	0.088572196	3.008430896	0.898142308	0.82587609	0.901150881	PREDICTED: acetyl-coenzyme A transporter 1
RS008929	GO:0022857	transmembrane transporter activity	MF	-0.139724103	-0.236598473	-0.091823273	4.57881475	0.895160984	0.826595415	0.901623303	PREDICTED: cytochrome c oxidase assembly protein COX15 homolog
RS011500	GO:0022857	transmembrane transporter activity	MF	-0.54663125	-0.559810746	-0.038971768	0.470844775	0.876166842	0.831175153	0.904842	PREDICTED: zinc transporter ZIP8
RS013172	GO:0022857	transmembrane transporter activity	MF	0.064707769	0.050177661	0.256276698	4.54520608	0.870549691	0.832528393	0.905630734	PREDICTED: N-acetyl-D-glucosamine kinase
RS001970	GO:0022857	transmembrane transporter activity	MF	0.084033429	-0.012169719	-0.157558613	5.885614174	0.833122903	0.84152916	0.911638874	PREDICTED: glutamate receptor ionotropic, kainate 2
RS005586	GO:0022857	transmembrane transporter activity	MF	0.165076617	-0.125717332	0.501179805	0.440219349	0.817686596	0.845232123	0.914113642	big brain
RS006019	GO:0022857	transmembrane transporter activity	MF	0.016800687	-0.569398535	-0.206623396	0.539126613	0.775635116	0.855285471	0.920559373	PREDICTED: cationic amino acid transporter 2
RS000298	GO:0022857	transmembrane transporter activity	MF	-0.477815194	-0.328207506	-0.189725936	1.140735658	0.76073894	0.858832832	0.922721437	PREDICTED: glutamate receptor ionotropic, NMDA 3A-like
RS010574	GO:0022857	transmembrane transporter activity	MF	-0.354727613	-0.373915239	-0.481676921	0.828534929	0.681580005	0.877527727	0.933866301	PREDICTED: G protein-activated inward rectifier potassium channel 3-like
RS001040	GO:0022857	transmembrane transporter activity	MF	-0.098603565	-0.201658646	0.076364834	3.089669028	0.652037571	0.884422889	0.938003319	PREDICTED: sideroflexin-2
RS003957	GO:0022857	transmembrane transporter activity	MF	0.345462579	0.413965389	0.154609396	1.754535313	0.612321805	0.893605875	0.942823504	PREDICTED: solute carrier family 2, facilitated glucose transporter member 1-like
RS003729	GO:0022857	transmembrane transporter activity	MF	-0.067068908	-0.31722074	-0.453287028	1.039779299	0.608180172	0.894557183	0.943318527	PREDICTED: neuronal acetylcholine receptor subunit alpha-7-like
RS003394	GO:0022857	transmembrane transporter activity	MF	0.180289073	0.135383955	0.089380518	6.008505153	0.597460048	0.89701359	0.944722291	PREDICTED: vesicle transport protein USE1
RS001538	GO:0022857	transmembrane transporter activity	MF	0.320201018	0.172065415	-0.020378547	1.614496317	0.588836165	0.898983255	0.94603515	PREDICTED: allatostatin-A receptor-like
RS014940	GO:0022857	transmembrane transporter activity	MF	0.00473671	-0.350880149	-0.354295712	0.736422598	0.582482205	0.900430699	0.946555742	PREDICTED: potassium voltage-gated channel protein Shal
RS005560	GO:0022857	transmembrane transporter activity	MF	0.073648066	0.070694895	-0.087436803	5.960124723	0.555903739	0.906448712	0.950525819	PREDICTED: protein catecholamines up
RS004110	GO:0022857	transmembrane transporter activity	MF	-0.109326082	-0.245764423	-0.393853677	2.856144774	0.536964028	0.910698682	0.95276119	PREDICTED: phospholipase A2 inhibitor and Ly6/PLAUR domain-containing protein
RS010583	GO:0022857	transmembrane transporter activity	MF	-0.204132095	-0.051330039	0.096105151	3.162457527	0.486088584	0.921936886	0.959149956	PREDICTED: partitioning defective protein 6
RS000285	GO:0022857	transmembrane transporter activity	MF	0.119687123	0.031475064	-0.04428715	4.541388379	0.422896761	0.935472985	0.966238561	PREDICTED: facilitated trehalose transporter Tret1-like
RS014584	GO:0022857	transmembrane transporter activity	MF	0.129219334	0.060601072	0.090579059	6.064060989	0.417243203	0.936657865	0.966828377	PREDICTED: wolframin
RS007128	GO:0022857	transmembrane transporter activity	MF	0.030616457	-0.137872106	-0.048843691	6.138938535	0.365078568	0.947358098	0.972178127	PREDICTED: probable multidrug resistance-associated protein lethal(2)03659
RS002672	GO:0022857	transmembrane transporter activity	MF	0.112781414	0.136232963	0.12426065	4.219978077	0.286688066	0.96251234	0.980258807	PREDICTED: mitochondrial import inner membrane translocase subunit Tim22
RS007404	GO:0022857	transmembrane transporter activity	MF	0.341548622	0.423696565	0.486833568	3.079113058	0.267214539	0.9660718	0.982215891	PREDICTED: sodium-independent sulfate anion transporter-like
RS009012	GO:0022857	transmembrane transporter activity	MF	0.165169583	0.002447419	-0.023721248	2.359677794	0.265786109	0.966329283	0.982398368	PREDICTED: zinc transporter 2-like
RS010152	GO:0022857	transmembrane transporter activity	MF	-0.129723253	-0.161823215	-0.203053025	1.88639679	0.263658934	0.966711776	0.98246999	PREDICTED: zinc transporter ZIP3
RS007304	GO:0022857	transmembrane transporter activity	MF	-0.03874979	-0.117709759	-0.024621974	4.815387852	0.237457293	0.971327222	0.985126901	PREDICTED: solute carrier family 17 member 9
RS002585	GO:0022857	transmembrane transporter activity	MF	0.061337763	0.015683494	0.119579633	4.655608643	0.205533408	0.976691112	0.987602807	PREDICTED: mitochondrial pyruvate carrier 1
RS008629	GO:0022857	transmembrane transporter activity	MF	0.051370738	-0.258316351	-0.177872255	0.376549123	0.205807073	0.976646438	0.987602807	PREDICTED: probable glutamate receptor
RS008706	GO:0022857	transmembrane transporter activity	MF	0.063443874	0.058144534	-0.01579188	5.633533699	0.196321115	0.978180999	0.988383465	PREDICTED: ATP-binding cassette sub-family D member 3
RS005614	GO:0022857	transmembrane transporter activity	MF	-0.026120006	0.069729297	0.030053445	4.472662817	0.146084989	0.985784124	0.992693667	PREDICTED: mitochondrial folate transporter/carrier
RS006774	GO:0022857	transmembrane transporter activity	MF	-0.005561161	-0.002818033	0.022604747	3.411433614	0.005716578	0.999885243	0.999885243	PREDICTED: equilibrative nucleoside transporter 1
RS011718	GO:0055085	transmembrane transport	BP	-0.059496373	8.716087878	3.96160781	4.311804415	501.7385592	2.004E-108	4.762E-106	PREDICTED: glucose dehydrogenase [FAD, quinone]
RS001948	GO:0055085	transmembrane transport	BP	-0.792763253	2.803258949	1.759563877	6.650188786	291.8180989	5.86789E-63	6.65767E-61	Major facilitator superfamily domain-containing protein 6-A
RS007652	GO:0055085	transmembrane transport	BP	0.585883623	0.528505008	4.851177054	4.584889963	291.2117195	7.93792E-63	8.92591E-61	PREDICTED: solute carrier family 26 member 10-like
RS005246	GO:0055085	transmembrane transport	BP	-1.039759025	2.52725738	3.661947996	5.83723879	286.4562689	8.48768E-62	9.29512E-60	PREDICTED: high-affinity choline transporter 1-like
RS012132	GO:0055085	transmembrane transport	BP	-0.641537099	0.463569382	4.164552381	6.993055111	251.5268374	3.05907E-54	2.69412E-52	PREDICTED: putative inorganic phosphate cotransporter
RS009299	GO:0055085	transmembrane transport	BP	-0.833536193	5.147671256	1.07098716	3.418953047	224.4882423	2.15009E-48	1.67149E-46	PREDICTED: organic cation transporter protein-like
RS003905	GO:0055085	transmembrane transport	BP	-1.535395853	-3.809880637	1.439384598	5.164045266	219.9356219	2.07317E-47	1.56345E-45	PREDICTED: facilitated trehalose transporter Tret1-like
RS009243	GO:0055085	transmembrane transport	BP	-0.589487895	2.064901326	2.91752274	4.715025796	173.1275538	2.68806E-37	1.47832E-35	PREDICTED: multidrug resistance protein homolog 49
RS009074	GO:0055085	transmembrane transport	BP	-0.999061123	1.569100076	2.399331412	5.711615811	162.1907409	6.1707E-35	3.12104E-33	PREDICTED: solute carrier family 46 member 3-like
RS003129	GO:0055085	transmembrane transport	BP	-1.935857211	0.013218622	3.698610157	4.556804627	145.8197805	2.10104E-31	9.2418E-30	PREDICTED: organic cation transporter protein
RS003584	GO:0055085	transmembrane transport	BP	-1.221802531	0.359585466	1.950408164	5.803684621	116.60715	4.15011E-25	1.41644E-23	PREDICTED: monocarboxylate transporter 12
RS003228	GO:0055085	transmembrane transport	BP	-0.561103954	2.12048168	1.83683218	5.406987214	114.6440514	1.09829E-24	3.71824E-23	PREDICTED: b(0,+)-type amino acid transporter 1-like
RS013974	GO:0055085	transmembrane transport	BP	1.308057058	2.315023189	0.876327879	6.127768048	111.7248777	4.66769E-24	1.53887E-22	PREDICTED: monocarboxylate transporter 10
RS001344	GO:0055085	transmembrane transport	BP	-1.224665802	0.504065929	2.789839008	4.816787403	108.7807423	2.00778E-23	6.30574E-22	PREDICTED: sodium/potassium/calcium exchanger 4
RS011780	GO:0055085	transmembrane transport	BP	-1.685309235	-4.03663067	-0.682423211	4.573186577	105.6851221	9.30608E-23	2.78387E-21	PREDICTED: facilitated trehalose transporter Tret1-2 homolog
RS004436	GO:0055085	transmembrane transport	BP	-1.6353822	-2.198323513	1.659074242	6.594585322	104.8919255	1.37848E-22	4.08485E-21	PREDICTED: monocarboxylate transporter 14
RS000720	GO:0055085	transmembrane transport	BP	-1.414180497	1.484024302	-1.819002501	5.701350612	100.1058089	1.47484E-21	4.12758E-20	aquaporin
RS012045	GO:0055085	transmembrane transport	BP	-2.417547613	-3.846942398	-2.948472949	3.138267013	95.12499238	1.73561E-20	4.58246E-19	PREDICTED: sodium-independent sulfate anion transporter
RS013239	GO:0055085	transmembrane transport	BP	1.866830916	1.827955171	-0.150888215	4.857533717	91.49789483	1.04424E-19	2.60936E-18	PREDICTED: feline leukemia virus subgroup C receptor-related protein 2
RS007494	GO:0055085	transmembrane transport	BP	-1.905744217	-3.744457543	0.74513791	6.016536186	89.73338778	2.49931E-19	6.00605E-18	PREDICTED: sodium-coupled monocarboxylate transporter 1
RS013889	GO:0055085	transmembrane transport	BP	-1.663206004	0.794911338	2.587607867	5.380228282	87.07326483	9.31253E-19	2.15989E-17	PREDICTED: sialin
RS009298	GO:0055085	transmembrane transport	BP	-2.524665679	0.320369244	1.997080797	2.729083611	85.53693786	1.99021E-18	4.50803E-17	PREDICTED: organic cation transporter protein-like
RS009300	GO:0055085	transmembrane transport	BP	-0.334428722	2.777656488	1.996146097	5.84981916	85.14092159	2.42051E-18	5.44355E-17	PREDICTED: organic cation transporter protein-like
RS013891	GO:0055085	transmembrane transport	BP	-3.03546779	0.898282569	1.370032508	5.013144301	84.17578439	3.9E-18	8.61695E-17	PREDICTED: sialin
RS015390	GO:0055085	transmembrane transport	BP	-1.260588417	-0.88728058	1.174441311	5.643186003	81.54774944	1.42892E-17	3.01438E-16	PREDICTED: sodium-independent sulfate anion transporter
RS014579	GO:0055085	transmembrane transport	BP	-1.833927302	-1.637670215	-2.03241566	4.946947748	80.73878787	2.13088E-17	4.42843E-16	PREDICTED: transient receptor potential cation channel protein painless
RS011973	GO:0055085	transmembrane transport	BP	-0.861945869	1.222753935	-0.568825813	4.359430432	80.33905671	2.59602E-17	5.34219E-16	PREDICTED: zinc transporter 1
RS005077	GO:0055085	transmembrane transport	BP	-1.853453594	-1.879553398	-0.229787216	4.527312475	77.70992995	9.50968E-17	1.89802E-15	PREDICTED: sodium-coupled monocarboxylate transporter 1-like
RS004424	GO:0055085	transmembrane transport	BP	-0.154114067	2.332271731	0.36051487	5.646491566	77.24068683	1.19889E-16	2.38151E-15	PREDICTED: TWiK family of potassium channels protein 7
RS002366	GO:0055085	transmembrane transport	BP	-2.013469227	2.285528889	0.940362283	2.009763796	76.37983445	1.83374E-16	3.57494E-15	PREDICTED: sodium-dependent serotonin transporter
RS014237	GO:0055085	transmembrane transport	BP	-1.358203588	-0.858052145	0.808855596	6.87948729	73.79033318	6.58177E-16	1.22983E-14	PREDICTED: calcium-binding mitochondrial carrier protein Aralar1
RS006074	GO:0055085	transmembrane transport	BP	0.061476322	1.293176276	1.550346639	6.752584934	73.22602795	8.69474E-16	1.6009E-14	PREDICTED: viral IAP-associated factor homolog
RS004435	GO:0055085	transmembrane transport	BP	-2.355016468	-4.840360235	-1.508287833	6.256644026	72.98893691	9.7736E-16	1.78908E-14	membrane transporter
RS006315	GO:0055085	transmembrane transport	BP	-2.445857294	-4.975199992	0.147442902	3.069628072	71.7469443	1.80352E-15	3.24017E-14	PREDICTED: bumetanide-sensitive sodium-(potassium)-chloride cotransporter
RS008278	GO:0055085	transmembrane transport	BP	0.642268679	2.750452439	1.283258456	3.961355993	71.14168969	2.43087E-15	4.34246E-14	PREDICTED: potassium voltage-gated channel protein Shaw-like
RS000228	GO:0055085	transmembrane transport	BP	-1.198401056	0.748426917	1.708285335	5.232498935	70.64756833	3.1016E-15	5.48618E-14	PREDICTED: facilitated trehalose transporter Tret1-like
RS001403	GO:0055085	transmembrane transport	BP	-2.730002539	-3.225210407	0.21862196	5.256977583	69.09878372	6.65609E-15	1.15147E-13	PREDICTED: putative metabolite transport protein HI_1104
RS006271	GO:0055085	transmembrane transport	BP	1.985922002	1.711587011	-1.594562947	3.353974541	68.37067995	9.52991E-15	1.62849E-13	PREDICTED: organic cation transporter 1

RS008443	GO:0055085	transmembrane transport	BP	-1.979883882	-8.057075206	-1.206380805	2.046366836	67.84965365	1.23201E-14	2.07155E-13	PREDICTED: facilitated trehalose transporter Tret1-2 homolog
RS014695	GO:0055085	transmembrane transport	BP	-3.182123286	-4.605585624	-2.434488323	1.709736198	64.954873	5.12876E-14	8.27038E-13	PREDICTED: organic cation transporter protein
RS001006	GO:0055085	transmembrane transport	BP	-1.308055843	0.748595123	2.011362686	7.151837524	64.31436805	7.03086E-14	1.12512E-12	PREDICTED: sodium-dependent nutrient amino acid transporter 1-like
RS006994	GO:0055085	transmembrane transport	BP	-0.66398235	-0.594278945	1.697528097	4.919565	60.59841537	4.37914E-13	6.597E-12	PREDICTED: putative inorganic phosphate cotransporter
RS007831	GO:0055085	transmembrane transport	BP	-0.285689079	2.23184291	-0.54998832	3.347851609	60.46667692	4.67238E-13	7.00523E-12	PREDICTED: sodium- and chloride-dependent GABA transporter ine-like
RS009004	GO:0055085	transmembrane transport	BP	-1.724057836	-4.564536642	-0.819207065	2.965758365	59.37486634	7.99449E-13	1.17895E-11	PREDICTED: mitochondrial basic amino acids transporter-like
RS010878	GO:0055085	transmembrane transport	BP	-0.202147251	0.219397642	1.259334773	5.8954575	54.42606082	9.10217E-12	1.23527E-10	PREDICTED: band 3 anion transport protein
RS008617	GO:0055085	transmembrane transport	BP	-1.758654905	-1.933395291	0.119722157	4.240860888	52.46640405	2.38231E-11	3.09308E-10	PREDICTED: proton-coupled amino acid transporter 1
RS006021	GO:0055085	transmembrane transport	BP	-1.978389488	-0.987147201	0.23457041	5.767022167	52.1310105	2.80858E-11	3.63527E-10	PREDICTED: cationic amino acid transporter 3
RS005693	GO:0055085	transmembrane transport	BP	-1.530868766	-3.126670283	-3.45883824	4.672689231	51.54252942	3.74885E-11	4.81275E-10	PREDICTED: facilitated trehalose transporter Tret1-2 homolog
RS009073	GO:0055085	transmembrane transport	BP	-1.510567785	0.927876147	1.809212406	5.164332774	50.87027386	5.21352E-11	6.61886E-10	PREDICTED: solute carrier family 46 member 3
RS000134	GO:0055085	transmembrane transport	BP	-2.137136524	0.447963208	0.262936854	3.43282157	50.22811762	7.14362E-11	8.98769E-10	PREDICTED: putative ammonium transporter 1
RS008254	GO:0055085	transmembrane transport	BP	-1.84400551	0.769806662	1.172103168	3.667348911	48.7792472	1.45353E-10	1.78592E-09	PREDICTED: facilitated trehalose transporter Tret1-like
RS009380	GO:0055085	transmembrane transport	BP	1.181487238	0.230316163	-1.011984835	4.090504935	45.23692145	8.23976E-10	9.50289E-09	PREDICTED: b(0,+)-type amino acid transporter 1-like
RS005293	GO:0055085	transmembrane transport	BP	-0.81903625	-1.714660195	-0.381289809	5.252711507	45.14972751	8.59898E-10	9.89E-09	PREDICTED: putative inorganic phosphate cotransporter
RS001492	GO:0055085	transmembrane transport	BP	-1.047661717	-2.014014601	-0.725945308	5.708393329	45.11153039	8.76123E-10	1.00308E-08	PREDICTED: multidrug resistance-associated protein 4-like
RS006648	GO:0055085	transmembrane transport	BP	-1.472287059	-1.627468546	0.001448714	3.845832603	45.04057983	9.07077E-10	1.03664E-08	PREDICTED: voltage-dependent L-type calcium channel subunit beta-2
RS006270	GO:0055085	transmembrane transport	BP	-0.625556145	-0.022131593	2.665286326	2.775447041	44.95816183	9.44409E-10	1.07832E-08	PREDICTED: organic cation transporter 1
RS004437	GO:0055085	transmembrane transport	BP	-1.425888965	-2.280207862	-0.816193317	4.489021524	44.20951888	1.36218E-09	1.53172E-08	Monocarboxylate transporter 9
RS011778	GO:0055085	transmembrane transport	BP	-1.482690204	-0.163981472	-1.627972092	5.097898358	44.03120475	1.48632E-09	1.66834E-08	PREDICTED: facilitated trehalose transporter Tret1-2 homolog
RS005640	GO:0055085	transmembrane transport	BP	0.969003673	1.718434974	-0.876644168	4.401662752	43.66119041	1.78117E-09	1.9764E-08	PREDICTED: glycine receptor subunit alpha-4
RS009578	GO:0055085	transmembrane transport	BP	-2.77838182	-2.247321578	-2.454512864	4.019688809	42.39569844	3.30671E-09	3.59937E-08	PREDICTED: solute carrier family 28 member 3
RS001007	GO:0055085	transmembrane transport	BP	-0.057453873	2.550863666	4.593751253	0.34449975	41.36132174	5.4813E-09	5.84023E-08	PREDICTED: sodium-dependent nutrient amino acid transporter 1-like
RS010452	GO:0055085	transmembrane transport	BP	-1.264493454	-3.411377807	-0.656775569	3.802175748	40.96006363	6.66799E-09	7.05094E-08	PREDICTED: transient receptor potential cation channel protein painless
RS014047	GO:0055085	transmembrane transport	BP	-0.592092988	1.748032789	5.355436168	1.817327481	40.93864928	6.73809E-09	7.11311E-08	PREDICTED: zinc transporter ZIP1-like
RS005851	GO:0055085	transmembrane transport	BP	0.526397294	1.41413322	1.673681755	7.829535198	40.74316203	7.413E-09	7.77347E-08	PREDICTED: proton-coupled amino acid transporter 4
RS012191	GO:0055085	transmembrane transport	BP	-0.827958312	0.960594434	-0.015691014	7.391207833	39.39645508	1.43044E-08	1.45634E-07	PREDICTED: V-type proton ATPase 116 kDa subunit a
RS009947	GO:0055085	transmembrane transport	BP	-0.133867435	1.065636208	0.708920051	6.792021792	39.18991216	1.58209E-08	1.60039E-07	PREDICTED: calcium-binding mitochondrial carrier protein SCaMC-2
RS011831	GO:0055085	transmembrane transport	BP	-1.435266558	0.383547956	3.056810998	0.766698698	36.38839562	6.19812E-08	5.87352E-07	PREDICTED: P protein-like
RS014465	GO:0055085	transmembrane transport	BP	-2.203563111	-3.568610423	-0.279641755	5.849726949	35.76065503	8.4137E-08	7.87237E-07	PREDICTED: probable multidrug resistance-associated protein lethal(2)03659
RS011779	GO:0055085	transmembrane transport	BP	-2.22356949	-3.571952783	-1.230326148	2.104606825	35.6579628	8.84493E-08	8.24523E-07	PREDICTED: facilitated trehalose transporter Tret1-2 homolog
RS011079	GO:0055085	transmembrane transport	BP	1.606302982	0.958734861	0.270199034	7.619499542	35.24300998	1.08241E-07	1.0016E-06	PREDICTED: monocarboxylate transporter 12
RS012786	GO:0055085	transmembrane transport	BP	-1.725772589	-2.922030511	-0.315113572	3.189291251	34.971992	1.23496E-07	1.13444E-06	PREDICTED: facilitated trehalose transporter Tret1-2 homolog
RS004248	GO:0055085	transmembrane transport	BP	-2.357800174	-1.138542703	0.799750155	3.47254249	34.60029849	1.47968E-07	1.34356E-06	PREDICTED: facilitated trehalose transporter Tret1-like
RS009116	GO:0055085	transmembrane transport	BP	-2.774708917	-1.965598127	1.306984186	1.044379091	34.54483465	1.52014E-07	1.3783E-06	PREDICTED: urea transporter 1-like
RS011418	GO:0055085	transmembrane transport	BP	-0.328802728	1.522013603	-0.732215375	3.957425952	34.42904716	1.60818E-07	1.45395E-06	PREDICTED: putative inorganic phosphate cotransporter
RS000365	GO:0055085	transmembrane transport	BP	-2.043121889	-2.26896537	0.189661525	11.00673959	34.00204383	1.97922E-07	1.77664E-06	PREDICTED: calcium-transporting ATPase sarcoplasmic/endoplasmic reticulum type
RS007032	GO:0055085	transmembrane transport	BP	-1.972036202	-0.832866298	0.156731051	5.195017285	33.86656984	2.11394E-07	1.89487E-06	PREDICTED: ammonium transporter Rh type A
RS001722	GO:0055085	transmembrane transport	BP	-0.334283753	-2.198611554	-0.278419111	3.485639997	32.69185314	3.74056E-07	3.27598E-06	PREDICTED: solute carrier family 41 member 1-like
RS007693	GO:0055085	transmembrane transport	BP	-0.242378739	-0.621851837	1.493665703	5.076496585	32.00947491	5.20946E-07	4.51534E-06	PREDICTED: zinc transporter ZIP14
RS004347	GO:0055085	transmembrane transport	BP	-0.916171765	-2.042094379	0.238772477	3.454218425	31.9850429	5.27159E-07	4.55665E-06	PREDICTED: organic cation transporter protein-like
RS013209	GO:0055085	transmembrane transport	BP	-0.459455181	-0.353978647	1.234885473	6.428228507	31.52472151	6.59072E-07	5.61974E-06	PREDICTED: sodium-coupled monocarboxylate transporter 1
RS006814	GO:0055085	transmembrane transport	BP	-0.514796382	1.014347124	0.975336891	4.041760153	30.97725487	8.59474E-07	7.23543E-06	PREDICTED: large neutral amino acids transporter small subunit 2
RS006320	GO:0055085	transmembrane transport	BP	1.668458777	1.549661536	0.932473469	6.183206129	30.51118393	1.07731E-06	8.96741E-06	PREDICTED: bumetanide-sensitive sodium-(potassium)-chloride cotransporter
RS007713	GO:0055085	transmembrane transport	BP	-1.593472957	-0.392083561	-0.180235411	5.938924251	30.40817878	1.13245E-06	9.40771E-06	PREDICTED: ATP-binding cassette sub-family D member 2
RS003904	GO:0055085	transmembrane transport	BP	-2.706690593	-2.534495324	0.490780854	4.216110096	30.29657835	1.19537E-06	9.89777E-06	PREDICTED: facilitated trehalose transporter Tret1-like
RS010500	GO:0055085	transmembrane transport	BP	1.19026545	2.873457875	1.841995752	12.00045446	30.1935168	1.25657E-06	1.03772E-05	PREDICTED: tubulin alpha-1 chain
RS000193	GO:0055085	transmembrane transport	BP	-0.612906575	0.552707825	0.864925878	6.395375097	30.19397061	1.25629E-06	1.03772E-05	PREDICTED: b(0,+)-type amino acid transporter 1
RS000996	GO:0055085	transmembrane transport	BP	-1.508231266	-0.156640833	0.876498273	2.831732428	29.09435446	2.13949E-06	1.70644E-05	PREDICTED: zinc transporter ZIP3-like
RS005491	GO:0055085	transmembrane transport	BP	-0.937465516	-1.630388798	-0.209059704	4.107611972	28.88192352	2.37108E-06	1.88281E-05	PREDICTED: voltage-gated potassium channel subunit beta-2
RS009579	GO:0055085	transmembrane transport	BP	-0.361377028	0.937501921	0.004604885	5.160969196	28.85056945	2.40732E-06	1.91039E-05	PREDICTED: solute carrier family 28 member 3-like
RS011850	GO:0055085	transmembrane transport	BP	-0.767278606	-0.313949384	0.713484854	8.083358391	28.67013092	2.62688E-06	2.07027E-05	PREDICTED: trimeric intracellular cation channel type B
RS008885	GO:0055085	transmembrane transport	BP	-1.592550754	-2.358375405	-0.949039702	4.983900477	28.41800515	2.96751E-06	2.32998E-05	PREDICTED: facilitated trehalose transporter Tret1-2 homolog
RS010090	GO:0055085	transmembrane transport	BP	0.323234855	1.546465547	-0.132352818	2.938456182	28.16134763	3.35955E-06	2.60531E-05	PREDICTED: cyclic nucleotide-gated cation channel subunit A-like
RS008631	GO:0055085	transmembrane transport	BP	-0.45372484	0.018443887	2.172529191	1.173123173	28.08258513	3.48992E-06	2.69976E-05	PREDICTED: glutamate receptor ionotropic, delta-1-like
RS008887	GO:0055085	transmembrane transport	BP	-0.760329305	-4.339267866	-1.430676324	3.320036492	27.63642804	4.32958E-06	3.3078E-05	PREDICTED: facilitated trehalose transporter Tret1-like
RS006124	GO:0055085	transmembrane transport	BP	1.059219965	0.250242816	-0.925285807	3.554006542	27.48741307	4.65272E-06	3.54057E-05	PREDICTED: putative sodium-dependent multivitamin transporter
RS006269	GO:0055085	transmembrane transport	BP	-1.78180142	-1.191554952	-0.830372469	4.550094908	27.39894348	4.85584E-06	3.68178E-05	PREDICTED: organic cation transporter 1
RS012590	GO:0055085	transmembrane transport	BP	-0.22021637	0.861437679	0.641734274	6.761709937	27.36974453	4.9248E-06	3.72958E-05	PREDICTED: high affinity copper uptake protein 1
RS012741	GO:0055085	transmembrane transport	BP	-1.060191661	0.585439948	0.026171952	4.628484614	26.6687965	6.90781E-06	5.10845E-05	PREDICTED: organic cation transporter protein
RS000895	GO:0055085	transmembrane transport	BP	1.014348253	1.385977981	0.68384576	7.930053973	26.65746374	6.94569E-06	5.13345E-05	PREDICTED: translocon-associated protein subunit beta
RS014872	GO:0055085	transmembrane transport	BP	0.103900993	0.11224879	1.250966703	5.119341431	25.84071442	1.02986E-05	7.43747E-05	PREDICTED: putative inorganic phosphate cotransporter
RS003902	GO:0055085	transmembrane transport	BP	0.212010943	1.72144821	0.98329227	4.242513254	25.33598899	1.31339E-05	9.3504E-05	PREDICTED: facilitated trehalose transporter Tret1
RS013310	GO:0055085	transmembrane transport	BP	-1.594173173	0.30942411	0.502900611	3.784418211	25.32704004	1.31907E-05	9.38549E-05	PREDICTED: sodium- and chloride-dependent transporter XTRP3
RS005461	GO:0055085	transmembrane transport	BP	-1.570191565	-0.758659476	-0.541009138	7.57624513	24.31985122	2.1419E-05	0.000146843	PREDICTED: solute carrier family 22 member 21-like
RS003139	GO:0055085	transmembrane transport	BP	-0.462942241	-0.484276182	0.670801183	4.994154039	24.0689115	2.4166E-05	0.000164334	PREDICTED: zinc transporter 1
RS006319	GO:0055085	transmembrane transport	BP	-1.264071762	-1.378688832	-0.296664377	4.499770473	23.99784591	2.50059E-05	0.000169587	PREDICTED: bumetanide-sensitive sodium-(potassium)-chloride cotransporter
RS008934	GO:0055085	transmembrane transport	BP	-2.249974024	-1.610242152	-0.986553809	2.596021099	23.90344612	2.61667E-05	0.000177078	ionotropic receptor 25a
RS014152	GO:0055085	transmembrane transport	BP	-1.430499389	-1.354570713	-0.671668718	4.505777329	23.40073256	3.33153E-05	0.000221644	PREDICTED: potassium channel subfamily K member 1-like
RS012504	GO:0055085	transmembrane transport	BP	1.701019285	0.103598753	-0.952358557	2.60795793	23.14266371	3.77102E-05	0.000248475	PREDICTED: zinc transporter ZIP3
RS007091	GO:0055085	transmembrane transport	BP	-0.260344997	-0.000107201	0.982798512	6.405504865	23.00178887	4.03484E-05	0.000263836	PREDICTED: probable multidrug resistance-associated protein lethal(2)03659
RS014340	GO:0055085	transmembrane transport	BP	-0.92887959	-0.092715464	0.219765138	5.620832256	22.82606695	4.38983E-05	0.00028483	PREDICTED: ileal sodium/bile acid cotransporter-like
RS011507	GO:0055085	transmembrane transport	BP	0.081672207	0.439418944	1.152509484	5.150989282	22.16598652	6.02438E-05	0.000382801	PREDICTED: sialin
RS003117	GO:0055085	transmembrane transport	BP	-1.023316483	-1.406983411	-1.703899952	3.289864327	21.79101183	7.20993E-05	0.000451301	PREDICTED: sodium/myo-inositol cotransporter
RS005054	GO:0055085	transmembrane transport	BP	-0.882564902	-1.772840386	-2.09465887	2.914764324	21.56658479	8.02786E-05	0.000498781	PREDICTED: ETS domain-containing protein Elk-3-like
RS012462	GO:0055085	transmembrane transport	BP	-1.887439376	-1.068323927	-1.822700455	2.935725927	21.16526065	9.72757E-05	0.000593839	PREDICTED: synaptic vesicle glycoprotein 2B
RS012046	GO:0055085	transmembrane transport	BP	-0.044612109	1.318321788	-0.182662099	3.873303092	21.0021542	0.000105168	0.000636465	PREDICTED: sodium-independent sulfate anion transporter-like
RS015154	GO:0055085	transmembrane transport	BP	0.77118859	1.275597924	0.795023023	8.092063536	20.820501	0.00011471	0.000690232	PREDICTED: protein transport protein Sec61 subunit gamma
RS015395	GO:0055085	transmembrane transport	BP	-1.227128143	-0.999149743	-0.38673491	5.789022217	20.66182653	0.000123747	0.000741733	PREDICTED: ryanodine receptor 44F
RS000851	GO:0055085	transmembrane transport	BP	-2.325107834	-2.129776094	-2.544043742	0.519579293	20.5603303	0.000129896	0.000774215	PREDICTED: monocarboxylate transporter 14-like
RS010854	GO:0055085	transmembrane transport	BP	-0.464601417	0.421962906	0.417617417	6.7210824	20.51691341	0.000132619	0.000789321	PREDICTED: V-type proton ATPase subunit e 2-like
RS006273	GO:0055085	transmembrane transport	BP	-0.841668922	-0.780206767	0.412687786	4.250127088	20.41178555	0.000139449	0.000826843	PREDICTED: organic cation transporter 1
RS014287	GO:0055085	transmembrane transport	BP	-0.825809928	1.486619083	1.592270521	1.389357496	20.39684624	0.000140447	0.000831501	PREDICTED: sialin
RS007340	GO:0055085	transmembrane transport	BP	1.72289566	2.228109689	-0.488523344	0.853368064	20.24210443	0.000151218	0.000888679	PREDICTED: mitochondrial sodium/hydrogen exchanger 9B2
RS004393	GO:0055085	transmembrane transport	BP	-1.227790861	-2.264108903	-0.817189459	2.289519889	20.23673107	0.000151606	0.000890546	PREDICTED: putative tricarboxylate transport protein, mitochondrial
RS015388	GO:0055085	transmembrane transport	BP	-2.602050655	-3.074067005	-0.457367858	0.547921075	20.16611512	0.000156804	0.000918937	PREDICTED: glutamate receptor ionotropic, delta-1
RS000135	GO:0055085	transmembrane transport	BP	-0.631745801	-0.940404648	0.237026994	4.32458574	20.11793543	0.000160452	0.000937697	PREDICTED: putative ammonium transporter 1
RS011787	GO:0055085	transmembrane transport	BP	-0.438940613	0.619762966	1.077554296	6.634288702	19.98955295	0.000170591	0.000992342	Zinc finger protein 782
RS006499	GO:0055085	transmembrane transport	BP	-1.047637087	0.301924919	0.728056025	3.319116729	19.94751042	0.000174047	0.001009187	PREDICTED: transient receptor potential cation channel subfamily V member 5
RS015396	GO:0055085	transmembrane transport	BP	-1.256182982	-1.010805264	-0.293857127	6.048626436	19.73517564	0.0001926	0.00110506	PREDICTED: ryanodine receptor 44F
RS008662	GO:0055085	transmembrane transport	BP	-0.323694319	-0.887991823	-0.04413226	5.702043181	19.66407708	0.000199242	0.001138501	PREDICTED: transient receptor potential cation channel subfamily V member 5
RS005651	GO:0055085	transmembrane transport	BP	-0.219646792	-1.283329628	-0.275521678	5.060327004	19.22158667	0.000246019	0.001380116	PREDICTED: transient-receptor-potential-like protein
RS006140	GO:0055085	transmembrane transport	BP	0.033292599	1.273516036	1.00013418	2.720807394	19.17927372	0.000251027	0.00140446	PREDICTED: potassium channel subfamily K member 18
RS002503	GO:0055085	transmembrane transport	BP	-0.009517009	0.124352347	0.864548115	5.245674668	19.05817022	0.000265931	0.001479298	PREDICTED: sugar phosphate exchanger 2
RS003726	GO:0055085	transmembrane transport	BP	-0.35022698	0.054528944	0.659374478	4.976150075	18.98906357	0.000274826	0.001524592	PREDICTED: mitochondrial coenzyme A transporter SLC25A42
RS000152	GO:0055085	transmembrane transport	BP	-1.205241827	-0.522965397	-0.166378556	6.69155609	18.98450384	0.000275423	0.001526708	PREDICTED: synaptic vesicle glycoprotein 2B-like
RS007614	GO:0055085	transmembrane transport	BP	0.699165628	-0.052782673	-0.817546641	5.472681929	18.94140065	0.000281133	0.001554478	PREDICTED: facilitated trehalose transporter Tret1-like
RS007694	GO:0055085	transmembrane transport	BP	-1.402205119	-1.429007484	-0.154037672	3.235518975	18.72862507	0.000311092	0.001703432	PREDICTED: proton myo-inositol cotransporter-like
RS009272	GO:0055085	transmembrane transport	BP	-0.152985688	-1.205701524	-0.53386679	4.590610282	18.71946093	0.000312451	0.001710132	PREDICTED: mitochondrial sodium/hydrogen exchanger 9B2
RS013136	GO:0055085	transmembrane transport	BP	-0.851364149	-0.302892822	0.222344744	5.914157083	18.57902045	0.000334038	0.001818795	PREDICTED: sialin
RS009604	GO:0055085	transmembrane transport	BP	-1.648345631	0.550771769	0.526514629	2.368500525	18.41833332	0.000360561	0.001945804	PREDICTED: sodium-dependent noradrenaline transporter
RS010007	GO:0055085	transmembrane transport	BP	-0.719830127	-2.070294597	-0.711910489	2.61204502	18.21562653	0.000397027	0.002115128	PREDICTED: monocarboxylate transporter 9-like
RS014410	GO:0055085	transmembrane transport	BP	-0.538555444	-0.069436224	0.424307658	5.574699261	17.86202393	0.00046963	0.002466436	PREDICTED: sodium- and chloride-dependent neutral and basic amino acid transporter B(0+)
RS000837	GO:0055085	transmembrane transport	BP	-1.260815808	-0.582381283	0.014148923	5.012164404	17.85146996	0.000471988	0.002476758	PREDICTED: excitatory amino acid transporter 1-like
RS003799	GO:0055085	transmembrane transport	BP	-0.143761693	0.372761559	-0.639546518	5.875204513	17.46636866	0.000566606	0.002928125	PREDICTED: ATP-binding cassette sub-family C member Sur
RS007085	GO:0055085	transmembrane transport	BP	-2.123520843	-1.442400071	-1.654436852	1.159522622	17.26753428	0.000622612	0.00317714	PREDICTED: cyclic nucleotide-gated cation channel alpha-3
RS000849	GO:0055085	transmembrane transport	BP	-0.753175709	-1.571391853	-1.058453667	2.708513766	17.06922787	0.000683946	0.003464852	PREDICTED: monocarboxylate transporter 9-like
RS012432	GO:0055085	transmembrane transport	BP	-1.318193047	-1.41640057	-1.093486391	3.821570882	17.02394884	0.000698772	0.003535692	PREDICTED: acetylcholine receptor subunit alpha-type acr-16-like
RS013332	GO:0055085	transmembrane transport	BP	-1.915754341	-0.761067399	-0.092972296	1.96508851	16.98236489	0.000712669	0.003598779	PREDICTED: potassium channel subfamily T member 2
RS004044	GO:0055085	transmembrane transport	BP	-0.216859326	0.637026253	1.004892576	2.871056733	16.9549321	0.000721987	0.003638536	PREDICTED: glutamate-gated chloride channel
RS007073	GO:0055085	transmembrane transport	BP	-0.603049329	-0.874713843	-0.994791182	5.376371075	16.7683237	0.000788663	0.003950846	PREDICTED: major facilitator superfamily domain-containing protein 6

RS012047	GO:0055085	transmembrane transport	BP	-1.033691566	-1.204387105	-0.573210977	3.717195265	16.53395031	0.000881135	0.004370625	PREDICTED: sodium-independent sulfate anion transporter-like
RS010364	GO:0055085	transmembrane transport	BP	-0.052944401	0.405459181	0.815688442	7.091949691	16.43172938	0.000924767	0.004565472	PREDICTED: cytochrome c oxidase subunit 7A2, mitochondrial
RS002855	GO:0055085	transmembrane transport	BP	-1.74839573	-1.535258414	-0.16366084	1.79539144	16.39328726	0.000941725	0.00463647	PREDICTED: glycine receptor subunit alpha-3
RS000927	GO:0055085	transmembrane transport	BP	-2.570527298	-2.804707147	-0.755163456	0.667619183	16.35029216	0.000961058	0.004715063	PREDICTED: b(0,+)-type amino acid transporter 1-like
RS009094	GO:0055085	transmembrane transport	BP	0.810509918	0.868482563	0.707370764	6.315762678	16.22341499	0.001020437	0.004986955	PREDICTED: mitochondrial import inner membrane translocase subunit Tim17-B-like
RS003544	GO:0055085	transmembrane transport	BP	-0.410771227	-0.348918681	1.010423069	3.516674428	16.0029682	0.001132396	0.005455777	PREDICTED: glutamate receptor ionotropic, kainate 2
RS006141	GO:0055085	transmembrane transport	BP	0.186642093	0.509454722	0.888482209	5.536921472	15.98603197	0.001141486	0.005494665	Potassium channel subfamily K member 9
RS003914	GO:0055085	transmembrane transport	BP	-0.408304954	-1.308021582	-1.113950825	3.842131166	15.96402321	0.001153407	0.005540047	PREDICTED: facilitated trehalose transporter Tret1-like
RS013870	GO:0055085	transmembrane transport	BP	-1.185159015	-0.789079424	-0.942978819	4.024142004	15.86729932	0.001207277	0.005765813	PREDICTED: sodium- and chloride-dependent glycine transporter 1-like
RS013164	GO:0055085	transmembrane transport	BP	-0.720894807	-1.573394185	-0.567112816	2.35012097	15.85215695	0.001215934	0.005800558	PREDICTED: sodium/hydrogen exchanger 3
RS014761	GO:0055085	transmembrane transport	BP	0.850386459	0.827647699	0.741742978	6.600879234	15.79201097	0.001250932	0.005936034	PREDICTED: mitochondrial import receptor subunit TOM40 homolog 1
RS007887	GO:0055085	transmembrane transport	BP	0.740875807	0.982728981	0.368061586	5.30134597	15.7441554	0.001279491	0.006053309	PREDICTED: mitochondrial thiamine pyrophosphate carrier-like
RS012806	GO:0055085	transmembrane transport	BP	-0.671548237	-0.771532863	-0.831977584	6.320380877	15.5512679	0.001401314	0.006555774	PREDICTED: glutamate [NMDA] receptor subunit 1
RS004534	GO:0055085	transmembrane transport	BP	-0.580382142	2.035273319	0.497901415	1.025113205	15.51468926	0.001425682	0.006654944	PREDICTED: sodium channel protein Nach-like
RS007810	GO:0055085	transmembrane transport	BP	-0.820216184	-1.018356117	-0.958242629	3.620004773	15.51103249	0.001428141	0.006659019	PREDICTED: vesicular glutamate transporter 1
RS000928	GO:0055085	transmembrane transport	BP	-2.818387407	-2.387215456	-1.195316578	0.324906115	15.29049888	0.001584497	0.007309578	B(0,+)-type amino acid transporter 1
RS006610	GO:0055085	transmembrane transport	BP	0.2664942	0.92285009	0.741592285	5.324734931	15.2782684	0.001593649	0.007342643	PREDICTED: zinc transporter 7
RS008896	GO:0055085	transmembrane transport	BP	-1.274733506	-1.006034401	-0.767037953	2.62762816	15.13459455	0.001705167	0.007799012	PREDICTED: mitoferrin-1
RS011193	GO:0055085	transmembrane transport	BP	0.23153241	-0.655301507	0.610210215	6.072153027	15.07588643	0.001752934	0.007978478	PREDICTED: organic cation transporter protein
RS006757	GO:0055085	transmembrane transport	BP	-0.233100515	0.411940029	0.749443033	4.26814335	14.98825356	0.001826715	0.008260555	PREDICTED: cell death-inducing p53-target protein 1
RS009126	GO:0055085	transmembrane transport	BP	0.004334845	0.550905396	0.691970165	7.068681097	14.88906784	0.001913944	0.008599435	PREDICTED: V-type proton ATPase subunit F
RS001526	GO:0055085	transmembrane transport	BP	-0.188683928	0.519017421	0.650345185	7.644232128	14.87803596	0.001923898	0.008634916	PREDICTED: V-type proton ATPase subunit G
RS006318	GO:0055085	transmembrane transport	BP	-3.618949223	-2.702749807	-1.072386578	0.467850565	14.73641507	0.002056334	0.009147816	PREDICTED: bumetanide-sensitive sodium-(potassium)-chloride cotransporter
RS005708	GO:0055085	transmembrane transport	BP	-2.57707626	-0.767810406	-0.121337824	1.208165742	14.61680361	0.002175202	0.009598631	PREDICTED: sodium- and chloride-dependent glycine transporter 1-like
RS013194	GO:0055085	transmembrane transport	BP	-0.402235939	1.468418978	0.620702468	1.800456379	14.57025873	0.002223278	0.009779937	PREDICTED: gamma-aminobutyric acid receptor alpha-like
RS008446	GO:0055085	transmembrane transport	BP	0.363470434	-0.27703378	-1.408383647	4.990252537	14.55183725	0.002242595	0.009858024	PREDICTED: facilitated trehalose transporter Tret1-2 homolog
RS012689	GO:0055085	transmembrane transport	BP	-0.335162446	-0.922302748	-1.067051914	4.164281375	14.43562454	0.002368352	0.010353914	PREDICTED: solute carrier family 41 member 1-like
RS014548	GO:0055085	transmembrane transport	BP	-0.284980468	-1.173909226	-1.493304567	2.859916837	14.21947648	0.00262113	0.011285646	PREDICTED: MFS-type transporter SLC18B1-like
RS005190	GO:0055085	transmembrane transport	BP	-0.561761317	0.20318587	0.563166122	6.506172496	14.19948267	0.002645822	0.011376401	PREDICTED: calcium-activated potassium channel slowpoke
RS013212	GO:0055085	transmembrane transport	BP	-0.878244991	-0.110123051	0.018139925	7.487946573	14.16068921	0.002694389	0.011553674	PREDICTED: V-type proton ATPase subunit B
RS011379	GO:0055085	transmembrane transport	BP	-0.945895165	-0.137739549	0.02413553	6.197611827	14.11352967	0.002754622	0.01177587	PREDICTED: chloride intracellular channel exc-4
RS014046	GO:0055085	transmembrane transport	BP	-0.827652517	0.123541367	0.39500677	4.371003694	13.78924417	0.003206526	0.0134386	PREDICTED: zinc transporter ZIP1-like
RS004592	GO:0055085	transmembrane transport	BP	-0.294046429	0.156068735	0.564893198	6.831343324	13.651508	0.003420014	0.014243271	PREDICTED: ATP-binding cassette sub-family B member 7, mitochondrial
RS009681	GO:0055085	transmembrane transport	BP	-0.687137615	-0.85904435	-0.276874723	5.270172907	13.64338362	0.003433037	0.014288059	PREDICTED: peroxisomal membrane protein PMP34
RS006255	GO:0055085	transmembrane transport	BP	-0.565848028	-0.409978879	0.214379631	6.839035823	13.48918712	0.003689736	0.015225603	PREDICTED: copper-transporting ATPase 1
RS012010	GO:0055085	transmembrane transport	BP	-0.658055692	-0.163236513	0.25753802	6.662027841	13.45566221	0.003748014	0.01543574	PREDICTED: sodium-dependent phosphate transporter 1-B-like
RS010731	GO:0055085	transmembrane transport	BP	-0.395182325	-0.639006133	-0.675231861	6.20607259	13.34648856	0.003944186	0.016153848	PREDICTED: V-type proton ATPase 116 kDa subunit a
RS006343	GO:0055085	transmembrane transport	BP	0.700005517	0.678494058	0.385858572	5.661988764	13.33113205	0.003972583	0.016264862	PREDICTED: zinc transporter 6
RS010905	GO:0055085	transmembrane transport	BP	-0.573248945	-0.768712677	-0.837001645	4.71486708	13.23543162	0.00415417	0.016871208	PREDICTED: gamma-aminobutyric acid receptor subunit beta
RS015097	GO:0055085	transmembrane transport	BP	0.838222973	0.450337326	-0.261713518	6.777317829	13.04891082	0.004532018	0.018148247	PREDICTED: tubulin alpha-1 chain
RS014000	GO:0055085	transmembrane transport	BP	0.486986842	0.493065666	-0.511287156	7.326747337	12.99389808	0.00464982	0.018525729	TWiK family of potassium channels protein 18
RS007615	GO:0055085	transmembrane transport	BP	0.842199769	0.08001472	-0.354074907	7.382223287	12.96671251	0.004709147	0.018738389	PREDICTED: facilitated trehalose transporter Tret1-like
RS005166	GO:0055085	transmembrane transport	BP	0.797745292	0.959053152	1.137980998	8.88871257	12.83598972	0.005005051	0.019744379	PREDICTED: nascent polypeptide-associated complex subunit alpha
RS005460	GO:0055085	transmembrane transport	BP	-0.996149896	-0.953790558	-0.537751487	3.769830749	12.77993102	0.005137514	0.020193827	PREDICTED: solute carrier family 22 member 21-like
RS005241	GO:0055085	transmembrane transport	BP	-1.24648029	-0.946331544	-0.692073388	4.41212653	12.5745733	0.005653054	0.021933014	PREDICTED: high-affinity choline transporter 1
RS006299	GO:0055085	transmembrane transport	BP	0.929966764	0.90845916	-0.169322471	3.276482954	12.53656321	0.005753939	0.022276393	PREDICTED: facilitated trehalose transporter Tret1-like
RS008051	GO:0055085	transmembrane transport	BP	-0.405673123	-1.320265794	-1.066020494	4.63196021	12.49923358	0.00585475	0.022555742	PREDICTED: transient receptor potential channel pyrexia
RS000227	GO:0055085	transmembrane transport	BP	-1.32952285	-0.75353739	-1.12184367	1.981758767	12.38833226	0.00616466	0.023605269	PREDICTED: facilitated trehalose transporter Tret1-2 homolog
RS012250	GO:0055085	transmembrane transport	BP	-1.126562781	-0.381786444	0.539034757	1.985904525	12.14501042	0.00690257	0.026042829	PREDICTED: organic cation transporter protein-like
RS002985	GO:0055085	transmembrane transport	BP	-0.571114377	-0.476136182	-0.859409917	5.548946497	12.07709757	0.007123669	0.026732864	PREDICTED: Y+L amino acid transporter 2
RS003288	GO:0055085	transmembrane transport	BP	-0.468141457	-0.663116971	0.331112622	4.523147937	12.02307867	0.007304519	0.027292266	PREDICTED: sodium- and chloride-dependent GABA transporter ine
RS011665	GO:0055085	transmembrane transport	BP	-0.460489579	-1.326999046	-1.061984026	2.228596867	11.95023512	0.007555593	0.028094223	PREDICTED: vesicular inhibitory amino acid transporter
RS000866	GO:0055085	transmembrane transport	BP	-0.851954648	-0.389874807	-0.092110043	7.989972168	11.90615845	0.007711634	0.028547895	PREDICTED: sodium/potassium-transporting ATPase subunit alpha
RS003756	GO:0055085	transmembrane transport	BP	-0.408342321	-0.364800511	-0.786353931	7.1095919	11.79402076	0.00812312	0.029860645	PREDICTED: multidrug resistance-associated protein 1
RS015096	GO:0055085	transmembrane transport	BP	-0.857980934	-0.446788274	-0.194471442	5.697782265	11.71197794	0.008437836	0.030900291	PREDICTED: V-type proton ATPase subunit D
RS008458	GO:0055085	transmembrane transport	BP	-1.182823715	-0.196910535	-0.390818396	8.429616967	11.6884148	0.008530428	0.031131399	PREDICTED: probable cation-transporting ATPase 13A3
RS014992	GO:0055085	transmembrane transport	BP	0.229136004	0.615757621	0.676877541	5.913523856	11.67233841	0.008594176	0.031318014	PREDICTED: sodium-dependent phosphate transport protein 1, chloroplastic-like
RS011196	GO:0055085	transmembrane transport	BP	0.675575742	0.753360958	0.761120595	5.457606567	11.66397815	0.008627513	0.031412228	PREDICTED: 39S ribosomal protein L19, mitochondrial
RS004997	GO:0055085	transmembrane transport	BP	-0.851696275	-1.530670443	-0.345032758	2.982640462	11.64463017	0.008705154	0.031658304	PREDICTED: kinesin-like protein KIF17
RS002363	GO:0055085	transmembrane transport	BP	-0.329275879	-0.729371826	0.101654401	6.167217704	11.55948198	0.009055119	0.032732541	PREDICTED: multidrug resistance-associated protein 5
RS008898	GO:0055085	transmembrane transport	BP	-0.172960297	-0.478698026	-0.774628888	4.936093792	11.53878925	0.009142244	0.033009582	PREDICTED: sideroflexin-1
RS002295	GO:0055085	transmembrane transport	BP	-0.760632417	-0.881718429	-1.336627404	7.31207469	11.50237509	0.009297576	0.033436228	PREDICTED: sodium- and chloride-dependent GABA transporter 1-like
RS012787	GO:0055085	transmembrane transport	BP	-1.042887095	-1.14108662	-0.848878197	2.917253142	11.38739346	0.009805347	0.03491082	PREDICTED: facilitated trehalose transporter Tret1-2 homolog
RS000505	GO:0055085	transmembrane transport	BP	-0.550172818	-0.780268786	-0.465996455	5.329225797	11.32824795	0.010077099	0.035769725	PREDICTED: synaptic vesicular amine transporter
RS000219	GO:0055085	transmembrane transport	BP	0.217052434	-0.673773561	-1.028588766	6.485154642	11.32601418	0.010087507	0.035776418	PREDICTED: innexin inx2
RS014273	GO:0055085	transmembrane transport	BP	-0.268192968	-0.584630007	0.366094757	4.564432181	11.2907457	0.010253243	0.036262102	PREDICTED: sialin-like
RS007389	GO:0055085	transmembrane transport	BP	-0.71821859	-0.059688714	0.178211598	8.141021926	11.17756615	0.010803454	0.037931058	PREDICTED: V-type proton ATPase catalytic subunit A
RS007388	GO:0055085	transmembrane transport	BP	-1.012601041	-0.502641916	-0.211964145	6.238906794	11.11536226	0.011118147	0.038884183	PREDICTED: V-type proton ATPase catalytic subunit A
RS006740	GO:0055085	transmembrane transport	BP	-0.43064001	-0.612335737	-0.738849827	6.295225865	10.98541247	0.011805018	0.040967869	PREDICTED: glutamate-gated chloride channel
RS014286	GO:0055085	transmembrane transport	BP	-1.116813321	-0.446472365	-0.712367183	4.323236502	10.94398368	0.012032666	0.041654588	PREDICTED: sialin
RS002816	GO:0055085	transmembrane transport	BP	-0.748548229	0.030574886	-0.160819942	5.861566925	10.7303973	0.013276729	0.045264516	PREDICTED: multidrug resistance protein 1-like
RS012007	GO:0055085	transmembrane transport	BP	-0.8805953	0.446042174	-0.616249485	2.603532973	10.7020781	0.013450915	0.045771636	PREDICTED: sodium-dependent phosphate transporter 2
RS002237	GO:0055085	transmembrane transport	BP	-0.77443485	-0.782975074	-0.864409769	3.087700691	10.69522479	0.013493406	0.045891426	PREDICTED: transient receptor potential cation channel protein painless
RS014643	GO:0055085	transmembrane transport	BP	0.254581862	0.726345297	-0.48680769	3.008929688	10.68030533	0.013586364	0.046182636	PREDICTED: monocarboxylate transporter 12-like
RS010501	GO:0055085	transmembrane transport	BP	-0.249581193	0.164542324	0.830736506	8.23731792	10.57427827	0.014265398	0.048114201	PREDICTED: ATP synthase subunit e, mitochondrial
RS013211	GO:0055085	transmembrane transport	BP	-0.46592983	-1.425136997	0.127678346	1.648820506	10.50692227	0.014714013	0.049415541	PREDICTED: pickpocket protein 19-like
RS005829	GO:0055085	transmembrane transport	BP	-0.565901365	-0.398114437	0.075675202	5.880326068	10.47839461	0.014908177	0.04996104	PREDICTED: facilitated trehalose transporter Tret1-like
RS012348	GO:0055085	transmembrane transport	BP	-1.171136152	-0.623448827	-0.241561177	4.399276235	10.47489738	0.014932153	0.050028076	V-type proton ATPase subunit S1
RS004574	GO:0055085	transmembrane transport	BP	-0.256414135	0.160648707	0.765161971	8.179741228	10.46991438	0.01496638	0.050122195	PREDICTED: ATP synthase subunit delta, mitochondrial
RS005209	GO:0055085	transmembrane transport	BP	-0.863563851	-1.023827568	-0.616859801	5.79782502	10.40323263	0.015431901	0.051374401	PREDICTED: multidrug resistance protein homolog 49
RS012105	GO:0055085	transmembrane transport	BP	-0.587037702	-0.604268783	0.220956679	3.910659327	10.37503836	0.015632991	0.051906642	Inward rectifier potassium channel 2
RS000868	GO:0055085	transmembrane transport	BP	-0.929392451	-0.475108864	-0.289097862	8.324170965	10.36013171	0.01574035	0.052235565	PREDICTED: sodium/potassium-transporting ATPase subunit alpha
RS005239	GO:0055085	transmembrane transport	BP	-0.442689839	-0.720832595	-0.092125906	4.79919063	10.32683206	0.015982804	0.05290077	PREDICTED: vesicular acetylcholine transporter
RS008445	GO:0055085	transmembrane transport	BP	-0.794376386	-2.696738816	-1.11925367	1.261432481	10.25254337	0.016537016	0.054463175	PREDICTED: facilitated trehalose transporter Tret1-2 homolog
RS008079	GO:0055085	transmembrane transport	BP	-1.109710923	-0.938014135	-0.296800028	2.900944855	10.13930135	0.017418391	0.056816166	PREDICTED: potassium voltage-gated channel protein Shaker
RS009153	GO:0055085	transmembrane transport	BP	0.613293967	0.998084897	0.966219795	3.330543492	10.10124836	0.017724806	0.057621634	PREDICTED: mitochondrial import inner membrane translocase subunit Tim9
RS003950	GO:0055085	transmembrane transport	BP	-0.587671115	-0.868990799	-0.714405964	4.844171479	10.0941278	0.017782729	0.057765203	PREDICTED: cationic amino acid transporter 2
RS005883	GO:0055085	transmembrane transport	BP	0.579677196	0.662723942	0.941061143	6.190916775	10.02257363	0.018375223	0.059324417	PREDICTED: mitochondrial import inner membrane translocase subunit Tim10
RS014544	GO:0055085	transmembrane transport	BP	-0.492312404	-0.805748281	-0.168707308	4.565520148	9.9569744	0.018935432	0.06081939	PREDICTED: monocarboxylate transporter 10-like
RS004575	GO:0055085	transmembrane transport	BP	-0.352734321	0.032623146	0.48704132	4.732722518	9.882231696	0.019594198	0.062460406	PREDICTED: sialin
RS002739	GO:0055085	transmembrane transport	BP	0.211694114	0.16146795	0.629767437	5.898527948	9.844331169	0.019936798	0.063405059	PREDICTED: UDP-xylose and UDP-N-acetylglucosamine transporter
RS009608	GO:0055085	transmembrane transport	BP	-0.650681402	-0.131877234	-0.002301287	6.339415917	9.831393766	0.020055087	0.063636624	PREDICTED: V-type proton ATPase subunit C
RS002046	GO:0055085	transmembrane transport	BP	-1.040945999	-1.141609481	-1.378037143	3.132734296	9.81352734	0.020219576	0.06404561	PREDICTED: sodium- and chloride-dependent GABA transporter 1-like
RS012325	GO:0055085	transmembrane transport	BP	-0.610322555	-0.73163567	0.587302153	2.890049632	9.633930261	0.021948434	0.068590218	PREDICTED: serine/threonine-protein phosphatase 6 regulatory ankyrin repeat subunit A
RS001773	GO:0055085	transmembrane transport	BP	-1.179645502	-1.391479741	-1.203064835	2.188248107	9.497351264	0.023359555	0.072211644	PREDICTED: acetylcholine receptor subunit alpha-like
RS006809	GO:0055085	transmembrane transport	BP	-0.069599294	0.161390813	-0.669189997	5.521140821	9.427681054	0.02411329	0.074118459	PREDICTED: sodium bicarbonate cotransporter 3
RS007719	GO:0055085	transmembrane transport	BP	-0.363554514	0.201823484	0.241238455	6.872179235	9.390306687	0.024527407	0.075102884	PREDICTED: V-type proton ATPase 21 kDa proteolipid subunit
RS005942	GO:0055085	transmembrane transport	BP	0.222003058	0.357500804	-0.707071584	4.173704825	9.375192754	0.024696847	0.075474905	PREDICTED: mitochondrial sodium/hydrogen exchanger 9B2-like
RS003802	GO:0055085	transmembrane transport	BP	0.811082921	0.486372202	-0.08894724	2.933919036	9.26719754	0.025941417	0.078459703	PREDICTED: cGMP-dependent protein kinase, isozyme 1
RS009928	GO:0055085	transmembrane transport	BP	-0.835456878	-2.347674561	-0.849585016	1.053053669	9.23428246	0.02633282	0.079385129	PREDICTED: sodium-dependent noradrenaline transporter-like
RS003234	GO:0055085	transmembrane transport	BP	-0.402942012	0.351806881	0.253791792	7.730694199	9.198914238	0.026759846	0.080432815	PREDICTED: V-type proton ATPase 16 kDa proteolipid subunit
RS001330	GO:0055085	transmembrane transport	BP	-1.668504431	0.166119722	0.427397074	0.85487177	9.12227714	0.027708555	0.082731519	PREDICTED: mitochondrial dicarboxylate carrier
RS007617	GO:0055085	transmembrane transport	BP	-1.32510407	-1.458516586	-0.870134155	3.353068646	9.039095435	0.028775582	0.085310659	PREDICTED: zinc transporter ZIP10
RS009139	GO:0055085	transmembrane transport	BP	0.649351007	1.292845053	0.336142374	2.039377777	9.031613663	0.028873505	0.085540561	PREDICTED: Y+L amino acid transporter 2
RS002268	GO:0055085	transmembrane transport	BP	-0.663213265	-0.543123609	-0.277002394	5.289469542	8.984030819	0.029503961	0.086978671	PREDICTED: inositol 1,4,5-trisphosphate receptor
RS007528	GO:0055085	transmembrane transport	BP	1.281551474	0.79778074	0.871132273	9.026899419	8.962975306	0.029787222	0.087649596	ubiquitin
RS000078	GO:0055085	transmembrane transport	BP	0.468513621	0.680037401	0.281061525	6.456939604	8.950555866	0.029955548	0.088000973	PREDICTED: peroxisomal membrane protein PEX14
RS013576	GO:0055085	transmembrane transport	BP	-0.152692106	0.186422925	0.739608273	8.524782718	8.933399856	0.030189603	0.088564608	PREDICTED: cytochrome c oxidase subunit 7A2, mitochondrial-like
RS008214	GO:0055085	transmembrane transport	BP	-0.591657749	-0.60801793	-0.342781866	5.622477097	8.89592842	0.030707059	0.089873274	PREDICTED: sodium channel protein paralytic
RS007859	GO:0055085	transmembrane transport	BP	-1.037355681	-0.953164057	-1.137934678	2.631254112	8.895408464	0.0307143	0.08987358	PREDICTED: proton-coupled amino acid transporter 4
RS013818	GO:0055085	transmembrane transport	BP	-0.091150147	0.704869775	0.985919631	2.320087932	8.860612664	0.031202686	0.091133262	PREDICTED: kidney mitochondrial carrier protein 1

RS003404	GO:0055085	transmembrane transport	BP	-0.282230962	0.285723529	0.434899732	4.438872301	8.825502366	0.031703186	0.092359456	PREDICTED: DET1- and DDB1-associated protein 1
RS000001	GO:0055085	transmembrane transport	BP	-0.923753843	-0.774146522	-0.309728113	8.964042611	8.801674733	0.032047317	0.093060619	PREDICTED: ryanodine receptor 44F
RS011130	GO:0055085	transmembrane transport	BP	0.62266454	0.316671327	-0.198111492	8.646709884	8.691352167	0.03368886	0.096889131	PREDICTED: 60 kDa heat shock protein, mitochondrial-like
RS014303	GO:0055085	transmembrane transport	BP	1.015519516	-0.140449015	-1.617687421	1.096446077	8.671576225	0.033991683	0.097559539	PREDICTED: sodium channel protein Nach-like
RS001434	GO:0055085	transmembrane transport	BP	-2.10642209	-0.940504046	-0.189980266	0.468874389	8.555806562	0.035818546	0.101461711	PREDICTED: sodium channel protein Nach-like
RS012852	GO:0055085	transmembrane transport	BP	0.095937018	0.366584098	-0.497735083	4.298329529	8.535728142	0.036145003	0.102133761	PREDICTED: glutamate receptor ionotropic, kainate 2-like
RS006017	GO:0055085	transmembrane transport	BP	-0.226333875	0.829010789	0.173010283	3.457042927	8.42538268	0.037991671	0.106302401	PREDICTED: cationic amino acid transporter 2
RS012784	GO:0055085	transmembrane transport	BP	-0.964188065	-1.053670411	-1.487092291	6.693074429	8.119987423	0.043595935	0.118762068	PREDICTED: excitatory amino acid transporter-like
RS002239	GO:0055085	transmembrane transport	BP	-0.518558054	-0.887931947	-1.328774582	2.771875246	7.996103171	0.04609231	0.124359208	PREDICTED: transient receptor potential cation channel protein painless
RS005210	GO:0055085	transmembrane transport	BP	-0.986436868	-1.240920184	-1.092022056	3.917975026	7.935991254	0.047353263	0.126994675	PREDICTED: multidrug resistance protein homolog 49
RS007665	GO:0055085	transmembrane transport	BP	-0.631881952	-0.428365421	0.007983181	8.098194771	7.871009593	0.048754144	0.129949141	PREDICTED: plasma membrane calcium-transporting ATPase 2
RS003841	GO:0055085	transmembrane transport	BP	-0.018830809	0.667905762	-0.171179406	4.919354176	7.849821458	0.04921958	0.130930145	PREDICTED: major facilitator superfamily domain-containing protein 10
RS008434	GO:0055085	transmembrane transport	BP	-0.152418883	-0.335351871	-0.836822092	4.94412872	7.808586514	0.050137806	0.132702402	PREDICTED: potassium voltage-gated channel subfamily H member 2
RS010293	GO:0055085	transmembrane transport	BP	-1.323536549	-0.537537624	-0.40616185	2.969834016	7.797939139	0.050377598	0.133149101	PREDICTED: sodium/hydrogen exchanger 3
RS002931	GO:0055085	transmembrane transport	BP	-0.784498789	-0.645948105	-0.625581434	3.345246421	7.781766391	0.050743963	0.133976828	PREDICTED: acetylcholine receptor subunit beta-like 2
RS012051	GO:0055085	transmembrane transport	BP	-1.206457775	-0.320139517	-0.400442703	3.93903891	7.774642457	0.050906164	0.13434875	PREDICTED: sodium-independent sulfate anion transporter-like
RS012317	GO:0055085	transmembrane transport	BP	-0.648427381	0.001131192	-0.007541087	6.300999393	7.771586789	0.050975892	0.13447641	PREDICTED: calcium-transporting ATPase type 2C member 1
RS003265	GO:0055085	transmembrane transport	BP	0.67065611	0.570992468	0.622458253	5.669387556	7.641310173	0.054036623	0.140868812	PREDICTED: 28S ribosomal protein S9, mitochondrial
RS005847	GO:0055085	transmembrane transport	BP	-0.009134422	0.240630937	0.785370217	8.208365721	7.589016609	0.055314812	0.143517665	PREDICTED: ATP synthase subunit epsilon, mitochondrial-like
RS014918	GO:0055085	transmembrane transport	BP	-0.265116474	0.084232844	0.568475259	7.79189049	7.503165544	0.057477171	0.147999897	PREDICTED: cytochrome c1, heme protein, mitochondrial
RS009082	GO:0055085	transmembrane transport	BP	-1.344763505	-0.953478298	-1.303298962	1.512508029	7.470773642	0.058314177	0.149663762	PREDICTED: glycine receptor subunit alpha-2-like
RS008448	GO:0055085	transmembrane transport	BP	-0.874319748	-1.179805365	-0.159545862	2.543187294	7.449190097	0.058878449	0.150714469	PREDICTED: facilitated trehalose transporter Tret1-2 homolog
RS002799	GO:0055085	transmembrane transport	BP	-0.659046602	-0.211273757	-0.16274335	6.419710777	7.409924332	0.059918615	0.152787009	PREDICTED: V-type proton ATPase subunit H
RS002292	GO:0055085	transmembrane transport	BP	-0.849226044	-0.724590211	-0.752733052	3.999828127	7.339934983	0.06181703	0.156330055	V-type proton ATPase subunit S1
RS004018	GO:0055085	transmembrane transport	BP	-0.854655712	-0.687918747	0.356622885	2.251863917	7.340140368	0.061811374	0.156330055	PREDICTED: potassium/sodium hyperpolarization-activated cyclic nucleotide-gated channel 2
RS010902	GO:0055085	transmembrane transport	BP	-0.413608261	-0.854721106	-0.933757255	3.754238005	7.337994128	0.061870497	0.156389668	PREDICTED: glutamate receptor 1
RS012800	GO:0055085	transmembrane transport	BP	0.051891265	0.181532958	0.60875329	6.827676128	7.290457164	0.063194134	0.158823972	PREDICTED: major facilitator superfamily domain-containing protein 8
RS006810	GO:0055085	transmembrane transport	BP	-0.692918241	-0.709642301	-0.469276201	5.552917252	7.276110601	0.063598968	0.159523084	PREDICTED: Y+L amino acid transporter 2
RS006920	GO:0055085	transmembrane transport	BP	-0.632866598	-0.974633547	-0.967627708	3.248752993	7.241310635	0.064591429	0.161433709	PREDICTED: glutamate receptor ionotropic, NMDA 2B
RS008996	GO:0055085	transmembrane transport	BP	-0.818894069	-0.144607229	0.175883673	2.981815036	7.237819263	0.064691824	0.161620478	PREDICTED: cytochrome c oxidase assembly factor 6 homolog
RS002605	GO:0055085	transmembrane transport	BP	-0.518735488	0.392463203	-0.08725497	4.03235098	7.22499608	0.065061855	0.162287383	PREDICTED: solute carrier family 40 member 1
RS000609	GO:0055085	transmembrane transport	BP	0.612008555	0.732502903	0.190053663	8.31658549	7.219606678	0.065217984	0.162580225	PREDICTED: muscle calcium channel subunit alpha-1
RS006503	GO:0055085	transmembrane transport	BP	-0.789357073	-0.069673576	-0.042357301	4.655019811	7.200877419	0.065763394	0.163551378	PREDICTED: transient receptor potential cation channel trpm
RS015105	GO:0055085	transmembrane transport	BP	-0.678666411	-2.224992259	-0.562865466	0.559101899	7.185153044	0.066224707	0.164438873	PREDICTED: organic cation transporter 1
RS009995	GO:0055085	transmembrane transport	BP	-0.926877817	0.790504308	1.027579647	0.931565014	7.179681543	0.066385961	0.164741831	PREDICTED: ras-related protein Rab-30
RS005264	GO:0055085	transmembrane transport	BP	-1.488549143	-1.308665565	-1.379377385	1.600885014	7.17732657	0.066455483	0.164816926	PREDICTED: sodium-dependent neutral amino acid transporter B(0)AT3
RS006739	GO:0055085	transmembrane transport	BP	-0.215562063	-0.194338635	-0.661480143	5.23328973	7.126492884	0.067973454	0.167558754	PREDICTED: potassium voltage-gated channel protein Shab
RS014661	GO:0055085	transmembrane transport	BP	-0.982950645	-0.377451355	-0.15917073	2.646015483	7.120938203	0.068141345	0.167841207	PREDICTED: sialin
RS000133	GO:0055085	transmembrane transport	BP	-0.045454902	0.182331435	0.994722318	2.687399163	7.116976504	0.068261333	0.168071011	PREDICTED: putative ammonium transporter 1
RS003449	GO:0055085	transmembrane transport	BP	-0.271604031	-0.623910119	-0.38398901	6.1655336	7.019104607	0.07129133	0.173865804	PREDICTED: glutamate receptor ionotropic, kainate 2
RS014295	GO:0055085	transmembrane transport	BP	0.412981653	0.489533068	0.464743455	5.949145628	7.0080526	0.071641551	0.174618482	PREDICTED: zinc transporter 5
RS002500	GO:0055085	transmembrane transport	BP	0.488671628	0.505919803	0.380713572	6.032071153	6.857741521	0.076572944	0.183547707	PREDICTED: gamma-secretase subunit pen-2
RS004291	GO:0055085	transmembrane transport	BP	0.781190319	-1.037280119	-0.352171624	0.672722301	6.843951183	0.07704145	0.184460079	PREDICTED: synaptic vesicle glycoprotein 2B-like
RS003427	GO:0055085	transmembrane transport	BP	-0.513964722	-0.550228674	-0.09809219	5.664872118	6.801016027	0.078517892	0.186983778	PREDICTED: voltage-dependent calcium channel type A subunit alpha-1
RS012028	GO:0055085	transmembrane transport	BP	0.541573501	0.614749029	0.44673468	5.702316197	6.784699514	0.079086109	0.187995021	PREDICTED: mitochondrial import inner membrane translocase subunit TIM44
RS014551	GO:0055085	transmembrane transport	BP	-0.502782747	-0.474145105	0.559209026	3.220569618	6.760015845	0.079953256	0.189622234	PREDICTED: MFS-type transporter SLC18B1-like
RS002296	GO:0055085	transmembrane transport	BP	0.050929101	-1.155041098	-1.453067074	0.872761787	6.743534644	0.080537346	0.190763087	PREDICTED: glycine receptor subunit alpha-4
RS003145	GO:0055085	transmembrane transport	BP	-1.309880541	-0.628756059	-0.991127574	1.553035926	6.713260678	0.081620986	0.19245815	PREDICTED: neuronal acetylcholine receptor subunit alpha-7-like
RS012048	GO:0055085	transmembrane transport	BP	-0.674894361	-1.155560739	-0.486644875	2.649535097	6.691851744	0.082395778	0.193852499	PREDICTED: sodium-independent sulfate anion transporter-like
RS009471	GO:0055085	transmembrane transport	BP	0.454440582	0.068570398	0.92902968	1.975531925	6.680170813	0.082821497	0.194672252	PREDICTED: potassium voltage-gated channel protein Shal
RS002982	GO:0055085	transmembrane transport	BP	0.437159386	0.690352357	0.482073212	5.918755174	6.64375724	0.084162258	0.197455193	PREDICTED: multidrug resistance-associated protein 5-like
RS000035	GO:0055085	transmembrane transport	BP	-0.59529335	-0.304820032	0.347972721	8.866264808	6.597065948	0.085912068	0.200794204	PREDICTED: cytochrome b-c1 complex subunit Rieske, mitochondrial-like
RS000318	GO:0055085	transmembrane transport	BP	-0.409411201	-0.696964023	-0.524046279	5.146315283	6.540394841	0.088082972	0.204696733	PREDICTED: alpha-aminoadipic semialdehyde dehydrogenase
RS001821	GO:0055085	transmembrane transport	BP	-0.416559115	-0.243117375	0.420607418	9.114591662	6.45061119	0.091630759	0.210852874	PREDICTED: cytochrome c oxidase subunit 4 isoform 1, mitochondrial
RS003508	GO:0055085	transmembrane transport	BP	-0.169271487	0.401452192	-0.239447825	4.192461457	6.422099786	0.092785845	0.212888492	PREDICTED: organic cation transporter 1-like
RS010561	GO:0055085	transmembrane transport	BP	0.077383384	1.01828952	0.325351919	10.99254901	6.38788389	0.094190516	0.215171867	PREDICTED: tubulin alpha-1 chain
RS007738	GO:0055085	transmembrane transport	BP	-2.236721279	-1.816342735	0.104485909	0.00738012	6.381695156	0.094446755	0.215638584	PREDICTED: dynein heavy chain 10, axonemal
RS006697	GO:0055085	transmembrane transport	BP	-0.137394972	-0.578930188	0.149350385	8.454117513	6.353830787	0.095608773	0.21750305	PREDICTED: zinc transporter ZIP13 homolog
RS010275	GO:0055085	transmembrane transport	BP	-0.327809837	0.151537852	-0.881426827	3.129420115	6.335110353	0.096397162	0.218625222	PREDICTED: equilibrative nucleoside transporter 4
RS015389	GO:0055085	transmembrane transport	BP	-1.014604708	-1.708407111	-0.480189784	0.854123779	6.332870963	0.096491888	0.218718663	PREDICTED: glutamate receptor ionotropic, delta-2
RS003087	GO:0055085	transmembrane transport	BP	0.028997549	0.644052003	-0.01338752	4.256601246	6.270105267	0.099183515	0.223136333	PREDICTED: solute carrier family 35 member E1 homolog
RS009966	GO:0055085	transmembrane transport	BP	-0.243614202	-0.508062646	-0.78761656	3.789270855	6.228722713	0.100997397	0.225925617	PREDICTED: transient receptor potential channel pyrexia-like
RS013777	GO:0055085	transmembrane transport	BP	0.279495049	0.245639381	0.652098837	5.139040067	6.110578071	0.106352562	0.234983187	PREDICTED: copper homeostasis protein cutC homolog
RS007563	GO:0055085	transmembrane transport	BP	-1.202835431	-1.462716201	-1.791419977	1.4245361	6.089904579	0.107317164	0.236574893	PREDICTED: uncharacterized MFS-type transporter C09D4.1
RS005351	GO:0055085	transmembrane transport	BP	-0.397867942	-0.039889938	0.245645216	7.41412513	6.082183312	0.107679573	0.237000444	PREDICTED: glucose transporter type 1
RS009881	GO:0055085	transmembrane transport	BP	-0.797297236	-0.318713435	0.370791229	1.650545529	6.023916498	0.110452416	0.241877538	PREDICTED: sodium/potassium/calcium exchanger Nckx30C
RS007852	GO:0055085	transmembrane transport	BP	-0.4463895	-0.500436907	-0.353366809	5.359879642	5.946281355	0.114253205	0.24733912	PREDICTED: multidrug resistance-associated protein 7
RS000704	GO:0055085	transmembrane transport	BP	-0.474456632	-0.423285145	-0.406316616	6.32955037	5.894533373	0.116855681	0.251739139	PREDICTED: voltage-dependent calcium channel subunit alpha-2/delta-3
RS008209	GO:0055085	transmembrane transport	BP	-0.797469436	0.00047394	-0.19086895	5.236915618	5.860312794	0.118607657	0.254297723	PREDICTED: protein spinster
RS000168	GO:0055085	transmembrane transport	BP	-0.555511687	-0.887978015	-1.234124807	1.652741901	5.837936606	0.119766759	0.255954957	PREDICTED: potassium voltage-gated channel protein eag
RS001145	GO:0055085	transmembrane transport	BP	-0.53262649	-0.12107554	0.117285332	6.762749099	5.723923744	0.125842321	0.265692906	PREDICTED: solute carrier family 2, facilitated glucose transporter member 1-like
RS003358	GO:0055085	transmembrane transport	BP	-0.353225898	-0.121840444	0.371616686	8.291426996	5.633056759	0.130893435	0.272925814	PREDICTED: ATP synthase-coupling factor 6, mitochondrial
RS007510	GO:0055085	transmembrane transport	BP	-0.474258119	-0.815348826	-0.098467285	4.567105163	5.618317073	0.131730741	0.274127057	PREDICTED: sodium-coupled monocarboxylate transporter 1-like
RS008279	GO:0055085	transmembrane transport	BP	-0.484679453	-0.433954641	0.086422434	3.880506245	5.606020043	0.132433181	0.27531586	PREDICTED: potassium voltage-gated channel protein Shaw-like
RS006497	GO:0055085	transmembrane transport	BP	-0.499541328	-1.800371012	-1.244184902	0.270751657	5.580972087	0.133875009	0.277534463	PREDICTED: facilitated trehalose transporter Tret1-2 homolog
RS009361	GO:0055085	transmembrane transport	BP	-0.469548252	-1.030089652	-0.272125463	2.379260128	5.571170404	0.134443268	0.278300217	PREDICTED: sodium- and chloride-dependent glycine transporter 2-like
RS003947	GO:0055085	transmembrane transport	BP	0.438161137	0.271388201	0.493351708	5.499648986	5.567309687	0.134667724	0.278719033	PREDICTED: cleavage stimulation factor subunit 1
RS002972	GO:0055085	transmembrane transport	BP	-0.311172151	-0.506842424	-0.237806006	6.495567074	5.534163734	0.136609467	0.281857737	PREDICTED: sodium leak channel non-selective protein
RS012532	GO:0055085	transmembrane transport	BP	0.52422059	0.27169055	0.170626759	5.950381512	5.527522768	0.137001687	0.282351902	PREDICTED: adenosine 3'-phospho 5'-phosphosulfate transporter 2
RS007587	GO:0055085	transmembrane transport	BP	0.548015678	0.376775275	0.454348959	4.86046888	5.504926438	0.138344259	0.284411948	PREDICTED: magnesium transporter NIPA2
RS012098	GO:0055085	transmembrane transport	BP	-0.722635213	-0.863193595	-0.599560773	3.5591329	5.486483871	0.13944927	0.285797251	PREDICTED: protein tweety
RS009262	GO:0055085	transmembrane transport	BP	-0.25857572	0.038639212	0.546361074	8.794488927	5.469506202	0.140473903	0.28738277	PREDICTED: cytochrome c oxidase subunit 7C, mitochondrial-like
RS012521	GO:0055085	transmembrane transport	BP	-0.27911124	0.207963234	0.176143396	5.682101261	5.464162095	0.140797903	0.287847294	PREDICTED: sodium/hydrogen exchanger 8
RS011419	GO:0055085	transmembrane transport	BP	0.142670051	0.566918683	0.76286631	3.172442667	5.332525292	0.149005743	0.30010848	PREDICTED: putative inorganic phosphate cotransporter
RS000402	GO:0055085	transmembrane transport	BP	-0.26718069	0.008072239	0.51581876	8.673900207	5.301862053	0.150981611	0.302828859	PREDICTED: cytochrome c oxidase subunit 6A, mitochondrial
RS014310	GO:0055085	transmembrane transport	BP	-0.394503871	-0.23364982	-0.939036808	2.647212913	5.221628248	0.156269422	0.310811292	PREDICTED: neuronal acetylcholine receptor subunit alpha-7
RS004262	GO:0055085	transmembrane transport	BP	-0.304556625	0.103631535	0.676510563	10.41026235	5.204184057	0.157441978	0.312748307	PREDICTED: ATP synthase lipid-binding protein, mitochondrial
RS007787	GO:0055085	transmembrane transport	BP	-0.305912555	0.11337882	0.601620189	9.43857381	5.186627171	0.15863048	0.314655166	PREDICTED: ATP synthase subunit b, mitochondrial
RS008202	GO:0055085	transmembrane transport	BP	0.24252685	0.342844048	-0.965951499	1.493557986	5.185516154	0.158705973	0.314677881	PREDICTED: voltage-dependent calcium channel gamma-5 subunit
RS000666	GO:0055085	transmembrane transport	BP	-0.080539122	0.013403368	0.652885093	9.136399161	5.147116783	0.161336103	0.31832475	PREDICTED: ATP synthase subunit g, mitochondrial
RS006076	GO:0055085	transmembrane transport	BP	-0.399500112	-0.234112198	-0.791572834	3.218719557	5.135126595	0.162165734	0.319411206	PREDICTED: cationic amino acid transporter 4
RS002494	GO:0055085	transmembrane transport	BP	0.23516431	-0.326148106	0.31597262	3.648514777	5.092877143	0.165121192	0.323864864	PREDICTED: proton-coupled folate transporter
RS008395	GO:0055085	transmembrane transport	BP	0.758334632	-0.043191485	0.423582919	3.204444173	5.092897421	0.165119762	0.323864864	PREDICTED: transient receptor potential cation channel protein painless
RS015132	GO:0055085	transmembrane transport	BP	-0.311288053	0.021217655	0.441252337	8.852842665	5.035692944	0.169202112	0.329814486	PREDICTED: ATP synthase subunit O, mitochondrial
RS001012	GO:0055085	transmembrane transport	BP	-0.050637771	0.65837124	0.220417241	5.028311487	5.034189195	0.169310694	0.329872366	PREDICTED: sodium/calcium exchanger 2
RS001954	GO:0055085	transmembrane transport	BP	-0.511023769	-1.121622332	-0.950494549	1.29802559	4.996217481	0.172074329	0.333041969	PREDICTED: major facilitator superfamily domain-containing protein 6-like
RS003210	GO:0055085	transmembrane transport	BP	0.457872677	0.832393815	0.705350714	3.97283813	4.972582281	0.173815849	0.33553599	PREDICTED: solute carrier family 12 member 8
RS007406	GO:0055085	transmembrane transport	BP	1.53218285	1.416234104	0.234676903	0.509107572	4.915967777	0.17805483	0.341676447	PREDICTED: sodium-independent sulfate anion transporter-like
RS007390	GO:0055085	transmembrane transport	BP	-0.350929593	-1.171722101	-0.259139814	1.654126139	4.900550276	0.179225874	0.343191525	PREDICTED: open rectifier potassium channel protein 1
RS012974	GO:0055085	transmembrane transport	BP	0.386362631	0.350475987	0.590578301	4.958398051	4.857275707	0.182551447	0.347919632	PREDICTED: mitochondrial import inner membrane translocase subunit Tim16
RS002766	GO:0055085	transmembrane transport	BP	-0.496155234	-0.78863534	-0.632950682	4.168210328	4.786567039	0.188109698	0.354852096	PREDICTED: solute carrier family 25 member 36-A
RS015035	GO:0055085	transmembrane transport	BP	-0.486032243	-0.438869682	-0.112800307	5.262898327	4.77200592	0.189273765	0.356097071	PREDICTED: solute carrier family 2, facilitated glucose transporter member 10-like
RS011272	GO:0055085	transmembrane transport	BP	-0.449550793	-0.842262029	0.317704025	1.68576224	4.742178986	0.191679247	0.358960362	PREDICTED: transient receptor potential-gamma protein
RS013062	GO:0055085	transmembrane transport	BP	-0.00162788	-0.815422885	-0.945361754	1.375980209	4.702121991	0.194954616	0.363203911	PREDICTED: transient receptor potential channel pyrexia
RS005980	GO:0055085	transmembrane transport	BP	-0.519964769	-0.115820264	0.014177289	6.649793844	4.674091701	0.197277507	0.365853766	PREDICTED: V-type proton ATPase subunit d
RS003391	GO:0055085	transmembrane transport	BP	-0.542480622	-0.539981143	-0.585509501	9.138318653	4.6421131	0.199959035	0.36995506	ubiquitin
RS000148	GO:0055085	transmembrane transport	BP	-0.122107004	0.195755414	0.48968258	6.587092416	4.638860432	0.200233675	0.370299985	PREDICTED: mitochondrial dicarboxylate carrier
RS013193	GO:0055085	transmembrane transport	BP	-0.571318105	-0.638002435	-0.492911161	3.125045391	4.612135042	0.202503545	0.372595481	PREDICTED: gamma-aminobutyric acid receptor subunit beta-like
RS002889	GO:0055085	transmembrane transport	BP	-0.128703142	0.3529612	0.286577104	4.360050122	4.605415706	0.203077987	0.373149135	PREDICTED: bifunctional arginine demethylase and lysyl-hydroxylase PSR
RS007095	GO:0055085	transmembrane transport	BP	0.192687609	0.009365646	-0.454674091	8.985348469	4.560229052	0.206980499	0.378277813	PREDICTED: UDP-glucose:glycoprotein glucosyltransferase
RS013960	GO:0055085	transmembrane transport	BP	-0.472338012	-0.452157922	0.021607712	3.915657005	4.540751566	0.208684017	0.380067463	PREDICTED: multidrug resistance-associated protein 5-like

RS009973	GO:0055085	transmembrane transport	BP	0.1676973	0.302067179	0.694181931	3.324934717	4.531641986	0.2094852	0.381030706 PREDICTED: sugar transporter SWEET1
RS002890	GO:0055085	transmembrane transport	BP	-0.213107922	-0.035236467	0.494651933	9.00551627	4.518839864	0.210615958	0.382259313 PREDICTED: cytochrome c oxidase subunit 5A, mitochondrial
RS000217	GO:0055085	transmembrane transport	BP	-0.750015472	-0.258912471	-0.859143521	4.771460381	4.479971735	0.214083735	0.38649234 PREDICTED: innexin inx1
RS011433	GO:0055085	transmembrane transport	BP	-0.117163554	0.472067962	0.209582221	5.304247486	4.450589701	0.216740144	0.389556925 PREDICTED: graves disease carrier protein-like
RS012324	GO:0055085	transmembrane transport	BP	-0.639112315	-1.234689077	-0.08776879	1.305640976	4.37485531	0.22372824	0.397633848 PREDICTED: serine/threonine-protein phosphatase 6 regulatory ankyrin repeat subunit A
RS009362	GO:0055085	transmembrane transport	BP	-0.151734251	-1.379851033	-0.853593219	0.577299208	4.353138426	0.225770076	0.399764452 PREDICTED: sodium- and chloride-dependent glycine transporter 2-like
RS001539	GO:0055085	transmembrane transport	BP	-0.380736825	-1.0292407	-0.36481089	2.349632725	4.316399244	0.229263428	0.403767811 PREDICTED: transient receptor potential cation channel protein painless
RS012560	GO:0055085	transmembrane transport	BP	-0.041301573	-0.515464379	-0.426603809	4.255404315	4.307057209	0.230159613	0.404677505 PREDICTED: ATP-binding cassette sub-family B member 6, mitochondrial
RS005280	GO:0055085	transmembrane transport	BP	0.572920685	0.052379455	0.938447567	3.032392488	4.273149565	0.233439508	0.40894939 PREDICTED: acid-sensing ion channel 4
RS001911	GO:0055085	transmembrane transport	BP	-0.354858321	-0.511067736	-0.530111822	4.46637499	4.265443861	0.234190845	0.409944281 PREDICTED: palmitoyltransferase ZDHHC17
RS007791	GO:0055085	transmembrane transport	BP	-0.379787464	0.049869261	0.105041138	7.48004628	4.225689753	0.238102407	0.41486742 PREDICTED: V-type proton ATPase subunit E
RS015079	GO:0055085	transmembrane transport	BP	-1.376295246	0.661290456	0.618943801	0.209343525	4.174647883	0.243212401	0.420568148 PREDICTED: facilitated trehalose transporter Tret1-like
RS012398	GO:0055085	transmembrane transport	BP	-0.181909214	0.045983675	0.559515665	9.326609998	4.173573583	0.243321024	0.420643099 PREDICTED: cytochrome c oxidase subunit 5B, mitochondrial-like
RS007439	GO:0055085	transmembrane transport	BP	-0.177322528	-0.488733711	0.259153647	3.425894364	4.099962898	0.250870294	0.42974095 PREDICTED: major facilitator superfamily domain-containing protein 9
RS010122	GO:0055085	transmembrane transport	BP	-0.765374966	-0.665862456	0.101094282	12.40753779	4.068317122	0.254180983	0.433408517 PREDICTED: ADP,ATP carrier protein 2
RS001774	GO:0055085	transmembrane transport	BP	-0.556967124	-0.686693785	0.064399051	2.246327006	4.014186998	0.25993626	0.440531132 PREDICTED: acetylcholine receptor subunit alpha-like
RS012212	GO:0055085	transmembrane transport	BP	1.298461473	1.241884362	0.487723216	0.415207106	3.920422645	0.270186252	0.451829193 PREDICTED: sodium channel protein 60E
RS000049	GO:0055085	transmembrane transport	BP	-0.167016261	-0.078808593	0.315739518	4.19490186	3.918242209	0.270428905	0.452114912 PREDICTED: protein I'm not dead yet-like
RS004419	GO:0055085	transmembrane transport	BP	-0.39398529	0.162481593	-1.185227216	0.809494392	3.887905498	0.273825486	0.455851742 PREDICTED: transient receptor potential-gamma protein
RS003691	GO:0055085	transmembrane transport	BP	-0.396884624	-0.571293958	0.253637659	1.856747806	3.871872414	0.275636118	0.457912129 PREDICTED: transient receptor potential cation channel subfamily A member 1
RS010418	GO:0055085	transmembrane transport	BP	-0.19455437	0.000394051	0.481391778	9.395600975	3.855673322	0.277476464	0.459791431 PREDICTED: cytochrome c oxidase subunit 6B1-like
RS011747	GO:0055085	transmembrane transport	BP	-0.380454491	0.007681606	0.358897081	9.615965078	3.849118736	0.278224261	0.460864966 PREDICTED: ATP synthase subunit gamma, mitochondrial
RS000693	GO:0055085	transmembrane transport	BP	-0.697277451	-0.63859341	-1.105155719	1.668516432	3.761841056	0.288355965	0.47128419 PREDICTED: solute carrier family 12 member 4
RS004593	GO:0055085	transmembrane transport	BP	0.262368668	-0.314833663	-0.606891149	2.686998086	3.735102132	0.29152562	0.474414272 PREDICTED: SET domain-containing protein 4
RS014392	GO:0055085	transmembrane transport	BP	-0.97591621	-0.707349255	0.232142575	0.530622375	3.721584252	0.293139895	0.476361786 PREDICTED: probable multidrug resistance-associated protein lethal(2)03659
RS002930	GO:0055085	transmembrane transport	BP	-0.451673532	-0.71437108	-0.718954078	3.306419228	3.701408177	0.295564164	0.478695356 PREDICTED: acetylcholine receptor subunit alpha-L1
RS005072	GO:0055085	transmembrane transport	BP	-0.654987192	-0.4219481	-0.18940484	3.26003723	3.685352455	0.297506145	0.480656437 PREDICTED: voltage-dependent T-type calcium channel subunit alpha-1G
RS006990	GO:0055085	transmembrane transport	BP	-0.310134595	-0.4355977	-0.507024072	4.194119455	3.681544826	0.297968358	0.480765048 PREDICTED: BTB/POZ domain-containing protein KCTD3
RS003147	GO:0055085	transmembrane transport	BP	-0.505550451	-0.027606287	-0.18120612	3.786984288	3.627252198	0.30462908	0.487532937 PREDICTED: acetylcholine receptor subunit beta-like 1
RS012817	GO:0055085	transmembrane transport	BP	-0.287960684	-0.628111107	-0.66343646	3.635326683	3.625082508	0.304897998	0.48766642 PREDICTED: facilitated trehalose transporter Tret1-like
RS015381	GO:0055085	transmembrane transport	BP	-0.14122205	0.494615068	-0.457720223	1.269481569	3.572397731	0.311493056	0.493953414 PREDICTED: potassium voltage-gated channel subfamily KQT member 1
RS013677	GO:0055085	transmembrane transport	BP	0.034211804	0.280550659	0.595537596	9.222031987	3.437461301	0.328964218	0.510658864 PREDICTED: cytochrome c oxidase subunit 6C
RS002729	GO:0055085	transmembrane transport	BP	0.044674654	0.345192364	0.263948476	5.488708832	3.423687042	0.330795361	0.512553738 PREDICTED: S-adenosylmethionine mitochondrial carrier protein-like
RS009119	GO:0055085	transmembrane transport	BP	-0.633398641	0.027582619	-0.254763808	2.374824839	3.400802845	0.333857375	0.51571198 PREDICTED: lateral signaling target protein 2 homolog
RS006561	GO:0055085	transmembrane transport	BP	0.353995304	0.237572917	0.419480704	5.635873753	3.395244005	0.334604917	0.516387054 PREDICTED: EH domain-containing protein 3
RS015453	GO:0055085	transmembrane transport	BP	-0.09263959	0.415348464	0.277714417	5.686672294	3.355959113	0.33992981	0.521573591 PREDICTED: sodium-dependent phosphate transporter 2
RS008482	GO:0055085	transmembrane transport	BP	0.456620351	0.231742272	0.664992756	4.899508409	3.329273867	0.343589006	0.524567819 PREDICTED: organic cation transporter protein
RS000775	GO:0055085	transmembrane transport	BP	0.833279028	0.566215982	0.85702564	2.313556006	3.298690339	0.347824967	0.528153802 PREDICTED: uncharacterized MFS-type transporter C09D4.1
RS008880	GO:0055085	transmembrane transport	BP	0.14769946	0.553054828	0.336408558	3.369176491	3.28851771	0.349243966	0.529733651 PREDICTED: pickpocket protein 19-like
RS001341	GO:0055085	transmembrane transport	BP	-0.6048269	-1.642468152	-0.431664777	0.247813466	3.229275082	0.357608157	0.538786592 PREDICTED: sodium/potassium/calcium exchanger 4-like
RS015449	GO:0055085	transmembrane transport	BP	0.241533289	-0.182689412	0.023986423	5.25590239	3.209399612	0.360452886	0.541776304 PREDICTED: peroxisome biogenesis factor 10
RS008726	GO:0055085	transmembrane transport	BP	-0.239198332	0.278689624	0.146315272	4.384525019	3.151643572	0.368830479	0.550035649 PREDICTED: equilibrative nucleoside transporter 3
RS008888	GO:0055085	transmembrane transport	BP	-0.294502555	-0.567523121	-0.206094758	5.180319559	3.112412951	0.374615949	0.554657096 PREDICTED: facilitated trehalose transporter Tret1-2 homolog
RS003956	GO:0055085	transmembrane transport	BP	-0.323676554	0.116745023	-0.07691896	6.422899031	3.056887659	0.382937112	0.562262205 PREDICTED: solute carrier family 2, facilitated glucose transporter member 1-like
RS003609	GO:0055085	transmembrane transport	BP	-0.051627285	0.355758402	0.153478437	5.36998255	3.050524042	0.383900772	0.56337058 PREDICTED: zinc transporter ZIP1
RS002264	GO:0055085	transmembrane transport	BP	-0.256910292	-0.386576341	-0.233202222	5.959043483	3.02437981	0.387881542	0.567097764 PREDICTED: sodium-dependent glucose transporter 1
RS000591	GO:0055085	transmembrane transport	BP	-0.210745642	-0.711938748	-0.062273638	2.249015287	2.914724059	0.404960781	0.581735608 PREDICTED: solute carrier family 41 member 1-like
RS003109	GO:0055085	transmembrane transport	BP	-0.563508854	-0.456515106	-0.489126671	3.799327004	2.910695341	0.405600131	0.582321939 PREDICTED: organic cation transporter protein-like
RS005802	GO:0055085	transmembrane transport	BP	-0.201437577	-0.069914946	-0.593279778	3.766942431	2.895793271	0.407972418	0.584129676 PREDICTED: two pore potassium channel protein sup-9
RS001904	GO:0055085	transmembrane transport	BP	-0.27687136	-0.081401122	0.3505686	9.687890931	2.842286187	0.416586082	0.592334615 PREDICTED: ATP synthase subunit d, mitochondrial
RS011686	GO:0055085	transmembrane transport	BP	-0.652816077	-0.692313812	-0.834928913	1.23665669	2.748017073	0.432128872	0.606039089 PREDICTED: TWiK family of potassium channels protein 18-like
RS003847	GO:0055085	transmembrane transport	BP	-0.07803106	-0.071687271	0.272229928	4.838857373	2.707139566	0.439015363	0.612694978 PREDICTED: DNL-type zinc finger protein-like
RS012396	GO:0055085	transmembrane transport	BP	0.327124195	0.012418108	0.069947446	5.465353023	2.691227933	0.441720074	0.615449672 PREDICTED: peroxisome biogenesis factor 13
RS005905	GO:0055085	transmembrane transport	BP	-0.707729301	-1.010882877	-0.25518329	0.794458623	2.663576794	0.446452565	0.62012801 AAEL002484-PA
RS012107	GO:0055085	transmembrane transport	BP	-0.368799719	0.188292375	-0.013404979	5.272470317	2.653348424	0.448213543	0.621757305 PREDICTED: G protein-activated inward rectifier potassium channel 3-like
RS014879	GO:0055085	transmembrane transport	BP	-0.246283404	-0.362594032	0.41505142	1.337328694	2.627851228	0.452627775	0.625660651 PREDICTED: glycine receptor subunit alphaZ1-like
RS007790	GO:0055085	transmembrane transport	BP	0.13034852	0.225712601	0.386180211	4.625198528	2.606288996	0.456388071	0.628856824 PREDICTED: mitochondrial glutamate carrier 1-like
RS010426	GO:0055085	transmembrane transport	BP	-0.0320805	-0.19528696	-0.535965982	2.761673686	2.581268642	0.460782863	0.632865926 PREDICTED: glutamate receptor ionotropic, NMDA 2B
RS013293	GO:0055085	transmembrane transport	BP	0.198558902	1.083035633	0.686996214	0.580149697	2.56014753	0.464519072	0.636353747 PREDICTED: synaptic vesicle glycoprotein 2B-like
RS007973	GO:0055085	transmembrane transport	BP	0.255068646	0.370011576	0.284397768	6.620720548	2.53491853	0.469013562	0.640437689 PREDICTED: zinc transporter 9
RS009279	GO:0055085	transmembrane transport	BP	0.294401775	0.208728083	0.372173078	5.384293591	2.517191015	0.472192292	0.643035221 PREDICTED: probable low affinity copper uptake protein 2
RS006138	GO:0055085	transmembrane transport	BP	0.077942232	0.584419518	-0.370544993	1.33370078	2.490635188	0.476985892	0.646552279 PREDICTED: potassium channel subfamily K member 18-like
RS000905	GO:0055085	transmembrane transport	BP	0.022025399	0.042858662	0.42533997	8.298571127	2.483368496	0.478304268	0.647507681 PREDICTED: cytochrome b-c1 complex subunit 10-like
RS003585	GO:0055085	transmembrane transport	BP	-0.526969653	-0.325999045	-0.519425311	4.846026814	2.460616462	0.482450634	0.65141528 PREDICTED: monocarboxylate transporter 3
RS007514	GO:0055085	transmembrane transport	BP	-0.393644456	0.206862252	-0.369998274	2.851975701	2.440356048	0.48616657	0.653933758 PREDICTED: solute carrier family 26 member 10-like
RS004471	GO:0055085	transmembrane transport	BP	0.302505199	0.1016203	-0.272900349	3.077100879	2.396738775	0.494241988	0.660492741 PREDICTED: Y+L amino acid transporter 2
RS003266	GO:0055085	transmembrane transport	BP	-0.36926559	-0.039795183	-0.401685153	5.103564049	2.37990733	0.497385826	0.663356676 PREDICTED: transient receptor potential channel pyrexia
RS006811	GO:0055085	transmembrane transport	BP	-0.091072902	-0.032205255	0.237958262	6.439935586	2.353693428	0.502312778	0.667124828 PREDICTED: angio-associated migratory cell protein
RS012376	GO:0055085	transmembrane transport	BP	-0.443120005	0.021687504	0.233976036	10.92588788	2.350839235	0.50285148	0.667312245 PREDICTED: phosphate carrier protein, mitochondrial
RS014502	GO:0055085	transmembrane transport	BP	-0.219647182	0.224994314	0.605975653	1.297553296	2.311846878	0.51025516	0.673954219 PREDICTED: small conductance calcium-activated potassium channel protein
RS000079	GO:0055085	transmembrane transport	BP	-0.412658669	-0.332670739	-0.441767601	3.811839291	2.286255317	0.515159136	0.677730509 PREDICTED: CTD nuclear envelope phosphatase 1 homolog
RS005763	GO:0055085	transmembrane transport	BP	-0.049864591	0.331988566	0.418799139	2.785560138	2.265395088	0.519182685	0.681106738 PREDICTED: TWiK family of potassium channels protein 7
RS014798	GO:0055085	transmembrane transport	BP	-0.301610744	-0.249820159	-0.529578201	3.17969966	2.231637207	0.525743741	0.685852151 PREDICTED: proton-coupled folate transporter-like
RS002929	GO:0055085	transmembrane transport	BP	-0.39492811	-0.310803863	-0.368352585	3.630774923	2.110337646	0.54982422	0.704208911 PREDICTED: acetylcholine receptor subunit alpha-like 1
RS006817	GO:0055085	transmembrane transport	BP	0.215767809	0.330522088	0.120729712	5.269453245	2.097871442	0.552343505	0.706429786 PREDICTED: mitochondrial import inner membrane translocase subunit Tim21
RS009552	GO:0055085	transmembrane transport	BP	-0.317174334	0.102956036	0.402195532	11.51695741	2.083221242	0.555314671	0.708862049 PREDICTED: ATP synthase subunit beta, mitochondrial
RS002902	GO:0055085	transmembrane transport	BP	-0.415647108	-0.152738152	0.265175951	11.47381982	2.020686865	0.56812419	0.719524944 PREDICTED: ATP synthase subunit alpha, mitochondrial
RS010798	GO:0055085	transmembrane transport	BP	0.021270494	0.09209859	-1.197682465	0.555972403	2.01223563	0.569871042	0.720350873 PREDICTED: zinc transporter ZIP11
RS005231	GO:0055085	transmembrane transport	BP	-0.391467446	-0.412384168	-0.179305009	4.071451755	1.941339648	0.584670698	0.731588315 PREDICTED: organic cation transporter protein
RS014789	GO:0055085	transmembrane transport	BP	-0.384309417	-0.340794963	-0.701560358	2.91880799	1.866727447	0.600523037	0.745342183 PREDICTED: zinc transporter ZIP9
RS001343	GO:0055085	transmembrane transport	BP	-0.182055731	-0.078317754	-1.122984871	0.25409944	1.840228913	0.606220045	0.749804104 PREDICTED: sodium/potassium/calcium exchanger 4
RS005281	GO:0055085	transmembrane transport	BP	0.147692306	-0.427946659	0.130849224	2.285946009	1.819237088	0.610757704	0.752790867 PREDICTED: acid-sensing ion channel 4
RS008886	GO:0055085	transmembrane transport	BP	-0.289536803	-0.51024615	-0.479741606	2.079554922	1.757477457	0.624231737	0.763037416 PREDICTED: facilitated trehalose transporter Tret1
RS014536	GO:0055085	transmembrane transport	BP	-0.137268948	-0.07750242	-0.358531119	4.210868086	1.71200184	0.634268817	0.771080465 PREDICTED: cyclic nucleotide-gated cation channel beta-3
RS004660	GO:0055085	transmembrane transport	BP	-0.375866955	-0.237419671	-0.141671824	3.773053923	1.687031734	0.639820776	0.774277204 PREDICTED: adenosine 3'-phospho 5'-phosphosulfate transporter 1
RS006928	GO:0055085	transmembrane transport	BP	-0.363135133	-0.220144308	0.11249691	2.701409449	1.667496458	0.644184087	0.776870094 PREDICTED: hippocampus abundant transcript 1 protein
RS015165	GO:0055085	transmembrane transport	BP	0.203835116	0.086955701	-0.092770826	6.68983845	1.645463293	0.649125814	0.780587272 PREDICTED: sodium channel protein 60E
RS003602	GO:0055085	transmembrane transport	BP	-0.963739329	-0.484991816	-0.758608967	0.476696176	1.634124817	0.651677236	0.782683875 PREDICTED: solute carrier family 28 member 3
RS010427	GO:0055085	transmembrane transport	BP	-0.256640259	-0.293251267	-0.831731644	1.070368751	1.607645315	0.657657541	0.78679007 PREDICTED: glutamate receptor ionotropic, NMDA 2B
RS000107	GO:0055085	transmembrane transport	BP	0.215541555	0.120252028	-0.217617098	3.595365183	1.517996661	0.678122983	0.800549125 PREDICTED: multidrug resistance protein 1A
RS004131	GO:0055085	transmembrane transport	BP	0.244077702	0.428184836	0.166004547	3.05086288	1.47403705	0.688275398	0.807916894 PREDICTED: synaptic vesicle 2-related protein
RS009066	GO:0055085	transmembrane transport	BP	-0.284333504	-0.421360118	-0.423891407	2.706723454	1.434128224	0.697554745	0.814482149 PREDICTED: solute carrier family 35 member E3-like
RS013170	GO:0055085	transmembrane transport	BP	-0.32607493	-0.406051581	-0.605910078	1.623065456	1.429779426	0.698569354	0.815288893 PREDICTED: probable sodium/potassium/calcium exchanger CG1090
RS015498	GO:0055085	transmembrane transport	BP	0.184679301	0.472371696	0.26432428	2.413896137	1.414695214	0.702093736	0.818113296 PREDICTED: calcium uniporter protein, mitochondrial
RS010347	GO:0055085	transmembrane transport	BP	-0.186689215	0.108500444	0.151129076	3.585218724	1.414002621	0.702255747	0.81816069 Cyclic nucleotide-gated cation channel alpha-3
RS014968	GO:0055085	transmembrane transport	BP	0.070753676	0.338611082	0.008973976	4.167384444	1.401868016	0.705096916	0.82085326 PREDICTED: solute carrier family 41 member 1-like
RS011446	GO:0055085	transmembrane transport	BP	-0.226602437	0.017489836	-0.173735734	4.50551615	1.392641248	0.707260567	0.822459795 PREDICTED: stress-activated protein kinase JNK
RS004705	GO:0055085	transmembrane transport	BP	-0.430701847	-0.465359916	-0.484712083	3.064343052	1.370281204	0.712515526	0.826890945 PREDICTED: sodium/hydrogen exchanger 7
RS005130	GO:0055085	transmembrane transport	BP	-0.120932052	-0.228887603	-0.254860555	4.886019147	1.367159764	0.713250393	0.827286374 PREDICTED: major facilitator superfamily domain-containing protein 6
RS001366	GO:0055085	transmembrane transport	BP	-0.202685308	-0.234557446	-0.316543451	6.418554265	1.353303228	0.71651627	0.829698961 PREDICTED: glutamate receptor ionotropic, kainate 2
RS010673	GO:0055085	transmembrane transport	BP	0.194686816	0.144745443	0.237663354	5.4673327	1.348021968	0.717762586	0.830648431 PREDICTED: zinc transporter foi-like
RS014588	GO:0055085	transmembrane transport	BP	-0.270138594	0.249327239	-0.142196498	2.721477241	1.322545255	0.723786572	0.834587812 PREDICTED: G protein-coupled receptor kinase 2
RS009390	GO:0055085	transmembrane transport	BP	-0.711993959	-0.803385471	-0.355292865	0.366452897	1.289104566	0.731721917	0.839984739 PREDICTED: gamma-aminobutyric acid receptor alpha-like
RS002882	GO:0055085	transmembrane transport	BP	0.362043543	0.12287268	0.14951259	3.567153224	1.277605364	0.734457649	0.842342193 PREDICTED: putative sodium-coupled neutral amino acid transporter 7
RS015540	GO:0055085	transmembrane transport	BP	-0.463597962	-0.808832157	-0.297537157	1.427647784	1.257020479	0.73936339	0.845811839 PREDICTED: zinc transporter ZIP11
RS013953	GO:0055085	transmembrane transport	BP	-0.420067551	-0.07959435	-0.044577109	2.09881014	1.242877248	0.742739999	0.848223421 PREDICTED: two pore potassium channel protein sup-9
RS000297	GO:0055085	transmembrane transport	BP	-0.078397003	0.556951886	0.159163891	1.385608421	1.213828293	0.749689662	0.85220612 PREDICTED: probable glutamate receptor
RS005062	GO:0055085	transmembrane transport	BP	0.042915661	-0.171071911	-0.235081974	3.733684216	1.211821777	0.75017038	0.852564992 PREDICTED: mitochondrial uncoupling protein 4
RS005898	GO:0055085	transmembrane transport	BP	-0.109533007	-0.24419355	-0.181501684	5.469138988	1.210081204	0.750587452	0.852649778 PREDICTED: ATP-binding cassette sub-family B member 10, mitochondrial
RS012877	GO:0055085	transmembrane transport	BP	0.012158985	-0.173894892	-0.21613122	4.155231751	1.200465777	0.752892601	0.854381818 PREDICTED: solute carrier family 25 member 38-like
RS008405	GO:0055085	transmembrane transport	BP	-0.507380646	-0.391727947	-0.160990784	2.501471485	1.183658987	0.75692617	0.857267824 PREDICTED: solute carrier family 12 member 9

RS002702	GO:0055085	transmembrane transport	BP	0.241523641	0.372283207	0.450735855	2.97728399	1.149201676	0.765211808	0.862694666	PREDICTED: congested-like trachea protein
RS001035	GO:0055085	transmembrane transport	BP	0.124094061	0.276516429	0.187901485	8.14568359	1.113792392	0.773745632	0.868231611	PREDICTED: tubulin alpha-1 chain
RS010672	GO:0055085	transmembrane transport	BP	-0.302029346	0.22206596	0.191043022	1.595359022	1.095814464	0.778084584	0.871039755	Zinc transporter 2
RS014288	GO:0055085	transmembrane transport	BP	-0.267562999	-0.21719274	-0.273627167	4.00532462	1.061069676	0.786479339	0.876852054	PREDICTED: ATP-binding cassette sub-family B member 8, mitochondrial
RS004706	GO:0055085	transmembrane transport	BP	-0.281248638	-0.101038833	-0.415774939	3.347688564	1.047260677	0.789818336	0.878882504	PREDICTED: sodium/hydrogen exchanger 7
RS012225	GO:0055085	transmembrane transport	BP	0.123374918	0.239169678	0.240173626	4.155168052	1.019975888	0.796418536	0.88302073	PREDICTED: mitochondrial 2-oxodicarboxylate carrier
RS001501	GO:0055085	transmembrane transport	BP	-0.162735947	-0.319720165	-0.011856727	3.268041292	0.998514331	0.801611445	0.885725087	PREDICTED: MFS-type transporter SLC18B1-like
RS007150	GO:0055085	transmembrane transport	BP	0.246217518	0.212666126	0.29750827	5.406495568	0.963851814	0.80999779	0.890926827	PREDICTED: probable multidrug resistance-associated protein lethal(2)03659
RS007717	GO:0055085	transmembrane transport	BP	0.066529061	0.463299961	0.014408782	1.808915589	0.956769727	0.811710752	0.892106596	PREDICTED: V-type proton ATPase 21 kDa proteolipid subunit
RS000442	GO:0055085	transmembrane transport	BP	-0.030981455	-0.235190752	-0.143405567	4.312058358	0.936944795	0.816504251	0.894645427	PREDICTED: proton-coupled folate transporter-like
RS008929	GO:0055085	transmembrane transport	BP	-0.139724103	-0.236598473	-0.091823273	4.57881475	0.895160984	0.826595415	0.901623303	PREDICTED: cytochrome c oxidase assembly protein COX15 homolog
RS011500	GO:0055085	transmembrane transport	BP	-0.54663125	-0.559810746	-0.038971768	0.470844775	0.876166842	0.831175153	0.904842	PREDICTED: zinc transporter ZIP8
RS014606	GO:0055085	transmembrane transport	BP	-0.143915834	0.212649167	-0.24267723	3.367536213	0.853642153	0.836598099	0.908207608	PREDICTED: major facilitator superfamily domain-containing protein 1-like
RS001970	GO:0055085	transmembrane transport	BP	0.084033429	-0.012169719	-0.157558613	5.885614174	0.833122903	0.84152916	0.911638874	PREDICTED: glutamate receptor ionotropic, kainate 2
RS005586	GO:0055085	transmembrane transport	BP	0.165076617	-0.125717332	0.501179805	0.440219349	0.817686596	0.845232123	0.914113642	big brain
RS006019	GO:0055085	transmembrane transport	BP	0.016800687	-0.569398535	-0.206623396	0.539126613	0.775635116	0.855285471	0.920559373	PREDICTED: cationic amino acid transporter 2
RS004416	GO:0055085	transmembrane transport	BP	-0.214358133	0.042991977	0.181506365	2.545521498	0.775084362	0.855416767	0.920596693	PREDICTED: monocarboxylate transporter 12-like
RS000298	GO:0055085	transmembrane transport	BP	-0.477815194	-0.328207506	-0.189725936	1.140735658	0.76073894	0.858832832	0.922721437	PREDICTED: glutamate receptor ionotropic, NMDA 3A-like
RS010574	GO:0055085	transmembrane transport	BP	-0.354727613	-0.373915239	-0.481676921	0.828534929	0.681580005	0.877527727	0.933866301	PREDICTED: G protein-activated inward rectifier potassium channel 3-like
RS004601	GO:0055085	transmembrane transport	BP	-0.128635596	-0.15423878	-0.239776755	5.581079946	0.672581672	0.879633272	0.935315761	PREDICTED: ankyrin-3
RS001040	GO:0055085	transmembrane transport	BP	-0.098603565	-0.201658646	0.076364834	3.089669028	0.652037571	0.884422889	0.938003319	PREDICTED: sideroflexin-2
RS003957	GO:0055085	transmembrane transport	BP	0.345462579	0.413965389	0.154609396	1.754535313	0.612321805	0.893605875	0.942823504	PREDICTED: solute carrier family 2, facilitated glucose transporter member 1-like
RS003729	GO:0055085	transmembrane transport	BP	-0.067068908	-0.31722074	-0.453287028	1.039779299	0.608180172	0.894557183	0.943318527	PREDICTED: neuronal acetylcholine receptor subunit alpha-7-like
RS003394	GO:0055085	transmembrane transport	BP	0.180289073	0.135383955	0.089380518	6.008505153	0.597460048	0.89701359	0.944722291	PREDICTED: vesicle transport protein USE1
RS001538	GO:0055085	transmembrane transport	BP	0.320201018	0.172065415	-0.020378547	1.614496317	0.588836165	0.898983255	0.94603515	PREDICTED: allatostatin-A receptor-like
RS014940	GO:0055085	transmembrane transport	BP	0.00473671	-0.350880149	-0.354295712	0.736422598	0.582482205	0.900430699	0.946555742	PREDICTED: potassium voltage-gated channel protein Shal
RS005560	GO:0055085	transmembrane transport	BP	0.073648066	0.070694895	-0.087436803	5.960124723	0.555903739	0.906448712	0.950525819	PREDICTED: protein catecholamines up
RS004110	GO:0055085	transmembrane transport	BP	-0.109326082	-0.245764423	-0.393853677	2.856144774	0.536964028	0.910698682	0.95276119	PREDICTED: phospholipase A2 inhibitor and Ly6/PLAUR domain-containing protein
RS015459	GO:0055085	transmembrane transport	BP	0.206612935	0.259250681	-0.103050012	2.119306922	0.506263052	0.917513308	0.95726639	PREDICTED: synaptic vesicular amine transporter
RS010583	GO:0055085	transmembrane transport	BP	-0.204132095	-0.051330039	0.096105151	3.162457527	0.486088584	0.921936886	0.959149956	PREDICTED: partitioning defective protein 6
RS000285	GO:0055085	transmembrane transport	BP	0.119687123	0.031475064	-0.04428715	4.541388379	0.422896761	0.935472985	0.966238561	PREDICTED: facilitated trehalose transporter Tret1-like
RS007128	GO:0055085	transmembrane transport	BP	0.030616457	-0.137872106	-0.048843691	6.138938535	0.365078568	0.947358098	0.972178127	PREDICTED: probable multidrug resistance-associated protein lethal(2)03659
RS009877	GO:0055085	transmembrane transport	BP	-0.00905179	0.103629481	0.021581239	6.403084093	0.354667887	0.949439292	0.973082555	PREDICTED: integral membrane protein GPR155
RS002672	GO:0055085	transmembrane transport	BP	0.112781414	0.136232963	0.12426065	4.219978077	0.286688066	0.96251234	0.980258807	PREDICTED: mitochondrial import inner membrane translocase subunit Tim22
RS005492	GO:0055085	transmembrane transport	BP	0.113658098	0.003287766	-0.018147387	4.461362746	0.267426626	0.966033527	0.982215891	PREDICTED: cell differentiation protein RCD1 homolog
RS007404	GO:0055085	transmembrane transport	BP	0.341548622	0.423696565	0.486833568	3.079113058	0.267214539	0.9660718	0.982215891	PREDICTED: sodium-independent sulfate anion transporter-like
RS009012	GO:0055085	transmembrane transport	BP	0.165169583	0.002447419	-0.023721248	2.359677794	0.265786109	0.966329283	0.982398368	PREDICTED: zinc transporter 2-like
RS010152	GO:0055085	transmembrane transport	BP	-0.129723253	-0.161823215	-0.203053025	1.88639679	0.263658934	0.966711776	0.98246999	PREDICTED: zinc transporter ZIP3
RS007304	GO:0055085	transmembrane transport	BP	-0.03874979	-0.117709759	-0.024621974	4.815387852	0.237457293	0.971327222	0.985126901	PREDICTED: solute carrier family 17 member 9
RS000440	GO:0055085	transmembrane transport	BP	0.057290939	0.025965614	-0.040667409	6.697035751	0.213954489	0.975305737	0.98698276	MFS transporter
RS002585	GO:0055085	transmembrane transport	BP	0.061337763	0.015683494	0.119579633	4.655608643	0.205533408	0.976691112	0.987602807	PREDICTED: mitochondrial pyruvate carrier 1
RS008706	GO:0055085	transmembrane transport	BP	0.063443874	0.058144534	-0.01579188	5.633533699	0.196321115	0.978180999	0.988383465	PREDICTED: ATP-binding cassette sub-family D member 3
RS005614	GO:0055085	transmembrane transport	BP	-0.026120006	0.069729297	0.030053445	4.472662817	0.146084989	0.985784124	0.992693667	PREDICTED: mitochondrial folate transporter/carrier
RS008855	GO:0055085	transmembrane transport	BP	0.02954866	-0.046352565	0.025055269	3.71091616	0.056927361	0.996448634	0.998351161	PREDICTED: solute carrier family 35 member B1
RS006774	GO:0055085	transmembrane transport	BP	-0.005561161	-0.002818033	0.022604747	3.411433614	0.005716578	0.999885243	0.999885243	PREDICTED: equilibrative nucleoside transporter 1
RS015383	GO:0005975	carbohydrate metabolic process	BP	-1.569834839	6.664361201	3.144862999	6.506097454	295.0381467	1.1793E-63	1.35019E-61	PREDICTED: mucin-19-like
RS005688	GO:0005975	carbohydrate metabolic process	BP	2.672306162	6.482393232	4.855110265	7.197261589	222.5847093	5.54588E-48	4.25883E-46	PREDICTED: endochitinase
RS012687	GO:0005975	carbohydrate metabolic process	BP	-2.409771896	-4.903293647	-1.083108244	4.98106813	185.3939853	6.03281E-40	3.7062E-38	endo-beta-1,4-glucanase
RS006382	GO:0005975	carbohydrate metabolic process	BP	2.806378091	4.140514995	2.619769373	8.160379228	161.4739314	8.81125E-35	4.43876E-33	PREDICTED: chitooligosaccharidolytic beta-N-acetylglucosaminidase
RS011942	GO:0005975	carbohydrate metabolic process	BP	-0.498390242	3.974481158	0.858281392	5.803436912	146.0821711	1.84434E-31	8.15005E-30	PREDICTED: 2-hydroxyacylsphingosine 1-beta-galactosyltransferase
RS012684	GO:0005975	carbohydrate metabolic process	BP	-3.104964921	-3.782144518	-3.589335926	3.211411943	144.6587701	3.73965E-31	1.61846E-29	endo-beta-1,4-glucanase
RS004137	GO:0005975	carbohydrate metabolic process	BP	-2.896148666	-5.161794939	-2.574026235	4.923055906	138.5902716	7.61047E-30	3.1425E-28	beta-glucosidase
RS008547	GO:0005975	carbohydrate metabolic process	BP	4.257822268	5.315943791	2.804489573	3.078313137	133.1027165	1.15977E-28	4.55021E-27	PREDICTED: lysozyme-like
RS005687	GO:0005975	carbohydrate metabolic process	BP	2.9402359	5.210386405	2.373472922	3.936368	114.1458443	1.40595E-24	4.73438E-23	PREDICTED: endochitinase
RS004624	GO:0005975	carbohydrate metabolic process	BP	-3.348252991	-4.51370637	-4.324589052	1.820204257	107.1496767	4.50481E-23	1.38375E-21	PREDICTED: myrosinase 1-like
RS002214	GO:0005975	carbohydrate metabolic process	BP	2.792978943	3.034362842	0.560488122	3.879155465	104.1646791	1.97623E-22	5.81512E-21	PREDICTED: carbohydrate sulfotransferase 11-like
RS010790	GO:0005975	carbohydrate metabolic process	BP	-0.856541903	2.066285721	1.962202671	8.506195557	94.18028693	2.76994E-20	7.22249E-19	PREDICTED: alpha-N-acetylgalactosaminidase
RS009651	GO:0005975	carbohydrate metabolic process	BP	-3.154372842	-4.783001161	-2.730899772	3.45854155	93.57471858	3.73763E-20	9.60647E-19	PREDICTED: maltase 1-like
RS007234	GO:0005975	carbohydrate metabolic process	BP	0.052513072	1.943902873	1.008743446	6.066222799	84.95578614	2.65245E-18	5.93338E-17	PREDICTED: phosphoacetylglucosamine mutase
RS012436	GO:0005975	carbohydrate metabolic process	BP	-3.287733804	-9.017974104	-2.90703098	4.455905937	84.49872826	3.32469E-18	7.38468E-17	beta-glucosidase
RS005235	GO:0005975	carbohydrate metabolic process	BP	-0.316190702	0.021797675	2.848580935	8.976414933	82.72590425	7.98393E-18	1.71003E-16	PREDICTED: glutamine--fructose-6-phosphate aminotransferase [isomerizing] 2-like
RS004205	GO:0005975	carbohydrate metabolic process	BP	1.324987867	5.035657228	-0.333819443	2.580237392	81.31706065	1.6014E-17	3.35576E-16	AAEL005685-PA
RS010860	GO:0005975	carbohydrate metabolic process	BP	-0.513060367	-1.867151173	-2.038852609	4.807472629	80.18504484	2.8012E-17	5.72699E-16	PREDICTED: fucose mutarotase
RS010590	GO:0005975	carbohydrate metabolic process	BP	-2.57016287	-4.977699687	-4.728351418	1.94352856	79.20706072	4.54057E-17	9.19355E-16	PREDICTED: acyl-CoA synthetase short-chain family member 3, mitochondrial
RS011853	GO:0005975	carbohydrate metabolic process	BP	-1.313648639	-2.478519389	-2.71556709	4.85148492	76.50416751	1.72458E-16	3.38308E-15	PREDICTED: aldehyde dehydrogenase, dimeric NADP-preferring
RS013075	GO:0005975	carbohydrate metabolic process	BP	-0.648211227	0.977122303	2.242125965	5.568091919	75.83995915	2.39369E-16	4.61657E-15	maltase A8
RS002034	GO:0005975	carbohydrate metabolic process	BP	-2.76074966	-3.66938775	0.182700124	4.670360092	71.90234509	1.67045E-15	3.00969E-14	PREDICTED: 4-hydroxyphenylpyruvate dioxygenase
RS008613	GO:0005975	carbohydrate metabolic process	BP	-1.584932115	4.282216911	3.741683308	2.697038089	68.69402362	8.1259E-15	1.39424E-13	lysozyme
RS009152	GO:0005975	carbohydrate metabolic process	BP	-0.076188961	2.318086608	-0.327763265	4.849879578	67.57555546	1.4102E-14	2.35857E-13	PREDICTED: alpha-(1,6)-fucosyltransferase
RS005437	GO:0005975	carbohydrate metabolic process	BP	-0.899257268	-2.245545308	-2.877195377	7.631293432	66.09329323	2.92732E-14	4.80661E-13	PREDICTED: acylphosphatase-2
RS013980	GO:0005975	carbohydrate metabolic process	BP	1.70171747	1.349492418	0.304429583	5.412132669	65.9064818	3.20951E-14	5.24943E-13	PREDICTED: phosphomannomutase
RS008102	GO:0005975	carbohydrate metabolic process	BP	1.547668341	1.36254646	-0.943790628	4.934583524	63.75992297	9.23805E-14	1.46161E-12	PREDICTED: glucosamine-6-phosphate isomerase
RS014869	GO:0005975	carbohydrate metabolic process	BP	-1.914036711	-6.718981489	-3.106950957	9.206887901	62.42555436	1.78189E-13	2.75352E-12	PREDICTED: glucosylceramidase-like
RS002511	GO:0005975	carbohydrate metabolic process	BP	-0.69900862	-1.454715384	-1.592361101	6.212273362	61.91143998	2.29498E-13	3.52906E-12	PREDICTED: glycogen [starch] synthase
RS015462	GO:0005975	carbohydrate metabolic process	BP	-2.562181511	-1.874035905	0.960657982	8.649528892	59.49483899	7.53639E-13	1.1127E-11	PREDICTED: probable citrate synthase 2, mitochondrial
RS009917	GO:0005975	carbohydrate metabolic process	BP	-2.92853977	-3.567066743	-0.105474701	4.514065574	58.59810319	1.17137E-12	1.7035E-11	PREDICTED: phosphoenolpyruvate carboxykinase [GTP]-like
RS000937	GO:0005975	carbohydrate metabolic process	BP	-3.809269902	1.450883882	1.905975853	4.944456301	56.13721158	3.92703E-12	5.47697E-11	PREDICTED: probable chitinase 3
RS002266	GO:0005975	carbohydrate metabolic process	BP	-1.93030545	-2.304011818	0.984730367	5.553897465	53.11063075	1.73645E-11	2.28038E-10	PREDICTED: ADP-sugar pyrophosphatase
RS011602	GO:0005975	carbohydrate metabolic process	BP	-1.476553362	-1.897162637	0.231276491	7.109501682	52.29111173	2.59635E-11	3.3675E-10	PREDICTED: acetyl-coenzyme A synthetase
RS001567	GO:0005975	carbohydrate metabolic process	BP	0.94709573	2.078531773	2.495021424	5.4172828	48.88614692	1.37933E-10	1.70139E-09	PREDICTED: beta-glucuronidase
RS002240	GO:0005975	carbohydrate metabolic process	BP	-0.851489546	0.204102513	1.146678862	4.735548097	48.55844091	1.61966E-10	1.98231E-09	PREDICTED: probable pyruvate dehydrogenase E1 component subunit alpha, mitochondrial
RS013820	GO:0005975	carbohydrate metabolic process	BP	3.31440563	1.763128357	-0.025225406	6.982603202	48.49815976	1.66822E-10	2.03779E-09	PREDICTED: probable chitinase 3
RS014755	GO:0005975	carbohydrate metabolic process	BP	-1.512321011	0.776695379	0.224272966	4.668653569	47.24300788	3.08558E-10	3.66602E-09	PREDICTED: aldose 1-epimerase
RS004116	GO:0005975	carbohydrate metabolic process	BP	0.34130734	-0.013101169	-1.46458415	7.02828743	45.18490377	8.45222E-10	9.73009E-09	PREDICTED: alpha,alpha-trehalose-phosphate synthase [UDP-forming]
RS002376	GO:0005975	carbohydrate metabolic process	BP	-0.179988011	-1.296681685	-1.811164701	3.809033977	41.44787524	5.25439E-09	5.6032E-08	PREDICTED: beta-glucuronidase
RS015505	GO:0005975	carbohydrate metabolic process	BP	-0.873277592	-1.775991461	-0.249713989	6.67190604	41.13584257	6.11945E-09	6.48178E-08	PREDICTED: probable methylmalonate-semialdehyde dehydrogenase [acylating], mitochondrial
RS008454	GO:0005975	carbohydrate metabolic process	BP	-2.842795397	-1.221885642	-1.98495974	4.87291922	40.59570098	7.96643E-09	8.31917E-08	PREDICTED: hyaluronidase-like
RS007194	GO:0005975	carbohydrate metabolic process	BP	-0.781842764	0.15844059	0.993190363	7.241530464	39.30278224	1.49733E-08	1.51708E-07	PREDICTED: hexokinase type 2
RS001009	GO:0005975	carbohydrate metabolic process	BP	-1.20218242	0.618094069	-0.379338777	5.010024276	39.11586683	1.64028E-08	1.65394E-07	PREDICTED: alpha-glucosidase-like
RS001234	GO:0005975	carbohydrate metabolic process	BP	-3.049313173	1.196358565	1.454889377	7.193983845	38.01690606	2.80328E-08	2.73043E-07	chitin deacetylase 4
RS009918	GO:0005975	carbohydrate metabolic process	BP	-3.041004311	-4.02502758	-0.121253316	1.30445851	36.72193195	5.26883E-08	5.02694E-07	PREDICTED: phosphoenolpyruvate carboxykinase [GTP]-like
RS005966	GO:0005975	carbohydrate metabolic process	BP	0.813382742	1.201323264	-0.273628464	6.163653891	36.410532	6.13166E-08	5.8193E-07	PREDICTED: protein O-mannosyltransferase 1
RS002163	GO:0005975	carbohydrate metabolic process	BP	-0.458782111	1.077749636	0.903170703	4.233761172	36.05963192	7.27411E-08	6.84679E-07	PREDICTED: beta-1,3-galactosyltransferase 5
RS007780	GO:0005975	carbohydrate metabolic process	BP	-0.274718828	-1.384032675	-1.005743733	7.135030002	35.91237691	7.81471E-08	7.33372E-07	PREDICTED: UTP--glucose-1-phosphate uridylyltransferase
RS013303	GO:0005975	carbohydrate metabolic process	BP	-1.533487737	-1.764835534	-1.412789568	4.061167435	34.36253174	1.66105E-07	1.49959E-06	PREDICTED: trehalase
RS003758	GO:0005975	carbohydrate metabolic process	BP	-0.559608852	2.208187894	1.597392533	6.933108648	31.62779144	6.2693E-07	5.36383E-06	chitin deacetylase 3
RS007469	GO:0005975	carbohydrate metabolic process	BP	-0.888748458	-2.227463608	-1.919844509	7.982695057	31.31984973	7.27926E-07	6.17756E-06	PREDICTED: retinal dehydrogenase 1
RS004623	GO:0005975	carbohydrate metabolic process	BP	-2.503638382	-2.762777991	-1.571976523	3.240115219	31.12897897	7.98518E-07	6.75388E-06	beta-glucosidase
RS004149	GO:0005975	carbohydrate metabolic process	BP	-0.781883137	-1.38009237	-0.360021571	5.743385186	29.8826953	1.46073E-06	1.20003E-05	beta-glucosidase
RS011175	GO:0005975	carbohydrate metabolic process	BP	-0.885455574	-1.277486072	0.737920476	7.001021188	29.37999588	1.86326E-06	1.5023E-05	PREDICTED: sorbitol dehydrogenase-like
RS006150	GO:0005975	carbohydrate metabolic process	BP	-1.731195553	-1.104009887	-0.435849878	6.16428314	28.13074802	3.40961E-06	2.6425E-05	PREDICTED: 6-phosphofructo-2-kinase/fructose-2,6-bisphosphatase 1
RS007102	GO:0005975	carbohydrate metabolic process	BP	-0.78190331	-0.653917209	0.250142998	5.561485709	27.98775305	3.6536E-06	2.81945E-05	PREDICTED: glycerol-3-phosphate dehydrogenase [NAD(+)], cytoplasmic
RS011800	GO:0005975	carbohydrate metabolic process	BP	-1.018570964	-2.603412306	-0.804726968	3.250067261	27.61409107	4.37655E-06	3.33848E-05	PREDICTED: serine dehydratase-like
RS012315	GO:0005975	carbohydrate metabolic process	BP	1.697220002	1.23705615	0.222802261	5.173892057	26.82332703	6.41142E-06	4.78067E-05	PREDICTED: chitooligosaccharidolytic beta-N-acetylglucosaminidase
RS005093	GO:0005975	carbohydrate metabolic process	BP	-1.490825093	-1.761448973	-3.067688799	4.130528041	26.76253338	6.60231E-06	4.90268E-05	PREDICTED: trehalase
RS015186	GO:0005975	carbohydrate metabolic process	BP	-1.21066846	0.888184276	1.336908068	10.2190989	26.71453317	6.75703E-06	5.00577E-05	chitin deacetylase 1
RS012511	GO:0005975	carbohydrate metabolic process	BP	-0.895186296	-1.106741169	-0.043602132	7.221167871	25.40830777	1.26843E-05	9.06102E-05	PREDICTED: uncharacterized family 31 glucosidase KIAA1161
RS004146	GO:0005975	carbohydrate metabolic process	BP	-1.688114425	-2.072876715	0.538613737	2.834411706	25.07930577	1.48621E-05	0.000104683	beta-glucosidase
RS010653	GO:0005975	carbohydrate metabolic process	BP	0.929493425	1.239889553	0.672676716	6.281928504	24.97161216	1.5653E-05	0.000109579	PREDICTED: ribulose-phosphate 3-epimerase
RS008744	GO:0005975	carbohydrate metabolic process	BP	0.963910383	0.936155852	-0.621160591	5.690387724	24.6305045	1.84458E-05	0.000127641	Poly(ADP-ribose) glycohydrolase
RS007582	GO:0005975	carbohydrate metabolic process	BP	-1.229970149	-1.592228859	-1.074598552	7.903923744	24.61081903	1.86213E-05	0.000128785	PREDICTED: malate dehydrogenase, cytoplasmic

RS008160	GO:0005975	carbohydrate metabolic process	BP	-1.86019723	-1.119619791	1.214513641	3.193269805	24.5107924	1.95394E-05	0.000134543	PREDICTED: UDP-glucuronosyltransferase 2C1-like
RS002847	GO:0005975	carbohydrate metabolic process	BP	-0.668596166	-2.688690132	-1.743374025	5.123821249	23.39560667	3.33974E-05	0.000222073	gram negative bacteria binding protein 2
RS100006	GO:0005975	carbohydrate metabolic process	BP	-0.939930455	-1.245686996	-0.06937368	6.4757339	22.86303754	4.31264E-05	0.00028011	beta-glucosidase
RS006348	GO:0005975	carbohydrate metabolic process	BP	0.461223391	1.781215986	0.898709787	2.791849317	22.54176148	5.03124E-05	0.000323944	PREDICTED: polypeptide N-acetylgalactosaminyltransferase 2
RS012439	GO:0005975	carbohydrate metabolic process	BP	-1.325809551	-2.415216704	-0.443242541	6.490526607	22.01883933	6.4645E-05	0.000408294	beta-glucosidase
RS012932	GO:0005975	carbohydrate metabolic process	BP	-1.049335672	-1.635763072	-1.382307762	3.469160191	21.88763407	6.88387E-05	0.000432396	PREDICTED: beta-1,4-mannosyltransferase egh
RS004183	GO:0005975	carbohydrate metabolic process	BP	0.222949707	0.663380198	-0.959888903	8.761852965	21.44964401	8.49003E-05	0.000525681	PREDICTED: 6-phosphofructo-2-kinase/fructose-2,6-bisphosphatase-like
RS011502	GO:0005975	carbohydrate metabolic process	BP	0.997139085	1.110937602	0.643258028	7.455027006	21.29469842	9.14347E-05	0.000561174	PREDICTED: 3-hydroxyacyl-CoA dehydrogenase type-2
RS004136	GO:0005975	carbohydrate metabolic process	BP	-2.318814572	-3.717518761	-2.158526792	0.752259207	20.84449334	0.000113402	0.000682687	beta-glucosidase
RS004147	GO:0005975	carbohydrate metabolic process	BP	-1.800178068	-2.349728235	-0.154511794	3.857268857	20.69549558	0.000121772	0.000730634	beta-glucosidase
RS004481	GO:0005975	carbohydrate metabolic process	BP	1.158258886	1.648956931	2.316008472	2.609786787	20.65713141	0.000124025	0.000742383	PREDICTED: beta-1,3-galactosyltransferase 5
RS015187	GO:0005975	carbohydrate metabolic process	BP	0.013748187	1.340727689	1.795231312	10.35011632	20.46140263	0.000136183	0.000809002	chitin deacetylase 1 precursor
RS013083	GO:0005975	carbohydrate metabolic process	BP	4.085119458	3.369322613	2.838285311	1.429700382	20.16145088	0.000157154	0.000920129	maltase B2
RS009017	GO:0005975	carbohydrate metabolic process	BP	-0.045189261	0.043390885	0.916173056	6.536550332	19.702086	0.000195664	0.001120594	PREDICTED: beta-1,3-galactosyltransferase 5-like
RS100005	GO:0005975	carbohydrate metabolic process	BP	-2.639688266	-3.107895143	0.367307699	1.207651888	19.4971158	0.00021575	0.001225045	beta-glucosidase
RS001711	GO:0005975	carbohydrate metabolic process	BP	0.690769604	1.181462709	0.955368065	6.847403504	19.20530543	0.000247934	0.001389623	PREDICTED: probable dolichol-phosphate mannosyltransferase
RS013798	GO:0005975	carbohydrate metabolic process	BP	0.748146493	0.320682105	-0.252653913	7.524922409	19.18495899	0.000250349	0.001401285	PREDICTED: neutral alpha-glucosidase AB
RS014754	GO:0005975	carbohydrate metabolic process	BP	-0.658876088	-1.058888095	-0.553090263	5.968197725	19.17453226	0.000251595	0.001406386	PREDICTED: aldose 1-epimerase-like
RS002509	GO:0005975	carbohydrate metabolic process	BP	0.07347544	-0.720252922	-0.547625565	6.183077943	18.51719795	0.000344005	0.001866607	PREDICTED: poly [ADP-ribose] polymerase
RS012721	GO:0005975	carbohydrate metabolic process	BP	-0.370726844	-1.082307086	-1.179144733	3.763910521	18.2954932	0.000382241	0.002044138	phosphoenolpyruvate phosphomutase
RS005416	GO:0005975	carbohydrate metabolic process	BP	-0.412284439	-1.050899627	-2.077424575	2.957561508	17.37798325	0.000590855	0.003035999	PREDICTED: threo-3-hydroxyaspartate ammonia-lyase
RS003705	GO:0005975	carbohydrate metabolic process	BP	0.057476585	-0.874131429	-1.326214826	5.440373792	17.33177411	0.000603941	0.003090627	PREDICTED: alpha-L-fucosidase-like
RS000041	GO:0005975	carbohydrate metabolic process	BP	-0.970092086	-1.799060718	-1.441059447	4.392482413	16.56903986	0.000866634	0.004310581	PREDICTED: inositol oxygenase
RS007611	GO:0005975	carbohydrate metabolic process	BP	0.85433042	0.661811427	0.970900853	6.298492276	16.20186994	0.001030876	0.005030163	Cytochrome b-c1 complex subunit 2, mitochondrial
RS010935	GO:0005975	carbohydrate metabolic process	BP	-0.955349572	-0.110515396	0.342411356	6.328877639	15.71272059	0.001298602	0.006129907	PREDICTED: integrin-linked protein kinase
RS100020	GO:0005975	carbohydrate metabolic process	BP	1.876366294	-0.446669319	-0.081800473	0.756075062	15.23454326	0.001626799	0.00747461	gram negative bacteria binding protein 1
RS006136	GO:0005975	carbohydrate metabolic process	BP	-2.199659373	0.052075633	2.322759219	0.896680658	15.0374978	0.001784883	0.008094642	alpha-amylase
RS006197	GO:0005975	carbohydrate metabolic process	BP	-1.725611249	-5.831665088	-0.587837998	0.462052021	15.01327909	0.001805336	0.008178562	PREDICTED: maltase 1-like
RS014418	GO:0005975	carbohydrate metabolic process	BP	-0.478113679	-0.820374615	0.805474325	2.345331841	14.9793906	0.001834346	0.008283168	PREDICTED: alpha-1,6-mannosyl-glycoprotein 2-beta-N-acetylglucosaminyltransferase
RS014698	GO:0005975	carbohydrate metabolic process	BP	4.124573171	5.181690304	3.981684124	-0.013147706	14.75841544	0.002035182	0.009076163	PREDICTED: lysozyme-like
RS011405	GO:0005975	carbohydrate metabolic process	BP	-1.520391219	-1.806294063	-1.56720976	8.916277625	14.69755053	0.002094233	0.00929016	PREDICTED: inositol-3-phosphate synthase 1-B
RS011851	GO:0005975	carbohydrate metabolic process	BP	-2.062046011	-1.929191517	-0.666540417	3.362371455	14.66062266	0.002130884	0.009432446	PREDICTED: putative glycerol kinase 5
RS002096	GO:0005975	carbohydrate metabolic process	BP	0.464617661	0.653577558	0.843133376	7.017229304	14.60721112	0.002185025	0.009631853	PREDICTED: lactoylglutathione lyase
RS100007	GO:0005975	carbohydrate metabolic process	BP	-2.427088717	-2.224912825	-0.099362221	6.432736706	14.45449785	0.002347463	0.010275964	beta-glucosidase
RS015477	GO:0005975	carbohydrate metabolic process	BP	-1.101064815	-0.393684254	-0.930550173	3.969326348	14.3061796	0.002516672	0.010910488	PREDICTED: pyruvate carboxylase, mitochondrial
RS006137	GO:0005975	carbohydrate metabolic process	BP	-1.750611457	-0.496084864	1.146600014	1.717250447	14.23346103	0.002603996	0.011227225	alpha-amylase
RS015559	GO:0005975	carbohydrate metabolic process	BP	-2.383612554	-2.605044757	-1.26251376	0.481679586	14.22941496	0.002608942	0.0112447	PREDICTED: probable beta-hexosaminidase fdl
RS004154	GO:0005975	carbohydrate metabolic process	BP	-1.099636409	-0.574925083	-0.313075483	5.604418011	13.91327386	0.00302561	0.012748256	PREDICTED: trehalase
RS011854	GO:0005975	carbohydrate metabolic process	BP	-1.112002956	0.070911347	-0.516191421	6.87646831	13.85956875	0.003102671	0.013038052	PREDICTED: aldehyde dehydrogenase, dimeric NADP-preferring
RS012437	GO:0005975	carbohydrate metabolic process	BP	-1.605717173	-1.261098214	0.809372784	1.627644662	13.7577251	0.003254184	0.013629264	beta-glucosidase
RS012390	GO:0005975	carbohydrate metabolic process	BP	-0.716673568	-0.893126341	-0.183509175	7.550045761	13.67242105	0.003386715	0.014118599	phosphoglucose isomerase
RS009242	GO:0005975	carbohydrate metabolic process	BP	0.682073862	0.906384828	-0.119195863	3.436386452	13.55373124	0.003580051	0.014826428	PREDICTED: putative N-acetylglucosamine-6-phosphate deacetylase
RS004144	GO:0005975	carbohydrate metabolic process	BP	-1.68344632	-2.290583943	0.372324576	1.860972949	13.53943372	0.003604066	0.014911174	beta-glucosidase
RS000144	GO:0005975	carbohydrate metabolic process	BP	-0.589405346	0.96241749	0.84562015	7.342211715	13.21351442	0.0041969	0.017011829	PREDICTED: probable chitinase 2
RS002313	GO:0005975	carbohydrate metabolic process	BP	-0.451678341	0.180904234	-0.854553222	5.296298932	13.08468271	0.004457007	0.017899092	PREDICTED: glucose-6-phosphate 1-dehydrogenase
RS010291	GO:0005975	carbohydrate metabolic process	BP	-1.148046164	-1.387251265	0.083086546	1.759013597	13.03205805	0.004567788	0.018268253	PREDICTED: galactoside 2-alpha-L-fucosyltransferase 3-like
RS009335	GO:0005975	carbohydrate metabolic process	BP	0.731058414	-0.369381984	0.309156103	5.654254453	12.91117936	0.004832676	0.01918749	PREDICTED: beta-galactosidase
RS000004	GO:0005975	carbohydrate metabolic process	BP	-0.265184605	-0.172398945	0.468191605	5.017738015	12.78135077	0.005134116	0.020187032	PREDICTED: glycerol kinase-like
RS005740	GO:0005975	carbohydrate metabolic process	BP	0.03115726	1.018554939	1.081039447	5.490146434	12.59344835	0.005603609	0.021761285	PREDICTED: putative epidermal cell surface receptor
RS006766	GO:0005975	carbohydrate metabolic process	BP	-0.318612109	-0.764780567	0.962282512	4.270639859	12.4793297	0.005909213	0.022737743	PREDICTED: alpha-(1,3)-fucosyltransferase 7-like
RS011888	GO:0005975	carbohydrate metabolic process	BP	-0.067075112	1.226758207	-0.103918657	4.829095091	12.38516055	0.006173758	0.023618563	PREDICTED: mucin-5AC
RS006342	GO:0005975	carbohydrate metabolic process	BP	0.734709905	0.839191818	0.013573097	6.490494103	12.21838984	0.006671291	0.025291462	PREDICTED: N-acetylgalactosaminyltransferase 7
RS006443	GO:0005975	carbohydrate metabolic process	BP	0.143296238	-0.770738062	-0.789685541	3.548052193	12.1322361	0.006943634	0.026189915	PREDICTED: alpha,alpha-trehalose-phosphate synthase [UDP-forming]
RS007511	GO:0005975	carbohydrate metabolic process	BP	-1.07942099	-1.056011151	0.717242571	9.592507232	12.08601869	0.007094231	0.026640194	PREDICTED: chitinase-like protein Idgf4
RS006054	GO:0005975	carbohydrate metabolic process	BP	-0.032318639	0.735949508	1.059453249	7.179426322	11.93109062	0.007622982	0.028278006	PREDICTED: lysozyme-like
RS015567	GO:0005975	carbohydrate metabolic process	BP	0.376413076	-0.071504162	-0.448105985	7.311814141	11.60916555	0.008849263	0.032126729	PREDICTED: glucosidase 2 subunit beta
RS000672	GO:0005975	carbohydrate metabolic process	BP	-0.356115583	-0.633045826	0.306113021	7.264205469	11.12372943	0.011075297	0.038766616	PREDICTED: maltase A2-like
RS003966	GO:0005975	carbohydrate metabolic process	BP	1.003154417	0.913053421	1.091479083	5.538658211	11.03065715	0.011561251	0.040277286	PREDICTED: hexosaminidase D-like
RS011555	GO:0005975	carbohydrate metabolic process	BP	-0.493021355	-0.206875073	-1.670148888	2.916126599	11.00502936	0.011698711	0.040704945	PREDICTED: L-lactate dehydrogenase-like
RS005798	GO:0005975	carbohydrate metabolic process	BP	0.42141247	0.736875665	0.248473058	5.783054674	10.99667203	0.011743885	0.040797551	PREDICTED: protein O-mannosyl-transferase 2
RS014419	GO:0005975	carbohydrate metabolic process	BP	-0.812097852	-0.035393336	0.555610255	3.206085641	10.88864079	0.012343532	0.042590258	PREDICTED: alpha-1,6-mannosyl-glycoprotein 2-beta-N-acetylglucosaminyltransferase
RS007445	GO:0005975	carbohydrate metabolic process	BP	0.077554822	-0.548800395	-0.821734397	6.266486593	10.87094209	0.012444608	0.042915495	PREDICTED: lysosomal alpha-mannosidase
RS008390	GO:0005975	carbohydrate metabolic process	BP	0.086808415	1.001303883	0.637061954	4.646844201	10.60922931	0.014037947	0.047525244	PREDICTED: putative polypeptide N-acetylgalactosaminyltransferase 9
RS008660	GO:0005975	carbohydrate metabolic process	BP	-0.610580371	-0.844004978	-0.632634161	7.281264035	10.5950421	0.01412984	0.047746499	PREDICTED: glycogen debranching enzyme
RS010463	GO:0005975	carbohydrate metabolic process	BP	-0.857124822	-0.198171941	0.157726924	3.31811352	10.57466098	0.014262888	0.048114201	PREDICTED: carbohydrate sulfotransferase 11
RS015148	GO:0005975	carbohydrate metabolic process	BP	-1.963276978	0.635742593	-0.155791267	2.122363384	10.45700384	0.01505542	0.050360678	PREDICTED: L-threonine 3-dehydrogenase, mitochondrial
RS000485	GO:0005975	carbohydrate metabolic process	BP	-0.695391551	-0.618652868	-0.739307552	7.487588188	10.45272332	0.015085056	0.05039693	PREDICTED: transaldolase
RS003249	GO:0005975	carbohydrate metabolic process	BP	-0.207283219	-0.369134294	-0.971045776	4.452945458	10.45254875	0.015086265	0.05039693	PREDICTED: protein O-linked-mannose beta-1,2-N-acetylglucosaminyltransferase 1
RS014260	GO:0005975	carbohydrate metabolic process	BP	0.847652686	-0.135314424	0.806100051	5.052649555	10.30574059	0.016138267	0.053359236	PREDICTED: polypeptide N-acetylgalactosaminyltransferase 3
RS005196	GO:0005975	carbohydrate metabolic process	BP	-0.62031121	-0.946710459	0.084812792	3.467267912	10.09431996	0.017781164	0.057765203	PREDICTED: hexokinase-1-like
RS010825	GO:0005975	carbohydrate metabolic process	BP	-0.11178442	-0.611021406	-0.795698296	5.293267172	10.03266739	0.018290482	0.059109656	PREDICTED: fructose-1,6-bisphosphatase 1
RS008456	GO:0005975	carbohydrate metabolic process	BP	-1.182722026	-0.163606113	-0.490216356	6.804540813	9.986465731	0.018681533	0.060172691	PREDICTED: cyclin-dependent kinase 8-like
RS003668	GO:0005975	carbohydrate metabolic process	BP	0.304173488	0.556834022	-1.582773639	1.31138541	9.918990955	0.019267443	0.061643235	PREDICTED: lysosomal alpha-glucosidase
RS005636	GO:0005975	carbohydrate metabolic process	BP	-0.453071987	-0.038123733	0.527166131	7.855258218	9.838636626	0.019988779	0.063503274	PREDICTED: dihydrolipoyl dehydrogenase, mitochondrial
RS000414	GO:0005975	carbohydrate metabolic process	BP	-0.176674877	0.307712802	0.425329171	5.806265794	9.510889408	0.023215797	0.071908448	PREDICTED: FGGY carbohydrate kinase domain-containing protein
RS012938	GO:0005975	carbohydrate metabolic process	BP	-1.846939656	-0.327189547	0.081992913	1.097980344	9.391278764	0.024516548	0.075087891	PREDICTED: beta-1,3-galactosyltransferase 5
RS002848	GO:0005975	carbohydrate metabolic process	BP	-0.038556699	-1.074896303	0.55472811	3.748995474	9.313160824	0.02540438	0.077150414	beta-1,3-glucanase
RS011064	GO:0005975	carbohydrate metabolic process	BP	-1.082972026	-1.027491454	-0.806343821	3.290957675	9.019949401	0.029026821	0.085833243	PREDICTED: glycoprotein 3-alpha-L-fucosyltransferase A
RS007528	GO:0005975	carbohydrate metabolic process	BP	1.281551474	0.79778074	0.871132273	9.026899419	8.962975306	0.029787222	0.087649596	ubiquitin
RS002022	GO:0005975	carbohydrate metabolic process	BP	0.62041628	0.278617857	0.05008813	5.14866597	8.75565426	0.032722342	0.094736821	PREDICTED: ADP-ribosylation factor-like protein 1
RS010234	GO:0005975	carbohydrate metabolic process	BP	-0.015484323	0.41266545	0.730886441	7.257240265	8.642929696	0.034435063	0.098562543	PREDICTED: pyruvate dehydrogenase E1 component subunit beta, mitochondrial
RS014200	GO:0005975	carbohydrate metabolic process	BP	0.368512158	-0.23900377	-0.315539871	4.740713374	8.620559497	0.034785227	0.09929734	PREDICTED: sphingosine-1-phosphate lyase
RS000183	GO:0005975	carbohydrate metabolic process	BP	0.194496357	-0.265263435	-0.399764104	6.003449428	8.522470401	0.036362154	0.102591942	PREDICTED: uncharacterized family 31 glucosidase KIAA1161
RS015009	GO:0005975	carbohydrate metabolic process	BP	0.452819	0.56796968	0.343760656	6.19776174	8.407300491	0.038302942	0.107030675	PREDICTED: dolichyl-diphosphooligosaccharide--protein glycosyltransferase subunit DAD1
RS010148	GO:0005975	carbohydrate metabolic process	BP	0.609571952	0.691443267	0.551683094	6.423538019	8.396934265	0.03848251	0.107413283	PREDICTED: ADP-ribosylation factor 2
RS007468	GO:0005975	carbohydrate metabolic process	BP	0.457030598	-0.368844241	-0.214826608	7.85084108	8.327687594	0.039703285	0.110210089	PREDICTED: retinal dehydrogenase 1
RS014206	GO:0005975	carbohydrate metabolic process	BP	-0.331060594	-0.319039029	0.770529033	2.915958338	8.315762111	0.039917305	0.110682196	PREDICTED: mannosyl-oligosaccharide 1,2-alpha-mannosidase IA
RS000901	GO:0005975	carbohydrate metabolic process	BP	0.411349349	-0.685418002	-1.18852688	1.856570664	8.117241058	0.043649821	0.118859642	PREDICTED: glucose-6-phosphate 1-epimerase
RS012691	GO:0005975	carbohydrate metabolic process	BP	-0.172595861	-0.578904307	-0.031671583	6.933895552	8.088105422	0.044225497	0.120245231	PREDICTED: 1,4-alpha-glucan-branching enzyme
RS010542	GO:0005975	carbohydrate metabolic process	BP	0.116103344	-0.618699857	-0.860552226	2.745008544	7.913554572	0.047832468	0.127853488	PREDICTED: sialic acid synthase
RS013076	GO:0005975	carbohydrate metabolic process	BP	-0.620955721	-0.518605183	-0.435868886	7.477326793	7.683889148	0.053017124	0.13864154	PREDICTED: neutral and basic amino acid transport protein rBAT
RS003700	GO:0005975	carbohydrate metabolic process	BP	0.445500208	0.547805665	0.90099913	5.029149185	7.491054113	0.057788756	0.148619889	PREDICTED: UDP-glucose 6-dehydrogenase
RS005354	GO:0005975	carbohydrate metabolic process	BP	-1.00471418	-0.664056052	-0.408294102	2.883865853	7.337282876	0.061890101	0.156389668	PREDICTED: alpha-1,3-mannosyl-glycoprotein 4-beta-N-acetylglucosaminyltransferase B
RS012830	GO:0005975	carbohydrate metabolic process	BP	0.512471195	0.69308213	0.478993909	7.410547394	7.303674851	0.062823366	0.158207852	PREDICTED: glycosyltransferase-like protein LARGE1
RS002662	GO:0005975	carbohydrate metabolic process	BP	0.548562285	0.643121214	0.480591626	7.386807743	6.793922491	0.078764437	0.18733887	PREDICTED: phosphorylase b kinase gamma catalytic chain, skeletal muscle/heart isoform
RS011679	GO:0005975	carbohydrate metabolic process	BP	-0.054688789	-0.103097471	-0.893891243	3.451449564	6.752534052	0.080217901	0.190077939	PREDICTED: N-acetylgalactosamine kinase
RS014429	GO:0005975	carbohydrate metabolic process	BP	0.590745261	0.872389057	0.269920982	4.617355118	6.586926525	0.086296659	0.201486859	PREDICTED: cytosolic endo-beta-N-acetylglucosaminidase
RS000279	GO:0005975	carbohydrate metabolic process	BP	-0.156609271	-0.975620437	-1.13346223	1.519250101	6.401800896	0.093616731	0.214191532	PREDICTED: glycerophosphodiester phosphodiesterase domain-containing protein 1-like
RS001568	GO:0005975	carbohydrate metabolic process	BP	-0.664679343	-0.257471752	-0.520595381	5.009946007	6.3478878	0.095858382	0.2177955	PREDICTED: beta-glucuronidase
RS004097	GO:0005975	carbohydrate metabolic process	BP	-0.320696314	-0.761974874	-0.592051235	7.045695376	6.243550756	0.100343827	0.224854953	PREDICTED: probable chitinase 2
RS007508	GO:0005975	carbohydrate metabolic process	BP	-0.206277528	-0.50989722	0.094040606	5.606337763	6.210422615	0.101809632	0.227222865	PREDICTED: alpha-mannosidase 2
RS006890	GO:0005975	carbohydrate metabolic process	BP	0.282954507	-1.7084021	-0.060666224	0.535603492	6.133135493	0.105309529	0.233251531	PREDICTED: malate synthase, glyoxysomal-like
RS013727	GO:0005975	carbohydrate metabolic process	BP	-0.694005627	-0.555219064	-0.764422667	3.314031676	6.109365741	0.106408898	0.235066421	PREDICTED: WD repeat and FYVE domain-containing protein 3
RS008343	GO:0005975	carbohydrate metabolic process	BP	0.425195192	0.621020602	0.508117655	4.703481584	6.084095192	0.107589727	0.236885493	PREDICTED: GDP-mannose 4,6 dehydratase
RS013092	GO:0005975	carbohydrate metabolic process	BP	-1.21991486	-0.311211194	-1.526773013	0.464605072	5.999428186	0.111638048	0.243540502	PREDICTED: polypeptide N-acetylgalactosaminyltransferase 2
RS002734	GO:0005975	carbohydrate metabolic process	BP	0.299931428	-0.05370325	-0.483067767	7.336391207	5.907492348	0.116198696	0.250669128	PREDICTED: dolichyl-diphosphooligosaccharide--protein glycosyltransferase subunit 1
RS014729	GO:0005975	carbohydrate metabolic process	BP	-1.272976392	-1.097970962	-0.527420749	2.348202298	5.808104611	0.121328885	0.258635068	PREDICTED: phosphoglycerate mutase 1
RS009661	GO:0005975	carbohydrate metabolic process	BP	0.59943041	0.211937076	-0.085571172	7.380254275	5.737164679	0.125121951	0.264971558	PREDICTED: dolichyl-diphosphooligosaccharide--protein glycosyltransferase subunit 2
RS013091	GO:0005975	carbohydrate metabolic process	BP	-0.634795047	0.023977642	-0.224683025	3.598027978	5.731282535	0.125441482	0.265335912	PREDICTED: polypeptide N-acetylgalactosaminyltransferase 14
RS010624	GO:0005975	carbohydrate metabolic process	BP	-1.888144745	-0.350620517	-1.036675402	0.280983459	5.728136435	0.125612703	0.265452684	PREDICTED: exostosin-1
RS011781	GO:0005975	carbohydrate metabolic process	BP	-0.445660127	-0.864112419	-0.573881668	9.678543904	5.72543243	0.125760042	0.265569301	PREDICTED: enolase

RS011118	GO:0005975	carbohydrate metabolic process	BP	-0.543786326	-0.028160748	-0.033265838	4.447786153	5.690180454	0.127696006	0.268526214	PREDICTED: xylulose kinase
RS006444	GO:0005975	carbohydrate metabolic process	BP	0.247684833	-1.158327215	-1.023157283	0.52416464	5.678360053	0.128351481	0.269372103	PREDICTED: alpha,alpha-trehalose-phosphate synthase [UDP-forming]
RS012338	GO:0005975	carbohydrate metabolic process	BP	-0.563620536	-0.376492761	0.024551568	7.936118918	5.617244954	0.131791842	0.274208898	PREDICTED: ATP-dependent 6-phosphofructokinase
RS004143	GO:0005975	carbohydrate metabolic process	BP	0.171248717	-0.378526389	-1.177546392	1.582100775	5.605188731	0.132480796	0.275369391	beta-glucosidase
RS008628	GO:0005975	carbohydrate metabolic process	BP	0.413632194	-0.00514203	-0.172918432	4.324500738	5.494355553	0.138976608	0.285106923	PREDICTED: glycoprotein endo-alpha-1,2-mannosidase
RS005181	GO:0005975	carbohydrate metabolic process	BP	-0.168717926	0.322198012	0.688998249	2.903388389	5.420247571	0.143487277	0.292076736	PREDICTED: beta-1,3-galactosyltransferase 1-like
RS007245	GO:0005975	carbohydrate metabolic process	BP	-0.109691638	-0.538704272	-0.521010048	4.600455628	5.356909305	0.147451966	0.297788657	PREDICTED: beta-1,3-galactosyltransferase 6
RS009599	GO:0005975	carbohydrate metabolic process	BP	-0.629337009	-0.652873003	-0.495532334	9.067326827	5.336457354	0.148754145	0.29979352	PREDICTED: fructose-bisphosphate aldolase
RS011885	GO:0005975	carbohydrate metabolic process	BP	0.560958428	0.381144006	0.226682277	7.447775785	5.334338605	0.148889666	0.299945651	PREDICTED: dolichyl-diphosphooligosaccharide--protein glycosyltransferase subunit STT3B
RS008006	GO:0005975	carbohydrate metabolic process	BP	-1.537762826	-0.128930159	-0.462894442	1.415657251	5.194123574	0.158121985	0.31385158	PREDICTED: beta-1,3-galactosyltransferase 1-like
RS002769	GO:0005975	carbohydrate metabolic process	BP	-0.180596666	-0.510025269	-0.494314256	5.266272897	5.147923967	0.161280396	0.318264698	PREDICTED: mitochondrial 2-oxoglutarate/malate carrier protein
RS003918	GO:0005975	carbohydrate metabolic process	BP	-0.223881737	-0.404666274	-0.599000058	7.422761893	5.141574758	0.161719072	0.318680956	PREDICTED: 6-phosphogluconate dehydrogenase, decarboxylating
RS010331	GO:0005975	carbohydrate metabolic process	BP	0.144296164	0.303288083	0.51514905	5.774666221	5.090804659	0.165267464	0.324101282	PREDICTED: mannose-1-phosphate guanyltransferase beta
RS010665	GO:0005975	carbohydrate metabolic process	BP	0.402212319	0.474203193	0.468533425	6.815729487	5.021744853	0.170211786	0.330665996	PREDICTED: mannose-1-phosphate guanyltransferase alpha-A
RS011641	GO:0005975	carbohydrate metabolic process	BP	-2.074551338	-0.763334889	-1.763867752	0.058250841	4.989449686	0.172571322	0.333696182	PREDICTED: exostosin-1
RS012633	GO:0005975	carbohydrate metabolic process	BP	0.3400712	-0.042971241	-0.128072376	5.95450125	4.828059073	0.184829233	0.350404841	PREDICTED: probable C-mannosyltransferase DPY19L1
RS009184	GO:0005975	carbohydrate metabolic process	BP	0.558555184	0.791658239	0.809029067	4.580170248	4.800804346	0.186977982	0.353149475	PREDICTED: chitinase-3-like protein 1
RS003558	GO:0005975	carbohydrate metabolic process	BP	0.316644445	-0.155731661	-0.244479979	4.492310398	4.785634623	0.188184038	0.354895144	PREDICTED: beta-1,4-N-acetylgalactosaminyltransferase bre-4
RS015579	GO:0005975	carbohydrate metabolic process	BP	0.884534215	1.24377824	0.555762766	1.68070004	4.75061201	0.190996266	0.358213994	PREDICTED: lysozyme-like
RS011065	GO:0005975	carbohydrate metabolic process	BP	-0.875027536	-0.699860567	-0.617836046	1.97912169	4.744920077	0.191457	0.358704174	PREDICTED: glycoprotein 3-alpha-L-fucosyltransferase A
RS012958	GO:0005975	carbohydrate metabolic process	BP	-0.475751151	-0.659652499	-0.592909608	4.258116367	4.709280662	0.194365468	0.362426678	PREDICTED: inositol monophosphatase 1-like
RS003712	GO:0005975	carbohydrate metabolic process	BP	0.03100834	-0.555218793	-0.842108264	3.063114051	4.678534462	0.196907622	0.365525714	PREDICTED: probable dolichyl pyrophosphate Glc1Man9GlcNAc2 alpha-1,3-glucosyltransferase
RS003391	GO:0005975	carbohydrate metabolic process	BP	-0.542480622	-0.539981143	-0.585509501	9.138318653	4.6421131	0.199959035	0.36995506	ubiquitin
RS008973	GO:0005975	carbohydrate metabolic process	BP	0.730569281	0.542878845	0.061507904	5.674408765	4.571253884	0.206021982	0.37712803	PREDICTED: collagen alpha-1(I) chain-like
RS007095	GO:0005975	carbohydrate metabolic process	BP	0.192687609	0.009365646	-0.454674091	8.985348469	4.560229052	0.206980499	0.378277813	PREDICTED: UDP-glucose:glycoprotein glucosyltransferase
RS006209	GO:0005975	carbohydrate metabolic process	BP	0.112226125	-0.275290936	-0.382508167	4.609935365	4.55572841	0.20737298	0.378610512	PREDICTED: acylphosphatase-1-like
RS009944	GO:0005975	carbohydrate metabolic process	BP	-0.459546546	-0.584097686	-0.413731607	3.698979882	4.541470139	0.208620939	0.380067463	PREDICTED: 6-phosphogluconolactonase
RS006396	GO:0005975	carbohydrate metabolic process	BP	-0.48548481	-0.011456222	-0.668681102	4.551562016	4.541901165	0.208583112	0.380067463	PREDICTED: endoglucanase 15-like
RS001183	GO:0005975	carbohydrate metabolic process	BP	0.381173523	0.452466843	0.551870902	6.511626778	4.513584138	0.211081809	0.382773838	PREDICTED: golgi-specific brefeldin A-resistance guanine nucleotide exchange factor 1
RS010206	GO:0005975	carbohydrate metabolic process	BP	0.32104555	-0.070314324	-0.136158126	4.839251455	4.503129415	0.212011321	0.383906624	PREDICTED: JNK1/MAPK8-associated membrane protein
RS012567	GO:0005975	carbohydrate metabolic process	BP	-0.01050941	0.11184936	-0.618760535	3.320616018	4.350385561	0.226030125	0.400031393	PREDICTED: sedoheptulokinase-like
RS011126	GO:0005975	carbohydrate metabolic process	BP	-0.425342683	-0.53256198	0.048696446	8.514475953	4.320223652	0.228897478	0.403292507	PREDICTED: triosephosphate isomerase
RS014997	GO:0005975	carbohydrate metabolic process	BP	-0.068392036	-0.306113841	0.205901561	4.99125296	4.308069794	0.23006232	0.404608974	PREDICTED: lethal(2)neighbour of Tid protein
RS012440	GO:0005975	carbohydrate metabolic process	BP	-0.304237482	-0.482968883	-0.452739024	5.758889288	4.219485676	0.238718226	0.415652888	beta-glucosidase
RS010424	GO:0005975	carbohydrate metabolic process	BP	-0.694975982	-0.087445348	-0.640520311	1.938820184	4.193601382	0.241303289	0.418188197	PREDICTED: glycerophosphodiester phosphodiesterase 1
RS009088	GO:0005975	carbohydrate metabolic process	BP	-0.297613061	0.064032419	0.430473257	9.578338225	4.093175996	0.251576991	0.430249948	PREDICTED: pyruvate kinase-like
RS005564	GO:0005975	carbohydrate metabolic process	BP	-0.206877879	-0.522391276	0.331053646	2.948665139	4.090061953	0.251901852	0.430571652	PREDICTED: glycerol kinase
RS005765	GO:0005975	carbohydrate metabolic process	BP	0.260355785	0.37953603	0.346515019	6.377998666	3.969301547	0.264798145	0.445978195	PREDICTED: glucosidase 2 subunit beta
RS008791	GO:0005975	carbohydrate metabolic process	BP	0.569561428	0.339283371	0.086755342	6.518064701	3.923862465	0.269803845	0.451369504	PREDICTED: dolichyl-diphosphooligosaccharide--protein glycosyltransferase 48 kDa subunit
RS011286	GO:0005975	carbohydrate metabolic process	BP	-0.457520568	-0.346669627	-0.668905633	8.593001753	3.910184477	0.271327336	0.453015574	PREDICTED: moesin/ezrin/radixin homolog 1
RS012431	GO:0005975	carbohydrate metabolic process	BP	0.101037027	-0.018627158	-0.475372339	3.924987682	3.858618333	0.277141065	0.459554256	PREDICTED: UDP-GalNAc:beta-1,3-N-acetylgalactosaminyltransferase 2
RS007814	GO:0005975	carbohydrate metabolic process	BP	-0.022934896	0.013283399	0.724460106	4.088570218	3.831671389	0.280223647	0.463019761	PREDICTED: fringe glycosyltransferase
RS012037	GO:0005975	carbohydrate metabolic process	BP	0.395424512	-0.837435285	-0.608327801	0.739724273	3.818678561	0.281720964	0.464457889	PREDICTED: alpha-N-acetylgalactosaminidase
RS000878	GO:0005975	carbohydrate metabolic process	BP	-0.536410077	-0.620333003	-0.480574696	3.333423954	3.791921767	0.284827164	0.467988351	PREDICTED: protein O-linked-mannose beta-1,2-N-acetylglucosaminyltransferase 1
RS012042	GO:0005975	carbohydrate metabolic process	BP	-0.192210076	-0.454192797	-0.212867275	5.055235089	3.746474687	0.29017371	0.473067663	PREDICTED: galactokinase
RS000280	GO:0005975	carbohydrate metabolic process	BP	0.027606087	0.060990489	-0.378460024	4.875576289	3.658145711	0.300822907	0.483050324	PREDICTED: septin-2
RS000968	GO:0005975	carbohydrate metabolic process	BP	-0.021551596	-0.437622733	0.46889812	2.096576649	3.63634133	0.303504841	0.486179086	Tankyrase-1
RS002269	GO:0005975	carbohydrate metabolic process	BP	-0.261710878	-0.14856721	0.173629907	6.885308754	3.554684137	0.313738721	0.496137057	PREDICTED: succinate-semialdehyde dehydrogenase, mitochondrial
RS009725	GO:0005975	carbohydrate metabolic process	BP	0.2924611	0.113694369	-0.187787882	4.640487227	3.516938508	0.318571889	0.501135944	PREDICTED: ribokinase-like
RS010149	GO:0005975	carbohydrate metabolic process	BP	-0.154098234	-0.466690935	-0.860655645	2.124840163	3.506315269	0.319943975	0.502353125	PREDICTED: O-glucosyltransferase rumi homolog
RS004742	GO:0005975	carbohydrate metabolic process	BP	-0.218056463	-0.458003523	-0.503460198	5.681995461	3.494130187	0.321524214	0.50389198	gram negative bacteria binding protein 1
RS005197	GO:0005975	carbohydrate metabolic process	BP	-0.624767222	-1.128834355	-0.039615784	1.616773135	3.471714778	0.324449191	0.50659968	PREDICTED: hexokinase-1-like
RS009945	GO:0005975	carbohydrate metabolic process	BP	0.395028173	0.529875207	0.572925362	5.902550872	3.471207811	0.324515615	0.506625717	PREDICTED: deoxyribose-phosphate aldolase
RS003202	GO:0005975	carbohydrate metabolic process	BP	0.017348138	0.376526889	0.008547262	4.505003572	3.45732396	0.326339386	0.508275813	PREDICTED: dynactin subunit 6
RS010925	GO:0005975	carbohydrate metabolic process	BP	0.558114996	0.463617513	0.367707791	6.895613326	3.343813411	0.341591039	0.522784973	PREDICTED: protein O-mannose kinase
RS003939	GO:0005975	carbohydrate metabolic process	BP	0.199968947	-0.031637855	-0.234477887	5.351704009	3.333286984	0.343036523	0.524296355	meiotic recombination 11
RS009125	GO:0005975	carbohydrate metabolic process	BP	-0.499166098	-0.260741424	-0.718448365	2.806103389	3.328550691	0.343688647	0.524656343	PREDICTED: isoprenoid synthase domain-containing protein
RS009458	GO:0005975	carbohydrate metabolic process	BP	0.629613425	0.709306495	0.406933765	2.907642854	3.270452692	0.351776297	0.532292525	PREDICTED: phosphoglucomutase
RS002950	GO:0005975	carbohydrate metabolic process	BP	0.051940014	0.251144097	-0.140848098	6.508547037	3.237096775	0.356493993	0.537429417	PREDICTED: GPI mannosyltransferase 2
RS012442	GO:0005975	carbohydrate metabolic process	BP	0.514798175	0.097974639	0.213608993	4.776141243	3.204017959	0.361226506	0.542677801	PREDICTED: beta-galactosidase-1-like protein 2
RS011421	GO:0005975	carbohydrate metabolic process	BP	-0.503303964	-0.41943895	-0.020163436	3.152894245	3.177804188	0.365015293	0.546351629	PREDICTED: beta-1,4-N-acetylgalactosaminyltransferase bre-4
RS011891	GO:0005975	carbohydrate metabolic process	BP	0.166598582	-0.184444429	-0.411265441	3.861165815	3.078602873	0.379664218	0.559821156	PREDICTED: alpha-L-iduronidase
RS002223	GO:0005975	carbohydrate metabolic process	BP	-0.661288042	-0.746174898	-0.52242263	5.161861513	2.989987319	0.393171512	0.571350951	PREDICTED: chitinase-3-like protein 1
RS004857	GO:0005975	carbohydrate metabolic process	BP	0.001882822	-0.133955239	-0.647468617	2.077593454	2.936643799	0.40149696	0.578409142	PREDICTED: alpha-1,3-mannosyl-glycoprotein 2-beta-N-acetylglucosaminyltransferase
RS012102	GO:0005975	carbohydrate metabolic process	BP	-0.031795079	-0.166508744	0.201878328	6.886076703	2.892771633	0.408454851	0.584588334	PREDICTED: hexosaminidase D-like
RS002983	GO:0005975	carbohydrate metabolic process	BP	0.377379875	0.206577437	0.23042339	5.703954918	2.872033248	0.41177882	0.587908679	PREDICTED: mannose-6-phosphate isomerase
RS011230	GO:0005975	carbohydrate metabolic process	BP	0.34834624	0.215690427	0.018096064	4.617698053	2.862743697	0.413275065	0.589307294	PREDICTED: glycerol kinase-like
RS006685	GO:0005975	carbohydrate metabolic process	BP	0.007325471	-0.293223436	-0.432311597	3.533055228	2.809344423	0.421963968	0.597572721	PREDICTED: GDP-fucose protein O-fucosyltransferase 1
RS001701	GO:0005975	carbohydrate metabolic process	BP	-0.428537809	-0.354853252	-0.359559576	8.130709933	2.794774192	0.424360901	0.599618668	PREDICTED: ornithine decarboxylase 1-like
RS003842	GO:0005975	carbohydrate metabolic process	BP	-0.202795939	-1.198204704	-0.607414579	0.64530997	2.785488426	0.425894355	0.600838644	PREDICTED: hematopoietically-expressed homeobox protein hhex
RS003389	GO:0005975	carbohydrate metabolic process	BP	-0.057326789	-0.435286332	-0.210979187	6.207606112	2.780907596	0.426652518	0.601506976	PREDICTED: alpha-mannosidase 2
RS009556	GO:0005975	carbohydrate metabolic process	BP	0.087611418	0.264903488	0.451450402	3.833800657	2.712576642	0.438094255	0.611571875	PREDICTED: chitinase-3-like protein 2
RS011076	GO:0005975	carbohydrate metabolic process	BP	0.672813702	0.671977204	0.36668554	1.736169215	2.564903592	0.463675648	0.635649811	CG1504
RS012264	GO:0005975	carbohydrate metabolic process	BP	0.056631713	0.03848964	-0.253035463	6.466737478	2.48425876	0.478142596	0.647358402	PREDICTED: galactosylgalactosylxylosylprotein 3-beta-glucuronosyltransferase I
RS011473	GO:0005975	carbohydrate metabolic process	BP	-0.143652931	-0.426507896	-0.271073598	3.841726772	2.460355232	0.482498404	0.65141528	PREDICTED: probable galactose-1-phosphate uridylyltransferase
RS002497	GO:0005975	carbohydrate metabolic process	BP	-0.212234533	-0.495353126	-0.227222594	7.913801195	2.430343888	0.488011114	0.655434784	PREDICTED: phosphoglycerate kinase
RS014064	GO:0005975	carbohydrate metabolic process	BP	0.4036205	0.091122921	0.315423844	4.209003705	2.392802345	0.49497587	0.66126298	PREDICTED: dolichyldiphosphatase 1-like
RS012274	GO:0005975	carbohydrate metabolic process	BP	0.250868348	0.130831382	-0.091269758	5.86768545	2.371175161	0.499022911	0.66490632	PREDICTED: probable GDP-L-fucose synthase
RS007200	GO:0005975	carbohydrate metabolic process	BP	0.073307321	-0.029382786	-0.363746794	6.43576106	2.351240248	0.502775765	0.667312245	PREDICTED: mannosyl-oligosaccharide glucosidase GCS1
RS010265	GO:0005975	carbohydrate metabolic process	BP	-0.38152137	-0.419775112	-0.428316336	3.45744259	2.30644957	0.511286467	0.674467556	PREDICTED: lysosomal alpha-glucosidase-like
RS004896	GO:0005975	carbohydrate metabolic process	BP	0.258725126	0.153243084	0.316020819	6.888961678	2.281927063	0.515992041	0.678432466	PREDICTED: ER degradation-enhancing alpha-mannosidase-like protein 3
RS015050	GO:0005975	carbohydrate metabolic process	BP	-0.017041537	-0.193053418	-0.353464811	4.694919112	2.25775821	0.520661577	0.682332144	PREDICTED: putative GPI-anchor transamidase
RS005067	GO:0005975	carbohydrate metabolic process	BP	-0.011472266	0.28377031	0.501312581	2.737680145	2.244794511	0.523179224	0.683779775	PREDICTED: N-acylneuraminate cytidylyltransferase
RS007162	GO:0005975	carbohydrate metabolic process	BP	0.352804678	-0.213394026	-0.027296829	4.583000343	2.241629936	0.523795185	0.684160606	PREDICTED: dolichyl-diphosphooligosaccharide--protein glycosyltransferase subunit STT3A
RS004440	GO:0005975	carbohydrate metabolic process	BP	-0.006850436	0.369271357	0.390768265	3.839992898	2.235571801	0.524975863	0.685283913	PREDICTED: protein prickle-like
RS003240	GO:0005975	carbohydrate metabolic process	BP	0.255011601	0.2449423	0.024189273	7.260073137	2.220353778	0.527950447	0.687888957	PREDICTED: ER degradation-enhancing alpha-mannosidase-like protein 2
RS010519	GO:0005975	carbohydrate metabolic process	BP	-0.037930597	-0.360925628	0.047413361	8.267918948	2.205787473	0.530809319	0.690248912	PREDICTED: phosphoglycerate mutase 1
RS012266	GO:0005975	carbohydrate metabolic process	BP	-0.472399722	-0.363524526	-0.351573067	4.356897101	2.197254643	0.532489323	0.691424331	PREDICTED: serine/threonine-protein phosphatase PP1-beta catalytic subunit
RS009176	GO:0005975	carbohydrate metabolic process	BP	-0.024380128	-0.369769368	-0.464884184	3.510259413	2.190263286	0.533868746	0.692292317	PREDICTED: phosphoglucomutase-2
RS009965	GO:0005975	carbohydrate metabolic process	BP	-0.364341407	-0.227420968	0.203520868	10.31015082	2.096719825	0.55257665	0.706584459	PREDICTED: malate dehydrogenase, mitochondrial
RS007966	GO:0005975	carbohydrate metabolic process	BP	0.327067464	0.065939638	0.241751561	4.896596253	2.070212177	0.557962509	0.711088832	Tankyrase-1
RS007232	GO:0005975	carbohydrate metabolic process	BP	0.05852288	0.30721745	0.280289014	7.191739753	2.01304291	0.569704018	0.720350873	PREDICTED: casein kinase I isoform alpha
RS013201	GO:0005975	carbohydrate metabolic process	BP	0.017552264	-0.289315883	-0.202265752	7.111279959	1.9075777	0.591809073	0.737725771	PREDICTED: probable phosphorylase b kinase regulatory subunit alpha
RS011544	GO:0005975	carbohydrate metabolic process	BP	0.377175568	-0.064832165	0.03084746	3.098145541	1.902260953	0.592938469	0.738768014	PREDICTED: endoplasmic reticulum mannosyl-oligosaccharide 1,2-alpha-mannosidase
RS009535	GO:0005975	carbohydrate metabolic process	BP	-0.371380403	-0.243526213	-0.109065039	6.875240596	1.879978368	0.597687259	0.742847463	PREDICTED: delta-1-pyrroline-5-carboxylate dehydrogenase, mitochondrial
RS002439	GO:0005975	carbohydrate metabolic process	BP	0.322813085	0.111104145	-0.161839796	6.005037147	1.846999235	0.604761168	0.748757585	PREDICTED: UDP-glucose 4-epimerase-like
RS012344	GO:0005975	carbohydrate metabolic process	BP	0.306723742	0.137385989	0.006060781	5.518269466	1.835403372	0.607261236	0.750524829	PREDICTED: heparanase-like
RS009803	GO:0005975	carbohydrate metabolic process	BP	0.213911252	0.218935271	0.288323068	5.18116662	1.76239907	0.623151298	0.762368939	PREDICTED: stromal cell-derived factor 2
RS015538	GO:0005975	carbohydrate metabolic process	BP	0.225640179	-0.10699019	0.104179018	4.908035582	1.70244913	0.636389435	0.77242565	PREDICTED: chitobiosyldiphosphodolichol beta-mannosyltransferase
RS015064	GO:0005975	carbohydrate metabolic process	BP	-0.234416859	-0.485764927	-0.405888112	6.699048012	1.631993179	0.652157533	0.782777605	PREDICTED: beta-hexosaminidase subunit beta-like
RS013111	GO:0005975	carbohydrate metabolic process	BP	0.243734991	-0.003665678	0.068325846	6.590986784	1.610092577	0.657103567	0.786351418	PREDICTED: ras-related protein Rab-10
RS001046	GO:0005975	carbohydrate metabolic process	BP	-0.274727443	-0.262613671	-0.086781178	6.540789003	1.480175107	0.686853406	0.806933497	PREDICTED: probable phosphorylase b kinase regulatory subunit beta
RS004233	GO:0005975	carbohydrate metabolic process	BP	0.217324559	-0.004738175	-0.010447668	5.688299795	1.395694182	0.706544349	0.822006423	PREDICTED: chitinase domain-containing protein 1
RS014710	GO:0005975	carbohydrate metabolic process	BP	0.13791826	0.280437844	0.246818414	7.961653377	1.394222752	0.706889507	0.822234092	PREDICTED: coatomer subunit alpha
RS006783	GO:0005975	carbohydrate metabolic process	BP	-0.192050553	-0.446936664	-0.038289298	3.261063321	1.349372965	0.717443685	0.830467442	PREDICTED: hexosaminidase D-like
RS009145	GO:0005975	carbohydrate metabolic process	BP	-0.08188239	-0.082633407	0.146015069	6.662956201	1.344623243	0.718565095	0.831181996	PREDICTED: ADP-dependent glucokinase
RS004098	GO:0005975	carbohydrate metabolic process	BP	0.338570455	-0.368556567	0.030527269	0.982012773	1.323061147	0.723664399	0.834587812	PREDICTED: probable chitinase 2
RS005230	GO:0005975	carbohydrate metabolic process	BP	-0.262472401	-0.091272997	-0.047101054	6.67466439	1.274786012	0.735128916	0.842540448	PREDICTED: probable pyruvate dehydrogenase E1 component subunit alpha, mitochondrial
RS014581	GO:0005975	carbohydrate metabolic process	BP	-0.061420057	0.165382081	0.066311593	5.341596955	1.209505455	0.750725425	0.852690838	PREDICTED: beta-1,4-galactosyltransferase 7
RS004127	GO:0005975	carbohydrate metabolic process	BP	0.115690403	0.165727139	0.345872341	7.087804839	1.185675978	0.756441814	0.857147907	PREDICTED: polypeptide N-acetylgalactosaminyltransferase 5
RS015552	GO:0005975	carbohydrate metabolic process	BP	0.031743656	-0.145572687	-0.190149689	5.50444468	1.149173771	0.765218526	0.862694666	PREDICTED: glucosylceramidase-like

RS010811	GO:0005975	carbohydrate metabolic process	BP	-0.327603428	-0.266768771	-0.322247022	2.816350152	1.145482098	0.766107395	0.863542158	PREDICTED: beta-galactoside alpha-2,6-sialyltransferase 2
RS011165	GO:0005975	carbohydrate metabolic process	BP	-0.210244823	0.020310001	-0.022065044	6.798726885	1.120227957	0.772193354	0.867529269	PREDICTED: polypeptide N-acetylgalactosaminyltransferase 35A
RS005984	GO:0005975	carbohydrate metabolic process	BP	0.152379143	-0.112920669	-0.068279543	4.98672439	1.116242857	0.773154511	0.868067033	PREDICTED: beta-1,3-galactosyltransferase brn
RS002699	GO:0005975	carbohydrate metabolic process	BP	0.18641679	-0.113157012	0.19259875	3.645600491	1.098441167	0.777450405	0.870639375	PREDICTED: galactosylgalactosylxylosylprotein 3-beta-glucuronosyltransferase S
RS004876	GO:0005975	carbohydrate metabolic process	BP	0.254514777	-0.013585511	0.016602412	4.233428366	1.08122198	0.781608997	0.873820125	PREDICTED: 3-hydroxyisobutyrate dehydrogenase, mitochondrial
RS009862	GO:0005975	carbohydrate metabolic process	BP	-0.060771506	0.139385422	0.123895862	5.976114235	1.046660744	0.789963425	0.878882504	PREDICTED: poly(ADP-ribose) glycohydrolase-like
RS007702	GO:0005975	carbohydrate metabolic process	BP	-0.207976473	0.196473721	0.085101443	3.379751314	1.033521878	0.793141391	0.880709569	PREDICTED: ER degradation-enhancing alpha-mannosidase-like protein 1
RS009476	GO:0005975	carbohydrate metabolic process	BP	0.070675817	-0.142437313	0.031156422	6.23550366	1.029018525	0.794230809	0.881436624	PREDICTED: N-acetylglucosamine-1-phosphotransferase subunits alpha/beta
RS007185	GO:0005975	carbohydrate metabolic process	BP	-0.40805096	-0.102942074	-0.188514429	3.229175551	1.01478824	0.7976737	0.883615901	PREDICTED: carbohydrate sulfotransferase 11
RS014637	GO:0005975	carbohydrate metabolic process	BP	0.066840247	0.600909401	0.396436471	2.051509931	1.008937941	0.799089251	0.884723519	PREDICTED: ER degradation-enhancing alpha-mannosidase-like protein 1
RS002770	GO:0005975	carbohydrate metabolic process	BP	-0.119360703	-0.359384597	-0.31109241	10.31331347	0.993680302	0.802781134	0.886207234	glyceraldehyde-3-phosphate dehydrogenase
RS002932	GO:0005975	carbohydrate metabolic process	BP	0.055970556	-0.003033501	0.220016606	7.070781008	0.921709314	0.820185942	0.897430213	PREDICTED: aldehyde dehydrogenase, mitochondrial
RS000150	GO:0005975	carbohydrate metabolic process	BP	0.215426876	0.132994308	0.193610407	5.217037063	0.918173637	0.821040024	0.898081827	PREDICTED: glucose-6-phosphatase 2
RS013172	GO:0005975	carbohydrate metabolic process	BP	0.064707769	0.050177661	0.256276698	4.54520608	0.870549691	0.832528393	0.905630734	PREDICTED: N-acetyl-D-glucosamine kinase
RS100018	GO:0005975	carbohydrate metabolic process	BP	0.098915616	-0.207748005	0.190436048	2.406369291	0.828987935	0.842521671	0.912149643	gram negative bacteria-binding protein 2
RS009311	GO:0005975	carbohydrate metabolic process	BP	0.17556667	0.058214485	-0.24291434	2.192551785	0.8273653	0.842911036	0.912465324	PREDICTED: bifunctional ATP-dependent dihydroxyacetone kinase/FAD-AMP lyase (cyclizing)-like
RS008464	GO:0005975	carbohydrate metabolic process	BP	-0.07818374	0.007615105	0.128259633	5.44261975	0.825685956	0.843313938	0.912744563	PREDICTED: polypeptide N-acetylgalactosaminyltransferase 1
RS009433	GO:0005975	carbohydrate metabolic process	BP	0.385144915	-0.153581998	0.101631697	1.554735334	0.779990692	0.854246764	0.919991769	gag-pol polyprotein
RS002959	GO:0005975	carbohydrate metabolic process	BP	-0.310320167	-0.096091889	-0.027692262	3.017853716	0.747218875	0.862045317	0.924989241	PREDICTED: 2-oxoisovalerate dehydrogenase subunit beta, mitochondrial
RS010425	GO:0005975	carbohydrate metabolic process	BP	0.170883627	0.369811289	0.502252155	0.509891817	0.615691468	0.892830962	0.942219758	PREDICTED: glycerophosphodiester phosphodiesterase 1
RS005270	GO:0005975	carbohydrate metabolic process	BP	-0.004544488	-0.216158524	-0.097291995	3.745309896	0.56443291	0.904524136	0.94929808	PREDICTED: xylosyltransferase oxt
RS012409	GO:0005975	carbohydrate metabolic process	BP	0.166649262	0.125364989	0.024833063	4.907461326	0.502930148	0.918247201	0.957419827	PREDICTED: UDP-glucose 4-epimerase
RS014134	GO:0005975	carbohydrate metabolic process	BP	0.202412564	0.022912512	0.074608072	10.02891467	0.337859024	0.952757814	0.975354966	PREDICTED: calmodulin
RS013735	GO:0005975	carbohydrate metabolic process	BP	0.104677793	0.022858359	-0.09563073	2.80861291	0.320642672	0.956100531	0.976888698	PREDICTED: alpha-(1,3)-fucosyltransferase 10
RS003843	GO:0005975	carbohydrate metabolic process	BP	-0.149866899	-0.119567997	-0.030363665	3.499148319	0.310983253	0.957949764	0.977604853	Homeobox protein ceh-9
RS004720	GO:0005975	carbohydrate metabolic process	BP	-0.146782098	-0.172357517	-0.158017647	3.893542108	0.308665541	0.958390572	0.977721415	PREDICTED: protein O-linked-mannose beta-1,2-N-acetylglucosaminyltransferase 1
RS012220	GO:0005975	carbohydrate metabolic process	BP	-0.029335723	0.050689391	0.128078453	3.427049826	0.305819403	0.958930318	0.977774901	PREDICTED: galactoside 2-alpha-L-fucosyltransferase 3-like
RS002156	GO:0005975	carbohydrate metabolic process	BP	-0.096166135	-0.089844853	-0.038945283	6.136484784	0.256806415	0.967936189	0.982956509	PREDICTED: hexosaminidase D
RS007819	GO:0005975	carbohydrate metabolic process	BP	-0.010489001	0.053195155	-0.069169533	4.283628557	0.169132117	0.982411503	0.991117631	PREDICTED: N-acetylgalactosaminyltransferase 6-like
RS007652	GO:0022891	substrate-specific transmembrane transporter activity	MF	0.585883623	0.528505008	4.851177054	4.584889963	291.2117195	7.93792E-63	8.92591E-61	PREDICTED: solute carrier family 26 member 10-like
RS003129	GO:0022891	substrate-specific transmembrane transporter activity	MF	-1.935857211	0.013218622	3.698610157	4.556804627	145.8197805	2.10104E-31	9.2418E-30	PREDICTED: organic cation transporter protein
RS004606	GO:0022891	substrate-specific transmembrane transporter activity	MF	-0.152775887	3.199032258	1.582974114	7.379011967	144.4003621	4.25168E-31	1.83376E-29	PREDICTED: peptide transporter family 1
RS004125	GO:0022891	substrate-specific transmembrane transporter activity	MF	1.407227463	2.012417871	-0.423642507	5.906215214	131.7530272	2.26607E-28	8.8083E-27	PREDICTED: ABC transporter G family member 9
RS003228	GO:0022891	substrate-specific transmembrane transporter activity	MF	-0.561103954	2.12048168	1.83683218	5.406987214	114.6440514	1.09829E-24	3.71824E-23	PREDICTED: b(0,+)-type amino acid transporter 1-like
RS013974	GO:0022891	substrate-specific transmembrane transporter activity	MF	1.308057058	2.315023189	0.876327879	6.127768048	111.7248777	4.66769E-24	1.53887E-22	PREDICTED: monocarboxylate transporter 10
RS000720	GO:0022891	substrate-specific transmembrane transporter activity	MF	-1.414180497	1.484024302	-1.819002501	5.701350612	100.1058089	1.47484E-21	4.12758E-20	aquaporin
RS012045	GO:0022891	substrate-specific transmembrane transporter activity	MF	-2.417547613	-3.846942398	-2.948472949	3.138267013	95.12499238	1.73561E-20	4.58246E-19	PREDICTED: sodium-independent sulfate anion transporter
RS002200	GO:0022891	substrate-specific transmembrane transporter activity	MF	-2.534854462	-2.965618885	0.446701011	4.187537984	94.94596227	1.89639E-20	4.97566E-19	PREDICTED: ATP-binding cassette sub-family G member 4
RS009300	GO:0022891	substrate-specific transmembrane transporter activity	MF	-0.334428722	2.777656488	1.996146097	5.84981916	85.14092159	2.42051E-18	5.44355E-17	PREDICTED: organic cation transporter protein-like
RS014732	GO:0022891	substrate-specific transmembrane transporter activity	MF	-2.171879817	-1.24728548	0.09932889	5.367265273	82.18961662	1.04062E-17	2.21004E-16	PREDICTED: solute carrier organic anion transporter family member 2A1
RS015390	GO:0022891	substrate-specific transmembrane transporter activity	MF	-1.260588417	-0.88728058	1.174441311	5.643186003	81.54774944	1.42892E-17	3.01438E-16	PREDICTED: sodium-independent sulfate anion transporter
RS014579	GO:0022891	substrate-specific transmembrane transporter activity	MF	-1.833927302	-1.637670215	-2.03241566	4.946947748	80.73878787	2.13088E-17	4.42843E-16	PREDICTED: transient receptor potential cation channel protein painless
RS011973	GO:0022891	substrate-specific transmembrane transporter activity	MF	-0.861945869	1.222753935	-0.568825813	4.359430432	80.33905671	2.59602E-17	5.34219E-16	PREDICTED: zinc transporter 1
RS004424	GO:0022891	substrate-specific transmembrane transporter activity	MF	-0.154114067	2.332271731	0.36051487	5.646491566	77.24068683	1.19889E-16	2.38151E-15	PREDICTED: TWiK family of potassium channels protein 7
RS002366	GO:0022891	substrate-specific transmembrane transporter activity	MF	-2.013469227	2.285528889	0.940362283	2.009763796	76.37983445	1.83374E-16	3.57494E-15	PREDICTED: sodium-dependent serotonin transporter
RS011988	GO:0022891	substrate-specific transmembrane transporter activity	MF	-1.045428446	0.08149937	1.776943069	7.507925055	72.44172426	1.28023E-15	2.31656E-14	PREDICTED: aquaporin-12
RS006315	GO:0022891	substrate-specific transmembrane transporter activity	MF	-2.445857294	-4.975199992	0.147442902	3.069628072	71.7469443	1.80352E-15	3.24017E-14	PREDICTED: bumetanide-sensitive sodium-(potassium)-chloride cotransporter
RS008278	GO:0022891	substrate-specific transmembrane transporter activity	MF	0.642268679	2.750452439	1.283258456	3.961355993	71.14168969	2.43087E-15	4.34246E-14	PREDICTED: potassium voltage-gated channel protein Shaw-like
RS006271	GO:0022891	substrate-specific transmembrane transporter activity	MF	1.985922002	1.711587011	-1.594562947	3.353974541	68.37067995	9.52991E-15	1.62849E-13	PREDICTED: organic cation transporter 1
RS008443	GO:0022891	substrate-specific transmembrane transporter activity	MF	-1.979883882	-8.057075206	-1.206380805	2.046366836	67.84965365	1.23201E-14	2.07155E-13	PREDICTED: facilitated trehalose transporter Tret1-2 homolog
RS001006	GO:0022891	substrate-specific transmembrane transporter activity	MF	-1.308055843	0.748595123	2.011362686	7.151837524	64.31436805	7.03086E-14	1.12512E-12	PREDICTED: sodium-dependent nutrient amino acid transporter 1-like
RS007831	GO:0022891	substrate-specific transmembrane transporter activity	MF	-0.285689079	2.23184291	-0.54998832	3.347851609	60.46667692	4.67238E-13	7.00523E-12	PREDICTED: sodium- and chloride-dependent GABA transporter ine-like
RS010878	GO:0022891	substrate-specific transmembrane transporter activity	MF	-0.202147251	0.219397642	1.259334773	5.8954575	54.42606082	9.10217E-12	1.23527E-10	PREDICTED: band 3 anion transport protein
RS008617	GO:0022891	substrate-specific transmembrane transporter activity	MF	-1.758654905	-1.933395291	0.119722157	4.240860888	52.46640405	2.38231E-11	3.09308E-10	PREDICTED: proton-coupled amino acid transporter 1
RS006021	GO:0022891	substrate-specific transmembrane transporter activity	MF	-1.978389488	-0.987147201	0.23457041	5.767022167	52.1310105	2.80858E-11	3.63527E-10	PREDICTED: cationic amino acid transporter 3
RS000134	GO:0022891	substrate-specific transmembrane transporter activity	MF	-2.137136524	0.447963208	0.262936854	3.43282157	50.22811762	7.14362E-11	8.98769E-10	PREDICTED: putative ammonium transporter 1
RS012141	GO:0022891	substrate-specific transmembrane transporter activity	MF	-0.795199967	0.272005658	1.157168	4.936082578	49.75842476	8.99389E-11	1.12594E-09	PREDICTED: UNC93-like protein MFSD11
RS008254	GO:0022891	substrate-specific transmembrane transporter activity	MF	-1.84400551	0.769806662	1.172103168	3.667348911	48.7792472	1.45353E-10	1.78592E-09	PREDICTED: facilitated trehalose transporter Tret1-like
RS009380	GO:0022891	substrate-specific transmembrane transporter activity	MF	1.181487238	0.230316163	-1.011984835	4.090504935	45.23692145	8.23976E-10	9.50289E-09	PREDICTED: b(0,+)-type amino acid transporter 1-like
RS006648	GO:0022891	substrate-specific transmembrane transporter activity	MF	-1.472287059	-1.627468546	0.001448714	3.845832603	45.04057983	9.07077E-10	1.03664E-08	PREDICTED: voltage-dependent L-type calcium channel subunit beta-2
RS006270	GO:0022891	substrate-specific transmembrane transporter activity	MF	-0.625556145	-0.022131593	2.665286326	2.775447041	44.95816183	9.44409E-10	1.07832E-08	PREDICTED: organic cation transporter 1
RS011778	GO:0022891	substrate-specific transmembrane transporter activity	MF	-1.482690204	-0.163981472	-1.627972092	5.097898358	44.03120475	1.48632E-09	1.66834E-08	PREDICTED: facilitated trehalose transporter Tret1-2 homolog
RS005640	GO:0022891	substrate-specific transmembrane transporter activity	MF	0.969003673	1.718434974	-0.876644168	4.401662752	43.66119041	1.78117E-09	1.9764E-08	PREDICTED: glycine receptor subunit alpha-4
RS009578	GO:0022891	substrate-specific transmembrane transporter activity	MF	-2.77838182	-2.247321578	-2.454512864	4.019688809	42.39569844	3.30671E-09	3.59937E-08	PREDICTED: solute carrier family 28 member 3
RS001007	GO:0022891	substrate-specific transmembrane transporter activity	MF	-0.057453873	2.550863666	4.593751253	0.34449975	41.36132174	5.4813E-09	5.84023E-08	PREDICTED: sodium-dependent nutrient amino acid transporter 1-like
RS010452	GO:0022891	substrate-specific transmembrane transporter activity	MF	-1.264493454	-3.411377807	-0.656775569	3.802175748	40.96006363	6.66799E-09	7.05094E-08	PREDICTED: transient receptor potential cation channel protein painless
RS014047	GO:0022891	substrate-specific transmembrane transporter activity	MF	-0.592092988	1.748032789	5.355436168	1.817327481	40.93864928	6.73809E-09	7.11311E-08	PREDICTED: zinc transporter ZIP1-like
RS005851	GO:0022891	substrate-specific transmembrane transporter activity	MF	0.526397294	1.41413322	1.673681755	7.829535198	40.74316203	7.413E-09	7.77347E-08	PREDICTED: proton-coupled amino acid transporter 4
RS012191	GO:0022891	substrate-specific transmembrane transporter activity	MF	-0.827958312	0.960594434	-0.015691014	7.391207833	39.39645508	1.43044E-08	1.45634E-07	PREDICTED: V-type proton ATPase 116 kDa subunit a
RS009711	GO:0022891	substrate-specific transmembrane transporter activity	MF	0.862276886	1.272132135	1.421293308	6.056371062	36.7986326	5.07562E-08	4.85732E-07	PREDICTED: tropinone reductase 2-like
RS013235	GO:0022891	substrate-specific transmembrane transporter activity	MF	-2.068035818	-1.64402774	1.426351822	0.894091033	34.98409736	1.22771E-07	1.1286E-06	PREDICTED: glutamate receptor 1-like
RS012786	GO:0022891	substrate-specific transmembrane transporter activity	MF	-1.725772589	-2.922030511	-0.315113572	3.189291251	34.971992	1.23496E-07	1.13444E-06	PREDICTED: facilitated trehalose transporter Tret1-2 homolog
RS004248	GO:0022891	substrate-specific transmembrane transporter activity	MF	-2.357800174	-1.138542703	0.799750155	3.47254249	34.60029849	1.47968E-07	1.34356E-06	PREDICTED: facilitated trehalose transporter Tret1-like
RS009116	GO:0022891	substrate-specific transmembrane transporter activity	MF	-2.774708917	-1.965598127	1.306984186	1.044379091	34.54483465	1.52014E-07	1.3783E-06	PREDICTED: urea transporter 1-like
RS000365	GO:0022891	substrate-specific transmembrane transporter activity	MF	-2.043121889	-2.26896537	0.189661525	11.00673959	34.00204383	1.97922E-07	1.77664E-06	PREDICTED: calcium-transporting ATPase sarcoplasmic/endoplasmic reticulum type
RS007032	GO:0022891	substrate-specific transmembrane transporter activity	MF	-1.972036202	-0.832866298	0.156731051	5.195017285	33.86656984	2.11394E-07	1.89487E-06	PREDICTED: ammonium transporter Rh type A
RS001722	GO:0022891	substrate-specific transmembrane transporter activity	MF	-0.334283753	-2.198611554	-0.278419111	3.485639997	32.69185314	3.74056E-07	3.27598E-06	PREDICTED: solute carrier family 41 member 1-like
RS007693	GO:0022891	substrate-specific transmembrane transporter activity	MF	-0.242378739	-0.621851837	1.493665703	5.076496585	32.00947491	5.20946E-07	4.51534E-06	PREDICTED: zinc transporter ZIP14
RS006814	GO:0022891	substrate-specific transmembrane transporter activity	MF	-0.514796382	1.014347124	0.975336891	4.041760153	30.97725487	8.59474E-07	7.23543E-06	PREDICTED: large neutral amino acids transporter small subunit 2
RS006320	GO:0022891	substrate-specific transmembrane transporter activity	MF	1.668458777	1.549661536	0.932473469	6.183206129	30.51118393	1.07731E-06	8.96741E-06	PREDICTED: bumetanide-sensitive sodium-(potassium)-chloride cotransporter
RS000193	GO:0022891	substrate-specific transmembrane transporter activity	MF	-0.612906575	0.552707825	0.864925878	6.395375097	30.19397061	1.25629E-06	1.03772E-05	PREDICTED: b(0,+)-type amino acid transporter 1
RS000996	GO:0022891	substrate-specific transmembrane transporter activity	MF	-1.508231266	-0.156640833	0.876498273	2.831732428	29.09435446	2.13949E-06	1.70644E-05	PREDICTED: zinc transporter ZIP3-like
RS005491	GO:0022891	substrate-specific transmembrane transporter activity	MF	-0.937465516	-1.630388798	-0.209059704	4.107611972	28.88192352	2.37108E-06	1.88281E-05	PREDICTED: voltage-gated potassium channel subunit beta-2
RS009579	GO:0022891	substrate-specific transmembrane transporter activity	MF	-0.361377028	0.937501921	0.004604885	5.160969196	28.85056945	2.40732E-06	1.91039E-05	PREDICTED: solute carrier family 28 member 3-like
RS011850	GO:0022891	substrate-specific transmembrane transporter activity	MF	-0.767278606	-0.313949384	0.713484854	8.083358391	28.67013092	2.62688E-06	2.07027E-05	PREDICTED: trimeric intracellular cation channel type B
RS008885	GO:0022891	substrate-specific transmembrane transporter activity	MF	-1.592550754	-2.358375405	-0.949039702	4.983900477	28.41800515	2.96751E-06	2.32998E-05	PREDICTED: facilitated trehalose transporter Tret1-2 homolog
RS010090	GO:0022891	substrate-specific transmembrane transporter activity	MF	0.323234855	1.546465547	-0.132352818	2.938456182	28.16134763	3.35955E-06	2.60531E-05	PREDICTED: cyclic nucleotide-gated cation channel subunit A-like
RS008631	GO:0022891	substrate-specific transmembrane transporter activity	MF	-0.45372484	0.018443887	2.172529191	1.173123173	28.08258513	3.48992E-06	2.69976E-05	PREDICTED: glutamate receptor ionotropic, delta-1-like
RS008887	GO:0022891	substrate-specific transmembrane transporter activity	MF	-0.760329305	-4.339267866	-1.430676324	3.320036492	27.63642804	4.32958E-06	3.3078E-05	PREDICTED: facilitated trehalose transporter Tret1-like
RS006269	GO:0022891	substrate-specific transmembrane transporter activity	MF	-1.78180142	-1.191554952	-0.830372469	4.550094908	27.39894348	4.85584E-06	3.68178E-05	PREDICTED: organic cation transporter 1
RS012590	GO:0022891	substrate-specific transmembrane transporter activity	MF	-0.22021637	0.861437679	0.641734274	6.761709937	27.36974453	4.9248E-06	3.72958E-05	PREDICTED: high affinity copper uptake protein 1
RS012741	GO:0022891	substrate-specific transmembrane transporter activity	MF	-1.060191661	0.585439948	0.026171952	4.628484614	26.6687965	6.90781E-06	5.10845E-05	PREDICTED: organic cation transporter protein
RS000895	GO:0022891	substrate-specific transmembrane transporter activity	MF	1.014348253	1.385977981	0.68384576	7.930053973	26.65746374	6.94569E-06	5.13345E-05	PREDICTED: translocon-associated protein subunit beta
RS000719	GO:0022891	substrate-specific transmembrane transporter activity	MF	-0.394780773	-1.247672358	-1.502851389	6.140116665	25.60119706	1.15586E-05	8.29929E-05	PREDICTED: aquaporin AQPAn.G
RS013310	GO:0022891	substrate-specific transmembrane transporter activity	MF	-1.594173173	0.30942411	0.502900611	3.784418211	25.32704004	1.31907E-05	9.38549E-05	PREDICTED: sodium- and chloride-dependent transporter XTRP3
RS003638	GO:0022891	substrate-specific transmembrane transporter activity	MF	-0.69625377	-0.738048152	0.351114728	5.7591544	24.53074045	1.93528E-05	0.00013355	PREDICTED: ATP-binding cassette sub-family G member 4
RS003139	GO:0022891	substrate-specific transmembrane transporter activity	MF	-0.462942241	-0.484276182	0.670801183	4.994154039	24.0689115	2.4166E-05	0.000164334	PREDICTED: zinc transporter 1
RS006319	GO:0022891	substrate-specific transmembrane transporter activity	MF	-1.264071762	-1.378688832	-0.296664377	4.499770473	23.99784591	2.50059E-05	0.000169587	PREDICTED: bumetanide-sensitive sodium-(potassium)-chloride cotransporter
RS008934	GO:0022891	substrate-specific transmembrane transporter activity	MF	-2.249974024	-1.610242152	-0.986553809	2.596021099	23.90344612	2.61667E-05	0.000177078	ionotropic receptor 25a
RS010443	GO:0022891	substrate-specific transmembrane transporter activity	MF	-2.227753564	-2.234532899	-0.56368196	2.617137088	23.82549146	2.71656E-05	0.000183248	PREDICTED: pickpocket protein 28-like
RS014152	GO:0022891	substrate-specific transmembrane transporter activity	MF	-1.430499389	-1.354570713	-0.671668718	4.505777329	23.40073256	3.33153E-05	0.000221644	PREDICTED: potassium channel subfamily K member 1-like
RS012504	GO:0022891	substrate-specific transmembrane transporter activity	MF	1.701019285	0.103598753	-0.952358557	2.60795793	23.14266371	3.77102E-05	0.000248475	PREDICTED: zinc transporter ZIP3
RS014340	GO:0022891	substrate-specific transmembrane transporter activity	MF	-0.92887959	-0.092715464	0.219765138	5.620832256	22.82606695	4.38983E-05	0.00028483	PREDICTED: ileal sodium/bile acid cotransporter-like
RS011507	GO:0022891	substrate-specific transmembrane transporter activity	MF	0.081672207	0.439418944	1.152509484	5.150989282	22.16598652	6.02438E-05	0.000382801	PREDICTED: sialin
RS005054	GO:0022891	substrate-specific transmembrane transporter activity	MF	-0.882564902	-1.772840386	-2.09465887	2.914764324	21.56658479	8.02786E-05	0.000498781	PREDICTED: ETS domain-containing protein Elk-3-like
RS012462	GO:0022891	substrate-specific transmembrane transporter activity	MF	-1.887439376	-1.068323927	-1.822700455	2.935725927	21.16526065	9.72757E-05	0.000593839	PREDICTED: synaptic vesicle glycoprotein 2B
RS012046	GO:0022891	substrate-specific transmembrane transporter activity	MF	-0.044612109	1.318321788	-0.182662099	3.873303092	21.0021542	0.000105168	0.000636465	PREDICTED: sodium-independent sulfate anion transporter-like
RS015154	GO:0022891	substrate-specific transmembrane transporter activity	MF	0.77118859	1.275597924	0.795023023	8.092063536	20.820501	0.00011471	0.000690232	PREDICTED: protein transport protein Sec61 subunit gamma
RS015395	GO:0022891	substrate-specific transmembrane transporter activity	MF	-1.227128143	-0.999149743	-0.38673491	5.789022217	20.66182653	0.000123747	0.000741733	PREDICTED: ryanodine receptor 44F
RS010854	GO:0022891	substrate-specific transmembrane transporter activity	MF	-0.464601417	0.421962906	0.417617417	6.7210824	20.51691341	0.000132619	0.000789321	PREDICTED: V-type proton ATPase subunit e 2-like
RS006273	GO:0022891	substrate-specific transmembrane transporter activity	MF	-0.841668922	-0.780206767	0.412687786	4.250127088	20.41178555	0.000139449	0.000826843	PREDICTED: organic cation transporter 1

RS014287	GO:0022891	substrate-specific transmembrane transporter activity	MF	-0.825809928	1.486619083	1.592270521	1.389357496	20.39684624	0.000140447	0.000831501	PREDICTED: sialin
RS007340	GO:0022891	substrate-specific transmembrane transporter activity	MF	1.72289566	2.228109689	-0.488523344	0.853368064	20.24210443	0.000151218	0.000888679	PREDICTED: mitochondrial sodium/hydrogen exchanger 9B2
RS004393	GO:0022891	substrate-specific transmembrane transporter activity	MF	-1.227790861	-2.264108903	-0.817189459	2.289519889	20.23673107	0.000151606	0.000890546	PREDICTED: putative tricarboxylate transport protein, mitochondrial
RS015388	GO:0022891	substrate-specific transmembrane transporter activity	MF	-2.602050655	-3.074067005	-0.457367858	0.547921075	20.16611512	0.000156804	0.000918937	PREDICTED: glutamate receptor ionotropic, delta-1
RS000135	GO:0022891	substrate-specific transmembrane transporter activity	MF	-0.631745801	-0.940404648	0.237026994	4.32458574	20.11793543	0.000160452	0.000937697	PREDICTED: putative ammonium transporter 1
RS006499	GO:0022891	substrate-specific transmembrane transporter activity	MF	-1.047637087	0.301924919	0.728056025	3.319116729	19.94751042	0.000174047	0.001009187	PREDICTED: transient receptor potential cation channel subfamily V member 5
RS015396	GO:0022891	substrate-specific transmembrane transporter activity	MF	-1.256182982	-1.010805264	-0.293857127	6.048626436	19.73517564	0.0001926	0.00110506	PREDICTED: ryanodine receptor 44F
RS008662	GO:0022891	substrate-specific transmembrane transporter activity	MF	-0.323694319	-0.887991823	-0.04413226	5.702043181	19.66407708	0.000199242	0.001138501	PREDICTED: transient receptor potential cation channel subfamily V member 5
RS005651	GO:0022891	substrate-specific transmembrane transporter activity	MF	-0.219646792	-1.283329628	-0.275521678	5.060327004	19.22158667	0.000246019	0.001380116	PREDICTED: transient-receptor-potential-like protein
RS012441	GO:0022891	substrate-specific transmembrane transporter activity	MF	-0.795629624	0.983276815	1.634723389	1.23435714	19.18509754	0.000250332	0.001401285	AGAP004474
RS006140	GO:0022891	substrate-specific transmembrane transporter activity	MF	0.033292599	1.273516036	1.00013418	2.720807394	19.17927372	0.000251027	0.00140446	PREDICTED: potassium channel subfamily K member 18
RS005838	GO:0022891	substrate-specific transmembrane transporter activity	MF	-1.272361428	-3.114064861	-3.237130599	0.429125999	19.0443607	0.000267685	0.001488399	PREDICTED: glutamate receptor U1-like
RS001336	GO:0022891	substrate-specific transmembrane transporter activity	MF	0.412169289	1.116247727	1.639275177	2.741362032	18.96980298	0.000277358	0.001536754	PREDICTED: glutamate receptor U1-like
RS007614	GO:0022891	substrate-specific transmembrane transporter activity	MF	0.699165628	-0.052782673	-0.817546641	5.472681929	18.94140065	0.000281133	0.001554478	PREDICTED: facilitated trehalose transporter Tret1-like
RS007694	GO:0022891	substrate-specific transmembrane transporter activity	MF	-1.402205119	-1.429007484	-0.154037672	3.235518975	18.72862507	0.000311092	0.001703432	PREDICTED: proton myo-inositol cotransporter-like
RS009272	GO:0022891	substrate-specific transmembrane transporter activity	MF	-0.152985688	-1.205701524	-0.53386679	4.590610282	18.71946093	0.000312451	0.001710132	PREDICTED: mitochondrial sodium/hydrogen exchanger 9B2
RS011525	GO:0022891	substrate-specific transmembrane transporter activity	MF	-0.806138244	0.427082827	0.274818707	7.810153084	18.69931455	0.000315461	0.001724354	PREDICTED: sodium/potassium-transporting ATPase subunit beta-2
RS009604	GO:0022891	substrate-specific transmembrane transporter activity	MF	-1.648345631	0.550771769	0.526514629	2.368500525	18.41833332	0.000360561	0.001945804	PREDICTED: sodium-dependent noradrenaline transporter
RS009304	GO:0022891	substrate-specific transmembrane transporter activity	MF	-2.056162023	-1.492683824	-1.148437663	1.440266038	18.0307206	0.000433479	0.002294761	PREDICTED: glutamate receptor ionotropic, delta-1-like
RS014410	GO:0022891	substrate-specific transmembrane transporter activity	MF	-0.538555444	-0.069436224	0.424307658	5.574699261	17.86202393	0.00046963	0.002466436	PREDICTED: sodium- and chloride-dependent neutral and basic amino acid transporter B(0+)
RS000837	GO:0022891	substrate-specific transmembrane transporter activity	MF	-1.260815808	-0.582381283	0.014148923	5.012164404	17.85146996	0.000471988	0.002476758	PREDICTED: excitatory amino acid transporter 1-like
RS007085	GO:0022891	substrate-specific transmembrane transporter activity	MF	-2.123520843	-1.442400071	-1.654436852	1.159522622	17.26753428	0.000622612	0.00317714	PREDICTED: cyclic nucleotide-gated cation channel alpha-3
RS000849	GO:0022891	substrate-specific transmembrane transporter activity	MF	-0.753175709	-1.571391853	-1.058453667	2.708513766	17.06922787	0.000683946	0.003464852	PREDICTED: monocarboxylate transporter 9-like
RS012432	GO:0022891	substrate-specific transmembrane transporter activity	MF	-1.318193047	-1.41640057	-1.093486391	3.821570882	17.02394884	0.000698772	0.003535692	PREDICTED: acetylcholine receptor subunit alpha-type acr-16-like
RS013332	GO:0022891	substrate-specific transmembrane transporter activity	MF	-1.915754341	-0.761067399	-0.092972296	1.96508851	16.98236489	0.000712669	0.003598779	PREDICTED: potassium channel subfamily T member 2
RS004044	GO:0022891	substrate-specific transmembrane transporter activity	MF	-0.216859326	0.637026253	1.004892576	2.871056733	16.9549321	0.000721987	0.003638536	PREDICTED: glutamate-gated chloride channel
RS012047	GO:0022891	substrate-specific transmembrane transporter activity	MF	-1.033691566	-1.204387105	-0.573210977	3.717195265	16.53395031	0.000881135	0.004370625	PREDICTED: sodium-independent sulfate anion transporter-like
RS010364	GO:0022891	substrate-specific transmembrane transporter activity	MF	-0.052944401	0.405459181	0.815688442	7.091949691	16.43172938	0.000924767	0.004565472	PREDICTED: cytochrome c oxidase subunit 7A2, mitochondrial
RS002855	GO:0022891	substrate-specific transmembrane transporter activity	MF	-1.74839573	-1.535258414	-0.16366084	1.79539144	16.39328726	0.000941725	0.00463647	PREDICTED: glycine receptor subunit alpha-3
RS000927	GO:0022891	substrate-specific transmembrane transporter activity	MF	-2.570527298	-2.804707147	-0.755163456	0.667619183	16.35029216	0.000961058	0.004715063	PREDICTED: b(0,+)-type amino acid transporter 1-like
RS009094	GO:0022891	substrate-specific transmembrane transporter activity	MF	0.810509918	0.868482563	0.707370764	6.315762678	16.22341499	0.001020437	0.004986955	PREDICTED: mitochondrial import inner membrane translocase subunit Tim17-B-like
RS003544	GO:0022891	substrate-specific transmembrane transporter activity	MF	-0.410771227	-0.348918681	1.010423069	3.516674428	16.0029682	0.001132396	0.005455777	PREDICTED: glutamate receptor ionotropic, kainate 2
RS006141	GO:0022891	substrate-specific transmembrane transporter activity	MF	0.186642093	0.509454722	0.888482209	5.536921472	15.98603197	0.001141486	0.005494665	Potassium channel subfamily K member 9
RS003914	GO:0022891	substrate-specific transmembrane transporter activity	MF	-0.408304954	-1.308021582	-1.113950825	3.842131166	15.96402321	0.001153407	0.005540047	PREDICTED: facilitated trehalose transporter Tret1-like
RS013870	GO:0022891	substrate-specific transmembrane transporter activity	MF	-1.185159015	-0.789079424	-0.942978819	4.024142004	15.86729932	0.001207277	0.005765813	PREDICTED: sodium- and chloride-dependent glycine transporter 1-like
RS013164	GO:0022891	substrate-specific transmembrane transporter activity	MF	-0.720894807	-1.573394185	-0.567112816	2.35012097	15.85215695	0.001215934	0.005800558	PREDICTED: sodium/hydrogen exchanger 3
RS014761	GO:0022891	substrate-specific transmembrane transporter activity	MF	0.850386459	0.827647699	0.741742978	6.600879234	15.79201097	0.001250932	0.005936034	PREDICTED: mitochondrial import receptor subunit TOM40 homolog 1
RS012306	GO:0022891	substrate-specific transmembrane transporter activity	MF	-1.171605666	-1.08070317	-0.099574832	5.667246046	15.66500563	0.001328153	0.006252994	PREDICTED: ABC transporter G family member 23
RS012806	GO:0022891	substrate-specific transmembrane transporter activity	MF	-0.671548237	-0.771532863	-0.831977584	6.320380877	15.5512679	0.001401314	0.006555774	PREDICTED: glutamate [NMDA] receptor subunit 1
RS004534	GO:0022891	substrate-specific transmembrane transporter activity	MF	-0.580382142	2.035273319	0.497901415	1.025113205	15.51468926	0.001425682	0.006654944	PREDICTED: sodium channel protein Nach-like
RS000928	GO:0022891	substrate-specific transmembrane transporter activity	MF	-2.818387407	-2.387215456	-1.195316578	0.324906115	15.29049888	0.001584497	0.007309578	B(0,+)-type amino acid transporter 1
RS006610	GO:0022891	substrate-specific transmembrane transporter activity	MF	0.2664942	0.92285009	0.741592285	5.324734931	15.2782684	0.001593649	0.007342643	PREDICTED: zinc transporter 7
RS008896	GO:0022891	substrate-specific transmembrane transporter activity	MF	-1.274733506	-1.006034401	-0.767037953	2.62762816	15.13459455	0.001705167	0.007799012	PREDICTED: mitoferrin-1
RS006757	GO:0022891	substrate-specific transmembrane transporter activity	MF	-0.233100515	0.411940029	0.749443033	4.26814335	14.98825356	0.001826715	0.008260555	PREDICTED: cell death-inducing p53-target protein 1
RS009126	GO:0022891	substrate-specific transmembrane transporter activity	MF	0.004334845	0.550905396	0.691970165	7.068681097	14.88906784	0.001913944	0.008599435	PREDICTED: V-type proton ATPase subunit F
RS006318	GO:0022891	substrate-specific transmembrane transporter activity	MF	-3.618949223	-2.702749807	-1.072386578	0.467850565	14.73641507	0.002056334	0.009147816	PREDICTED: bumetanide-sensitive sodium-(potassium)-chloride cotransporter
RS005708	GO:0022891	substrate-specific transmembrane transporter activity	MF	-2.57707626	-0.767810406	-0.121337824	1.208165742	14.61680361	0.002175202	0.009598631	PREDICTED: sodium- and chloride-dependent glycine transporter 1-like
RS013194	GO:0022891	substrate-specific transmembrane transporter activity	MF	-0.402235939	1.468418978	0.620702468	1.800456379	14.57025873	0.002223278	0.009779937	PREDICTED: gamma-aminobutyric acid receptor alpha-like
RS008446	GO:0022891	substrate-specific transmembrane transporter activity	MF	0.363470434	-0.27703378	-1.408383647	4.990252537	14.55183725	0.002242595	0.009858024	PREDICTED: facilitated trehalose transporter Tret1-2 homolog
RS008491	GO:0022891	substrate-specific transmembrane transporter activity	MF	-0.83498967	-1.675590127	-1.132486269	2.878684988	14.45091871	0.00235141	0.010286093	PREDICTED: callisulfakinin
RS012689	GO:0022891	substrate-specific transmembrane transporter activity	MF	-0.335162446	-0.922302748	-1.067051914	4.164281375	14.43562454	0.002368352	0.010353914	PREDICTED: solute carrier family 41 member 1-like
RS008723	GO:0022891	substrate-specific transmembrane transporter activity	MF	0.765724899	0.492155106	-0.726953396	2.604372289	14.25219643	0.002581214	0.011140444	PREDICTED: excitatory amino acid transporter
RS005190	GO:0022891	substrate-specific transmembrane transporter activity	MF	-0.561761317	0.20318587	0.563166122	6.506172496	14.19948267	0.002645822	0.011376401	PREDICTED: calcium-activated potassium channel slowpoke
RS013212	GO:0022891	substrate-specific transmembrane transporter activity	MF	-0.878244991	-0.110123051	0.018139925	7.487946573	14.16068921	0.002694389	0.011553674	PREDICTED: V-type proton ATPase subunit B
RS011379	GO:0022891	substrate-specific transmembrane transporter activity	MF	-0.945895165	-0.137739549	0.02413553	6.197611827	14.11352967	0.002754622	0.01177587	PREDICTED: chloride intracellular channel exc-4
RS014046	GO:0022891	substrate-specific transmembrane transporter activity	MF	-0.827652517	0.123541367	0.39500677	4.371003694	13.78924417	0.003206526	0.0134386	PREDICTED: zinc transporter ZIP1-like
RS006255	GO:0022891	substrate-specific transmembrane transporter activity	MF	-0.565848028	-0.409978879	0.214379631	6.839035823	13.48918712	0.003689736	0.015225603	PREDICTED: copper-transporting ATPase 1
RS012010	GO:0022891	substrate-specific transmembrane transporter activity	MF	-0.658055692	-0.163236513	0.25753802	6.662027841	13.45566221	0.003748014	0.01543574	PREDICTED: sodium-dependent phosphate transporter 1-B-like
RS010731	GO:0022891	substrate-specific transmembrane transporter activity	MF	-0.395182325	-0.639006133	-0.675231861	6.20607259	13.34648856	0.003944186	0.016153848	PREDICTED: V-type proton ATPase 116 kDa subunit a
RS006343	GO:0022891	substrate-specific transmembrane transporter activity	MF	0.700005517	0.678494058	0.385858572	5.661988764	13.33113205	0.003972583	0.016264862	PREDICTED: zinc transporter 6
RS010905	GO:0022891	substrate-specific transmembrane transporter activity	MF	-0.573248945	-0.768712677	-0.837001645	4.71486708	13.23543162	0.00415417	0.016871208	PREDICTED: gamma-aminobutyric acid receptor subunit beta
RS014000	GO:0022891	substrate-specific transmembrane transporter activity	MF	0.486986842	0.493065666	-0.511287156	7.326747337	12.99389808	0.00464982	0.018525729	TWiK family of potassium channels protein 18
RS007615	GO:0022891	substrate-specific transmembrane transporter activity	MF	0.842199769	0.08001472	-0.354074907	7.382223287	12.96671251	0.004709147	0.018738389	PREDICTED: facilitated trehalose transporter Tret1-like
RS009863	GO:0022891	substrate-specific transmembrane transporter activity	MF	0.247967949	1.994626146	0.840783905	0.741237487	12.85234652	0.004967042	0.019634316	PREDICTED: sodium- and chloride-dependent glycine transporter 1-like
RS005166	GO:0022891	substrate-specific transmembrane transporter activity	MF	0.797745292	0.959053152	1.137980998	8.88871257	12.83598972	0.005005051	0.019744379	PREDICTED: nascent polypeptide-associated complex subunit alpha
RS005241	GO:0022891	substrate-specific transmembrane transporter activity	MF	-1.24648029	-0.946331544	-0.692073388	4.41212653	12.5745733	0.005653054	0.021933014	PREDICTED: high-affinity choline transporter 1
RS006299	GO:0022891	substrate-specific transmembrane transporter activity	MF	0.929966764	0.90845916	-0.169322471	3.276482954	12.53656321	0.005753939	0.022276393	PREDICTED: facilitated trehalose transporter Tret1-like
RS008051	GO:0022891	substrate-specific transmembrane transporter activity	MF	-0.405673123	-1.320265794	-1.066020494	4.63196021	12.49923358	0.00585475	0.022555742	PREDICTED: transient receptor potential channel pyrexia
RS000227	GO:0022891	substrate-specific transmembrane transporter activity	MF	-1.32952285	-0.75353739	-1.12184367	1.981758767	12.38833226	0.00616466	0.023605269	PREDICTED: facilitated trehalose transporter Tret1-2 homolog
RS012250	GO:0022891	substrate-specific transmembrane transporter activity	MF	-1.126562781	-0.381786444	0.539034757	1.985904525	12.14501042	0.00690257	0.026042829	PREDICTED: organic cation transporter protein-like
RS002985	GO:0022891	substrate-specific transmembrane transporter activity	MF	-0.571114377	-0.476136182	-0.859409917	5.548946497	12.07709757	0.007123669	0.026732864	PREDICTED: Y+L amino acid transporter 2
RS008215	GO:0022891	substrate-specific transmembrane transporter activity	MF	0.162922308	0.46909669	0.671411093	6.515514341	12.06788131	0.007154208	0.026831478	PREDICTED: mitochondrial import receptor subunit TOM22 homolog
RS003288	GO:0022891	substrate-specific transmembrane transporter activity	MF	-0.468141457	-0.663116971	0.331112622	4.523147937	12.02307867	0.007304519	0.027292266	PREDICTED: sodium- and chloride-dependent GABA transporter ine
RS003997	GO:0022891	substrate-specific transmembrane transporter activity	MF	-1.222850277	-0.635965438	-0.411407393	6.247970682	11.97127476	0.007482212	0.027852345	PREDICTED: ABC transporter G family member 23
RS011468	GO:0022891	substrate-specific transmembrane transporter activity	MF	-0.318737056	-0.110014844	-1.568608212	1.963272184	11.9167036	0.007674014	0.028433815	PREDICTED: solute carrier family 35 member F6
RS000866	GO:0022891	substrate-specific transmembrane transporter activity	MF	-0.851954648	-0.389874807	-0.092110043	7.989972168	11.90615845	0.007711634	0.028547895	PREDICTED: sodium/potassium-transporting ATPase subunit alpha
RS015096	GO:0022891	substrate-specific transmembrane transporter activity	MF	-0.857980934	-0.446788274	-0.194471442	5.697782265	11.71197794	0.008437836	0.030900291	PREDICTED: V-type proton ATPase subunit D
RS009360	GO:0022891	substrate-specific transmembrane transporter activity	MF	-0.052778092	-0.516301747	0.304429459	6.031942967	11.69868265	0.008489957	0.031037017	PREDICTED: sodium-dependent nutrient amino acid transporter 1
RS008458	GO:0022891	substrate-specific transmembrane transporter activity	MF	-1.182823715	-0.196910535	-0.390818396	8.429616967	11.6884148	0.008530428	0.031131399	PREDICTED: probable cation-transporting ATPase 13A3
RS004997	GO:0022891	substrate-specific transmembrane transporter activity	MF	-0.851696275	-1.530670443	-0.345032758	2.982640462	11.64463017	0.008705154	0.031658304	PREDICTED: kinesin-like protein KIF17
RS008898	GO:0022891	substrate-specific transmembrane transporter activity	MF	-0.172960297	-0.478698026	-0.774628888	4.936093792	11.53878925	0.009142244	0.033009582	PREDICTED: sideroflexin-1
RS002295	GO:0022891	substrate-specific transmembrane transporter activity	MF	-0.760632417	-0.881718429	-1.336627404	7.31207469	11.50237509	0.009297576	0.033436228	PREDICTED: sodium- and chloride-dependent GABA transporter 1-like
RS012787	GO:0022891	substrate-specific transmembrane transporter activity	MF	-1.042887095	-1.14108662	-0.848878197	2.917253142	11.38739346	0.009805347	0.03491082	PREDICTED: facilitated trehalose transporter Tret1-2 homolog
RS007389	GO:0022891	substrate-specific transmembrane transporter activity	MF	-0.71821859	-0.059688714	0.178211598	8.141021926	11.17756615	0.010803454	0.037931058	PREDICTED: V-type proton ATPase catalytic subunit A
RS007388	GO:0022891	substrate-specific transmembrane transporter activity	MF	-1.012601041	-0.502641916	-0.211964145	6.238906794	11.11536226	0.011118147	0.038884183	PREDICTED: V-type proton ATPase catalytic subunit A
RS006740	GO:0022891	substrate-specific transmembrane transporter activity	MF	-0.43064001	-0.612335737	-0.738849827	6.295225865	10.98541247	0.011805018	0.040967869	PREDICTED: glutamate-gated chloride channel
RS007836	GO:0022891	substrate-specific transmembrane transporter activity	MF	-0.670404831	1.05713246	-0.759263575	1.495067002	10.91840834	0.012175354	0.042079146	PREDICTED: bestrophin-4
RS000123	GO:0022891	substrate-specific transmembrane transporter activity	MF	0.692109916	0.810423229	0.325162475	7.0801236	10.8417935	0.012612853	0.04340062	PREDICTED: ATP-binding cassette sub-family F member 3
RS012007	GO:0022891	substrate-specific transmembrane transporter activity	MF	-0.8805953	0.446042174	-0.616249485	2.603532973	10.7020781	0.013450915	0.045771636	PREDICTED: sodium-dependent phosphate transporter 2
RS002237	GO:0022891	substrate-specific transmembrane transporter activity	MF	-0.77443485	-0.782975074	-0.864409769	3.087700691	10.69522479	0.013493406	0.045891426	PREDICTED: transient receptor potential cation channel protein painless
RS010501	GO:0022891	substrate-specific transmembrane transporter activity	MF	-0.249581193	0.164542324	0.830736506	8.23731792	10.57427827	0.014265398	0.048114201	PREDICTED: ATP synthase subunit e, mitochondrial
RS013211	GO:0022891	substrate-specific transmembrane transporter activity	MF	-0.46592983	-1.425136997	0.127678346	1.648820506	10.50692227	0.014714013	0.049415541	PREDICTED: pickpocket protein 19-like
RS005829	GO:0022891	substrate-specific transmembrane transporter activity	MF	-0.565901365	-0.398114437	0.075675202	5.880326068	10.47839461	0.014908177	0.04996104	PREDICTED: facilitated trehalose transporter Tret1-like
RS012348	GO:0022891	substrate-specific transmembrane transporter activity	MF	-1.171136152	-0.623448827	-0.241561177	4.399276235	10.47489738	0.014932153	0.050028076	V-type proton ATPase subunit S1
RS004574	GO:0022891	substrate-specific transmembrane transporter activity	MF	-0.256414135	0.160648707	0.765161971	8.179741228	10.46991438	0.01496638	0.050122195	PREDICTED: ATP synthase subunit delta, mitochondrial
RS012105	GO:0022891	substrate-specific transmembrane transporter activity	MF	-0.587037702	-0.604268783	0.220956679	3.910659327	10.37503836	0.015632991	0.051906642	Inward rectifier potassium channel 2
RS000868	GO:0022891	substrate-specific transmembrane transporter activity	MF	-0.929392451	-0.475108864	-0.289097862	8.324170965	10.36013171	0.01574035	0.052235565	PREDICTED: sodium/potassium-transporting ATPase subunit alpha
RS008445	GO:0022891	substrate-specific transmembrane transporter activity	MF	-0.794376386	-2.696738816	-1.11925367	1.261432481	10.25254337	0.016537016	0.054463175	PREDICTED: facilitated trehalose transporter Tret1-2 homolog
RS008079	GO:0022891	substrate-specific transmembrane transporter activity	MF	-1.109710923	-0.938014135	-0.296800028	2.900944855	10.13930135	0.017418391	0.056816166	PREDICTED: potassium voltage-gated channel protein Shaker
RS009153	GO:0022891	substrate-specific transmembrane transporter activity	MF	0.613293967	0.998084897	0.966219795	3.330543492	10.10124836	0.017724806	0.057621634	PREDICTED: mitochondrial import inner membrane translocase subunit Tim9
RS003950	GO:0022891	substrate-specific transmembrane transporter activity	MF	-0.587671115	-0.868990799	-0.714405964	4.844171479	10.0941278	0.017782729	0.057765203	PREDICTED: cationic amino acid transporter 2
RS005883	GO:0022891	substrate-specific transmembrane transporter activity	MF	0.579677196	0.662723942	0.941061143	6.190916775	10.02257363	0.018375223	0.059324417	PREDICTED: mitochondrial import inner membrane translocase subunit Tim10
RS009608	GO:0022891	substrate-specific transmembrane transporter activity	MF	-0.650681402	-0.131877234	-0.002301287	6.339415917	9.831393766	0.020055087	0.063636624	PREDICTED: V-type proton ATPase subunit C
RS002046	GO:0022891	substrate-specific transmembrane transporter activity	MF	-1.040945999	-1.141609481	-1.378037143	3.132734296	9.81352734	0.020219576	0.06404561	PREDICTED: sodium- and chloride-dependent GABA transporter 1-like
RS001413	GO:0022891	substrate-specific transmembrane transporter activity	MF	-0.248212965	0.06615521	0.706495165	7.98044758	9.766834252	0.020655746	0.065132313	PREDICTED: cytochrome b-c1 complex subunit 7-like
RS015433	GO:0022891	substrate-specific transmembrane transporter activity	MF	-1.023491195	-0.871650733	-0.547044844	4.320060789	9.694765434	0.021347153	0.067027185	PREDICTED: glutamate receptor ionotropic, kainate 2
RS012325	GO:0022891	substrate-specific transmembrane transporter activity	MF	-0.610322555	-0.73163567	0.587302153	2.890049632	9.633930261	0.021948434	0.068590218	PREDICTED: serine/threonine-protein phosphatase 6 regulatory ankyrin repeat subunit A
RS001773	GO:0022891	substrate-specific transmembrane transporter activity	MF	-1.179645502	-1.391479741	-1.203064835	2.188248107	9.497351264	0.023359555	0.072211644	PREDICTED: acetylcholine receptor subunit alpha-like
RS006809	GO:0022891	substrate-specific transmembrane transporter activity	MF	-0.069599294	0.161390813	-0.669189997	5.521140821	9.427681054	0.02411329	0.074118459	PREDICTED: sodium bicarbonate cotransporter 3
RS003636	GO:0022891	substrate-specific transmembrane transporter activity	MF	0.658899301	-0.027122995	0.775979951	5.812404377	9.412838955	0.024276916	0.074486992	PREDICTED: ATP-binding cassette sub-family G member 4
RS007719	GO:0022891	substrate-specific transmembrane transporter activity	MF	-0.363554514	0.201823484	0.241238455	6.872179235	9.390306687	0.024527407	0.075102884	PREDICTED: V-type proton ATPase 21 kDa proteolipid subunit

RS005942	GO:0022891	substrate-specific transmembrane transporter activity	MF	0.222003058	0.357500804	-0.707071584	4.173704825	9.375192754	0.024696847	0.075474905	PREDICTED: mitochondrial sodium/hydrogen exchanger 9B2-like
RS009928	GO:0022891	substrate-specific transmembrane transporter activity	MF	-0.835456878	-2.347674561	-0.849585016	1.053053669	9.23428246	0.02633282	0.079385129	PREDICTED: sodium-dependent noradrenaline transporter-like
RS003234	GO:0022891	substrate-specific transmembrane transporter activity	MF	-0.402942012	0.351806881	0.253791792	7.730694199	9.198914238	0.026759846	0.080432815	PREDICTED: V-type proton ATPase 16 kDa proteolipid subunit
RS012808	GO:0022891	substrate-specific transmembrane transporter activity	MF	0.436531101	0.768358177	0.047570539	8.601906761	9.12311113	0.027698056	0.082719781	PREDICTED: ATP-binding cassette sub-family E member 1
RS007617	GO:0022891	substrate-specific transmembrane transporter activity	MF	-1.32510407	-1.458516586	-0.870134155	3.353068646	9.039095435	0.028775582	0.085310659	PREDICTED: zinc transporter ZIP10
RS009139	GO:0022891	substrate-specific transmembrane transporter activity	MF	0.649351007	1.292845053	0.336142374	2.039377777	9.031613663	0.028873505	0.085540561	PREDICTED: Y+L amino acid transporter 2
RS002268	GO:0022891	substrate-specific transmembrane transporter activity	MF	-0.663213265	-0.543123609	-0.277002394	5.289469542	8.984030819	0.029503961	0.086978671	PREDICTED: inositol 1,4,5-trisphosphate receptor
RS013576	GO:0022891	substrate-specific transmembrane transporter activity	MF	-0.152692106	0.186422925	0.739608273	8.524782718	8.933399856	0.030189603	0.088564608	PREDICTED: cytochrome c oxidase subunit 7A2, mitochondrial-like
RS008214	GO:0022891	substrate-specific transmembrane transporter activity	MF	-0.591657749	-0.60801793	-0.342781866	5.622477097	8.89592842	0.030707059	0.089873274	PREDICTED: sodium channel protein paralytic
RS007859	GO:0022891	substrate-specific transmembrane transporter activity	MF	-1.037355681	-0.953164057	-1.137934678	2.631254112	8.895408464	0.0307143	0.08987358	PREDICTED: proton-coupled amino acid transporter 4
RS003404	GO:0022891	substrate-specific transmembrane transporter activity	MF	-0.282230962	0.285723529	0.434899732	4.438872301	8.825502366	0.031703186	0.092359456	PREDICTED: DET1- and DDB1-associated protein 1
RS013668	GO:0022891	substrate-specific transmembrane transporter activity	MF	-1.170027407	-0.729178035	0.872714223	0.383894951	8.820674505	0.031772619	0.092540325	PREDICTED: glutamate receptor 3.4-like
RS000001	GO:0022891	substrate-specific transmembrane transporter activity	MF	-0.923753843	-0.774146522	-0.309728113	8.964042611	8.801674733	0.032047317	0.093060619	PREDICTED: ryanodine receptor 44F
RS009135	GO:0022891	substrate-specific transmembrane transporter activity	MF	-2.013397474	0.050668944	0.558887993	0.476879882	8.748046843	0.032835258	0.094920773	PREDICTED: sodium channel protein Nach-like
RS011130	GO:0022891	substrate-specific transmembrane transporter activity	MF	0.62266454	0.316671327	-0.198111492	8.646709884	8.691352167	0.03368886	0.096889131	PREDICTED: 60 kDa heat shock protein, mitochondrial-like
RS014303	GO:0022891	substrate-specific transmembrane transporter activity	MF	1.015519516	-0.140449015	-1.617687421	1.096446077	8.671576225	0.033991683	0.097559539	PREDICTED: sodium channel protein Nach-like
RS001434	GO:0022891	substrate-specific transmembrane transporter activity	MF	-2.10642209	-0.940504046	-0.189980266	0.468874389	8.555806562	0.035818546	0.101461711	PREDICTED: sodium channel protein Nach-like
RS012852	GO:0022891	substrate-specific transmembrane transporter activity	MF	0.095937018	0.366584098	-0.497735083	4.298329529	8.535728142	0.036145003	0.102133761	PREDICTED: glutamate receptor ionotropic, kainate 2-like
RS005358	GO:0022891	substrate-specific transmembrane transporter activity	MF	0.260075089	0.471390925	0.694718137	6.608164203	8.461122113	0.037383693	0.104938933	PREDICTED: surfeit locus protein 1
RS006017	GO:0022891	substrate-specific transmembrane transporter activity	MF	-0.226333875	0.829010789	0.173010283	3.457042927	8.42538268	0.037991671	0.106302401	PREDICTED: cationic amino acid transporter 2
RS003858	GO:0022891	substrate-specific transmembrane transporter activity	MF	-0.359815662	-0.446595841	0.382189615	3.498410245	8.385315512	0.038684752	0.10786673	PREDICTED: sodium/potassium-transporting ATPase subunit beta-2-like
RS012784	GO:0022891	substrate-specific transmembrane transporter activity	MF	-0.964188065	-1.053670411	-1.487092291	6.693074429	8.119987423	0.043595935	0.118762068	PREDICTED: excitatory amino acid transporter-like
RS005143	GO:0022891	substrate-specific transmembrane transporter activity	MF	0.352665656	0.523610265	0.606871417	5.289665213	8.000205041	0.046007468	0.124178752	PREDICTED: protein SCO1 homolog, mitochondrial
RS002239	GO:0022891	substrate-specific transmembrane transporter activity	MF	-0.518558054	-0.887931947	-1.328774582	2.771875246	7.996103171	0.04609231	0.124359208	PREDICTED: transient receptor potential cation channel protein painless
RS008614	GO:0022891	substrate-specific transmembrane transporter activity	MF	-0.377384484	0.178151297	0.139169662	6.960985845	7.911936368	0.047867212	0.127882831	PREDICTED: H(+)/Cl(-) exchange transporter 5
RS007665	GO:0022891	substrate-specific transmembrane transporter activity	MF	-0.631881952	-0.428365421	0.007983181	8.098194771	7.871009593	0.048754144	0.129949141	PREDICTED: plasma membrane calcium-transporting ATPase 2
RS003841	GO:0022891	substrate-specific transmembrane transporter activity	MF	-0.018830809	0.667905762	-0.171179406	4.919354176	7.849821458	0.04921958	0.130930145	PREDICTED: major facilitator superfamily domain-containing protein 10
RS008434	GO:0022891	substrate-specific transmembrane transporter activity	MF	-0.152418883	-0.335351871	-0.836822092	4.94412872	7.808586514	0.050137806	0.132702402	PREDICTED: potassium voltage-gated channel subfamily H member 2
RS010293	GO:0022891	substrate-specific transmembrane transporter activity	MF	-1.323536549	-0.537537624	-0.40616185	2.969834016	7.797939139	0.050377598	0.133149101	PREDICTED: sodium/hydrogen exchanger 3
RS002931	GO:0022891	substrate-specific transmembrane transporter activity	MF	-0.784498789	-0.645948105	-0.625581434	3.345246421	7.781766391	0.050743963	0.133976828	PREDICTED: acetylcholine receptor subunit beta-like 2
RS012051	GO:0022891	substrate-specific transmembrane transporter activity	MF	-1.206457775	-0.320139517	-0.400442703	3.93903891	7.774642457	0.050906164	0.13434875	PREDICTED: sodium-independent sulfate anion transporter-like
RS012317	GO:0022891	substrate-specific transmembrane transporter activity	MF	-0.648427381	0.001131192	-0.007541087	6.300999393	7.771586789	0.050975892	0.13447641	PREDICTED: calcium-transporting ATPase type 2C member 1
RS003265	GO:0022891	substrate-specific transmembrane transporter activity	MF	0.67065611	0.570992468	0.622458253	5.669387556	7.641310173	0.054036623	0.140868812	PREDICTED: 28S ribosomal protein S9, mitochondrial
RS015407	GO:0022891	substrate-specific transmembrane transporter activity	MF	-0.53343531	-0.824506724	0.003613607	6.554411162	7.589715422	0.055297539	0.143502413	PREDICTED: sodium/potassium-transporting ATPase subunit beta-2
RS005847	GO:0022891	substrate-specific transmembrane transporter activity	MF	-0.009134422	0.240630937	0.785370217	8.208365721	7.589016609	0.055314812	0.143517665	PREDICTED: ATP synthase subunit epsilon, mitochondrial-like
RS014918	GO:0022891	substrate-specific transmembrane transporter activity	MF	-0.265116474	0.084232844	0.568475259	7.79189049	7.503165544	0.057477171	0.147999897	PREDICTED: cytochrome c1, heme protein, mitochondrial
RS007235	GO:0022891	substrate-specific transmembrane transporter activity	MF	-0.411884685	-0.555436113	-0.88679334	5.718214674	7.500689334	0.057540743	0.148133302	PREDICTED: melanotransferrin
RS009082	GO:0022891	substrate-specific transmembrane transporter activity	MF	-1.344763505	-0.953478298	-1.303298962	1.512508029	7.470773642	0.058314177	0.149663762	PREDICTED: glycine receptor subunit alpha-2-like
RS008448	GO:0022891	substrate-specific transmembrane transporter activity	MF	-0.874319748	-1.179805365	-0.159545862	2.543187294	7.449190097	0.058878449	0.150714469	PREDICTED: facilitated trehalose transporter Tret1-2 homolog
RS002799	GO:0022891	substrate-specific transmembrane transporter activity	MF	-0.659046602	-0.211273757	-0.16274335	6.419710777	7.409924332	0.059918615	0.152787009	PREDICTED: V-type proton ATPase subunit H
RS002292	GO:0022891	substrate-specific transmembrane transporter activity	MF	-0.849226044	-0.724590211	-0.752733052	3.999828127	7.339934983	0.06181703	0.156330055	V-type proton ATPase subunit S1
RS004018	GO:0022891	substrate-specific transmembrane transporter activity	MF	-0.854655712	-0.687918747	0.356622885	2.251863917	7.340140368	0.061811374	0.156330055	PREDICTED: potassium/sodium hyperpolarization-activated cyclic nucleotide-gated channel 2
RS010902	GO:0022891	substrate-specific transmembrane transporter activity	MF	-0.413608261	-0.854721106	-0.933757255	3.754238005	7.337994128	0.061870497	0.156389668	PREDICTED: glutamate receptor 1
RS006810	GO:0022891	substrate-specific transmembrane transporter activity	MF	-0.692918241	-0.709642301	-0.469276201	5.552917252	7.276110601	0.063598968	0.159523084	PREDICTED: Y+L amino acid transporter 2
RS006920	GO:0022891	substrate-specific transmembrane transporter activity	MF	-0.632866598	-0.974633547	-0.967627708	3.248752993	7.241310635	0.064591429	0.161433709	PREDICTED: glutamate receptor ionotropic, NMDA 2B
RS008996	GO:0022891	substrate-specific transmembrane transporter activity	MF	-0.818894069	-0.144607229	0.175883673	2.981815036	7.237819263	0.064691824	0.161620478	PREDICTED: cytochrome c oxidase assembly factor 6 homolog
RS002605	GO:0022891	substrate-specific transmembrane transporter activity	MF	-0.518735488	0.392463203	-0.08725497	4.03235098	7.22499608	0.065061855	0.162287383	PREDICTED: solute carrier family 40 member 1
RS000609	GO:0022891	substrate-specific transmembrane transporter activity	MF	0.612008555	0.732502903	0.190053663	8.31658549	7.219606678	0.065217984	0.162580225	PREDICTED: muscle calcium channel subunit alpha-1
RS006503	GO:0022891	substrate-specific transmembrane transporter activity	MF	-0.789357073	-0.069673576	-0.042357301	4.655019811	7.200877419	0.065763394	0.163551378	PREDICTED: transient receptor potential cation channel trpm
RS015105	GO:0022891	substrate-specific transmembrane transporter activity	MF	-0.678666411	-2.224992259	-0.562865466	0.559101899	7.185153044	0.066224707	0.164438873	PREDICTED: organic cation transporter 1
RS005264	GO:0022891	substrate-specific transmembrane transporter activity	MF	-1.488549143	-1.308665565	-1.379377385	1.600885014	7.17732657	0.066455483	0.164816926	PREDICTED: sodium-dependent neutral amino acid transporter B(0)AT3
RS006739	GO:0022891	substrate-specific transmembrane transporter activity	MF	-0.215562063	-0.194338635	-0.661480143	5.23328973	7.126492884	0.067973454	0.167558754	PREDICTED: potassium voltage-gated channel protein Shab
RS000133	GO:0022891	substrate-specific transmembrane transporter activity	MF	-0.045454902	0.182331435	0.994722318	2.687399163	7.116976504	0.068261333	0.168071011	PREDICTED: putative ammonium transporter 1
RS015404	GO:0022891	substrate-specific transmembrane transporter activity	MF	-0.033173942	0.491254687	-0.13268681	6.364272838	7.035330626	0.07078015	0.172954058	PREDICTED: sodium/potassium-transporting ATPase subunit beta-2
RS003449	GO:0022891	substrate-specific transmembrane transporter activity	MF	-0.271604031	-0.623910119	-0.38398901	6.1655336	7.019104607	0.07129133	0.173865804	PREDICTED: glutamate receptor ionotropic, kainate 2
RS014295	GO:0022891	substrate-specific transmembrane transporter activity	MF	0.412981653	0.489533068	0.464743455	5.949145628	7.0080526	0.071641551	0.174618482	PREDICTED: zinc transporter 5
RS003825	GO:0022891	substrate-specific transmembrane transporter activity	MF	-0.116552603	-0.74841501	-0.195189608	7.935069367	6.915883201	0.07462774	0.18005155	PREDICTED: H(+)/Cl(-) exchange transporter 7
RS003427	GO:0022891	substrate-specific transmembrane transporter activity	MF	-0.513964722	-0.550228674	-0.09809219	5.664872118	6.801016027	0.078517892	0.186983778	PREDICTED: voltage-dependent calcium channel type A subunit alpha-1
RS002296	GO:0022891	substrate-specific transmembrane transporter activity	MF	0.050929101	-1.155041098	-1.453067074	0.872761787	6.743534644	0.080537346	0.190763087	PREDICTED: glycine receptor subunit alpha-4
RS003145	GO:0022891	substrate-specific transmembrane transporter activity	MF	-1.309880541	-0.628756059	-0.991127574	1.553035926	6.713260678	0.081620986	0.19245815	PREDICTED: neuronal acetylcholine receptor subunit alpha-7-like
RS012048	GO:0022891	substrate-specific transmembrane transporter activity	MF	-0.674894361	-1.155560739	-0.486644875	2.649535097	6.691851744	0.082395778	0.193852499	PREDICTED: sodium-independent sulfate anion transporter-like
RS009471	GO:0022891	substrate-specific transmembrane transporter activity	MF	0.454440582	0.068570398	0.92902968	1.975531925	6.680170813	0.082821497	0.194672252	PREDICTED: potassium voltage-gated channel protein Shal
RS000035	GO:0022891	substrate-specific transmembrane transporter activity	MF	-0.59529335	-0.304820032	0.347972721	8.866264808	6.597065948	0.085912068	0.200794204	PREDICTED: cytochrome b-c1 complex subunit Rieske, mitochondrial-like
RS000318	GO:0022891	substrate-specific transmembrane transporter activity	MF	-0.409411201	-0.696964023	-0.524046279	5.146315283	6.540394841	0.088082972	0.204696733	PREDICTED: alpha-aminoadipic semialdehyde dehydrogenase
RS001821	GO:0022891	substrate-specific transmembrane transporter activity	MF	-0.416559115	-0.243117375	0.420607418	9.114591662	6.45061119	0.091630759	0.210852874	PREDICTED: cytochrome c oxidase subunit 4 isoform 1, mitochondrial
RS007738	GO:0022891	substrate-specific transmembrane transporter activity	MF	-2.236721279	-1.816342735	0.104485909	0.00738012	6.381695156	0.094446755	0.215638584	PREDICTED: dynein heavy chain 10, axonemal
RS006697	GO:0022891	substrate-specific transmembrane transporter activity	MF	-0.137394972	-0.578930188	0.149350385	8.454117513	6.353830787	0.095608773	0.21750305	PREDICTED: zinc transporter ZIP13 homolog
RS007190	GO:0022891	substrate-specific transmembrane transporter activity	MF	-0.878979816	-0.595509543	0.227085088	2.704210207	6.34629855	0.095925237	0.217838484	PREDICTED: sodium channel protein Nach-like
RS010275	GO:0022891	substrate-specific transmembrane transporter activity	MF	-0.327809837	0.151537852	-0.881426827	3.129420115	6.335110353	0.096397162	0.218625222	PREDICTED: equilibrative nucleoside transporter 4
RS015389	GO:0022891	substrate-specific transmembrane transporter activity	MF	-1.014604708	-1.708407111	-0.480189784	0.854123779	6.332870963	0.096491888	0.218718663	PREDICTED: glutamate receptor ionotropic, delta-2
RS009966	GO:0022891	substrate-specific transmembrane transporter activity	MF	-0.243614202	-0.508062646	-0.78761656	3.789270855	6.228722713	0.100997397	0.225925617	PREDICTED: transient receptor potential channel pyrexia-like
RS005351	GO:0022891	substrate-specific transmembrane transporter activity	MF	-0.397867942	-0.039889938	0.245645216	7.41412513	6.082183312	0.107679573	0.237000444	PREDICTED: glucose transporter type 1
RS011409	GO:0022891	substrate-specific transmembrane transporter activity	MF	-0.278990895	0.058673971	0.653521491	10.28933054	5.992909669	0.111955695	0.243939451	PREDICTED: voltage-dependent anion-selective channel protein 2
RS000168	GO:0022891	substrate-specific transmembrane transporter activity	MF	-0.555511687	-0.887978015	-1.234124807	1.652741901	5.837936606	0.119766759	0.255954957	PREDICTED: potassium voltage-gated channel protein eag
RS000398	GO:0022891	substrate-specific transmembrane transporter activity	MF	0.277198819	0.379861004	-0.066027388	5.894739848	5.761774521	0.123793477	0.26277685	PREDICTED: RNA polymerase I-specific transcription initiation factor RRN3
RS001145	GO:0022891	substrate-specific transmembrane transporter activity	MF	-0.53262649	-0.12107554	0.117285332	6.762749099	5.723923744	0.125842321	0.265692906	PREDICTED: solute carrier family 2, facilitated glucose transporter member 1-like
RS003358	GO:0022891	substrate-specific transmembrane transporter activity	MF	-0.353225898	-0.121840444	0.371616686	8.291426996	5.633056759	0.130893435	0.272925814	PREDICTED: ATP synthase-coupling factor 6, mitochondrial
RS008279	GO:0022891	substrate-specific transmembrane transporter activity	MF	-0.484679453	-0.433954641	0.086422434	3.880506245	5.606020043	0.132433181	0.27531586	PREDICTED: potassium voltage-gated channel protein Shaw-like
RS009361	GO:0022891	substrate-specific transmembrane transporter activity	MF	-0.469548252	-1.030089652	-0.272125463	2.379260128	5.571170404	0.134443268	0.278300217	PREDICTED: sodium- and chloride-dependent glycine transporter 2-like
RS002972	GO:0022891	substrate-specific transmembrane transporter activity	MF	-0.311172151	-0.506842424	-0.237806006	6.495567074	5.534163734	0.136609467	0.281857737	PREDICTED: sodium leak channel non-selective protein
RS012532	GO:0022891	substrate-specific transmembrane transporter activity	MF	0.52422059	0.27169055	0.170626759	5.950381512	5.527522768	0.137001687	0.282351902	PREDICTED: adenosine 3'-phospho 5'-phosphosulfate transporter 2
RS007587	GO:0022891	substrate-specific transmembrane transporter activity	MF	0.548015678	0.376775275	0.454348959	4.86046888	5.504926438	0.138344259	0.284411948	PREDICTED: magnesium transporter NIPA2
RS012098	GO:0022891	substrate-specific transmembrane transporter activity	MF	-0.722635213	-0.863193595	-0.599560773	3.5591329	5.486483871	0.13944927	0.285797251	PREDICTED: protein tweety
RS009262	GO:0022891	substrate-specific transmembrane transporter activity	MF	-0.25857572	0.038639212	0.546361074	8.794488927	5.469506202	0.140473903	0.28738277	PREDICTED: cytochrome c oxidase subunit 7C, mitochondrial-like
RS012521	GO:0022891	substrate-specific transmembrane transporter activity	MF	-0.27911124	0.207963234	0.176143396	5.682101261	5.464162095	0.140797903	0.287847294	PREDICTED: sodium/hydrogen exchanger 8
RS000402	GO:0022891	substrate-specific transmembrane transporter activity	MF	-0.26718069	0.008072239	0.51581876	8.673900207	5.301862053	0.150981611	0.302828859	PREDICTED: cytochrome c oxidase subunit 6A, mitochondrial
RS006927	GO:0022891	substrate-specific transmembrane transporter activity	MF	-0.523545819	-0.19238077	0.403170712	9.665847911	5.252945184	0.154185014	0.307637209	PREDICTED: mitochondrial-processing peptidase subunit beta
RS014310	GO:0022891	substrate-specific transmembrane transporter activity	MF	-0.394503871	-0.23364982	-0.939036808	2.647212913	5.221628248	0.156269422	0.310811292	PREDICTED: neuronal acetylcholine receptor subunit alpha-7
RS004262	GO:0022891	substrate-specific transmembrane transporter activity	MF	-0.304556625	0.103631535	0.676510563	10.41026235	5.204184057	0.157441978	0.312748307	PREDICTED: ATP synthase lipid-binding protein, mitochondrial
RS007787	GO:0022891	substrate-specific transmembrane transporter activity	MF	-0.305912555	0.11337882	0.601620189	9.43857381	5.186627171	0.15863048	0.314655166	PREDICTED: ATP synthase subunit b, mitochondrial
RS008202	GO:0022891	substrate-specific transmembrane transporter activity	MF	0.24252685	0.342844048	-0.965951499	1.493557986	5.185516154	0.158705973	0.314677881	PREDICTED: voltage-dependent calcium channel gamma-5 subunit
RS002769	GO:0022891	substrate-specific transmembrane transporter activity	MF	-0.180596666	-0.510025269	-0.494314256	5.266272897	5.147923967	0.161280396	0.318264698	PREDICTED: mitochondrial 2-oxoglutarate/malate carrier protein
RS000666	GO:0022891	substrate-specific transmembrane transporter activity	MF	-0.080539122	0.013403368	0.652885093	9.136399161	5.147116783	0.161336103	0.31832475	PREDICTED: ATP synthase subunit g, mitochondrial
RS006076	GO:0022891	substrate-specific transmembrane transporter activity	MF	-0.399500112	-0.234112198	-0.791572834	3.218719557	5.135126595	0.162165734	0.319411206	PREDICTED: cationic amino acid transporter 4
RS015132	GO:0022891	substrate-specific transmembrane transporter activity	MF	-0.311288053	0.021217655	0.441252337	8.852842665	5.035692944	0.169202112	0.329814486	PREDICTED: ATP synthase subunit O, mitochondrial
RS001012	GO:0022891	substrate-specific transmembrane transporter activity	MF	-0.050637771	0.65837124	0.220417241	5.028311487	5.034189195	0.169310694	0.329872366	PREDICTED: sodium/calcium exchanger 2
RS007406	GO:0022891	substrate-specific transmembrane transporter activity	MF	1.53218285	1.416234104	0.234676903	0.509107572	4.915967777	0.17805483	0.341676447	PREDICTED: sodium-independent sulfate anion transporter-like
RS007390	GO:0022891	substrate-specific transmembrane transporter activity	MF	-0.350929593	-1.171722101	-0.259139814	1.654126139	4.900550276	0.179225874	0.343191525	PREDICTED: open rectifier potassium channel protein 1
RS012974	GO:0022891	substrate-specific transmembrane transporter activity	MF	0.386362631	0.350475987	0.590578301	4.958398051	4.857275707	0.182551447	0.347919632	PREDICTED: mitochondrial import inner membrane translocase subunit Tim16
RS004430	GO:0022891	substrate-specific transmembrane transporter activity	MF	-0.579491375	-0.057917442	0.425475066	2.309902759	4.852857272	0.182894224	0.348520179	Glutamate [NMDA] receptor subunit 3A
RS015406	GO:0022891	substrate-specific transmembrane transporter activity	MF	-0.548835798	-0.34994381	-0.083936965	5.176471396	4.839807661	0.183910115	0.349694714	PREDICTED: sodium/potassium-transporting ATPase subunit beta-2
RS015035	GO:0022891	substrate-specific transmembrane transporter activity	MF	-0.486032243	-0.438869682	-0.112800307	5.262898327	4.77200592	0.189273765	0.356097071	PREDICTED: solute carrier family 2, facilitated glucose transporter member 10-like
RS011272	GO:0022891	substrate-specific transmembrane transporter activity	MF	-0.449550793	-0.842262029	0.317704025	1.68576224	4.742178986	0.191679247	0.358960362	PREDICTED: transient receptor potential-gamma protein
RS013062	GO:0022891	substrate-specific transmembrane transporter activity	MF	-0.00162788	-0.815422885	-0.945361754	1.375980209	4.702121991	0.194954616	0.363203911	PREDICTED: transient receptor potential channel pyrexia
RS008733	GO:0022891	substrate-specific transmembrane transporter activity	MF	-0.069301153	-0.696190609	0.565661049	1.222507914	4.700705743	0.195071368	0.363260212	PREDICTED: pickpocket protein 28-like
RS005980	GO:0022891	substrate-specific transmembrane transporter activity	MF	-0.519964769	-0.115820264	0.014177289	6.649793844	4.674091701	0.197277507	0.365853766	PREDICTED: V-type proton ATPase subunit d
RS013193	GO:0022891	substrate-specific transmembrane transporter activity	MF	-0.571318105	-0.638002435	-0.492911161	3.125045391	4.612135042	0.202503545	0.372595481	PREDICTED: gamma-aminobutyric acid receptor subunit beta-like
RS012106	GO:0022891	substrate-specific transmembrane transporter activity	MF	-0.818692691	-0.45460686	0.366734327	1.141769569	4.599841297	0.203555696	0.373808755	Inward rectifier potassium channel 2
RS009973	GO:0022891	substrate-specific transmembrane transporter activity	MF	0.1676973	0.302067179	0.694181931	3.324934717	4.531641986	0.2094852	0.381030706	PREDICTED: sugar transporter SWEET1
RS002890	GO:0022891	substrate-specific transmembrane transporter activity	MF	-0.213107922	-0.035236467	0.494651933	9.00551627	4.518839864	0.210615958	0.382259313	PREDICTED: cytochrome c oxidase subunit 5A, mitochondrial
RS012324	GO:0022891	substrate-specific transmembrane transporter activity	MF	-0.639112315	-1.234689077	-0.08776879	1.305640976	4.37485531	0.22372824	0.397633848	PREDICTED: serine/threonine-protein phosphatase 6 regulatory ankyrin repeat subunit A

RS009362	GO:0022891	substrate-specific transmembrane transporter activity	MF	-0.151734251	-1.379851033	-0.853593219	0.577299208	4.353138426	0.225770076	0.399764452	PREDICTED: sodium- and chloride-dependent glycine transporter 2-like
RS001539	GO:0022891	substrate-specific transmembrane transporter activity	MF	-0.380736825	-1.0292407	-0.36481089	2.349632725	4.316399244	0.229263428	0.403767811	PREDICTED: transient receptor potential cation channel protein painless
RS005280	GO:0022891	substrate-specific transmembrane transporter activity	MF	0.572920685	0.052379455	0.938447567	3.032392488	4.273149565	0.233439508	0.40894939	PREDICTED: acid-sensing ion channel 4
RS001911	GO:0022891	substrate-specific transmembrane transporter activity	MF	-0.354858321	-0.511067736	-0.530111822	4.46637499	4.265443861	0.234190845	0.409944281	PREDICTED: palmitoyltransferase ZDHHC17
RS007791	GO:0022891	substrate-specific transmembrane transporter activity	MF	-0.379787464	0.049869261	0.105041138	7.48004628	4.225689753	0.238102407	0.41486742	PREDICTED: V-type proton ATPase subunit E
RS015079	GO:0022891	substrate-specific transmembrane transporter activity	MF	-1.376295246	0.661290456	0.618943801	0.209343525	4.174647883	0.243212401	0.420568148	PREDICTED: facilitated trehalose transporter Tret1-like
RS012398	GO:0022891	substrate-specific transmembrane transporter activity	MF	-0.181909214	0.045983675	0.559515665	9.326609998	4.173573583	0.243321024	0.420643099	PREDICTED: cytochrome c oxidase subunit 5B, mitochondrial-like
RS011634	GO:0022891	substrate-specific transmembrane transporter activity	MF	-1.482237858	-0.869085331	-1.279144023	0.748490273	4.162431432	0.24445023	0.422083595	PREDICTED: glutamate receptor ionotropic, delta-2-like
RS001774	GO:0022891	substrate-specific transmembrane transporter activity	MF	-0.556967124	-0.686693785	0.064399051	2.246327006	4.014186998	0.25993626	0.440531132	PREDICTED: acetylcholine receptor subunit alpha-like
RS012212	GO:0022891	substrate-specific transmembrane transporter activity	MF	1.298461473	1.241884362	0.487723216	0.415207106	3.920422645	0.270186252	0.451829193	PREDICTED: sodium channel protein 60E
RS000049	GO:0022891	substrate-specific transmembrane transporter activity	MF	-0.167016261	-0.078808593	0.315739518	4.19490186	3.918242209	0.270428905	0.452114912	PREDICTED: protein I'm not dead yet-like
RS004419	GO:0022891	substrate-specific transmembrane transporter activity	MF	-0.39398529	0.162481593	-1.185227216	0.809494392	3.887905498	0.273825486	0.455851742	PREDICTED: transient receptor potential-gamma protein
RS003691	GO:0022891	substrate-specific transmembrane transporter activity	MF	-0.396884624	-0.571293958	0.253637659	1.856747806	3.871872414	0.275636118	0.457912129	PREDICTED: transient receptor potential cation channel subfamily A member 1
RS010418	GO:0022891	substrate-specific transmembrane transporter activity	MF	-0.19455437	0.000394051	0.481391778	9.395600975	3.855673322	0.277476464	0.459791431	PREDICTED: cytochrome c oxidase subunit 6B1-like
RS011747	GO:0022891	substrate-specific transmembrane transporter activity	MF	-0.380454491	0.007681606	0.358897081	9.615965078	3.849118736	0.278224261	0.460864966	PREDICTED: ATP synthase subunit gamma, mitochondrial
RS000693	GO:0022891	substrate-specific transmembrane transporter activity	MF	-0.697277451	-0.63859341	-1.105155719	1.668516432	3.761841056	0.288355965	0.47128419	PREDICTED: solute carrier family 12 member 4
RS006724	GO:0022891	substrate-specific transmembrane transporter activity	MF	1.072023102	0.112416307	0.609701895	0.789605252	3.761098346	0.288443587	0.47128419	PREDICTED: glutamate receptor ionotropic, delta-1-like
RS002930	GO:0022891	substrate-specific transmembrane transporter activity	MF	-0.451673532	-0.71437108	-0.718954078	3.306419228	3.701408177	0.295564164	0.478695356	PREDICTED: acetylcholine receptor subunit alpha-L1
RS005072	GO:0022891	substrate-specific transmembrane transporter activity	MF	-0.654987192	-0.4219481	-0.18940484	3.26003723	3.685352455	0.297506145	0.480656437	PREDICTED: voltage-dependent T-type calcium channel subunit alpha-1G
RS008197	GO:0022891	substrate-specific transmembrane transporter activity	MF	-0.307158998	-0.373647818	-0.769382915	3.163133414	3.663806527	0.300130093	0.48236835	PREDICTED: lysophospholipid acyltransferase 7
RS003147	GO:0022891	substrate-specific transmembrane transporter activity	MF	-0.505550451	-0.027606287	-0.18120612	3.786984288	3.627252198	0.30462908	0.487532937	PREDICTED: acetylcholine receptor subunit beta-like 1
RS014617	GO:0022891	substrate-specific transmembrane transporter activity	MF	-0.072631645	0.084385518	0.526454273	9.138635249	3.625994331	0.304784959	0.487596216	PREDICTED: putative ATP synthase subunit f, mitochondrial
RS012817	GO:0022891	substrate-specific transmembrane transporter activity	MF	-0.287960684	-0.628111107	-0.66343646	3.635326683	3.625082508	0.304897998	0.48766642	PREDICTED: facilitated trehalose transporter Tret1-like
RS015381	GO:0022891	substrate-specific transmembrane transporter activity	MF	-0.14122205	0.494615068	-0.457720223	1.269481569	3.572397731	0.311493056	0.493953414	PREDICTED: potassium voltage-gated channel subfamily KQT member 1
RS008419	GO:0022891	substrate-specific transmembrane transporter activity	MF	-0.579270886	-0.22684126	-0.07023873	5.06982487	3.507414936	0.319801701	0.502353125	PREDICTED: chloride channel protein 2
RS013677	GO:0022891	substrate-specific transmembrane transporter activity	MF	0.034211804	0.280550659	0.595537596	9.222031987	3.437461301	0.328964218	0.510658864	PREDICTED: cytochrome c oxidase subunit 6C
RS009119	GO:0022891	substrate-specific transmembrane transporter activity	MF	-0.633398641	0.027582619	-0.254763808	2.374824839	3.400802845	0.333857375	0.51571198	PREDICTED: lateral signaling target protein 2 homolog
RS006561	GO:0022891	substrate-specific transmembrane transporter activity	MF	0.353995304	0.237572917	0.419480704	5.635873753	3.395244005	0.334604917	0.516387054	PREDICTED: EH domain-containing protein 3
RS015453	GO:0022891	substrate-specific transmembrane transporter activity	MF	-0.09263959	0.415348464	0.277714417	5.686672294	3.355959113	0.33992981	0.521573591	PREDICTED: sodium-dependent phosphate transporter 2
RS008482	GO:0022891	substrate-specific transmembrane transporter activity	MF	0.456620351	0.231742272	0.664992756	4.899508409	3.329273867	0.343589006	0.524567819	PREDICTED: organic cation transporter protein
RS008880	GO:0022891	substrate-specific transmembrane transporter activity	MF	0.14769946	0.553054828	0.336408558	3.369176491	3.28851771	0.349243966	0.529733651	PREDICTED: pickpocket protein 19-like
RS008726	GO:0022891	substrate-specific transmembrane transporter activity	MF	-0.239198332	0.278689624	0.146315272	4.384525019	3.151643572	0.368830479	0.550035649	PREDICTED: equilibrative nucleoside transporter 3
RS008888	GO:0022891	substrate-specific transmembrane transporter activity	MF	-0.294502555	-0.567523121	-0.206094758	5.180319559	3.112412951	0.374615949	0.554657096	PREDICTED: facilitated trehalose transporter Tret1-2 homolog
RS003956	GO:0022891	substrate-specific transmembrane transporter activity	MF	-0.323676554	0.116745023	-0.07691896	6.422899031	3.056887659	0.382937112	0.562262205	PREDICTED: solute carrier family 2, facilitated glucose transporter member 1-like
RS003609	GO:0022891	substrate-specific transmembrane transporter activity	MF	-0.051627285	0.355758402	0.153478437	5.36998255	3.050524042	0.383900772	0.56337058	PREDICTED: zinc transporter ZIP1
RS000591	GO:0022891	substrate-specific transmembrane transporter activity	MF	-0.210745642	-0.711938748	-0.062273638	2.249015287	2.914724059	0.404960781	0.581735608	PREDICTED: solute carrier family 41 member 1-like
RS005802	GO:0022891	substrate-specific transmembrane transporter activity	MF	-0.201437577	-0.069914946	-0.593279778	3.766942431	2.895793271	0.407972418	0.584129676	PREDICTED: two pore potassium channel protein sup-9
RS001904	GO:0022891	substrate-specific transmembrane transporter activity	MF	-0.27687136	-0.081401122	0.3505686	9.687890931	2.842286187	0.416586082	0.592334615	PREDICTED: ATP synthase subunit d, mitochondrial
RS011686	GO:0022891	substrate-specific transmembrane transporter activity	MF	-0.652816077	-0.692313812	-0.834928913	1.23665669	2.748017073	0.432128872	0.606039089	PREDICTED: TWiK family of potassium channels protein 18-like
RS012107	GO:0022891	substrate-specific transmembrane transporter activity	MF	-0.368799719	0.188292375	-0.013404979	5.272470317	2.653348424	0.448213543	0.621757305	PREDICTED: G protein-activated inward rectifier potassium channel 3-like
RS014879	GO:0022891	substrate-specific transmembrane transporter activity	MF	-0.246283404	-0.362594032	0.41505142	1.337328694	2.627851228	0.452627775	0.625660651	PREDICTED: glycine receptor subunit alphaZ1-like
RS007790	GO:0022891	substrate-specific transmembrane transporter activity	MF	0.13034852	0.225712601	0.386180211	4.625198528	2.606288996	0.456388071	0.628856824	PREDICTED: mitochondrial glutamate carrier 1-like
RS010426	GO:0022891	substrate-specific transmembrane transporter activity	MF	-0.0320805	-0.19528696	-0.535965982	2.761673686	2.581268642	0.460782863	0.632865926	PREDICTED: glutamate receptor ionotropic, NMDA 2B
RS007973	GO:0022891	substrate-specific transmembrane transporter activity	MF	0.255068646	0.370011576	0.284397768	6.620720548	2.53491853	0.469013562	0.640437689	PREDICTED: zinc transporter 9
RS009279	GO:0022891	substrate-specific transmembrane transporter activity	MF	0.294401775	0.208728083	0.372173078	5.384293591	2.517191015	0.472192292	0.643035221	PREDICTED: probable low affinity copper uptake protein 2
RS003198	GO:0022891	substrate-specific transmembrane transporter activity	MF	-1.073241752	-1.028205771	-0.905668183	0.370380606	2.506990701	0.474029032	0.644352037	PREDICTED: glutamate receptor ionotropic, delta-1-like
RS003214	GO:0022891	substrate-specific transmembrane transporter activity	MF	0.817513479	0.952415195	0.900489695	0.817958486	2.496776884	0.475873854	0.64588375	PREDICTED: potassium voltage-gated channel subfamily KQT member 4
RS006138	GO:0022891	substrate-specific transmembrane transporter activity	MF	0.077942232	0.584419518	-0.370544993	1.33370078	2.490635188	0.476985892	0.646552279	PREDICTED: potassium channel subfamily K member 18-like
RS000905	GO:0022891	substrate-specific transmembrane transporter activity	MF	0.022025399	0.042858662	0.42533997	8.298571127	2.483368496	0.478304268	0.647507681	PREDICTED: cytochrome b-c1 complex subunit 10-like
RS012899	GO:0022891	substrate-specific transmembrane transporter activity	MF	0.204967532	0.199038052	0.483016212	5.559314101	2.452259782	0.483980622	0.652426585	PREDICTED: SID1 transmembrane family member 1-like
RS009087	GO:0022891	substrate-specific transmembrane transporter activity	MF	-0.753493655	-0.466320961	-0.51762636	2.340610515	2.452889414	0.483865213	0.652426585	PREDICTED: probable glutamate receptor
RS007514	GO:0022891	substrate-specific transmembrane transporter activity	MF	-0.393644456	0.206862252	-0.369998274	2.851975701	2.440356048	0.48616657	0.653933758	PREDICTED: solute carrier family 26 member 10-like
RS002311	GO:0022891	substrate-specific transmembrane transporter activity	MF	0.21167088	0.356479307	0.364913574	7.124016358	2.415178378	0.490815427	0.65773111	PREDICTED: putative GTP-binding protein 6
RS004471	GO:0022891	substrate-specific transmembrane transporter activity	MF	0.302505199	0.1016203	-0.272900349	3.077100879	2.396738775	0.494241988	0.660492741	PREDICTED: Y+L amino acid transporter 2
RS003266	GO:0022891	substrate-specific transmembrane transporter activity	MF	-0.36926559	-0.039795183	-0.401685153	5.103564049	2.37990733	0.497385826	0.663356676	PREDICTED: transient receptor potential channel pyrexia
RS006811	GO:0022891	substrate-specific transmembrane transporter activity	MF	-0.091072902	-0.032205255	0.237958262	6.439935586	2.353693428	0.502312778	0.667124828	PREDICTED: angio-associated migratory cell protein
RS005021	GO:0022891	substrate-specific transmembrane transporter activity	MF	-0.06781788	-0.474899669	-0.350734329	3.155367473	2.352458366	0.50254583	0.667200314	PREDICTED: molybdate-anion transporter-like
RS001365	GO:0022891	substrate-specific transmembrane transporter activity	MF	0.237278489	0.141297916	0.554160331	3.551638791	2.315424336	0.509572456	0.67338898	PREDICTED: glutamate receptor ionotropic, kainate 2
RS014502	GO:0022891	substrate-specific transmembrane transporter activity	MF	-0.219647182	0.224994314	0.605975653	1.297553296	2.311846878	0.51025516	0.673954219	PREDICTED: small conductance calcium-activated potassium channel protein
RS001131	GO:0022891	substrate-specific transmembrane transporter activity	MF	-0.46672796	-0.438731687	-0.485486742	3.00696323	2.28306329	0.515773294	0.678325905	PREDICTED: two pore calcium channel protein 1-like
RS005763	GO:0022891	substrate-specific transmembrane transporter activity	MF	-0.049864591	0.331988566	0.418799139	2.785560138	2.265395088	0.519182685	0.681106738	PREDICTED: TWiK family of potassium channels protein 7
RS002215	GO:0022891	substrate-specific transmembrane transporter activity	MF	-0.459893543	-0.373663745	-0.327616751	3.245153068	2.258109459	0.520593488	0.682313913	PREDICTED: CMP-sialic acid transporter 1
RS010646	GO:0022891	substrate-specific transmembrane transporter activity	MF	-0.387797302	-0.153467445	-0.201059514	5.65127642	2.191739529	0.533577259	0.692056848	PREDICTED: probable phospholipid-transporting ATPase IA
RS002929	GO:0022891	substrate-specific transmembrane transporter activity	MF	-0.39492811	-0.310803863	-0.368352585	3.630774923	2.110337646	0.54982422	0.704208911	PREDICTED: acetylcholine receptor subunit alpha-like 1
RS009552	GO:0022891	substrate-specific transmembrane transporter activity	MF	-0.317174334	0.102956036	0.402195532	11.51695741	2.083221242	0.555314671	0.708862049	PREDICTED: ATP synthase subunit beta, mitochondrial
RS002902	GO:0022891	substrate-specific transmembrane transporter activity	MF	-0.415647108	-0.152738152	0.265175951	11.47381982	2.020686865	0.56812419	0.719524944	PREDICTED: ATP synthase subunit alpha, mitochondrial
RS010798	GO:0022891	substrate-specific transmembrane transporter activity	MF	0.021270494	0.09209859	-1.197682465	0.555972403	2.01223563	0.569871042	0.720350873	PREDICTED: zinc transporter ZIP11
RS005231	GO:0022891	substrate-specific transmembrane transporter activity	MF	-0.391467446	-0.412384168	-0.179305009	4.071451755	1.941339648	0.584670698	0.731588315	PREDICTED: organic cation transporter protein
RS014789	GO:0022891	substrate-specific transmembrane transporter activity	MF	-0.384309417	-0.340794963	-0.701560358	2.91880799	1.866727447	0.600523037	0.745342183	PREDICTED: zinc transporter ZIP9
RS010157	GO:0022891	substrate-specific transmembrane transporter activity	MF	0.252451639	0.308689007	0.216067222	5.404322605	1.826234541	0.609242714	0.751719677	PREDICTED: exportin-T
RS005281	GO:0022891	substrate-specific transmembrane transporter activity	MF	0.147692306	-0.427946659	0.130849224	2.285946009	1.819237088	0.610757704	0.752790867	PREDICTED: acid-sensing ion channel 4
RS008886	GO:0022891	substrate-specific transmembrane transporter activity	MF	-0.289536803	-0.51024615	-0.479741606	2.079554922	1.757477457	0.624231737	0.763037416	PREDICTED: facilitated trehalose transporter Tret1
RS014536	GO:0022891	substrate-specific transmembrane transporter activity	MF	-0.137268948	-0.07750242	-0.358531119	4.210868086	1.71200184	0.634268817	0.771080465	PREDICTED: cyclic nucleotide-gated cation channel beta-3
RS015165	GO:0022891	substrate-specific transmembrane transporter activity	MF	0.203835116	0.086955701	-0.092770826	6.68983845	1.645463293	0.649125814	0.780587272	PREDICTED: sodium channel protein 60E
RS003602	GO:0022891	substrate-specific transmembrane transporter activity	MF	-0.963739329	-0.484991816	-0.758608967	0.476696176	1.634124817	0.651677236	0.782683875	PREDICTED: solute carrier family 28 member 3
RS010427	GO:0022891	substrate-specific transmembrane transporter activity	MF	-0.256640259	-0.293251267	-0.831731644	1.070368751	1.607645315	0.657657541	0.78679007	PREDICTED: glutamate receptor ionotropic, NMDA 2B
RS004131	GO:0022891	substrate-specific transmembrane transporter activity	MF	0.244077702	0.428184836	0.166004547	3.05086288	1.47403705	0.688275398	0.807916894	PREDICTED: synaptic vesicle 2-related protein
RS003990	GO:0022891	substrate-specific transmembrane transporter activity	MF	-0.055368209	0.238070575	0.293320892	4.25914611	1.454595738	0.692788544	0.811066898	PREDICTED: UDP-galactose translocator
RS009066	GO:0022891	substrate-specific transmembrane transporter activity	MF	-0.284333504	-0.421360118	-0.423891407	2.706723454	1.434128224	0.697554745	0.814482149	PREDICTED: solute carrier family 35 member E3-like
RS015498	GO:0022891	substrate-specific transmembrane transporter activity	MF	0.184679301	0.472371696	0.26432428	2.413896137	1.414695214	0.702093736	0.818113296	PREDICTED: calcium uniporter protein, mitochondrial
RS014968	GO:0022891	substrate-specific transmembrane transporter activity	MF	0.070753676	0.338611082	0.008973976	4.167384444	1.401868016	0.705096916	0.82085326	PREDICTED: solute carrier family 41 member 1-like
RS011446	GO:0022891	substrate-specific transmembrane transporter activity	MF	-0.226602437	0.017489836	-0.173735734	4.50551615	1.392641248	0.707260567	0.822459795	PREDICTED: stress-activated protein kinase JNK
RS005975	GO:0022891	substrate-specific transmembrane transporter activity	MF	0.184305527	-0.340054217	-0.375631512	2.198485768	1.388734626	0.708177509	0.823222037	PREDICTED: piezo-type mechanosensitive ion channel component
RS004705	GO:0022891	substrate-specific transmembrane transporter activity	MF	-0.430701847	-0.465359916	-0.484712083	3.064343052	1.370281204	0.712515526	0.826890945	PREDICTED: sodium/hydrogen exchanger 7
RS001366	GO:0022891	substrate-specific transmembrane transporter activity	MF	-0.202685308	-0.234557446	-0.316543451	6.418554265	1.353303228	0.71651627	0.829698961	PREDICTED: glutamate receptor ionotropic, kainate 2
RS010673	GO:0022891	substrate-specific transmembrane transporter activity	MF	0.194686816	0.144745443	0.237663354	5.4673327	1.348021968	0.717762586	0.830648431	PREDICTED: zinc transporter foi-like
RS005974	GO:0022891	substrate-specific transmembrane transporter activity	MF	-0.266864024	-0.195937093	-0.171697422	7.122329353	1.335637313	0.720688539	0.832387331	PREDICTED: piezo-type mechanosensitive ion channel component
RS009390	GO:0022891	substrate-specific transmembrane transporter activity	MF	-0.711993959	-0.803385471	-0.355292865	0.366452897	1.289104566	0.731721917	0.839984739	PREDICTED: gamma-aminobutyric acid receptor alpha-like
RS002882	GO:0022891	substrate-specific transmembrane transporter activity	MF	0.362043543	0.12287268	0.14951259	3.567153224	1.277605364	0.734457649	0.842342193	PREDICTED: putative sodium-coupled neutral amino acid transporter 7
RS015540	GO:0022891	substrate-specific transmembrane transporter activity	MF	-0.463597962	-0.808832157	-0.297537157	1.427647784	1.257020479	0.73936339	0.845811839	PREDICTED: zinc transporter ZIP11
RS013953	GO:0022891	substrate-specific transmembrane transporter activity	MF	-0.420067551	-0.07959435	-0.044577109	2.09881014	1.242877248	0.742739999	0.848223421	PREDICTED: two pore potassium channel protein sup-9
RS000297	GO:0022891	substrate-specific transmembrane transporter activity	MF	-0.078397003	0.556951886	0.159163891	1.385608421	1.213828293	0.749689662	0.85220612	PREDICTED: probable glutamate receptor
RS008405	GO:0022891	substrate-specific transmembrane transporter activity	MF	-0.507380646	-0.391727947	-0.160990784	2.501471485	1.183658987	0.75692617	0.857267824	PREDICTED: solute carrier family 12 member 9
RS010672	GO:0022891	substrate-specific transmembrane transporter activity	MF	-0.302029346	0.22206596	0.191043022	1.595359022	1.095814464	0.778084584	0.871039755	Zinc transporter 2
RS003408	GO:0022891	substrate-specific transmembrane transporter activity	MF	0.14875062	-0.250114218	0.329780306	2.915781806	1.09604514	0.778028887	0.871039755	PREDICTED: probable glutamate receptor
RS011943	GO:0022891	substrate-specific transmembrane transporter activity	MF	0.336788752	0.302655858	-0.329953803	0.718404792	1.062395748	0.786158762	0.876649854	PREDICTED: glutamate receptor ionotropic, delta-1-like
RS001346	GO:0022891	substrate-specific transmembrane transporter activity	MF	-0.305712719	-0.459994451	-0.225727855	2.010578526	1.048797397	0.789446703	0.878744111	PREDICTED: glutamate receptor ionotropic, delta-1-like
RS004706	GO:0022891	substrate-specific transmembrane transporter activity	MF	-0.281248638	-0.101038833	-0.415774939	3.347688564	1.047260677	0.789818336	0.878882504	PREDICTED: sodium/hydrogen exchanger 7
RS007717	GO:0022891	substrate-specific transmembrane transporter activity	MF	0.066529061	0.463299961	0.014408782	1.808915589	0.956769727	0.811710752	0.892106596	PREDICTED: V-type proton ATPase 21 kDa proteolipid subunit
RS006075	GO:0022891	substrate-specific transmembrane transporter activity	MF	-0.226394382	-0.065362369	0.088572196	3.008430896	0.898142308	0.82587609	0.901150881	PREDICTED: acetyl-coenzyme A transporter 1
RS008929	GO:0022891	substrate-specific transmembrane transporter activity	MF	-0.139724103	-0.236598473	-0.091823273	4.57881475	0.895160984	0.826595415	0.901623303	PREDICTED: cytochrome c oxidase assembly protein COX15 homolog
RS011500	GO:0022891	substrate-specific transmembrane transporter activity	MF	-0.54663125	-0.559810746	-0.038971768	0.470844775	0.876166842	0.831175153	0.904842	PREDICTED: zinc transporter ZIP8
RS013172	GO:0022891	substrate-specific transmembrane transporter activity	MF	0.064707769	0.050177661	0.256276698	4.54520608	0.870549691	0.832528393	0.905630734	PREDICTED: N-acetyl-D-glucosamine kinase
RS001970	GO:0022891	substrate-specific transmembrane transporter activity	MF	0.084033429	-0.012169719	-0.157558613	5.885614174	0.833122903	0.84152916	0.911638874	PREDICTED: glutamate receptor ionotropic, kainate 2
RS005586	GO:0022891	substrate-specific transmembrane transporter activity	MF	0.165076617	-0.125717332	0.501179805	0.440219349	0.817686596	0.845232123	0.914113642	big brain
RS006019	GO:0022891	substrate-specific transmembrane transporter activity	MF	0.016800687	-0.569398535	-0.206623396	0.539126613	0.775635116	0.855285471	0.920559373	PREDICTED: cationic amino acid transporter 2
RS000298	GO:0022891	substrate-specific transmembrane transporter activity	MF	-0.477815194	-0.328207506	-0.189725936	1.140735658	0.76073894	0.858832832	0.922721437	PREDICTED: glutamate receptor ionotropic, NMDA 3A-like
RS010574	GO:0022891	substrate-specific transmembrane transporter activity	MF	-0.354727613	-0.373915239	-0.481676921	0.828534929	0.681580005	0.877527727	0.933866301	PREDICTED: G protein-activated inward rectifier potassium channel 3-like
RS001040	GO:0022891	substrate-specific transmembrane transporter activity	MF	-0.098603565	-0.201658646	0.076364834	3.089669028	0.652037571	0.884422889	0.938003319	PREDICTED: sideroflexin-2
RS003957	GO:0022891	substrate-specific transmembrane transporter activity	MF	0.345462579	0.413965389	0.154609396	1.754535313	0.612321805	0.893605875	0.942823504	PREDICTED: solute carrier family 2, facilitated glucose transporter member 1-like
RS003729	GO:0022891	substrate-specific transmembrane transporter activity	MF	-0.067068908	-0.31722074	-0.453287028	1.039779299	0.608180172	0.894557183	0.943318527	PREDICTED: neuronal acetylcholine receptor subunit alpha-7-like

RS003394	GO:0022891	substrate-specific transmembrane transporter activity	MF	0.180289073	0.135383955	0.089380518	6.008505153	0.597460048	0.89701359	0.944722291	PREDICTED: vesicle transport protein USE1
RS001538	GO:0022891	substrate-specific transmembrane transporter activity	MF	0.320201018	0.172065415	-0.020378547	1.614496317	0.588836165	0.898983255	0.94603515	PREDICTED: allatostatin-A receptor-like
RS014940	GO:0022891	substrate-specific transmembrane transporter activity	MF	0.00473671	-0.350880149	-0.354295712	0.736422598	0.582482205	0.900430699	0.946555742	PREDICTED: potassium voltage-gated channel protein Shal
RS005560	GO:0022891	substrate-specific transmembrane transporter activity	MF	0.073648066	0.070694895	-0.087436803	5.960124723	0.555903739	0.906448712	0.950525819	PREDICTED: protein catecholamines up
RS004110	GO:0022891	substrate-specific transmembrane transporter activity	MF	-0.109326082	-0.245764423	-0.393853677	2.856144774	0.536964028	0.910698682	0.95276119	PREDICTED: phospholipase A2 inhibitor and Ly6/PLAUR domain-containing protein
RS000285	GO:0022891	substrate-specific transmembrane transporter activity	MF	0.119687123	0.031475064	-0.04428715	4.541388379	0.422896761	0.935472985	0.966238561	PREDICTED: facilitated trehalose transporter Tret1-like
RS014584	GO:0022891	substrate-specific transmembrane transporter activity	MF	0.129219334	0.060601072	0.090579059	6.064060989	0.417243203	0.936657865	0.966828377	PREDICTED: wolframin
RS002672	GO:0022891	substrate-specific transmembrane transporter activity	MF	0.112781414	0.136232963	0.12426065	4.219978077	0.286688066	0.96251234	0.980258807	PREDICTED: mitochondrial import inner membrane translocase subunit Tim22
RS007404	GO:0022891	substrate-specific transmembrane transporter activity	MF	0.341548622	0.423696565	0.486833568	3.079113058	0.267214539	0.9660718	0.982215891	PREDICTED: sodium-independent sulfate anion transporter-like
RS009012	GO:0022891	substrate-specific transmembrane transporter activity	MF	0.165169583	0.002447419	-0.023721248	2.359677794	0.265786109	0.966329283	0.982398368	PREDICTED: zinc transporter 2-like
RS010152	GO:0022891	substrate-specific transmembrane transporter activity	MF	-0.129723253	-0.161823215	-0.203053025	1.88639679	0.263658934	0.966711776	0.98246999	PREDICTED: zinc transporter ZIP3
RS007304	GO:0022891	substrate-specific transmembrane transporter activity	MF	-0.03874979	-0.117709759	-0.024621974	4.815387852	0.237457293	0.971327222	0.985126901	PREDICTED: solute carrier family 17 member 9
RS002585	GO:0022891	substrate-specific transmembrane transporter activity	MF	0.061337763	0.015683494	0.119579633	4.655608643	0.205533408	0.976691112	0.987602807	PREDICTED: mitochondrial pyruvate carrier 1
RS008629	GO:0022891	substrate-specific transmembrane transporter activity	MF	0.051370738	-0.258316351	-0.177872255	0.376549123	0.205807073	0.976646438	0.987602807	PREDICTED: probable glutamate receptor
RS006774	GO:0022891	substrate-specific transmembrane transporter activity	MF	-0.005561161	-0.002818033	0.022604747	3.411433614	0.005716578	0.999885243	0.999885243	PREDICTED: equilibrative nucleoside transporter 1
RS007652	GO:0022892	substrate-specific transporter activity	MF	0.585883623	0.528505008	4.851177054	4.584889963	291.2117195	7.93792E-63	8.92591E-61	PREDICTED: solute carrier family 26 member 10-like
RS003129	GO:0022892	substrate-specific transporter activity	MF	-1.935857211	0.013218622	3.698610157	4.556804627	145.8197805	2.10104E-31	9.2418E-30	PREDICTED: organic cation transporter protein
RS004606	GO:0022892	substrate-specific transporter activity	MF	-0.152775887	3.199032258	1.582974114	7.379011967	144.4003621	4.25168E-31	1.83376E-29	PREDICTED: peptide transporter family 1
RS004125	GO:0022892	substrate-specific transporter activity	MF	1.407227463	2.012417871	-0.423642507	5.906215214	131.7530272	2.26607E-28	8.8083E-27	PREDICTED: ABC transporter G family member 9
RS003228	GO:0022892	substrate-specific transporter activity	MF	-0.561103954	2.12048168	1.83683218	5.406987214	114.6440514	1.09829E-24	3.71824E-23	PREDICTED: b(0,+)-type amino acid transporter 1-like
RS013974	GO:0022892	substrate-specific transporter activity	MF	1.308057058	2.315023189	0.876327879	6.127768048	111.7248777	4.66769E-24	1.53887E-22	PREDICTED: monocarboxylate transporter 10
RS000720	GO:0022892	substrate-specific transporter activity	MF	-1.414180497	1.484024302	-1.819002501	5.701350612	100.1058089	1.47484E-21	4.12758E-20	aquaporin
RS012045	GO:0022892	substrate-specific transporter activity	MF	-2.417547613	-3.846942398	-2.948472949	3.138267013	95.12499238	1.73561E-20	4.58246E-19	PREDICTED: sodium-independent sulfate anion transporter
RS002200	GO:0022892	substrate-specific transporter activity	MF	-2.534854462	-2.965618885	0.446701011	4.187537984	94.94596227	1.89639E-20	4.97566E-19	PREDICTED: ATP-binding cassette sub-family G member 4
RS009300	GO:0022892	substrate-specific transporter activity	MF	-0.334428722	2.777656488	1.996146097	5.84981916	85.14092159	2.42051E-18	5.44355E-17	PREDICTED: organic cation transporter protein-like
RS014732	GO:0022892	substrate-specific transporter activity	MF	-2.171879817	-1.24728548	0.09932889	5.367265273	82.18961662	1.04062E-17	2.21004E-16	PREDICTED: solute carrier organic anion transporter family member 2A1
RS015390	GO:0022892	substrate-specific transporter activity	MF	-1.260588417	-0.88728058	1.174441311	5.643186003	81.54774944	1.42892E-17	3.01438E-16	PREDICTED: sodium-independent sulfate anion transporter
RS014579	GO:0022892	substrate-specific transporter activity	MF	-1.833927302	-1.637670215	-2.03241566	4.946947748	80.73878787	2.13088E-17	4.42843E-16	PREDICTED: transient receptor potential cation channel protein painless
RS011973	GO:0022892	substrate-specific transporter activity	MF	-0.861945869	1.222753935	-0.568825813	4.359430432	80.33905671	2.59602E-17	5.34219E-16	PREDICTED: zinc transporter 1
RS004424	GO:0022892	substrate-specific transporter activity	MF	-0.154114067	2.332271731	0.36051487	5.646491566	77.24068683	1.19889E-16	2.38151E-15	PREDICTED: TWiK family of potassium channels protein 7
RS002366	GO:0022892	substrate-specific transporter activity	MF	-2.013469227	2.285528889	0.940362283	2.009763796	76.37983445	1.83374E-16	3.57494E-15	PREDICTED: sodium-dependent serotonin transporter
RS011988	GO:0022892	substrate-specific transporter activity	MF	-1.045428446	0.08149937	1.776943069	7.507925055	72.44172426	1.28023E-15	2.31656E-14	PREDICTED: aquaporin-12
RS006315	GO:0022892	substrate-specific transporter activity	MF	-2.445857294	-4.975199992	0.147442902	3.069628072	71.7469443	1.80352E-15	3.24017E-14	PREDICTED: bumetanide-sensitive sodium-(potassium)-chloride cotransporter
RS008278	GO:0022892	substrate-specific transporter activity	MF	0.642268679	2.750452439	1.283258456	3.961355993	71.14168969	2.43087E-15	4.34246E-14	PREDICTED: potassium voltage-gated channel protein Shaw-like
RS006271	GO:0022892	substrate-specific transporter activity	MF	1.985922002	1.711587011	-1.594562947	3.353974541	68.37067995	9.52991E-15	1.62849E-13	PREDICTED: organic cation transporter 1
RS008443	GO:0022892	substrate-specific transporter activity	MF	-1.979883882	-8.057075206	-1.206380805	2.046366836	67.84965365	1.23201E-14	2.07155E-13	PREDICTED: facilitated trehalose transporter Tret1-2 homolog
RS001006	GO:0022892	substrate-specific transporter activity	MF	-1.308055843	0.748595123	2.011362686	7.151837524	64.31436805	7.03086E-14	1.12512E-12	PREDICTED: sodium-dependent nutrient amino acid transporter 1-like
RS007831	GO:0022892	substrate-specific transporter activity	MF	-0.285689079	2.23184291	-0.54998832	3.347851609	60.46667692	4.67238E-13	7.00523E-12	PREDICTED: sodium- and chloride-dependent GABA transporter ine-like
RS011073	GO:0022892	substrate-specific transporter activity	MF	1.681239408	1.71053558	-0.220987815	5.116873023	56.90571814	2.69177E-12	3.80474E-11	PREDICTED: vitellogenin-like
RS010878	GO:0022892	substrate-specific transporter activity	MF	-0.202147251	0.219397642	1.259334773	5.8954575	54.42606082	9.10217E-12	1.23527E-10	PREDICTED: band 3 anion transport protein
RS008617	GO:0022892	substrate-specific transporter activity	MF	-1.758654905	-1.933395291	0.119722157	4.240860888	52.46640405	2.38231E-11	3.09308E-10	PREDICTED: proton-coupled amino acid transporter 1
RS006021	GO:0022892	substrate-specific transporter activity	MF	-1.978389488	-0.987147201	0.23457041	5.767022167	52.1310105	2.80858E-11	3.63527E-10	PREDICTED: cationic amino acid transporter 3
RS000134	GO:0022892	substrate-specific transporter activity	MF	-2.137136524	0.447963208	0.262936854	3.43282157	50.22811762	7.14362E-11	8.98769E-10	PREDICTED: putative ammonium transporter 1
RS012141	GO:0022892	substrate-specific transporter activity	MF	-0.795199967	0.272005658	1.157168	4.936082578	49.75842476	8.99389E-11	1.12594E-09	PREDICTED: UNC93-like protein MFSD11
RS008254	GO:0022892	substrate-specific transporter activity	MF	-1.84400551	0.769806662	1.172103168	3.667348911	48.7792472	1.45353E-10	1.78592E-09	PREDICTED: facilitated trehalose transporter Tret1-like
RS009380	GO:0022892	substrate-specific transporter activity	MF	1.181487238	0.230316163	-1.011984835	4.090504935	45.23692145	8.23976E-10	9.50289E-09	PREDICTED: b(0,+)-type amino acid transporter 1-like
RS006648	GO:0022892	substrate-specific transporter activity	MF	-1.472287059	-1.627468546	0.001448714	3.845832603	45.04057983	9.07077E-10	1.03664E-08	PREDICTED: voltage-dependent L-type calcium channel subunit beta-2
RS006270	GO:0022892	substrate-specific transporter activity	MF	-0.625556145	-0.022131593	2.665286326	2.775447041	44.95816183	9.44409E-10	1.07832E-08	PREDICTED: organic cation transporter 1
RS011778	GO:0022892	substrate-specific transporter activity	MF	-1.482690204	-0.163981472	-1.627972092	5.097898358	44.03120475	1.48632E-09	1.66834E-08	PREDICTED: facilitated trehalose transporter Tret1-2 homolog
RS007743	GO:0022892	substrate-specific transporter activity	MF	-1.804323011	-1.669176003	-0.470211701	5.80929799	43.79813802	1.66579E-09	1.85163E-08	PREDICTED: probable phospholipid-transporting ATPase IF
RS005640	GO:0022892	substrate-specific transporter activity	MF	0.969003673	1.718434974	-0.876644168	4.401662752	43.66119041	1.78117E-09	1.9764E-08	PREDICTED: glycine receptor subunit alpha-4
RS009578	GO:0022892	substrate-specific transporter activity	MF	-2.77838182	-2.247321578	-2.454512864	4.019688809	42.39569844	3.30671E-09	3.59937E-08	PREDICTED: solute carrier family 28 member 3
RS001007	GO:0022892	substrate-specific transporter activity	MF	-0.057453873	2.550863666	4.593751253	0.34449975	41.36132174	5.4813E-09	5.84023E-08	PREDICTED: sodium-dependent nutrient amino acid transporter 1-like
RS010452	GO:0022892	substrate-specific transporter activity	MF	-1.264493454	-3.411377807	-0.656775569	3.802175748	40.96006363	6.66799E-09	7.05094E-08	PREDICTED: transient receptor potential cation channel protein painless
RS014047	GO:0022892	substrate-specific transporter activity	MF	-0.592092988	1.748032789	5.355436168	1.817327481	40.93864928	6.73809E-09	7.11311E-08	PREDICTED: zinc transporter ZIP1-like
RS005851	GO:0022892	substrate-specific transporter activity	MF	0.526397294	1.41413322	1.673681755	7.829535198	40.74316203	7.413E-09	7.77347E-08	PREDICTED: proton-coupled amino acid transporter 4
RS012191	GO:0022892	substrate-specific transporter activity	MF	-0.827958312	0.960594434	-0.015691014	7.391207833	39.39645508	1.43044E-08	1.45634E-07	PREDICTED: V-type proton ATPase 116 kDa subunit a
RS003335	GO:0022892	substrate-specific transporter activity	MF	0.43066806	0.052969002	-1.65671712	5.277276724	38.32248305	2.41526E-08	2.37825E-07	PREDICTED: apolipophorins-like
RS009711	GO:0022892	substrate-specific transporter activity	MF	0.862276886	1.272132135	1.421293308	6.056371062	36.7986326	5.07562E-08	4.85732E-07	PREDICTED: tropinone reductase 2-like
RS013235	GO:0022892	substrate-specific transporter activity	MF	-2.068035818	-1.64402774	1.426351822	0.894091033	34.98409736	1.22771E-07	1.1286E-06	PREDICTED: glutamate receptor 1-like
RS012786	GO:0022892	substrate-specific transporter activity	MF	-1.725772589	-2.922030511	-0.315113572	3.189291251	34.971992	1.23496E-07	1.13444E-06	PREDICTED: facilitated trehalose transporter Tret1-2 homolog
RS004248	GO:0022892	substrate-specific transporter activity	MF	-2.357800174	-1.138542703	0.799750155	3.47254249	34.60029849	1.47968E-07	1.34356E-06	PREDICTED: facilitated trehalose transporter Tret1-like
RS009116	GO:0022892	substrate-specific transporter activity	MF	-2.774708917	-1.965598127	1.306984186	1.044379091	34.54483465	1.52014E-07	1.3783E-06	PREDICTED: urea transporter 1-like
RS000365	GO:0022892	substrate-specific transporter activity	MF	-2.043121889	-2.26896537	0.189661525	11.00673959	34.00204383	1.97922E-07	1.77664E-06	PREDICTED: calcium-transporting ATPase sarcoplasmic/endoplasmic reticulum type
RS007032	GO:0022892	substrate-specific transporter activity	MF	-1.972036202	-0.832866298	0.156731051	5.195017285	33.86656984	2.11394E-07	1.89487E-06	PREDICTED: ammonium transporter Rh type A
RS001722	GO:0022892	substrate-specific transporter activity	MF	-0.334283753	-2.198611554	-0.278419111	3.485639997	32.69185314	3.74056E-07	3.27598E-06	PREDICTED: solute carrier family 41 member 1-like
RS007693	GO:0022892	substrate-specific transporter activity	MF	-0.242378739	-0.621851837	1.493665703	5.076496585	32.00947491	5.20946E-07	4.51534E-06	PREDICTED: zinc transporter ZIP14
RS006814	GO:0022892	substrate-specific transporter activity	MF	-0.514796382	1.014347124	0.975336891	4.041760153	30.97725487	8.59474E-07	7.23543E-06	PREDICTED: large neutral amino acids transporter small subunit 2
RS006320	GO:0022892	substrate-specific transporter activity	MF	1.668458777	1.549661536	0.932473469	6.183206129	30.51118393	1.07731E-06	8.96741E-06	PREDICTED: bumetanide-sensitive sodium-(potassium)-chloride cotransporter
RS000193	GO:0022892	substrate-specific transporter activity	MF	-0.612906575	0.552707825	0.864925878	6.395375097	30.19397061	1.25629E-06	1.03772E-05	PREDICTED: b(0,+)-type amino acid transporter 1
RS001411	GO:0022892	substrate-specific transporter activity	MF	-1.439234611	-1.190473147	0.602093209	4.878972892	30.16795384	1.27223E-06	1.04928E-05	PREDICTED: globin
RS005497	GO:0022892	substrate-specific transporter activity	MF	1.170662464	-1.020287306	-0.883108523	1.992265709	29.45354334	1.79809E-06	1.45254E-05	PREDICTED: low-density lipoprotein receptor-related protein 2
RS014898	GO:0022892	substrate-specific transporter activity	MF	-0.634960839	-1.801610032	0.576713947	3.693735802	29.21635917	2.01683E-06	1.61783E-05	PREDICTED: ATP-binding cassette sub-family G member 1
RS000996	GO:0022892	substrate-specific transporter activity	MF	-1.508231266	-0.156640833	0.876498273	2.831732428	29.09435446	2.13949E-06	1.70644E-05	PREDICTED: zinc transporter ZIP3-like
RS005491	GO:0022892	substrate-specific transporter activity	MF	-0.937465516	-1.630388798	-0.209059704	4.107611972	28.88192352	2.37108E-06	1.88281E-05	PREDICTED: voltage-gated potassium channel subunit beta-2
RS009579	GO:0022892	substrate-specific transporter activity	MF	-0.361377028	0.937501921	0.004604885	5.160969196	28.85056945	2.40732E-06	1.91039E-05	PREDICTED: solute carrier family 28 member 3-like
RS011850	GO:0022892	substrate-specific transporter activity	MF	-0.767278606	-0.313949384	0.713484854	8.083358391	28.67013092	2.62688E-06	2.07027E-05	PREDICTED: trimeric intracellular cation channel type B
RS008885	GO:0022892	substrate-specific transporter activity	MF	-1.592550754	-2.358375405	-0.949039702	4.983900477	28.41800515	2.96751E-06	2.32998E-05	PREDICTED: facilitated trehalose transporter Tret1-2 homolog
RS010090	GO:0022892	substrate-specific transporter activity	MF	0.323234855	1.546465547	-0.132352818	2.938456182	28.16134763	3.35955E-06	2.60531E-05	PREDICTED: cyclic nucleotide-gated cation channel subunit A-like
RS008631	GO:0022892	substrate-specific transporter activity	MF	-0.45372484	0.018443887	2.172529191	1.173123173	28.08258513	3.48992E-06	2.69976E-05	PREDICTED: glutamate receptor ionotropic, delta-1-like
RS004528	GO:0022892	substrate-specific transporter activity	MF	0.600875429	1.093315864	0.014760964	6.884013552	27.64527681	4.31111E-06	3.29855E-05	PREDICTED: microsomal triglyceride transfer protein large subunit
RS008887	GO:0022892	substrate-specific transporter activity	MF	-0.760329305	-4.339267866	-1.430676324	3.320036492	27.63642804	4.32958E-06	3.3078E-05	PREDICTED: facilitated trehalose transporter Tret1-like
RS006269	GO:0022892	substrate-specific transporter activity	MF	-1.78180142	-1.191554952	-0.830372469	4.550094908	27.39894348	4.85584E-06	3.68178E-05	PREDICTED: organic cation transporter 1
RS012590	GO:0022892	substrate-specific transporter activity	MF	-0.22021637	0.861437679	0.641734274	6.761709937	27.36974453	4.9248E-06	3.72958E-05	PREDICTED: high affinity copper uptake protein 1
RS012741	GO:0022892	substrate-specific transporter activity	MF	-1.060191661	0.585439948	0.026171952	4.628484614	26.6687965	6.90781E-06	5.10845E-05	PREDICTED: organic cation transporter protein
RS000895	GO:0022892	substrate-specific transporter activity	MF	1.014348253	1.385977981	0.68384576	7.930053973	26.65746374	6.94569E-06	5.13345E-05	PREDICTED: translocon-associated protein subunit beta
RS000719	GO:0022892	substrate-specific transporter activity	MF	-0.394780773	-1.247672358	-1.502851389	6.140116665	25.60119706	1.15586E-05	8.29929E-05	PREDICTED: aquaporin AQPAn.G
RS013310	GO:0022892	substrate-specific transporter activity	MF	-1.594173173	0.30942411	0.502900611	3.784418211	25.32704004	1.31907E-05	9.38549E-05	PREDICTED: sodium- and chloride-dependent transporter XTRP3
RS003638	GO:0022892	substrate-specific transporter activity	MF	-0.69625377	-0.738048152	0.351114728	5.7591544	24.53074045	1.93528E-05	0.00013355	PREDICTED: ATP-binding cassette sub-family G member 4
RS004915	GO:0022892	substrate-specific transporter activity	MF	-0.614675035	-1.44127198	-1.283158317	7.861317536	24.36804538	2.09282E-05	0.000143713	Apolipophorin
RS003139	GO:0022892	substrate-specific transporter activity	MF	-0.462942241	-0.484276182	0.670801183	4.994154039	24.0689115	2.4166E-05	0.000164334	PREDICTED: zinc transporter 1
RS007526	GO:0022892	substrate-specific transporter activity	MF	-1.223662153	-0.387211114	-0.507976241	5.27455292	24.05874887	2.42843E-05	0.00016505	PREDICTED: phospholipid-transporting ATPase ID
RS006319	GO:0022892	substrate-specific transporter activity	MF	-1.264071762	-1.378688832	-0.296664377	4.499770473	23.99784591	2.50059E-05	0.000169587	PREDICTED: bumetanide-sensitive sodium-(potassium)-chloride cotransporter
RS008934	GO:0022892	substrate-specific transporter activity	MF	-2.249974024	-1.610242152	-0.986553809	2.596021099	23.90344612	2.61667E-05	0.000177078	ionotropic receptor 25a
RS010443	GO:0022892	substrate-specific transporter activity	MF	-2.227753564	-2.234532899	-0.56368196	2.617137088	23.82549146	2.71656E-05	0.000183248	PREDICTED: pickpocket protein 28-like
RS014152	GO:0022892	substrate-specific transporter activity	MF	-1.430499389	-1.354570713	-0.671668718	4.505777329	23.40073256	3.33153E-05	0.000221644	PREDICTED: potassium channel subfamily K member 1-like
RS012504	GO:0022892	substrate-specific transporter activity	MF	1.701019285	0.103598753	-0.952358557	2.60795793	23.14266371	3.77102E-05	0.000248475	PREDICTED: zinc transporter ZIP3
RS014340	GO:0022892	substrate-specific transporter activity	MF	-0.92887959	-0.092715464	0.219765138	5.620832256	22.82606695	4.38983E-05	0.00028483	PREDICTED: ileal sodium/bile acid cotransporter-like
RS011507	GO:0022892	substrate-specific transporter activity	MF	0.081672207	0.439418944	1.152509484	5.150989282	22.16598652	6.02438E-05	0.000382801	PREDICTED: sialin
RS005054	GO:0022892	substrate-specific transporter activity	MF	-0.882564902	-1.772840386	-2.09465887	2.914764324	21.56658479	8.02786E-05	0.000498781	PREDICTED: ETS domain-containing protein Elk-3-like
RS012462	GO:0022892	substrate-specific transporter activity	MF	-1.887439376	-1.068323927	-1.822700455	2.935725927	21.16526065	9.72757E-05	0.000593839	PREDICTED: synaptic vesicle glycoprotein 2B
RS012046	GO:0022892	substrate-specific transporter activity	MF	-0.044612109	1.318321788	-0.182662099	3.873303092	21.0021542	0.000105168	0.000636465	PREDICTED: sodium-independent sulfate anion transporter-like
RS015154	GO:0022892	substrate-specific transporter activity	MF	0.77118859	1.275597924	0.795023023	8.092063536	20.820501	0.00011471	0.000690232	PREDICTED: protein transport protein Sec61 subunit gamma
RS015395	GO:0022892	substrate-specific transporter activity	MF	-1.227128143	-0.999149743	-0.38673491	5.789022217	20.66182653	0.000123747	0.000741733	PREDICTED: ryanodine receptor 44F
RS010854	GO:0022892	substrate-specific transporter activity	MF	-0.464601417	0.421962906	0.417617417	6.7210824	20.51691341	0.000132619	0.000789321	PREDICTED: V-type proton ATPase subunit e 2-like
RS006273	GO:0022892	substrate-specific transporter activity	MF	-0.841668922	-0.780206767	0.412687786	4.250127088	20.41178555	0.000139449	0.000826843	PREDICTED: organic cation transporter 1
RS014287	GO:0022892	substrate-specific transporter activity	MF	-0.825809928	1.486619083	1.592270521	1.389357496	20.39684624	0.000140447	0.000831501	PREDICTED: sialin
RS007340	GO:0022892	substrate-specific transporter activity	MF	1.72289566	2.228109689	-0.488523344	0.853368064	20.24210443	0.000151218	0.000888679	PREDICTED: mitochondrial sodium/hydrogen exchanger 9B2
RS004393	GO:0022892	substrate-specific transporter activity	MF	-1.227790861	-2.264108903	-0.817189459	2.289519889	20.23673107	0.000151606	0.000890546	PREDICTED: putative tricarboxylate transport protein, mitochondrial
RS015388	GO:0022892	substrate-specific transporter activity	MF	-2.602050655	-3.074067005	-0.457367858	0.547921075	20.16611512	0.000156804	0.000918937	PREDICTED: glutamate receptor ionotropic, delta-1
RS000135	GO:0022892	substrate-specific transporter activity	MF	-0.631745801	-0.940404648	0.237026994	4.32458574	20.11793543	0.000160452	0.000937697	PREDICTED: putative ammonium transporter 1

RS006499	GO:0022892	substrate-specific transporter activity	MF	-1.047637087	0.301924919	0.728056025	3.319116729	19.94751042	0.000174047	0.001009187	PREDICTED: transient receptor potential cation channel subfamily V member 5
RS015396	GO:0022892	substrate-specific transporter activity	MF	-1.256182982	-1.010805264	-0.293857127	6.048626436	19.73517564	0.0001926	0.00110506	PREDICTED: ryanodine receptor 44F
RS008662	GO:0022892	substrate-specific transporter activity	MF	-0.323694319	-0.887991823	-0.04413226	5.702043181	19.66407708	0.000199242	0.001138501	PREDICTED: transient receptor potential cation channel subfamily V member 5
RS001822	GO:0022892	substrate-specific transporter activity	MF	0.374152151	-0.235220993	-0.559194155	6.339069228	19.66077025	0.000199557	0.00113978	PREDICTED: exportin-2
RS100005	GO:0022892	substrate-specific transporter activity	MF	-2.639688266	-3.107895143	0.367307699	1.207651888	19.4971158	0.00021575	0.001225045	beta-glucosidase
RS005651	GO:0022892	substrate-specific transporter activity	MF	-0.219646792	-1.283329628	-0.275521678	5.060327004	19.22158667	0.000246019	0.001380116	PREDICTED: transient-receptor-potential-like protein
RS012441	GO:0022892	substrate-specific transporter activity	MF	-0.795629624	0.983276815	1.634723389	1.23435714	19.18509754	0.000250332	0.001401285	AGAP004474
RS006140	GO:0022892	substrate-specific transporter activity	MF	0.033292599	1.273516036	1.00013418	2.720807394	19.17927372	0.000251027	0.00140446	PREDICTED: potassium channel subfamily K member 18
RS005838	GO:0022892	substrate-specific transporter activity	MF	-1.272361428	-3.114064861	-3.237130599	0.429125999	19.0443607	0.000267685	0.001488399	PREDICTED: glutamate receptor U1-like
RS001336	GO:0022892	substrate-specific transporter activity	MF	0.412169289	1.116247727	1.639275177	2.741362032	18.96980298	0.000277358	0.001536754	PREDICTED: glutamate receptor U1-like
RS007614	GO:0022892	substrate-specific transporter activity	MF	0.699165628	-0.052782673	-0.817546641	5.472681929	18.94140065	0.000281133	0.001554478	PREDICTED: facilitated trehalose transporter Tret1-like
RS007694	GO:0022892	substrate-specific transporter activity	MF	-1.402205119	-1.429007484	-0.154037672	3.235518975	18.72862507	0.000311092	0.001703432	PREDICTED: proton myo-inositol cotransporter-like
RS009272	GO:0022892	substrate-specific transporter activity	MF	-0.152985688	-1.205701524	-0.53386679	4.590610282	18.71946093	0.000312451	0.001710132	PREDICTED: mitochondrial sodium/hydrogen exchanger 9B2
RS011525	GO:0022892	substrate-specific transporter activity	MF	-0.806138244	0.427082827	0.274818707	7.810153084	18.69931455	0.000315461	0.001724354	PREDICTED: sodium/potassium-transporting ATPase subunit beta-2
RS009604	GO:0022892	substrate-specific transporter activity	MF	-1.648345631	0.550771769	0.526514629	2.368500525	18.41833332	0.000360561	0.001945804	PREDICTED: sodium-dependent noradrenaline transporter
RS009304	GO:0022892	substrate-specific transporter activity	MF	-2.056162023	-1.492683824	-1.148437663	1.440266038	18.0307206	0.000433479	0.002294761	PREDICTED: glutamate receptor ionotropic, delta-1-like
RS014410	GO:0022892	substrate-specific transporter activity	MF	-0.538555444	-0.069436224	0.424307658	5.574699261	17.86202393	0.00046963	0.002466436	PREDICTED: sodium- and chloride-dependent neutral and basic amino acid transporter B(0+)
RS000837	GO:0022892	substrate-specific transporter activity	MF	-1.260815808	-0.582381283	0.014148923	5.012164404	17.85146996	0.000471988	0.002476758	PREDICTED: excitatory amino acid transporter 1-like
RS007085	GO:0022892	substrate-specific transporter activity	MF	-2.123520843	-1.442400071	-1.654436852	1.159522622	17.26753428	0.000622612	0.00317714	PREDICTED: cyclic nucleotide-gated cation channel alpha-3
RS000849	GO:0022892	substrate-specific transporter activity	MF	-0.753175709	-1.571391853	-1.058453667	2.708513766	17.06922787	0.000683946	0.003464852	PREDICTED: monocarboxylate transporter 9-like
RS012432	GO:0022892	substrate-specific transporter activity	MF	-1.318193047	-1.41640057	-1.093486391	3.821570882	17.02394884	0.000698772	0.003535692	PREDICTED: acetylcholine receptor subunit alpha-type acr-16-like
RS013332	GO:0022892	substrate-specific transporter activity	MF	-1.915754341	-0.761067399	-0.092972296	1.96508851	16.98236489	0.000712669	0.003598779	PREDICTED: potassium channel subfamily T member 2
RS004044	GO:0022892	substrate-specific transporter activity	MF	-0.216859326	0.637026253	1.004892576	2.871056733	16.9549321	0.000721987	0.003638536	PREDICTED: glutamate-gated chloride channel
RS002706	GO:0022892	substrate-specific transporter activity	MF	-0.740169025	-1.766156812	-2.037640472	11.03901016	16.70844226	0.00081133	0.004054717	PREDICTED: apolipophorins
RS012047	GO:0022892	substrate-specific transporter activity	MF	-1.033691566	-1.204387105	-0.573210977	3.717195265	16.53395031	0.000881135	0.004370625	PREDICTED: sodium-independent sulfate anion transporter-like
RS010364	GO:0022892	substrate-specific transporter activity	MF	-0.052944401	0.405459181	0.815688442	7.091949691	16.43172938	0.000924767	0.004565472	PREDICTED: cytochrome c oxidase subunit 7A2, mitochondrial
RS002855	GO:0022892	substrate-specific transporter activity	MF	-1.74839573	-1.535258414	-0.16366084	1.79539144	16.39328726	0.000941725	0.00463647	PREDICTED: glycine receptor subunit alpha-3
RS000927	GO:0022892	substrate-specific transporter activity	MF	-2.570527298	-2.804707147	-0.755163456	0.667619183	16.35029216	0.000961058	0.004715063	PREDICTED: b(0,+)-type amino acid transporter 1-like
RS009094	GO:0022892	substrate-specific transporter activity	MF	0.810509918	0.868482563	0.707370764	6.315762678	16.22341499	0.001020437	0.004986955	PREDICTED: mitochondrial import inner membrane translocase subunit Tim17-B-like
RS003544	GO:0022892	substrate-specific transporter activity	MF	-0.410771227	-0.348918681	1.010423069	3.516674428	16.0029682	0.001132396	0.005455777	PREDICTED: glutamate receptor ionotropic, kainate 2
RS006141	GO:0022892	substrate-specific transporter activity	MF	0.186642093	0.509454722	0.888482209	5.536921472	15.98603197	0.001141486	0.005494665	Potassium channel subfamily K member 9
RS003914	GO:0022892	substrate-specific transporter activity	MF	-0.408304954	-1.308021582	-1.113950825	3.842131166	15.96402321	0.001153407	0.005540047	PREDICTED: facilitated trehalose transporter Tret1-like
RS013870	GO:0022892	substrate-specific transporter activity	MF	-1.185159015	-0.789079424	-0.942978819	4.024142004	15.86729932	0.001207277	0.005765813	PREDICTED: sodium- and chloride-dependent glycine transporter 1-like
RS013164	GO:0022892	substrate-specific transporter activity	MF	-0.720894807	-1.573394185	-0.567112816	2.35012097	15.85215695	0.001215934	0.005800558	PREDICTED: sodium/hydrogen exchanger 3
RS014761	GO:0022892	substrate-specific transporter activity	MF	0.850386459	0.827647699	0.741742978	6.600879234	15.79201097	0.001250932	0.005936034	PREDICTED: mitochondrial import receptor subunit TOM40 homolog 1
RS012306	GO:0022892	substrate-specific transporter activity	MF	-1.171605666	-1.08070317	-0.099574832	5.667246046	15.66500563	0.001328153	0.006252994	PREDICTED: ABC transporter G family member 23
RS012806	GO:0022892	substrate-specific transporter activity	MF	-0.671548237	-0.771532863	-0.831977584	6.320380877	15.5512679	0.001401314	0.006555774	PREDICTED: glutamate [NMDA] receptor subunit 1
RS004534	GO:0022892	substrate-specific transporter activity	MF	-0.580382142	2.035273319	0.497901415	1.025113205	15.51468926	0.001425682	0.006654944	PREDICTED: sodium channel protein Nach-like
RS000928	GO:0022892	substrate-specific transporter activity	MF	-2.818387407	-2.387215456	-1.195316578	0.324906115	15.29049888	0.001584497	0.007309578	B(0,+)-type amino acid transporter 1
RS006610	GO:0022892	substrate-specific transporter activity	MF	0.2664942	0.92285009	0.741592285	5.324734931	15.2782684	0.001593649	0.007342643	PREDICTED: zinc transporter 7
RS008896	GO:0022892	substrate-specific transporter activity	MF	-1.274733506	-1.006034401	-0.767037953	2.62762816	15.13459455	0.001705167	0.007799012	PREDICTED: mitoferrin-1
RS006757	GO:0022892	substrate-specific transporter activity	MF	-0.233100515	0.411940029	0.749443033	4.26814335	14.98825356	0.001826715	0.008260555	PREDICTED: cell death-inducing p53-target protein 1
RS009126	GO:0022892	substrate-specific transporter activity	MF	0.004334845	0.550905396	0.691970165	7.068681097	14.88906784	0.001913944	0.008599435	PREDICTED: V-type proton ATPase subunit F
RS006318	GO:0022892	substrate-specific transporter activity	MF	-3.618949223	-2.702749807	-1.072386578	0.467850565	14.73641507	0.002056334	0.009147816	PREDICTED: bumetanide-sensitive sodium-(potassium)-chloride cotransporter
RS005708	GO:0022892	substrate-specific transporter activity	MF	-2.57707626	-0.767810406	-0.121337824	1.208165742	14.61680361	0.002175202	0.009598631	PREDICTED: sodium- and chloride-dependent glycine transporter 1-like
RS013194	GO:0022892	substrate-specific transporter activity	MF	-0.402235939	1.468418978	0.620702468	1.800456379	14.57025873	0.002223278	0.009779937	PREDICTED: gamma-aminobutyric acid receptor alpha-like
RS008446	GO:0022892	substrate-specific transporter activity	MF	0.363470434	-0.27703378	-1.408383647	4.990252537	14.55183725	0.002242595	0.009858024	PREDICTED: facilitated trehalose transporter Tret1-2 homolog
RS008491	GO:0022892	substrate-specific transporter activity	MF	-0.83498967	-1.675590127	-1.132486269	2.878684988	14.45091871	0.00235141	0.010286093	PREDICTED: callisulfakinin
RS012689	GO:0022892	substrate-specific transporter activity	MF	-0.335162446	-0.922302748	-1.067051914	4.164281375	14.43562454	0.002368352	0.010353914	PREDICTED: solute carrier family 41 member 1-like
RS008723	GO:0022892	substrate-specific transporter activity	MF	0.765724899	0.492155106	-0.726953396	2.604372289	14.25219643	0.002581214	0.011140444	PREDICTED: excitatory amino acid transporter
RS005190	GO:0022892	substrate-specific transporter activity	MF	-0.561761317	0.20318587	0.563166122	6.506172496	14.19948267	0.002645822	0.011376401	PREDICTED: calcium-activated potassium channel slowpoke
RS013212	GO:0022892	substrate-specific transporter activity	MF	-0.878244991	-0.110123051	0.018139925	7.487946573	14.16068921	0.002694389	0.011553674	PREDICTED: V-type proton ATPase subunit B
RS011379	GO:0022892	substrate-specific transporter activity	MF	-0.945895165	-0.137739549	0.02413553	6.197611827	14.11352967	0.002754622	0.01177587	PREDICTED: chloride intracellular channel exc-4
RS014046	GO:0022892	substrate-specific transporter activity	MF	-0.827652517	0.123541367	0.39500677	4.371003694	13.78924417	0.003206526	0.0134386	PREDICTED: zinc transporter ZIP1-like
RS006255	GO:0022892	substrate-specific transporter activity	MF	-0.565848028	-0.409978879	0.214379631	6.839035823	13.48918712	0.003689736	0.015225603	PREDICTED: copper-transporting ATPase 1
RS012010	GO:0022892	substrate-specific transporter activity	MF	-0.658055692	-0.163236513	0.25753802	6.662027841	13.45566221	0.003748014	0.01543574	PREDICTED: sodium-dependent phosphate transporter 1-B-like
RS010731	GO:0022892	substrate-specific transporter activity	MF	-0.395182325	-0.639006133	-0.675231861	6.20607259	13.34648856	0.003944186	0.016153848	PREDICTED: V-type proton ATPase 116 kDa subunit a
RS006343	GO:0022892	substrate-specific transporter activity	MF	0.700005517	0.678494058	0.385858572	5.661988764	13.33113205	0.003972583	0.016264862	PREDICTED: zinc transporter 6
RS010905	GO:0022892	substrate-specific transporter activity	MF	-0.573248945	-0.768712677	-0.837001645	4.71486708	13.23543162	0.00415417	0.016871208	PREDICTED: gamma-aminobutyric acid receptor subunit beta
RS009404	GO:0022892	substrate-specific transporter activity	MF	0.443476694	0.123699806	-0.392860437	6.921259727	13.00862403	0.004617993	0.018439758	PREDICTED: AP-2 complex subunit sigma
RS014000	GO:0022892	substrate-specific transporter activity	MF	0.486986842	0.493065666	-0.511287156	7.326747337	12.99389808	0.00464982	0.018525729	TWiK family of potassium channels protein 18
RS007615	GO:0022892	substrate-specific transporter activity	MF	0.842199769	0.08001472	-0.354074907	7.382223287	12.96671251	0.004709147	0.018738389	PREDICTED: facilitated trehalose transporter Tret1-like
RS009863	GO:0022892	substrate-specific transporter activity	MF	0.247967949	1.994626146	0.840783905	0.741237487	12.85234652	0.004967042	0.019634316	PREDICTED: sodium- and chloride-dependent glycine transporter 1-like
RS005166	GO:0022892	substrate-specific transporter activity	MF	0.797745292	0.959053152	1.137980998	8.88871257	12.83598972	0.005005051	0.019744379	PREDICTED: nascent polypeptide-associated complex subunit alpha
RS005241	GO:0022892	substrate-specific transporter activity	MF	-1.24648029	-0.946331544	-0.692073388	4.41212653	12.5745733	0.005653054	0.021933014	PREDICTED: high-affinity choline transporter 1
RS006299	GO:0022892	substrate-specific transporter activity	MF	0.929966764	0.90845916	-0.169322471	3.276482954	12.53656321	0.005753939	0.022276393	PREDICTED: facilitated trehalose transporter Tret1-like
RS008051	GO:0022892	substrate-specific transporter activity	MF	-0.405673123	-1.320265794	-1.066020494	4.63196021	12.49923358	0.00585475	0.022555742	PREDICTED: transient receptor potential channel pyrexia
RS000227	GO:0022892	substrate-specific transporter activity	MF	-1.32952285	-0.75353739	-1.12184367	1.981758767	12.38833226	0.00616466	0.023605269	PREDICTED: facilitated trehalose transporter Tret1-2 homolog
RS012250	GO:0022892	substrate-specific transporter activity	MF	-1.126562781	-0.381786444	0.539034757	1.985904525	12.14501042	0.00690257	0.026042829	PREDICTED: organic cation transporter protein-like
RS002985	GO:0022892	substrate-specific transporter activity	MF	-0.571114377	-0.476136182	-0.859409917	5.548946497	12.07709757	0.007123669	0.026732864	PREDICTED: Y+L amino acid transporter 2
RS008215	GO:0022892	substrate-specific transporter activity	MF	0.162922308	0.46909669	0.671411093	6.515514341	12.06788131	0.007154208	0.026831478	PREDICTED: mitochondrial import receptor subunit TOM22 homolog
RS003288	GO:0022892	substrate-specific transporter activity	MF	-0.468141457	-0.663116971	0.331112622	4.523147937	12.02307867	0.007304519	0.027292266	PREDICTED: sodium- and chloride-dependent GABA transporter ine
RS003997	GO:0022892	substrate-specific transporter activity	MF	-1.222850277	-0.635965438	-0.411407393	6.247970682	11.97127476	0.007482212	0.027852345	PREDICTED: ABC transporter G family member 23
RS011468	GO:0022892	substrate-specific transporter activity	MF	-0.318737056	-0.110014844	-1.568608212	1.963272184	11.9167036	0.007674014	0.028433815	PREDICTED: solute carrier family 35 member F6
RS000866	GO:0022892	substrate-specific transporter activity	MF	-0.851954648	-0.389874807	-0.092110043	7.989972168	11.90615845	0.007711634	0.028547895	PREDICTED: sodium/potassium-transporting ATPase subunit alpha
RS015096	GO:0022892	substrate-specific transporter activity	MF	-0.857980934	-0.446788274	-0.194471442	5.697782265	11.71197794	0.008437836	0.030900291	PREDICTED: V-type proton ATPase subunit D
RS009360	GO:0022892	substrate-specific transporter activity	MF	-0.052778092	-0.516301747	0.304429459	6.031942967	11.69868265	0.008489957	0.031037017	PREDICTED: sodium-dependent nutrient amino acid transporter 1
RS008458	GO:0022892	substrate-specific transporter activity	MF	-1.182823715	-0.196910535	-0.390818396	8.429616967	11.6884148	0.008530428	0.031131399	PREDICTED: probable cation-transporting ATPase 13A3
RS004997	GO:0022892	substrate-specific transporter activity	MF	-0.851696275	-1.530670443	-0.345032758	2.982640462	11.64463017	0.008705154	0.031658304	PREDICTED: kinesin-like protein KIF17
RS008898	GO:0022892	substrate-specific transporter activity	MF	-0.172960297	-0.478698026	-0.774628888	4.936093792	11.53878925	0.009142244	0.033009582	PREDICTED: sideroflexin-1
RS002295	GO:0022892	substrate-specific transporter activity	MF	-0.760632417	-0.881718429	-1.336627404	7.31207469	11.50237509	0.009297576	0.033436228	PREDICTED: sodium- and chloride-dependent GABA transporter 1-like
RS010641	GO:0022892	substrate-specific transporter activity	MF	-0.807148767	-0.226247423	-0.169065413	4.942005241	11.48155079	0.009387573	0.033673341	PREDICTED: probable phospholipid-transporting ATPase VD
RS012787	GO:0022892	substrate-specific transporter activity	MF	-1.042887095	-1.14108662	-0.848878197	2.917253142	11.38739346	0.009805347	0.03491082	PREDICTED: facilitated trehalose transporter Tret1-2 homolog
RS007389	GO:0022892	substrate-specific transporter activity	MF	-0.71821859	-0.059688714	0.178211598	8.141021926	11.17756615	0.010803454	0.037931058	PREDICTED: V-type proton ATPase catalytic subunit A
RS007388	GO:0022892	substrate-specific transporter activity	MF	-1.012601041	-0.502641916	-0.211964145	6.238906794	11.15536226	0.011118147	0.038884183	PREDICTED: V-type proton ATPase catalytic subunit A
RS006740	GO:0022892	substrate-specific transporter activity	MF	-0.43064001	-0.612335737	-0.738849827	6.295225865	10.98541247	0.011805018	0.040967869	PREDICTED: glutamate-gated chloride channel
RS007836	GO:0022892	substrate-specific transporter activity	MF	-0.670404831	1.05713246	-0.759263575	1.495067002	10.91840834	0.012175354	0.042079146	PREDICTED: bestrophin-4
RS000123	GO:0022892	substrate-specific transporter activity	MF	0.692109916	0.810423229	0.325162475	7.0801236	10.8417935	0.012612853	0.04340062	PREDICTED: ATP-binding cassette sub-family F member 3
RS012007	GO:0022892	substrate-specific transporter activity	MF	-0.8805953	0.446042174	-0.616249485	2.603532973	10.7020781	0.013450915	0.045771636	PREDICTED: sodium-dependent phosphate transporter 2
RS002237	GO:0022892	substrate-specific transporter activity	MF	-0.77443485	-0.782975074	-0.864409769	3.087700691	10.69522479	0.013493406	0.045891426	PREDICTED: transient receptor potential cation channel protein painless
RS010501	GO:0022892	substrate-specific transporter activity	MF	-0.249581193	0.164542324	0.830736506	8.23731792	10.57427827	0.014265398	0.048114201	PREDICTED: ATP synthase subunit e, mitochondrial
RS013211	GO:0022892	substrate-specific transporter activity	MF	-0.46592983	-1.425136997	0.127678346	1.648820506	10.50692227	0.014714013	0.049415541	PREDICTED: pickpocket protein 19-like
RS005829	GO:0022892	substrate-specific transporter activity	MF	-0.565901365	-0.398114437	0.075675202	5.880326068	10.47839461	0.014908177	0.04996104	PREDICTED: facilitated trehalose transporter Tret1-like
RS012348	GO:0022892	substrate-specific transporter activity	MF	-1.171136152	-0.623448827	-0.241561177	4.399276235	10.47489738	0.014932153	0.050028076	V-type proton ATPase subunit S1
RS004574	GO:0022892	substrate-specific transporter activity	MF	-0.256414135	0.160648707	0.765161971	8.179741228	10.46991438	0.01496638	0.050122195	PREDICTED: ATP synthase subunit delta, mitochondrial
RS005224	GO:0022892	substrate-specific transporter activity	MF	0.634008216	0.005237384	-0.014917096	6.79959133	10.44569532	0.015133838	0.050532387	PREDICTED: importin subunit alpha
RS012105	GO:0022892	substrate-specific transporter activity	MF	-0.587037702	-0.604268783	0.220956679	3.910659327	10.37503836	0.015632991	0.051906642	Inward rectifier potassium channel 2
RS000868	GO:0022892	substrate-specific transporter activity	MF	-0.929392451	-0.475108864	-0.289097862	8.324170965	10.36013171	0.01574035	0.052235565	PREDICTED: sodium/potassium-transporting ATPase subunit alpha
RS008445	GO:0022892	substrate-specific transporter activity	MF	-0.794376386	-2.696738816	-1.11925367	1.261432481	10.25254337	0.016537016	0.054463175	PREDICTED: facilitated trehalose transporter Tret1-2 homolog
RS008079	GO:0022892	substrate-specific transporter activity	MF	-1.109710923	-0.938014135	-0.296800028	2.900944855	10.13930135	0.017418391	0.056816166	PREDICTED: potassium voltage-gated channel protein Shaker
RS009153	GO:0022892	substrate-specific transporter activity	MF	0.613293967	0.998084897	0.966219795	3.330543492	10.10124836	0.017724806	0.057621634	PREDICTED: mitochondrial import inner membrane translocase subunit Tim9
RS003950	GO:0022892	substrate-specific transporter activity	MF	-0.587671115	-0.868990799	-0.714405964	4.844171479	10.0941278	0.017782729	0.057765203	PREDICTED: cationic amino acid transporter 2
RS005883	GO:0022892	substrate-specific transporter activity	MF	0.579677196	0.662723942	0.941061143	6.190916775	10.02257363	0.018375223	0.059324417	PREDICTED: mitochondrial import inner membrane translocase subunit Tim10
RS009608	GO:0022892	substrate-specific transporter activity	MF	-0.650681402	-0.131877234	-0.002301287	6.339415917	9.831393766	0.020055087	0.063636624	PREDICTED: V-type proton ATPase subunit C
RS002046	GO:0022892	substrate-specific transporter activity	MF	-1.040945999	-1.141609481	-1.378037143	3.132734296	9.81352734	0.020219576	0.06404561	PREDICTED: sodium- and chloride-dependent GABA transporter 1-like
RS001413	GO:0022892	substrate-specific transporter activity	MF	-0.248212965	0.06615521	0.706495165	7.98044758	9.766834252	0.020655746	0.065132313	PREDICTED: cytochrome b-c1 complex subunit 7-like
RS015433	GO:0022892	substrate-specific transporter activity	MF	-1.023491195	-0.871650733	-0.547044844	4.320060789	9.694765434	0.021347153	0.067027185	PREDICTED: glutamate receptor ionotropic, kainate 2
RS012325	GO:0022892	substrate-specific transporter activity	MF	-0.610322555	-0.73163567	0.587302153	2.890049632	9.633930261	0.021948434	0.068590218	PREDICTED: serine/threonine-protein phosphatase 6 regulatory ankyrin repeat subunit A
RS001773	GO:0022892	substrate-specific transporter activity	MF	-1.179645502	-1.391479741	-1.203064835	2.188248107	9.497351264	0.023359555	0.072211644	PREDICTED: acetylcholine receptor subunit alpha-like
RS006809	GO:0022892	substrate-specific transporter activity	MF	-0.069599294	0.161390813	-0.669189997	5.521140821	9.427681054	0.02411329	0.074118459	PREDICTED: sodium bicarbonate cotransporter 3
RS003636	GO:0022892	substrate-specific transporter activity	MF	0.658899301	-0.027122995	0.775979951	5.812404377	9.412838955	0.024276916	0.074486992	PREDICTED: ATP-binding cassette sub-family G member 4

RS007719	GO:0022892	substrate-specific transporter activity	MF	-0.363554514	0.201823484	0.241238455	6.872179235	9.390306687	0.024527407	0.075102884	PREDICTED: V-type proton ATPase 21 kDa proteolipid subunit
RS005942	GO:0022892	substrate-specific transporter activity	MF	0.222003058	0.357500804	-0.707071584	4.173704825	9.375192754	0.024696847	0.075474905	PREDICTED: mitochondrial sodium/hydrogen exchanger 9B2-like
RS009928	GO:0022892	substrate-specific transporter activity	MF	-0.835456878	-2.347674561	-0.849585016	1.053053669	9.23428246	0.02633282	0.079385129	PREDICTED: sodium-dependent noradrenaline transporter-like
RS003234	GO:0022892	substrate-specific transporter activity	MF	-0.402942012	0.351806881	0.253791792	7.730694199	9.198914238	0.026759846	0.080432815	PREDICTED: V-type proton ATPase 16 kDa proteolipid subunit
RS012808	GO:0022892	substrate-specific transporter activity	MF	0.436531101	0.768358177	0.047570539	8.601906761	9.12311113	0.027698056	0.082719781	PREDICTED: ATP-binding cassette sub-family E member 1
RS007617	GO:0022892	substrate-specific transporter activity	MF	-1.32510407	-1.458516586	-0.870134155	3.353068646	9.039095435	0.028775582	0.085310659	PREDICTED: zinc transporter ZIP10
RS009139	GO:0022892	substrate-specific transporter activity	MF	0.649351007	1.292845053	0.336142374	2.039377777	9.031613663	0.028873505	0.085540561	PREDICTED: Y+L amino acid transporter 2
RS002268	GO:0022892	substrate-specific transporter activity	MF	-0.663213265	-0.543123609	-0.277002394	5.289469542	8.984030819	0.029503961	0.086978671	PREDICTED: inositol 1,4,5-trisphosphate receptor
RS013576	GO:0022892	substrate-specific transporter activity	MF	-0.152692106	0.186422925	0.739608273	8.524782718	8.933399856	0.030189603	0.088564608	PREDICTED: cytochrome c oxidase subunit 7A2, mitochondrial-like
RS008214	GO:0022892	substrate-specific transporter activity	MF	-0.591657749	-0.60801793	-0.342781866	5.622477097	8.89592842	0.030707059	0.089873274	PREDICTED: sodium channel protein paralytic
RS007859	GO:0022892	substrate-specific transporter activity	MF	-1.037355681	-0.953164057	-1.137934678	2.631254112	8.895408464	0.0307143	0.08987358	PREDICTED: proton-coupled amino acid transporter 4
RS003404	GO:0022892	substrate-specific transporter activity	MF	-0.282230962	0.285723529	0.434899732	4.438872301	8.825502366	0.031703186	0.092359456	PREDICTED: DET1- and DDB1-associated protein 1
RS013668	GO:0022892	substrate-specific transporter activity	MF	-1.170027407	-0.729178035	0.872714223	0.383894951	8.820674505	0.031772619	0.092540325	PREDICTED: glutamate receptor 3.4-like
RS000001	GO:0022892	substrate-specific transporter activity	MF	-0.923753843	-0.774146522	-0.309728113	8.964042611	8.801674733	0.032047317	0.093060619	PREDICTED: ryanodine receptor 44F
RS009135	GO:0022892	substrate-specific transporter activity	MF	-2.013397474	0.050668944	0.558887993	0.476879882	8.748046843	0.032835258	0.094920773	PREDICTED: sodium channel protein Nach-like
RS011130	GO:0022892	substrate-specific transporter activity	MF	0.62266454	0.316671327	-0.198111492	8.646709884	8.691352167	0.03368886	0.096889131	PREDICTED: 60 kDa heat shock protein, mitochondrial-like
RS014303	GO:0022892	substrate-specific transporter activity	MF	1.015519516	-0.140449015	-1.617687421	1.096446077	8.671576225	0.033991683	0.097559539	PREDICTED: sodium channel protein Nach-like
RS001434	GO:0022892	substrate-specific transporter activity	MF	-2.10642209	-0.940504046	-0.189980266	0.468874389	8.555806562	0.035818546	0.101461711	PREDICTED: sodium channel protein Nach-like
RS012852	GO:0022892	substrate-specific transporter activity	MF	0.095937018	0.366584098	-0.497735083	4.298329529	8.535728142	0.036145003	0.102133761	PREDICTED: glutamate receptor ionotropic, kainate 2-like
RS005358	GO:0022892	substrate-specific transporter activity	MF	0.260075089	0.471390925	0.694718137	6.608164203	8.461122113	0.037383693	0.104938933	PREDICTED: surfeit locus protein 1
RS006017	GO:0022892	substrate-specific transporter activity	MF	-0.226333875	0.829010789	0.173010283	3.457042927	8.42538268	0.037991671	0.106302401	PREDICTED: cationic amino acid transporter 2
RS003858	GO:0022892	substrate-specific transporter activity	MF	-0.359815662	-0.446595841	0.382189615	3.498410245	8.385315512	0.038684752	0.10786673	PREDICTED: sodium/potassium-transporting ATPase subunit beta-2-like
RS012784	GO:0022892	substrate-specific transporter activity	MF	-0.964188065	-1.053670411	-1.487092291	6.693074429	8.119987423	0.043595935	0.118762068	PREDICTED: excitatory amino acid transporter-like
RS014867	GO:0022892	substrate-specific transporter activity	MF	-0.157489035	-0.255961916	-0.998134687	2.846044163	8.112060193	0.04375165	0.11908543	PREDICTED: neuroglobin-like
RS005143	GO:0022892	substrate-specific transporter activity	MF	0.352665656	0.523610265	0.606871417	5.289665213	8.000205041	0.046007468	0.124178752	PREDICTED: protein SCO1 homolog, mitochondrial
RS002239	GO:0022892	substrate-specific transporter activity	MF	-0.518558054	-0.887931947	-1.328774582	2.771875246	7.996103171	0.04609231	0.124359208	PREDICTED: transient receptor potential cation channel protein painless
RS008614	GO:0022892	substrate-specific transporter activity	MF	-0.377384484	0.178151297	0.139169662	6.960985845	7.911936368	0.047867212	0.127882831	PREDICTED: H(+)/Cl(-) exchange transporter 5
RS002728	GO:0022892	substrate-specific transporter activity	MF	0.212818207	0.643840285	0.425436189	7.554169008	7.882815481	0.048496668	0.129395018	PREDICTED: protein RFT1 homolog
RS007665	GO:0022892	substrate-specific transporter activity	MF	-0.631881952	-0.428365421	0.007983181	8.098194771	7.871009593	0.048754144	0.129949141	PREDICTED: plasma membrane calcium-transporting ATPase 2
RS014326	GO:0022892	substrate-specific transporter activity	MF	0.497414651	0.118895049	-0.126889249	6.198656401	7.849052394	0.049236555	0.130930145	PREDICTED: UPF0505 protein C16orf62 homolog
RS003841	GO:0022892	substrate-specific transporter activity	MF	-0.018830809	0.667905762	-0.171179406	4.919354176	7.849821458	0.04921958	0.130930145	PREDICTED: major facilitator superfamily domain-containing protein 10
RS008434	GO:0022892	substrate-specific transporter activity	MF	-0.152418883	-0.335351871	-0.836822092	4.94412872	7.808586514	0.050137806	0.132702402	PREDICTED: potassium voltage-gated channel subfamily H member 2
RS010293	GO:0022892	substrate-specific transporter activity	MF	-1.323536549	-0.537537624	-0.40616185	2.969834016	7.797939139	0.050377598	0.133149101	PREDICTED: sodium/hydrogen exchanger 3
RS002931	GO:0022892	substrate-specific transporter activity	MF	-0.784498789	-0.645948105	-0.625581434	3.345246421	7.781766391	0.050743963	0.133976828	PREDICTED: acetylcholine receptor subunit beta-like 2
RS012051	GO:0022892	substrate-specific transporter activity	MF	-1.206457775	-0.320139517	-0.400442703	3.93903891	7.774642457	0.050906164	0.13434875	PREDICTED: sodium-independent sulfate anion transporter-like
RS012317	GO:0022892	substrate-specific transporter activity	MF	-0.648427381	0.001131192	-0.007541087	6.300999393	7.771586789	0.050975892	0.13447641	PREDICTED: calcium-transporting ATPase type 2C member 1
RS003265	GO:0022892	substrate-specific transporter activity	MF	0.67065611	0.570992468	0.622458253	5.669387556	7.641310173	0.054036623	0.140868812	PREDICTED: 28S ribosomal protein S9, mitochondrial
RS015407	GO:0022892	substrate-specific transporter activity	MF	-0.53343531	-0.824506724	0.003613607	6.554411162	7.589715422	0.055297539	0.143502413	PREDICTED: sodium/potassium-transporting ATPase subunit beta-2
RS005847	GO:0022892	substrate-specific transporter activity	MF	-0.009134422	0.240630937	0.785370217	8.208365721	7.589016609	0.055314812	0.143517665	PREDICTED: ATP synthase subunit epsilon, mitochondrial-like
RS014918	GO:0022892	substrate-specific transporter activity	MF	-0.265116474	0.084232844	0.568475259	7.79189049	7.503165544	0.057477171	0.147999897	PREDICTED: cytochrome c1, heme protein, mitochondrial
RS007235	GO:0022892	substrate-specific transporter activity	MF	-0.411884685	-0.555436113	-0.88679334	5.718214674	7.500689334	0.057540743	0.148133302	PREDICTED: melanotransferrin
RS009082	GO:0022892	substrate-specific transporter activity	MF	-1.344763505	-0.953478298	-1.303298962	1.512508029	7.470773642	0.058314177	0.149663762	PREDICTED: glycine receptor subunit alpha-2-like
RS008448	GO:0022892	substrate-specific transporter activity	MF	-0.874319748	-1.179805365	-0.159545862	2.543187294	7.449190097	0.058878449	0.150714469	PREDICTED: facilitated trehalose transporter Tret1-2 homolog
RS002799	GO:0022892	substrate-specific transporter activity	MF	-0.659046602	-0.211273757	-0.16274335	6.419710777	7.409924332	0.059918615	0.152787009	PREDICTED: V-type proton ATPase subunit H
RS002292	GO:0022892	substrate-specific transporter activity	MF	-0.849226044	-0.724590211	-0.752733052	3.999828127	7.339934983	0.06181703	0.156330055	V-type proton ATPase subunit S1
RS004018	GO:0022892	substrate-specific transporter activity	MF	-0.854655712	-0.687918747	0.356622885	2.251863917	7.340140368	0.061811374	0.156330055	PREDICTED: potassium/sodium hyperpolarization-activated cyclic nucleotide-gated channel 2
RS010902	GO:0022892	substrate-specific transporter activity	MF	-0.413608261	-0.854721106	-0.933757255	3.754238005	7.337994128	0.061870497	0.156389668	PREDICTED: glutamate receptor 1
RS006810	GO:0022892	substrate-specific transporter activity	MF	-0.692918241	-0.709642301	-0.469276201	5.552917252	7.276110601	0.063598968	0.159523084	PREDICTED: Y+L amino acid transporter 2
RS006920	GO:0022892	substrate-specific transporter activity	MF	-0.632866598	-0.974633547	-0.967627708	3.248752993	7.241310635	0.064591429	0.161433709	PREDICTED: glutamate receptor ionotropic, NMDA 2B
RS008996	GO:0022892	substrate-specific transporter activity	MF	-0.818894069	-0.144607229	0.175883673	2.981815036	7.237819263	0.064691824	0.161620478	PREDICTED: cytochrome c oxidase assembly factor 6 homolog
RS002605	GO:0022892	substrate-specific transporter activity	MF	-0.518735488	0.392463203	-0.08725497	4.03235098	7.22499608	0.065061855	0.162287383	PREDICTED: solute carrier family 40 member 1
RS000609	GO:0022892	substrate-specific transporter activity	MF	0.612008555	0.732502903	0.190053663	8.31658549	7.219606678	0.065217984	0.162580225	PREDICTED: muscle calcium channel subunit alpha-1
RS006503	GO:0022892	substrate-specific transporter activity	MF	-0.789357073	-0.069673576	-0.042357301	4.655019811	7.200877419	0.065763394	0.163551378	PREDICTED: transient receptor potential cation channel trpm
RS015105	GO:0022892	substrate-specific transporter activity	MF	-0.678666411	-2.224992259	-0.562865466	0.559101899	7.185153044	0.066224707	0.164438873	PREDICTED: organic cation transporter 1
RS005264	GO:0022892	substrate-specific transporter activity	MF	-1.488549143	-1.308665565	-1.379377385	1.600885014	7.17732657	0.066455483	0.164816926	PREDICTED: sodium-dependent neutral amino acid transporter B(0)AT3
RS006739	GO:0022892	substrate-specific transporter activity	MF	-0.215562063	-0.194338635	-0.661480143	5.23328973	7.126492884	0.067973454	0.167558754	PREDICTED: potassium voltage-gated channel protein Shab
RS000133	GO:0022892	substrate-specific transporter activity	MF	-0.045454902	0.182331435	0.994722318	2.687399163	7.116976504	0.068261333	0.168071011	PREDICTED: putative ammonium transporter 1
RS015404	GO:0022892	substrate-specific transporter activity	MF	-0.033173942	0.491254687	-0.13268681	6.364272838	7.035330626	0.07078015	0.172954058	PREDICTED: sodium/potassium-transporting ATPase subunit beta-2
RS003449	GO:0022892	substrate-specific transporter activity	MF	-0.271604031	-0.623910119	-0.38398901	6.1655336	7.019104607	0.07129133	0.173865804	PREDICTED: glutamate receptor ionotropic, kainate 2
RS014295	GO:0022892	substrate-specific transporter activity	MF	0.412981653	0.489533068	0.464743455	5.949145628	7.0080526	0.071641551	0.174618482	PREDICTED: zinc transporter 5
RS003825	GO:0022892	substrate-specific transporter activity	MF	-0.116552603	-0.74841501	-0.195189608	7.935069367	6.915883201	0.07462774	0.18005155	PREDICTED: H(+)/Cl(-) exchange transporter 7
RS003427	GO:0022892	substrate-specific transporter activity	MF	-0.513964722	-0.550228674	-0.09809219	5.664872118	6.801016027	0.078517892	0.186983778	PREDICTED: voltage-dependent calcium channel type A subunit alpha-1
RS002296	GO:0022892	substrate-specific transporter activity	MF	0.050929101	-1.155041098	-1.453067074	0.872761787	6.743534644	0.080537346	0.190763087	PREDICTED: glycine receptor subunit alpha-4
RS003145	GO:0022892	substrate-specific transporter activity	MF	-1.309880541	-0.628756059	-0.991127574	1.553035926	6.713260678	0.081620986	0.19245815	PREDICTED: neuronal acetylcholine receptor subunit alpha-7-like
RS012048	GO:0022892	substrate-specific transporter activity	MF	-0.674894361	-1.155560739	-0.486644875	2.649535097	6.691851744	0.082395778	0.193852499	PREDICTED: sodium-independent sulfate anion transporter-like
RS009471	GO:0022892	substrate-specific transporter activity	MF	0.454440582	0.068570398	0.92902968	1.975531925	6.680170813	0.082821497	0.194672252	PREDICTED: potassium voltage-gated channel protein Shal
RS000035	GO:0022892	substrate-specific transporter activity	MF	-0.59529335	-0.304820032	0.347972721	8.866264808	6.597065948	0.085912068	0.200794204	PREDICTED: cytochrome b-c1 complex subunit Rieske, mitochondrial-like
RS000318	GO:0022892	substrate-specific transporter activity	MF	-0.409411201	-0.696964023	-0.524046279	5.146315283	6.540394841	0.088082972	0.204696733	PREDICTED: alpha-aminoadipic semialdehyde dehydrogenase
RS001410	GO:0022892	substrate-specific transporter activity	MF	-0.833940378	-1.145871699	-0.470397772	2.458966391	6.454844784	0.091460426	0.210576345	PREDICTED: globin
RS001821	GO:0022892	substrate-specific transporter activity	MF	-0.416559115	-0.243117375	0.420607418	9.114591662	6.45061119	0.091630759	0.210852874	PREDICTED: cytochrome c oxidase subunit 4 isoform 1, mitochondrial
RS007738	GO:0022892	substrate-specific transporter activity	MF	-2.236721279	-1.816342735	0.104485909	0.00738012	6.381695156	0.094446755	0.215638584	PREDICTED: dynein heavy chain 10, axonemal
RS006697	GO:0022892	substrate-specific transporter activity	MF	-0.137394972	-0.578930188	0.149350385	8.454117513	6.353830787	0.095608773	0.21750305	PREDICTED: zinc transporter ZIP13 homolog
RS007190	GO:0022892	substrate-specific transporter activity	MF	-0.878979816	-0.595509543	0.227085088	2.704210207	6.34629855	0.095925237	0.217838484	PREDICTED: sodium channel protein Nach-like
RS010275	GO:0022892	substrate-specific transporter activity	MF	-0.327809837	0.151537852	-0.881426827	3.129420115	6.335110353	0.096397162	0.218625222	PREDICTED: equilibrative nucleoside transporter 4
RS015389	GO:0022892	substrate-specific transporter activity	MF	-1.014604708	-1.708407111	-0.480189784	0.854123779	6.332870963	0.096491888	0.218718663	PREDICTED: glutamate receptor ionotropic, delta-2
RS009966	GO:0022892	substrate-specific transporter activity	MF	-0.243614202	-0.508062646	-0.78761656	3.789270855	6.228722713	0.100997397	0.225925617	PREDICTED: transient receptor potential channel pyrexia-like
RS005351	GO:0022892	substrate-specific transporter activity	MF	-0.397867942	-0.039889938	0.245645216	7.41412513	6.082183312	0.107679573	0.237000444	PREDICTED: glucose transporter type 1
RS011409	GO:0022892	substrate-specific transporter activity	MF	-0.278990895	0.058673971	0.653521491	10.28933054	5.992909669	0.111955695	0.243939451	PREDICTED: voltage-dependent anion-selective channel protein 2
RS000168	GO:0022892	substrate-specific transporter activity	MF	-0.555511687	-0.887978015	-1.234124807	1.652741901	5.837936606	0.119766759	0.255954957	PREDICTED: potassium voltage-gated channel protein eag
RS000398	GO:0022892	substrate-specific transporter activity	MF	0.277198819	0.379861004	-0.066027388	5.894739848	5.761774521	0.123793477	0.26277685	PREDICTED: RNA polymerase I-specific transcription initiation factor RRN3
RS001145	GO:0022892	substrate-specific transporter activity	MF	-0.53262649	-0.12107554	0.117285332	6.762749099	5.723923744	0.125842321	0.265692906	PREDICTED: solute carrier family 2, facilitated glucose transporter member 1-like
RS003358	GO:0022892	substrate-specific transporter activity	MF	-0.353225898	-0.121840444	0.371616686	8.291426996	5.633056759	0.130893435	0.272925814	PREDICTED: ATP synthase-coupling factor 6, mitochondrial
RS008279	GO:0022892	substrate-specific transporter activity	MF	-0.484679453	-0.433954641	0.086422434	3.880506245	5.606020043	0.132433181	0.27531586	PREDICTED: potassium voltage-gated channel protein Shaw-like
RS009361	GO:0022892	substrate-specific transporter activity	MF	-0.469548252	-1.030089652	-0.272125463	2.379260128	5.571170404	0.134443268	0.278300217	PREDICTED: sodium- and chloride-dependent glycine transporter 2-like
RS002972	GO:0022892	substrate-specific transporter activity	MF	-0.311172151	-0.506842424	-0.237806006	6.495567074	5.534163734	0.136609467	0.281857737	PREDICTED: sodium leak channel non-selective protein
RS002378	GO:0022892	substrate-specific transporter activity	MF	0.189638401	-0.292415431	-0.3313077	5.146004911	5.532246854	0.13672257	0.281998697	PREDICTED: exportin-6
RS012532	GO:0022892	substrate-specific transporter activity	MF	0.52422059	0.27169055	0.170626759	5.950381512	5.527522768	0.137001687	0.282351902	PREDICTED: adenosine 3'-phospho 5'-phosphosulfate transporter 2
RS007587	GO:0022892	substrate-specific transporter activity	MF	0.548015678	0.376775275	0.454348959	4.86046888	5.504926438	0.138344259	0.284411948	PREDICTED: magnesium transporter NIPA2
RS012098	GO:0022892	substrate-specific transporter activity	MF	-0.722635213	-0.863193595	-0.599560773	3.5591329	5.486483871	0.13944927	0.285797251	PREDICTED: protein tweety
RS009262	GO:0022892	substrate-specific transporter activity	MF	-0.25857572	0.038639212	0.546361074	8.794488927	5.469506202	0.140473903	0.28738277	PREDICTED: cytochrome c oxidase subunit 7C, mitochondrial-like
RS012521	GO:0022892	substrate-specific transporter activity	MF	-0.27911124	0.207963234	0.176143396	5.682101261	5.464162095	0.140797903	0.287847294	PREDICTED: sodium/hydrogen exchanger 8
RS000402	GO:0022892	substrate-specific transporter activity	MF	-0.26718069	0.008072239	0.51581876	8.673900207	5.301862053	0.150981611	0.302828859	PREDICTED: cytochrome c oxidase subunit 6A, mitochondrial
RS006927	GO:0022892	substrate-specific transporter activity	MF	-0.523545819	-0.19238077	0.403170712	9.665847911	5.252945184	0.154185014	0.307637209	PREDICTED: mitochondrial-processing peptidase subunit beta
RS014310	GO:0022892	substrate-specific transporter activity	MF	-0.394503871	-0.23364982	-0.939036808	2.647212913	5.221628248	0.156269422	0.310811292	PREDICTED: neuronal acetylcholine receptor subunit alpha-7
RS004262	GO:0022892	substrate-specific transporter activity	MF	-0.304556625	0.103631535	0.676510563	10.41026235	5.204184057	0.157441978	0.312748307	PREDICTED: ATP synthase lipid-binding protein, mitochondrial
RS007787	GO:0022892	substrate-specific transporter activity	MF	-0.305912555	0.11337882	0.601620189	9.43857381	5.186627171	0.15863048	0.314655166	PREDICTED: ATP synthase subunit b, mitochondrial
RS008202	GO:0022892	substrate-specific transporter activity	MF	0.24252685	0.342844048	-0.965951499	1.493557986	5.185516154	0.158705973	0.314677881	PREDICTED: voltage-dependent calcium channel gamma-5 subunit
RS002769	GO:0022892	substrate-specific transporter activity	MF	-0.180596666	-0.510025269	-0.494314256	5.266272897	5.147923967	0.161280396	0.318264698	PREDICTED: mitochondrial 2-oxoglutarate/malate carrier protein
RS000666	GO:0022892	substrate-specific transporter activity	MF	-0.080539122	0.013403368	0.652885093	9.136399161	5.147116783	0.161336103	0.31832475	PREDICTED: ATP synthase subunit g, mitochondrial
RS006076	GO:0022892	substrate-specific transporter activity	MF	-0.399500112	-0.234112198	-0.791572834	3.218719557	5.135126595	0.162165734	0.319411206	PREDICTED: cationic amino acid transporter 4
RS015132	GO:0022892	substrate-specific transporter activity	MF	-0.311288053	0.021217655	0.441252337	8.852842665	5.035692944	0.169202112	0.329814486	PREDICTED: ATP synthase subunit O, mitochondrial
RS001012	GO:0022892	substrate-specific transporter activity	MF	-0.050637771	0.65837124	0.220417241	5.028311487	5.034189195	0.169310694	0.329872366	PREDICTED: sodium/calcium exchanger 2
RS013006	GO:0022892	substrate-specific transporter activity	MF	-0.538272733	-0.817432042	-1.008287916	2.921270255	4.979088813	0.173334784	0.334904704	PREDICTED: exportin-4-like
RS007406	GO:0022892	substrate-specific transporter activity	MF	1.53218285	1.416234104	0.234676903	0.509107572	4.915967777	0.17805483	0.341676447	PREDICTED: sodium-independent sulfate anion transporter-like
RS007390	GO:0022892	substrate-specific transporter activity	MF	-0.350929593	-1.171722101	-0.259139814	1.654126139	4.900550276	0.179225874	0.343191525	PREDICTED: open rectifier potassium channel protein 1
RS012974	GO:0022892	substrate-specific transporter activity	MF	0.386362631	0.350475987	0.590578301	4.958398051	4.857275707	0.182551447	0.347919632	PREDICTED: mitochondrial import inner membrane translocase subunit Tim16
RS004430	GO:0022892	substrate-specific transporter activity	MF	-0.579491375	-0.057917442	0.425475066	2.309902759	4.852857272	0.182894224	0.348520179	Glutamate [NMDA] receptor subunit 3A
RS015406	GO:0022892	substrate-specific transporter activity	MF	-0.548835798	-0.34994381	-0.083936965	5.176471396	4.839807661	0.183910115	0.349694714	PREDICTED: sodium/potassium-transporting ATPase subunit beta-2
RS015035	GO:0022892	substrate-specific transporter activity	MF	-0.486032243	-0.438869682	-0.112800307	5.262898327	4.77200592	0.189273765	0.356097071	PREDICTED: solute carrier family 2, facilitated glucose transporter member 10-like
RS011272	GO:0022892	substrate-specific transporter activity	MF	-0.449550793	-0.842262029	0.317704025	1.68576224	4.742178986	0.191679247	0.358960362	PREDICTED: transient receptor potential-gamma protein
RS013062	GO:0022892	substrate-specific transporter activity	MF	-0.00162788	-0.815422885	-0.945361754	1.375980209	4.702121991	0.194954616	0.363203911	PREDICTED: transient receptor potential channel pyrexia

RS008733	GO:0022892	substrate-specific transporter activity	MF	-0.069301153	-0.696190609	0.565661049	1.222507914	4.700705743	0.195071368	0.363260212	PREDICTED: pickpocket protein 28-like
RS009963	GO:0022892	substrate-specific transporter activity	MF	-0.732608205	-0.778239432	-0.747787888	2.188684887	4.691092768	0.195865561	0.364308208	PREDICTED: oxysterol-binding protein 1
RS005980	GO:0022892	substrate-specific transporter activity	MF	-0.519964769	-0.115820264	0.014177289	6.649793844	4.674091701	0.197277507	0.365853766	PREDICTED: V-type proton ATPase subunit d
RS013193	GO:0022892	substrate-specific transporter activity	MF	-0.571318105	-0.638002435	-0.492911161	3.125045391	4.612135042	0.202503545	0.372595481	PREDICTED: gamma-aminobutyric acid receptor subunit beta-like
RS012106	GO:0022892	substrate-specific transporter activity	MF	-0.818692691	-0.45460686	0.366734327	1.141769569	4.599841297	0.203555696	0.373808755	Inward rectifier potassium channel 2
RS009973	GO:0022892	substrate-specific transporter activity	MF	0.1676973	0.302067179	0.694181931	3.324934717	4.531641986	0.2094852	0.381030706	PREDICTED: sugar transporter SWEET1
RS002890	GO:0022892	substrate-specific transporter activity	MF	-0.213107922	-0.035236467	0.494651933	9.00551627	4.518839864	0.210615958	0.382259313	PREDICTED: cytochrome c oxidase subunit 5A, mitochondrial
RS012324	GO:0022892	substrate-specific transporter activity	MF	-0.639112315	-1.234689077	-0.08776879	1.305640976	4.37485531	0.22372824	0.397633848	PREDICTED: serine/threonine-protein phosphatase 6 regulatory ankyrin repeat subunit A
RS009362	GO:0022892	substrate-specific transporter activity	MF	-0.151734251	-1.379851033	-0.853593219	0.577299208	4.353138426	0.225770076	0.399764452	PREDICTED: sodium- and chloride-dependent glycine transporter 2-like
RS001539	GO:0022892	substrate-specific transporter activity	MF	-0.380736825	-1.0292407	-0.36481089	2.349632725	4.316399244	0.229263428	0.403767811	PREDICTED: transient receptor potential cation channel protein painless
RS005280	GO:0022892	substrate-specific transporter activity	MF	0.572920685	0.052379455	0.938447567	3.032392488	4.273149565	0.233439508	0.40894939	PREDICTED: acid-sensing ion channel 4
RS001911	GO:0022892	substrate-specific transporter activity	MF	-0.354858321	-0.511067736	-0.530111822	4.46637499	4.265443861	0.234190845	0.409944281	PREDICTED: palmitoyltransferase ZDHHC17
RS007791	GO:0022892	substrate-specific transporter activity	MF	-0.379787464	0.049869261	0.105041138	7.48004628	4.225689753	0.238102407	0.41486742	PREDICTED: V-type proton ATPase subunit E
RS015079	GO:0022892	substrate-specific transporter activity	MF	-1.376295246	0.661290456	0.618943801	0.209343525	4.174647883	0.243212401	0.420568148	PREDICTED: facilitated trehalose transporter Tret1-like
RS012398	GO:0022892	substrate-specific transporter activity	MF	-0.181909214	0.045983675	0.559515665	9.326609998	4.173573583	0.243321024	0.420643099	PREDICTED: cytochrome c oxidase subunit 5B, mitochondrial-like
RS011634	GO:0022892	substrate-specific transporter activity	MF	-1.482237858	-0.869085331	-1.279144023	0.748490273	4.162431432	0.24445023	0.422083595	PREDICTED: glutamate receptor ionotropic, delta-2-like
RS001433	GO:0022892	substrate-specific transporter activity	MF	-0.877308965	-1.067905037	-0.581830951	1.405281912	4.044182144	0.25673261	0.436813089	PREDICTED: Niemann-Pick C1 protein
RS001774	GO:0022892	substrate-specific transporter activity	MF	-0.556967124	-0.686693785	0.064399051	2.246327006	4.014186998	0.25993626	0.440531132	PREDICTED: acetylcholine receptor subunit alpha-like
RS012212	GO:0022892	substrate-specific transporter activity	MF	1.298461473	1.241884362	0.487723216	0.415207106	3.920422645	0.270186252	0.451829193	PREDICTED: sodium channel protein 60E
RS000049	GO:0022892	substrate-specific transporter activity	MF	-0.167016261	-0.078808593	0.315739518	4.19490186	3.918242209	0.270428905	0.452114912	PREDICTED: protein l'm not dead yet-like
RS004111	GO:0022892	substrate-specific transporter activity	MF	0.320806788	0.270359069	0.505810835	5.451871069	3.897981521	0.272693095	0.454693081	PREDICTED: pleckstrin homology domain-containing family A member 3
RS004419	GO:0022892	substrate-specific transporter activity	MF	-0.39398529	0.162481593	-1.185227216	0.809494392	3.887905498	0.273825486	0.455851742	PREDICTED: transient receptor potential-gamma protein
RS003691	GO:0022892	substrate-specific transporter activity	MF	-0.396884624	-0.571293958	0.253637659	1.856747806	3.871872414	0.275636118	0.457912129	PREDICTED: transient receptor potential cation channel subfamily A member 1
RS010418	GO:0022892	substrate-specific transporter activity	MF	-0.19455437	0.000394051	0.481391778	9.395600975	3.855673322	0.277476464	0.459791431	PREDICTED: cytochrome c oxidase subunit 6B1-like
RS011747	GO:0022892	substrate-specific transporter activity	MF	-0.380454491	0.007681606	0.358897081	9.615965078	3.849118736	0.278224261	0.460864966	PREDICTED: ATP synthase subunit gamma, mitochondrial
RS002977	GO:0022892	substrate-specific transporter activity	MF	0.118604573	0.441224603	0.037913535	4.957205925	3.761211507	0.288430235	0.47128419	PREDICTED: AP-1 complex subunit sigma-2
RS000693	GO:0022892	substrate-specific transporter activity	MF	-0.697277451	-0.63859341	-1.105155719	1.668516432	3.761841056	0.288355965	0.47128419	PREDICTED: solute carrier family 12 member 4
RS006724	GO:0022892	substrate-specific transporter activity	MF	1.072023102	0.112416307	0.609701895	0.789605252	3.761098346	0.288443587	0.47128419	PREDICTED: glutamate receptor ionotropic, delta-1-like
RS002930	GO:0022892	substrate-specific transporter activity	MF	-0.451673532	-0.71437108	-0.718954078	3.306419228	3.701408177	0.295564164	0.478695356	PREDICTED: acetylcholine receptor subunit alpha-L1
RS005072	GO:0022892	substrate-specific transporter activity	MF	-0.654987192	-0.4219481	-0.18940484	3.26003723	3.685352455	0.297506145	0.480656437	PREDICTED: voltage-dependent T-type calcium channel subunit alpha-1G
RS008197	GO:0022892	substrate-specific transporter activity	MF	-0.307158998	-0.373647818	-0.769382915	3.163133414	3.663806527	0.300130093	0.48236835	PREDICTED: lysophospholipid acyltransferase 7
RS003147	GO:0022892	substrate-specific transporter activity	MF	-0.505550451	-0.027606287	-0.18120612	3.786984288	3.627252198	0.30462908	0.487532937	PREDICTED: acetylcholine receptor subunit beta-like 1
RS014617	GO:0022892	substrate-specific transporter activity	MF	-0.072631645	0.084385518	0.526454273	9.138635249	3.625994331	0.304784959	0.487596216	PREDICTED: putative ATP synthase subunit f, mitochondrial
RS012817	GO:0022892	substrate-specific transporter activity	MF	-0.287960684	-0.628111107	-0.66343646	3.635326683	3.625082508	0.304897998	0.48766642	PREDICTED: facilitated trehalose transporter Tret1-like
RS015381	GO:0022892	substrate-specific transporter activity	MF	-0.14122205	0.494615068	-0.457720223	1.269481569	3.572397731	0.311493056	0.493953414	PREDICTED: potassium voltage-gated channel subfamily KQT member 1
RS008419	GO:0022892	substrate-specific transporter activity	MF	-0.579270886	-0.22684126	-0.07023873	5.06982487	3.507414936	0.319801701	0.502353125	PREDICTED: chloride channel protein 2
RS013707	GO:0022892	substrate-specific transporter activity	MF	-0.500388832	-0.162680031	-0.110632879	6.260634623	3.457673335	0.326293381	0.508267019	PREDICTED: phospholipid scramblase 2
RS013677	GO:0022892	substrate-specific transporter activity	MF	0.034211804	0.280550659	0.595537596	9.222031987	3.437461301	0.328964218	0.510658864	PREDICTED: cytochrome c oxidase subunit 6C
RS009119	GO:0022892	substrate-specific transporter activity	MF	-0.633398641	0.027582619	-0.254763808	2.374824839	3.400802845	0.333857375	0.51571198	PREDICTED: lateral signaling target protein 2 homolog
RS006561	GO:0022892	substrate-specific transporter activity	MF	0.353995304	0.237572917	0.419480704	5.635873753	3.395244005	0.334604917	0.516387054	PREDICTED: EH domain-containing protein 3
RS015453	GO:0022892	substrate-specific transporter activity	MF	-0.09263959	0.415348464	0.277714417	5.686672294	3.355959113	0.33992981	0.521573591	PREDICTED: sodium-dependent phosphate transporter 2
RS008482	GO:0022892	substrate-specific transporter activity	MF	0.456620351	0.231742272	0.664992756	4.899508409	3.329273867	0.343589006	0.524567819	PREDICTED: organic cation transporter protein
RS008880	GO:0022892	substrate-specific transporter activity	MF	0.14769946	0.553054828	0.336408558	3.369176491	3.28851771	0.349243966	0.529733651	PREDICTED: pickpocket protein 19-like
RS009580	GO:0022892	substrate-specific transporter activity	MF	-0.333340287	-0.479408474	-0.582222382	3.302563198	3.277032573	0.350852097	0.531532696	PREDICTED: ceramide-1-phosphate transfer protein
RS013351	GO:0022892	substrate-specific transporter activity	MF	0.199508499	-0.221355739	-0.354559239	3.592239645	3.258354324	0.353481134	0.534116858	PREDICTED: Down syndrome critical region protein 3 homolog
RS008726	GO:0022892	substrate-specific transporter activity	MF	-0.239198332	0.278689624	0.146315272	4.384525019	3.151643572	0.368830479	0.550035649	PREDICTED: equilibrative nucleoside transporter 3
RS005874	GO:0022892	substrate-specific transporter activity	MF	0.356190643	0.298141688	0.106337447	6.648518844	3.145329906	0.369756365	0.550764242	PREDICTED: importin subunit alpha-3
RS008888	GO:0022892	substrate-specific transporter activity	MF	-0.294502555	-0.567523121	-0.206094758	5.180319559	3.112412951	0.374615949	0.554657096	PREDICTED: facilitated trehalose transporter Tret1-2 homolog
RS001554	GO:0022892	substrate-specific transporter activity	MF	-0.434675524	-0.248984799	-0.186000304	5.41833871	3.07154736	0.380724991	0.560358769	PREDICTED: AP-1 complex subunit gamma-1
RS003956	GO:0022892	substrate-specific transporter activity	MF	-0.323676554	0.116745023	-0.07691896	6.422899031	3.056887659	0.382937112	0.562262205	PREDICTED: solute carrier family 2, facilitated glucose transporter member 1-like
RS003609	GO:0022892	substrate-specific transporter activity	MF	-0.051627285	0.355758402	0.153478437	5.36998255	3.050524042	0.383900772	0.56337058	PREDICTED: zinc transporter ZIP1
RS002254	GO:0022892	substrate-specific transporter activity	MF	0.026779126	-0.240562604	-0.407019639	8.0023415	2.926808228	0.403048115	0.579800873	PREDICTED: non-specific lipid-transfer protein
RS000591	GO:0022892	substrate-specific transporter activity	MF	-0.210745642	-0.711938748	-0.062273638	2.249015287	2.914724059	0.404960781	0.581735608	PREDICTED: solute carrier family 41 member 1-like
RS005802	GO:0022892	substrate-specific transporter activity	MF	-0.201437577	-0.069914946	-0.593279778	3.766942431	2.895793271	0.407972418	0.584129676	PREDICTED: two pore potassium channel protein sup-9
RS001904	GO:0022892	substrate-specific transporter activity	MF	-0.27687136	-0.081401122	0.3505686	9.687890931	2.842286187	0.416586082	0.592334615	PREDICTED: ATP synthase subunit d, mitochondrial
RS011686	GO:0022892	substrate-specific transporter activity	MF	-0.652816077	-0.692313812	-0.834928913	1.23665669	2.748017073	0.432128872	0.606039089	PREDICTED: TWiK family of potassium channels protein 18-like
RS004207	GO:0022892	substrate-specific transporter activity	MF	-0.450748093	-0.259258272	-0.448403469	6.976635555	2.715150178	0.437658817	0.611208155	PREDICTED: Niemann-Pick C1 protein
RS012107	GO:0022892	substrate-specific transporter activity	MF	-0.368799719	0.188292375	-0.013404979	5.272470317	2.653348424	0.448213543	0.621757305	PREDICTED: G protein-activated inward rectifier potassium channel 3-like
RS014879	GO:0022892	substrate-specific transporter activity	MF	-0.246283404	-0.362594032	0.41505142	1.337328694	2.627851228	0.452627775	0.625660651	PREDICTED: glycine receptor subunit alphaZ1-like
RS007790	GO:0022892	substrate-specific transporter activity	MF	0.13034852	0.225712601	0.386180211	4.625198528	2.606288996	0.456388071	0.628856824	PREDICTED: mitochondrial glutamate carrier 1-like
RS010426	GO:0022892	substrate-specific transporter activity	MF	-0.0320805	-0.19528696	-0.535965982	2.761673686	2.581268642	0.460782863	0.632865926	PREDICTED: glutamate receptor ionotropic, NMDA 2B
RS007973	GO:0022892	substrate-specific transporter activity	MF	0.255068646	0.370011576	0.284397768	6.620720548	2.53491853	0.469013562	0.640437689	PREDICTED: zinc transporter 9
RS009279	GO:0022892	substrate-specific transporter activity	MF	0.294401775	0.208728083	0.372173078	5.384293591	2.517191015	0.472192292	0.643035221	PREDICTED: probable low affinity copper uptake protein 2
RS003198	GO:0022892	substrate-specific transporter activity	MF	-1.073241752	-1.028205771	-0.905668183	0.370380606	2.506990701	0.474029032	0.644352037	PREDICTED: glutamate receptor ionotropic, delta-1-like
RS011365	GO:0022892	substrate-specific transporter activity	MF	-0.252603307	-0.167521421	-0.434236646	6.323749924	2.498431248	0.475574659	0.645682607	PREDICTED: translocation protein SEC62
RS003214	GO:0022892	substrate-specific transporter activity	MF	0.817513479	0.952415195	0.900489695	0.817958486	2.496776884	0.475873854	0.64588375	PREDICTED: potassium voltage-gated channel subfamily KQT member 4
RS006138	GO:0022892	substrate-specific transporter activity	MF	0.077942232	0.584419518	-0.370544993	1.33370078	2.490635188	0.476985892	0.646552279	PREDICTED: potassium channel subfamily K member 18-like
RS000905	GO:0022892	substrate-specific transporter activity	MF	0.022025399	0.042858662	0.42533997	8.298571127	2.483368496	0.478304268	0.647507681	PREDICTED: cytochrome b-c1 complex subunit 10-like
RS012899	GO:0022892	substrate-specific transporter activity	MF	0.204967532	0.199038052	0.483016212	5.559314101	2.452259782	0.483980622	0.652426585	PREDICTED: SID1 transmembrane family member 1-like
RS009087	GO:0022892	substrate-specific transporter activity	MF	-0.753493655	-0.466320961	-0.51762636	2.340610515	2.452889414	0.483865213	0.652426585	PREDICTED: probable glutamate receptor
RS007514	GO:0022892	substrate-specific transporter activity	MF	-0.393644456	0.206862252	-0.369998274	2.851975701	2.440356048	0.48616657	0.653933758	PREDICTED: solute carrier family 26 member 10-like
RS002311	GO:0022892	substrate-specific transporter activity	MF	0.21167088	0.356479307	0.364913574	7.124016358	2.415178378	0.490815427	0.65773111	PREDICTED: putative GTP-binding protein 6
RS004471	GO:0022892	substrate-specific transporter activity	MF	0.302505199	0.1016203	-0.272900349	3.077100879	2.396738775	0.494241988	0.660492741	PREDICTED: Y+L amino acid transporter 2
RS003266	GO:0022892	substrate-specific transporter activity	MF	-0.36926559	-0.039795183	-0.401685153	5.103564049	2.37990733	0.497385826	0.663356676	PREDICTED: transient receptor potential channel pyrexia
RS006811	GO:0022892	substrate-specific transporter activity	MF	-0.091072902	-0.032205255	0.237958262	6.439935586	2.353693428	0.502312778	0.667124828	PREDICTED: angio-associated migratory cell protein
RS005021	GO:0022892	substrate-specific transporter activity	MF	-0.06781788	-0.474899669	-0.350734329	3.155367473	2.352458366	0.50254583	0.667200314	PREDICTED: molybdate-anion transporter-like
RS001365	GO:0022892	substrate-specific transporter activity	MF	0.237278489	0.141297916	0.554160331	3.551638791	2.315424336	0.509572456	0.67338898	PREDICTED: glutamate receptor ionotropic, kainate 2
RS014502	GO:0022892	substrate-specific transporter activity	MF	-0.219647182	0.224994314	0.605975653	1.297553296	2.311846878	0.51025516	0.673954219	PREDICTED: small conductance calcium-activated potassium channel protein
RS001131	GO:0022892	substrate-specific transporter activity	MF	-0.46672796	-0.438731687	-0.485486742	3.00696323	2.28306329	0.515773294	0.678325905	PREDICTED: two pore calcium channel protein 1-like
RS005763	GO:0022892	substrate-specific transporter activity	MF	-0.049864591	0.331988566	0.418799139	2.785560138	2.265395088	0.519182685	0.681106738	PREDICTED: TWiK family of potassium channels protein 7
RS002215	GO:0022892	substrate-specific transporter activity	MF	-0.459893543	-0.373663745	-0.327616751	3.245153068	2.258109459	0.520593488	0.682313913	PREDICTED: CMP-sialic acid transporter 1
RS010646	GO:0022892	substrate-specific transporter activity	MF	-0.387797302	-0.153467445	-0.201059514	5.65127642	2.191739529	0.533577259	0.692056848	PREDICTED: probable phospholipid-transporting ATPase IA
RS002929	GO:0022892	substrate-specific transporter activity	MF	-0.39492811	-0.310803863	-0.368352585	3.630774923	2.110337646	0.54982422	0.704208911	PREDICTED: acetylcholine receptor subunit alpha-like 1
RS006952	GO:0022892	substrate-specific transporter activity	MF	0.196623599	-0.003036232	-0.225453179	4.529529399	2.10907617	0.550078776	0.704320059	PREDICTED: AP-3 complex subunit sigma-2
RS009552	GO:0022892	substrate-specific transporter activity	MF	-0.317174334	0.102956036	0.402195532	11.51695741	2.083221242	0.555314671	0.708862049	PREDICTED: ATP synthase subunit beta, mitochondrial
RS002902	GO:0022892	substrate-specific transporter activity	MF	-0.415647108	-0.152738152	0.265175951	11.47381982	2.020686865	0.56812419	0.719524944	PREDICTED: ATP synthase subunit alpha, mitochondrial
RS010798	GO:0022892	substrate-specific transporter activity	MF	0.021270494	0.09209859	-1.197682465	0.555972403	2.01223563	0.569871042	0.720350873	PREDICTED: zinc transporter ZIP11
RS001956	GO:0022892	substrate-specific transporter activity	MF	0.077958348	-0.099402593	-0.22241688	5.819513808	1.957973342	0.581175153	0.729303142	PREDICTED: conserved oligomeric Golgi complex subunit 3
RS005231	GO:0022892	substrate-specific transporter activity	MF	-0.391467446	-0.412384168	-0.179305009	4.071451755	1.941339648	0.584670698	0.731588315	PREDICTED: organic cation transporter protein
RS013007	GO:0022892	substrate-specific transporter activity	MF	-0.002282553	0.125821076	-0.165712957	6.097798672	1.893235166	0.59485901	0.740400689	PREDICTED: exportin-4-like
RS014789	GO:0022892	substrate-specific transporter activity	MF	-0.384309417	-0.340794963	-0.701560358	2.91880799	1.866727447	0.600523037	0.745342183	PREDICTED: zinc transporter ZIP9
RS001105	GO:0022892	substrate-specific transporter activity	MF	-0.219614449	-0.26220832	-0.327519535	7.371351073	1.827185164	0.609037083	0.751645402	PREDICTED: AP-1 complex subunit beta-1
RS010157	GO:0022892	substrate-specific transporter activity	MF	0.252451639	0.308689007	0.216067222	5.404322605	1.826234541	0.609242714	0.751719677	PREDICTED: exportin-T
RS005281	GO:0022892	substrate-specific transporter activity	MF	0.147692306	-0.427946659	0.130849224	2.285946009	1.819237088	0.610757704	0.752790867	PREDICTED: acid-sensing ion channel 4
RS008886	GO:0022892	substrate-specific transporter activity	MF	-0.289536803	-0.51024615	-0.479741606	2.079554922	1.757477457	0.624231737	0.763037416	PREDICTED: facilitated trehalose transporter Tret1
RS014536	GO:0022892	substrate-specific transporter activity	MF	-0.137268948	-0.07750242	-0.358531119	4.210868086	1.71200184	0.634268817	0.771080465	PREDICTED: cyclic nucleotide-gated cation channel beta-3
RS010358	GO:0022892	substrate-specific transporter activity	MF	0.306976969	0.175025435	-0.575621634	0.810862965	1.706529117	0.6354832	0.771892784	PREDICTED: neuroglobin-like
RS015410	GO:0022892	substrate-specific transporter activity	MF	-0.258061334	-0.246025988	-0.260658269	5.743258539	1.692882375	0.638517364	0.773443079	PREDICTED: protein slowmo
RS012406	GO:0022892	substrate-specific transporter activity	MF	-0.118916496	-0.22943197	0.145617021	8.722210769	1.650202623	0.648061031	0.779972717	PREDICTED: probable phospholipid-transporting ATPase IIB
RS015165	GO:0022892	substrate-specific transporter activity	MF	0.203835116	0.086955701	-0.092770826	6.68983845	1.645463293	0.649125814	0.780587272	PREDICTED: sodium channel protein 60E
RS003602	GO:0022892	substrate-specific transporter activity	MF	-0.963739329	-0.484991816	-0.758608967	0.476696176	1.634124817	0.651677236	0.782683875	PREDICTED: solute carrier family 28 member 3
RS010427	GO:0022892	substrate-specific transporter activity	MF	-0.256640259	-0.293251267	-0.831731644	1.070368751	1.607645315	0.657657541	0.78679007	PREDICTED: glutamate receptor ionotropic, NMDA 2B
RS012989	GO:0022892	substrate-specific transporter activity	MF	0.065692611	0.13851955	0.297427673	4.628786845	1.545719967	0.671759087	0.796098047	PREDICTED: snurportin-1
RS004131	GO:0022892	substrate-specific transporter activity	MF	0.244077702	0.428184836	0.166004547	3.05086288	1.47403705	0.688275398	0.807916894	PREDICTED: synaptic vesicle 2-related protein
RS014210	GO:0022892	substrate-specific transporter activity	MF	-0.099864709	-0.261063694	-0.008158269	5.601094807	1.464186895	0.690560295	0.809316615	PREDICTED: importin subunit alpha-7
RS003990	GO:0022892	substrate-specific transporter activity	MF	-0.055368209	0.238070575	0.293320892	4.25914611	1.454595738	0.692788544	0.811066898	PREDICTED: UDP-galactose translocator
RS009066	GO:0022892	substrate-specific transporter activity	MF	-0.284333504	-0.421360118	-0.423891407	2.706723454	1.434128224	0.697554745	0.814482149	PREDICTED: solute carrier family 35 member E3-like
RS015498	GO:0022892	substrate-specific transporter activity	MF	0.184679301	0.472371696	0.26432428	2.413896137	1.414695214	0.702093736	0.818113296	PREDICTED: calcium uniporter protein, mitochondrial
RS014968	GO:0022892	substrate-specific transporter activity	MF	0.070753676	0.338611082	0.008973976	4.167384444	1.401868016	0.705096916	0.82085326	PREDICTED: solute carrier family 41 member 1-like
RS011446	GO:0022892	substrate-specific transporter activity	MF	-0.226602437	0.017489836	-0.173735734	4.50551615	1.392641248	0.707260567	0.822459795	PREDICTED: stress-activated protein kinase JNK
RS005975	GO:0022892	substrate-specific transporter activity	MF	0.184305527	-0.340054217	-0.375631512	2.198485768	1.388734626	0.708177509	0.823222037	PREDICTED: piezo-type mechanosensitive ion channel component

RS004705	GO:0022892	substrate-specific transporter activity	MF	-0.430701847	-0.465359916	-0.484712083	3.064343052	1.370281204	0.712515526	0.826890945	PREDICTED: sodium/hydrogen exchanger 7
RS001366	GO:0022892	substrate-specific transporter activity	MF	-0.202685308	-0.234557446	-0.316543451	6.418554265	1.353303228	0.71651627	0.829698961	PREDICTED: glutamate receptor ionotropic, kainate 2
RS010673	GO:0022892	substrate-specific transporter activity	MF	0.194686816	0.144745443	0.237663354	5.4673327	1.348021968	0.717762586	0.830648431	PREDICTED: zinc transporter foi-like
RS005974	GO:0022892	substrate-specific transporter activity	MF	-0.266864024	-0.195937093	-0.171697422	7.122329353	1.335637313	0.720688539	0.832387331	PREDICTED: piezo-type mechanosensitive ion channel component
RS009390	GO:0022892	substrate-specific transporter activity	MF	-0.711993959	-0.803385471	-0.355292865	0.366452897	1.289104566	0.731721917	0.839984739	PREDICTED: gamma-aminobutyric acid receptor alpha-like
RS002882	GO:0022892	substrate-specific transporter activity	MF	0.362043543	0.12287268	0.14951259	3.567153224	1.277605364	0.734457649	0.842342193	PREDICTED: putative sodium-coupled neutral amino acid transporter 7
RS015540	GO:0022892	substrate-specific transporter activity	MF	-0.463597962	-0.808832157	-0.297537157	1.427647784	1.257020479	0.73936339	0.845811839	PREDICTED: zinc transporter ZIP11
RS013953	GO:0022892	substrate-specific transporter activity	MF	-0.420067551	-0.07959435	-0.044577109	2.09881014	1.242877248	0.742739999	0.848223421	PREDICTED: two pore potassium channel protein sup-9
RS014987	GO:0022892	substrate-specific transporter activity	MF	-0.129443831	0.074498835	0.080188563	6.502399376	1.215933035	0.749185503	0.85187288	PREDICTED: vacuolar protein sorting-associated protein 26B-like
RS000297	GO:0022892	substrate-specific transporter activity	MF	-0.078397003	0.556951886	0.159163891	1.385608421	1.213828293	0.749689662	0.85220612	PREDICTED: probable glutamate receptor
RS008405	GO:0022892	substrate-specific transporter activity	MF	-0.507380646	-0.391727947	-0.160990784	2.501471485	1.183658987	0.75692617	0.857267824	PREDICTED: solute carrier family 12 member 9
RS013461	GO:0022892	substrate-specific transporter activity	MF	0.139158606	-0.101013347	0.199986874	6.071677902	1.15813645	0.763061416	0.861033552	PREDICTED: ATP-binding cassette sub-family G member 5
RS010672	GO:0022892	substrate-specific transporter activity	MF	-0.302029346	0.22206596	0.191043022	1.595359022	1.095814464	0.778084584	0.871039755	Zinc transporter 2
RS003408	GO:0022892	substrate-specific transporter activity	MF	0.14875062	-0.250114218	0.329780306	2.915781806	1.09604514	0.778028887	0.871039755	PREDICTED: probable glutamate receptor
RS011943	GO:0022892	substrate-specific transporter activity	MF	0.336788752	0.302655858	-0.329953803	0.718404792	1.062395748	0.786158762	0.876649854	PREDICTED: glutamate receptor ionotropic, delta-1-like
RS001346	GO:0022892	substrate-specific transporter activity	MF	-0.305712719	-0.459994451	-0.225727855	2.010578526	1.048797397	0.789446703	0.878744111	PREDICTED: glutamate receptor ionotropic, delta-1-like
RS004706	GO:0022892	substrate-specific transporter activity	MF	-0.281248638	-0.101038833	-0.415774939	3.347688564	1.047260677	0.789818336	0.878882504	PREDICTED: sodium/hydrogen exchanger 7
RS003711	GO:0022892	substrate-specific transporter activity	MF	-0.209105883	-0.210486061	-0.224489958	5.489527424	1.011980558	0.798353047	0.8841416	PREDICTED: AP-2 complex subunit alpha
RS007717	GO:0022892	substrate-specific transporter activity	MF	0.066529061	0.463299961	0.014408782	1.808915589	0.956769727	0.811710752	0.892106596	PREDICTED: V-type proton ATPase 21 kDa proteolipid subunit
RS006075	GO:0022892	substrate-specific transporter activity	MF	-0.226394382	-0.065362369	0.088572196	3.008430896	0.898142308	0.82587609	0.901150881	PREDICTED: acetyl-coenzyme A transporter 1
RS008929	GO:0022892	substrate-specific transporter activity	MF	-0.139724103	-0.236598473	-0.091823273	4.57881475	0.895160984	0.826595415	0.901623303	PREDICTED: cytochrome c oxidase assembly protein COX15 homolog
RS011500	GO:0022892	substrate-specific transporter activity	MF	-0.54663125	-0.559810746	-0.038971768	0.470844775	0.876166842	0.831175153	0.904842	PREDICTED: zinc transporter ZIP8
RS013172	GO:0022892	substrate-specific transporter activity	MF	0.064707769	0.050177661	0.256276698	4.54520608	0.870549691	0.832528393	0.905630734	PREDICTED: N-acetyl-D-glucosamine kinase
RS001970	GO:0022892	substrate-specific transporter activity	MF	0.084033429	-0.012169719	-0.157558613	5.885614174	0.833122903	0.84152916	0.911638874	PREDICTED: glutamate receptor ionotropic, kainate 2
RS005586	GO:0022892	substrate-specific transporter activity	MF	0.165076617	-0.125717332	0.501179805	0.440219349	0.817686596	0.845232123	0.914113642	big brain
RS006019	GO:0022892	substrate-specific transporter activity	MF	0.016800687	-0.569398535	-0.206623396	0.539126613	0.775635116	0.855285471	0.920559373	PREDICTED: cationic amino acid transporter 2
RS000298	GO:0022892	substrate-specific transporter activity	MF	-0.477815194	-0.328207506	-0.189725936	1.140735658	0.76073894	0.858832832	0.922721437	PREDICTED: glutamate receptor ionotropic, NMDA 3A-like
RS002865	GO:0022892	substrate-specific transporter activity	MF	0.139107175	-0.024859854	-0.057863489	4.933967608	0.745084535	0.862551797	0.925368639	PREDICTED: glycolipid transfer protein
RS000066	GO:0022892	substrate-specific transporter activity	MF	-0.032220815	-0.172850098	-0.01678773	5.03444559	0.692162711	0.875045792	0.932661367	PREDICTED: vacuolar protein sorting-associated protein 29
RS010574	GO:0022892	substrate-specific transporter activity	MF	-0.354727613	-0.373915239	-0.481676921	0.828534929	0.681580005	0.877527727	0.933866301	PREDICTED: G protein-activated inward rectifier potassium channel 3-like
RS001040	GO:0022892	substrate-specific transporter activity	MF	-0.098603565	-0.201658646	0.076364834	3.089669028	0.652037571	0.884422889	0.938003319	PREDICTED: sideroflexin-2
RS003957	GO:0022892	substrate-specific transporter activity	MF	0.345462579	0.413965389	0.154609396	1.754535313	0.612321805	0.893605875	0.942823504	PREDICTED: solute carrier family 2, facilitated glucose transporter member 1-like
RS003729	GO:0022892	substrate-specific transporter activity	MF	-0.067068908	-0.31722074	-0.453287028	1.039779299	0.608180172	0.894557183	0.943318527	PREDICTED: neuronal acetylcholine receptor subunit alpha-7-like
RS003394	GO:0022892	substrate-specific transporter activity	MF	0.180289073	0.135383955	0.089380518	6.008505153	0.597460048	0.89701359	0.944722291	PREDICTED: vesicle transport protein USE1
RS001538	GO:0022892	substrate-specific transporter activity	MF	0.320201018	0.172065415	-0.020378547	1.614496317	0.588836165	0.898983255	0.94603515	PREDICTED: allatostatin-A receptor-like
RS014940	GO:0022892	substrate-specific transporter activity	MF	0.00473671	-0.350880149	-0.354295712	0.736422598	0.582482205	0.900430699	0.946555742	PREDICTED: potassium voltage-gated channel protein Shal
RS005560	GO:0022892	substrate-specific transporter activity	MF	0.073648066	0.070694895	-0.087436803	5.960124723	0.555903739	0.906448712	0.950525819	PREDICTED: protein catecholamines up
RS007670	GO:0022892	substrate-specific transporter activity	MF	0.130593495	0.003348502	0.052901794	6.519330394	0.542845203	0.90938257	0.952321145	PREDICTED: importin-9
RS004110	GO:0022892	substrate-specific transporter activity	MF	-0.109326082	-0.245764423	-0.393853677	2.856144774	0.536964028	0.910698682	0.95276119	PREDICTED: phospholipase A2 inhibitor and Ly6/PLAUR domain-containing protein
RS004521	GO:0022892	substrate-specific transporter activity	MF	-0.132126314	-0.000916743	-0.081420203	5.759811737	0.517058893	0.915127978	0.955543481	PREDICTED: general vesicular transport factor p115
RS001839	GO:0022892	substrate-specific transporter activity	MF	-0.04688838	-0.096047629	-0.142499158	5.750316042	0.476768464	0.923964819	0.960258535	PREDICTED: vacuolar protein sorting-associated protein 35
RS000077	GO:0022892	substrate-specific transporter activity	MF	0.022424805	0.042099988	-0.112334789	7.781323573	0.465871302	0.926322751	0.961835991	PREDICTED: AP-2 complex subunit mu
RS009649	GO:0022892	substrate-specific transporter activity	MF	-0.036772899	-0.220878583	-0.152031025	3.870532721	0.442143891	0.931405334	0.964172183	PREDICTED: transportin-2
RS000285	GO:0022892	substrate-specific transporter activity	MF	0.119687123	0.031475064	-0.04428715	4.541388379	0.422896761	0.935472985	0.966238561	PREDICTED: facilitated trehalose transporter Tret1-like
RS014584	GO:0022892	substrate-specific transporter activity	MF	0.129219334	0.060601072	0.090579059	6.064060989	0.417243203	0.936657865	0.966828377	PREDICTED: wolframin
RS002672	GO:0022892	substrate-specific transporter activity	MF	0.112781414	0.136232963	0.12426065	4.219978077	0.286688066	0.96251234	0.980258807	PREDICTED: mitochondrial import inner membrane translocase subunit Tim22
RS007404	GO:0022892	substrate-specific transporter activity	MF	0.341548622	0.423696565	0.486833568	3.079113058	0.267214539	0.9660718	0.982215891	PREDICTED: sodium-independent sulfate anion transporter-like
RS009012	GO:0022892	substrate-specific transporter activity	MF	0.165169583	0.002447419	-0.023721248	2.359677794	0.265786109	0.966329283	0.982398368	PREDICTED: zinc transporter 2-like
RS010152	GO:0022892	substrate-specific transporter activity	MF	-0.129723253	-0.161823215	-0.203053025	1.88639679	0.263658934	0.966711776	0.98246999	PREDICTED: zinc transporter ZIP3
RS007304	GO:0022892	substrate-specific transporter activity	MF	-0.03874979	-0.117709759	-0.024621974	4.815387852	0.237457293	0.971327222	0.985126901	PREDICTED: solute carrier family 17 member 9
RS002585	GO:0022892	substrate-specific transporter activity	MF	0.061337763	0.015683494	0.119579633	4.655608643	0.205533408	0.976691112	0.987602807	PREDICTED: mitochondrial pyruvate carrier 1
RS008629	GO:0022892	substrate-specific transporter activity	MF	0.051370738	-0.258316351	-0.177872255	0.376549123	0.205807073	0.976646438	0.987602807	PREDICTED: probable glutamate receptor
RS006774	GO:0022892	substrate-specific transporter activity	MF	-0.005561161	-0.002818033	0.022604747	3.411433614	0.005716578	0.999885243	0.999885243	PREDICTED: equilibrative nucleoside transporter 1
RS004555	GO:0044699	single-organism process	BP	-1.77422606	10.14745237	0.20411529	4.914736893	836.3719593	5.5949E-181	4.1449E-178	PREDICTED: cuticle protein 19-like
RS009563	GO:0044699	single-organism process	BP	2.374454834	10.18887902	1.125958506	8.244301045	812.4741887	8.5281E-176	5.3701E-173	PREDICTED: glycine-rich cell wall structural protein
RS009564	GO:0044699	single-organism process	BP	0.230716979	10.22816175	3.600547482	6.659804203	691.4792195	1.4779E-149	6.6475E-147	PREDICTED: proline-rich extensin-like protein EPR1
RS004559	GO:0044699	single-organism process	BP	5.645078627	11.73038235	5.038078452	4.755641597	518.5309297	4.5983E-112	1.2065E-109	PREDICTED: cuticle protein 19-like
RS011720	GO:0044699	single-organism process	BP	-2.78993926	6.666162583	5.530650553	4.728455414	504.2844383	5.6255E-109	1.3892E-106	PREDICTED: glucose dehydrogenase [FAD, quinone]
RS011718	GO:0044699	single-organism process	BP	-0.059496373	8.716087878	3.96160781	4.311804415	501.7385592	2.004E-108	4.762E-106	PREDICTED: glucose dehydrogenase [FAD, quinone]
RS001828	GO:0044699	single-organism process	BP	1.084411871	7.432507455	1.171021987	8.21689075	467.9453916	4.2161E-101	9.6541E-99	UPF0667 protein C1orf55-like
RS013971	GO:0044699	single-organism process	BP	0.699864391	6.735423711	1.2897367	5.600344024	422.6098593	2.80136E-91	5.51255E-89	PREDICTED: larval cuticle protein A2B-like
RS011049	GO:0044699	single-organism process	BP	-2.29908087	3.299439657	8.25278355	7.490502686	408.7578628	2.80601E-88	5.12158E-86	PREDICTED: cysteine sulfinic acid decarboxylase
RS013142	GO:0044699	single-organism process	BP	4.457376693	6.040934995	1.867046236	5.863115196	407.5103843	5.22776E-88	9.27301E-86	Spermatogenesis-associated protein 18-like protein
RS006489	GO:0044699	single-organism process	BP	2.510686859	5.223337247	0.044092099	6.002257611	377.9796278	1.30192E-81	2.18619E-79	PREDICTED: probable nuclear hormone receptor HR3
RS000375	GO:0044699	single-organism process	BP	-0.34491686	5.884963026	2.082677396	7.234288024	328.8906103	5.54743E-71	7.7627E-69	putative allantoinase 1
RS000202	GO:0044699	single-organism process	BP	-1.20201424	6.865435341	3.307157572	6.02876929	323.5281227	8.03513E-70	1.08811E-67	PREDICTED: probable cytochrome P450 49a1
RS011560	GO:0044699	single-organism process	BP	-2.826521884	1.009412093	3.934530665	5.719581406	321.6146418	2.08555E-69	2.79419E-67	PREDICTED: dehydrogenase/reductase SDR family member 11-like
RS001531	GO:0044699	single-organism process	BP	-0.568996735	7.455231832	2.891986124	6.325697782	314.7812613	6.28688E-68	8.16258E-66	PREDICTED: clavesin-1
RS002054	GO:0044699	single-organism process	BP	0.655571472	3.958444332	0.432136406	5.231550382	314.0290944	9.1464E-68	1.1519E-65	PREDICTED: laccase-4-like
RS010208	GO:0044699	single-organism process	BP	-0.084960135	7.892665833	1.075100468	4.896692372	313.5860255	1.14066E-67	1.42233E-65	PREDICTED: acyl-CoA Delta(11) desaturase-like
RS013784	GO:0044699	single-organism process	BP	3.358922598	9.954724644	4.200151343	2.991428299	310.2015642	6.16228E-67	7.53474E-65	Cytochrome P450 4C1
RS012128	GO:0044699	single-organism process	BP	1.527833822	8.238015641	3.354969162	3.846530051	299.8011385	1.09852E-64	1.281E-62	Chorion peroxidase
RS001948	GO:0044699	single-organism process	BP	-0.792763253	2.803258949	1.759563877	6.650188786	291.8180989	5.86789E-63	6.65767E-61	Major facilitator superfamily domain-containing protein 6-A
RS007652	GO:0044699	single-organism process	BP	0.585883623	0.528505008	4.851177054	4.584889963	291.2117195	7.93792E-63	8.92591E-61	PREDICTED: solute carrier family 26 member 10-like
RS005246	GO:0044699	single-organism process	BP	-1.039759025	2.52725738	3.661947996	5.83723879	286.4562689	8.48768E-62	9.29512E-60	PREDICTED: high-affinity choline transporter 1-like
RS002523	GO:0044699	single-organism process	BP	-0.106349467	2.390006294	4.141325792	6.188052257	274.8835771	2.70941E-59	2.79692E-57	PREDICTED: putative fatty acyl-CoA reductase CG5065
RS014534	GO:0044699	single-organism process	BP	-0.920891257	5.10098414	5.913154476	4.616080779	274.3087891	3.60776E-59	3.694E-57	PREDICTED: larval cuticle protein A3A
RS013281	GO:0044699	single-organism process	BP	-0.67475512	7.412691306	-0.864970782	5.931563279	274.2657315	3.68599E-59	3.74366E-57	osiris 9
RS001671	GO:0044699	single-organism process	BP	1.607767839	7.027479089	1.35245618	3.520751217	273.0690443	6.69065E-59	6.74097E-57	catalase
RS013966	GO:0044699	single-organism process	BP	-1.184955047	6.847280362	6.132025519	5.068962085	269.9551868	3.15618E-58	3.12984E-56	PREDICTED: larval cuticle protein A2B-like
RS008116	GO:0044699	single-organism process	BP	-1.267428214	1.552900586	3.203973031	7.311161645	266.5897912	1.6875E-57	1.61003E-55	PREDICTED: dehydrogenase/reductase SDR family member 7
RS004543	GO:0044699	single-organism process	BP	1.737579014	10.48554524	0	3.477978359	257.893392	1.2838E-55	1.18883E-53	PREDICTED: cuticle protein 19-like
RS005133	GO:0044699	single-organism process	BP	0.479107556	4.239486439	2.055495409	6.672951297	251.9385588	2.49194E-54	2.22578E-52	PREDICTED: activating signal cointegrator 1 complex subunit 2 homolog
RS012132	GO:0044699	single-organism process	BP	-0.641537099	0.463569382	4.164552381	6.993055111	251.5268374	3.05907E-54	2.69412E-52	PREDICTED: putative inorganic phosphate cotransporter
RS007037	GO:0044699	single-organism process	BP	0.123547036	8.1085325	2.157110744	6.427068403	246.1743028	4.39789E-53	3.69247E-51	CG4702
RS000976	GO:0044699	single-organism process	BP	-5.16065417	-1.385229108	4.799547049	3.069597401	243.5736887	1.60573E-52	1.33924E-50	neuropeptide like precursor 3-like
RS014505	GO:0044699	single-organism process	BP	-1.520148085	-1.804455673	4.078799864	3.578432441	240.6616227	6.84588E-52	5.59851E-50	AAEL007250-PA
RS009299	GO:0044699	single-organism process	BP	-0.833536193	5.147671256	1.07098716	3.418953047	224.4882423	2.15009E-48	1.67149E-46	PREDICTED: organic cation transporter protein-like
RS015506	GO:0044699	single-organism process	BP	0.832991555	4.202066924	3.727868957	6.35398696	220.1468977	1.86623E-47	1.41586E-45	PREDICTED: nose resistant to fluoxetine protein 6
RS003905	GO:0044699	single-organism process	BP	-1.535395853	-3.809880637	1.439384598	5.164045266	219.9356219	2.07317E-47	1.56345E-45	PREDICTED: facilitated trehalose transporter Tret1-like
RS005672	GO:0044699	single-organism process	BP	-1.733793607	4.733583232	2.714558296	5.986161479	215.2357865	2.15052E-46	1.57463E-44	PREDICTED: probable cytochrome P450 49a1
RS004931	GO:0044699	single-organism process	BP	-0.974873999	2.736457506	0.991127956	5.448489905	213.8177257	4.35562E-46	3.15257E-44	PREDICTED: inactive hydroxysteroid dehydrogenase-like protein 1
RS009630	GO:0044699	single-organism process	BP	-3.7994905	1.185051274	4.347466708	6.191232232	208.8321844	5.2068E-45	3.70477E-43	PREDICTED: protein lethal(3)malignant blood neoplasm 1
RS005312	GO:0044699	single-organism process	BP	2.694423701	11.36569557	6.88574002	9.476352687	205.0586546	3.40456E-44	2.38206E-42	PREDICTED: glycine-rich cell wall structural protein
RS010242	GO:0044699	single-organism process	BP	-1.845535492	-4.484162024	-2.219215826	7.022944235	191.9213788	2.3472E-41	1.53961E-39	PREDICTED: 15-hydroxyprostaglandin dehydrogenase [NAD(+)]-like
RS009746	GO:0044699	single-organism process	BP	-2.752522237	-5.141049143	-2.028825144	5.976115232	190.5917258	4.54764E-41	2.96751E-39	PREDICTED: sphingomyelin phosphodiesterase-like
RS005356	GO:0044699	single-organism process	BP	-1.113173524	4.433335808	-1.46018865	4.569036402	189.7047295	7.06955E-41	4.51949E-39	PREDICTED: probable cytochrome P450 303a1
RS006639	GO:0044699	single-organism process	BP	-3.083868425	-7.637695989	-8.133777043	4.628300865	186.0851837	4.27786E-40	2.64095E-38	PREDICTED: aromatic-L-amino-acid decarboxylase-like
RS012687	GO:0044699	single-organism process	BP	-2.409771896	-4.903293647	-1.083108244	4.98106813	185.3939853	6.03281E-40	3.7062E-38	endo-beta-1,4-glucanase
RS015531	GO:0044699	single-organism process	BP	0.046825306	2.516345677	0.523451541	5.739280554	184.8461325	7.92225E-40	4.81994E-38	PREDICTED: patched domain-containing protein 3
RS010587	GO:0044699	single-organism process	BP	-0.817567239	1.406623819	2.936998472	6.643230199	180.2959822	7.61285E-39	4.52246E-37	PREDICTED: dual oxidase maturation factor 1
RS003967	GO:0044699	single-organism process	BP	1.184875011	3.494756351	6.294613809	9.437666813	175.8735837	6.86318E-38	3.8587E-36	ebony
RS003373	GO:0044699	single-organism process	BP	2.394141754	0.518855653	-1.476597425	5.561080032	174.1882806	1.58642E-37	8.84041E-36	PREDICTED: UDP-glucuronosyltransferase 2C1-like
RS009243	GO:0044699	single-organism process	BP	-0.589487895	2.064901326	2.91752274	4.715025796	173.1275538	2.68806E-37	1.47832E-35	PREDICTED: multidrug resistance protein homolog 49
RS014063	GO:0044699	single-organism process	BP	-2.219641238	3.697202499	2.654852855	9.863295277	166.236498	8.2622E-36	4.35373E-34	PREDICTED: endocuticle structural glycoprotein SgAbd-5
RS009074	GO:0044699	single-organism process	BP	-0.999061123	1.569100076	2.399331412	5.711615811	162.1907409	6.1707E-35	3.12104E-33	PREDICTED: solute carrier family 46 member 3-like
RS006382	GO:0044699	single-organism process	BP	2.806378091	4.140514995	2.619769373	8.160379228	161.4739314	8.81125E-35	4.43876E-33	PREDICTED: chitooligosaccharidolytic beta-N-acetylglucosaminidase
RS011712	GO:0044699	single-organism process	BP	-1.230020342	4.072791347	0.764053697	2.678048388	161.0891676	1.06678E-34	5.35262E-33	PREDICTED: glucose dehydrogenase [FAD, quinone]
RS014580	GO:0044699	single-organism process	BP	-2.976523608	-6.808866752	-1.942701116	5.214122185	156.927034	8.43836E-34	4.16756E-32	PREDICTED: kynurenine formamidase-like
RS005837	GO:0044699	single-organism process	BP	-3.657782779	-9.217465737	-5.915245655	4.470527867	156.4779613	1.05477E-33	5.16879E-32	PREDICTED: gastrin-releasing peptide receptor-like
RS010164	GO:0044699	single-organism process	BP	-2.449946147	-5.759887047	-4.69205969	6.572436586	156.0284659	1.31871E-33	6.38761E-32	PREDICTED: cytochrome P450 6k1-like

RS013327	GO:0044699	single-organism process	BP	4.160581503	4.46067403	1.925626339	4.485054186	152.472056	7.71738E-33	3.62659E-31	PREDICTED: latent-transforming growth factor beta-binding protein 4
RS004549	GO:0044699	single-organism process	BP	-0.718067112	5.195357268	-0.92260103	2.031520878	150.6729405	1.88625E-32	8.76583E-31	PREDICTED: cuticle protein 8
RS010601	GO:0044699	single-organism process	BP	-2.057712014	-5.959536073	-2.672840145	5.417440088	149.9236278	2.73679E-32	1.25792E-30	PREDICTED: cytochrome P450 9e2-like
RS009516	GO:0044699	single-organism process	BP	-1.060411828	2.092027896	2.629324285	7.147037886	149.0647342	4.19292E-32	1.90634E-30	PREDICTED: putative fatty acyl-CoA reductase CG5065
RS000766	GO:0044699	single-organism process	BP	2.752254264	5.580693581	4.191992628	7.600438501	148.294296	6.14759E-32	2.77501E-30	PREDICTED: hormone receptor 4
RS002966	GO:0044699	single-organism process	BP	-1.449397511	4.316039174	4.18727517	6.997406999	148.2173215	6.38717E-32	2.86263E-30	PREDICTED: peroxidase
RS011942	GO:0044699	single-organism process	BP	-0.498390242	3.974481158	0.858281392	5.803436912	146.0821711	1.84434E-31	8.15005E-30	PREDICTED: 2-hydroxyacylsphingosine 1-beta-galactosyltransferase
RS003129	GO:0044699	single-organism process	BP	-1.935857211	0.013218622	3.698610157	4.556804627	145.8197805	2.10104E-31	9.2418E-30	PREDICTED: organic cation transporter protein
RS012684	GO:0044699	single-organism process	BP	-3.104964921	-3.782144518	-3.589335926	3.211411943	144.6587701	3.73965E-31	1.61846E-29	endo-beta-1,4-glucanase
RS004606	GO:0044699	single-organism process	BP	-0.152775887	3.199032258	1.582974114	7.379011967	144.4003621	4.25168E-31	1.83376E-29	PREDICTED: peptide transporter family 1
RS015149	GO:0044699	single-organism process	BP	-0.699191537	2.926752741	0.224484556	3.215733009	140.1439713	3.51898E-30	1.47236E-28	PREDICTED: chaoptin
RS008125	GO:0044699	single-organism process	BP	-2.416273617	4.060119856	1.427250248	9.208999704	139.6656385	4.46225E-30	1.86085E-28	PREDICTED: endocuticle structural glycoprotein ABD-4
RS001262	GO:0044699	single-organism process	BP	-2.399000696	4.824243006	1.184902522	2.908556297	139.6588544	4.4773E-30	1.86096E-28	PREDICTED: elongation of very long chain fatty acids protein AAEL008004
RS006854	GO:0044699	single-organism process	BP	6.283120461	6.552275357	-0.537889305	5.75451878	138.70125	7.20252E-30	2.98383E-28	AAEL007579-PA
RS002686	GO:0044699	single-organism process	BP	2.763811404	2.611957119	-0.723707642	4.513758678	135.3970454	3.71383E-29	1.48013E-27	PREDICTED: micronuclear linker histone polyprotein
RS005111	GO:0044699	single-organism process	BP	3.78451671	3.022726549	-0.512162157	3.220438756	134.9581256	4.61783E-29	1.8174E-27	PREDICTED: enhancer of split malpha protein-like
RS004125	GO:0044699	single-organism process	BP	1.407227463	2.012417871	-0.423642507	5.906215214	131.7530272	2.26607E-28	8.8083E-27	PREDICTED: ABC transporter G family member 9
RS003669	GO:0044699	single-organism process	BP	-0.372973382	3.273118337	-1.095397675	2.330404728	128.9803887	8.97006E-28	3.4337E-26	PREDICTED: retinol dehydrogenase 12-like
RS003386	GO:0044699	single-organism process	BP	0.126302997	-0.926691146	2.846839447	6.952013651	127.4048432	1.96016E-27	7.39111E-26	PREDICTED: alcohol dehydrogenase [NADP(+)]
RS009442	GO:0044699	single-organism process	BP	-2.053221047	-0.511261449	3.451856895	7.01573551	127.3138567	2.05068E-27	7.70932E-26	PREDICTED: E3 ubiquitin-protein ligase SIAH1-like
RS010487	GO:0044699	single-organism process	BP	0.299211974	1.908799826	3.01367764	7.532839574	126.2279928	3.51446E-27	1.30564E-25	PREDICTED: probable chitinase 3
RS000536	GO:0044699	single-organism process	BP	3.948686772	5.799780814	5.453271459	8.019750929	123.4676022	1.38211E-26	4.95906E-25	PREDICTED: LIRP-like
RS011221	GO:0044699	single-organism process	BP	1.299997407	3.647486912	0.869752941	6.470449193	122.767789	1.95565E-26	6.97718E-25	PREDICTED: protein eiger
RS000212	GO:0044699	single-organism process	BP	-1.359638547	-4.566079113	-2.08395694	4.979139945	122.5054278	2.22743E-26	7.92438E-25	cytochrome P450
RS010066	GO:0044699	single-organism process	BP	-2.152102653	0.574586566	2.415584484	5.620353404	119.856004	8.28798E-26	2.90749E-24	PREDICTED: collagen alpha-1(I) chain
RS004452	GO:0044699	single-organism process	BP	-0.736699183	-0.175370284	2.422374147	4.446989456	119.427265	1.02514E-25	3.56646E-24	PREDICTED: tyrosine-protein kinase Src42A
RS013125	GO:0044699	single-organism process	BP	2.481132043	2.876581195	-0.019668684	4.44585997	118.6121689	1.53573E-25	5.32811E-24	PREDICTED: GTP-binding protein RAD
RS003150	GO:0044699	single-organism process	BP	-1.696898363	-4.037494955	-1.303342477	4.152151436	116.6159855	4.13197E-25	1.41408E-23	PREDICTED: probable cytochrome P450 6a14
RS003584	GO:0044699	single-organism process	BP	-1.221802531	0.359585466	1.950408164	5.803684621	116.60715	4.15011E-25	1.41644E-23	PREDICTED: monocarboxylate transporter 12
RS006867	GO:0044699	single-organism process	BP	1.545187713	9.468733383	7.242700694	4.540942609	116.1783728	5.13311E-25	1.7472E-23	PREDICTED: soluble guanylate cyclase 89Db-like
RS002050	GO:0044699	single-organism process	BP	-2.617490939	-7.068375023	-3.318747569	6.701449754	115.4815057	7.25135E-25	2.46155E-23	laccase-2
RS003228	GO:0044699	single-organism process	BP	-0.561103954	2.12048168	1.83683218	5.406987214	114.6440514	1.09829E-24	3.71824E-23	PREDICTED: b(0,+)-type amino acid transporter 1-like
RS009512	GO:0044699	single-organism process	BP	0.928691276	1.871849051	-0.285447106	6.651716807	113.8771328	1.60627E-24	5.38014E-23	PREDICTED: putative fatty acyl-CoA reductase CG5065
RS013159	GO:0044699	single-organism process	BP	-0.798180063	-1.858281034	2.789942887	3.101482083	112.2571221	3.58543E-24	1.19142E-22	PREDICTED: probable cytochrome P450 4aa1
RS013974	GO:0044699	single-organism process	BP	1.308057058	2.315023189	0.876327879	6.127768048	111.7248777	4.66769E-24	1.53887E-22	PREDICTED: monocarboxylate transporter 10
RS001968	GO:0044699	single-organism process	BP	-2.059672848	-5.407063175	-2.355052572	8.05739758	111.5421304	5.11018E-24	1.67163E-22	PREDICTED: prostaglandin reductase 1-like
RS011123	GO:0044699	single-organism process	BP	-0.064367361	6.039195805	1.496286249	1.119543417	111.0087632	6.6563E-24	2.16613E-22	PREDICTED: phospholipase A2
RS011719	GO:0044699	single-organism process	BP	-1.396212466	3.56799454	1.196979804	5.060980959	110.7270473	7.65358E-24	2.47152E-22	PREDICTED: glucose dehydrogenase [FAD, quinone]
RS014350	GO:0044699	single-organism process	BP	0.394221017	3.916656328	2.472104182	3.250648745	110.2945537	9.48297E-24	3.05444E-22	Matrix metalloproteinase-16
RS007016	GO:0044699	single-organism process	BP	-1.016570529	1.612262307	-0.298013395	6.087776173	110.0375814	1.07708E-23	3.44283E-22	PREDICTED: D-beta-hydroxybutyrate dehydrogenase, mitochondrial
RS001883	GO:0044699	single-organism process	BP	-2.384568661	-3.311370279	-1.801982984	5.447832656	109.4829382	1.41778E-23	4.47507E-22	PREDICTED: protein croquemort-like
RS001344	GO:0044699	single-organism process	BP	-1.224665802	0.504065929	2.789839008	4.816787403	108.7807423	2.00778E-23	6.30574E-22	PREDICTED: sodium/potassium/calcium exchanger 4
RS014484	GO:0044699	single-organism process	BP	-2.032033569	-3.025181863	-1.160613076	6.653281311	108.725895	2.06309E-23	6.46334E-22	PREDICTED: cytochrome P450 6k1-like
RS014587	GO:0044699	single-organism process	BP	1.404300629	3.384393819	0.219702824	4.326573332	107.7034868	3.42387E-23	1.06208E-21	PREDICTED: lysyl oxidase homolog 3
RS003506	GO:0044699	single-organism process	BP	2.500641759	2.214328227	-0.294085399	7.071545239	106.6773518	5.69245E-23	1.7443E-21	PREDICTED: tubulin beta chain-like
RS010975	GO:0044699	single-organism process	BP	-3.237644952	-3.683659794	-2.505401587	3.871263699	106.1678334	7.32688E-23	2.22349E-21	PREDICTED: cytochrome P450 4c3
RS005642	GO:0044699	single-organism process	BP	-0.158087639	3.50903035	0.033669894	7.188992405	105.9789213	8.04565E-23	2.4299E-21	PREDICTED: elongation of very long chain fatty acids protein 4-like
RS011319	GO:0044699	single-organism process	BP	-2.200812301	2.311506339	4.542956934	3.965152586	105.9687966	8.0861E-23	2.43628E-21	PREDICTED: elongation of very long chain fatty acids protein AAEL008004-like
RS011780	GO:0044699	single-organism process	BP	-1.685309235	-4.03663067	-0.682423211	4.573186577	105.6851221	9.30608E-23	2.78387E-21	PREDICTED: facilitated trehalose transporter Tret1-2 homolog
RS008286	GO:0044699	single-organism process	BP	1.416045047	2.242632072	-0.540840835	4.520196143	105.4062775	1.06845E-22	3.1811E-21	PREDICTED: toll-like receptor 13
RS004436	GO:0044699	single-organism process	BP	-1.6353822	-2.198323513	1.659074242	6.594585322	104.8919255	1.37848E-22	4.08485E-21	PREDICTED: monocarboxylate transporter 14
RS009523	GO:0044699	single-organism process	BP	0.933204298	3.492404054	0.512137378	2.728986468	104.3236738	1.82658E-22	5.38734E-21	PREDICTED: putative fatty acyl-CoA reductase CG5065
RS002214	GO:0044699	single-organism process	BP	2.792978943	3.034362842	0.560488122	3.879155465	104.1646791	1.97623E-22	5.81512E-21	PREDICTED: carbohydrate sulfotransferase 11-like
RS013663	GO:0044699	single-organism process	BP	-1.653643168	-4.62554876	-0.789996175	3.227127882	103.6938228	2.49527E-22	7.25762E-21	cytochrome P450
RS012767	GO:0044699	single-organism process	BP	-1.080176195	1.794194957	2.213308124	7.335239134	103.6003524	2.6135E-22	7.58397E-21	PREDICTED: D-beta-hydroxybutyrate dehydrogenase, mitochondrial
RS001246	GO:0044699	single-organism process	BP	-1.809510857	-3.746707307	-2.305954392	4.950153695	102.9321488	3.63867E-22	1.04624E-20	PREDICTED: gastrin-releasing peptide receptor-like
RS013283	GO:0044699	single-organism process	BP	3.670823051	9.162133659	0.973649447	3.986247177	102.9332907	3.63661E-22	1.04624E-20	osiris 7
RS014291	GO:0044699	single-organism process	BP	-1.581546423	-3.964154112	-2.419882402	4.827091015	102.7077738	4.06631E-22	1.16654E-20	PREDICTED: probable cytochrome P450 49a1
RS010490	GO:0044699	single-organism process	BP	-1.102168741	0.224078961	2.790482278	7.266154347	102.1283006	5.41784E-22	1.54023E-20	PREDICTED: UDP-N-acetylhexosamine pyrophosphorylase
RS000720	GO:0044699	single-organism process	BP	-1.414180497	1.484024302	-1.819002501	5.701350612	100.1058089	1.47484E-21	4.12758E-20	aquaporin
RS002557	GO:0044699	single-organism process	BP	1.71837254	0.555805156	-2.680377852	8.326223872	99.81567395	1.70266E-21	4.75462E-20	PREDICTED: ankyrin-1-like
RS009320	GO:0044699	single-organism process	BP	-3.489735764	-0.455154282	3.302382355	4.78831433	99.22062542	2.28596E-21	6.31346E-20	PREDICTED: superoxide dismutase [Cu-Zn]-like
RS010849	GO:0044699	single-organism process	BP	-1.356133269	-5.188301121	-4.010261417	3.745442042	99.05971644	2.47551E-21	6.822E-20	PREDICTED: glycine N-methyltransferase
RS003672	GO:0044699	single-organism process	BP	-2.021765402	-4.256841834	-0.243015197	4.841936154	98.14132048	3.90039E-21	1.06324E-19	Cytochrome P450 4C1
RS010608	GO:0044699	single-organism process	BP	-2.103950908	-3.081106465	-1.868132201	5.146780914	97.70861522	4.83202E-21	1.3087E-19	PREDICTED: cytochrome P450 9e2-like
RS010604	GO:0044699	single-organism process	BP	-2.596737331	-4.018149402	-1.968313631	2.892489978	96.73070525	7.84041E-21	2.1009E-19	PREDICTED: cytochrome P450 9e2-like
RS014619	GO:0044699	single-organism process	BP	0.135810517	1.948291035	0.695319866	6.46161189	96.1170343	1.06229E-20	2.83441E-19	PREDICTED: dual oxidase
RS009744	GO:0044699	single-organism process	BP	-0.046452796	6.48102378	2.439435417	1.487925442	95.45473823	1.4743E-20	3.92544E-19	PREDICTED: sphingomyelin phosphodiesterase-like
RS004588	GO:0044699	single-organism process	BP	0.455957454	2.020684969	0.541747519	6.211789985	95.36099226	1.54431E-20	4.09453E-19	PREDICTED: decaprenyl-diphosphate synthase subunit 2
RS014609	GO:0044699	single-organism process	BP	-0.53137706	3.86263829	1.755847184	3.509463235	95.29323245	1.59697E-20	4.22526E-19	GD21471
RS012045	GO:0044699	single-organism process	BP	-2.417547613	-3.846942398	-2.948472949	3.138267013	95.12499238	1.73561E-20	4.58246E-19	PREDICTED: sodium-independent sulfate anion transporter
RS002200	GO:0044699	single-organism process	BP	-2.534854462	-2.965618885	0.446701011	4.187537984	94.94596227	1.89639E-20	4.97566E-19	PREDICTED: ATP-binding cassette sub-family G member 4
RS003670	GO:0044699	single-organism process	BP	0	7.322187147	0	0.672496202	94.93746329	1.90439E-20	4.98625E-19	PREDICTED: retinol dehydrogenase 11-like
RS100017	GO:0044699	single-organism process	BP	-2.375948362	-6.540524088	-2.832559384	2.986513272	93.62826533	3.63991E-20	9.39365E-19	PREDICTED: geranylgeranyl pyrophosphate synthase
RS004449	GO:0044699	single-organism process	BP	-2.134330565	-2.618389909	-0.942621108	6.590990632	93.57612815	3.73502E-20	9.60647E-19	cytochrome P450
RS009651	GO:0044699	single-organism process	BP	-3.154372842	-4.783001161	-2.730899772	3.45854155	93.57471858	3.73763E-20	9.60647E-19	PREDICTED: maltase 1-like
RS013239	GO:0044699	single-organism process	BP	1.866830916	1.827955171	-0.150888215	4.857533717	91.49789483	1.04424E-19	2.60936E-18	PREDICTED: feline leukemia virus subgroup C receptor-related protein 2
RS001847	GO:0044699	single-organism process	BP	-2.509626326	-4.299827518	-0.240987804	4.342755604	91.2307621	1.19175E-19	2.96035E-18	Cytochrome P450 4C1
RS003347	GO:0044699	single-organism process	BP	1.380593252	1.266525956	3.318324244	5.492117256	91.01864054	1.32359E-19	3.24938E-18	PREDICTED: A disintegrin and metalloproteinase with thrombospondin motifs 18-like
RS001965	GO:0044699	single-organism process	BP	-0.943336884	-1.128601737	1.539172053	5.381812793	90.96116488	1.36176E-19	3.33657E-18	PREDICTED: venom carboxylesterase-6
RS004558	GO:0044699	single-organism process	BP	-5.510261675	-4.322944833	3.428707167	11.35830114	90.14866931	2.03528E-19	4.9579E-18	PREDICTED: cuticle protein 7
RS002322	GO:0044699	single-organism process	BP	-2.116430532	-0.063380901	1.840937811	5.106622206	89.82502084	2.38858E-19	5.76279E-18	PREDICTED: Krueppel-like factor 2
RS007494	GO:0044699	single-organism process	BP	-1.905744217	-3.744457543	0.74513791	6.016536186	89.73338778	2.49931E-19	6.00605E-18	PREDICTED: sodium-coupled monocarboxylate transporter 1
RS000265	GO:0044699	single-organism process	BP	-1.566831111	2.04965793	-0.177069096	2.908279706	89.72983325	2.50371E-19	6.00605E-18	PREDICTED: phospholipase A2
RS009515	GO:0044699	single-organism process	BP	-2.342451636	0.818736611	0.533407054	6.330219835	89.56330692	2.71863E-19	6.49686E-18	PREDICTED: putative fatty acyl-CoA reductase CG5065
RS003553	GO:0044699	single-organism process	BP	-1.896615492	-3.604139345	-1.858803184	8.22403702	88.21801298	5.28765E-19	1.24472E-17	PREDICTED: xanthine dehydrogenase/oxidase-like
RS010600	GO:0044699	single-organism process	BP	-1.965880285	-2.595180035	-2.025766755	3.501649819	88.03748055	5.78135E-19	1.35587E-17	PREDICTED: cytochrome P450 9e2-like
RS007080	GO:0044699	single-organism process	BP	-0.867799573	2.923130763	2.721632003	3.820195406	87.39968469	7.92467E-19	1.84821E-17	putative E3 ubiquitin-protein ligase sinah
RS010610	GO:0044699	single-organism process	BP	-2.192804333	-3.183357387	-2.464569112	5.200370422	87.3467206	8.13492E-19	1.89374E-17	PREDICTED: cytochrome P450 9e2-like
RS013889	GO:0044699	single-organism process	BP	-1.663206004	0.794911338	2.587607867	5.380228282	87.07326483	9.31253E-19	2.15989E-17	PREDICTED: sialin
RS014290	GO:0044699	single-organism process	BP	-0.136150141	-2.239974624	-1.856066463	6.291958295	86.88373783	1.02273E-18	2.36335E-17	PREDICTED: cytochrome P450 CYP12A2-like
RS004161	GO:0044699	single-organism process	BP	-0.641780189	2.854806475	3.433103727	4.043935479	86.6921254	1.12435E-18	2.59341E-17	PREDICTED: decaprenyl-diphosphate synthase subunit 1
RS003197	GO:0044699	single-organism process	BP	-1.902499307	-1.808382325	0.314261681	5.846256191	86.59882123	1.17742E-18	2.71087E-17	PREDICTED: estradiol 17-beta-dehydrogenase 2
RS004065	GO:0044699	single-organism process	BP	1.058473307	3.921943748	2.533827009	4.458300808	86.3745619	1.31546E-18	3.01766E-17	Pancreatic lipase-related protein 1
RS006477	GO:0044699	single-organism process	BP	-2.466158458	-0.106225577	1.489284681	8.740232495	86.0306644	1.55922E-18	3.57034E-17	yellow-c
RS001669	GO:0044699	single-organism process	BP	0.222207808	2.563484364	1.861666726	5.8935478	85.64961074	1.88239E-18	4.27922E-17	PREDICTED: UPF0676 protein C1494.01
RS009298	GO:0044699	single-organism process	BP	-2.524665679	0.320369244	1.997080797	2.729083611	85.53693786	1.99021E-18	4.50803E-17	PREDICTED: organic cation transporter protein-like
RS004448	GO:0044699	single-organism process	BP	-2.423580791	-5.214804966	-2.367165741	3.341394571	85.22354932	2.32365E-18	5.24445E-17	PREDICTED: cytochrome P450 4c3
RS009300	GO:0044699	single-organism process	BP	-0.334428722	2.777656488	1.996146097	5.84981916	85.14092159	2.42051E-18	5.44355E-17	PREDICTED: organic cation transporter protein-like
RS010908	GO:0044699	single-organism process	BP	-0.943181794	0.036107559	2.049952254	7.213001489	84.97338301	2.62948E-18	5.89246E-17	PREDICTED: ras-related and estrogen-regulated growth inhibitor
RS007234	GO:0044699	single-organism process	BP	0.052513072	1.943902873	1.008743446	6.066222799	84.95578614	2.65245E-18	5.93338E-17	PREDICTED: phosphoacetylglucosamine mutase
RS006480	GO:0044699	single-organism process	BP	-1.982319187	-2.457116034	-0.131752411	6.935989039	84.74132818	2.94903E-18	6.57347E-17	PREDICTED: protein 5NUC-like
RS001654	GO:0044699	single-organism process	BP	-2.493682135	-3.567429027	0.297619687	2.726724729	84.3718169	3.53989E-18	7.84884E-17	PREDICTED: catalase
RS013891	GO:0044699	single-organism process	BP	-3.03546779	0.898282569	1.370032508	5.013144301	84.17578439	3.9E-18	8.61695E-17	PREDICTED: sialin
RS013586	GO:0044699	single-organism process	BP	1.963091519	2.580507008	-0.247710486	3.610162984	84.11093426	4.02702E-18	8.882E-17	PREDICTED: GTP-binding protein RAD
RS003546	GO:0044699	single-organism process	BP	2.700076628	3.489431185	0.636738182	4.405317492	84.09974075	4.04935E-18	8.91566E-17	PREDICTED: amine oxidase [flavin-containing] A
RS000583	GO:0044699	single-organism process	BP	-0.501317303	3.210271228	0.843907421	2.742897173	83.99954617	4.2549E-18	9.33558E-17	PREDICTED: dehydrogenase/reductase SDR family member 11
RS007081	GO:0044699	single-organism process	BP	-3.228897268	-1.965361483	0.820827398	4.722808911	83.32123205	5.94927E-18	1.28737E-16	E3 ubiquitin-protein ligase SINAT3
RS005404	GO:0044699	single-organism process	BP	-1.657150549	-2.930223644	-1.37987084	5.26638522	83.27344041	6.09144E-18	1.31588E-16	PREDICTED: probable 3',5'-cyclic phosphodiesterase pde-5
RS011716	GO:0044699	single-organism process	BP	-6.01820819	3.489449018	2.81410635	3.308115746	83.10682532	6.61417E-18	1.42635E-16	PREDICTED: glucose dehydrogenase [FAD, quinone]-like
RS012299	GO:0044699	single-organism process	BP	-2.492567056	1.677811294	-0.786240613	4.794484547	83.06835031	6.74111E-18	1.45124E-16	Peroxidasin-like protein

RS005235	GO:0044699	single-organism process	BP	-0.316190702	0.021797675	2.848580935	8.976414933	82.72590425	7.98393E-18	1.71003E-16	PREDICTED: glutamine--fructose-6-phosphate aminotransferase [isomerizing] 2-like
RS014377	GO:0044699	single-organism process	BP	-2.879618381	-2.202364727	0.22442043	5.12611155	82.5458203	8.72689E-18	1.86282E-16	PREDICTED: UDP-glucuronosyltransferase 1-9-like
RS001885	GO:0044699	single-organism process	BP	-2.76489906	-4.755081501	-1.884555645	2.547127138	82.44560035	9.16989E-18	1.95077E-16	PREDICTED: protein croquemort-like
RS014732	GO:0044699	single-organism process	BP	-2.171879817	-1.24728548	0.09932889	5.367265273	82.18961662	1.04062E-17	2.21004E-16	PREDICTED: solute carrier organic anion transporter family member 2A1
RS002687	GO:0044699	single-organism process	BP	3.182608916	3.046354965	-1.369458287	2.06064219	81.71825502	1.31349E-17	2.78018E-16	branchless
RS015390	GO:0044699	single-organism process	BP	-1.260588417	-0.88728058	1.174441311	5.643186003	81.54774944	1.42892E-17	3.01438E-16	PREDICTED: sodium-independent sulfate anion transporter
RS010611	GO:0044699	single-organism process	BP	-1.874440611	-3.117963163	-1.762661	5.623164733	81.37661471	1.55498E-17	3.26935E-16	PREDICTED: cytochrome P450 9e2-like
RS006415	GO:0044699	single-organism process	BP	-3.059456516	-3.953414729	-1.888715959	4.231267415	80.90022593	1.96755E-17	4.11617E-16	PREDICTED: glucose dehydrogenase [FAD, quinone]-like
RS014485	GO:0044699	single-organism process	BP	-2.876103439	-4.859730307	-1.669594344	3.096724346	80.81553027	2.05161E-17	4.27075E-16	PREDICTED: cytochrome P450 6k1-like
RS014579	GO:0044699	single-organism process	BP	-1.833927302	-1.637670215	-2.03241566	4.946947748	80.73878787	2.13088E-17	4.42843E-16	PREDICTED: transient receptor potential cation channel protein painless
RS010634	GO:0044699	single-organism process	BP	-1.040329997	-2.397752271	-0.988326981	6.391698647	80.5838793	2.30033E-17	4.75703E-16	PREDICTED: prostaglandin reductase 1-like
RS011973	GO:0044699	single-organism process	BP	-0.861945869	1.222753935	-0.568825813	4.359430432	80.33905671	2.59602E-17	5.34219E-16	PREDICTED: zinc transporter 1
RS000216	GO:0044699	single-organism process	BP	0.006923045	3.155966968	0.729591328	2.670233893	80.26935787	2.68694E-17	5.52028E-16	PREDICTED: innexin shaking-B
RS010860	GO:0044699	single-organism process	BP	-0.513060367	-1.867151173	-2.038852609	4.807472629	80.18504484	2.8012E-17	5.72699E-16	PREDICTED: fucose mutarotase
RS010590	GO:0044699	single-organism process	BP	-2.57016287	-4.977699687	-4.728351418	1.94352856	79.20706072	4.54057E-17	9.19355E-16	PREDICTED: acyl-CoA synthetase short-chain family member 3, mitochondrial
RS013829	GO:0044699	single-organism process	BP	-3.015607204	1.594605805	1.65676505	3.934101852	78.84573345	5.42754E-17	1.09192E-15	PREDICTED: nuclear hormone receptor FTZ-F1
RS008250	GO:0044699	single-organism process	BP	-1.320752861	-4.449222575	-1.991229095	4.875325306	77.72414306	9.44318E-17	1.88774E-15	PREDICTED: dehydrogenase/reductase SDR family member 11
RS005077	GO:0044699	single-organism process	BP	-1.853453594	-1.879553398	-0.229787216	4.527312475	77.70992995	9.50968E-17	1.89802E-15	PREDICTED: sodium-coupled monocarboxylate transporter 1-like
RS004383	GO:0044699	single-organism process	BP	1.531726105	1.066158773	-0.120151447	6.626366457	77.64194245	9.83431E-17	1.9597E-15	PREDICTED: insulin-degrading enzyme
RS002222	GO:0044699	single-organism process	BP	1.040237914	2.76223951	2.556623912	5.259858563	77.56540926	1.0213E-16	2.03195E-15	PREDICTED: pancreatic lipase-related protein 2-like
RS004424	GO:0044699	single-organism process	BP	-0.154114067	2.332271731	0.36051487	5.646491566	77.24068683	1.19889E-16	2.38151E-15	PREDICTED: TWiK family of potassium channels protein 7
RS000815	GO:0044699	single-organism process	BP	-1.262984526	-2.453584338	-1.561433711	5.065217685	77.01022079	1.34335E-16	2.6601E-15	PREDICTED: cytochrome P450 6k1-like
RS013664	GO:0044699	single-organism process	BP	-2.172206706	-3.603858445	-1.432660958	6.909404613	76.93178914	1.39639E-16	2.76077E-15	PREDICTED: probable cytochrome P450 6a13
RS011853	GO:0044699	single-organism process	BP	-1.313648639	-2.478519389	-2.71556709	4.85148492	76.50416751	1.72458E-16	3.38308E-15	PREDICTED: aldehyde dehydrogenase, dimeric NADP-preferring
RS003149	GO:0044699	single-organism process	BP	-2.041224545	-4.252171047	-2.371690984	2.549963522	76.39474375	1.82029E-16	3.55423E-15	PREDICTED: probable cytochrome P450 6a14
RS002366	GO:0044699	single-organism process	BP	-2.013469227	2.285528889	0.940362283	2.009763796	76.37983445	1.83374E-16	3.57494E-15	PREDICTED: sodium-dependent serotonin transporter
RS004871	GO:0044699	single-organism process	BP	0.288015696	3.09715123	1.160078455	3.818460573	76.32733992	1.88188E-16	3.66311E-15	G-protein coupled receptor Mth2
RS004723	GO:0044699	single-organism process	BP	-0.605681875	0.81470227	2.160175202	4.779063462	76.01797748	2.19234E-16	4.24775E-15	Liprin-beta-1
RS013785	GO:0044699	single-organism process	BP	-1.824353655	1.515123028	1.759409539	6.020912494	75.89329217	2.3315E-16	4.50352E-15	PREDICTED: nuclear hormone receptor FTZ-F1
RS010778	GO:0044699	single-organism process	BP	-2.374219528	-5.181629311	-2.196074053	5.244928834	75.69892556	2.56625E-16	4.9418E-15	PREDICTED: lipase 3-like
RS008850	GO:0044699	single-organism process	BP	-0.034048793	-3.919275437	-3.888425234	2.887389913	75.68574566	2.583E-16	4.96646E-15	PREDICTED: cytochrome P450 6k1-like
RS014496	GO:0044699	single-organism process	BP	-2.44814153	-3.696803576	-1.083788116	3.702386291	75.5252221	2.79597E-16	5.36775E-15	Dehydrogenase/reductase SDR family member 11
RS008819	GO:0044699	single-organism process	BP	-1.032692902	-2.222642533	-1.657320465	5.534608709	75.39402177	2.98301E-16	5.70943E-15	PREDICTED: glutaminase liver isoform, mitochondrial
RS005453	GO:0044699	single-organism process	BP	0.668735883	1.879757994	0.412329477	5.54953181	75.34668517	3.05352E-16	5.83551E-15	PREDICTED: ATP-binding cassette sub-family G member 1
RS000975	GO:0044699	single-organism process	BP	-6.513903292	-7.688604171	2.940067135	11.1479828	75.07664463	3.48883E-16	6.63721E-15	neuropeptide like precursor 3-like
RS003156	GO:0044699	single-organism process	BP	-1.731849332	-3.299783923	-1.878218617	6.179838024	75.03990047	3.55267E-16	6.71807E-15	cytochrome P450
RS008510	GO:0044699	single-organism process	BP	-1.907443864	0.513471025	2.887314324	3.573724567	74.77986444	4.03912E-16	7.61507E-15	PREDICTED: GTP cyclohydrolase 1
RS000948	GO:0044699	single-organism process	BP	-1.424649633	-0.799295095	1.234430326	6.77421009	74.06852264	5.7376E-16	1.07529E-14	Protein disulfide-isomerase A5
RS014237	GO:0044699	single-organism process	BP	-1.358203588	-0.858052145	0.808855596	6.87948729	73.79033318	6.58177E-16	1.22983E-14	PREDICTED: calcium-binding mitochondrial carrier protein Aralar1
RS006640	GO:0044699	single-organism process	BP	-1.791513421	5.598946144	0.991323579	0.785411688	73.73810476	6.75358E-16	1.25635E-14	PREDICTED: aromatic-L-amino-acid decarboxylase-like
RS000980	GO:0044699	single-organism process	BP	-1.142427852	-5.738335179	-2.9180828	5.801501745	73.65585145	7.0333E-16	1.30453E-14	PREDICTED: probable cytochrome P450 305a1
RS001840	GO:0044699	single-organism process	BP	-0.121553778	2.536459905	-0.70159401	2.5556935	73.24989778	8.59295E-16	1.58448E-14	PREDICTED: AF4/FMR2 family member 4-like
RS006074	GO:0044699	single-organism process	BP	0.061476322	1.293176276	1.550346639	6.752584934	73.22602795	8.69474E-16	1.6009E-14	PREDICTED: viral IAP-associated factor homolog
RS004435	GO:0044699	single-organism process	BP	-2.355016468	-4.840360235	-1.508287833	6.256644026	72.98893691	9.7736E-16	1.78908E-14	membrane transporter
RS014494	GO:0044699	single-organism process	BP	-2.432481365	-5.196280499	-2.034004727	3.984278095	72.57188313	1.20062E-15	2.17876E-14	PREDICTED: dehydrogenase/reductase SDR family member 11-like
RS011988	GO:0044699	single-organism process	BP	-1.045428446	0.08149937	1.776943069	7.507925055	72.44172426	1.28023E-15	2.31656E-14	PREDICTED: aquaporin-12
RS006641	GO:0044699	single-organism process	BP	0	7.485546798	0	0.803640671	72.21979448	1.42834E-15	2.58085E-14	PREDICTED: aromatic-L-amino-acid decarboxylase-like
RS002034	GO:0044699	single-organism process	BP	-2.76074966	-3.66938775	0.182700124	4.670360092	71.90234509	1.67045E-15	3.00969E-14	PREDICTED: 4-hydroxyphenylpyruvate dioxygenase
RS006315	GO:0044699	single-organism process	BP	-2.445857294	-4.975199992	0.147442902	3.069628072	71.7469443	1.80352E-15	3.24017E-14	PREDICTED: bumetanide-sensitive sodium-(potassium)-chloride cotransporter
RS008278	GO:0044699	single-organism process	BP	0.642268679	2.750452439	1.283258456	3.961355993	71.14168969	2.43087E-15	4.34246E-14	PREDICTED: potassium voltage-gated channel protein Shaw-like
RS000228	GO:0044699	single-organism process	BP	-1.198401056	0.748426917	1.708285335	5.232498935	70.64756833	3.1016E-15	5.48618E-14	PREDICTED: facilitated trehalose transporter Tret1-like
RS010209	GO:0044699	single-organism process	BP	-1.199116022	0.810918744	-0.247836435	5.677637623	69.45798048	5.5759E-15	9.71271E-14	PREDICTED: acyl-CoA Delta(11) desaturase
RS009071	GO:0044699	single-organism process	BP	-1.098903341	1.887027204	0.517588265	5.781996572	69.43996733	5.62564E-15	9.78582E-14	PREDICTED: angiotensin-converting enzyme
RS013835	GO:0044699	single-organism process	BP	-3.138201275	-5.46811686	-2.708237319	1.967351202	69.40811596	5.71467E-15	9.92698E-14	cytochrome P450
RS009553	GO:0044699	single-organism process	BP	-1.014221448	-0.49963889	0.896520456	6.75591274	69.40067409	5.73568E-15	9.94974E-14	PREDICTED: prostatic acid phosphatase-like
RS001403	GO:0044699	single-organism process	BP	-2.730002539	-3.225210407	0.21862196	5.256977583	69.09878372	6.65609E-15	1.15147E-13	PREDICTED: putative metabolite transport protein HI_1104
RS004166	GO:0044699	single-organism process	BP	-2.051391916	0.351054893	0.641192255	5.773153891	69.05783117	6.79183E-15	1.17173E-13	PREDICTED: laccase-1-like
RS004102	GO:0044699	single-organism process	BP	-1.281622227	2.367984903	0.792781013	2.704679545	68.39908264	9.39742E-15	1.60803E-13	PREDICTED: heparan sulfate 2-O-sulfotransferase pipe
RS006271	GO:0044699	single-organism process	BP	1.985922002	1.711587011	-1.594562947	3.353974541	68.37067995	9.52991E-15	1.62849E-13	PREDICTED: organic cation transporter 1
RS006665	GO:0044699	single-organism process	BP	-0.823102205	-1.375582878	0.852023692	5.282609853	68.30800501	9.8289E-15	1.67731E-13	PREDICTED: breast cancer anti-estrogen resistance protein 3
RS012246	GO:0044699	single-organism process	BP	-0.570469265	-2.940993589	-0.072505664	3.698768333	68.13212965	1.07189E-14	1.82178E-13	PREDICTED: cytochrome P450 302a1, mitochondrial
RS013098	GO:0044699	single-organism process	BP	-2.126898304	-3.4336079	-2.804919227	4.337624443	68.06603945	1.10738E-14	1.87704E-13	PREDICTED: xanthine dehydrogenase/oxidase-like
RS007436	GO:0044699	single-organism process	BP	-1.373814305	0.238018607	1.128809843	7.410193397	67.9550388	1.16965E-14	1.97461E-13	PREDICTED: sorbin and SH3 domain-containing protein 2-like
RS008443	GO:0044699	single-organism process	BP	-1.979883882	-8.057075206	-1.206380805	2.046366836	67.84965365	1.23201E-14	2.07155E-13	PREDICTED: facilitated trehalose transporter Tret1-2 homolog
RS009152	GO:0044699	single-organism process	BP	-0.076188961	2.318086608	-0.327763265	4.849879578	67.57555546	1.4102E-14	2.35857E-13	PREDICTED: alpha-(1,6)-fucosyltransferase
RS013033	GO:0044699	single-organism process	BP	-2.44596251	-3.881013688	-4.124427842	5.607467213	67.36154464	1.56705E-14	2.61743E-13	PREDICTED: glucose dehydrogenase [FAD, quinone]
RS005262	GO:0044699	single-organism process	BP	-0.644057425	1.484637721	1.372046282	6.910372909	66.70945052	2.16088E-14	3.58552E-13	Intersectin-2
RS006903	GO:0044699	single-organism process	BP	0.773688372	2.857651838	1.403228733	4.679118286	66.6189813	2.25938E-14	3.73911E-13	PREDICTED: pancreatic triacylglycerol lipase-like
RS005437	GO:0044699	single-organism process	BP	-0.899257268	-2.245545308	-2.877195377	7.631293432	66.09329323	2.92732E-14	4.80661E-13	PREDICTED: acylphosphatase-2
RS005780	GO:0044699	single-organism process	BP	-2.209524268	-3.568309813	-0.61439872	4.088152173	66.00896266	3.0515E-14	4.99748E-13	PREDICTED: homogentisate 1,2-dioxygenase
RS013980	GO:0044699	single-organism process	BP	1.70171747	1.349492418	0.304429583	5.412132669	65.9064818	3.20951E-14	5.24943E-13	PREDICTED: phosphomannomutase
RS010041	GO:0044699	single-organism process	BP	-0.841304888	-1.311133394	1.016447212	5.056278579	65.67682339	3.59396E-14	5.85541E-13	PREDICTED: peptidoglycan-recognition protein LC-like
RS007018	GO:0044699	single-organism process	BP	-1.769441031	-2.893314898	-0.078411849	4.392691737	65.37012463	4.18008E-14	6.78402E-13	PREDICTED: insulin-like receptor
RS013013	GO:0044699	single-organism process	BP	-1.540094428	-1.189185544	1.548167541	3.519055593	65.31696378	4.29099E-14	6.95504E-13	PREDICTED: peptidoglycan-recognition protein LB-like
RS014695	GO:0044699	single-organism process	BP	-3.182123286	-4.605585624	-2.434488323	1.709736198	64.954873	5.12876E-14	8.27038E-13	PREDICTED: organic cation transporter protein
RS005658	GO:0044699	single-organism process	BP	0.207452045	2.219852023	2.409164965	3.733589056	64.80484621	5.52209E-14	8.88188E-13	PREDICTED: probable cytochrome P450 301a1, mitochondrial
RS001006	GO:0044699	single-organism process	BP	-1.308055843	0.748595123	2.011362686	7.151837524	64.31436805	7.03086E-14	1.12512E-12	PREDICTED: sodium-dependent nutrient amino acid transporter 1-like
RS003016	GO:0044699	single-organism process	BP	0.355128266	-1.63524154	-2.121597333	3.87230946	64.30058562	7.07874E-14	1.13134E-12	PREDICTED: glucose dehydrogenase [FAD, quinone]
RS010386	GO:0044699	single-organism process	BP	1.385615233	2.177722429	1.08390832	5.303798446	64.21218649	7.3937E-14	1.18018E-12	PREDICTED: ketimine reductase mu-crystallin
RS010163	GO:0044699	single-organism process	BP	-2.550994651	-4.854888232	-3.071576004	6.587744208	64.1927153	7.46493E-14	1.19004E-12	PREDICTED: cytochrome P450 6k1-like
RS011173	GO:0044699	single-organism process	BP	-0.925107219	-1.604021027	-1.115433762	6.20820018	63.76408718	9.21913E-14	1.46045E-12	PREDICTED: L-galactose dehydrogenase-like
RS010606	GO:0044699	single-organism process	BP	-2.368811611	-3.144344494	-2.367074066	3.691341387	63.45365154	1.07417E-13	1.69101E-12	PREDICTED: cytochrome P450 9e2-like
RS013244	GO:0044699	single-organism process	BP	-1.611287022	-4.550784717	-2.084168564	3.087514358	63.42642333	1.08867E-13	1.71169E-12	PREDICTED: acyl-CoA Delta(11) desaturase-like
RS006840	GO:0044699	single-organism process	BP	-1.528923895	-1.666690338	0.003915845	5.78754987	63.18992323	1.2231E-13	1.91351E-12	PREDICTED: probable hydroxyacid-oxoacid transhydrogenase, mitochondrial
RS014962	GO:0044699	single-organism process	BP	-0.778249315	-3.477526571	-0.968757046	7.419767245	62.95916817	1.37025E-13	2.14106E-12	PREDICTED: delta(24)-sterol reductase
RS013848	GO:0044699	single-organism process	BP	0.385280105	-0.451376677	-1.360242751	6.103207414	62.73512141	1.53003E-13	2.38481E-12	PREDICTED: protein CREG1
RS000213	GO:0044699	single-organism process	BP	-2.775159969	-5.407059204	-1.896008339	1.358103957	62.58039536	1.65112E-13	2.55772E-12	PREDICTED: innexin shaking-B
RS012642	GO:0044699	single-organism process	BP	1.82812389	1.487792365	0.508984734	6.246596161	62.55806624	1.66937E-13	2.58281E-12	PREDICTED: protein yippee-like 1
RS014869	GO:0044699	single-organism process	BP	-1.914036711	-6.718981489	-3.106950957	9.206887901	62.42555436	1.78189E-13	2.75352E-12	PREDICTED: glucosylceramidase-like
RS002203	GO:0044699	single-organism process	BP	-0.12814185	2.306500337	1.518575612	3.797272355	62.10896556	2.08237E-13	3.21389E-12	PREDICTED: ATP-binding cassette sub-family G member 4
RS003495	GO:0044699	single-organism process	BP	-1.321761447	-2.422499478	0.06766072	3.777578236	62.06524239	2.12767E-13	3.27979E-12	PREDICTED: oxidative stress-induced growth inhibitor 2-like
RS002511	GO:0044699	single-organism process	BP	-0.69900862	-1.454715384	-1.592361101	6.212273362	61.91143998	2.29498E-13	3.52906E-12	PREDICTED: glycogen [starch] synthase
RS000919	GO:0044699	single-organism process	BP	0.070629326	1.524717298	0.182975292	7.336844113	61.36491303	3.00324E-13	4.57904E-12	PREDICTED: 3-hydroxy-3-methylglutaryl-coenzyme A reductase
RS010746	GO:0044699	single-organism process	BP	1.925894311	3.013679552	0.800152242	2.938831742	60.67298927	4.22138E-13	6.36695E-12	Amine oxidase [flavin-containing] B
RS006994	GO:0044699	single-organism process	BP	-0.66398235	-0.594278945	1.697528097	4.919565	60.59841537	4.37914E-13	6.597E-12	PREDICTED: putative inorganic phosphate cotransporter
RS006876	GO:0044699	single-organism process	BP	-1.906405126	-4.198252583	-2.68639647	6.979759297	60.5192047	4.55318E-13	6.83465E-12	PREDICTED: acyl-CoA Delta(11) desaturase-like
RS007831	GO:0044699	single-organism process	BP	-0.285689079	2.23184291	-0.54998832	3.347851609	60.46667692	4.67238E-13	7.00523E-12	PREDICTED: sodium- and chloride-dependent GABA transporter ine-like
RS003153	GO:0044699	single-organism process	BP	-1.574922188	-4.570570746	-2.687308064	2.667699686	60.33326147	4.98936E-13	7.47158E-12	PREDICTED: probable cytochrome P450 6a14
RS011713	GO:0044699	single-organism process	BP	-2.342114325	-5.146606544	-5.648708234	2.431264584	60.21279143	5.294E-13	7.91837E-12	PREDICTED: glucose dehydrogenase [FAD, quinone]
RS013647	GO:0044699	single-organism process	BP	-1.115440718	-2.582939113	-1.288503389	4.886525189	60.18904281	5.35621E-13	8.00192E-12	PREDICTED: probable cytochrome P450 6a13
RS003152	GO:0044699	single-organism process	BP	-1.039074276	-3.749023978	-2.116976898	4.07470091	59.88983701	6.20557E-13	9.24887E-12	PREDICTED: probable cytochrome P450 6a14
RS008251	GO:0044699	single-organism process	BP	-1.012307933	-3.255692772	-0.950692528	5.034390269	59.83246475	6.3832E-13	9.50237E-12	PREDICTED: dehydrogenase/reductase SDR family member 11-like
RS007228	GO:0044699	single-organism process	BP	-2.560463409	0.195134217	-0.521000497	6.368385994	59.52585567	7.42229E-13	1.09714E-11	PREDICTED: retinol dehydrogenase 14
RS015462	GO:0044699	single-organism process	BP	-2.562181511	-1.874035905	0.960657982	8.649528892	59.49483899	7.53639E-13	1.1127E-11	PREDICTED: probable citrate synthase 2, mitochondrial
RS009004	GO:0044699	single-organism process	BP	-1.724057836	-4.564536642	-0.819207065	2.965758365	59.37486634	7.99449E-13	1.17895E-11	PREDICTED: mitochondrial basic amino acids transporter-like
RS006664	GO:0044699	single-organism process	BP	-2.643047657	0.520074633	1.717541658	7.376730422	58.96651027	9.77269E-13	1.43113E-11	Transforming growth factor-beta-induced protein ig-h3
RS001672	GO:0044699	single-organism process	BP	-1.155357851	-1.706499334	-2.840101545	5.412224306	58.94003971	9.90074E-13	1.44652E-11	PREDICTED: catalase
RS002052	GO:0044699	single-organism process	BP	-2.167253342	2.826832687	1.748485901	5.595702905	58.74140273	1.09167E-12	1.58942E-11	PREDICTED: laccase-5-like
RS009917	GO:0044699	single-organism process	BP	-2.92853977	-3.567066743	-0.105474701	4.514065574	58.59810319	1.17137E-12	1.7035E-11	PREDICTED: phosphoenolpyruvate carboxykinase [GTP]-like
RS002789	GO:0044699	single-organism process	BP	-1.372012942	-1.490274038	-0.050279956	6.171605621	58.36997718	1.31043E-12	1.89478E-11	PREDICTED: long-chain-fatty-acid--CoA ligase 5
RS011717	GO:0044699	single-organism process	BP	-5.032597315	2.326479602	1.338171742	5.261254656	58.26088503	1.38264E-12	1.99461E-11	PREDICTED: glucose dehydrogenase [FAD, quinone]-like

RS010906	GO:0044699	single-organism process	BP	-4.255006548	-1.567644761	2.696115347	8.985641519	58.14209697	1.4658E-12	2.10975E-11	PREDICTED: tyrosine 3-monooxygenase
RS007482	GO:0044699	single-organism process	BP	-1.928508957	-3.965096441	-3.707408816	3.476799558	57.92648171	1.62973E-12	2.34301E-11	PREDICTED: geranylgeranyl pyrophosphate synthase
RS009221	GO:0044699	single-organism process	BP	-0.121969881	1.574996175	1.697054704	5.919275299	57.89701083	1.65351E-12	2.37179E-11	PREDICTED: serine-threonine kinase receptor-associated protein
RS004187	GO:0044699	single-organism process	BP	0.051852857	0.716857623	1.52846695	7.094613975	57.08224889	2.46806E-12	3.49637E-11	PREDICTED: NAD kinase-like
RS011073	GO:0044699	single-organism process	BP	1.681239408	1.71053558	-0.220987815	5.116873023	56.90571814	2.69177E-12	3.80474E-11	PREDICTED: vitellogenin-like
RS008191	GO:0044699	single-organism process	BP	-3.021136905	-0.272400079	1.298364757	4.630032009	56.86531108	2.74577E-12	3.8767E-11	PREDICTED: trichohyalin
RS004722	GO:0044699	single-organism process	BP	-0.688116409	0.284058705	1.281169675	5.989720641	56.50964862	3.27023E-12	4.58633E-11	PREDICTED: liprin-beta-1
RS010228	GO:0044699	single-organism process	BP	-0.603654192	-3.938345019	-0.506568246	3.3634338	56.06539856	4.06808E-12	5.66115E-11	PREDICTED: xanthine dehydrogenase/oxidase-like
RS006060	GO:0044699	single-organism process	BP	-0.510179653	2.010318247	0.930442243	4.838598392	56.04175354	4.11562E-12	5.72099E-11	forked
RS009993	GO:0044699	single-organism process	BP	-0.660386169	1.329644951	-0.126272013	4.440804092	55.87977965	4.45657E-12	6.16093E-11	PREDICTED: dehydrogenase/reductase SDR family member on chromosome X
RS005485	GO:0044699	single-organism process	BP	-1.618889345	-1.998591571	-1.47354184	6.882241188	55.52024724	5.31763E-12	7.32716E-11	PREDICTED: retinol dehydrogenase 13-like
RS003713	GO:0044699	single-organism process	BP	-2.006533724	-2.265969826	-2.407654301	4.003209248	55.48714921	5.40481E-12	7.43915E-11	PREDICTED: growth arrest-specific protein 2-like
RS010272	GO:0044699	single-organism process	BP	-3.250896666	-4.41990282	-0.187787053	3.271423774	55.38395874	5.68588E-12	7.81746E-11	PREDICTED: glucose dehydrogenase [FAD, quinone]-like
RS003292	GO:0044699	single-organism process	BP	-3.305241102	-6.333825568	-1.772035881	11.1765296	55.22912463	6.13525E-12	8.41692E-11	PREDICTED: ejaculatory bulb-specific protein 3
RS008170	GO:0044699	single-organism process	BP	0.253513984	1.733052836	0.806515481	5.816940129	54.81424338	7.52215E-12	1.02972E-10	PREDICTED: glucose dehydrogenase [FAD, quinone]
RS001261	GO:0044699	single-organism process	BP	-0.735144857	-2.614482301	-2.129153596	5.833143755	54.62533616	8.25351E-12	1.12616E-10	PREDICTED: elongation of very long chain fatty acids protein 7
RS003745	GO:0044699	single-organism process	BP	0.166576035	1.591069186	1.721030016	7.861189432	54.5929302	8.38593E-12	1.14299E-10	PREDICTED: cGMP-dependent protein kinase, isozyme 2 forms cD4/T1/T3A/T3B
RS000584	GO:0044699	single-organism process	BP	-3.218559687	-0.666315986	0.361963046	8.571279444	54.50851473	8.74093E-12	1.19009E-10	chemosensory protein
RS010878	GO:0044699	single-organism process	BP	-0.202147251	0.219397642	1.259334773	5.8954575	54.42606082	9.10217E-12	1.23527E-10	PREDICTED: band 3 anion transport protein
RS013635	GO:0044699	single-organism process	BP	-3.132556281	-4.708910583	-3.489353155	0.683770869	54.42024633	9.1282E-12	1.23747E-10	Cytochrome P450 4C1
RS009484	GO:0044699	single-organism process	BP	0.531159268	-0.753055165	-3.522846028	2.912098997	54.18479776	1.02472E-11	1.3832E-10	PREDICTED: G-protein coupled receptor 143-like
RS007731	GO:0044699	single-organism process	BP	-0.212130184	2.422258994	-1.511673278	4.25447991	54.14547158	1.0447E-11	1.40867E-10	PREDICTED: metabotropic glutamate receptor 2-like
RS008252	GO:0044699	single-organism process	BP	1.087955	5.332121384	-0.588233895	1.221679144	54.04196925	1.09917E-11	1.47895E-10	PREDICTED: dehydrogenase/reductase SDR family member 11-like
RS009449	GO:0044699	single-organism process	BP	0.217859906	0.894808756	-2.254717648	4.298914796	53.88906176	1.18488E-11	1.59257E-10	PREDICTED: very-long-chain 3-oxoacyl-CoA reductase
RS003509	GO:0044699	single-organism process	BP	-0.36042749	1.933660325	1.876806238	6.361690814	53.86819358	1.19709E-11	1.60519E-10	PREDICTED: venom metalloproteinase 3
RS003155	GO:0044699	single-organism process	BP	-0.932305778	-3.619417471	-2.546802201	4.482591272	53.85296725	1.20607E-11	1.61416E-10	PREDICTED: probable cytochrome P450 6a14
RS000947	GO:0044699	single-organism process	BP	-1.65511431	-1.392425323	0.73931174	8.722692002	53.78336359	1.248E-11	1.66851E-10	PREDICTED: FK506-binding protein 5
RS004117	GO:0044699	single-organism process	BP	-0.833839389	-1.498856462	0.389559148	5.929206642	53.74612284	1.27103E-11	1.6975E-10	PREDICTED: FMRFamide receptor-like
RS003633	GO:0044699	single-organism process	BP	2.406817281	4.495780859	0.555172218	1.526066816	53.73260343	1.2795E-11	1.70699E-10	PREDICTED: lipase 3-like
RS002521	GO:0044699	single-organism process	BP	-0.871873311	-2.489627168	-0.374270696	6.536689236	53.54809917	1.40083E-11	1.86491E-10	PREDICTED: dehydrogenase/reductase SDR family member 11-like
RS008717	GO:0044699	single-organism process	BP	-0.547135716	1.669312441	1.069669447	5.830198322	53.44032639	1.47695E-11	1.96003E-10	PREDICTED: CD151 antigen
RS008935	GO:0044699	single-organism process	BP	-0.003333114	1.673408999	0.502619815	5.286993351	53.33008857	1.55909E-11	2.06686E-10	PREDICTED: leucine zipper putative tumor suppressor 2 homolog
RS013151	GO:0044699	single-organism process	BP	0.240198914	1.905291964	0.499372157	4.816395016	53.28933142	1.5906E-11	2.1042E-10	PREDICTED: cAMP-specific 3',5'-cyclic phosphodiesterase 4A-like
RS006397	GO:0044699	single-organism process	BP	2.677889399	3.536925796	2.975240508	5.435489187	53.28452354	1.59436E-11	2.10696E-10	PREDICTED: glucose dehydrogenase [FAD, quinone]
RS010162	GO:0044699	single-organism process	BP	-2.448268703	-4.852982151	-3.40180898	4.27254499	53.25379016	1.6186E-11	2.13451E-10	PREDICTED: cytochrome P450 6k1-like
RS010609	GO:0044699	single-organism process	BP	-2.078534702	-2.906704826	-2.369304453	6.160164309	53.20623791	1.65683E-11	2.18265E-10	PREDICTED: cytochrome P450 9e2-like
RS002266	GO:0044699	single-organism process	BP	-1.93030545	-2.304011818	0.984730367	5.553897465	53.11063075	1.73645E-11	2.28038E-10	PREDICTED: ADP-sugar pyrophosphatase
RS000708	GO:0044699	single-organism process	BP	-1.23499554	-2.729140829	-1.377527606	4.320776854	53.03106634	1.80561E-11	2.36628E-10	PREDICTED: aldose reductase-like
RS006694	GO:0044699	single-organism process	BP	0.434554837	2.61722148	1.159617999	3.14099825	52.98400949	1.84781E-11	2.41906E-10	PREDICTED: bcl-2-related ovarian killer protein-like
RS008873	GO:0044699	single-organism process	BP	0.214275635	1.741156477	1.29973684	6.340063699	52.95544053	1.87391E-11	2.45067E-10	GL14064
RS000797	GO:0044699	single-organism process	BP	-1.906331881	-1.987387567	-0.799925012	5.057556376	52.80550918	2.01703E-11	2.63238E-10	stripe
RS014958	GO:0044699	single-organism process	BP	-1.228360328	-1.847606458	-0.667973568	7.113057913	52.54514506	2.292E-11	2.98197E-10	PREDICTED: quinone oxidoreductase
RS000961	GO:0044699	single-organism process	BP	-0.707857267	-2.959502955	-2.707049538	2.485173352	52.53629744	2.30197E-11	2.99185E-10	Lipase 3
RS008617	GO:0044699	single-organism process	BP	-1.758654905	-1.933395291	0.119722157	4.240860888	52.46640405	2.38231E-11	3.09308E-10	PREDICTED: proton-coupled amino acid transporter 1
RS011602	GO:0044699	single-organism process	BP	-1.476553362	-1.897162637	0.231276491	7.109501682	52.29111173	2.59635E-11	3.3675E-10	PREDICTED: acetyl-coenzyme A synthetase
RS006021	GO:0044699	single-organism process	BP	-1.978389488	-0.987147201	0.23457041	5.767022167	52.1310105	2.80858E-11	3.63527E-10	PREDICTED: cationic amino acid transporter 3
RS000960	GO:0044699	single-organism process	BP	-1.97268983	2.572463394	2.330300941	1.703317672	52.07353344	2.88893E-11	3.73544E-10	PREDICTED: lipase 3-like
RS009161	GO:0044699	single-organism process	BP	-0.295774945	-1.280493549	0.533120662	6.698267524	51.95530641	3.0615E-11	3.95451E-10	PREDICTED: 4-coumarate--CoA ligase 1
RS002910	GO:0044699	single-organism process	BP	-1.969650787	-1.815116369	-1.894484106	3.888046225	51.87661209	3.18203E-11	4.106E-10	cytochrome P450
RS005693	GO:0044699	single-organism process	BP	-1.530868766	-3.126670283	-3.45883824	4.672689231	51.54252942	3.74885E-11	4.81275E-10	PREDICTED: facilitated trehalose transporter Tret1-2 homolog
RS009596	GO:0044699	single-organism process	BP	1.093926571	2.520157329	1.243834608	5.223653192	51.28018671	4.26381E-11	5.42408E-10	PREDICTED: T-box transcription factor TBX1
RS009073	GO:0044699	single-organism process	BP	-1.510567785	0.927876147	1.809212406	5.164332774	50.87027386	5.21352E-11	6.61886E-10	PREDICTED: solute carrier family 46 member 3
RS011642	GO:0044699	single-organism process	BP	-2.363975484	-1.131041885	-0.186002278	3.85755016	50.84894851	5.26835E-11	6.68173E-10	PREDICTED: carboxylesterase 1C
RS001249	GO:0044699	single-organism process	BP	-0.845862568	0.222883144	2.176673324	6.011338737	50.43913468	6.44129E-11	8.12841E-10	PREDICTED: 17-beta-hydroxysteroid dehydrogenase 13-like
RS000134	GO:0044699	single-organism process	BP	-2.137136524	0.447963208	0.262936854	3.43282157	50.22811762	7.14362E-11	8.98769E-10	PREDICTED: putative ammonium transporter 1
RS013213	GO:0044699	single-organism process	BP	-1.712589731	-2.286901003	0.483545885	7.415178553	50.10415403	7.59137E-11	9.53198E-10	PREDICTED: 4-hydroxybutyrate coenzyme A transferase
RS014292	GO:0044699	single-organism process	BP	-2.056763959	-3.747670529	-0.583961639	4.094602278	49.98172238	8.06111E-11	1.01117E-09	PREDICTED: probable cytochrome P450 49a1
RS012141	GO:0044699	single-organism process	BP	-0.795199967	0.272005658	1.157168	4.936082578	49.75842476	8.99389E-11	1.12594E-09	PREDICTED: UNC93-like protein MFSD11
RS000986	GO:0044699	single-organism process	BP	-1.612598154	-1.562338284	-1.987921038	4.387068382	49.65417221	9.46559E-11	1.18146E-09	PREDICTED: probable cytochrome P450 305a1
RS000962	GO:0044699	single-organism process	BP	-1.267212099	-3.948910387	-2.907002134	2.707713789	49.36323106	1.09169E-10	1.35722E-09	PREDICTED: lipase 3-like
RS013665	GO:0044699	single-organism process	BP	-2.735006342	-4.935001894	-0.754376068	5.921729894	49.2507131	1.1536E-10	1.42996E-09	cytochrome P450
RS001763	GO:0044699	single-organism process	BP	-1.883006032	-3.946455627	-1.232693773	5.510373929	49.24591945	1.15631E-10	1.43147E-09	Dehydrogenase/reductase SDR family member 11
RS008254	GO:0044699	single-organism process	BP	-1.84400551	0.769806662	1.172103168	3.667348911	48.7792472	1.45353E-10	1.78592E-09	PREDICTED: facilitated trehalose transporter Tret1-like
RS002240	GO:0044699	single-organism process	BP	-0.851489546	0.204102513	1.146678862	4.735548097	48.55844091	1.61966E-10	1.98231E-09	PREDICTED: probable pyruvate dehydrogenase E1 component subunit alpha, mitochondrial
RS001805	GO:0044699	single-organism process	BP	-1.712509312	0.709000933	-0.641501281	4.340777854	48.29287055	1.84479E-10	2.24693E-09	PREDICTED: elongation of very long chain fatty acids protein AAEL008004-like
RS001964	GO:0044699	single-organism process	BP	-0.08889768	-1.094615495	-1.709346424	4.842490732	48.28139375	1.85519E-10	2.25524E-09	carboxylesterase
RS007002	GO:0044699	single-organism process	BP	-2.468593161	-5.0492494	-2.627899474	3.73092382	48.053052	2.07484E-10	2.50773E-09	UDP-glucuronosyltransferase 2C1
RS009686	GO:0044699	single-organism process	BP	-2.882458924	-3.707652316	-2.499775747	2.735891641	47.57890032	2.61744E-10	3.13943E-09	PREDICTED: gamma-glutamyl hydrolase-like
RS000211	GO:0044699	single-organism process	BP	-1.735345083	-6.508275359	-1.173336997	2.558478767	47.43806859	2.8044E-10	3.35409E-09	cytochrome P450 6BQ10
RS014755	GO:0044699	single-organism process	BP	-1.512321011	0.776695379	0.224272966	4.668653569	47.24300788	3.08558E-10	3.66602E-09	PREDICTED: aldose 1-epimerase
RS006422	GO:0044699	single-organism process	BP	-0.088130055	1.300502444	2.226547033	7.201832072	46.72364419	3.97934E-10	4.71014E-09	PREDICTED: GAS2-like protein 1
RS007667	GO:0044699	single-organism process	BP	-2.905496692	-3.771454356	-1.959403868	2.847931386	46.70215936	4.02143E-10	4.75549E-09	PREDICTED: xanthine dehydrogenase/oxidase-like
RS001834	GO:0044699	single-organism process	BP	-0.603389323	0.374444079	1.306874145	4.748010336	46.63034204	4.16539E-10	4.92109E-09	PREDICTED: 5'-nucleotidase
RS007483	GO:0044699	single-organism process	BP	-2.426509726	-4.888542858	-1.641942338	2.479463759	46.53228086	4.37029E-10	5.14868E-09	PREDICTED: geranylgeranyl pyrophosphate synthase
RS005415	GO:0044699	single-organism process	BP	0.752364721	2.105225135	1.227482192	5.12894411	46.44013978	4.57199E-10	5.37625E-09	PREDICTED: whirlin
RS008573	GO:0044699	single-organism process	BP	-2.260713301	-3.030774322	-1.815246754	3.622901311	46.35220856	4.77314E-10	5.60233E-09	PREDICTED: probable aspartate aminotransferase, cytoplasmic
RS010573	GO:0044699	single-organism process	BP	-2.709572748	-2.377809387	-2.920421811	2.150943813	46.34585331	4.78802E-10	5.61456E-09	PREDICTED: cytochrome P450 9e2-like
RS014413	GO:0044699	single-organism process	BP	-3.721287275	-4.168286165	-3.100878197	0.868660601	46.12101612	5.34522E-10	6.25049E-09	PREDICTED: cytochrome P450 4V2
RS004422	GO:0044699	single-organism process	BP	0.746127292	-0.326413016	2.562442218	2.668262701	46.10990948	5.37437E-10	6.27874E-09	PREDICTED: cytochrome P450 4V2
RS009248	GO:0044699	single-organism process	BP	-1.191615658	-2.527981487	-0.234228501	8.921044612	45.94720912	5.81997E-10	6.78046E-09	PREDICTED: aldose reductase-like
RS005686	GO:0044699	single-organism process	BP	1.19862273	1.910481277	0.396698682	3.820462343	45.90603934	5.93847E-10	6.91211E-09	PREDICTED: lutropin-choriogonadotropic hormone receptor
RS001705	GO:0044699	single-organism process	BP	-2.66056254	-2.283004556	-1.241073988	3.766397219	45.84467431	6.11957E-10	7.11633E-09	PREDICTED: uncharacterized peptidase C1-like protein F26E4.3
RS002053	GO:0044699	single-organism process	BP	1.006786076	2.654271724	1.608006217	4.814861737	45.82056053	6.19224E-10	7.18865E-09	laccase 1
RS010777	GO:0044699	single-organism process	BP	1.24320996	0.865682303	0.068334999	6.870269993	45.82025182	6.19317E-10	7.18865E-09	PREDICTED: 26S protease regulatory subunit 6B
RS010737	GO:0044699	single-organism process	BP	-0.645730611	1.009936317	1.210261513	4.679785325	45.64187507	6.75826E-10	7.83014E-09	PREDICTED: elongation of very long chain fatty acids protein AAEL008004
RS009380	GO:0044699	single-organism process	BP	1.181487238	0.230316163	-1.011984835	4.090504935	45.23692145	8.23976E-10	9.50289E-09	PREDICTED: b(0,+)-type amino acid transporter 1-like
RS004116	GO:0044699	single-organism process	BP	0.34130734	-0.013101169	-1.46458415	7.02828743	45.18490377	8.45222E-10	9.73009E-09	PREDICTED: alpha,alpha-trehalose-phosphate synthase [UDP-forming]
RS005293	GO:0044699	single-organism process	BP	-0.81903625	-1.714660195	-0.381289809	5.252711507	45.14972751	8.59898E-10	9.89E-09	PREDICTED: putative inorganic phosphate cotransporter
RS011857	GO:0044699	single-organism process	BP	-2.520329028	-3.351704626	0.098895726	4.019687658	45.14359349	8.62483E-10	9.90887E-09	PREDICTED: 2-amino-3-ketobutyrate coenzyme A ligase, mitochondrial
RS001492	GO:0044699	single-organism process	BP	-1.047661717	-2.014014601	-0.725945308	5.708393329	45.11153039	8.76123E-10	1.00308E-08	PREDICTED: multidrug resistance-associated protein 4-like
RS006648	GO:0044699	single-organism process	BP	-1.472287059	-1.627468546	0.001448714	3.845832603	45.04057983	9.07077E-10	1.03664E-08	PREDICTED: voltage-dependent L-type calcium channel subunit beta-2
RS006270	GO:0044699	single-organism process	BP	-0.625556145	-0.022131593	2.665286326	2.775447041	44.95816183	9.44409E-10	1.07832E-08	PREDICTED: organic cation transporter 1
RS009576	GO:0044699	single-organism process	BP	-0.50213676	-1.291905438	-2.310240899	3.627997372	44.77505702	1.03293E-09	1.17726E-08	hypothetical protein
RS011737	GO:0044699	single-organism process	BP	-0.771192452	-2.179003544	-1.238139899	5.818282126	44.67524622	1.08463E-09	1.23172E-08	apolipophorin-III
RS014293	GO:0044699	single-organism process	BP	-1.255076548	-7.106748552	-2.698014487	1.18789233	44.66669901	1.08917E-09	1.23577E-08	PREDICTED: probable cytochrome P450 301a1, mitochondrial
RS014374	GO:0044699	single-organism process	BP	-2.757401794	-1.293839431	0.61208896	5.598754173	44.48882029	1.18821E-09	1.3445E-08	PREDICTED: UDP-glucuronosyltransferase 1-9-like
RS004408	GO:0044699	single-organism process	BP	-1.413333885	-1.270757399	0.830141825	5.260573821	44.44877453	1.21172E-09	1.36987E-08	PREDICTED: 3'(2'),5'-bisphosphate nucleotidase 1
RS001352	GO:0044699	single-organism process	BP	-0.632507825	-0.87685044	0.773411943	6.362623028	44.41562096	1.23153E-09	1.38899E-08	PREDICTED: dual 3',5'-cyclic-AMP and -GMP phosphodiesterase 11-like
RS006479	GO:0044699	single-organism process	BP	-1.113422679	0.312593808	1.487887601	5.120184715	44.22549144	1.35157E-09	1.52115E-08	PREDICTED: apyrase
RS004437	GO:0044699	single-organism process	BP	-1.425888965	-2.280207862	-0.816193317	4.489021524	44.20951888	1.36218E-09	1.53172E-08	Monocarboxylate transporter 9
RS002911	GO:0044699	single-organism process	BP	-1.809470992	-2.126048817	-2.690821339	2.723721955	44.05334773	1.47031E-09	1.65184E-08	PREDICTED: cytochrome P450 9e2-like
RS011778	GO:0044699	single-organism process	BP	-1.482690204	-0.163981472	-1.627972092	5.097898358	44.03120475	1.48632E-09	1.66834E-08	PREDICTED: facilitated trehalose transporter Tret1-2 homolog
RS013976	GO:0044699	single-organism process	BP	1.262683995	1.371831144	1.673256576	6.401824333	43.93535672	1.55766E-09	1.7422E-08	PREDICTED: aminoacyl tRNA synthase complex-interacting multifunctional protein 1
RS005258	GO:0044699	single-organism process	BP	-0.331266579	-2.189481258	-2.018410103	5.385456428	43.85577504	1.61949E-09	1.80654E-08	PREDICTED: lipid storage droplets surface-binding protein 1
RS007743	GO:0044699	single-organism process	BP	-1.804323011	-1.669176003	-0.470211701	5.80929799	43.79813802	1.66579E-09	1.85163E-08	PREDICTED: probable phospholipid-transporting ATPase IF
RS005640	GO:0044699	single-organism process	BP	0.969003673	1.718434974	-0.876644168	4.401662752	43.66119041	1.78117E-09	1.9764E-08	PREDICTED: glycine receptor subunit alpha-4
RS012213	GO:0044699	single-organism process	BP	1.307278104	3.558142726	2.111093584	3.711355131	43.61369277	1.82303E-09	2.02106E-08	cypher
RS006807	GO:0044699	single-organism process	BP	-0.111659914	0.788394818	1.55691057	7.684190982	43.55638032	1.87485E-09	2.07668E-08	PREDICTED: peroxiredoxin-6-like
RS008795	GO:0044699	single-organism process	BP	-0.669431828	0.077730985	0.924956243	5.298185262	43.29432987	2.13116E-09	2.35437E-08	PREDICTED: CD151 antigen-like
RS006836	GO:0044699	single-organism process	BP	-2.843096066	0.476407858	1.216523551	2.112665612	43.25863411	2.16868E-09	2.39163E-08	PREDICTED: solute carrier organic anion transporter family member 2A1
RS003050	GO:0044699	single-organism process	BP	-0.547628467	1.078731713	0.376015467	5.671667378	43.14241527	2.29548E-09	2.52925E-08	PREDICTED: NADPH oxidase 5

RS012318	GO:0044699	single-organism process	BP	-1.322255463	-1.602824961	-1.080974124	6.711324032	43.08397409	2.36201E-09	2.60028E-08	PREDICTED: protein FAM13A
RS006296	GO:0044699	single-organism process	BP	0.686204551	1.485233683	0.889580255	6.856267464	42.92668636	2.55081E-09	2.80566E-08	PREDICTED: signal recognition particle receptor subunit beta
RS011734	GO:0044699	single-organism process	BP	-2.971337729	0.243659454	-0.177326697	3.7415488	42.72791998	2.81109E-09	3.08119E-08	PREDICTED: glucose dehydrogenase [FAD, quinone]-like
RS003751	GO:0044699	single-organism process	BP	2.518764	2.048772185	0.932913542	3.838567576	42.72131253	2.82019E-09	3.08847E-08	PREDICTED: protein drumstick
RS009969	GO:0044699	single-organism process	BP	-3.153874476	-7.593721927	-2.696137695	1.315074121	42.48204851	3.17006E-09	3.4566E-08	PREDICTED: aldose reductase-like
RS009578	GO:0044699	single-organism process	BP	-2.77838182	-2.247321578	-2.454512864	4.019688809	42.39569844	3.30671E-09	3.59937E-08	PREDICTED: solute carrier family 28 member 3
RS011394	GO:0044699	single-organism process	BP	-1.130512888	-4.311859926	-0.867929923	2.581686454	42.35833137	3.36765E-09	3.65938E-08	PREDICTED: hydroxyacyl-coenzyme A dehydrogenase, mitochondrial-like
RS010165	GO:0044699	single-organism process	BP	-2.978712613	-4.129324569	-4.260231483	1.233097625	42.25155161	3.54805E-09	3.84877E-08	PREDICTED: cytochrome P450 6k1-like
RS008118	GO:0044699	single-organism process	BP	-0.562153282	-1.452871599	0.194081934	4.903898162	42.23530312	3.57634E-09	3.87611E-08	PREDICTED: dehydrogenase/reductase SDR family member 7
RS003206	GO:0044699	single-organism process	BP	-0.294947237	-0.743983498	2.922416727	0.864847928	42.10964368	3.80283E-09	4.11804E-08	PREDICTED: cytochrome P450 4c3
RS009656	GO:0044699	single-organism process	BP	-0.476171665	-0.276323371	0.841955979	6.857227985	41.94653446	4.11832E-09	4.45203E-08	PREDICTED: rho GTPase-activating protein 18
RS002535	GO:0044699	single-organism process	BP	-0.91575853	-1.304494659	-0.314668602	6.514376839	41.85373159	4.30936E-09	4.64261E-08	PREDICTED: acetyl-CoA carboxylase
RS013524	GO:0044699	single-organism process	BP	-1.108042663	0.09814092	0.458444535	6.588074135	41.78012353	4.46716E-09	4.80849E-08	hypothetical protein
RS004454	GO:0044699	single-organism process	BP	1.479270216	1.56058516	0.252252337	4.193920246	41.7175568	4.60582E-09	4.94929E-08	PREDICTED: ras-related protein Rab-23
RS001737	GO:0044699	single-organism process	BP	0.795534613	1.750543461	1.243157639	8.11677925	41.64579554	4.77015E-09	5.11715E-08	PREDICTED: translocon-associated protein subunit gamma
RS001544	GO:0044699	single-organism process	BP	0.080055725	-1.814392956	-3.057255425	5.225820738	41.53629056	5.03228E-09	5.38458E-08	PREDICTED: alkaline phosphatase 4-like
RS001862	GO:0044699	single-organism process	BP	-1.83463814	-2.520018561	-0.230784861	3.148640444	41.46093404	5.22098E-09	5.57701E-08	PREDICTED: bombesin receptor subtype-3
RS001007	GO:0044699	single-organism process	BP	-0.057453873	2.550863666	4.593751253	0.34449975	41.36132174	5.4813E-09	5.84023E-08	PREDICTED: sodium-dependent nutrient amino acid transporter 1-like
RS015505	GO:0044699	single-organism process	BP	-0.873277592	-1.775991461	-0.249713989	6.67190604	41.13584257	6.11945E-09	6.48178E-08	PREDICTED: probable methylmalonate-semialdehyde dehydrogenase [acylating], mitochondrial
RS010452	GO:0044699	single-organism process	BP	-1.264493454	-3.411377807	-0.656775569	3.802175748	40.96006363	6.66799E-09	7.05094E-08	PREDICTED: transient receptor potential cation channel protein painless
RS014047	GO:0044699	single-organism process	BP	-0.592092988	1.748032789	5.355436168	1.817327481	40.93864928	6.73809E-09	7.11311E-08	PREDICTED: zinc transporter ZIP1-like
RS009628	GO:0044699	single-organism process	BP	-1.046734727	-1.827307043	0.144731166	7.022153366	40.8042965	7.19498E-09	7.56374E-08	PREDICTED: Niemann-Pick C1 protein-like
RS009440	GO:0044699	single-organism process	BP	-1.905319583	0.388036879	1.943885223	4.104469726	40.78879703	7.24964E-09	7.61485E-08	PREDICTED: E3 ubiquitin-protein ligase SIAH1
RS008334	GO:0044699	single-organism process	BP	-1.249649981	-2.54409993	-1.48513853	9.150611402	40.76024334	7.35143E-09	7.71533E-08	PREDICTED: 1,5-anhydro-D-fructose reductase-like
RS005851	GO:0044699	single-organism process	BP	0.526397294	1.41413322	1.673681755	7.829535198	40.74316203	7.413E-09	7.77347E-08	PREDICTED: proton-coupled amino acid transporter 4
RS007222	GO:0044699	single-organism process	BP	0.871635934	1.295444052	-1.298295918	2.421213351	40.60411611	7.93377E-09	8.29882E-08	PREDICTED: muscle segmentation homeobox
RS004948	GO:0044699	single-organism process	BP	0.372490505	1.46727742	2.155346295	8.583374689	40.45310847	8.5408E-09	8.88215E-08	Chymotrypsin BI
RS009222	GO:0044699	single-organism process	BP	-1.042858273	1.531981881	0.833944459	3.778338416	40.34089406	9.02174E-09	9.36684E-08	Peroxidasin-like
RS015221	GO:0044699	single-organism process	BP	-1.706285672	-2.059581608	-1.637011217	3.868239538	40.0532125	1.03819E-08	1.07172E-07	PREDICTED: cytochrome P450 6a9-like
RS007879	GO:0044699	single-organism process	BP	-0.496017353	-0.166118237	0.918665204	6.582220728	39.96605472	1.08331E-08	1.11555E-07	PREDICTED: serine/threonine-protein kinase N
RS006496	GO:0044699	single-organism process	BP	0.251522081	1.149469438	1.045735947	6.062426778	39.80904864	1.16958E-08	1.20145E-07	PREDICTED: ubiquitin-fold modifier-conjugating enzyme 1
RS005087	GO:0044699	single-organism process	BP	-1.968225029	0.898040261	2.102745618	2.388969678	39.77259646	1.19058E-08	1.22201E-07	PREDICTED: GTP cyclohydrolase 1
RS009089	GO:0044699	single-organism process	BP	0.038317941	-1.422332309	-1.528666477	5.001621096	39.70999923	1.22751E-08	1.25583E-07	PREDICTED: probable phosphoserine aminotransferase
RS000244	GO:0044699	single-organism process	BP	-2.077293385	0.322822201	0.471009763	5.277306481	39.6052201	1.2919E-08	1.32063E-07	PREDICTED: gamma-glutamyltranspeptidase 1-like
RS007757	GO:0044699	single-organism process	BP	-2.173834034	-1.722758699	0.546076306	4.342986412	39.5273811	1.34191E-08	1.37065E-07	cytochrome P450
RS012191	GO:0044699	single-organism process	BP	-0.827958312	0.960594434	-0.015691014	7.391207833	39.39645508	1.43044E-08	1.45634E-07	PREDICTED: V-type proton ATPase 116 kDa subunit a
RS005515	GO:0044699	single-organism process	BP	-1.535828202	-1.305216981	-0.409435273	6.908912824	39.33630525	1.47304E-08	1.49608E-07	PREDICTED: A disintegrin and metalloproteinase with thrombospondin motifs 7-like
RS007194	GO:0044699	single-organism process	BP	-0.781842764	0.15844059	0.993190363	7.241530464	39.30278224	1.49733E-08	1.51708E-07	PREDICTED: hexokinase type 2
RS009947	GO:0044699	single-organism process	BP	-0.133867435	1.065636208	0.708920051	6.792021792	39.18991216	1.58209E-08	1.60039E-07	PREDICTED: calcium-binding mitochondrial carrier protein SCaMC-2
RS100013	GO:0044699	single-organism process	BP	-2.527163929	-5.669615447	-2.347573994	2.218055905	39.15268701	1.61109E-08	1.62711E-07	PREDICTED: geranylgeranyl pyrophosphate synthase
RS001009	GO:0044699	single-organism process	BP	-1.20218242	0.618094069	-0.379338777	5.010024276	39.11586683	1.64028E-08	1.65394E-07	PREDICTED: alpha-glucosidase-like
RS010602	GO:0044699	single-organism process	BP	-1.308613642	-0.58422639	-1.999476075	4.600438381	38.94993325	1.77858E-08	1.78339E-07	PREDICTED: cytochrome P450 9e2-like
RS006676	GO:0044699	single-organism process	BP	-1.643188787	-1.466584143	0.183981342	3.745621255	38.83785809	1.87851E-08	1.87613E-07	PREDICTED: adenylate kinase isoenzyme 1
RS006626	GO:0044699	single-organism process	BP	-2.700806188	0.747961514	2.346199654	7.636078588	38.74712028	1.96351E-08	1.95173E-07	chitin synthase
RS007540	GO:0044699	single-organism process	BP	-1.390529334	-1.312124577	-0.217851181	4.81798822	38.69565819	2.01341E-08	1.99881E-07	PREDICTED: atypical protein kinase C
RS003335	GO:0044699	single-organism process	BP	0.43066806	0.052969002	-1.65671712	5.277276724	38.32248305	2.41526E-08	2.37825E-07	PREDICTED: apolipophorins-like
RS007182	GO:0044699	single-organism process	BP	-1.832618585	-0.998643447	0.230873124	4.04126445	38.17861289	2.59077E-08	2.53718E-07	PREDICTED: receptor-type tyrosine-protein phosphatase kappa
RS003552	GO:0044699	single-organism process	BP	-2.156474624	-2.867477226	-2.328741761	3.097576061	38.13053428	2.65221E-08	2.59131E-07	PREDICTED: xanthine dehydrogenase/oxidase-like
RS000859	GO:0044699	single-organism process	BP	-2.022801794	-5.883127386	-3.431068172	2.40785442	38.11492496	2.67247E-08	2.60908E-07	PREDICTED: probable cytochrome P450 6a14
RS010451	GO:0044699	single-organism process	BP	0.264412147	-1.428926603	-1.961517952	3.661432931	38.10668964	2.68322E-08	2.61755E-07	PREDICTED: cytochrome P450 315a1, mitochondrial
RS011264	GO:0044699	single-organism process	BP	-0.733835915	-2.946626233	-1.607155614	3.298835636	37.81625782	3.09132E-08	3.0017E-07	PREDICTED: arrestin domain-containing protein 3
RS010663	GO:0044699	single-organism process	BP	0.715601749	4.612398326	3.003724411	0.401836624	37.79706814	3.12037E-08	3.02757E-07	PREDICTED: carbonic anhydrase 2-like
RS013610	GO:0044699	single-organism process	BP	-1.884472907	-1.020334627	-0.783788058	5.426462338	37.74288835	3.20387E-08	3.1062E-07	PREDICTED: glucose dehydrogenase [FAD, quinone]-like
RS010472	GO:0044699	single-organism process	BP	-0.707846719	-1.359223275	-1.145612806	6.054168603	37.56822123	3.48855E-08	3.377E-07	PREDICTED: isovaleryl-CoA dehydrogenase, mitochondrial
RS012781	GO:0044699	single-organism process	BP	-0.777757392	1.093076306	1.566350271	6.253867256	36.99757405	4.60682E-08	4.43565E-07	PREDICTED: transforming growth factor-beta-induced protein ig-h3-like
RS013592	GO:0044699	single-organism process	BP	-1.435884935	1.094620871	0.340591193	2.672110311	36.86431737	4.91579E-08	4.70795E-07	E3 ubiquitin-protein ligase sina
RS009711	GO:0044699	single-organism process	BP	0.862276886	1.272132135	1.421293308	6.056371062	36.7986326	5.07562E-08	4.85732E-07	PREDICTED: tropinone reductase 2-like
RS003151	GO:0044699	single-organism process	BP	-0.685456445	-3.390981876	-1.99177962	2.900662756	36.76012323	5.17172E-08	4.94178E-07	cytochrome P450
RS007724	GO:0044699	single-organism process	BP	-0.685070011	-3.51329955	0.336899658	1.729555574	36.73271354	5.24123E-08	5.0044E-07	PREDICTED: histidine ammonia-lyase
RS009918	GO:0044699	single-organism process	BP	-3.041004311	-4.02502758	-0.121253316	1.30445851	36.72193195	5.26883E-08	5.02694E-07	PREDICTED: phosphoenolpyruvate carboxykinase [GTP]-like
RS006313	GO:0044699	single-organism process	BP	-1.886967838	-0.586437774	0.058839309	6.903111498	36.59852665	5.59522E-08	5.33431E-07	PREDICTED: mucin-17
RS003709	GO:0044699	single-organism process	BP	-2.735568398	-3.204306593	-3.328272875	0.911281484	36.5712199	5.67013E-08	5.40163E-07	PREDICTED: cytochrome P450 4V2
RS008524	GO:0044699	single-organism process	BP	1.31697469	1.43235307	1.01851404	5.440871033	36.52475366	5.7999E-08	5.51692E-07	PREDICTED: signal recognition particle 9 kDa protein
RS005966	GO:0044699	single-organism process	BP	0.813382742	1.201323264	-0.273628464	6.163653891	36.410532	6.13166E-08	5.8193E-07	PREDICTED: protein O-mannosyltransferase 1
RS011831	GO:0044699	single-organism process	BP	-1.435266558	0.383547956	3.056810998	0.766698698	36.38839562	6.19812E-08	5.87352E-07	PREDICTED: P protein-like
RS000735	GO:0044699	single-organism process	BP	-0.822808135	-0.566823481	0.604494363	4.683837559	36.36127792	6.2805E-08	5.93819E-07	PREDICTED: FMRFamide receptor
RS005308	GO:0044699	single-organism process	BP	-0.596892188	-2.606531853	-0.637963065	3.492025149	36.06925496	7.24011E-08	6.81989E-07	PREDICTED: GTP-binding protein Rhes-like
RS002163	GO:0044699	single-organism process	BP	-0.458782111	1.077749636	0.903170703	4.233761172	36.05963192	7.27411E-08	6.84679E-07	PREDICTED: beta-1,3-galactosyltransferase 5
RS001295	GO:0044699	single-organism process	BP	-2.533567544	-3.585652321	-0.069082698	1.419163476	35.94001788	7.71026E-08	7.2465E-07	PREDICTED: lipase 3-like
RS007780	GO:0044699	single-organism process	BP	-0.274718828	-1.384032675	-1.005743733	7.135030002	35.91237691	7.81471E-08	7.33372E-07	PREDICTED: UTP--glucose-1-phosphate uridylyltransferase
RS002285	GO:0044699	single-organism process	BP	-0.388215159	0.429000459	-0.963951905	6.572062919	35.8619284	8.009E-08	7.51045E-07	Metabotropic glutamate receptor 7
RS003648	GO:0044699	single-organism process	BP	0.799121324	0.883024374	-0.745381312	6.113323662	35.82944046	8.13666E-08	7.62449E-07	PREDICTED: protein singed
RS014465	GO:0044699	single-organism process	BP	-2.203563111	-3.568610423	-0.279641755	5.849726949	35.76065503	8.4137E-08	7.87237E-07	PREDICTED: probable multidrug resistance-associated protein lethal(2)03659
RS011779	GO:0044699	single-organism process	BP	-2.22356949	-3.571952783	-1.230326148	2.104606825	35.6579628	8.84493E-08	8.24523E-07	PREDICTED: facilitated trehalose transporter Tret1-2 homolog
RS005999	GO:0044699	single-organism process	BP	-1.570970326	0.530440776	-0.11933938	4.712096327	35.61481783	9.03262E-08	8.40775E-07	PREDICTED: neurotrimin-like
RS012862	GO:0044699	single-organism process	BP	-0.607441674	-0.230065514	1.014673933	5.194678981	35.47494967	9.66887E-08	8.9867E-07	PREDICTED: fibrillin-2-like
RS011079	GO:0044699	single-organism process	BP	1.606302982	0.958734861	0.270199034	7.619499542	35.24300998	1.08241E-07	1.0016E-06	PREDICTED: monocarboxylate transporter 12
RS007692	GO:0044699	single-organism process	BP	1.298753706	1.017547395	-0.037961486	4.240216468	35.23679427	1.08569E-07	1.0039E-06	PREDICTED: F-box only protein 30
RS006848	GO:0044699	single-organism process	BP	-0.66754309	0.9581648	1.223015946	5.733467437	35.19938959	1.10563E-07	1.02159E-06	PREDICTED: protocadherin-15
RS010978	GO:0044699	single-organism process	BP	-1.244943903	-1.839221985	-1.263048219	7.621355649	35.10486661	1.15766E-07	1.0681E-06	PREDICTED: fatty acid synthase
RS013959	GO:0044699	single-organism process	BP	-0.0336679	-2.765874442	-1.736786071	2.907541159	35.00582949	1.2148E-07	1.11754E-06	PREDICTED: D-arabinitol dehydrogenase 1-like
RS013235	GO:0044699	single-organism process	BP	-2.068035818	-1.64402774	1.426351822	0.894091033	34.98409736	1.22771E-07	1.1286E-06	PREDICTED: glutamate receptor 1-like
RS012786	GO:0044699	single-organism process	BP	-1.725772589	-2.922030511	-0.315113572	3.189291251	34.971992	1.23496E-07	1.13444E-06	PREDICTED: facilitated trehalose transporter Tret1-2 homolog
RS008217	GO:0044699	single-organism process	BP	-0.058063532	0.427477589	-1.164768064	4.782352072	34.91230534	1.27134E-07	1.16531E-06	PREDICTED: calcium-binding protein E63-1
RS009788	GO:0044699	single-organism process	BP	-1.186410373	-2.74936292	-1.181314734	2.694540135	34.80409703	1.34005E-07	1.22294E-06	PREDICTED: D-beta-hydroxybutyrate dehydrogenase, mitochondrial
RS011873	GO:0044699	single-organism process	BP	-1.551877292	-1.463388789	-0.669233879	3.779876253	34.75570287	1.37197E-07	1.25026E-06	PREDICTED: 1-acyl-sn-glycerol-3-phosphate acyltransferase alpha
RS004130	GO:0044699	single-organism process	BP	-0.253006741	0.983334659	-0.778902298	3.6246462	34.60903051	1.47341E-07	1.33883E-06	PREDICTED: kinesin-like protein CG14535
RS004248	GO:0044699	single-organism process	BP	-2.357800174	-1.138542703	0.799750155	3.47254249	34.60029849	1.47968E-07	1.34356E-06	PREDICTED: facilitated trehalose transporter Tret1-like
RS003293	GO:0044699	single-organism process	BP	2.481596928	1.309372061	-1.050028141	1.947806195	34.57037048	1.50138E-07	1.36227E-06	pigment-dispersing factor I precursor
RS009116	GO:0044699	single-organism process	BP	-2.774708917	-1.965598127	1.306984186	1.044379091	34.54483465	1.52014E-07	1.3783E-06	PREDICTED: urea transporter 1-like
RS008881	GO:0044699	single-organism process	BP	-2.536456712	-1.798085063	-1.917971777	2.118086368	34.5143941	1.54281E-07	1.39785E-06	PREDICTED: apolipoprotein D
RS011418	GO:0044699	single-organism process	BP	-0.328802728	1.522013603	-0.732215375	3.957425952	34.42904716	1.60818E-07	1.45395E-06	PREDICTED: putative inorganic phosphate cotransporter
RS013303	GO:0044699	single-organism process	BP	-1.533487737	-1.764835534	-1.412789568	4.061167435	34.36253174	1.66105E-07	1.49959E-06	PREDICTED: trehalase
RS009950	GO:0044699	single-organism process	BP	-0.960859919	1.366385705	0.445541223	3.310327115	34.33223063	1.6857E-07	1.52075E-06	PREDICTED: pancreatic triacylglycerol lipase-like
RS013012	GO:0044699	single-organism process	BP	-1.470953782	-1.559057804	-1.338853711	3.899322122	34.27053677	1.73703E-07	1.56482E-06	PREDICTED: peptidoglycan-recognition protein LB-like
RS013773	GO:0044699	single-organism process	BP	-1.94450872	-1.634357728	0.481509832	8.982406035	34.18942032	1.8069E-07	1.6266E-06	PREDICTED: bifunctional 3'-phosphoadenosine 5'-phosphosulfate synthase
RS002524	GO:0044699	single-organism process	BP	-0.578991198	1.381359439	0.45288263	4.143576647	34.14564597	1.84577E-07	1.6604E-06	PREDICTED: putative fatty acyl-CoA reductase CG5065
RS000365	GO:0044699	single-organism process	BP	-2.043121889	-2.26896537	0.189661525	11.00673959	34.00204383	1.97922E-07	1.77664E-06	PREDICTED: calcium-transporting ATPase sarcoplasmic/endoplasmic reticulum type
RS007032	GO:0044699	single-organism process	BP	-1.972036202	-0.832866298	0.156731051	5.195017285	33.86656984	2.11394E-07	1.89487E-06	PREDICTED: ammonium transporter Rh type A
RS008323	GO:0044699	single-organism process	BP	0.172300495	1.684288353	1.434517397	6.128767325	33.74577634	2.24176E-07	2.00374E-06	PREDICTED: ras and EF-hand domain-containing protein
RS007481	GO:0044699	single-organism process	BP	-3.004029121	-4.75496431	-0.652750642	2.796051045	33.57332715	2.43772E-07	2.17581E-06	PREDICTED: geranylgeranyl pyrophosphate synthase
RS009773	GO:0044699	single-organism process	BP	-2.811480866	0.140271451	0.013519957	2.881280508	33.55652115	2.45771E-07	2.19209E-06	PREDICTED: uncharacterized oxidoreductase YjmC-like
RS001808	GO:0044699	single-organism process	BP	-1.457307761	-1.90386158	0.642631365	1.825702934	33.50863405	2.51557E-07	2.24211E-06	PREDICTED: elongation of very long chain fatty acids protein AAEL008004-like
RS007554	GO:0044699	single-organism process	BP	-1.13592152	0.547754454	-0.035456045	4.77074889	33.47775643	2.55359E-07	2.27279E-06	PREDICTED: rho GTPase-activating protein 7
RS008021	GO:0044699	single-organism process	BP	0.628636726	1.444295451	1.247605819	5.849848653	33.37369484	2.68602E-07	2.38391E-06	PREDICTED: ets DNA-binding protein pokkuri
RS100015	GO:0044699	single-organism process	BP	-3.534953526	-5.250842496	0.994426729	0.454465563	33.26503764	2.83162E-07	2.5096E-06	PREDICTED: geranylgeranyl pyrophosphate synthase
RS013686	GO:0044699	single-organism process	BP	0.294389451	-2.065909015	-0.50139354	4.039478596	33.22682692	2.88467E-07	2.55303E-06	PREDICTED: acyl-CoA Delta(11) desaturase-like
RS013408	GO:0044699	single-organism process	BP	-2.171831305	-2.46836901	-0.048988623	1.521331242	33.15573846	2.98603E-07	2.64087E-06	PREDICTED: gastrin-releasing peptide receptor-like
RS001764	GO:0044699	single-organism process	BP	-1.556165295	-2.551692524	-0.716104852	5.377278034	33.13465313	3.01677E-07	2.66619E-06	Dehydrogenase/reductase SDR family member 11
RS010161	GO:0044699	single-organism process	BP	-2.438095508	-2.352258281	-0.643986362	7.162442842	33.12541031	3.03034E-07	2.67631E-06	PREDICTED: glucose dehydrogenase [FAD, quinone]
RS000843	GO:0044699	single-organism process	BP	-1.977565527	-5.004696471	-4.240016481	1.674804222	33.12335597	3.03337E-07	2.6771E-06	cytochrome P450

RS010847	GO:0044699	single-organism process	BP	1.534407003	2.818886789	1.313018929	2.952010688	32.94017085	3.31563E-07	2.92007E-06	PREDICTED: helix-loop-helix protein delilah
RS000674	GO:0044699	single-organism process	BP	-1.552503519	-0.893745123	-1.927098955	3.032780219	32.90956314	3.36529E-07	2.96173E-06	Broad-complex core protein isoforms 1/2/3/4/5
RS000985	GO:0044699	single-organism process	BP	-0.888120769	-2.33944472	-0.322516664	6.215621628	32.89500862	3.38916E-07	2.98066E-06	PREDICTED: probable cytochrome P450 305a1
RS014538	GO:0044699	single-organism process	BP	-2.188112515	-1.958581739	-1.468968725	1.93400113	32.7768474	3.58932E-07	3.1523E-06	carboxylesterase
RS013606	GO:0044699	single-organism process	BP	2.703077388	2.005591113	-0.078311465	4.987158317	32.7122742	3.70365E-07	3.24592E-06	PREDICTED: glucose dehydrogenase [FAD, quinone]-like
RS001722	GO:0044699	single-organism process	BP	-0.334283753	-2.198611554	-0.278419111	3.485639997	32.69185314	3.74056E-07	3.27598E-06	PREDICTED: solute carrier family 41 member 1-like
RS013669	GO:0044699	single-organism process	BP	-2.349130665	-3.706281097	-0.990999783	3.956179734	32.54254674	4.0218E-07	3.50765E-06	PREDICTED: lipase 3-like
RS008570	GO:0044699	single-organism process	BP	1.045999857	1.589147815	0.921746869	7.502617442	32.52585395	4.05452E-07	3.53375E-06	PREDICTED: protein transport protein Sec61 subunit beta
RS014414	GO:0044699	single-organism process	BP	-3.315038519	-3.830436798	-4.44334774	0.947691129	32.34601125	4.42442E-07	3.85347E-06	PREDICTED: cytochrome P450 4c3
RS000194	GO:0044699	single-organism process	BP	-1.531358076	-6.832056874	-4.047407244	0.911286362	32.30334845	4.517E-07	3.92867E-06	PREDICTED: bone morphogenetic protein 2-like
RS007693	GO:0044699	single-organism process	BP	-0.242378739	-0.621851837	1.493665703	5.076496585	32.00947491	5.20946E-07	4.51534E-06	PREDICTED: zinc transporter ZIP14
RS004347	GO:0044699	single-organism process	BP	-0.916171765	-2.042094379	0.238772477	3.454218425	31.9850429	5.27159E-07	4.55665E-06	PREDICTED: organic cation transporter protein-like
RS004445	GO:0044699	single-organism process	BP	-0.658892664	-1.562277504	-1.82473577	4.229772353	31.970129	5.30988E-07	4.5866E-06	cytochrome P450
RS009061	GO:0044699	single-organism process	BP	-0.381717049	0.872229307	1.423987272	3.354243538	31.77457362	5.83839E-07	5.01903E-06	PREDICTED: elongation of very long chain fatty acids protein AAEL008004
RS007455	GO:0044699	single-organism process	BP	-1.046545392	-0.635037178	0.340581557	5.866349225	31.66588775	6.1545E-07	5.27637E-06	PREDICTED: glutathione peroxidase 6
RS014319	GO:0044699	single-organism process	BP	0.279760306	1.081767409	-0.500139447	8.021288646	31.64386012	6.22062E-07	5.3258E-06	PREDICTED: nuclear hormone receptor E75
RS007019	GO:0044699	single-organism process	BP	-1.271050296	-1.444219505	-0.860996008	5.239606583	31.58048012	6.41485E-07	5.4772E-06	PREDICTED: insulin-like receptor
RS013209	GO:0044699	single-organism process	BP	-0.459455181	-0.353978647	1.234885473	6.428228507	31.52472151	6.59072E-07	5.61974E-06	PREDICTED: sodium-coupled monocarboxylate transporter 1
RS014192	GO:0044699	single-organism process	BP	0.688964938	1.567107088	0.870440333	7.888499222	31.51414601	6.62462E-07	5.64482E-06	PREDICTED: myosin regulatory light chain sqh
RS007469	GO:0044699	single-organism process	BP	-0.888748458	-2.227463608	-1.919844509	7.982695057	31.31984973	7.27926E-07	6.17756E-06	PREDICTED: retinal dehydrogenase 1
RS002862	GO:0044699	single-organism process	BP	-0.125821873	-1.516493062	-1.54493594	3.292859449	31.03344956	8.36374E-07	7.0504E-06	PREDICTED: cytochrome P450 306a1
RS006814	GO:0044699	single-organism process	BP	-0.514796382	1.014347124	0.975336891	4.041760153	30.97725487	8.59474E-07	7.23543E-06	PREDICTED: large neutral amino acids transporter small subunit 2
RS003154	GO:0044699	single-organism process	BP	-0.695769745	-2.602251526	-2.026678427	5.229628303	30.89958714	8.92452E-07	7.49803E-06	PREDICTED: probable cytochrome P450 6a14
RS002614	GO:0044699	single-organism process	BP	0.68095135	-0.62219164	-2.045010142	3.816147986	30.86882145	9.05862E-07	7.60055E-06	PREDICTED: MOXD1 homolog 1
RS003761	GO:0044699	single-organism process	BP	0.226917268	2.017264642	-0.464263505	1.612100723	30.83684088	9.20015E-07	7.70903E-06	PREDICTED: isthmin-1-like
RS010237	GO:0044699	single-organism process	BP	-0.598284446	-0.986044671	0.3758501	5.887852127	30.75720854	9.56222E-07	8.00709E-06	PREDICTED: calcium/calmodulin-dependent protein kinase type 1
RS001674	GO:0044699	single-organism process	BP	-0.725177387	-0.370012813	0.932400504	6.657705976	30.72741593	9.70131E-07	8.11276E-06	PREDICTED: cystathionine beta-synthase
RS011736	GO:0044699	single-organism process	BP	-0.840871335	-2.1975492	-1.372225226	8.82923978	30.69100612	9.87403E-07	8.24625E-06	apolipophorin-III
RS006320	GO:0044699	single-organism process	BP	1.668458777	1.549661536	0.932473469	6.183206129	30.51118393	1.07731E-06	8.96741E-06	PREDICTED: bumetanide-sensitive sodium-(potassium)-chloride cotransporter
RS003917	GO:0044699	single-organism process	BP	0.904473895	0.736473947	-0.596479059	4.896495196	30.47299505	1.09744E-06	9.12563E-06	PREDICTED: asparagine synthetase [glutamine-hydrolyzing]
RS007713	GO:0044699	single-organism process	BP	-1.593472957	-0.392083561	-0.180235411	5.938924251	30.40817878	1.13245E-06	9.40771E-06	PREDICTED: ATP-binding cassette sub-family D member 2
RS003904	GO:0044699	single-organism process	BP	-2.706690593	-2.534495324	0.490780854	4.216110096	30.29657835	1.19537E-06	9.89777E-06	PREDICTED: facilitated trehalose transporter Tret1-like
RS012480	GO:0044699	single-organism process	BP	-2.211703227	-4.570647765	-2.199566529	0.771754643	30.21889923	1.24121E-06	1.02639E-05	PREDICTED: retinol dehydrogenase 11-like
RS010500	GO:0044699	single-organism process	BP	1.19026545	2.873457875	1.841995752	12.00045446	30.1935168	1.25657E-06	1.03772E-05	PREDICTED: tubulin alpha-1 chain
RS000193	GO:0044699	single-organism process	BP	-0.612906575	0.552707825	0.864925878	6.395375097	30.19397061	1.25629E-06	1.03772E-05	PREDICTED: b(0,+)-type amino acid transporter 1
RS003370	GO:0044699	single-organism process	BP	1.148136507	-0.965311261	-0.4811304	3.625502469	30.18481425	1.26188E-06	1.04142E-05	PREDICTED: UDP-glucuronosyltransferase 1-7C-like
RS001411	GO:0044699	single-organism process	BP	-1.439234611	-1.190473147	0.602093209	4.878972892	30.16795384	1.27223E-06	1.04928E-05	PREDICTED: globin
RS009521	GO:0044699	single-organism process	BP	-0.593455229	1.121217687	0.256757641	3.629605813	30.1364228	1.29181E-06	1.06473E-05	PREDICTED: putative fatty acyl-CoA reductase CG5065
RS002792	GO:0044699	single-organism process	BP	1.416282241	0.881694727	0.182668444	4.447509939	29.97406772	1.3975E-06	1.14958E-05	PREDICTED: mitogen-activated protein kinase kinase kinase 7
RS006007	GO:0044699	single-organism process	BP	-0.453303397	0.571846832	0.972958891	4.133697317	29.94667021	1.41617E-06	1.16418E-05	PREDICTED: innexin inx7
RS006642	GO:0044699	single-organism process	BP	-2.419823612	0.944025566	1.645528914	4.053624516	29.85374943	1.48135E-06	1.21538E-05	PREDICTED: aromatic-L-amino-acid decarboxylase
RS015219	GO:0044699	single-organism process	BP	-2.352696213	-1.674333088	-1.332318565	3.405274693	29.83241182	1.49674E-06	1.22641E-05	PREDICTED: cytochrome P450 6k1-like
RS006355	GO:0044699	single-organism process	BP	0.969374194	1.345486678	1.0281371	5.974897614	29.7627744	1.54807E-06	1.26512E-05	PREDICTED: D-tyrosyl-tRNA(Tyr) deacylase 1
RS004450	GO:0044699	single-organism process	BP	-0.62579013	-1.60519533	0.087177481	5.243141125	29.71290013	1.58591E-06	1.29108E-05	PREDICTED: cytochrome P450 4c3
RS004782	GO:0044699	single-organism process	BP	-0.56235577	-0.281140516	0.947317924	4.616609736	29.69315735	1.60115E-06	1.30264E-05	tartan
RS003469	GO:0044699	single-organism process	BP	-1.12741584	-1.814734525	-1.321813002	5.031194954	29.68202165	1.6098E-06	1.30799E-05	PREDICTED: PBAN-type neuropeptides-like
RS015086	GO:0044699	single-organism process	BP	-0.031343432	1.660408125	0.049542551	2.372004734	29.58636931	1.68611E-06	1.36559E-05	PREDICTED: G-protein coupled receptor moody
RS014705	GO:0044699	single-organism process	BP	-0.846250988	-1.231073838	-0.973701558	6.39649342	29.48407024	1.77171E-06	1.43215E-05	PREDICTED: tyrosine-protein phosphatase 99A
RS005497	GO:0044699	single-organism process	BP	1.170662464	-1.020287306	-0.883108523	1.992265709	29.45354334	1.79809E-06	1.45254E-05	PREDICTED: low-density lipoprotein receptor-related protein 2
RS011175	GO:0044699	single-organism process	BP	-0.885455574	-1.277486072	0.737920476	7.001021188	29.37999588	1.86326E-06	1.5023E-05	PREDICTED: sorbitol dehydrogenase-like
RS000410	GO:0044699	single-organism process	BP	1.366752185	1.500582927	1.519736248	4.986074654	29.36983146	1.87245E-06	1.50874E-05	PREDICTED: metabotropic glutamate receptor 6
RS003348	GO:0044699	single-organism process	BP	0.435104118	0.896643807	1.206838528	5.259653033	29.30705068	1.93022E-06	1.5533E-05	PREDICTED: 39S ribosomal protein L40, mitochondrial
RS011271	GO:0044699	single-organism process	BP	-0.083239972	2.141765807	-0.104489587	2.286387209	29.30458209	1.93253E-06	1.55417E-05	PREDICTED: dynein beta chain, ciliary
RS014898	GO:0044699	single-organism process	BP	-0.634960839	-1.801610032	0.576713947	3.693735802	29.21635917	2.01683E-06	1.61783E-05	PREDICTED: ATP-binding cassette sub-family G member 1
RS012711	GO:0044699	single-organism process	BP	-1.349382482	-1.024824699	0.156872307	5.314908717	29.15556389	2.07705E-06	1.66401E-05	PREDICTED: beta,beta-carotene 9',10'-oxygenase
RS012844	GO:0044699	single-organism process	BP	0.349465935	0.921460552	-0.225472148	6.495114856	29.13494206	2.09788E-06	1.6775E-05	PREDICTED: glutamyl aminopeptidase-like
RS010599	GO:0044699	single-organism process	BP	-1.733278397	-1.466298329	-1.577728369	3.172823323	29.12935359	2.10356E-06	1.68098E-05	PREDICTED: gastrin-releasing peptide receptor-like
RS002190	GO:0044699	single-organism process	BP	-1.585867798	-2.762372308	0.098229096	2.73757327	29.10799577	2.12541E-06	1.69629E-05	PREDICTED: fatty acid 2-hydroxylase
RS000996	GO:0044699	single-organism process	BP	-1.508231266	-0.156640833	0.876498273	2.831732428	29.09435446	2.13949E-06	1.70644E-05	PREDICTED: zinc transporter ZIP3-like
RS013347	GO:0044699	single-organism process	BP	-0.641509733	-2.419819909	-1.425319202	2.273203864	29.08562847	2.14854E-06	1.71258E-05	PREDICTED: tyramine beta-hydroxylase
RS014930	GO:0044699	single-organism process	BP	0.323746001	-0.667582543	-0.725755425	6.121124277	29.05530974	2.18029E-06	1.73569E-05	PREDICTED: bifunctional purine biosynthesis protein PURH
RS001816	GO:0044699	single-organism process	BP	-0.83021237	0.593772319	0.329841861	5.05828422	28.9658157	2.27678E-06	1.81135E-05	PREDICTED: rhophilin-1
RS005491	GO:0044699	single-organism process	BP	-0.937465516	-1.630388798	-0.209059704	4.107611972	28.88192352	2.37108E-06	1.88281E-05	PREDICTED: voltage-gated potassium channel subunit beta-2
RS009579	GO:0044699	single-organism process	BP	-0.361377028	0.937501921	0.004604885	5.160969196	28.85056945	2.40732E-06	1.91039E-05	PREDICTED: solute carrier family 28 member 3-like
RS001887	GO:0044699	single-organism process	BP	-2.77939121	-1.454591278	0.286615073	3.02093845	28.79531455	2.47254E-06	1.95721E-05	PREDICTED: protein croquemort-like
RS007069	GO:0044699	single-organism process	BP	-1.090693094	-0.787452036	0.487574628	8.688494739	28.79647484	2.47115E-06	1.95721E-05	PREDICTED: gelsolin
RS001245	GO:0044699	single-organism process	BP	-1.523639946	-1.345745342	0.215937374	5.416585779	28.67759633	2.61741E-06	2.0641E-05	PREDICTED: short-chain dehydrogenase/reductase family 16C member 6-like
RS011850	GO:0044699	single-organism process	BP	-0.767278606	-0.313949384	0.713484854	8.083358391	28.67013092	2.62688E-06	2.07027E-05	PREDICTED: trimeric intracellular cation channel type B
RS000987	GO:0044699	single-organism process	BP	-1.331119303	-1.642345687	-1.067316453	3.442657955	28.43189591	2.94765E-06	2.31582E-05	PREDICTED: probable cytochrome P450 305a1
RS008885	GO:0044699	single-organism process	BP	-1.592550754	-2.358375405	-0.949039702	4.983900477	28.41800515	2.96751E-06	2.32998E-05	PREDICTED: facilitated trehalose transporter Tret1-2 homolog
RS008269	GO:0044699	single-organism process	BP	1.176389545	-0.872424518	-2.454278616	1.905046295	28.39266837	3.00409E-06	2.35576E-05	PREDICTED: protein NDNF
RS010803	GO:0044699	single-organism process	BP	0.859925193	0.265538059	-0.203254243	6.865383484	28.26911395	3.18901E-06	2.48992E-05	PREDICTED: F-box-like/WD repeat-containing protein TBL1XR1
RS004073	GO:0044699	single-organism process	BP	-1.342551195	-2.1206434	-1.672678297	4.715867182	28.24980452	3.21892E-06	2.50861E-05	PREDICTED: cytochrome P450 9e2-like
RS009528	GO:0044699	single-organism process	BP	-1.162111469	-0.889324468	-0.730012253	6.348609225	28.24598445	3.22487E-06	2.51169E-05	PREDICTED: extended synaptotagmin-2
RS005732	GO:0044699	single-organism process	BP	1.042793426	1.73269194	-0.726780556	1.781153007	28.23770506	3.2378E-06	2.5202E-05	PREDICTED: protein Wnt-5b-like
RS010090	GO:0044699	single-organism process	BP	0.323234855	1.546465547	-0.132352818	2.938456182	28.16134763	3.35955E-06	2.60531E-05	PREDICTED: cyclic nucleotide-gated cation channel subunit A-like
RS006150	GO:0044699	single-organism process	BP	-1.731195553	-1.104009887	-0.435849878	6.16428314	28.13074802	3.40961E-06	2.6425E-05	PREDICTED: 6-phosphofructo-2-kinase/fructose-2,6-bisphosphatase 1
RS010957	GO:0044699	single-organism process	BP	0.104578349	-0.713610498	-0.868724544	6.502009154	28.08418135	3.48723E-06	2.69933E-05	PREDICTED: ornithine aminotransferase, mitochondrial
RS008631	GO:0044699	single-organism process	BP	-0.45372484	0.018443887	2.172529191	1.173123173	28.08258513	3.48992E-06	2.69976E-05	PREDICTED: glutamate receptor ionotropic, delta-1-like
RS013770	GO:0044699	single-organism process	BP	-2.729831026	-1.299649688	-0.212543539	6.435468894	28.01025099	3.61409E-06	2.79238E-05	PREDICTED: peroxidase-like
RS007102	GO:0044699	single-organism process	BP	-0.78190331	-0.653917209	0.250142998	5.561485709	27.98775305	3.6536E-06	2.81945E-05	PREDICTED: glycerol-3-phosphate dehydrogenase [NAD(+)], cytoplasmic
RS007624	GO:0044699	single-organism process	BP	-1.33998228	-3.692172416	0.752205035	1.953523445	27.98010021	3.66714E-06	2.82816E-05	PREDICTED: tyrosine aminotransferase
RS008591	GO:0044699	single-organism process	BP	-0.810554716	-1.195261701	-0.70340811	6.355902668	27.88153137	3.84605E-06	2.95348E-05	PREDICTED: myosin light chain kinase, smooth muscle-like
RS011364	GO:0044699	single-organism process	BP	-0.545238764	-0.491708172	1.286107834	5.019541499	27.75006355	4.09829E-06	3.13953E-05	PREDICTED: tetratricopeptide repeat protein 36
RS004528	GO:0044699	single-organism process	BP	0.600875429	1.093315864	0.014760964	6.884013552	27.64527681	4.31111E-06	3.29855E-05	PREDICTED: microsomal triglyceride transfer protein large subunit
RS008887	GO:0044699	single-organism process	BP	-0.760329305	-4.339267866	-1.430676324	3.320036492	27.63642804	4.32958E-06	3.3078E-05	PREDICTED: facilitated trehalose transporter Tret1-like
RS011800	GO:0044699	single-organism process	BP	-1.018570964	-2.603412306	-0.804726968	3.250067261	27.61409107	4.37655E-06	3.33848E-05	PREDICTED: serine dehydratase-like
RS009913	GO:0044699	single-organism process	BP	1.394144455	1.465286213	0.840669485	8.199178881	27.5665156	4.4783E-06	3.41402E-05	PREDICTED: glycine--tRNA ligase
RS012143	GO:0044699	single-organism process	BP	-0.727254854	-1.471091081	0.213555621	4.71227261	27.4899169	4.6471E-06	3.53842E-05	PREDICTED: ATP-binding cassette sub-family A member 3
RS006124	GO:0044699	single-organism process	BP	1.059219965	0.250242816	-0.925285807	3.554006542	27.48741307	4.65272E-06	3.54057E-05	PREDICTED: putative sodium-dependent multivitamin transporter
RS003901	GO:0044699	single-organism process	BP	-0.740860349	-0.481682425	0.865536129	4.881046728	27.44281021	4.75404E-06	3.61112E-05	PREDICTED: PR domain zinc finger protein 5
RS004201	GO:0044699	single-organism process	BP	-2.055567806	1.418055941	1.915555039	0.827431243	27.43373258	4.77493E-06	3.6248E-05	PREDICTED: peroxidase
RS006269	GO:0044699	single-organism process	BP	-1.78180142	-1.191554952	-0.830372469	4.550094908	27.39894348	4.85584E-06	3.68178E-05	PREDICTED: organic cation transporter 1
RS012590	GO:0044699	single-organism process	BP	-0.22021637	0.861437679	0.641734274	6.761709937	27.36974453	4.9248E-06	3.72958E-05	PREDICTED: high affinity copper uptake protein 1
RS011231	GO:0044699	single-organism process	BP	0.18553788	-1.400278484	-0.907078394	4.077363686	27.29383163	5.10869E-06	3.86419E-05	PREDICTED: prolyl 3-hydroxylase 2-like
RS006155	GO:0044699	single-organism process	BP	-0.731406145	0.899350938	0.931563636	7.523650564	27.17761832	5.40356E-06	4.08478E-05	PREDICTED: integrin alpha-PS2
RS006613	GO:0044699	single-organism process	BP	0.89197901	-0.33137349	-0.039728003	6.264408579	27.12230889	5.5498E-06	4.1903E-05	PREDICTED: enolase-phosphatase E1
RS010268	GO:0044699	single-organism process	BP	0.024988664	0.644055147	1.144265924	5.03520594	27.10742401	5.58982E-06	4.21799E-05	PREDICTED: putative serine/threonine-protein kinase YPL150W
RS011875	GO:0044699	single-organism process	BP	-0.988365188	-4.039282611	-1.254284329	7.158723379	27.0943218	5.62529E-06	4.23967E-05	cytochrome P450
RS011038	GO:0044699	single-organism process	BP	-1.808830614	-0.668580619	0.921251651	1.961697276	27.07718778	5.67202E-06	4.27233E-05	PREDICTED: protein giant-lens-like
RS006251	GO:0044699	single-organism process	BP	-1.563247844	-1.222856026	-0.413903114	4.037779446	27.07508163	5.67779E-06	4.27412E-05	PREDICTED: eye-specific diacylglycerol kinase
RS013123	GO:0044699	single-organism process	BP	0.964292094	1.142038786	0.572450646	6.991719618	27.01145786	5.85488E-06	4.38907E-05	GM20973
RS009033	GO:0044699	single-organism process	BP	-0.740817184	-4.219002234	-1.406053468	2.237145053	26.99292634	5.90749E-06	4.42588E-05	PREDICTED: aminomethyltransferase, mitochondrial
RS008499	GO:0044699	single-organism process	BP	-0.606497585	1.732175829	-0.382830219	1.497372509	26.96568663	5.98568E-06	4.47913E-05	PREDICTED: patched domain-containing protein 3
RS006511	GO:0044699	single-organism process	BP	1.156899451	0.373824996	0.48135166	6.95930305	26.95322278	6.0218E-06	4.50348E-05	PREDICTED: ubiquitin-conjugating enzyme E2 R2
RS002048	GO:0044699	single-organism process	BP	-1.620721672	-1.803353702	-1.262769398	6.263303332	26.83922426	6.36242E-06	4.74694E-05	PREDICTED: leucine-rich repeat-containing protein let-4
RS014856	GO:0044699	single-organism process	BP	0.647633895	0.713937322	1.209349979	7.147306971	26.81562073	6.43531E-06	4.79315E-05	PREDICTED: lysine--tRNA ligase
RS004063	GO:0044699	single-organism process	BP	0.893241263	0.022671451	-0.849442139	7.363097612	26.8149568	6.43738E-06	4.79315E-05	PREDICTED: endoplasmin
RS005093	GO:0044699	single-organism process	BP	-1.490825093	-1.761448973	-3.067688799	4.130528041	26.76253338	6.60231E-06	4.90268E-05	PREDICTED: trehalase
RS012741	GO:0044699	single-organism process	BP	-1.060191661	0.585439948	0.026171952	4.628484614	26.6687965	6.90781E-06	5.10845E-05	PREDICTED: organic cation transporter protein
RS000895	GO:0044699	single-organism process	BP	1.014348253	1.385977981	0.68384576	7.930053973	26.65746374	6.94569E-06	5.13345E-05	PREDICTED: translocon-associated protein subunit beta

RS007480	GO:0044699	single-organism process	BP	-1.878202197	-3.699684825	-2.385750326	0.760702052	26.65614984	6.95009E-06	5.13369E-05	PREDICTED: geranylgeranyl pyrophosphate synthase
RS005548	GO:0044699	single-organism process	BP	-0.600191617	0.2714556	0.994050165	5.95748448	26.64118427	7.00046E-06	5.16484E-05	PREDICTED: coronin-6
RS013178	GO:0044699	single-organism process	BP	-0.611525718	0.155938726	1.161874022	8.971086766	26.55931826	7.2825E-06	5.35411E-05	PREDICTED: pericentriolar material 1 protein
RS100009	GO:0044699	single-organism process	BP	-1.446320627	-1.976955707	-3.482764589	2.944589037	26.3560744	8.03267E-06	5.88502E-05	PREDICTED: cysteine dioxygenase type 1
RS000627	GO:0044699	single-organism process	BP	-1.23214239	-1.256196129	-0.507713368	5.68807099	26.33446348	8.11684E-06	5.93977E-05	PREDICTED: 5-aminolevulinate synthase, erythroid-specific, mitochondrial
RS015101	GO:0044699	single-organism process	BP	-0.869924153	-1.646224769	-1.333222707	4.11216481	26.27474776	8.35401E-06	6.10623E-05	PREDICTED: 2-aminoethylphosphonate--pyruvate transaminase-like
RS004895	GO:0044699	single-organism process	BP	-0.841584594	-0.937470527	2.218371569	3.156774089	26.261831	8.40621E-06	6.14082E-05	PREDICTED: carbonic anhydrase 2-like
RS010954	GO:0044699	single-organism process	BP	-1.068257688	-1.034828789	-1.13235545	6.908854825	26.1509141	8.86808E-06	6.46697E-05	CG2082
RS005259	GO:0044699	single-organism process	BP	-1.342701938	-1.55617572	-1.419719902	6.173858534	26.03421943	9.38135E-06	6.82546E-05	PREDICTED: lipid storage droplets surface-binding protein 2
RS001429	GO:0044699	single-organism process	BP	-0.615461223	1.011192126	1.583319425	5.894267382	25.95527059	9.7453E-06	7.07799E-05	expansion
RS006683	GO:0044699	single-organism process	BP	-1.231113811	0.666436506	0.417461744	3.798041372	25.93631354	9.83477E-06	7.13474E-05	PREDICTED: peroxiredoxin-6-like
RS003158	GO:0044699	single-organism process	BP	-1.077129559	-2.015740945	0.055274694	4.91360724	25.89613232	1.00271E-05	7.27009E-05	PREDICTED: probable cytochrome P450 6a14
RS010567	GO:0044699	single-organism process	BP	0.24659093	0.125949775	1.097396623	5.963000975	25.88149489	1.00981E-05	7.31315E-05	PREDICTED: 4-hydroxybenzoate polyprenyltransferase, mitochondrial
RS014872	GO:0044699	single-organism process	BP	0.103900993	0.11224879	1.250966703	5.119341431	25.84071442	1.02986E-05	7.43747E-05	PREDICTED: putative inorganic phosphate cotransporter
RS004692	GO:0044699	single-organism process	BP	-1.533477303	-1.581672673	-0.440490513	3.98590693	25.83868371	1.03087E-05	7.43996E-05	PREDICTED: histone deacetylase 4
RS010135	GO:0044699	single-organism process	BP	-1.587997138	-2.925621596	-0.408732266	3.739912878	25.80914978	1.04564E-05	7.5423E-05	PREDICTED: protein henna
RS000229	GO:0044699	single-organism process	BP	-0.215915746	-1.250240772	-1.120772701	4.633851177	25.70129786	1.10143E-05	7.92202E-05	PREDICTED: condensin complex subunit 1
RS011771	GO:0044699	single-organism process	BP	-1.004497255	-1.956314668	-1.351147219	2.492829585	25.68494255	1.11015E-05	7.97559E-05	PREDICTED: putative phosphatidate phosphatase
RS000719	GO:0044699	single-organism process	BP	-0.394780773	-1.247672358	-1.502851389	6.140116665	25.60119706	1.15586E-05	8.29929E-05	PREDICTED: aquaporin AQPAn.G
RS005367	GO:0044699	single-organism process	BP	0.752759732	-0.818531129	-1.255874136	1.958767366	25.57018925	1.17326E-05	8.40505E-05	PREDICTED: serendipity locus protein alpha-like
RS014309	GO:0044699	single-organism process	BP	-0.256294696	0.568800721	-0.467831763	5.72893143	25.41365969	1.26516E-05	9.04795E-05	PREDICTED: high affinity cAMP-specific and IBMX-insensitive 3',5'-cyclic phosphodiesterase 8A
RS002844	GO:0044699	single-organism process	BP	-2.433049289	0.181852058	0.537757818	2.82301503	25.3938178	1.27731E-05	9.11415E-05	dumpy
RS003282	GO:0044699	single-organism process	BP	-1.578996502	-3.030953253	-0.903078628	1.646890882	25.35897562	1.29893E-05	9.26315E-05	PREDICTED: aldose reductase-like
RS000061	GO:0044699	single-organism process	BP	1.966649145	0.548084611	1.579526008	5.408238621	25.35538124	1.30118E-05	9.26871E-05	PREDICTED: somatomedin-B and thrombospondin type-1 domain-containing protein-like
RS003902	GO:0044699	single-organism process	BP	0.212010943	1.72144821	0.98329227	4.242513254	25.33598899	1.31339E-05	9.3504E-05	PREDICTED: facilitated trehalose transporter Tret1
RS013310	GO:0044699	single-organism process	BP	-1.594173173	0.30942411	0.502900611	3.784418211	25.32704004	1.31907E-05	9.38549E-05	PREDICTED: sodium- and chloride-dependent transporter XTRP3
RS009968	GO:0044699	single-organism process	BP	1.704437228	-0.761060399	-0.737051902	1.342557732	25.27753952	1.35089E-05	9.59027E-05	PREDICTED: citron Rho-interacting kinase
RS001122	GO:0044699	single-organism process	BP	-0.967052962	-1.40095345	-0.827981443	5.092727615	25.12581142	1.45329E-05	0.000102479	PREDICTED: cytochrome P450 9e2-like
RS000863	GO:0044699	single-organism process	BP	-0.29909992	-1.336766347	-1.031318313	6.934182187	25.07064659	1.49241E-05	0.000105003	PREDICTED: DNA-directed RNA polymerases I, II, and III subunit RPABC3
RS004290	GO:0044699	single-organism process	BP	-1.463452305	0.814025605	0.947980611	6.036220009	25.03938128	1.51505E-05	0.000106358	PREDICTED: peroxidase-like
RS007848	GO:0044699	single-organism process	BP	1.344212016	1.824281189	1.757263797	8.823057104	25.01649455	1.53184E-05	0.000107416	PREDICTED: 60S ribosomal protein L44
RS010653	GO:0044699	single-organism process	BP	0.929493425	1.239889553	0.672676716	6.281928504	24.97161216	1.5653E-05	0.000109579	PREDICTED: ribulose-phosphate 3-epimerase
RS011188	GO:0044699	single-organism process	BP	0.675723205	1.035772933	1.05815662	6.319099093	24.89921245	1.62081E-05	0.00011334	PREDICTED: 39S ribosomal protein L36, mitochondrial
RS011335	GO:0044699	single-organism process	BP	-0.98070178	-0.732116906	0.489172582	4.044187601	24.89783035	1.62189E-05	0.000113352	Neuromedin-B receptor
RS000748	GO:0044699	single-organism process	BP	1.344647193	1.566125604	1.310427631	8.634755939	24.87013453	1.64366E-05	0.00011481	PREDICTED: 40S ribosomal protein S23
RS007662	GO:0044699	single-organism process	BP	0.47851066	0.113879849	-0.926079851	4.757841791	24.85870331	1.65273E-05	0.000115316	PREDICTED: inactive ubiquitin thioesterase FAM105A
RS010160	GO:0044699	single-organism process	BP	-2.894349966	-2.510383916	-0.914696982	6.182434793	24.85038105	1.65936E-05	0.000115714	PREDICTED: glucose dehydrogenase [FAD, quinone]
RS002115	GO:0044699	single-organism process	BP	0.639862723	-0.551456572	-0.969952879	3.381687325	24.75074446	1.74087E-05	0.000120997	PREDICTED: protein regulator of cytokinesis 1-like
RS007989	GO:0044699	single-organism process	BP	-0.054366714	0.548253106	1.63458217	5.065417283	24.68932094	1.7931E-05	0.000124489	PREDICTED: glutaredoxin domain-containing cysteine-rich protein CG31559-like
RS013319	GO:0044699	single-organism process	BP	1.360955707	-0.219010225	-1.347093545	2.439854767	24.65746026	1.8208E-05	0.000126273	PREDICTED: transcription factor E2F7
RS010605	GO:0044699	single-organism process	BP	-3.36317076	-3.777857152	-2.173207779	0.169050824	24.65530687	1.82269E-05	0.000126335	cytochrome P450
RS000419	GO:0044699	single-organism process	BP	0.040399421	-0.493996515	-1.164051968	4.981441137	24.64016581	1.83602E-05	0.000127118	PREDICTED: peroxisomal acyl-coenzyme A oxidase 3
RS007582	GO:0044699	single-organism process	BP	-1.229970149	-1.592228859	-1.074598552	7.903923744	24.61081903	1.86213E-05	0.000128785	PREDICTED: malate dehydrogenase, cytoplasmic
RS005123	GO:0044699	single-organism process	BP	0.781802156	0.484030121	-1.078810264	3.903771807	24.55032113	1.91713E-05	0.00013237	PREDICTED: kynurenine formamidase-like
RS003638	GO:0044699	single-organism process	BP	-0.69625377	-0.738048152	0.351114728	5.7591544	24.53074045	1.93528E-05	0.00013355	PREDICTED: ATP-binding cassette sub-family G member 4
RS008160	GO:0044699	single-organism process	BP	-1.86019723	-1.119619791	1.214513641	3.193269805	24.5107924	1.95394E-05	0.000134543	PREDICTED: UDP-glucuronosyltransferase 2C1-like
RS011742	GO:0044699	single-organism process	BP	-0.336125278	0.161852025	0.904475844	7.200905914	24.4380114	2.02356E-05	0.000139033	PREDICTED: NADH dehydrogenase [ubiquinone] iron-sulfur protein 8, mitochondrial-like
RS004915	GO:0044699	single-organism process	BP	-0.614675035	-1.44127198	-1.283158317	7.861317536	24.36804538	2.09282E-05	0.000143713	Apolipophorin
RS005461	GO:0044699	single-organism process	BP	-1.570191565	-0.758659476	-0.541009138	7.57624513	24.31985122	2.1419E-05	0.000146843	PREDICTED: solute carrier family 22 member 21-like
RS003144	GO:0044699	single-organism process	BP	-1.685219874	-7.396574181	-4.642253479	1.314837961	24.25775	2.20684E-05	0.000151048	PREDICTED: ejaculatory bulb-specific protein 3
RS003551	GO:0044699	single-organism process	BP	-1.777164237	-2.890195639	-1.40533891	2.518693384	24.2447302	2.2207E-05	0.000151915	PREDICTED: xanthine dehydrogenase/oxidase-like
RS000465	GO:0044699	single-organism process	BP	-1.538170058	-1.210552577	0.127049257	2.419553677	24.23973387	2.22604E-05	0.000152197	cytochrome P450
RS013890	GO:0044699	single-organism process	BP	-2.124685921	-2.486942049	-1.307623114	4.055795719	24.21562886	2.25199E-05	0.000153888	PREDICTED: dual specificity protein phosphatase 18
RS000400	GO:0044699	single-organism process	BP	-1.44281387	-0.212784112	-0.371668202	5.011133563	24.18843219	2.28164E-05	0.000155829	PREDICTED: sn1-specific diacylglycerol lipase beta-like
RS002863	GO:0044699	single-organism process	BP	-2.617591534	-0.821640073	-0.653622318	4.232875894	24.11659321	2.36183E-05	0.000161044	PREDICTED: cytochrome P450 18a1
RS011584	GO:0044699	single-organism process	BP	-0.339551107	1.336210164	-0.58024509	1.751457913	24.07277721	2.41211E-05	0.000164117	PREDICTED: cardioacceleratory peptide receptor-like
RS003139	GO:0044699	single-organism process	BP	-0.462942241	-0.484276182	0.670801183	4.994154039	24.0689115	2.4166E-05	0.000164334	PREDICTED: zinc transporter 1
RS007526	GO:0044699	single-organism process	BP	-1.223662153	-0.387211114	-0.507976241	5.27455292	24.05874887	2.42843E-05	0.00016505	PREDICTED: phospholipid-transporting ATPase ID
RS013553	GO:0044699	single-organism process	BP	-1.464621695	0.395111675	-1.145633191	5.594700246	24.02664772	2.4662E-05	0.000167526	PREDICTED: transforming growth factor-beta-induced protein ig-h3
RS000811	GO:0044699	single-organism process	BP	-1.065863601	-1.07637172	0.584455389	3.196340646	24.02535768	2.46773E-05	0.00016754	PREDICTED: tyramine/octopamine receptor
RS005434	GO:0044699	single-organism process	BP	-0.778815577	-1.85986502	-1.933168126	2.416920689	24.0141541	2.48106E-05	0.000168354	PREDICTED: ATP-dependent DNA helicase DDM1
RS006319	GO:0044699	single-organism process	BP	-1.264071762	-1.378688832	-0.296664377	4.499770473	23.99784591	2.50059E-05	0.000169587	PREDICTED: bumetanide-sensitive sodium-(potassium)-chloride cotransporter
RS015357	GO:0044699	single-organism process	BP	-0.911353669	-1.05463231	-0.283669707	6.234625568	23.92431044	2.59056E-05	0.000175595	PREDICTED: cAMP-specific 3',5'-cyclic phosphodiesterase
RS007454	GO:0044699	single-organism process	BP	-1.467063692	-1.20611441	-0.186463361	3.937246861	23.90841929	2.61042E-05	0.000176751	Glutathione peroxidase 6
RS008934	GO:0044699	single-organism process	BP	-2.249974024	-1.610242152	-0.986553809	2.596021099	23.90344612	2.61667E-05	0.000177078	ionotropic receptor 25a
RS009890	GO:0044699	single-organism process	BP	-0.075711281	-1.041786728	-1.208804803	4.540282181	23.87182517	2.65674E-05	0.000179597	PREDICTED: structural maintenance of chromosomes protein 2
RS010494	GO:0044699	single-organism process	BP	-0.810924227	0.149689075	0.777799549	7.85206277	23.8544144	2.67906E-05	0.000180912	gelsolin
RS010443	GO:0044699	single-organism process	BP	-2.227753564	-2.234532899	-0.56368196	2.617137088	23.82549146	2.71656E-05	0.000183248	PREDICTED: pickpocket protein 28-like
RS002724	GO:0044699	single-organism process	BP	-1.451282908	-0.057440091	-1.790777903	2.779290653	23.81887075	2.72522E-05	0.000183734	PREDICTED: alpha-aminoadipic semialdehyde synthase, mitochondrial
RS008852	GO:0044699	single-organism process	BP	-0.529528854	-1.927991847	-1.523960967	3.134162128	23.80331774	2.74567E-05	0.000185013	PREDICTED: cytochrome P450 6k1-like
RS013682	GO:0044699	single-organism process	BP	-0.527896702	-1.581213796	-0.852078873	3.414021714	23.78876132	2.76494E-05	0.000186212	PREDICTED: regulator of telomere elongation helicase 1 homolog
RS006004	GO:0044699	single-organism process	BP	-1.905451888	-2.638161092	-1.101882392	2.62544082	23.78684346	2.76749E-05	0.000186284	PREDICTED: 1-acyl-sn-glycerol-3-phosphate acyltransferase alpha
RS003119	GO:0044699	single-organism process	BP	1.412801751	0.162091509	-1.555286206	1.807407953	23.76729604	2.79361E-05	0.000187942	PREDICTED: cyclin-dependent kinase 1
RS001128	GO:0044699	single-organism process	BP	1.808591761	2.123019753	-1.973037331	0.645601271	23.62881903	2.98581E-05	0.000200444	PREDICTED: ankyrin repeat and SOCS box protein 10-like
RS015025	GO:0044699	single-organism process	BP	-0.50239966	0.605172425	0.217457472	6.798020919	23.53862414	3.11803E-05	0.000208542	PREDICTED: E3 ubiquitin-protein ligase AMFR-like
RS009217	GO:0044699	single-organism process	BP	-3.054072352	-3.080853801	0.02675188	2.733421312	23.488354	3.19424E-05	0.000213299	Cytochrome P450 4C1
RS002965	GO:0044699	single-organism process	BP	-0.436341634	0.333737355	2.141168519	1.378955234	23.4459247	3.26E-05	0.000217264	PREDICTED: eclosion hormone
RS006732	GO:0044699	single-organism process	BP	0.232254618	-0.514178369	-1.093467207	6.112128041	23.44559627	3.26051E-05	0.000217264	PREDICTED: plexin-B
RS011367	GO:0044699	single-organism process	BP	-0.022151105	-0.99577985	-0.967372584	5.465736233	23.42836442	3.28761E-05	0.000218838	PREDICTED: trifunctional purine biosynthetic protein adenosine-3
RS014152	GO:0044699	single-organism process	BP	-1.430499389	-1.354570713	-0.671668718	4.505777329	23.40073256	3.33153E-05	0.000221644	PREDICTED: potassium channel subfamily K member 1-like
RS002116	GO:0044699	single-organism process	BP	0.917058864	0.016189191	-0.108622566	6.557134635	23.36174257	3.3945E-05	0.000225595	PREDICTED: nuclear pore complex protein Nup93-like
RS011522	GO:0044699	single-organism process	BP	0.688820055	1.111205213	-0.440982183	4.101681825	23.32897813	3.44833E-05	0.00022869	PREDICTED: retinol dehydrogenase 11-like
RS005619	GO:0044699	single-organism process	BP	0.261636641	0.96256037	1.225010795	4.96659594	23.16367482	3.73318E-05	0.000246284	mitochondrial 50S ribosomal protein L53, putative
RS012504	GO:0044699	single-organism process	BP	1.701019285	0.103598753	-0.952358557	2.60795793	23.14266371	3.77102E-05	0.000248475	PREDICTED: zinc transporter ZIP3
RS011207	GO:0044699	single-organism process	BP	0.526909114	0.672637237	1.070322125	5.664986164	23.1408246	3.77435E-05	0.000248475	PREDICTED: group XIIA secretory phospholipase A2
RS007610	GO:0044699	single-organism process	BP	-0.937977299	-0.674413913	0.09856611	5.486082389	23.09307983	3.86186E-05	0.000253578	PREDICTED: short-chain specific acyl-CoA dehydrogenase, mitochondrial
RS005929	GO:0044699	single-organism process	BP	1.193026005	0.720506621	-1.327106608	2.816765242	23.08508406	3.87671E-05	0.00025442	PREDICTED: inactive tyrosine-protein kinase 7-like
RS013918	GO:0044699	single-organism process	BP	0.094481673	0.85169144	0.99774783	5.041999784	23.0537233	3.9355E-05	0.000257876	PREDICTED: tetraspanin-5
RS010502	GO:0044699	single-organism process	BP	-1.112761591	-0.974871229	-1.135223235	4.74695728	23.01510441	4.00913E-05	0.000262427	PREDICTED: glycerol-3-phosphate acyltransferase 3
RS007091	GO:0044699	single-organism process	BP	-0.260344997	-0.000107201	0.982798512	6.405504865	23.00178887	4.03484E-05	0.000263836	PREDICTED: probable multidrug resistance-associated protein lethal(2)03659
RS000970	GO:0044699	single-organism process	BP	-1.826169573	-1.767109952	-3.471752553	2.26650892	22.95978906	4.11699E-05	0.000268372	PREDICTED: alkaline ceramidase
RS014431	GO:0044699	single-organism process	BP	-1.235538497	-1.028714924	-0.243461798	4.612597454	22.93297852	4.1703E-05	0.000271706	PREDICTED: extracellular serine/threonine protein kinase FAM20C
RS009293	GO:0044699	single-organism process	BP	1.071127962	1.229444588	-0.109748778	7.893660582	22.90924169	4.21808E-05	0.000274251	thymosin
RS006486	GO:0044699	single-organism process	BP	-0.152887236	1.834548681	-0.487172522	2.256934592	22.88335857	4.27079E-05	0.000277536	PREDICTED: runt-related transcription factor 3
RS014340	GO:0044699	single-organism process	BP	-0.92887959	-0.092715464	0.219765138	5.620832256	22.82606695	4.38983E-05	0.00028483	PREDICTED: ileal sodium/bile acid cotransporter-like
RS013250	GO:0044699	single-organism process	BP	-1.523650907	-1.743852951	-1.808125659	6.883037152	22.68498455	4.69723E-05	0.000303836	PREDICTED: cytochrome P450 6k1-like
RS004203	GO:0044699	single-organism process	BP	-1.695215415	2.456278506	1.854617147	0.351195749	22.61931625	4.84754E-05	0.000312756	PREDICTED: peroxidase
RS006149	GO:0044699	single-organism process	BP	0.234679756	0.441701686	-0.670485793	5.245280711	22.58573184	4.92625E-05	0.000317509	PREDICTED: endothelin-converting enzyme 1
RS006348	GO:0044699	single-organism process	BP	0.461223391	1.781215986	0.898709787	2.791849317	22.54176148	5.03124E-05	0.000323944	PREDICTED: polypeptide N-acetylgalactosaminyltransferase 2
RS013417	GO:0044699	single-organism process	BP	-0.932938762	-0.802444269	0.119486378	7.142436574	22.52961093	5.06064E-05	0.000325535	PREDICTED: protein sprint
RS010628	GO:0044699	single-organism process	BP	0.154730341	-1.430708521	-0.348593286	6.670494332	22.45057473	5.25612E-05	0.000337216	PREDICTED: phytanoyl-CoA dioxygenase domain-containing protein 1 homolog
RS006066	GO:0044699	single-organism process	BP	-1.68415098	-2.69333348	-1.541267202	1.967459328	22.44424809	5.27209E-05	0.000337897	PREDICTED: D-arabinitol dehydrogenase 1-like
RS007239	GO:0044699	single-organism process	BP	-0.751585402	-1.063608644	-2.049559478	8.039978062	22.36996591	5.46324E-05	0.000349259	PREDICTED: D-3-phosphoglycerate dehydrogenase
RS010687	GO:0044699	single-organism process	BP	0.308688922	-0.324256536	0.937976283	4.1425546	22.35934713	5.49112E-05	0.000350685	PREDICTED: F-box/LRR-repeat protein 20
RS013455	GO:0044699	single-organism process	BP	1.064643482	1.451845871	1.387567298	8.555199834	22.22021108	5.86984E-05	0.000373924	PREDICTED: 60S ribosomal protein L37
RS011507	GO:0044699	single-organism process	BP	0.081672207	0.439418944	1.152509484	5.150989282	22.16598652	6.02438E-05	0.000382801	PREDICTED: sialin
RS000681	GO:0044699	single-organism process	BP	-1.508540822	0.325287212	0.756664918	5.674807397	22.14032278	6.09894E-05	0.000387147	PREDICTED: elongation of very long chain fatty acids protein AAEL008004-like
RS011826	GO:0044699	single-organism process	BP	-1.201418844	-0.474560935	-0.544171933	6.720722124	22.09874094	6.22168E-05	0.000394144	PREDICTED: TBC1 domain family member 1
RS005544	GO:0044699	single-organism process	BP	-0.308180157	0.689248167	-0.738011841	4.466023101	22.07108314	6.30469E-05	0.000399001	PREDICTED: beta-ureidopropionase
RS010514	GO:0044699	single-organism process	BP	0.123856296	0.052206416	-3.120394885	2.960812945	22.02953661	6.43145E-05	0.000406819	PREDICTED: cytochrome P450 307a1
RS007249	GO:0044699	single-organism process	BP	0.571583495	0.957851271	0.170821299	6.396887116	22.0216419	6.45582E-05	0.000408156	PREDICTED: inhibitor of growth protein 5

RS006965	GO:0044699	single-organism process	BP	-0.648544941	-1.308040109	-1.400072153	4.447763652	22.02015753	6.46042E-05	0.000408241	PREDICTED: integrin beta-PS-like
RS005296	GO:0044699	single-organism process	BP	-0.666577041	-0.650920673	0.350048094	4.593415922	21.99923013	6.52552E-05	0.000411529	PREDICTED: protein kinase C, brain isozyme
RS012932	GO:0044699	single-organism process	BP	-1.049335672	-1.635763072	-1.382307762	3.469160191	21.88763407	6.88387E-05	0.000432396	PREDICTED: beta-1,4-mannosyltransferase egh
RS015076	GO:0044699	single-organism process	BP	-1.152386752	-2.00550275	0.22240639	2.013103871	21.86954435	6.94377E-05	0.000435942	PREDICTED: gastrin-releasing peptide receptor-like
RS015008	GO:0044699	single-organism process	BP	-0.623113856	1.344350634	-2.110770817	5.532077098	21.84433607	7.02812E-05	0.000440798	PREDICTED: growth arrest and DNA damage-inducible protein GADD45 alpha
RS009247	GO:0044699	single-organism process	BP	-0.433803981	-2.61636981	-1.684487129	1.770337047	21.79473359	7.19709E-05	0.000450722	PREDICTED: aldose reductase-like
RS003117	GO:0044699	single-organism process	BP	-1.023316483	-1.406983411	-1.703899952	3.289864327	21.79101183	7.20993E-05	0.000451301	PREDICTED: sodium/myo-inositol cotransporter
RS000667	GO:0044699	single-organism process	BP	0.426019576	0.799348926	1.2430101	6.670189859	21.78701685	7.22373E-05	0.000451941	PREDICTED: tubulin-specific chaperone C
RS011862	GO:0044699	single-organism process	BP	1.26876485	0.185394973	-0.470917451	2.593625104	21.77747232	7.25683E-05	0.000453786	PREDICTED: aurora kinase A
RS004760	GO:0044699	single-organism process	BP	0.156406904	-0.487895142	-1.092169147	4.26170621	21.74333442	7.37644E-05	0.00046058	Mitotic checkpoint serine/threonine-protein kinase BUB1
RS005054	GO:0044699	single-organism process	BP	-0.882564902	-1.772840386	-2.09465887	2.914764324	21.56658479	8.02786E-05	0.000498781	PREDICTED: ETS domain-containing protein Elk-3-like
RS001377	GO:0044699	single-organism process	BP	-2.038255092	-0.72878571	1.665144534	0.432779214	21.45218701	8.47971E-05	0.0005253	PREDICTED: gustatory and odorant receptor 24
RS004183	GO:0044699	single-organism process	BP	0.222949707	0.663380198	-0.959888903	8.761852965	21.44964401	8.49003E-05	0.000525681	PREDICTED: 6-phosphofructo-2-kinase/fructose-2,6-bisphosphatase-like
RS010848	GO:0044699	single-organism process	BP	0.497660837	1.496767868	0.106326531	4.247510649	21.44357076	8.51475E-05	0.000526693	PREDICTED: helix-loop-helix protein delilah-like
RS013036	GO:0044699	single-organism process	BP	-1.044771795	-1.760053035	-2.149354476	2.634243274	21.44128572	8.52407E-05	0.000527011	PREDICTED: steroid hormone receptor ERR2
RS006775	GO:0044699	single-organism process	BP	0.962498876	0.838572639	-0.15620984	4.112988908	21.43289788	8.55835E-05	0.000528612	PREDICTED: THO complex subunit 3
RS003830	GO:0044699	single-organism process	BP	-1.002612763	-3.480041102	-5.543788803	0.155703269	21.409331	8.65543E-05	0.000533561	PREDICTED: slit homolog 1 protein
RS000899	GO:0044699	single-organism process	BP	0.969793956	0.241634505	-0.680374371	8.897928901	21.40765273	8.66238E-05	0.000533728	PREDICTED: protein disulfide-isomerase A3
RS000378	GO:0044699	single-organism process	BP	0.790910759	1.677968261	0.802501388	2.754205587	21.34414601	8.92968E-05	0.000549391	PREDICTED: probable G-protein coupled receptor Mth-like 1
RS001132	GO:0044699	single-organism process	BP	1.169061141	0.859228333	0.575845094	7.651899109	21.34274064	8.93568E-05	0.000549492	PREDICTED: mevalonate kinase
RS008848	GO:0044699	single-organism process	BP	1.031712174	0.171089737	-1.324822101	2.881649271	21.33965826	8.94887E-05	0.000550035	PREDICTED: inositol 2-dehydrogenase-like
RS100016	GO:0044699	single-organism process	BP	-2.857073439	-0.80670961	-0.981032078	5.721199604	21.329776	8.99129E-05	0.000552103	PREDICTED: geranylgeranyl pyrophosphate synthase
RS011502	GO:0044699	single-organism process	BP	0.997139085	1.110937602	0.643258028	7.455027006	21.29469842	9.14347E-05	0.000561174	PREDICTED: 3-hydroxyacyl-CoA dehydrogenase type-2
RS015509	GO:0044699	single-organism process	BP	0.906898648	1.035001423	0.502187156	6.057869967	21.29281626	9.15171E-05	0.000561406	PREDICTED: anamorsin
RS003580	GO:0044699	single-organism process	BP	-0.349903781	0.97880878	-0.297171717	4.29862202	21.28103667	9.20343E-05	0.000564029	PREDICTED: uncharacterized aarF domain-containing protein kinase 1
RS004224	GO:0044699	single-organism process	BP	1.136231346	1.228854145	0.864409331	4.792423538	21.28117299	9.20283E-05	0.000564029	PREDICTED: COX assembly mitochondrial protein homolog
RS003788	GO:0044699	single-organism process	BP	-0.001493548	-1.056703896	-0.768443435	5.436926906	21.17659873	9.67495E-05	0.000590913	PREDICTED: probable cytochrome P450 304a1
RS012462	GO:0044699	single-organism process	BP	-1.887439376	-1.068323927	-1.822700455	2.935725927	21.16526065	9.72757E-05	0.000593839	PREDICTED: synaptic vesicle glycoprotein 2B
RS000984	GO:0044699	single-organism process	BP	-0.502231476	-1.761380933	0.042197395	5.64477335	21.16261014	9.73991E-05	0.000594304	PREDICTED: probable cytochrome P450 303a1
RS014088	GO:0044699	single-organism process	BP	-0.848449663	-1.58863489	-0.444766302	5.50367593	21.14381657	9.82786E-05	0.00059909	PREDICTED: multifunctional protein ADE2
RS003042	GO:0044699	single-organism process	BP	0.331617356	2.020868632	0.38642443	5.177405281	21.10261342	0.000100235	0.000609833	AGAP005313-PA
RS009050	GO:0044699	single-organism process	BP	0.063363337	0.694270572	-0.929018582	7.754862813	21.06312261	0.000102146	0.000620561	PREDICTED: MAP kinase-interacting serine/threonine-protein kinase 1
RS008760	GO:0044699	single-organism process	BP	1.198265037	0.816217907	0.515151983	5.821617631	21.01285917	0.000104631	0.000633824	PREDICTED: peroxisome assembly protein 12
RS011901	GO:0044699	single-organism process	BP	-1.015874652	-1.526152304	-0.434414637	3.8222502	21.00653206	0.000104948	0.000635439	PREDICTED: inverted formin-2-like
RS012046	GO:0044699	single-organism process	BP	-0.044612109	1.318321788	-0.182662099	3.873303092	21.0021542	0.000105168	0.000636465	PREDICTED: sodium-independent sulfate anion transporter-like
RS008367	GO:0044699	single-organism process	BP	-1.514192668	-1.058445236	-0.928363315	5.216707665	20.99291854	0.000105633	0.000638975	PREDICTED: vascular endothelial growth factor receptor 1
RS015539	GO:0044699	single-organism process	BP	0.844191092	0.614154003	0.051818114	6.072749755	20.94493919	0.000108085	0.000652863	PREDICTED: NHP2-like protein 1
RS009432	GO:0044699	single-organism process	BP	-1.776105984	-2.872925288	-1.291840277	2.676092105	20.84637639	0.0001133	0.000682399	ace-orthologous acetylcholinesterase
RS015154	GO:0044699	single-organism process	BP	0.77118859	1.275597924	0.795023023	8.092063536	20.820501	0.00011471	0.000690232	PREDICTED: protein transport protein Sec61 subunit gamma
RS008597	GO:0044699	single-organism process	BP	1.561774478	2.596567639	-1.448241182	0.970670851	20.76853675	0.000117595	0.000706241	PREDICTED: insulin-like growth factor I
RS013245	GO:0044699	single-organism process	BP	-0.919192882	-3.212762255	-1.960378095	3.150082705	20.75615985	0.000118292	0.000710093	PREDICTED: acyl-CoA Delta(11) desaturase-like
RS015395	GO:0044699	single-organism process	BP	-1.227128143	-0.999149743	-0.38673491	5.789022217	20.66182653	0.000123747	0.000741733	PREDICTED: ryanodine receptor 44F
RS004481	GO:0044699	single-organism process	BP	1.158258886	1.648956931	2.316008472	2.609786787	20.65713141	0.000124025	0.000742383	PREDICTED: beta-1,3-galactosyltransferase 5
RS000851	GO:0044699	single-organism process	BP	-2.325107834	-2.129776094	-2.544043742	0.519579293	20.5603303	0.000129896	0.000774215	PREDICTED: monocarboxylate transporter 14-like
RS010854	GO:0044699	single-organism process	BP	-0.464601417	0.421962906	0.417617417	6.7210824	20.51691341	0.000132619	0.000789321	PREDICTED: V-type proton ATPase subunit e 2-like
RS010764	GO:0044699	single-organism process	BP	-0.233938613	1.236746291	1.602346303	5.688370295	20.50609795	0.000133306	0.000793035	CG1397-PA
RS006273	GO:0044699	single-organism process	BP	-0.841668922	-0.780206767	0.412687786	4.250127088	20.41178555	0.000139449	0.000826843	PREDICTED: organic cation transporter 1
RS000033	GO:0044699	single-organism process	BP	-0.830072013	-0.118029911	0.444856927	5.272741207	20.40538442	0.000139875	0.000828984	PREDICTED: protein spire
RS013957	GO:0044699	single-organism process	BP	-0.723811943	-0.038931871	0.450923188	7.537654892	20.40186349	0.000140111	0.000829989	PREDICTED: LIM and senescent cell antigen-like-containing domain protein 1
RS014287	GO:0044699	single-organism process	BP	-0.825809928	1.486619083	1.592270521	1.389357496	20.39684624	0.000140447	0.000831501	PREDICTED: sialin
RS010248	GO:0044699	single-organism process	BP	-0.777404788	-0.826975424	0.026640425	7.19452614	20.39608396	0.000140498	0.000831501	PREDICTED: TPPP family protein CG45057
RS007296	GO:0044699	single-organism process	BP	-0.732019662	-1.271421086	-0.560500848	5.629888683	20.38749695	0.000141076	0.000834526	PREDICTED: protein toll
RS004338	GO:0044699	single-organism process	BP	-0.04258427	1.052652401	-0.505496651	4.425105975	20.35193586	0.000143492	0.000847625	PREDICTED: adenylate cyclase type 2-like
RS003734	GO:0044699	single-organism process	BP	0.488070469	0.663359159	2.246155071	1.47414568	20.28161972	0.000148392	0.0008737	Transposable element Tcb1 transposase
RS007340	GO:0044699	single-organism process	BP	1.72289566	2.228109689	-0.488523344	0.853368064	20.24210443	0.000151218	0.000888679	PREDICTED: mitochondrial sodium/hydrogen exchanger 9B2
RS004393	GO:0044699	single-organism process	BP	-1.227790861	-2.264108903	-0.817189459	2.289519889	20.23673107	0.000151606	0.000890546	PREDICTED: putative tricarboxylate transport protein, mitochondrial
RS002393	GO:0044699	single-organism process	BP	-1.699733524	-2.645144912	-0.81293239	1.789894619	20.21821595	0.000152952	0.000897616	PREDICTED: G-protein coupled receptor Mth2-like
RS008430	GO:0044699	single-organism process	BP	-0.063415715	0.3360037	0.984091257	7.0279237	20.19641705	0.000154553	0.000906162	PREDICTED: NADH dehydrogenase [ubiquinone] 1 alpha subcomplex subunit 7-like
RS015388	GO:0044699	single-organism process	BP	-2.602050655	-3.074067005	-0.457367858	0.547921075	20.16611512	0.000156804	0.000918937	PREDICTED: glutamate receptor ionotropic, delta-1
RS000135	GO:0044699	single-organism process	BP	-0.631745801	-0.940404648	0.237026994	4.32458574	20.11793543	0.000160452	0.000937697	PREDICTED: putative ammonium transporter 1
RS006448	GO:0044699	single-organism process	BP	-0.438550285	-0.842818516	-1.111661943	7.029395929	20.1090662	0.000161133	0.000940802	PREDICTED: glycogen phosphorylase
RS005472	GO:0044699	single-organism process	BP	0.842994714	-0.578505121	-0.381283947	4.095980328	20.0834513	0.000163115	0.000951493	PREDICTED: slit homolog 3 protein-like
RS014437	GO:0044699	single-organism process	BP	-0.988357721	-0.015003324	-0.20706366	6.1139878	20.07392409	0.000163859	0.000955386	PREDICTED: alanine--glyoxylate aminotransferase 2-like
RS011787	GO:0044699	single-organism process	BP	-0.438940613	0.619762966	1.077554296	6.634288702	19.98955295	0.000170591	0.000992342	Zinc finger protein 782
RS009040	GO:0044699	single-organism process	BP	-1.66586434	1.595228541	-2.749094748	4.815736325	19.98075033	0.000171309	0.000996059	PREDICTED: F-box/LRR-repeat protein 20
RS001872	GO:0044699	single-organism process	BP	0.988217893	0.341580329	-0.047218689	5.506069821	19.96958697	0.000172224	0.001000916	PREDICTED: histone H2A-like
RS002951	GO:0044699	single-organism process	BP	-0.713484452	-1.159017956	-0.981253249	5.089094554	19.9574752	0.000173222	0.001005326	PREDICTED: dual oxidase 2-like
RS006499	GO:0044699	single-organism process	BP	-1.047637087	0.301924919	0.728056025	3.319116729	19.94751042	0.000174047	0.001009187	PREDICTED: transient receptor potential cation channel subfamily V member 5
RS007172	GO:0044699	single-organism process	BP	-1.06076279	-1.246026476	-1.103197614	4.121058791	19.9312671	0.000175402	0.001016571	Diuretic hormone
RS013197	GO:0044699	single-organism process	BP	-1.613311304	-1.711943527	-0.937859313	5.506175009	19.92371087	0.000176035	0.001019304	PREDICTED: fatty acid hydroxylase domain-containing protein 2
RS014438	GO:0044699	single-organism process	BP	-1.208921125	-1.470936279	-1.254270348	5.660833974	19.92442343	0.000175975	0.001019304	PREDICTED: protein 5NUC-like
RS014669	GO:0044699	single-organism process	BP	1.199508884	0.060809762	-0.59772612	4.68343803	19.91473829	0.00017679	0.001023206	PREDICTED: frizzled-9-like
RS013198	GO:0044699	single-organism process	BP	-0.170174414	-2.032426041	-0.722029511	3.416328583	19.90072697	0.000177976	0.001029596	PREDICTED: fatty acid hydroxylase domain-containing protein 2
RS001856	GO:0044699	single-organism process	BP	-1.518501715	-1.505189008	-1.496793556	1.860974125	19.87755541	0.000179954	0.001040563	PREDICTED: cholecystokinin receptor-like
RS001942	GO:0044699	single-organism process	BP	-1.320906139	-1.706899574	-0.491622586	3.988989165	19.86811055	0.000180767	0.001044303	PREDICTED: beta-arrestin-1
RS005307	GO:0044699	single-organism process	BP	-0.999906753	-0.633406591	-0.150885239	6.796027517	19.86120028	0.000181364	0.001047271	PREDICTED: protoheme IX farnesyltransferase, mitochondrial
RS012429	GO:0044699	single-organism process	BP	0.262891211	0.743274576	1.096842522	7.24219902	19.82285947	0.000184711	0.001065556	PREDICTED: NADH dehydrogenase [ubiquinone] 1 subunit C2
RS002517	GO:0044699	single-organism process	BP	1.387227473	1.312659509	1.21909409	5.707023875	19.80644487	0.000186163	0.001072036	PREDICTED: adenylate kinase isoenzyme 6
RS010799	GO:0044699	single-organism process	BP	1.037559953	-0.640351901	-0.304056164	2.431167164	19.73585517	0.000192538	0.00110506	PREDICTED: DNA replication licensing factor Mcm7
RS015396	GO:0044699	single-organism process	BP	-1.256182982	-1.010805264	-0.293857127	6.048626436	19.73517564	0.0001926	0.00110506	PREDICTED: ryanodine receptor 44F
RS010614	GO:0044699	single-organism process	BP	-3.98675211	-0.987773588	0.464520287	0.726187727	19.72870801	0.000193195	0.001107969	PREDICTED: uricase
RS009017	GO:0044699	single-organism process	BP	-0.045189261	0.043390885	0.916173056	6.536550332	19.702086	0.000195664	0.001120594	PREDICTED: beta-1,3-galactosyltransferase 5-like
RS004622	GO:0044699	single-organism process	BP	-0.068975245	-0.987566547	-0.794611159	5.37593922	19.69257847	0.000196553	0.001124663	PREDICTED: DNA repair protein RAD50
RS008662	GO:0044699	single-organism process	BP	-0.323694319	-0.887991823	-0.04413226	5.702043181	19.66407708	0.000199242	0.001138501	PREDICTED: transient receptor potential cation channel subfamily V member 5
RS001822	GO:0044699	single-organism process	BP	0.374152151	-0.235220993	-0.559194155	6.339069228	19.66077025	0.000199557	0.00113978	PREDICTED: exportin-2
RS008466	GO:0044699	single-organism process	BP	0.258194523	0.713995144	0.861375799	6.29704795	19.55042679	0.000210336	0.001197005	PREDICTED: protein deglycase DJ-1-like
RS007960	GO:0044699	single-organism process	BP	-0.842611461	-1.224378529	-0.873963772	6.473372778	19.54389387	0.000210992	0.001200196	PREDICTED: MAGUK p55 subfamily member 5-A
RS011404	GO:0044699	single-organism process	BP	-0.228168042	-1.092916273	-0.693824639	5.087351847	19.52950106	0.000212445	0.001207368	PREDICTED: serine-protein kinase ATM
RS003207	GO:0044699	single-organism process	BP	-0.800648585	-1.285989549	0.05453666	3.673665732	19.51472965	0.000213946	0.001215351	PREDICTED: cytochrome P450 4c3
RS100005	GO:0044699	single-organism process	BP	-2.639688266	-3.107895143	0.367307699	1.207651888	19.4971158	0.00021575	0.001225045	beta-glucosidase
RS004901	GO:0044699	single-organism process	BP	0.866375197	1.48127266	0.824794906	9.108540307	19.4576958	0.000219842	0.001247717	PREDICTED: staphylococcal nuclease domain-containing protein 1
RS013881	GO:0044699	single-organism process	BP	-0.670719982	-0.118819236	0.483986011	7.673500387	19.41420712	0.000224446	0.001270412	PREDICTED: rho GTPase-activating protein 21
RS002288	GO:0044699	single-organism process	BP	0.867549004	1.162917378	1.440662369	6.537251033	19.38644465	0.000227435	0.001286753	no mechanoreceptor potential A
RS010210	GO:0044699	single-organism process	BP	-0.994802094	1.493392713	-0.157135873	2.117526785	19.31894942	0.000234868	0.001324644	PREDICTED: acyl-CoA Delta(11) desaturase-like
RS006338	GO:0044699	single-organism process	BP	-1.23237985	-1.464800018	-1.246209284	4.869588358	19.31342459	0.000235487	0.001327541	PREDICTED: prohormone-4
RS004443	GO:0044699	single-organism process	BP	0.054717098	0.932950948	0.929941517	4.868833385	19.26483314	0.000241002	0.001356203	PREDICTED: kinesin-like protein KIF2A
RS009896	GO:0044699	single-organism process	BP	-0.657326089	-0.896052656	-0.286680156	5.734157752	19.24633255	0.000243136	0.001365866	PREDICTED: tyrosine-protein phosphatase corkscrew
RS005651	GO:0044699	single-organism process	BP	-0.219646792	-1.283329628	-0.275521678	5.060327004	19.22158667	0.000246019	0.001380116	PREDICTED: transient-receptor-potential-like protein
RS001711	GO:0044699	single-organism process	BP	0.690769604	1.181462709	0.955368065	6.847403504	19.20530543	0.000247934	0.001389623	PREDICTED: probable dolichol-phosphate mannosyltransferase
RS012059	GO:0044699	single-organism process	BP	-0.487537417	-1.155775031	-1.008744238	4.503721781	19.20044033	0.000248509	0.001392227	PREDICTED: serine hydroxymethyltransferase, cytosolic
RS012441	GO:0044699	single-organism process	BP	-0.795629624	0.983276815	1.634723389	1.23435714	19.18509754	0.000250332	0.001401285	AGAP004474
RS006140	GO:0044699	single-organism process	BP	0.033292599	1.273516036	1.00013418	2.720807394	19.17927372	0.000251027	0.00140446	PREDICTED: potassium channel subfamily K member 18
RS014754	GO:0044699	single-organism process	BP	-0.658876088	-1.058888095	-0.553090263	5.968197725	19.17453226	0.000251595	0.001406386	PREDICTED: aldose 1-epimerase-like
RS003313	GO:0044699	single-organism process	BP	-0.809695085	-1.073895206	-0.821194513	5.250458407	19.16975752	0.000252168	0.001408963	PREDICTED: calcium-independent phospholipase A2-gamma-like
RS012729	GO:0044699	single-organism process	BP	0.67341266	0.986455945	1.0660316	6.776322119	19.11539228	0.000258782	0.001442719	PREDICTED: disheveled-associated activator of morphogenesis 1
RS013204	GO:0044699	single-organism process	BP	0.2252278	-1.626669386	-2.139314176	1.777992775	19.06163958	0.000265492	0.001478816	PREDICTED: G1/S-specific cyclin-D2
RS008851	GO:0044699	single-organism process	BP	-1.026874425	-2.686216909	-1.27484501	1.194799866	19.0588559	0.000265844	0.001479298	PREDICTED: cytochrome P450 6k1-like
RS002503	GO:0044699	single-organism process	BP	-0.009517009	0.124352347	0.864548115	5.245674668	19.05817022	0.000265931	0.001479298	PREDICTED: sugar phosphate exchanger 2
RS008907	GO:0044699	single-organism process	BP	-1.38488	-0.062498976	0.498893705	4.635174182	19.05931997	0.000265785	0.001479298	PREDICTED: metabotropic glutamate receptor 3-like
RS005838	GO:0044699	single-organism process	BP	-1.272361428	-3.114064861	-3.237130599	0.429125999	19.0443607	0.000267685	0.001488399	PREDICTED: glutamate receptor U1-like
RS003726	GO:0044699	single-organism process	BP	-0.35022698	0.054528944	0.659374478	4.976150075	18.98906357	0.000274826	0.001524592	PREDICTED: mitochondrial coenzyme A transporter SLC25A42

RS000152	GO:0044699	single-organism process	BP	-1.205241827	-0.522965397	-0.166378556	6.69155609	18.98450384	0.000275423	0.001526708	PREDICTED: synaptic vesicle glycoprotein 2B-like
RS001336	GO:0044699	single-organism process	BP	0.412169289	1.116247727	1.639275177	2.741362032	18.96980298	0.000277358	0.001536754	PREDICTED: glutamate receptor U1-like
RS007614	GO:0044699	single-organism process	BP	0.699165628	-0.052782673	-0.817546641	5.472681929	18.94140065	0.000281133	0.001554478	PREDICTED: facilitated trehalose transporter Tret1-like
RS015408	GO:0044699	single-organism process	BP	-0.990750885	-0.815651047	-0.405889031	7.467064085	18.907004	0.000285774	0.001576452	PREDICTED: sodium/potassium-transporting ATPase subunit beta-2-like
RS007875	GO:0044699	single-organism process	BP	0.743606352	-0.046039924	0.105888728	5.909432159	18.82016803	0.000297832	0.001640095	PREDICTED: splicing factor 3B subunit 6-like protein
RS004267	GO:0044699	single-organism process	BP	0.923435776	0.461949323	0.046778356	5.790570644	18.81684727	0.000298303	0.001641971	PREDICTED: tubulin epsilon chain-like
RS003954	GO:0044699	single-organism process	BP	-4.25472229	-2.186202621	-0.625435753	0.514613298	18.80035159	0.000300654	0.001654188	PREDICTED: guanine nucleotide-binding protein subunit beta-2
RS001158	GO:0044699	single-organism process	BP	0.872931495	-0.210046718	-2.199477253	1.873855939	18.78473436	0.000302897	0.001665304	PREDICTED: fork head domain-containing protein FD4-like
RS013988	GO:0044699	single-organism process	BP	-0.868304934	-0.581730433	1.156036538	1.862819607	18.77711082	0.000303997	0.001670394	PREDICTED: echinoderm microtubule-associated protein-like CG42247
RS003226	GO:0044699	single-organism process	BP	-3.288280783	-2.544414567	-1.03339834	0.925036246	18.76617374	0.000305584	0.001676915	PREDICTED: putative nuclease HARBI1
RS008344	GO:0044699	single-organism process	BP	0.997945072	0.536467708	0.242324791	5.73621232	18.75619131	0.000307039	0.001684165	PREDICTED: small nuclear ribonucleoprotein Sm D1
RS009401	GO:0044699	single-organism process	BP	0.854447285	0.929289648	0.445031622	5.521211063	18.75438305	0.000307303	0.001684881	PREDICTED: trafficking protein particle complex subunit 6B
RS011715	GO:0044699	single-organism process	BP	-1.02776739	-1.798297028	-1.766096501	7.764040057	18.73944327	0.000309495	0.001695424	PREDICTED: glucose dehydrogenase [FAD, quinone]-like
RS007694	GO:0044699	single-organism process	BP	-1.402205119	-1.429007484	-0.154037672	3.235518975	18.72862507	0.000311092	0.001703432	PREDICTED: proton myo-inositol cotransporter-like
RS009272	GO:0044699	single-organism process	BP	-0.152985688	-1.205701524	-0.53386679	4.590610282	18.71946093	0.000312451	0.001710132	PREDICTED: mitochondrial sodium/hydrogen exchanger 9B2
RS005897	GO:0044699	single-organism process	BP	1.150102697	-1.510175908	-1.635972119	0.550138227	18.70930417	0.000313965	0.001717669	PREDICTED: tethering factor for nuclear proteasome sts1
RS011525	GO:0044699	single-organism process	BP	-0.806138244	0.427082827	0.274818707	7.810153084	18.69931455	0.000315461	0.001724354	PREDICTED: sodium/potassium-transporting ATPase subunit beta-2
RS009874	GO:0044699	single-organism process	BP	0.701368874	1.02507259	0.9119133	5.08095509	18.69168808	0.000316607	0.001729871	PREDICTED: spermidine synthase
RS000605	GO:0044699	single-organism process	BP	0.99400035	0.382921961	-2.550989273	0.932876977	18.6495758	0.000323014	0.001763345	PREDICTED: aurora kinase B
RS013136	GO:0044699	single-organism process	BP	-0.851364149	-0.302892822	0.222344744	5.914157083	18.57902045	0.000334038	0.001818795	PREDICTED: sialin
RS006581	GO:0044699	single-organism process	BP	-0.306542325	-0.987050981	-0.658423051	5.75989163	18.52006556	0.000343536	0.001864867	PREDICTED: copper chaperone for superoxide dismutase
RS002509	GO:0044699	single-organism process	BP	0.07347544	-0.720252922	-0.547625565	6.183077943	18.51719795	0.000344005	0.001866607	PREDICTED: poly [ADP-ribose] polymerase
RS009604	GO:0044699	single-organism process	BP	-1.648345631	0.550771769	0.526514629	2.368500525	18.41833332	0.000360561	0.001945804	PREDICTED: sodium-dependent noradrenaline transporter
RS012270	GO:0044699	single-organism process	BP	-0.182394127	0.327985779	0.811767902	6.544185044	18.41805613	0.000360609	0.001945804	PREDICTED: histone-lysine N-methyltransferase setd3
RS000466	GO:0044699	single-organism process	BP	-2.166029677	-1.851606374	-0.236502645	3.196981426	18.39313728	0.000364906	0.001967306	cytochrome P450
RS014713	GO:0044699	single-organism process	BP	0.018067912	0.424164392	-1.237214228	3.948162684	18.38267981	0.000366725	0.001976264	Secreted frizzled-related protein 5
RS003219	GO:0044699	single-organism process	BP	0.030734368	-1.119228443	-0.51621698	3.887749858	18.36336163	0.000370108	0.001991939	PREDICTED: probable peroxisomal acyl-coenzyme A oxidase 1
RS002546	GO:0044699	single-organism process	BP	-1.996319911	-0.628990503	-0.486595829	4.86185156	18.31880565	0.000378029	0.002025916	PREDICTED: scavenger receptor class B member 1
RS010411	GO:0044699	single-organism process	BP	-0.654350162	0.196745787	0.465032285	7.059348208	18.31448963	0.000378806	0.002028947	PREDICTED: 3-phosphoinositide-dependent protein kinase 1
RS001941	GO:0044699	single-organism process	BP	0.892160393	1.088657994	1.159229799	5.534219954	18.31177893	0.000379294	0.002030102	PREDICTED: methionine-R-sulfoxide reductase B1
RS013158	GO:0044699	single-organism process	BP	-1.989910388	0.460189662	-0.420554799	7.049048028	18.30634638	0.000380275	0.002034486	PREDICTED: putative fatty acyl-CoA reductase CG5065
RS012721	GO:0044699	single-organism process	BP	-0.370726844	-1.082307086	-1.179144733	3.763910521	18.2954932	0.000382241	0.002044138	phosphoenolpyruvate phosphomutase
RS007466	GO:0044699	single-organism process	BP	1.218797087	2.13669878	1.837909061	1.863580807	18.26800425	0.000387268	0.002068383	PREDICTED: ras-related and estrogen-regulated growth inhibitor-like
RS010080	GO:0044699	single-organism process	BP	-1.225591648	-1.424043287	-0.670731436	9.443830356	18.26507346	0.000387807	0.002070388	PREDICTED: 2-oxoglutarate dehydrogenase, mitochondrial
RS012501	GO:0044699	single-organism process	BP	-1.250093155	-1.758275813	-0.217147236	3.060515632	18.24989505	0.000390615	0.002083609	PREDICTED: collagen and calcium-binding EGF domain-containing protein 1
RS010007	GO:0044699	single-organism process	BP	-0.719830127	-2.070294597	-0.711910489	2.61204502	18.21562653	0.000397027	0.002115128	PREDICTED: monocarboxylate transporter 9-like
RS005291	GO:0044699	single-organism process	BP	-0.384191963	-0.901778096	-0.889974383	6.001215612	18.17315177	0.000405121	0.002155512	PREDICTED: DNA ligase 1
RS013893	GO:0044699	single-organism process	BP	0.865893227	0.613314033	1.324344129	4.789113349	18.12489455	0.000414516	0.002203638	PREDICTED: G-protein coupled receptor 161-like
RS008541	GO:0044699	single-organism process	BP	0.555640398	0.958575568	0.469787621	6.558218358	18.11924045	0.000415631	0.002208632	PREDICTED: alpha-tubulin N-acetyltransferase-like
RS007772	GO:0044699	single-organism process	BP	1.194382491	0.710383458	0.569322256	5.741877059	18.10577673	0.000418298	0.002219993	PREDICTED: DNA-directed RNA polymerase III subunit RPC7-like
RS013623	GO:0044699	single-organism process	BP	-0.572979083	-1.747015153	-0.730223745	3.430703168	18.10154502	0.00041914	0.002223523	PREDICTED: neo-calmodulin-like
RS009304	GO:0044699	single-organism process	BP	-2.056162023	-1.492683824	-1.148437663	1.440266038	18.0307206	0.000433479	0.002294761	PREDICTED: glutamate receptor ionotropic, delta-1-like
RS010564	GO:0044699	single-organism process	BP	-0.595765531	-0.950640759	-0.006337065	5.273966828	17.9050916	0.000460125	0.002423594	ACYPI003318
RS000897	GO:0044699	single-organism process	BP	1.16874833	1.097549551	0.978511526	6.230404895	17.90546659	0.000460043	0.002423594	PREDICTED: adenylate kinase isoenzyme 6
RS003346	GO:0044699	single-organism process	BP	0.185204858	-0.316496604	0.908686897	3.535712188	17.89446056	0.000462453	0.002434839	PREDICTED: DNA ligase 4
RS002555	GO:0044699	single-organism process	BP	-1.140121066	-0.73879484	-0.227892694	7.987636916	17.89107673	0.000463197	0.002437735	PREDICTED: ankyrin-2-like
RS007227	GO:0044699	single-organism process	BP	1.175219171	0.8252835	0.752372621	5.540119208	17.88973221	0.000463492	0.002438272	PREDICTED: uroporphyrinogen-III synthase
RS013246	GO:0044699	single-organism process	BP	-1.373852886	-4.090748051	-2.164995168	2.336943256	17.88036325	0.000465559	0.002448119	PREDICTED: acyl-CoA Delta(11) desaturase-like
RS014036	GO:0044699	single-organism process	BP	0.055154792	-0.971344742	-0.534644837	5.913555109	17.87876759	0.000465911	0.002448952	Neurotrypsin
RS014410	GO:0044699	single-organism process	BP	-0.538555444	-0.069436224	0.424307658	5.574699261	17.86202393	0.00046963	0.002466436	PREDICTED: sodium- and chloride-dependent neutral and basic amino acid transporter B(0+)
RS000837	GO:0044699	single-organism process	BP	-1.260815808	-0.582381283	0.014148923	5.012164404	17.85146996	0.000471988	0.002476758	PREDICTED: excitatory amino acid transporter 1-like
RS012415	GO:0044699	single-organism process	BP	-1.109242119	-1.5975963	-0.338883168	3.45173542	17.81063049	0.000481227	0.002523137	PREDICTED: zinc finger protein PLAG1
RS000805	GO:0044699	single-organism process	BP	-0.741332789	-1.773884811	-0.626522213	4.210559745	17.80066494	0.000483509	0.002534045	PREDICTED: protein SERAC1
RS010652	GO:0044699	single-organism process	BP	0.706911894	0.574603078	-0.126390712	5.931654177	17.75520496	0.000494054	0.002587159	PREDICTED: CTP synthase
RS001961	GO:0044699	single-organism process	BP	-1.00080434	1.41660757	1.066880848	2.170829105	17.74147664	0.000497283	0.002601244	carboxylesterase
RS012231	GO:0044699	single-organism process	BP	0.305167569	0.871014785	0.884514802	6.802223658	17.7132479	0.00050399	0.002632619	PREDICTED: probable 39S ribosomal protein L49, mitochondrial
RS003685	GO:0044699	single-organism process	BP	-0.539796958	-0.98347808	-0.875859761	5.981260133	17.64846278	0.000519722	0.002709182	PREDICTED: dynein heavy chain 5, axonemal
RS000327	GO:0044699	single-organism process	BP	-0.00726001	-1.146145516	-1.664085652	4.090173459	17.63943013	0.000521954	0.002719691	PREDICTED: farnesyl pyrophosphate synthase
RS008650	GO:0044699	single-organism process	BP	-0.150761137	0.057954786	0.953488041	3.599213119	17.62923206	0.000524486	0.002731751	PREDICTED: POC1 centriolar protein homolog
RS009810	GO:0044699	single-organism process	BP	-1.393669765	-0.799726174	-0.408687427	4.462416695	17.62535716	0.000525451	0.002735646	PREDICTED: 1-phosphatidylinositol 4,5-bisphosphate phosphodiesterase delta-4
RS010313	GO:0044699	single-organism process	BP	-1.001125903	-4.868885875	1.067576862	0.36061951	17.62041212	0.000526685	0.002739806	PREDICTED: protein maelstrom homolog
RS009828	GO:0044699	single-organism process	BP	-0.293669849	0.251880322	0.656532524	6.962358331	17.59297378	0.000533585	0.002771122	PREDICTED: puromycin-sensitive aminopeptidase
RS006223	GO:0044699	single-organism process	BP	-0.457154045	0.525936273	0.222262374	5.569768578	17.50973148	0.000555074	0.002876172	allatotropin
RS013806	GO:0044699	single-organism process	BP	1.125065024	0.762651662	0.536660145	6.030701282	17.50931715	0.000555183	0.002876172	PREDICTED: elongation factor G, mitochondrial
RS014365	GO:0044699	single-organism process	BP	0.85655384	0.755877469	0.728396016	5.752752663	17.48862594	0.000560657	0.002903339	PREDICTED: thioredoxin domain-containing protein 17-like
RS012271	GO:0044699	single-organism process	BP	-0.281748742	0.071249927	1.279091697	3.12916002	17.47213454	0.000565059	0.002923729	PREDICTED: ras-related protein Rab-32-like
RS013819	GO:0044699	single-organism process	BP	-1.107033055	-2.944700776	-1.549867549	2.010059666	17.46942746	0.000565785	0.002926282	PREDICTED: urocanate hydratase
RS003799	GO:0044699	single-organism process	BP	-0.143761693	0.372761559	-0.639546518	5.875204513	17.46636866	0.000566606	0.002928125	PREDICTED: ATP-binding cassette sub-family C member Sur
RS014628	GO:0044699	single-organism process	BP	-0.742367417	-0.611313702	0.354118192	3.683582866	17.44515899	0.000572334	0.002954086	PREDICTED: calcium/calmodulin-dependent protein kinase kinase 1-like
RS013814	GO:0044699	single-organism process	BP	-0.210474819	0.719689803	0.248188508	5.412785882	17.42219412	0.0005786	0.002977885	PREDICTED: protein kinase DC2
RS013961	GO:0044699	single-organism process	BP	0.36854056	0.734046356	-0.074989852	5.464061023	17.39152223	0.000587075	0.003019038	PREDICTED: golgin-45
RS005416	GO:0044699	single-organism process	BP	-0.412284439	-1.050899627	-2.077424575	2.957561508	17.37798325	0.000590855	0.003035999	PREDICTED: threo-3-hydroxyaspartate ammonia-lyase
RS004930	GO:0044699	single-organism process	BP	-0.497639986	-0.980023186	-0.354132401	5.164789843	17.37464784	0.00059179	0.003039563	PREDICTED: phosphatidylinositol 3-kinase regulatory subunit alpha
RS000435	GO:0044699	single-organism process	BP	0.515479598	0.900602724	0.726672321	5.243663251	17.3682661	0.000593584	0.00304753	PREDICTED: 39S ribosomal protein L13, mitochondrial
RS008703	GO:0044699	single-organism process	BP	0.891413178	0.410697322	-0.681554837	9.477370831	17.33816384	0.000602115	0.003082533	PREDICTED: calreticulin
RS009509	GO:0044699	single-organism process	BP	0.806427471	0.776544181	1.154394405	5.747456563	17.33967998	0.000601682	0.003082533	DNA-directed RNA polymerase III subunit RPC9
RS003705	GO:0044699	single-organism process	BP	0.057476585	-0.874131429	-1.326214826	5.440373792	17.33177411	0.000603941	0.003090627	PREDICTED: alpha-L-fucosidase-like
RS013208	GO:0044699	single-organism process	BP	0.299641009	0.680206066	0.926035838	6.944401511	17.31297112	0.000609348	0.003115764	PREDICTED: glutaredoxin-3
RS011383	GO:0044699	single-organism process	BP	-0.592908391	-0.975532691	-0.419225628	5.386382038	17.30604756	0.000611351	0.003123469	PREDICTED: 2-hydroxyacyl-CoA lyase 1
RS010863	GO:0044699	single-organism process	BP	1.056959359	1.019389795	1.044382134	5.466013138	17.28355255	0.000617904	0.00315439	PREDICTED: mimitin, mitochondrial
RS007085	GO:0044699	single-organism process	BP	-2.123520843	-1.442400071	-1.654436852	1.159522622	17.26753428	0.000622612	0.00317714	PREDICTED: cyclic nucleotide-gated cation channel alpha-3
RS007435	GO:0044699	single-organism process	BP	-1.357174472	-1.765931713	-0.506725895	4.089208263	17.26317136	0.000623901	0.003182427	PREDICTED: A disintegrin and metalloproteinase with thrombospondin motifs 3-like
RS008837	GO:0044699	single-organism process	BP	1.048782795	1.201451837	0.639944589	5.270709436	17.25285317	0.000626959	0.003196732	PREDICTED: translation initiation factor IF-2, mitochondrial
RS010509	GO:0044699	single-organism process	BP	-1.527063033	-1.135110705	-1.09559939	2.940762741	17.22393729	0.00063561	0.003239526	PREDICTED: cysteine sulfinic acid decarboxylase
RS013191	GO:0044699	single-organism process	BP	0.105641406	-0.473422065	-1.100786682	4.568604849	17.21352583	0.000638753	0.003251735	PREDICTED: low-density lipoprotein receptor-related protein 4
RS011194	GO:0044699	single-organism process	BP	-0.043178438	0.101493976	0.864859407	6.406781775	17.21343829	0.00063878	0.003251735	PREDICTED: mitochondrial pyruvate carrier 2-like
RS012692	GO:0044699	single-organism process	BP	-0.691907497	-0.984121611	-0.240522391	4.80822272	17.19367051	0.000644791	0.003278361	PREDICTED: serine/threonine-protein phosphatase 2A 56 kDa regulatory subunit gamma isoform-like
RS006294	GO:0044699	single-organism process	BP	0.715429068	0.199968336	-0.917251363	3.513742435	17.1175034	0.000668484	0.003394712	PREDICTED: protein deadpan-like
RS011797	GO:0044699	single-organism process	BP	0.90598073	0.776839649	0.523519079	6.985288917	17.09418962	0.000675907	0.003426883	PREDICTED: tyrosine--tRNA ligase, cytoplasmic
RS015400	GO:0044699	single-organism process	BP	-2.223152999	-3.76726411	-1.749155038	1.545557081	17.08779982	0.000677956	0.003435888	PREDICTED: fatty acyl-CoA reductase 1-like
RS000849	GO:0044699	single-organism process	BP	-0.753175709	-1.571391853	-1.058453667	2.708513766	17.06922787	0.000683946	0.003464852	PREDICTED: monocarboxylate transporter 9-like
RS012432	GO:0044699	single-organism process	BP	-1.318193047	-1.41640057	-1.093486391	3.821570882	17.02394884	0.000698772	0.003535692	PREDICTED: acetylcholine receptor subunit alpha-type acr-16-like
RS007219	GO:0044699	single-organism process	BP	-0.802609438	-5.215789315	-2.442780211	0.047839307	17.01391627	0.0007021	0.003551104	PREDICTED: transcription factor LBX1-like
RS013332	GO:0044699	single-organism process	BP	-1.915754341	-0.761067399	-0.092972296	1.96508851	16.98236489	0.000712669	0.003598779	PREDICTED: potassium channel subfamily T member 2
RS004774	GO:0044699	single-organism process	BP	0.866820361	0.213840863	-0.023111661	7.683781356	16.95671717	0.000721377	0.003638374	PREDICTED: 26S protease regulatory subunit 10B
RS004044	GO:0044699	single-organism process	BP	-0.216859326	0.637026253	1.004892576	2.871056733	16.9549321	0.000721987	0.003638536	PREDICTED: glutamate-gated chloride channel
RS009749	GO:0044699	single-organism process	BP	-0.44682439	-0.87081526	-0.341573949	6.643967653	16.95006253	0.000723653	0.003645477	PREDICTED: tetraspanin-1
RS001926	GO:0044699	single-organism process	BP	0.91116243	1.13513027	1.076018868	8.148556654	16.94137237	0.000726637	0.003659044	PREDICTED: 40S ribosomal protein S28
RS009694	GO:0044699	single-organism process	BP	0.63774292	0.924020801	0.928262356	7.139015542	16.9030198	0.000739952	0.003724602	PREDICTED: serine--tRNA ligase, cytoplasmic
RS000809	GO:0044699	single-organism process	BP	-0.701195951	0.021692306	0.322683644	4.895816175	16.88899392	0.000744881	0.003747917	PREDICTED: inositol-trisphosphate 3-kinase A
RS012541	GO:0044699	single-organism process	BP	-0.984280487	-1.389163223	-0.815862587	2.880809307	16.87130659	0.000751144	0.003776412	PREDICTED: nucleolysin TIAR
RS003877	GO:0044699	single-organism process	BP	-0.65387437	0.297873351	0.698651676	5.601194508	16.86557951	0.000753184	0.003785153	PREDICTED: SET and MYND domain-containing protein 4
RS007073	GO:0044699	single-organism process	BP	-0.603049329	-0.874713843	-0.994791182	5.376371075	16.7683237	0.000788663	0.003950846	PREDICTED: major facilitator superfamily domain-containing protein 6
RS002706	GO:0044699	single-organism process	BP	-0.740169025	-1.766156812	-2.037640472	11.03901016	16.70844226	0.00081133	0.004054717	PREDICTED: apolipophorins
RS011660	GO:0044699	single-organism process	BP	-0.230746972	-1.178905272	-0.378126279	5.905869902	16.64895332	0.000834488	0.004162195	PREDICTED: ribonuclease UK114
RS008042	GO:0044699	single-organism process	BP	0.748039054	0.869303872	0.524727487	7.223922118	16.64134071	0.000837499	0.004173905	PREDICTED: threonine--tRNA ligase, cytoplasmic
RS000041	GO:0044699	single-organism process	BP	-0.970092086	-1.799060718	-1.441059447	4.392482413	16.56903986	0.000866634	0.004310581	PREDICTED: inositol oxygenase
RS012880	GO:0044699	single-organism process	BP	-0.30658397	-1.009628453	-0.362263592	6.906985464	16.5529187	0.000873267	0.004340143	PREDICTED: C-1-tetrahydrofolate synthase, cytoplasmic
RS010586	GO:0044699	single-organism process	BP	0.623952368	-0.256309112	-0.51417404	5.593140423	16.55051363	0.000874261	0.004343368	PREDICTED: lon protease homolog 2, peroxisomal
RS001085	GO:0044699	single-organism process	BP	0.380680709	-0.189722762	-0.809714628	5.874285554	16.53535798	0.000880549	0.004369438	PREDICTED: endoplasmic reticulum resident protein 29
RS012047	GO:0044699	single-organism process	BP	-1.033691566	-1.204387105	-0.573210977	3.717195265	16.53395031	0.000881135	0.004370625	PREDICTED: sodium-independent sulfate anion transporter-like

RS008487	GO:0044699	single-organism process	BP	-1.659056586	0.950292602	0.71211177	2.293134942	16.52586712	0.00088451	0.004385636	PREDICTED: probable nuclear hormone receptor HR38
RS015168	GO:0044699	single-organism process	BP	0.488237482	0.26526474	-0.417654449	6.233148413	16.52067499	0.000886684	0.004394687	PREDICTED: prolyl 4-hydroxylase subunit alpha-2
RS001615	GO:0044699	single-organism process	BP	0.36152593	-0.39751857	-1.048113601	3.908433802	16.49288964	0.00089841	0.004449304	PREDICTED: E3 ubiquitin-protein ligase RAD18-like
RS003885	GO:0044699	single-organism process	BP	-0.60905754	-0.139328874	0.430103655	5.306744699	16.46129051	0.000911933	0.004503878	PREDICTED: amyloid beta A4 precursor protein-binding family B member 1-interacting protein
RS010364	GO:0044699	single-organism process	BP	-0.052944401	0.405459181	0.815688442	7.091949691	16.43172938	0.000924767	0.004565472	PREDICTED: cytochrome c oxidase subunit 7A2, mitochondrial
RS008835	GO:0044699	single-organism process	BP	0.714093156	1.017689251	0.694563384	4.883435711	16.42237232	0.000928867	0.004583914	PREDICTED: signal recognition particle 14 kDa protein
RS003625	GO:0044699	single-organism process	BP	-0.919867298	-0.750704213	-0.704835111	6.933958906	16.40972231	0.000934438	0.004607796	PREDICTED: synaptotagmin 1
RS002855	GO:0044699	single-organism process	BP	-1.74839573	-1.535258414	-0.16366084	1.79539144	16.39328726	0.000941725	0.00463647	PREDICTED: glycine receptor subunit alpha-3
RS001900	GO:0044699	single-organism process	BP	0.920032231	0.681311079	0.164965465	7.432319705	16.37741042	0.000948819	0.004665093	PREDICTED: ATP-dependent RNA helicase p62-like
RS014196	GO:0044699	single-organism process	BP	0.27402419	-0.508228049	-0.515526922	5.019419054	16.37592758	0.000949484	0.00466555	PREDICTED: protoporphyrinogen oxidase
RS000927	GO:0044699	single-organism process	BP	-2.570527298	-2.804707147	-0.755163456	0.667619183	16.35029216	0.000961058	0.004715063	PREDICTED: b(0,+)-type amino acid transporter 1-like
RS009136	GO:0044699	single-organism process	BP	0.453201531	1.18574391	0.527049129	5.684115152	16.33142348	0.000969667	0.004753593	PREDICTED: glutamate receptor-interacting protein 1
RS010910	GO:0044699	single-organism process	BP	-0.748105138	0.071569831	0.008155293	6.575547514	16.32656533	0.000971895	0.004760813	PREDICTED: serine/threonine-protein kinase tricorner
RS000323	GO:0044699	single-organism process	BP	0.958578604	1.001886888	0.695262009	6.065570937	16.30861249	0.000980176	0.004797642	PREDICTED: 39S ribosomal protein L50, mitochondrial
RS009094	GO:0044699	single-organism process	BP	0.810509918	0.868482563	0.707370764	6.315762678	16.22341499	0.001020437	0.004986955	PREDICTED: mitochondrial import inner membrane translocase subunit Tim17-B-like
RS010632	GO:0044699	single-organism process	BP	-0.601981369	-2.724628152	-1.346468504	1.085526658	16.2209042	0.001021648	0.004989001	PREDICTED: E3 ubiquitin-protein ligase SIAH1-like
RS007611	GO:0044699	single-organism process	BP	0.85433042	0.661811427	0.970900853	6.298492276	16.20186994	0.001030876	0.005030163	Cytochrome b-c1 complex subunit 2, mitochondrial
RS015045	GO:0044699	single-organism process	BP	-0.779817217	0.120556874	0.367497615	7.201359537	16.19804265	0.001032741	0.005037313	PREDICTED: putative ferric-chelate reductase 1 homolog
RS012234	GO:0044699	single-organism process	BP	-0.237721619	0.668719697	0.406400015	6.348460931	16.1630065	0.001049975	0.005115428	PREDICTED: dihydropteridine reductase
RS009524	GO:0044699	single-organism process	BP	-0.581068435	-0.703536129	0.80952874	2.472581079	16.15868974	0.001052118	0.005123886	PREDICTED: tyrosine-protein phosphatase non-receptor type 5-like
RS011739	GO:0044699	single-organism process	BP	0.480441824	0.81604698	0.958356963	6.198337685	16.13176967	0.00106558	0.005181434	PREDICTED: 39S ribosomal protein L44, mitochondrial
RS006224	GO:0044699	single-organism process	BP	0.458860561	0.806125659	-0.013738201	7.161415157	16.10048245	0.001081441	0.005248428	PREDICTED: ubiquitin carboxyl-terminal hydrolase 10
RS011005	GO:0044699	single-organism process	BP	-0.555589131	-2.624022106	1.401997531	0.450600041	16.09549582	0.001083991	0.005258775	PREDICTED: histamine H2 receptor
RS012395	GO:0044699	single-organism process	BP	-0.33294168	0.344699786	0.795487985	7.263451467	16.0796385	0.001092138	0.005292185	PREDICTED: reticulon-4-interacting protein 1 homolog, mitochondrial-like
RS015162	GO:0044699	single-organism process	BP	0.636728789	0.736805617	-0.009824935	6.699510264	16.07671339	0.001093648	0.005297461	PREDICTED: fibroblast growth factor receptor homolog 1-like
RS003418	GO:0044699	single-organism process	BP	-0.329155165	1.093289904	1.059155473	3.11674268	16.06532959	0.001099542	0.005321919	PREDICTED: phosrestin-2-like
RS015297	GO:0044699	single-organism process	BP	-1.015455816	-2.24509318	-0.470758517	1.387764422	16.04189955	0.001111773	0.0053708	PREDICTED: frizzled-2-like
RS002936	GO:0044699	single-organism process	BP	-0.1501232	0.247765204	0.8156572	6.076227226	16.02267088	0.001121912	0.00541355	PREDICTED: arrestin domain-containing protein 3
RS008099	GO:0044699	single-organism process	BP	0.427050523	-0.25028056	-0.602969715	5.831752321	16.02158313	0.001122489	0.005414256	PREDICTED: centrosomal protein of 135 kDa
RS003544	GO:0044699	single-organism process	BP	-0.410771227	-0.348918681	1.010423069	3.516674428	16.0029682	0.001132396	0.005455777	PREDICTED: glutamate receptor ionotropic, kainate 2
RS010726	GO:0044699	single-organism process	BP	-0.948746358	-0.647876443	0.359072765	3.48816878	15.9874998	0.001140696	0.00549366	PREDICTED: activin receptor type-2A
RS006141	GO:0044699	single-organism process	BP	0.186642093	0.509454722	0.888482209	5.536921472	15.98603197	0.001141486	0.005494665	Potassium channel subfamily K member 9
RS008762	GO:0044699	single-organism process	BP	-0.443958619	-1.307107122	-1.617489214	6.243657005	15.97477402	0.001147568	0.005518319	PREDICTED: phenoloxidase 2-like
RS015474	GO:0044699	single-organism process	BP	-0.709288445	-0.784579568	-1.561754476	5.780194154	15.96582735	0.001152425	0.005537444	PREDICTED: mucin-5AC
RS003914	GO:0044699	single-organism process	BP	-0.408304954	-1.308021582	-1.113950825	3.842131166	15.96402321	0.001153407	0.005540047	PREDICTED: facilitated trehalose transporter Tret1-like
RS002082	GO:0044699	single-organism process	BP	0.782834803	0.605864374	0.18619874	6.629641607	15.93394391	0.001169899	0.005608566	PREDICTED: small nuclear ribonucleoprotein-associated protein B
RS002338	GO:0044699	single-organism process	BP	0.516863935	0.450314897	-0.275417004	6.799668193	15.91557113	0.001180087	0.005653106	PREDICTED: rab proteins geranylgeranyltransferase component A 1
RS001319	GO:0044699	single-organism process	BP	1.511864955	1.284108306	2.990762134	0.453423176	15.91081499	0.001182739	0.005663655	PREDICTED: prostaglandin E2 receptor EP3 subtype
RS012240	GO:0044699	single-organism process	BP	0.589927687	0.240581316	-0.821227102	3.702761451	15.88875944	0.001195113	0.005716391	PREDICTED: bipolar kinesin KRP-130-like
RS008709	GO:0044699	single-organism process	BP	-0.858383544	-1.607869787	-1.185848102	5.60680269	15.87116741	0.001205076	0.005757482	PREDICTED: glycine dehydrogenase (decarboxylating), mitochondrial
RS013870	GO:0044699	single-organism process	BP	-1.185159015	-0.789079424	-0.942978819	4.024142004	15.86729932	0.001207277	0.005765813	PREDICTED: sodium- and chloride-dependent glycine transporter 1-like
RS013164	GO:0044699	single-organism process	BP	-0.720894807	-1.573394185	-0.567112816	2.35012097	15.85215695	0.001215934	0.005800558	PREDICTED: sodium/hydrogen exchanger 3
RS004133	GO:0044699	single-organism process	BP	-1.405737616	-1.5407592	-0.871825746	8.438126432	15.84692631	0.001218939	0.00581269	superoxide dismutase
RS014330	GO:0044699	single-organism process	BP	-0.904307343	-1.006861252	-0.448014081	6.451669659	15.83661629	0.001224883	0.005834407	PREDICTED: NADPH--cytochrome P450 reductase
RS001333	GO:0044699	single-organism process	BP	-0.39244885	-1.244052895	0.568596523	4.759499994	15.83188612	0.001227619	0.005843621	PREDICTED: kinesin-like protein KIF19
RS011887	GO:0044699	single-organism process	BP	0.896958966	0.96953015	0.804343524	6.976362895	15.81322065	0.001238478	0.005883586	PREDICTED: probable cardiolipin synthase (CMP-forming)
RS007657	GO:0044699	single-organism process	BP	0.118922712	-1.465382499	-0.981922701	2.419012684	15.79975793	0.001246369	0.00591884	PREDICTED: aurora kinase B
RS014761	GO:0044699	single-organism process	BP	0.850386459	0.827647699	0.741742978	6.600879234	15.79201097	0.001250932	0.005936034	PREDICTED: mitochondrial import receptor subunit TOM40 homolog 1
RS010670	GO:0044699	single-organism process	BP	0.11586169	0.434196754	0.975472626	6.943668226	15.76519389	0.001266857	0.006007076	PREDICTED: NADH dehydrogenase [ubiquinone] 1 beta subcomplex subunit 3
RS012655	GO:0044699	single-organism process	BP	-0.287961251	-0.117441863	0.618178853	7.425528178	15.76413742	0.001267488	0.006007809	PREDICTED: protein phosphatase Slingshot
RS015083	GO:0044699	single-organism process	BP	-1.353576966	-1.726252304	-1.030363606	3.43648112	15.75786481	0.001271244	0.006018815	PREDICTED: G-protein coupled receptor moody
RS007887	GO:0044699	single-organism process	BP	0.740875807	0.982728981	0.368061586	5.30134597	15.7441554	0.001279491	0.006053309	PREDICTED: mitochondrial thiamine pyrophosphate carrier-like
RS014120	GO:0044699	single-organism process	BP	-0.922827123	-1.289252361	-1.238323899	2.633512953	15.71916541	0.001294661	0.006118183	PREDICTED: nicotinamidase
RS010935	GO:0044699	single-organism process	BP	-0.955349572	-0.110515396	0.342411356	6.328877639	15.71272059	0.001298602	0.006129907	PREDICTED: integrin-linked protein kinase
RS012448	GO:0044699	single-organism process	BP	0.432921331	0.502551129	1.044792421	6.158806875	15.70768354	0.00130169	0.006140931	PREDICTED: 39S ribosomal protein L23, mitochondrial
RS002871	GO:0044699	single-organism process	BP	-0.296751524	0.736699117	-1.141900235	3.342833192	15.70483332	0.001303441	0.00614584	cytochrome P450
RS012306	GO:0044699	single-organism process	BP	-1.171605666	-1.08070317	-0.099574832	5.667246046	15.66500563	0.001328153	0.006252994	PREDICTED: ABC transporter G family member 23
RS014405	GO:0044699	single-organism process	BP	0.958360321	1.312682736	-0.124130207	2.991400315	15.64637713	0.001339871	0.006301227	PREDICTED: vang-like protein 2
RS012739	GO:0044699	single-organism process	BP	-0.719207681	-0.968714942	-0.43280243	4.857531521	15.60802319	0.00136432	0.006404116	PREDICTED: ribosomal protein S6 kinase alpha-3
RS015210	GO:0044699	single-organism process	BP	0.885212806	0.92739167	0.921146058	7.166370556	15.59681265	0.001371549	0.006429811	PREDICTED: protein lin-52 homolog
RS012806	GO:0044699	single-organism process	BP	-0.671548237	-0.771532863	-0.831977584	6.320380877	15.5512679	0.001401314	0.006555774	PREDICTED: glutamate [NMDA] receptor subunit 1
RS012955	GO:0044699	single-organism process	BP	-0.728677213	0.000354407	1.251744202	10.76578598	15.54939983	0.001402548	0.006558257	PREDICTED: flightin
RS014795	GO:0044699	single-organism process	BP	-0.083269799	0.255358346	0.921540584	7.700274461	15.54888907	0.001402886	0.006558257	PREDICTED: NADH dehydrogenase [ubiquinone] 1 beta subcomplex subunit 8, mitochondrial
RS004534	GO:0044699	single-organism process	BP	-0.580382142	2.035273319	0.497901415	1.025113205	15.51468926	0.001425682	0.006654944	PREDICTED: sodium channel protein Nach-like
RS001884	GO:0044699	single-organism process	BP	-0.95805652	-1.257839612	-1.050763923	4.518398722	15.51303432	0.001426794	0.006657669	PREDICTED: protein croquemort-like
RS008500	GO:0044699	single-organism process	BP	0.002272108	1.869228123	0.257768405	1.255041308	15.51205513	0.001427453	0.006658275	PREDICTED: patched domain-containing protein 3
RS007810	GO:0044699	single-organism process	BP	-0.820216184	-1.018356117	-0.958242629	3.620004773	15.51103249	0.001428141	0.006659019	PREDICTED: vesicular glutamate transporter 1
RS003951	GO:0044699	single-organism process	BP	-0.089340755	0.817298999	-0.331077746	5.263967312	15.4731895	0.001453838	0.006768814	PREDICTED: procollagen-lysine,2-oxoglutarate 5-dioxygenase 3
RS009281	GO:0044699	single-organism process	BP	0.3749826	-0.436310081	-0.776142013	4.026435371	15.4687186	0.001456905	0.006778078	PREDICTED: probable serine/threonine-protein kinase mps1
RS000189	GO:0044699	single-organism process	BP	-0.489811484	-1.010756521	-0.978147615	5.972836071	15.4661139	0.001458694	0.006781392	PREDICTED: cytochrome b5
RS008144	GO:0044699	single-organism process	BP	0.604388754	0.174208176	-0.195414694	6.220683848	15.46317209	0.001460717	0.006785789	PREDICTED: cyclin-dependent kinase 10
RS008562	GO:0044699	single-organism process	BP	-0.534931045	0.469487333	-0.516456474	4.343438394	15.44564485	0.001472831	0.006831982	PREDICTED: cGMP-specific 3',5'-cyclic phosphodiesterase
RS002756	GO:0044699	single-organism process	BP	-0.963241844	-1.229404023	-1.218821516	7.34033313	15.41968201	0.001490958	0.00691352	PREDICTED: kinesin-like protein unc-104
RS011298	GO:0044699	single-organism process	BP	-1.012083432	0.044059061	0.235404354	8.917200046	15.41459917	0.001494532	0.006927545	PREDICTED: myophilin
RS012386	GO:0044699	single-organism process	BP	-1.113494458	-1.408132111	-1.035003478	4.276380866	15.3721214	0.00152474	0.007059771	PREDICTED: calbindin-32
RS001401	GO:0044699	single-organism process	BP	-0.75955343	-0.918377897	-0.01602954	4.379386426	15.3535465	0.001538139	0.007115841	PREDICTED: isocitrate dehydrogenase [NADP] cytoplasmic-like
RS013810	GO:0044699	single-organism process	BP	-0.907513315	-1.338803536	-1.386188117	1.974248304	15.35298665	0.001538545	0.007115841	PREDICTED: calpain-5
RS006403	GO:0044699	single-organism process	BP	-0.681016731	0.542395772	-0.474618093	4.101875379	15.33937628	0.00154844	0.007158975	PREDICTED: G-protein coupled receptor Mth2-like
RS006044	GO:0044699	single-organism process	BP	-0.676228211	-0.513648981	0.462911231	3.973647365	15.33152104	0.001554179	0.007182874	PREDICTED: allantoinase, mitochondrial-like
RS007742	GO:0044699	single-organism process	BP	0.316811125	0.69266755	-0.130137718	6.055518966	15.32081631	0.001562034	0.00721653	PREDICTED: ras-related GTP-binding protein A
RS002204	GO:0044699	single-organism process	BP	-1.650105815	-1.734472246	-1.621814081	2.284635926	15.3188973	0.001563447	0.007220407	PREDICTED: cell adhesion molecule 3-like
RS000010	GO:0044699	single-organism process	BP	0.426923599	-0.624982642	-0.893462554	2.701038655	15.2949068	0.001581211	0.007297239	PREDICTED: cell division control protein 6 homolog
RS000928	GO:0044699	single-organism process	BP	-2.818387407	-2.387215456	-1.195316578	0.324906115	15.29049888	0.001584497	0.007309578	B(0,+)-type amino acid transporter 1
RS006610	GO:0044699	single-organism process	BP	0.2664942	0.92285009	0.741592285	5.324734931	15.2782684	0.001593649	0.007342643	PREDICTED: zinc transporter 7
RS013812	GO:0044699	single-organism process	BP	-0.90352517	-0.658147253	-0.537176094	4.464856555	15.24382038	0.001619709	0.007447467	PREDICTED: propionyl-CoA carboxylase beta chain, mitochondrial
RS011250	GO:0044699	single-organism process	BP	-1.135486465	-1.214258358	-1.040330075	3.222301555	15.1532963	0.001690224	0.007743426	eater
RS001241	GO:0044699	single-organism process	BP	-1.170912786	-2.106369837	-0.733700322	11.51564159	15.14249671	0.001698837	0.007780056	PREDICTED: arginine kinase
RS005593	GO:0044699	single-organism process	BP	0.627280874	-0.724664401	-0.502373046	3.104326898	15.1371369	0.001703128	0.007794039	PREDICTED: kinesin-like protein KIF18A
RS008896	GO:0044699	single-organism process	BP	-1.274733506	-1.006034401	-0.767037953	2.62762816	15.13459455	0.001705167	0.007799012	PREDICTED: mitoferrin-1
RS007163	GO:0044699	single-organism process	BP	-0.713121743	-1.68663287	-0.132093276	4.212153829	15.12739922	0.001710951	0.00781814	PREDICTED: adenosine deaminase
RS012755	GO:0044699	single-organism process	BP	0.293553114	-0.126828854	-0.885519174	8.465049801	15.11046803	0.001724639	0.007869602	PREDICTED: lysosomal aspartic protease
RS007779	GO:0044699	single-organism process	BP	0.463802087	-0.235076553	0.817903268	4.17313915	15.09027569	0.001741105	0.00793324	PREDICTED: microtubule-associated proteins 1A/1B light chain 3C
RS000178	GO:0044699	single-organism process	BP	-0.901112574	-1.878487348	-1.266039758	3.225951619	15.07720821	0.001751844	0.0079764	PREDICTED: argininosuccinate synthase
RS011193	GO:0044699	single-organism process	BP	0.23153241	-0.655301507	0.610210215	6.072153027	15.07588643	0.001752934	0.007978478	PREDICTED: organic cation transporter protein
RS000495	GO:0044699	single-organism process	BP	-0.516925659	-0.37793687	-1.159432206	3.991777882	15.07108362	0.0017569	0.007987868	acyltransferase
RS000210	GO:0044699	single-organism process	BP	-0.955252781	-5.253337838	-2.469969213	0.028530126	15.06419366	0.001762605	0.008010914	cytochrome P450
RS009127	GO:0044699	single-organism process	BP	0.427832283	0.765474122	0.889802594	6.51609746	15.05604992	0.001769372	0.008038768	PREDICTED: mitotic-spindle organizing protein 1-like
RS002291	GO:0044699	single-organism process	BP	-0.050285067	1.11315863	0.096165399	5.093284164	15.05231582	0.001772483	0.00805	PREDICTED: cat eye syndrome critical region protein 5 homolog
RS006022	GO:0044699	single-organism process	BP	-1.813369421	-1.750081698	-0.062538905	2.776091628	15.04967814	0.001774684	0.008054187	PREDICTED: beta-ureidopropionase
RS008827	GO:0044699	single-organism process	BP	0.074981221	-0.72918138	-0.860942434	4.364697528	15.04602812	0.001777734	0.008065124	PREDICTED: ribonucleoside-diphosphate reductase subunit M2
RS006136	GO:0044699	single-organism process	BP	-2.199659373	0.052075633	2.322759219	0.896680658	15.0374978	0.001784883	0.008094642	alpha-amylase
RS004916	GO:0044699	single-organism process	BP	-1.150368436	-0.282872283	-1.326860021	2.723099194	15.0312974	0.001790098	0.008115367	PREDICTED: dual specificity protein phosphatase CDC14A-like
RS006197	GO:0044699	single-organism process	BP	-1.725611249	-5.831665088	-0.587837998	0.462052021	15.01327909	0.001805336	0.008178562	PREDICTED: maltase 1-like
RS006757	GO:0044699	single-organism process	BP	-0.233100515	0.411940029	0.749443033	4.26814335	14.98825356	0.001826715	0.008260555	PREDICTED: cell death-inducing p53-target protein 1
RS014418	GO:0044699	single-organism process	BP	-0.478113679	-0.820374615	0.805474325	2.345331841	14.9793906	0.001834346	0.008283168	PREDICTED: alpha-1,6-mannosyl-glycoprotein 2-beta-N-acetylglucosaminyltransferase
RS007861	GO:0044699	single-organism process	BP	-0.580634106	-2.782707671	-3.148613229	1.366193435	14.96245395	0.001849017	0.008340446	juvenile hormone acid methyltransferase
RS010696	GO:0044699	single-organism process	BP	-0.661039084	-1.417412032	-0.955228318	4.894679825	14.94772627	0.00186187	0.008389405	PREDICTED: fumarylacetoacetase
RS001628	GO:0044699	single-organism process	BP	-0.066987655	0.88088736	0.583161669	4.399335423	14.93361565	0.001874267	0.008442244	PREDICTED: apoptosis-inducing factor 3
RS013391	GO:0044699	single-organism process	BP	0.240838892	-0.336953256	-0.607480362	5.48514497	14.91761643	0.001888422	0.008496887	PREDICTED: intraflagellar transport protein 81 homolog
RS009126	GO:0044699	single-organism process	BP	0.004334845	0.550905396	0.691970165	7.068681097	14.88906784	0.001913944	0.008599435	PREDICTED: V-type proton ATPase subunit F
RS009530	GO:0044699	single-organism process	BP	-0.92327297	-0.184426165	0.206235076	3.842166244	14.88499805	0.001917611	0.008612834	PREDICTED: 43 kDa receptor-associated protein of the synapse homolog

RS001426	GO:0044699	single-organism process	BP	-0.051094175	0.393849454	1.27557231	2.251827973	14.88393125	0.001918573	0.008614083	PREDICTED: solute carrier organic anion transporter family member 5A1
RS001526	GO:0044699	single-organism process	BP	-0.188683928	0.519017421	0.650345185	7.644232128	14.87803596	0.001923898	0.008634916	PREDICTED: V-type proton ATPase subunit G
RS009254	GO:0044699	single-organism process	BP	0.276763099	0.307712871	0.901515863	7.284745885	14.831862	0.00196612	0.008811857	PREDICTED: histidine triad nucleotide-binding protein 1
RS000313	GO:0044699	single-organism process	BP	-0.954310763	-3.153266548	-0.807609981	1.098288284	14.81595689	0.001980876	0.008868522	PREDICTED: 3 beta-hydroxysteroid dehydrogenase/Delta 5-->4-isomerase type 1
RS000883	GO:0044699	single-organism process	BP	0.721822534	0.660596012	0.315198713	6.086831525	14.79749299	0.001998143	0.00892914	PREDICTED: ras-related protein Rab-8A
RS006318	GO:0044699	single-organism process	BP	-3.618949223	-2.702749807	-1.072386578	0.467850565	14.73641507	0.002056334	0.009147816	PREDICTED: bumetanide-sensitive sodium-(potassium)-chloride cotransporter
RS011547	GO:0044699	single-organism process	BP	-0.263001265	-0.778562912	-0.771649351	6.075833911	14.71604685	0.00207611	0.009222762	PREDICTED: ribose-phosphate pyrophosphokinase 1
RS011711	GO:0044699	single-organism process	BP	-0.932783616	-1.108902154	-0.464136879	4.215366662	14.7138405	0.002078264	0.009229074	PREDICTED: flotillin-2
RS011405	GO:0044699	single-organism process	BP	-1.520391219	-1.806294063	-1.56720976	8.916277625	14.69755053	0.002094233	0.00929016	PREDICTED: inositol-3-phosphate synthase 1-B
RS010102	GO:0044699	single-organism process	BP	0.602034968	-0.002372206	-0.350450464	5.052828537	14.65995786	0.00213155	0.009432446	proliferating cell nuclear antigen
RS008426	GO:0044699	single-organism process	BP	0.21033902	-0.068768175	-0.671070305	5.440837237	14.65031422	0.002141229	0.009468622	PREDICTED: tubulin gamma-2 chain
RS009443	GO:0044699	single-organism process	BP	-1.459569756	-2.612200948	-0.494425769	0.982671322	14.62163541	0.002170271	0.009583587	seven in absentia 1A
RS005708	GO:0044699	single-organism process	BP	-2.57707626	-0.767810406	-0.121337824	1.208165742	14.61680361	0.002175202	0.009598631	PREDICTED: sodium- and chloride-dependent glycine transporter 1-like
RS000651	GO:0044699	single-organism process	BP	-1.237192962	-0.764493307	0.341738456	10.32717692	14.61687954	0.002175124	0.009598631	PREDICTED: nesprin-1
RS002096	GO:0044699	single-organism process	BP	0.464617661	0.653577558	0.843133376	7.017229304	14.60721112	0.002185025	0.009631853	PREDICTED: lactoylglutathione lyase
RS008575	GO:0044699	single-organism process	BP	-0.895887835	-1.094241053	-0.498680763	6.153628076	14.59558766	0.002196986	0.009674422	PREDICTED: probable aspartate aminotransferase, cytoplasmic
RS013194	GO:0044699	single-organism process	BP	-0.402235939	1.468418978	0.620702468	1.800456379	14.57025873	0.002223278	0.009779937	PREDICTED: gamma-aminobutyric acid receptor alpha-like
RS008446	GO:0044699	single-organism process	BP	0.363470434	-0.27703378	-1.408383647	4.990252537	14.55183725	0.002242595	0.009858024	PREDICTED: facilitated trehalose transporter Tret1-2 homolog
RS009719	GO:0044699	single-organism process	BP	0.563582355	0.295013848	0.853666183	5.794037182	14.50872049	0.002288461	0.010045616	PREDICTED: coiled-coil domain-containing protein 25
RS005115	GO:0044699	single-organism process	BP	1.546667092	0.997186707	-1.046806406	1.090183288	14.49382648	0.002304519	0.010109063	PREDICTED: enhancer of split mbeta protein-like
RS005493	GO:0044699	single-organism process	BP	0.723695416	0.886760965	1.006140641	5.151193943	14.46545589	0.002335419	0.010230352	PREDICTED: dehydrogenase/reductase SDR family member 11-like
RS008491	GO:0044699	single-organism process	BP	-0.83498967	-1.675590127	-1.132486269	2.878684988	14.45091871	0.00235141	0.010286093	PREDICTED: callisulfakinin
RS012689	GO:0044699	single-organism process	BP	-0.335162446	-0.922302748	-1.067051914	4.164281375	14.43562454	0.002368352	0.010353914	PREDICTED: solute carrier family 41 member 1-like
RS003961	GO:0044699	single-organism process	BP	-0.750984742	-0.876045305	-0.479254703	5.20742512	14.41029185	0.00239668	0.010462317	PREDICTED: sterol regulatory element-binding protein cleavage-activating protein
RS011611	GO:0044699	single-organism process	BP	-0.479731095	-0.109387378	0.576801966	5.275549716	14.40716498	0.0024002	0.010470424	PREDICTED: ras-related protein Rac1
RS004810	GO:0044699	single-organism process	BP	-0.563533832	-0.496909413	0.504144261	5.97356833	14.40436631	0.002403354	0.010480555	Rho GTPase-activating protein 6
RS012514	GO:0044699	single-organism process	BP	-0.809786811	-1.025425535	-1.196386573	6.957494555	14.3933263	0.002415839	0.010524833	PREDICTED: microtubule-associated protein futsch
RS015058	GO:0044699	single-organism process	BP	0.697002167	0.949496893	0.739628343	7.396841716	14.39407361	0.002414992	0.010524833	PREDICTED: eukaryotic translation initiation factor 3 subunit J
RS003513	GO:0044699	single-organism process	BP	-0.999789437	-1.703665788	-1.124485665	2.357938576	14.39084011	0.002418659	0.010532709	PREDICTED: retinol dehydrogenase 14
RS013687	GO:0044699	single-organism process	BP	-0.15225672	-2.860275429	-1.652752871	2.658895556	14.35252769	0.002462536	0.010708972	PREDICTED: acyl-CoA Delta(11) desaturase-like
RS015477	GO:0044699	single-organism process	BP	-1.101064815	-0.393684254	-0.930550173	3.969326348	14.3061796	0.002516672	0.010910488	PREDICTED: pyruvate carboxylase, mitochondrial
RS009849	GO:0044699	single-organism process	BP	-1.123044544	-0.698755878	0.301998954	2.897014168	14.28861394	0.002537496	0.010989417	PREDICTED: mothers against decapentaplegic homolog 6
RS001558	GO:0044699	single-organism process	BP	-0.498500357	0.339634653	0.275416805	6.573968032	14.2819524	0.002545438	0.011020022	PREDICTED: clustered mitochondria protein homolog
RS013787	GO:0044699	single-organism process	BP	0.379837104	-0.516409715	0.818329231	4.525575001	14.2636528	0.002567381	0.011088341	PREDICTED: probable cytochrome P450 305a1
RS009859	GO:0044699	single-organism process	BP	-0.988585761	-1.845941859	-1.665851706	3.480828702	14.26090821	0.002570689	0.011098818	PREDICTED: ganglioside GM2 activator
RS008723	GO:0044699	single-organism process	BP	0.765724899	0.492155106	-0.726953396	2.604372289	14.25219643	0.002581214	0.011140444	PREDICTED: excitatory amino acid transporter
RS006137	GO:0044699	single-organism process	BP	-1.750611457	-0.496084864	1.146600014	1.717250447	14.23346103	0.002603996	0.011227225	alpha-amylase
RS015559	GO:0044699	single-organism process	BP	-2.383612554	-2.605044757	-1.26251376	0.481679586	14.22941496	0.002608942	0.0112447	PREDICTED: probable beta-hexosaminidase fdl
RS004493	GO:0044699	single-organism process	BP	0.747582847	0.822851494	0.673066996	7.064713526	14.22392986	0.002615662	0.011265952	PREDICTED: eukaryotic translation initiation factor 2 subunit 2
RS014548	GO:0044699	single-organism process	BP	-0.284980468	-1.173909226	-1.493304567	2.859916837	14.21947648	0.00262113	0.011285646	PREDICTED: MFS-type transporter SLC18B1-like
RS007565	GO:0044699	single-organism process	BP	-0.276040168	0.053025641	0.824680101	7.619542531	14.20253495	0.002642037	0.011367617	PREDICTED: NADH dehydrogenase [ubiquinone] 1 beta subcomplex subunit 6
RS005190	GO:0044699	single-organism process	BP	-0.561761317	0.20318587	0.563166122	6.506172496	14.19948267	0.002645822	0.011376401	PREDICTED: calcium-activated potassium channel slowpoke
RS013212	GO:0044699	single-organism process	BP	-0.878244991	-0.110123051	0.018139925	7.487946573	14.16068921	0.002694389	0.011553674	PREDICTED: V-type proton ATPase subunit B
RS014977	GO:0044699	single-organism process	BP	0.405657427	-0.54233367	-0.425006151	5.3256547	14.11996649	0.002746323	0.011752162	PREDICTED: gamma-butyrobetaine dioxygenase
RS015220	GO:0044699	single-organism process	BP	-1.725263737	-0.823509933	-0.447260682	3.097046424	14.11927767	0.00274721	0.011752162	PREDICTED: cytochrome P450 6k1-like
RS011379	GO:0044699	single-organism process	BP	-0.945895165	-0.137739549	0.02413553	6.197611827	14.11352967	0.002754622	0.01177587	PREDICTED: chloride intracellular channel exc-4
RS007462	GO:0044699	single-organism process	BP	0.36030726	-0.423018692	-0.296632439	5.494309337	14.09396548	0.00278	0.011872269	PREDICTED: charged multivesicular body protein 2b-B
RS011435	GO:0044699	single-organism process	BP	-0.366557147	-0.19145709	-1.542817591	2.894938423	14.09263118	0.002781739	0.011875669	PREDICTED: guanine nucleotide-binding protein subunit beta-2-like 1
RS001584	GO:0044699	single-organism process	BP	-1.279974575	-1.282037826	-0.766338036	5.895975843	14.07137958	0.002809585	0.011982364	PREDICTED: superoxide dismutase [Cu-Zn], chloroplastic-like
RS000121	GO:0044699	single-organism process	BP	-0.019315387	0.635582011	0.716261319	4.467640052	14.06831177	0.002813628	0.011991483	PREDICTED: WAS/WASL-interacting protein family member 2
RS010917	GO:0044699	single-organism process	BP	0.542714268	0.449972149	-0.281829843	5.363560163	14.0470616	0.002841789	0.01208696	PREDICTED: RNA/RNP complex-1-interacting phosphatase
RS011122	GO:0044699	single-organism process	BP	0.082189494	1.196958457	0.74482172	3.346133987	14.0443711	0.002845374	0.012090866	PREDICTED: phospholipase A2
RS009329	GO:0044699	single-organism process	BP	-0.948520503	0.072910629	0.92431105	1.718714513	14.02878534	0.002866232	0.012170371	PREDICTED: ras-related protein Rab-7L1
RS000944	GO:0044699	single-organism process	BP	-1.104302196	-0.930660283	-0.256032322	7.618878836	14.02469536	0.00287173	0.012185502	PREDICTED: glutamate dehydrogenase, mitochondrial
RS010551	GO:0044699	single-organism process	BP	-0.821090597	-0.543834668	-0.366492633	5.842626515	14.01366646	0.002886609	0.01223627	PREDICTED: nicotinate phosphoribosyltransferase
RS008574	GO:0044699	single-organism process	BP	-1.398204707	-1.439633479	-0.772632276	3.648197129	13.9848864	0.002925797	0.012394044	PREDICTED: probable aspartate aminotransferase, cytoplasmic
RS003160	GO:0044699	single-organism process	BP	-2.458757465	-3.448259619	0.224465111	6.176120812	13.98050671	0.002931807	0.012415325	PREDICTED: probable cytochrome P450 6a14
RS013441	GO:0044699	single-organism process	BP	-1.118538724	-1.066918701	-0.417843975	2.984566996	13.96108545	0.002958604	0.012516175	PREDICTED: ras-related protein Rab-37
RS008584	GO:0044699	single-organism process	BP	1.571981097	0.220870531	-0.598786915	1.2790483	13.94920659	0.002975113	0.01258179	PREDICTED: thymidylate synthase
RS003220	GO:0044699	single-organism process	BP	-0.919595261	-1.193174547	-0.639458968	5.160771178	13.94596359	0.002979636	0.012596688	PREDICTED: probable peroxisomal acyl-coenzyme A oxidase 1
RS015500	GO:0044699	single-organism process	BP	-1.105288316	-1.790627726	-0.834079521	2.387317289	13.93598546	0.002993595	0.012636758	PREDICTED: fatty acyl-CoA reductase 1-like
RS004154	GO:0044699	single-organism process	BP	-1.099636409	-0.574925083	-0.313075483	5.604418011	13.91327386	0.00302561	0.012748256	PREDICTED: trehalase
RS011854	GO:0044699	single-organism process	BP	-1.112002956	0.070911347	-0.516191421	6.87646831	13.85956875	0.003102671	0.013038052	PREDICTED: aldehyde dehydrogenase, dimeric NADP-preferring
RS010970	GO:0044699	single-organism process	BP	-0.654166537	0.492469445	-0.220477149	4.881203789	13.84299924	0.003126837	0.013126461	PREDICTED: alanine aminotransferase 1
RS014046	GO:0044699	single-organism process	BP	-0.827652517	0.123541367	0.39500677	4.371003694	13.78924417	0.003206526	0.0134386	PREDICTED: zinc transporter ZIP1-like
RS000957	GO:0044699	single-organism process	BP	-2.010012831	-0.338712626	2.309453527	0.190518781	13.7331006	0.003291905	0.013768931	PREDICTED: lipase 3-like
RS011402	GO:0044699	single-organism process	BP	-0.518766755	-1.355522075	-0.785081892	3.459941294	13.73057373	0.0032958	0.013776073	PREDICTED: glyoxylate reductase/hydroxypyruvate reductase-like
RS004446	GO:0044699	single-organism process	BP	-0.453642352	-1.168928872	-1.463577764	5.14345374	13.7113448	0.003325592	0.01388677	cytochrome P450
RS000848	GO:0044699	single-organism process	BP	-0.491509622	-0.039197542	0.539051459	7.220480495	13.7028662	0.003338812	0.013932736	PREDICTED: NADH dehydrogenase [ubiquinone] 1 alpha subcomplex subunit 9, mitochondrial
RS012390	GO:0044699	single-organism process	BP	-0.716673568	-0.893126341	-0.183509175	7.550045761	13.67242105	0.003386715	0.014118599	phosphoglucose isomerase
RS004592	GO:0044699	single-organism process	BP	-0.294046429	0.156068735	0.564893198	6.831343324	13.651508	0.003420014	0.014243271	PREDICTED: ATP-binding cassette sub-family B member 7, mitochondrial
RS008428	GO:0044699	single-organism process	BP	0.726073094	0.817785022	0.602141689	6.883790517	13.64572037	0.003429286	0.014277167	PREDICTED: signal peptidase complex subunit 1
RS009681	GO:0044699	single-organism process	BP	-0.687137615	-0.85904435	-0.276874723	5.270172907	13.64338362	0.003433037	0.014288059	PREDICTED: peroxisomal membrane protein PMP34
RS010082	GO:0044699	single-organism process	BP	0.977742003	1.040191834	0.66275259	8.591648811	13.59639781	0.003509324	0.014576658	PREDICTED: nucleoside diphosphate kinase
RS013109	GO:0044699	single-organism process	BP	0.631944911	0.678140132	0.924971577	5.281011599	13.58711883	0.003524587	0.014635228	PREDICTED: endonuclease G, mitochondrial
RS014215	GO:0044699	single-organism process	BP	0.558889204	0.180769849	-0.366265028	6.244068247	13.56345187	0.003563815	0.014772207	PREDICTED: alcohol dehydrogenase class-3
RS012490	GO:0044699	single-organism process	BP	-0.915529552	-0.48670375	0.180774422	7.265494326	13.56297283	0.003564613	0.014772207	PREDICTED: dihydrolipoyllysine-residue succinyltransferase component of 2-oxoglutarate dehydrogenase complex, mitochondrial
RS009429	GO:0044699	single-organism process	BP	-0.617325589	-1.392127645	-0.307783204	4.922754521	13.52755287	0.003624144	0.014974562	PREDICTED: very-long-chain enoyl-CoA reductase
RS010366	GO:0044699	single-organism process	BP	0.382759498	0.630321986	0.894464068	7.0334464	13.5075993	0.003658112	0.015105005	PREDICTED: thioredoxin, mitochondrial
RS006255	GO:0044699	single-organism process	BP	-0.565848028	-0.409978879	0.214379631	6.839035823	13.48918712	0.003689736	0.015225603	PREDICTED: copper-transporting ATPase 1
RS001967	GO:0044699	single-organism process	BP	-1.292199525	-4.907642283	0.13038607	0.114212979	13.45666724	0.003746254	0.015433537	carboxylesterase
RS012010	GO:0044699	single-organism process	BP	-0.658055692	-0.163236513	0.25753802	6.662027841	13.45566221	0.003748014	0.01543574	PREDICTED: sodium-dependent phosphate transporter 1-B-like
RS008477	GO:0044699	single-organism process	BP	-0.3330518	-0.89397836	-0.27696967	6.646440173	13.43354958	0.003786952	0.015585815	PREDICTED: iodotyrosine dehalogenase 1
RS006304	GO:0044699	single-organism process	BP	0.422940736	0.353105918	-0.357159997	6.653898423	13.40015991	0.003846507	0.015799678	PREDICTED: retinol dehydrogenase 13-like
RS012776	GO:0044699	single-organism process	BP	0.789690393	1.167067408	1.229484622	9.213801709	13.39739167	0.003851486	0.015810174	40S ribosomal protein S13
RS012241	GO:0044699	single-organism process	BP	0.040785999	-0.886212197	-0.897512931	3.97496532	13.38709279	0.003870066	0.015876093	PREDICTED: phosphatidate phosphatase PPAPDC1B
RS010731	GO:0044699	single-organism process	BP	-0.395182325	-0.639006133	-0.675231861	6.20607259	13.34648856	0.003944186	0.016153848	PREDICTED: V-type proton ATPase 116 kDa subunit a
RS006343	GO:0044699	single-organism process	BP	0.700005517	0.678494058	0.385858572	5.661988764	13.33113205	0.003972583	0.016264862	PREDICTED: zinc transporter 6
RS011208	GO:0044699	single-organism process	BP	-0.482430328	-0.966724395	-0.684724818	4.789360952	13.30902134	0.004013826	0.016423044	PREDICTED: centrosomal protein of 290 kDa
RS005589	GO:0044699	single-organism process	BP	-0.596440903	0.149886416	0.534975348	6.378552287	13.30055833	0.004029724	0.016466693	PREDICTED: beta-parvin
RS001644	GO:0044699	single-organism process	BP	0.156342426	-0.571907409	-0.712089954	4.678494016	13.28665341	0.004055981	0.016552501	PREDICTED: condensin complex subunit 3
RS005159	GO:0044699	single-organism process	BP	0.663011183	1.016331225	1.065582964	9.044461009	13.27695529	0.004074393	0.016611495	PREDICTED: cofilin/actin-depolymerizing factor homolog
RS009785	GO:0044699	single-organism process	BP	0.335842818	0.756139448	0.899027229	6.65430016	13.2620304	0.004102891	0.016711453	PREDICTED: glutaredoxin-related protein 5, mitochondrial
RS005402	GO:0044699	single-organism process	BP	-0.379275472	-0.086413517	0.400493634	5.978759037	13.26009271	0.004106606	0.016721174	PREDICTED: ras-specific guanine nucleotide-releasing factor RalGPS2
RS015561	GO:0044699	single-organism process	BP	-0.850385576	-0.319041778	-1.043739094	4.615795366	13.2424681	0.004140543	0.01683215	malate dehydrogenase
RS014890	GO:0044699	single-organism process	BP	-2.326302792	-1.929909571	0.173695568	0.355706774	13.23858014	0.004148067	0.016851857	PREDICTED: secretin receptor-like
RS008148	GO:0044699	single-organism process	BP	-0.182411272	0.33937493	0.804327555	8.139861375	13.23875929	0.00414772	0.016851857	PREDICTED: NADH dehydrogenase [ubiquinone] iron-sulfur protein 3, mitochondrial
RS010905	GO:0044699	single-organism process	BP	-0.573248945	-0.768712677	-0.837001645	4.71486708	13.23543162	0.00415417	0.016871208	PREDICTED: gamma-aminobutyric acid receptor subunit beta
RS013741	GO:0044699	single-organism process	BP	-0.437564905	0.321626957	1.01206774	6.184564336	13.22901631	0.004166632	0.016910915	PREDICTED: cysteine-rich protein 1-like
RS014143	GO:0044699	single-organism process	BP	0.691610154	0.789866634	0.915978507	6.771675255	13.20990433	0.004203979	0.017029692	PREDICTED: dihydroorotate dehydrogenase (quinone), mitochondrial
RS014537	GO:0044699	single-organism process	BP	0.226106828	-0.47363146	-0.693319269	5.358848092	13.17406228	0.004274914	0.017272464	carboxylesterase
RS014495	GO:0044699	single-organism process	BP	-2.130730125	-2.379705587	-0.504966123	0.486362894	13.13791982	0.004347643	0.017526959	Dehydrogenase/reductase SDR family member 11
RS004022	GO:0044699	single-organism process	BP	0.446259896	-0.09190358	-0.876496708	3.32323364	13.1157164	0.004392929	0.017692531	PREDICTED: centromere protein X
RS007147	GO:0044699	single-organism process	BP	-0.183438953	-1.074134638	-0.371314656	3.810955234	13.11351454	0.004397445	0.017702537	PREDICTED: hydroxyacid oxidase 1
RS013657	GO:0044699	single-organism process	BP	0.592912262	0.820666836	1.014808098	7.401076254	13.11313477	0.004398224	0.017702537	PREDICTED: 39S ribosomal protein L39, mitochondrial
RS010139	GO:0044699	single-organism process	BP	0.261430241	0.997416812	0.564250626	4.010572694	13.10462664	0.004415722	0.017755938	PREDICTED: kinesin-like protein KIF12
RS002313	GO:0044699	single-organism process	BP	-0.451678341	0.180904234	-0.854553222	5.296298932	13.08468271	0.004457007	0.017899092	PREDICTED: glucose-6-phosphate 1-dehydrogenase
RS005864	GO:0044699	single-organism process	BP	-0.832779988	-0.598675317	0.034192681	5.983079267	13.07753281	0.004471901	0.01795318	PREDICTED: laminin subunit gamma-1
RS003686	GO:0044699	single-organism process	BP	-0.829001752	-1.737132543	-0.769993978	1.792228626	13.05859208	0.004511595	0.018098763	PREDICTED: dynein heavy chain 5, axonemal
RS005301	GO:0044699	single-organism process	BP	0.054695424	0.943656704	0.357347462	5.262032246	13.05593276	0.004517196	0.018107055	PREDICTED: apolipoprotein D
RS015097	GO:0044699	single-organism process	BP	0.838222973	0.450337326	-0.261713518	6.777317829	13.04891082	0.004532018	0.018148247	PREDICTED: tubulin alpha-1 chain

RS014395	GO:0044699	single-organism process	BP	0.315687369	0.588236519	0.952986831	7.967008333	13.03931815	0.004552345	0.018212271	PREDICTED: ATPase ASNA1 homolog
RS007058	GO:0044699	single-organism process	BP	0.259575136	-0.23976162	-0.538042359	6.617702274	13.01457146	0.0046052	0.018406183	PREDICTED: replication protein A 70 kDa DNA-binding subunit
RS009404	GO:0044699	single-organism process	BP	0.443476694	0.123699806	-0.392860437	6.921259727	13.00862403	0.004617993	0.018439758	PREDICTED: AP-2 complex subunit sigma
RS003535	GO:0044699	single-organism process	BP	-0.7581095	-1.259736634	-1.152250668	3.440687943	12.99767818	0.004641629	0.018518434	PREDICTED: lipase 3-like
RS014000	GO:0044699	single-organism process	BP	0.486986842	0.493065666	-0.511287156	7.326747337	12.99389808	0.00464982	0.018525729	TWiK family of potassium channels protein 18
RS010857	GO:0044699	single-organism process	BP	0.764148813	0.870742281	0.980352619	4.070999981	12.98153732	0.004676703	0.018621054	PREDICTED: AP-5 complex subunit sigma-1
RS007615	GO:0044699	single-organism process	BP	0.842199769	0.08001472	-0.354074907	7.382223287	12.96671251	0.004709147	0.018738389	PREDICTED: facilitated trehalose transporter Tret1-like
RS008090	GO:0044699	single-organism process	BP	0.823286106	0.508776808	-0.0807306	4.788950509	12.9625918	0.004718205	0.018768501	PREDICTED: Bardet-Biedl syndrome 4 protein
RS014527	GO:0044699	single-organism process	BP	0.569815179	0.595367911	0.893346442	6.881844181	12.93354807	0.004782538	0.019006399	PREDICTED: transcription and mRNA export factor ENY2
RS009335	GO:0044699	single-organism process	BP	0.731058414	-0.369381984	0.309156103	5.654254453	12.91117936	0.004832676	0.01918749	PREDICTED: beta-galactosidase
RS004856	GO:0044699	single-organism process	BP	0.852844308	0.720679081	0.580581782	6.068734238	12.91031498	0.004834624	0.019189174	PREDICTED: aspartate--tRNA ligase, cytoplasmic
RS001835	GO:0044699	single-organism process	BP	0.805773835	0.650362199	0.718101486	5.354738576	12.8981259	0.004862174	0.019280297	PREDICTED: protein SYS1 homolog
RS014993	GO:0044699	single-organism process	BP	0.045913843	0.758103294	0.764834294	4.621384277	12.88648713	0.004888626	0.019372988	PREDICTED: transcriptional activator protein Pur-beta
RS004184	GO:0044699	single-organism process	BP	-0.64484503	-0.705907962	-1.121612296	3.508665996	12.88356376	0.004895292	0.019393304	PREDICTED: PI-PLC X domain-containing protein 3
RS004058	GO:0044699	single-organism process	BP	-0.732025658	-0.494762615	0.44879129	4.228368541	12.87647487	0.004911495	0.019451374	PREDICTED: SH3 and cysteine-rich domain-containing protein 2
RS015215	GO:0044699	single-organism process	BP	-1.515846961	-0.31728745	0.336886429	1.860768736	12.86370702	0.004940812	0.019542897	PREDICTED: cytochrome P450 4c3
RS009863	GO:0044699	single-organism process	BP	0.247967949	1.994626146	0.840783905	0.741237487	12.85234652	0.004967042	0.019634316	PREDICTED: sodium- and chloride-dependent glycine transporter 1-like
RS007711	GO:0044699	single-organism process	BP	-0.29668746	-0.658074436	-0.677818273	5.328856467	12.83578234	0.005005535	0.019744379	PREDICTED: membrane-associated protein Hem
RS003166	GO:0044699	single-organism process	BP	0.923028584	1.385520407	1.271308157	9.837403841	12.8356431	0.00500586	0.019744379	PREDICTED: 60S ribosomal protein L23
RS005166	GO:0044699	single-organism process	BP	0.797745292	0.959053152	1.137980998	8.88871257	12.83598972	0.005005051	0.019744379	PREDICTED: nascent polypeptide-associated complex subunit alpha
RS007237	GO:0044699	single-organism process	BP	0.458239901	-0.486538959	-1.248359032	1.882172727	12.79602085	0.005099144	0.020074591	PREDICTED: pyridine nucleotide-disulfide oxidoreductase domain-containing protein 2
RS010780	GO:0044699	single-organism process	BP	-0.697464891	-1.313825464	-0.263747329	3.243099766	12.78869749	0.005116573	0.020130621	Protostome-specific GEF
RS005524	GO:0044699	single-organism process	BP	-1.220098668	-1.714891222	-1.517818703	2.425389351	12.78149174	0.005133779	0.020187032	PREDICTED: gonadotropin-releasing hormone II receptor
RS000004	GO:0044699	single-organism process	BP	-0.265184605	-0.172398945	0.468191605	5.017738015	12.78135077	0.005134116	0.020187032	PREDICTED: glycerol kinase-like
RS005460	GO:0044699	single-organism process	BP	-0.996149896	-0.953790558	-0.537751487	3.769830749	12.77993102	0.005137514	0.020193827	PREDICTED: solute carrier family 22 member 21-like
RS013357	GO:0044699	single-organism process	BP	0.435058831	0.347921288	-0.38140622	5.378689111	12.77702873	0.005144465	0.020198398	PREDICTED: PCI domain-containing protein 2
RS007447	GO:0044699	single-organism process	BP	-0.998379247	-0.601198746	-0.20487521	4.319039793	12.77088025	0.005159223	0.02024151	PREDICTED: HIG1 domain family member 1A, mitochondrial-like
RS013556	GO:0044699	single-organism process	BP	0.690755036	0.669338842	-0.517703345	3.064715758	12.76443692	0.005174733	0.02029604	PREDICTED: cardioacceleratory peptide receptor-like
RS007598	GO:0044699	single-organism process	BP	0.827042594	0.45973922	0.026042821	4.793261851	12.75657898	0.00519371	0.020351459	PREDICTED: nucleolar protein 12
RS001256	GO:0044699	single-organism process	BP	0.746468746	0.212019743	-0.274350079	6.384861873	12.75431264	0.005199197	0.02036662	PREDICTED: protein disulfide-isomerase A6
RS012757	GO:0044699	single-organism process	BP	0.070512735	-1.04697516	-0.817513738	3.319678238	12.73995861	0.005234077	0.020484142	PREDICTED: cell division cycle protein 20 homolog
RS004076	GO:0044699	single-organism process	BP	-1.141656326	-0.063293219	0.525187867	2.734823933	12.67761469	0.00538828	0.021048388	PREDICTED: free fatty acid receptor 4-like
RS003529	GO:0044699	single-organism process	BP	0.881391282	0.664029112	0.740069814	7.280539789	12.67459049	0.005395874	0.021071514	PREDICTED: serine--tRNA ligase, mitochondrial
RS002029	GO:0044699	single-organism process	BP	-0.13328777	0.727266831	0.310534802	4.952812509	12.66589221	0.005417773	0.021124282	PREDICTED: MOB kinase activator-like 2
RS010471	GO:0044699	single-organism process	BP	0.366315986	0.745162152	0.508533944	6.75081825	12.61429099	0.005549506	0.021584458	PREDICTED: valine--tRNA ligase
RS000117	GO:0044699	single-organism process	BP	0.856813981	0.766148652	0.4845856	4.570251762	12.60353146	0.00557737	0.021672756	PREDICTED: dual specificity protein phosphatase 19
RS010247	GO:0044699	single-organism process	BP	-0.356860545	-0.683501085	-0.645803449	7.120569921	12.59740166	0.005593307	0.021727979	PREDICTED: nucleoprotein TPR
RS005740	GO:0044699	single-organism process	BP	0.03115726	1.018554939	1.081039447	5.490146434	12.59344835	0.005603609	0.021761285	PREDICTED: putative epidermal cell surface receptor
RS002274	GO:0044699	single-organism process	BP	1.331323712	0.880001876	0.882713794	4.900841985	12.58372928	0.005629015	0.021846477	PAN domain containing protein 1
RS005241	GO:0044699	single-organism process	BP	-1.24648029	-0.946331544	-0.692073388	4.41212653	12.5745733	0.005653054	0.021933014	PREDICTED: high-affinity choline transporter 1
RS002245	GO:0044699	single-organism process	BP	0.706012832	0.884331936	0.688633334	5.827708866	12.56036255	0.005690565	0.022071752	PREDICTED: dolichol-phosphate mannosyltransferase subunit 3
RS001428	GO:0044699	single-organism process	BP	-0.868061308	-0.865227926	0.015938791	5.656555355	12.55011961	0.005717755	0.022156742	PREDICTED: solute carrier organic anion transporter family member 4A1
RS006299	GO:0044699	single-organism process	BP	0.929966764	0.90845916	-0.169322471	3.276482954	12.53656321	0.005753939	0.022276393	PREDICTED: facilitated trehalose transporter Tret1-like
RS012881	GO:0044699	single-organism process	BP	0.665873857	0.515282975	-0.219908381	8.237769757	12.5305365	0.005770097	0.022314995	PREDICTED: 26S protease regulatory subunit 4
RS004258	GO:0044699	single-organism process	BP	0.652929099	0.433035473	-0.111504098	4.927446963	12.53020143	0.005770997	0.022314995	PREDICTED: methylthioribose-1-phosphate isomerase
RS015184	GO:0044699	single-organism process	BP	1.039615177	0.841127939	1.341816666	3.384450441	12.5313472	0.005767921	0.022314995	PREDICTED: ras-related and estrogen-regulated growth inhibitor-like protein
RS014138	GO:0044699	single-organism process	BP	0.31436076	0.038655478	-0.908473672	3.026423122	12.52239561	0.005791996	0.022375581	PREDICTED: HAUS augmin-like complex subunit 6
RS012434	GO:0044699	single-organism process	BP	-1.482379263	-1.438022634	-0.987309697	2.418516034	12.51006701	0.005825315	0.022469837	PREDICTED: protein decapentaplegic
RS008051	GO:0044699	single-organism process	BP	-0.405673123	-1.320265794	-1.066020494	4.63196021	12.49923358	0.00585475	0.022555742	PREDICTED: transient receptor potential channel pyrexia
RS006766	GO:0044699	single-organism process	BP	-0.318612109	-0.764780567	0.962282512	4.270639859	12.4793297	0.005909213	0.022737743	PREDICTED: alpha-(1,3)-fucosyltransferase 7-like
RS011700	GO:0044699	single-organism process	BP	0.123275019	-0.824221198	-1.02267516	2.529023274	12.45616107	0.005973241	0.022959384	PREDICTED: cell division control protein 45 homolog
RS011677	GO:0044699	single-organism process	BP	0.770186673	1.322492184	-0.833866673	2.584628121	12.45352527	0.005980569	0.022963196	PREDICTED: ecdysone-induced protein 78C
RS001133	GO:0044699	single-organism process	BP	1.00904016	0.474949441	0.334976687	4.645037343	12.45381435	0.005979765	0.022963196	PREDICTED: bolA-like protein 2
RS006902	GO:0044699	single-organism process	BP	0.862607351	0.755707819	0.498838266	6.615986417	12.4480253	0.005995887	0.023014997	PREDICTED: conserved oligomeric Golgi complex subunit 5
RS008149	GO:0044699	single-organism process	BP	-0.682097649	-0.786633441	-0.697613254	4.651426879	12.43598979	0.006029544	0.023130088	PREDICTED: centrosome-associated protein 350-like
RS007784	GO:0044699	single-organism process	BP	-0.688772057	0.750724914	0.712844616	3.529213198	12.40986621	0.006103242	0.023398546	PREDICTED: ras-like protein 2
RS005478	GO:0044699	single-organism process	BP	-0.317320863	0.181393706	0.628036643	7.037516725	12.40117718	0.006127951	0.023478983	NADH:ubiquinone dehydrogenase
RS000227	GO:0044699	single-organism process	BP	-1.32952285	-0.75353739	-1.12184367	1.981758767	12.38833226	0.00616466	0.023605269	PREDICTED: facilitated trehalose transporter Tret1-2 homolog
RS014031	GO:0044699	single-organism process	BP	-0.521995584	-0.786620499	-1.18332395	3.371095243	12.38651585	0.006169869	0.023610856	PREDICTED: glutathione S-transferase-like
RS011032	GO:0044699	single-organism process	BP	-0.526292146	-0.829736458	-0.646061965	4.781042562	12.38343437	0.006178715	0.023623174	PREDICTED: D-aspartate oxidase
RS009438	GO:0044699	single-organism process	BP	-1.808479608	-1.529808712	-2.421969601	0.542438384	12.37208909	0.006211393	0.023740904	PREDICTED: E3 ubiquitin-protein ligase SIAH1-like
RS008171	GO:0044699	single-organism process	BP	-1.67818786	-2.37701163	-1.564123169	0.367095425	12.35670743	0.006255969	0.023875053	PREDICTED: alpha-1A adrenergic receptor
RS003447	GO:0044699	single-organism process	BP	-0.205250547	-0.841085384	-0.843141784	4.078788691	12.27106946	0.006509993	0.024774348	PREDICTED: protein tiptop
RS014920	GO:0044699	single-organism process	BP	-0.165620846	-0.81666407	-0.627381341	4.272840612	12.22470061	0.006651762	0.025232617	PREDICTED: 15-hydroxyprostaglandin dehydrogenase [NAD(+)]-like
RS001115	GO:0044699	single-organism process	BP	-0.445852787	-0.566595293	0.166847494	6.214720571	12.22049672	0.006664765	0.025274329	PREDICTED: Ca(2+)/calmodulin-responsive adenylate cyclase
RS006342	GO:0044699	single-organism process	BP	0.734709905	0.839191818	0.013573097	6.490494103	12.21838984	0.006671291	0.025291462	PREDICTED: N-acetylgalactosaminyltransferase 7
RS003528	GO:0044699	single-organism process	BP	0.030278166	0.673651934	0.772131025	7.357292374	12.16799176	0.006829297	0.025820525	PREDICTED: multiple inositol polyphosphate phosphatase 1-like
RS003371	GO:0044699	single-organism process	BP	0.472707825	-0.12338578	-0.39256252	5.540350268	12.16672405	0.006833319	0.025827977	PREDICTED: UDP-glucuronosyltransferase 2B15-like
RS012250	GO:0044699	single-organism process	BP	-1.126562781	-0.381786444	0.539034757	1.985904525	12.14501042	0.00690257	0.026042829	PREDICTED: organic cation transporter protein-like
RS006443	GO:0044699	single-organism process	BP	0.143296238	-0.770738062	-0.789685541	3.548052193	12.1322361	0.006943634	0.026189915	PREDICTED: alpha,alpha-trehalose-phosphate synthase [UDP-forming]
RS009864	GO:0044699	single-organism process	BP	-0.647431205	-0.951712197	-0.350535749	4.647846858	12.11603837	0.00699605	0.026348164	PREDICTED: acyl-CoA synthetase family member 2, mitochondrial-like
RS009199	GO:0044699	single-organism process	BP	0.023382962	0.544949349	0.623954223	6.289729047	12.11452547	0.007000966	0.026358795	PREDICTED: 39S ribosomal protein L2, mitochondrial
RS007161	GO:0044699	single-organism process	BP	-0.468052015	-0.115533583	0.490115432	4.815745054	12.11343226	0.00700452	0.026364294	PREDICTED: methionine-R-sulfoxide reductase B1
RS004483	GO:0044699	single-organism process	BP	0.059907484	0.360300197	0.720781759	6.766342642	12.11043105	0.007014287	0.02638843	PREDICTED: mitochondrial inner membrane protein OXA1L
RS003527	GO:0044699	single-organism process	BP	-0.020649905	0.221205466	-0.508347411	6.143757947	12.10770359	0.007023174	0.026410826	PREDICTED: protein LMBR1L
RS004676	GO:0044699	single-organism process	BP	-0.546152282	0.147502295	0.556615017	4.228252059	12.09936204	0.007050424	0.026497476	PREDICTED: formin-binding protein 1-like
RS009081	GO:0044699	single-organism process	BP	-0.352538222	-1.124692397	-0.854023886	3.040871436	12.09356451	0.007069425	0.026560959	PREDICTED: zinc finger protein 37 homolog
RS002985	GO:0044699	single-organism process	BP	-0.571114377	-0.476136182	-0.859409917	5.548946497	12.07709757	0.007123669	0.026732864	PREDICTED: Y+L amino acid transporter 2
RS000658	GO:0044699	single-organism process	BP	0.659057673	0.426168092	0.082378635	6.04702022	12.06164842	0.007174935	0.026877196	PREDICTED: trafficking protein particle complex subunit 4
RS003288	GO:0044699	single-organism process	BP	-0.468141457	-0.663116971	0.331112622	4.523147937	12.02307867	0.007304519	0.027292266	PREDICTED: sodium- and chloride-dependent GABA transporter ine
RS007097	GO:0044699	single-organism process	BP	0.340367486	-0.138491072	-0.70896225	4.143803222	11.99504412	0.007400157	0.027589571	PREDICTED: methylcrotonoyl-CoA carboxylase subunit alpha, mitochondrial
RS005266	GO:0044699	single-organism process	BP	-0.969208784	-1.627785117	-0.854089636	2.260064915	11.99115604	0.007413518	0.027631205	PREDICTED: gastrin-releasing peptide receptor-like
RS008754	GO:0044699	single-organism process	BP	-0.126337377	0.390181258	0.766731787	6.919760824	11.97078614	0.007483908	0.027852345	PREDICTED: dynein light chain 2, cytoplasmic
RS003997	GO:0044699	single-organism process	BP	-1.222850277	-0.635965438	-0.411407393	6.247970682	11.97127476	0.007482212	0.027852345	PREDICTED: ABC transporter G family member 23
RS009676	GO:0044699	single-organism process	BP	0.692787818	0.692803944	0.715971572	5.777323004	11.96171195	0.007515477	0.02796157	PREDICTED: small nuclear ribonucleoprotein F
RS010973	GO:0044699	single-organism process	BP	0.220348063	-1.900264308	-1.052818685	1.491257599	11.95394616	0.007542598	0.028054188	PREDICTED: gastrin-releasing peptide receptor-like
RS011665	GO:0044699	single-organism process	BP	-0.460489579	-1.326999046	-1.061984026	2.228596867	11.95023512	0.007555593	0.028094223	PREDICTED: vesicular inhibitory amino acid transporter
RS001450	GO:0044699	single-organism process	BP	-0.506441485	-0.627602418	-0.685886129	5.506186136	11.94564203	0.007571707	0.028137525	PREDICTED: neurofibromin
RS010964	GO:0044699	single-organism process	BP	-1.004995812	-0.588637144	-0.424145768	8.038347207	11.94194578	0.007584699	0.028169182	PREDICTED: low-density lipoprotein receptor-related protein 2
RS009001	GO:0044699	single-organism process	BP	0.383702821	0.510464719	0.758420865	6.143991765	11.9364335	0.007604115	0.02821633	PREDICTED: 39S ribosomal protein L48, mitochondrial
RS006235	GO:0044699	single-organism process	BP	0.69957514	1.168387755	0.896391877	8.425585193	11.92747243	0.007635784	0.028308821	PREDICTED: probable aminoacyl tRNA synthase complex-interacting multifunctional protein 2
RS003989	GO:0044699	single-organism process	BP	0.310667742	0.580977423	0.80300078	6.925603516	11.91982245	0.007662923	0.028401074	PREDICTED: transcription initiation factor TFIID subunit 10-like
RS011468	GO:0044699	single-organism process	BP	-0.318737056	-0.110014844	-1.568608212	1.963272184	11.9167036	0.007674014	0.028433815	PREDICTED: solute carrier family 35 member F6
RS000866	GO:0044699	single-organism process	BP	-0.851954648	-0.389874807	-0.092110043	7.989972168	11.90615845	0.007711634	0.028547895	PREDICTED: sodium/potassium-transporting ATPase subunit alpha
RS000574	GO:0044699	single-organism process	BP	-0.885042738	-1.056461743	-1.076450855	4.349937012	11.90553297	0.007713871	0.028547895	allatostatin neuropeptide precursor
RS008554	GO:0044699	single-organism process	BP	0.990986195	0.682629014	0.463563703	6.643926243	11.89255172	0.007760444	0.028678121	PREDICTED: probable phenylalanine--tRNA ligase beta subunit
RS009834	GO:0044699	single-organism process	BP	0.440417977	0.629739022	0.755048365	6.326460704	11.86532553	0.007859029	0.028974418	PREDICTED: 39S ribosomal protein L21, mitochondrial
RS007818	GO:0044699	single-organism process	BP	0.457706947	0.681077476	0.184430982	6.661999168	11.8654647	0.007858522	0.028974418	PREDICTED: protein flightless-1
RS011788	GO:0044699	single-organism process	BP	-0.501112324	-0.797613147	-0.632406168	6.743212918	11.79762557	0.008109561	0.029828215	PREDICTED: afadin
RS004829	GO:0044699	single-organism process	BP	0.235783952	-0.878250286	-0.950976758	2.167082869	11.79806585	0.008107906	0.029828215	PREDICTED: serine/threonine-protein kinase Nek2
RS003756	GO:0044699	single-organism process	BP	-0.408342321	-0.364800511	-0.786353931	7.1095919	11.79402076	0.00812312	0.029860645	PREDICTED: multidrug resistance-associated protein 1
RS007809	GO:0044699	single-organism process	BP	-1.465331767	-1.532092907	-1.031211145	3.519019597	11.74408596	0.008313261	0.030479536	PREDICTED: gremlin-1
RS010467	GO:0044699	single-organism process	BP	0.793039659	-0.679498755	-0.472159802	2.19594104	11.73705171	0.008340396	0.030561232	PREDICTED: kinesin-like protein KIF23
RS015096	GO:0044699	single-organism process	BP	-0.857980934	-0.446788274	-0.194471442	5.697782265	11.71197794	0.008437836	0.030900291	PREDICTED: V-type proton ATPase subunit D
RS010014	GO:0044699	single-organism process	BP	0.35427573	-0.016418124	-0.422862839	5.19528713	11.70149308	0.008478913	0.031024326	PREDICTED: DNA-3-methyladenine glycosylase-like
RS009360	GO:0044699	single-organism process	BP	-0.052778092	-0.516301747	0.304429459	6.031942967	11.69868265	0.008489957	0.031037017	PREDICTED: sodium-dependent nutrient amino acid transporter 1
RS008458	GO:0044699	single-organism process	BP	-1.182823715	-0.196910535	-0.390818396	8.429616967	11.6884148	0.008530428	0.031131399	PREDICTED: probable cation-transporting ATPase 13A3
RS014992	GO:0044699	single-organism process	BP	0.229136004	0.615757621	0.676877541	5.913523856	11.67233841	0.008594176	0.031318014	PREDICTED: sodium-dependent phosphate transport protein 1, chloroplastic-like
RS010422	GO:0044699	single-organism process	BP	-0.891847421	-1.189840939	-1.580420114	2.046452779	11.66510339	0.008623019	0.031412228	PREDICTED: noggin-2-like
RS011196	GO:0044699	single-organism process	BP	0.675575742	0.753360958	0.761120595	5.457606567	11.66397815	0.008627513	0.031412228	PREDICTED: 39S ribosomal protein L19, mitochondrial
RS011756	GO:0044699	single-organism process	BP	0.566418511	0.699886652	0.753619757	6.355142205	11.65708835	0.008655082	0.031503498	PREDICTED: prostaglandin E synthase 2

RS001952	GO:0044699	single-organism process	BP	-0.796459757	-0.904276224	-1.349830192	3.620084555	11.65393196	0.008667741	0.03154046	PREDICTED: delta and Notch-like epidermal growth factor-related receptor
RS012631	GO:0044699	single-organism process	BP	-1.865512908	-2.096612173	-0.212150587	1.249055231	11.64984926	0.008684143	0.031591014	E3 ubiquitin-protein ligase sina
RS004997	GO:0044699	single-organism process	BP	-0.851696275	-1.530670443	-0.345032758	2.982640462	11.64463017	0.008705154	0.031658304	PREDICTED: kinesin-like protein KIF17
RS015567	GO:0044699	single-organism process	BP	0.376413076	-0.071504162	-0.448105985	7.311814141	11.60916555	0.008849263	0.032126729	PREDICTED: glucosidase 2 subunit beta
RS013049	GO:0044699	single-organism process	BP	0.534016123	0.720426157	0.617615712	5.498431269	11.59548651	0.008905475	0.032302867	PREDICTED: dual specificity protein phosphatase 23-like
RS004333	GO:0044699	single-organism process	BP	-0.614786023	-0.06913771	0.157411092	6.656175065	11.58624491	0.008943651	0.032404011	PREDICTED: sulfide:quinone oxidoreductase, mitochondrial
RS000186	GO:0044699	single-organism process	BP	0.094099037	0.039473519	-0.72472192	7.551125593	11.56473727	0.009033123	0.032681171	PREDICTED: cyclic AMP response element-binding protein A-like
RS002363	GO:0044699	single-organism process	BP	-0.329275879	-0.729371826	0.101654401	6.167217704	11.55948198	0.009055119	0.032732541	PREDICTED: multidrug resistance-associated protein 5
RS008946	GO:0044699	single-organism process	BP	-0.997190713	-0.414222367	-1.262775862	2.122161935	11.55237631	0.009084944	0.032830929	PREDICTED: B9 domain-containing protein 2
RS008898	GO:0044699	single-organism process	BP	-0.172960297	-0.478698026	-0.774628888	4.936093792	11.53878925	0.009142244	0.033009582	PREDICTED: sideroflexin-1
RS009060	GO:0044699	single-organism process	BP	-0.445440747	0.052931611	1.34094544	4.027025825	11.53172946	0.009172158	0.033098611	PREDICTED: elongation of very long chain fatty acids protein 4-like
RS004828	GO:0044699	single-organism process	BP	0.100031683	0.494832512	0.956002198	6.978957936	11.52373086	0.009206166	0.033202305	PREDICTED: NADH dehydrogenase [ubiquinone] 1 beta subcomplex subunit 1
RS007363	GO:0044699	single-organism process	BP	-0.383482262	-1.417236113	-0.774050401	4.8160579	11.5194449	0.00922444	0.033258283	PREDICTED: patatin-like phospholipase domain-containing protein 3
RS100010	GO:0044699	single-organism process	BP	-1.411018608	-2.350967327	-0.955704779	0.944710369	11.51684529	0.009235541	0.033279657	PREDICTED: geranylgeranyl pyrophosphate synthase
RS003297	GO:0044699	single-organism process	BP	-0.227427683	-0.016437135	0.553846864	5.31488792	11.50297001	0.009295017	0.033436228	PREDICTED: pyruvate dehydrogenase phosphatase regulatory subunit, mitochondrial-like
RS002295	GO:0044699	single-organism process	BP	-0.760632417	-0.881718429	-1.336627404	7.31207469	11.50237509	0.009297576	0.033436228	PREDICTED: sodium- and chloride-dependent GABA transporter 1-like
RS012916	GO:0044699	single-organism process	BP	-0.746562811	-0.727766733	-0.234283384	4.938742363	11.49914688	0.009311471	0.03347664	PREDICTED: phospholipase D2
RS011114	GO:0044699	single-organism process	BP	0.183353558	0.829780316	0.406972459	4.727187822	11.49727609	0.009319533	0.033496062	PREDICTED: 3-oxoacyl-[acyl-carrier-protein] reductase FabG-like
RS013954	GO:0044699	single-organism process	BP	-1.055619515	-0.668164846	-0.471918087	5.887744052	11.48893867	0.009355547	0.033596737	PREDICTED: renin receptor
RS008273	GO:0044699	single-organism process	BP	-0.474841961	-0.70204137	-0.865745137	6.751270883	11.48819486	0.009358766	0.033598718	PREDICTED: histone acetyltransferase KAT6B
RS002359	GO:0044699	single-organism process	BP	0.586201128	0.978364088	-0.589225833	1.498220939	11.48711924	0.009363424	0.03360586	PREDICTED: alpha-1B adrenergic receptor
RS004482	GO:0044699	single-organism process	BP	0.583687449	0.821155136	0.729658871	6.729625612	11.48194801	0.009385849	0.033673341	PREDICTED: MIP18 family protein CG7949
RS010641	GO:0044699	single-organism process	BP	-0.807148767	-0.226247423	-0.169065413	4.942005241	11.48155079	0.009387573	0.033673341	PREDICTED: probable phospholipid-transporting ATPase VD
RS006548	GO:0044699	single-organism process	BP	0.059898012	0.416718771	0.839565831	7.671508238	11.47459596	0.009417822	0.033753001	PREDICTED: acyl carrier protein, mitochondrial
RS001253	GO:0044699	single-organism process	BP	-1.492058856	-1.192715108	-0.868809965	2.190673979	11.45902163	0.009485907	0.033939067	PREDICTED: phospholipase ABHD3
RS014933	GO:0044699	single-organism process	BP	-0.642346894	-0.209430891	-0.767671226	4.860259423	11.44341112	0.009554638	0.034126805	PREDICTED: probable cytochrome P450 304a1
RS010715	GO:0044699	single-organism process	BP	-0.691275094	-0.650068955	-0.591774492	7.210638046	11.41681174	0.009672886	0.034519786	PREDICTED: diacylglycerol kinase theta
RS002391	GO:0044699	single-organism process	BP	0.444122363	0.61799603	0.876500108	7.743786052	11.41361504	0.009687194	0.034551265	PREDICTED: complement component 1 Q subcomponent-binding protein, mitochondrial
RS012787	GO:0044699	single-organism process	BP	-1.042887095	-1.14108662	-0.848878197	2.917253142	11.38739346	0.009805347	0.03491082	PREDICTED: facilitated trehalose transporter Tret1-2 homolog
RS000698	GO:0044699	single-organism process	BP	0.214834477	-0.527958335	-0.778262071	3.682560769	11.38730067	0.009805768	0.03491082	PREDICTED: DNA replication licensing factor MCM4
RS010245	GO:0044699	single-organism process	BP	-1.987623381	2.222422249	0.2664132	2.039098054	11.36271722	0.009917835	0.035273994	PREDICTED: vascular endothelial growth factor A-like
RS001899	GO:0044699	single-organism process	BP	-1.010441966	-1.763289491	0.943059021	0.256564282	11.34768176	0.009986998	0.035489914	PREDICTED: odorant receptor 49b-like
RS004743	GO:0044699	single-organism process	BP	-0.687052251	0.374482001	0.129333798	5.270250719	11.33865564	0.010028747	0.035628218	PREDICTED: FERM and PDZ domain-containing protein 4
RS000505	GO:0044699	single-organism process	BP	-0.550172818	-0.780268786	-0.465996455	5.329225797	11.32824795	0.010077099	0.035769725	PREDICTED: synaptic vesicular amine transporter
RS000219	GO:0044699	single-organism process	BP	0.217052434	-0.673773561	-1.028588766	6.485154642	11.32601418	0.010087507	0.035776418	PREDICTED: innexin inx2
RS012907	GO:0044699	single-organism process	BP	-0.836480451	0.263478228	0.21997425	3.849870347	11.31256718	0.010150384	0.035958912	PREDICTED: multiple inositol polyphosphate phosphatase 1-like
RS005321	GO:0044699	single-organism process	BP	-1.514373496	-1.388973316	-1.386050817	1.60456008	11.30958913	0.010164361	0.035998303	PREDICTED: COUP transcription factor 2
RS014273	GO:0044699	single-organism process	BP	-0.268192968	-0.584630007	0.366094757	4.564432181	11.2907457	0.010253243	0.036262102	PREDICTED: sialin-like
RS007896	GO:0044699	single-organism process	BP	0.744637369	1.164990107	-0.247013701	3.910217304	11.29168244	0.010248807	0.036262102	PREDICTED: M-phase inducer phosphatase-like
RS008942	GO:0044699	single-organism process	BP	-1.103445792	-0.654369525	-0.512935524	4.286093906	11.2746722	0.010329665	0.036511873	PREDICTED: rho guanine nucleotide exchange factor 11
RS006104	GO:0044699	single-organism process	BP	-1.23967714	-0.389611593	-0.470245408	8.765952961	11.26951111	0.010354323	0.03658876	PREDICTED: glutamine synthetase 2 cytoplasmic
RS010694	GO:0044699	single-organism process	BP	-1.376784469	-1.151365045	-1.019735694	3.615734954	11.25799445	0.010409554	0.036761977	PREDICTED: glutamate decarboxylase
RS007213	GO:0044699	single-organism process	BP	0.838702364	0.625882382	0.393178148	5.329226936	11.24943271	0.010450803	0.036888287	PREDICTED: ribose-5-phosphate isomerase
RS008780	GO:0044699	single-organism process	BP	-1.708644245	-1.134360468	-1.179254192	3.130067319	11.24406261	0.010476757	0.03695918	PREDICTED: receptor-type tyrosine-protein phosphatase N2
RS006779	GO:0044699	single-organism process	BP	2.113003052	0.36019819	1.956574207	0.455420701	11.23781765	0.010507019	0.037055558	PREDICTED: neurophysin 1-like
RS012570	GO:0044699	single-organism process	BP	-0.317030466	-0.896613556	0.124483324	3.719243132	11.23592539	0.010516206	0.037077574	PREDICTED: adenosine receptor A2b-like
RS003971	GO:0044699	single-organism process	BP	-0.368274612	-0.238258011	0.34922185	6.920050921	11.22343656	0.010577036	0.037281611	PREDICTED: glycerol-3-phosphate acyltransferase 1, mitochondrial
RS005095	GO:0044699	single-organism process	BP	0.664372765	0.123971195	-0.105061691	6.434923294	11.21954757	0.010596049	0.037318309	PREDICTED: endoplasmic reticulum-Golgi intermediate compartment protein 2
RS008687	GO:0044699	single-organism process	BP	-0.359897482	-0.945966528	-0.989157806	3.133501312	11.19321575	0.010725675	0.037731607	PREDICTED: origin recognition complex subunit 1
RS007770	GO:0044699	single-organism process	BP	0.590420984	0.680667049	0.570518779	6.237334319	11.18677044	0.010757642	0.037812375	PREDICTED: probable trafficking protein particle complex subunit 2
RS014519	GO:0044699	single-organism process	BP	0.689544211	0.908547366	0.415941488	5.248194553	11.17920863	0.010795265	0.037912875	PREDICTED: irregular chiasm C-roughest protein
RS007389	GO:0044699	single-organism process	BP	-0.71821859	-0.059688714	0.178211598	8.141021926	11.17756615	0.010803454	0.037931058	PREDICTED: V-type proton ATPase catalytic subunit A
RS003157	GO:0044699	single-organism process	BP	-0.488485782	-0.878257668	-1.341628209	5.535906607	11.17557061	0.010813412	0.037955438	cytochrome P450
RS014033	GO:0044699	single-organism process	BP	-0.436152357	-0.743556646	0.220958975	4.477033022	11.14471657	0.010968532	0.03845704	PREDICTED: caspase-1-like
RS000910	GO:0044699	single-organism process	BP	-0.675555848	0.753469081	0.450845434	5.001071772	11.14284403	0.010978016	0.038468874	PREDICTED: arrestin domain-containing protein 2
RS010026	GO:0044699	single-organism process	BP	-0.646643137	-0.709006409	-1.120496805	4.44474718	11.12682891	0.011059465	0.038721963	PREDICTED: myosin-I heavy chain-like
RS005128	GO:0044699	single-organism process	BP	-0.986352968	-0.941562794	-0.824497555	5.605286461	11.12261317	0.011081004	0.038775816	PREDICTED: laminin subunit alpha
RS005275	GO:0044699	single-organism process	BP	0.581857125	-0.215495669	-0.358640095	4.487704057	11.11828097	0.011103181	0.038842628	PREDICTED: mismatch repair endonuclease PMS2
RS007388	GO:0044699	single-organism process	BP	-1.012601041	-0.502641916	-0.211964145	6.238906794	11.11536226	0.011118147	0.038884183	PREDICTED: V-type proton ATPase catalytic subunit A
RS015007	GO:0044699	single-organism process	BP	0.885187507	0.582553314	0.351169832	5.093595343	11.10985933	0.011146418	0.038961417	PREDICTED: CDK-activating kinase assembly factor MAT1
RS007155	GO:0044699	single-organism process	BP	-0.903016144	-1.587086013	-0.164756246	1.514002329	11.0922904	0.011237152	0.03925678	PREDICTED: neuroligin-4, Y-linked
RS008519	GO:0044699	single-organism process	BP	0.816487648	0.653005857	0.626787247	7.118148321	11.06710417	0.011368497	0.039671613	PREDICTED: rRNA 2'-O-methyltransferase fibrillarin
RS010385	GO:0044699	single-organism process	BP	0.463471266	0.71663601	0.037275502	4.562838647	11.0559059	0.011427381	0.039855007	PREDICTED: diphthamide biosynthesis protein 1
RS011176	GO:0044699	single-organism process	BP	0.473395066	0.419893985	-0.115628867	6.32671568	11.02928748	0.011568557	0.040291594	PREDICTED: serine/threonine-protein kinase RIO2
RS011555	GO:0044699	single-organism process	BP	-0.493021355	-0.206875073	-1.670148888	2.916126599	11.00502936	0.011698711	0.040704945	PREDICTED: L-lactate dehydrogenase-like
RS005798	GO:0044699	single-organism process	BP	0.42141247	0.736875665	0.248473058	5.783054674	10.99667203	0.011743885	0.040797551	PREDICTED: protein O-mannosyl-transferase 2
RS006740	GO:0044699	single-organism process	BP	-0.43064001	-0.612335737	-0.738849827	6.295225865	10.98541247	0.011805018	0.040967869	PREDICTED: glutamate-gated chloride channel
RS005985	GO:0044699	single-organism process	BP	-1.322196093	-1.78301114	-0.537217086	3.036385127	10.98323564	0.011816873	0.040986422	PREDICTED: retinoic acid receptor RXR-alpha-B
RS014019	GO:0044699	single-organism process	BP	-1.467988986	-0.712901687	-0.931718893	2.804647652	10.96588862	0.011911766	0.041281448	PREDICTED: rho GTPase-activating protein 26
RS004025	GO:0044699	single-organism process	BP	-1.263851415	-1.586868114	-0.216299788	1.435280902	10.96515279	0.011915808	0.041284094	PREDICTED: ubiquitin-conjugating enzyme E2 T
RS014286	GO:0044699	single-organism process	BP	-1.116813321	-0.446472365	-0.712367183	4.323236502	10.94398368	0.012032666	0.041654588	PREDICTED: sialin
RS012905	GO:0044699	single-organism process	BP	-0.815133052	-0.311118279	-0.474363818	5.281977326	10.93527671	0.012081057	0.041776175	PREDICTED: multiple inositol polyphosphate phosphatase 1-like
RS007836	GO:0044699	single-organism process	BP	-0.670404831	1.05713246	-0.759263575	1.495067002	10.91840834	0.012175354	0.042079146	PREDICTED: bestrophin-4
RS004697	GO:0044699	single-organism process	BP	0.790201286	0.5264892	0.024119998	4.515727811	10.8956717	0.012303604	0.042487412	PREDICTED: ras-related protein Ral-a
RS014419	GO:0044699	single-organism process	BP	-0.812097852	-0.035393336	0.555610255	3.206085641	10.88864079	0.012343532	0.042590258	PREDICTED: alpha-1,6-mannosyl-glycoprotein 2-beta-N-acetylglucosaminyltransferase
RS007445	GO:0044699	single-organism process	BP	0.077554822	-0.548800395	-0.821734397	6.266486593	10.87094209	0.012444608	0.042915495	PREDICTED: lysosomal alpha-mannosidase
RS000123	GO:0044699	single-organism process	BP	0.692109916	0.810423229	0.325162475	7.0801236	10.8417935	0.012612853	0.04340062	PREDICTED: ATP-binding cassette sub-family F member 3
RS011600	GO:0044699	single-organism process	BP	0.005589325	-0.389349094	-0.731140975	5.193526696	10.83468742	0.012654208	0.04351914	PREDICTED: protein arginine N-methyltransferase 5
RS013389	GO:0044699	single-organism process	BP	-0.224647569	-0.44483051	0.622610016	5.896195007	10.82662015	0.012701318	0.043633497	PREDICTED: propionyl-CoA carboxylase alpha chain, mitochondrial
RS007571	GO:0044699	single-organism process	BP	0.913385032	-0.678933443	-0.78278962	1.033669734	10.81624565	0.012762156	0.043818592	PREDICTED: E3 ubiquitin-protein ligase UHRF1
RS011127	GO:0044699	single-organism process	BP	0.510801033	0.668255039	0.469000295	5.728845819	10.81526531	0.01276792	0.043826433	PREDICTED: ATP-binding cassette sub-family F member 2
RS011903	GO:0044699	single-organism process	BP	0.432407009	-0.458087969	-0.635284859	4.879343122	10.81435958	0.012773247	0.043832773	PREDICTED: CAD protein
RS012898	GO:0044699	single-organism process	BP	-0.529595109	-0.13068788	-0.976420386	5.311956508	10.81357589	0.012777859	0.043836653	PREDICTED: GTPase-activating protein CdGAPr
RS014259	GO:0044699	single-organism process	BP	-0.353117713	-1.404057398	-0.722855844	3.450892887	10.80902748	0.012804654	0.043916617	PREDICTED: caspase-1-like
RS010927	GO:0044699	single-organism process	BP	0.992769091	0.708372335	0.399922008	3.940909255	10.80220798	0.012844933	0.044042768	PREDICTED: ribonuclease H2 subunit A
RS014452	GO:0044699	single-organism process	BP	-1.602073465	-1.251692781	-1.769543689	0.985867301	10.79983575	0.012858974	0.04407891	PREDICTED: E3 ubiquitin-protein ligase SIAH1-like
RS006456	GO:0044699	single-organism process	BP	-0.783553697	-0.497624014	-0.801985163	4.688121476	10.77538946	0.013004549	0.044517339	PREDICTED: kinesin-like protein KIF13B
RS014586	GO:0044699	single-organism process	BP	-0.877324504	-0.868386378	-1.448297031	1.926393117	10.77189768	0.013025474	0.044540544	bride of sevenless
RS005245	GO:0044699	single-organism process	BP	0.43477401	-0.090232398	-0.271084535	5.858068656	10.77028504	0.01303515	0.044549437	PREDICTED: histone H2A.V
RS001731	GO:0044699	single-organism process	BP	0.142527026	-0.406357233	-0.531526792	4.89952821	10.76460748	0.013069269	0.044641817	PREDICTED: rac GTPase-activating protein 1-like
RS013598	GO:0044699	single-organism process	BP	0.512037549	0.631132246	0.94499001	7.210942389	10.75511349	0.013126521	0.044813068	PREDICTED: mcKusick-Kaufman/Bardet-Biedl syndromes putative chaperonin
RS002816	GO:0044699	single-organism process	BP	-0.748548229	0.030574886	-0.160819942	5.861566925	10.7303973	0.013276729	0.045264516	PREDICTED: multidrug resistance protein 1-like
RS013048	GO:0044699	single-organism process	BP	0.717862274	0.808293829	0.95039709	8.4203493	10.71635095	0.013362846	0.045521147	PREDICTED: bifunctional glutamate/proline--tRNA ligase
RS014624	GO:0044699	single-organism process	BP	-0.324041413	-0.901957414	-1.083684327	4.835018422	10.71534176	0.013369054	0.045529981	PREDICTED: probable cytochrome P450 304a1
RS012007	GO:0044699	single-organism process	BP	-0.8805953	0.446042174	-0.616249485	2.603532973	10.7020781	0.013450915	0.045771636	PREDICTED: sodium-dependent phosphate transporter 2
RS013256	GO:0044699	single-organism process	BP	-2.118263679	-1.029697671	-1.151968074	0.484712211	10.70124469	0.013456075	0.045776826	PREDICTED: alpha-2A adrenergic receptor
RS002237	GO:0044699	single-organism process	BP	-0.77443485	-0.782975074	-0.864409769	3.087700691	10.69522479	0.013493406	0.045891426	PREDICTED: transient receptor potential cation channel protein painless
RS014643	GO:0044699	single-organism process	BP	0.254581862	0.726345297	-0.48680769	3.008929688	10.68030533	0.013586364	0.046182636	PREDICTED: monocarboxylate transporter 12-like
RS006300	GO:0044699	single-organism process	BP	-0.511538755	-0.90580939	-0.346492661	4.099933328	10.62089422	0.013962833	0.04732183	PREDICTED: pyridoxine-5'-phosphate oxidase
RS008390	GO:0044699	single-organism process	BP	0.086808415	1.001303883	0.637061954	4.646844201	10.60922931	0.014037947	0.047525244	PREDICTED: putative polypeptide N-acetylgalactosaminyltransferase 9
RS004674	GO:0044699	single-organism process	BP	-0.134689749	0.146434239	1.045058219	5.894682998	10.59658774	0.0141198	0.04773819	PREDICTED: nuclear hormone receptor FTZ-F1 beta
RS008660	GO:0044699	single-organism process	BP	-0.610580371	-0.844004978	-0.632634161	7.281264035	10.5950421	0.01412984	0.047746499	PREDICTED: glycogen debranching enzyme
RS010463	GO:0044699	single-organism process	BP	-0.857124822	-0.198171941	0.157726924	3.31811352	10.57466098	0.014262888	0.048114201	PREDICTED: carbohydrate sulfotransferase 11
RS000727	GO:0044699	single-organism process	BP	0.187570541	0.166933639	0.666638121	5.613818637	10.57499723	0.014260683	0.048114201	PREDICTED: delta-aminolevulinic acid dehydratase
RS010501	GO:0044699	single-organism process	BP	-0.249581193	0.164542324	0.830736506	8.23731792	10.57427827	0.014265398	0.048114201	PREDICTED: ATP synthase subunit e, mitochondrial
RS004883	GO:0044699	single-organism process	BP	-0.29441679	0.069643558	0.453675773	5.141081662	10.57019709	0.014292193	0.048178769	PREDICTED: phosphatidylinositol 4-phosphate 5-kinase type-1 alpha-like
RS002726	GO:0044699	single-organism process	BP	0.463459962	0.70932778	0.691403342	6.3457007	10.53629476	0.014516699	0.048844059	PREDICTED: ragulator complex protein LAMTOR5 homolog
RS009823	GO:0044699	single-organism process	BP	-1.238124462	-1.581039379	-1.214320102	3.555672218	10.51169425	0.014681778	0.04934676	PREDICTED: gastrin-releasing peptide receptor-like
RS014520	GO:0044699	single-organism process	BP	0.368825623	-0.038875833	-0.392085998	5.571469339	10.50878035	0.014701453	0.04938653	PREDICTED: retinol dehydrogenase 11-like
RS013211	GO:0044699	single-organism process	BP	-0.46592983	-1.425136997	0.127678346	1.648820506	10.50692227	0.014714013	0.049415541	PREDICTED: pickpocket protein 19-like
RS001709	GO:0044699	single-organism process	BP	0.777840332	0.406931537	0.261860802	4.756023495	10.48782829	0.014843693	0.049758175	PREDICTED: MRG/MORF4L-binding protein

RS005829	GO:0044699	single-organism process	BP	-0.565901365	-0.398114437	0.075675202	5.880326068	10.47839461	0.014908177	0.04996104	PREDICTED: facilitated trehalose transporter Tret1-like
RS012348	GO:0044699	single-organism process	BP	-1.171136152	-0.623448827	-0.241561177	4.399276235	10.47489738	0.014932153	0.050028076	V-type proton ATPase subunit S1
RS004574	GO:0044699	single-organism process	BP	-0.256414135	0.160648707	0.765161971	8.179741228	10.46991438	0.01496638	0.050122195	PREDICTED: ATP synthase subunit delta, mitochondrial
RS015148	GO:0044699	single-organism process	BP	-1.963276978	0.635742593	-0.155791267	2.122363384	10.45700384	0.01505542	0.050360678	PREDICTED: L-threonine 3-dehydrogenase, mitochondrial
RS009559	GO:0044699	single-organism process	BP	-1.102537153	-0.013791304	0.087900083	4.220199202	10.45541365	0.015066422	0.050384101	PREDICTED: cytochrome b561-like
RS003249	GO:0044699	single-organism process	BP	-0.207283219	-0.369134294	-0.971045776	4.452945458	10.45254875	0.015086265	0.05039693	PREDICTED: protein O-linked-mannose beta-1,2-N-acetylglucosaminyltransferase 1
RS000485	GO:0044699	single-organism process	BP	-0.695391551	-0.618652868	-0.739307552	7.487588188	10.45272332	0.015085056	0.05039693	PREDICTED: transaldolase
RS005224	GO:0044699	single-organism process	BP	0.634008216	0.005237384	-0.014917096	6.79959133	10.44569532	0.015133838	0.050532387	PREDICTED: importin subunit alpha
RS011984	GO:0044699	single-organism process	BP	-0.529428927	-1.115110606	-0.420809217	4.865160658	10.42681322	0.015265673	0.050901744	PREDICTED: synaptic vesicle membrane protein VAT-1 homolog
RS005209	GO:0044699	single-organism process	BP	-0.863563851	-1.023827568	-0.616859801	5.79782502	10.40323263	0.015431901	0.051374401	PREDICTED: multidrug resistance protein homolog 49
RS003304	GO:0044699	single-organism process	BP	-1.31453369	-1.211089405	-1.381955811	3.144933806	10.40242865	0.0154376	0.051379791	PREDICTED: ion transport peptide-like
RS005888	GO:0044699	single-organism process	BP	0.888406764	0.376409719	0.484352959	6.270344694	10.4000221	0.01545467	0.051423017	PREDICTED: H/ACA ribonucleoprotein complex subunit 4
RS014917	GO:0044699	single-organism process	BP	0.400078534	-1.067678011	-0.179731157	3.962692977	10.39566749	0.015485607	0.051499818	PREDICTED: serine/threonine-protein kinase Nek1
RS012563	GO:0044699	single-organism process	BP	-0.988721616	-1.629093038	-1.547898297	1.395248421	10.37792036	0.015612318	0.051865119	PREDICTED: polycomb complex protein BMI-1
RS012105	GO:0044699	single-organism process	BP	-0.587037702	-0.604268783	0.220956679	3.910659327	10.37503836	0.015632991	0.051906642	Inward rectifier potassium channel 2
RS000868	GO:0044699	single-organism process	BP	-0.929392451	-0.475108864	-0.289097862	8.324170965	10.36013171	0.01574035	0.052235565	PREDICTED: sodium/potassium-transporting ATPase subunit alpha
RS000328	GO:0044699	single-organism process	BP	-0.579267699	-0.762242335	0.061515272	4.205756282	10.34334596	0.015862111	0.052611605	PREDICTED: farnesyl pyrophosphate synthase
RS000682	GO:0044699	single-organism process	BP	-0.126315522	0.259410143	0.757618967	7.322018397	10.33657632	0.015911478	0.052728055	PREDICTED: NADH dehydrogenase [ubiquinone] 1 beta subcomplex subunit 7
RS014648	GO:0044699	single-organism process	BP	-1.552586697	-1.42084672	-1.27626509	1.788880832	10.33267874	0.01593997	0.052786742	PREDICTED: synaptotagmin-7
RS005239	GO:0044699	single-organism process	BP	-0.442689839	-0.720832595	-0.092125906	4.79919063	10.32683206	0.015982804	0.05290077	PREDICTED: vesicular acetylcholine transporter
RS010341	GO:0044699	single-organism process	BP	0.724454995	0.510479084	0.009111094	5.152584391	10.32038846	0.016030142	0.053043512	PREDICTED: transcription initiation protein SPT3 homolog
RS000355	GO:0044699	single-organism process	BP	0.186905278	-0.53481783	-0.634700084	3.813658966	10.30949379	0.016110494	0.053295392	PREDICTED: ras GTPase-activating-like protein IQGAP1
RS014260	GO:0044699	single-organism process	BP	0.847652686	-0.135314424	0.806100051	5.052649555	10.30574059	0.016138267	0.053359236	PREDICTED: polypeptide N-acetylgalactosaminyltransferase 3
RS006468	GO:0044699	single-organism process	BP	-0.231878205	-0.728845307	-0.532238669	5.359397877	10.30159738	0.01616898	0.053446755	PREDICTED: dynactin subunit 1
RS005511	GO:0044699	single-organism process	BP	0.600916857	-0.522438809	-0.062691298	3.783949071	10.28751405	0.01627381	0.053757033	PREDICTED: protein dispatched
RS003096	GO:0044699	single-organism process	BP	0.221396945	0.218154194	0.698843797	5.992034129	10.28029031	0.01632784	0.053915263	PREDICTED: quinone oxidoreductase-like protein 2
RS011355	GO:0044699	single-organism process	BP	0.253608749	0.879943744	0.418994898	5.031456652	10.27103637	0.016397312	0.054102107	PREDICTED: deoxynucleoside triphosphate triphosphohydrolase SAMHD1
RS004470	GO:0044699	single-organism process	BP	0.543979307	-0.185028907	-0.226883369	4.441356704	10.25789431	0.016496473	0.054358079	PREDICTED: meiosis-specific nuclear structural protein 1
RS012239	GO:0044699	single-organism process	BP	0.482459701	0.838142166	1.02225669	9.155423628	10.25350664	0.01652971	0.054453354	PREDICTED: succinyl-CoA ligase [ADP/GDP-forming] subunit alpha, mitochondrial
RS008445	GO:0044699	single-organism process	BP	-0.794376386	-2.696738816	-1.11925367	1.261432481	10.25254337	0.016537016	0.054463175	PREDICTED: facilitated trehalose transporter Tret1-2 homolog
RS009128	GO:0044699	single-organism process	BP	0.602850609	0.618849101	0.428865547	6.715423229	10.23923324	0.016638291	0.054710872	PREDICTED: LDLR chaperone boca
RS001157	GO:0044699	single-organism process	BP	0.896529095	0.226411579	-0.658824297	2.329234103	10.23735633	0.016652621	0.054729413	PREDICTED: fork head domain-containing protein FD4-like
RS014543	GO:0044699	single-organism process	BP	0.157306782	0.31225489	0.813449271	5.130563443	10.22788653	0.016725108	0.054910326	PREDICTED: cytochrome b5 reductase 4
RS005019	GO:0044699	single-organism process	BP	-0.683071201	-0.821364423	-0.163028794	3.587633592	10.22051853	0.016781721	0.055038801	PREDICTED: serine/threonine-protein kinase NIM1
RS010025	GO:0044699	single-organism process	BP	-0.732876867	-0.934197135	-0.624089721	4.91488863	10.21589436	0.016817348	0.055126934	PREDICTED: protein kinase C
RS011227	GO:0044699	single-organism process	BP	0.334619246	0.843464653	0.334391141	4.925842673	10.20266648	0.016919673	0.055419079	hypothetical protein
RS002767	GO:0044699	single-organism process	BP	-0.220649299	-0.818802321	-1.153514956	3.513282722	10.19354792	0.016990566	0.055636815	PREDICTED: 2-phosphoxylose phosphatase 1
RS004192	GO:0044699	single-organism process	BP	-0.780718028	-0.778170358	-0.860196259	4.007314836	10.19014864	0.017017069	0.055709115	PREDICTED: myosin-3-like
RS012944	GO:0044699	single-organism process	BP	-0.51867479	-0.880358905	-1.034452936	4.443229138	10.14864263	0.017343972	0.056599143	PREDICTED: isopentenyl-diphosphate Delta-isomerase 1
RS008079	GO:0044699	single-organism process	BP	-1.109710923	-0.938014135	-0.296800028	2.900944855	10.13930135	0.017418391	0.056816166	PREDICTED: potassium voltage-gated channel protein Shaker
RS004798	GO:0044699	single-organism process	BP	0.527491496	0.725225005	0.274836226	6.810708913	10.13566285	0.017447463	0.056894838	PREDICTED: polyprenol reductase
RS008621	GO:0044699	single-organism process	BP	0.693704268	0.750272119	0.010010283	5.172186846	10.13274418	0.017470817	0.056929316	PREDICTED: katanin p60 ATPase-containing subunit A1
RS000303	GO:0044699	single-organism process	BP	-0.686213864	-0.317192717	0.380423788	4.708375082	10.12045274	0.01756951	0.057190593	PREDICTED: vitamin K epoxide reductase complex subunit 1-like protein 1
RS010405	GO:0044699	single-organism process	BP	0.474393779	0.873037775	0.408572486	5.968952056	10.10643309	0.017682747	0.057499747	PREDICTED: aprataxin
RS013466	GO:0044699	single-organism process	BP	-0.500235206	-0.734799003	-0.5747966	5.583326396	10.1065781	0.017681572	0.057499747	PREDICTED: retinal guanylyl cyclase 2
RS009153	GO:0044699	single-organism process	BP	0.613293967	0.998084897	0.966219795	3.330543492	10.10124836	0.017724806	0.057621634	PREDICTED: mitochondrial import inner membrane translocase subunit Tim9
RS004081	GO:0044699	single-organism process	BP	1.119342481	0.133579247	-0.12061345	1.599096738	10.09767322	0.017753865	0.057701208	PREDICTED: mitotic spindle assembly checkpoint protein MAD2A
RS005196	GO:0044699	single-organism process	BP	-0.62031121	-0.946710459	0.084812792	3.467267912	10.09431996	0.017781164	0.057765203	PREDICTED: hexokinase-1-like
RS003950	GO:0044699	single-organism process	BP	-0.587671115	-0.868990799	-0.714405964	4.844171479	10.0941278	0.017782729	0.057765203	PREDICTED: cationic amino acid transporter 2
RS008955	GO:0044699	single-organism process	BP	0.403724441	0.637447413	0.728368892	5.510987945	10.08992696	0.017816989	0.057861566	PREDICTED: phosphopantothenate--cysteine ligase
RS001463	GO:0044699	single-organism process	BP	-0.705115576	-0.592987043	-0.53974017	6.388106555	10.08445396	0.01786172	0.057947065	hypothetical protein
RS003251	GO:0044699	single-organism process	BP	-0.926007043	-0.972699255	-0.243788129	4.618803471	10.07731546	0.01792023	0.058121911	PREDICTED: alkyldihydroxyacetonephosphate synthase
RS003654	GO:0044699	single-organism process	BP	0.687784934	0.169974473	0.001761567	7.865910242	10.07014476	0.017979194	0.058253143	PREDICTED: craniofacial development protein 2-like
RS001239	GO:0044699	single-organism process	BP	0.958677384	0.665870745	0.196846134	4.037018866	10.0385642	0.018241154	0.058965373	PREDICTED: zinc finger protein 706-like
RS010825	GO:0044699	single-organism process	BP	-0.11178442	-0.611021406	-0.795698296	5.293267172	10.03266739	0.018290482	0.059109656	PREDICTED: fructose-1,6-bisphosphatase 1
RS005813	GO:0044699	single-organism process	BP	-0.028906362	-0.855787459	-0.320403172	4.418608219	10.02713591	0.018336873	0.059229182	PREDICTED: hemicentin-2
RS005238	GO:0044699	single-organism process	BP	-1.154648901	-1.302063311	-0.817071902	3.097380454	10.02250762	0.018375778	0.059324417	PREDICTED: choline O-acetyltransferase
RS005883	GO:0044699	single-organism process	BP	0.579677196	0.662723942	0.941061143	6.190916775	10.02257363	0.018375223	0.059324417	PREDICTED: mitochondrial import inner membrane translocase subunit Tim10
RS008876	GO:0044699	single-organism process	BP	-0.044076832	-0.679757915	-1.127823688	2.636096118	10.01713287	0.01842106	0.059455363	PREDICTED: DNA replication licensing factor Mcm5
RS004124	GO:0044699	single-organism process	BP	-0.61907943	-0.690409053	-0.007043237	5.012696694	9.991139429	0.018641604	0.060074811	PREDICTED: probable trans-2-enoyl-CoA reductase, mitochondrial
RS009706	GO:0044699	single-organism process	BP	0.008362474	-0.509311807	-0.5812572	6.894368711	9.981766648	0.018721763	0.060286854	PREDICTED: structural maintenance of chromosomes protein 1A
RS005779	GO:0044699	single-organism process	BP	0.112656963	-0.512247284	-0.571575845	5.471924331	9.976469478	0.018767216	0.060386898	PREDICTED: probable histone-binding protein Caf1
RS001404	GO:0044699	single-organism process	BP	-0.441742771	-1.044901466	0.066385313	2.875083466	9.975183883	0.018778264	0.060407012	AGAP002511-PA-like protein
RS000722	GO:0044699	single-organism process	BP	0.655146436	0.259480921	-0.435617731	8.518476501	9.973943049	0.018788933	0.060425898	PREDICTED: heat shock 70 kDa protein cognate 3
RS002591	GO:0044699	single-organism process	BP	-0.862816032	-0.632941949	0.029415913	8.699755638	9.964280052	0.018872221	0.060647296	PREDICTED: succinate dehydrogenase [ubiquinone] flavoprotein subunit, mitochondrial
RS014544	GO:0044699	single-organism process	BP	-0.492312404	-0.805748281	-0.168707308	4.565520148	9.9569744	0.018935432	0.06081939	PREDICTED: monocarboxylate transporter 10-like
RS005900	GO:0044699	single-organism process	BP	-1.106268929	-1.125208311	-0.429605979	3.784570883	9.955753683	0.018946014	0.060837864	PREDICTED: protein 60A
RS002232	GO:0044699	single-organism process	BP	0.119289335	0.50794716	0.803561551	7.6433151	9.952596024	0.018973414	0.060879282	PREDICTED: NADH dehydrogenase [ubiquinone] 1 beta subcomplex subunit 9
RS009482	GO:0044699	single-organism process	BP	0.656409358	0.589041006	-0.674416821	2.174541161	9.949687744	0.018998685	0.060928106	PREDICTED: FMRFamide receptor-like
RS008027	GO:0044699	single-organism process	BP	0.628402022	0.552311615	0.584221727	6.50145483	9.949174963	0.019003144	0.060928106	PREDICTED: 39S ribosomal protein L27, mitochondrial
RS006180	GO:0044699	single-organism process	BP	0.307870402	0.596768941	0.701851598	6.457677105	9.950085895	0.018995224	0.060928106	PREDICTED: OCIA domain-containing protein 1
RS013196	GO:0044699	single-organism process	BP	-0.742787586	-0.82296517	-0.584288713	4.338589721	9.929359295	0.019176251	0.061420575	PREDICTED: adenylate cyclase type 9
RS000167	GO:0044699	single-organism process	BP	-1.102687093	0.106083975	0.527503968	2.917063029	9.929771479	0.019172634	0.061420575	PREDICTED: probable WRKY transcription factor protein 1
RS004049	GO:0044699	single-organism process	BP	0.140062401	0.173125772	-0.953882435	2.844517803	9.917563627	0.01928003	0.061643235	PREDICTED: ras-related protein Rab-27A
RS004269	GO:0044699	single-organism process	BP	0.264997402	0.496560686	0.739100443	6.92568149	9.917628354	0.019279459	0.061643235	PREDICTED: signal recognition particle 19 kDa protein
RS003532	GO:0044699	single-organism process	BP	0.639024623	0.539875333	0.278180553	6.230710506	9.913650076	0.019314584	0.061722373	PREDICTED: arginine--tRNA ligase, cytoplasmic
RS008729	GO:0044699	single-organism process	BP	0.336616977	0.599284474	0.715731577	7.669808717	9.906724803	0.019375878	0.06188684	PREDICTED: leucine-rich PPR motif-containing protein, mitochondrial
RS002778	GO:0044699	single-organism process	BP	0.683580252	0.576127296	0.643441611	5.379786914	9.904304363	0.019397346	0.061939699	PREDICTED: N-alpha-acetyltransferase 40
RS012242	GO:0044699	single-organism process	BP	-0.364304586	-0.900139498	-0.18093339	4.065360265	9.89725823	0.019459974	0.062123932	PREDICTED: cardioactive peptide
RS004575	GO:0044699	single-organism process	BP	-0.352734321	0.032623146	0.48704132	4.732722518	9.882231696	0.019594198	0.062460406	PREDICTED: sialin
RS002067	GO:0044699	single-organism process	BP	0.818239453	0.645181246	0.817422465	6.592031154	9.882127771	0.01959513	0.062460406	PREDICTED: ribonuclease P protein subunit p30
RS012602	GO:0044699	single-organism process	BP	0.506574671	0.594015734	0.65612311	6.018722653	9.872779785	0.019679093	0.06271217	PREDICTED: thioredoxin-like protein 4A
RS013771	GO:0044699	single-organism process	BP	-0.724487617	-1.407505507	-0.968416348	7.909349388	9.865437308	0.019745291	0.062875396	PREDICTED: putative ferric-chelate reductase 1 homolog
RS007233	GO:0044699	single-organism process	BP	-0.66254078	-0.44136293	-0.524369626	5.805626877	9.86301746	0.019767155	0.062929108	hypothetical protein
RS004406	GO:0044699	single-organism process	BP	0.125398184	0.827131322	0.610658506	4.59644428	9.85991184	0.019795251	0.063002625	PREDICTED: ADP-ribosylation factor-like protein 16
RS004428	GO:0044699	single-organism process	BP	-0.678036321	-1.656818353	-0.858035526	3.622037903	9.858029749	0.019812297	0.063040946	PREDICTED: cytochrome P450 4g15
RS002739	GO:0044699	single-organism process	BP	0.211694114	0.16146795	0.629767437	5.898527948	9.844331169	0.019936798	0.063405059	PREDICTED: UDP-xylose and UDP-N-acetylglucosamine transporter
RS005636	GO:0044699	single-organism process	BP	-0.453071987	-0.038123733	0.527166131	7.855258218	9.838636626	0.019988779	0.063503274	PREDICTED: dihydrolipoyl dehydrogenase, mitochondrial
RS014176	GO:0044699	single-organism process	BP	0.177224614	-0.223975103	-0.611807087	4.673089603	9.837440203	0.019999717	0.063508936	PREDICTED: histone acetyltransferase type B catalytic subunit
RS009608	GO:0044699	single-organism process	BP	-0.650681402	-0.131877234	-0.002301287	6.339415917	9.831393766	0.020055087	0.063636624	PREDICTED: V-type proton ATPase subunit C
RS004728	GO:0044699	single-organism process	BP	-0.39010325	-0.518105196	0.186521429	5.063484231	9.819942673	0.020160361	0.063897618	PREDICTED: adenylate cyclase type 2
RS002046	GO:0044699	single-organism process	BP	-1.040945999	-1.141609481	-1.378037143	3.132734296	9.81352734	0.020219576	0.06404561	PREDICTED: sodium- and chloride-dependent GABA transporter 1-like
RS005643	GO:0044699	single-organism process	BP	0.001628945	-0.599904596	-0.980408504	3.651885374	9.812499581	0.020229079	0.064047956	PREDICTED: alpha-crystallin A chain-like
RS014760	GO:0044699	single-organism process	BP	0.346858397	0.752518643	0.342834138	5.774843462	9.812347181	0.020230488	0.064047956	PREDICTED: retinoblastoma-binding protein 5 homolog
RS009315	GO:0044699	single-organism process	BP	0.557410134	0.146594931	-0.05792173	5.618545668	9.808484282	0.020266245	0.064145035	PREDICTED: cullin-5
RS000267	GO:0044699	single-organism process	BP	-0.975049617	-1.281753931	-0.054139098	2.075306639	9.804681806	0.020301504	0.064240487	PREDICTED: argininosuccinate lyase
RS015026	GO:0044699	single-organism process	BP	-2.067999384	-1.445287582	-0.623763088	0.948319182	9.800718222	0.02033832	0.06434082	PREDICTED: hepatocyte nuclear factor 4-gamma
RS011326	GO:0044699	single-organism process	BP	-3.559882472	-1.675919738	-0.069245797	1.058615186	9.796058365	0.020381688	0.064428604	PREDICTED: putative fatty acyl-CoA reductase CG5065
RS003051	GO:0044699	single-organism process	BP	-0.064961478	1.836288981	0.63869261	0.362805235	9.79580548	0.020384044	0.064428604	PREDICTED: NADPH oxidase 5
RS001413	GO:0044699	single-organism process	BP	-0.248212965	0.06615521	0.706495165	7.98044758	9.766834252	0.020655746	0.065132313	PREDICTED: cytochrome b-c1 complex subunit 7-like
RS009910	GO:0044699	single-organism process	BP	0.313194263	0.730742635	0.275971395	4.823306289	9.760044134	0.020719938	0.065318373	PREDICTED: ADP-ribosylation factor-like protein 5B
RS008926	GO:0044699	single-organism process	BP	-1.534010989	-0.385501145	-0.729597294	1.610423423	9.752885364	0.020787828	0.065499602	PREDICTED: octopamine receptor beta-2R-like
RS005470	GO:0044699	single-organism process	BP	-0.591341731	-1.24800673	-0.982044765	2.918949317	9.727285999	0.021032391	0.066229478	PREDICTED: slit homolog 2 protein
RS012906	GO:0044699	single-organism process	BP	-0.77406038	0.16023721	0.255652978	3.857520339	9.726166779	0.021043148	0.066229478	Multiple inositol polyphosphate phosphatase 1
RS007419	GO:0044699	single-organism process	BP	-0.810509574	-0.496169472	0.271583652	8.964283994	9.715009245	0.021150677	0.066476571	PREDICTED: probable isocitrate dehydrogenase [NAD] subunit alpha, mitochondrial
RS008813	GO:0044699	single-organism process	BP	-0.966072111	-1.905422688	0.196893498	0.740833766	9.694980284	0.021345058	0.067027185	FERM domain (protein4.1-ezrin-radixin-moesin) family
RS015433	GO:0044699	single-organism process	BP	-1.023491195	-0.871650733	-0.547044844	4.320060789	9.694765434	0.021347153	0.067027185	PREDICTED: glutamate receptor ionotropic, kainate 2
RS001721	GO:0044699	single-organism process	BP	0.082556812	-0.281594977	-0.73498889	4.716641345	9.695756603	0.021337491	0.067027185	PREDICTED: 5-oxoprolinase
RS011957	GO:0044699	single-organism process	BP	-0.480515765	-2.279558072	-0.771986192	1.619506529	9.693257754	0.021361857	0.067056637	PREDICTED: probable cytochrome P450 CYP44
RS011557	GO:0044699	single-organism process	BP	0.238740865	1.416257966	1.208471941	1.630049229	9.691416693	0.021379826	0.067096319	Cubilin
RS000129	GO:0044699	single-organism process	BP	0.575945381	0.333253167	-0.128471307	6.41956033	9.680605885	0.021485642	0.067344493	PREDICTED: ruvB-like 1

RS001208	GO:0044699	single-organism process	BP	-0.016389681	0.720095866	0.500772791	6.241378143	9.673849648	0.021552033	0.067527851	PREDICTED: GSK-3-binding protein-like
RS013502	GO:0044699	single-organism process	BP	-1.39922311	-0.803927126	-1.016110582	2.326956106	9.673561923	0.021554864	0.067527851	PREDICTED: single Ig IL-1-related receptor-like
RS002533	GO:0044699	single-organism process	BP	0.598821326	0.052547223	0.359146454	5.76070108	9.639508094	0.021892619	0.068449762	PREDICTED: sepiapterin reductase-like
RS012325	GO:0044699	single-organism process	BP	-0.610322555	-0.73163567	0.587302153	2.890049632	9.633930261	0.021948434	0.068590218	PREDICTED: serine/threonine-protein phosphatase 6 regulatory ankyrin repeat subunit A
RS002956	GO:0044699	single-organism process	BP	-2.928056308	-0.897843684	-0.292887679	1.412347179	9.602623126	0.022264315	0.069370802	PREDICTED: glucose dehydrogenase [FAD, quinone]-like
RS012064	GO:0044699	single-organism process	BP	0.420784618	0.720623592	0.420429533	7.093139482	9.589511139	0.022397933	0.069752614	PREDICTED: signal recognition particle subunit SRP68
RS010393	GO:0044699	single-organism process	BP	-1.177557798	-0.645121351	-1.493993137	3.341621176	9.557412969	0.022728362	0.070572236	PREDICTED: purine nucleoside phosphorylase-like
RS013348	GO:0044699	single-organism process	BP	-0.376569369	-0.677221706	-0.537214255	5.144895409	9.550689575	0.022798178	0.070771569	PREDICTED: guanine nucleotide-releasing factor 2
RS000425	GO:0044699	single-organism process	BP	-0.489453497	0.45298053	0.550696036	3.881523413	9.546181524	0.022845108	0.070899775	PREDICTED: RNA-binding protein Musashi homolog Rbp6
RS003296	GO:0044699	single-organism process	BP	-0.446533967	-0.429432028	0.180148858	6.98986577	9.535872508	0.022952784	0.0711638	PREDICTED: dual specificity protein phosphatase 3-like
RS013899	GO:0044699	single-organism process	BP	-0.041892099	0.289722966	0.753875941	7.322200491	9.516745919	0.023153878	0.071751953	PREDICTED: NADH dehydrogenase [ubiquinone] iron-sulfur protein 4, mitochondrial
RS003938	GO:0044699	single-organism process	BP	-0.544460525	-0.47253041	-0.796253481	4.546392138	9.512451754	0.023199263	0.071874912	PREDICTED: adenylate cyclase type 6
RS006691	GO:0044699	single-organism process	BP	-0.046408564	0.042237177	0.543267145	5.501535519	9.506155456	0.023265967	0.072028413	PREDICTED: ankyrin-3
RS004401	GO:0044699	single-organism process	BP	0.352119513	0.452013829	0.772165286	6.786052696	9.503035986	0.023299085	0.072092718	PREDICTED: DNA repair protein XRCC4-like
RS013499	GO:0044699	single-organism process	BP	-0.283648325	-0.605727083	-0.940470074	3.563352368	9.501690017	0.023313388	0.072104325	PREDICTED: regulating synaptic membrane exocytosis protein 2
RS001773	GO:0044699	single-organism process	BP	-1.179645502	-1.391479741	-1.203064835	2.188248107	9.497351264	0.023359555	0.072211644	PREDICTED: acetylcholine receptor subunit alpha-like
RS002606	GO:0044699	single-organism process	BP	0.727776854	0.044705319	0.194370573	5.767585194	9.496670082	0.023366812	0.07221635	PREDICTED: actin-related protein 5
RS012801	GO:0044699	single-organism process	BP	-0.524225323	0.265871902	-0.897489264	2.617033793	9.476085022	0.023587141	0.072796642	PREDICTED: trimethyllysine dioxygenase, mitochondrial
RS003696	GO:0044699	single-organism process	BP	-0.832828581	-0.854637452	-2.229427046	0.666392921	9.465733793	0.023698703	0.07302703	PREDICTED: microcephalin
RS001282	GO:0044699	single-organism process	BP	0.417935678	0.544644884	0.732222098	5.614550128	9.453370051	0.023832634	0.073403814	PREDICTED: retinol dehydrogenase 13-like
RS015576	GO:0044699	single-organism process	BP	-0.604832605	-0.776770217	-0.354955504	5.578036943	9.446482694	0.023907563	0.073612854	PREDICTED: PI-PLC X domain-containing protein 1-like
RS015367	GO:0044699	single-organism process	BP	0.208837877	0.169293528	1.730491115	0.595360665	9.444985213	0.023923885	0.073612854	PREDICTED: tubulin alpha-1C chain-like
RS000492	GO:0044699	single-organism process	BP	-0.350891295	-0.858661191	-0.877219269	3.396135103	9.445821042	0.023914774	0.073612854	PREDICTED: tyrosine-protein phosphatase 10D
RS006809	GO:0044699	single-organism process	BP	-0.069599294	0.161390813	-0.669189997	5.521140821	9.427681054	0.02411329	0.074118459	PREDICTED: sodium bicarbonate cotransporter 3
RS006843	GO:0044699	single-organism process	BP	0.072888437	-0.12337594	0.631562026	4.421322729	9.426131696	0.02413032	0.074139363	PREDICTED: biogenesis of lysosome-related organelles complex 1 subunit 5
RS011343	GO:0044699	single-organism process	BP	0.705971244	0.543114848	0.427844676	7.343583445	9.423971108	0.024154088	0.074194289	PREDICTED: UV excision repair protein RAD23 homolog B
RS015575	GO:0044699	single-organism process	BP	0.492225129	-0.602318287	-1.145138069	2.605181572	9.416013738	0.024241825	0.074409344	PREDICTED: protein notum homolog
RS001408	GO:0044699	single-organism process	BP	-0.807514723	-0.968606634	-0.441028126	4.690280881	9.412655047	0.024278951	0.074486992	PREDICTED: protein winged eye
RS003636	GO:0044699	single-organism process	BP	0.658899301	-0.027122995	0.775979951	5.812404377	9.412838955	0.024276916	0.074486992	PREDICTED: ATP-binding cassette sub-family G member 4
RS000109	GO:0044699	single-organism process	BP	1.11237011	0.292104085	0.413906997	2.41513452	9.404421786	0.024370195	0.074748717	PREDICTED: selenocysteine lyase
RS006751	GO:0044699	single-organism process	BP	0.318033216	0.612725646	0.706826078	5.088000425	9.400847536	0.024409911	0.074852304	PREDICTED: riboflavin kinase
RS005428	GO:0044699	single-organism process	BP	-0.062034998	-2.807856927	-2.085450138	0.079634062	9.400298956	0.024416012	0.074852788	PREDICTED: E3 ubiquitin-protein ligase SIAH1-like
RS006916	GO:0044699	single-organism process	BP	-0.687565061	-0.785032465	-0.460125521	4.499473968	9.393768985	0.024488752	0.075021004	PREDICTED: SRSF protein kinase 2
RS012938	GO:0044699	single-organism process	BP	-1.846939656	-0.327189547	0.081992913	1.097980344	9.391278764	0.024516548	0.075087891	PREDICTED: beta-1,3-galactosyltransferase 5
RS007719	GO:0044699	single-organism process	BP	-0.363554514	0.201823484	0.241238455	6.872179235	9.390306687	0.024527407	0.075102884	PREDICTED: V-type proton ATPase 21 kDa proteolipid subunit
RS001677	GO:0044699	single-organism process	BP	0.732018626	1.345208564	0.883061373	2.970019988	9.388133465	0.0245517	0.075158996	Formin-like protein 2
RS007215	GO:0044699	single-organism process	BP	-0.354778299	0.092886137	0.424760533	4.515646467	9.383572717	0.024602759	0.075278704	PREDICTED: PRADC1-like protein
RS011516	GO:0044699	single-organism process	BP	-0.473275427	-0.68290119	-0.690615739	5.161656614	9.379057986	0.024653405	0.075415347	PREDICTED: patronin
RS001656	GO:0044699	single-organism process	BP	-0.726450565	-0.798902297	1.105142878	1.008896657	9.378417658	0.024660597	0.075419027	PREDICTED: ankycorbin-like
RS001780	GO:0044699	single-organism process	BP	-0.83440823	-0.675360673	-0.062232735	3.12040852	9.377698714	0.024668674	0.075425412	CD63 antigen
RS005942	GO:0044699	single-organism process	BP	0.222003058	0.357500804	-0.707071584	4.173704825	9.375192754	0.024696847	0.075474905	PREDICTED: mitochondrial sodium/hydrogen exchanger 9B2-like
RS006823	GO:0044699	single-organism process	BP	-1.796015766	-1.90024873	-0.974318408	1.062644789	9.364491028	0.024817515	0.075788504	PREDICTED: prolactin-releasing peptide receptor-like
RS000408	GO:0044699	single-organism process	BP	-1.046267284	-0.87882332	-0.26104022	3.64779078	9.360881799	0.024858342	0.075821254	PREDICTED: G-protein coupled receptor Mth2-like
RS000146	GO:0044699	single-organism process	BP	0.201922556	-0.781716042	-0.183638845	5.004160987	9.356926226	0.024903162	0.075921187	PREDICTED: serine hydroxymethyltransferase, mitochondrial
RS005671	GO:0044699	single-organism process	BP	-1.126425574	-0.035686297	-1.017174804	1.852902018	9.333442544	0.025170888	0.076644625	PREDICTED: branched-chain-amino-acid aminotransferase, cytosolic
RS002099	GO:0044699	single-organism process	BP	0.616949357	0.296958619	0.122554602	5.735215826	9.329089072	0.025220829	0.076759576	PREDICTED: protein MIS12 homolog
RS005539	GO:0044699	single-organism process	BP	0.583175168	0.777154512	0.547995138	4.732854484	9.324473045	0.025273888	0.076883901	PREDICTED: ADP-ribosylation factor 6
RS011133	GO:0044699	single-organism process	BP	-0.331501428	-0.235298864	0.393756894	5.223707678	9.315845045	0.025373357	0.077090385	PREDICTED: ras-related protein Rab-18
RS007160	GO:0044699	single-organism process	BP	-0.549555066	-1.005296558	-0.449020655	6.369743433	9.315822987	0.025373612	0.077090385	cytoplasmic NADP+-dependent isocitrate dehydrogenase
RS004356	GO:0044699	single-organism process	BP	-0.203062517	-0.534836144	-0.655563335	5.538704348	9.289500759	0.02567945	0.077760757	PREDICTED: mitogen-activated protein kinase kinase kinase 12
RS003802	GO:0044699	single-organism process	BP	0.811082921	0.486372202	-0.08894724	2.933919036	9.26719754	0.025941417	0.078459703	PREDICTED: cGMP-dependent protein kinase, isozyme 1
RS005076	GO:0044699	single-organism process	BP	-1.115248473	-1.35071287	-3.252547736	0.628097357	9.266085702	0.025954544	0.07848056	PREDICTED: orexin receptor type 2-like
RS010223	GO:0044699	single-organism process	BP	0.60835214	0.559996282	0.648387849	7.460242383	9.245407714	0.026199883	0.079108445	PREDICTED: gustatory receptor for sugar taste 64f-like
RS004326	GO:0044699	single-organism process	BP	-0.320433456	0.005509373	0.528151824	7.246771849	9.242882358	0.026230001	0.079161426	PREDICTED: NADH dehydrogenase [ubiquinone] 1 alpha subcomplex subunit 8
RS009928	GO:0044699	single-organism process	BP	-0.835456878	-2.347674561	-0.849585016	1.053053669	9.23428246	0.02633282	0.079385129	PREDICTED: sodium-dependent noradrenaline transporter-like
RS008567	GO:0044699	single-organism process	BP	-0.395964157	0.055050505	0.329555756	6.356343245	9.231251867	0.026369147	0.079448096	PREDICTED: glycerol-3-phosphate dehydrogenase, mitochondrial
RS012839	GO:0044699	single-organism process	BP	0.765070034	0.767441076	0.269444249	4.770997415	9.223490228	0.026462409	0.079644425	PREDICTED: tyrosine--tRNA ligase, mitochondrial
RS000170	GO:0044699	single-organism process	BP	-0.544657161	0.096205811	-0.031088519	7.09217039	9.202880768	0.026711619	0.080307026	PREDICTED: probable Ras GTPase-activating protein
RS003234	GO:0044699	single-organism process	BP	-0.402942012	0.351806881	0.253791792	7.730694199	9.198914238	0.026759846	0.080432815	PREDICTED: V-type proton ATPase 16 kDa proteolipid subunit
RS013599	GO:0044699	single-organism process	BP	-0.182689665	0.395528696	0.666677894	4.079879837	9.186285421	0.026913961	0.080798775	PREDICTED: alcohol dehydrogenase [NADP(+)]-like
RS009514	GO:0044699	single-organism process	BP	0.645819746	0.6092827	0.519808652	6.857573544	9.185784321	0.026920094	0.080798775	PREDICTED: H/ACA ribonucleoprotein complex subunit 3
RS007025	GO:0044699	single-organism process	BP	-0.646756609	-0.209658007	0.290941208	7.803114963	9.184468997	0.026936199	0.08082785	PREDICTED: fumarate hydratase, mitochondrial-like
RS010591	GO:0044699	single-organism process	BP	-0.801595268	-0.939760531	-0.841892471	4.241724584	9.178118556	0.027014087	0.08100367	PREDICTED: myosuppressin
RS004650	GO:0044699	single-organism process	BP	0.850673803	0.816552711	0.93883888	5.469693425	9.162260223	0.027209555	0.081531559	PREDICTED: protein grainyhead
RS004289	GO:0044699	single-organism process	BP	-0.745647812	-0.447361458	-0.613342816	7.891760836	9.157810686	0.027264649	0.081677208	PREDICTED: peroxidase-like
RS012178	GO:0044699	single-organism process	BP	-1.027741074	-0.1980121	0.153777121	4.415822931	9.157127984	0.027273111	0.08168313	PREDICTED: tyrosine-protein phosphatase 10D
RS010129	GO:0044699	single-organism process	BP	-0.216635499	-0.615347417	-0.539422062	5.171989793	9.148529898	0.027379913	0.081908682	PREDICTED: breast cancer type 2 susceptibility protein homolog
RS006944	GO:0044699	single-organism process	BP	-0.448114803	-1.113816169	-0.583334766	3.52112084	9.137339539	0.027519529	0.082303715	PREDICTED: pro-neuropeptide Y-like
RS004247	GO:0044699	single-organism process	BP	0.461639087	-0.103575911	-0.16666483	5.236023139	9.127198863	0.02764665	0.082605437	PREDICTED: U4/U6 small nuclear ribonucleoprotein Prp4
RS012808	GO:0044699	single-organism process	BP	0.436531101	0.768358177	0.047570539	8.601906761	9.12311113	0.027698056	0.082719781	PREDICTED: ATP-binding cassette sub-family E member 1
RS001330	GO:0044699	single-organism process	BP	-1.668504431	0.166119722	0.427397074	0.85487177	9.12227714	0.027708555	0.082731519	PREDICTED: mitochondrial dicarboxylate carrier
RS006509	GO:0044699	single-organism process	BP	0.093266267	0.278590645	0.708464019	6.298118015	9.118160861	0.027760434	0.082807888	PREDICTED: MIP18 family protein CG30152
RS013242	GO:0044699	single-organism process	BP	0.588121021	0.810761	0.637726782	6.366112122	9.106707706	0.027905282	0.083180857	PREDICTED: minor histocompatibility protein HA-1-like
RS014177	GO:0044699	single-organism process	BP	0.64345759	0.743657112	0.536940233	7.590947089	9.092444334	0.028086706	0.083642464	PREDICTED: profilin
RS001454	GO:0044699	single-organism process	BP	0.290328526	0.793256603	0.701232326	4.771700319	9.086226372	0.028166158	0.08381961	PREDICTED: tryptophan--tRNA ligase, cytoplasmic
RS008392	GO:0044699	single-organism process	BP	-0.746220384	-0.183091018	0.227040902	5.634504061	9.08305956	0.028206707	0.08392045	PREDICTED: probable cation-transporting ATPase 13A3
RS002136	GO:0044699	single-organism process	BP	0.750220748	1.080167298	0.829874657	9.257649854	9.077326625	0.028280259	0.084079693	PREDICTED: myotubularin-related protein 8
RS011015	GO:0044699	single-organism process	BP	-0.12196467	0.343730769	0.605371117	5.791134914	9.069640156	0.028379169	0.084254422	PREDICTED: protein extra-macrochaetae
RS010105	GO:0044699	single-organism process	BP	-0.567206255	-1.412112129	-0.886187691	5.676367558	9.069724408	0.028378083	0.084254422	PREDICTED: elongation of very long chain fatty acids protein 6
RS014324	GO:0044699	single-organism process	BP	-0.34257466	0.558612034	-1.393013712	1.322479778	9.042564169	0.028730293	0.085196447	PREDICTED: guanine nucleotide-binding protein subunit alpha homolog
RS007617	GO:0044699	single-organism process	BP	-1.32510407	-1.458516586	-0.870134155	3.353068646	9.039095435	0.028775582	0.085310659	PREDICTED: zinc transporter ZIP10
RS009334	GO:0044699	single-organism process	BP	0.254491937	0.542690228	-0.328792	5.144186147	9.037592254	0.02879523	0.085348817	PREDICTED: thioredoxin domain-containing protein 9
RS009139	GO:0044699	single-organism process	BP	0.649351007	1.292845053	0.336142374	2.039377777	9.031613663	0.028873505	0.085540561	PREDICTED: Y+L amino acid transporter 2
RS006938	GO:0044699	single-organism process	BP	-1.36465961	-1.65981816	-0.774038627	3.586593371	9.027650125	0.028925513	0.08561408	PREDICTED: carboxylesterase 1E
RS005831	GO:0044699	single-organism process	BP	-0.45966863	-0.501653004	-0.576157257	5.427123424	9.023124457	0.028985009	0.085770019	PREDICTED: TBC1 domain family member 14
RS015211	GO:0044699	single-organism process	BP	0.030199313	0.344318338	0.583878025	7.160814049	9.021892779	0.029001222	0.085786944	PREDICTED: ubiquitin-conjugating enzyme E2-17 kDa
RS011494	GO:0044699	single-organism process	BP	0.202411998	0.165418769	-0.691185184	7.134946488	9.021655055	0.029004352	0.085786944	PREDICTED: laminin subunit beta-1
RS011064	GO:0044699	single-organism process	BP	-1.082972026	-1.027491454	-0.806343821	3.290957675	9.019949401	0.029026821	0.085833243	PREDICTED: glycoprotein 3-alpha-L-fucosyltransferase A
RS005713	GO:0044699	single-organism process	BP	0.27965859	1.390496131	1.129673048	2.871314037	8.995574636	0.029349782	0.086668373	PREDICTED: cyclic AMP response element-binding protein A
RS009757	GO:0044699	single-organism process	BP	0.640503403	0.649550604	0.49019916	5.965200425	8.985261288	0.029487489	0.086950465	PREDICTED: trafficking protein particle complex subunit 1
RS002268	GO:0044699	single-organism process	BP	-0.663213265	-0.543123609	-0.277002394	5.289469542	8.984030819	0.029503961	0.086978671	PREDICTED: inositol 1,4,5-trisphosphate receptor
RS013684	GO:0044699	single-organism process	BP	-0.892438207	-0.036472036	-0.439366306	3.419195067	8.971652748	0.029670163	0.087407258	PREDICTED: centaurin-gamma-1A
RS007528	GO:0044699	single-organism process	BP	1.281551474	0.79778074	0.871132273	9.026899419	8.962975306	0.029787222	0.087649596	ubiquitin
RS000078	GO:0044699	single-organism process	BP	0.468513621	0.680037401	0.281061525	6.456939604	8.950555866	0.029955548	0.088000973	PREDICTED: peroxisomal membrane protein PEX14
RS012407	GO:0044699	single-organism process	BP	0.402719322	0.564945555	0.711127243	6.500525743	8.936168409	0.030151712	0.088494676	PREDICTED: probable phenylalanine--tRNA ligase, mitochondrial
RS001927	GO:0044699	single-organism process	BP	0.771616176	0.674937655	0.707792447	6.929359786	8.935578229	0.030159785	0.088497748	PREDICTED: alanyl-tRNA editing protein Aarsd1
RS013576	GO:0044699	single-organism process	BP	-0.152692106	0.186422925	0.739608273	8.524782718	8.933399856	0.030189603	0.088564608	PREDICTED: cytochrome c oxidase subunit 7A2, mitochondrial-like
RS014990	GO:0044699	single-organism process	BP	-0.847768343	-1.496659591	-1.543821536	2.005749414	8.920079077	0.030372569	0.089059868	PREDICTED: ninjurin-1-like
RS006922	GO:0044699	single-organism process	BP	-1.434864836	-1.711777799	-1.847138408	0.586150524	8.915938687	0.030429659	0.089206502	PREDICTED: reticulon-4 receptor-like 2
RS001623	GO:0044699	single-organism process	BP	-1.619350615	-1.891194927	0.366953342	0.439561173	8.905530474	0.030573639	0.089605739	PREDICTED: 5-hydroxytryptamine receptor 1
RS006567	GO:0044699	single-organism process	BP	1.188624292	0.01513041	0.042556045	1.61880047	8.903943977	0.030595644	0.089630506	PREDICTED: exonuclease 1
RS001227	GO:0044699	single-organism process	BP	0.41183477	0.56957695	0.128982257	5.629565936	8.899239622	0.030660986	0.089780157	PREDICTED: guanylate kinase
RS008214	GO:0044699	single-organism process	BP	-0.591657749	-0.60801793	-0.342781866	5.622477097	8.89592842	0.030707059	0.089873274	PREDICTED: sodium channel protein paralytic
RS007859	GO:0044699	single-organism process	BP	-1.037355681	-0.953164057	-1.137934678	2.631254112	8.895408464	0.0307143	0.08987358	PREDICTED: proton-coupled amino acid transporter 4
RS015133	GO:0044699	single-organism process	BP	0.62924298	0.657422051	0.31187198	7.333767054	8.873750789	0.031017395	0.090697255	PREDICTED: eukaryotic translation initiation factor 2 subunit 3-like
RS013818	GO:0044699	single-organism process	BP	-0.091150147	0.704869775	0.985919631	2.320087932	8.860612664	0.031202686	0.091133262	PREDICTED: kidney mitochondrial carrier protein 1
RS011670	GO:0044699	single-organism process	BP	-0.500688314	-0.906644814	-0.001083981	3.985350954	8.857204244	0.031250932	0.091231859	PREDICTED: protein slit
RS010186	GO:0044699	single-organism process	BP	1.836830068	0.632048386	0.803862589	1.840047339	8.830539051	0.031630908	0.092212884	PREDICTED: probable proline dehydrogenase 2
RS003404	GO:0044699	single-organism process	BP	-0.282230962	0.285723529	0.434899732	4.438872301	8.825502366	0.031703186	0.092359456	PREDICTED: DET1- and DDB1-associated protein 1
RS013668	GO:0044699	single-organism process	BP	-1.170027407	-0.729178035	0.872714223	0.383894951	8.820674505	0.031772619	0.092540325	PREDICTED: glutamate receptor 3.4-like
RS010255	GO:0044699	single-organism process	BP	1.018238849	0.227228558	-0.036585953	2.079744763	8.817858736	0.031813184	0.092637048	PREDICTED: dynein intermediate chain 2, axonemal

RS006943	GO:0044699	single-organism process	BP	-0.745844928	-0.858790002	-0.719751741	4.133949221	8.813227185	0.031880016	0.092788751	PREDICTED: diacylglycerol kinase 1
RS014121	GO:0044699	single-organism process	BP	-0.032184465	-0.279490394	-0.777791457	4.111467091	8.810465743	0.031919929	0.092883454	PREDICTED: DNA mismatch repair protein Msh6
RS009134	GO:0044699	single-organism process	BP	-0.8891536	-0.06070733	-0.116917154	4.724648661	8.808122123	0.031953841	0.092939186	PREDICTED: putative protein kinase C delta type homolog
RS000888	GO:0044699	single-organism process	BP	0.606197762	0.501709067	0.782757085	6.085163388	8.803152867	0.032025863	0.093060619	PREDICTED: 39S ribosomal protein L54, mitochondrial
RS000001	GO:0044699	single-organism process	BP	-0.923753843	-0.774146522	-0.309728113	8.964042611	8.801674733	0.032047317	0.093060619	PREDICTED: ryanodine receptor 44F
RS006058	GO:0044699	single-organism process	BP	-0.056910353	-0.595195322	-0.351052445	6.583193568	8.79853438	0.032092943	0.093150155	PREDICTED: GMP synthase [glutamine-hydrolyzing]
RS001476	GO:0044699	single-organism process	BP	-1.145211616	-1.733503349	-0.560856033	2.306347247	8.780028535	0.032363107	0.093847795	PREDICTED: glucose-dependent insulinotropic receptor-like
RS015412	GO:0044699	single-organism process	BP	-0.797867905	-0.553260932	-0.632470904	4.484132016	8.764763712	0.03258763	0.094433643	PREDICTED: diacylglycerol O-acyltransferase 1
RS014767	GO:0044699	single-organism process	BP	0.908823423	0.780719592	0.404104689	4.410402715	8.756690788	0.032706986	0.094714137	PREDICTED: phosphatidylserine decarboxylase proenzyme
RS002022	GO:0044699	single-organism process	BP	0.62041628	0.278617857	0.05008813	5.14866597	8.75565426	0.032722342	0.094736821	PREDICTED: ADP-ribosylation factor-like protein 1
RS009114	GO:0044699	single-organism process	BP	-0.050493186	-1.398246154	-1.036381905	2.348641228	8.75155828	0.032783091	0.094847288	PREDICTED: DNA replication licensing factor Mcm6
RS009135	GO:0044699	single-organism process	BP	-2.013397474	0.050668944	0.558887993	0.476879882	8.748046843	0.032835258	0.094920773	PREDICTED: sodium channel protein Nach-like
RS006176	GO:0044699	single-organism process	BP	0.428128298	0.059517256	-0.293113923	5.9989529	8.74465504	0.032885725	0.095013265	PREDICTED: ubiquitin carboxyl-terminal hydrolase isozyme L3
RS010568	GO:0044699	single-organism process	BP	0.386193784	0.686631425	0.844148867	6.978022235	8.738768615	0.03297349	0.095244985	PREDICTED: protein Simiate
RS011899	GO:0044699	single-organism process	BP	-0.131765772	-0.709553389	-0.319477363	5.321098546	8.728454249	0.033127827	0.095626909	PREDICTED: intraflagellar transport protein 80 homolog
RS012083	GO:0044699	single-organism process	BP	0.284978601	0.31601334	0.727329773	4.646874778	8.707376247	0.033445424	0.096409171	PREDICTED: casein kinase I isoform epsilon
RS013696	GO:0044699	single-organism process	BP	0.605064909	0.576813725	0.60067116	6.352280709	8.698822867	0.033575151	0.096628303	PREDICTED: methylmalonic aciduria and homocystinuria type D homolog, mitochondrial
RS007024	GO:0044699	single-organism process	BP	-0.128844159	0.682616829	-0.186562737	3.98321222	8.695142083	0.033631128	0.09676729	PREDICTED: arginase, hepatic
RS011130	GO:0044699	single-organism process	BP	0.62266454	0.316671327	-0.198111492	8.646709884	8.691352167	0.03368886	0.096889131	PREDICTED: 60 kDa heat shock protein, mitochondrial-like
RS014012	GO:0044699	single-organism process	BP	-1.016533135	-0.664013291	-0.585003747	3.026158548	8.678661326	0.033882886	0.097313813	PREDICTED: thyrotropin-releasing hormone receptor-like
RS014303	GO:0044699	single-organism process	BP	1.015519516	-0.140449015	-1.617687421	1.096446077	8.671576225	0.033991683	0.097559539	PREDICTED: sodium channel protein Nach-like
RS001683	GO:0044699	single-organism process	BP	-0.662301892	-0.390743135	0.127930711	5.093296427	8.667052181	0.034061332	0.097714882	Inactive phospholipase C-like 1
RS014632	GO:0044699	single-organism process	BP	-0.68677021	-0.539157917	-0.302904262	5.052169026	8.66245247	0.034132288	0.09787387	PREDICTED: serine/threonine-protein phosphatase 2A 56 kDa regulatory subunit epsilon isoform
RS010093	GO:0044699	single-organism process	BP	-0.728071992	-0.235326584	0.394446618	2.873319327	8.661280723	0.034150387	0.097897452	PREDICTED: phosphatidate cytidylyltransferase, photoreceptor-specific
RS012707	GO:0044699	single-organism process	BP	0.321560918	-0.670702602	-0.628783261	2.037388916	8.651044035	0.034308902	0.098268436	PREDICTED: rho GTPase-activating protein 19-like
RS010234	GO:0044699	single-organism process	BP	-0.015484323	0.41266545	0.730886441	7.257240265	8.642929696	0.034435063	0.098562543	PREDICTED: pyruvate dehydrogenase E1 component subunit beta, mitochondrial
RS002788	GO:0044699	single-organism process	BP	0.112865471	1.766259932	0.869293526	0.546463984	8.641892628	0.03445122	0.098583582	PREDICTED: bone morphogenetic protein 10
RS009100	GO:0044699	single-organism process	BP	0.342349417	0.166795763	-0.532068138	4.09432898	8.63830405	0.034507186	0.098679268	PREDICTED: homeotic protein deformed
RS001966	GO:0044699	single-organism process	BP	-0.643121314	-0.728339763	-0.033251824	5.175342657	8.637273014	0.034523281	0.098702884	carboxylesterase
RS011648	GO:0044699	single-organism process	BP	0.507387692	0.63293481	0.104468555	5.07515494	8.635539851	0.034550355	0.098740666	PREDICTED: FAD-linked sulfhydryl oxidase ALR
RS007561	GO:0044699	single-organism process	BP	0.605175865	0.578016944	0.41319379	6.223245073	8.631943265	0.034606603	0.098873765	PREDICTED: histidine--tRNA ligase, cytoplasmic
RS002253	GO:0044699	single-organism process	BP	0.296156782	0.238201065	-0.309563994	5.640625565	8.627730521	0.034672601	0.099039859	PREDICTED: protein Hook homolog 3
RS013703	GO:0044699	single-organism process	BP	0.065758707	-0.676009631	-0.491825966	4.341509589	8.620485247	0.034786395	0.09929734	PREDICTED: DNA mismatch repair protein Msh2
RS014200	GO:0044699	single-organism process	BP	0.368512158	-0.23900377	-0.315539871	4.740713374	8.620559497	0.034785227	0.09929734	PREDICTED: sphingosine-1-phosphate lyase
RS012263	GO:0044699	single-organism process	BP	-0.683736045	-1.767581064	-1.46798217	0.795763765	8.618519886	0.034817326	0.099341099	PREDICTED: fasciclin-2
RS007513	GO:0044699	single-organism process	BP	-1.005284005	-1.097119765	-0.564348321	2.866106464	8.604803517	0.035033944	0.099868152	PREDICTED: gamma-aminobutyric acid type B receptor subunit 2
RS013390	GO:0044699	single-organism process	BP	0.39926361	0.726410071	0.413971661	6.912999481	8.599223653	0.035122442	0.100097767	PREDICTED: probable signal peptidase complex subunit 2
RS006776	GO:0044699	single-organism process	BP	0.71638764	0.579864743	0.458766163	5.992561337	8.584837864	0.035351611	0.10061428	PREDICTED: NADPH-dependent diflavin oxidoreductase 1
RS013383	GO:0044699	single-organism process	BP	-0.958350269	-0.933135261	-1.046963183	2.885636215	8.584230757	0.035361315	0.100619158	PREDICTED: solute carrier organic anion transporter family member 3A1
RS002608	GO:0044699	single-organism process	BP	0.023311756	0.589903849	0.93280004	9.464511211	8.57996396	0.035429585	0.100699659	PREDICTED: cytochrome c-2
RS005165	GO:0044699	single-organism process	BP	0.075414856	-0.442218337	0.462888	5.369391281	8.566759673	0.035641674	0.101165482	PREDICTED: receptor-type tyrosine-protein phosphatase F-like
RS010884	GO:0044699	single-organism process	BP	-0.877259234	-0.855709811	-0.755005157	3.207767966	8.564522758	0.035677727	0.101199389	PREDICTED: inositol polyphosphate 1-phosphatase
RS009970	GO:0044699	single-organism process	BP	-0.427278507	-0.034042587	0.939067586	1.319946371	8.562659198	0.035707789	0.101216271	PREDICTED: bicaudal D-related protein homolog
RS002100	GO:0044699	single-organism process	BP	0.521250686	0.500182617	0.580767602	6.787528007	8.558799736	0.035770127	0.101347352	PREDICTED: methylosome subunit pICln
RS001434	GO:0044699	single-organism process	BP	-2.10642209	-0.940504046	-0.189980266	0.468874389	8.555806562	0.035818546	0.101461711	PREDICTED: sodium channel protein Nach-like
RS001886	GO:0044699	single-organism process	BP	-1.267711199	-1.679853662	-0.927754955	0.85492185	8.555164787	0.035828936	0.10146832	PREDICTED: protein croquemort-like
RS009755	GO:0044699	single-organism process	BP	-0.043169603	0.458963811	0.35352347	6.296109057	8.549721708	0.035917175	0.101633978	PREDICTED: phosphatidylinositol 4-kinase beta
RS003873	GO:0044699	single-organism process	BP	0.683138248	0.499702921	0.376504248	5.547932665	8.539451523	0.036084246	0.101984962	PREDICTED: N-acetylglucosaminyl-phosphatidylinositol biosynthetic protein
RS012852	GO:0044699	single-organism process	BP	0.095937018	0.366584098	-0.497735083	4.298329529	8.535728142	0.036145003	0.102133761	PREDICTED: glutamate receptor ionotropic, kainate 2-like
RS003907	GO:0044699	single-organism process	BP	-0.780904403	-0.676585263	-0.418988272	8.536828109	8.530481048	0.036230795	0.102353214	PREDICTED: serine/threonine-protein kinase PINK1, mitochondrial
RS011109	GO:0044699	single-organism process	BP	0.640129642	0.546672973	0.174750688	7.959476904	8.527314593	0.036282663	0.102430813	PREDICTED: latrophilin Cirl-like
RS004893	GO:0044699	single-organism process	BP	0.028836543	-0.29186153	-0.578578001	6.468371045	8.500242671	0.036729089	0.103436079	PREDICTED: another transcription unit protein
RS005033	GO:0044699	single-organism process	BP	-1.307098604	-0.07215234	-0.796767707	3.08063394	8.493682136	0.036838079	0.103650307	PREDICTED: hydroxymethylglutaryl-CoA synthase 1
RS014953	GO:0044699	single-organism process	BP	0.853189222	0.551273025	0.027539056	4.191494556	8.492895911	0.036851162	0.103663957	PREDICTED: H/ACA ribonucleoprotein complex subunit 2-like protein
RS005057	GO:0044699	single-organism process	BP	-0.033025012	0.199673677	0.677121448	6.846017574	8.479134724	0.037080885	0.104217064	PREDICTED: NADH dehydrogenase [ubiquinone] 1 alpha subcomplex subunit 2
RS010661	GO:0044699	single-organism process	BP	-1.196464064	-1.125289107	-2.512040374	1.571507161	8.471850824	0.037203044	0.104537068	long-wavelength opsin
RS005326	GO:0044699	single-organism process	BP	0.532492415	0.058581044	-0.08323301	4.899932392	8.464558485	0.037325739	0.104835048	PREDICTED: DNA mismatch repair protein Msh3
RS005094	GO:0044699	single-organism process	BP	0.776797656	0.509601441	0.126828145	3.579945988	8.463680805	0.037340533	0.104853214	PREDICTED: B9 domain-containing protein 1
RS000445	GO:0044699	single-organism process	BP	-0.654177928	0.616204789	0.505661822	2.230372795	8.460389722	0.037396056	0.104938933	cytochrome P450
RS005358	GO:0044699	single-organism process	BP	0.260075089	0.471390925	0.694718137	6.608164203	8.461122113	0.037383693	0.104938933	PREDICTED: surfeit locus protein 1
RS005160	GO:0044699	single-organism process	BP	-0.164248663	-0.503625548	-0.947201397	3.860185221	8.444286072	0.037668905	0.105610461	PREDICTED: RNA polymerase-associated protein RTF1 homolog
RS014087	GO:0044699	single-organism process	BP	-0.743002326	-1.020886612	-0.697919932	3.048117664	8.443176869	0.03768777	0.105639834	PREDICTED: amidophosphoribosyltransferase-like
RS001488	GO:0044699	single-organism process	BP	0.51791782	0.710327442	0.83324624	7.761417394	8.425639535	0.037987268	0.106302401	PREDICTED: growth arrest and DNA damage-inducible proteins-interacting protein 1
RS006017	GO:0044699	single-organism process	BP	-0.226333875	0.829010789	0.173010283	3.457042927	8.42538268	0.037991671	0.106302401	PREDICTED: cationic amino acid transporter 2
RS006818	GO:0044699	single-organism process	BP	0.176324187	-0.432331746	-0.512902229	3.880176402	8.423627123	0.038021783	0.106363025	PREDICTED: transmembrane protein 231
RS011681	GO:0044699	single-organism process	BP	-0.591852065	-1.543222476	-0.819954651	1.994853741	8.417741291	0.038122909	0.106622234	Metabotropic glutamate receptor 2
RS013655	GO:0044699	single-organism process	BP	-0.201023512	0.012381247	-1.007472533	9.301050197	8.410677765	0.038244617	0.106938877	PREDICTED: transforming growth factor-beta-induced protein ig-h3-like
RS015009	GO:0044699	single-organism process	BP	0.452819	0.56796968	0.343760656	6.19776174	8.407300491	0.038302942	0.107030675	PREDICTED: dolichyl-diphosphooligosaccharide--protein glycosyltransferase subunit DAD1
RS010148	GO:0044699	single-organism process	BP	0.609571952	0.691443267	0.551683094	6.423538019	8.396934265	0.03848251	0.107413283	PREDICTED: ADP-ribosylation factor 2
RS003361	GO:0044699	single-organism process	BP	0.378052871	-0.2200652	-0.320610465	4.480880112	8.384945637	0.038691207	0.10786673	PREDICTED: rho GTPase-activating protein 11A-like
RS003858	GO:0044699	single-organism process	BP	-0.359815662	-0.446595841	0.382189615	3.498410245	8.385315512	0.038684752	0.10786673	PREDICTED: sodium/potassium-transporting ATPase subunit beta-2-like
RS010213	GO:0044699	single-organism process	BP	0.581736903	0.680717263	0.303984593	4.864342298	8.383716362	0.038712668	0.107886718	PREDICTED: tail-anchored protein insertion receptor WRB
RS009879	GO:0044699	single-organism process	BP	-0.425445258	0.392810445	0.467887213	5.118012521	8.377640701	0.038818912	0.108136558	PREDICTED: semaphorin-1A-like
RS004836	GO:0044699	single-organism process	BP	-0.726951085	-0.940049622	-0.81039797	4.415915436	8.366935638	0.039006801	0.108563902	PREDICTED: protein furry
RS013514	GO:0044699	single-organism process	BP	-0.925172564	-1.255322847	-1.149531993	1.178848556	8.347306403	0.039353632	0.109408309	PREDICTED: E3 ubiquitin-protein ligase SIAH1
RS001974	GO:0044699	single-organism process	BP	-0.162323419	-0.634665262	-0.632134264	4.375637983	8.332457006	0.039618005	0.109997609	PREDICTED: histone acetyltransferase KAT7
RS007468	GO:0044699	single-organism process	BP	0.457030598	-0.368844241	-0.214826608	7.85084108	8.327687594	0.039703285	0.110210089	PREDICTED: retinal dehydrogenase 1
RS008527	GO:0044699	single-organism process	BP	-0.169342875	-0.534096372	0.094011382	5.723617218	8.325889957	0.039735474	0.110275135	PREDICTED: ubiquitin-like-conjugating enzyme ATG3
RS009617	GO:0044699	single-organism process	BP	-1.035857016	0.816350748	-0.206897462	0.644945079	8.323758533	0.039773674	0.110339769	PREDICTED: centaurin-gamma-1A-like
RS014206	GO:0044699	single-organism process	BP	-0.331060594	-0.319039029	0.770529033	2.915958338	8.315762111	0.039917305	0.110682196	PREDICTED: mannosyl-oligosaccharide 1,2-alpha-mannosidase IA
RS013253	GO:0044699	single-organism process	BP	-0.531293038	-0.391779248	-0.887161561	3.90741334	8.310855501	0.040005687	0.110854043	PREDICTED: serine/threonine-protein kinase PLK4
RS009561	GO:0044699	single-organism process	BP	0.130900151	-0.477143343	-0.33797038	5.293328557	8.310233248	0.040016909	0.110860747	PREDICTED: pre-mRNA-processing factor 40 homolog A
RS001931	GO:0044699	single-organism process	BP	0.595939461	0.383048109	0.664722454	5.650828206	8.303677696	0.040135325	0.111115472	PREDICTED: NEDD8-conjugating enzyme UBE2F-like
RS002076	GO:0044699	single-organism process	BP	0.48711958	0.589170119	0.638551254	6.216982065	8.282426802	0.040521543	0.11201236	PREDICTED: inosine triphosphate pyrophosphatase
RS007413	GO:0044699	single-organism process	BP	0.401896552	0.598678698	-0.364632407	4.683481911	8.275295278	0.040651963	0.112274304	PREDICTED: monocyte to macrophage differentiation factor 2
RS003765	GO:0044699	single-organism process	BP	-0.267216254	-0.562886151	-1.191320732	2.534249501	8.259184855	0.040948098	0.112918842	PREDICTED: protein cornichon homolog 4
RS011895	GO:0044699	single-organism process	BP	-0.993627827	-0.703134464	-0.934337098	2.974091488	8.239608151	0.041310782	0.113645258	PREDICTED: SPRY domain-containing SOCS box protein 3
RS011366	GO:0044699	single-organism process	BP	0.020458032	0.159346075	0.718831212	7.623264922	8.219712526	0.041682585	0.114542978	PREDICTED: dnaJ homolog subfamily C member 28
RS002298	GO:0044699	single-organism process	BP	0.444685109	0.888871791	0.46931162	5.877833769	8.218233291	0.041710358	0.114594295	PREDICTED: battenin
RS007346	GO:0044699	single-organism process	BP	-0.638416263	0.123750631	0.995715645	12.76023637	8.208372814	0.041895955	0.114953738	PREDICTED: tropomyosin
RS010700	GO:0044699	single-organism process	BP	0.471430016	0.541282621	0.196614123	5.717274917	8.200768266	0.04203964	0.115247546	PREDICTED: thioredoxin-like protein 1
RS002073	GO:0044699	single-organism process	BP	-0.730372331	-0.653911276	-1.441098065	2.248321382	8.194678957	0.042155042	0.115463374	PREDICTED: neural-cadherin-like
RS001745	GO:0044699	single-organism process	BP	0.384909957	0.516054681	-0.3759302	4.817337581	8.192128882	0.042203461	0.115545737	PREDICTED: integrin alpha-PS1
RS003910	GO:0044699	single-organism process	BP	-0.595590646	-0.505583068	-0.068935582	5.32940786	8.185259112	0.042334171	0.115853226	carboxylesterase
RS002872	GO:0044699	single-organism process	BP	0.439579363	0.885948659	0.120051824	4.137670654	8.182874063	0.042379643	0.11595247	cytochrome P450
RS014407	GO:0044699	single-organism process	BP	-0.503325604	-1.05334215	-1.272939128	2.313306279	8.174699872	0.042535849	0.116304056	PREDICTED: molybdenum cofactor biosynthesis protein 1
RS011509	GO:0044699	single-organism process	BP	-0.530402083	-0.738074094	-1.271752002	3.986259296	8.140966438	0.043186444	0.11780162	PREDICTED: cytochrome b5-related protein-like
RS012784	GO:0044699	single-organism process	BP	-0.964188065	-1.053670411	-1.487092291	6.693074429	8.119987423	0.043595935	0.118762068	PREDICTED: excitatory amino acid transporter-like
RS014867	GO:0044699	single-organism process	BP	-0.157489035	-0.255961916	-0.998134687	2.846044163	8.112060193	0.04375165	0.11908543	PREDICTED: neuroglobin-like
RS001233	GO:0044699	single-organism process	BP	-0.691058221	-0.027290652	-0.036947076	5.25394964	8.110482484	0.043782706	0.119144209	PREDICTED: carbonic anhydrase 2
RS012691	GO:0044699	single-organism process	BP	-0.172595861	-0.578904307	-0.031671583	6.933895552	8.088105422	0.044225497	0.120245231	PREDICTED: 1,4-alpha-glucan-branching enzyme
RS013024	GO:0044699	single-organism process	BP	0.519161235	-0.028154291	0.169521169	5.686176167	8.078299435	0.044420908	0.120717943	PREDICTED: uroporphyrinogen decarboxylase
RS004779	GO:0044699	single-organism process	BP	-0.55306309	-0.562273758	-0.634999193	6.963712019	8.075096886	0.044484909	0.120820131	PREDICTED: 1-phosphatidylinositol 4,5-bisphosphate phosphodiesterase epsilon-1-like
RS006764	GO:0044699	single-organism process	BP	-0.051805943	0.184711003	0.558414735	6.83348825	8.073084384	0.044525174	0.120903416	PREDICTED: ragulator complex protein LAMTOR2
RS006910	GO:0044699	single-organism process	BP	-0.883620755	-0.757147404	-1.087681892	2.025636733	8.046070105	0.045069109	0.122143396	PREDICTED: GTP-binding protein Di-Ras2
RS014128	GO:0044699	single-organism process	BP	0.289503877	0.600035109	0.531285614	6.062217666	8.023392892	0.045530711	0.123208804	PREDICTED: ubiquitin-conjugating enzyme E2-17 kDa
RS010172	GO:0044699	single-organism process	BP	-0.481661904	-0.635138059	-0.336556682	6.471234663	8.019147909	0.045617629	0.123406231	PREDICTED: histone-lysine N-methyltransferase 2C-like
RS013365	GO:0044699	single-organism process	BP	-0.770264669	-1.02060252	-0.979223709	3.590470415	8.015114149	0.045700372	0.123535198	PREDICTED: peptide methionine sulfoxide reductase
RS008516	GO:0044699	single-organism process	BP	-0.470687748	-0.703651294	-0.654662903	7.419176436	8.008239799	0.04584172	0.123837543	PREDICTED: saccharopine dehydrogenase-like oxidoreductase
RS015155	GO:0044699	single-organism process	BP	0.639478075	0.516896094	0.44209348	6.319006926	8.002127166	0.045967764	0.12412479	PREDICTED: protein NPC2 homolog
RS005143	GO:0044699	single-organism process	BP	0.352665656	0.523610265	0.606871417	5.289665213	8.000205041	0.046007468	0.124178752	PREDICTED: protein SCO1 homolog, mitochondrial

RS002239	GO:0044699	single-organism process	BP	-0.518558054	-0.887931947	-1.328774582	2.771875246	7.996103171	0.04609231	0.124359208	PREDICTED: transient receptor potential cation channel protein painless
RS006971	GO:0044699	single-organism process	BP	-0.330033212	-1.64912161	-0.134661802	1.450654065	7.996017904	0.046094075	0.124359208	PREDICTED: secretin receptor-like
RS005572	GO:0044699	single-organism process	BP	-0.063350657	-0.485549058	-0.635829538	6.080941742	7.993630754	0.046143522	0.124412657	PREDICTED: histone-lysine N-methyltransferase ash1
RS013199	GO:0044699	single-organism process	BP	-0.830868079	-0.654963754	-0.904770904	4.065881842	7.990715751	0.046203973	0.124522328	PREDICTED: fatty acid hydroxylase domain-containing protein 2
RS001938	GO:0044699	single-organism process	BP	0.222675437	0.100399432	0.605261905	6.750945362	7.982114591	0.046382793	0.124924058	PREDICTED: mitochondrial intermediate peptidase
RS007516	GO:0044699	single-organism process	BP	-1.054756668	-0.26952049	1.034103673	15.46958455	7.975854067	0.046513374	0.125168682	actin
RS015504	GO:0044699	single-organism process	BP	-0.346792054	-0.919939272	-0.688384249	5.423422413	7.961040774	0.046823775	0.125842642	NAD(P) transhydrogenase, mitochondrial
RS010028	GO:0044699	single-organism process	BP	-0.660251529	-0.727004979	-0.471042699	3.72377673	7.944090491	0.04718143	0.126695719	PREDICTED: kinesin-like protein KIF16B
RS005203	GO:0044699	single-organism process	BP	0.549530222	0.550484134	0.290145942	6.388995069	7.938656006	0.047296661	0.126897753	PREDICTED: FGFR1 oncogene partner
RS005210	GO:0044699	single-organism process	BP	-0.986436868	-1.240920184	-1.092022056	3.917975026	7.935991254	0.047353263	0.126994675	PREDICTED: multidrug resistance protein homolog 49
RS013982	GO:0044699	single-organism process	BP	0.796086126	1.164886475	0.862624202	9.90157022	7.933713993	0.047401687	0.127097477	PREDICTED: 40S ribosomal protein S9
RS015189	GO:0044699	single-organism process	BP	-0.712049522	-0.413267466	0.027260785	4.577437281	7.933109118	0.047414557	0.127104925	PREDICTED: adenylate cyclase type 3
RS005703	GO:0044699	single-organism process	BP	-0.059967398	0.557072618	0.344174957	4.906279033	7.921012176	0.047672666	0.127674845	PREDICTED: signal recognition particle subunit SRP72
RS010542	GO:0044699	single-organism process	BP	0.116103344	-0.618699857	-0.860552226	2.745008544	7.913554572	0.047832468	0.127853488	PREDICTED: sialic acid synthase
RS006194	GO:0044699	single-organism process	BP	0.361384182	0.803747373	-0.293940475	3.413679546	7.912785294	0.047848982	0.127861251	PREDICTED: ecdysone receptor
RS008614	GO:0044699	single-organism process	BP	-0.377384484	0.178151297	0.139169662	6.960985845	7.911936368	0.047867212	0.127882831	PREDICTED: H(+)/Cl(-) exchange transporter 5
RS010967	GO:0044699	single-organism process	BP	0.614024398	-0.376867979	-0.234778695	3.413421752	7.903104824	0.048057263	0.128308919	PREDICTED: protein FAM135A
RS011310	GO:0044699	single-organism process	BP	0.512463955	0.266450018	-0.590116921	4.721320355	7.903303067	0.048052989	0.128308919	PREDICTED: protocadherin-like wing polarity protein stan
RS002728	GO:0044699	single-organism process	BP	0.212818207	0.643840285	0.425436189	7.554169008	7.882815481	0.048496668	0.129395018	PREDICTED: protein RFT1 homolog
RS012827	GO:0044699	single-organism process	BP	0.270811737	0.00479393	-0.664455338	6.443908707	7.879526255	0.04856827	0.129535957	PREDICTED: calnexin
RS007665	GO:0044699	single-organism process	BP	-0.631881952	-0.428365421	0.007983181	8.098194771	7.871009593	0.048754144	0.129949141	PREDICTED: plasma membrane calcium-transporting ATPase 2
RS007762	GO:0044699	single-organism process	BP	0.472872954	0.175202644	-0.261101136	4.994018262	7.854476871	0.049116946	0.13075012	PREDICTED: mitochondrial import inner membrane translocase subunit Tim8 A
RS003841	GO:0044699	single-organism process	BP	-0.018830809	0.667905762	-0.171179406	4.919354176	7.849821458	0.04921958	0.130930145	PREDICTED: major facilitator superfamily domain-containing protein 10
RS014326	GO:0044699	single-organism process	BP	0.497414651	0.118895049	-0.126889249	6.198656401	7.849052394	0.049236555	0.130930145	PREDICTED: UPF0505 protein C16orf62 homolog
RS005725	GO:0044699	single-organism process	BP	-0.139929744	-0.747433941	0.682163756	2.165224256	7.841793188	0.049397062	0.131246118	Retrovirus-related Pol polyprotein from transposon
RS007166	GO:0044699	single-organism process	BP	-0.621306361	-0.446886661	-1.302335205	1.763190005	7.8397232	0.049442925	0.131312567	PREDICTED: 5-hydroxytryptamine receptor 2A
RS005925	GO:0044699	single-organism process	BP	-0.192287424	0.588168671	-0.314585038	3.493046409	7.837253835	0.04949769	0.131430299	PREDICTED: nephrin
RS008109	GO:0044699	single-organism process	BP	-0.547779539	-0.931279331	-1.767829636	1.712240618	7.83613061	0.04952262	0.131468777	PREDICTED: insulin-like growth factor-binding protein complex acid labile subunit
RS004034	GO:0044699	single-organism process	BP	-0.208823665	-0.581751124	-0.634982994	4.436435309	7.824898102	0.049772602	0.13192465	PREDICTED: origin recognition complex subunit 2
RS008434	GO:0044699	single-organism process	BP	-0.152418883	-0.335351871	-0.836822092	4.94412872	7.808586514	0.050137806	0.132702402	PREDICTED: potassium voltage-gated channel subfamily H member 2
RS010293	GO:0044699	single-organism process	BP	-1.323536549	-0.537537624	-0.40616185	2.969834016	7.797939139	0.050377598	0.133149101	PREDICTED: sodium/hydrogen exchanger 3
RS002931	GO:0044699	single-organism process	BP	-0.784498789	-0.645948105	-0.625581434	3.345246421	7.781766391	0.050743963	0.133976828	PREDICTED: acetylcholine receptor subunit beta-like 2
RS001210	GO:0044699	single-organism process	BP	-0.156036564	-0.477461749	0.221069939	5.507603598	7.778628958	0.050815336	0.134137149	PREDICTED: intraflagellar transport protein 74 homolog
RS012051	GO:0044699	single-organism process	BP	-1.206457775	-0.320139517	-0.400442703	3.93903891	7.774642457	0.050906164	0.13434875	PREDICTED: sodium-independent sulfate anion transporter-like
RS012317	GO:0044699	single-organism process	BP	-0.648427381	0.001131192	-0.007541087	6.300999393	7.771586789	0.050975892	0.13447641	PREDICTED: calcium-transporting ATPase type 2C member 1
RS013440	GO:0044699	single-organism process	BP	-0.636222533	-0.934297367	-0.595689136	2.922678358	7.746146156	0.05156004	0.135733098	F-box/LRR-repeat protein 2
RS012751	GO:0044699	single-organism process	BP	-0.577850405	-0.795884178	-0.492958446	6.531780063	7.74261211	0.051641699	0.135834493	PREDICTED: cAMP-dependent protein kinase catalytic subunit
RS011113	GO:0044699	single-organism process	BP	-0.734065104	-0.772083364	0.314417947	1.839190076	7.734385648	0.05183227	0.136221956	PREDICTED: tubulin alpha-1C chain-like
RS000053	GO:0044699	single-organism process	BP	-0.581337051	-0.604699475	-0.828922976	4.14463726	7.729731173	0.051940397	0.136420722	PREDICTED: vacuolar protein sorting-associated protein 33A
RS008678	GO:0044699	single-organism process	BP	-0.676293204	-0.385200901	-0.071994287	4.611206664	7.717817688	0.052218156	0.137007387	zinc finger protein, putative
RS007092	GO:0044699	single-organism process	BP	0.089476635	-0.622782824	-0.592047442	3.832833636	7.714044317	0.052306432	0.137210414	PREDICTED: replication factor C subunit 4
RS001190	GO:0044699	single-organism process	BP	0.595914093	1.050394293	0.805952275	9.945959972	7.712932183	0.052332477	0.137250149	PREDICTED: 40S ribosomal protein S3
RS004595	GO:0044699	single-organism process	BP	0.458645321	0.171189243	-0.213779238	6.701384486	7.69001904	0.0528719	0.138376705	PREDICTED: DNA-(apurinic or apyrimidinic site) lyase
RS007740	GO:0044699	single-organism process	BP	-1.881332081	-0.475084205	-1.553001032	0.397357369	7.673203792	0.0532712	0.139277038	PREDICTED: dynein heavy chain 10, axonemal
RS000100	GO:0044699	single-organism process	BP	-0.365378747	-0.640466631	-0.535150561	6.92743209	7.672351081	0.053291527	0.139301263	PREDICTED: kinesin heavy chain
RS008221	GO:0044699	single-organism process	BP	0.494584107	0.577949482	0.596443217	6.228276146	7.64532803	0.053939612	0.140674151	PREDICTED: S-adenosyl-L-methionine-dependent tRNA 4-demethylwyosine synthase
RS003265	GO:0044699	single-organism process	BP	0.67065611	0.570992468	0.622458253	5.669387556	7.641310173	0.054036623	0.140868812	PREDICTED: 28S ribosomal protein S9, mitochondrial
RS005856	GO:0044699	single-organism process	BP	0.584869974	0.206275451	0.424307139	5.438984465	7.623424627	0.054470529	0.141823825	PREDICTED: small ubiquitin-related modifier 3
RS002584	GO:0044699	single-organism process	BP	-0.38019887	-0.726008621	-0.802464256	6.091882598	7.622877541	0.054483854	0.141829199	putative G-protein coupled receptor 158
RS004672	GO:0044699	single-organism process	BP	0.353656262	0.61047509	0.327590838	7.102946281	7.619168949	0.05457427	0.142005858	PREDICTED: coatomer subunit epsilon
RS004318	GO:0044699	single-organism process	BP	0.491549724	0.757087987	0.844223327	5.176959997	7.614963704	0.05467697	0.142243701	PREDICTED: glyoxylate/hydroxypyruvate reductase A-like
RS002101	GO:0044699	single-organism process	BP	-0.144951689	-0.554820667	-0.459503979	7.127596683	7.614008776	0.054700317	0.14227505	PREDICTED: long-chain-fatty-acid--CoA ligase 4
RS003565	GO:0044699	single-organism process	BP	0.009379947	-0.588411186	-0.622600778	4.36081212	7.606274085	0.054889782	0.1427089	PREDICTED: dedicator of cytokinesis protein 7
RS010768	GO:0044699	single-organism process	BP	0.081909498	-0.543954744	-0.957622884	2.342725479	7.599016154	0.055068148	0.143110009	PREDICTED: serine/threonine-protein kinase greatwall
RS004949	GO:0044699	single-organism process	BP	-0.136902988	-0.407301819	-0.704907953	4.613075273	7.593655487	0.055200249	0.143338544	PREDICTED: neural Wiskott-Aldrich syndrome protein-like
RS015407	GO:0044699	single-organism process	BP	-0.53343531	-0.824506724	0.003613607	6.554411162	7.589715422	0.055297539	0.143502413	PREDICTED: sodium/potassium-transporting ATPase subunit beta-2
RS005847	GO:0044699	single-organism process	BP	-0.009134422	0.240630937	0.785370217	8.208365721	7.589016609	0.055314812	0.143517665	PREDICTED: ATP synthase subunit epsilon, mitochondrial-like
RS011336	GO:0044699	single-organism process	BP	-0.879188978	-1.233405357	-1.234396951	2.133042356	7.583735869	0.055445508	0.143708732	PREDICTED: receptor-type guanylate cyclase daf-11
RS011462	GO:0044699	single-organism process	BP	-0.000757058	-0.523845676	-0.405417273	5.076518471	7.580389939	0.055528473	0.143894155	PREDICTED: H/ACA ribonucleoprotein complex non-core subunit NAF1
RS005855	GO:0044699	single-organism process	BP	0.058122704	0.338586634	0.567482864	6.250681808	7.56879813	0.055816836	0.144552176	PREDICTED: DNA-directed RNA polymerases I, II, and III subunit RPABC5
RS008140	GO:0044699	single-organism process	BP	-0.361857045	-0.714572238	-0.480772878	5.655369201	7.561721547	0.055993591	0.14489075	PREDICTED: kinesin-like protein KIF21A
RS014124	GO:0044699	single-organism process	BP	0.444731341	-0.111272035	-0.039155407	5.479298892	7.550475121	0.056275616	0.145500945	PREDICTED: PRKC apoptosis WT1 regulator protein-like
RS003764	GO:0044699	single-organism process	BP	-1.154969853	-0.552489744	-0.181870194	2.19921009	7.540167043	0.056535321	0.146030227	PREDICTED: 1-phosphatidylinositol 4,5-bisphosphate phosphodiesterase classes I and II
RS011021	GO:0044699	single-organism process	BP	0.642615063	0.849810005	0.523625278	4.438603171	7.538269452	0.056583257	0.146116369	PREDICTED: glucose-fructose oxidoreductase domain-containing protein 1
RS007829	GO:0044699	single-organism process	BP	-0.263506913	-0.868406959	-1.240307969	3.387506175	7.533681033	0.056699328	0.146356086	PREDICTED: neurotactin
RS004754	GO:0044699	single-organism process	BP	-0.342169634	-0.743078845	-0.417925461	4.970480981	7.522387592	0.056986001	0.147005672	PREDICTED: condensin-2 complex subunit D3
RS004115	GO:0044699	single-organism process	BP	-0.627603385	-1.062312103	-2.423539101	0.810737055	7.504090363	0.057453446	0.147969059	PREDICTED: pyrroline-5-carboxylate reductase 2
RS014918	GO:0044699	single-organism process	BP	-0.265116474	0.084232844	0.568475259	7.79189049	7.503165544	0.057477171	0.147999897	PREDICTED: cytochrome c1, heme protein, mitochondrial
RS007235	GO:0044699	single-organism process	BP	-0.411884685	-0.555436113	-0.88679334	5.718214674	7.500689334	0.057540743	0.148133302	PREDICTED: melanotransferrin
RS002518	GO:0044699	single-organism process	BP	0.487075817	0.195434226	0.865280956	9.446754703	7.493415036	0.057727889	0.148493676	PREDICTED: S-adenosylmethionine synthase
RS003700	GO:0044699	single-organism process	BP	0.445500208	0.547805665	0.90099913	5.029149185	7.491054113	0.057788756	0.148619889	PREDICTED: UDP-glucose 6-dehydrogenase
RS001491	GO:0044699	single-organism process	BP	0.093273478	-0.939776398	0.008331587	2.343336972	7.487647322	0.057876696	0.148785285	PREDICTED: DNA replication complex GINS protein SLD5
RS012492	GO:0044699	single-organism process	BP	-0.930779787	-0.788218054	-0.940010835	4.262815585	7.482344431	0.058013839	0.149076981	PREDICTED: homeobox protein prospero
RS009082	GO:0044699	single-organism process	BP	-1.344763505	-0.953478298	-1.303298962	1.512508029	7.470773642	0.058314177	0.149663762	PREDICTED: glycine receptor subunit alpha-2-like
RS001254	GO:0044699	single-organism process	BP	0.293780314	0.053368454	-0.472044391	5.211924016	7.461905116	0.058545394	0.150167147	PREDICTED: adenylate kinase
RS000291	GO:0044699	single-organism process	BP	0.357652774	0.553120416	0.724586151	5.358817226	7.45908553	0.058619092	0.15022775	PREDICTED: intraflagellar transport protein 22 homolog
RS008448	GO:0044699	single-organism process	BP	-0.874319748	-1.179805365	-0.159545862	2.543187294	7.449190097	0.058878449	0.150714469	PREDICTED: facilitated trehalose transporter Tret1-2 homolog
RS009769	GO:0044699	single-organism process	BP	-0.395928216	-0.595374077	-0.361609944	6.747353089	7.447755342	0.058916146	0.150775783	PREDICTED: CREB-binding protein
RS006107	GO:0044699	single-organism process	BP	-0.171681696	0.054277683	0.595145921	4.366410076	7.439495858	0.059133613	0.151213952	PREDICTED: dnaJ homolog subfamily C member 16
RS005466	GO:0044699	single-organism process	BP	0.967187642	0.688490249	0.567140756	6.477903469	7.433729173	0.059285907	0.151511102	PREDICTED: slit homolog 1 protein
RS006100	GO:0044699	single-organism process	BP	0.421624175	0.050581823	-0.504986901	8.46224366	7.432655532	0.059314303	0.151544411	PREDICTED: failed axon connections
RS010972	GO:0044699	single-organism process	BP	0.104108788	-1.061139396	-0.662375696	3.700090739	7.413436876	0.059824845	0.152609702	PREDICTED: gastrin-releasing peptide receptor-like
RS002799	GO:0044699	single-organism process	BP	-0.659046602	-0.211273757	-0.16274335	6.419710777	7.409924332	0.059918615	0.152787009	PREDICTED: V-type proton ATPase subunit H
RS000995	GO:0044699	single-organism process	BP	-0.530293894	-0.87454566	-0.699019546	4.953108181	7.403657421	0.060086269	0.153183497	colmedin
RS002684	GO:0044699	single-organism process	BP	0.147173374	-0.355631952	-0.26819976	5.677394813	7.400935341	0.060159233	0.153276427	PREDICTED: protein phosphatase methylesterase 1
RS005737	GO:0044699	single-organism process	BP	0.302243019	-0.40559564	-0.905264064	4.115692831	7.398482299	0.060225059	0.153382081	PREDICTED: protein jagged-1
RS010786	GO:0044699	single-organism process	BP	0.093212042	0.39376767	0.547822381	6.051605832	7.383979187	0.06061567	0.15414857	PREDICTED: BET1 homolog
RS012405	GO:0044699	single-organism process	BP	-0.931163046	-0.915195858	-0.91055185	1.793494323	7.379252012	0.060743517	0.154296864	PREDICTED: orexin receptor type 1-like
RS013100	GO:0044699	single-organism process	BP	-0.492079473	-0.824138133	-0.742917603	4.396518564	7.37502299	0.060858113	0.154525619	PREDICTED: sarcosine dehydrogenase, mitochondrial
RS008853	GO:0044699	single-organism process	BP	-0.347268363	-0.073583389	0.495367682	8.287236859	7.368575082	0.061033239	0.154845408	PREDICTED: NADH dehydrogenase [ubiquinone] 1 alpha subcomplex subunit 10, mitochondrial
RS000535	GO:0044699	single-organism process	BP	-0.375887342	-1.106633389	-1.780301278	3.250236621	7.367872547	0.061052349	0.15485807	PREDICTED: LIRP-like
RS013516	GO:0044699	single-organism process	BP	-0.071257629	-0.443466359	-0.469509849	6.036825379	7.367035791	0.061075118	0.15485807	PREDICTED: exostosin-3
RS015425	GO:0044699	single-organism process	BP	-0.493172114	-1.644318381	-1.432561787	1.899493423	7.36634539	0.061093911	0.15487454	PREDICTED: protein Wnt-6
RS005808	GO:0044699	single-organism process	BP	-0.542979089	-0.958110907	-1.208799287	1.800811493	7.365296655	0.061122468	0.15488458	PREDICTED: disintegrin and metalloproteinase domain-containing protein 11
RS006544	GO:0044699	single-organism process	BP	0.228299441	0.486965949	0.575473907	5.998771703	7.363808306	0.061163019	0.154924991	PREDICTED: elongation factor Tu, mitochondrial
RS005172	GO:0044699	single-organism process	BP	0.362832633	-1.252356801	-2.026621278	0.54057572	7.34273227	0.061740049	0.156198106	PREDICTED: homeobox protein engrailed-1a-like
RS004018	GO:0044699	single-organism process	BP	-0.854655712	-0.687918747	0.356622885	2.251863917	7.340140368	0.061811374	0.156330055	PREDICTED: potassium/sodium hyperpolarization-activated cyclic nucleotide-gated channel 2
RS002292	GO:0044699	single-organism process	BP	-0.849226044	-0.724590211	-0.752733052	3.999828127	7.339934983	0.06181703	0.156330055	V-type proton ATPase subunit S1
RS010902	GO:0044699	single-organism process	BP	-0.413608261	-0.854721106	-0.933757255	3.754238005	7.337994128	0.061870497	0.156389668	PREDICTED: glutamate receptor 1
RS000570	GO:0044699	single-organism process	BP	0.518702229	0.116476368	-0.226939285	5.273182682	7.320315563	0.062359576	0.157266002	PREDICTED: peroxisomal membrane protein PEX16
RS012830	GO:0044699	single-organism process	BP	0.512471195	0.69308213	0.478993909	7.410547394	7.303674851	0.062823366	0.158207852	PREDICTED: glycosyltransferase-like protein LARGE1
RS015346	GO:0044699	single-organism process	BP	-0.035897868	0.497726136	0.291117237	6.601582051	7.299752842	0.062933161	0.158389335	PREDICTED: cystathionine gamma-lyase
RS015431	GO:0044699	single-organism process	BP	-1.315909641	-2.868077276	-0.442125086	1.513185213	7.297381393	0.062999639	0.158501028	PREDICTED: nucleoside diphosphate kinase homolog 5-like
RS012388	GO:0044699	single-organism process	BP	-0.033544073	1.164865903	0.505870592	2.336669345	7.291026055	0.063178132	0.158815449	PREDICTED: transforming growth factor beta receptor type 3
RS012800	GO:0044699	single-organism process	BP	0.051891265	0.181532958	0.60875329	6.827676128	7.290457164	0.063194134	0.158823972	PREDICTED: major facilitator superfamily domain-containing protein 8
RS010104	GO:0044699	single-organism process	BP	0.474163025	0.520379393	0.535549754	5.754453844	7.288350376	0.063253427	0.158909567	PREDICTED: fas-associated death domain protein
RS000090	GO:0044699	single-organism process	BP	-0.660367689	-0.043314301	0.197399386	5.536396839	7.284013478	0.063375653	0.15914734	PREDICTED: integrin beta-PS
RS000192	GO:0044699	single-organism process	BP	0.64926176	-0.741502205	-0.554920299	2.15935723	7.277266734	0.063566251	0.159504556	PREDICTED: cell division cycle 7-related protein kinase-like
RS006810	GO:0044699	single-organism process	BP	-0.692918241	-0.709642301	-0.469276201	5.552917252	7.276110601	0.063598968	0.159523084	PREDICTED: Y+L amino acid transporter 2
RS003797	GO:0044699	single-organism process	BP	-0.779191865	-0.909904321	-0.8699466	6.927393112	7.246172474	0.064451878	0.1612129	PREDICTED: 4-aminobutyrate aminotransferase, mitochondrial
RS015303	GO:0044699	single-organism process	BP	-0.508205047	-0.927948724	-1.998037566	0.782466667	7.245628342	0.064467482	0.16121991	PREDICTED: leucine-rich repeat transmembrane neuronal protein 4

RS007579	GO:0044699	single-organism process	BP	-0.161689677	-0.938732224	-1.006211109	3.317416238	7.24303802	0.064541814	0.161373755	PREDICTED: WD repeat-containing protein 19
RS006920	GO:0044699	single-organism process	BP	-0.632866598	-0.974633547	-0.967627708	3.248752993	7.241310635	0.064591429	0.161433709	PREDICTED: glutamate receptor ionotropic, NMDA 2B
RS011743	GO:0044699	single-organism process	BP	0.613155845	0.739434445	0.12169262	4.329604651	7.238987185	0.064658223	0.161568584	PREDICTED: vacuole membrane protein 1
RS008996	GO:0044699	single-organism process	BP	-0.818894069	-0.144607229	0.175883673	2.981815036	7.237819263	0.064691824	0.161620478	PREDICTED: cytochrome c oxidase assembly factor 6 homolog
RS004181	GO:0044699	single-organism process	BP	0.712841894	0.728346217	0.487631593	7.763445766	7.232819048	0.064835869	0.161916108	PREDICTED: DNA-directed RNA polymerase, mitochondrial
RS002605	GO:0044699	single-organism process	BP	-0.518735488	0.392463203	-0.08725497	4.03235098	7.22499608	0.065061855	0.162287383	PREDICTED: solute carrier family 40 member 1
RS000609	GO:0044699	single-organism process	BP	0.612008555	0.732502903	0.190053663	8.31658549	7.219606678	0.065217984	0.162580225	PREDICTED: muscle calcium channel subunit alpha-1
RS005953	GO:0044699	single-organism process	BP	0.473941806	-0.137921347	-0.448948452	4.899827349	7.212284421	0.06543069	0.162949201	PREDICTED: dynein heavy chain 7, axonemal
RS012154	GO:0044699	single-organism process	BP	0.444265091	0.153308266	-0.1266241	6.291615632	7.206731242	0.065592453	0.163287479	PREDICTED: methyl-CpG-binding domain protein 4
RS006327	GO:0044699	single-organism process	BP	0.513873286	-0.208197793	-0.634892764	3.419932453	7.203690519	0.065681193	0.163436151	PREDICTED: ecdysone 20-monooxygenase
RS006503	GO:0044699	single-organism process	BP	-0.789357073	-0.069673576	-0.042357301	4.655019811	7.200877419	0.065763394	0.163551378	PREDICTED: transient receptor potential cation channel trpm
RS015164	GO:0044699	single-organism process	BP	0.689107788	0.434945645	0.336893172	5.916865798	7.194398876	0.06595308	0.163958366	PREDICTED: protein arginine N-methyltransferase 6
RS006781	GO:0044699	single-organism process	BP	0.526897874	0.596395483	0.356615489	6.037413706	7.193909569	0.065967428	0.16396167	PREDICTED: ras-related protein Rab-28-like
RS015105	GO:0044699	single-organism process	BP	-0.678666411	-2.224992259	-0.562865466	0.559101899	7.185153044	0.066224707	0.164438873	PREDICTED: organic cation transporter 1
RS000480	GO:0044699	single-organism process	BP	-0.001096276	0.487561407	0.488428304	8.018008075	7.180741969	0.066354679	0.164716286	PREDICTED: signal recognition particle receptor subunit alpha homolog
RS009995	GO:0044699	single-organism process	BP	-0.926877817	0.790504308	1.027579647	0.931565014	7.179681543	0.066385961	0.164741831	PREDICTED: ras-related protein Rab-30
RS005264	GO:0044699	single-organism process	BP	-1.488549143	-1.308665565	-1.379377385	1.600885014	7.17732657	0.066455483	0.164816926	PREDICTED: sodium-dependent neutral amino acid transporter B(0)AT3
RS005580	GO:0044699	single-organism process	BP	0.50168489	0.255888995	-0.148930096	4.887037059	7.175704799	0.0665034	0.164870832	PREDICTED: DNA polymerase subunit gamma-2, mitochondrial
RS000758	GO:0044699	single-organism process	BP	0.436016025	0.658391235	0.438858138	7.421745581	7.173549798	0.066567125	0.164996334	PREDICTED: kynurenine--oxoglutarate transaminase 3
RS007230	GO:0044699	single-organism process	BP	-0.595941154	-0.25795038	0.362903371	9.093119676	7.171002242	0.066642534	0.165140653	PREDICTED: isocitrate dehydrogenase [NAD] subunit beta, mitochondrial
RS012628	GO:0044699	single-organism process	BP	-0.579111491	-0.949115471	-0.471871476	9.193123975	7.165063336	0.06681865	0.165404616	PREDICTED: sterile alpha and TIR motif-containing protein 1
RS013435	GO:0044699	single-organism process	BP	0.372961317	-0.259369735	-0.262791659	4.32688553	7.159682329	0.06697861	0.165690162	PREDICTED: nudC domain-containing protein 3
RS015010	GO:0044699	single-organism process	BP	-0.519984491	-0.303036255	-1.027229467	4.186866021	7.157457195	0.067044865	0.165788931	PREDICTED: guanine nucleotide-binding protein G(o) subunit alpha
RS000452	GO:0044699	single-organism process	BP	-0.553587383	0.348993435	0.009026134	3.540268279	7.135177972	0.067711748	0.167011702	PREDICTED: GTP-binding protein Rit2
RS006739	GO:0044699	single-organism process	BP	-0.215562063	-0.194338635	-0.661480143	5.23328973	7.126492884	0.067973454	0.167558754	PREDICTED: potassium voltage-gated channel protein Shab
RS014661	GO:0044699	single-organism process	BP	-0.982950645	-0.377451355	-0.15917073	2.646015483	7.120938203	0.068141345	0.167841207	PREDICTED: sialin
RS000133	GO:0044699	single-organism process	BP	-0.045454902	0.182331435	0.994722318	2.687399163	7.116976504	0.068261333	0.168071011	PREDICTED: putative ammonium transporter 1
RS012960	GO:0044699	single-organism process	BP	-0.376002699	-0.49425854	-0.692985711	4.353867983	7.106069265	0.068592737	0.168784702	PREDICTED: probable cation-transporting ATPase 13A3
RS013301	GO:0044699	single-organism process	BP	0.293016657	0.414659226	0.757851184	6.361707162	7.096024609	0.06889931	0.169277783	PREDICTED: metalloendopeptidase OMA1, mitochondrial
RS012610	GO:0044699	single-organism process	BP	-2.163853558	-1.407578521	-1.490670102	1.440727739	7.082903676	0.069301772	0.170166995	PREDICTED: arrestin homolog
RS005368	GO:0044699	single-organism process	BP	-0.867637256	-0.828889719	-0.697658978	3.330677082	7.082129861	0.069325578	0.170192268	PREDICTED: serine/threonine-protein kinase Nek8-like
RS007029	GO:0044699	single-organism process	BP	0.30951981	-0.243087357	-0.216035825	5.153399253	7.07759246	0.06946533	0.170468895	PREDICTED: leucine-rich repeat-containing protein 58
RS009815	GO:0044699	single-organism process	BP	-1.467100978	-1.416460789	-1.526760447	0.458433519	7.069890334	0.06970318	0.171019257	trissin precursor
RS014164	GO:0044699	single-organism process	BP	-0.323373255	-0.480954149	-0.674381743	7.10845976	7.054385301	0.070184382	0.171965391	PREDICTED: exostosin-2
RS006462	GO:0044699	single-organism process	BP	-0.413832115	-0.032686193	-1.689293195	1.347147133	7.047382369	0.070402772	0.172232422	PREDICTED: homeobox protein slou-like
RS001963	GO:0044699	single-organism process	BP	-0.727767318	-1.343201941	-1.251547757	1.437734943	7.043327631	0.070529521	0.172442008	Neuromedin-B receptor
RS003660	GO:0044699	single-organism process	BP	0.41031539	0.497158098	0.06003262	6.120516822	7.043694021	0.070518059	0.172442008	PREDICTED: DNA-directed RNA polymerase I subunit RPA49-like
RS007710	GO:0044699	single-organism process	BP	0.254808015	0.164133518	0.635618538	4.712550891	7.037094945	0.070724782	0.172885851	PREDICTED: alpha-ketoglutarate-dependent dioxygenase alkB homolog 7, mitochondrial
RS011063	GO:0044699	single-organism process	BP	-0.677442669	-0.379381664	-0.180305524	5.081987275	7.036051205	0.070757532	0.172932342	7-dehydrocholesterol reductase
RS015404	GO:0044699	single-organism process	BP	-0.033173942	0.491254687	-0.13268681	6.364272838	7.035330626	0.07078015	0.172954058	PREDICTED: sodium/potassium-transporting ATPase subunit beta-2
RS013008	GO:0044699	single-organism process	BP	0.700396501	0.575214376	0.540127857	4.751571873	7.034606657	0.070802882	0.172976043	PREDICTED: probable phenylalanine--tRNA ligase alpha subunit
RS013479	GO:0044699	single-organism process	BP	-0.912044125	-0.88198527	-0.393912656	3.548387807	7.023342256	0.071157485	0.173572994	ace-paralogous acetylcholinesterase
RS003449	GO:0044699	single-organism process	BP	-0.271604031	-0.623910119	-0.38398901	6.1655336	7.019104607	0.07129133	0.173865804	PREDICTED: glutamate receptor ionotropic, kainate 2
RS003560	GO:0044699	single-organism process	BP	-0.560443517	-0.851259813	-1.019009863	3.375107429	7.009139154	0.071607046	0.174568165	G/T mismatch-specific thymine DNA glycosylase
RS014295	GO:0044699	single-organism process	BP	0.412981653	0.489533068	0.464743455	5.949145628	7.0080526	0.071641551	0.174618482	PREDICTED: zinc transporter 5
RS003688	GO:0044699	single-organism process	BP	0.121215068	-1.088834194	-0.415477272	3.417019683	6.997230352	0.071986103	0.175220909	PREDICTED: probable cytochrome P450 49a1
RS002642	GO:0044699	single-organism process	BP	0.38648925	0.622840374	0.048143591	4.971006951	6.995050777	0.072055688	0.175356394	PREDICTED: acetyl-CoA acetyltransferase, cytosolic
RS011623	GO:0044699	single-organism process	BP	0.600618447	-0.341170034	-0.132850423	2.74046665	6.982416041	0.07246035	0.176204991	PREDICTED: baculoviral IAP repeat-containing protein 5
RS004794	GO:0044699	single-organism process	BP	-1.194960627	-1.570619893	-1.298915827	0.604298427	6.98008147	0.072535361	0.176353346	PREDICTED: probable G-protein coupled receptor 158
RS000729	GO:0044699	single-organism process	BP	0.36128273	0.630576784	0.108395063	6.09974523	6.970092612	0.072857156	0.176930781	PREDICTED: surfeit locus protein 4 homolog
RS012985	GO:0044699	single-organism process	BP	-0.969985528	-0.457725587	-0.632358924	3.192395806	6.96564327	0.073000938	0.177211606	PREDICTED: discoidin domain-containing receptor 2-like
RS011306	GO:0044699	single-organism process	BP	-0.537330602	0.387024584	1.020375573	1.123255473	6.964902709	0.073024896	0.177235602	PREDICTED: cytochrome P450 4c3
RS004371	GO:0044699	single-organism process	BP	-0.114512892	-0.471597485	-0.476274739	6.333445083	6.937918615	0.073903063	0.178831489	PREDICTED: structural maintenance of chromosomes protein 3
RS005477	GO:0044699	single-organism process	BP	0.511856699	0.424979628	0.431601432	6.288256837	6.930446026	0.074148048	0.179305015	PREDICTED: trafficking protein particle complex subunit 5
RS010328	GO:0044699	single-organism process	BP	-0.290368041	-0.923328347	-0.569995373	3.378511577	6.926120151	0.074290228	0.179545411	PREDICTED: ubiquitin carboxyl-terminal hydrolase 37
RS014190	GO:0044699	single-organism process	BP	-0.446715996	-0.848688203	-0.433442952	9.178280474	6.924826259	0.074332806	0.179575887	PREDICTED: probable phospholipid hydroperoxide glutathione peroxidase
RS006016	GO:0044699	single-organism process	BP	-0.615049522	-0.707734062	-0.49788903	4.507497874	6.924437162	0.074345615	0.179575887	PREDICTED: ORM1-like protein 3
RS009219	GO:0044699	single-organism process	BP	-1.093292862	-1.25664422	-0.723042855	1.921374802	6.915865584	0.074628322	0.18005155	PREDICTED: protein sprouty
RS000909	GO:0044699	single-organism process	BP	-0.60899635	-0.75123901	0.2180877	2.813140803	6.916479696	0.074608033	0.18005155	PREDICTED: mitochondrial uncoupling protein 2-like
RS003825	GO:0044699	single-organism process	BP	-0.116552603	-0.74841501	-0.195189608	7.935069367	6.915883201	0.07462774	0.18005155	PREDICTED: H(+)/Cl(-) exchange transporter 7
RS011014	GO:0044699	single-organism process	BP	-0.505402207	0.19156047	0.062497002	5.153414157	6.906978993	0.074922516	0.180692103	PREDICTED: transcription factor 21
RS008028	GO:0044699	single-organism process	BP	0.080000091	-0.951859812	-0.482192541	2.340477948	6.904949413	0.074989863	0.180819898	target of wingless
RS014119	GO:0044699	single-organism process	BP	-0.394332155	-0.685680238	0.020826764	4.620891066	6.896383256	0.075274755	0.181356544	PREDICTED: oxysterol-binding protein-related protein 8
RS003462	GO:0044699	single-organism process	BP	0.697721441	0.025606853	-0.264330568	4.0131306	6.886632368	0.075600322	0.181943522	PREDICTED: cysteine--tRNA ligase, cytoplasmic
RS013768	GO:0044699	single-organism process	BP	0.510925536	0.38519901	-0.038122074	4.699791831	6.884520717	0.075671005	0.182042975	PREDICTED: serine--tRNA ligase, mitochondrial
RS007855	GO:0044699	single-organism process	BP	-1.424425864	-1.269720145	-1.006702932	1.128088952	6.877386185	0.075910293	0.182420197	PREDICTED: gamma-aminobutyric acid type B receptor subunit 2
RS009627	GO:0044699	single-organism process	BP	0.274232803	0.091820577	1.094974551	11.41302523	6.866987602	0.076260363	0.183077205	PREDICTED: heat shock 70 kDa protein cognate 4
RS005611	GO:0044699	single-organism process	BP	-0.853521593	-0.896293105	-0.339873022	4.001870039	6.863381331	0.076382133	0.183264733	PREDICTED: ras-related protein Rab-32
RS002500	GO:0044699	single-organism process	BP	0.488671628	0.505919803	0.380713572	6.032071153	6.857741521	0.076572944	0.183547707	PREDICTED: gamma-secretase subunit pen-2
RS002556	GO:0044699	single-organism process	BP	-0.245828331	-0.654733652	-0.414341086	5.508180753	6.856253413	0.076623367	0.183618406	PREDICTED: inositol polyphosphate 5-phosphatase OCRL-1
RS010959	GO:0044699	single-organism process	BP	-0.01139964	0.540038099	0.153812167	4.863464778	6.854286188	0.076690074	0.183723568	PREDICTED: probable sulfite oxidase, mitochondrial
RS004291	GO:0044699	single-organism process	BP	0.781190319	-1.037280119	-0.352171624	0.672722301	6.843951183	0.07704145	0.184460079	PREDICTED: synaptic vesicle glycoprotein 2B-like
RS007471	GO:0044699	single-organism process	BP	-0.166455797	0.50637605	-0.01116969	8.121929428	6.835928055	0.077315296	0.184975082	PREDICTED: lambda-crystallin homolog
RS007094	GO:0044699	single-organism process	BP	0.028984738	-2.016221238	-1.615755551	0.158728832	6.830527417	0.077500159	0.185311753	Protein Wnt-5a
RS009169	GO:0044699	single-organism process	BP	0.588023497	0.240183125	-0.031263491	4.026762678	6.821027282	0.077826384	0.18605647	PREDICTED: protein cornichon
RS000292	GO:0044699	single-organism process	BP	-0.077606235	-0.220452652	-0.638433725	4.826834935	6.819185275	0.07788979	0.186102071	PREDICTED: alpha-ketoglutarate-dependent dioxygenase alkB homolog 4
RS004888	GO:0044699	single-organism process	BP	0.119671255	-0.203948356	-0.501086439	5.219576584	6.818135325	0.077925954	0.186117858	PREDICTED: nuclear autoantigenic sperm protein
RS004873	GO:0044699	single-organism process	BP	-0.321107174	-0.728253018	-0.434082813	4.829123654	6.815753931	0.078008038	0.186207965	PREDICTED: centrosomal protein of 131 kDa
RS002341	GO:0044699	single-organism process	BP	0.503403219	0.718208946	0.415316153	7.707757154	6.80564451	0.078357426	0.186758785	PREDICTED: leucine--tRNA ligase, cytoplasmic
RS003427	GO:0044699	single-organism process	BP	-0.513964722	-0.550228674	-0.09809219	5.664872118	6.801016027	0.078517892	0.186983778	PREDICTED: voltage-dependent calcium channel type A subunit alpha-1
RS013090	GO:0044699	single-organism process	BP	-0.401051453	-0.648615716	-0.200667915	5.427851955	6.796633362	0.078670129	0.187220636	PREDICTED: linear gramicidin synthase subunit D-like
RS002662	GO:0044699	single-organism process	BP	0.548562285	0.643121214	0.480591626	7.386807743	6.793922491	0.078764437	0.18733887	PREDICTED: phosphorylase b kinase gamma catalytic chain, skeletal muscle/heart isoform
RS012028	GO:0044699	single-organism process	BP	0.541573501	0.614749029	0.44673468	5.702316197	6.784699514	0.079086109	0.187995021	PREDICTED: mitochondrial import inner membrane translocase subunit TIM44
RS013443	GO:0044699	single-organism process	BP	-0.491548722	-0.260820185	-0.544882026	5.768203832	6.776025779	0.079389782	0.188541376	PREDICTED: unconventional myosin-IXa-like
RS014551	GO:0044699	single-organism process	BP	-0.502782747	-0.474145105	0.559209026	3.220569618	6.760015845	0.079953256	0.189622234	PREDICTED: MFS-type transporter SLC18B1-like
RS000856	GO:0044699	single-organism process	BP	0.50025501	0.656647327	0.389966239	5.460683953	6.755556114	0.080110904	0.189895863	PREDICTED: presenilin-1
RS011679	GO:0044699	single-organism process	BP	-0.054688789	-0.103097471	-0.893891243	3.451449564	6.752534052	0.080217901	0.190077939	PREDICTED: N-acetylgalactosamine kinase
RS002296	GO:0044699	single-organism process	BP	0.050929101	-1.155041098	-1.453067074	0.872761787	6.743534644	0.080537346	0.190763087	PREDICTED: glycine receptor subunit alpha-4
RS009997	GO:0044699	single-organism process	BP	0.343885955	0.512684404	0.747641932	4.621337129	6.741569173	0.080607276	0.190892823	PREDICTED: probable asparagine--tRNA ligase, mitochondrial
RS005549	GO:0044699	single-organism process	BP	0.522647095	0.199448201	-0.050575822	4.924616279	6.735233086	0.080833107	0.191247821	PREDICTED: cell cycle checkpoint protein RAD1
RS002615	GO:0044699	single-organism process	BP	-0.06386033	-0.985822281	-0.297735703	2.791023397	6.734264746	0.080867675	0.191293669	PREDICTED: gastrin-releasing peptide receptor-like
RS012374	GO:0044699	single-organism process	BP	0.63903748	0.325681205	0.279143903	5.091561417	6.731419919	0.080969311	0.191498121	PREDICTED: intraflagellar transport protein 43 homolog
RS006344	GO:0044699	single-organism process	BP	0.529733961	0.375791502	0.226115552	6.083041877	6.727616725	0.081105378	0.191711924	PREDICTED: centromere/kinetochore protein zw10 homolog
RS009043	GO:0044699	single-organism process	BP	0.166334153	0.474828815	0.489350025	5.98714761	6.719718617	0.081388654	0.192165113	PREDICTED: nucleoside diphosphate-linked moiety X motif 17
RS002422	GO:0044699	single-organism process	BP	0.174915618	0.361222955	0.71291441	7.775093031	6.719016781	0.081413873	0.192188625	PREDICTED: NADH dehydrogenase [ubiquinone] iron-sulfur protein 5-like
RS008960	GO:0044699	single-organism process	BP	0.090988571	0.403212139	-0.202866112	7.716840214	6.716670207	0.081498245	0.192315701	PREDICTED: coatomer subunit gamma
RS000293	GO:0044699	single-organism process	BP	0.419346925	-0.045258063	-0.727855325	2.968837848	6.716868372	0.081491116	0.192315701	PREDICTED: TNF receptor-associated factor 4
RS003145	GO:0044699	single-organism process	BP	-1.309880541	-0.628756059	-0.991127574	1.553035926	6.713260678	0.081620986	0.19245815	PREDICTED: neuronal acetylcholine receptor subunit alpha-7-like
RS003697	GO:0044699	single-organism process	BP	-0.943247872	-1.064539386	-0.892443959	2.112477374	6.708443234	0.081794715	0.192654319	PREDICTED: microcephalin-like
RS012825	GO:0044699	single-organism process	BP	0.519876278	0.380407444	0.607764994	5.674018807	6.701163938	0.082057901	0.193238072	PREDICTED: lipoyl synthase, mitochondrial
RS012048	GO:0044699	single-organism process	BP	-0.674894361	-1.155560739	-0.486644875	2.649535097	6.691851744	0.082395778	0.193852499	PREDICTED: sodium-independent sulfate anion transporter-like
RS006778	GO:0044699	single-organism process	BP	-0.217267845	-0.468162815	-0.605610758	6.34143178	6.692271923	0.082380504	0.193852499	RNA polymerase II second largest subunit
RS011039	GO:0044699	single-organism process	BP	-0.244819279	-0.634405128	-0.5346659	4.962293715	6.690040947	0.082461635	0.193971205	PREDICTED: enoyl-CoA delta isomerase 2, mitochondrial
RS002152	GO:0044699	single-organism process	BP	0.266936548	0.870150214	0.249395764	3.060745526	6.686619633	0.082586204	0.194227947	PREDICTED: cytoplasmic dynein 2 light intermediate chain 1
RS009471	GO:0044699	single-organism process	BP	0.454440582	0.068570398	0.92902968	1.975531925	6.680170813	0.082821497	0.194672252	PREDICTED: potassium voltage-gated channel protein Shal
RS012176	GO:0044699	single-organism process	BP	0.51158448	0.516166901	0.483188159	6.915068808	6.678947522	0.082866203	0.194704656	PREDICTED: casein kinase II subunit beta
RS002982	GO:0044699	single-organism process	BP	0.437159386	0.690352357	0.482073212	5.918755174	6.64375724	0.084162258	0.197455193	PREDICTED: multidrug resistance-associated protein 5-like
RS005486	GO:0044699	single-organism process	BP	-1.503171466	-1.396938356	-1.46976547	1.83314743	6.637867085	0.084381091	0.197821196	PREDICTED: PDF receptor
RS002576	GO:0044699	single-organism process	BP	-0.620693831	-0.682180231	-0.612309734	6.887876847	6.631270757	0.084626811	0.198360331	PREDICTED: NADP-dependent malic enzyme
RS005349	GO:0044699	single-organism process	BP	0.450765862	0.364329504	0.520620409	5.976312653	6.621075578	0.085007948	0.199050612	PREDICTED: F-box/WD repeat-containing protein 7
RS005939	GO:0044699	single-organism process	BP	-0.197409925	-0.641883766	-0.472598309	4.303446703	6.605077678	0.08560935	0.200216184	PREDICTED: serologically defined colon cancer antigen 8 homolog

RS002289	GO:0044699	single-organism process	BP	0.240927005	-0.159443346	-0.419758048	5.335124408	6.602382935	0.085711054	0.200416824	PREDICTED: DNA-(apurinic or apyrimidinic site) lyase 2
RS000035	GO:0044699	single-organism process	BP	-0.59529335	-0.304820032	0.347972721	8.866264808	6.597065948	0.085912068	0.200794204	PREDICTED: cytochrome b-c1 complex subunit Rieske, mitochondrial-like
RS003791	GO:0044699	single-organism process	BP	-0.678787605	-0.019757464	-0.126441983	4.54718096	6.597089055	0.085911194	0.200794204	PREDICTED: guanylate cyclase 32E
RS004850	GO:0044699	single-organism process	BP	0.061794982	-0.298019961	-0.539408818	4.403735747	6.584518292	0.086388247	0.201663314	PREDICTED: negative elongation factor B
RS009122	GO:0044699	single-organism process	BP	0.244148657	0.447879654	0.657487362	4.861582845	6.566356812	0.08708197	0.202944177	PREDICTED: zinc finger protein 543-like
RS013666	GO:0044699	single-organism process	BP	-1.146187094	0.124030689	-1.340835201	0.632768848	6.551429578	0.087656166	0.203956157	PREDICTED: adhesion G protein-coupled receptor L2-like
RS008514	GO:0044699	single-organism process	BP	-0.150431282	-0.260030975	0.291128497	5.641661731	6.546812332	0.087834511	0.204267946	PREDICTED: saccharopine dehydrogenase-like oxidoreductase
RS012134	GO:0044699	single-organism process	BP	0.326084156	-1.133391396	-0.337804959	1.638216142	6.546550782	0.087844624	0.204267946	PREDICTED: thymidine kinase, cytosolic
RS000318	GO:0044699	single-organism process	BP	-0.409411201	-0.696964023	-0.524046279	5.146315283	6.540394841	0.088082972	0.204696733	PREDICTED: alpha-aminoadipic semialdehyde dehydrogenase
RS009403	GO:0044699	single-organism process	BP	-1.114148368	-0.446207588	-1.216817584	1.305782432	6.540109468	0.088094036	0.204696733	Pax9
RS008478	GO:0044699	single-organism process	BP	-0.36286792	0.257674612	-0.077485053	6.266919719	6.539508192	0.088117353	0.204713142	PREDICTED: ribosomal protein S6 kinase alpha-5
RS002854	GO:0044699	single-organism process	BP	0.231156139	0.235908421	0.689826813	6.843181501	6.51305293	0.089149148	0.206652745	PREDICTED: mitochondrial inner membrane protease ATP23 homolog
RS015377	GO:0044699	single-organism process	BP	0.064687264	-0.135391647	-0.540226237	5.050775931	6.503445798	0.089526709	0.207299021	PREDICTED: probable glutamine-dependent NAD(+) synthetase
RS010721	GO:0044699	single-organism process	BP	0.47718921	-0.210912584	-0.002742229	5.164624711	6.501021843	0.089622214	0.207405763	PREDICTED: thioredoxin domain-containing protein 5
RS005573	GO:0044699	single-organism process	BP	0.321059524	0.53789242	0.37867597	6.429686541	6.49661781	0.089795985	0.20770489	PREDICTED: exocyst complex component 7
RS012259	GO:0044699	single-organism process	BP	-0.47689811	-0.550626227	-0.829212685	8.367846941	6.487657393	0.090150539	0.2083745	PREDICTED: putative glutamate synthase [NADPH]
RS004374	GO:0044699	single-organism process	BP	0.389774982	0.318158623	-0.152087121	5.582839423	6.483562425	0.09031302	0.208509549	PREDICTED: transmembrane emp24 domain-containing protein eca
RS003275	GO:0044699	single-organism process	BP	-0.32112423	-1.225335524	-0.48854162	1.810755093	6.476832132	0.090580679	0.209047658	PREDICTED: metabotropic glutamate receptor 1
RS011292	GO:0044699	single-organism process	BP	-1.41507544	-0.227362679	0.078175978	1.07662831	6.474948975	0.090655707	0.20918248	PREDICTED: headcase protein
RS003413	GO:0044699	single-organism process	BP	0.449978058	0.401321124	-0.013517671	6.374995689	6.473027673	0.090732317	0.209282564	PREDICTED: asparagine--tRNA ligase, cytoplasmic
RS001410	GO:0044699	single-organism process	BP	-0.833940378	-1.145871699	-0.470397772	2.458966391	6.454844784	0.091460426	0.210576345	PREDICTED: globin
RS004460	GO:0044699	single-organism process	BP	-0.079558878	0.148430003	-0.716345774	4.095003994	6.452192903	0.091567085	0.21074486	PREDICTED: MTSS1-like protein
RS001821	GO:0044699	single-organism process	BP	-0.416559115	-0.243117375	0.420607418	9.114591662	6.45061119	0.091630759	0.210852874	PREDICTED: cytochrome c oxidase subunit 4 isoform 1, mitochondrial
RS010984	GO:0044699	single-organism process	BP	-0.275678709	0.022395231	0.433282592	7.347025378	6.449036943	0.091694175	0.210960255	PREDICTED: probable NADH dehydrogenase [ubiquinone] 1 alpha subcomplex subunit 12
RS004484	GO:0044699	single-organism process	BP	-0.568391639	-0.664978914	-0.194206782	3.934285841	6.441114928	0.092013938	0.211541355	hypothetical protein
RS005788	GO:0044699	single-organism process	BP	0.578132848	0.243978273	-0.277885533	4.597372349	6.42881166	0.092512673	0.212416519	PREDICTED: frizzled-4
RS003393	GO:0044699	single-organism process	BP	0.415730181	0.169253589	-0.163673807	6.750170107	6.428868474	0.092510364	0.212416519	PREDICTED: leukotriene A-4 hydrolase
RS003508	GO:0044699	single-organism process	BP	-0.169271487	0.401452192	-0.239447825	4.192461457	6.422099786	0.092785845	0.212888492	PREDICTED: organic cation transporter 1-like
RS001873	GO:0044699	single-organism process	BP	0.173459976	-0.247876129	-0.411131878	5.728085444	6.416248826	0.09302461	0.213397439	PREDICTED: protein sly1 homolog
RS015472	GO:0044699	single-organism process	BP	-0.630677411	-0.397195879	-0.181320944	4.290556076	6.413140935	0.093151676	0.213544767	PREDICTED: dedicator of cytokinesis protein 1
RS008224	GO:0044699	single-organism process	BP	0.284136312	0.497342106	0.600474921	5.055240003	6.411920646	0.093201614	0.213608939	PREDICTED: notchless protein homolog 1
RS008061	GO:0044699	single-organism process	BP	-0.693395661	-0.146881088	-0.693247085	3.663791087	6.405616828	0.093459991	0.214053476	GD17939
RS007788	GO:0044699	single-organism process	BP	-0.078223265	-0.643029265	-0.327697088	5.100907289	6.405454125	0.093466669	0.214053476	PREDICTED: src substrate cortactin
RS000279	GO:0044699	single-organism process	BP	-0.156609271	-0.975620437	-1.13346223	1.519250101	6.401800896	0.093616731	0.214191532	PREDICTED: glycerophosphodiester phosphodiesterase domain-containing protein 1-like
RS010561	GO:0044699	single-organism process	BP	0.077383384	1.01828952	0.325351919	10.99254901	6.38788389	0.094190516	0.215171867	PREDICTED: tubulin alpha-1 chain
RS006789	GO:0044699	single-organism process	BP	-0.855729601	-1.129016513	-0.749857227	2.631838766	6.387043941	0.094225254	0.215171867	PREDICTED: serine/threonine-protein kinase 32A
RS005635	GO:0044699	single-organism process	BP	-0.897094157	-0.864685475	-0.332013128	3.330968755	6.387093907	0.094223187	0.215171867	PREDICTED: dedicator of cytokinesis protein 3
RS007738	GO:0044699	single-organism process	BP	-2.236721279	-1.816342735	0.104485909	0.00738012	6.381695156	0.094446755	0.215638584	PREDICTED: dynein heavy chain 10, axonemal
RS005623	GO:0044699	single-organism process	BP	-0.868293114	-0.040367169	-0.805108588	4.47095866	6.376337371	0.09466913	0.215950557	PREDICTED: sphingomyelin phosphodiesterase
RS014213	GO:0044699	single-organism process	BP	0.613464183	0.699717114	0.629898893	4.817107609	6.372622901	0.094823595	0.216185437	PREDICTED: phosphatidylinositol-glycan biosynthesis class F protein
RS006997	GO:0044699	single-organism process	BP	-0.010496639	0.262604646	0.509544222	5.688605341	6.36713105	0.095052414	0.216578909	PREDICTED: charged multivesicular body protein 6
RS014925	GO:0044699	single-organism process	BP	-0.459700932	0.108784662	0.214102952	4.180136496	6.358699728	0.09540474	0.217156569	PREDICTED: RNA demethylase ALKBH5
RS006697	GO:0044699	single-organism process	BP	-0.137394972	-0.578930188	0.149350385	8.454117513	6.353830787	0.095608773	0.21750305	PREDICTED: zinc transporter ZIP13 homolog
RS008551	GO:0044699	single-organism process	BP	-0.043226161	-0.397341447	-0.514548196	6.962546876	6.350807698	0.095735666	0.217634473	PREDICTED: alpha-2-macroglobulin receptor-associated protein
RS014526	GO:0044699	single-organism process	BP	-0.300967845	-0.598909751	-0.228196474	6.554181924	6.351262623	0.095716561	0.217634473	PREDICTED: probable histone-lysine N-methyltransferase CG1716
RS012061	GO:0044699	single-organism process	BP	-0.770276006	-0.094935186	-0.31150429	3.245572638	6.349445225	0.095792909	0.21768821	PREDICTED: 72 kDa inositol polyphosphate 5-phosphatase
RS001568	GO:0044699	single-organism process	BP	-0.664679343	-0.257471752	-0.520595381	5.009946007	6.3478878	0.095858382	0.2177955	PREDICTED: beta-glucuronidase
RS003504	GO:0044699	single-organism process	BP	0.081633737	1.126411428	0.674413768	12.00398265	6.345032644	0.095978522	0.217838484	PREDICTED: tubulin beta-1 chain
RS008449	GO:0044699	single-organism process	BP	-0.853683481	-0.526886778	-1.027937023	2.277355601	6.346530323	0.095915484	0.217838484	PREDICTED: cytochrome P450 4C1-like
RS014141	GO:0044699	single-organism process	BP	-0.505666266	-0.291663535	0.286304823	8.214373843	6.345876379	0.095943004	0.217838484	PREDICTED: succinate dehydrogenase cytochrome b560 subunit, mitochondrial-like
RS015501	GO:0044699	single-organism process	BP	-0.403397557	-0.84271726	-0.472116094	4.072578673	6.344595061	0.095996948	0.217838484	PREDICTED: mitotic spindle assembly checkpoint protein MAD1
RS007190	GO:0044699	single-organism process	BP	-0.878979816	-0.595509543	0.227085088	2.704210207	6.34629855	0.095925237	0.217838484	PREDICTED: sodium channel protein Nach-like
RS001140	GO:0044699	single-organism process	BP	-0.967102714	-1.562646946	-0.525847776	1.042696025	6.341021064	0.096147567	0.218137716	PREDICTED: ras-related protein Rab-37
RS002276	GO:0044699	single-organism process	BP	-0.237172699	0.220188611	0.255899427	6.467350094	6.339825053	0.096198022	0.218212876	PREDICTED: ras-like GTP-binding protein Rho1
RS010275	GO:0044699	single-organism process	BP	-0.327809837	0.151537852	-0.881426827	3.129420115	6.335110353	0.096397162	0.218625222	PREDICTED: equilibrative nucleoside transporter 4
RS005070	GO:0044699	single-organism process	BP	0.362058703	0.570306267	0.494225948	5.36041377	6.334041217	0.096442375	0.218688382	PREDICTED: evolutionarily conserved signaling intermediate in Toll pathway, mitochondrial
RS015389	GO:0044699	single-organism process	BP	-1.014604708	-1.708407111	-0.480189784	0.854123779	6.332870963	0.096491888	0.218718663	PREDICTED: glutamate receptor ionotropic, delta-2
RS000177	GO:0044699	single-organism process	BP	-0.81078162	-0.557481321	-0.666750888	4.385726245	6.332494295	0.09650783	0.218718663	PREDICTED: protein ROP
RS006464	GO:0044699	single-organism process	BP	-0.381451516	-0.307702845	0.198104486	7.290272282	6.321947105	0.09695525	0.219534001	PREDICTED: peroxiredoxin-like
RS002667	GO:0044699	single-organism process	BP	0.618978476	0.38868353	0.054093718	5.478854661	6.321332868	0.096981367	0.219534001	PREDICTED: 46 kDa FK506-binding nuclear protein
RS006385	GO:0044699	single-organism process	BP	-0.838024192	-0.78400016	0.867821594	3.612055094	6.321138979	0.096989613	0.219534001	PREDICTED: odorant receptor coreceptor
RS003658	GO:0044699	single-organism process	BP	-0.041216828	-0.331006227	-0.650832232	8.423732837	6.322317544	0.096939502	0.219534001	PREDICTED: AMP deaminase 2
RS010836	GO:0044699	single-organism process	BP	-0.205213922	-0.639747089	-0.112922325	7.96156317	6.313679716	0.09730735	0.220068025	PREDICTED: protein mahjong
RS009228	GO:0044699	single-organism process	BP	0.586816853	0.889891772	-0.04387833	2.001393975	6.31392221	0.097297005	0.220068025	PREDICTED: guanine nucleotide-binding protein G(o) subunit alpha-like
RS004301	GO:0044699	single-organism process	BP	0.516426719	0.082345289	0.055377044	5.507212987	6.311701946	0.097391763	0.22016727	PREDICTED: actin-like protein 6B
RS007217	GO:0044699	single-organism process	BP	-0.213511141	-0.633476745	-0.712194127	3.426922326	6.306997918	0.097592818	0.220542606	PREDICTED: spermine oxidase-like
RS005313	GO:0044699	single-organism process	BP	-0.428776531	-1.360279383	-0.739073472	3.028416412	6.298699016	0.097948492	0.221259517	PREDICTED: caspase-1-like
RS005104	GO:0044699	single-organism process	BP	0.273973478	0.438590321	-0.388664674	3.999027317	6.296419795	0.098046393	0.221368998	PREDICTED: frizzled-2
RS009797	GO:0044699	single-organism process	BP	0.426579917	0.511875772	0.490593681	4.892308108	6.28964535	0.098337934	0.221920201	PREDICTED: inositol-tetrakisphosphate 1-kinase-like
RS003141	GO:0044699	single-organism process	BP	-0.660872409	-0.640737006	-0.559876126	3.667938691	6.288486219	0.098387901	0.221941111	PREDICTED: DNA repair protein complementing XP-C cells homolog
RS008515	GO:0044699	single-organism process	BP	-0.40213246	-0.709209343	-0.013039291	6.109855772	6.285767151	0.098505208	0.222087323	PREDICTED: saccharopine dehydrogenase-like oxidoreductase
RS010476	GO:0044699	single-organism process	BP	0.62891303	0.463842577	0.253884727	5.644628114	6.285757581	0.098505621	0.222087323	PREDICTED: alanine--tRNA ligase, mitochondrial
RS000296	GO:0044699	single-organism process	BP	-0.154575641	-0.731686684	-0.647867681	5.62740596	6.283265117	0.09861327	0.222250451	PREDICTED: peroxisomal acyl-coenzyme A oxidase 3
RS003087	GO:0044699	single-organism process	BP	0.028997549	0.644052003	-0.01338752	4.256601246	6.270105267	0.099183515	0.223136333	PREDICTED: solute carrier family 35 member E1 homolog
RS007689	GO:0044699	single-organism process	BP	-0.001241457	-0.315989083	-0.51994008	5.021722545	6.270137031	0.099182135	0.223136333	PREDICTED: DNA-directed RNA polymerase I subunit RPA1
RS013640	GO:0044699	single-organism process	BP	-0.29404632	0.060804484	-0.643889244	4.146029871	6.26842733	0.099256451	0.223220669	PREDICTED: alkaline phosphatase-like
RS002385	GO:0044699	single-organism process	BP	0.014701725	-0.545074871	-0.366471276	4.261364025	6.263899379	0.099453526	0.223447811	PREDICTED: short transient receptor potential channel 2-like
RS008832	GO:0044699	single-organism process	BP	0.472745752	0.442092553	-0.037580189	7.784276167	6.261293539	0.099567114	0.223599898	PREDICTED: protein transport protein Sec23A
RS009976	GO:0044699	single-organism process	BP	-1.439029575	-0.947606699	-0.68706721	1.990896776	6.256892631	0.09975923	0.223754275	PREDICTED: sugar transporter SWEET1
RS004573	GO:0044699	single-organism process	BP	0.753728353	0.336120354	0.570790604	6.159109254	6.256904363	0.099758717	0.223754275	PREDICTED: tryptophan--tRNA ligase, mitochondrial
RS004625	GO:0044699	single-organism process	BP	-0.573753282	-1.547083191	-1.204971987	0.53975011	6.250118	0.100055662	0.224297083	PREDICTED: tigger transposable element-derived protein 6-like
RS001042	GO:0044699	single-organism process	BP	0.377604165	0.526506941	0.382246054	6.008306124	6.243226347	0.100358082	0.224854953	PREDICTED: leishmanolysin-like peptidase
RS002397	GO:0044699	single-organism process	BP	-0.785633516	-0.551479914	-0.462523853	4.24650265	6.237338434	0.10061715	0.225275091	PREDICTED: phosphoserine phosphatase
RS009966	GO:0044699	single-organism process	BP	-0.243614202	-0.508062646	-0.78761656	3.789270855	6.228722713	0.100997397	0.225925617	PREDICTED: transient receptor potential channel pyrexia-like
RS011890	GO:0044699	single-organism process	BP	-1.392418589	-1.074426686	-1.054267848	0.7052205	6.227658726	0.101044451	0.225950607	G-protein coupled receptor Mth2
RS012556	GO:0044699	single-organism process	BP	-0.867790348	-0.745098015	-0.538886637	3.387614638	6.221177603	0.101331526	0.226433169	PREDICTED: rho GTPase-activating protein 12-like
RS007508	GO:0044699	single-organism process	BP	-0.206277528	-0.50989722	0.094040606	5.606337763	6.210422615	0.101809632	0.227222865	PREDICTED: alpha-mannosidase 2
RS009603	GO:0044699	single-organism process	BP	-0.501280182	-1.117738503	-0.812897186	2.721266532	6.196487419	0.10243233	0.228364802	PREDICTED: serine/threonine-protein kinase Chk2
RS003124	GO:0044699	single-organism process	BP	-0.86644645	-0.412003649	-0.470173886	7.170618958	6.190262179	0.102711685	0.228825572	PREDICTED: cytochrome b reductase 1
RS003748	GO:0044699	single-organism process	BP	0.364001788	-0.607553046	-0.366220247	2.710492767	6.180838039	0.103135978	0.229648958	PREDICTED: chromosome transmission fidelity protein 18 homolog
RS007309	GO:0044699	single-organism process	BP	-0.215890885	-0.384966284	0.175524897	6.831394536	6.173301657	0.103476489	0.230244329	PREDICTED: acyl-CoA synthetase family member 3, mitochondrial
RS007566	GO:0044699	single-organism process	BP	-0.165234921	0.059394139	0.503185195	7.013491926	6.159113281	0.104120476	0.231431923	PREDICTED: NADH dehydrogenase [ubiquinone] 1 alpha subcomplex subunit 5
RS001264	GO:0044699	single-organism process	BP	-0.540370506	1.321850389	-0.246643474	0.379907904	6.157830262	0.104178899	0.231480074	PREDICTED: elongation of very long chain fatty acids protein AAEL008004-like
RS009439	GO:0044699	single-organism process	BP	-1.186695259	0.203393487	-1.767899195	0.281563813	6.156088869	0.104258245	0.231556603	E3 ubiquitin-protein ligase sina
RS006890	GO:0044699	single-organism process	BP	0.282954507	-1.7084021	-0.060666224	0.535603492	6.133135493	0.105309529	0.233251531	PREDICTED: malate synthase, glyoxysomal-like
RS006085	GO:0044699	single-organism process	BP	0.313144343	-1.142187451	-1.259770219	0.317838505	6.127419627	0.105572894	0.233752643	PREDICTED: broad-complex core protein
RS015558	GO:0044699	single-organism process	BP	0.735235021	0.211778509	-0.087325057	4.719840591	6.125360241	0.105667938	0.233880845	PREDICTED: meckelin
RS002444	GO:0044699	single-organism process	BP	-0.011627925	0.672200489	0.08763332	3.635921824	6.114559304	0.106167758	0.234724996	PREDICTED: transmembrane protein 110-like
RS004313	GO:0044699	single-organism process	BP	0.621468124	0.534257852	0.733146046	4.224360103	6.113898076	0.10619843	0.234724996	PREDICTED: succinate dehydrogenase assembly factor 3, mitochondrial
RS000810	GO:0044699	single-organism process	BP	-1.210705026	-0.849166162	-0.220752937	1.746960004	6.113953549	0.106195856	0.234724996	PREDICTED: tyramine/octopamine receptor
RS003123	GO:0044699	single-organism process	BP	-0.197461368	-0.487406663	-0.423747548	6.686252954	6.114150154	0.106186736	0.234724996	PREDICTED: phosphatidylinositol 4-kinase alpha
RS013777	GO:0044699	single-organism process	BP	0.279495049	0.245639381	0.652098837	5.139040067	6.110578071	0.106352562	0.234983187	PREDICTED: copper homeostasis protein cutC homolog
RS013631	GO:0044699	single-organism process	BP	-0.316146783	0.108706914	0.300525721	5.814889926	6.105248441	0.10660044	0.235406968	PREDICTED: protein enhancer of sevenless 2B
RS002569	GO:0044699	single-organism process	BP	0.036202504	-0.472182537	-0.345853379	4.593360763	6.097340118	0.10696927	0.235901077	PREDICTED: sodium/bile acid cotransporter 7
RS013555	GO:0044699	single-organism process	BP	-0.99697134	-0.719203134	-0.951350799	3.196207758	6.091871837	0.107225015	0.236413136	PREDICTED: insulin-like growth factor-binding protein complex acid labile subunit
RS007563	GO:0044699	single-organism process	BP	-1.202835431	-1.462716201	-1.791419977	1.4245361	6.089904579	0.107317164	0.236574893	PREDICTED: uncharacterized MFS-type transporter C09D4.1
RS014604	GO:0044699	single-organism process	BP	-0.747668141	-0.333345803	-0.791183082	4.087231012	6.087300146	0.107439276	0.23667837	PREDICTED: probable G-protein coupled receptor 158
RS005591	GO:0044699	single-organism process	BP	-0.522540063	-0.858322328	-0.735800656	2.37867999	6.084869669	0.107553352	0.236846812	PREDICTED: phosphatidylserine synthase 2
RS008343	GO:0044699	single-organism process	BP	0.425195192	0.621020602	0.508117655	4.703481584	6.084095192	0.107589727	0.236885493	PREDICTED: GDP-mannose 4,6 dehydratase
RS005351	GO:0044699	single-organism process	BP	-0.397867942	-0.039889938	0.245645216	7.41412513	6.082183312	0.107679573	0.237000444	PREDICTED: glucose transporter type 1
RS013850	GO:0044699	single-organism process	BP	0.226155429	-0.16034169	-0.376830354	4.968715242	6.079013068	0.107828712	0.237100249	PREDICTED: cubilin-like

RS000218	GO:0044699	single-organism process	BP	0.043076242	-0.548093507	-1.430844745	1.31715817	6.078418505	0.107856704	0.237100249	PREDICTED: innexin inx7
RS006109	GO:0044699	single-organism process	BP	-0.795549779	-1.137521865	-0.533770767	1.634658204	6.050342102	0.109186495	0.239521812	PREDICTED: centromere protein S
RS009881	GO:0044699	single-organism process	BP	-0.797297236	-0.318713435	0.370791229	1.650545529	6.023916498	0.110452416	0.241877538	PREDICTED: sodium/potassium/calcium exchanger Nckx30C
RS006166	GO:0044699	single-organism process	BP	0.025488575	0.569370333	-0.260601387	4.376208038	6.019501158	0.110665296	0.242178767	PREDICTED: peroxidasin homolog
RS002399	GO:0044699	single-organism process	BP	-1.131266147	-0.620330872	-1.563865647	1.022179287	6.015745455	0.110846682	0.242403736	beat-VI
RS005652	GO:0044699	single-organism process	BP	-0.969696053	-0.993586662	-0.315128041	1.541678254	6.012945224	0.110982108	0.242602571	PREDICTED: ras-related protein Rab-7L1
RS009550	GO:0044699	single-organism process	BP	-0.635389354	-0.332728298	-0.404356373	4.663601717	6.01181261	0.111036928	0.242609138	PREDICTED: polycomb complex protein BMI-1
RS008841	GO:0044699	single-organism process	BP	0.467422341	0.594810988	-0.250124864	3.867866615	6.010773559	0.111087243	0.242645952	PREDICTED: platelet-activating factor acetylhydrolase
RS013092	GO:0044699	single-organism process	BP	-1.21991486	-0.311211194	-1.526773013	0.464605072	5.999428186	0.111638048	0.243540502	PREDICTED: polypeptide N-acetylgalactosaminyltransferase 2
RS008094	GO:0044699	single-organism process	BP	-0.917228522	-0.679216209	-0.41399674	3.095856104	5.996385207	0.111786225	0.24369668	PREDICTED: electron transfer flavoprotein-ubiquinone oxidoreductase, mitochondrial
RS015569	GO:0044699	single-organism process	BP	-0.128331611	-0.760589397	-0.492329527	3.790653976	5.993743108	0.111915034	0.24393526	PREDICTED: cyclin-H
RS013082	GO:0044699	single-organism process	BP	-0.038269835	-0.543335982	-0.164986164	7.037010843	5.993036247	0.111949519	0.243939451	PREDICTED: carbonyl reductase [NADPH] 1-like
RS003875	GO:0044699	single-organism process	BP	-0.961695284	-0.241376636	-0.535058303	4.159161175	5.990427609	0.112076873	0.244119013	PREDICTED: growth factor receptor-bound protein 14-like
RS013566	GO:0044699	single-organism process	BP	-0.157588116	0.520916419	-1.587874113	0.395549287	5.986097896	0.112288555	0.244537794	beat protein
RS006406	GO:0044699	single-organism process	BP	0.612520104	-0.00332516	-0.151026738	3.645552596	5.982561902	0.112461716	0.244656992	AGAP004293-PA-like protein
RS001184	GO:0044699	single-organism process	BP	-4.147618499	0.081714403	-0.543245406	0.713984789	5.981807323	0.112498701	0.244656992	PREDICTED: putative fatty acyl-CoA reductase CG5065
RS009677	GO:0044699	single-organism process	BP	-0.408447797	-0.081762186	0.324630792	8.122332392	5.983940879	0.112394156	0.244656992	PREDICTED: NADH dehydrogenase [ubiquinone] flavoprotein 2, mitochondrial
RS004745	GO:0044699	single-organism process	BP	0.161397126	-0.223153389	-0.31612292	5.470584727	5.981295365	0.112523801	0.244669328	PREDICTED: intraflagellar transport protein 46 homolog
RS007456	GO:0044699	single-organism process	BP	-0.576442849	-0.23827504	-0.616910623	8.133018648	5.977700241	0.112700212	0.244948417	PREDICTED: ankyrin-2-like
RS007735	GO:0044699	single-organism process	BP	-0.503769274	-1.027659606	0.506197934	1.152999129	5.977362774	0.112716784	0.244948417	PREDICTED: dynein heavy chain 10, axonemal
RS008677	GO:0044699	single-organism process	BP	-0.134647501	0.41411484	0.101144302	5.164586238	5.97517261	0.112824399	0.245111348	PREDICTED: twinfilin
RS008507	GO:0044699	single-organism process	BP	-0.534017422	0.001864636	0.460916761	2.352125317	5.968964121	0.11312999	0.24564812	PREDICTED: dorsal-ventral patterning protein Sog
RS010301	GO:0044699	single-organism process	BP	0.554864705	0.504110275	0.293166126	4.535879289	5.967134906	0.113220177	0.245801571	PREDICTED: thioredoxin domain-containing protein 12
RS009685	GO:0044699	single-organism process	BP	-0.727384329	-0.47287616	-0.746026536	3.938086398	5.964332856	0.113358462	0.246056979	PREDICTED: gamma-glutamyl hydrolase-like
RS005653	GO:0044699	single-organism process	BP	0.053349091	0.30239504	-1.0528009	2.80168646	5.961151969	0.113515639	0.246273206	PREDICTED: cyclin-dependent kinase inhibitor 1C
RS010173	GO:0044699	single-organism process	BP	-0.653474603	-0.670329541	-0.018744995	2.781255318	5.960250908	0.113560201	0.24628503	PREDICTED: histone-lysine N-methyltransferase 2C-like
RS011794	GO:0044699	single-organism process	BP	0.54458062	0.299455757	0.208575016	6.472060352	5.950784891	0.114029352	0.24704725	PREDICTED: DNA-directed RNA polymerase III subunit RPC2
RS007852	GO:0044699	single-organism process	BP	-0.4463895	-0.500436907	-0.353366809	5.359879642	5.946281355	0.114253205	0.24733912	PREDICTED: multidrug resistance-associated protein 7
RS012979	GO:0044699	single-organism process	BP	0.618469103	0.473188915	0.244616989	4.685711162	5.934546112	0.114838488	0.248415651	PREDICTED: U6 snRNA-associated Sm-like protein LSm2
RS009673	GO:0044699	single-organism process	BP	-0.65890353	-1.419644883	-1.07791182	0.918069721	5.929609579	0.115085548	0.248864592	PREDICTED: dynein heavy chain 9, axonemal
RS010656	GO:0044699	single-organism process	BP	-1.049567645	-1.38587029	-0.733928491	1.012615895	5.923136956	0.115410253	0.249310487	PREDICTED: ras-related protein Rab-8A
RS002945	GO:0044699	single-organism process	BP	0.649828836	-0.442519103	-0.201002517	1.825683656	5.920320177	0.115551832	0.249491258	PREDICTED: DNA helicase MCM9
RS004766	GO:0044699	single-organism process	BP	0.503166061	-0.227587613	-0.178208236	4.383163414	5.919991168	0.11556838	0.249491258	PREDICTED: thioredoxin-related transmembrane protein 1-like
RS002734	GO:0044699	single-organism process	BP	0.299931428	-0.05370325	-0.483067767	7.336391207	5.907492348	0.116198696	0.250669128	PREDICTED: dolichyl-diphosphooligosaccharide--protein glycosyltransferase subunit 1
RS006445	GO:0044699	single-organism process	BP	-0.639463442	-0.446387769	0.056703064	3.503852672	5.903016491	0.11642521	0.251028779	PREDICTED: serine palmitoyltransferase 2
RS000704	GO:0044699	single-organism process	BP	-0.474456632	-0.423285145	-0.406316616	6.32955037	5.894533373	0.116855681	0.251739139	PREDICTED: voltage-dependent calcium channel subunit alpha-2/delta-3
RS014582	GO:0044699	single-organism process	BP	-0.433611466	0.299788286	-0.18823496	3.593128362	5.892219468	0.116973362	0.251822654	PREDICTED: nicotinamide/nicotinic acid mononucleotide adenylyltransferase 1
RS001519	GO:0044699	single-organism process	BP	-0.472653361	0.203286284	0.353773033	3.177377624	5.881974043	0.117495788	0.252635562	PREDICTED: beta carbonic anhydrase 1
RS005363	GO:0044699	single-organism process	BP	0.06250066	-0.509821841	-0.317977893	4.739870694	5.881667678	0.117511444	0.252635562	PREDICTED: DNA ligase 3
RS005923	GO:0044699	single-organism process	BP	-0.29583379	-0.408740057	-0.606163424	4.718333096	5.881905023	0.117499315	0.252635562	PREDICTED: ALK tyrosine kinase receptor
RS008776	GO:0044699	single-organism process	BP	0.176397475	0.407614872	0.550184837	6.705291978	5.872153438	0.11799864	0.253423409	PREDICTED: actin-related protein 2/3 complex subunit 3
RS014344	GO:0044699	single-organism process	BP	0.401508425	0.14567646	-0.540803526	3.263857413	5.864287836	0.118402869	0.254118223	PREDICTED: tektin-1
RS008420	GO:0044699	single-organism process	BP	0.059381848	0.13717831	0.48004901	6.228946149	5.863111354	0.118463444	0.254161604	PREDICTED: vesicle-associated membrane protein 2
RS008209	GO:0044699	single-organism process	BP	-0.797469436	0.00047394	-0.19086895	5.236915618	5.860312794	0.118607657	0.254297723	PREDICTED: protein spinster
RS009820	GO:0044699	single-organism process	BP	0.026507268	-0.679608892	-0.508640644	3.076979217	5.859298786	0.118659951	0.254366539	PREDICTED: eyes absent homolog 1
RS015217	GO:0044699	single-organism process	BP	-1.918332283	0.656528436	-0.710886007	0.308559129	5.858582753	0.118696891	0.254402424	PREDICTED: probable G-protein coupled receptor B0563.6
RS008518	GO:0044699	single-organism process	BP	0.072303746	-0.419490042	-0.319932956	6.262352468	5.856393956	0.118809879	0.254601263	PREDICTED: replication protein A 32 kDa subunit
RS001001	GO:0044699	single-organism process	BP	-0.795446363	0.222492307	-0.593398043	1.447294358	5.843906605	0.119456457	0.255595415	PREDICTED: lachesin-like
RS006877	GO:0044699	single-organism process	BP	-1.565839401	-1.137352864	-1.254175337	0.6979186	5.839978249	0.119660554	0.255813437	PREDICTED: acyl-CoA Delta(11) desaturase
RS003623	GO:0044699	single-organism process	BP	0.492750706	0.473603418	0.434689901	5.095752538	5.83959995	0.119680226	0.255813437	PREDICTED: male-enhanced antigen 1
RS000168	GO:0044699	single-organism process	BP	-0.555511687	-0.887978015	-1.234124807	1.652741901	5.837936606	0.119766759	0.255954957	PREDICTED: potassium voltage-gated channel protein eag
RS001871	GO:0044699	single-organism process	BP	0.079598677	-0.410279911	0.141060918	5.713758628	5.833134417	0.12001692	0.256359072	PREDICTED: serine/threonine-protein kinase grp
RS003890	GO:0044699	single-organism process	BP	-1.399572918	-0.662104974	-1.231074075	0.647625922	5.829029429	0.120231156	0.256599081	PREDICTED: probable G-protein coupled receptor No18
RS011228	GO:0044699	single-organism process	BP	0.334084549	-0.045062798	-0.253509042	5.326355936	5.828624641	0.120252301	0.256600726	PREDICTED: actin-related protein 2
RS009585	GO:0044699	single-organism process	BP	-0.155397372	0.077187945	0.529927416	8.081329361	5.820729463	0.12066544	0.257395079	PREDICTED: NADH dehydrogenase [ubiquinone] 1 beta subcomplex subunit 5, mitochondrial
RS014729	GO:0044699	single-organism process	BP	-1.272976392	-1.097970962	-0.527420749	2.348202298	5.808104611	0.121328885	0.258635068	PREDICTED: phosphoglycerate mutase 1
RS011886	GO:0044699	single-organism process	BP	0.204713045	0.342290862	0.652481344	6.702554752	5.80647803	0.121414616	0.258730232	PREDICTED: probable complex I intermediate-associated protein 30, mitochondrial
RS011597	GO:0044699	single-organism process	BP	0.120865252	0.3884333	0.497900193	7.018899084	5.793582234	0.122096347	0.260094959	PREDICTED: charged multivesicular body protein 1b
RS011833	GO:0044699	single-organism process	BP	-0.577940263	-0.306121354	-0.163344325	4.887916805	5.788906916	0.122344406	0.260451612	PREDICTED: brain-specific angiogenesis inhibitor 3
RS013104	GO:0044699	single-organism process	BP	-0.255874279	-0.939218493	-1.405518767	1.263593869	5.774891314	0.123090913	0.261770848	hypothetical protein
RS006582	GO:0044699	single-organism process	BP	-0.576013873	-1.031647124	-0.839421999	2.650540137	5.771045921	0.123296486	0.26216376	PREDICTED: probable G-protein coupled receptor No9
RS004055	GO:0044699	single-organism process	BP	-0.954511167	-0.790556597	-0.764680904	3.527651994	5.768285115	0.12344428	0.262345133	PREDICTED: ras-related protein Rab-3
RS006400	GO:0044699	single-organism process	BP	0.568217135	-0.041409934	-0.370464485	3.331561104	5.766495959	0.123540148	0.262504576	PREDICTED: leucine-rich repeat-containing protein 47-like
RS011030	GO:0044699	single-organism process	BP	-0.251290958	-0.699850301	-0.014462638	3.890220076	5.76416074	0.123665383	0.262682042	PREDICTED: SRSF protein kinase 3
RS014830	GO:0044699	single-organism process	BP	-0.613523633	-0.385881539	-0.491426715	5.84635008	5.763433182	0.123704425	0.262720663	PREDICTED: patched domain-containing protein 3-like
RS000398	GO:0044699	single-organism process	BP	0.277198819	0.379861004	-0.066027388	5.894739848	5.761774521	0.123793477	0.26277685	PREDICTED: RNA polymerase I-specific transcription initiation factor RRN3
RS013046	GO:0044699	single-organism process	BP	-0.192559498	-0.410187946	-0.766212712	4.050990539	5.757141628	0.124042537	0.26312813	PREDICTED: egl nine homolog 1
RS011753	GO:0044699	single-organism process	BP	0.306224541	0.343514137	0.5706241	7.209833327	5.746385689	0.124622606	0.26404729	PREDICTED: glutaredoxin-C4-like
RS009661	GO:0044699	single-organism process	BP	0.59943041	0.211937076	-0.085571172	7.380254275	5.737164679	0.125121951	0.264971558	PREDICTED: dolichyl-diphosphooligosaccharide--protein glycosyltransferase subunit 2
RS006127	GO:0044699	single-organism process	BP	-0.259350556	0.027967109	0.415460673	7.54256678	5.736162685	0.125176327	0.265042142	PREDICTED: NADH dehydrogenase [ubiquinone] 1 beta subcomplex subunit 10
RS013091	GO:0044699	single-organism process	BP	-0.634795047	0.023977642	-0.224683025	3.598027978	5.731282535	0.125441482	0.265335912	PREDICTED: polypeptide N-acetylgalactosaminyltransferase 14
RS006504	GO:0044699	single-organism process	BP	0.680835382	0.337313251	0.107389767	3.996147763	5.728961633	0.125567772	0.265424726	PREDICTED: 5-formyltetrahydrofolate cyclo-ligase
RS010624	GO:0044699	single-organism process	BP	-1.888144745	-0.350620517	-1.036675402	0.280983459	5.728136435	0.125612703	0.265452684	PREDICTED: exostosin-1
RS011781	GO:0044699	single-organism process	BP	-0.445660127	-0.864112419	-0.573881668	9.678543904	5.72543243	0.125760042	0.265569301	PREDICTED: enolase
RS001145	GO:0044699	single-organism process	BP	-0.53262649	-0.12107554	0.117285332	6.762749099	5.723923744	0.125842321	0.265692906	PREDICTED: solute carrier family 2, facilitated glucose transporter member 1-like
RS011958	GO:0044699	single-organism process	BP	-1.045130484	-0.946209805	-2.006970418	0.460382455	5.711212314	0.126537595	0.267026554	PREDICTED: MOXD1 homolog 2-like
RS009467	GO:0044699	single-organism process	BP	0.105282131	0.575829771	0.630071704	6.50153031	5.708395503	0.12669216	0.267269998	PREDICTED: gamma-glutamyltransferase 7
RS005236	GO:0044699	single-organism process	BP	-0.445853089	0.203787183	0.177320673	4.467206789	5.708336462	0.126695402	0.267269998	PREDICTED: calcium/calmodulin-dependent protein kinase type II alpha chain
RS012209	GO:0044699	single-organism process	BP	-0.458083923	-0.170342222	0.3412592	3.687183544	5.702533014	0.127014424	0.267808414	PREDICTED: SHC-transforming protein 1
RS000273	GO:0044699	single-organism process	BP	-1.300251871	-0.732876516	-0.366313312	1.089297814	5.699094456	0.127203806	0.268028231	PREDICTED: DNA excision repair protein ERCC-6
RS006951	GO:0044699	single-organism process	BP	0.362871559	0.199149505	-0.106154987	6.457125486	5.692470091	0.127569407	0.268446696	PREDICTED: lysine-specific histone demethylase 1A
RS002077	GO:0044699	single-organism process	BP	-0.0224398	-0.068473309	0.408097099	5.040364207	5.684367699	0.128017941	0.269068416	PREDICTED: endonuclease/exonuclease/phosphatase family domain-containing protein 1-like
RS005274	GO:0044699	single-organism process	BP	0.57201853	0.219701086	0.430708717	5.513139031	5.683865728	0.128045778	0.269082018	PREDICTED: glutamate-rich WD repeat-containing protein 1
RS009626	GO:0044699	single-organism process	BP	-1.579002054	-1.487462508	-1.045248076	4.049931115	5.682494232	0.128121866	0.269173334	eater
RS009998	GO:0044699	single-organism process	BP	-0.052218738	-1.067407497	-0.062787963	3.059610981	5.676902406	0.128432533	0.269372103	PREDICTED: tryptophan 2,3-dioxygenase
RS012118	GO:0044699	single-organism process	BP	-0.636280503	-0.481948741	-0.088616276	4.20929643	5.676745583	0.128441256	0.269372103	PREDICTED: heparan sulfate glucosamine 3-O-sulfotransferase 6
RS000174	GO:0044699	single-organism process	BP	-0.933724731	-0.521167867	-0.720878603	3.374530574	5.675332301	0.128519893	0.269372103	PREDICTED: band 7 protein AGAP004871-like
RS006444	GO:0044699	single-organism process	BP	0.247684833	-1.158327215	-1.023157283	0.52416464	5.678360053	0.128351481	0.269372103	PREDICTED: alpha,alpha-trehalose-phosphate synthase [UDP-forming]
RS000747	GO:0044699	single-organism process	BP	0.450510585	0.423416615	0.213910119	5.988812706	5.676438738	0.128458326	0.269372103	PREDICTED: sec1 family domain-containing protein 2-like
RS009629	GO:0044699	single-organism process	BP	-0.691618563	-0.413021374	0.205371865	9.600427322	5.679143987	0.128307911	0.269372103	PREDICTED: isocitrate dehydrogenase [NAD] subunit gamma, mitochondrial
RS010555	GO:0044699	single-organism process	BP	0.929255584	0.186969273	-0.279650258	2.292744501	5.673113421	0.128643447	0.269528459	PREDICTED: tyrosine-protein kinase Dnt-like
RS002083	GO:0044699	single-organism process	BP	-0.323137174	-0.49516432	0.148595856	8.605719851	5.666822715	0.128994345	0.270039027	PREDICTED: aspartate aminotransferase, mitochondrial
RS014207	GO:0044699	single-organism process	BP	-0.610018037	-0.793941432	-0.296572058	2.637696837	5.663067597	0.129204241	0.270343615	PREDICTED: ADP-ribosylation factor-like protein 13B
RS000854	GO:0044699	single-organism process	BP	0.086388548	-0.448920684	-0.876676062	3.261541941	5.654967215	0.129658127	0.271203197	PREDICTED: chromosome-associated kinesin KIF4
RS005398	GO:0044699	single-organism process	BP	0.260717452	0.422740272	-0.108311338	4.832779292	5.648569798	0.130017662	0.271819793	PREDICTED: cyclin-dependent kinase 9
RS006326	GO:0044699	single-organism process	BP	-0.089521494	0.415516352	0.565958597	5.148928649	5.645303686	0.130201583	0.271920355	PREDICTED: ankyrin repeat and death domain-containing protein 1A-like
RS003622	GO:0044699	single-organism process	BP	0.437288663	-0.123031371	-0.22124586	6.02604864	5.645973331	0.130163854	0.271920355	PREDICTED: dynein heavy chain 2, axonemal
RS003823	GO:0044699	single-organism process	BP	-0.312627517	-0.255132414	-0.725939607	3.723623892	5.646108513	0.130156238	0.271920355	PREDICTED: G-protein coupled receptor GRL101-like
RS003358	GO:0044699	single-organism process	BP	-0.353225898	-0.121840444	0.371616686	8.291426996	5.633056759	0.130893435	0.272925814	PREDICTED: ATP synthase-coupling factor 6, mitochondrial
RS014390	GO:0044699	single-organism process	BP	0.510425399	0.309243454	-0.10472795	6.434540969	5.6307243	0.131025595	0.273011931	PREDICTED: DNA methyltransferase 1-associated protein 1
RS007510	GO:0044699	single-organism process	BP	-0.474258119	-0.815348826	-0.098467285	4.567105163	5.618317073	0.131730741	0.274127057	PREDICTED: sodium-coupled monocarboxylate transporter 1-like
RS012338	GO:0044699	single-organism process	BP	-0.563620536	-0.376492761	0.024551568	7.936118918	5.617244954	0.131791842	0.274208898	PREDICTED: ATP-dependent 6-phosphofructokinase
RS008279	GO:0044699	single-organism process	BP	-0.484679453	-0.433954641	0.086422434	3.880506245	5.606020043	0.132433181	0.27531586	PREDICTED: potassium voltage-gated channel protein Shaw-like
RS009448	GO:0044699	single-organism process	BP	-1.39837706	-1.14434035	-1.417540583	0.89502985	5.600775455	0.132733847	0.275667888	E3 ubiquitin-protein ligase sina
RS012618	GO:0044699	single-organism process	BP	0.640367169	-0.198864572	-0.120314847	2.600306879	5.600260708	0.132763392	0.275683785	PREDICTED: serine/threonine-protein kinase NLK
RS003182	GO:0044699	single-organism process	BP	-0.258483588	0.744929838	-0.534274495	2.131381648	5.599624998	0.132799888	0.27571411	PREDICTED: cyclin-dependent kinase 4
RS003225	GO:0044699	single-organism process	BP	-0.433425834	-0.58821296	0.083272594	4.76902773	5.5944249	0.133098785	0.276198073	PREDICTED: syndecan
RS006497	GO:0044699	single-organism process	BP	-0.499541328	-1.800371012	-1.244184902	0.270751657	5.580972087	0.133875009	0.277534463	PREDICTED: facilitated trehalose transporter Tret1-2 homolog
RS003719	GO:0044699	single-organism process	BP	0.60785672	0.311286476	0.122882378	5.366656437	5.579691181	0.133949141	0.277642442	PREDICTED: DNA-directed RNA polymerase III subunit RPC3
RS015424	GO:0044699	single-organism process	BP	0.774413179	0.35481848	-0.480300837	2.127448003	5.577639521	0.13406796	0.277797284	PREDICTED: protein Wnt-10a
RS009361	GO:0044699	single-organism process	BP	-0.469548252	-1.030089652	-0.272125463	2.379260128	5.571170404	0.134443268	0.278300217	PREDICTED: sodium- and chloride-dependent glycine transporter 2-like

RS003947	GO:0044699	single-organism process	BP	0.438161137	0.271388201	0.493351708	5.499648986	5.567309687	0.134667724	0.278719033	PREDICTED: cleavage stimulation factor subunit 1
RS011541	GO:0044699	single-organism process	BP	0.328380656	-0.23808386	-0.089473223	5.292394638	5.561473505	0.135007706	0.279330877	PREDICTED: ran GTPase-activating protein 1
RS009697	GO:0044699	single-organism process	BP	0.569668677	0.126888506	0.047327614	6.260897588	5.554895439	0.135391882	0.279849887	PREDICTED: vezatin
RS005269	GO:0044699	single-organism process	BP	0.033876174	-0.502906721	-0.030003777	5.135665287	5.554442412	0.135418378	0.279858723	PREDICTED: protein SGT1 homolog ecdysoneless
RS007949	GO:0044699	single-organism process	BP	-0.12886544	-0.339712403	-0.474911852	5.46833021	5.550428572	0.13565335	0.280206379	PREDICTED: ribonuclease 3
RS000237	GO:0044699	single-organism process	BP	-0.122122545	0.34733073	-0.422707365	3.88004886	5.545752767	0.135927562	0.28068072	PREDICTED: gamma-glutamyltranspeptidase 1-like
RS012883	GO:0044699	single-organism process	BP	0.593893674	0.092849465	0.398639901	4.518991514	5.535656668	0.13652144	0.281722271	PREDICTED: probable DNA-directed RNA polymerase III subunit RPC6
RS002972	GO:0044699	single-organism process	BP	-0.311172151	-0.506842424	-0.237806006	6.495567074	5.534163734	0.136609467	0.281857737	PREDICTED: sodium leak channel non-selective protein
RS002378	GO:0044699	single-organism process	BP	0.189638401	-0.292415431	-0.3313077	5.146004911	5.532246854	0.13672257	0.281998697	PREDICTED: exportin-6
RS012532	GO:0044699	single-organism process	BP	0.52422059	0.27169055	0.170626759	5.950381512	5.527522768	0.137001687	0.282351902	PREDICTED: adenosine 3'-phospho 5'-phosphosulfate transporter 2
RS005188	GO:0044699	single-organism process	BP	0.543893153	0.111055458	-0.040744688	6.235121469	5.527796455	0.136985502	0.282351902	PREDICTED: nucleolar complex protein 2 homolog
RS011880	GO:0044699	single-organism process	BP	-0.086524241	0.085080431	0.499597912	7.945836466	5.525046466	0.137148213	0.282552689	PREDICTED: adenylosuccinate synthetase
RS007587	GO:0044699	single-organism process	BP	0.548015678	0.376775275	0.454348959	4.86046888	5.504926438	0.138344259	0.284411948	PREDICTED: magnesium transporter NIPA2
RS004797	GO:0044699	single-organism process	BP	0.459004562	-0.000986695	-0.496189284	4.718991441	5.504275332	0.138383129	0.284445426	PREDICTED: dystroglycan-like
RS008412	GO:0044699	single-organism process	BP	-0.558904773	-1.47615803	-1.995578468	0.264227687	5.502174534	0.138508614	0.284477308	PREDICTED: dynein assembly factor 1, axonemal
RS006147	GO:0044699	single-organism process	BP	-0.356527382	0.242750606	-0.324230967	4.139518639	5.494451268	0.13897087	0.285106923	Uncharacterized protein CG3556
RS008847	GO:0044699	single-organism process	BP	0.340494887	0.472162049	0.51678103	6.538622182	5.489856779	0.139246555	0.285567678	PREDICTED: inositol 2-dehydrogenase-like
RS012098	GO:0044699	single-organism process	BP	-0.722635213	-0.863193595	-0.599560773	3.5591329	5.486483871	0.13944927	0.285797251	PREDICTED: protein tweety
RS014059	GO:0044699	single-organism process	BP	-0.981423525	-2.483264786	-1.45423058	0.238549299	5.473171168	0.140252114	0.287068929	PREDICTED: allatostatin-A receptor-like
RS009262	GO:0044699	single-organism process	BP	-0.25857572	0.038639212	0.546361074	8.794488927	5.469506202	0.140473903	0.28738277	PREDICTED: cytochrome c oxidase subunit 7C, mitochondrial-like
RS011105	GO:0044699	single-organism process	BP	0.120646907	-0.265641169	-0.297512323	6.388868085	5.466399933	0.140662142	0.287627703	PREDICTED: putative pre-mRNA-splicing factor ATP-dependent RNA helicase DHX16
RS014794	GO:0044699	single-organism process	BP	0.684288907	0.57343907	0.452752305	7.409120301	5.463876404	0.140815244	0.287847294	PREDICTED: serine/threonine-protein phosphatase 2A regulatory subunit B'' subunit gamma-like
RS012521	GO:0044699	single-organism process	BP	-0.27911124	0.207963234	0.176143396	5.682101261	5.464162095	0.140797903	0.287847294	PREDICTED: sodium/hydrogen exchanger 8
RS003699	GO:0044699	single-organism process	BP	-0.478691962	-0.337253077	0.505415439	2.071752133	5.462191092	0.140917579	0.288009735	PREDICTED: TGF-beta receptor type-1
RS004077	GO:0044699	single-organism process	BP	-1.170967132	-1.184318721	-0.844919743	10.17432667	5.460351222	0.14102938	0.288191466	PREDICTED: transferrin
RS006161	GO:0044699	single-organism process	BP	0.38084584	0.495760539	0.355981582	6.479692283	5.457274253	0.141216541	0.288504088	PREDICTED: endothelial differentiation-related factor 1 homolog
RS011045	GO:0044699	single-organism process	BP	0.187705096	-0.108025719	-0.308975176	6.706258871	5.45380424	0.141427892	0.288865208	PREDICTED: cAMP-dependent protein kinase type I regulatory subunit
RS001940	GO:0044699	single-organism process	BP	0.014624858	0.538479708	0.31160031	4.747114522	5.447607577	0.141806064	0.289590654	PREDICTED: ras-related protein Rab6
RS008470	GO:0044699	single-organism process	BP	-0.519995165	-0.339577271	-0.281098773	6.678033077	5.445239444	0.141950839	0.289803056	PREDICTED: E3 ubiquitin-protein ligase Su(dx)
RS014889	GO:0044699	single-organism process	BP	-0.711641029	-0.622090454	-0.23012999	2.99436616	5.444661033	0.141986222	0.289817581	PREDICTED: UDP-sugar transporter UST74c-like
RS015384	GO:0044699	single-organism process	BP	-0.654037834	-0.476456827	0.275335892	10.72611864	5.440034908	0.142269511	0.290163428	PREDICTED: probable aconitate hydratase, mitochondrial
RS007980	GO:0044699	single-organism process	BP	0.197066924	0.488438041	0.322953674	6.235166424	5.432059662	0.142759147	0.290923737	PREDICTED: centrin-2
RS012857	GO:0044699	single-organism process	BP	-0.105625886	0.201123089	0.498522584	7.453971122	5.428494018	0.142978573	0.291276634	PREDICTED: NADH dehydrogenase [ubiquinone] 1 alpha subcomplex subunit 13
RS011806	GO:0044699	single-organism process	BP	-0.63141387	-0.906953768	-0.479906313	2.978714318	5.426575255	0.143096785	0.291470307	PREDICTED: POU domain protein CF1A
RS005181	GO:0044699	single-organism process	BP	-0.168717926	0.322198012	0.688998249	2.903388389	5.420247571	0.143487277	0.292076736	PREDICTED: beta-1,3-galactosyltransferase 1-like
RS004257	GO:0044699	single-organism process	BP	0.051261261	0.565692392	0.504493374	8.106681345	5.410703222	0.144078184	0.293090075	PREDICTED: tropomodulin
RS001154	GO:0044699	single-organism process	BP	0.311304583	-0.272735866	-0.327283925	4.041313895	5.409347304	0.144162318	0.293100272	PREDICTED: tRNA wybutosine-synthesizing protein 5
RS006553	GO:0044699	single-organism process	BP	0.415089736	0.499812934	0.239034811	5.166117832	5.404991496	0.144432908	0.293432496	PREDICTED: PHD finger-like domain-containing protein 5A
RS000759	GO:0044699	single-organism process	BP	-0.433403951	-0.385527821	-0.401367398	5.389311713	5.404124782	0.144486807	0.293494653	PREDICTED: gamma-aminobutyric acid type B receptor subunit 1
RS008228	GO:0044699	single-organism process	BP	-0.371378043	-0.79623786	-0.326576187	3.818023202	5.402709771	0.144574845	0.293626124	PREDICTED: serine/threonine-protein kinase ULK2
RS008382	GO:0044699	single-organism process	BP	0.266656973	0.812272633	-0.199148228	3.251303257	5.39864168	0.144828231	0.29385641	PREDICTED: uridine phosphorylase 1
RS001924	GO:0044699	single-organism process	BP	0.249143226	0.393529678	0.558679779	6.115571957	5.387149473	0.14554631	0.295075697	PREDICTED: putative inositol monophosphatase 3
RS008894	GO:0044699	single-organism process	BP	-0.514062757	-0.696879917	-0.505523514	4.11078946	5.375092365	0.146303308	0.296306434	PREDICTED: nuclear hormone receptor HR96
RS010668	GO:0044699	single-organism process	BP	-0.489947037	-0.580808276	-0.366666726	4.924551177	5.371105693	0.146554427	0.296689672	PREDICTED: acyloxyacyl hydrolase
RS005474	GO:0044699	single-organism process	BP	-0.34611367	0.188705029	-0.350387077	6.225257583	5.369759174	0.146639335	0.296766156	PREDICTED: slit homolog 3 protein
RS013503	GO:0044699	single-organism process	BP	-0.119576598	-0.85479082	-0.51412057	2.369599699	5.369864542	0.146632689	0.296766156	PREDICTED: single Ig IL-1-related receptor-like
RS013833	GO:0044699	single-organism process	BP	-1.470670204	-0.063183436	-1.492054285	0.260360657	5.367809086	0.146762387	0.296919758	PREDICTED: gastrin/cholecystokinin type B receptor-like
RS012697	GO:0044699	single-organism process	BP	-0.622221402	-0.796015617	-1.208933851	2.506197036	5.366195078	0.146864305	0.297078229	PREDICTED: G-protein coupled receptor Mth2-like
RS001062	GO:0044699	single-organism process	BP	0.537274595	0.911534042	0.688261052	2.651358759	5.364074352	0.146998322	0.297206111	PREDICTED: UPF0769 protein C21orf59 homolog
RS013345	GO:0044699	single-organism process	BP	-0.323347678	-0.952835707	-0.628418917	2.452265869	5.361101816	0.147186363	0.297490781	PREDICTED: transmembrane channel-like protein 3
RS007245	GO:0044699	single-organism process	BP	-0.109691638	-0.538704272	-0.521010048	4.600455628	5.356909305	0.147451966	0.297788657	PREDICTED: beta-1,3-galactosyltransferase 6
RS004631	GO:0044699	single-organism process	BP	0.5608506	0.387863411	0.518040567	5.664766756	5.357108262	0.147439352	0.297788657	PREDICTED: kaptin-like
RS006573	GO:0044699	single-organism process	BP	0.485262475	0.662382533	0.577118833	8.828001204	5.349733011	0.14790765	0.298469627	PREDICTED: tubulin beta-1 chain
RS010154	GO:0044699	single-organism process	BP	-0.098048802	-0.878061324	-0.368300145	2.474373048	5.341149622	0.14845443	0.29938112	PREDICTED: ADP-ribosylation factor-like protein 6
RS009599	GO:0044699	single-organism process	BP	-0.629337009	-0.652873003	-0.495532334	9.067326827	5.336457354	0.148754145	0.29979352	PREDICTED: fructose-bisphosphate aldolase
RS011885	GO:0044699	single-organism process	BP	0.560958428	0.381144006	0.226682277	7.447775785	5.334338605	0.148889666	0.299945651	PREDICTED: dolichyl-diphosphooligosaccharide--protein glycosyltransferase subunit STT3B
RS011419	GO:0044699	single-organism process	BP	0.142670051	0.566918683	0.76286631	3.172442667	5.332525292	0.149005743	0.30010848	PREDICTED: putative inorganic phosphate cotransporter
RS013055	GO:0044699	single-organism process	BP	0.191296626	-0.56501285	-0.569387129	3.196188907	5.317906407	0.149944682	0.30171007	PREDICTED: serine/threonine-protein kinase VRK1-like
RS014327	GO:0044699	single-organism process	BP	0.383151527	0.403145502	0.506472801	6.956924125	5.312603933	0.150286629	0.302103546	PREDICTED: SUMO-conjugating enzyme UBC9-B
RS000993	GO:0044699	single-organism process	BP	0.577956101	0.48635634	0.37411447	6.953624448	5.311197155	0.150377473	0.302194653	PREDICTED: 28S ribosomal protein S29, mitochondrial
RS004964	GO:0044699	single-organism process	BP	0.172623637	-0.249053349	-0.488374307	6.739440872	5.306159171	0.150703231	0.302615295	PREDICTED: hydroxysteroid dehydrogenase-like protein 2
RS013551	GO:0044699	single-organism process	BP	-0.40069454	-0.157960658	0.518470598	5.501819228	5.304168783	0.150832114	0.302721855	PREDICTED: mannose-P-dolichol utilization defect 1 protein homolog
RS013646	GO:0044699	single-organism process	BP	-0.987549481	-1.420507085	-0.873106927	1.419380886	5.303427679	0.150880129	0.302769972	PREDICTED: E3 ubiquitin-protein ligase SIAH1
RS000402	GO:0044699	single-organism process	BP	-0.26718069	0.008072239	0.51581876	8.673900207	5.301862053	0.150981611	0.302828859	PREDICTED: cytochrome c oxidase subunit 6A, mitochondrial
RS012262	GO:0044699	single-organism process	BP	-0.819169073	-0.272724588	-0.743023701	3.673134324	5.29753969	0.151262117	0.303246593	PREDICTED: fasciclin-2
RS006238	GO:0044699	single-organism process	BP	0.238584558	-0.04397443	-0.231491456	5.794637148	5.284717858	0.152097106	0.304823512	PREDICTED: mitochondrial Rho GTPase
RS002158	GO:0044699	single-organism process	BP	-0.886158538	-1.427951308	-0.912508156	5.195968979	5.283943126	0.152147697	0.304876388	PREDICTED: hypertrehalosaemic prohormone-like
RS001338	GO:0044699	single-organism process	BP	0.292519	-0.064509471	0.329201742	5.760278361	5.281390401	0.152314508	0.305065031	PREDICTED: pre-mRNA 3' end processing protein WDR33
RS008683	GO:0044699	single-organism process	BP	-0.685747102	-0.486113188	-0.249052476	4.253825687	5.276867736	0.152610471	0.305512044	PREDICTED: aminoacylase-1-like
RS005928	GO:0044699	single-organism process	BP	-0.102628163	-0.343614326	-0.463905142	5.406755596	5.273770603	0.15281346	0.305821185	PREDICTED: DNA polymerase eta
RS011575	GO:0044699	single-organism process	BP	-0.541502238	-0.734592233	0.432127154	1.021805414	5.272019849	0.152928319	0.305905218	PREDICTED: neuromedin-K receptor
RS009042	GO:0044699	single-organism process	BP	0.233063403	0.223182355	0.508790511	5.488764188	5.271630729	0.152953859	0.305907718	PREDICTED: ubiquitin-conjugating enzyme E2 Z
RS002645	GO:0044699	single-organism process	BP	0.149218139	-0.965306677	-0.005280552	1.52336568	5.253108871	0.154174188	0.307637209	PREDICTED: homeobox protein B-H1-like
RS006927	GO:0044699	single-organism process	BP	-0.523545819	-0.19238077	0.403170712	9.665847911	5.252945184	0.154185014	0.307637209	PREDICTED: mitochondrial-processing peptidase subunit beta
RS005391	GO:0044699	single-organism process	BP	-1.075324442	-0.478499139	-0.682066037	1.522651837	5.251404954	0.154286913	0.307781317	PREDICTED: melatonin receptor type 1B-A-like
RS015426	GO:0044699	single-organism process	BP	0.395205204	-1.655341944	-0.943243357	0.885746424	5.240240588	0.15502743	0.308926497	PREDICTED: protein Wnt-1
RS003706	GO:0044699	single-organism process	BP	-0.086259747	-0.426738092	-0.697826068	8.567985902	5.233843645	0.155453241	0.309481207	pleiotrophin-like protein
RS012759	GO:0044699	single-organism process	BP	0.271971861	0.067389685	-0.299949221	4.772548689	5.232066304	0.155571745	0.309668177	PREDICTED: probable methylthioribulose-1-phosphate dehydratase
RS009644	GO:0044699	single-organism process	BP	0.608522719	0.516200541	0.329168013	5.056830245	5.228808488	0.15578918	0.309954019	PREDICTED: peptide chain release factor 1-like, mitochondrial
RS006570	GO:0044699	single-organism process	BP	0.568241673	0.482723202	0.243074239	4.31572301	5.224099031	0.156104009	0.310531337	PREDICTED: DNA repair protein XRCC3
RS014310	GO:0044699	single-organism process	BP	-0.394503871	-0.23364982	-0.939036808	2.647212913	5.221628248	0.156269422	0.310811292	PREDICTED: neuronal acetylcholine receptor subunit alpha-7
RS007988	GO:0044699	single-organism process	BP	-0.369402898	-0.504998826	-0.58785137	3.966519503	5.216602306	0.156606406	0.311284892	PREDICTED: guanylate cyclase soluble subunit beta-1
RS004262	GO:0044699	single-organism process	BP	-0.304556625	0.103631535	0.676510563	10.41026235	5.204184057	0.157441978	0.312748307	PREDICTED: ATP synthase lipid-binding protein, mitochondrial
RS002640	GO:0044699	single-organism process	BP	-0.121065485	0.449419508	0.131214602	8.120561122	5.199872229	0.157733084	0.313129013	PREDICTED: MAP7 domain-containing protein 1-like
RS008006	GO:0044699	single-organism process	BP	-1.537762826	-0.128930159	-0.462894442	1.415657251	5.194123574	0.158121985	0.31385158	PREDICTED: beta-1,3-galactosyltransferase 1-like
RS007787	GO:0044699	single-organism process	BP	-0.305912555	0.11337882	0.601620189	9.43857381	5.186627171	0.15863048	0.314655166	PREDICTED: ATP synthase subunit b, mitochondrial
RS008202	GO:0044699	single-organism process	BP	0.24252685	0.342844048	-0.965951499	1.493557986	5.185516154	0.158705973	0.314677881	PREDICTED: voltage-dependent calcium channel gamma-5 subunit
RS010273	GO:0044699	single-organism process	BP	-0.896135709	-0.199190815	-1.162541175	1.102505166	5.184035257	0.158806653	0.314813629	PREDICTED: pancreatic triacylglycerol lipase-like
RS007576	GO:0044699	single-organism process	BP	-0.644577079	-0.203811144	0.166523241	2.787509599	5.183496259	0.158843312	0.314836743	PREDICTED: protein lin-9 homolog
RS001690	GO:0044699	single-organism process	BP	-0.6256747	-0.444109809	-0.279387949	4.197593305	5.180609453	0.159039788	0.31512698	PREDICTED: mitogen-activated protein kinase kinase kinase 1
RS015270	GO:0044699	single-organism process	BP	0.522835531	-0.133237839	1.37147116	0.709937191	5.174487982	0.159457174	0.315692517	Longitudinals lacking protein, isoforms A/B/D/L
RS007152	GO:0044699	single-organism process	BP	-0.79033743	-0.938189121	-1.446925399	0.97652372	5.151356345	0.161043715	0.317897264	PREDICTED: neuroligin-4, Y-linked-like
RS002769	GO:0044699	single-organism process	BP	-0.180596666	-0.510025269	-0.494314256	5.266272897	5.147923967	0.161280396	0.318264698	PREDICTED: mitochondrial 2-oxoglutarate/malate carrier protein
RS000666	GO:0044699	single-organism process	BP	-0.080539122	0.013403368	0.652885093	9.136399161	5.147116783	0.161336103	0.31832475	PREDICTED: ATP synthase subunit g, mitochondrial
RS012430	GO:0044699	single-organism process	BP	0.261867046	0.422261115	-0.14760571	4.558549487	5.143813334	0.161564277	0.318571648	PREDICTED: UDP-N-acetylglucosamine--dolichyl-phosphate N-acetylglucosaminephosphotransferase
RS003918	GO:0044699	single-organism process	BP	-0.223881737	-0.404666274	-0.599000058	7.422761893	5.141574758	0.161719072	0.318680956	PREDICTED: 6-phosphogluconate dehydrogenase, decarboxylating
RS006076	GO:0044699	single-organism process	BP	-0.399500112	-0.234112198	-0.791572834	3.218719557	5.135126595	0.162165734	0.319411206	PREDICTED: cationic amino acid transporter 4
RS009932	GO:0044699	single-organism process	BP	0.560077045	0.563075794	0.51794201	5.62049798	5.134655651	0.162198402	0.319425593	PREDICTED: hexaprenyldihydroxybenzoate methyltransferase, mitochondrial
RS005979	GO:0044699	single-organism process	BP	0.60197583	0.542202012	-0.014023095	8.718917948	5.122655216	0.16303292	0.320754029	PREDICTED: adenosylhomocysteinase
RS010388	GO:0044699	single-organism process	BP	-0.099542584	-0.346736555	-0.781715474	4.088145889	5.122392447	0.163051238	0.320754029	PREDICTED: phosducin-like protein
RS011782	GO:0044699	single-organism process	BP	-0.750002623	-0.673657332	-0.855032559	1.947701654	5.11964637	0.16324279	0.321030558	PREDICTED: WNT1-inducible-signaling pathway protein 1
RS010553	GO:0044699	single-organism process	BP	-0.343321114	-1.029907294	-1.07262003	5.072393174	5.119779104	0.163233526	0.321030558	PREDICTED: cholinesterase
RS012289	GO:0044699	single-organism process	BP	-0.142508395	0.116281098	1.510012562	0.385772306	5.101772345	0.16449477	0.322988327	RNA directed DNA polymerase reverse transcriptase
RS006834	GO:0044699	single-organism process	BP	0.290732775	-0.325890232	-1.403209591	0.687144952	5.100420183	0.164589848	0.323124637	defective proventriculus
RS002494	GO:0044699	single-organism process	BP	0.23516431	-0.326148106	0.31597262	3.648514777	5.092877143	0.165121192	0.323864864	PREDICTED: proton-coupled folate transporter
RS008395	GO:0044699	single-organism process	BP	0.758334632	-0.043191485	0.423582919	3.204444173	5.092897421	0.165119762	0.323864864	PREDICTED: transient receptor potential cation channel protein painless
RS010331	GO:0044699	single-organism process	BP	0.144296164	0.303288083	0.51514905	5.774666221	5.090804659	0.165267464	0.324101282	PREDICTED: mannose-1-phosphate guanyltransferase beta
RS002856	GO:0044699	single-organism process	BP	-0.890643141	-0.642130738	-0.327432805	2.814706402	5.087431306	0.165505808	0.324467644	PREDICTED: ras-related protein Rap-2a
RS013471	GO:0044699	single-organism process	BP	-0.268550146	-0.73606256	-0.856219193	2.957884939	5.081197756	0.165947091	0.325128613	PREDICTED: gamma-tubulin complex component 3
RS001719	GO:0044699	single-organism process	BP	0.056240993	0.353605259	0.507377244	7.900530978	5.074050324	0.16645443	0.325769556	PREDICTED: trifunctional enzyme subunit beta, mitochondrial
RS013705	GO:0044699	single-organism process	BP	-0.27093063	-1.214537102	0.444444118	1.807352185	5.071958687	0.166603174	0.325961122	AGAP004577-PA

RS007666	GO:0044699	single-organism process	BP	-0.598394258	-0.634131169	-0.344891733	4.649544539	5.068273893	0.166865518	0.326269885	PREDICTED: putative adenosylhomocysteinase 2
RS014216	GO:0044699	single-organism process	BP	-0.410521539	-0.222608491	-0.785892482	7.084822935	5.060121692	0.167447305	0.327255021	PREDICTED: testican-1
RS014862	GO:0044699	single-organism process	BP	-0.717021268	-0.337097413	0.432780493	11.96886137	5.0593915	0.167499508	0.327306254	PREDICTED: alpha-actinin, sarcomeric
RS010369	GO:0044699	single-organism process	BP	0.147175962	0.244403927	0.543037161	6.747114426	5.057948766	0.167602698	0.327457087	PREDICTED: DIS3-like exonuclease 2
RS000918	GO:0044699	single-organism process	BP	0.439097278	0.010140394	-0.056475099	4.235800708	5.05319332	0.16794325	0.32791896	PREDICTED: DNA repair protein complementing XP-A cells homolog
RS010155	GO:0044699	single-organism process	BP	0.538451032	0.273103235	0.187832848	6.01569993	5.050627616	0.168127258	0.328181548	PREDICTED: transforming growth factor-beta receptor-associated protein 1
RS004892	GO:0044699	single-organism process	BP	-0.767060843	-0.510725904	-0.665741296	2.855255385	5.048273456	0.168296261	0.328455463	PREDICTED: somatostatin receptor type 2-like
RS007741	GO:0044699	single-organism process	BP	0.107251572	0.288771113	-0.421173633	3.710462745	5.044015914	0.168602311	0.328899861	PREDICTED: serine/threonine-protein kinase PAK 3
RS003405	GO:0044699	single-organism process	BP	-1.140128483	-0.638902826	-0.572644895	5.169618842	5.041425771	0.168788757	0.329161599	PREDICTED: integrin alpha-PS4-like
RS015132	GO:0044699	single-organism process	BP	-0.311288053	0.021217655	0.441252337	8.852842665	5.035692944	0.169202112	0.329814486	PREDICTED: ATP synthase subunit O, mitochondrial
RS011147	GO:0044699	single-organism process	BP	-0.188033853	-0.620082869	-0.241475019	3.95825095	5.034776157	0.169268303	0.329872366	PREDICTED: 2-aminoethanethiol dioxygenase-like
RS001012	GO:0044699	single-organism process	BP	-0.050637771	0.65837124	0.220417241	5.028311487	5.034189195	0.169310694	0.329872366	PREDICTED: sodium/calcium exchanger 2
RS005142	GO:0044699	single-organism process	BP	-1.030388396	-1.05356009	-1.275985831	1.089542545	5.023698294	0.170070041	0.330636322	PREDICTED: methylenetetrahydrofolate reductase
RS003115	GO:0044699	single-organism process	BP	0.149468626	-0.331087446	-0.225382824	5.139921936	5.023083349	0.170114651	0.330665186	PREDICTED: structure-specific endonuclease subunit slx1
RS001935	GO:0044699	single-organism process	BP	-0.556723983	-0.187232259	0.067267248	4.269623268	5.022769816	0.170137399	0.330665186	PREDICTED: mitogen-activated protein kinase 14B-like
RS002485	GO:0044699	single-organism process	BP	-0.948741805	-0.406017768	0.30302886	2.771982877	5.021612128	0.170221421	0.330665996	PREDICTED: tubulin alpha-2/alpha-4 chain-like
RS010665	GO:0044699	single-organism process	BP	0.402212319	0.474203193	0.468533425	6.815729487	5.021744853	0.170211786	0.330665996	PREDICTED: mannose-1-phosphate guanyltransferase alpha-A
RS014542	GO:0044699	single-organism process	BP	0.536529737	0.713142956	-0.037940125	3.557905423	5.0204721	0.1703042	0.33073417	PREDICTED: ATP-dependent DNA helicase PIF1
RS005728	GO:0044699	single-organism process	BP	-0.003940707	-0.83645467	-0.385133659	3.698580247	5.012712258	0.170868653	0.331676915	PREDICTED: adenine phosphoribosyltransferase
RS006122	GO:0044699	single-organism process	BP	-0.228454338	0.117824137	0.242789183	5.731526127	5.009554387	0.171098861	0.331919139	PREDICTED: serine/threonine-protein kinase OSR1
RS005650	GO:0044699	single-organism process	BP	-0.263569768	-0.537739458	-1.085021089	2.249426757	5.007889356	0.171220358	0.332052538	PREDICTED: FMRFamide-related peptides
RS004629	GO:0044699	single-organism process	BP	-0.227092865	-0.656235331	-0.597841111	3.612233491	5.00372413	0.171524648	0.332335603	PREDICTED: DNA polymerase iota
RS008531	GO:0044699	single-organism process	BP	0.524536046	0.534614429	0.42432679	6.09351041	5.004019427	0.171503059	0.332335603	PREDICTED: peroxisome biogenesis factor 1
RS001165	GO:0044699	single-organism process	BP	0.61157654	0.563908095	0.587673492	4.578939757	4.998339378	0.171918784	0.332846379	PREDICTED: DNA-directed RNA polymerase I subunit RPA12
RS001954	GO:0044699	single-organism process	BP	-0.511023769	-1.121622332	-0.950494549	1.29802559	4.996217481	0.172074329	0.333041969	PREDICTED: major facilitator superfamily domain-containing protein 6-like
RS011641	GO:0044699	single-organism process	BP	-2.074551338	-0.763334889	-1.763867752	0.058250841	4.989449686	0.172571322	0.333696182	PREDICTED: exostosin-1
RS013885	GO:0044699	single-organism process	BP	-0.653620991	-1.203365067	-0.496719101	1.563650614	4.985052582	0.172894945	0.334270639	PREDICTED: tyrosine decarboxylase
RS014860	GO:0044699	single-organism process	BP	0.206368674	0.225727975	0.534712532	5.744307673	4.979732854	0.173287234	0.334874854	PREDICTED: general transcription factor IIH subunit 4
RS013006	GO:0044699	single-organism process	BP	-0.538272733	-0.817432042	-1.008287916	2.921270255	4.979088813	0.173334784	0.334904704	PREDICTED: exportin-4-like
RS014641	GO:0044699	single-organism process	BP	-0.068024044	0.218551578	0.40443894	6.971557568	4.978803328	0.173355865	0.334904704	PREDICTED: ras-related protein Rab-39B
RS004391	GO:0044699	single-organism process	BP	-0.797503593	-0.701477499	-0.082653753	3.597373187	4.977631633	0.173442413	0.334969137	PREDICTED: rap guanine nucleotide exchange factor 4
RS003210	GO:0044699	single-organism process	BP	0.457872677	0.832393815	0.705350714	3.97283813	4.972582281	0.173815849	0.33553599	PREDICTED: solute carrier family 12 member 8
RS001929	GO:0044699	single-organism process	BP	0.509975884	0.459388731	-0.309620866	3.64304367	4.971420074	0.173901909	0.335650673	PREDICTED: ataxin-1-like
RS002853	GO:0044699	single-organism process	BP	-0.67671843	-0.979269865	-0.818772724	2.537388531	4.961141773	0.174664748	0.336785905	PREDICTED: mitogen-activated protein kinase 1
RS012145	GO:0044699	single-organism process	BP	0.15858941	0.02286931	0.43233142	5.36574869	4.959483806	0.174788094	0.336947995	PREDICTED: SRA stem-loop-interacting RNA-binding protein, mitochondrial
RS012947	GO:0044699	single-organism process	BP	-0.799151497	-0.339003975	-1.415086944	0.6304954	4.951586688	0.175376725	0.337782643	PREDICTED: protein Wnt-7b
RS012451	GO:0044699	single-organism process	BP	0.194839248	-0.091247506	-0.367796028	4.528361561	4.951792882	0.175361332	0.337782643	PREDICTED: transcription factor stalky
RS009851	GO:0044699	single-organism process	BP	0.04948956	-0.27876713	-0.396957993	7.248526228	4.947807104	0.175659103	0.338109543	PREDICTED: splicing factor 3A subunit 1
RS002009	GO:0044699	single-organism process	BP	0.194963927	0.862223882	0.465391228	2.435073123	4.94708067	0.175713424	0.338162418	PREDICTED: ankyrin repeat and SOCS box protein 12
RS005862	GO:0044699	single-organism process	BP	-0.063721	0.064308402	0.483380733	6.661557905	4.943935204	0.175948819	0.338511981	pastrel
RS003663	GO:0044699	single-organism process	BP	-1.647383031	-1.021936201	-0.831759249	0.547854387	4.94039762	0.176213912	0.338923324	fork head
RS007114	GO:0044699	single-organism process	BP	0.028844431	-0.280621663	-0.465664762	7.87805453	4.935906797	0.176550976	0.339463052	PREDICTED: acidic leucine-rich nuclear phosphoprotein 32 family member A
RS002609	GO:0044699	single-organism process	BP	-0.442925672	-0.118232305	0.103593655	5.128787566	4.916894353	0.17798468	0.34160332	PREDICTED: rap1 GTPase-activating protein 1
RS007406	GO:0044699	single-organism process	BP	1.53218285	1.416234104	0.234676903	0.509107572	4.915967777	0.17805483	0.341676447	PREDICTED: sodium-independent sulfate anion transporter-like
RS001311	GO:0044699	single-organism process	BP	0.38050087	0.40875068	0.443186312	5.192231702	4.912765487	0.17829747	0.342037828	PREDICTED: sorting nexin-12
RS000039	GO:0044699	single-organism process	BP	-0.207479061	0.295867587	-0.138315971	5.999069609	4.911691996	0.178378879	0.342089782	PREDICTED: calcium/calmodulin-dependent 3',5'-cyclic nucleotide phosphodiesterase 1A-like
RS006240	GO:0044699	single-organism process	BP	-0.456558706	-0.421545107	-0.731005218	5.012200364	4.908981407	0.178584593	0.342327908	PREDICTED: annexin B9-like
RS010450	GO:0044699	single-organism process	BP	-0.765817623	-0.824667038	-0.145855989	2.532327432	4.90912998	0.178573311	0.342327908	PREDICTED: homeotic protein distal-less
RS005738	GO:0044699	single-organism process	BP	-0.378294814	-0.071697406	0.312780479	7.87711044	4.907000397	0.178735077	0.342512106	PREDICTED: putative succinate dehydrogenase [ubiquinone] cytochrome b small subunit, mitochondrial
RS000125	GO:0044699	single-organism process	BP	0.391186248	0.173793981	-0.178124604	4.016953676	4.900984056	0.179192828	0.343180425	PRDM9
RS007390	GO:0044699	single-organism process	BP	-0.350929593	-1.171722101	-0.259139814	1.654126139	4.900550276	0.179225874	0.343191525	PREDICTED: open rectifier potassium channel protein 1
RS011518	GO:0044699	single-organism process	BP	0.346347099	-0.325462494	-0.882224584	1.61921728	4.89701379	0.179495505	0.343290264	PREDICTED: E3 ubiquitin-protein ligase UHRF1
RS014676	GO:0044699	single-organism process	BP	-0.54914954	-0.212189377	-0.188111039	4.762158836	4.897349239	0.179469913	0.343290264	PREDICTED: serine/threonine-protein kinase Warts
RS005987	GO:0044699	single-organism process	BP	-0.712414943	0.091463404	0.025113033	3.724448751	4.897471924	0.179460554	0.343290264	PREDICTED: ral guanine nucleotide dissociation stimulator
RS009720	GO:0044699	single-organism process	BP	0.537516493	0.386389967	0.319813219	6.16758859	4.899477131	0.179307653	0.343290264	PREDICTED: probable queuine tRNA-ribosyltransferase
RS006946	GO:0044699	single-organism process	BP	-0.485699781	-0.103029911	-0.223784241	6.515830906	4.898324769	0.179395508	0.343290264	PREDICTED: ornithine decarboxylase antizyme 1
RS006865	GO:0044699	single-organism process	BP	-0.674092785	-0.804033489	-0.307415293	2.653923004	4.882017443	0.180643088	0.345170543	PREDICTED: soluble guanylate cyclase 89Db-like
RS014094	GO:0044699	single-organism process	BP	-0.881856016	-1.069505928	-1.868414774	0.170558582	4.879025282	0.180872881	0.345504787	brinker
RS006350	GO:0044699	single-organism process	BP	0.249286194	0.090755988	-0.343024603	4.752495149	4.870415953	0.181535589	0.34667152	PREDICTED: KRR1 small subunit processome component homolog
RS011898	GO:0044699	single-organism process	BP	-0.584444256	-0.264407425	-0.156097104	5.321728706	4.869243988	0.181625977	0.34674071	PREDICTED: serine/threonine-protein kinase 25
RS002228	GO:0044699	single-organism process	BP	-0.754859268	-0.244716646	0.001009	3.254531674	4.863581224	0.182063313	0.347304659	PREDICTED: guanine nucleotide-binding protein G(s) subunit alpha
RS012974	GO:0044699	single-organism process	BP	0.386362631	0.350475987	0.590578301	4.958398051	4.857275707	0.182551447	0.347919632	PREDICTED: mitochondrial import inner membrane translocase subunit Tim16
RS004430	GO:0044699	single-organism process	BP	-0.579491375	-0.057917442	0.425475066	2.309902759	4.852857272	0.182894224	0.348520179	Glutamate [NMDA] receptor subunit 3A
RS014875	GO:0044699	single-organism process	BP	-1.243468354	0.025730545	0.504864087	0.758811237	4.849831777	0.183129286	0.348757028	PREDICTED: titin-like
RS004747	GO:0044699	single-organism process	BP	0.134852391	0.085980981	0.695845276	3.134277611	4.847982789	0.183273079	0.348978101	PREDICTED: PDZ domain-containing protein 8
RS003264	GO:0044699	single-organism process	BP	-0.247243349	0.028958919	-0.383654367	5.924635862	4.844804899	0.183520467	0.34929073	PREDICTED: katanin p80 WD40 repeat-containing subunit B1
RS014449	GO:0044699	single-organism process	BP	0.02252753	-0.435635201	-0.256205382	5.053779272	4.837178753	0.184115409	0.349694714	PREDICTED: putative DNA helicase Ino80
RS002164	GO:0044699	single-organism process	BP	-0.115674773	-0.565652978	-0.630495566	4.331344889	4.839306305	0.18394925	0.349694714	PREDICTED: iron/zinc purple acid phosphatase-like protein
RS011682	GO:0044699	single-organism process	BP	-0.971592876	-0.747634782	-0.371222458	1.955229859	4.839217081	0.183956215	0.349694714	Metabotropic glutamate receptor 2
RS014693	GO:0044699	single-organism process	BP	0.609023931	0.599563264	0.558081139	3.442400107	4.837033933	0.184126724	0.349694714	PREDICTED: DDB1- and CUL4-associated factor 7
RS002607	GO:0044699	single-organism process	BP	-0.193226241	-0.559233157	-0.467691917	4.445319078	4.836745968	0.184149226	0.349694714	PREDICTED: DNA-directed RNA polymerase I subunit RPA2
RS015406	GO:0044699	single-organism process	BP	-0.548835798	-0.34994381	-0.083936965	5.176471396	4.839807661	0.183910115	0.349694714	PREDICTED: sodium/potassium-transporting ATPase subunit beta-2
RS013883	GO:0044699	single-organism process	BP	-1.119764452	-0.852715943	0.034262468	6.463866364	4.838736295	0.183993753	0.349694714	PREDICTED: aromatic-L-amino-acid decarboxylase-like
RS009223	GO:0044699	single-organism process	BP	0.341121543	0.430957586	0.5323603	4.700673698	4.836241026	0.184188688	0.349716921	PREDICTED: peptidyl-tRNA hydrolase ICT1, mitochondrial
RS010115	GO:0044699	single-organism process	BP	0.049934466	-0.018094849	-0.700558611	2.678564854	4.834458418	0.184328067	0.349780004	PREDICTED: tryptophan 5-hydroxylase 1
RS008549	GO:0044699	single-organism process	BP	0.14330619	0.406068691	0.419243198	5.37556194	4.834681525	0.184310617	0.349780004	PREDICTED: BRCA1-A complex subunit BRE
RS000834	GO:0044699	single-organism process	BP	0.027272592	0.531296961	0.204952902	4.410747552	4.828431315	0.184800046	0.350402255	PREDICTED: MAP kinase-activated protein kinase 2
RS012633	GO:0044699	single-organism process	BP	0.3400712	-0.042971241	-0.128072376	5.95450125	4.828059073	0.184829233	0.350404841	PREDICTED: probable C-mannosyltransferase DPY19L1
RS006281	GO:0044699	single-organism process	BP	0.246368129	-0.248191376	-0.206269072	6.321107095	4.821950304	0.18530883	0.351024091	PREDICTED: tetratricopeptide repeat protein 21B-like
RS008784	GO:0044699	single-organism process	BP	-0.593134085	-0.245991961	-0.60199577	5.264515055	4.821997138	0.185305148	0.351024091	guanine nucleotide exchange factor
RS005557	GO:0044699	single-organism process	BP	0.303265508	0.259353221	0.507067636	7.094617312	4.820839095	0.185396195	0.351045831	PREDICTED: pyridine nucleotide-disulfide oxidoreductase domain-containing protein 1
RS012838	GO:0044699	single-organism process	BP	0.378531586	0.499243749	0.391510283	7.013197	4.820929049	0.185389121	0.351045831	PREDICTED: eukaryotic peptide chain release factor GTP-binding subunit ERF3A
RS015486	GO:0044699	single-organism process	BP	0.142496913	0.244726276	0.445776716	6.006109719	4.814517426	0.18589395	0.351629378	PREDICTED: heme oxygenase
RS000028	GO:0044699	single-organism process	BP	0.222938088	0.127584655	-0.473780265	3.781246522	4.810530689	0.186208498	0.35206573	PREDICTED: group XV phospholipase A2-like
RS001425	GO:0044699	single-organism process	BP	-0.206238113	-0.472926335	0.121297171	8.034122348	4.806328385	0.186540593	0.352481955	PREDICTED: protein tyrosine phosphatase type IVA 1
RS009880	GO:0044699	single-organism process	BP	0.343349524	0.094460002	-0.701170484	2.346330806	4.805230969	0.186627409	0.352593098	PREDICTED: apoptosis regulator BAX
RS014523	GO:0044699	single-organism process	BP	-0.340895935	-0.487246878	-0.330281542	7.025294852	4.789646773	0.187864351	0.354557715	PREDICTED: ras-related protein Rab-5B
RS011603	GO:0044699	single-organism process	BP	0.360137787	0.174900039	-0.094452522	5.901142243	4.78995928	0.187839472	0.354557715	PREDICTED: syntaxin-17
RS002766	GO:0044699	single-organism process	BP	-0.496155234	-0.78863534	-0.632950682	4.168210328	4.786567039	0.188109698	0.354852096	PREDICTED: solute carrier family 25 member 36-A
RS003363	GO:0044699	single-organism process	BP	0.16959335	0.457195032	0.086889481	6.839770755	4.786274295	0.188133035	0.354852096	PREDICTED: E3 ubiquitin-protein ligase synoviolin
RS001757	GO:0044699	single-organism process	BP	-0.581179614	-0.137368528	0.380454103	2.203664504	4.783768521	0.188332901	0.355069545	PREDICTED: ras-related protein M-Ras-like
RS005814	GO:0044699	single-organism process	BP	-0.759832602	-0.899813821	-0.376932251	1.964440063	4.782071734	0.188468354	0.355219028	PREDICTED: dopamine receptor 1
RS004695	GO:0044699	single-organism process	BP	0.513915231	0.257267281	0.117948351	5.229345941	4.780656224	0.188581421	0.355219028	PREDICTED: replication protein A 14 kDa subunit-like
RS015488	GO:0044699	single-organism process	BP	0.477041226	0.549897843	0.422724836	7.058766336	4.781664348	0.188500888	0.355219028	PREDICTED: metaxin-1
RS003248	GO:0044699	single-organism process	BP	-0.403402665	-0.740390341	-0.54475869	3.388113846	4.780916092	0.188560659	0.355219028	PREDICTED: synaptojanin-1
RS009307	GO:0044699	single-organism process	BP	0.178587151	0.573714687	0.468396304	6.05696173	4.773689809	0.189138806	0.35594951	PREDICTED: guanine nucleotide-binding protein subunit gamma-1
RS005996	GO:0044699	single-organism process	BP	0.10545181	-0.331820343	0.053049731	6.441303762	4.772916155	0.189200801	0.356012982	PREDICTED: thyroid receptor-interacting protein 11-like
RS015035	GO:0044699	single-organism process	BP	-0.486032243	-0.438869682	-0.112800307	5.262898327	4.77200592	0.189273765	0.356097071	PREDICTED: solute carrier family 2, facilitated glucose transporter member 10-like
RS005546	GO:0044699	single-organism process	BP	0.22008949	-0.311385066	0.164057107	5.968386842	4.769105886	0.189506405	0.356428266	PREDICTED: Fanconi anemia group D2 protein
RS010397	GO:0044699	single-organism process	BP	-0.247875997	-0.791333928	-1.062124793	1.988946652	4.764358819	0.18988779	0.356932362	PREDICTED: 15-hydroxyprostaglandin dehydrogenase [NAD(+)]-like
RS009196	GO:0044699	single-organism process	BP	0.36522098	0.502228647	0.180964663	4.487979595	4.761038407	0.190154981	0.357221334	PREDICTED: protein krueppel-like
RS004772	GO:0044699	single-organism process	BP	0.470924495	0.409139587	0.16242472	6.145484968	4.757758559	0.190419252	0.357611103	PREDICTED: lys-63-specific deubiquitinase BRCC36
RS002678	GO:0044699	single-organism process	BP	-0.444950422	-1.104137516	-0.306756919	3.80292744	4.750004591	0.191045384	0.358244364	PREDICTED: fatty acid synthase-like
RS011065	GO:0044699	single-organism process	BP	-0.875027536	-0.699860567	-0.617836046	1.97912169	4.744920077	0.191457	0.358704174	PREDICTED: glycoprotein 3-alpha-L-fucosyltransferase A
RS014914	GO:0044699	single-organism process	BP	-0.388013822	-0.3450635	0.180623926	3.998879616	4.743988992	0.191532465	0.358738826	PREDICTED: GTP-binding protein Rhes
RS011272	GO:0044699	single-organism process	BP	-0.449550793	-0.842262029	0.317704025	1.68576224	4.742178986	0.191679247	0.358960362	PREDICTED: transient receptor potential-gamma protein
RS006954	GO:0044699	single-organism process	BP	-0.240760684	0.005140101	0.617138509	3.425234611	4.731183517	0.192573178	0.360205941	PREDICTED: arf-GAP with Rho-GAP domain, ANK repeat and PH domain-containing protein 1
RS015084	GO:0044699	single-organism process	BP	-1.170238128	0.951638351	0.245785168	0.310335995	4.729152449	0.192738728	0.36030155	PREDICTED: G-protein coupled receptor moody
RS003616	GO:0044699	single-organism process	BP	-0.633195871	-0.632196145	-0.816138038	2.788858931	4.729664269	0.192696998	0.36030155	PREDICTED: complexin
RS011016	GO:0044699	single-organism process	BP	0.197192571	-0.15030133	-0.347371365	4.855516765	4.72645006	0.192959203	0.360660166	PREDICTED: coiled-coil and C2 domain-containing protein 2A
RS008232	GO:0044699	single-organism process	BP	-0.666480677	-0.601520392	-0.268527096	3.191166108	4.724370799	0.193128999	0.360923968	PREDICTED: rho GTPase-activating protein 1

RS011432	GO:0044699	single-organism process	BP	-0.206587007	-0.213127101	0.237104291	5.945152012	4.720197897	0.193470187	0.3613181	PREDICTED: cytochrome c oxidase copper chaperone
RS004490	GO:0044699	single-organism process	BP	0.426070235	0.412678234	0.244675287	5.869522534	4.714478533	0.19393873	0.361901662	PREDICTED: uncharacterized aarF domain-containing protein kinase 5
RS006401	GO:0044699	single-organism process	BP	-0.3161354	-0.264283002	-0.746985347	4.177079266	4.714468898	0.19393952	0.361901662	PREDICTED: CDP-diacylglycerol--inositol 3-phosphatidyltransferase
RS004093	GO:0044699	single-organism process	BP	0.012526749	0.260466659	-0.204883198	5.895452712	4.7145195	0.19393537	0.361901662	PREDICTED: beta-TrCP
RS012958	GO:0044699	single-organism process	BP	-0.475751151	-0.659652499	-0.592909608	4.258116367	4.709280662	0.194365468	0.362426678	PREDICTED: inositol monophosphatase 1-like
RS002725	GO:0044699	single-organism process	BP	0.495615238	-0.317090284	-0.119643975	2.808672366	4.708478783	0.194431379	0.362443574	PREDICTED: flap endonuclease GEN
RS010695	GO:0044699	single-organism process	BP	0.518417779	0.062491693	-0.045494596	3.878001861	4.706009864	0.194634444	0.362661072	PREDICTED: dihydrofolate reductase
RS013062	GO:0044699	single-organism process	BP	-0.00162788	-0.815422885	-0.945361754	1.375980209	4.702121991	0.194954616	0.363203911	PREDICTED: transient receptor potential channel pyrexia
RS008733	GO:0044699	single-organism process	BP	-0.069301153	-0.696190609	0.565661049	1.222507914	4.700705743	0.195071368	0.363260212	PREDICTED: pickpocket protein 28-like
RS005692	GO:0044699	single-organism process	BP	-0.714519036	-1.046561661	-0.368392221	1.614510198	4.692226236	0.195771761	0.364187528	PREDICTED: ras-related and estrogen-regulated growth inhibitor
RS009963	GO:0044699	single-organism process	BP	-0.732608205	-0.778239432	-0.747787888	2.188684887	4.691092768	0.195865561	0.364308208	PREDICTED: oxysterol-binding protein 1
RS013862	GO:0044699	single-organism process	BP	0.062570052	0.429054094	0.297850698	5.081715497	4.689811588	0.195971635	0.364345296	PREDICTED: alpha-ketoglutarate-dependent taurine dioxygenase-like
RS001682	GO:0044699	single-organism process	BP	0.427496794	0.162370784	-0.166794293	4.20654832	4.689803666	0.195972291	0.364345296	PREDICTED: putative Dol-P-Glc:Glc(2)Man(9)GlcNAc(2)-PP-Dol alpha-1,2-glucosyltransferase
RS009359	GO:0044699	single-organism process	BP	0.28026819	-0.408253169	0.031587479	4.49329609	4.682466065	0.196580833	0.365311231	PREDICTED: probable malonyl-CoA-acyl carrier protein transacylase, mitochondrial
RS001462	GO:0044699	single-organism process	BP	0.742880569	0.623358898	0.408018193	6.10251623	4.680620584	0.196734164	0.36538417	PREDICTED: tRNA modification GTPase GTPBP3, mitochondrial
RS010236	GO:0044699	single-organism process	BP	0.404534573	0.389941764	0.598338898	6.378798066	4.679342248	0.196840439	0.365473757	PREDICTED: prolyl 3-hydroxylase OGFOD1
RS003712	GO:0044699	single-organism process	BP	0.03100834	-0.555218793	-0.842108264	3.063114051	4.678534462	0.196907622	0.365525714	PREDICTED: probable dolichyl pyrophosphate Glc1Man9GlcNAc2 alpha-1,3-glucosyltransferase
RS015204	GO:0044699	single-organism process	BP	-0.40385631	-0.267684874	0.256413441	8.673377735	4.675269768	0.197179364	0.365763575	PREDICTED: NADH dehydrogenase [ubiquinone] flavoprotein 1, mitochondrial
RS014191	GO:0044699	single-organism process	BP	0.388726094	0.139670183	0.008081604	6.147606593	4.675024038	0.197199831	0.365763575	PREDICTED: DNA-directed RNA polymerases I, II, and III subunit RPABC1
RS005980	GO:0044699	single-organism process	BP	-0.519964769	-0.115820264	0.014177289	6.649793844	4.674091701	0.197277507	0.365853766	PREDICTED: V-type proton ATPase subunit d
RS001430	GO:0044699	single-organism process	BP	0.020093121	-0.191599336	0.388272246	4.621577215	4.669696933	0.197644032	0.366362711	PREDICTED: hydroxymethylglutaryl-CoA lyase, mitochondrial
RS005140	GO:0044699	single-organism process	BP	-0.416469633	-0.263839851	-0.01550935	6.34712109	4.659101598	0.198530293	0.36774386	PREDICTED: D-2-hydroxyglutarate dehydrogenase, mitochondrial
RS008188	GO:0044699	single-organism process	BP	-0.933844664	-0.891654518	-0.589913532	1.772787712	4.652646048	0.199072086	0.368476463	PREDICTED: slit homolog 3 protein
RS003391	GO:0044699	single-organism process	BP	-0.542480622	-0.539981143	-0.585509501	9.138318653	4.6421131	0.199959035	0.36995506	ubiquitin
RS015319	GO:0044699	single-organism process	BP	0.211353187	-0.36009673	-0.190242368	4.816999791	4.64109435	0.200045016	0.370005424	PREDICTED: intraflagellar transport protein 172 homolog
RS000148	GO:0044699	single-organism process	BP	-0.122107004	0.195755414	0.48968258	6.587092416	4.638860432	0.200233675	0.370299985	PREDICTED: mitochondrial dicarboxylate carrier
RS012770	GO:0044699	single-organism process	BP	0.315811511	-0.092673305	-1.083722411	0.988774343	4.634832629	0.200574249	0.37067814	PREDICTED: kinesin-like protein KIF20B
RS005911	GO:0044699	single-organism process	BP	0.374556135	0.14320806	-0.002684491	6.465311096	4.630699081	0.200924323	0.371149337	PREDICTED: transcription initiation factor TFIID subunit 5
RS005031	GO:0044699	single-organism process	BP	-0.143156983	-0.610278504	-0.891062591	3.276334664	4.626253582	0.201301451	0.37145648	PREDICTED: tyrosine-protein phosphatase 69D
RS011922	GO:0044699	single-organism process	BP	-0.649385456	-0.169072995	-1.344964409	1.159816594	4.617457825	0.202049567	0.372151441	PREDICTED: protein eyes shut
RS012971	GO:0044699	single-organism process	BP	0.144384823	0.048672472	-0.316436227	5.216939055	4.613896001	0.202353248	0.37245905	PREDICTED: ralA-binding protein 1
RS013193	GO:0044699	single-organism process	BP	-0.571318105	-0.638002435	-0.492911161	3.125045391	4.612135042	0.202503545	0.372595481	PREDICTED: gamma-aminobutyric acid receptor subunit beta-like
RS002481	GO:0044699	single-organism process	BP	-0.166331763	-0.74476705	-0.928309039	2.223054766	4.608815039	0.202787186	0.372849849	PREDICTED: 7,8-dihydro-8-oxoguanine triphosphatase
RS007040	GO:0044699	single-organism process	BP	0.784219344	-0.014888112	0.504920578	3.394706463	4.608011433	0.202855897	0.372849849	PREDICTED: H2.0-like homeobox protein
RS009642	GO:0044699	single-organism process	BP	-0.332622261	-0.587763162	-0.788046414	3.037828859	4.607638111	0.202887824	0.372854116	PREDICTED: mutS protein homolog 5-like
RS002889	GO:0044699	single-organism process	BP	-0.128703142	0.3529612	0.286577104	4.360050122	4.605415706	0.203077987	0.373149135	PREDICTED: bifunctional arginine demethylase and lysyl-hydroxylase PSR
RS006340	GO:0044699	single-organism process	BP	-0.481524043	-0.576099618	-0.452634393	4.933016446	4.600128758	0.203531036	0.373808755	PREDICTED: soluble NSF attachment protein
RS012106	GO:0044699	single-organism process	BP	-0.818692691	-0.45460686	0.366734327	1.141769569	4.599841297	0.203555696	0.373808755	Inward rectifier potassium channel 2
RS006788	GO:0044699	single-organism process	BP	-0.425454397	-1.3482247	-0.668715921	1.875814215	4.594118457	0.204047211	0.374383825	PREDICTED: protein Star-like
RS000359	GO:0044699	single-organism process	BP	0.154714236	0.686551175	-0.230261008	1.933392001	4.59137418	0.204283298	0.374707815	PREDICTED: forkhead box protein D3-like
RS008219	GO:0044699	single-organism process	BP	0.352735213	0.394765771	0.053226017	5.309963286	4.590496302	0.204358875	0.374791855	PREDICTED: target of rapamycin complex subunit lst8
RS010613	GO:0044699	single-organism process	BP	0.166802636	0.436915148	0.362481091	6.693929741	4.586856986	0.204672459	0.375257673	PREDICTED: transcription initiation factor IIB
RS009285	GO:0044699	single-organism process	BP	0.085000423	0.40215963	0.387666311	4.994657563	4.58478738	0.204850988	0.375428519	PREDICTED: NADH dehydrogenase [ubiquinone] complex I, assembly factor 7
RS008579	GO:0044699	single-organism process	BP	-0.510716851	-0.374224714	-0.311157995	5.569014869	4.57979745	0.205282024	0.376101515	PREDICTED: NADH-cytochrome b5 reductase 2
RS010546	GO:0044699	single-organism process	BP	0.364633638	0.109816219	-0.173184364	4.655756997	4.573003416	0.205870254	0.37695987	PREDICTED: cilia- and flagella-associated protein 20
RS008973	GO:0044699	single-organism process	BP	0.730569281	0.542878845	0.061507904	5.674408765	4.571253884	0.206021982	0.37712803	PREDICTED: collagen alpha-1(I) chain-like
RS013367	GO:0044699	single-organism process	BP	-0.060901637	-0.335506202	-0.499370525	4.595141744	4.567868374	0.206315885	0.377501418	PREDICTED: palmitoyl-protein thioesterase 1
RS011849	GO:0044699	single-organism process	BP	0.407048694	0.387681559	0.347666334	5.896226356	4.56328191	0.206714666	0.377901655	PREDICTED: integrin-alpha FG-GAP repeat-containing protein 2
RS007095	GO:0044699	single-organism process	BP	0.192687609	0.009365646	-0.454674091	8.985348469	4.560229052	0.206980499	0.378277813	PREDICTED: UDP-glucose:glycoprotein glucosyltransferase
RS006638	GO:0044699	single-organism process	BP	-1.448956343	-1.207961983	-1.300822057	0.363513528	4.558683913	0.207115166	0.378469008	PREDICTED: LIM/homeobox protein Lhx3
RS008564	GO:0044699	single-organism process	BP	0.035044616	-0.3795368	-0.288048528	5.833061411	4.55795336	0.207178866	0.378530486	PREDICTED: tyrosine-protein phosphatase non-receptor type 23
RS005244	GO:0044699	single-organism process	BP	-0.065249759	0.38909471	-0.560413614	7.914524942	4.557440342	0.207223609	0.378541045	PREDICTED: SPARC
RS000111	GO:0044699	single-organism process	BP	-0.493551406	-0.558539536	0.006439579	9.004522239	4.55700476	0.207261605	0.378541045	PREDICTED: succinate dehydrogenase [ubiquinone] iron-sulfur subunit, mitochondrial
RS003701	GO:0044699	single-organism process	BP	0.448313442	0.529646515	0.511182253	6.976340521	4.556497178	0.20730589	0.3785429	PREDICTED: probable deoxyhypusine synthase
RS006209	GO:0044699	single-organism process	BP	0.112226125	-0.275290936	-0.382508167	4.609935365	4.55572841	0.20737298	0.378610512	PREDICTED: acylphosphatase-1-like
RS007975	GO:0044699	single-organism process	BP	-1.218044673	-0.868864741	-1.116123575	1.298086845	4.549037269	0.20795776	0.379403162	PREDICTED: toll-like receptor 13
RS008075	GO:0044699	single-organism process	BP	-0.434191288	-0.074441076	0.254824545	8.687521875	4.547639571	0.208080106	0.379571387	PREDICTED: talin-1
RS003994	GO:0044699	single-organism process	BP	-0.134307551	0.372977114	-0.150034493	4.481208671	4.545193142	0.208294413	0.379792461	PREDICTED: charged multivesicular body protein 3
RS001638	GO:0044699	single-organism process	BP	1.106129118	0.529630066	-0.178427791	1.261230424	4.541867395	0.208586075	0.380067463	PREDICTED: spindle and kinetochore-associated protein 1
RS013960	GO:0044699	single-organism process	BP	-0.472338012	-0.452157922	0.021607712	3.915657005	4.540751566	0.208684017	0.380067463	PREDICTED: multidrug resistance-associated protein 5-like
RS006396	GO:0044699	single-organism process	BP	-0.48548481	-0.011456222	-0.668681102	4.551562016	4.541901165	0.208583112	0.380067463	PREDICTED: endoglucanase 15-like
RS009944	GO:0044699	single-organism process	BP	-0.459546546	-0.584097686	-0.413731607	3.698979882	4.541470139	0.208620939	0.380067463	PREDICTED: 6-phosphogluconolactonase
RS008566	GO:0044699	single-organism process	BP	-0.574477742	-0.469706951	-0.054247022	8.931217496	4.541048439	0.208657955	0.380067463	PREDICTED: NADH-ubiquinone oxidoreductase 75 kDa subunit, mitochondrial
RS012975	GO:0044699	single-organism process	BP	0.040208154	0.239103933	0.847702251	3.944106322	4.53757653	0.208962937	0.380465408	PREDICTED: F-box/LRR-repeat protein 7-like
RS004007	GO:0044699	single-organism process	BP	0.453002142	0.540248202	0.663134739	7.357198428	4.53665787	0.209043703	0.380557444	PREDICTED: 39S ribosomal protein L47, mitochondrial
RS011392	GO:0044699	single-organism process	BP	-0.004739108	-0.111082146	-0.62224778	3.54204359	4.531913122	0.209461313	0.381030706	PREDICTED: general transcription factor IIH subunit 5
RS009973	GO:0044699	single-organism process	BP	0.1676973	0.302067179	0.694181931	3.324934717	4.531641986	0.2094852	0.381030706	PREDICTED: sugar transporter SWEET1
RS009075	GO:0044699	single-organism process	BP	0.454074585	0.27825264	0.575811284	5.679136781	4.531167855	0.209526978	0.381051661	PREDICTED: transcriptional activator cubitus interruptus
RS004971	GO:0044699	single-organism process	BP	-0.846062962	-1.02817738	-0.860554404	2.115405232	4.529503457	0.209673695	0.38126343	PREDICTED: arrestin domain-containing protein 3-like
RS007359	GO:0044699	single-organism process	BP	-0.033186439	-0.296037869	-0.421053457	5.816316114	4.527960563	0.209809787	0.381376047	PREDICTED: putative oxidoreductase GLYR1 homolog
RS007043	GO:0044699	single-organism process	BP	0.600228763	0.40873804	0.100098369	3.870522798	4.523452304	0.210207911	0.3818489	PREDICTED: diablo homolog, mitochondrial-like
RS008715	GO:0044699	single-organism process	BP	0.152832653	0.181455734	0.585787892	4.09558654	4.520522168	0.210467045	0.382129469	PREDICTED: U8 snoRNA-decapping enzyme-like
RS002890	GO:0044699	single-organism process	BP	-0.213107922	-0.035236467	0.494651933	9.00551627	4.518839864	0.210615958	0.382259313	PREDICTED: cytochrome c oxidase subunit 5A, mitochondrial
RS001183	GO:0044699	single-organism process	BP	0.381173523	0.452466843	0.551870902	6.511626778	4.513584138	0.211081809	0.382773838	PREDICTED: golgi-specific brefeldin A-resistance guanine nucleotide exchange factor 1
RS008117	GO:0044699	single-organism process	BP	-0.156476954	0.219557131	0.36239921	5.492050905	4.508719139	0.211513877	0.383446923	PREDICTED: dehydrogenase/reductase SDR family member 7
RS014790	GO:0044699	single-organism process	BP	0.427670174	0.279813355	0.299823815	5.791211765	4.508041725	0.211574105	0.383451075	PREDICTED: enhancer of rudimentary homolog
RS013266	GO:0044699	single-organism process	BP	-0.052572572	-0.278010266	0.268293936	5.046125939	4.506155635	0.211741876	0.383639358	PREDICTED: 4-hydroxy-2-oxoglutarate aldolase, mitochondrial
RS006237	GO:0044699	single-organism process	BP	0.434807153	0.854097634	0.572501888	9.728203859	4.505190752	0.211827751	0.383707537	PREDICTED: 40S ribosomal protein S15Aa
RS005951	GO:0044699	single-organism process	BP	-0.227228711	-0.724219387	-0.592674383	4.090941552	4.502174471	0.212096411	0.383998369	PREDICTED: villin-1
RS003913	GO:0044699	single-organism process	BP	-0.267728215	-0.516651127	-0.317459739	5.939188132	4.498305545	0.212441479	0.384464432	PREDICTED: pre-mRNA-processing factor 6
RS005084	GO:0044699	single-organism process	BP	0.448911254	0.44033824	0.465332767	5.676050757	4.497575834	0.212506619	0.384471824	PREDICTED: coiled-coil-helix-coiled-coil-helix domain-containing protein 7
RS007491	GO:0044699	single-organism process	BP	-0.446508158	-0.228275875	-0.036390422	6.67592503	4.497785152	0.212487932	0.384471824	PREDICTED: NADP-dependent malic enzyme
RS013550	GO:0044699	single-organism process	BP	-0.451027373	-1.090824466	-0.810848917	2.641058017	4.495135897	0.212724565	0.38470034	PREDICTED: transcription factor Sox-2
RS001049	GO:0044699	single-organism process	BP	0.642048509	0.381053105	0.461044754	3.47973328	4.494241054	0.212804548	0.384789731	PREDICTED: F-box/SPRY domain-containing protein 1
RS000915	GO:0044699	single-organism process	BP	0.44594725	0.315990325	0.305853323	6.17815639	4.490252329	0.213161407	0.385158502	PREDICTED: ubiquitin-conjugating enzyme E2 N
RS011879	GO:0044699	single-organism process	BP	0.304820475	0.511797719	0.256613173	7.478764321	4.486494573	0.213498109	0.385600915	PREDICTED: transmembrane emp24 domain-containing protein bai
RS000217	GO:0044699	single-organism process	BP	-0.750015472	-0.258912471	-0.859143521	4.771460381	4.479971735	0.214083735	0.38649234	PREDICTED: innexin inx1
RS007299	GO:0044699	single-organism process	BP	0.694477623	0.166166886	-0.086300779	2.713419968	4.478820934	0.214187209	0.386568317	PREDICTED: substance-P receptor-like
RS002717	GO:0044699	single-organism process	BP	-0.136045677	-0.272610205	0.21743988	7.359575075	4.474417506	0.21458357	0.387079325	PREDICTED: serine/threonine-protein kinase WNK1
RS012065	GO:0044699	single-organism process	BP	0.388994696	-0.047014138	-0.15109159	4.208510895	4.468401141	0.215126211	0.387560818	PREDICTED: uridine 5'-monophosphate synthase
RS015353	GO:0044699	single-organism process	BP	0.674564948	0.647369511	0.362530993	6.01737716	4.466200823	0.215324983	0.387843655	PREDICTED: ubiquitin carboxyl-terminal hydrolase 37
RS006293	GO:0044699	single-organism process	BP	-0.303341476	-0.533978762	-0.369378241	4.443899898	4.45776568	0.216088571	0.38888532	PREDICTED: tRNA wybutosine-synthesizing protein 4
RS011297	GO:0044699	single-organism process	BP	-0.425649339	-0.131741867	0.376112855	9.23992399	4.454555531	0.216379826	0.389187022	PREDICTED: muscle-specific protein 20-like
RS012625	GO:0044699	single-organism process	BP	-0.296469095	-0.501683362	0.092051806	3.988116726	4.45384837	0.216444035	0.389246919	PREDICTED: rotatin
RS010528	GO:0044699	single-organism process	BP	-0.621566999	-0.212435009	-0.136067735	5.379200146	4.452810755	0.21653828	0.389312616	PREDICTED: regulator of G-protein signaling loco
RS011433	GO:0044699	single-organism process	BP	-0.117163554	0.472067962	0.209582221	5.304247486	4.450589701	0.216740144	0.389556925	PREDICTED: graves disease carrier protein-like
RS015201	GO:0044699	single-organism process	BP	0.123426566	0.337198506	-0.095887941	5.352533015	4.444357707	0.217307476	0.39029811	PREDICTED: mRNA-capping enzyme
RS015271	GO:0044699	single-organism process	BP	0.402691797	-1.007786202	-0.878562213	0.496484741	4.443683697	0.217368917	0.390352793	PREDICTED: longitudinals lacking protein, isoform G-like
RS000051	GO:0044699	single-organism process	BP	0.219533059	-0.381729979	-0.112189092	5.572527138	4.441004254	0.217613326	0.390680289	PREDICTED: cellular tumor antigen p53
RS015489	GO:0044699	single-organism process	BP	-0.347437816	-0.476837561	-0.670947437	3.589980165	4.437575151	0.217926487	0.391075261	PREDICTED: vacuolar protein sorting-associated protein 45
RS010053	GO:0044699	single-organism process	BP	-0.089230706	-0.43091418	0.03265712	6.379954566	4.427132948	0.218882682	0.392455151	PREDICTED: biogenesis of lysosome-related organelles complex 1 subunit 2
RS012509	GO:0044699	single-organism process	BP	-0.454363735	-0.4194122	-0.04422351	5.121726307	4.41974461	0.219561572	0.393225033	PREDICTED: sorting nexin-27
RS000599	GO:0044699	single-organism process	BP	-0.545322181	0.317225331	0.414175701	2.267147313	4.420272975	0.219512957	0.393225033	PREDICTED: myotubularin-related protein 14
RS009286	GO:0044699	single-organism process	BP	0.620857569	0.545318248	0.557703239	5.963331539	4.4189701	0.219632851	0.393240849	PREDICTED: GRIP and coiled-coil domain-containing protein 1
RS007608	GO:0044699	single-organism process	BP	-0.313919656	-0.221843514	-0.598786917	6.860573137	4.416671791	0.219844495	0.393527242	PREDICTED: ATP-citrate synthase
RS003530	GO:0044699	single-organism process	BP	0.552512287	0.354723827	0.48126928	4.389900012	4.416554483	0.219855302	0.393527242	PREDICTED: zinc finger protein 593 homolog
RS010017	GO:0044699	single-organism process	BP	0.509465749	0.783290973	0.186844021	3.788149078	4.410846351	0.220381783	0.394021604	PREDICTED: DNA-3-methyladenine glycosylase-like
RS009942	GO:0044699	single-organism process	BP	0.409219716	0.347978408	0.200169616	6.173031128	4.407804328	0.220662835	0.394412112	PREDICTED: rab-like protein 3
RS007131	GO:0044699	single-organism process	BP	0.416097573	0.483443439	0.316904503	4.880419797	4.406664747	0.220768205	0.394488476	PREDICTED: probable glutamate--tRNA ligase, mitochondrial
RS007759	GO:0044699	single-organism process	BP	-0.727888511	0.088802775	-0.395453856	2.340238306	4.406238604	0.22080762	0.394502932	PREDICTED: alpha-ketoglutarate-dependent dioxygenase alkB homolog 6
RS008592	GO:0044699	single-organism process	BP	-0.478319437	-0.717645594	-0.537537042	4.454749447	4.405354139	0.220889447	0.394593149	PREDICTED: myosin light chain kinase, smooth muscle-like

RS006442	GO:0044699	single-organism process	BP	-0.49190499	0.170187732	0.109297644	2.966623919	4.401421445	0.221253622	0.395075588	PREDICTED: phosphatidylinositol 3-kinase regulatory subunit alpha
RS000409	GO:0044699	single-organism process	BP	-0.556925433	-0.508529383	0.013855524	3.706596652	4.395731219	0.221781529	0.395737684	PREDICTED: inositol polyphosphate 5-phosphatase K-like
RS013567	GO:0044699	single-organism process	BP	0.452931493	0.545824856	0.697046303	3.165531843	4.392024195	0.22212607	0.395922591	Transposable element Tcb1 transposase
RS000620	GO:0044699	single-organism process	BP	0.499413378	-0.222945669	-0.506492163	1.534940773	4.387111113	0.222583466	0.396494508	PREDICTED: DEP domain-containing protein 1A
RS005156	GO:0044699	single-organism process	BP	-0.623810488	-0.249490705	-0.24287695	5.3844119	4.385809209	0.222704816	0.396542408	PREDICTED: serine/threonine-protein kinase Genghis Khan
RS000054	GO:0044699	single-organism process	BP	-0.235636075	-0.773053343	-0.434628691	4.474106447	4.385938282	0.222692782	0.396542408	putative G-protein coupled receptor 112
RS009494	GO:0044699	single-organism process	BP	-0.091763742	0.258616765	0.376342216	5.855661683	4.38183706	0.223075434	0.396888329	mTerf5
RS012032	GO:0044699	single-organism process	BP	0.075382703	-0.598762121	-0.648027479	2.214483488	4.379237748	0.223318269	0.397218311	PREDICTED: mini-chromosome maintenance complex-binding protein
RS012324	GO:0044699	single-organism process	BP	-0.639112315	-1.234689077	-0.08776879	1.305640976	4.37485531	0.22372824	0.397633848	PREDICTED: serine/threonine-protein phosphatase 6 regulatory ankyrin repeat subunit A
RS001807	GO:0044699	single-organism process	BP	-0.129068087	-0.955111529	-0.333434619	2.612993734	4.374145983	0.223794661	0.397639668	PREDICTED: histone-lysine N-methyltransferase EHMT2
RS008952	GO:0044699	single-organism process	BP	-0.404836787	-0.11441651	0.355860301	8.876459072	4.374443881	0.223766764	0.397639668	PREDICTED: cytochrome c oxidase subunit NDUFA4
RS009362	GO:0044699	single-organism process	BP	-0.151734251	-1.379851033	-0.853593219	0.577299208	4.353138426	0.225770076	0.399764452	PREDICTED: sodium- and chloride-dependent glycine transporter 2-like
RS012567	GO:0044699	single-organism process	BP	-0.01050941	0.11184936	-0.618760535	3.320616018	4.350385561	0.226030125	0.400031393	PREDICTED: sedoheptulokinase-like
RS002090	GO:0044699	single-organism process	BP	0.2011381	0.539249055	0.349907191	5.119496939	4.350497366	0.226019558	0.400031393	PREDICTED: mitochondrial ribosome-associated GTPase 2
RS002757	GO:0044699	single-organism process	BP	-0.513013105	-0.366927339	-0.051947584	8.044085888	4.347488937	0.226304051	0.400336226	mitochondrial manganese superoxide dismutase
RS010199	GO:0044699	single-organism process	BP	-0.184513202	-0.397576289	-0.473387729	5.197408164	4.337501536	0.22725088	0.401502303	PREDICTED: GPI transamidase component PIG-T
RS008031	GO:0044699	single-organism process	BP	0.179743824	0.116308483	-0.361381561	4.562200837	4.335196439	0.227469926	0.401618349	PREDICTED: estradiol 17-beta-dehydrogenase 8
RS001473	GO:0044699	single-organism process	BP	-0.132327419	-0.412257946	-0.576652187	5.421135015	4.335895644	0.227403462	0.401618349	PREDICTED: DNA mismatch repair protein Mlh3
RS002807	GO:0044699	single-organism process	BP	0.287796549	-0.082276361	-0.371894573	3.970498847	4.33487152	0.227500818	0.401618349	PREDICTED: bifunctional coenzyme A synthase
RS005426	GO:0044699	single-organism process	BP	0.304371374	0.396062737	0.395138703	6.672682358	4.329148443	0.228045572	0.402185503	PREDICTED: 5'-AMP-activated protein kinase subunit beta-2
RS011126	GO:0044699	single-organism process	BP	-0.425342683	-0.53256198	0.048696446	8.514475953	4.320223652	0.228897478	0.403292507	PREDICTED: triosephosphate isomerase
RS001539	GO:0044699	single-organism process	BP	-0.380736825	-1.0292407	-0.36481089	2.349632725	4.316399244	0.229263428	0.403767811	PREDICTED: transient receptor potential cation channel protein painless
RS012561	GO:0044699	single-organism process	BP	-0.06337451	-0.316466781	0.29051754	4.249095264	4.315883376	0.229312832	0.403798351	PREDICTED: polycomb complex protein BMI-1
RS005227	GO:0044699	single-organism process	BP	0.455821833	0.259038003	0.102179355	5.644742258	4.314230826	0.229471159	0.403821294	PREDICTED: DNA polymerase beta-like
RS008402	GO:0044699	single-organism process	BP	0.08413133	-0.311339849	-0.389541279	4.262499687	4.313408868	0.229549946	0.403877064	PREDICTED: DNA mismatch repair protein Mlh1
RS014997	GO:0044699	single-organism process	BP	-0.068392036	-0.306113841	0.205901561	4.99125296	4.308069794	0.23006232	0.404608974	PREDICTED: lethal(2)neighbour of Tid protein
RS012560	GO:0044699	single-organism process	BP	-0.041301573	-0.515464379	-0.426603809	4.255404315	4.307057209	0.230159613	0.404677505	PREDICTED: ATP-binding cassette sub-family B member 6, mitochondrial
RS001078	GO:0044699	single-organism process	BP	0.318616011	0.447688701	0.420750106	5.614321212	4.302274624	0.230619654	0.405193069	PREDICTED: fukutin
RS015158	GO:0044699	single-organism process	BP	-0.221959913	0.017696906	0.233149674	6.687630933	4.297117886	0.231116631	0.405839774	PREDICTED: calcineurin subunit B type 2
RS007168	GO:0044699	single-organism process	BP	-0.665607539	-1.37473132	-0.363737727	1.361668693	4.29592032	0.231232187	0.405986082	PREDICTED: 5-hydroxytryptamine receptor 2B
RS008230	GO:0044699	single-organism process	BP	-0.371541256	0.155315236	0.053609737	7.613849202	4.29031154	0.231774096	0.40679682	PREDICTED: protein-methionine sulfoxide oxidase Mical
RS011177	GO:0044699	single-organism process	BP	-0.147795675	-0.468213815	-0.154094949	5.086535011	4.289589226	0.23184397	0.40679682	PREDICTED: 3-ketodihydrosphingosine reductase
RS009057	GO:0044699	single-organism process	BP	0.410364117	1.199880509	1.610875782	0.294179101	4.290361138	0.231769299	0.40679682	PREDICTED: leucine-rich repeat-containing G-protein coupled receptor 4
RS008517	GO:0044699	single-organism process	BP	0.192039661	0.227550564	-0.202510499	7.07712527	4.287608369	0.232035688	0.406999645	PREDICTED: coatomer subunit delta
RS000072	GO:0044699	single-organism process	BP	0.29579404	0.088321019	-0.161135845	6.127110061	4.277508343	0.233015491	0.408490686	PREDICTED: vacuolar protein sorting-associated protein 53 homolog
RS006541	GO:0044699	single-organism process	BP	-0.648048957	-0.689832557	-0.251732785	3.138463299	4.276826437	0.233081779	0.408550024	PREDICTED: syntaxin-1A
RS005280	GO:0044699	single-organism process	BP	0.572920685	0.052379455	0.938447567	3.032392488	4.273149565	0.233439508	0.40894939	PREDICTED: acid-sensing ion channel 4
RS001911	GO:0044699	single-organism process	BP	-0.354858321	-0.511067736	-0.530111822	4.46637499	4.265443861	0.234190845	0.409944281	PREDICTED: palmitoyltransferase ZDHHC17
RS013373	GO:0044699	single-organism process	BP	0.30791963	-0.26735448	-0.049890947	4.380750886	4.255882131	0.235126238	0.411046619	PREDICTED: probable ATP-dependent RNA helicase DDX52
RS005177	GO:0044699	single-organism process	BP	-0.469793391	-0.028312976	-0.027112499	5.899236932	4.252708788	0.235437433	0.411533523	PREDICTED: methionine--tRNA ligase, cytoplasmic
RS003617	GO:0044699	single-organism process	BP	-1.062213293	-0.649703445	-0.536049715	2.007279057	4.249683399	0.23573447	0.411948805	PREDICTED: complexin
RS005705	GO:0044699	single-organism process	BP	-0.315799028	0.314162418	-0.216981043	3.349316124	4.249445874	0.235757806	0.411948805	PREDICTED: coiled-coil domain-containing protein R3HCC1L
RS003810	GO:0044699	single-organism process	BP	-0.803977268	-1.196117277	-0.166070638	1.492786124	4.242763049	0.236415215	0.412862393	PREDICTED: lutropin-choriogonadotropic hormone receptor-like
RS002935	GO:0044699	single-organism process	BP	-0.221135437	-0.844900766	-0.747380477	1.585722909	4.23509852	0.237171267	0.413702899	hypothetical protein
RS014952	GO:0044699	single-organism process	BP	-0.182386035	-0.493073257	-0.206399499	5.389916505	4.229413722	0.237733464	0.414453937	PREDICTED: GTP:AMP phosphotransferase AK3, mitochondrial
RS007791	GO:0044699	single-organism process	BP	-0.379787464	0.049869261	0.105041138	7.48004628	4.225689753	0.238102407	0.41486742	PREDICTED: V-type proton ATPase subunit E
RS012052	GO:0044699	single-organism process	BP	0.275111344	0.301401645	0.429317077	5.823951798	4.214060162	0.239257961	0.416304886	PREDICTED: CDP-diacylglycerol--glycerol-3-phosphate 3-phosphatidyltransferase, mitochondrial
RS005053	GO:0044699	single-organism process	BP	-0.615464722	-0.814503784	-0.589684708	4.951955423	4.206995467	0.239962439	0.416897083	PREDICTED: contactin
RS002653	GO:0044699	single-organism process	BP	-0.286495154	-0.544151982	-0.337620666	4.702765343	4.195534228	0.241109374	0.417967166	PREDICTED: septin-1
RS010424	GO:0044699	single-organism process	BP	-0.694975982	-0.087445348	-0.640520311	1.938820184	4.193601382	0.241303289	0.418188197	PREDICTED: glycerophosphodiester phosphodiesterase 1
RS006558	GO:0044699	single-organism process	BP	0.42066103	-0.248688123	-0.586603156	2.253369248	4.177489737	0.242925275	0.420131956	PREDICTED: uncharacterized oxidoreductase C663.09c
RS015079	GO:0044699	single-organism process	BP	-1.376295246	0.661290456	0.618943801	0.209343525	4.174647883	0.243212401	0.420568148	PREDICTED: facilitated trehalose transporter Tret1-like
RS012392	GO:0044699	single-organism process	BP	0.339871188	0.761377897	0.718083895	10.3892291	4.174332608	0.243244274	0.420568148	PREDICTED: 40S ribosomal protein S20
RS012398	GO:0044699	single-organism process	BP	-0.181909214	0.045983675	0.559515665	9.326609998	4.173573583	0.243321024	0.420643099	PREDICTED: cytochrome c oxidase subunit 5B, mitochondrial-like
RS011925	GO:0044699	single-organism process	BP	0.388435501	0.174573955	-0.032448816	4.88050701	4.171615824	0.243519088	0.420927724	mitochondrial transcription termination factor
RS003664	GO:0044699	single-organism process	BP	-0.498766635	-0.07762555	0.027433905	6.12845622	4.16486432	0.24420326	0.421877707	PREDICTED: dihydroxyacetone phosphate acyltransferase
RS012982	GO:0044699	single-organism process	BP	0.198305119	0.368962485	0.445914277	5.300185415	4.161162588	0.244579125	0.422083595	PREDICTED: ribosome-recycling factor, mitochondrial
RS007096	GO:0044699	single-organism process	BP	0.429341549	0.499225342	0.319605252	6.59354325	4.161253118	0.244569926	0.422083595	PREDICTED: guanosine-3',5'-bis(diphosphate) 3'-pyrophosphohydrolase MESH1
RS008772	GO:0044699	single-organism process	BP	0.318184031	-0.174315554	0.020873609	4.815050346	4.148979324	0.245819923	0.423741597	PREDICTED: histone-lysine N-methyltransferase pr-set7
RS001829	GO:0044699	single-organism process	BP	0.13908193	0.456910021	0.272417381	4.9206264	4.141242682	0.246610841	0.424809521	PREDICTED: trafficking protein particle complex subunit 2-like protein
RS007089	GO:0044699	single-organism process	BP	-0.283226924	-0.348037131	-0.428049003	5.400538835	4.140940004	0.246641831	0.424809521	PREDICTED: protein ECT2
RS012575	GO:0044699	single-organism process	BP	-0.349652146	-0.758075662	-0.220454604	3.97087506	4.137638051	0.246980136	0.425217749	PREDICTED: phosphatidylinositol 5-phosphate 4-kinase type-2 alpha
RS014054	GO:0044699	single-organism process	BP	-0.695685696	-0.323415064	-0.428068674	4.673415478	4.129457837	0.247820075	0.426372408	PREDICTED: rap guanine nucleotide exchange factor 2-like
RS007453	GO:0044699	single-organism process	BP	-0.313175917	-0.643873466	-0.543888051	5.046096398	4.126644489	0.24810955	0.426753848	PREDICTED: 3-hydroxyisobutyryl-CoA hydrolase, mitochondrial
RS005592	GO:0044699	single-organism process	BP	-0.408049599	-0.607376572	-0.512216376	6.303486981	4.102236304	0.250633977	0.429713316	PREDICTED: SPARC-related modular calcium-binding protein 2
RS007439	GO:0044699	single-organism process	BP	-0.177322528	-0.488733711	0.259153647	3.425894364	4.099962898	0.250870294	0.42974095	PREDICTED: major facilitator superfamily domain-containing protein 9
RS015378	GO:0044699	single-organism process	BP	-0.11464752	-0.47318258	-0.790743101	2.363076796	4.098614325	0.251010572	0.429864991	PREDICTED: probable glutamine-dependent NAD(+) synthetase
RS014056	GO:0044699	single-organism process	BP	-0.307786794	0.024183851	-0.622076043	3.844393156	4.096025189	0.251280093	0.430170067	PREDICTED: ras-GEF domain-containing family member 1B-like
RS003792	GO:0044699	single-organism process	BP	-0.051296063	0.11818854	-0.599943163	4.868518492	4.095076911	0.251378872	0.430186541	PREDICTED: guanylate cyclase 32E-like
RS009088	GO:0044699	single-organism process	BP	-0.297613061	0.064032419	0.430473257	9.578338225	4.093175996	0.251576991	0.430249948	PREDICTED: pyruvate kinase-like
RS006623	GO:0044699	single-organism process	BP	-1.922871643	-0.290114595	-0.243187504	0.180645122	4.091714317	0.251729428	0.430393757	PREDICTED: tachykinin-like peptides receptor 86C
RS005564	GO:0044699	single-organism process	BP	-0.206877879	-0.522391276	0.331053646	2.948665139	4.090061953	0.251901852	0.430571652	PREDICTED: glycerol kinase
RS014714	GO:0044699	single-organism process	BP	-0.013655297	0.542376204	0.362211526	3.317353074	4.08561393	0.25236654	0.431014945	PREDICTED: E3 ubiquitin-protein ligase SIAH1-like
RS006215	GO:0044699	single-organism process	BP	0.353967808	0.02087474	-0.504165922	7.694470755	4.081159889	0.252832639	0.431693907	PREDICTED: protein disulfide-isomerase
RS009855	GO:0044699	single-organism process	BP	0.309966628	-0.244784452	-0.16779766	5.16344605	4.075402859	0.253436254	0.432463909	PREDICTED: protein disulfide-isomerase A5
RS009341	GO:0044699	single-organism process	BP	0.151083647	0.239981822	0.690112883	8.452787692	4.07531685	0.253445282	0.432463909	PREDICTED: protein SDA1 homolog
RS010122	GO:0044699	single-organism process	BP	-0.765374966	-0.665862456	0.101094282	12.40753779	4.068317122	0.254180983	0.433408517	PREDICTED: ADP,ATP carrier protein 2
RS014037	GO:0044699	single-organism process	BP	-0.055767744	0.147297367	0.42107924	5.140895492	4.065379367	0.254490333	0.433818524	PREDICTED: mitochondrial inner membrane protein COX18
RS008779	GO:0044699	single-organism process	BP	-0.520767416	-0.062936729	-0.289743839	4.99774316	4.063022308	0.254738782	0.434123405	PREDICTED: cytospin-A-like
RS011961	GO:0044699	single-organism process	BP	-0.621707386	-0.351172328	-1.036617646	1.30141316	4.046203386	0.256518026	0.436567445	PREDICTED: MOXD1 homolog 2
RS000571	GO:0044699	single-organism process	BP	-0.309899163	-0.83125256	-0.530161449	2.485162009	4.045605819	0.25658145	0.436614887	PREDICTED: cardio acceleratory peptide 2b
RS013386	GO:0044699	single-organism process	BP	0.344498303	0.461767843	0.375892385	5.338715675	4.038523574	0.257334215	0.437600203	PREDICTED: probable proline--tRNA ligase, mitochondrial
RS012677	GO:0044699	single-organism process	BP	0.052999836	-0.267798448	-0.411399371	5.561277549	4.036103121	0.257591943	0.437920211	PREDICTED: DNA repair protein complementing XP-G cells
RS012572	GO:0044699	single-organism process	BP	-0.033530493	-0.070144158	0.420324183	6.938987919	4.033084121	0.257913732	0.438348927	PREDICTED: NADH dehydrogenase [ubiquinone] 1 alpha subcomplex subunit 6
RS009632	GO:0044699	single-organism process	BP	0.339545386	0.360953145	0.444037887	5.099058717	4.030337811	0.258206774	0.438728564	PREDICTED: tumor suppressor candidate 2
RS001071	GO:0044699	single-organism process	BP	-0.876930207	-0.229687445	-0.857127014	1.449912461	4.027091563	0.258553552	0.439140044	PREDICTED: 1-acyl-sn-glycerol-3-phosphate acyltransferase alpha
RS007422	GO:0044699	single-organism process	BP	0.502598123	0.256270296	0.296811298	5.99380803	4.025616867	0.258711224	0.439230137	PREDICTED: G kinase-anchoring protein 1-like
RS013313	GO:0044699	single-organism process	BP	-0.437353383	0.051765726	-1.02692538	1.648766752	4.024520684	0.258828484	0.439369986	PREDICTED: calcitonin gene-related peptide type 1 receptor-like
RS002496	GO:0044699	single-organism process	BP	0.300148394	0.236519543	0.47025147	6.2846586	4.023173444	0.258972665	0.439496259	PREDICTED: cation-dependent mannose-6-phosphate receptor
RS013133	GO:0044699	single-organism process	BP	0.090755825	0.303378857	0.373115699	5.975301029	4.022654923	0.259028176	0.439531238	PREDICTED: protein Mo25
RS014949	GO:0044699	single-organism process	BP	-0.322028009	0.232388952	-0.284319267	3.462216166	4.019236252	0.25939444	0.440017429	PREDICTED: UMP-CMP kinase
RS009951	GO:0044699	single-organism process	BP	-0.297383727	-0.368112525	-0.736085978	3.621798761	4.01659476	0.259677762	0.440315381	PREDICTED: protein unc-119 homolog
RS001774	GO:0044699	single-organism process	BP	-0.556967124	-0.686693785	0.064399051	2.246327006	4.014186998	0.25993626	0.440531132	PREDICTED: acetylcholine receptor subunit alpha-like
RS006416	GO:0044699	single-organism process	BP	-0.455449744	-0.785897929	-0.399409126	2.606477879	4.013898616	0.259967236	0.440531132	PREDICTED: SH2B adapter protein 1
RS001949	GO:0044699	single-organism process	BP	0.436689786	0.3106474	0.171255603	5.354182783	4.005922061	0.260825364	0.441510031	PREDICTED: nucleoside diphosphate kinase 7
RS005924	GO:0044699	single-organism process	BP	-0.297017548	-0.405613194	-0.513435526	5.234961629	4.005053562	0.260918953	0.441609098	PREDICTED: leukocyte tyrosine kinase receptor
RS000424	GO:0044699	single-organism process	BP	0.344918054	-0.041153314	-0.104963434	5.167688014	4.004430264	0.260986139	0.441663455	PREDICTED: mitotic spindle assembly checkpoint protein MAD2B
RS008854	GO:0044699	single-organism process	BP	-0.17805427	-0.516288835	-0.566251357	3.97716752	4.002335321	0.261212068	0.441867667	CASPL1
RS011633	GO:0044699	single-organism process	BP	-0.725429276	-0.391291537	-1.591427502	0.581647617	3.998167637	0.26166206	0.442450589	PREDICTED: uracil-DNA glycosylase
RS004035	GO:0044699	single-organism process	BP	-0.316720688	-0.100307737	0.281750596	3.910110528	3.996890358	0.261800111	0.442624594	PREDICTED: protein roadkill
RS006644	GO:0044699	single-organism process	BP	0.218546353	0.122029449	0.462159426	6.416700673	3.996438773	0.261848935	0.442629138	PREDICTED: FAD-dependent oxidoreductase domain-containing protein 1-like
RS002014	GO:0044699	single-organism process	BP	0.036539526	-0.332351867	0.222033102	8.334994989	3.995693418	0.261929539	0.442664335	PREDICTED: cystathionine gamma-lyase
RS013721	GO:0044699	single-organism process	BP	0.465402258	0.161671699	-0.014919029	5.889660753	3.990400825	0.262502535	0.44339551	PREDICTED: peroxiredoxin-2
RS005063	GO:0044699	single-organism process	BP	0.017944639	-0.401939594	-0.05804259	5.322551021	3.987451176	0.262822368	0.443757194	PREDICTED: S-formylglutathione hydrolase
RS004188	GO:0044699	single-organism process	BP	0.414432737	0.244282265	0.066639236	6.071930318	3.978503796	0.263794707	0.444921727	PREDICTED: cyclin-dependent kinase 7
RS000326	GO:0044699	single-organism process	BP	-0.22680592	0.003848613	0.324328015	8.109325185	3.972399064	0.264459998	0.445815046	PREDICTED: NADH-ubiquinone oxidoreductase 20 kDa subunit-like
RS010926	GO:0044699	single-organism process	BP	0.254824514	0.395156365	0.422047001	6.39620994	3.972494832	0.264449549	0.445815046	PREDICTED: short/branched chain specific acyl-CoA dehydrogenase, mitochondrial
RS014912	GO:0044699	single-organism process	BP	0.459283472	0.258927496	-0.211345316	3.514847173	3.969475205	0.264779177	0.445978195	PREDICTED: meiosis-specific with OB domain-containing protein
RS013099	GO:0044699	single-organism process	BP	-0.560764356	-0.269109274	-0.640459518	2.716772585	3.969895209	0.264733307	0.445978195	PREDICTED: CUB and sushi domain-containing protein 2
RS005765	GO:0044699	single-organism process	BP	0.260355785	0.37953603	0.346515019	6.377998666	3.969301547	0.264798145	0.445978195	PREDICTED: glucosidase 2 subunit beta
RS008065	GO:0044699	single-organism process	BP	0.473400163	-0.049869664	0.555426708	4.851567836	3.96813281	0.264925835	0.446052937	PREDICTED: protein kinase C

RS002455	GO:0044699	single-organism process	BP	-0.194242358	0.195237976	0.397980266	8.707600168	3.965482633	0.265215586	0.446421423	PREDICTED: very long-chain specific acyl-CoA dehydrogenase, mitochondrial
RS001759	GO:0044699	single-organism process	BP	-0.042942023	-0.665774245	-0.496631128	2.879134841	3.964710699	0.265300037	0.446503898	PREDICTED: RISC-loading complex subunit tarbp2-like
RS015190	GO:0044699	single-organism process	BP	-0.178822527	0.125844763	-0.676021497	2.927459761	3.960710402	0.26573807	0.446782062	PREDICTED: clavesin-2
RS004770	GO:0044699	single-organism process	BP	0.472178397	0.425856706	0.489620432	6.480104907	3.959582324	0.265861713	0.446783122	PREDICTED: TFIIH basal transcription factor complex helicase XPD subunit
RS009464	GO:0044699	single-organism process	BP	-0.502171207	-0.451204069	-0.531419295	3.621112164	3.958052407	0.266029483	0.446895467	PREDICTED: protein MCM10 homolog
RS009019	GO:0044699	single-organism process	BP	-0.374676852	-0.083964815	-0.359583241	5.442610253	3.956059206	0.266248201	0.44719179	Ras association domain-containing protein 1
RS015072	GO:0044699	single-organism process	BP	0.41054768	0.376328418	0.334473924	6.31964088	3.95241829	0.266648146	0.447636197	PREDICTED: coiled-coil domain-containing protein 124
RS008328	GO:0044699	single-organism process	BP	-0.720270979	-0.673219644	-0.913076457	2.068222114	3.949683391	0.266948925	0.447988979	PREDICTED: synaptotagmin-14-like
RS011939	GO:0044699	single-organism process	BP	-0.481911697	-0.084828234	-0.2742772	4.763648336	3.942464907	0.267744278	0.448877986	PREDICTED: atrial natriuretic peptide receptor 1-like
RS006038	GO:0044699	single-organism process	BP	-0.174265845	-0.020333193	0.31772091	7.640911117	3.934202534	0.268657282	0.45004919	PREDICTED: transmembrane GTPase Marf
RS012116	GO:0044699	single-organism process	BP	0.430475775	0.296659774	0.137983498	6.087217849	3.926618434	0.269497816	0.451097222	PREDICTED: diacylglycerol kinase epsilon
RS005199	GO:0044699	single-organism process	BP	0.225950096	0.537294963	0.468500964	8.733132264	3.925147849	0.269661074	0.451310508	PREDICTED: eukaryotic translation initiation factor 1A, X-chromosomal
RS012908	GO:0044699	single-organism process	BP	-1.031954639	-0.189011594	-0.455811654	4.06156748	3.924168721	0.269769822	0.451369504	PREDICTED: multiple inositol polyphosphate phosphatase 1-like
RS008791	GO:0044699	single-organism process	BP	0.569561428	0.339283371	0.086755342	6.518064701	3.923862465	0.269803845	0.451369504	PREDICTED: dolichyl-diphosphooligosaccharide--protein glycosyltransferase 48 kDa subunit
RS014973	GO:0044699	single-organism process	BP	0.296196044	-0.232758974	-0.117632416	4.617234243	3.920646319	0.270161371	0.451829193	PREDICTED: histone acetyltransferase KAT8-like
RS012212	GO:0044699	single-organism process	BP	1.298461473	1.241884362	0.487723216	0.415207106	3.920422645	0.270186252	0.451829193	PREDICTED: sodium channel protein 60E
RS000049	GO:0044699	single-organism process	BP	-0.167016261	-0.078808593	0.315739518	4.19490186	3.918242209	0.270428905	0.452114912	PREDICTED: protein I'm not dead yet-like
RS014090	GO:0044699	single-organism process	BP	-0.625737072	-0.444031972	-0.693341244	2.207480279	3.912693473	0.271047296	0.452668034	PREDICTED: autophagy protein 5
RS011286	GO:0044699	single-organism process	BP	-0.457520568	-0.346669627	-0.668905633	8.593001753	3.910184477	0.271327336	0.453015574	PREDICTED: moesin/ezrin/radixin homolog 1
RS015140	GO:0044699	single-organism process	BP	-0.426413598	-0.012528137	-0.217227405	5.917000686	3.905110368	0.271894479	0.453601679	PREDICTED: WD and tetratricopeptide repeats protein 1
RS006260	GO:0044699	single-organism process	BP	-0.911671778	-1.203709643	-0.508265233	0.937109996	3.898666817	0.272616232	0.454639894	PREDICTED: serine/threonine-protein kinase BRSK2
RS004111	GO:0044699	single-organism process	BP	0.320806788	0.270359069	0.505810835	5.451871069	3.897981521	0.272693095	0.454693081	PREDICTED: pleckstrin homology domain-containing family A member 3
RS003443	GO:0044699	single-organism process	BP	-0.469553175	-0.466729369	-0.575026889	6.444976583	3.896875988	0.272817133	0.454839685	PREDICTED: armadillo segment polarity protein
RS006501	GO:0044699	single-organism process	BP	0.245925198	-0.186915412	0.62414392	3.232220761	3.895097721	0.273016757	0.454998046	PREDICTED: protein scabrous-like
RS008423	GO:0044699	single-organism process	BP	-0.379048222	-0.141509895	0.240294359	8.745130768	3.887175909	0.273907646	0.455851742	PREDICTED: gamma-aminobutyric acid receptor-associated protein
RS004419	GO:0044699	single-organism process	BP	-0.39398529	0.162481593	-1.185227216	0.809494392	3.887905498	0.273825486	0.455851742	PREDICTED: transient receptor potential-gamma protein
RS005292	GO:0044699	single-organism process	BP	0.14939254	-0.391184552	-0.290812662	7.864912454	3.885986603	0.274041622	0.455975715	PREDICTED: ubiquitin-like modifier-activating enzyme 1
RS010106	GO:0044699	single-organism process	BP	0.319180641	0.179884108	-0.326863066	3.10196542	3.884554499	0.274203028	0.4561568	PREDICTED: protein regulator of cytokinesis 1
RS003691	GO:0044699	single-organism process	BP	-0.396884624	-0.571293958	0.253637659	1.856747806	3.871872414	0.275636118	0.457912129	PREDICTED: transient receptor potential cation channel subfamily A member 1
RS011003	GO:0044699	single-organism process	BP	-0.476136553	-0.825914591	-0.168758494	3.800595326	3.862674949	0.276679667	0.459091401	PREDICTED: serine/threonine-protein kinase SMG1
RS013054	GO:0044699	single-organism process	BP	0.511099579	0.508935842	0.181571968	4.890056381	3.862283345	0.276724178	0.459104769	PREDICTED: protein ARV1
RS003247	GO:0044699	single-organism process	BP	0.069914699	-0.374607603	-0.423624681	3.439245554	3.860098797	0.276972597	0.459395877	PREDICTED: cytosolic carboxypeptidase 2-like
RS012431	GO:0044699	single-organism process	BP	0.101037027	-0.018627158	-0.475372339	3.924987682	3.858618333	0.277141065	0.459554256	PREDICTED: UDP-GalNAc:beta-1,3-N-acetylgalactosaminyltransferase 2
RS004350	GO:0044699	single-organism process	BP	0.280918864	0.306909298	-0.037073186	6.781816446	3.85543914	0.27750315	0.459791431	PREDICTED: folylpolyglutamate synthase, mitochondrial
RS010418	GO:0044699	single-organism process	BP	-0.19455437	0.000394051	0.481391778	9.395600975	3.855673322	0.277476464	0.459791431	PREDICTED: cytochrome c oxidase subunit 6B1-like
RS001415	GO:0044699	single-organism process	BP	-0.744437253	-0.516131445	-0.52746823	2.101158601	3.852618087	0.277824802	0.46026382	PREDICTED: alpha-catulin
RS011747	GO:0044699	single-organism process	BP	-0.380454491	0.007681606	0.358897081	9.615965078	3.849118736	0.278224261	0.460864966	PREDICTED: ATP synthase subunit gamma, mitochondrial
RS001134	GO:0044699	single-organism process	BP	-0.472202252	-0.066367351	-0.014278242	4.816744369	3.847768291	0.278378555	0.46099928	PREDICTED: dolichyl pyrophosphate Man9GlcNAc2 alpha-1,3-glucosyltransferase
RS012217	GO:0044699	single-organism process	BP	-0.796593052	0.153868312	-0.263902197	1.491997951	3.845659113	0.278619693	0.461337945	PREDICTED: bone morphogenetic protein receptor type-2
RS001504	GO:0044699	single-organism process	BP	1.18540917	-0.006449946	1.178302281	0.613775435	3.839710707	0.279300775	0.462096147	PREDICTED: gustatory receptor for sugar taste 64f-like
RS007814	GO:0044699	single-organism process	BP	-0.022934896	0.013283399	0.724460106	4.088570218	3.831671389	0.280223647	0.463019761	PREDICTED: fringe glycosyltransferase
RS014583	GO:0044699	single-organism process	BP	-0.370492992	-0.697880158	-0.036922724	3.313261775	3.826612848	0.280805749	0.463677409	PREDICTED: vesicular integral-membrane protein VIP36
RS005452	GO:0044699	single-organism process	BP	-0.406225183	0.261839883	0.228164558	2.399361105	3.82672589	0.280792729	0.463677409	PREDICTED: palmitoyltransferase ZDHHC3
RS009227	GO:0044699	single-organism process	BP	0.112733416	0.226866135	0.435481621	5.02768326	3.822411736	0.28129001	0.464186902	PREDICTED: phosphoribosyl pyrophosphate synthase-associated protein 2
RS006073	GO:0044699	single-organism process	BP	-0.655045206	-0.531220638	-0.148121833	3.315319524	3.822723198	0.281254082	0.464186902	PREDICTED: NADH dehydrogenase [ubiquinone] 1 alpha subcomplex assembly factor 3
RS012037	GO:0044699	single-organism process	BP	0.395424512	-0.837435285	-0.608327801	0.739724273	3.818678561	0.281720964	0.464457889	PREDICTED: alpha-N-acetylgalactosaminidase
RS008383	GO:0044699	single-organism process	BP	-0.894290585	-0.445846498	-0.267768075	2.195389047	3.816496528	0.28197313	0.464751943	PREDICTED: probable muscarinic acetylcholine receptor gar-2
RS002754	GO:0044699	single-organism process	BP	0.232163177	-0.618847	-0.488073371	3.37505688	3.812514338	0.282433855	0.465389503	PREDICTED: cell division cycle-associated protein 7-like
RS007857	GO:0044699	single-organism process	BP	0.019057041	0.160899051	0.363191345	6.304461316	3.810263323	0.282694589	0.465502367	PREDICTED: xanthine dehydrogenase
RS009912	GO:0044699	single-organism process	BP	-0.007471245	-0.317003797	-0.486745701	4.012886914	3.810008462	0.282724123	0.465502367	PREDICTED: phosphatidylinositol N-acetylglucosaminyltransferase subunit P
RS013693	GO:0044699	single-organism process	BP	0.070724722	-0.379036433	0.167092826	4.279852199	3.804162069	0.283402383	0.466375227	PREDICTED: clusterin-associated protein 1
RS008249	GO:0044699	single-organism process	BP	-0.391953891	1.193441996	1.153073924	1.121001183	3.791832584	0.284837569	0.467988351	PREDICTED: glucose dehydrogenase [FAD, quinone]-like
RS000878	GO:0044699	single-organism process	BP	-0.536410077	-0.620333003	-0.480574696	3.333423954	3.791921767	0.284827164	0.467988351	PREDICTED: protein O-linked-mannose beta-1,2-N-acetylglucosaminyltransferase 1
RS013362	GO:0044699	single-organism process	BP	0.4425472	0.190205413	0.349137702	5.59674962	3.792154238	0.284800044	0.467988351	PREDICTED: transmembrane protein 223
RS004827	GO:0044699	single-organism process	BP	-0.266017746	-1.073095568	-0.488955466	3.291066059	3.790814623	0.284956354	0.468030394	PREDICTED: LIRP
RS002113	GO:0044699	single-organism process	BP	-0.310068083	-0.200145174	0.161852954	4.561355774	3.789195603	0.285145367	0.468142452	Suppressor of cytokine signaling 5
RS001681	GO:0044699	single-organism process	BP	-0.413729733	-0.679395811	-0.380973277	3.503026248	3.786679393	0.285439347	0.46836187	PREDICTED: galactosylgalactosylxylosylprotein 3-beta-glucuronosyltransferase P-like
RS006716	GO:0044699	single-organism process	BP	-0.244490549	0.153640219	-0.138699419	6.086703304	3.784813257	0.285657552	0.468431445	PREDICTED: sphingolipid delta(4)-desaturase DES1
RS006700	GO:0044699	single-organism process	BP	0.49463998	0.248253118	0.064484491	4.284883257	3.784971912	0.285638995	0.468431445	PREDICTED: methionine--tRNA ligase, mitochondrial
RS014351	GO:0044699	single-organism process	BP	0.156227108	0.422293573	0.344826693	6.658243688	3.776584112	0.286621565	0.469477085	PREDICTED: thioredoxin reductase 1, mitochondrial
RS011308	GO:0044699	single-organism process	BP	0.354601056	-0.027381385	0.181923114	6.868671023	3.772230039	0.287132809	0.470118382	PREDICTED: guanine deaminase
RS010159	GO:0044699	single-organism process	BP	-0.348706938	-0.240534596	-0.923335701	3.326116345	3.763603184	0.28814817	0.471201178	PREDICTED: delta-1-pyrroline-5-carboxylate synthase
RS005866	GO:0044699	single-organism process	BP	-0.551694843	-0.065495423	-0.351623158	3.517411343	3.763432496	0.288168292	0.471201178	PREDICTED: syntaxin-6
RS006724	GO:0044699	single-organism process	BP	1.072023102	0.112416307	0.609701895	0.789605252	3.761098346	0.288443587	0.47128419	PREDICTED: glutamate receptor ionotropic, delta-1-like
RS002977	GO:0044699	single-organism process	BP	0.118604573	0.441224603	0.037913535	4.957205925	3.761211507	0.288430235	0.47128419	PREDICTED: AP-1 complex subunit sigma-2
RS000693	GO:0044699	single-organism process	BP	-0.697277451	-0.63859341	-1.105155719	1.668516432	3.761841056	0.288355965	0.47128419	PREDICTED: solute carrier family 12 member 4
RS006159	GO:0044699	single-organism process	BP	-0.427103513	-1.091983299	-0.903838157	1.479388146	3.756667236	0.288966851	0.471771911	PREDICTED: G2/mitotic-specific cyclin-B
RS002165	GO:0044699	single-organism process	BP	-0.409757647	-0.51419189	-0.430750075	5.606018497	3.755761984	0.289073856	0.47182428	PREDICTED: iron/zinc purple acid phosphatase-like protein
RS012042	GO:0044699	single-organism process	BP	-0.192210076	-0.454192797	-0.212867275	5.055235089	3.746474687	0.29017371	0.473067663	PREDICTED: galactokinase
RS010937	GO:0044699	single-organism process	BP	-0.11149478	0.175540035	0.373137602	4.805084257	3.745187713	0.290326416	0.473155872	PREDICTED: ataxin-3-like
RS002954	GO:0044699	single-organism process	BP	-0.588664935	0.129947261	-0.44183827	3.296295807	3.738395845	0.291133501	0.474080077	PREDICTED: arrestin domain-containing protein 3
RS003190	GO:0044699	single-organism process	BP	-0.100853157	-0.242764002	0.247383351	4.936434407	3.737424627	0.291249076	0.474206964	PREDICTED: mothers against decapentaplegic homolog 3
RS013554	GO:0044699	single-organism process	BP	-0.499772988	0.023998288	0.225519408	5.709572253	3.736268552	0.291386703	0.474308406	PREDICTED: anoctamin-10
RS004593	GO:0044699	single-organism process	BP	0.262368668	-0.314833663	-0.606891149	2.686998086	3.735102132	0.29152562	0.474414272	PREDICTED: SET domain-containing protein 4
RS005441	GO:0044699	single-organism process	BP	0.262065053	-0.027964427	0.320352653	5.632316838	3.733445463	0.291723027	0.474671809	PREDICTED: tetratricopeptide repeat protein 8
RS002566	GO:0044699	single-organism process	BP	0.207478392	-0.303199333	-0.27669186	3.569058625	3.728835434	0.292272983	0.475321015	PREDICTED: deoxynucleoside kinase-like
RS004928	GO:0044699	single-organism process	BP	-0.90690334	-0.12406779	-0.215472756	1.967342897	3.728023521	0.292369936	0.475417299	PREDICTED: protein tyrosine phosphatase domain-containing protein 1-like
RS012810	GO:0044699	single-organism process	BP	0.088984599	-0.187574767	-0.570276294	3.098238898	3.726517241	0.292549883	0.47562109	PREDICTED: DNA repair protein RAD51 homolog 3
RS001789	GO:0044699	single-organism process	BP	-0.073594352	-0.136739575	-0.4213873	5.307211889	3.722647394	0.293012648	0.476216452	PREDICTED: 5' exonuclease Apollo
RS014392	GO:0044699	single-organism process	BP	-0.97591621	-0.707349255	0.232142575	0.530622375	3.721584252	0.293139895	0.476361786	PREDICTED: probable multidrug resistance-associated protein lethal(2)03659
RS001666	GO:0044699	single-organism process	BP	0.117948739	0.758402671	0.221099192	2.577675346	3.714077603	0.294039775	0.477208366	PREDICTED: homeobox protein SIX1
RS009835	GO:0044699	single-organism process	BP	-0.060454727	0.315981781	0.029106827	5.959693048	3.710913503	0.29441982	0.477702037	PREDICTED: cholinephosphotransferase 1
RS008074	GO:0044699	single-organism process	BP	0.895038239	0.848148357	0.82905262	1.501203956	3.7112003	0.294385354	0.477702037	PREDICTED: protein kinase C
RS007739	GO:0044699	single-organism process	BP	-0.795148844	-1.727750335	-0.75551632	0.47258517	3.704652962	0.295173079	0.478307997	PREDICTED: dynein heavy chain 10, axonemal
RS009113	GO:0044699	single-organism process	BP	0.334735575	0.39936985	0.20445746	6.485206118	3.703479509	0.295314459	0.47847553	PREDICTED: WD repeat-containing protein 61
RS013872	GO:0044699	single-organism process	BP	-0.427727966	0.250232996	-0.272408159	3.409604747	3.702156523	0.295473927	0.478610757	PREDICTED: manganese-transporting ATPase 13A1
RS002930	GO:0044699	single-organism process	BP	-0.451673532	-0.71437108	-0.718954078	3.306419228	3.701408177	0.295564164	0.478695356	PREDICTED: acetylcholine receptor subunit alpha-L1
RS012946	GO:0044699	single-organism process	BP	-0.297256477	-0.452772751	-0.368238054	4.68789869	3.698131714	0.295959538	0.479089257	PREDICTED: dephospho-CoA kinase domain-containing protein
RS000314	GO:0044699	single-organism process	BP	-0.241069359	-0.168114078	-0.586009631	4.362662924	3.697392619	0.296048791	0.479159473	PREDICTED: active breakpoint cluster region-related protein
RS000301	GO:0044699	single-organism process	BP	0.140861067	-0.356984937	-0.26583636	3.914176067	3.694246208	0.296429019	0.479602654	PREDICTED: transcription factor E2F4
RS007353	GO:0044699	single-organism process	BP	-0.304792096	-0.067839808	0.305784648	3.508690169	3.693919818	0.296468486	0.479604896	PREDICTED: SH3 domain-containing RING finger protein 3
RS006023	GO:0044699	single-organism process	BP	0.300521306	-0.09222393	-0.085871364	4.538846326	3.688654362	0.297105842	0.480265817	PREDICTED: general transcription factor IIH subunit 2
RS004654	GO:0044699	single-organism process	BP	0.416624429	0.212691527	0.475217673	7.129737028	3.685363202	0.297504842	0.480656437	PREDICTED: protein lethal(2)essential for life
RS013027	GO:0044699	single-organism process	BP	-0.238999734	-0.765613467	-0.573817745	1.677304247	3.684544351	0.297604188	0.480656437	PREDICTED: lutropin-choriogonadotropic hormone receptor-like
RS005072	GO:0044699	single-organism process	BP	-0.654987192	-0.4219481	-0.18940484	3.26003723	3.685352455	0.297506145	0.480656437	PREDICTED: voltage-dependent T-type calcium channel subunit alpha-1G
RS001013	GO:0044699	single-organism process	BP	0.443299861	0.21880778	0.21999306	5.122358405	3.685264876	0.29751677	0.480656437	PREDICTED: polyribonucleotide nucleotidyltransferase 1, mitochondrial
RS003919	GO:0044699	single-organism process	BP	-0.236371861	-0.488480595	-0.189569942	6.71848387	3.682891397	0.297804823	0.480716988	High affinity cGMP-specific 3',5'-cyclic phosphodiesterase 9A
RS000240	GO:0044699	single-organism process	BP	-0.600573278	-0.607829638	-0.158787656	4.031758924	3.682980404	0.297794016	0.480716988	PREDICTED: phytanoyl-CoA dioxygenase, peroxisomal
RS005931	GO:0044699	single-organism process	BP	-0.187863689	-0.557912258	-0.389758882	4.004554102	3.682204482	0.297888235	0.480765048	PREDICTED: protein artemis
RS006990	GO:0044699	single-organism process	BP	-0.310134595	-0.4355977	-0.507024072	4.194119455	3.681544826	0.297968358	0.480765048	PREDICTED: BTB/POZ domain-containing protein KCTD3
RS003923	GO:0044699	single-organism process	BP	0.390351906	0.456762952	0.318357313	5.429261663	3.675338141	0.298723169	0.481273888	PREDICTED: DNA-directed RNA polymerase II 16 kDa polypeptide
RS011274	GO:0044699	single-organism process	BP	-0.018915184	0.184236622	0.341807321	6.169763506	3.674556472	0.298818351	0.481304298	PREDICTED: 39S ribosomal protein L18, mitochondrial
RS001466	GO:0044699	single-organism process	BP	-0.508985756	-1.094149248	-0.887721815	1.104977813	3.67365661	0.298927958	0.481412918	PREDICTED: protein unc-13 homolog A-like
RS008767	GO:0044699	single-organism process	BP	-0.071185743	-0.285368576	0.183550764	4.880406744	3.670316078	0.299335164	0.481890203	PREDICTED: histone deacetylase 3
RS014832	GO:0044699	single-organism process	BP	0.199346151	0.16463845	-0.314909589	3.820826104	3.669737264	0.299405771	0.481942265	PREDICTED: protein-S-isoprenylcysteine O-methyltransferase
RS007885	GO:0044699	single-organism process	BP	0.233179417	-0.249988458	-0.051343102	4.818955098	3.668926146	0.299504741	0.48199112	PREDICTED: cell division cycle protein 16 homolog
RS006765	GO:0044699	single-organism process	BP	0.361769579	-0.165062346	0.341742359	3.839196918	3.665879365	0.299876759	0.482145782	PREDICTED: zinc finger protein 37 homolog
RS008197	GO:0044699	single-organism process	BP	-0.307158998	-0.373647818	-0.769382915	3.163133414	3.663806527	0.300130093	0.48236835	PREDICTED: lysophospholipid acyltransferase 7
RS008805	GO:0044699	single-organism process	BP	-0.126144331	-0.406779076	-0.093364642	5.860518388	3.663047097	0.300222955	0.482456028	PREDICTED: KAT8 regulatory NSL complex subunit 3
RS001914	GO:0044699	single-organism process	BP	-0.110256652	0.082475422	-0.327605066	5.647487618	3.660588721	0.300523738	0.482693018	PREDICTED: vesicle-associated membrane protein 7
RS000280	GO:0044699	single-organism process	BP	0.027606087	0.060990489	-0.378460024	4.875576289	3.658145711	0.300822907	0.483050324	PREDICTED: septin-2

RS000633	GO:0044699	single-organism process	BP	-0.245959706	-0.480650328	-0.268781586	4.706722594	3.650699393	0.301736417	0.484085151	PREDICTED: ankyrin repeat and fibronectin type-III domain-containing protein 1
RS011215	GO:0044699	single-organism process	BP	0.144642587	-0.292630102	-0.045170464	4.999931895	3.644097085	0.302548453	0.485078958	PREDICTED: nucleolin-like
RS003768	GO:0044699	single-organism process	BP	0.363512172	0.076859047	0.245878102	5.801656836	3.638753746	0.30320707	0.485798664	PREDICTED: probable ATP-dependent RNA helicase DDX28
RS010217	GO:0044699	single-organism process	BP	-0.095562697	-0.173800591	-0.490593873	4.155048772	3.638577758	0.303228784	0.485798664	PREDICTED: inhibitor of nuclear factor kappa-B kinase subunit alpha
RS000968	GO:0044699	single-organism process	BP	-0.021551596	-0.437622733	0.46889812	2.096576649	3.63634133	0.303504841	0.486179086	Tankyrase-1
RS010655	GO:0044699	single-organism process	BP	-0.016745185	0.462584396	0.634465997	3.095211793	3.634701145	0.303707443	0.486347858	CG9760
RS003147	GO:0044699	single-organism process	BP	-0.505550451	-0.027606287	-0.18120612	3.786984288	3.627252198	0.30462908	0.487532937	PREDICTED: acetylcholine receptor subunit beta-like 1
RS014617	GO:0044699	single-organism process	BP	-0.072631645	0.084385518	0.526454273	9.138635249	3.625994331	0.304784959	0.487596216	PREDICTED: putative ATP synthase subunit f, mitochondrial
RS012817	GO:0044699	single-organism process	BP	-0.287960684	-0.628111107	-0.66343646	3.635326683	3.625082508	0.304897998	0.48766642	PREDICTED: facilitated trehalose transporter Tret1-like
RS005465	GO:0044699	single-organism process	BP	-0.255589849	-0.533680261	-0.609552513	2.6851131	3.620655703	0.305447325	0.488173047	PREDICTED: lysine-specific histone demethylase 1A
RS009411	GO:0044699	single-organism process	BP	-0.181431225	0.040614699	0.430286683	4.766244138	3.618525603	0.305711965	0.488387287	PREDICTED: formin-like protein CG32138
RS008839	GO:0044699	single-organism process	BP	-0.227694172	-0.426905121	-0.461391998	6.717778568	3.618328275	0.305736491	0.488387287	RNA polymerase II largest subunit
RS002504	GO:0044699	single-organism process	BP	-0.071875925	-0.111183804	0.317337709	6.127914535	3.612731629	0.306432832	0.489189388	PREDICTED: serine/threonine-protein kinase SIK3-like
RS011307	GO:0044699	single-organism process	BP	0.41048066	0.421207639	0.448357526	7.756469586	3.611633244	0.306569659	0.489326422	PREDICTED: 26S proteasome complex subunit DSS1
RS011301	GO:0044699	single-organism process	BP	-0.798119998	-0.388058978	0.133350522	2.218059865	3.607472367	0.30708848	0.489610436	PREDICTED: ras association domain-containing protein 4
RS004348	GO:0044699	single-organism process	BP	0.458523279	0.508729278	0.520757084	6.978813474	3.600477766	0.307962398	0.490512007	PREDICTED: ankyrin repeat and SOCS box protein 3
RS012848	GO:0044699	single-organism process	BP	0.217948337	-0.831073649	-0.020401587	1.446963224	3.599306911	0.308108903	0.490675361	PREDICTED: neuromedin-U receptor 2-like
RS005374	GO:0044699	single-organism process	BP	0.156154551	0.4895025	0.635662696	3.718621443	3.593858232	0.30879149	0.491583874	PREDICTED: disintegrin and metalloproteinase domain-containing protein 10
RS014352	GO:0044699	single-organism process	BP	-0.405692259	-0.007251687	-0.394574135	4.119166488	3.592852908	0.30891758	0.491722447	PREDICTED: uncharacterized oxidoreductase C663.09c
RS006894	GO:0044699	single-organism process	BP	-0.686998359	-0.181790129	-0.16040124	3.196602102	3.587663966	0.309569114	0.492407803	PREDICTED: rho GTPase-activating protein 39
RS013044	GO:0044699	single-organism process	BP	-0.331223889	-0.146943464	-0.901803683	1.724670353	3.585091749	0.309892539	0.492651683	PREDICTED: DNA polymerase epsilon subunit 2
RS004204	GO:0044699	single-organism process	BP	0.123950547	-0.145554708	-0.274706165	5.273847584	3.585175806	0.309881965	0.492651683	PREDICTED: GATA zinc finger domain-containing protein 21
RS004874	GO:0044699	single-organism process	BP	-0.386498026	-0.557788415	-0.205318852	4.223261841	3.583563387	0.310084854	0.492770807	PREDICTED: CCR4-NOT transcription complex subunit 3
RS008730	GO:0044699	single-organism process	BP	0.014745387	0.239913504	0.337320818	6.316621512	3.582890386	0.310169572	0.492781076	PREDICTED: syntaxin-5
RS008786	GO:0044699	single-organism process	BP	0.071257145	0.088316562	-0.483739065	3.538308674	3.57613499	0.311021085	0.493760185	PREDICTED: Bardet-Biedl syndrome 1 protein
RS015381	GO:0044699	single-organism process	BP	-0.14122205	0.494615068	-0.457720223	1.269481569	3.572397731	0.311493056	0.493953414	PREDICTED: potassium voltage-gated channel subfamily KQT member 1
RS001242	GO:0044699	single-organism process	BP	-0.29273257	-0.083387009	-0.505833143	5.07480907	3.567700142	0.312087205	0.494704376	PREDICTED: 1,2-dihydroxy-3-keto-5-methylthiopentene dioxygenase
RS007547	GO:0044699	single-organism process	BP	-0.409773516	-0.482458992	-0.258852864	5.091140141	3.566625413	0.312223278	0.494795516	PREDICTED: serine/threonine-protein kinase MARK2-like
RS000861	GO:0044699	single-organism process	BP	-0.055798041	-0.372825703	-0.382585377	4.749435008	3.563119255	0.312667563	0.495125774	PREDICTED: ribosomal protein S6 kinase delta-1
RS002269	GO:0044699	single-organism process	BP	-0.261710878	-0.14856721	0.173629907	6.885308754	3.554684137	0.313738721	0.496137057	PREDICTED: succinate-semialdehyde dehydrogenase, mitochondrial
RS008522	GO:0044699	single-organism process	BP	0.134383151	0.311651473	0.388955483	6.660634256	3.55467785	0.313739521	0.496137057	PREDICTED: mitochondrial import receptor subunit TOM20 homolog
RS010557	GO:0044699	single-organism process	BP	0.009935888	-0.224548088	-0.438482038	4.714259077	3.550615019	0.314256612	0.496705293	PREDICTED: U5 small nuclear ribonucleoprotein 40 kDa protein
RS010659	GO:0044699	single-organism process	BP	-0.269715753	-0.3594563	-0.806493701	3.164694791	3.543965144	0.315104596	0.497775097	PREDICTED: COMM domain-containing protein 2
RS000739	GO:0044699	single-organism process	BP	0.259445931	-0.090346934	-0.110568136	6.500027832	3.543472178	0.315167539	0.497775097	PREDICTED: exocyst complex component 1
RS005011	GO:0044699	single-organism process	BP	0.468893027	0.29037197	0.201515764	5.543220753	3.543350008	0.31518314	0.497775097	PREDICTED: rabenosyn-5
RS014614	GO:0044699	single-organism process	BP	0.17118901	-0.326282215	-0.090631117	4.420046044	3.544195221	0.315075223	0.497775097	PREDICTED: phosphatidylinositol N-acetylglucosaminyltransferase subunit Q
RS006242	GO:0044699	single-organism process	BP	-0.424071152	-0.371038933	0.131683439	3.119144436	3.540526578	0.315543873	0.498177201	PREDICTED: intraflagellar transport protein 20 homolog
RS005467	GO:0044699	single-organism process	BP	0.676741581	0.380751093	0.512853019	2.637868892	3.538021229	0.315864272	0.498396739	PREDICTED: lysine-specific histone demethylase 1A
RS015321	GO:0044699	single-organism process	BP	-0.064263828	-0.363517178	-0.336803268	4.877826598	3.531324754	0.316722073	0.499223753	PREDICTED: probable kinetochore protein nuf2
RS008959	GO:0044699	single-organism process	BP	0.333762401	-0.243983015	0.018643418	4.262626361	3.530506551	0.316827024	0.499326685	PREDICTED: thiamin pyrophosphokinase 1
RS007856	GO:0044699	single-organism process	BP	-0.527927895	-0.44836958	-0.316928476	3.63806015	3.524541502	0.317593092	0.500131237	PREDICTED: gamma-aminobutyric acid type B receptor subunit 2
RS000158	GO:0044699	single-organism process	BP	0.100628242	0.213464071	-0.277743956	6.067227973	3.522556443	0.317848388	0.500372825	SH3 domain-binding protein 5-like
RS009725	GO:0044699	single-organism process	BP	0.2924611	0.113694369	-0.187787882	4.640487227	3.516938508	0.318571889	0.501135944	PREDICTED: ribokinase-like
RS005839	GO:0044699	single-organism process	BP	-0.135352814	-0.412362885	-0.484066483	4.174169403	3.51549575	0.318757928	0.501365973	PREDICTED: DNA-directed RNA polymerase II subunit RPB7
RS006152	GO:0044699	single-organism process	BP	-0.748986783	-0.594128336	-0.87114847	2.407340807	3.514000553	0.318950831	0.501481493	PREDICTED: neprilysin-2-like
RS014514	GO:0044699	single-organism process	BP	0.458849962	0.461206587	0.309540175	8.341540804	3.513253966	0.31904719	0.50157038	PREDICTED: trifunctional enzyme subunit alpha, mitochondrial
RS006801	GO:0044699	single-organism process	BP	0.369669196	0.224508328	-0.001681508	6.983073564	3.508843193	0.319616998	0.502340756	PREDICTED: X-ray repair cross-complementing protein 6
RS003909	GO:0044699	single-organism process	BP	-0.28403924	-0.595331771	-0.404111292	3.54996971	3.506397065	0.31993339	0.502353125	PREDICTED: twist-related protein 2
RS010149	GO:0044699	single-organism process	BP	-0.154098234	-0.466690935	-0.860655645	2.124840163	3.506315269	0.319943975	0.502353125	PREDICTED: O-glucosyltransferase rumi homolog
RS008419	GO:0044699	single-organism process	BP	-0.579270886	-0.22684126	-0.07023873	5.06982487	3.507414936	0.319801701	0.502353125	PREDICTED: chloride channel protein 2
RS009076	GO:0044699	single-organism process	BP	-0.298411511	-0.653779919	-0.744142137	2.374750456	3.498058392	0.321014028	0.503455183	PREDICTED: structural maintenance of chromosomes protein 3
RS002059	GO:0044699	single-organism process	BP	0.325315345	-0.138226136	-0.067334548	4.647825416	3.488075311	0.322312009	0.504561522	PREDICTED: DNA-directed RNA polymerase II subunit RPB3
RS002850	GO:0044699	single-organism process	BP	0.054918911	0.385582416	0.426039373	8.052818651	3.480247363	0.323333022	0.505782646	PREDICTED: protein pelota
RS014381	GO:0044699	single-organism process	BP	-0.705869133	-0.34609985	-0.428285662	3.522815445	3.475455372	0.323959457	0.506245345	PREDICTED: serine/threonine-protein kinase pelle
RS001633	GO:0044699	single-organism process	BP	0.409054643	0.118433167	0.197648052	4.998918179	3.473753873	0.324182143	0.50641645	PREDICTED: ADP-ribosylation factor-like protein 2
RS005197	GO:0044699	single-organism process	BP	-0.624767222	-1.128834355	-0.039615784	1.616773135	3.471714778	0.324449191	0.50659968	PREDICTED: hexokinase-1-like
RS009945	GO:0044699	single-organism process	BP	0.395028173	0.529875207	0.572925362	5.902550872	3.471207811	0.324515615	0.506625717	PREDICTED: deoxyribose-phosphate aldolase
RS002742	GO:0044699	single-organism process	BP	-0.253489353	-0.27680429	0.079511831	5.757116744	3.470639826	0.324590049	0.506679112	PREDICTED: catenin delta-2
RS011793	GO:0044699	single-organism process	BP	0.159121641	0.31789259	0.375071534	6.885176518	3.461918317	0.325734878	0.507914397	PREDICTED: vacuolar protein sorting-associated protein VTA1 homolog
RS012645	GO:0044699	single-organism process	BP	0.330011745	0.290246406	0.357264622	6.751888566	3.462117049	0.325708752	0.507914397	PREDICTED: derlin-2
RS009202	GO:0044699	single-organism process	BP	0.202808858	-0.0060625	-0.293576568	4.147181216	3.458067044	0.326241547	0.508260903	PREDICTED: DNA replication complex GINS protein PSF1
RS013707	GO:0044699	single-organism process	BP	-0.500388832	-0.162680031	-0.110632879	6.260634623	3.457673335	0.326293381	0.508267019	PREDICTED: phospholipid scramblase 2
RS003202	GO:0044699	single-organism process	BP	0.017348138	0.376526889	0.008547262	4.505003572	3.45732396	0.326339386	0.508275813	PREDICTED: dynactin subunit 6
RS007890	GO:0044699	single-organism process	BP	-0.696206211	0.347963331	0.009276738	1.666115317	3.448521467	0.327500344	0.509328147	PREDICTED: ADP-ribosylation factor-like protein 4C
RS001689	GO:0044699	single-organism process	BP	-0.126908424	0.123316768	0.352676594	8.118770546	3.448072313	0.32755968	0.509357527	PREDICTED: 5'-AMP-activated protein kinase catalytic subunit alpha-2
RS005558	GO:0044699	single-organism process	BP	0.219671473	-0.292074721	-0.325633645	3.310500659	3.439939223	0.328635752	0.510276886	PREDICTED: kinesin-like protein KIF14
RS013677	GO:0044699	single-organism process	BP	0.034211804	0.280550659	0.595537596	9.222031987	3.437461301	0.328964218	0.510658864	PREDICTED: cytochrome c oxidase subunit 6C
RS000067	GO:0044699	single-organism process	BP	0.438230689	0.237825515	0.064775335	4.242332687	3.426782753	0.330383041	0.512166914	PREDICTED: cell cycle checkpoint control protein RAD9A
RS002729	GO:0044699	single-organism process	BP	0.044674654	0.345192364	0.263948476	5.488708832	3.423687042	0.330795361	0.512553738	PREDICTED: S-adenosylmethionine mitochondrial carrier protein-like
RS006660	GO:0044699	single-organism process	BP	0.235499911	0.419615542	0.129667593	5.023551202	3.420676164	0.331196817	0.512986436	PREDICTED: golgin subfamily A member 1
RS009393	GO:0044699	single-organism process	BP	-0.046854497	0.27112668	0.560417085	3.082873219	3.417764947	0.331585391	0.513209588	PREDICTED: general transcription factor IIH subunit 1
RS007431	GO:0044699	single-organism process	BP	0.339936526	0.102928169	0.53827734	7.570432343	3.409547472	0.332684378	0.514467959	PREDICTED: cell division cycle and apoptosis regulator protein 1-like
RS009119	GO:0044699	single-organism process	BP	-0.633398641	0.027582619	-0.254763808	2.374824839	3.400802845	0.333857375	0.51571198	PREDICTED: lateral signaling target protein 2 homolog
RS010718	GO:0044699	single-organism process	BP	0.305301299	0.000108971	-0.170457454	4.847912933	3.396379497	0.334452099	0.516377312	PREDICTED: pyrroline-5-carboxylate reductase 2
RS006561	GO:0044699	single-organism process	BP	0.353995304	0.237572917	0.419480704	5.635873753	3.395244005	0.334604917	0.516387054	PREDICTED: EH domain-containing protein 3
RS000867	GO:0044699	single-organism process	BP	6.18244E-05	0.256523791	0.336462055	5.338612801	3.391260764	0.335141479	0.517064425	PREDICTED: electron transfer flavoprotein subunit beta
RS012226	GO:0044699	single-organism process	BP	-0.205500751	-0.508001907	-0.511476983	4.612114998	3.388598173	0.335500563	0.517298492	PREDICTED: serine/threonine-protein kinase PAK mbt
RS011766	GO:0044699	single-organism process	BP	-0.075763637	-0.513683733	-0.864812575	2.04257582	3.383880843	0.336137582	0.517900258	PREDICTED: protein Wnt-16
RS010881	GO:0044699	single-organism process	BP	-0.388739446	-0.194274119	0.054595471	4.906511942	3.383536546	0.336184117	0.517908595	PREDICTED: triple functional domain protein
RS015110	GO:0044699	single-organism process	BP	-0.075114035	0.056180732	0.466052101	5.236733039	3.378954547	0.33680395	0.518546327	PREDICTED: dehydrogenase/reductase SDR family member 4
RS006577	GO:0044699	single-organism process	BP	-0.11277365	-0.172635618	0.398797935	3.46652672	3.372722797	0.337648562	0.519529137	PREDICTED: regulator of G-protein signaling 7
RS000799	GO:0044699	single-organism process	BP	-0.865915011	-0.703100612	-0.200350079	1.588681696	3.368404404	0.338234936	0.519923201	PREDICTED: peroxisomal N(1)-acetyl-spermine/spermidine oxidase
RS003017	GO:0044699	single-organism process	BP	-1.452388745	-1.397193115	-1.124449019	0.856106819	3.362332122	0.33906097	0.520811545	PREDICTED: probable cytochrome P450 6a13
RS001164	GO:0044699	single-organism process	BP	0.445628494	0.248131241	0.141768408	6.910318189	3.359263499	0.339479077	0.521199499	PREDICTED: RNA-binding protein 8A
RS004489	GO:0044699	single-organism process	BP	-0.49287906	-0.290129923	-0.514718357	3.478984221	3.356184306	0.339899076	0.521573591	PREDICTED: 1-phosphatidylinositol 4,5-bisphosphate phosphodiesterase gamma-1
RS015453	GO:0044699	single-organism process	BP	-0.09263959	0.415348464	0.277714417	5.686672294	3.355959113	0.33992981	0.521573591	PREDICTED: sodium-dependent phosphate transporter 2
RS011863	GO:0044699	single-organism process	BP	0.321832165	0.16282681	-0.112218819	5.645169918	3.354057141	0.340189485	0.521596292	PREDICTED: nibrin
RS003241	GO:0044699	single-organism process	BP	-0.11427916	-0.337421798	-0.306523126	6.067048434	3.354240435	0.340164453	0.521596292	PREDICTED: aldehyde dehydrogenase family 16 member A1
RS012694	GO:0044699	single-organism process	BP	-0.02754663	-0.226841758	-0.47714326	4.025670054	3.355285162	0.340021805	0.521596292	PREDICTED: probable G-protein coupled receptor Mth-like 5
RS010621	GO:0044699	single-organism process	BP	0.319373764	0.387129475	0.372445429	7.228363378	3.353578394	0.340254876	0.521596292	PREDICTED: probable medium-chain specific acyl-CoA dehydrogenase, mitochondrial
RS001866	GO:0044699	single-organism process	BP	0.040123917	-0.623017482	-0.214094236	5.514447489	3.354957946	0.340066477	0.521596292	PREDICTED: cadherin-related tumor suppressor
RS013385	GO:0044699	single-organism process	BP	0.215351397	0.365963937	0.527615652	7.926892912	3.35273376	0.340370268	0.521613915	PREDICTED: translation initiation factor eIF-2B subunit beta
RS003557	GO:0044699	single-organism process	BP	0.363950058	0.502299661	0.556587335	3.173988838	3.34627251	0.341254124	0.522459815	PREDICTED: nuclear receptor subfamily 2 group C member 2
RS010925	GO:0044699	single-organism process	BP	0.558114996	0.463617513	0.367707791	6.895613326	3.343813411	0.341591039	0.522784973	PREDICTED: protein O-mannose kinase
RS002744	GO:0044699	single-organism process	BP	-0.056964262	0.592194235	0.470764603	2.406605109	3.343188582	0.341676691	0.522788999	PREDICTED: serine/threonine-protein kinase pim-3
RS010016	GO:0044699	single-organism process	BP	0.327183332	0.140688951	-0.730471087	1.033959693	3.341064119	0.341968056	0.522917156	PREDICTED: zinc finger protein 271-like
RS014217	GO:0044699	single-organism process	BP	0.049300671	-0.330628229	0.015177052	6.939810457	3.341764506	0.341871976	0.522917156	PREDICTED: inorganic pyrophosphatase
RS003337	GO:0044699	single-organism process	BP	0.355486434	0.282271733	0.022330894	4.881633894	3.336115149	0.342647637	0.523765546	PREDICTED: centromere protein C
RS003939	GO:0044699	single-organism process	BP	0.199968947	-0.031637855	-0.234477887	5.351704009	3.333286984	0.343036523	0.524296355	meiotic recombination 11
RS011499	GO:0044699	single-organism process	BP	0.219446926	0.142215963	-0.270678974	4.576594625	3.332692581	0.343118305	0.524357715	PREDICTED: Bardet-Biedl syndrome 2 protein
RS005894	GO:0044699	single-organism process	BP	0.128483759	-0.263173817	-0.218260239	7.883484878	3.330941129	0.343359381	0.524361855	PREDICTED: pantothenate kinase 3
RS011074	GO:0044699	single-organism process	BP	0.053760579	-0.248033721	0.174401823	6.088249935	3.330728667	0.343388635	0.524361855	PREDICTED: PAX3- and PAX7-binding protein 1
RS012473	GO:0044699	single-organism process	BP	0.219440918	-0.17327645	-0.012648584	5.480329156	3.330724461	0.343389214	0.524361855	PREDICTED: phosphomevalonate kinase
RS008482	GO:0044699	single-organism process	BP	0.456620351	0.231742272	0.664992756	4.899508409	3.329273867	0.343589006	0.524567819	PREDICTED: organic cation transporter protein
RS009125	GO:0044699	single-organism process	BP	-0.499166098	-0.260741424	-0.718448365	2.806103389	3.328550691	0.343688647	0.524656343	PREDICTED: isoprenoid synthase domain-containing protein
RS015137	GO:0044699	single-organism process	BP	0.34283683	0.528993292	0.15762418	3.868031538	3.324536024	0.344242261	0.525354807	PREDICTED: oxygen-dependent coproporphyrinogen-III oxidase
RS003949	GO:0044699	single-organism process	BP	-0.598521789	-0.743311449	-0.73665729	2.167226911	3.323325607	0.344409327	0.525355013	PREDICTED: cadherin EGF LAG seven-pass G-type receptor 1-like
RS009016	GO:0044699	single-organism process	BP	-0.358127247	-0.324658837	-0.114733336	6.411062696	3.322511298	0.344521761	0.525355013	PREDICTED: rho GTPase-activating protein 190
RS010623	GO:0044699	single-organism process	BP	0.371350039	-0.115137788	-0.057468537	4.775466483	3.318024509	0.345141837	0.525982127	PREDICTED: serine/threonine-protein kinase 3
RS000562	GO:0044699	single-organism process	BP	0.212427527	-0.134542831	-0.23850818	4.591882481	3.316530041	0.345348589	0.526054964	PREDICTED: Bardet-Biedl syndrome 7 protein
RS009782	GO:0044699	single-organism process	BP	0.446042183	0.196556863	0.143386096	5.302965272	3.313692918	0.345741388	0.526386248	PREDICTED: histone acetyltransferase Tip60

RS012619	GO:0044699	single-organism process	BP	-0.001912662	-0.215315901	-0.586382377	3.697107794	3.309289444	0.346351818	0.526944563	PREDICTED: methylcytosine dioxygenase TET2-like
RS006605	GO:0044699	single-organism process	BP	-0.065709099	-0.47669722	-0.100819036	4.593971479	3.308331991	0.346484669	0.526944563	PREDICTED: zinc finger protein 271-like
RS008644	GO:0044699	single-organism process	BP	-0.796257461	-0.687818746	-0.5824022	2.146115836	3.308618795	0.346444869	0.526944563	PREDICTED: synapsin
RS007207	GO:0044699	single-organism process	BP	-0.249910057	0.062141868	0.149417087	5.652494964	3.309208427	0.346363058	0.526944563	PREDICTED: DALR anticodon-binding domain-containing protein 3
RS005218	GO:0044699	single-organism process	BP	0.242330565	-0.145861453	-0.071995416	6.091152187	3.306778099	0.346700373	0.527145298	PREDICTED: splicing factor 3A subunit 3
RS012548	GO:0044699	single-organism process	BP	0.298419466	0.393153997	0.299749674	6.430122129	3.30282543	0.347249591	0.527661842	PREDICTED: condensin complex subunit 2
RS000775	GO:0044699	single-organism process	BP	0.833279028	0.566215982	0.85702564	2.313556006	3.298690339	0.347824967	0.528153802	PREDICTED: uncharacterized MFS-type transporter C09D4.1
RS014765	GO:0044699	single-organism process	BP	0.405799525	-0.236424421	-0.827160936	1.162763803	3.298867386	0.347800315	0.528153802	PREDICTED: HORMA domain-containing protein 1-like
RS007871	GO:0044699	single-organism process	BP	-0.031905381	0.017165893	0.608341948	2.0955426	3.294591499	0.348396117	0.528882752	leucine-rich transmembrane protein
RS008880	GO:0044699	single-organism process	BP	0.14769946	0.553054828	0.336408558	3.369176491	3.28851771	0.349243966	0.529733651	PREDICTED: pickpocket protein 19-like
RS004159	GO:0044699	single-organism process	BP	-0.238470338	-0.545850828	-0.707838928	3.832246777	3.288118548	0.349299748	0.529754459	PREDICTED: queuine tRNA-ribosyltransferase subunit QTRTD1 homolog
RS009580	GO:0044699	single-organism process	BP	-0.333340287	-0.479408474	-0.582222382	3.302563198	3.277032573	0.350852097	0.531532696	PREDICTED: ceramide-1-phosphate transfer protein
RS002545	GO:0044699	single-organism process	BP	-0.486437272	-0.387085875	0.043929873	9.369782347	3.274374469	0.351225196	0.531905979	PREDICTED: NADH-ubiquinone oxidoreductase 49 kDa subunit
RS013743	GO:0044699	single-organism process	BP	0.561665254	-0.245927413	-0.110092498	2.165573591	3.271543924	0.351622878	0.532124313	PREDICTED: protein pangolin, isoforms A/H/I/S
RS009458	GO:0044699	single-organism process	BP	0.629613425	0.709306495	0.406933765	2.907642854	3.270452692	0.351776297	0.532292525	PREDICTED: phosphoglucomutase
RS005895	GO:0044699	single-organism process	BP	0.35152058	0.235243354	0.402810814	4.743748069	3.268563023	0.352042108	0.532566764	PREDICTED: charged multivesicular body protein 7
RS009407	GO:0044699	single-organism process	BP	-0.055373153	-0.327300962	-0.377162054	4.676573478	3.267656302	0.352169714	0.532695818	PREDICTED: ras guanyl-releasing protein 3
RS015098	GO:0044699	single-organism process	BP	0.322561978	0.43400492	0.278910192	5.526390339	3.259010345	0.353388507	0.534090347	PREDICTED: alkylated DNA repair protein alkB homolog 1
RS012085	GO:0044699	single-organism process	BP	0.198359697	0.187020438	0.486788396	5.612109249	3.252913508	0.354250158	0.534808818	PREDICTED: L-xylulose reductase
RS010805	GO:0044699	single-organism process	BP	-0.452936408	0.121429711	-0.302941434	3.598720367	3.250889077	0.354536669	0.535119224	PREDICTED: ras GTPase-activating protein 3
RS013255	GO:0044699	single-organism process	BP	-1.459435183	-0.987305084	-0.89736578	0.172081245	3.24840536	0.354888454	0.53552183	PREDICTED: alpha-2A adrenergic receptor
RS002950	GO:0044699	single-organism process	BP	0.051940014	0.251144097	-0.140848098	6.508547037	3.237096775	0.356493993	0.537429417	PREDICTED: GPI mannosyltransferase 2
RS000631	GO:0044699	single-organism process	BP	0.055217091	-0.475322053	-0.565131935	2.008427122	3.236628279	0.356560644	0.537465559	PREDICTED: dual specificity protein phosphatase 12-like
RS001341	GO:0044699	single-organism process	BP	-0.6048269	-1.642468152	-0.431664777	0.247813466	3.229275082	0.357608157	0.538786592	PREDICTED: sodium/potassium/calcium exchanger 4-like
RS011579	GO:0044699	single-organism process	BP	0.235438118	0.422326103	0.263749824	5.358762783	3.227710905	0.357831328	0.538894287	PREDICTED: quinone oxidoreductase-like protein 1
RS010779	GO:0044699	single-organism process	BP	0.212439189	-0.035024314	-0.595729899	2.244190775	3.227818371	0.357815991	0.538894287	PREDICTED: histone deacetylase 8
RS011629	GO:0044699	single-organism process	BP	0.210972852	-0.170607966	-0.214366493	4.538422472	3.224718253	0.358258644	0.539342221	G-protein coupled receptor Mth2
RS002195	GO:0044699	single-organism process	BP	0.022226137	0.401047718	-0.653344262	1.681549745	3.224591722	0.358276721	0.539342221	PREDICTED: E3 ubiquitin-protein ligase TRIM9
RS000260	GO:0044699	single-organism process	BP	-0.372718301	-0.338894098	-0.272046466	6.198010954	3.213472528	0.359868351	0.541220685	PREDICTED: X-box-binding protein 1
RS000373	GO:0044699	single-organism process	BP	-0.064635806	-0.443417447	-0.538534356	2.605903843	3.21103807	0.36021764	0.541551983	PREDICTED: alpha-ketoglutarate-dependent dioxygenase alkB homolog 2
RS015449	GO:0044699	single-organism process	BP	0.241533289	-0.182689412	0.023986423	5.25590239	3.209399612	0.360452886	0.541776304	PREDICTED: peroxisome biogenesis factor 10
RS011450	GO:0044699	single-organism process	BP	-0.483723311	-0.287583959	0.17586215	2.345735713	3.205016344	0.361082879	0.542552185	PREDICTED: phosphatidylinositol 3,4,5-trisphosphate 3-phosphatase and dual-specificity protein phosphatase PTEN
RS005200	GO:0044699	single-organism process	BP	0.209681203	-0.087757717	-0.134216095	6.003210529	3.203728765	0.361268119	0.542677801	PREDICTED: cell division cycle 5-like protein
RS012442	GO:0044699	single-organism process	BP	0.514798175	0.097974639	0.213608993	4.776141243	3.204017959	0.361226506	0.542677801	PREDICTED: beta-galactosidase-1-like protein 2
RS006492	GO:0044699	single-organism process	BP	-1.67132853	-0.607302412	-1.11579327	0.298965767	3.20278412	0.361404074	0.542817282	PREDICTED: E3 ubiquitin-protein ligase Siah2
RS012716	GO:0044699	single-organism process	BP	-0.051044967	-0.320757452	0.121612058	7.402757929	3.199133315	0.361929921	0.543477456	PREDICTED: protein-L-isoaspartate O-methyltransferase domain-containing protein 1-like
RS000922	GO:0044699	single-organism process	BP	-0.314267249	-0.636681757	-0.28029371	8.107288492	3.198808416	0.36197675	0.543482975	PREDICTED: insulin-like receptor
RS014023	GO:0044699	single-organism process	BP	0.353927952	0.192699647	0.391595026	6.342763502	3.197833502	0.3621173	0.543506784	PREDICTED: N-acetyltransferase 10
RS009382	GO:0044699	single-organism process	BP	0.235396002	0.394473241	0.371943487	4.670508831	3.198210791	0.362062902	0.543506784	PREDICTED: sorting nexin-16
RS003035	GO:0044699	single-organism process	BP	0.29010656	0.167235009	-0.235171176	4.062927787	3.195251918	0.362489706	0.5436339	PREDICTED: TBC1 domain family member 31
RS014882	GO:0044699	single-organism process	BP	-0.797340425	-0.942761802	-0.17161927	0.865056139	3.191662338	0.363008069	0.544172311	PREDICTED: inhibin beta chain
RS007033	GO:0044699	single-organism process	BP	0.270223086	0.401266333	0.04279188	7.039693445	3.191435871	0.363040793	0.544172311	hypothetical protein
RS014329	GO:0044699	single-organism process	BP	0.400769299	-0.282149171	-0.119652405	3.115811912	3.18180945	0.364434188	0.545860803	PREDICTED: ras-related protein Rab-34
RS007020	GO:0044699	single-organism process	BP	-0.349966267	-1.365217591	-1.075173917	0.979069608	3.18007576	0.364685623	0.545987962	PREDICTED: breast cancer type 1 susceptibility protein homolog
RS005978	GO:0044699	single-organism process	BP	0.413928876	0.207487647	0.17759189	6.185014054	3.180127385	0.364678134	0.545987962	PREDICTED: eukaryotic translation initiation factor 2-alpha kinase 1
RS005362	GO:0044699	single-organism process	BP	-0.152031174	0.225071251	-0.176971093	5.583015541	3.178651218	0.364892335	0.546232506	PREDICTED: orcokinin peptides type A-like
RS013145	GO:0044699	single-organism process	BP	-0.37845992	-0.293629087	-0.461905316	4.08174033	3.170920199	0.366015926	0.547393964	PREDICTED: BTB/POZ domain-containing protein KCTD9
RS014840	GO:0044699	single-organism process	BP	-0.175383987	-0.325833617	-0.325229019	5.697533573	3.167201054	0.36655751	0.547897912	PREDICTED: 1-phosphatidylinositol 4,5-bisphosphate phosphodiesterase-like
RS014852	GO:0044699	single-organism process	BP	0.33891669	0.310861208	0.06051804	6.448661632	3.166654746	0.366637121	0.547932587	PREDICTED: putative ATP-dependent RNA helicase TDRD12
RS009732	GO:0044699	single-organism process	BP	0.298605963	0.037089023	-0.235488835	3.970429665	3.166281931	0.366691459	0.54794877	PREDICTED: protein TIPIN homolog
RS013434	GO:0044699	single-organism process	BP	-0.010399702	0.373315152	-0.444905959	5.140528188	3.165001383	0.366878151	0.548126247	hypothetical protein
RS006996	GO:0044699	single-organism process	BP	0.252915482	0.158669624	-0.134625355	5.142962692	3.162956281	0.367176478	0.548219099	PREDICTED: phosphatidylinositol-glycan biosynthesis class X protein
RS008726	GO:0044699	single-organism process	BP	-0.239198332	0.278689624	0.146315272	4.384525019	3.151643572	0.368830479	0.550035649	PREDICTED: equilibrative nucleoside transporter 3
RS011469	GO:0044699	single-organism process	BP	0.151818118	0.193552771	-0.286378592	4.361178005	3.150262741	0.369032805	0.550207073	PREDICTED: solute carrier family 35 member F6
RS005874	GO:0044699	single-organism process	BP	0.356190643	0.298141688	0.106337447	6.648518844	3.145329906	0.369756365	0.550764242	PREDICTED: importin subunit alpha-3
RS008565	GO:0044699	single-organism process	BP	0.20838658	-0.120639906	0.327030442	5.996896306	3.1405613	0.370456994	0.551090949	PREDICTED: nucleolar MIF4G domain-containing protein 1
RS013366	GO:0044699	single-organism process	BP	0.196726148	0.191605852	0.436578687	5.924766775	3.137498132	0.370907652	0.551469208	PREDICTED: peptide methionine sulfoxide reductase
RS010211	GO:0044699	single-organism process	BP	1.071391991	0.905933246	0.557883471	0.690909896	3.137344567	0.370930257	0.551469208	PREDICTED: frequenin-1
RS004237	GO:0044699	single-organism process	BP	0.295118164	0.456384044	0.284810202	7.976036798	3.13763398	0.370887656	0.551469208	PREDICTED: eukaryotic initiation factor 4A-III
RS013467	GO:0044699	single-organism process	BP	0.412853916	0.254135471	0.26313331	5.045845788	3.126817797	0.372482641	0.552939062	PREDICTED: nuclear cap-binding protein subunit 2
RS003163	GO:0044699	single-organism process	BP	-0.037442444	-0.26394933	0.589685811	1.945902825	3.124345212	0.37284808	0.553210264	PREDICTED: adenylate cyclase type 2
RS001275	GO:0044699	single-organism process	BP	-0.70266797	-1.181757347	-0.365027789	0.749154187	3.121510919	0.373267357	0.553441146	PREDICTED: ras-related protein RabJ
RS012286	GO:0044699	single-organism process	BP	0.002948276	-0.532373512	-0.387966955	2.890872337	3.120946978	0.373350829	0.553499745	PREDICTED: rab-like protein 2B
RS012606	GO:0044699	single-organism process	BP	0.28216868	0.411654103	0.321220882	4.518084361	3.119652511	0.37354249	0.553718705	PREDICTED: thioredoxin-like protein 4A
RS012661	GO:0044699	single-organism process	BP	0.473122938	0.375093299	0.193663626	5.395833491	3.116471522	0.374013832	0.554260429	PREDICTED: SPRY domain-containing SOCS box protein 3-like
RS008180	GO:0044699	single-organism process	BP	-0.502662562	-0.240208011	-0.273240204	4.094017652	3.115494384	0.374158721	0.554305956	PREDICTED: intraflagellar transport protein 140 homolog
RS008888	GO:0044699	single-organism process	BP	-0.294502555	-0.567523121	-0.206094758	5.180319559	3.112412951	0.374615949	0.554657096	PREDICTED: facilitated trehalose transporter Tret1-2 homolog
RS009422	GO:0044699	single-organism process	BP	-0.285243328	-0.114572361	-0.482333904	4.81030392	3.110008243	0.374973095	0.555055379	PREDICTED: peptidylglycine alpha-hydroxylating monooxygenase
RS014625	GO:0044699	single-organism process	BP	0.707872264	-0.337541701	-0.642304502	0.776519168	3.105701231	0.3756135	0.555676779	PREDICTED: sonic hedgehog protein A
RS004220	GO:0044699	single-organism process	BP	0.210757719	-0.2013515	0.052038459	5.129953712	3.089349868	0.378053291	0.558163198	PREDICTED: F-box/LRR-repeat protein 15
RS014963	GO:0044699	single-organism process	BP	0.448226608	0.466746786	0.508611245	6.710153007	3.088795217	0.378136288	0.558163198	PREDICTED: cell cycle checkpoint protein RAD17
RS009053	GO:0044699	single-organism process	BP	-0.088550678	0.553640015	1.127275643	0.51309617	3.086083713	0.378542255	0.55856604	GD19989
RS009894	GO:0044699	single-organism process	BP	0.039716983	0.28446316	0.457933808	8.000714796	3.079939673	0.379463519	0.559610507	PREDICTED: dynamin-like 120 kDa protein, mitochondrial
RS011891	GO:0044699	single-organism process	BP	0.166598582	-0.184444429	-0.411265441	3.861165815	3.078602873	0.379664218	0.559821156	PREDICTED: alpha-L-iduronidase
RS002320	GO:0044699	single-organism process	BP	0.438166269	0.445303386	0.458626323	4.1628893	3.073343634	0.380454687	0.560337419	PREDICTED: Krueppel-like factor 5
RS015048	GO:0044699	single-organism process	BP	-0.740243311	-0.374333487	-0.470195995	10.45326798	3.0714738	0.380736064	0.560358769	PREDICTED: microtubule-actin cross-linking factor 1
RS001303	GO:0044699	single-organism process	BP	0.024239257	-0.336651953	-0.152400984	6.676481854	3.07225571	0.380618379	0.560358769	PREDICTED: proto-oncogene tyrosine-protein kinase ROS
RS012682	GO:0044699	single-organism process	BP	-0.537981604	-1.499248749	-0.927951299	0.552006568	3.070157864	0.380934195	0.560519365	PREDICTED: DNA polymerase nu-like
RS004958	GO:0044699	single-organism process	BP	0.227373234	0.474424462	0.122806485	5.328517003	3.065717212	0.381603441	0.56096812	PRDM9
RS007473	GO:0044699	single-organism process	BP	0.209454242	-0.283393779	0.012406391	4.174919671	3.064690556	0.38175831	0.561010986	PREDICTED: transmembrane protein 138
RS006542	GO:0044699	single-organism process	BP	0.328869281	0.186312148	-0.175041812	8.579084039	3.063606839	0.381921844	0.561176443	PREDICTED: GTP-binding nuclear protein Ran
RS003956	GO:0044699	single-organism process	BP	-0.323676554	0.116745023	-0.07691896	6.422899031	3.056887659	0.382937112	0.562262205	PREDICTED: solute carrier family 2, facilitated glucose transporter member 1-like
RS003609	GO:0044699	single-organism process	BP	-0.051627285	0.355758402	0.153478437	5.36998255	3.050524042	0.383900772	0.56337058	PREDICTED: zinc transporter ZIP1
RS014020	GO:0044699	single-organism process	BP	0.235013967	-0.158275896	0.007675978	6.76906365	3.04779978	0.384313945	0.56369083	PREDICTED: phosphoribosylformylglycinamidine synthase
RS002091	GO:0044699	single-organism process	BP	-0.439707875	-0.065831858	-0.115185791	5.6709785	3.047162437	0.384410662	0.563724718	PREDICTED: alanine--tRNA ligase, cytoplasmic
RS012380	GO:0044699	single-organism process	BP	-0.351152527	-0.007544774	0.171487215	4.800505707	3.044675949	0.384788184	0.563955189	PREDICTED: GMP reductase 1-like
RS008421	GO:0044699	single-organism process	BP	-0.407699918	-0.309603982	-0.266011294	5.56065966	3.045367269	0.38468319	0.563955189	PREDICTED: vesicle-associated membrane protein 2
RS013590	GO:0044699	single-organism process	BP	0.916624006	0.203331243	0.594896403	1.144463385	3.04326547	0.385002477	0.564083172	PREDICTED: zinc finger and SCAN domain-containing protein 12
RS005578	GO:0044699	single-organism process	BP	0.320826952	0.071363043	-0.057340171	5.536137514	3.038481115	0.385730115	0.565001752	PREDICTED: GPI inositol-deacylase
RS004046	GO:0044699	single-organism process	BP	0.380079947	0.285827761	0.419501086	4.864041297	3.037253475	0.385917011	0.565209773	PREDICTED: sperm-associated antigen 1
RS006067	GO:0044699	single-organism process	BP	-0.504575739	-0.251744329	-0.278379685	3.977652098	3.03568145	0.386156449	0.565428941	PREDICTED: synaptotagmin-7
RS011819	GO:0044699	single-organism process	BP	-0.564377143	-0.061987604	-0.810820848	3.212596367	3.033391379	0.38650548	0.565808441	PREDICTED: lachesin
RS013077	GO:0044699	single-organism process	BP	0.320745853	-0.047417166	-0.242885267	3.632774111	3.029654065	0.387075662	0.566379794	PREDICTED: phosphatidylinositol N-acetylglucosaminyltransferase subunit H
RS002264	GO:0044699	single-organism process	BP	-0.256910292	-0.386576341	-0.233202222	5.959043483	3.02437981	0.387881542	0.567097764	PREDICTED: sodium-dependent glucose transporter 1
RS002179	GO:0044699	single-organism process	BP	-0.05600211	-1.565631247	-0.488033424	0.578890362	3.02525752	0.387747333	0.567097764	PREDICTED: paired box protein Pax-6
RS007151	GO:0044699	single-organism process	BP	-0.208884581	-0.529618051	-0.401679863	4.143839607	3.022784144	0.388125632	0.567257074	PREDICTED: E3 ubiquitin-protein ligase RNF169-like
RS014629	GO:0044699	single-organism process	BP	-0.214678935	-0.383760696	-0.386614036	4.518097369	3.022169962	0.388219619	0.567272978	PREDICTED: arf-GAP with coiled-coil, ANK repeat and PH domain-containing protein 2
RS009930	GO:0044699	single-organism process	BP	-0.164680453	-0.22422223	-0.458745434	4.39174756	3.013997337	0.389472093	0.568499251	PREDICTED: vacuolar protein sorting-associated protein 28 homolog
RS004751	GO:0044699	single-organism process	BP	-0.742938454	-0.316630208	-1.110114663	0.942724402	3.013484825	0.389550751	0.56854817	PREDICTED: fibroblast growth factor 1-like
RS014385	GO:0044699	single-organism process	BP	0.324478996	0.165168981	0.43192841	4.234877987	3.009582619	0.390150083	0.568955529	PREDICTED: SOSS complex subunit C homolog
RS002387	GO:0044699	single-organism process	BP	0.411007636	0.065695965	0.063256072	4.124318324	3.004407781	0.390946082	0.569528625	PREDICTED: deoxyhypusine hydroxylase
RS100011	GO:0044699	single-organism process	BP	-0.187119175	-0.458379002	0.039139154	3.720929443	2.997212276	0.392055188	0.570485081	PREDICTED: geranylgeranyl pyrophosphate synthase
RS001976	GO:0044699	single-organism process	BP	-0.251391891	0.159463551	0.065403536	6.34133909	2.996329079	0.392191506	0.570485081	PREDICTED: formin-2
RS002089	GO:0044699	single-organism process	BP	-0.250948009	0.149321798	0.140990093	4.605226999	2.997849109	0.39195692	0.570485081	PREDICTED: ferredoxin-fold anticodon-binding domain-containing protein 1
RS007370	GO:0044699	single-organism process	BP	0.366879659	0.305144166	0.211380847	5.31062455	2.989248601	0.393285803	0.571350951	PREDICTED: WD repeat domain phosphoinositide-interacting protein 4-like
RS001591	GO:0044699	single-organism process	BP	0.025793299	0.263307601	-0.227967144	4.103259518	2.988175269	0.393451913	0.57146043	PREDICTED: myotubularin-related protein 2
RS100012	GO:0044699	single-organism process	BP	-0.480756966	0.267174146	0.019201116	3.217213517	2.982312811	0.39436024	0.572449615	PREDICTED: geranylgeranyl pyrophosphate synthase
RS001687	GO:0044699	single-organism process	BP	-0.719731145	-1.222301468	-0.648751586	0.570538248	2.979640994	0.394774798	0.572670789	PREDICTED: transcription factor RFX4
RS014634	GO:0044699	single-organism process	BP	0.185252175	-0.433726736	-0.57445767	1.576125573	2.976357818	0.395284718	0.573196976	PREDICTED: scaffold protein salvador
RS011470	GO:0044699	single-organism process	BP	-0.851403	-0.368190617	-1.419428633	0.475746125	2.972833288	0.395832742	0.573706362	PREDICTED: gustatory and odorant receptor 22-like
RS000283	GO:0044699	single-organism process	BP	0.387162419	0.173844023	0.20820605	5.733738134	2.970267551	0.396232089	0.574041979	PREDICTED: replication factor C subunit 5

RS004180	GO:0044699	single-organism process	BP	0.181244663	0.016093174	-0.195437889	5.503718069	2.968472005	0.39651176	0.574315021	PREDICTED: actin-related protein 2/3 complex subunit 2
RS000516	GO:0044699	single-organism process	BP	0.082112591	-0.030225782	-0.31775252	6.094076998	2.967302563	0.396694	0.574446848	PREDICTED: persulfide dioxygenase ETHE1, mitochondrial
RS011425	GO:0044699	single-organism process	BP	-0.421495798	-0.219418599	-0.328836876	4.809940968	2.965714873	0.396941531	0.57473921	PREDICTED: wiskott-Aldrich syndrome protein family member 3
RS013715	GO:0044699	single-organism process	BP	0.253463228	0.392667158	0.178778214	6.770284307	2.963354317	0.397309798	0.575140183	PREDICTED: dnaJ homolog subfamily C member 10-like
RS006833	GO:0044699	single-organism process	BP	0.508844783	-0.926836516	0.134300311	0.616587013	2.957970235	0.398150837	0.575600088	PREDICTED: sulfhydryl oxidase 1-like
RS000068	GO:0044699	single-organism process	BP	-0.396609694	-0.510016286	-0.175292961	3.520752812	2.957257498	0.398262285	0.575600088	PREDICTED: SH3 domain-binding protein 5 homolog
RS006760	GO:0044699	single-organism process	BP	-0.079958688	0.455256853	-0.08438531	2.501847147	2.959039176	0.39798374	0.575600088	PREDICTED: ras-related protein Rab-40C
RS000892	GO:0044699	single-organism process	BP	0.338138191	0.424482879	0.167447565	4.601167068	2.953865432	0.398793051	0.576108366	PREDICTED: calcium and integrin-binding family member 2
RS008113	GO:0044699	single-organism process	BP	-0.380396575	-0.235567209	0.170748265	4.686839393	2.953515927	0.398847772	0.576108366	PREDICTED: dual specificity protein phosphatase 10
RS005950	GO:0044699	single-organism process	BP	0.000537452	-0.326108944	0.039117289	6.989500858	2.952562975	0.398997008	0.576257834	PREDICTED: rho-associated protein kinase 2
RS011521	GO:0044699	single-organism process	BP	-0.751442034	-0.710386156	0.307766899	0.820244242	2.951499606	0.39916359	0.576300155	PREDICTED: retinol dehydrogenase 14
RS010226	GO:0044699	single-organism process	BP	-0.42640398	-0.691946426	-0.284833479	2.60959204	2.949041227	0.399548932	0.5765921	hypothetical protein
RS009591	GO:0044699	single-organism process	BP	-0.300835761	-0.461383255	-0.155026388	4.330389462	2.947890985	0.399729336	0.576770925	PREDICTED: DNA-binding protein SMUBP-2
RS001008	GO:0044699	single-organism process	BP	0.574766861	1.06264067	0.570653886	1.233181577	2.945083382	0.400169969	0.577223753	PREDICTED: 4-coumarate--CoA ligase 1
RS015004	GO:0044699	single-organism process	BP	1.027892445	1.271859333	0.69201976	0.986973614	2.939315228	0.40107652	0.578009705	PREDICTED: protein Wnt-1-like
RS002780	GO:0044699	single-organism process	BP	-0.020401082	0.243861311	-0.325967901	4.058126261	2.938988617	0.401127904	0.578009705	PREDICTED: S-adenosylmethionine decarboxylase proenzyme
RS004857	GO:0044699	single-organism process	BP	0.001882822	-0.133955239	-0.647468617	2.077593454	2.936643799	0.40149696	0.578409142	PREDICTED: alpha-1,3-mannosyl-glycoprotein 2-beta-N-acetylglucosaminyltransferase
RS002525	GO:0044699	single-organism process	BP	-0.583871468	-0.142039694	-0.260997572	4.004468135	2.935752421	0.401637331	0.57847902	PREDICTED: protein diaphanous
RS001050	GO:0044699	single-organism process	BP	0.016105631	-0.186255044	-0.337510647	4.754684371	2.928229901	0.402823594	0.579657261	PREDICTED: transcription factor Dp-1
RS002254	GO:0044699	single-organism process	BP	0.026779126	-0.240562604	-0.407019639	8.0023415	2.926808228	0.403048115	0.579800873	PREDICTED: non-specific lipid-transfer protein
RS000591	GO:0044699	single-organism process	BP	-0.210745642	-0.711938748	-0.062273638	2.249015287	2.914724059	0.404960781	0.581735608	PREDICTED: solute carrier family 41 member 1-like
RS013662	GO:0044699	single-organism process	BP	-0.35109086	-0.779115266	-0.381597224	2.892946474	2.912561078	0.405303937	0.582029393	PREDICTED: pyroglutamylated RFamide peptide receptor-like
RS001451	GO:0044699	single-organism process	BP	-0.56655501	-0.598725296	-0.286511941	3.41381086	2.912930447	0.405245319	0.582029393	PREDICTED: serine palmitoyltransferase small subunit A
RS003109	GO:0044699	single-organism process	BP	-0.563508854	-0.456515106	-0.489126671	3.799327004	2.910695341	0.405600131	0.582321939	PREDICTED: organic cation transporter protein-like
RS000507	GO:0044699	single-organism process	BP	0.333205088	0.034412845	-0.104515492	5.628089258	2.908006768	0.406027273	0.582369507	PREDICTED: Fanconi anemia group I protein
RS013241	GO:0044699	single-organism process	BP	-0.358652827	-0.673997448	-0.312616232	3.582135599	2.909240758	0.405831178	0.582369507	PREDICTED: plexin-A4
RS001459	GO:0044699	single-organism process	BP	0.281600556	0.369346526	0.129178152	6.415090299	2.908158384	0.406003175	0.582369507	PREDICTED: beclin-1-like protein
RS009503	GO:0044699	single-organism process	BP	-0.288639608	-0.114228373	0.099675684	5.337685499	2.905950405	0.406354228	0.582740282	Sorting nexin-29
RS005802	GO:0044699	single-organism process	BP	-0.201437577	-0.069914946	-0.593279778	3.766942431	2.895793271	0.407972418	0.584129676	PREDICTED: two pore potassium channel protein sup-9
RS007684	GO:0044699	single-organism process	BP	0.246558198	0.109156297	-0.12390993	5.372733164	2.884511863	0.409776038	0.586045813	PREDICTED: putative hydroxypyruvate isomerase
RS001823	GO:0044699	single-organism process	BP	-0.036751111	0.231020128	0.247332775	6.237547041	2.878220965	0.41078469	0.586955115	PREDICTED: dynein light chain roadblock-type 2
RS005563	GO:0044699	single-organism process	BP	-0.287333382	-0.127875056	0.182185235	4.579647806	2.875984629	0.411143752	0.587334893	PREDICTED: E3 ubiquitin-protein ligase CBL-B
RS002983	GO:0044699	single-organism process	BP	0.377379875	0.206577437	0.23042339	5.703954918	2.872033248	0.41177882	0.587908679	PREDICTED: mannose-6-phosphate isomerase
RS004668	GO:0044699	single-organism process	BP	0.149575289	0.400022809	0.167225784	7.451102141	2.863945114	0.4130813	0.589167146	PREDICTED: signal recognition particle 54 kDa protein
RS011230	GO:0044699	single-organism process	BP	0.34834624	0.215690427	0.018096064	4.617698053	2.862743697	0.413275065	0.589307294	PREDICTED: glycerol kinase-like
RS006563	GO:0044699	single-organism process	BP	-0.489174516	-0.417725507	-0.674213578	3.687949172	2.85405574	0.41467851	0.590575728	PREDICTED: EH domain-containing protein 3
RS012027	GO:0044699	single-organism process	BP	-0.231736103	0.053376129	-0.36568925	5.253210532	2.852106525	0.41499393	0.590824503	PREDICTED: Krueppel homolog 2
RS015274	GO:0044699	single-organism process	BP	0.190516592	0.903204914	0.887088726	0.57665691	2.849622605	0.415396163	0.59115192	PREDICTED: longitudinals lacking protein, isoform G-like
RS000021	GO:0044699	single-organism process	BP	0.352698076	0.223428782	0.088380723	6.096238225	2.849526925	0.415411664	0.59115192	PREDICTED: KDEL motif-containing protein 1-like
RS005365	GO:0044699	single-organism process	BP	0.361826129	0.155291905	0.110843753	6.532690167	2.845176087	0.416117021	0.59175449	PREDICTED: probable ATP-dependent RNA helicase DDX56
RS001904	GO:0044699	single-organism process	BP	-0.27687136	-0.081401122	0.3505686	9.687890931	2.842286187	0.416586082	0.592334615	PREDICTED: ATP synthase subunit d, mitochondrial
RS004572	GO:0044699	single-organism process	BP	0.133626501	0.187489315	0.446168466	7.392149193	2.839995652	0.416958171	0.59268298	PREDICTED: reticulon-4-interacting protein 1, mitochondrial
RS010277	GO:0044699	single-organism process	BP	0.378750319	-0.005930032	0.308961094	4.357809539	2.83238491	0.418196494	0.593949885	PREDICTED: low molecular weight phosphotyrosine protein phosphatase-like
RS006427	GO:0044699	single-organism process	BP	0.128215159	0.420409835	0.189186973	7.734054421	2.828202212	0.418878349	0.594406076	PREDICTED: protein transport protein Sec24C
RS008083	GO:0044699	single-organism process	BP	-0.156978799	-0.394347077	0.118142017	4.047485283	2.82626452	0.41919454	0.594787746	PREDICTED: E3 ubiquitin-protein ligase CHIP
RS008538	GO:0044699	single-organism process	BP	-0.336935574	-0.037576603	0.029262022	4.864941767	2.823630813	0.419624624	0.595140247	PREDICTED: protein tamozhennic
RS007450	GO:0044699	single-organism process	BP	-0.472518932	-0.276576764	-0.555924351	4.528276271	2.816126644	0.42085206	0.596362528	PREDICTED: cGMP-dependent protein kinase, isozyme 1
RS012188	GO:0044699	single-organism process	BP	0.093610346	0.354131078	0.236809978	4.973492608	2.814705945	0.421084775	0.596595979	PREDICTED: guanine nucleotide exchange factor MSS4 homolog
RS005369	GO:0044699	single-organism process	BP	0.201414303	0.403119805	0.336262142	5.699731667	2.814401367	0.421134679	0.596599567	PREDICTED: non-homologous end-joining factor 1-like
RS006685	GO:0044699	single-organism process	BP	0.007325471	-0.293223436	-0.432311597	3.533055228	2.809344423	0.421963968	0.597572721	PREDICTED: GDP-fucose protein O-fucosyltransferase 1
RS009693	GO:0044699	single-organism process	BP	0.246804497	0.323445058	0.103062807	5.738123837	2.80490925	0.422692405	0.598335185	PREDICTED: actin-related protein 2/3 complex subunit 4
RS005325	GO:0044699	single-organism process	BP	0.257540318	0.304747707	-0.031160316	6.755234502	2.802829983	0.423034263	0.598528799	PREDICTED: DNA-dependent protein kinase catalytic subunit
RS001701	GO:0044699	single-organism process	BP	-0.428537809	-0.354853252	-0.359559576	8.130709933	2.794774192	0.424360901	0.599618668	PREDICTED: ornithine decarboxylase 1-like
RS003724	GO:0044699	single-organism process	BP	-0.563027663	-0.354338625	-0.171950323	3.785653889	2.790806177	0.425015621	0.600139784	PREDICTED: signal transducer and activator of transcription 5B
RS003893	GO:0044699	single-organism process	BP	-0.636740638	0.394784506	-0.728645752	0.650265231	2.789115462	0.425294842	0.600332127	PREDICTED: formin-J
RS007765	GO:0044699	single-organism process	BP	-0.115537356	-0.332127172	-0.312950076	7.359507191	2.789151021	0.425288967	0.600332127	PREDICTED: kinesin-like protein KIF3A
RS011918	GO:0044699	single-organism process	BP	-0.283368406	-0.591171038	-0.500171174	2.742116745	2.787050879	0.425636011	0.600611757	PREDICTED: dedicator of cytokinesis protein 9
RS000487	GO:0044699	single-organism process	BP	0.164758079	-0.207833338	-0.024547781	5.972297581	2.785136249	0.425952604	0.600838644	PREDICTED: protein broad-minded-like
RS003842	GO:0044699	single-organism process	BP	-0.202795939	-1.198204704	-0.607414579	0.64530997	2.785488426	0.425894355	0.600838644	PREDICTED: hematopoietically-expressed homeobox protein hhex
RS013884	GO:0044699	single-organism process	BP	-1.483581091	-0.28373006	-0.000921218	2.848064172	2.784924475	0.425987633	0.600838644	PREDICTED: aromatic-L-amino-acid decarboxylase-like
RS013821	GO:0044699	single-organism process	BP	0.50131938	0.258448114	-0.078761892	2.315318862	2.78133735	0.426581343	0.601473963	PREDICTED: transmembrane protein 107
RS003389	GO:0044699	single-organism process	BP	-0.057326789	-0.435286332	-0.210979187	6.207606112	2.780907596	0.426652518	0.601506976	PREDICTED: alpha-mannosidase 2
RS003489	GO:0044699	single-organism process	BP	0.434058064	0.053799645	0.547507794	3.924561486	2.780217171	0.426766885	0.601533537	caspase-1
RS011315	GO:0044699	single-organism process	BP	-0.020934269	-0.334379922	-0.189927492	5.449158477	2.775948082	0.427474611	0.602126748	PREDICTED: protein PTHB1
RS004472	GO:0044699	single-organism process	BP	0.23338312	0.299367112	-0.155614298	3.964638541	2.775417072	0.427562709	0.602126862	PREDICTED: exosome complex component RRP43-like
RS010988	GO:0044699	single-organism process	BP	0.372032907	0.563714151	0.72777503	2.023536873	2.77132253	0.428242522	0.602871264	PREDICTED: dolichol phosphate-mannose biosynthesis regulatory protein
RS005764	GO:0044699	single-organism process	BP	-0.087456614	0.263340135	0.009281276	5.815827041	2.768997792	0.428628893	0.603212903	PREDICTED: UPF0565 protein C2orf69 homolog
RS004582	GO:0044699	single-organism process	BP	-0.73583249	-0.347054977	-0.318238203	3.047739544	2.76763295	0.428855862	0.603397816	PREDICTED: netrin receptor UNC5C-like
RS004407	GO:0044699	single-organism process	BP	0.25797716	-0.172857716	0.145052432	8.088414476	2.766051469	0.429118983	0.603632801	PREDICTED: DNA damage-binding protein 1
RS002907	GO:0044699	single-organism process	BP	-0.275272942	-0.300983974	-1.055264998	1.533528744	2.761518018	0.429873976	0.604289859	ankyrin repeat protein
RS002668	GO:0044699	single-organism process	BP	0.005092215	0.291856379	0.276868518	5.647493836	2.756251541	0.430752417	0.604718374	PREDICTED: UPF0729 protein GD16342
RS005020	GO:0044699	single-organism process	BP	0.496667832	0.268219535	0.273665044	4.956460924	2.75576276	0.43083402	0.604718374	PREDICTED: diphthamide biosynthesis protein 2
RS011659	GO:0044699	single-organism process	BP	-0.154486133	-0.403035914	-0.349850862	4.578285897	2.755660836	0.430851038	0.604718374	PREDICTED: ribonucleases P/MRP protein subunit POP1
RS004134	GO:0044699	single-organism process	BP	0.004171617	-0.295632624	-0.495731886	5.679937701	2.753463703	0.431218022	0.605098581	PREDICTED: ribonucleoside-diphosphate reductase large subunit
RS015070	GO:0044699	single-organism process	BP	-0.286375301	-0.09814047	-0.700137809	2.142659578	2.750463547	0.431719548	0.60559991	PREDICTED: unconventional myosin-Ie
RS013346	GO:0044699	single-organism process	BP	-0.095021443	-0.55837102	-0.473417441	6.796225014	2.749473682	0.431885126	0.605764704	PREDICTED: neurogenic locus Notch protein
RS011686	GO:0044699	single-organism process	BP	-0.652816077	-0.692313812	-0.834928913	1.23665669	2.748017073	0.432128872	0.606039089	PREDICTED: TWiK family of potassium channels protein 18-like
RS009427	GO:0044699	single-organism process	BP	-0.141296429	-0.597579347	-0.600562142	2.512316691	2.74363063	0.432863574	0.606529133	PREDICTED: peroxidase
RS008409	GO:0044699	single-organism process	BP	-0.063013939	-0.296980216	-0.260966083	6.681205059	2.73900254	0.43363986	0.607346575	PREDICTED: transformation/transcription domain-associated protein
RS008625	GO:0044699	single-organism process	BP	-0.28446001	-0.40339653	-0.325442643	4.728523792	2.735354216	0.434252612	0.607934397	PREDICTED: Bardet-Biedl syndrome 5 protein
RS010806	GO:0044699	single-organism process	BP	0.417731943	-0.214862196	0.112210512	3.575135255	2.731397887	0.434917896	0.608527495	PREDICTED: matrix metalloproteinase-14
RS012080	GO:0044699	single-organism process	BP	0.191298298	0.061195457	0.414787636	4.58343694	2.725171136	0.435966658	0.609656239	PREDICTED: intraflagellar transport protein 27 homolog
RS001922	GO:0044699	single-organism process	BP	-0.088945977	-0.394803448	-0.247618856	5.06916862	2.717551953	0.43725276	0.610859546	PREDICTED: CAS1 domain-containing protein 1
RS006283	GO:0044699	single-organism process	BP	0.06605322	0.18971651	0.362569658	6.613446347	2.716610089	0.43741196	0.610973711	PREDICTED: charged multivesicular body protein 5
RS004207	GO:0044699	single-organism process	BP	-0.450748093	-0.259258272	-0.448403469	6.976635555	2.715150178	0.437658817	0.611208155	PREDICTED: Niemann-Pick C1 protein
RS003847	GO:0044699	single-organism process	BP	-0.07803106	-0.071687271	0.272229928	4.838857373	2.707139566	0.439015363	0.612694978	PREDICTED: DNL-type zinc finger protein-like
RS014996	GO:0044699	single-organism process	BP	0.226630703	-0.211533947	-0.36248209	3.237412291	2.699093235	0.440381412	0.614193078	PREDICTED: membrane-associated tyrosine- and threonine-specific cdc2-inhibitory kinase-like
RS002522	GO:0044699	single-organism process	BP	-0.532539074	0.383587923	0.093594657	0.9041521	2.694414048	0.441177403	0.614989081	PREDICTED: tachykinin-like peptides receptor 99D
RS012396	GO:0044699	single-organism process	BP	0.327124195	0.012418108	0.069947446	5.465353023	2.691227933	0.441720074	0.615449672	PREDICTED: peroxisome biogenesis factor 13
RS006959	GO:0044699	single-organism process	BP	0.218906083	-0.157184986	-0.030285587	6.750313653	2.691729854	0.441634549	0.615449672	PREDICTED: serrate RNA effector molecule homolog
RS002934	GO:0044699	single-organism process	BP	0.218014659	0.303665602	0.379570495	6.73161799	2.682472139	0.443214194	0.617118801	PREDICTED: phosphatidylinositol 3-kinase catalytic subunit type 3
RS010387	GO:0044699	single-organism process	BP	0.273309892	0.271638567	0.040204127	6.12761374	2.679872387	0.443658615	0.617464537	PREDICTED: actin-related protein 2/3 complex subunit 1A
RS010893	GO:0044699	single-organism process	BP	0.040129704	-0.138943638	-0.371704357	6.935894095	2.67917968	0.443777093	0.617492952	PREDICTED: thioredoxin domain-containing protein
RS007943	GO:0044699	single-organism process	BP	0.102316261	-0.7855998	0.472804112	0.660304745	2.678707542	0.44385786	0.617537107	PREDICTED: leucine-rich repeat-containing protein 15
RS007347	GO:0044699	single-organism process	BP	0.551585526	0.378242267	0.201825987	3.076235953	2.671994293	0.44500757	0.618697209	PREDICTED: mortality factor 4-like protein 1
RS008861	GO:0044699	single-organism process	BP	-0.372985263	-0.686791432	-0.964879982	1.391079077	2.670480351	0.445267181	0.618882561	PREDICTED: partner of bursicon
RS005905	GO:0044699	single-organism process	BP	-0.707729301	-1.010882877	-0.25518329	0.794458623	2.663576794	0.446452565	0.62012801	AAEL002484-PA
RS009679	GO:0044699	single-organism process	BP	0.401967086	0.328288672	0.532346962	4.297978762	2.663541194	0.446458684	0.62012801	PREDICTED: U6 snRNA phosphodiesterase
RS008753	GO:0044699	single-organism process	BP	0.164095151	0.237972147	0.430890072	5.134544592	2.659489406	0.447155601	0.620981671	PREDICTED: 2-methoxy-6-polyprenyl-1,4-benzoquinol methylase, mitochondrial
RS014759	GO:0044699	single-organism process	BP	0.297402268	0.349036548	0.391747272	4.965839912	2.656479362	0.447673905	0.621404735	PREDICTED: homologous-pairing protein 2 homolog
RS005803	GO:0044699	single-organism process	BP	-0.002024478	-0.530272126	0.012443909	3.547775495	2.654489256	0.448016852	0.621743718	Adenosine receptor A2b
RS012107	GO:0044699	single-organism process	BP	-0.368799719	0.188292375	-0.013404979	5.272470317	2.653348424	0.448213543	0.621757305	PREDICTED: G protein-activated inward rectifier potassium channel 3-like
RS004681	GO:0044699	single-organism process	BP	-0.505077425	-0.166995306	-0.065709253	4.449641166	2.649307118	0.448910868	0.622367181	PREDICTED: parafibromin
RS010212	GO:0044699	single-organism process	BP	-0.044467639	0.041882794	0.391177961	4.199952764	2.647868881	0.449159246	0.62256752	PREDICTED: glutamyl-tRNA(Gln) amidotransferase subunit A, mitochondrial
RS006518	GO:0044699	single-organism process	BP	0.371021394	0.1479039	0.105703534	6.00773277	2.647615178	0.449203072	0.62256752	PREDICTED: intraflagellar transport protein 122 homolog
RS000379	GO:0044699	single-organism process	BP	0.238639889	0.002576451	-0.1801865	4.995979504	2.646519158	0.44939244	0.622623585	PREDICTED: protein MTO1 homolog, mitochondrial
RS000882	GO:0044699	single-organism process	BP	0.409867145	0.118344836	0.242213936	6.775044333	2.641914509	0.450188729	0.623383931	PREDICTED: general transcription factor IIF subunit 1
RS006771	GO:0044699	single-organism process	BP	0.475535507	0.066379896	0.054545274	5.13346181	2.640006509	0.450519017	0.623635579	PREDICTED: C-terminal-binding protein
RS015262	GO:0044699	single-organism process	BP	-0.248404397	0.139144696	-0.956013952	0.939962441	2.639400897	0.450623893	0.6237122	PREDICTED: longitudinals lacking protein
RS009567	GO:0044699	single-organism process	BP	-0.033721687	0.291703775	0.477768837	3.867183318	2.631573468	0.451981177	0.625041281	PREDICTED: ephrin type-B receptor 1-B
RS001310	GO:0044699	single-organism process	BP	-0.326851591	-0.081171675	-0.290330675	5.399229597	2.630190851	0.452221267	0.625235991	PREDICTED: DNA polymerase zeta catalytic subunit

RS014879	GO:0044699	single-organism process	BP	-0.246283404	-0.362594032	0.41505142	1.337328694	2.627851228	0.452627775	0.625660651	PREDICTED: glycine receptor subunit alphaZ1-like
RS007750	GO:0044699	single-organism process	BP	0.574488725	0.504255201	0.399021795	3.006361843	2.625674098	0.453006314	0.62588798	PREDICTED: heparan-sulfate 6-O-sulfotransferase 2
RS006187	GO:0044699	single-organism process	BP	0.296007208	0.259817588	0.073669551	5.442707435	2.624060722	0.453286997	0.62592491	PREDICTED: cyclin-dependent-like kinase 5
RS009493	GO:0044699	single-organism process	BP	-0.431408946	-0.208879752	-0.440237613	4.096404936	2.61948123	0.454084467	0.626711209	PREDICTED: raf homolog serine/threonine-protein kinase phl
RS013339	GO:0044699	single-organism process	BP	0.29787968	0.142046103	-0.062634811	6.486462438	2.616990878	0.45451861	0.627241658	PREDICTED: actin-related protein 3
RS009023	GO:0044699	single-organism process	BP	-0.037015581	0.692056765	0.134440297	1.505404877	2.615326347	0.454808974	0.627573596	PREDICTED: transmembrane emp24 domain-containing protein 5-like
RS005105	GO:0044699	single-organism process	BP	-0.835967915	-0.676145164	-0.984137452	0.462005287	2.614453848	0.454961233	0.627714918	PREDICTED: segmentation protein Runt
RS013156	GO:0044699	single-organism process	BP	0.360567235	0.492762353	0.357878004	4.027595579	2.613384946	0.455147824	0.62790357	PREDICTED: tyrosine-protein phosphatase non-receptor type 2
RS008169	GO:0044699	single-organism process	BP	-0.945986481	-0.538298521	-0.571457627	0.987678192	2.612051091	0.455380751	0.628118647	PREDICTED: alpha-1A adrenergic receptor
RS000270	GO:0044699	single-organism process	BP	-0.344979442	-0.238162884	-0.139757992	5.591606358	2.607428323	0.456188754	0.628723492	PREDICTED: kinase suppressor of Ras 2
RS007790	GO:0044699	single-organism process	BP	0.13034852	0.225712601	0.386180211	4.625198528	2.606288996	0.456388071	0.628856824	PREDICTED: mitochondrial glutamate carrier 1-like
RS009287	GO:0044699	single-organism process	BP	-0.152507426	-0.649151389	-0.513900002	1.89610463	2.604067288	0.456776945	0.62925496	PREDICTED: ero1-like protein
RS000015	GO:0044699	single-organism process	BP	0.020068586	-0.335498379	-0.389018644	4.072380722	2.601854425	0.457164535	0.629720021	PREDICTED: phosphatidylinositol-glycan biosynthesis class W protein-like
RS007076	GO:0044699	single-organism process	BP	-0.333532028	0.014299236	-0.014087004	5.372273562	2.600146824	0.457463807	0.629994444	PREDICTED: serine palmitoyltransferase 1
RS010765	GO:0044699	single-organism process	BP	0.276309513	0.246865122	0.398690978	5.334170587	2.597772984	0.457880105	0.630292059	PREDICTED: nitrogen permease regulator 3-like protein
RS013643	GO:0044699	single-organism process	BP	-0.274291247	0.028833824	0.392182073	4.734266638	2.593688814	0.458597054	0.630865242	PREDICTED: gustatory receptor for sugar taste 64f-like
RS009038	GO:0044699	single-organism process	BP	-0.068830404	0.280776911	0.068709268	6.963301239	2.590030142	0.459240073	0.631213738	PREDICTED: ras-related protein Rab-11A
RS000050	GO:0044699	single-organism process	BP	0.009682946	0.212878195	-0.114950849	6.0212319	2.590229463	0.459205023	0.631213738	PREDICTED: GPI transamidase component PIG-S
RS006111	GO:0044699	single-organism process	BP	0.385986662	0.472088444	0.3365299	4.200617162	2.587948401	0.459606266	0.631563701	PREDICTED: protein yippee-like 5
RS010990	GO:0044699	single-organism process	BP	0.270197659	0.242457664	0.475127184	4.208211224	2.583900634	0.460318967	0.632405047	PREDICTED: mitotic spindle assembly checkpoint protein MAD1
RS010426	GO:0044699	single-organism process	BP	-0.0320805	-0.19528696	-0.535965982	2.761673686	2.581268642	0.460782863	0.632865926	PREDICTED: glutamate receptor ionotropic, NMDA 2B
RS011498	GO:0044699	single-organism process	BP	-0.021091891	-0.187939322	-1.146135739	0.625316914	2.572342043	0.462358988	0.634308181	PREDICTED: protein trapped in endoderm-1
RS011111	GO:0044699	single-organism process	BP	0.247529122	0.335154268	0.426754458	5.263341647	2.570885268	0.462616613	0.634592487	PREDICTED: cell division cycle protein 123 homolog
RS009814	GO:0044699	single-organism process	BP	1.646314621	1.402135716	0.697258946	0.548777018	2.569907062	0.462789668	0.634760737	PREDICTED: probable kinetochore protein NUF2
RS003046	GO:0044699	single-organism process	BP	0.197255301	0.326104291	0.090024596	6.676753805	2.564536477	0.463740707	0.635649811	PREDICTED: membrane-bound transcription factor site-1 protease
RS011076	GO:0044699	single-organism process	BP	0.672813702	0.671977204	0.36668554	1.736169215	2.564903592	0.463675648	0.635649811	CG1504
RS011721	GO:0044699	single-organism process	BP	0.19190141	0.061457593	0.321440339	6.123202877	2.564611469	0.463727417	0.635649811	PREDICTED: serine/threonine-protein phosphatase 4 catalytic subunit
RS013293	GO:0044699	single-organism process	BP	0.198558902	1.083035633	0.686996214	0.580149697	2.56014753	0.464519072	0.636353747	PREDICTED: synaptic vesicle glycoprotein 2B-like
RS014139	GO:0044699	single-organism process	BP	-0.011390372	0.270176171	0.212987304	5.391904902	2.555043661	0.465425537	0.637127088	PREDICTED: ras-related protein Rab-4B
RS001412	GO:0044699	single-organism process	BP	-0.201139827	-0.362439088	-0.669833899	10.04170695	2.551863008	0.465991144	0.637762711	catalase
RS011987	GO:0044699	single-organism process	BP	-0.488068572	-0.15342032	0.523787862	0.777826252	2.550027389	0.466317816	0.638071119	PREDICTED: rho-related GTP-binding protein RhoE
RS004026	GO:0044699	single-organism process	BP	0.361893932	0.287993844	0.385913985	4.470336965	2.550278546	0.466273109	0.638071119	PREDICTED: ubiquinone biosynthesis protein COQ4 homolog, mitochondrial
RS006942	GO:0044699	single-organism process	BP	0.361497889	0.092609345	0.223627816	4.685458904	2.535985268	0.468822828	0.640316093	PREDICTED: NAD kinase 2, mitochondrial
RS007973	GO:0044699	single-organism process	BP	0.255068646	0.370011576	0.284397768	6.620720548	2.53491853	0.469013562	0.640437689	PREDICTED: zinc transporter 9
RS012653	GO:0044699	single-organism process	BP	0.056734656	-0.372769409	-0.163659265	4.115912994	2.527360648	0.470366688	0.641826581	PREDICTED: 3-oxoacyl-[acyl-carrier-protein] synthase, mitochondrial
RS014174	GO:0044699	single-organism process	BP	0.243141358	0.291049728	-0.048014052	4.335741869	2.526539923	0.470513813	0.641828652	PREDICTED: DNA repair protein XRCC1
RS012287	GO:0044699	single-organism process	BP	0.600207066	0.30325307	0.303579049	3.807779021	2.522761118	0.47119168	0.642297653	PREDICTED: four-jointed box protein 1-like
RS013118	GO:0044699	single-organism process	BP	-0.001785963	0.210424479	0.358880231	4.813609609	2.51932422	0.471808885	0.642784962	PREDICTED: ubiquinone biosynthesis protein COQ9, mitochondrial
RS009279	GO:0044699	single-organism process	BP	0.294401775	0.208728083	0.372173078	5.384293591	2.517191015	0.472192292	0.643035221	PREDICTED: probable low affinity copper uptake protein 2
RS009008	GO:0044699	single-organism process	BP	0.292080821	0.274945291	0.343598243	6.179710033	2.513516153	0.472853363	0.643656576	PREDICTED: vacuolar protein sorting-associated protein 54
RS001843	GO:0044699	single-organism process	BP	0.125103861	0.358523006	0.276895591	5.751467293	2.512786402	0.472984725	0.64369674	pastrel
RS005529	GO:0044699	single-organism process	BP	-0.488235901	-0.539862818	-0.211684148	4.097895297	2.512486448	0.473038728	0.643700675	PREDICTED: ras-related protein Rab-2A
RS002192	GO:0044699	single-organism process	BP	-0.380790599	-0.281163701	-0.149636554	4.800742827	2.507854396	0.47387329	0.644209868	PREDICTED: L-2-hydroxyglutarate dehydrogenase, mitochondrial
RS003198	GO:0044699	single-organism process	BP	-1.073241752	-1.028205771	-0.905668183	0.370380606	2.506990701	0.474029032	0.644352037	PREDICTED: glutamate receptor ionotropic, delta-1-like
RS005885	GO:0044699	single-organism process	BP	0.175747782	0.335001627	0.089694216	6.271918403	2.50323783	0.474706219	0.645133282	PREDICTED: eukaryotic translation initiation factor 6
RS014929	GO:0044699	single-organism process	BP	-0.66549843	0.463934316	-0.414340811	1.378765976	2.500427323	0.475213861	0.645506655	PREDICTED: sclerostin domain-containing protein 1
RS013776	GO:0044699	single-organism process	BP	0.324546544	0.227202309	0.236664132	6.156639201	2.498135106	0.475628206	0.645682607	PREDICTED: general transcription factor IIH subunit 3
RS009609	GO:0044699	single-organism process	BP	0.237545237	0.05526396	-0.190935826	4.176700496	2.498126856	0.475629697	0.645682607	PREDICTED: S-methyl-5'-thioadenosine phosphorylase
RS003214	GO:0044699	single-organism process	BP	0.817513479	0.952415195	0.900489695	0.817958486	2.496776884	0.475873854	0.64588375	PREDICTED: potassium voltage-gated channel subfamily KQT member 4
RS010552	GO:0044699	single-organism process	BP	-0.474155216	-0.15204609	-0.166244758	7.298549599	2.496174275	0.475982874	0.645962103	PREDICTED: adenylyl cyclase-associated protein 1
RS010395	GO:0044699	single-organism process	BP	-0.288904532	-0.397767207	-0.731992737	2.398518934	2.495812833	0.476048274	0.645981247	PREDICTED: beta-1-syntrophin
RS015275	GO:0044699	single-organism process	BP	-0.597370658	0.178654375	-0.229128419	1.566798085	2.494770435	0.476236924	0.646028417	PREDICTED: longitudinals lacking protein
RS011106	GO:0044699	single-organism process	BP	0.010939481	-0.320842065	-0.095148007	5.595805295	2.495293912	0.476142179	0.646028417	PREDICTED: cytoplasmic dynein 1 light intermediate chain 2
RS009713	GO:0044699	single-organism process	BP	0.276518514	0.284318052	0.365753819	7.640318284	2.489379064	0.477213583	0.646552279	PREDICTED: peptidyl-alpha-hydroxyglycine alpha-amidating lyase 1
RS006138	GO:0044699	single-organism process	BP	0.077942232	0.584419518	-0.370544993	1.33370078	2.490635188	0.476985892	0.646552279	PREDICTED: potassium channel subfamily K member 18-like
RS009637	GO:0044699	single-organism process	BP	0.194650044	0.636197094	0.06162723	3.173885651	2.489834821	0.47713096	0.646552279	PREDICTED: ephrin-B2
RS009263	GO:0044699	single-organism process	BP	0.377755056	0.2256657	0.072028892	4.676821532	2.490710429	0.476972256	0.646552279	PREDICTED: methylcrotonoyl-CoA carboxylase beta chain, mitochondrial
RS008976	GO:0044699	single-organism process	BP	0.75451769	0.313876671	0.317858045	3.733164683	2.484580804	0.478084123	0.647348828	PREDICTED: zinc finger protein 271-like
RS012264	GO:0044699	single-organism process	BP	0.056631713	0.03848964	-0.253035463	6.466737478	2.48425876	0.478142596	0.647358402	PREDICTED: galactosylgalactosylxylosylprotein 3-beta-glucuronosyltransferase I
RS000905	GO:0044699	single-organism process	BP	0.022025399	0.042858662	0.42533997	8.298571127	2.483368496	0.478304268	0.647507681	PREDICTED: cytochrome b-c1 complex subunit 10-like
RS007113	GO:0044699	single-organism process	BP	-0.384136599	0.319103771	-0.084532698	2.002723032	2.478429289	0.47920201	0.648444194	hypothetical protein
RS001213	GO:0044699	single-organism process	BP	0.297681102	0.175792368	0.444019573	6.821186204	2.475167442	0.479795603	0.648968514	PREDICTED: probable leucine--tRNA ligase, mitochondrial
RS002551	GO:0044699	single-organism process	BP	-0.485756409	0.01884223	-0.455567996	3.575771237	2.474501092	0.479916937	0.64906292	PREDICTED: B-cell lymphoma 3 protein
RS002425	GO:0044699	single-organism process	BP	0.181684868	-0.400483054	0.16026535	2.40842669	2.472215923	0.480333221	0.649556167	PREDICTED: DNA repair protein RAD52 homolog
RS002759	GO:0044699	single-organism process	BP	0.028967615	0.090840031	0.328940162	5.233792198	2.466480112	0.481379347	0.650551721	PREDICTED: bifunctional methylenetetrahydrofolate dehydrogenase/cyclohydrolase, mitochondrial
RS007258	GO:0044699	single-organism process	BP	0.165246919	0.394416853	-0.249399331	2.357145913	2.461663179	0.482259262	0.651391371	PREDICTED: male-specific lethal 1 homolog
RS003585	GO:0044699	single-organism process	BP	-0.526969653	-0.325999045	-0.519425311	4.846026814	2.460616462	0.482450634	0.65141528	PREDICTED: monocarboxylate transporter 3
RS011473	GO:0044699	single-organism process	BP	-0.143652931	-0.426507896	-0.271073598	3.841726772	2.460355232	0.482498404	0.65141528	PREDICTED: probable galactose-1-phosphate uridylyltransferase
RS001868	GO:0044699	single-organism process	BP	-0.245802404	-0.46166884	-0.481659652	2.636431688	2.458988236	0.482748441	0.651450759	PREDICTED: extracellular tyrosine-protein kinase PKDCC
RS013504	GO:0044699	single-organism process	BP	0.005631654	-0.378075339	-0.198417431	7.124669527	2.458877101	0.482768773	0.651450759	PREDICTED: GRIP and coiled-coil domain-containing protein 2
RS006131	GO:0044699	single-organism process	BP	-0.352165306	0.039176788	-0.281451791	4.380937286	2.456575471	0.483190008	0.651879481	PREDICTED: thioredoxin domain-containing protein 11
RS009733	GO:0044699	single-organism process	BP	0.312382918	0.283612789	0.271443928	5.620793269	2.453710167	0.483714805	0.652349219	PREDICTED: U6 snRNA-associated Sm-like protein LSm6
RS009282	GO:0044699	single-organism process	BP	-0.523115983	-0.205799461	-0.290286585	9.304493683	2.454208505	0.4836235	0.652349219	PREDICTED: spectrin alpha chain
RS009087	GO:0044699	single-organism process	BP	-0.753493655	-0.466320961	-0.51762636	2.340610515	2.452889414	0.483865213	0.652426585	PREDICTED: probable glutamate receptor
RS012899	GO:0044699	single-organism process	BP	0.204967532	0.199038052	0.483016212	5.559314101	2.452259782	0.483980622	0.652426585	PREDICTED: SID1 transmembrane family member 1-like
RS010757	GO:0044699	single-organism process	BP	0.19221425	-0.12605948	-0.032665115	6.370075282	2.45159303	0.484102858	0.652426585	PREDICTED: WASH complex subunit 7
RS014728	GO:0044699	single-organism process	BP	0.289084042	-0.158860536	0.433007903	3.145521858	2.44396679	0.485502697	0.653389716	pastrel
RS014448	GO:0044699	single-organism process	BP	0.063218695	0.360518634	0.2332296	4.966069177	2.442711039	0.485733499	0.653560651	PREDICTED: neurogenic locus protein delta
RS013267	GO:0044699	single-organism process	BP	-0.615367071	-0.558981829	-0.137728349	2.506265497	2.442381539	0.485794075	0.653572329	PREDICTED: neuropeptide F receptor
RS007514	GO:0044699	single-organism process	BP	-0.393644456	0.206862252	-0.369998274	2.851975701	2.440356048	0.48616657	0.653933758	PREDICTED: solute carrier family 26 member 10-like
RS000334	GO:0044699	single-organism process	BP	-0.423750188	-0.108130859	-0.305232423	3.908975308	2.437902331	0.486618116	0.65440134	PREDICTED: G protein-coupled receptor kinase 1
RS005082	GO:0044699	single-organism process	BP	-0.615167969	-0.515665964	-0.377307828	2.030498416	2.438057713	0.486589511	0.65440134	PREDICTED: ethanolaminephosphotransferase 1-like
RS011305	GO:0044699	single-organism process	BP	-0.404511495	-0.299486895	-0.550946754	9.870902688	2.437547415	0.486683456	0.65441933	PREDICTED: prosaposin
RS006905	GO:0044699	single-organism process	BP	-0.790316136	-0.068876999	0.391201443	1.132571279	2.436707651	0.486838085	0.654487494	PREDICTED: pancreatic lipase-related protein 2-like
RS003185	GO:0044699	single-organism process	BP	-1.123448995	-0.350035568	-0.063446673	0.692769609	2.436988799	0.486786312	0.654487494	sanpodo
RS003540	GO:0044699	single-organism process	BP	-0.647024675	-0.24831789	-0.831592146	2.164398377	2.434440616	0.487255714	0.654909121	Synaptotagmin-like protein 5
RS005714	GO:0044699	single-organism process	BP	0.681969754	1.448973415	1.269842108	0.332142713	2.433618669	0.487407201	0.655042822	PREDICTED: cyclic AMP response element-binding protein A
RS002497	GO:0044699	single-organism process	BP	-0.212234533	-0.495353126	-0.227222594	7.913801195	2.430343888	0.488011114	0.655434784	PREDICTED: phosphoglycerate kinase
RS011684	GO:0044699	single-organism process	BP	-0.692170143	-0.042506964	-0.922207534	1.283681737	2.430556184	0.487971946	0.655434784	Titin
RS007216	GO:0044699	single-organism process	BP	-0.177290189	-0.250540079	-0.343800823	5.202114494	2.428633119	0.488326834	0.655788883	PREDICTED: probable G-protein coupled receptor 125
RS001011	GO:0044699	single-organism process	BP	0.31200213	0.242431579	0.363583596	6.468484747	2.423265963	0.489318366	0.65684028	PREDICTED: leukocyte tyrosine kinase receptor
RS006793	GO:0044699	single-organism process	BP	0.21984434	0.344651115	0.203000906	5.387685985	2.415927078	0.490676689	0.65773111	PREDICTED: TATA box-binding protein-like protein 1
RS002311	GO:0044699	single-organism process	BP	0.21167088	0.356479307	0.364913574	7.124016358	2.415178378	0.490815427	0.65773111	PREDICTED: putative GTP-binding protein 6
RS010636	GO:0044699	single-organism process	BP	0.088918176	0.399373514	0.287267336	7.168378761	2.414984703	0.490851321	0.65773111	PREDICTED: rapamycin-insensitive companion of mTOR
RS005567	GO:0044699	single-organism process	BP	-0.629090951	0.153446724	-0.226041023	2.346109897	2.410435898	0.49169494	0.658299907	PREDICTED: transmembrane protein 145
RS012768	GO:0044699	single-organism process	BP	0.513703894	0.439908485	0.04771159	2.824489396	2.407464471	0.492246626	0.658734886	PREDICTED: DNA fragmentation factor subunit alpha-like
RS005327	GO:0044699	single-organism process	BP	0.594762406	0.291633704	0.291495001	2.777375117	2.400363324	0.493566994	0.660151096	DNA mismatch repair protein Msh3
RS007812	GO:0044699	single-organism process	BP	0.194272698	-0.14705421	-0.205244538	4.63586196	2.399467902	0.49373368	0.660303915	PREDICTED: MOXD1 homolog 2
RS008036	GO:0044699	single-organism process	BP	-0.91530135	-0.720765274	-0.735078429	0.719805004	2.397350586	0.494128001	0.660480582	PREDICTED: muscarinic acetylcholine receptor DM1
RS004471	GO:0044699	single-organism process	BP	0.302505199	0.1016203	-0.272900349	3.077100879	2.396738775	0.494241988	0.660492741	PREDICTED: Y+L amino acid transporter 2
RS014064	GO:0044699	single-organism process	BP	0.4036205	0.091122921	0.315423844	4.209003705	2.392802345	0.49497587	0.66126298	PREDICTED: dolichyldiphosphatase 1-like
RS008673	GO:0044699	single-organism process	BP	-0.420529009	-0.633051142	-0.599758098	2.219976693	2.386148983	0.496218193	0.662435362	PREDICTED: BBSome-interacting protein 1
RS012814	GO:0044699	single-organism process	BP	-0.136459218	-0.546290696	0.233200678	1.706530127	2.383035606	0.496800352	0.66310796	PREDICTED: carbonic anhydrase-related protein 10
RS005655	GO:0044699	single-organism process	BP	-0.366916241	-0.556778303	-0.335965357	3.236628275	2.382873011	0.496830769	0.66310796	PREDICTED: cyclin-dependent kinase inhibitor 1
RS006410	GO:0044699	single-organism process	BP	-0.237071073	-0.551422214	-0.407318951	3.379585522	2.380304993	0.497311372	0.663327623	PREDICTED: optomotor-blind protein
RS013452	GO:0044699	single-organism process	BP	0.469213761	0.184190107	-0.021150747	3.385565932	2.381445454	0.497097891	0.663327623	PREDICTED: putative lipoyltransferase 2, mitochondrial
RS003266	GO:0044699	single-organism process	BP	-0.36926559	-0.039795183	-0.401685153	5.103564049	2.37990733	0.497385826	0.663356676	PREDICTED: transient receptor potential channel pyrexia
RS004251	GO:0044699	single-organism process	BP	-0.025798497	-0.409224862	-0.408151338	3.246556755	2.374365749	0.498424268	0.664249231	PREDICTED: protein TEX261
RS012274	GO:0044699	single-organism process	BP	0.250868348	0.130831382	-0.091269758	5.86768545	2.371175161	0.499022911	0.66490632	PREDICTED: probable GDP-L-fucose synthase
RS011135	GO:0044699	single-organism process	BP	0.388131325	0.380813733	0.357031631	6.716192438	2.370025737	0.49923871	0.664982794	PREDICTED: exocyst complex component 8
RS012142	GO:0044699	single-organism process	BP	-0.227035903	-0.211326043	0.002070951	5.872416907	2.366057537	0.499984274	0.665694253	PREDICTED: rho GTPase-activating protein 44-like

RS010070	GO:0044699	single-organism process	BP	0.426102461	0.249569785	0.229186741	6.662299066	2.36347465	0.500470017	0.666130374	PREDICTED: DNA-directed RNA polymerase III subunit RPC4
RS008616	GO:0044699	single-organism process	BP	-0.370025045	0.099244548	-0.058320642	6.404208835	2.360771737	0.500978721	0.666454633	PREDICTED: nocturnin
RS013447	GO:0044699	single-organism process	BP	0.409495455	0.318927019	0.272173129	7.71635361	2.360181871	0.50108979	0.666531983	PREDICTED: cytoplasmic dynein 1 intermediate chain-like
RS003747	GO:0044699	single-organism process	BP	-0.363859986	-0.459320975	-0.333882132	3.597356509	2.357773902	0.501543394	0.666672784	PREDICTED: cone-rod homeobox protein
RS011195	GO:0044699	single-organism process	BP	-0.170691699	-0.271625919	-0.36664943	4.826980326	2.35841319	0.501422937	0.666672784	PREDICTED: serine/threonine-protein kinase 40
RS002616	GO:0044699	single-organism process	BP	-0.604425909	-0.227288728	-0.686758455	1.493498107	2.357805247	0.501537488	0.666672784	PREDICTED: gastrin-releasing peptide receptor-like
RS006346	GO:0044699	single-organism process	BP	0.186201906	-0.455433392	-0.048979366	2.812014352	2.355871741	0.501901939	0.666897886	PREDICTED: HMG box-containing protein 1
RS001414	GO:0044699	single-organism process	BP	-0.55594124	-0.392713779	-0.306676768	3.216845169	2.356121749	0.501854803	0.666897886	PREDICTED: sorting nexin-17
RS006811	GO:0044699	single-organism process	BP	-0.091072902	-0.032205255	0.237958262	6.439935586	2.353693428	0.502312778	0.667124828	PREDICTED: angio-associated migratory cell protein
RS005021	GO:0044699	single-organism process	BP	-0.06781788	-0.474899669	-0.350734329	3.155367473	2.352458366	0.50254583	0.667200314	PREDICTED: molybdate-anion transporter-like
RS011395	GO:0044699	single-organism process	BP	-0.578499552	-0.892720519	-0.795986283	1.325618075	2.350327789	0.502948057	0.667312245	PREDICTED: bone morphogenetic protein 3B
RS011022	GO:0044699	single-organism process	BP	0.198126699	0.09507206	-0.217276641	4.42516984	2.351418872	0.502742043	0.667312245	PREDICTED: diphosphoinositol polyphosphate phosphohydrolase 1
RS012376	GO:0044699	single-organism process	BP	-0.443120005	0.021687504	0.233976036	10.92588788	2.350839235	0.50285148	0.667312245	PREDICTED: phosphate carrier protein, mitochondrial
RS007200	GO:0044699	single-organism process	BP	0.073307321	-0.029382786	-0.363746794	6.43576106	2.351240248	0.502775765	0.667312245	PREDICTED: mannosyl-oligosaccharide glucosidase GCS1
RS001067	GO:0044699	single-organism process	BP	0.507736955	-0.043386115	-0.060869807	1.996213167	2.348289815	0.503333032	0.667484003	PREDICTED: sulfiredoxin-1
RS012598	GO:0044699	single-organism process	BP	-0.038030099	-0.161823372	-0.522614402	8.926773296	2.348756832	0.503244792	0.667484003	PREDICTED: titin-like
RS002859	GO:0044699	single-organism process	BP	0.316926002	0.369286141	0.159193529	6.339147897	2.340119633	0.504878649	0.668816841	PREDICTED: multiple inositol polyphosphate phosphatase 1
RS001322	GO:0044699	single-organism process	BP	0.073624118	0.258253815	0.407760776	4.899676072	2.337266058	0.505419336	0.66925151	PREDICTED: probable 28S ribosomal protein S23, mitochondrial
RS015179	GO:0044699	single-organism process	BP	0.333736139	0.333427341	0.163436813	4.70925399	2.331365628	0.50653873	0.670170057	PREDICTED: endonuclease V
RS009234	GO:0044699	single-organism process	BP	0.126988243	0.342457159	0.395519048	7.837797102	2.3304594	0.506710822	0.67032732	PREDICTED: putative RNA-binding protein Luc7-like 2
RS001507	GO:0044699	single-organism process	BP	0.129222299	0.009527171	0.648931001	3.409800651	2.326584629	0.507447138	0.671160392	PREDICTED: gustatory receptor for sugar taste 64f-like
RS001365	GO:0044699	single-organism process	BP	0.237278489	0.141297916	0.554160331	3.551638791	2.315424336	0.509572456	0.67338898	PREDICTED: glutamate receptor ionotropic, kainate 2
RS012298	GO:0044699	single-organism process	BP	0.306831729	0.211160455	0.107490212	6.095236613	2.312559577	0.510119096	0.673845175	myd88 protein
RS014502	GO:0044699	single-organism process	BP	-0.219647182	0.224994314	0.605975653	1.297553296	2.311846878	0.51025516	0.673954219	PREDICTED: small conductance calcium-activated potassium channel protein
RS007930	GO:0044699	single-organism process	BP	-0.061248022	0.433464271	0.198126107	2.703360678	2.310130945	0.510582866	0.674174943	PREDICTED: doublesex- and mab-3-related transcription factor 2
RS011967	GO:0044699	single-organism process	BP	-0.487444254	-0.165820874	-0.461490275	5.204715649	2.301911612	0.512154792	0.67540078	PREDICTED: flavin-containing monooxygenase FMO GS-OX-like 4
RS013131	GO:0044699	single-organism process	BP	0.056169741	-0.557814894	-0.295891595	2.318662367	2.294275324	0.51361849	0.676693301	PREDICTED: tubulin delta chain-like
RS009936	GO:0044699	single-organism process	BP	-0.405774511	-0.244025799	-0.380793029	5.042984997	2.288314551	0.514763225	0.67756355	PREDICTED: O-acetyl-ADP-ribose deacetylase 1
RS000079	GO:0044699	single-organism process	BP	-0.412658669	-0.332670739	-0.441767601	3.811839291	2.286255317	0.515159136	0.677730509	PREDICTED: CTD nuclear envelope phosphatase 1 homolog
RS011683	GO:0044699	single-organism process	BP	-0.264189218	-0.305236115	-0.007459881	5.107411445	2.284385591	0.515518811	0.67813285	PREDICTED: metabotropic glutamate receptor 2
RS004896	GO:0044699	single-organism process	BP	0.258725126	0.153243084	0.316020819	6.888961678	2.281927063	0.515992041	0.678432466	PREDICTED: ER degradation-enhancing alpha-mannosidase-like protein 3
RS012071	GO:0044699	single-organism process	BP	0.150655962	0.742763871	0.223400409	11.75709097	2.279137353	0.516529414	0.678611667	actin-4
RS011874	GO:0044699	single-organism process	BP	-0.595755719	0.321795189	-0.247478571	1.978968576	2.276081836	0.517118472	0.679173014	PREDICTED: knirps-related protein-like
RS005007	GO:0044699	single-organism process	BP	-0.059586696	0.152333746	-0.306054991	6.311528508	2.275400933	0.517249808	0.67927467	PREDICTED: thioredoxin-2
RS004583	GO:0044699	single-organism process	BP	0.326349741	0.164058096	-0.458342213	1.389904921	2.27262369	0.517785759	0.679836723	PREDICTED: neuropeptide FF receptor 2-like
RS005538	GO:0044699	single-organism process	BP	-0.01085029	0.184290766	0.249960669	6.267863964	2.272155956	0.517876063	0.679854115	PREDICTED: trans-1,2-dihydrobenzene-1,2-diol dehydrogenase-like
RS008712	GO:0044699	single-organism process	BP	-0.094748499	-0.364291756	-0.122624002	5.390572284	2.271854674	0.517934237	0.679854115	PREDICTED: traB domain-containing protein
RS009745	GO:0044699	single-organism process	BP	-0.628822026	-0.856847196	-0.715872376	0.753964619	2.271716323	0.517960953	0.679854115	PREDICTED: sphingomyelin phosphodiesterase-like
RS007305	GO:0044699	single-organism process	BP	0.349416491	0.2525404	0.172641186	6.448297777	2.269931701	0.518305655	0.680168555	PREDICTED: elongator complex protein 3
RS005763	GO:0044699	single-organism process	BP	-0.049864591	0.331988566	0.418799139	2.785560138	2.265395088	0.519182685	0.681106738	PREDICTED: TWiK family of potassium channels protein 7
RS000824	GO:0044699	single-organism process	BP	-0.657577232	-0.360235458	-1.178447729	0.499455064	2.262961041	0.5196537	0.681507726	PREDICTED: G-protein coupled receptor 64
RS009581	GO:0044699	single-organism process	BP	-0.082624917	-0.318869549	-0.362976424	4.40579628	2.261904579	0.519858236	0.681630794	PREDICTED: retinal rod rhodopsin-sensitive cGMP 3',5'-cyclic phosphodiesterase subunit delta
RS002215	GO:0044699	single-organism process	BP	-0.459893543	-0.373663745	-0.327616751	3.245153068	2.258109459	0.520593488	0.682313913	PREDICTED: CMP-sialic acid transporter 1
RS015050	GO:0044699	single-organism process	BP	-0.017041537	-0.193053418	-0.353464811	4.694919112	2.25775821	0.520661577	0.682332144	PREDICTED: putative GPI-anchor transamidase
RS012601	GO:0044699	single-organism process	BP	0.259922278	0.071030626	0.356475327	4.969754492	2.255145168	0.521168319	0.682854121	PREDICTED: RING finger protein 121
RS013061	GO:0044699	single-organism process	BP	0.055667037	0.39151864	0.322142146	3.784294836	2.253027649	0.521579236	0.683037215	PREDICTED: ubiquinone biosynthesis monooxygenase COQ6
RS008513	GO:0044699	single-organism process	BP	-0.025656425	-0.314395818	-0.176127512	5.41461175	2.251555276	0.521865101	0.683269476	PREDICTED: serine/threonine-protein kinase VRK1-like
RS005213	GO:0044699	single-organism process	BP	-0.168198261	-0.115865072	-0.354402757	5.076326521	2.249372679	0.522289074	0.68354033	PREDICTED: leucine-rich repeat serine/threonine-protein kinase 1
RS005150	GO:0044699	single-organism process	BP	1.108316077	0.167275937	-0.016216729	0.318216831	2.24954746	0.522255113	0.68354033	PREDICTED: homeobox protein OTX2-like
RS005067	GO:0044699	single-organism process	BP	-0.011472266	0.28377031	0.501312581	2.737680145	2.244794511	0.523179224	0.683779775	PREDICTED: N-acylneuraminate cytidylyltransferase
RS009984	GO:0044699	single-organism process	BP	-0.459044562	0.235336007	0.260152452	2.09044652	2.243088243	0.52351127	0.68398293	PREDICTED: transmembrane protein 47
RS007162	GO:0044699	single-organism process	BP	0.352804678	-0.213394026	-0.027296829	4.583000343	2.241629936	0.523795185	0.684160606	PREDICTED: dolichyl-diphosphooligosaccharide--protein glycosyltransferase subunit STT3A
RS004440	GO:0044699	single-organism process	BP	-0.006850436	0.369271357	0.390768265	3.839992898	2.235571801	0.524975863	0.685283913	PREDICTED: protein prickle-like
RS010188	GO:0044699	single-organism process	BP	0.086805335	-0.305670556	0.194209296	3.618005131	2.234682919	0.525149265	0.685383914	PREDICTED: Meckel syndrome type 1 protein
RS007873	GO:0044699	single-organism process	BP	0.352825721	0.238673302	0.160827331	6.81640946	2.231854824	0.525701249	0.685852151	PREDICTED: X-ray repair cross-complementing protein 5
RS014798	GO:0044699	single-organism process	BP	-0.301610744	-0.249820159	-0.529578201	3.17969966	2.231637207	0.525743741	0.685852151	PREDICTED: proton-coupled folate transporter-like
RS008765	GO:0044699	single-organism process	BP	-0.093493748	0.105458444	0.210619456	6.51494372	2.224185502	0.527200306	0.687467452	PREDICTED: myotubularin-related protein 3
RS006366	GO:0044699	single-organism process	BP	-0.14949043	-0.014558263	0.204413029	5.662962205	2.222493645	0.527531425	0.687649911	PREDICTED: cell division cycle protein 27 homolog
RS004221	GO:0044699	single-organism process	BP	-0.012306305	-0.374097254	0.006451511	4.348769073	2.220302402	0.52796051	0.687888957	PREDICTED: protein smoothened
RS014644	GO:0044699	single-organism process	BP	-0.185798477	-0.138679411	-0.737410043	1.872318804	2.21867774	0.528278815	0.688035933	PREDICTED: ras-related C3 botulinum toxin substrate 1-like
RS000677	GO:0044699	single-organism process	BP	-0.068097477	-0.368137342	-0.403982594	4.114617662	2.21923163	0.52817028	0.688035933	PREDICTED: ferrochelatase, mitochondrial
RS010270	GO:0044699	single-organism process	BP	-0.499816687	-0.179901857	-0.776722233	3.403430526	2.21634321	0.528736447	0.688401407	PREDICTED: protein N-terminal asparagine amidohydrolase
RS002128	GO:0044699	single-organism process	BP	0.24220864	0.390584804	0.530367508	2.888041968	2.20595072	0.530777216	0.690248912	PREDICTED: tyrosine-protein kinase Btk29A
RS009005	GO:0044699	single-organism process	BP	0.041714921	-0.201638008	-0.423306268	2.818304646	2.204831378	0.530997367	0.690248912	PREDICTED: glycoprotein hormone beta-5
RS007386	GO:0044699	single-organism process	BP	0.314086827	0.128584769	0.168612897	6.074658863	2.207367602	0.530498642	0.690248912	PREDICTED: hormone-sensitive lipase
RS010519	GO:0044699	single-organism process	BP	-0.037930597	-0.360925628	0.047413361	8.267918948	2.205787473	0.530809319	0.690248912	PREDICTED: phosphoglycerate mutase 1
RS011401	GO:0044699	single-organism process	BP	0.081549062	0.242069802	-0.257176864	3.111325918	2.201994704	0.531555584	0.690644007	PREDICTED: protein atonal homolog 8
RS012266	GO:0044699	single-organism process	BP	-0.472399722	-0.363524526	-0.351573067	4.356897101	2.197254643	0.532489323	0.691424331	PREDICTED: serine/threonine-protein phosphatase PP1-beta catalytic subunit
RS000284	GO:0044699	single-organism process	BP	-0.182180605	-0.326297855	-0.31886269	4.780780807	2.196047683	0.532727274	0.691524148	PREDICTED: protein lin-37 homolog
RS013249	GO:0044699	single-organism process	BP	-0.123210494	-0.264878471	-0.331783693	4.970226168	2.193604405	0.533209202	0.691864485	PREDICTED: interleukin-1 receptor-associated kinase 4-like
RS012835	GO:0044699	single-organism process	BP	-0.278764114	-0.378756734	-0.344077129	4.799600271	2.19192366	0.53354091	0.692056848	PREDICTED: protein XRP2
RS010646	GO:0044699	single-organism process	BP	-0.387797302	-0.153467445	-0.201059514	5.65127642	2.191739529	0.533577259	0.692056848	PREDICTED: probable phospholipid-transporting ATPase IA
RS012546	GO:0044699	single-organism process	BP	0.245310054	-0.049733462	-0.041489293	6.494753916	2.186370918	0.534637862	0.693075577	PREDICTED: WASH complex subunit strumpellin
RS011651	GO:0044699	single-organism process	BP	-0.351250373	-0.35252654	-0.287462621	4.967773394	2.18354716	0.535196334	0.693184484	PREDICTED: fatty acid synthase-like
RS015375	GO:0044699	single-organism process	BP	0.212267401	0.099848906	-0.115148087	5.42805291	2.181916206	0.535519093	0.693432804	Transcriptional regulator ATRX
RS002260	GO:0044699	single-organism process	BP	-0.844003423	-0.819264903	-0.423327512	1.50342137	2.175127996	0.536863981	0.694443515	PREDICTED: probable imidazolonepropionase
RS009383	GO:0044699	single-organism process	BP	0.391561702	0.048258756	0.267351301	5.560294073	2.17434817	0.537018639	0.694517636	PREDICTED: queuine tRNA-ribosyltransferase subunit QTRTD1
RS000792	GO:0044699	single-organism process	BP	0.263950133	0.071560722	0.276490197	5.557409031	2.170277281	0.537826523	0.695276866	PREDICTED: U4/U6.U5 small nuclear ribonucleoprotein 27 kDa protein
RS004078	GO:0044699	single-organism process	BP	-0.25730047	-0.277820059	-0.008138502	4.872635972	2.167174742	0.538442829	0.695645157	PREDICTED: telomerase reverse transcriptase
RS009174	GO:0044699	single-organism process	BP	-0.423002788	0.196548036	-0.205246354	4.127087177	2.166809446	0.538515428	0.695667586	PREDICTED: protein mothers against dpp
RS007897	GO:0044699	single-organism process	BP	0.062440003	-0.169160121	-0.302568052	5.867401431	2.164590839	0.538956504	0.696023196	PREDICTED: enoyl-CoA delta isomerase 1, mitochondrial-like
RS003783	GO:0044699	single-organism process	BP	0.35774402	-0.001104624	0.315382476	4.238118822	2.163108013	0.539251448	0.696261302	PREDICTED: sprT-like domain-containing protein Spartan
RS000590	GO:0044699	single-organism process	BP	0.152694733	-0.065582463	-0.43365666	3.203936127	2.16153955	0.539563554	0.696592865	PREDICTED: guanine nucleotide-binding protein G(i) subunit alpha
RS007159	GO:0044699	single-organism process	BP	0.090456011	-0.045162262	-0.528662069	1.960245612	2.161128556	0.539645359	0.696622187	PREDICTED: protein tramtrack, beta isoform
RS004277	GO:0044699	single-organism process	BP	-0.361527942	-0.072534399	-0.270080371	4.469820531	2.16072785	0.539725125	0.696622187	PREDICTED: disintegrin and metalloproteinase domain-containing protein 10
RS013116	GO:0044699	single-organism process	BP	-0.223367245	-0.449314941	-0.527136168	2.883999154	2.15911181	0.540046906	0.696931113	Suppressor of cytokine signaling 6
RS005865	GO:0044699	single-organism process	BP	-0.391290711	-0.580951392	-0.40868115	2.234548587	2.154665193	0.540933023	0.697535069	PREDICTED: syntaxin-6
RS002812	GO:0044699	single-organism process	BP	-0.695840769	-0.030688493	-0.3213982	1.581211751	2.154148644	0.541036029	0.697564266	PREDICTED: basic-leucine zipper transcription factor A
RS005332	GO:0044699	single-organism process	BP	0.03211789	-0.20554948	-0.243739032	6.261746697	2.15056386	0.541751269	0.698032794	PREDICTED: proline dehydrogenase 1, mitochondrial
RS012643	GO:0044699	single-organism process	BP	0.368633588	0.30532466	0.275081455	4.933664509	2.150175792	0.541828738	0.698032794	PREDICTED: INO80 complex subunit E
RS000118	GO:0044699	single-organism process	BP	-0.538237076	-0.248716882	-0.399559621	3.257659828	2.150813072	0.541701524	0.698032794	PREDICTED: protein melted
RS001108	GO:0044699	single-organism process	BP	-0.351307875	-0.364078375	-0.192697335	4.467218055	2.150105206	0.54184283	0.698032794	PREDICTED: short-chain specific acyl-CoA dehydrogenase, mitochondrial
RS000760	GO:0044699	single-organism process	BP	-0.373118902	-0.388261482	-0.671688535	2.590553751	2.144476326	0.542967433	0.699124	PREDICTED: inosine-5'-monophosphate dehydrogenase
RS007832	GO:0044699	single-organism process	BP	0.183402512	0.459331821	0.297289886	4.980169739	2.144553241	0.542952054	0.699124	PREDICTED: copper transport protein ATOX1
RS010759	GO:0044699	single-organism process	BP	0.088910614	0.234370013	0.342907191	6.066619154	2.142722424	0.543318193	0.69943262	PREDICTED: mitochondrial tRNA-specific 2-thiouridylase 1
RS013874	GO:0044699	single-organism process	BP	0.284914808	0.126074464	0.028714206	6.077549896	2.142330533	0.543396589	0.699439052	PREDICTED: peroxiredoxin-6
RS001004	GO:0044699	single-organism process	BP	0.351831481	0.250476663	0.218204399	5.115358526	2.13679893	0.544504037	0.699957522	PREDICTED: REM2- and Rab-like small GTPase 1
RS005581	GO:0044699	single-organism process	BP	0.372372642	0.180290678	-0.234718283	3.817007712	2.131792389	0.545507771	0.700818614	PREDICTED: ER lumen protein-retaining receptor
RS003167	GO:0044699	single-organism process	BP	0.231123427	0.300471952	0.30027721	5.892033491	2.131163175	0.545634013	0.700837814	PREDICTED: deoxyuridine 5'-triphosphate nucleotidohydrolase
RS014524	GO:0044699	single-organism process	BP	-0.161068997	-0.282848979	-0.333877227	5.633359602	2.126907819	0.546488338	0.701279359	PREDICTED: pre-mRNA-splicing factor CWC22 homolog
RS008915	GO:0044699	single-organism process	BP	-0.12485235	-0.438336721	-0.155700693	4.175396238	2.125742074	0.546722547	0.701449038	PREDICTED: A-kinase anchor protein 17A
RS000700	GO:0044699	single-organism process	BP	-0.325577143	-0.391074394	-0.272103698	7.157092092	2.124799381	0.546911995	0.70155141	PREDICTED: synaptic vesicle membrane protein VAT-1 homolog-like
RS007808	GO:0044699	single-organism process	BP	-0.305811494	-0.451112655	-0.188271036	8.749048076	2.12119125	0.54763754	0.702363066	PREDICTED: neuroendocrine protein 7B2
RS013219	GO:0044699	single-organism process	BP	0.286759539	0.315059003	0.077696978	8.095205709	2.121089372	0.547658036	0.702363066	PREDICTED: coatomer subunit beta'
RS012676	GO:0044699	single-organism process	BP	0.328234174	0.122797012	0.091228035	6.949367387	2.11972792	0.547931993	0.702547336	PREDICTED: conserved oligomeric Golgi complex subunit 4
RS001483	GO:0044699	single-organism process	BP	-0.159090076	-0.387245504	-0.302062286	4.993392435	2.11926654	0.548024856	0.702547336	PREDICTED: mothers against decapentaplegic homolog 4
RS001746	GO:0044699	single-organism process	BP	0.427924428	0.890866718	0.763147152	0.595497837	2.113941261	0.549097504	0.703707537	PREDICTED: forkhead box protein J3
RS006678	GO:0044699	single-organism process	BP	0.139354119	0.346144069	0.216283711	4.801909094	2.111453968	0.549599025	0.704135313	PREDICTED: calcium and integrin-binding protein 1-like
RS002929	GO:0044699	single-organism process	BP	-0.39492811	-0.310803863	-0.368352585	3.630774923	2.110337646	0.54982422	0.704208911	PREDICTED: acetylcholine receptor subunit alpha-like 1
RS006952	GO:0044699	single-organism process	BP	0.196623599	-0.003036232	-0.225453179	4.529529399	2.10907617	0.550078776	0.704320059	PREDICTED: AP-3 complex subunit sigma-2
RS006973	GO:0044699	single-organism process	BP	0.695576665	1.024137969	1.156284247	0.486288114	2.107958774	0.550304328	0.704394014	novel protein similar to DNA polymerases

RS001514	GO:0044699	single-organism process	BP	0.054174875	-0.249066955	0.029148299	6.065449686	2.108035373	0.550288864	0.704394014	PREDICTED: pantothenate kinase 4
RS015000	GO:0044699	single-organism process	BP	-0.383767528	-0.292514056	-0.346036053	3.582315118	2.09803705	0.552309984	0.706429786	PREDICTED: coiled-coil domain-containing protein 103
RS006817	GO:0044699	single-organism process	BP	0.215767809	0.330522088	0.120729712	5.269453245	2.097871442	0.552343505	0.706429786	PREDICTED: mitochondrial import inner membrane translocase subunit Tim21
RS009965	GO:0044699	single-organism process	BP	-0.364341407	-0.227420968	0.203520868	10.31015082	2.096719825	0.55257665	0.706584459	PREDICTED: malate dehydrogenase, mitochondrial
RS001595	GO:0044699	single-organism process	BP	-0.185258595	-0.184191027	0.184221317	3.898304226	2.093447138	0.553239589	0.707073207	PREDICTED: acyl-CoA Delta(11) desaturase
RS010044	GO:0044699	single-organism process	BP	-0.032740719	-0.492484393	-0.25544283	2.787037183	2.091316821	0.553671426	0.707481528	PREDICTED: solute carrier family 35 member C2
RS010577	GO:0044699	single-organism process	BP	0.095307992	-0.282992845	-0.522627351	2.176162312	2.090499603	0.553837148	0.707529581	PREDICTED: zinc finger MYND domain-containing protein 10
RS009552	GO:0044699	single-organism process	BP	-0.317174334	0.102956036	0.402195532	11.51695741	2.083221242	0.555314671	0.708862049	PREDICTED: ATP synthase subunit beta, mitochondrial
RS000520	GO:0044699	single-organism process	BP	-0.435291767	-0.212206011	-0.252475205	5.009676129	2.081646327	0.555634751	0.709198749	PREDICTED: RAC serine/threonine-protein kinase
RS010012	GO:0044699	single-organism process	BP	0.432969561	0.295211484	0.296888773	5.40665096	2.080106109	0.555947906	0.709526543	PREDICTED: probable low-specificity L-threonine aldolase 2
RS012614	GO:0044699	single-organism process	BP	-0.05396488	0.094282901	-0.213596456	6.025448718	2.078550311	0.556264355	0.70974586	PREDICTED: coiled-coil and C2 domain-containing protein 1-like
RS014317	GO:0044699	single-organism process	BP	0.159539053	0.240574537	0.297498962	6.660395876	2.068721716	0.558266444	0.711404189	PREDICTED: dehydrogenase/reductase SDR family protein 7-like
RS011585	GO:0044699	single-organism process	BP	0.074885187	0.199416499	0.564512656	2.528878013	2.065843457	0.558853711	0.711864431	PREDICTED: neuromedin-K receptor
RS008585	GO:0044699	single-organism process	BP	0.102088105	-0.133124434	-0.227228227	5.49512115	2.059668874	0.560115014	0.713110442	PREDICTED: cytoplasmic dynein 2 heavy chain 1
RS004653	GO:0044699	single-organism process	BP	-0.204326	-0.435783026	-0.52970613	2.188246229	2.056823883	0.560696846	0.713631606	PREDICTED: FERM domain-containing protein 4A
RS011534	GO:0044699	single-organism process	BP	-0.042855122	-0.37351784	0.198544715	4.461853423	2.047180727	0.562672139	0.714902558	PREDICTED: D-beta-hydroxybutyrate dehydrogenase, mitochondrial-like
RS004983	GO:0044699	single-organism process	BP	-0.251588885	-0.230618152	0.013157616	4.627906751	2.046973161	0.56271471	0.714902558	PRDM9 zinc finger domain protein
RS009198	GO:0044699	single-organism process	BP	0.246688789	-0.081204816	-0.064292575	5.356640501	2.043235233	0.563481736	0.71551613	PREDICTED: protein fantom
RS010099	GO:0044699	single-organism process	BP	0.196128807	0.291404844	0.296335058	7.662118239	2.036970563	0.564768893	0.716651044	PREDICTED: protein transport protein Sec24A
RS012569	GO:0044699	single-organism process	BP	0.131839839	-0.031650727	-0.243472933	4.510108976	2.030828549	0.566032843	0.717525679	PREDICTED: probable DNA replication complex GINS protein PSF2
RS002902	GO:0044699	single-organism process	BP	-0.415647108	-0.152738152	0.265175951	11.47381982	2.020686865	0.56812419	0.719524944	PREDICTED: ATP synthase subunit alpha, mitochondrial
RS008546	GO:0044699	single-organism process	BP	0.247324836	0.186193186	-0.033147877	6.689047743	2.020746085	0.568111962	0.719524944	PREDICTED: neuroblastoma-amplified sequence
RS007609	GO:0044699	single-organism process	BP	0.299959252	0.232454062	0.343283097	4.439499292	2.01965726	0.568336808	0.719721846	PREDICTED: serine/threonine-protein kinase 16
RS008702	GO:0044699	single-organism process	BP	-0.049560893	0.21376574	-0.016973749	6.107003009	2.016087618	0.569074383	0.719931971	PREDICTED: E3 ubiquitin-protein ligase TRIM23
RS008640	GO:0044699	single-organism process	BP	0.156563792	0.244356442	0.284513408	6.017118287	2.016428321	0.569003956	0.719931971	PREDICTED: transmembrane protein 115
RS000486	GO:0044699	single-organism process	BP	0.232389203	-0.011565709	-0.040844754	6.74387093	2.012991222	0.569714711	0.720350873	PREDICTED: apoptosis-inducing factor 1, mitochondrial
RS011393	GO:0044699	single-organism process	BP	-0.041730957	-0.230300536	0.07476639	6.804211163	2.01329809	0.569651229	0.720350873	PREDICTED: hydroxyacyl-coenzyme A dehydrogenase, mitochondrial-like
RS007793	GO:0044699	single-organism process	BP	0.384044636	0.154735346	0.08700257	7.659265624	2.011997642	0.569920287	0.720350873	PREDICTED: RNA polymerase-associated protein CTR9 homolog
RS007232	GO:0044699	single-organism process	BP	0.05852288	0.30721745	0.280289014	7.191739753	2.01304291	0.569704018	0.720350873	PREDICTED: casein kinase I isoform alpha
RS010798	GO:0044699	single-organism process	BP	0.021270494	0.09209859	-1.197682465	0.555972403	2.01223563	0.569871042	0.720350873	PREDICTED: zinc transporter ZIP11
RS001563	GO:0044699	single-organism process	BP	0.209916554	0.179625921	-0.058722842	5.49902195	2.010573736	0.570214989	0.720506429	PREDICTED: nucleoporin GLE1
RS002410	GO:0044699	single-organism process	BP	0.427440896	0.104952702	-0.036807537	3.940256848	2.006457354	0.571067539	0.720978167	PREDICTED: mitochondrial enolase superfamily member 1-like
RS009146	GO:0044699	single-organism process	BP	0.273209024	0.243574118	0.076497435	5.63647883	2.001763487	0.572040767	0.721944225	PREDICTED: breast cancer metastasis-suppressor 1-like protein
RS010987	GO:0044699	single-organism process	BP	0.029732183	0.51637298	0.136724477	2.61614082	2.001250298	0.572147241	0.722006248	PREDICTED: nicotinamide riboside kinase 1
RS015021	GO:0044699	single-organism process	BP	0.211893583	0.297159583	0.29750376	5.512348772	2.000151262	0.57237531	0.722153537	PREDICTED: neuralized-like protein 2
RS012746	GO:0044699	single-organism process	BP	-0.022595911	0.268824622	0.197727808	4.959043313	1.996263834	0.573182522	0.722713775	PREDICTED: liprin-alpha-1
RS005701	GO:0044699	single-organism process	BP	-0.391198295	-0.223595779	-0.856901858	0.773984583	1.995915581	0.573254874	0.722713775	PREDICTED: dopamine receptor 2
RS008058	GO:0044699	single-organism process	BP	-0.409713032	-0.274953713	-0.342896401	4.945538751	1.99375875	0.573703109	0.723100176	PREDICTED: guanine nucleotide-binding protein G(q) subunit alpha
RS012104	GO:0044699	single-organism process	BP	0.309980164	0.263293194	0.289793404	7.022205172	1.990253206	0.574432151	0.723746181	PREDICTED: DNA excision repair protein ERCC-6
RS012611	GO:0044699	single-organism process	BP	-0.045044208	-0.266187656	-0.496953526	3.594449833	1.990262588	0.574430199	0.723746181	PREDICTED: peroxisomal targeting signal 2 receptor
RS011000	GO:0044699	single-organism process	BP	0.311209564	-0.851695004	-0.061991307	0.663662117	1.990188972	0.574445515	0.723746181	PREDICTED: meiotic recombination protein DMC1/LIM15 homolog
RS003906	GO:0044699	single-organism process	BP	0.077939755	-0.212889937	-0.228799708	4.947105077	1.989438429	0.57460169	0.723798128	PREDICTED: WW domain-containing oxidoreductase
RS000513	GO:0044699	single-organism process	BP	0.166972537	0.295794213	0.246815675	6.542776388	1.980299961	0.576505573	0.725978521	PREDICTED: eukaryotic translation initiation factor 3 subunit F
RS011823	GO:0044699	single-organism process	BP	0.016033338	-0.16483323	-0.243264329	6.78294302	1.974666968	0.577681284	0.726761626	PREDICTED: histone-lysine N-methyltransferase eggless
RS003569	GO:0044699	single-organism process	BP	0.019446207	-0.182843453	-0.227529492	6.806978843	1.972641864	0.578104361	0.727046767	PREDICTED: nipped-B-like protein
RS006279	GO:0044699	single-organism process	BP	0.731956001	0.312619525	-0.157127015	1.511408191	1.970524751	0.578546886	0.727453039	PREDICTED: fatty acid synthase-like
RS008962	GO:0044699	single-organism process	BP	-0.044534925	-0.129293512	-0.286592954	5.622513162	1.967541314	0.579170884	0.727734024	PREDICTED: cyclin-G-associated kinase
RS012747	GO:0044699	single-organism process	BP	-0.713364102	0.286694361	0.251163895	0.771822647	1.959310148	0.58089484	0.729245376	PREDICTED: liprin-alpha-1
RS001956	GO:0044699	single-organism process	BP	0.077958348	-0.099402593	-0.22241688	5.819513808	1.957973342	0.581175153	0.729303142	PREDICTED: conserved oligomeric Golgi complex subunit 3
RS014730	GO:0044699	single-organism process	BP	-0.332871202	-0.302940951	-0.071966649	4.568536248	1.957689168	0.581234752	0.729303142	PREDICTED: ras-related protein Rab-24-like
RS004331	GO:0044699	single-organism process	BP	0.52533185	0.333475056	0.107304878	3.351388691	1.95543419	0.581707835	0.729404638	PREDICTED: guanine nucleotide-binding protein G(f) subunit alpha
RS000780	GO:0044699	single-organism process	BP	-0.151918465	-0.367027163	-0.082403445	7.305505482	1.949956894	0.582858029	0.730348366	PREDICTED: insulin receptor substrate 1
RS002943	GO:0044699	single-organism process	BP	-0.030438927	-0.1436328	-0.336559017	4.365840246	1.950399501	0.582765028	0.730348366	PREDICTED: methyl-CpG-binding domain protein 3
RS008534	GO:0044699	single-organism process	BP	0.478793271	0.306173793	0.418879374	3.414660537	1.949275894	0.583001142	0.730360726	PREDICTED: deoxynucleoside kinase
RS001124	GO:0044699	single-organism process	BP	0.040015909	0.174855229	0.304720951	5.441777148	1.945432468	0.583809285	0.731082245	PREDICTED: Golgi SNAP receptor complex member 1
RS005231	GO:0044699	single-organism process	BP	-0.391467446	-0.412384168	-0.179305009	4.071451755	1.941339648	0.584670698	0.731588315	PREDICTED: organic cation transporter protein
RS001474	GO:0044699	single-organism process	BP	0.194890329	0.338508393	-0.06376494	5.204773489	1.940752655	0.584794312	0.731588315	PREDICTED: homeobox protein homothorax
RS002150	GO:0044699	single-organism process	BP	0.002744289	0.108693157	-0.316967638	4.719449259	1.941248125	0.58468997	0.731588315	PREDICTED: intraflagellar transport protein 56
RS013708	GO:0044699	single-organism process	BP	-0.249101338	-0.4189226	-0.046087928	3.594256898	1.942033403	0.584524624	0.731588315	PREDICTED: ras-related protein Rap-2a
RS002129	GO:0044699	single-organism process	BP	0.474836474	0.860051892	0.944335717	1.159053463	1.939476566	0.585063103	0.731851879	PREDICTED: tyrosine-protein kinase Btk29A-like
RS010963	GO:0044699	single-organism process	BP	0.219826287	0.108252837	0.320948457	4.896240447	1.936757829	0.585636044	0.732350347	PREDICTED: protein virilizer
RS003845	GO:0044699	single-organism process	BP	0.105130982	-0.655648098	-0.636390592	0.795528085	1.931028835	0.586844596	0.733480672	PREDICTED: homeobox protein Nkx-6.1
RS009793	GO:0044699	single-organism process	BP	0.201805337	0.148978427	-0.108678135	5.304207448	1.923324278	0.588472532	0.734875862	PREDICTED: aprataxin and PNK-like factor
RS003278	GO:0044699	single-organism process	BP	-0.229798233	-0.363111867	-0.210643548	4.730975205	1.917947367	0.589610433	0.735931991	PREDICTED: malonyl-CoA decarboxylase, mitochondrial-like
RS015293	GO:0044699	single-organism process	BP	-0.750597248	-0.271746642	-0.413090169	1.165270449	1.915505839	0.590127611	0.736431543	PREDICTED: longitudinals lacking protein, isoforms A/B/D/L
RS002886	GO:0044699	single-organism process	BP	-0.091313315	-0.213541392	-0.292711592	5.35479834	1.915051386	0.590223909	0.736478739	PREDICTED: SLIT-ROBO Rho GTPase-activating protein 1-like
RS008914	GO:0044699	single-organism process	BP	-0.237701584	-0.530611571	0.561510058	0.60959975	1.913724094	0.590505221	0.736756761	PREDICTED: gonadotropin-releasing hormone receptor
RS014738	GO:0044699	single-organism process	BP	-0.165142767	-0.491585118	-0.17814123	3.320934373	1.912387175	0.590788663	0.737037386	PREDICTED: acidic phospholipase A2 PA4
RS008830	GO:0044699	single-organism process	BP	-0.136742747	-0.373540687	-0.697137823	2.022953767	1.911959752	0.5908793	0.737077447	PREDICTED: zygotic gap protein knirps-like
RS000732	GO:0044699	single-organism process	BP	-0.155711315	0.122158053	-0.262813147	4.114742654	1.910210735	0.591250286	0.737394147	PREDICTED: probable uridine-cytidine kinase
RS005170	GO:0044699	single-organism process	BP	0.622629572	-0.2007686	-0.31881309	0.328943855	1.907919441	0.591736529	0.737708359	PREDICTED: homeobox protein E60-like
RS013201	GO:0044699	single-organism process	BP	0.017552264	-0.289315883	-0.202265752	7.111279959	1.9075777	0.591809073	0.737725771	PREDICTED: probable phosphorylase b kinase regulatory subunit alpha
RS006603	GO:0044699	single-organism process	BP	0.458433756	-0.102217738	-0.12302605	2.407708913	1.898898397	0.593653487	0.739439424	PREDICTED: zinc finger protein 2 homolog
RS013326	GO:0044699	single-organism process	BP	-0.569421903	-0.210489639	-0.092201112	1.678462327	1.897566829	0.593936791	0.739645995	PREDICTED: probable G-protein coupled receptor 52
RS006599	GO:0044699	single-organism process	BP	-0.229353532	-0.166005786	0.057722818	7.486641568	1.896642027	0.594133604	0.739817936	PREDICTED: baculoviral IAP repeat-containing protein 6
RS001786	GO:0044699	single-organism process	BP	0.218253759	-0.126277735	0.52095378	1.939016679	1.894123567	0.594669791	0.7403392	PREDICTED: E3 ubiquitin-protein ligase SIAH1-like
RS013007	GO:0044699	single-organism process	BP	-0.002282553	0.125821076	-0.165712957	6.097798672	1.893235166	0.59485901	0.740400689	PREDICTED: exportin-4-like
RS015109	GO:0044699	single-organism process	BP	0.437383882	0.300982134	0.110886528	4.356407084	1.886434865	0.596308708	0.74186098	PREDICTED: opsin, ultraviolet-sensitive
RS005889	GO:0044699	single-organism process	BP	0.180854398	0.010978214	-0.100281595	6.088378416	1.885276905	0.596555795	0.741953751	PREDICTED: exocyst complex component 4
RS013005	GO:0044699	single-organism process	BP	0.358685922	0.036444866	-0.047275094	9.304453427	1.884981644	0.596618809	0.741958851	PREDICTED: superoxide dismutase [Cu-Zn]
RS009535	GO:0044699	single-organism process	BP	-0.371380403	-0.243526213	-0.109065039	6.875240596	1.879978368	0.597687259	0.742847463	PREDICTED: delta-1-pyrroline-5-carboxylate dehydrogenase, mitochondrial
RS003230	GO:0044699	single-organism process	BP	0.241270944	-0.072234236	-0.032023792	4.201954258	1.871554283	0.599489051	0.744572496	PREDICTED: IQ calmodulin-binding motif-containing protein 1
RS005517	GO:0044699	single-organism process	BP	-0.040617769	0.277956167	0.454102457	9.66049094	1.870199645	0.599779119	0.744859307	PREDICTED: ATP-dependent RNA helicase p62-like
RS014789	GO:0044699	single-organism process	BP	-0.384309417	-0.340794963	-0.701560358	2.91880799	1.866727447	0.600523037	0.745342183	PREDICTED: zinc transporter ZIP9
RS012954	GO:0044699	single-organism process	BP	-0.176644427	-0.317583234	-0.156924207	5.388438607	1.862359781	0.601459661	0.746080227	PREDICTED: neurocalcin homolog
RS007597	GO:0044699	single-organism process	BP	-0.446120938	-0.224236273	-0.438920293	3.37848861	1.861925411	0.601552862	0.746080227	PREDICTED: endonuclease III-like protein 1
RS013804	GO:0044699	single-organism process	BP	-0.121672386	0.019007874	0.170391643	6.253876544	1.859455709	0.602082949	0.746542548	PREDICTED: programmed cell death protein 10
RS010807	GO:0044699	single-organism process	BP	0.001269449	-0.509367404	-0.252962804	2.656086521	1.8581118	0.602371528	0.746826839	PREDICTED: matrix metalloproteinase-14
RS006631	GO:0044699	single-organism process	BP	0.417265301	0.366540752	0.352742367	8.674144117	1.857566742	0.602488595	0.746898451	PREDICTED: ATP-dependent RNA helicase WM6
RS000450	GO:0044699	single-organism process	BP	0.16069023	-0.44102839	-0.465031076	1.86959629	1.849290979	0.604267853	0.748335328	PREDICTED: odorant receptor 13a-like
RS002439	GO:0044699	single-organism process	BP	0.322813085	0.111104145	-0.161839796	6.005037147	1.846999235	0.604761168	0.748757585	PREDICTED: UDP-glucose 4-epimerase-like
RS007441	GO:0044699	single-organism process	BP	0.372211922	0.171604384	0.334018641	4.066166374	1.846453659	0.604878646	0.748829418	PREDICTED: DNA excision repair protein ERCC-1
RS000074	GO:0044699	single-organism process	BP	-0.127272604	-0.404669152	-0.102237074	3.987379332	1.845992434	0.604977972	0.748878767	PREDICTED: 5-hydroxytryptamine receptor
RS010664	GO:0044699	single-organism process	BP	0.206014271	-0.150395144	-0.048170963	5.065806217	1.844132908	0.605378532	0.749153702	PREDICTED: mRNA export factor
RS004421	GO:0044699	single-organism process	BP	0.237715808	-0.140769631	-0.073201592	4.162439897	1.840037471	0.60626133	0.749804104	PREDICTED: probable tyrosyl-DNA phosphodiesterase
RS001343	GO:0044699	single-organism process	BP	-0.182055731	-0.078317754	-1.122984871	0.25409944	1.840228913	0.606220045	0.749804104	PREDICTED: sodium/potassium/calcium exchanger 4
RS015335	GO:0044699	single-organism process	BP	-0.124297709	-0.419949503	-0.289750147	3.819367875	1.838300279	0.606636043	0.749899325	PREDICTED: transcription factor SOX-8
RS014266	GO:0044699	single-organism process	BP	-0.268154724	-0.478207871	-0.16661673	3.494657479	1.833486238	0.607675207	0.750668127	PREDICTED: protein gustavus
RS000563	GO:0044699	single-organism process	BP	0.315930573	0.28704086	0.276676793	5.982143714	1.832575182	0.607871997	0.750837576	PREDICTED: alkylated DNA repair protein alkB homolog 8
RS010526	GO:0044699	single-organism process	BP	0.048522394	0.243990412	0.337228358	8.570219697	1.83160295	0.608082046	0.750933698	PREDICTED: protein krasavietz
RS003806	GO:0044699	single-organism process	BP	0.24967023	0.596582329	0.028772767	2.167333516	1.827881633	0.608886457	0.751615857	PREDICTED: ribosomal protein S6 kinase beta-1-like
RS001141	GO:0044699	single-organism process	BP	0.003845601	-0.255771841	-0.248015311	4.913983338	1.827727222	0.60891985	0.751615857	PREDICTED: DNA polymerase lambda
RS001105	GO:0044699	single-organism process	BP	-0.219614449	-0.26220832	-0.327519535	7.371351073	1.827185164	0.609037083	0.751645402	PREDICTED: AP-1 complex subunit beta-1
RS003259	GO:0044699	single-organism process	BP	0.345067689	0.214790695	0.161470789	5.364845303	1.826626715	0.609157877	0.751688644	PREDICTED: tRNA-dihydrouridine(20) synthase [NAD(P)+]-like
RS010157	GO:0044699	single-organism process	BP	0.252451639	0.308689007	0.216067222	5.404322605	1.826234541	0.609242714	0.751719677	PREDICTED: exportin-T
RS009309	GO:0044699	single-organism process	BP	-0.23123993	-0.246980014	0.121570988	8.816929097	1.822999249	0.609942877	0.752288766	PREDICTED: iron-sulfur cluster assembly enzyme ISCU, mitochondrial
RS009317	GO:0044699	single-organism process	BP	-0.122121145	-0.087507899	-0.451264739	3.371075008	1.819120611	0.610782942	0.752790867	PREDICTED: protein sarah
RS005281	GO:0044699	single-organism process	BP	0.147692306	-0.427946659	0.130849224	2.285946009	1.819237088	0.610757704	0.752790867	PREDICTED: acid-sensing ion channel 4
RS002510	GO:0044699	single-organism process	BP	0.348650911	0.071659367	0.314644185	7.42752718	1.817730592	0.611084182	0.7530327	PREDICTED: Fanconi anemia group M protein
RS014616	GO:0044699	single-organism process	BP	0.250099502	0.079398829	-0.140121384	4.299112717	1.813412248	0.612020639	0.75381789	PREDICTED: serine/threonine-protein kinase PLK1
RS005621	GO:0044699	single-organism process	BP	-0.009813837	-0.316643841	-0.164836557	4.503621957	1.810659523	0.612618056	0.75440616	PREDICTED: protein argonaute-2

RS009180	GO:0044699	single-organism process	BP	0.332801217	0.12935444	0.191700052	6.497052643	1.808880891	0.613004264	0.754493035	Structure-specific endonuclease subunit SLX4
RS001590	GO:0044699	single-organism process	BP	0.800175461	0.243014294	0.361100751	1.206112949	1.808896988	0.613000768	0.754493035	AAA protein
RS009092	GO:0044699	single-organism process	BP	-0.184657287	-0.393843086	-0.11606951	4.040945019	1.806786702	0.613459188	0.754556599	PREDICTED: unconventional myosin-Ia
RS009860	GO:0044699	single-organism process	BP	0.162324623	0.32969451	0.216154039	5.027765659	1.806224902	0.613581265	0.754633052	PREDICTED: frataxin homolog, mitochondrial
RS014908	GO:0044699	single-organism process	BP	-0.182791767	-0.391383048	-0.263236776	4.097287057	1.80593089	0.613645159	0.754637939	Regulator of chromosome condensation
RS000733	GO:0044699	single-organism process	BP	-0.118924727	0.190147955	0.059240361	6.90602201	1.805271446	0.613788483	0.754666811	PREDICTED: protein transport protein Sec16B-like
RS010009	GO:0044699	single-organism process	BP	-0.028917549	0.125879721	0.232911505	6.177450618	1.796378041	0.615723432	0.756528868	PREDICTED: hydroxyacylglutathione hydrolase, mitochondrial
RS004342	GO:0044699	single-organism process	BP	-0.080876857	0.054160052	0.283602986	7.947576541	1.792680803	0.616528969	0.757223116	PREDICTED: paraplegin
RS001986	GO:0044699	single-organism process	BP	0.190706131	0.196633314	-0.073424922	5.311443205	1.789874802	0.617140767	0.757752835	PREDICTED: transcription factor grauzone-like
RS014916	GO:0044699	single-organism process	BP	0.230960206	0.267918017	0.093618919	5.622479388	1.788586718	0.617421738	0.757791045	PREDICTED: ubiquitin-conjugating enzyme E2 variant 2
RS004252	GO:0044699	single-organism process	BP	0.082186267	-0.171991664	-0.154928187	6.499291827	1.788352939	0.617472741	0.757791045	PREDICTED: exocyst complex component 6B
RS002973	GO:0044699	single-organism process	BP	0.129234058	0.595120427	-0.169312302	1.075854896	1.783305209	0.618574631	0.758625855	PREDICTED: ras-related C3 botulinum toxin substrate 1
RS003568	GO:0044699	single-organism process	BP	0.103992631	-0.335499443	-0.229845068	5.37382267	1.780400481	0.61920927	0.758982221	PREDICTED: cytosolic 10-formyltetrahydrofolate dehydrogenase
RS000223	GO:0044699	single-organism process	BP	0.257509082	0.106070944	0.293588131	6.716748584	1.780633001	0.619158453	0.758982221	PREDICTED: sphingomyelin phosphodiesterase 4
RS011599	GO:0044699	single-organism process	BP	-0.333977819	-0.372121193	-0.704602405	3.567133965	1.774616841	0.620474111	0.759919378	PREDICTED: dynein heavy chain 6, axonemal
RS008416	GO:0044699	single-organism process	BP	-0.193466134	-0.158001591	-0.375061899	6.588148689	1.772814265	0.620868649	0.760180805	PREDICTED: guanine nucleotide-binding protein G(I)/G(S)/G(T) subunit beta-1
RS010896	GO:0044699	single-organism process	BP	-0.330497398	-0.006471207	-0.302632139	3.723704066	1.772970128	0.620834528	0.760180805	PREDICTED: diacylglycerol kinase eta
RS005013	GO:0044699	single-organism process	BP	-0.104544829	-0.283209041	0.185378786	3.478361859	1.772364764	0.620967057	0.760227386	PREDICTED: tyrosine-protein kinase transmembrane receptor Ror
RS000691	GO:0044699	single-organism process	BP	0.144637227	-0.294490096	0.290386798	1.985497226	1.764506244	0.622689062	0.76196522	PREDICTED: pachytene checkpoint protein 2 homolog
RS009803	GO:0044699	single-organism process	BP	0.213911252	0.218935271	0.288323068	5.18116662	1.76239907	0.623151298	0.762368939	PREDICTED: stromal cell-derived factor 2
RS007596	GO:0044699	single-organism process	BP	-0.43536767	-0.490752696	-0.330090188	3.448549998	1.761913314	0.623257885	0.762368939	PREDICTED: fatty acid synthase-like
RS010249	GO:0044699	single-organism process	BP	0.351517878	-0.022497083	0.36598575	3.644847054	1.761898553	0.623261124	0.762368939	PREDICTED: type 1 phosphatidylinositol 4,5-bisphosphate 4-phosphatase
RS003940	GO:0044699	single-organism process	BP	0.355944096	0.015136174	0.161986665	5.007962335	1.758035998	0.624109063	0.762961517	PREDICTED: DEAD-box helicase Dbp80
RS008886	GO:0044699	single-organism process	BP	-0.289536803	-0.51024615	-0.479741606	2.079554922	1.757477457	0.624231737	0.763037416	PREDICTED: facilitated trehalose transporter Tret1
RS004905	GO:0044699	single-organism process	BP	-0.152478881	-0.125822337	0.089757805	5.66267141	1.756021667	0.624551546	0.763243955	PREDICTED: protein lin-7 homolog C
RS003681	GO:0044699	single-organism process	BP	0.085803928	-0.429040718	-0.172407369	2.598676023	1.749886099	0.625900512	0.764462367	PREDICTED: phosphatidylinositol N-acetylglucosaminyltransferase subunit C
RS014201	GO:0044699	single-organism process	BP	-0.421296402	-0.187472298	-0.591723695	1.959335562	1.745309148	0.626907956	0.765120535	PREDICTED: tubby-related protein 4
RS006120	GO:0044699	single-organism process	BP	0.282310094	0.26654938	0.010128256	4.191105626	1.744965367	0.626983667	0.765138788	PREDICTED: tRNA-dihydrouridine(47) synthase [NAD(P)(+)]-like
RS006376	GO:0044699	single-organism process	BP	0.036035271	-0.042639612	-0.239924224	5.347626836	1.735186466	0.62913958	0.767323636	PREDICTED: protein disulfide-isomerase TMX3
RS014301	GO:0044699	single-organism process	BP	0.040186155	-0.255484225	-0.528426906	1.945675602	1.733554783	0.629499746	0.767391327	PREDICTED: photoreceptor-specific nuclear receptor-like
RS007865	GO:0044699	single-organism process	BP	0.189614824	-0.107146276	0.466934837	3.905162157	1.731854836	0.629875113	0.767457844	PREDICTED: protein dachsous
RS008357	GO:0044699	single-organism process	BP	-0.295874931	-0.558240786	-0.403769987	2.274669311	1.730852433	0.630096517	0.767524474	PREDICTED: transcriptional enhancer factor TEF-1
RS011504	GO:0044699	single-organism process	BP	0.16917503	-0.151465524	0.162772761	4.241192785	1.722085218	0.63203496	0.769455221	PREDICTED: myeloid differentiation primary response protein MyD88
RS006113	GO:0044699	single-organism process	BP	-0.097796407	-0.153577541	-0.309295441	5.110127356	1.721473557	0.632170332	0.769455221	Armadillo type fold
RS001270	GO:0044699	single-organism process	BP	0.521684303	-0.020109254	0.42194779	1.619895984	1.720002688	0.632495935	0.769777136	PREDICTED: checkpoint protein HUS1
RS007048	GO:0044699	single-organism process	BP	-0.803309293	-0.099756871	-0.346898083	1.413242127	1.718790334	0.632764386	0.769955042	PREDICTED: dual 3',5'-cyclic-AMP and -GMP phosphodiesterase 11A
RS003731	GO:0044699	single-organism process	BP	0.302671865	0.481623589	0.352453939	2.364864784	1.716406033	0.63329254	0.770428736	PREDICTED: E3 ubiquitin-protein ligase parkin
RS012115	GO:0044699	single-organism process	BP	0.165628601	0.216441768	-0.027555978	5.450424984	1.715928392	0.633398375	0.770428736	PREDICTED: dual specificity mitogen-activated protein kinase kinase 6
RS014536	GO:0044699	single-organism process	BP	-0.137268948	-0.07750242	-0.358531119	4.210868086	1.71200184	0.634268817	0.771080465	PREDICTED: cyclic nucleotide-gated cation channel beta-3
RS002596	GO:0044699	single-organism process	BP	0.185252017	0.028392534	0.351365092	3.868060209	1.711535085	0.634372335	0.771080465	PREDICTED: cytidine deaminase-like
RS005423	GO:0044699	single-organism process	BP	-0.381627978	-0.344176933	-0.367676587	3.307053472	1.709787739	0.634759954	0.771265496	PREDICTED: histone-lysine N-methyltransferase Suv4-20
RS010358	GO:0044699	single-organism process	BP	0.306976969	0.175025435	-0.575621634	0.810862965	1.706529117	0.6354832	0.771892784	PREDICTED: neuroglobin-like
RS011067	GO:0044699	single-organism process	BP	0.025939029	0.198008277	0.271866084	7.216112793	1.705585924	0.635692632	0.772026326	PREDICTED: acetyl-CoA acetyltransferase, mitochondrial
RS008136	GO:0044699	single-organism process	BP	0.401812039	-0.12673825	-0.036704417	3.435602026	1.705041182	0.635813608	0.772098792	PREDICTED: protein patched
RS015538	GO:0044699	single-organism process	BP	0.225640179	-0.10699019	0.104179018	4.908035582	1.70244913	0.636389435	0.77242565	PREDICTED: chitobiosyldiphosphodolichol beta-mannosyltransferase
RS000553	GO:0044699	single-organism process	BP	0.64730182	0.862092501	0.056726366	0.305601663	1.700531981	0.63681553	0.772658973	PREDICTED: G1/S-specific cyclin-D2
RS013963	GO:0044699	single-organism process	BP	-0.123647317	-0.556084473	0.053667978	1.62151211	1.696404756	0.637733396	0.773136842	PREDICTED: calcitonin gene-related peptide type 1 receptor-like
RS003243	GO:0044699	single-organism process	BP	0.308946546	0.311732646	-0.170468752	1.692340436	1.694667413	0.638120002	0.773279771	Rho GTPase-activating protein 20
RS015410	GO:0044699	single-organism process	BP	-0.258061334	-0.246025988	-0.260658269	5.743258539	1.692882375	0.638517364	0.773443079	PREDICTED: protein slowmo
RS009891	GO:0044699	single-organism process	BP	-0.757696241	-0.494102871	-0.600706255	0.815486787	1.690663236	0.639011563	0.773818426	PREDICTED: atrial natriuretic peptide receptor 1
RS006985	GO:0044699	single-organism process	BP	0.006933679	0.269486901	-0.078587833	5.040871731	1.689258661	0.639324476	0.774122916	PREDICTED: probable 2-oxoglutarate dehydrogenase E1 component DHKTD1 homolog, mitochondrial
RS000768	GO:0044699	single-organism process	BP	0.270429843	0.24022112	0.220600039	5.318853214	1.687253729	0.639771292	0.774277204	PREDICTED: serine/threonine-protein phosphatase 6 catalytic subunit
RS004660	GO:0044699	single-organism process	BP	-0.375866955	-0.237419671	-0.141671824	3.773053923	1.687031734	0.639820776	0.774277204	PREDICTED: adenosine 3'-phospho 5'-phosphosulfate transporter 1
RS005648	GO:0044699	single-organism process	BP	0.422345316	0.329528901	0.151118532	3.905859633	1.684838589	0.640309768	0.774601232	PREDICTED: CD82 antigen-like
RS000783	GO:0044699	single-organism process	BP	-0.124790609	-0.354938505	-0.080239083	4.821761199	1.682545189	0.640821347	0.774931943	Beta-sarcoglycan
RS005988	GO:0044699	single-organism process	BP	-0.682720425	-0.216046027	-0.655597455	0.968663044	1.681276697	0.641104406	0.775160223	PREDICTED: whirlin
RS003921	GO:0044699	single-organism process	BP	0.221700722	0.079943729	-0.150862655	3.57985918	1.680094714	0.641368226	0.775200881	CG6175
RS012278	GO:0044699	single-organism process	BP	0.448431944	0.626519695	-0.013476653	2.933846509	1.679632149	0.641471488	0.775200881	PREDICTED: radical S-adenosyl methionine domain-containing protein 2
RS002117	GO:0044699	single-organism process	BP	0.21920613	0.287876127	0.152581754	5.189853985	1.673232271	0.642901179	0.775918424	PREDICTED: ADP-ribosylation factor-like protein 2-binding protein
RS005198	GO:0044699	single-organism process	BP	-0.039434026	-0.322075231	-0.254045164	5.027143615	1.671960987	0.643185395	0.776111609	PREDICTED: intraflagellar transport protein 88 homolog
RS005122	GO:0044699	single-organism process	BP	-0.335937031	-0.276756834	-0.328451579	4.001594434	1.667864735	0.644101672	0.776845092	PREDICTED: GPI ethanolamine phosphate transferase 1
RS006928	GO:0044699	single-organism process	BP	-0.363135133	-0.220144308	0.11249691	2.701409449	1.667496458	0.644184087	0.776870094	PREDICTED: hippocampus abundant transcript 1 protein
RS007059	GO:0044699	single-organism process	BP	0.403340577	-0.250644084	0.412306049	1.578648447	1.664819496	0.644783338	0.777443883	PREDICTED: PI-PLC X domain-containing protein 1-like
RS007484	GO:0044699	single-organism process	BP	-0.117372652	-0.36503658	-0.439316499	2.808678182	1.663521482	0.64507402	0.777571039	PREDICTED: geranylgeranyl pyrophosphate synthase
RS012536	GO:0044699	single-organism process	BP	0.047139577	-0.258366711	-0.192122824	5.403598014	1.660854375	0.645671538	0.77814233	PREDICTED: histone deacetylase Rpd3
RS009498	GO:0044699	single-organism process	BP	0.136698246	-0.219322505	0.000773036	5.008025386	1.660051847	0.645851393	0.778284608	PREDICTED: coiled-coil domain-containing protein 39
RS000266	GO:0044699	single-organism process	BP	0.151837872	0.279762783	0.07684077	6.684593801	1.655430255	0.646887694	0.779011629	PREDICTED: DEP domain-containing protein 5
RS012406	GO:0044699	single-organism process	BP	-0.118916496	-0.22943197	0.145617021	8.722210769	1.650202623	0.648061031	0.779972717	PREDICTED: probable phospholipid-transporting ATPase IIB
RS001269	GO:0044699	single-organism process	BP	0.099230055	-0.152014882	0.551858094	1.166065908	1.649373225	0.6482473	0.780052216	PREDICTED: checkpoint protein HUS1
RS015165	GO:0044699	single-organism process	BP	0.203835116	0.086955701	-0.092770826	6.68983845	1.645463293	0.649125814	0.780587272	PREDICTED: sodium channel protein 60E
RS003602	GO:0044699	single-organism process	BP	-0.963739329	-0.484991816	-0.758608967	0.476696176	1.634124817	0.651677236	0.782683875	PREDICTED: solute carrier family 28 member 3
RS010127	GO:0044699	single-organism process	BP	0.170429473	0.30068794	0.135511369	5.722930489	1.631571734	0.652252516	0.782777605	PREDICTED: probable glutamine--tRNA ligase
RS015064	GO:0044699	single-organism process	BP	-0.234416859	-0.485764927	-0.405888112	6.699048012	1.631993179	0.652157533	0.782777605	PREDICTED: beta-hexosaminidase subunit beta-like
RS007623	GO:0044699	single-organism process	BP	-0.043985273	0.172463342	0.184999253	6.287744401	1.629636466	0.652688775	0.782965625	PREDICTED: ER membrane protein complex subunit 4
RS003435	GO:0044699	single-organism process	BP	-0.265036011	-0.457016415	-0.330200465	4.234282109	1.62949783	0.652720033	0.782965625	PREDICTED: regulator of G-protein signaling 9
RS010829	GO:0044699	single-organism process	BP	-0.088922983	-0.143734243	0.205870504	5.245264757	1.629734927	0.652666576	0.782965625	PREDICTED: isoleucine--tRNA ligase, cytoplasmic
RS007589	GO:0044699	single-organism process	BP	-0.435229241	-0.37419876	0.172373107	1.34129675	1.61905116	0.655077853	0.784822154	PREDICTED: fork head domain transcription factor slp2-like
RS001545	GO:0044699	single-organism process	BP	0.256979278	0.132354415	0.05596066	6.254781443	1.611421238	0.656802912	0.786237773	PREDICTED: protection of telomeres protein 1
RS006057	GO:0044699	single-organism process	BP	0.242586341	0.333532639	0.095589819	8.338996539	1.611064002	0.656883742	0.786237773	PREDICTED: 26S protease regulatory subunit 7
RS013111	GO:0044699	single-organism process	BP	0.243734991	-0.003665678	0.068325846	6.590986784	1.610092577	0.657103567	0.786351418	PREDICTED: ras-related protein Rab-10
RS010427	GO:0044699	single-organism process	BP	-0.256640259	-0.293251267	-0.831731644	1.070368751	1.607645315	0.657657541	0.78679007	PREDICTED: glutamate receptor ionotropic, NMDA 2B
RS007101	GO:0044699	single-organism process	BP	0.302041092	0.019148607	0.249776245	3.7895307	1.606596267	0.657895087	0.786999499	FAD-linked oxidoreductase
RS001560	GO:0044699	single-organism process	BP	-0.177702139	-0.072258119	-0.309350954	7.149968374	1.601750288	0.658993019	0.787657085	PREDICTED: lissencephaly-1 homolog
RS014486	GO:0044699	single-organism process	BP	-0.345895533	-0.296211489	-0.130080409	3.378564789	1.595537379	0.660402112	0.788575348	PREDICTED: type I inositol 1,4,5-trisphosphate 5-phosphatase
RS014553	GO:0044699	single-organism process	BP	-0.735733025	0.024315289	-0.582972029	0.379469694	1.581739377	0.663537345	0.791044048	PREDICTED: fatty acid synthase-like
RS013558	GO:0044699	single-organism process	BP	-0.082986615	-0.139743726	-0.318419282	4.570668193	1.579865284	0.6639638	0.791402621	PREDICTED: phosphatidylserine synthase 1
RS012399	GO:0044699	single-organism process	BP	0.27036231	0.136969162	0.049276245	5.890506614	1.579065686	0.664145795	0.791469733	PREDICTED: probable splicing factor 3B subunit 5
RS000745	GO:0044699	single-organism process	BP	0.349751231	0.222717717	0.146141045	4.165636942	1.578118797	0.66436135	0.791540107	PREDICTED: DNA repair protein XRCC2
RS009537	GO:0044699	single-organism process	BP	-0.2081333	-0.164903777	0.057137416	5.354589713	1.576868679	0.66464599	0.791616379	PREDICTED: prostatic acid phosphatase-like
RS002544	GO:0044699	single-organism process	BP	0.191643692	-0.092357281	-0.10550711	4.149457625	1.576422288	0.664747645	0.791662585	PREDICTED: DNA-directed RNA polymerase II subunit RPB9
RS005554	GO:0044699	single-organism process	BP	-0.377665086	-0.114327538	-0.112630181	3.603732064	1.573723285	0.665362455	0.791987138	PREDICTED: glycerophosphocholine phosphodiesterase GPCPD1-like
RS005336	GO:0044699	single-organism process	BP	0.446715966	-0.092505303	0.171556857	2.747618541	1.574279874	0.665235644	0.791987138	PREDICTED: coiled-coil domain-containing protein 63
RS011960	GO:0044699	single-organism process	BP	0.158138847	0.38416202	-0.39100016	0.830892015	1.571089141	0.665962782	0.792293049	PREDICTED: MOXD1 homolog 2
RS010986	GO:0044699	single-organism process	BP	0.253721879	0.185004267	0.228015552	5.911597412	1.570101178	0.666188016	0.792478688	PREDICTED: COP9 signalosome complex subunit 3
RS002730	GO:0044699	single-organism process	BP	0.273731849	0.243679505	0.179335078	5.847607944	1.567473925	0.666787166	0.792742148	PREDICTED: tRNA-dihydrouridine(20a/20b) synthase [NAD(P)+]-like
RS004292	GO:0044699	single-organism process	BP	0.329201153	0.103869218	0.213950703	8.299117956	1.560734675	0.668325369	0.793682461	PREDICTED: T-complex protein 1 subunit epsilon
RS005010	GO:0044699	single-organism process	BP	-0.284832376	-0.446843104	-0.370459198	2.721108227	1.559929183	0.668509343	0.793714758	PREDICTED: asparagine synthetase domain-containing protein 1
RS013397	GO:0044699	single-organism process	BP	-0.201757507	-0.230383602	-0.008206272	6.648735594	1.554620765	0.669722452	0.79473142	PREDICTED: delta(3,5)-Delta(2,4)-dienoyl-CoA isomerase, mitochondrial
RS011647	GO:0044699	single-organism process	BP	-0.265493546	-0.458726124	-0.343198876	2.618456764	1.552213011	0.670273065	0.795160039	PREDICTED: insulin-like growth factor-binding protein complex acid labile subunit
RS002384	GO:0044699	single-organism process	BP	-0.214338327	-0.292600051	-0.19835915	6.014577236	1.551214898	0.670501385	0.79535598	PREDICTED: cold shock domain-containing protein CG9705
RS005045	GO:0044699	single-organism process	BP	0.21270016	0.223935052	0.00629873	5.48428509	1.549262736	0.670948064	0.795735937	PREDICTED: zinc finger protein 271-like
RS009168	GO:0044699	single-organism process	BP	0.010370353	-0.180214798	-0.244163034	5.228808153	1.546856649	0.671498819	0.796010584	PREDICTED: KIF1-binding protein homolog
RS011146	GO:0044699	single-organism process	BP	0.047436805	-0.146169888	-0.197023734	6.983925612	1.546594349	0.671558873	0.796010584	PREDICTED: phosphoinositide 3-kinase regulatory subunit 4
RS013617	GO:0044699	single-organism process	BP	0.186548808	0.116425849	0.271354635	5.471792039	1.546182325	0.671653214	0.796047485	PREDICTED: CCR4-NOT transcription complex subunit 11
RS012989	GO:0044699	single-organism process	BP	0.065692611	0.13851955	0.297427673	4.628786845	1.545719967	0.671759087	0.796098047	PREDICTED: snurportin-1
RS015471	GO:0044699	single-organism process	BP	0.133117621	-0.560311077	-0.541944047	0.858512045	1.544871634	0.671953366	0.796253358	PREDICTED: protein zwilch homolog
RS011517	GO:0044699	single-organism process	BP	-0.425201269	-0.694105233	-0.809419044	0.768896127	1.538649762	0.673379139	0.79748469	PREDICTED: patronin
RS004731	GO:0044699	single-organism process	BP	-0.229574689	-0.122344617	-0.277681282	5.517826795	1.535656733	0.674065561	0.797930414	PREDICTED: 1-phosphatidylinositol 3-phosphate 5-kinase
RS010826	GO:0044699	single-organism process	BP	0.138208531	-0.101859985	-0.15150157	5.915468023	1.53076441	0.675188338	0.798809012	PREDICTED: nuclear cap-binding protein subunit 1
RS005129	GO:0044699	single-organism process	BP	-0.329505756	-0.578443302	-0.349527898	2.358715219	1.526347183	0.6762029	0.799634314	PREDICTED: PQ-loop repeat-containing protein 3
RS012038	GO:0044699	single-organism process	BP	-0.298498954	-0.429697941	-0.08853967	2.876862474	1.523032867	0.67696465	0.800220491	PREDICTED: guanylate cyclase 32E

RS014702	GO:0044699	single-organism process	BP	0.048428453	-0.238349601	-0.12992294	6.529352259	1.523281632	0.67690746	0.800220491	PREDICTED: glutaryl-CoA dehydrogenase, mitochondrial
RS003360	GO:0044699	single-organism process	BP	-0.014456754	-0.152106383	-0.250776241	4.736201145	1.519502809	0.677776462	0.800421353	PREDICTED: diphthine synthase
RS000107	GO:0044699	single-organism process	BP	0.215541555	0.120252028	-0.217617098	3.595365183	1.517996661	0.678122983	0.800549125	PREDICTED: multidrug resistance protein 1A
RS010274	GO:0044699	single-organism process	BP	-0.328476294	0.178673468	-0.060831732	2.941831503	1.517530124	0.678230337	0.800549125	PREDICTED: GTP-binding protein Di-Ras2
RS008748	GO:0044699	single-organism process	BP	0.362941432	0.111884559	0.143279106	4.512203371	1.504356184	0.681265291	0.802906146	PREDICTED: phosphatidate cytidylyltransferase, mitochondrial
RS013875	GO:0044699	single-organism process	BP	0.237848383	0.485622911	0.188044452	3.133678997	1.50277884	0.681629124	0.803109476	PREDICTED: actin-related protein 2/3 complex subunit 5
RS002189	GO:0044699	single-organism process	BP	0.053141311	0.253737742	0.050076175	5.618416132	1.501775601	0.681860583	0.803193487	PREDICTED: leucine-rich repeat-containing protein C10orf11 homolog
RS012079	GO:0044699	single-organism process	BP	0.239219771	0.02432342	0.187017072	5.848565127	1.497472914	0.682853703	0.803950597	PREDICTED: pyridoxal-dependent decarboxylase domain-containing protein 1
RS007072	GO:0044699	single-organism process	BP	0.057769024	0.288369246	0.156218878	6.314877502	1.494618764	0.683512873	0.804425859	PREDICTED: U4/U6 small nuclear ribonucleoprotein Prp3
RS005179	GO:0044699	single-organism process	BP	0.105882197	0.22624227	-0.024420666	5.410537537	1.490831892	0.684387936	0.80530521	PREDICTED: protein prune homolog 2
RS009872	GO:0044699	single-organism process	BP	-0.245142475	-0.255602656	-0.247812004	4.543401333	1.489615581	0.684669115	0.805485552	PREDICTED: segment polarity protein dishevelled homolog DVL-3
RS002299	GO:0044699	single-organism process	BP	0.474202893	0.476796195	0.477543891	2.345993252	1.488127273	0.685013248	0.805739876	PREDICTED: ferritin, lower subunit
RS001875	GO:0044699	single-organism process	BP	0.161509628	-0.228939888	-0.193566399	3.958788455	1.486598784	0.68536676	0.805966114	PREDICTED: pyridoxal kinase
RS007177	GO:0044699	single-organism process	BP	0.062205783	-0.161256518	-0.189988088	4.691464586	1.484922633	0.685754525	0.806084795	PREDICTED: baculoviral IAP repeat-containing protein 6-like
RS001046	GO:0044699	single-organism process	BP	-0.274727443	-0.262613671	-0.086781178	6.540789003	1.480175107	0.686853406	0.806933497	PREDICTED: probable phosphorylase b kinase regulatory subunit beta
RS014157	GO:0044699	single-organism process	BP	-0.542553385	-0.255540081	-0.17193539	2.124694822	1.477157184	0.687552386	0.807433396	PREDICTED: tyrosine-protein phosphatase non-receptor type 14
RS004131	GO:0044699	single-organism process	BP	0.244077702	0.428184836	0.166004547	3.05086288	1.47403705	0.688275398	0.807916894	PREDICTED: synaptic vesicle 2-related protein
RS003723	GO:0044699	single-organism process	BP	-0.004420393	0.320740261	-0.251711753	2.646606579	1.469163281	0.689405495	0.808866481	PREDICTED: growth arrest-specific protein 8
RS002803	GO:0044699	single-organism process	BP	0.343367226	0.291007198	0.436673754	9.264575701	1.464726265	0.690435087	0.809286647	PREDICTED: splicing factor 3B subunit 1
RS006130	GO:0044699	single-organism process	BP	-0.262914657	-0.459395724	-0.515401475	3.28664966	1.466187192	0.690096004	0.809286647	PREDICTED: transcription factor stalky
RS001204	GO:0044699	single-organism process	BP	-0.193538244	-0.033752226	-0.200100469	5.895767362	1.466039539	0.690130271	0.809286647	PREDICTED: phosphatidylinositol glycan anchor biosynthesis class U protein
RS014210	GO:0044699	single-organism process	BP	-0.099864709	-0.261063694	-0.008158269	5.601094807	1.464186895	0.690560295	0.809316615	PREDICTED: importin subunit alpha-7
RS012275	GO:0044699	single-organism process	BP	-0.212121307	-0.202343571	0.047777621	6.588625972	1.463343167	0.690756177	0.809470857	PREDICTED: sn1-specific diacylglycerol lipase alpha
RS001715	GO:0044699	single-organism process	BP	-0.10748083	0.311329715	0.140851688	3.35289137	1.461276326	0.691236132	0.809957931	PREDICTED: longitudinals lacking protein-like
RS004390	GO:0044699	single-organism process	BP	-0.099480159	0.217151829	0.0017329	4.396736241	1.459502115	0.691648258	0.810230171	PREDICTED: MOB kinase activator-like 4
RS015243	GO:0044699	single-organism process	BP	-0.688661163	-0.086472172	-0.520258795	0.446087205	1.457617986	0.692086043	0.81050136	PREDICTED: longitudinals lacking protein, isoforms A/B/D/L-like
RS003990	GO:0044699	single-organism process	BP	-0.055368209	0.238070575	0.293320892	4.25914611	1.454595738	0.692788544	0.811066898	PREDICTED: UDP-galactose translocator
RS009185	GO:0044699	single-organism process	BP	0.014907563	-0.22840943	-0.289226944	4.455742642	1.4529144	0.693179504	0.811178468	PREDICTED: glia maturation factor beta
RS013572	GO:0044699	single-organism process	BP	-0.046510172	-0.01028718	-0.267362163	5.187624113	1.448524287	0.694200814	0.811920974	PREDICTED: protein FAM50 homolog
RS011273	GO:0044699	single-organism process	BP	0.225938591	-0.009214176	0.213519481	4.443074423	1.44680938	0.694599956	0.812312364	PREDICTED: E3 ubiquitin-protein ligase Siah1-like
RS009283	GO:0044699	single-organism process	BP	-0.139541385	-0.36672288	-0.08881878	4.012094905	1.439453864	0.69631314	0.813484943	PREDICTED: dephospho-CoA kinase domain-containing protein
RS002826	GO:0044699	single-organism process	BP	0.250919424	0.008700772	-0.045827627	4.112833325	1.438213941	0.696602122	0.813671594	PREDICTED: cyclin-dependent kinase 8
RS001756	GO:0044699	single-organism process	BP	0.196282527	-0.01818291	-0.032103809	6.702691645	1.437540698	0.696759054	0.813779424	PREDICTED: DNA excision repair protein haywire
RS009066	GO:0044699	single-organism process	BP	-0.284333504	-0.421360118	-0.423891407	2.706723454	1.434128224	0.697554745	0.814482149	PREDICTED: solute carrier family 35 member E3-like
RS013170	GO:0044699	single-organism process	BP	-0.32607493	-0.406051581	-0.605910078	1.623065456	1.429779426	0.698569354	0.815288893	PREDICTED: probable sodium/potassium/calcium exchanger CG1090
RS003678	GO:0044699	single-organism process	BP	-0.334465949	-0.114289915	-0.203522905	4.076361414	1.425503068	0.69956771	0.815865352	PREDICTED: tRNA-dihydrouridine(16/17) synthase [NAD(P)(+)]-like
RS009867	GO:0044699	single-organism process	BP	0.177380013	-0.109506066	-0.054245699	6.130179775	1.426200875	0.699404757	0.815865352	PREDICTED: S1 RNA-binding domain-containing protein 1
RS002953	GO:0044699	single-organism process	BP	-0.005347516	-0.199511217	-0.214304267	5.127054414	1.42336328	0.700067503	0.816280912	PREDICTED: Bloom syndrome protein homolog
RS007066	GO:0044699	single-organism process	BP	-0.267884143	-0.3289022	-0.200858946	4.312380835	1.417467274	0.701445461	0.817603063	PREDICTED: single-strand selective monofunctional uracil DNA glycosylase
RS013983	GO:0044699	single-organism process	BP	0.174688496	-0.087823924	-0.199199758	3.791091714	1.417139914	0.701522004	0.817603063	PREDICTED: valine--tRNA ligase-like
RS015498	GO:0044699	single-organism process	BP	0.184679301	0.472371696	0.26432428	2.413896137	1.414695214	0.702093736	0.818113296	PREDICTED: calcium uniporter protein, mitochondrial
RS010347	GO:0044699	single-organism process	BP	-0.186689215	0.108500444	0.151129076	3.585218724	1.414002621	0.702255747	0.81816069	Cyclic nucleotide-gated cation channel alpha-3
RS014968	GO:0044699	single-organism process	BP	0.070753676	0.338611082	0.008973976	4.167384444	1.401868016	0.705096916	0.82085326	PREDICTED: solute carrier family 41 member 1-like
RS014710	GO:0044699	single-organism process	BP	0.13791826	0.280437844	0.246818414	7.961653377	1.394222752	0.706889507	0.822234092	PREDICTED: coatomer subunit alpha
RS013905	GO:0044699	single-organism process	BP	-0.194636933	-0.034295338	-0.183390318	6.587442494	1.394206188	0.706893393	0.822234092	PREDICTED: exocyst complex component 3
RS011446	GO:0044699	single-organism process	BP	-0.226602437	0.017489836	-0.173735734	4.50551615	1.392641248	0.707260567	0.822459795	PREDICTED: stress-activated protein kinase JNK
RS011487	GO:0044699	single-organism process	BP	0.307555722	0.055105083	-0.182513134	2.933490158	1.379075172	0.710446874	0.825195632	PREDICTED: DNA repair protein RAD51 homolog 1
RS007613	GO:0044699	single-organism process	BP	-0.178822031	-0.166838759	-0.615818192	1.442423618	1.377955684	0.710710079	0.825264136	PREDICTED: carotenoid isomerooxygenase
RS008330	GO:0044699	single-organism process	BP	-0.130690066	-0.089214412	-0.33095204	4.021503763	1.372516643	0.711989437	0.826605506	PREDICTED: dimethyladenosine transferase 2, mitochondrial
RS006365	GO:0044699	single-organism process	BP	0.255219257	-0.131076203	0.089534139	3.395779717	1.371431999	0.712244678	0.826805187	PREDICTED: renalase
RS004594	GO:0044699	single-organism process	BP	0.239340739	0.0549206	0.159140698	5.376544784	1.370887354	0.712372859	0.826835397	PREDICTED: trafficking protein particle complex subunit 3
RS004705	GO:0044699	single-organism process	BP	-0.430701847	-0.465359916	-0.484712083	3.064343052	1.370281204	0.712515526	0.826890945	PREDICTED: sodium/hydrogen exchanger 7
RS001777	GO:0044699	single-organism process	BP	0.12387802	0.116824063	0.320015937	4.772677013	1.368884078	0.712844407	0.827120183	PREDICTED: serine/threonine-protein kinase RIO1
RS005130	GO:0044699	single-organism process	BP	-0.120932052	-0.228887603	-0.254860555	4.886019147	1.367159764	0.713250393	0.827286374	PREDICTED: major facilitator superfamily domain-containing protein 6
RS008713	GO:0044699	single-organism process	BP	0.27828907	0.19697421	0.275456017	6.153632425	1.365920619	0.713542205	0.827548626	PREDICTED: histone-lysine N-methyltransferase SETMAR
RS002758	GO:0044699	single-organism process	BP	0.23530849	0.11520272	-0.020421955	5.252459564	1.361413404	0.714604037	0.828246203	PREDICTED: thioredoxin domain-containing protein 15
RS001231	GO:0044699	single-organism process	BP	0.326472133	0.01191445	0.006751878	4.576542697	1.358277243	0.715343245	0.828874121	PREDICTED: cleft lip and palate transmembrane protein 1-like protein
RS001366	GO:0044699	single-organism process	BP	-0.202685308	-0.234557446	-0.316543451	6.418554265	1.353303228	0.71651627	0.829698961	PREDICTED: glutamate receptor ionotropic, kainate 2
RS010673	GO:0044699	single-organism process	BP	0.194686816	0.144745443	0.237663354	5.4673327	1.348021968	0.717762586	0.830648431	PREDICTED: zinc transporter foi-like
RS003740	GO:0044699	single-organism process	BP	0.188038637	0.141769883	0.335411585	4.728973031	1.347872335	0.71779791	0.830648431	PREDICTED: nucleoside diphosphate kinase 6
RS008862	GO:0044699	single-organism process	BP	0.540100521	0.133587909	0.205179113	1.360982431	1.345269174	0.718412551	0.831181996	PREDICTED: bursicon
RS014513	GO:0044699	single-organism process	BP	0.245831721	0.239105658	0.17414925	5.204247819	1.344995027	0.718477293	0.831181996	PREDICTED: ras-related protein Rab-6.2
RS000453	GO:0044699	single-organism process	BP	-0.163238634	0.06408679	0.006017898	6.312087033	1.340423108	0.719557318	0.831601015	PREDICTED: proline-rich protein PRCC
RS012931	GO:0044699	single-organism process	BP	0.314299687	0.243515274	0.072230637	3.574858405	1.339862175	0.71968987	0.831615215	PREDICTED: mitochondrial inner membrane protease subunit 1
RS002564	GO:0044699	single-organism process	BP	-0.055922393	-0.07295524	-0.365301811	3.220132904	1.33924172	0.719836499	0.831708337	PREDICTED: wee1-like protein kinase
RS012736	GO:0044699	single-organism process	BP	-0.057541182	0.306260911	0.204181391	4.431666009	1.337745824	0.720190065	0.831964197	PREDICTED: sine oculis-binding protein homolog
RS013018	GO:0044699	single-organism process	BP	0.13429137	0.046847466	-0.138938024	6.093970377	1.323740511	0.723503525	0.834567082	PREDICTED: integrin alpha-8-like
RS014588	GO:0044699	single-organism process	BP	-0.270138594	0.249327239	-0.142196498	2.721477241	1.322545255	0.723786572	0.834587812	PREDICTED: G protein-coupled receptor kinase 2
RS002401	GO:0044699	single-organism process	BP	-0.020315698	-0.107263354	-0.486568077	2.823609491	1.303779178	0.728235869	0.838183379	PREDICTED: TP53-regulating kinase
RS013374	GO:0044699	single-organism process	BP	0.016832719	0.278203004	0.197636103	3.921804526	1.301786339	0.728708936	0.838631898	PREDICTED: solute carrier family 15 member 4
RS007403	GO:0044699	single-organism process	BP	-0.184042388	-0.039960933	0.093322889	4.5559264	1.301370618	0.728807635	0.838631898	PREDICTED: regulatory-associated protein of mTOR
RS007655	GO:0044699	single-organism process	BP	0.011292384	-0.268436054	0.015653774	8.659516926	1.294598436	0.730416129	0.839312111	PREDICTED: peroxiredoxin 1
RS009390	GO:0044699	single-organism process	BP	-0.711993959	-0.803385471	-0.355292865	0.366452897	1.289104566	0.731721917	0.839984739	PREDICTED: gamma-aminobutyric acid receptor alpha-like
RS012744	GO:0044699	single-organism process	BP	-0.167883712	-0.020348239	-0.366472815	4.551424063	1.284276609	0.732870095	0.841057588	PREDICTED: 8-oxo-dGDP phosphatase NUDT18
RS001353	GO:0044699	single-organism process	BP	-0.287844104	-0.294211151	-0.104699816	3.917468146	1.281374336	0.733560605	0.841635256	PREDICTED: probable G-protein coupled receptor CG31760
RS002733	GO:0044699	single-organism process	BP	-0.131086022	-0.223478474	-0.168696314	6.671969789	1.279089307	0.734104415	0.842090446	PREDICTED: tyrosine-protein phosphatase non-receptor type 12
RS002882	GO:0044699	single-organism process	BP	0.362043543	0.12287268	0.14951259	3.567153224	1.277605364	0.734457649	0.842342193	PREDICTED: putative sodium-coupled neutral amino acid transporter 7
RS005230	GO:0044699	single-organism process	BP	-0.262472401	-0.091272997	-0.047101054	6.67466439	1.274786012	0.735128916	0.842540448	PREDICTED: probable pyruvate dehydrogenase E1 component subunit alpha, mitochondrial
RS001950	GO:0044699	single-organism process	BP	0.20325267	0.237480272	0.18707812	5.440940647	1.275559735	0.734944678	0.842540448	PREDICTED: RNA-binding motif protein, X-linked 2
RS012886	GO:0044699	single-organism process	BP	0.043889614	0.045935169	-0.172991381	6.198524418	1.274010735	0.73531354	0.842556521	PREDICTED: RAD50-interacting protein 1
RS003837	GO:0044699	single-organism process	BP	-0.163629332	-0.238266443	-0.158286361	6.375287983	1.268537336	0.736617406	0.843743484	PREDICTED: myotubularin-related protein 13
RS006565	GO:0044699	single-organism process	BP	0.277314691	0.123626202	0.145229407	6.096135504	1.268756312	0.736565227	0.843743484	PREDICTED: isoleucine--tRNA ligase, mitochondrial
RS011746	GO:0044699	single-organism process	BP	-0.39884548	-0.351698614	-0.321292158	2.434881592	1.267908853	0.736767171	0.843838282	PREDICTED: rabphilin-3A
RS012127	GO:0044699	single-organism process	BP	-0.431174895	-0.666271308	-0.435645091	1.992998089	1.263117574	0.737909239	0.844912628	PREDICTED: guanine nucleotide-binding protein subunit gamma-e
RS001155	GO:0044699	single-organism process	BP	0.231488002	0.16782116	0.267286333	7.542611217	1.261267446	0.738350396	0.845036794	PREDICTED: 26S protease regulatory subunit 6A-B
RS015540	GO:0044699	single-organism process	BP	-0.463597962	-0.808832157	-0.297537157	1.427647784	1.257020479	0.73936339	0.845811839	PREDICTED: zinc transporter ZIP11
RS001831	GO:0044699	single-organism process	BP	-0.177972614	-0.541729148	-0.455569709	1.818459057	1.248862293	0.741310524	0.847423504	saxophone
RS013491	GO:0044699	single-organism process	BP	0.50959189	0.011563311	0.381662714	3.356752204	1.246647485	0.741839413	0.847577485	PREDICTED: amine oxidase [flavin-containing] B
RS000181	GO:0044699	single-organism process	BP	-0.209336428	-0.237338166	-0.224925346	4.582710421	1.24606193	0.741979261	0.847649384	PREDICTED: probable ATP-dependent RNA helicase DHX34
RS006056	GO:0044699	single-organism process	BP	0.097307775	-0.123216002	-0.15886195	4.780986891	1.242574999	0.74281221	0.848223421	PREDICTED: DNA repair protein REV1
RS013953	GO:0044699	single-organism process	BP	-0.420067551	-0.07959435	-0.044577109	2.09881014	1.242877248	0.742739999	0.848223421	PREDICTED: two pore potassium channel protein sup-9
RS000122	GO:0044699	single-organism process	BP	0.288651375	0.278795684	0.189466353	4.361874643	1.242736903	0.742773529	0.848223421	PREDICTED: biogenesis of lysosome-related organelles complex 1 subunit 1
RS007878	GO:0044699	single-organism process	BP	0.237698949	0.116278875	0.031546913	5.542687623	1.242544299	0.742819545	0.848223421	PREDICTED: conserved oligomeric Golgi complex subunit 6
RS007210	GO:0044699	single-organism process	BP	0.104668927	0.085929796	-0.201752304	3.844405488	1.238566021	0.743770216	0.8488484	PREDICTED: suppressor of fused homolog
RS001192	GO:0044699	single-organism process	BP	0.284689986	0.054619982	0.012801589	3.953759168	1.234817587	0.744666295	0.849473216	PREDICTED: NADPH:adrenodoxin oxidoreductase, mitochondrial
RS003464	GO:0044699	single-organism process	BP	0.172598356	-0.041210389	0.142351133	6.065811923	1.22736415	0.746449016	0.850210627	lobe protein
RS003459	GO:0044699	single-organism process	BP	0.001382138	0.062656571	0.219503831	6.205280639	1.224130894	0.747222734	0.850784117	PREDICTED: succinate dehydrogenase assembly factor 2, mitochondrial-like
RS010370	GO:0044699	single-organism process	BP	-0.028241889	-0.212720469	-0.248480983	4.787856893	1.223719893	0.747321103	0.850819199	PREDICTED: 85/88 kDa calcium-independent phospholipase A2
RS008693	GO:0044699	single-organism process	BP	-0.559486277	-0.01474303	-0.323993212	0.848527572	1.221198886	0.747924562	0.851148317	PREDICTED: rho GTPase-activating protein 100F
RS006015	GO:0044699	single-organism process	BP	0.202251624	0.17761	0.20909812	6.744213337	1.220874472	0.748002228	0.851148317	PREDICTED: probable dynactin subunit 2
RS012487	GO:0044699	single-organism process	BP	0.253977322	-0.126048889	0.116418283	4.017698188	1.219410378	0.748352765	0.851301122	PREDICTED: katanin p60 ATPase-containing subunit A-like 2
RS014148	GO:0044699	single-organism process	BP	0.186545119	0.030709626	-0.045353955	5.475449872	1.2188916	0.748476983	0.851365528	PREDICTED: pleiotropic regulator 1
RS014987	GO:0044699	single-organism process	BP	-0.129443831	0.074498835	0.080188563	6.502399376	1.215933035	0.749185503	0.85187288	PREDICTED: vacuolar protein sorting-associated protein 26B-like
RS000297	GO:0044699	single-organism process	BP	-0.078397003	0.556951886	0.159163891	1.385608421	1.213828293	0.749689662	0.85220612	PREDICTED: probable glutamate receptor
RS005062	GO:0044699	single-organism process	BP	0.042915661	-0.171071911	-0.235081974	3.733684216	1.211821777	0.75017038	0.852564992	PREDICTED: mitochondrial uncoupling protein 4
RS014703	GO:0044699	single-organism process	BP	0.074865729	-0.455038306	-0.581190522	1.069092191	1.21166288	0.750208452	0.852564992	PREDICTED: caveolin-3-like
RS005898	GO:0044699	single-organism process	BP	-0.109533007	-0.24419355	-0.181501684	5.469138988	1.210081204	0.750587452	0.852649778	PREDICTED: ATP-binding cassette sub-family B member 10, mitochondrial
RS009090	GO:0044699	single-organism process	BP	0.562021791	0.109785238	0.650716386	1.113219948	1.204865274	0.751837656	0.853571243	PREDICTED: unconventional myosin-Ib-like
RS007071	GO:0044699	single-organism process	BP	-0.142708288	-0.13599678	-0.267687329	5.853678032	1.201905454	0.752547341	0.854143945	PREDICTED: dihydropyrimidine dehydrogenase [NADP(+)]
RS007868	GO:0044699	single-organism process	BP	-0.076067947	0.164128919	-0.310728926	2.087794039	1.200913409	0.752785247	0.854336974	PREDICTED: platelet-activating factor acetylhydrolase IB subunit beta homolog
RS012877	GO:0044699	single-organism process	BP	0.012158985	-0.173894892	-0.21613122	4.155231751	1.200465777	0.752892601	0.854381818	PREDICTED: solute carrier family 25 member 38-like

RS014971	GO:0044699	single-organism process	BP	-0.420354002	-0.582733202	-0.554399168	0.64336528	1.189225943	0.755589519	0.856558389	PREDICTED: growth/differentiation factor 8
RS004127	GO:0044699	single-organism process	BP	0.115690403	0.165727139	0.345872341	7.087804839	1.185675978	0.756441814	0.857147907	PREDICTED: polypeptide N-acetylgalactosaminyltransferase 5
RS002324	GO:0044699	single-organism process	BP	0.259123946	0.10530511	-0.239819052	5.644760979	1.184734988	0.756667772	0.85716077	PREDICTED: dynein heavy chain 12, axonemal
RS008405	GO:0044699	single-organism process	BP	-0.507380646	-0.391727947	-0.160990784	2.501471485	1.183658987	0.75692617	0.857267824	PREDICTED: solute carrier family 12 member 9
RS014462	GO:0044699	single-organism process	BP	-0.015529693	-0.23758928	-0.210393211	4.275966799	1.18306732	0.757068267	0.857343562	PREDICTED: limbic system-associated membrane protein
RS012518	GO:0044699	single-organism process	BP	0.281741139	0.043270074	0.049925166	4.919976993	1.180147602	0.757769568	0.857906323	PREDICTED: tonsoku-like protein
RS002255	GO:0044699	single-organism process	BP	-0.368416253	-0.138403963	-0.433771239	1.887732821	1.178695988	0.758118297	0.85813451	PREDICTED: lipoamide acyltransferase component of branched-chain alpha-keto acid dehydrogenase complex, mitochondrial
RS003577	GO:0044699	single-organism process	BP	0.047553007	-0.050524719	-0.207374013	5.413423156	1.178457743	0.758175535	0.85813451	PREDICTED: probable phenylalanine--tRNA ligase alpha subunit
RS014402	GO:0044699	single-organism process	BP	0.25203639	-0.096764054	0.084994005	2.964440161	1.178148368	0.758249864	0.858141516	PRDM9
RS004358	GO:0044699	single-organism process	BP	0.383805277	0.088334542	0.197444303	2.523673705	1.176597363	0.758622528	0.858466262	PREDICTED: docking protein 1
RS012872	GO:0044699	single-organism process	BP	0.347866735	0.308908424	-0.160370575	1.790115903	1.170755672	0.760026511	0.8593028	PREDICTED: molybdopterin synthase sulfur carrier subunit-like
RS006785	GO:0044699	single-organism process	BP	-0.17122852	0.190651478	0.214491802	2.43808167	1.163769113	0.761706435	0.860198246	PREDICTED: tetraspanin-33-like
RS013461	GO:0044699	single-organism process	BP	0.139158606	-0.101013347	0.199986874	6.071677902	1.15813645	0.763061416	0.861033552	PREDICTED: ATP-binding cassette sub-family G member 5
RS005932	GO:0044699	single-organism process	BP	-0.675269705	-0.476368159	-0.327546775	0.669205916	1.156004095	0.763574507	0.861458152	sex peptide receptor
RS002702	GO:0044699	single-organism process	BP	0.241523641	0.372283207	0.450735855	2.97728399	1.149201676	0.765211808	0.862694666	PREDICTED: congested-like trachea protein
RS015552	GO:0044699	single-organism process	BP	0.031743656	-0.145572687	-0.190149689	5.50444468	1.149173771	0.765218526	0.862694666	PREDICTED: glucosylceramidase-like
RS008427	GO:0044699	single-organism process	BP	-0.30220029	-0.34550505	-0.371729561	3.913115141	1.146374634	0.765892474	0.863377177	PREDICTED: protein wntless
RS010811	GO:0044699	single-organism process	BP	-0.327603428	-0.266768771	-0.322247022	2.816350152	1.145482098	0.766107395	0.863542158	PREDICTED: beta-galactoside alpha-2,6-sialyltransferase 2
RS002978	GO:0044699	single-organism process	BP	-0.234829207	-0.123824467	-0.149396436	6.676853808	1.140160941	0.767388972	0.864445144	PREDICTED: splicing factor 1
RS007246	GO:0044699	single-organism process	BP	0.180026918	0.030073467	-0.012525648	6.009929697	1.139773026	0.767482416	0.864473084	PREDICTED: histone acetyltransferase KAT2A
RS010513	GO:0044699	single-organism process	BP	-0.102013328	0.539013863	0.3463181	0.734193901	1.131469419	0.769483181	0.865903825	PREDICTED: cholesterol desaturase daf-36-like
RS015334	GO:0044699	single-organism process	BP	-0.016521369	-0.128326626	-0.191063523	6.308133993	1.126425415	0.770699015	0.866468791	PREDICTED: THO complex subunit 1
RS014787	GO:0044699	single-organism process	BP	0.1326594	-0.131057385	0.010031006	5.422788051	1.125716782	0.770869856	0.866583502	PREDICTED: E3 ubiquitin-protein ligase FANCL
RS000934	GO:0044699	single-organism process	BP	0.300050957	0.566960801	0.17692926	1.72779898	1.121157382	0.771969218	0.867354834	PREDICTED: serine/threonine-protein kinase D3
RS011165	GO:0044699	single-organism process	BP	-0.210244823	0.020310001	-0.022065044	6.798726885	1.120227957	0.772193354	0.867529269	PREDICTED: polypeptide N-acetylgalactosaminyltransferase 35A
RS004027	GO:0044699	single-organism process	BP	0.288457433	0.017397432	-0.40888073	0.587381213	1.118588901	0.77258865	0.867737221	PREDICTED: E3 ubiquitin-protein ligase CCNB1IP1
RS005984	GO:0044699	single-organism process	BP	0.152379143	-0.112920669	-0.068279543	4.98672439	1.116242857	0.773154511	0.868067033	PREDICTED: beta-1,3-galactosyltransferase brn
RS004523	GO:0044699	single-organism process	BP	0.264113833	0.369495276	0.162485791	4.59919441	1.115907837	0.773235323	0.868080376	PREDICTED: SH2 domain-containing protein 4A
RS007813	GO:0044699	single-organism process	BP	0.34178505	-0.155591001	0.135384575	1.821901332	1.113348932	0.773852615	0.868231611	PREDICTED: MOXD1 homolog 2
RS001035	GO:0044699	single-organism process	BP	0.124094061	0.276516429	0.187901485	8.14568359	1.113792392	0.773745632	0.868231611	PREDICTED: tubulin alpha-1 chain
RS009462	GO:0044699	single-organism process	BP	-0.11557416	-0.137615478	0.105349632	4.573469797	1.113369435	0.773847669	0.868231611	PREDICTED: DNA fragmentation factor subunit beta
RS006358	GO:0044699	single-organism process	BP	0.376653322	0.295506773	0.377360215	3.440937942	1.105396194	0.775771583	0.869764739	PREDICTED: engulfment and cell motility protein 1
RS011532	GO:0044699	single-organism process	BP	-0.004883213	-0.304988147	-0.240714936	3.048059116	1.100344088	0.77699102	0.870485316	PREDICTED: D-beta-hydroxybutyrate dehydrogenase, mitochondrial
RS002699	GO:0044699	single-organism process	BP	0.18641679	-0.113157012	0.19259875	3.645600491	1.098441167	0.777450405	0.870639375	PREDICTED: galactosylgalactosylxylosylprotein 3-beta-glucuronosyltransferase S
RS003408	GO:0044699	single-organism process	BP	0.14875062	-0.250114218	0.329780306	2.915781806	1.09604514	0.778028887	0.871039755	PREDICTED: probable glutamate receptor
RS010672	GO:0044699	single-organism process	BP	-0.302029346	0.22206596	0.191043022	1.595359022	1.095814464	0.778084584	0.871039755	Zinc transporter 2
RS006651	GO:0044699	single-organism process	BP	-0.359324357	0.180318882	0.018972841	2.860812982	1.090007528	0.779486837	0.87198945	PREDICTED: putative mediator of RNA polymerase II transcription subunit 24
RS004876	GO:0044699	single-organism process	BP	0.254514777	-0.013585511	0.016602412	4.233428366	1.08122198	0.781608997	0.873820125	PREDICTED: 3-hydroxyisobutyrate dehydrogenase, mitochondrial
RS006804	GO:0044699	single-organism process	BP	-0.106727555	-0.311113867	0.044117663	3.55950746	1.080563363	0.781768115	0.873920437	PREDICTED: Kv channel-interacting protein 4
RS002898	GO:0044699	single-organism process	BP	-0.017250263	-0.424041525	-0.125803688	2.460106405	1.07679768	0.782677961	0.874248185	PREDICTED: tyrosine-protein kinase hopscotch
RS011082	GO:0044699	single-organism process	BP	0.177813024	0.166124529	0.204835906	6.747747806	1.077932566	0.782403742	0.874248185	PREDICTED: charged multivesicular body protein 2a
RS006050	GO:0044699	single-organism process	BP	0.110341354	0.097937695	-0.09319607	5.196452558	1.074872731	0.783143105	0.874526003	PREDICTED: nuclear RNA export factor 1-like
RS005863	GO:0044699	single-organism process	BP	0.219553251	0.135242119	0.19794836	5.869991134	1.071267093	0.784014453	0.874955962	PREDICTED: quinone oxidoreductase
RS006408	GO:0044699	single-organism process	BP	-0.44008577	-0.058685903	-0.12050687	2.264244562	1.067646937	0.784889416	0.875777204	PREDICTED: LIM domain kinase 1
RS011943	GO:0044699	single-organism process	BP	0.336788752	0.302655858	-0.329953803	0.718404792	1.062395748	0.786158762	0.876649854	PREDICTED: glutamate receptor ionotropic, delta-1-like
RS014288	GO:0044699	single-organism process	BP	-0.267562999	-0.21719274	-0.273627167	4.00532462	1.061069676	0.786479339	0.876852054	PREDICTED: ATP-binding cassette sub-family B member 8, mitochondrial
RS009923	GO:0044699	single-organism process	BP	0.114543292	0.251147653	0.19532454	5.007055297	1.059756621	0.786796781	0.877128323	PREDICTED: NF-kappa-B inhibitor-interacting Ras-like protein
RS005955	GO:0044699	single-organism process	BP	0.23219394	-0.012384952	0.075718692	4.142489583	1.059248208	0.786919698	0.877187703	PREDICTED: zinc finger and BTB domain-containing protein 49
RS011765	GO:0044699	single-organism process	BP	-0.144177159	-0.040223372	-0.541930342	1.051839678	1.057913309	0.787242438	0.877330106	PREDICTED: protein Wnt-16-like
RS015467	GO:0044699	single-organism process	BP	-0.145058898	0.071434556	0.080110335	4.868082576	1.053836135	0.788228252	0.878024642	PREDICTED: GPI mannosyltransferase 3
RS009768	GO:0044699	single-organism process	BP	-0.008029071	-0.40798174	-0.080090492	3.716070833	1.051268396	0.788849155	0.878327846	PREDICTED: membrane-associated progesterone receptor component 1-like
RS001346	GO:0044699	single-organism process	BP	-0.305712719	-0.459994451	-0.225727855	2.010578526	1.048797397	0.789446703	0.878744111	PREDICTED: glutamate receptor ionotropic, delta-1-like
RS004706	GO:0044699	single-organism process	BP	-0.281248638	-0.101038833	-0.415774939	3.347688564	1.047260677	0.789818336	0.878882504	PREDICTED: sodium/hydrogen exchanger 7
RS000185	GO:0044699	single-organism process	BP	-0.037221089	0.214766432	-0.051178796	4.757717504	1.046611305	0.789975381	0.878882504	PREDICTED: casein kinase I isoform gamma-3
RS005157	GO:0044699	single-organism process	BP	-0.118250937	-0.0439441	-0.240634957	5.607866798	1.039532981	0.791687355	0.880009757	PREDICTED: serine/threonine-protein kinase Genghis Khan
RS014911	GO:0044699	single-organism process	BP	0.085265571	0.151578786	-0.134476351	5.426560488	1.039739383	0.79163743	0.880009757	PREDICTED: methylated-DNA--protein-cysteine methyltransferase-like
RS010416	GO:0044699	single-organism process	BP	0.102688878	0.052544299	-0.105001885	6.444797474	1.033324222	0.793189205	0.880709569	PREDICTED: 2-oxoglutarate and iron-dependent oxygenase domain-containing protein 3-like
RS007702	GO:0044699	single-organism process	BP	-0.207976473	0.196473721	0.085101443	3.379751314	1.033521878	0.793141391	0.880709569	PREDICTED: ER degradation-enhancing alpha-mannosidase-like protein 1
RS001480	GO:0044699	single-organism process	BP	-0.167209719	0.042692043	-0.020765468	6.882416052	1.032304741	0.793435824	0.880709569	PREDICTED: GTP-binding protein Rheb homolog
RS009476	GO:0044699	single-organism process	BP	0.070675817	-0.142437313	0.031156422	6.23550366	1.029018525	0.794230809	0.881436624	PREDICTED: N-acetylglucosamine-1-phosphotransferase subunits alpha/beta
RS004899	GO:0044699	single-organism process	BP	0.083677265	0.261971071	0.014438767	4.041577121	1.022966523	0.795694972	0.882594899	PREDICTED: DNA polymerase epsilon subunit 3
RS010394	GO:0044699	single-organism process	BP	0.114789658	0.020660296	-0.171961491	4.340996112	1.019930784	0.796429449	0.88302073	PREDICTED: Fanconi anemia-associated protein of 24 kDa
RS012225	GO:0044699	single-organism process	BP	0.123374918	0.239169678	0.240173626	4.155168052	1.019975888	0.796418536	0.88302073	PREDICTED: mitochondrial 2-oxodicarboxylate carrier
RS007599	GO:0044699	single-organism process	BP	0.23961498	-0.031443355	-0.044630992	4.997069358	1.019570467	0.796516627	0.883039647	PREDICTED: TATA-binding protein-associated factor 172
RS007185	GO:0044699	single-organism process	BP	-0.40805096	-0.102942074	-0.188514429	3.229175551	1.01478824	0.7976737	0.883615901	PREDICTED: carbohydrate sulfotransferase 11
RS014637	GO:0044699	single-organism process	BP	0.066840247	0.600909401	0.396436471	2.051509931	1.008937941	0.799089251	0.884723519	PREDICTED: ER degradation-enhancing alpha-mannosidase-like protein 1
RS003722	GO:0044699	single-organism process	BP	-0.060079024	0.09677149	-0.140630114	7.508919419	1.005963793	0.799808898	0.885105534	PREDICTED: cytoplasmic aconitate hydratase-like
RS011666	GO:0044699	single-organism process	BP	0.194502435	-0.083841257	0.107781796	4.058911746	0.999234825	0.801437107	0.885725087	PREDICTED: flap endonuclease 1
RS001501	GO:0044699	single-organism process	BP	-0.162735947	-0.319720165	-0.011856727	3.268041292	0.998514331	0.801611445	0.885725087	PREDICTED: MFS-type transporter SLC18B1-like
RS008007	GO:0044699	single-organism process	BP	-0.299753326	-0.158742463	0.220604965	1.716465146	1.000828824	0.801051406	0.885725087	PREDICTED: soluble guanylate cyclase 88E
RS008177	GO:0044699	single-organism process	BP	-0.207235443	-0.193747342	-0.174587667	4.90122724	1.000799571	0.801058484	0.885725087	PREDICTED: adenylosuccinate lyase
RS001032	GO:0044699	single-organism process	BP	-0.035675482	-0.19444943	-0.243524005	6.174925441	0.996317401	0.802143037	0.886178947	PREDICTED: dynein heavy chain 3, axonemal
RS001592	GO:0044699	single-organism process	BP	-0.031932856	-0.21203954	0.020473628	5.518186721	0.994927465	0.802479359	0.88620662	PREDICTED: sarcosine dehydrogenase, mitochondrial
RS011965	GO:0044699	single-organism process	BP	0.317265942	-0.117437467	-0.074967125	1.692671759	0.994636233	0.802549828	0.88620662	PREDICTED: MOXD1 homolog 2
RS005521	GO:0044699	single-organism process	BP	0.071891096	-0.700368041	-0.118874224	0.50692932	0.994536759	0.802573898	0.88620662	PREDICTED: cyclin-dependent kinases regulatory subunit
RS002770	GO:0044699	single-organism process	BP	-0.119360703	-0.359384597	-0.31109241	10.31331347	0.993680302	0.802781134	0.886207234	glyceraldehyde-3-phosphate dehydrogenase
RS014368	GO:0044699	single-organism process	BP	-0.289633023	-0.138434263	-0.088387792	4.620332903	0.992830213	0.802986828	0.886311666	PREDICTED: calcium release-activated calcium channel protein 1-like
RS010660	GO:0044699	single-organism process	BP	0.206965433	0.02100087	0.364453235	2.788303671	0.98771275	0.804225074	0.88744507	opsin, long-wavelength sensitive type
RS012778	GO:0044699	single-organism process	BP	-0.018722594	-0.192886376	-0.083182665	6.08277868	0.984119419	0.805094511	0.888015438	PREDICTED: hsp90 co-chaperone Cdc37
RS004456	GO:0044699	single-organism process	BP	0.366045518	0.210318536	0.311608774	2.475173309	0.982563731	0.805470916	0.888275019	PREDICTED: armadillo repeat-containing protein 4
RS003644	GO:0044699	single-organism process	BP	-0.112541068	0.045579425	0.179657323	4.365690395	0.981222887	0.805795334	0.888418842	PREDICTED: mitochondrial genome maintenance exonuclease 1-like
RS000019	GO:0044699	single-organism process	BP	0.015778594	-0.335849458	-0.242941961	4.003640077	0.978625733	0.806423705	0.888773172	PREDICTED: basic salivary proline-rich protein 2
RS007027	GO:0044699	single-organism process	BP	0.046638135	0.211656467	0.202947562	5.933716015	0.976651636	0.806901318	0.888996168	PREDICTED: beta-1,4-glucuronyltransferase 1
RS009545	GO:0044699	single-organism process	BP	0.048908783	-0.239577504	-0.201089225	6.838828622	0.974337729	0.80746113	0.889424188	PREDICTED: adipocyte plasma membrane-associated protein-like
RS002306	GO:0044699	single-organism process	BP	-0.217902651	-0.104333884	-0.179384255	5.311832399	0.97144951	0.808159864	0.889574066	PREDICTED: lysine-specific demethylase lid
RS011093	GO:0044699	single-organism process	BP	-0.05744554	-0.329366907	-0.456864573	1.66329981	0.971819987	0.808070237	0.889574066	PREDICTED: protein kinase C
RS007150	GO:0044699	single-organism process	BP	0.246217518	0.212666126	0.29750827	5.406495568	0.963851814	0.80999779	0.890926827	PREDICTED: probable multidrug resistance-associated protein lethal(2)03659
RS015108	GO:0044699	single-organism process	BP	0.185335437	-0.0665085	0.107755691	3.971128253	0.963097754	0.810180188	0.89097182	PREDICTED: mitochondrial cardiolipin hydrolase
RS002016	GO:0044699	single-organism process	BP	-0.219720189	-0.011165184	0.043784713	5.130790684	0.958891453	0.811197592	0.891857047	PREDICTED: embryonic polarity protein dorsal-like
RS007717	GO:0044699	single-organism process	BP	0.066529061	0.463299961	0.014408782	1.808915589	0.956769727	0.811710752	0.892106596	PREDICTED: V-type proton ATPase 21 kDa proteolipid subunit
RS003105	GO:0044699	single-organism process	BP	0.209748722	0.196596402	0.069569969	4.899802605	0.955619911	0.811988837	0.892181767	PREDICTED: alkylglycerol monooxygenase-like
RS003294	GO:0044699	single-organism process	BP	-0.223323713	-0.091859915	-0.086004838	5.394693822	0.952084787	0.812843763	0.892576018	PREDICTED: exocyst complex component 2
RS008272	GO:0044699	single-organism process	BP	0.079621716	0.102426005	0.381084129	2.970121429	0.950880254	0.813135048	0.892579513	PREDICTED: adenosine kinase 2
RS012547	GO:0044699	single-organism process	BP	0.14825459	0.028003325	-0.066423805	5.477711778	0.949162398	0.81355045	0.892729317	PREDICTED: condensin complex subunit 2
RS002685	GO:0044699	single-organism process	BP	-0.106623202	0.129132903	-0.002060714	5.277398632	0.946440961	0.814208493	0.893217924	PREDICTED: calcium uptake protein 3, mitochondrial
RS015273	GO:0044699	single-organism process	BP	-0.063991481	-0.323635658	0.409483072	0.354255725	0.944929709	0.814573892	0.893337407	PREDICTED: longitudinals lacking protein
RS004689	GO:0044699	single-organism process	BP	-0.612760571	-0.6607617	-0.621054484	0.296820601	0.945248721	0.814496761	0.893337407	PREDICTED: diuretic hormone receptor-like
RS005532	GO:0044699	single-organism process	BP	-0.030306406	-0.135903277	0.086199672	5.499589864	0.94359213	0.814897286	0.893506566	PREDICTED: RNA polymerase II subunit A C-terminal domain phosphatase
RS008895	GO:0044699	single-organism process	BP	-0.026855739	-0.00671437	0.176077167	6.14530021	0.94096375	0.815532726	0.893981253	PREDICTED: ATP-dependent RNA helicase SUV3 homolog, mitochondrial
RS000442	GO:0044699	single-organism process	BP	-0.030981455	-0.235190752	-0.143405567	4.312058358	0.936944795	0.816504251	0.894645427	PREDICTED: proton-coupled folate transporter-like
RS001469	GO:0044699	single-organism process	BP	-0.317934529	-0.305329378	-0.244489188	3.485731847	0.93468792	0.817049761	0.895009541	PREDICTED: transcriptional adapter 2B
RS009674	GO:0044699	single-organism process	BP	0.363941417	0.284951845	0.044204898	6.008768547	0.930722767	0.818008075	0.895747648	PREDICTED: dynein beta chain, ciliary
RS002814	GO:0044699	single-organism process	BP	0.014206663	0.225083985	-0.196562045	3.228829268	0.930926008	0.817958959	0.895747648	PREDICTED: D-glucuronyl C5-epimerase
RS002932	GO:0044699	single-organism process	BP	0.055970556	-0.003033501	0.220016606	7.070781008	0.921709314	0.820185942	0.897430213	PREDICTED: aldehyde dehydrogenase, mitochondrial
RS007659	GO:0044699	single-organism process	BP	-0.114888907	0.141143829	-0.162452328	4.019033592	0.921749708	0.820176183	0.897430213	PREDICTED: ubiquitin carboxyl-terminal hydrolase 4
RS013224	GO:0044699	single-organism process	BP	0.122086809	-0.150594114	-0.259318034	3.533638766	0.920892955	0.820383154	0.897490049	PREDICTED: low-density lipoprotein receptor-related protein 6
RS000150	GO:0044699	single-organism process	BP	0.215426876	0.132994308	0.193610407	5.217037063	0.918173637	0.821040024	0.898081827	PREDICTED: glucose-6-phosphatase 2
RS002763	GO:0044699	single-organism process	BP	-0.046028975	-0.042891999	-0.320861965	3.723402786	0.916658152	0.821406066	0.89821898	PREDICTED: WD repeat-containing protein 48
RS002381	GO:0044699	single-organism process	BP	0.251538422	-0.184815243	0.026115519	2.109803288	0.915441681	0.821699867	0.898306261	PREDICTED: dynein light chain 4, axonemal
RS014043	GO:0044699	single-organism process	BP	-0.148156353	0.203034172	0.127649507	2.873911963	0.906496586	0.823859757	0.900042486	PREDICTED: histone acetyltransferase KAT8
RS009843	GO:0044699	single-organism process	BP	-0.172356069	-0.30206292	-0.092446383	3.8192117	0.906528704	0.823852003	0.900042486	PREDICTED: 7-methylguanosine phosphate-specific 5'-nucleotidase

RS009351	GO:0044699	single-organism process	BP	-0.121565336	-0.143470121	-0.207423994	6.852684229	0.904669389	0.824300834	0.90044624	PREDICTED: vesicle-associated membrane protein-associated protein B
RS006075	GO:0044699	single-organism process	BP	-0.226394382	-0.065362369	0.088572196	3.008430896	0.898142308	0.82587609	0.901150881	PREDICTED: acetyl-coenzyme A transporter 1
RS015527	GO:0044699	single-organism process	BP	0.095932391	-0.126719206	0.030481933	5.507190068	0.897198446	0.826103835	0.901321295	PREDICTED: acyl-CoA dehydrogenase family member 9, mitochondrial
RS008929	GO:0044699	single-organism process	BP	-0.139724103	-0.236598473	-0.091823273	4.57881475	0.895160984	0.826595415	0.901623303	PREDICTED: cytochrome c oxidase assembly protein COX15 homolog
RS009425	GO:0044699	single-organism process	BP	0.10798095	0.061380806	-0.158803348	7.481137034	0.883754565	0.829346331	0.903958365	PREDICTED: histone-lysine N-methyltransferase, H3 lysine-79 specific
RS003379	GO:0044699	single-organism process	BP	-0.109446268	0.122333667	0.075109531	7.929044814	0.881096492	0.829987097	0.904255802	PREDICTED: ras-related protein Rab-1A
RS007987	GO:0044699	single-organism process	BP	-0.108783046	-0.260376904	-0.017555578	4.226643361	0.877727216	0.830799144	0.904790531	PREDICTED: head-specific guanylate cyclase
RS011500	GO:0044699	single-organism process	BP	-0.54663125	-0.559810746	-0.038971768	0.470844775	0.876166842	0.831175153	0.904842	PREDICTED: zinc transporter ZIP8
RS004596	GO:0044699	single-organism process	BP	0.06304095	0.073784272	-0.155201973	5.372603719	0.872303114	0.832106031	0.905516578	PREDICTED: SH3 domain-binding glutamic acid-rich protein homolog
RS013172	GO:0044699	single-organism process	BP	0.064707769	0.050177661	0.256276698	4.54520608	0.870549691	0.832528393	0.905630734	PREDICTED: N-acetyl-D-glucosamine kinase
RS005799	GO:0044699	single-organism process	BP	-0.170095951	-0.166816491	-0.322385565	3.5068153	0.871097028	0.832396557	0.905630734	PREDICTED: probable maleylacetoacetate isomerase 2
RS006825	GO:0044699	single-organism process	BP	-0.276515626	-0.143960507	0.082362978	2.762529123	0.86935597	0.832815903	0.90567245	PREDICTED: ras-related protein Rab-9B
RS012029	GO:0044699	single-organism process	BP	-0.276393946	-0.049076576	-0.007451282	3.393647443	0.867360029	0.833296571	0.905988649	PREDICTED: thioredoxin, mitochondrial
RS014606	GO:0044699	single-organism process	BP	-0.143915834	0.212649167	-0.24267723	3.367536213	0.853642153	0.836598099	0.908207608	PREDICTED: major facilitator superfamily domain-containing protein 1-like
RS004634	GO:0044699	single-organism process	BP	-0.276624638	0.028539956	-0.191250909	3.694247185	0.850639774	0.837320188	0.908678195	PREDICTED: protein pellino
RS013919	GO:0044699	single-organism process	BP	0.009501196	0.169264606	-0.027306846	6.022790259	0.850141837	0.837439927	0.908684035	PREDICTED: serine/threonine-protein kinase RIO3
RS006384	GO:0044699	single-organism process	BP	-0.172858547	-0.282112872	-0.212856479	4.183875927	0.847784731	0.838006666	0.909188142	PREDICTED: ras-like protein family member 10B
RS001713	GO:0044699	single-organism process	BP	-0.023623329	0.232569309	0.03284288	3.878331878	0.838151447	0.840321611	0.910993404	PREDICTED: serine/threonine-protein kinase Sgk3
RS005927	GO:0044699	single-organism process	BP	0.138944119	0.107334365	0.197785171	6.406998597	0.835186065	0.841033787	0.911530078	PREDICTED: sprouty-related, EVH1 domain-containing protein 1
RS014784	GO:0044699	single-organism process	BP	-0.033507375	0.041403523	-0.177899145	4.971404331	0.833957051	0.84132889	0.911638874	PREDICTED: NADH-cytochrome b5 reductase-like
RS001970	GO:0044699	single-organism process	BP	0.084033429	-0.012169719	-0.157558613	5.885614174	0.833122903	0.84152916	0.911638874	PREDICTED: glutamate receptor ionotropic, kainate 2
RS009311	GO:0044699	single-organism process	BP	0.17556667	0.058214485	-0.24291434	2.192551785	0.8273653	0.842911036	0.912465324	PREDICTED: bifunctional ATP-dependent dihydroxyacetone kinase/FAD-AMP lyase (cyclizing)-like
RS008464	GO:0044699	single-organism process	BP	-0.07818374	0.007615105	0.128259633	5.44261975	0.825685956	0.843313938	0.912744563	PREDICTED: polypeptide N-acetylgalactosaminyltransferase 1
RS014821	GO:0044699	single-organism process	BP	-0.252579087	-0.384297106	-0.265729211	2.565842443	0.81795654	0.84516742	0.914113642	PREDICTED: spermine oxidase-like
RS008948	GO:0044699	single-organism process	BP	-0.139209169	-0.445981598	-0.384340527	1.178240838	0.817697319	0.845229553	0.914113642	hypothetical protein
RS005586	GO:0044699	single-organism process	BP	0.165076617	-0.125717332	0.501179805	0.440219349	0.817686596	0.845232123	0.914113642	big brain
RS007067	GO:0044699	single-organism process	BP	-0.005563285	-0.115207847	-0.172105232	5.226229812	0.811554498	0.8467014	0.914916983	PREDICTED: protein HEXIM-like
RS013781	GO:0044699	single-organism process	BP	0.193638779	-0.537237581	0.052129311	0.617025573	0.806231879	0.847975877	0.915587122	PREDICTED: neurexin-1
RS004240	GO:0044699	single-organism process	BP	0.049479372	-0.048650348	-0.13481177	6.340483542	0.801952368	0.848999996	0.916300107	PREDICTED: metastasis-associated protein MTA3
RS001074	GO:0044699	single-organism process	BP	0.209133952	0.10331734	-0.085594621	2.799301808	0.800021453	0.849461902	0.916575594	5-hydroxyisourate hydrolase
RS006940	GO:0044699	single-organism process	BP	0.23315657	0.184699143	0.232741985	5.696286415	0.798391233	0.84985179	0.916728432	PREDICTED: ATP-dependent RNA helicase bel
RS009813	GO:0044699	single-organism process	BP	-0.010458943	-0.044123502	-0.222894149	4.074724376	0.793540037	0.851011539	0.917684676	PREDICTED: kelch-like protein 18
RS012809	GO:0044699	single-organism process	BP	0.138894759	-0.067618824	-0.049182961	6.532772502	0.790924641	0.851636486	0.918122744	PREDICTED: ATP-dependent DNA helicase Q5-like
RS013860	GO:0044699	single-organism process	BP	0.220808872	0.226587436	0.176488663	3.873415312	0.782047995	0.853755921	0.919777764	PREDICTED: cyclin-dependent kinase 2-like
RS009433	GO:0044699	single-organism process	BP	0.385144915	-0.153581998	0.101631697	1.554735334	0.779990692	0.854246764	0.919991769	gag-pol polyprotein
RS008022	GO:0044699	single-organism process	BP	-0.008491466	-0.090368246	-0.241833023	4.214597047	0.776767956	0.855015376	0.920504672	PREDICTED: E3 ubiquitin-protein ligase parkin
RS003423	GO:0044699	single-organism process	BP	-0.204426608	-0.211392288	-0.35583628	3.26361471	0.777317342	0.854884374	0.920504672	PREDICTED: histone-lysine N-methyltransferase EHMT2
RS006019	GO:0044699	single-organism process	BP	0.016800687	-0.569398535	-0.206623396	0.539126613	0.775635116	0.855285471	0.920559373	PREDICTED: cationic amino acid transporter 2
RS000592	GO:0044699	single-organism process	BP	0.023504386	-0.152053903	-0.130032456	4.686848899	0.775930989	0.855214932	0.920559373	PREDICTED: phosphatidylinositol 4,5-bisphosphate 3-kinase catalytic subunit delta isoform
RS014955	GO:0044699	single-organism process	BP	-0.057432753	-0.004844162	-0.218290687	4.164955338	0.774569745	0.855539439	0.920596693	PREDICTED: SWI/SNF complex subunit SMARCC1-like
RS004416	GO:0044699	single-organism process	BP	-0.214358133	0.042991977	0.181506365	2.545521498	0.775084362	0.855416767	0.920596693	PREDICTED: monocarboxylate transporter 12-like
RS008622	GO:0044699	single-organism process	BP	0.280784236	-0.039365013	-0.064153371	2.135336557	0.771858031	0.856185692	0.920985865	PREDICTED: katanin p60 ATPase-containing subunit A-like 1
RS010914	GO:0044699	single-organism process	BP	-0.347466111	-0.225374731	-0.327929208	1.867203108	0.771517813	0.856266754	0.920985865	PREDICTED: transcription factor collier
RS004824	GO:0044699	single-organism process	BP	-0.119817424	-0.002510339	-0.218462131	5.836600219	0.766555667	0.857448594	0.921706008	PREDICTED: tumor suppressor candidate 3
RS012537	GO:0044699	single-organism process	BP	-0.212067979	-0.140687452	-0.284974938	4.062566229	0.76303063	0.858287615	0.922293022	PREDICTED: tyrosine-protein kinase Src64B
RS000298	GO:0044699	single-organism process	BP	-0.477815194	-0.328207506	-0.189725936	1.140735658	0.76073894	0.858832832	0.922721437	PREDICTED: glutamate receptor ionotropic, NMDA 3A-like
RS001482	GO:0044699	single-organism process	BP	-0.208896326	-0.157781308	-0.17016301	4.389662984	0.7595889	0.859106364	0.922936582	PREDICTED: ubiquitin carboxyl-terminal hydrolase 8
RS006755	GO:0044699	single-organism process	BP	0.06229291	0.176504231	0.172369871	5.353781931	0.759280259	0.859179765	0.922936707	PREDICTED: Parkinson disease 7 domain-containing protein 1
RS004042	GO:0044699	single-organism process	BP	0.203741087	0.362883244	0.585874991	5.682798387	0.758371704	0.859395815	0.923090055	PREDICTED: fatty acid synthase
RS000764	GO:0044699	single-organism process	BP	0.020886652	0.003406521	0.189564929	4.777359509	0.752422008	0.860809848	0.924057214	PREDICTED: GTPase-activating protein and VPS9 domain-containing protein 1
RS002959	GO:0044699	single-organism process	BP	-0.310320167	-0.096091889	-0.027692262	3.017853716	0.747218875	0.862045317	0.924989241	PREDICTED: 2-oxoisovalerate dehydrogenase subunit beta, mitochondrial
RS002865	GO:0044699	single-organism process	BP	0.139107175	-0.024859854	-0.057863489	4.933967608	0.745084535	0.862551797	0.925368639	PREDICTED: glycolipid transfer protein
RS002815	GO:0044699	single-organism process	BP	0.393824696	0.312417268	0.029549746	1.049567949	0.739584358	0.863856135	0.926353127	PREDICTED: dynein assembly factor 3, axonemal
RS015091	GO:0044699	single-organism process	BP	-0.134281621	-0.160424628	-0.198718771	6.519985926	0.738356389	0.86414717	0.92645522	PREDICTED: kinesin light chain
RS009424	GO:0044699	single-organism process	BP	-0.142588946	-0.138930413	-0.14960561	6.66400614	0.73306479	0.865400579	0.927009857	PREDICTED: collagen type IV alpha-3-binding protein
RS007369	GO:0044699	single-organism process	BP	0.057173494	-0.085692682	0.135206562	4.674891271	0.733546583	0.865286507	0.927009857	PREDICTED: vacuolar-sorting protein SNF8
RS005850	GO:0044699	single-organism process	BP	0.077070408	-0.110943737	-0.009607352	5.995328644	0.732458319	0.865544156	0.927084801	PREDICTED: tumor susceptibility gene 101 protein
RS011224	GO:0044699	single-organism process	BP	0.00980749	0.112477412	0.184400596	5.967450382	0.730589269	0.865986537	0.927401734	PREDICTED: GPI mannosyltransferase 4
RS001978	GO:0044699	single-organism process	BP	-0.330141653	-0.237382533	-0.097004779	3.877946755	0.725987594	0.867075045	0.928329772	PREDICTED: hemicentin-2
RS012554	GO:0044699	single-organism process	BP	-0.267727564	-0.325817741	-0.178689085	3.94502166	0.725108546	0.867282873	0.928394433	PREDICTED: rho-related BTB domain-containing protein 1
RS002905	GO:0044699	single-organism process	BP	0.088090998	0.253655374	0.156881191	4.076313798	0.723831965	0.867584626	0.928559597	PREDICTED: elongation factor Tu, mitochondrial-like
RS008884	GO:0044699	single-organism process	BP	0.08087288	0.024205228	0.419370624	1.16557718	0.722862304	0.867813782	0.928725932	PREDICTED: apolipoprotein D
RS002666	GO:0044699	single-organism process	BP	0.127068677	-0.094588635	0.428370884	1.329270369	0.709147508	0.871050283	0.931081927	PREDICTED: 46 kDa FK506-binding nuclear protein
RS008938	GO:0044699	single-organism process	BP	0.215794238	0.189677042	0.236190469	8.930167625	0.706178908	0.871749654	0.931592291	PREDICTED: heat shock 70 kDa protein cognate 5
RS004842	GO:0044699	single-organism process	BP	0.05115507	-0.142968429	-0.093764274	4.319758607	0.702780855	0.87254967	0.931784791	PREDICTED: WD repeat-containing protein 73
RS000701	GO:0044699	single-organism process	BP	-0.210234157	-0.305070462	-0.137758538	3.562312096	0.700924435	0.872986491	0.931965065	PREDICTED: protein O-linked-mannose beta-1,4-N-acetylglucosaminyltransferase 2
RS010145	GO:0044699	single-organism process	BP	0.22501962	0.086276598	0.114988845	8.071473402	0.700263135	0.873142055	0.931973137	PREDICTED: F-actin-capping protein subunit alpha
RS015134	GO:0044699	single-organism process	BP	-0.226754082	-0.238391391	-0.223410296	3.370253838	0.698915605	0.87345898	0.932074428	PREDICTED: histone-lysine N-methyltransferase SUV39H2-like
RS014156	GO:0044699	single-organism process	BP	-0.058031861	0.001665872	-0.209341233	5.730992757	0.696637201	0.873994627	0.93233003	PREDICTED: 2-oxoisovalerate dehydrogenase subunit alpha, mitochondrial
RS000066	GO:0044699	single-organism process	BP	-0.032220815	-0.172850098	-0.01678773	5.03444559	0.692162711	0.875045792	0.932661367	PREDICTED: vacuolar protein sorting-associated protein 29
RS013873	GO:0044699	single-organism process	BP	0.184186733	0.079457296	0.053956788	6.30963553	0.690319283	0.875478555	0.932877837	PREDICTED: ras-related protein Rab-14
RS015233	GO:0044699	single-organism process	BP	-0.028168318	0.124444095	-0.146279858	3.77098604	0.6887061	0.875857119	0.933052322	PREDICTED: kynurenine formamidase
RS011344	GO:0044699	single-organism process	BP	-0.159583809	-0.219183185	-0.241957742	3.786489846	0.684898687	0.876750051	0.933750515	PREDICTED: ceramide glucosyltransferase
RS010574	GO:0044699	single-organism process	BP	-0.354727613	-0.373915239	-0.481676921	0.828534929	0.681580005	0.877527727	0.933866301	PREDICTED: G protein-activated inward rectifier potassium channel 3-like
RS004375	GO:0044699	single-organism process	BP	-0.439923774	-0.123751868	-0.173141227	1.274835456	0.678853987	0.878166071	0.934326561	PREDICTED: Kv channel-interacting protein 1
RS004601	GO:0044699	single-organism process	BP	-0.128635596	-0.15423878	-0.239776755	5.581079946	0.672581672	0.879633272	0.935315761	PREDICTED: ankyrin-3
RS000238	GO:0044699	single-organism process	BP	-0.050837525	-0.611359765	-0.107163686	0.567644784	0.665998955	0.881170673	0.936336775	G-protein coupled receptor 161
RS000459	GO:0044699	single-organism process	BP	-0.078440734	0.084608735	-0.034836862	6.245747856	0.664175004	0.881596213	0.936551894	PREDICTED: Golgi phosphoprotein 3 homolog rotini
RS009410	GO:0044699	single-organism process	BP	-0.021024962	-0.164944243	-0.123826627	5.58134017	0.65876902	0.882856308	0.937179058	PREDICTED: cyclin-dependent kinase 12
RS000176	GO:0044699	single-organism process	BP	0.087073889	-0.057775582	0.095474446	5.229956059	0.659136625	0.882770677	0.937179058	PREDICTED: N-glycosylase/DNA lyase
RS004545	GO:0044699	single-organism process	BP	-0.089423892	-0.138498359	-0.249800109	3.713413066	0.658786227	0.8828523	0.937179058	PREDICTED: chromatin modification-related protein MEAF6
RS001040	GO:0044699	single-organism process	BP	-0.098603565	-0.201658646	0.076364834	3.089669028	0.652037571	0.884422889	0.938003319	PREDICTED: sideroflexin-2
RS014130	GO:0044699	single-organism process	BP	0.393138432	0.253437286	0.451794066	1.035306574	0.652814694	0.884242175	0.938003319	PREDICTED: ras guanyl-releasing protein 3
RS014622	GO:0044699	single-organism process	BP	-0.047075902	-0.128467641	0.089339876	4.333738603	0.647347266	0.885512787	0.938417035	PREDICTED: glyoxal reductase-like
RS004932	GO:0044699	single-organism process	BP	-0.107358821	-0.273048279	-0.380139022	2.306685824	0.645070288	0.886041394	0.93887791	PREDICTED: succinyl-CoA ligase [GDP-forming] subunit beta, mitochondrial
RS007364	GO:0044699	single-organism process	BP	-0.091098515	0.061208309	0.058281616	5.553320345	0.641700559	0.886823078	0.939254915	PREDICTED: STAGA complex 65 subunit gamma
RS002505	GO:0044699	single-organism process	BP	0.357481159	0.105045367	0.191570684	1.883917881	0.637078351	0.887894104	0.939757962	PREDICTED: transforming protein p68/c-ets-1-like
RS005225	GO:0044699	single-organism process	BP	-0.003239015	-0.199279073	-0.02219917	7.432966705	0.637526891	0.887790233	0.939757962	PREDICTED: cathepsin B
RS007595	GO:0044699	single-organism process	BP	-0.043630471	-0.222354057	0.080312883	5.057929375	0.63376465	0.888661066	0.940408157	PREDICTED: fatty acid synthase-like
RS009546	GO:0044699	single-organism process	BP	0.035774641	-0.136528477	-0.013542658	6.07588184	0.633008349	0.888836011	0.940435245	PREDICTED: golgin subfamily A member 4
RS010866	GO:0044699	single-organism process	BP	-0.039357915	-0.019276508	0.260698288	5.55455209	0.629921716	0.889549601	0.940745576	PREDICTED: epidermal growth factor receptor
RS005536	GO:0044699	single-organism process	BP	-0.192514907	-0.002056217	-0.037030388	6.598340113	0.628539533	0.889868935	0.940916748	PREDICTED: ras-like GTP-binding protein Rho1
RS000388	GO:0044699	single-organism process	BP	0.163881885	0.163236809	0.093750927	6.417536992	0.626420759	0.890358194	0.940948778	PREDICTED: ras-related protein Rab-43
RS000973	GO:0044699	single-organism process	BP	0.0849184	0.05775336	0.225359978	3.946600078	0.622551135	0.891250953	0.941217158	PREDICTED: 6-pyruvoyl tetrahydrobiopterin synthase
RS002453	GO:0044699	single-organism process	BP	0.258801838	0.127103034	0.082139649	4.295877362	0.622040556	0.891368671	0.941217158	PREDICTED: DNA polymerase delta subunit 3
RS010425	GO:0044699	single-organism process	BP	0.170883627	0.369811289	0.502252155	0.509891817	0.615691468	0.892830962	0.942219758	PREDICTED: glycerophosphodiester phosphodiesterase 1
RS003037	GO:0044699	single-organism process	BP	0.18532221	0.142190916	-0.199884767	1.414409688	0.613421112	0.89335316	0.942680327	PREDICTED: TBC1 domain family member 31
RS003957	GO:0044699	single-organism process	BP	0.345462579	0.413965389	0.154609396	1.754535313	0.612321805	0.893605875	0.942823504	PREDICTED: solute carrier family 2, facilitated glucose transporter member 1-like
RS003729	GO:0044699	single-organism process	BP	-0.067068908	-0.31722074	-0.453287028	1.039779299	0.608180172	0.894557183	0.943318527	PREDICTED: neuronal acetylcholine receptor subunit alpha-7-like
RS000147	GO:0044699	single-organism process	BP	-0.194312079	0.053736367	-0.070014322	4.918513595	0.607133119	0.894797485	0.943361649	PREDICTED: neogenin
RS007654	GO:0044699	single-organism process	BP	0.009311392	0.056329921	0.176145345	4.76807517	0.604164382	0.895478375	0.94375704	PREDICTED: tyrosine-protein kinase transmembrane receptor Ror2
RS003394	GO:0044699	single-organism process	BP	0.180289073	0.135383955	0.089380518	6.008505153	0.597460048	0.89701359	0.944722291	PREDICTED: vesicle transport protein USE1
RS013132	GO:0044699	single-organism process	BP	0.112449764	0.120524483	0.198181067	5.618714839	0.596632622	0.897202823	0.944842575	PREDICTED: pyruvate dehydrogenase phosphatase regulatory subunit, mitochondrial
RS001706	GO:0044699	single-organism process	BP	0.033602523	0.158734434	-0.006749049	5.288609056	0.590995424	0.898490636	0.945803333	PREDICTED: programmed cell death protein 6
RS001538	GO:0044699	single-organism process	BP	0.320201018	0.172065415	-0.020378547	1.614496317	0.588836165	0.898983255	0.94603515	PREDICTED: allatostatin-A receptor-like
RS004108	GO:0044699	single-organism process	BP	0.048512455	0.016995297	-0.151609859	5.239718172	0.588054192	0.899161565	0.94603515	PREDICTED: endoplasmic reticulum resident protein 44
RS009465	GO:0044699	single-organism process	BP	0.099688848	0.186059472	0.123089643	7.725505743	0.584687351	0.899928732	0.94652601	PREDICTED: ras-related protein Rab-7a
RS014940	GO:0044699	single-organism process	BP	0.00473671	-0.350880149	-0.354295712	0.736422598	0.582482205	0.900430699	0.946555742	PREDICTED: potassium voltage-gated channel protein Shal
RS011084	GO:0044699	single-organism process	BP	-0.276842157	-0.273241819	-0.12030894	2.495038084	0.576329104	0.90182925	0.947575302	PREDICTED: paired box protein Pax-6-like
RS012296	GO:0044699	single-organism process	BP	-0.276526091	-0.095232679	-0.053143451	3.613607819	0.569149321	0.903457157	0.9486709	PREDICTED: zinc finger FYVE domain-containing protein 1-like
RS013157	GO:0044699	single-organism process	BP	0.115195703	0.398542992	-0.12346977	0.94470257	0.569142202	0.903458769	0.9486709	Rm62

RS000550	GO:0044699	single-organism process	BP	-0.107303114	-0.150193748	-0.021630838	4.909164031	0.567296373	0.903876573	0.948855677	PREDICTED: ras-related protein Rab-33B
RS002652	GO:0044699	single-organism process	BP	-0.106248501	-0.150324784	-0.205824742	4.194524752	0.564648155	0.904475484	0.94929808	PREDICTED: serine/threonine-protein kinase GL21140
RS005270	GO:0044699	single-organism process	BP	-0.004544488	-0.216158524	-0.097291995	3.745309896	0.56443291	0.904524136	0.94929808	PREDICTED: xylosyltransferase oxt
RS009067	GO:0044699	single-organism process	BP	-0.057216322	-0.133941113	0.099775616	3.741902064	0.56109264	0.905278622	0.949931592	hypothetical protein
RS001309	GO:0044699	single-organism process	BP	0.105977595	-0.144048822	-0.114622842	2.982143032	0.558586257	0.905844108	0.950148239	PREDICTED: ashwin-like
RS009356	GO:0044699	single-organism process	BP	0.06777373	-0.078226225	-0.119045856	4.494731858	0.558495291	0.905864621	0.950148239	PREDICTED: differentially expressed in FDCP 8 homolog
RS005560	GO:0044699	single-organism process	BP	0.073648066	0.070694895	-0.087436803	5.960124723	0.555903739	0.906448712	0.950525819	PREDICTED: protein catecholamines up
RS003738	GO:0044699	single-organism process	BP	0.092942549	-0.028457633	0.13043239	5.068462041	0.555304577	0.906583668	0.95055187	PREDICTED: ras-like protein 1
RS008239	GO:0044699	single-organism process	BP	-0.207787893	-0.060400846	-0.137330675	4.238389148	0.551886149	0.907353012	0.95123648	PREDICTED: ras-related protein Rab-21
RS013041	GO:0044699	single-organism process	BP	0.191903812	0.143123438	0.050232882	7.325389002	0.548105795	0.908202567	0.951960557	PREDICTED: exocyst complex component 5
RS008043	GO:0044699	single-organism process	BP	0.089270351	-0.015942538	-0.065337621	6.129392354	0.545609807	0.908762765	0.952318044	PREDICTED: guanine nucleotide-binding protein subunit beta-5
RS007877	GO:0044699	single-organism process	BP	-0.04006425	0.125386876	0.039173322	5.581612122	0.5433599	0.909267233	0.952321145	PREDICTED: D-aspartate oxidase
RS004214	GO:0044699	single-organism process	BP	-0.139759174	-0.031556346	-0.100933337	6.102350376	0.544251586	0.909067358	0.952321145	PREDICTED: bone morphogenetic protein receptor type-1B
RS009901	GO:0044699	single-organism process	BP	-0.135786167	-0.138189382	-0.17298141	4.923905802	0.544889896	0.908924233	0.952321145	PREDICTED: dephospho-CoA kinase domain-containing protein
RS007670	GO:0044699	single-organism process	BP	0.130593495	0.003348502	0.052901794	6.519330394	0.542845203	0.90938257	0.952321145	PREDICTED: importin-9
RS007682	GO:0044699	single-organism process	BP	0.181703477	-0.010229708	0.051664986	5.162899017	0.537053718	0.910678636	0.95276119	PREDICTED: kinesin-like protein costa
RS000071	GO:0044699	single-organism process	BP	-0.142120932	-0.1661567	-0.111810727	5.400205502	0.537926912	0.910483431	0.95276119	PREDICTED: thioredoxin-related transmembrane protein 2 homolog
RS004110	GO:0044699	single-organism process	BP	-0.109326082	-0.245764423	-0.393853677	2.856144774	0.536964028	0.910698682	0.95276119	PREDICTED: phospholipase A2 inhibitor and Ly6/PLAUR domain-containing protein
RS013412	GO:0044699	single-organism process	BP	-0.596059828	-0.351142315	-0.491311856	0.145573986	0.533065101	0.911569367	0.953276144	PREDICTED: neuropeptides capa receptor-like
RS012170	GO:0044699	single-organism process	BP	0.044376062	-0.029149749	-0.102272667	6.387060354	0.532474838	0.911701051	0.953313256	PREDICTED: rab-like protein 6
RS011838	GO:0044699	single-organism process	BP	0.075328587	0.16154379	0.135711958	4.870803793	0.532227388	0.911756246	0.953313256	PREDICTED: GPI mannosyltransferase 1
RS000933	GO:0044699	single-organism process	BP	0.089195068	0.031072027	0.156423006	5.800088164	0.525786635	0.913190757	0.954654635	PREDICTED: ras-like protein 1
RS011946	GO:0044699	single-organism process	BP	0.167178742	0.07755664	0.302234259	2.38224942	0.524046656	0.913577582	0.954900496	PREDICTED: protein-L-isoaspartate(D-aspartate) O-methyltransferase-like
RS009391	GO:0044699	single-organism process	BP	0.17328093	0.072173211	0.110539737	6.966234536	0.522720262	0.913872256	0.954946215	PREDICTED: von Willebrand factor A domain-containing protein 8
RS013113	GO:0044699	single-organism process	BP	0.046645335	0.245804085	0.153626628	2.756131068	0.522388395	0.913945956	0.954946215	PREDICTED: uridine-cytidine kinase-like 1
RS013206	GO:0044699	single-organism process	BP	0.034480158	0.086958602	-0.090838409	5.052201951	0.521255264	0.914197515	0.954993655	PREDICTED: protein maelstrom homolog
RS012977	GO:0044699	single-organism process	BP	0.07077055	0.009763122	0.187870609	5.309676068	0.519508	0.914585156	0.955240127	PREDICTED: PMS1 protein homolog 1
RS004521	GO:0044699	single-organism process	BP	-0.132126314	-0.000916743	-0.081420203	5.759811737	0.517058893	0.915127978	0.955543481	PREDICTED: general vesicular transport factor p115
RS012006	GO:0044699	single-organism process	BP	-0.401459319	-0.176689266	-0.33799932	1.109446347	0.514024531	0.915799655	0.955874429	PREDICTED: neuroligin-1-like
RS000249	GO:0044699	single-organism process	BP	-0.037902467	-0.184358393	-0.037386112	4.55363631	0.507250978	0.91729554	0.957189111	PREDICTED: rab GTPase-binding effector protein 1
RS015459	GO:0044699	single-organism process	BP	0.206612935	0.259250681	-0.103050012	2.119306922	0.506263052	0.917513308	0.95726639	PREDICTED: synaptic vesicular amine transporter
RS004737	GO:0044699	single-organism process	BP	-0.151180156	0.003237402	0.02197263	4.191432688	0.502613638	0.918316833	0.957419827	PREDICTED: syntaxin-8
RS012409	GO:0044699	single-organism process	BP	0.166649262	0.125364989	0.024833063	4.907461326	0.502930148	0.918247201	0.957419827	PREDICTED: UDP-glucose 4-epimerase
RS012418	GO:0044699	single-organism process	BP	-0.078901438	-0.029572492	-0.266306984	2.339309267	0.496957281	0.919559374	0.957961251	PREDICTED: extracellular signal-regulated kinase 2
RS010097	GO:0044699	single-organism process	BP	0.1168828	0.00617676	0.188326472	3.775780964	0.495590834	0.919859013	0.958126243	PREDICTED: spermine synthase
RS011225	GO:0044699	single-organism process	BP	-0.091534002	-0.117631399	0.051910895	4.273754569	0.491379714	0.920781128	0.958611738	PREDICTED: serine/threonine-protein kinase MARK2-like
RS008845	GO:0044699	single-organism process	BP	-0.010804388	-0.092568632	-0.418289763	0.995903461	0.488737273	0.921358724	0.958974527	PREDICTED: pancreas transcription factor 1 subunit alpha
RS002297	GO:0044699	single-organism process	BP	0.074812549	-0.001009557	0.138813819	5.06516138	0.485522314	0.922060387	0.959149956	PREDICTED: WAS protein family homolog 1
RS010583	GO:0044699	single-organism process	BP	-0.204132095	-0.051330039	0.096105151	3.162457527	0.486088584	0.921936886	0.959149956	PREDICTED: partitioning defective protein 6
RS012100	GO:0044699	single-organism process	BP	0.014405596	-0.329139599	-0.17904822	1.447953403	0.484840248	0.922209095	0.959225416	PREDICTED: ubiquitin carboxyl-terminal hydrolase 22
RS002094	GO:0044699	single-organism process	BP	-0.070547906	-0.064339364	-0.159554953	6.864712402	0.484167197	0.922355784	0.959228097	PREDICTED: aspartate--tRNA ligase, mitochondrial
RS003224	GO:0044699	single-organism process	BP	-0.08323441	-0.480510952	-0.115193841	0.487478781	0.479798997	0.923306535	0.959934677	E3 ubiquitin-protein ligase sia-1
RS011964	GO:0044699	single-organism process	BP	-0.077033518	0.063002549	0.26787124	1.486081763	0.477456988	0.923815355	0.960258535	PREDICTED: MOXD1 homolog 2
RS001839	GO:0044699	single-organism process	BP	-0.04688838	-0.096047629	-0.142499158	5.750316042	0.476768464	0.923964819	0.960258535	PREDICTED: vacuolar protein sorting-associated protein 35
RS009988	GO:0044699	single-organism process	BP	-0.15655041	0.010613303	0.102002539	3.344484246	0.474547854	0.92444648	0.960600575	PREDICTED: tyrosine-protein phosphatase non-receptor type 4
RS013457	GO:0044699	single-organism process	BP	-0.019643874	-0.121686427	-0.132749688	5.531676154	0.466698183	0.926144339	0.96173003	PREDICTED: succinyl-CoA:3-ketoacid coenzyme A transferase 1, mitochondrial
RS000077	GO:0044699	single-organism process	BP	0.022424805	0.042099988	-0.112334789	7.781323573	0.465871302	0.926322751	0.961835991	PREDICTED: AP-2 complex subunit mu
RS001156	GO:0044699	single-organism process	BP	-0.181392398	-0.107611225	0.084069374	3.85316737	0.464681438	0.926579335	0.961964352	PREDICTED: repulsive guidance molecule A-like
RS004255	GO:0044699	single-organism process	BP	-0.198204019	-0.259034991	-0.092048485	10.50660242	0.46288683	0.926965994	0.961964352	PREDICTED: ferritin subunit
RS008674	GO:0044699	single-organism process	BP	-0.056806492	-0.145351105	-0.110188305	5.960621991	0.463181797	0.926902469	0.961964352	PREDICTED: prenylcysteine oxidase-like
RS000629	GO:0044699	single-organism process	BP	0.00837018	0.116143531	-0.015561228	6.338092866	0.458808573	0.927843179	0.962389796	PREDICTED: thioredoxin reductase 3
RS010642	GO:0044699	single-organism process	BP	0.102013501	-0.013594925	-0.08112989	7.265809889	0.456711872	0.928293336	0.962622345	PREDICTED: rab GDP dissociation inhibitor alpha
RS009705	GO:0044699	single-organism process	BP	0.125195543	0.088331077	0.03120065	6.048095277	0.457137741	0.928201948	0.962622345	PREDICTED: vacuolar protein sorting-associated protein 52 homolog
RS006815	GO:0044699	single-organism process	BP	0.108449613	-0.042019997	-0.051907391	4.813656212	0.451359512	0.929439922	0.963316356	PREDICTED: zinc finger protein 708-like
RS014391	GO:0044699	single-organism process	BP	-0.141622911	-0.011559207	-0.104994402	5.056860218	0.449084888	0.92992607	0.963591322	PREDICTED: oxysterol-binding protein-related protein 9
RS004206	GO:0044699	single-organism process	BP	0.000307965	-0.088318853	0.079752893	6.877461006	0.446319742	0.930516142	0.963806258	PREDICTED: dihydrolipoyllysine-residue acetyltransferase component of pyruvate dehydrogenase complex, mitochondrial
RS009649	GO:0044699	single-organism process	BP	-0.036772899	-0.220878583	-0.152031025	3.870532721	0.442143891	0.931405334	0.964172183	PREDICTED: transportin-2
RS010459	GO:0044699	single-organism process	BP	-0.05442216	0.166436102	-0.023821054	4.058899949	0.442266479	0.931379264	0.964172183	PREDICTED: F-actin-capping protein subunit beta
RS000370	GO:0044699	single-organism process	BP	-0.324119489	-0.104677593	0.006917945	5.382292536	0.439925008	0.931876866	0.964422487	PREDICTED: nitric oxide synthase, salivary gland
RS000385	GO:0044699	single-organism process	BP	0.062760932	0.164555437	0.091022996	4.775396122	0.438019762	0.932281217	0.964603158	PREDICTED: DNA repair protein RAD51 homolog 4
RS005846	GO:0044699	single-organism process	BP	0.101171538	0.048311308	-0.06237235	4.773789938	0.438136256	0.932256507	0.964603158	PREDICTED: diphosphomevalonate decarboxylase
RS000725	GO:0044699	single-organism process	BP	0.121486426	0.013260796	0.137270899	7.522004032	0.436242434	0.932657974	0.964685587	PREDICTED: neuropathy target esterase sws
RS001867	GO:0044699	single-organism process	BP	-0.219015309	-0.312481311	-0.195553489	2.164090749	0.435081401	0.932903856	0.964850234	PREDICTED: cadherin-related tumor suppressor
RS014642	GO:0044699	single-organism process	BP	0.159723207	0.060186377	0.052507328	4.3427757	0.427501088	0.934504616	0.965475893	PREDICTED: actin-related protein 6-like
RS001631	GO:0044699	single-organism process	BP	-0.11654592	0.079541288	-0.017499778	3.920893014	0.426035341	0.934813215	0.965704361	PREDICTED: putative tRNA (cytidine(32)/guanosine(34)-2'-O)-methyltransferase
RS000285	GO:0044699	single-organism process	BP	0.119687123	0.031475064	-0.04428715	4.541388379	0.422896761	0.935472985	0.966238561	PREDICTED: facilitated trehalose transporter Tret1-like
RS013716	GO:0044699	single-organism process	BP	0.02204544	-0.074823197	-0.193445943	2.77968161	0.419771439	0.936128566	0.966474263	PREDICTED: YY1-associated factor 2
RS003714	GO:0044699	single-organism process	BP	0.073593867	0.045834412	0.154226165	4.6890794	0.411422784	0.937872837	0.967685606	PREDICTED: deoxycytidylate deaminase
RS009294	GO:0044699	single-organism process	BP	-0.076821145	-0.177523409	-0.041037448	4.402559106	0.401366319	0.939960101	0.968769566	PREDICTED: probable cysteine--tRNA ligase, mitochondrial
RS013432	GO:0044699	single-organism process	BP	0.186161353	0.083115952	0.073559077	4.349613188	0.400756431	0.94008619	0.96877878	PREDICTED: glucose dehydrogenase [FAD, quinone]-like
RS003630	GO:0044699	single-organism process	BP	0.317028447	0.242451352	0.202902983	2.554704633	0.398794912	0.940491325	0.969116982	KRAB-A domain-containing protein
RS009195	GO:0044699	single-organism process	BP	0.040946668	-0.128586278	-0.145576563	3.519298225	0.397500853	0.940758275	0.969237982	PREDICTED: protein piccolo
RS011944	GO:0044699	single-organism process	BP	0.153962405	0.193046383	0.199498955	2.727566063	0.397191897	0.94082197	0.969237982	PREDICTED: protein-L-isoaspartate(D-aspartate) O-methyltransferase
RS008320	GO:0044699	single-organism process	BP	-0.037729257	-0.052265497	0.112994878	4.280613611	0.381723639	0.943991541	0.970591928	PREDICTED: oxysterol-binding protein-related protein 11
RS006226	GO:0044699	single-organism process	BP	-0.000697599	-0.093453208	-0.154614222	4.887176637	0.380294869	0.944282348	0.970591928	PREDICTED: alanine--glyoxylate aminotransferase 2, mitochondrial
RS003038	GO:0044699	single-organism process	BP	0.077168238	-0.005295284	0.118806655	6.256795152	0.377874095	0.944774291	0.970829587	PREDICTED: charged multivesicular body protein 4b
RS009451	GO:0044699	single-organism process	BP	0.099708273	0.02602253	-0.020039705	5.973886745	0.373552171	0.94565014	0.97112357	PREDICTED: sorting nexin lst-4
RS001169	GO:0044699	single-organism process	BP	0.021192247	0.181464539	0.41016591	0.73758366	0.372228166	0.945917821	0.971212216	PREDICTED: neuropeptide FF receptor 2-like
RS007128	GO:0044699	single-organism process	BP	0.030616457	-0.137872106	-0.048843691	6.138938535	0.365078568	0.947358098	0.972178127	PREDICTED: probable multidrug resistance-associated protein lethal(2)03659
RS008532	GO:0044699	single-organism process	BP	-0.287363401	-0.014264649	-0.092533105	2.88092717	0.359345764	0.948506525	0.97283909	Receptor-interacting serine/threonine-protein kinase 1
RS005533	GO:0044699	single-organism process	BP	0.07478252	-0.020377096	0.113953138	5.025280818	0.357239197	0.94892706	0.973052367	PREDICTED: ras-like GTP-binding protein Rho1
RS002326	GO:0044699	single-organism process	BP	0.013609713	0.055420704	-0.067127567	6.572410905	0.357142935	0.948946258	0.973052367	PREDICTED: kinesin-like protein KIF3B
RS009877	GO:0044699	single-organism process	BP	-0.00905179	0.103629481	0.021581239	6.403084093	0.354667887	0.949439292	0.973082555	PREDICTED: integral membrane protein GPR155
RS000806	GO:0044699	single-organism process	BP	-0.127630852	-0.084577756	0.05026157	4.674252953	0.355388053	0.949295947	0.973082555	PREDICTED: nuclear RNA export factor 1-like
RS014144	GO:0044699	single-organism process	BP	-0.121770658	-0.058425415	-0.173533176	3.572086428	0.352409822	0.949888135	0.973384148	PREDICTED: sphingomyelin phosphodiesterase 3-like
RS007821	GO:0044699	single-organism process	BP	4.01981E-05	0.148096469	0.015161294	8.710029191	0.343238755	0.951701461	0.974687176	PREDICTED: histone H3.3-like
RS008675	GO:0044699	single-organism process	BP	-0.016425296	0.110982572	0.092956654	5.295400621	0.342541881	0.951838607	0.974748367	PREDICTED: RPII140-upstream gene protein
RS014134	GO:0044699	single-organism process	BP	0.202412564	0.022912512	0.074608072	10.02891467	0.337859024	0.952757814	0.975354966	PREDICTED: calmodulin
RS010037	GO:0044699	single-organism process	BP	-0.185218212	-0.323706268	-0.222144535	1.208869074	0.337550753	0.952818179	0.975354966	PREDICTED: DNA-binding protein D-ETS-3-like
RS005300	GO:0044699	single-organism process	BP	0.136609392	0.084556551	0.12292061	5.147320274	0.336492194	0.953025322	0.975408444	PREDICTED: probable cytosolic Fe-S cluster assembly factor CPIJ010948
RS007915	GO:0044699	single-organism process	BP	0.174015546	0.154911991	0.062361059	3.358554509	0.329864651	0.954317283	0.975779156	PREDICTED: ras-related protein Rab-35
RS014512	GO:0044699	single-organism process	BP	-0.029889403	-0.146982534	-0.072958369	7.559121721	0.32842394	0.954596993	0.97588441	PREDICTED: cysteine desulfurase, mitochondrial
RS007783	GO:0044699	single-organism process	BP	-0.049575722	0.073396615	-0.039092986	5.629391925	0.325826477	0.95510024	0.976183446	PREDICTED: biotin--protein ligase
RS013747	GO:0044699	single-organism process	BP	-0.097825816	0.022663465	-0.094325017	4.213469082	0.323501709	0.955549509	0.976563379	PREDICTED: hypoxia-inducible factor 1-alpha inhibitor
RS013735	GO:0044699	single-organism process	BP	0.104677793	0.022858359	-0.09563073	2.80861291	0.320642672	0.956100531	0.976888698	PREDICTED: alpha-(1,3)-fucosyltransferase 10
RS012058	GO:0044699	single-organism process	BP	0.052902933	-0.081735984	-0.016513223	4.829080134	0.321347261	0.95596489	0.976888698	PREDICTED: Golgi to ER traffic protein 4 homolog
RS011309	GO:0044699	single-organism process	BP	-0.019311707	0.099298418	0.064239812	4.915784714	0.319686716	0.956284402	0.976930029	PREDICTED: beta-chimaerin
RS008359	GO:0044699	single-organism process	BP	-0.15895413	-0.058889898	-0.119101694	5.018440461	0.318142775	0.956580971	0.976930029	PREDICTED: phosphatidylinositol 4-phosphate 3-kinase C2 domain-containing subunit beta
RS000479	GO:0044699	single-organism process	BP	0.10484998	-0.020725948	0.022448788	5.60863949	0.317285357	0.956745457	0.976939321	PREDICTED: protein maelstrom homolog
RS003843	GO:0044699	single-organism process	BP	-0.149866899	-0.119567997	-0.030363665	3.499148319	0.310983253	0.957949764	0.977604853	Homeobox protein ceh-9
RS007594	GO:0044699	single-organism process	BP	-0.043954148	-0.103763802	0.083984987	7.697619471	0.311465254	0.957857949	0.977604853	PREDICTED: fatty acid synthase
RS001599	GO:0044699	single-organism process	BP	-0.065655632	0.224429989	-0.024901164	1.688688894	0.310898959	0.957965816	0.977604853	Sex determination protein fruitless
RS004720	GO:0044699	single-organism process	BP	-0.146782098	-0.172357517	-0.158017647	3.893542108	0.308665541	0.958390572	0.977721415	PREDICTED: protein O-linked-mannose beta-1,2-N-acetylglucosaminyltransferase 1
RS000601	GO:0044699	single-organism process	BP	0.130347414	0.102232377	0.053386194	5.634424164	0.309673025	0.958199098	0.977721415	PREDICTED: ras-like protein 3
RS007866	GO:0044699	single-organism process	BP	-0.123239502	0.339316842	0.191117175	0.229582542	0.305854213	0.958923727	0.977774901	PREDICTED: protein dachsous-like
RS013160	GO:0044699	single-organism process	BP	0.070991661	-0.1100282	-0.077216007	3.419820009	0.306373925	0.958825294	0.977774901	Suppressor of cytokine signaling 7
RS012220	GO:0044699	single-organism process	BP	-0.029335723	0.050689391	0.128078453	3.427049826	0.305819403	0.958930318	0.977774901	PREDICTED: galactoside 2-alpha-L-fucosyltransferase 3-like
RS007802	GO:0044699	single-organism process	BP	-0.074208781	-0.151087855	-0.139336444	8.750992462	0.304066997	0.959261782	0.977976434	PREDICTED: dynein heavy chain, cytoplasmic
RS001555	GO:0044699	single-organism process	BP	0.090413095	-0.088549935	0.005499565	3.539254563	0.302461129	0.959564946	0.978206324	PREDICTED: ADP-ribosylation factor-related protein 1
RS005074	GO:0044699	single-organism process	BP	-0.193266098	-0.005892579	-0.109917921	2.467034116	0.299037755	0.960209351	0.978704805	PREDICTED: lutropin-choriogonadotropic hormone receptor
RS011848	GO:0044699	single-organism process	BP	0.090463627	-0.079930202	-0.010998879	3.860717882	0.287972307	0.962274462	0.980175071	PREDICTED: mitochondrial inner membrane protease subunit 2

RS002672	GO:0044699	single-organism process	BP	0.112781414	0.136232963	0.12426065	4.219978077	0.286688066	0.96251234	0.980258807	PREDICTED: mitochondrial import inner membrane translocase subunit Tim22
RS008654	GO:0044699	single-organism process	BP	-0.095495158	-0.038135619	0.078540934	3.14330089	0.284037982	0.963002008	0.980519629	PREDICTED: serine/threonine-protein kinase STK11
RS006287	GO:0044699	single-organism process	BP	-0.1653583	0.007454873	0.002789099	2.879341795	0.282691268	0.963250222	0.980613798	PREDICTED: guanine nucleotide-binding protein G(q) subunit alpha
RS008338	GO:0044699	single-organism process	BP	0.14872949	-0.240669395	-0.009563551	0.41802839	0.281319083	0.963502695	0.980712272	PREDICTED: centrosomal and chromosomal factor
RS001209	GO:0044699	single-organism process	BP	0.077544034	-0.042162893	0.04839562	5.846254258	0.278588163	0.964003848	0.981063795	Bifunctional polynucleotide phosphatase/kinase
RS013615	GO:0044699	single-organism process	BP	-0.058342475	-0.14313437	-0.034392008	4.172960075	0.269146752	0.965722705	0.982098817	PREDICTED: ras-like protein 1
RS005492	GO:0044699	single-organism process	BP	0.113658098	0.003287766	-0.018147387	4.461362746	0.267426626	0.966033527	0.982215891	PREDICTED: cell differentiation protein RCD1 homolog
RS007404	GO:0044699	single-organism process	BP	0.341548622	0.423696565	0.486833568	3.079113058	0.267214539	0.9660718	0.982215891	PREDICTED: sodium-independent sulfate anion transporter-like
RS009012	GO:0044699	single-organism process	BP	0.165169583	0.002447419	-0.023721248	2.359677794	0.265786109	0.966329283	0.982398368	PREDICTED: zinc transporter 2-like
RS010152	GO:0044699	single-organism process	BP	-0.129723253	-0.161823215	-0.203053025	1.88639679	0.263658934	0.966711776	0.98246999	PREDICTED: zinc transporter ZIP3
RS001505	GO:0044699	single-organism process	BP	0.078680969	-0.134736852	0.279398738	0.376583472	0.261628451	0.967075826	0.982700975	PREDICTED: gustatory receptor for sugar taste 64f-like
RS002156	GO:0044699	single-organism process	BP	-0.096166135	-0.089844853	-0.038945283	6.136484784	0.256806415	0.967936189	0.982956509	PREDICTED: hexosaminidase D
RS009273	GO:0044699	single-organism process	BP	0.151319695	0.142235856	0.107040205	4.408147966	0.241184076	0.970681917	0.984835903	PREDICTED: neuroligin-1
RS007304	GO:0044699	single-organism process	BP	-0.03874979	-0.117709759	-0.024621974	4.815387852	0.237457293	0.971327222	0.985126901	PREDICTED: solute carrier family 17 member 9
RS014904	GO:0044699	single-organism process	BP	0.045818613	-0.062655331	-0.006051577	5.650113317	0.236403551	0.971508985	0.985126901	PREDICTED: DNA cross-link repair 1A protein
RS012720	GO:0044699	single-organism process	BP	-0.139415748	-0.017410373	-0.236213903	3.849303586	0.236821887	0.971436862	0.985126901	PREDICTED: syntaxin-1A
RS007476	GO:0044699	single-organism process	BP	0.044914002	-0.07417449	-0.014088609	7.111466563	0.228419276	0.972876082	0.986029404	PREDICTED: GTP-binding protein SAR1b
RS005904	GO:0044699	single-organism process	BP	-0.011040745	-0.076944928	0.058252451	6.145465042	0.224633883	0.973517866	0.986292869	Insulin-like peptide receptor
RS000440	GO:0044699	single-organism process	BP	0.057290939	0.025965614	-0.040667409	6.697035751	0.213954489	0.975305737	0.98698276	MFS transporter
RS011312	GO:0044699	single-organism process	BP	0.025050484	0.005525477	0.101396181	4.960673257	0.211785092	0.975664726	0.98722444	PREDICTED: ADP-ribosylation factor-like protein 8B-A
RS013349	GO:0044699	single-organism process	BP	-0.016065637	-0.008469226	-0.094122004	5.383025321	0.211563374	0.975701335	0.98722444	PREDICTED: hydroxymethylglutaryl-CoA synthase 1
RS013089	GO:0044699	single-organism process	BP	-0.22038153	-0.156563146	-0.112341596	3.391286063	0.209547546	0.976033477	0.987388333	PREDICTED: ras association domain-containing protein 8
RS003634	GO:0044699	single-organism process	BP	-0.01323716	-0.0152483	0.09920843	5.517923906	0.208599107	0.976189314	0.987400869	CG12283
RS008629	GO:0044699	single-organism process	BP	0.051370738	-0.258316351	-0.177872255	0.376549123	0.205807073	0.976646438	0.987602807	PREDICTED: probable glutamate receptor
RS002585	GO:0044699	single-organism process	BP	0.061337763	0.015683494	0.119579633	4.655608643	0.205533408	0.976691112	0.987602807	PREDICTED: mitochondrial pyruvate carrier 1
RS010651	GO:0044699	single-organism process	BP	0.10420223	-0.090245357	0.179436123	1.127351457	0.198174181	0.977883508	0.988268304	PREDICTED: L-aminoadipate-semialdehyde dehydrogenase-phosphopantetheinyl transferase
RS008706	GO:0044699	single-organism process	BP	0.063443874	0.058144534	-0.01579188	5.633533699	0.196321115	0.978180999	0.988383465	PREDICTED: ATP-binding cassette sub-family D member 3
RS000287	GO:0044699	single-organism process	BP	-0.130050153	-0.132670171	-0.120971952	2.922402922	0.190091258	0.979172831	0.989064089	PREDICTED: ARF GTPase-activating protein GIT2
RS013112	GO:0044699	single-organism process	BP	0.05541278	-0.05175788	0.105025219	3.490871533	0.190562319	0.979098288	0.989064089	PREDICTED: protein tilB homolog
RS005543	GO:0044699	single-organism process	BP	0.043488647	-0.039284661	0.110504781	4.748144745	0.171087805	0.98211595	0.991087201	PREDICTED: calcium-independent protein kinase C-like
RS007819	GO:0044699	single-organism process	BP	-0.010489001	0.053195155	-0.069169533	4.283628557	0.169132117	0.982411503	0.991117631	PREDICTED: N-acetylgalactosaminyltransferase 6-like
RS004690	GO:0044699	single-organism process	BP	0.010577797	0.147104201	0.053981904	2.401893968	0.169155877	0.98240792	0.991117631	PREDICTED: calcitonin gene-related peptide type 1 receptor-like
RS013043	GO:0044699	single-organism process	BP	-0.013935609	0.061215834	-0.014635593	5.695784627	0.158118062	0.984049167	0.992163574	PREDICTED: cyclin-dependent kinase 20
RS012709	GO:0044699	single-organism process	BP	-0.027523896	-0.136188214	0.011444807	2.83214618	0.151414847	0.985022841	0.992545067	PREDICTED: ADP-ribosylation factor-like protein 3
RS006991	GO:0044699	single-organism process	BP	0.019405506	0.107324217	0.052714682	4.701019396	0.149573617	0.985287141	0.992676747	PREDICTED: tetratricopeptide repeat protein 30A
RS010919	GO:0044699	single-organism process	BP	0.050386763	-0.027549645	-0.017107013	5.185210079	0.144069246	0.98606899	0.992693667	PREDICTED: zinc finger protein Gfi-1b
RS009931	GO:0044699	single-organism process	BP	-0.03797299	-0.085550863	-0.026581632	5.317899556	0.14544473	0.985874789	0.992693667	PREDICTED: intraflagellar transport protein 52 homolog
RS013001	GO:0044699	single-organism process	BP	-0.01460361	-0.111784649	-0.008501333	3.112916539	0.144028996	0.98607466	0.992693667	PREDICTED: condensin-2 complex subunit H2
RS005614	GO:0044699	single-organism process	BP	-0.026120006	0.069729297	0.030053445	4.472662817	0.146084989	0.985784124	0.992693667	PREDICTED: mitochondrial folate transporter/carrier
RS008050	GO:0044699	single-organism process	BP	0.01135696	0.052144522	-0.026243119	6.110921718	0.139562891	0.986699653	0.992848788	PREDICTED: probable arginine--tRNA ligase, mitochondrial
RS005661	GO:0044699	single-organism process	BP	0.019006785	-0.040864734	-0.119648478	2.535793738	0.132989548	0.987603929	0.993282533	PREDICTED: paired box protein Pax-6
RS009037	GO:0044699	single-organism process	BP	0.021100072	0.086349196	-0.007551463	5.274006194	0.132054739	0.987730987	0.993330995	PREDICTED: zinc phosphodiesterase ELAC protein 1-like
RS005997	GO:0044699	single-organism process	BP	0.068035409	-0.077672745	-0.014373732	3.687117976	0.128743502	0.988177895	0.993621749	PREDICTED: serine/threonine-protein kinase Nek7
RS015253	GO:0044699	single-organism process	BP	-0.187002018	-0.048518181	-0.080548556	1.343055934	0.127343932	0.988365299	0.993730846	PREDICTED: longitudinals lacking protein, isoform G-like
RS013888	GO:0044699	single-organism process	BP	-0.159221184	0.130652356	-0.162999387	0.153210176	0.125947719	0.98855136	0.993838575	Uncharacterized protein WH47_11177
RS005772	GO:0044699	single-organism process	BP	-0.105567683	-0.145655697	-0.123138729	2.010194556	0.124936903	0.988685502	0.993894094	PREDICTED: progestin and adipoQ receptor family member 4
RS000130	GO:0044699	single-organism process	BP	-0.028415589	-0.080265702	-0.035810993	5.36706926	0.122477335	0.989009917	0.993982196	PREDICTED: ubiquitin-like modifier-activating enzyme ATG7
RS004254	GO:0044699	single-organism process	BP	-0.026172328	0.069863977	0.113498135	10.91096352	0.122590708	0.988995025	0.993982196	PREDICTED: ferritin heavy polypeptide-like 17
RS006332	GO:0044699	single-organism process	BP	0.048362027	0.064108962	0.021989075	5.885207613	0.115490582	0.989915803	0.994495822	PREDICTED: RNA polymerase II-associated factor 1 homolog
RS009352	GO:0044699	single-organism process	BP	-0.054678595	-0.051909046	-0.070138268	7.168167116	0.11281971	0.990255835	0.994758075	PREDICTED: vacuolar protein sorting-associated protein 4A
RS006046	GO:0044699	single-organism process	BP	0.032603421	-0.052583212	0.006941694	7.328683846	0.106122647	0.991092694	0.995278893	PREDICTED: 97 kDa heat shock protein
RS003766	GO:0044699	single-organism process	BP	0.037221326	0.068863647	0.039138626	6.202580903	0.096224069	0.992286668	0.996091905	PREDICTED: intraflagellar transport protein 57 homolog
RS007567	GO:0044699	single-organism process	BP	0.071173881	-0.014147382	0.078373332	3.16219608	0.094380715	0.992503126	0.996220959	Uncharacterized protein C45G9.7
RS009776	GO:0044699	single-organism process	BP	0.090085711	0.091507347	0.075736594	2.571666375	0.083365319	0.993756023	0.996842959	PREDICTED: thymidylate kinase
RS014503	GO:0044699	single-organism process	BP	0.026137146	-0.011495671	-0.035651636	6.254362366	0.081061626	0.994008923	0.997017233	PREDICTED: kelch-like protein diablo
RS008855	GO:0044699	single-organism process	BP	0.02954866	-0.046352565	0.025055269	3.71091616	0.056927361	0.996448634	0.998351161	PREDICTED: solute carrier family 35 member B1
RS010657	GO:0044699	single-organism process	BP	0.027799466	0.029344701	-0.007157413	5.712009401	0.041629262	0.997769	0.998896998	PREDICTED: DNA polymerase alpha catalytic subunit
RS008734	GO:0044699	single-organism process	BP	0.009238119	-0.031702975	-0.013781447	5.697287383	0.04248393	0.997700532	0.998896998	PREDICTED: cGMP-dependent 3',5'-cyclic phosphodiesterase-like
RS015379	GO:0044699	single-organism process	BP	0.001443853	0.041729538	0.03884543	4.513273638	0.043606131	0.997609626	0.998896998	PREDICTED: vacuolar protein sorting-associated protein 33B
RS009905	GO:0044699	single-organism process	BP	0.043933578	0.057131618	0.032840345	4.976758851	0.048858778	0.997169417	0.998896998	PREDICTED: cdc42 homolog
RS005099	GO:0044699	single-organism process	BP	0.018705784	0.032141182	-0.02482591	4.150966739	0.041603756	0.997771033	0.998896998	PREDICTED: class E basic helix-loop-helix protein 22
RS011601	GO:0044699	single-organism process	BP	0.065232471	0.044854229	0.02428735	3.588838314	0.043711997	0.997600992	0.998896998	PREDICTED: glycine cleavage system H protein, mitochondrial
RS006474	GO:0044699	single-organism process	BP	0.028007466	-0.018976929	-0.009195392	7.361983844	0.037571185	0.998084812	0.998923116	PREDICTED: serine/threonine-protein phosphatase alpha-2 isoform
RS003494	GO:0044699	single-organism process	BP	0.026429046	0.046105564	0.02332654	4.336568543	0.032979113	0.998422808	0.999057433	PREDICTED: syntaxin-16
RS014833	GO:0044699	single-organism process	BP	0.02123593	0.003210275	0.030730838	4.623928817	0.020264711	0.999237411	0.99963428	PREDICTED: tyrosine-protein kinase PR2
RS014627	GO:0044699	single-organism process	BP	-0.067434836	-0.004088457	0.004889792	0.754706582	0.015050832	0.999511123	0.999788699	PREDICTED: prolactin-releasing peptide receptor
RS006774	GO:0044699	single-organism process	BP	-0.005561161	-0.002818033	0.022604747	3.411433614	0.005716578	0.999885243	0.999885243	PREDICTED: equilibrative nucleoside transporter 1

Table S7. Genes belonging to the significant GO terms specifically observed during the worker-presoldier molt.

LogFC.Stage means the log fold-change in expression between each developmental stage and worker/nymph. LR means the likelihood ratio of the full model and the null model that assumes no differences in the expression levels among developmental stages.

seq ID	GO ID	GO Name	GO category	logFC.Pre-GP	logFC.GP	logFC.Molt	logCPM	LR	P Value	FDR	Annotation
RS001442	GO:0016620	oxidoreductase activity, acting on the aldehyde or oxo group of donors, NAD or NADP as acceptor	MF	-1.586399246	2.933634676	4.376004948	9.622554704	76.64892549	1.61E-16	3.86E-14	PREDICTED: putative fatty acyl-CoA reductase CG5065
RS002523	GO:0016620	oxidoreductase activity, acting on the aldehyde or oxo group of donors, NAD or NADP as acceptor	MF	-0.086458424	4.245282488	2.9594565	6.37441706	46.87769729	3.69E-10	1.65E-08	PREDICTED: putative fatty acyl-CoA reductase CG5065
RS011327	GO:0016620	oxidoreductase activity, acting on the aldehyde or oxo group of donors, NAD or NADP as acceptor	MF	4.036858349	1.163915093	-0.59732214	3.649521505	34.49886194	1.55E-07	3.89E-06	PREDICTED: putative fatty acyl-CoA reductase CG5065
RS011853	GO:0016620	oxidoreductase activity, acting on the aldehyde or oxo group of donors, NAD or NADP as acceptor	MF	-1.539711363	-1.980697617	-2.111889965	4.895722934	33.41511771	2.63E-07	6.31E-06	PREDICTED: aldehyde dehydrogenase, dimeric NADP-preferring
RS009516	GO:0016620	oxidoreductase activity, acting on the aldehyde or oxo group of donors, NAD or NADP as acceptor	MF	-1.076031484	1.6558067	1.900095229	6.618233597	29.29942627	1.94E-06	3.64E-05	PREDICTED: putative fatty acyl-CoA reductase CG5065
RS015400	GO:0016620	oxidoreductase activity, acting on the aldehyde or oxo group of donors, NAD or NADP as acceptor	MF	-1.71002934	-3.764076007	-3.05181642	1.406500915	28.16615073	3.35E-06	5.94E-05	PREDICTED: fatty acyl-CoA reductase 1-like
RS009512	GO:0016620	oxidoreductase activity, acting on the aldehyde or oxo group of donors, NAD or NADP as acceptor	MF	1.17375944	2.33350653	-0.141698925	6.950500439	27.16679844	5.43E-06	9.24E-05	PREDICTED: putative fatty acyl-CoA reductase CG5065
RS002448	GO:0016620	oxidoreductase activity, acting on the aldehyde or oxo group of donors, NAD or NADP as acceptor	MF	4.625577563	2.670121748	3.786612691	1.364968679	22.01852978	6.47E-05	0.000819286	PREDICTED: putative fatty acyl-CoA reductase CG5065
RS009515	GO:0016620	oxidoreductase activity, acting on the aldehyde or oxo group of donors, NAD or NADP as acceptor	MF	-1.923477724	-0.346564502	0.335371421	5.902653633	20.76767136	0.000117643	0.001378974	PREDICTED: putative fatty acyl-CoA reductase CG5065
RS007468	GO:0016620	oxidoreductase activity, acting on the aldehyde or oxo group of donors, NAD or NADP as acceptor	MF	1.461435577	0.004846791	0.136545734	8.378746871	19.65605155	0.000200006	0.002180886	PREDICTED: retinal dehydrogenase 1
RS009521	GO:0016620	oxidoreductase activity, acting on the aldehyde or oxo group of donors, NAD or NADP as acceptor	MF	-0.272316809	1.409631421	0.357528897	3.809197435	19.06871797	0.000264598	0.002753187	PREDICTED: putative fatty acyl-CoA reductase CG5065
RS011854	GO:0016620	oxidoreductase activity, acting on the aldehyde or oxo group of donors, NAD or NADP as acceptor	MF	-1.35236157	0.287378657	-0.660124585	6.873563429	16.89098532	0.00074418	0.006720183	PREDICTED: aldehyde dehydrogenase, dimeric NADP-preferring
RS013158	GO:0016620	oxidoreductase activity, acting on the aldehyde or oxo group of donors, NAD or NADP as acceptor	MF	-2.523475416	-0.050651854	-0.622998335	6.75862469	16.44248	0.000920079	0.008069727	PREDICTED: putative fatty acyl-CoA reductase CG5065
RS007469	GO:0016620	oxidoreductase activity, acting on the aldehyde or oxo group of donors, NAD or NADP as acceptor	MF	-1.09984178	-1.575245179	-2.17486518	7.967693554	16.09288204	0.00108533	0.009253045	PREDICTED: retinal dehydrogenase 1
RS010159	GO:0016620	oxidoreductase activity, acting on the aldehyde or oxo group of donors, NAD or NADP as acceptor	MF	-1.336084764	-0.608202522	-1.699159609	2.898762963	13.43912624	0.003777094	0.025772652	PREDICTED: delta-1-pyrroline-5-carboxylate synthase
RS009523	GO:0016620	oxidoreductase activity, acting on the aldehyde or oxo group of donors, NAD or NADP as acceptor	MF	0.414541696	2.484569346	1.258947785	2.192862387	11.63149297	0.008758264	0.050595664	PREDICTED: putative fatty acyl-CoA reductase CG5065
RS011326	GO:0016620	oxidoreductase activity, acting on the aldehyde or oxo group of donors, NAD or NADP as acceptor	MF	-4.201069461	0.569943485	-0.647844648	1.358802356	11.21635139	0.010611701	0.058313068	PREDICTED: putative fatty acyl-CoA reductase CG5065
RS006264	GO:0016620	oxidoreductase activity, acting on the aldehyde or oxo group of donors, NAD or NADP as acceptor	MF	-0.802889558	-0.066245303	2.443772965	1.061581921	11.21401863	0.010623138	0.058350282	PREDICTED: putative fatty acyl-CoA reductase CG5065
RS015500	GO:0016620	oxidoreductase activity, acting on the aldehyde or oxo group of donors, NAD or NADP as acceptor	MF	-1.05332144	-1.708677167	-1.001113459	2.323787627	10.91793546	0.012178008	0.064955049	PREDICTED: fatty acyl-CoA reductase 1-like
RS015505	GO:0016620	oxidoreductase activity, acting on the aldehyde or oxo group of donors, NAD or NADP as acceptor	MF	-0.978556642	-0.958832908	-0.261061582	6.744664605	8.855720098	0.031271963	0.135569652	PREDICTED: probable methylmalonate-semialdehyde dehydrogenase [acylating], mitochondrial
RS001184	GO:0016620	oxidoreductase activity, acting on the aldehyde or oxo group of donors, NAD or NADP as acceptor	MF	-3.515844956	1.387936375	0.631197405	1.396215257	8.202888118	0.041999538	0.169666738	PREDICTED: putative fatty acyl-CoA reductase CG5065
RS008830	GO:0016620	oxidoreductase activity, acting on the aldehyde or oxo group of donors, NAD or NADP as acceptor	MF	-1.546272705	-0.486129482	-0.932581332	1.650402159	6.916340367	0.074612636	0.255385944	PREDICTED: zygotic gap protein knirps-like
RS009519	GO:0016620	oxidoreductase activity, acting on the aldehyde or oxo group of donors, NAD or NADP as acceptor	MF	0.116806708	0.15786105	-0.793527902	6.282026795	6.691617035	0.082404311	0.274103916	PREDICTED: putative fatty acyl-CoA reductase CG8306
RS000318	GO:0016620	oxidoreductase activity, acting on the aldehyde or oxo group of donors, NAD or NADP as acceptor	MF	-0.819793796	-0.410671544	-0.751228336	5.05180063	6.532344839	0.088395594	0.288132316	PREDICTED: alpha-aminoadipic semialdehyde dehydrogenase
RS013434	GO:0016620	oxidoreductase activity, acting on the aldehyde or oxo group of donors, NAD or NADP as acceptor	MF	-0.977659098	-0.021311101	-0.865681572	4.721682556	5.03686848	0.169117274	0.44001451	hypothetical protein
RS009535	GO:0016620	oxidoreductase activity, acting on the aldehyde or oxo group of donors, NAD or NADP as acceptor	MF	-0.747931195	-0.197281827	-0.499138908	6.700058131	4.619934419	0.201838661	0.490936627	PREDICTED: delta-1-pyrroline-5-carboxylate dehydrogenase, mitochondrial
RS001185	GO:0016620	oxidoreductase activity, acting on the aldehyde or oxo group of donors, NAD or NADP as acceptor	MF	-2.562558968	-1.040991284	-1.033231903	-0.160571283	3.372518647	0.337676262	0.658350275	PREDICTED: putative fatty acyl-CoA reductase CG5065
RS002524	GO:0016620	oxidoreductase activity, acting on the aldehyde or oxo group of donors, NAD or NADP as acceptor	MF	0.392469364	1.033888347	0.750805369	4.230147143	3.201207503	0.361631082	0.681142684	PREDICTED: putative fatty acyl-CoA reductase CG5065
RS003568	GO:0016620	oxidoreductase activity, acting on the aldehyde or oxo group of donors, NAD or NADP as acceptor	MF	-0.358954008	-0.163834855	0.276188326	5.413046387	2.747090596	0.432283966	0.74340916	PREDICTED: cytosolic 10-formyltetrahydrofolate dehydrogenase
RS002269	GO:0016620	oxidoreductase activity, acting on the aldehyde or oxo group of donors, NAD or NADP as acceptor	MF	-0.38241426	-0.288426799	-0.399182444	6.660770145	1.912856965	0.590689051	0.853723189	PREDICTED: succinate-semialdehyde dehydrogenase, mitochondrial
RS003241	GO:0016620	oxidoreductase activity, acting on the aldehyde or oxo group of donors, NAD or NADP as acceptor	MF	-0.013202747	-0.118347937	-0.276634704	6.133308229	1.046133956	0.790090825	0.953781304	PREDICTED: aldehyde dehydrogenase family 16 member A1
RS013804	GO:0016620	oxidoreductase activity, acting on the aldehyde or oxo group of donors, NAD or NADP as acceptor	MF	-0.100877476	0.06686151	0.154473621	6.248422898	0.883357177	0.829442134	0.96484922	PREDICTED: programmed cell death protein 10
RS001105	GO:0016620	oxidoreductase activity, acting on the aldehyde or oxo group of donors, NAD or NADP as acceptor	MF	-0.243906454	-0.198530275	-0.240884664	7.38276752	0.660307254	0.882497935	0.979034651	PREDICTED: AP-1 complex subunit beta-1
RS002770	GO:0016620	oxidoreductase activity, acting on the aldehyde or oxo group of donors, NAD or NADP as acceptor	MF	-0.432315078	-0.163031708	-0.288231784	10.27518516	0.594192027	0.89776068	0.982795617	glyceraldehyde-3-phosphate dehydrogenase
RS002932	GO:0016620	oxidoreductase activity, acting on the aldehyde or oxo group of donors, NAD or NADP as acceptor	MF	0.016901916	0.134159103	0.001207983	7.021670537	0.211268982	0.975749919	0.99704905	PREDICTED: aldehyde dehydrogenase, mitochondrial
RS005533	GO:0016620	oxidoreductase activity, acting on the aldehyde or oxo group of donors, NAD or NADP as acceptor	MF	0.056572281	0.084144994	0.064697944	5.012612582	0.076174865	0.994534457	0.999117259	PREDICTED: ras-like GTP-binding protein Rho1
RS001442	GO:0016903	oxidoreductase activity, acting on the aldehyde or oxo group of donors	MF	-1.586399246	2.933634676	4.376004948	9.622554704	76.64892549	1.60564E-16	3.86188E-14	PREDICTED: putative fatty acyl-CoA reductase CG5065
RS002240	GO:0016903	oxidoreductase activity, acting on the aldehyde or oxo group of donors	MF	-0.817495452	1.494943112	1.773626385	5.37490251	70.04797431	4.16856E-15	6.77093E-13	PREDICTED: probable pyruvate dehydrogenase E1 component subunit alpha, mitochondrial
RS002523	GO:0016903	oxidoreductase activity, acting on the aldehyde or oxo group of donors	MF	-0.086458424	4.245282488	2.9594565	6.37441706	46.87769729	3.69016E-10	1.64832E-08	PREDICTED: putative fatty acyl-CoA reductase CG5065
RS011327	GO:0016903	oxidoreductase activity, acting on the aldehyde or oxo group of donors	MF	4.036858349	1.163915093	-0.59732214	3.649521505	34.49886194	1.5545E-07	3.88844E-06	PREDICTED: putative fatty acyl-CoA reductase CG5065
RS011853	GO:0016903	oxidoreductase activity, acting on the aldehyde or oxo group of donors	MF	-1.539711363	-1.980697617	-2.111889965	4.895722934	33.41511771	2.6325E-07	6.30742E-06	PREDICTED: aldehyde dehydrogenase, dimeric NADP-preferring
RS009516	GO:0016903	oxidoreductase activity, acting on the aldehyde or oxo group of donors	MF	-1.076031484	1.6558067	1.900095229	6.618233597	29.29942627	1.93735E-06	3.64368E-05	PREDICTED: putative fatty acyl-CoA reductase CG5065
RS015400	GO:0016903	oxidoreductase activity, acting on the aldehyde or oxo group of donors	MF	-1.71002934	-3.764076007	-3.05181642	1.406500915	28.16615073	3.35176E-06	5.93627E-05	PREDICTED: fatty acyl-CoA reductase 1-like
RS009512	GO:0016903	oxidoreductase activity, acting on the aldehyde or oxo group of donors	MF	1.17375944	2.33350653	-0.141698925	6.950500439	27.16679844	5.43186E-06	9.24303E-05	PREDICTED: putative fatty acyl-CoA reductase CG5065
RS002448	GO:0016903	oxidoreductase activity, acting on the aldehyde or oxo group of donors	MF	4.625577563	2.670121748	3.786612691	1.364968679	22.01852978	6.46546E-05	0.000819286	PREDICTED: putative fatty acyl-CoA reductase CG5065
RS009515	GO:0016903	oxidoreductase activity, acting on the aldehyde or oxo group of donors	MF	-1.923477724	-0.346564502	0.335371421	5.902653633	20.76767136	0.000117643	0.001378974	PREDICTED: putative fatty acyl-CoA reductase CG5065
RS007468	GO:0016903	oxidoreductase activity, acting on the aldehyde or oxo group of donors	MF	1.461435577	0.004846791	0.136545734	8.378746871	19.65605155	0.000200006	0.002180886	PREDICTED: retinal dehydrogenase 1
RS009521	GO:0016903	oxidoreductase activity, acting on the aldehyde or oxo group of donors	MF	-0.272316809	1.409631421	0.357528897	3.809197435	19.06871797	0.000264598	0.002753187	PREDICTED: putative fatty acyl-CoA reductase CG5065
RS011854	GO:0016903	oxidoreductase activity, acting on the aldehyde or oxo group of donors	MF	-1.35236157	0.287378657	-0.660124585	6.873563429	16.89098532	0.00074418	0.006720183	PREDICTED: aldehyde dehydrogenase, dimeric NADP-preferring
RS013158	GO:0016903	oxidoreductase activity, acting on the aldehyde or oxo group of donors	MF	-2.523475416	-0.050651854	-0.622998335	6.75862469	16.44248	0.000920079	0.008069727	PREDICTED: putative fatty acyl-CoA reductase CG5065
RS007469	GO:0016903	oxidoreductase activity, acting on the aldehyde or oxo group of donors	MF	-1.09984178	-1.575245179	-2.17486518	7.967693554	16.09288204	0.00108533	0.009253045	PREDICTED: retinal dehydrogenase 1
RS007611	GO:0016903	oxidoreductase activity, acting on the aldehyde or oxo group of donors	MF	1.032582941	0.639648292	1.069533602	6.357548335	14.5935775	0.002199062	0.016618528	Cytochrome b-c1 complex subunit 2, mitochondrial
RS010159	GO:0016903	oxidoreductase activity, acting on the aldehyde or oxo group of donors	MF	-1.336084764	-0.608202522	-1.699159609	2.898762963	13.43912624	0.003777094	0.025772652	PREDICTED: delta-1-pyrroline-5-carboxylate synthase
RS009523	GO:0016903	oxidoreductase activity, acting on the aldehyde or oxo group of donors	MF	0.414541696	2.484569346	1.258947785	2.192862387	11.63149297	0.008758264	0.050595664	PREDICTED: putative fatty acyl-CoA reductase CG5065
RS011326	GO:0016903	oxidoreductase activity, acting on the aldehyde or oxo group of donors	MF	-4.201069461	0.569943485	-0.647844648	1.358802356	11.21635139	0.010611701	0.058313068	PREDICTED: putative fatty acyl-CoA reductase CG5065
RS006264	GO:0016903	oxidoreductase activity, acting on the aldehyde or oxo group of donors	MF	-0.802889558	-0.066245303	2.443772965	1.061581921	11.21401863	0.010623138	0.058350282	PREDICTED: putative fatty acyl-CoA reductase CG5065
RS015500	GO:0016903	oxidoreductase activity, acting on the aldehyde or oxo group of donors	MF	-1.05332144	-1.708677167	-1.001113459	2.323787627	10.91793546	0.012178008	0.064955049	PREDICTED: fatty acyl-CoA reductase 1-like
RS015505	GO:0016903	oxidoreductase activity, acting on the aldehyde or oxo group of donors	MF	-0.978556642	-0.958832908	-0.261061582	6.744664605	8.855720098	0.031271963	0.135569652	PREDICTED: probable methylmalonate-semialdehyde dehydrogenase [acylating], mitochondrial
RS001184	GO:0016903	oxidoreductase activity, acting on the aldehyde or oxo group of donors	MF	-3.515844956	1.387936375	0.631197405	1.396215257	8.202888118	0.041999538	0.169666738	PREDICTED: putative fatty acyl-CoA reductase CG5065
RS010080	GO:0016903	oxidoreductase activity, acting on the aldehyde or oxo group of donors	MF	-1.270090148	-0.999607277	-1.018639887	9.415700574	6.968026907	0.072923876	0.252089253	PREDICTED: 2-oxoglutarate dehydrogenase, mitochondrial
RS008830	GO:0016903	oxidoreductase activity, acting on the aldehyde or oxo group of donors	MF	-1.546272705	-0.486129482	-0.932581332	1.650402159	6.916340367	0.074612636	0.255385944	PREDICTED: zygotic gap protein knirps-like
RS009519	GO:0016903	oxidoreductase activity, acting on the aldehyde or oxo group of donors	MF	0.116806708	0.15786105	-0.793527902	6.282026795	6.691617035	0.082404311	0.274103916	PREDICTED: putative fatty acyl-CoA reductase CG8306
RS000318	GO:0016903	oxidoreductase activity, acting on the aldehyde or oxo group of donors	MF	-0.819793796	-0.410671544	-0.751228336	5.05180063	6.532344839	0.088395594	0.288132316	PREDICTED: alpha-aminoadipic semialdehyde dehydrogenase
RS013434	GO:0016903	oxidoreductase activity, acting on the aldehyde or oxo group of donors	MF	-0.977659098	-0.021311101	-0.865681572	4.721682556	5.03686848	0.169117274	0.44001451	hypothetical protein
RS009535	GO:0016903	oxidoreductase activity, acting on the aldehyde or oxo group of donors	MF	-0.747931195	-0.197281827	-0.499138908	6.700058131	4.619934419	0.201838661	0.490936627	PREDICTED: delta-1-pyrroline-5-carboxylate dehydrogenase, mitochondrial
RS001185	GO:0016903	oxidoreductase activity, acting on the aldehyde or oxo group of donors	MF	-2.562558968	-1.040991284	-1.033231903	-0.160571283	3.372518647	0.337676262	0.658350275	PREDICTED: putative fatty acyl-CoA reductase CG5065
RS002524	GO:0016903	oxidoreductase activity, acting on the aldehyde or oxo group of donors	MF	0.392469364	1.033888347	0.750805369	4.230147143	3.201207503	0.361631082	0.681142684	PREDICTED: putative fatty acyl-CoA reductase CG5065
RS003568	GO:0016903	oxidoreductase activity, acting on the aldehyde or oxo group of donors	MF	-0.358954008	-0.163834855	0.276188326	5.413046387	2.747090596	0.432283966	0.74340916	PREDICTED: cytosolic 10-formyltetrahydrofolate dehydrogenase
RS005230	GO:0016903	oxidoreductase activity, acting on the aldehyde or oxo group of donors	MF	-0.24608214	-0.146681187	-0.541827929	6.536342957	2.70170332	0.439937911	0.750952525	PREDICTED: probable pyruvate dehydrogenase E1 component subunit alpha, mitochondrial
RS002269	GO:0016903	oxidoreductase activity, acting on the aldehyde or oxo group of donors	MF	-0.38241426	-0.288426799	-0.399182444	6.660770145	1.912856965	0.590689051	0.853723189	PREDICTED: succinate-semialdehyde dehydrogenase, mitochondrial
RS003843	GO:0016903	oxidoreductase activity, acting on the aldehyde or oxo group of donors	MF	-0.227101251	-0.256610399	-0.379542493	3.347277451	1.126030654	0.770794186	0.946428714	Homeobox protein ceh-9
RS006985	GO:0016903	oxidoreductase activity, acting on the aldehyde or oxo group of donors	MF	-0.059879691	0.24664751	-0.060787349	5.004220186	1.086241389	0.780396462	0.950321585	PREDICTED: probable 2-oxoglutarate dehydrogenase E1 component DHKTD1 homolog, mitochondrial
RS003241	GO:0016903	oxidoreductase activity, acting on the aldehyde or oxo group of donors	MF	-0.013202747	-0.118347937	-0.276634704	6.133308229	1.046133956	0.790090825	0.953781304	PREDICTED: aldehyde dehydrogenase family 16 member A1
RS002959	GO:0016903	oxidoreductase activity, acting on the aldehyde or oxo group of donors	MF	0.10849891	-0.210449738	-0.203788709	3.029502993	0.89073147	0.827663923	0.963963396	PREDICTED: 2-oxoisovalerate dehydrogenase subunit beta, mitochondrial
RS013804	GO:0016903	oxidoreductase activity, acting on the aldehyde or oxo group of donors	MF	-0.100877476	0.06686151	0.154473621	6.248422898	0.883357177	0.829442134	0.96484922	PREDICTED: programmed cell death protein 10
RS010234	GO:0016903	oxidoreductase activity, acting on the aldehyde or oxo group of donors	MF	0.230092631	0.197002224	0.300579947	7.113384407	0.67048929	0.880122221	0.97863519	PREDICTED: pyruvate dehydrogenase E1 component subunit beta, mitochondrial
RS001105	GO:0016903	oxidoreductase activity, acting on the aldehyde or oxo group of donors	MF	-0.243906454	-0.198530275	-0.240884664	7.38276752	0.660307254	0.882497935	0.979034651	PREDICTED: AP-1 complex subunit beta-1
RS002770	GO:0016903	oxidoreductase activity, acting on the aldehyde or oxo group of donors	MF	-0.432315078	-0.163031708	-0.288231784	10.27518516	0.594192027	0.89776068	0.982795617	glyceraldehyde-3-phosphate dehydrogenase
RS014156	GO:0016903	oxidoreductase activity, acting on the aldehyde or oxo group of donors	MF	-0.134750199	-0.213308978	-0.063238525	5.674006491	0.421863311	0.935689922	0.989529968	PREDICTED: 2-oxoisovalerate dehydrogenase subunit alpha, mitochondrial
RS002932	GO:0016903	oxidoreductase activity, acting on the aldehyde or oxo group of donors	MF	0.016901916	0.134159103	0.001207983	7.021670537	0.211268982	0.975749919	0.99704905	PREDICTED: aldehyde dehydrogenase, mitochondrial
RS005533	GO:0016903	oxidoreductase activity, acting on the aldehyde or oxo group of donors	MF	0.056572281	0.084144994	0.064697944	5.012612582	0.076174865	0.994534457	0.999117259	PREDICTED: ras-like GTP-binding protein Rho1
RS003846	GO:0071466	cellular response to xenobiotic stimulus	BP	-1.038963412	2.94494667	3.749161043	6.765942147	177.7664	2.67796E-38	1.51845E-36	PREDICTED: sulfotransferase family cytosolic 1B member 1-like
RS012179	GO:0071466	cellular response to xenobiotic stimulus	BP	-0.120198925	-6.829209403	-6.139450628	7.700162804	101.7710374	6.46634E-22	2.01925E-20	PREDICTED: multiple inositol polyphosphate phosphatase 1-like
RS005780	GO:0071466	cellular response to xenobiotic stimulus	BP	-1.090603084	-1.954331392	1.708875507	4.823456587	91.57621383	1.00456E-19	2.80496E-18	PREDICTED: homogentisate 1,2-dioxygenase
RS013159	GO:0071466	cellular response to xenobiotic stimulus	BP	-1.152046773	-1.232208582	2.215346375	3.49161913	50.69445089	5.68312E-11	8.86265E-10	PREDICTED: probable cytochrome P450 4aa1
RS003181	GO:0071466	cellular response to xenobiotic stimulus	BP	-1.140903314	-3.135515894	-2.809886681	3.020558658	43.70092224	1.7469E-09	2.42057E-08	PREDICTED: serine/threonine-protein phosphatase alpha-2 isoform
RS011853	GO:0071466	cellular response to xenobiotic stimulus	BP	-0.198391061	-2.402376009	-1.315737207	5.883199768	42.14749002	3.73315E-09	5.06955E-08	PREDICTED: aldehyde dehydrogenase, dimeric NADP-preferring
RS001544	GO:0071466	cellular response to xenobiotic stimulus	BP	0.274963904	-1.09360695	-1.805361323	6.749172802	41.74350775	4.54779E-09	6.11798E-08	PREDICTED: alkaline phosphatase 4-like
RS012906	GO:0071466	cellular response to xenobiotic stimulus	BP	-1.065702124	1.788880632	1.156501433	6.060912588	30.43739468	1.11653E-06	1.13927E-05	Multiple inositol polyphosphate phosphatase 1
RS010696	GO:0071466	cellular response to xenobiotic stimulus	BP	-0.461445339	-1.655551863	-0.178166149	5.088306104	29.87793362	1.4641E-06	1.4799E-05	PREDICTED: fumarylacetoacetase
RS012907	GO:0071466	cellular response to xenobiotic stimulus	BP	-0.210791938	1.703354709	1.009332145	6.58618481	24.34301467	2.11817E-05	0.000183169	PREDICTED: multiple inositol polyphosphate phosphatase 1-like
RS007468	GO:0071466	cellular response to xenobiotic stimulus	BP	1.554961358	-0.015902902	0.482470573	9.656522243	22.44534397	5.26932E-05	0.0004279	PREDICTED: retinal dehydrogenase 1
RS009553	GO:0071466	cellular response to xenobiotic stimulus	BP	0.220029961	1.148171689	1.314074443	7.69406935	22.24804933	5.79204E-05	0.000467704	PREDICTED: prostatic acid phosphatase-like
RS010605	GO:0071466	cellular response to xenobiotic stimulus	BP	-1.737053607	-4.040674622	-2.749734436	0.598060261	20.07234291	0.000163982	0.00122683	cytochrome P450
RS008160	GO:0071466	cellular response to xenobiotic stimulus	BP	-2.41543084	1.144946968	-0.139456084	1.085418032	16.94702776	0.000724694	0.00484178	PREDICTED: UDP-glucuronosyltransferase 2C1-like
RS015505	GO:0071466	cellular response to xenobiotic stimulus	BP	-0.333963264	-1.254068775	-0.333900304	7.203178713	16.32816273	0.000971162	0.006353837	PREDICTED: probable methylmalonate-semialdehyde dehydrogenase [acylating], mitochondrial
RS012905	GO:0071466	cellular response to xenobiotic stimulus	BP	-0.444917968	0.744827628	0.362813316	5.719209673	15.03421911	0.001787639	0.011076502	PREDICTED: multiple inositol polyphosphate phosphatase 1-like
RS012908	GO:0071466	cellular response to xenobiotic stimulus	BP	-0.148291307	1.999497182	1.3045129	7.135023881	14.83576019	0.001962521	0.012024391	PREDICTED: multiple inositol polyphosphate phosphatase 1-like
RS007469	GO:0071466	cellular response to xenobiotic stimulus	BP	0.109921824	-1.512321655	-1.260766781	10.37063405	14.46509492	0.002335814	0.014022266	PREDICTED: retinal dehydrogenase 1
RS013640	GO:0071466	cellular response to xenobiotic stimulus	BP	-0.507605075	0.88378812	-0.370589145	3.007371753	13.90545704	0.003036708	0.017788199	PREDICTED: alkaline phosphatase-like
RS002859	GO:0071466	cellular response to xenobiotic stimulus	BP	0.189250546	0.851032571	0.013876855	6.303317671	12.79585099	0.005099547	0.028169544	PREDICTED: multiple inositol polyphosphate phosphatase 1
RS006561	GO:0071466	cellular response to xenobiotic stimulus	BP	0.564379861	-0.323284027	0.338585535	5.035478156	8.86647187	0.031119918	0.133882964	PREDICTED: EH domain-containing protein 3
RS003528	GO:0071466	cellular response to xenobiotic stimulus	BP	0.294815966	1.5271518	0.691426504	9.664094871	7.861102029	0.048971246	0.194321752	PREDICTED: multiple inositol polyphosphate phosphatase 1-like
RS011854	GO:0071466	cellular response to xenobiotic stimulus	BP	-0.744270551	0.041529434	-0.702768939	7.795747502	7.164883594	0.066823987	0.248104681	PREDICTED: aldehyde dehydrogenase, dimeric NADP-preferring
RS013013	GO:0071466	cellular response to xenobiotic stimulus	BP	0.208074223	1.342259058	-0.041659798	4.182064207	6.279402018	0.09878034	0.33526706	PREDICTED: peptidoglycan-recognition protein LB-like
RS003393	GO:0071466	cellular response to xenobiotic stimulus	BP	0.293569646	-0.648014171	-0.531549171	8.391513924	5.38529422	0.145662549	0.443610233	PREDICTED: leukotriene A-4 hydrolase
RS002767	GO:0071466	cellular response to xenobiotic stimulus	BP	-0.067461427	-0.59993482	-0.658322096	3.142861555	5.233644143	0.155466538	0.461933213	PREDICTED: 2-phosphoxylose phosphatase 1
RS009422	GO:0071466	cellular response to xenobiotic stimulus	BP	0.093862126	0.614096232	0.365855749	4.493355272	4.639942739	0.200142251	0.54911694	PREDICTED: peptidylglycine alpha-hydroxylating monooxygenase

RS003226	GO:0071466	cellular response to xenobiotic stimulus	BP	-0.415048721	-1.598366462	-0.067839473	0.542153418	3.714543094	0.293983901	0.692297595	PREDICTED: putative nuclease HARBI1
RS010277	GO:0071466	cellular response to xenobiotic stimulus	BP	0.43294049	-0.156404058	-0.063878996	4.226356086	3.715171891	0.29390844	0.692297595	PREDICTED: low molecular weight phosphotyrosine protein phosphatase-like
RS002164	GO:0071466	cellular response to xenobiotic stimulus	BP	0.51839823	-0.089308584	-0.136548467	5.351204227	3.691790815	0.296726044	0.695145504	PREDICTED: iron/zinc purple acid phosphatase-like protein
RS013622	GO:0071466	cellular response to xenobiotic stimulus	BP	1.304002696	-0.790491216	0.213979312	6.381917919	3.432062346	0.329680888	0.734589515	PREDICTED: membrane-bound alkaline phosphatase-like
RS009537	GO:0071466	cellular response to xenobiotic stimulus	BP	0.212069599	-0.420308914	-0.225831017	5.76759198	3.392997346	0.33490746	0.740507361	PREDICTED: prostatic acid phosphatase-like
RS002932	GO:0071466	cellular response to xenobiotic stimulus	BP	0.285330244	-0.183360297	-0.196925118	7.264005384	2.38943308	0.495604683	0.88489721	PREDICTED: aldehyde dehydrogenase, mitochondrial
RS006563	GO:0071466	cellular response to xenobiotic stimulus	BP	0.29335232	-0.606847586	-0.072946631	5.352277354	2.119455869	0.547986748	0.912111842	PREDICTED: EH domain-containing protein 3
RS002269	GO:0071466	cellular response to xenobiotic stimulus	BP	-0.115899934	-0.045489339	-0.35700385	6.156428651	1.746693481	0.626603143	0.953618312	PREDICTED: succinate-semialdehyde dehydrogenase, mitochondrial
RS002165	GO:0071466	cellular response to xenobiotic stimulus	BP	-0.196863778	-0.335644238	-0.4763521	6.442896406	1.665734489	0.644578476	0.962808844	PREDICTED: iron/zinc purple acid phosphatase-like protein
RS009535	GO:0071466	cellular response to xenobiotic stimulus	BP	-0.056065203	0.092504311	-0.098751469	6.062674479	0.266403412	0.966218072	0.998581907	PREDICTED: delta-1-pyrroline-5-carboxylate dehydrogenase, mitochondrial
RS005799	GO:0071466	cellular response to xenobiotic stimulus	BP	0.113612619	-0.164027646	0.198582604	3.989699328	1.050959988	0.788923734	0.998581907	PREDICTED: probable maleylacetoacetate isomerase 2
RS010395	GO:0071466	cellular response to xenobiotic stimulus	BP	-0.341120196	-0.109016823	-0.088861773	2.332771442	0.616668735	0.892606069	0.998581907	PREDICTED: beta-1-syntrophin
RS006474	GO:0071466	cellular response to xenobiotic stimulus	BP	0.045697876	-0.089721002	-0.017504278	7.527263086	0.144001552	0.986078527	0.999271462	PREDICTED: serine/threonine-protein phosphatase alpha-2 isoform
RS003846	GO:0009410	response to xenobiotic stimulus	BP	-1.038963412	2.94494667	3.749161043	6.765942147	177.7664	2.67796E-38	1.51845E-36	PREDICTED: sulfotransferase family cytosolic 1B member 1-like
RS012179	GO:0009410	response to xenobiotic stimulus	BP	-0.120198925	-6.829209403	-6.139450628	7.700162804	101.7710374	6.46634E-22	2.01925E-20	PREDICTED: multiple inositol polyphosphate phosphatase 1-like
RS005780	GO:0009410	response to xenobiotic stimulus	BP	-1.090603084	-1.954331392	1.708875507	4.823456587	91.57621383	1.00456E-19	2.80496E-18	PREDICTED: homogentisate 1,2-dioxygenase
RS013159	GO:0009410	response to xenobiotic stimulus	BP	-1.152046773	-1.232208582	2.215346375	3.49161913	50.69445089	5.68312E-11	8.86265E-10	PREDICTED: probable cytochrome P450 4aa1
RS003181	GO:0009410	response to xenobiotic stimulus	BP	-1.140903314	-3.135515894	-2.809886681	3.020558658	43.70092224	1.7469E-09	2.42057E-08	PREDICTED: serine/threonine-protein phosphatase alpha-2 isoform
RS011853	GO:0009410	response to xenobiotic stimulus	BP	-0.198391061	-2.402376009	-1.315737207	5.883199768	42.14749002	3.73315E-09	5.06955E-08	PREDICTED: aldehyde dehydrogenase, dimeric NADP-preferring
RS001544	GO:0009410	response to xenobiotic stimulus	BP	0.274963904	-1.09360695	-1.805361323	6.749172802	41.74350775	4.54779E-09	6.11798E-08	PREDICTED: alkaline phosphatase 4-like
RS012906	GO:0009410	response to xenobiotic stimulus	BP	-1.065702124	1.788880632	1.156501433	6.060912588	30.43739468	1.11653E-06	1.13927E-05	Multiple inositol polyphosphate phosphatase 1
RS010696	GO:0009410	response to xenobiotic stimulus	BP	-0.461445339	-1.655551863	-0.178166149	5.088306104	29.87793362	1.4641E-06	1.4799E-05	PREDICTED: fumarylacetoacetase
RS012907	GO:0009410	response to xenobiotic stimulus	BP	-0.210791938	1.703354709	1.009332145	6.58618481	24.34301467	2.11817E-05	0.000183169	PREDICTED: multiple inositol polyphosphate phosphatase 1-like
RS007468	GO:0009410	response to xenobiotic stimulus	BP	1.554961358	-0.015902902	0.482470573	9.656522243	22.44534397	5.26932E-05	0.0004279	PREDICTED: retinal dehydrogenase 1
RS009553	GO:0009410	response to xenobiotic stimulus	BP	0.220029961	1.148171689	1.314074443	7.69406935	22.24804933	5.79204E-05	0.000467704	PREDICTED: prostatic acid phosphatase-like
RS010605	GO:0009410	response to xenobiotic stimulus	BP	-1.737053607	-4.040674622	-2.749734436	0.598060261	20.07234291	0.000163982	0.00122683	cytochrome P450
RS008160	GO:0009410	response to xenobiotic stimulus	BP	-2.41543084	1.144946968	-0.139456084	1.085418032	16.94702776	0.000724694	0.00484178	PREDICTED: UDP-glucuronosyltransferase 2C1-like
RS015505	GO:0009410	response to xenobiotic stimulus	BP	-0.333963264	-1.254068775	-0.333900304	7.203178713	16.32816273	0.000971162	0.006353837	PREDICTED: probable methylmalonate-semialdehyde dehydrogenase [acylating], mitochondrial
RS012905	GO:0009410	response to xenobiotic stimulus	BP	-0.444917968	0.744827628	0.362813316	5.719209673	15.03421911	0.001787639	0.011076502	PREDICTED: multiple inositol polyphosphate phosphatase 1-like
RS012908	GO:0009410	response to xenobiotic stimulus	BP	-0.148291307	1.999497182	1.3045129	7.135023881	14.83576019	0.001962521	0.012024391	PREDICTED: multiple inositol polyphosphate phosphatase 1-like
RS007469	GO:0009410	response to xenobiotic stimulus	BP	0.109921824	-1.512321655	-1.260766781	10.37063405	14.46509492	0.002335814	0.014022266	PREDICTED: retinal dehydrogenase 1
RS013640	GO:0009410	response to xenobiotic stimulus	BP	-0.507605075	0.88378812	-0.370589145	3.007371753	13.90545704	0.003036708	0.017788199	PREDICTED: alkaline phosphatase-like
RS002859	GO:0009410	response to xenobiotic stimulus	BP	0.189250546	0.851032571	0.013876855	6.303317671	12.79585099	0.005099547	0.028169544	PREDICTED: multiple inositol polyphosphate phosphatase 1
RS006561	GO:0009410	response to xenobiotic stimulus	BP	0.564379861	-0.323284027	0.338585535	5.035478156	8.86647187	0.031119918	0.133882964	PREDICTED: EH domain-containing protein 3
RS003528	GO:0009410	response to xenobiotic stimulus	BP	0.294815966	1.5271518	0.691426504	9.664094871	7.861102029	0.048971246	0.194321752	PREDICTED: multiple inositol polyphosphate phosphatase 1-like
RS011854	GO:0009410	response to xenobiotic stimulus	BP	-0.744270551	0.041529434	-0.702768939	7.795747502	7.164883594	0.066823987	0.248104681	PREDICTED: aldehyde dehydrogenase, dimeric NADP-preferring
RS013013	GO:0009410	response to xenobiotic stimulus	BP	0.208074223	1.342259058	-0.041659798	4.182064207	6.279402018	0.09878034	0.33526706	PREDICTED: peptidoglycan-recognition protein LB-like
RS003393	GO:0009410	response to xenobiotic stimulus	BP	0.293569646	-0.648014171	-0.531549171	8.391513924	5.38529422	0.145662549	0.443610233	PREDICTED: leukotriene A-4 hydrolase
RS002767	GO:0009410	response to xenobiotic stimulus	BP	-0.067461427	-0.59993482	-0.658322096	3.142861555	5.233644143	0.155466538	0.461933213	PREDICTED: 2-phosphoxylose phosphatase 1
RS009422	GO:0009410	response to xenobiotic stimulus	BP	0.093862126	0.614096232	0.365855749	4.493355272	4.639942739	0.200142251	0.54911694	PREDICTED: peptidylglycine alpha-hydroxylating monooxygenase
RS003226	GO:0009410	response to xenobiotic stimulus	BP	-0.415048721	-1.598366462	-0.067839473	0.542153418	3.714543094	0.293983901	0.692297595	PREDICTED: putative nuclease HARBI1
RS010277	GO:0009410	response to xenobiotic stimulus	BP	0.43294049	-0.156404058	-0.063878996	4.226356086	3.715171891	0.29390844	0.692297595	PREDICTED: low molecular weight phosphotyrosine protein phosphatase-like
RS002164	GO:0009410	response to xenobiotic stimulus	BP	0.51839823	-0.089308584	-0.136548467	5.351204227	3.691790815	0.296726044	0.695145504	PREDICTED: iron/zinc purple acid phosphatase-like protein
RS013622	GO:0009410	response to xenobiotic stimulus	BP	1.304002696	-0.790491216	0.213979312	6.381917919	3.432062346	0.329680888	0.734589515	PREDICTED: membrane-bound alkaline phosphatase-like
RS009537	GO:0009410	response to xenobiotic stimulus	BP	0.212069599	-0.420308914	-0.225831017	5.76759198	3.392997346	0.33490746	0.740507361	PREDICTED: prostatic acid phosphatase-like
RS002932	GO:0009410	response to xenobiotic stimulus	BP	0.285330244	-0.183360297	-0.196925118	7.264005384	2.38943308	0.495604683	0.88489721	PREDICTED: aldehyde dehydrogenase, mitochondrial
RS006563	GO:0009410	response to xenobiotic stimulus	BP	0.29335232	-0.606847586	-0.072946631	5.352277354	2.119455869	0.547986748	0.912111842	PREDICTED: EH domain-containing protein 3
RS002269	GO:0009410	response to xenobiotic stimulus	BP	-0.115899934	-0.045489339	-0.35700385	6.156428651	1.746693481	0.626603143	0.953618312	PREDICTED: succinate-semialdehyde dehydrogenase, mitochondrial
RS002165	GO:0009410	response to xenobiotic stimulus	BP	-0.196863778	-0.335644238	-0.4763521	6.442896406	1.665734489	0.644578476	0.962808844	PREDICTED: iron/zinc purple acid phosphatase-like protein
RS010395	GO:0009410	response to xenobiotic stimulus	BP	-0.341120196	-0.109016823	-0.088861773	2.332771442	0.616668735	0.892606069	0.998581907	PREDICTED: beta-1-syntrophin
RS009535	GO:0009410	response to xenobiotic stimulus	BP	-0.056065203	0.092504311	-0.098751469	6.062674479	0.266403412	0.966218072	0.998581907	PREDICTED: delta-1-pyrroline-5-carboxylate dehydrogenase, mitochondrial
RS005799	GO:0009410	response to xenobiotic stimulus	BP	0.113612619	-0.164027646	0.198582604	3.989699328	1.050959988	0.788923734	0.998581907	PREDICTED: probable maleylacetoacetate isomerase 2
RS006474	GO:0009410	response to xenobiotic stimulus	BP	0.045697876	-0.089721002	-0.017504278	7.527263086	0.144001552	0.986078527	0.999271462	PREDICTED: serine/threonine-protein phosphatase alpha-2 isoform
RS003846	GO:0006805	xenobiotic metabolic process	BP	-1.038963412	2.94494667	3.749161043	6.765942147	177.7664	2.67796E-38	1.51845E-36	PREDICTED: sulfotransferase family cytosolic 1B member 1-like
RS012179	GO:0006805	xenobiotic metabolic process	BP	-0.120198925	-6.829209403	-6.139450628	7.700162804	101.7710374	6.46634E-22	2.01925E-20	PREDICTED: multiple inositol polyphosphate phosphatase 1-like
RS005780	GO:0006805	xenobiotic metabolic process	BP	-1.090603084	-1.954331392	1.708875507	4.823456587	91.57621383	1.00456E-19	2.80496E-18	PREDICTED: homogentisate 1,2-dioxygenase
RS013159	GO:0006805	xenobiotic metabolic process	BP	-1.152046773	-1.232208582	2.215346375	3.49161913	50.69445089	5.68312E-11	8.86265E-10	PREDICTED: probable cytochrome P450 4aa1
RS003181	GO:0006805	xenobiotic metabolic process	BP	-1.140903314	-3.135515894	-2.809886681	3.020558658	43.70092224	1.7469E-09	2.42057E-08	PREDICTED: serine/threonine-protein phosphatase alpha-2 isoform
RS011853	GO:0006805	xenobiotic metabolic process	BP	-0.198391061	-2.402376009	-1.315737207	5.883199768	42.14749002	3.73315E-09	5.06955E-08	PREDICTED: aldehyde dehydrogenase, dimeric NADP-preferring
RS001544	GO:0006805	xenobiotic metabolic process	BP	0.274963904	-1.09360695	-1.805361323	6.749172802	41.74350775	4.54779E-09	6.11798E-08	PREDICTED: alkaline phosphatase 4-like
RS012906	GO:0006805	xenobiotic metabolic process	BP	-1.065702124	1.788880632	1.156501433	6.060912588	30.43739468	1.11653E-06	1.13927E-05	Multiple inositol polyphosphate phosphatase 1
RS010696	GO:0006805	xenobiotic metabolic process	BP	-0.461445339	-1.655551863	-0.178166149	5.088306104	29.87793362	1.4641E-06	1.4799E-05	PREDICTED: fumarylacetoacetase
RS012907	GO:0006805	xenobiotic metabolic process	BP	-0.210791938	1.703354709	1.009332145	6.58618481	24.34301467	2.11817E-05	0.000183169	PREDICTED: multiple inositol polyphosphate phosphatase 1-like
RS007468	GO:0006805	xenobiotic metabolic process	BP	1.554961358	-0.015902902	0.482470573	9.656522243	22.44534397	5.26932E-05	0.0004279	PREDICTED: retinal dehydrogenase 1
RS009553	GO:0006805	xenobiotic metabolic process	BP	0.220029961	1.148171689	1.314074443	7.69406935	22.24804933	5.79204E-05	0.000467704	PREDICTED: prostatic acid phosphatase-like
RS010605	GO:0006805	xenobiotic metabolic process	BP	-1.737053607	-4.040674622	-2.749734436	0.598060261	20.07234291	0.000163982	0.00122683	cytochrome P450
RS008160	GO:0006805	xenobiotic metabolic process	BP	-2.41543084	1.144946968	-0.139456084	1.085418032	16.94702776	0.000724694	0.00484178	PREDICTED: UDP-glucuronosyltransferase 2C1-like
RS015505	GO:0006805	xenobiotic metabolic process	BP	-0.333963264	-1.254068775	-0.333900304	7.203178713	16.32816273	0.000971162	0.006353837	PREDICTED: probable methylmalonate-semialdehyde dehydrogenase [acylating], mitochondrial
RS012905	GO:0006805	xenobiotic metabolic process	BP	-0.444917968	0.744827628	0.362813316	5.719209673	15.03421911	0.001787639	0.011076502	PREDICTED: multiple inositol polyphosphate phosphatase 1-like
RS012908	GO:0006805	xenobiotic metabolic process	BP	-0.148291307	1.999497182	1.3045129	7.135023881	14.83576019	0.001962521	0.012024391	PREDICTED: multiple inositol polyphosphate phosphatase 1-like
RS007469	GO:0006805	xenobiotic metabolic process	BP	0.109921824	-1.512321655	-1.260766781	10.37063405	14.46509492	0.002335814	0.014022266	PREDICTED: retinal dehydrogenase 1
RS013640	GO:0006805	xenobiotic metabolic process	BP	-0.507605075	0.88378812	-0.370589145	3.007371753	13.90545704	0.003036708	0.017788199	PREDICTED: alkaline phosphatase-like
RS002859	GO:0006805	xenobiotic metabolic process	BP	0.189250546	0.851032571	0.013876855	6.303317671	12.79585099	0.005099547	0.028169544	PREDICTED: multiple inositol polyphosphate phosphatase 1
RS006561	GO:0006805	xenobiotic metabolic process	BP	0.564379861	-0.323284027	0.338585535	5.035478156	8.86647187	0.031119918	0.133882964	PREDICTED: EH domain-containing protein 3
RS003528	GO:0006805	xenobiotic metabolic process	BP	0.294815966	1.5271518	0.691426504	9.664094871	7.861102029	0.048971246	0.194321752	PREDICTED: multiple inositol polyphosphate phosphatase 1-like
RS011854	GO:0006805	xenobiotic metabolic process	BP	-0.744270551	0.041529434	-0.702768939	7.795747502	7.164883594	0.066823987	0.248104681	PREDICTED: aldehyde dehydrogenase, dimeric NADP-preferring
RS013013	GO:0006805	xenobiotic metabolic process	BP	0.208074223	1.342259058	-0.041659798	4.182064207	6.279402018	0.09878034	0.33526706	PREDICTED: peptidoglycan-recognition protein LB-like
RS003393	GO:0006805	xenobiotic metabolic process	BP	0.293569646	-0.648014171	-0.531549171	8.391513924	5.38529422	0.145662549	0.443610233	PREDICTED: leukotriene A-4 hydrolase
RS002767	GO:0006805	xenobiotic metabolic process	BP	-0.067461427	-0.59993482	-0.658322096	3.142861555	5.233644143	0.155466538	0.461933213	PREDICTED: 2-phosphoxylose phosphatase 1
RS009422	GO:0006805	xenobiotic metabolic process	BP	0.093862126	0.614096232	0.365855749	4.493355272	4.639942739	0.200142251	0.54911694	PREDICTED: peptidylglycine alpha-hydroxylating monooxygenase
RS003226	GO:0006805	xenobiotic metabolic process	BP	-0.415048721	-1.598366462	-0.067839473	0.542153418	3.714543094	0.293983901	0.692297595	PREDICTED: putative nuclease HARBI1
RS010277	GO:0006805	xenobiotic metabolic process	BP	0.43294049	-0.156404058	-0.063878996	4.226356086	3.715171891	0.29390844	0.692297595	PREDICTED: low molecular weight phosphotyrosine protein phosphatase-like
RS002164	GO:0006805	xenobiotic metabolic process	BP	0.51839823	-0.089308584	-0.136548467	5.351204227	3.691790815	0.296726044	0.695145504	PREDICTED: iron/zinc purple acid phosphatase-like protein
RS013622	GO:0006805	xenobiotic metabolic process	BP	1.304002696	-0.790491216	0.213979312	6.381917919	3.432062346	0.329680888	0.734589515	PREDICTED: membrane-bound alkaline phosphatase-like
RS009537	GO:0006805	xenobiotic metabolic process	BP	0.212069599	-0.420308914	-0.225831017	5.76759198	3.392997346	0.33490746	0.740507361	PREDICTED: prostatic acid phosphatase-like
RS002932	GO:0006805	xenobiotic metabolic process	BP	0.285330244	-0.183360297	-0.196925118	7.264005384	2.38943308	0.495604683	0.88489721	PREDICTED: aldehyde dehydrogenase, mitochondrial
RS006563	GO:0006805	xenobiotic metabolic process	BP	0.29335232	-0.606847586	-0.072946631	5.352277354	2.119455869	0.547986748	0.912111842	PREDICTED: EH domain-containing protein 3
RS002269	GO:0006805	xenobiotic metabolic process	BP	-0.115899934	-0.045489339	-0.35700385	6.156428651	1.746693481	0.626603143	0.953618312	PREDICTED: succinate-semialdehyde dehydrogenase, mitochondrial
RS002165	GO:0006805	xenobiotic metabolic process	BP	-0.196863778	-0.335644238	-0.4763521	6.442896406	1.665734489	0.644578476	0.962808844	PREDICTED: iron/zinc purple acid phosphatase-like protein
RS005799	GO:0006805	xenobiotic metabolic process	BP	0.113612619	-0.164027646	0.198582604	3.989699328	1.050959988	0.788923734	0.998581907	PREDICTED: probable maleylacetoacetate isomerase 2
RS010395	GO:0006805	xenobiotic metabolic process	BP	-0.341120196	-0.109016823	-0.088861773	2.332771442	0.616668735	0.892606069	0.998581907	PREDICTED: beta-1-syntrophin
RS009535	GO:0006805	xenobiotic metabolic process	BP	-0.056065203	0.092504311	-0.098751469	6.062674479	0.266403412	0.966218072	0.998581907	PREDICTED: delta-1-pyrroline-5-carboxylate dehydrogenase, mitochondrial
RS006474	GO:0006805	xenobiotic metabolic process	BP	0.045697876	-0.089721002	-0.017504278	7.527263086	0.144001552	0.986078527	0.999271462	PREDICTED: serine/threonine-protein phosphatase alpha-2 isoform
RS004161	GO:0006720	isoprenoid metabolic process	BP	0.505016069	3.835932721	3.267455071	4.506016406	133.4840545	9.59795E-29	4.12233E-27	PREDICTED: decaprenyl-diphosphate synthase subunit 1
RS001887	GO:0006720	isoprenoid metabolic process	BP	-2.121970627	-0.158682886	2.897491979	3.147887764	119.0269144	1.25026E-25	4.76795E-24	PREDICTED: protein croquemort-like
RS000980	GO:0006720	isoprenoid metabolic process	BP	-0.153209218	-5.17967259	-1.399075209	7.685275768	113.1637433	2.28764E-24	8.2842E-23	PREDICTED: probable cytochrome P450 305a1
RS012767	GO:0006720	isoprenoid metabolic process	BP	-1.335505277	1.927739084	1.097273196	5.721508311	82.36809216	9.52786E-18	2.35069E-16	PREDICTED: D-beta-hydroxybutyrate dehydrogenase, mitochondrial
RS004588	GO:0006720	isoprenoid metabolic process	BP	0.65072192	2.625374347	0.335731048	6.374966282	80.93341444	1.93556E-17	4.73023E-16	PREDICTED: decaprenyl-diphosphate synthase subunit 2
RS100014	GO:0006720	isoprenoid metabolic process	BP	6.044492419	-0.931046675	-0.57189216	3.244642924	69.48745755	5.49546E-15	1.13855E-13	PREDICTED: geranylgeranyl pyrophosphate synthase
RS001883	GO:0006720	isoprenoid metabolic process	BP	0.667857981	-3.252550219	-1.218789822	6.226556851	58.66456333	1.13371E-12	1.99681E-11	PREDICTED: protein croquemort-like
RS007861	GO:0006720	isoprenoid metabolic process	BP	-1.625270581	-4.456706967	-5.505477632	0.791119027	51.92467717	3.10786E-11	4.94199E-10	juvenile hormone acid methyltransferase
RS013788	GO:0006720	isoprenoid metabolic process	BP	4.523831791	5.522316775	6.082412849	3.873281439	46.08243774	5.44714E-10	7.87703E-09	PREDICTED: probable cytochrome P450 305a1
RS000919	GO:0006720	isoprenoid metabolic process	BP	0.299501456	1.741696639	-0.081450247	7.750885128	44.04920028	1.4733E-09	2.05246E-08	PREDICTED: 3-hydroxy-3-methylglutaryl-coenzyme A reductase
RS011853	GO:0006720	isoprenoid metabolic process	BP	-0.198391061	-2.402376009	-1.315737207	5.883199768	42.14749002	3.73315E-09	5.06955E-08	PREDICTED: aldehyde dehydrogenase, dimeric NADP-preferring
RS001884	GO:0006720	isoprenoid metabolic process	BP	0.795236464	0.438276657	1.724458417	6.06156771	38.99900607	1.73651E-08	2.18169E-07	PREDICTED: protein croquemort-like
RS007468	GO:0006720	isoprenoid metabolic process	BP	1.554961358	-0.015902902	0.482470573	9.656522243	22.44534397	5.26932E-05	0.0004279	PREDICTED: retinal dehydrogenase 1
RS012711	GO:0006720	isoprenoid metabolic process	BP	-0.261444278	-1.426954231	-0.747677472	6.901316322	20.57634898	0.000128906	0.000979142	PREDICTED: beta,beta-carotene 9',10'-oxygenase
RS000985	GO:0006720	isoprenoid metabolic process	BP	-0.29448114	-1.752268901	0.208476973	8.194696299	18.37775851	0.000367584	0.002589711	PREDICTED: probable cytochrome P450 305a1
RS007482	GO:0006720	isoprenoid metabolic process	BP	-0.326011516	1.454538363	0.106694628	1.929722985	17.82254603	0.000478513	0.003299317	PREDICTED: geranylgeranyl pyrophosphate synthase
RS005033	GO:0006720	isoprenoid metabolic process	BP	0.142636854	1.886458506	0.8717188	3.994789684	17.64333971	0.000520987	0.003567437	PREDICTED: hydroxymethylglutaryl-CoA synthase 1
RS001886	GO:0006720	isoprenoid metabolic process	BP	-2.408260642	-0.589495078	-1.004178279	3.150888145	16.77582032	0.00078587	0.005215469	PREDICTED: protein croquemort-like

RS015505	GO:0006720	isoprenoid metabolic process	BP	-0.333963264	-1.254068775	-0.333900304	7.203178713	16.32816273	0.000971162	0.006353837	PREDICTED: probable methylmalonate-semialdehyde dehydrogenase [acylating], mitochondrial
RS001763	GO:0006720	isoprenoid metabolic process	BP	-0.765106078	-2.406391493	-0.098466625	7.382023827	16.07185152	0.001096161	0.007103345	Dehydrogenase/reductase SDR family member 11
RS007469	GO:0006720	isoprenoid metabolic process	BP	0.109921824	-1.512321655	-1.260766781	10.37063405	14.46509492	0.002335814	0.014022266	PREDICTED: retinal dehydrogenase 1
RS007516	GO:0006720	isoprenoid metabolic process	BP	-1.028657455	1.245130415	1.086483676	13.85469833	14.31398947	0.002507468	0.014945387	actin
RS001885	GO:0006720	isoprenoid metabolic process	BP	-0.271637027	-1.360873271	0.03499008	3.248505684	13.75810288	0.003253609	0.018887587	PREDICTED: protein croquemort-like
RS000328	GO:0006720	isoprenoid metabolic process	BP	0.978084896	0.038022828	0.524967434	5.828289629	13.51663083	0.003642698	0.02092341	PREDICTED: farnesyl pyrophosphate synthase
RS007483	GO:0006720	isoprenoid metabolic process	BP	-2.357334054	-1.334091745	-2.336906267	0.504909953	12.14522038	0.006901897	0.036993251	PREDICTED: geranylgeranyl pyrophosphate synthase
RS001764	GO:0006720	isoprenoid metabolic process	BP	-0.34089142	-1.274576739	-0.08040122	6.629558203	7.318764875	0.062402654	0.235201607	Dehydrogenase/reductase SDR family member 11
RS100010	GO:0006720	isoprenoid metabolic process	BP	-1.155740681	-1.574587114	0.736520257	0.89623493	7.292285629	0.063142718	0.237299144	PREDICTED: geranylgeranyl pyrophosphate synthase
RS011854	GO:0006720	isoprenoid metabolic process	BP	-0.744270551	0.041529434	-0.702768939	7.795747502	7.164883594	0.066823987	0.248104681	PREDICTED: aldehyde dehydrogenase, dimeric NADP-preferring
RS009125	GO:0006720	isoprenoid metabolic process	BP	0.956044811	-0.148073708	0.932479134	2.076001482	7.124149978	0.068044219	0.251589467	PREDICTED: isoprenoid synthase domain-containing protein
RS000986	GO:0006720	isoprenoid metabolic process	BP	-0.740366011	-0.043299267	-0.829938683	5.994364596	5.511541444	0.137949939	0.425776905	PREDICTED: probable cytochrome P450 305a1
RS005846	GO:0006720	isoprenoid metabolic process	BP	-0.204371705	0.235880963	-0.413168784	4.909460384	5.205903352	0.157326043	0.465425878	PREDICTED: diphosphomevalonate decarboxylase
RS000987	GO:0006720	isoprenoid metabolic process	BP	-0.558625859	-0.603572019	-0.038627506	4.115748725	4.855302088	0.182704484	0.517869671	PREDICTED: probable cytochrome P450 305a1
RS000327	GO:0006720	isoprenoid metabolic process	BP	-0.280057357	-1.058934228	-0.674261538	7.058523817	4.443350347	0.21739931	0.577846178	PREDICTED: farnesyl pyrophosphate synthase
RS013397	GO:0006720	isoprenoid metabolic process	BP	0.276762068	-0.133208457	-0.437714221	7.582877367	4.164631585	0.244226876	0.623342031	PREDICTED: delta(3,5)-Delta(2,4)-dienoyl-CoA isomerase, mitochondrial
RS013349	GO:0006720	isoprenoid metabolic process	BP	-0.0079831	0.412785967	-0.237167699	5.649856826	4.070125368	0.253990743	0.63907986	PREDICTED: hydroxymethylglutaryl-CoA synthase 1
RS014514	GO:0006720	isoprenoid metabolic process	BP	0.454293395	0.189819706	-0.159566019	8.374000108	3.535332448	0.316208451	0.719511677	PREDICTED: trifunctional enzyme subunit alpha, mitochondrial
RS012473	GO:0006720	isoprenoid metabolic process	BP	0.306707128	0.418508405	-0.027591115	5.653573217	3.297735221	0.347957984	0.756542351	PREDICTED: phosphomevalonate kinase
RS007484	GO:0006720	isoprenoid metabolic process	BP	0.532156365	0.550474553	0.853413257	2.961241555	2.608173647	0.456058403	0.856582856	PREDICTED: geranylgeranyl pyrophosphate synthase
RS002932	GO:0006720	isoprenoid metabolic process	BP	0.285330244	-0.183360297	-0.196925118	7.264005384	2.38943308	0.495604683	0.88489721	PREDICTED: aldehyde dehydrogenase, mitochondrial
RS007481	GO:0006720	isoprenoid metabolic process	BP	0.46015371	0.979196535	0.821302629	1.24577587	2.355887167	0.501899031	0.886490057	PREDICTED: geranylgeranyl pyrophosphate synthase
RS010988	GO:0006720	isoprenoid metabolic process	BP	0.262058021	0.123117736	0.598878406	2.998961013	2.161958105	0.539480254	0.908038249	PREDICTED: dolichol phosphate-mannose biosynthesis regulatory protein
RS001711	GO:0006720	isoprenoid metabolic process	BP	0.090646339	0.318957455	-0.126739578	6.747827317	2.082452942	0.555470801	0.917038659	PREDICTED: probable dolichol-phosphate mannosyltransferase
RS100011	GO:0006720	isoprenoid metabolic process	BP	0.08619348	0.35129476	0.434471771	3.251884573	1.976103672	0.577381262	0.928860516	PREDICTED: geranylgeranyl pyrophosphate synthase
RS010567	GO:0006720	isoprenoid metabolic process	BP	-0.198427706	-0.253712569	0.126213578	5.507438126	1.886780944	0.596234875	0.939900556	PREDICTED: 4-hydroxybenzoate polyprenyltransferase, mitochondrial
RS100016	GO:0006720	isoprenoid metabolic process	BP	-0.571893807	0.01109718	0.152846641	3.456354556	1.848587936	0.604419161	0.945556612	PREDICTED: geranylgeranyl pyrophosphate synthase
RS002269	GO:0006720	isoprenoid metabolic process	BP	-0.115899934	-0.045489339	-0.35700385	6.156428651	1.746693481	0.626603143	0.953618312	PREDICTED: succinate-semialdehyde dehydrogenase, mitochondrial
RS000240	GO:0006720	isoprenoid metabolic process	BP	-0.381260852	-0.548825844	-0.265693149	5.841900743	1.544702564	0.671992089	0.975910688	PREDICTED: phytanoyl-CoA dioxygenase, peroxisomal
RS100012	GO:0006720	isoprenoid metabolic process	BP	0.192349859	0.555220807	0.053486602	3.947852311	1.497218821	0.682912374	0.977986414	PREDICTED: geranylgeranyl pyrophosphate synthase
RS013787	GO:0006720	isoprenoid metabolic process	BP	-0.274759984	0.074207597	0.175603802	3.188960439	1.257692569	0.739203052	0.996823246	PREDICTED: probable cytochrome P450 305a1
RS001132	GO:0006720	isoprenoid metabolic process	BP	0.266178506	0.185497446	-0.017903823	7.720936942	0.954946663	0.812151659	0.998581907	PREDICTED: mevalonate kinase
RS001133	GO:0006720	isoprenoid metabolic process	BP	0.121771934	-0.042701559	-0.115178897	4.779819281	0.434009831	0.933130627	0.998581907	PREDICTED: bolA-like protein 2
RS009545	GO:0006720	isoprenoid metabolic process	BP	0.380226632	0.162644242	0.158066156	7.506343047	0.762011484	0.858530104	0.998581907	PREDICTED: adipocyte plasma membrane-associated protein-like
RS012944	GO:0006720	isoprenoid metabolic process	BP	0.005963536	-0.089944286	-0.142741873	4.492809558	0.255251373	0.968212374	0.998581907	PREDICTED: isopentenyl-diphosphate Delta-isomerase 1
RS009535	GO:0006720	isoprenoid metabolic process	BP	-0.056065203	0.092504311	-0.098751469	6.062674479	0.266403412	0.966218072	0.998581907	PREDICTED: delta-1-pyrroline-5-carboxylate dehydrogenase, mitochondrial
RS010208	GO:0072330	monocarboxylic acid biosynthetic process	BP	0.40953932	8.566234616	-0.813747211	5.573361368	83.90745465	4.45302E-18	1.8964E-15	PREDICTED: acyl-CoA Delta(11) desaturase-like
RS005642	GO:0072330	monocarboxylic acid biosynthetic process	BP	-0.279552371	4.272858857	-0.313652117	7.78962837	67.40068434	1.53711E-14	1.9617E-12	PREDICTED: elongation of very long chain fatty acids protein 4-like
RS001262	GO:0072330	monocarboxylic acid biosynthetic process	BP	-0.338954796	5.446293974	1.268047261	3.476596747	54.93909298	7.0747E-12	4.58463E-10	PREDICTED: elongation of very long chain fatty acids protein AAEL008004
RS011853	GO:0072330	monocarboxylic acid biosynthetic process	BP	-1.539711363	-1.980697617	-2.111889965	4.895722934	33.41511771	2.6325E-07	6.30742E-06	PREDICTED: aldehyde dehydrogenase, dimeric NADP-preferring
RS013244	GO:0072330	monocarboxylic acid biosynthetic process	BP	-1.487782389	-2.692851641	-2.371678281	3.111913039	30.24667209	1.22463E-06	2.44671E-05	PREDICTED: acyl-CoA Delta(11) desaturase-like
RS006876	GO:0072330	monocarboxylic acid biosynthetic process	BP	-1.593839448	-2.768637972	-2.765096032	7.097794961	30.03235568	1.3586E-06	2.65915E-05	PREDICTED: acyl-CoA Delta(11) desaturase-like
RS011319	GO:0072330	monocarboxylic acid biosynthetic process	BP	1.297734018	3.937584594	3.842441852	4.109693124	29.5576257	1.70974E-06	3.25971E-05	PREDICTED: elongation of very long chain fatty acids protein AAEL008004-like
RS015477	GO:0072330	monocarboxylic acid biosynthetic process	BP	-1.357077531	-0.867133807	-1.530859338	3.686839363	25.33393955	1.31469E-05	0.000204767	PREDICTED: pyruvate carboxylase, mitochondrial
RS013198	GO:0072330	monocarboxylic acid biosynthetic process	BP	0.896132421	-0.645896353	-1.292717358	3.883106874	24.75729978	1.73539E-05	0.000261186	PREDICTED: fatty acid hydroxylase domain-containing protein 2
RS001805	GO:0072330	monocarboxylic acid biosynthetic process	BP	-2.68529829	0.221266129	-1.028409465	3.986669859	22.59725553	4.8991E-05	0.000643625	PREDICTED: elongation of very long chain fatty acids protein AAEL008004-like
RS007468	GO:0072330	monocarboxylic acid biosynthetic process	BP	1.461435577	0.004846791	0.136545734	8.378746871	19.65605155	0.000200006	0.002180886	PREDICTED: retinal dehydrogenase 1
RS010978	GO:0072330	monocarboxylic acid biosynthetic process	BP	-1.198682581	-1.433513591	-1.599911096	7.615086683	19.36240662	0.000230055	0.002454743	PREDICTED: fatty acid synthase
RS009061	GO:0072330	monocarboxylic acid biosynthetic process	BP	-0.864241624	1.167084696	1.330503034	3.332211443	19.26066799	0.000241481	0.002560502	PREDICTED: elongation of very long chain fatty acids protein AAEL008004
RS006022	GO:0072330	monocarboxylic acid biosynthetic process	BP	-3.002622861	-0.09536608	-0.106929644	2.953528405	18.57509887	0.000334662	0.003380946	PREDICTED: beta-ureidopropionase
RS001261	GO:0072330	monocarboxylic acid biosynthetic process	BP	-0.885066846	-1.491689635	-2.739426172	5.845553124	17.79676997	0.000484404	0.004656752	PREDICTED: elongation of very long chain fatty acids protein 7
RS000681	GO:0072330	monocarboxylic acid biosynthetic process	BP	-2.799883829	0.74888975	-0.624675575	5.350984403	17.58504858	0.000535595	0.005051799	PREDICTED: elongation of very long chain fatty acids protein AAEL008004-like
RS011394	GO:0072330	monocarboxylic acid biosynthetic process	BP	-1.284509298	-2.31025524	-1.982826031	2.39977127	17.48739502	0.000560985	0.005271403	PREDICTED: hydroxyacyl-coenzyme A dehydrogenase, mitochondrial-like
RS011854	GO:0072330	monocarboxylic acid biosynthetic process	BP	-1.35236157	0.287378657	-0.660124585	6.873563429	16.89098532	0.00074418	0.006720183	PREDICTED: aldehyde dehydrogenase, dimeric NADP-preferring
RS002535	GO:0072330	monocarboxylic acid biosynthetic process	BP	-1.052343191	-0.872433361	-0.808793642	6.419183283	16.55913521	0.000870703	0.007663536	PREDICTED: acetyl-CoA carboxylase
RS007469	GO:0072330	monocarboxylic acid biosynthetic process	BP	-1.09984178	-1.575245179	-2.17486518	7.967693554	16.09288204	0.00108533	0.009253045	PREDICTED: retinal dehydrogenase 1
RS002190	GO:0072330	monocarboxylic acid biosynthetic process	BP	-1.824923402	-1.484254245	-0.168377733	2.674698116	16.00743246	0.001130012	0.009542921	PREDICTED: fatty acid 2-hydroxylase
RS013246	GO:0072330	monocarboxylic acid biosynthetic process	BP	-0.956146185	-2.513310351	-2.839896025	2.400675533	15.93467978	0.001169492	0.009803513	PREDICTED: acyl-CoA Delta(11) desaturase-like
RS010210	GO:0072330	monocarboxylic acid biosynthetic process	BP	-0.185024992	1.873783975	0.24166857	2.442036791	13.52894892	0.003621779	0.024958593	PREDICTED: acyl-CoA Delta(11) desaturase-like
RS013245	GO:0072330	monocarboxylic acid biosynthetic process	BP	-0.101657773	-2.121665275	-1.150332926	3.544940819	12.27982883	0.006483549	0.040130175	PREDICTED: acyl-CoA Delta(11) desaturase-like
RS010209	GO:0072330	monocarboxylic acid biosynthetic process	BP	-0.736956301	0.474470079	-0.471752419	5.545819004	11.54825203	0.0091023	0.052137615	PREDICTED: acyl-CoA Delta(11) desaturase
RS010737	GO:0072330	monocarboxylic acid biosynthetic process	BP	-0.247692759	1.085794566	0.915381516	4.638859366	11.13690421	0.011008155	0.059938615	PREDICTED: elongation of very long chain fatty acids protein AAEL008004
RS005544	GO:0072330	monocarboxylic acid biosynthetic process	BP	-0.082655147	0.512723371	-0.903724344	4.410585135	9.868590242	0.019716838	0.094882067	PREDICTED: beta-ureidopropionase
RS003648	GO:0072330	monocarboxylic acid biosynthetic process	BP	0.335424074	0.609770127	-0.614739035	5.876670465	9.171002913	0.027101623	0.121928058	PREDICTED: protein singed
RS011502	GO:0072330	monocarboxylic acid biosynthetic process	BP	1.05705852	0.446891983	0.29693492	7.192950505	9.057303762	0.028538624	0.127067489	PREDICTED: 3-hydroxyacyl-CoA dehydrogenase type-2
RS013812	GO:0072330	monocarboxylic acid biosynthetic process	BP	-0.86621186	-0.658458955	-0.817815679	4.388253413	8.923687398	0.0303229	0.132743617	PREDICTED: propionyl-CoA carboxylase beta chain, mitochondrial
RS013197	GO:0072330	monocarboxylic acid biosynthetic process	BP	-1.612351267	-0.62106831	-0.592382332	5.772342887	8.889493327	0.030796792	0.134058205	PREDICTED: fatty acid hydroxylase domain-containing protein 2
RS015505	GO:0072330	monocarboxylic acid biosynthetic process	BP	-0.978556642	-0.958832908	-0.261061582	6.744664605	8.855720098	0.031271963	0.135569652	PREDICTED: probable methylmalonate-semialdehyde dehydrogenase [acylating], mitochondrial
RS007097	GO:0072330	monocarboxylic acid biosynthetic process	BP	0.373595636	-0.35778522	-0.525824672	4.113699054	8.093922933	0.044109964	0.175863346	PREDICTED: methylcrotonoyl-CoA carboxylase subunit alpha, mitochondrial
RS013686	GO:0072330	monocarboxylic acid biosynthetic process	BP	0.863108513	-0.546438339	-0.068479185	4.491043804	7.883479624	0.048482223	0.188488393	PREDICTED: acyl-CoA Delta(11) desaturase-like
RS008466	GO:0072330	monocarboxylic acid biosynthetic process	BP	0.23094468	0.606433909	0.709058821	6.195490356	7.847507042	0.049270681	0.19066473	PREDICTED: protein deglycase DJ-1-like
RS002807	GO:0072330	monocarboxylic acid biosynthetic process	BP	0.569520956	-0.229710319	-0.41934753	4.001241935	7.644261208	0.053965354	0.204157497	PREDICTED: bifunctional coenzyme A synthase
RS013199	GO:0072330	monocarboxylic acid biosynthetic process	BP	-0.520123082	-0.628040598	-1.25176871	4.061270269	7.377662596	0.060786562	0.221714064	PREDICTED: fatty acid hydroxylase domain-containing protein 2
RS006877	GO:0072330	monocarboxylic acid biosynthetic process	BP	-1.463048184	-0.603743205	-1.924867752	0.661629944	7.200875865	0.065763439	0.234732686	PREDICTED: acyl-CoA Delta(11) desaturase
RS009359	GO:0072330	monocarboxylic acid biosynthetic process	BP	0.759728316	0.141832502	0.609048514	4.874503124	6.794657901	0.078738842	0.26523657	PREDICTED: probable malonyl-CoA-acyl carrier protein transacylase, mitochondrial
RS008955	GO:0072330	monocarboxylic acid biosynthetic process	BP	0.49619909	0.538661905	0.874223714	5.531490277	6.409295268	0.093309138	0.299850305	PREDICTED: phosphopantothenate--cysteine ligase
RS002678	GO:0072330	monocarboxylic acid biosynthetic process	BP	-0.700472261	-1.032634555	-0.404958866	3.701627733	6.30501959	0.097677492	0.310429635	PREDICTED: fatty acid synthase-like
RS006548	GO:0072330	monocarboxylic acid biosynthetic process	BP	0.157530637	0.541952921	0.801596853	7.696201606	5.454525931	0.141383911	0.391363543	PREDICTED: acyl carrier protein, mitochondrial
RS013687	GO:0072330	monocarboxylic acid biosynthetic process	BP	-0.016961736	-1.37774066	-0.598056224	2.940563216	5.202822538	0.157533844	0.420399846	PREDICTED: acyl-CoA Delta(11) desaturase-like
RS007875	GO:0072330	monocarboxylic acid biosynthetic process	BP	0.649092018	0.224347975	0.426440348	5.992692439	4.900604249	0.179221762	0.456942118	PREDICTED: splicing factor 3B subunit 6-like protein
RS013655	GO:0072330	monocarboxylic acid biosynthetic process	BP	-1.080742374	-0.092997764	-0.767879495	9.110944973	4.889696223	0.18005462	0.45810787	PREDICTED: transforming growth factor-beta-induced protein ig-h3-like
RS002778	GO:0072330	monocarboxylic acid biosynthetic process	BP	0.579461063	0.538443294	0.620406302	5.314325197	4.831703938	0.184543629	0.465527867	PREDICTED: N-alpha-acetyltransferase 40
RS009535	GO:0072330	monocarboxylic acid biosynthetic process	BP	-0.747931195	-0.197281827	-0.499138908	6.700058131	4.619934419	0.201838661	0.490936627	PREDICTED: delta-1-pyrroline-5-carboxylate dehydrogenase, mitochondrial
RS010651	GO:0072330	monocarboxylic acid biosynthetic process	BP	0.602579698	-0.697714514	-0.422855969	1.029404074	4.457155227	0.216143929	0.513157199	PREDICTED: L-aminoadipate-semialdehyde dehydrogenase-phosphopantetheinyl transferase
RS012801	GO:0072330	monocarboxylic acid biosynthetic process	BP	-0.304924491	0.256722313	-0.759949671	2.652708114	4.441620744	0.21755707	0.515140203	PREDICTED: trimethyllysine dioxygenase, mitochondrial
RS001808	GO:0072330	monocarboxylic acid biosynthetic process	BP	-0.695225216	0.402436691	0.314794879	2.119921446	3.857603675	0.27725658	0.593470486	PREDICTED: elongation of very long chain fatty acids protein AAEL008004-like
RS005894	GO:0072330	monocarboxylic acid biosynthetic process	BP	0.335607631	-0.327079178	-0.25590607	7.906420492	3.655017863	0.301206329	0.620196675	PREDICTED: pantothenate kinase 3
RS000435	GO:0072330	monocarboxylic acid biosynthetic process	BP	0.488182536	0.488320685	0.50918498	5.04250979	3.473999324	0.324150011	0.645461581	PREDICTED: 39S ribosomal protein L13, mitochondrial
RS012946	GO:0072330	monocarboxylic acid biosynthetic process	BP	-0.206534933	-0.425434625	-0.506757887	4.664403836	3.380705817	0.336566928	0.657367518	PREDICTED: dephospho-CoA kinase domain-containing protein
RS007471	GO:0072330	monocarboxylic acid biosynthetic process	BP	-0.18452211	0.497674381	0.127043581	8.130803258	3.018784418	0.388738046	0.705243218	PREDICTED: lambda-crystallin homolog
RS003393	GO:0072330	monocarboxylic acid biosynthetic process	BP	0.408517998	0.061810543	-0.02613326	6.732440593	2.837271477	0.417401064	0.729111048	PREDICTED: leukotriene A-4 hydrolase
RS010105	GO:0072330	monocarboxylic acid biosynthetic process	BP	-0.234246941	-0.76390623	-0.644097976	5.900365636	2.720902747	0.436686771	0.748272564	PREDICTED: elongation of very long chain fatty acids protein 6
RS012825	GO:0072330	monocarboxylic acid biosynthetic process	BP	0.485099415	0.365377893	0.459504228	5.600595599	2.710720254	0.438408573	0.749477312	PREDICTED: lipoyl synthase, mitochondrial
RS003105	GO:0072330	monocarboxylic acid biosynthetic process	BP	0.183649196	0.315441312	0.466780551	5.007811733	2.322858108	0.508156049	0.801653344	PREDICTED: alkylglycerol monooxygenase-like
RS007595	GO:0072330	monocarboxylic acid biosynthetic process	BP	0.164727983	-0.295824155	0.427640289	5.169829636	2.181260808	0.535648833	0.821777339	PREDICTED: fatty acid synthase-like
RS007071	GO:0072330	monocarboxylic acid biosynthetic process	BP	0.16857672	0.265490742	-0.154555133	6.045163222	1.960750395	0.58059294	0.84820417	PREDICTED: dihydropyrimidine dehydrogenase [NADP(+)]
RS002269	GO:0072330	monocarboxylic acid biosynthetic process	BP	-0.38241426	-0.288426799	-0.399182444	6.660770145	1.912856965	0.590689051	0.853723189	PREDICTED: succinate-semialdehyde dehydrogenase, mitochondrial
RS001719	GO:0072330	monocarboxylic acid biosynthetic process	BP	0.23343797	0.310266487	0.517838343	7.916279315	1.789688939	0.617181305	0.872508939	PREDICTED: trifunctional enzyme subunit beta, mitochondrial
RS006279	GO:0072330	monocarboxylic acid biosynthetic process	BP	0.184880266	0.769627133	0.310533969	1.515285651	1.637744848	0.650862032	0.893952496	PREDICTED: fatty acid synthase-like
RS012653	GO:0072330	monocarboxylic acid biosynthetic process	BP	-0.084021737	-0.424032699	-0.259044725	4.02329399	1.490885128	0.68437563	0.911456289	PREDICTED: 3-oxoacyl-[acyl-carrier-protein] synthase, mitochondrial
RS006755	GO:0072330	monocarboxylic acid biosynthetic process	BP	-0.049717574	0.272485067	0.033381435	5.300785048	1.276407765	0.734742763	0.933131186	PREDICTED: Parkinson disease 7 domain-containing protein 1
RS007596	GO:0072330	monocarboxylic acid biosynthetic process	BP	-0.11747467	-0.624349711	-0.164407836	3.507338677	1.259288718	0.73882231	0.934889785	PREDICTED: fatty acid synthase-like
RS003278	GO:0072330	monocarboxylic acid biosynthetic process	BP	-0.346470018	-0.090411042	-0.13097421	4.764766138	1.241825949	0.742991179	0.93645498	PREDICTED: malonyl-CoA decarboxylase, mitochondrial-like
RS007594	GO:0072330	monocarboxylic acid biosynthetic process	BP	0.16106181	-0.093769718	0.38480713	7.815007876	1.191800479	0.75497156	0.940842433	PREDICTED: fatty acid synthase
RS003723	GO:0072330	monocarboxylic acid biosynthetic process	BP	0.039806794	-0.334350128	-0.507270784	2.419411604	1.179971307	0.757811918	0.941300394	PREDICTED: growth arrest-specific protein 8
RS009283	GO:0072330	monocarboxylic acid biosynthetic process	BP	0.074524983	-0.230554773	-0.188357118	4.050002278	1.158422364	0.762992624	0.943176193	PREDICTED: dephospho-CoA kinase domain-containing protein
RS013389	GO:0072330	monocarboxylic acid biosynthetic process	BP	0.096342723	0.102312458	0.439543216	5.998769053	1.155945592	0.763588586	0.943695893	PREDICTED: propionyl-CoA carboxylase alpha chain, mitochondrial
RS011651	GO:0072330	monocarboxylic acid biosynthetic process	BP	-0.068864474	-0.294321027	0.013768587	5.100802697	1.01332519	0.798027699	0.956638671	PREDICTED: fatty acid synthase-like
RS009901	GO:0072330	monocarboxylic acid biosynthetic process	BP	-0.036136626	-0.276401588	-0.171475769	4.897047645	1.003597488	0.800381471	0.957229551	PREDICTED: dephospho-CoA kinase domain-containing protein
RS011393	GO:0072330	monocarboxylic acid biosynthetic process	BP	-0.278112416	-0.24784575	-0.252527295	6.641784714	0.954350873	0.812295746	0.960451131	PREDICTED: hydroxyacyl-coenzyme A dehydrogenase, mitochondrial-like
RS014514	GO:0072330	monocarboxylic acid biosynthetic process	BP	0.362935044	0.26440136	0.179406902	8.212784804	0.917621347	0.821173424	0.963277045	PREDICTED: trifunctional enzyme subunit alpha, mitochondrial

RS002642	GO:0072330	monocarboxylic acid biosynthetic process	BP	0.059983825	0.054443803	-0.216162172	4.650958618	0.660213422	0.882519799	0.979034651	PREDICTED: acetyl-CoA acetyltransferase, cytosolic
RS013452	GO:0072330	monocarboxylic acid biosynthetic process	BP	0.304905755	0.196439232	0.279889149	3.383958731	0.649457914	0.885022501	0.979730727	PREDICTED: putative lipoyltransferase 2, mitochondrial
RS014553	GO:0072330	monocarboxylic acid biosynthetic process	BP	-0.210915049	-0.392664805	0.03762662	0.423909115	0.42546143	0.934933963	0.989525182	PREDICTED: fatty acid synthase-like
RS013397	GO:0072330	monocarboxylic acid biosynthetic process	BP	-0.177291621	-0.04387875	-0.006145775	6.681702268	0.386582905	0.942999992	0.991192522	PREDICTED: delta(3,5)-Delta(2,4)-dienoyl-CoA isomerase, mitochondrial
RS001514	GO:0072330	monocarboxylic acid biosynthetic process	BP	0.130694093	0.065208981	0.147240676	6.16999534	0.273507904	0.96493141	0.996194717	PREDICTED: pantothenate kinase 4
RS002932	GO:0072330	monocarboxylic acid biosynthetic process	BP	0.016901916	0.134159103	0.001207983	7.021670537	0.211268982	0.975749919	0.99704905	PREDICTED: aldehyde dehydrogenase, mitochondrial
RS006641	GO:0042430	indole-containing compound metabolic process	BP	0	8.850887518	4.982676945	2.190892633	150.9532201	1.6411E-32	8.16005E-31	PREDICTED: aromatic-L-amino-acid decarboxylase-like
RS014580	GO:0042430	indole-containing compound metabolic process	BP	-2.238409419	-4.554196395	-0.83576737	3.778928597	98.73572107	2.90617E-21	8.73744E-20	PREDICTED: kynurenine formamidase-like
RS006640	GO:0042430	indole-containing compound metabolic process	BP	0	8.362754097	5.318907063	1.840319552	94.18289464	2.76637E-20	7.86014E-19	PREDICTED: aromatic-L-amino-acid decarboxylase-like
RS001654	GO:0042430	indole-containing compound metabolic process	BP	1.733073604	-4.46574722	-0.501137925	5.066069368	73.06021491	9.4359E-16	2.10655E-14	PREDICTED: catalase
RS010135	GO:0042430	indole-containing compound metabolic process	BP	1.840019098	-2.32625182	0.732787728	7.759250511	67.76710296	1.28317E-14	2.58392E-13	PREDICTED: protein henna
RS007624	GO:0042430	indole-containing compound metabolic process	BP	-1.038705777	-2.417981438	1.4929059	2.541304599	63.78671286	9.11699E-14	1.74098E-12	PREDICTED: tyrosine aminotransferase
RS011394	GO:0042430	indole-containing compound metabolic process	BP	-0.808232837	-2.973798175	-1.001114062	5.701841087	63.34834224	1.13133E-13	2.15086E-12	PREDICTED: hydroxyacyl-coenzyme A dehydrogenase, mitochondrial-like
RS100005	GO:0042430	indole-containing compound metabolic process	BP	-2.512831781	-3.07482716	-0.793132985	4.249756653	57.0339543	2.52735E-12	4.2935E-11	beta-glucosidase
RS011853	GO:0042430	indole-containing compound metabolic process	BP	-0.198391061	-2.402376009	-1.315737207	5.883199768	42.14749002	3.73315E-09	5.06955E-08	PREDICTED: aldehyde dehydrogenase, dimeric NADP-preferring
RS005123	GO:0042430	indole-containing compound metabolic process	BP	2.214245853	4.384368384	1.931581004	5.434815601	37.74744679	3.19676E-08	3.88783E-07	PREDICTED: kynurenine formamidase-like
RS003648	GO:0042430	indole-containing compound metabolic process	BP	1.268807283	1.776217158	0.41179627	5.528161964	23.03387344	3.97318E-05	0.000329264	PREDICTED: protein singed
RS007468	GO:0042430	indole-containing compound metabolic process	BP	1.554961358	-0.015902902	0.482470573	9.656522243	22.44534397	5.26932E-05	0.0004279	PREDICTED: retinal dehydrogenase 1
RS006642	GO:0042430	indole-containing compound metabolic process	BP	-0.637747527	1.174042561	-0.357044754	3.154543638	21.5719212	8.00738E-05	0.000631986	PREDICTED: aromatic-L-amino-acid decarboxylase
RS009998	GO:0042430	indole-containing compound metabolic process	BP	-0.09363052	0.506668162	1.222395436	5.448013038	20.12058269	0.00016025	0.001201688	PREDICTED: tryptophan 2,3-dioxygenase
RS006985	GO:0042430	indole-containing compound metabolic process	BP	-0.522671357	0.739758227	-0.391833734	5.350293084	18.78443454	0.00030294	0.002167353	PREDICTED: probable 2-oxoglutarate dehydrogenase E1 component DHKTD1 homolog, mitochondrial
RS010115	GO:0042430	indole-containing compound metabolic process	BP	-0.130017178	1.261118944	-0.650626648	1.761465135	17.35059423	0.000598577	0.004053643	PREDICTED: tryptophan 5-hydroxylase 1
RS015505	GO:0042430	indole-containing compound metabolic process	BP	-0.333963264	-1.254068775	-0.333900304	7.203178713	16.32816273	0.000971162	0.006353837	PREDICTED: probable methylmalonate-semialdehyde dehydrogenase [acylating], mitochondrial
RS008573	GO:0042430	indole-containing compound metabolic process	BP	-0.877847198	-1.285642597	-0.317627515	5.276427543	15.89720024	0.001190362	0.007667666	PREDICTED: probable aspartate aminotransferase, cytoplasmic
RS007469	GO:0042430	indole-containing compound metabolic process	BP	0.109921824	-1.512321655	-1.260766781	10.37063405	14.46509492	0.002335814	0.014022266	PREDICTED: retinal dehydrogenase 1
RS002083	GO:0042430	indole-containing compound metabolic process	BP	0.177250401	-0.761079147	-0.577437436	8.133614126	10.062607	0.018041381	0.084660246	PREDICTED: aspartate aminotransferase, mitochondrial
RS008575	GO:0042430	indole-containing compound metabolic process	BP	0.285937384	-0.731842967	-0.265423735	6.102447533	9.775911583	0.020570235	0.094235294	PREDICTED: probable aspartate aminotransferase, cytoplasmic
RS001672	GO:0042430	indole-containing compound metabolic process	BP	0.307357992	0.966128919	-0.03567067	4.711156529	8.400416794	0.038422093	0.159922993	PREDICTED: catalase
RS007471	GO:0042430	indole-containing compound metabolic process	BP	0.488223208	0.356479238	-0.43697813	8.831136905	7.56070493	0.056019028	0.215925459	PREDICTED: lambda-crystallin homolog
RS011854	GO:0042430	indole-containing compound metabolic process	BP	-0.744270551	0.041529434	-0.702768939	7.795747502	7.164883594	0.066823987	0.248104681	PREDICTED: aldehyde dehydrogenase, dimeric NADP-preferring
RS006400	GO:0042430	indole-containing compound metabolic process	BP	0.791751698	-0.199311153	0.789475208	3.864467767	6.803637183	0.07842698	0.281327411	PREDICTED: leucine-rich repeat-containing protein 47-like
RS013013	GO:0042430	indole-containing compound metabolic process	BP	0.208074223	1.342259058	-0.041659798	4.182064207	6.279402018	0.09878034	0.33526706	PREDICTED: peptidoglycan-recognition protein LB-like
RS000758	GO:0042430	indole-containing compound metabolic process	BP	0.33512919	-0.05711749	-0.463250935	7.63864689	5.751926409	0.124323472	0.394805661	PREDICTED: kynurenine--oxoglutarate transaminase 3
RS011781	GO:0042430	indole-containing compound metabolic process	BP	0.194868998	-0.290152413	-0.647996521	9.541772745	5.013408504	0.170817937	0.49469854	PREDICTED: enolase
RS010080	GO:0042430	indole-containing compound metabolic process	BP	-0.624995317	-0.751441981	-0.311360855	8.760336944	4.839771278	0.183912955	0.520034833	PREDICTED: 2-oxoglutarate dehydrogenase, mitochondrial
RS013397	GO:0042430	indole-containing compound metabolic process	BP	0.276762068	-0.133208457	-0.437714221	7.582877367	4.164631585	0.244226876	0.623342031	PREDICTED: delta(3,5)-Delta(2,4)-dienoyl-CoA isomerase, mitochondrial
RS014514	GO:0042430	indole-containing compound metabolic process	BP	0.454293395	0.189819706	-0.159566019	8.374000108	3.535332448	0.316208451	0.719511677	PREDICTED: trifunctional enzyme subunit alpha, mitochondrial
RS015233	GO:0042430	indole-containing compound metabolic process	BP	0.540850916	0.558601533	0.185240719	3.554345825	3.3904938	0.335244879	0.740735907	PREDICTED: kynurenine formamidase
RS013491	GO:0042430	indole-containing compound metabolic process	BP	-0.181773747	-0.876367231	-0.869756564	4.885158359	3.065746684	0.381598996	0.790214932	PREDICTED: amine oxidase [flavin-containing] B
RS011393	GO:0042430	indole-containing compound metabolic process	BP	0.02917404	-0.508183169	0.050743806	7.352702277	3.024628885	0.387843452	0.796886111	PREDICTED: hydroxyacyl-coenzyme A dehydrogenase, mitochondrial-like
RS013885	GO:0042430	indole-containing compound metabolic process	BP	-1.296946996	-0.308203642	-1.037010877	0.265185356	2.784418498	0.426071337	0.832842713	PREDICTED: tyrosine decarboxylase
RS002090	GO:0042430	indole-containing compound metabolic process	BP	-0.035193593	0.360328133	-0.131582113	4.860304023	2.500878624	0.475132317	0.871772722	PREDICTED: mitochondrial ribosome-associated GTPase 2
RS002642	GO:0042430	indole-containing compound metabolic process	BP	0.327968475	-0.152878617	-0.267497897	5.475828266	2.41164637	0.491470337	0.881592142	PREDICTED: acetyl-CoA acetyltransferase, cytosolic
RS002932	GO:0042430	indole-containing compound metabolic process	BP	0.285330244	-0.183360297	-0.196925118	7.264005384	2.38943308	0.495604683	0.88489721	PREDICTED: aldehyde dehydrogenase, mitochondrial
RS011797	GO:0042430	indole-containing compound metabolic process	BP	0.384815831	0.110590536	0.031157322	6.971559534	2.046333709	0.562845874	0.920617663	PREDICTED: tyrosine--tRNA ligase, cytoplasmic
RS008554	GO:0042430	indole-containing compound metabolic process	BP	0.26061457	-0.150564831	0.073116667	6.805535643	2.011998083	0.569920196	0.923921867	PREDICTED: probable phenylalanine--tRNA ligase beta subunit
RS014702	GO:0042430	indole-containing compound metabolic process	BP	0.053889903	0.000945402	-0.337929036	6.872299671	1.971861167	0.578267518	0.929137767	PREDICTED: glutaryl-CoA dehydrogenase, mitochondrial
RS001412	GO:0042430	indole-containing compound metabolic process	BP	-0.432560841	0.07421703	-0.330687573	10.62227785	1.773660119	0.620683494	0.951636173	catalase
RS002269	GO:0042430	indole-containing compound metabolic process	BP	-0.115899934	-0.045489339	-0.35700385	6.156428651	1.746693481	0.626603143	0.953618312	PREDICTED: succinate-semialdehyde dehydrogenase, mitochondrial
RS012407	GO:0042430	indole-containing compound metabolic process	BP	-0.056883468	0.282034432	0.214431303	6.135655265	1.676615982	0.64214505	0.961518328	PREDICTED: probable phenylalanine--tRNA ligase, mitochondrial
RS013884	GO:0042430	indole-containing compound metabolic process	BP	-0.632046343	-0.62122136	-0.1334654	1.884245628	1.381585071	0.709856913	0.989330549	PREDICTED: aromatic-L-amino-acid decarboxylase-like
RS001454	GO:0042430	indole-containing compound metabolic process	BP	0.068681475	-0.060934606	-0.164757009	5.079370103	0.528175367	0.912659209	0.998581907	PREDICTED: tryptophan--tRNA ligase, cytoplasmic
RS004573	GO:0042430	indole-containing compound metabolic process	BP	0.017752395	0.123015371	-0.190111273	5.974861078	0.679282781	0.878065689	0.998581907	PREDICTED: tryptophan--tRNA ligase, mitochondrial
RS009535	GO:0042430	indole-containing compound metabolic process	BP	-0.056065203	0.092504311	-0.098751469	6.062674479	0.266403412	0.966218072	0.998581907	PREDICTED: delta-1-pyrroline-5-carboxylate dehydrogenase, mitochondrial
RS011502	GO:0042430	indole-containing compound metabolic process	BP	0.178723895	0.074408536	-0.154428329	7.266745855	1.03873978	0.791879214	0.998581907	PREDICTED: 3-hydroxyacyl-CoA dehydrogenase type-2
RS009545	GO:0042430	indole-containing compound metabolic process	BP	0.380226632	0.162644242	0.158066156	7.506343047	0.762011484	0.858530104	0.998581907	PREDICTED: adipocyte plasma membrane-associated protein-like
RS003577	GO:0042430	indole-containing compound metabolic process	BP	0.046547345	-0.14594425	0.038124755	5.480941987	0.544458042	0.90902107	0.998581907	PREDICTED: probable phenylalanine--tRNA ligase alpha subunit
RS010099	GO:0042430	indole-containing compound metabolic process	BP	0.100814249	0.052396139	-0.166198377	8.020158747	0.756965839	0.859730062	0.998581907	PREDICTED: protein transport protein Sec24A
RS012839	GO:0042430	indole-containing compound metabolic process	BP	0.292242717	0.226216567	0.09849742	4.671305028	0.96001386	0.810926117	0.998581907	PREDICTED: tyrosine--tRNA ligase, mitochondrial
RS013008	GO:0042430	indole-containing compound metabolic process	BP	0.250876024	0.120317607	0.240583891	4.769299517	0.556791171	0.906248768	0.998581907	PREDICTED: probable phenylalanine--tRNA ligase alpha subunit
RS014580	GO:0006586	indolalkylamine metabolic process	BP	-2.238409419	-4.554196395	-0.83576737	3.778928597	98.73572107	2.90617E-21	8.73744E-20	PREDICTED: kynurenine formamidase-like
RS006640	GO:0006586	indolalkylamine metabolic process	BP	0	8.362754097	5.318907063	1.840319552	94.18289464	2.76637E-20	7.86014E-19	PREDICTED: aromatic-L-amino-acid decarboxylase-like
RS001654	GO:0006586	indolalkylamine metabolic process	BP	1.733073604	-4.46574722	-0.501137925	5.066069368	73.06021491	9.4359E-16	2.10655E-14	PREDICTED: catalase
RS010135	GO:0006586	indolalkylamine metabolic process	BP	1.840019098	-2.32625182	0.732787728	7.759250511	67.76710296	1.28317E-14	2.58392E-13	PREDICTED: protein henna
RS007624	GO:0006586	indolalkylamine metabolic process	BP	-1.038705777	-2.417981438	1.4929059	2.541304599	63.78671286	9.11699E-14	1.74098E-12	PREDICTED: tyrosine aminotransferase
RS011394	GO:0006586	indolalkylamine metabolic process	BP	-0.808232837	-2.973798175	-1.001114062	5.701841087	63.34834224	1.13133E-13	2.15086E-12	PREDICTED: hydroxyacyl-coenzyme A dehydrogenase, mitochondrial-like
RS100005	GO:0006586	indolalkylamine metabolic process	BP	-2.512831781	-3.07482716	-0.793132985	4.249756653	57.0339543	2.52735E-12	4.2935E-11	beta-glucosidase
RS011853	GO:0006586	indolalkylamine metabolic process	BP	-0.198391061	-2.402376009	-1.315737207	5.883199768	42.14749002	3.73315E-09	5.06955E-08	PREDICTED: aldehyde dehydrogenase, dimeric NADP-preferring
RS005123	GO:0006586	indolalkylamine metabolic process	BP	2.214245853	4.384368384	1.931581004	5.434815601	37.74744679	3.19676E-08	3.88783E-07	PREDICTED: kynurenine formamidase-like
RS003648	GO:0006586	indolalkylamine metabolic process	BP	1.268807283	1.776217158	0.41179627	5.528161964	23.03387344	3.97318E-05	0.000329264	PREDICTED: protein singed
RS007468	GO:0006586	indolalkylamine metabolic process	BP	1.554961358	-0.015902902	0.482470573	9.656522243	22.44534397	5.26932E-05	0.0004279	PREDICTED: retinal dehydrogenase 1
RS006642	GO:0006586	indolalkylamine metabolic process	BP	-0.637747527	1.174042561	-0.357044754	3.154543638	21.5719212	8.00738E-05	0.000631986	PREDICTED: aromatic-L-amino-acid decarboxylase
RS009998	GO:0006586	indolalkylamine metabolic process	BP	-0.09363052	0.506668162	1.222395436	5.448013038	20.12058269	0.00016025	0.001201688	PREDICTED: tryptophan 2,3-dioxygenase
RS006985	GO:0006586	indolalkylamine metabolic process	BP	-0.522671357	0.739758227	-0.391833734	5.350293084	18.78443454	0.00030294	0.002167353	PREDICTED: probable 2-oxoglutarate dehydrogenase E1 component DHKTD1 homolog, mitochondrial
RS010115	GO:0006586	indolalkylamine metabolic process	BP	-0.130017178	1.261118944	-0.650626648	1.761465135	17.35059423	0.000598577	0.004053643	PREDICTED: tryptophan 5-hydroxylase 1
RS015505	GO:0006586	indolalkylamine metabolic process	BP	-0.333963264	-1.254068775	-0.333900304	7.203178713	16.32816273	0.000971162	0.006353837	PREDICTED: probable methylmalonate-semialdehyde dehydrogenase [acylating], mitochondrial
RS008573	GO:0006586	indolalkylamine metabolic process	BP	-0.877847198	-1.285642597	-0.317627515	5.276427543	15.89720024	0.001190362	0.007667666	PREDICTED: probable aspartate aminotransferase, cytoplasmic
RS007469	GO:0006586	indolalkylamine metabolic process	BP	0.109921824	-1.512321655	-1.260766781	10.37063405	14.46509492	0.002335814	0.014022266	PREDICTED: retinal dehydrogenase 1
RS002083	GO:0006586	indolalkylamine metabolic process	BP	0.177250401	-0.761079147	-0.577437436	8.133614126	10.062607	0.018041381	0.084660246	PREDICTED: aspartate aminotransferase, mitochondrial
RS008575	GO:0006586	indolalkylamine metabolic process	BP	0.285937384	-0.731842967	-0.265423735	6.102447533	9.775911583	0.020570235	0.094235294	PREDICTED: probable aspartate aminotransferase, cytoplasmic
RS001672	GO:0006586	indolalkylamine metabolic process	BP	0.307357992	0.966128919	-0.03567067	4.711156529	8.400416794	0.038422093	0.159922993	PREDICTED: catalase
RS007471	GO:0006586	indolalkylamine metabolic process	BP	0.488223208	0.356479238	-0.43697813	8.831136905	7.56070493	0.056019028	0.215925459	PREDICTED: lambda-crystallin homolog
RS011854	GO:0006586	indolalkylamine metabolic process	BP	-0.744270551	0.041529434	-0.702768939	7.795747502	7.164883594	0.066823987	0.248104681	PREDICTED: aldehyde dehydrogenase, dimeric NADP-preferring
RS006400	GO:0006586	indolalkylamine metabolic process	BP	0.791751698	-0.199311153	0.789475208	3.864467767	6.803637183	0.07842698	0.281327411	PREDICTED: leucine-rich repeat-containing protein 47-like
RS013013	GO:0006586	indolalkylamine metabolic process	BP	0.208074223	1.342259058	-0.041659798	4.182064207	6.279402018	0.09878034	0.33526706	PREDICTED: peptidoglycan-recognition protein LB-like
RS000758	GO:0006586	indolalkylamine metabolic process	BP	0.33512919	-0.05711749	-0.463250935	7.63864689	5.751926409	0.124323472	0.394805661	PREDICTED: kynurenine--oxoglutarate transaminase 3
RS011781	GO:0006586	indolalkylamine metabolic process	BP	0.194868998	-0.290152413	-0.647996521	9.541772745	5.013408504	0.170817937	0.49469854	PREDICTED: enolase
RS010080	GO:0006586	indolalkylamine metabolic process	BP	-0.624995317	-0.751441981	-0.311360855	8.760336944	4.839771278	0.183912955	0.520034833	PREDICTED: 2-oxoglutarate dehydrogenase, mitochondrial
RS013397	GO:0006586	indolalkylamine metabolic process	BP	0.276762068	-0.133208457	-0.437714221	7.582877367	4.164631585	0.244226876	0.623342031	PREDICTED: delta(3,5)-Delta(2,4)-dienoyl-CoA isomerase, mitochondrial
RS014514	GO:0006586	indolalkylamine metabolic process	BP	0.454293395	0.189819706	-0.159566019	8.374000108	3.535332448	0.316208451	0.719511677	PREDICTED: trifunctional enzyme subunit alpha, mitochondrial
RS015233	GO:0006586	indolalkylamine metabolic process	BP	0.540850916	0.558601533	0.185240719	3.554345825	3.3904938	0.335244879	0.740735907	PREDICTED: kynurenine formamidase
RS011393	GO:0006586	indolalkylamine metabolic process	BP	0.02917404	-0.508183169	0.050743806	7.352702277	3.024628885	0.387843452	0.796886111	PREDICTED: hydroxyacyl-coenzyme A dehydrogenase, mitochondrial-like
RS013885	GO:0006586	indolalkylamine metabolic process	BP	-1.296946996	-0.308203642	-1.037010877	0.265185356	2.784418498	0.426071337	0.832842713	PREDICTED: tyrosine decarboxylase
RS002090	GO:0006586	indolalkylamine metabolic process	BP	-0.035193593	0.360328133	-0.131582113	4.860304023	2.500878624	0.475132317	0.871772722	PREDICTED: mitochondrial ribosome-associated GTPase 2
RS002642	GO:0006586	indolalkylamine metabolic process	BP	0.327968475	-0.152878617	-0.267497897	5.475828266	2.41164637	0.491470337	0.881592142	PREDICTED: acetyl-CoA acetyltransferase, cytosolic
RS002932	GO:0006586	indolalkylamine metabolic process	BP	0.285330244	-0.183360297	-0.196925118	7.264005384	2.38943308	0.495604683	0.88489721	PREDICTED: aldehyde dehydrogenase, mitochondrial
RS011797	GO:0006586	indolalkylamine metabolic process	BP	0.384815831	0.110590536	0.031157322	6.971559534	2.046333709	0.562845874	0.920617663	PREDICTED: tyrosine--tRNA ligase, cytoplasmic
RS008554	GO:0006586	indolalkylamine metabolic process	BP	0.26061457	-0.150564831	0.073116667	6.805535643	2.011998083	0.569920196	0.923921867	PREDICTED: probable phenylalanine--tRNA ligase beta subunit
RS014702	GO:0006586	indolalkylamine metabolic process	BP	0.053889903	0.000945402	-0.337929036	6.872299671	1.971861167	0.578267518	0.929137767	PREDICTED: glutaryl-CoA dehydrogenase, mitochondrial
RS001412	GO:0006586	indolalkylamine metabolic process	BP	-0.432560841	0.07421703	-0.330687573	10.62227785	1.773660119	0.620683494	0.951636173	catalase
RS002269	GO:0006586	indolalkylamine metabolic process	BP	-0.115899934	-0.045489339	-0.35700385	6.156428651	1.746693481	0.626603143	0.953618312	PREDICTED: succinate-semialdehyde dehydrogenase, mitochondrial
RS012407	GO:0006586	indolalkylamine metabolic process	BP	-0.056883468	0.282034432	0.214431303	6.135655265	1.676615982	0.64214505	0.961518328	PREDICTED: probable phenylalanine--tRNA ligase, mitochondrial
RS013884	GO:0006586	indolalkylamine metabolic process	BP	-0.632046343	-0.62122136	-0.1334654	1.884245628	1.381585071	0.709856913	0.989330549	PREDICTED: aromatic-L-amino-acid decarboxylase-like
RS001454	GO:0006586	indolalkylamine metabolic process	BP	0.068681475	-0.060934606	-0.164757009	5.079370103	0.528175367	0.912659209	0.998581907	PREDICTED: tryptophan--tRNA ligase, cytoplasmic
RS004573	GO:0006586	indolalkylamine metabolic process	BP	0.017752395	0.123015371	-0.190111273	5.974861078	0.679282781	0.878065689	0.998581907	PREDICTED: tryptophan--tRNA ligase, mitochondrial
RS009535	GO:0006586	indolalkylamine metabolic process	BP	-0.056065203	0.092504311	-0.098751469	6.062674479	0.266403412	0.966218072	0.998581907	PREDICTED: delta-1-pyrroline-5-carboxylate dehydrogenase, mitochondrial
RS011502	GO:0006586	indolalkylamine metabolic process	BP	0.178723895	0.074408536	-0.154428329	7.266745855	1.03873978	0.791879214	0.998581907	PREDICTED: 3-hydroxyacyl-CoA dehydrogenase type-2
RS003577	GO:0006586	indolalkylamine metabolic process	BP	0.046547345	-0.14594425	0.038124755	5.480941987	0.544458042	0.90902107	0.998581907	PREDICTED: probable phenylalanine--tRNA ligase alpha subunit
RS010099	GO:0006586	indolalkylamine metabolic process	BP	0.100814249	0.052396139	-0.166198377	8.020158747	0.756965839	0.859730062	0.998581907	PREDICTED: protein transport protein Sec24A
RS012839	GO:0006586	indolalkylamine metabolic process	BP	0.292242717	0.226216567	0.09849742	4.671305028	0.96001386	0.810926117	0.998581907	PREDICTED: tyrosine--tRNA ligase, mitochondrial
RS013008	GO:0006586	indolalkylamine metabolic process	BP	0.250876024	0.120317607	0.240583891	4.769299517	0.556791171	0.906248768	0.998581907	PREDICTED: probable phenylalanine--tRNA ligase alpha subunit

RS014580	GO:0006568	tryptophan metabolic process	BP	-2.238409419	-4.554196395	-0.83576737	3.778928597	98.73572107	2.90617E-21	8.73744E-20	PREDICTED: kynurenine formamidase-like
RS006640	GO:0006568	tryptophan metabolic process	BP	0	8.362754097	5.318907063	1.840319552	94.18289464	2.76637E-20	7.86014E-19	PREDICTED: aromatic-L-amino-acid decarboxylase-like
RS001654	GO:0006568	tryptophan metabolic process	BP	1.733073604	-4.46574722	-0.501137925	5.066069368	73.06021491	9.4359E-16	2.10655E-14	PREDICTED: catalase
RS010135	GO:0006568	tryptophan metabolic process	BP	1.840019098	-2.32625182	0.732787728	7.759250511	67.76710296	1.28317E-14	2.58392E-13	PREDICTED: protein henna
RS007624	GO:0006568	tryptophan metabolic process	BP	-1.038705777	-2.417981438	1.4929059	2.541304599	63.78671286	9.11699E-14	1.74098E-12	PREDICTED: tyrosine aminotransferase
RS011394	GO:0006568	tryptophan metabolic process	BP	-0.808232837	-2.973798175	-1.001114062	5.701841087	63.34834224	1.13133E-13	2.15086E-12	PREDICTED: hydroxyacyl-coenzyme A dehydrogenase, mitochondrial-like
RS100005	GO:0006568	tryptophan metabolic process	BP	-2.512831781	-3.07482716	-0.793132985	4.249756653	57.0339543	2.52735E-12	4.2935E-11	beta-glucosidase
RS011853	GO:0006568	tryptophan metabolic process	BP	-0.198391061	-2.402376009	-1.315737207	5.883199768	42.14749002	3.73315E-09	5.06955E-08	PREDICTED: aldehyde dehydrogenase, dimeric NADP-preferring
RS005123	GO:0006568	tryptophan metabolic process	BP	2.214245853	4.384368384	1.931581004	5.434815601	37.74744679	3.19676E-08	3.88783E-07	PREDICTED: kynurenine formamidase-like
RS003648	GO:0006568	tryptophan metabolic process	BP	1.268807283	1.776217158	0.41179627	5.528161964	23.03387344	3.97318E-05	0.000329264	PREDICTED: protein singed
RS007468	GO:0006568	tryptophan metabolic process	BP	1.554961358	-0.015902902	0.482470573	9.656522243	22.44534397	5.26932E-05	0.0004279	PREDICTED: retinal dehydrogenase 1
RS006642	GO:0006568	tryptophan metabolic process	BP	-0.637747527	1.174042561	-0.357044754	3.154543638	21.5719212	8.00738E-05	0.000631986	PREDICTED: aromatic-L-amino-acid decarboxylase
RS009998	GO:0006568	tryptophan metabolic process	BP	-0.09363052	0.506668162	1.222395436	5.448013038	20.12058269	0.00016025	0.001201688	PREDICTED: tryptophan 2,3-dioxygenase
RS006985	GO:0006568	tryptophan metabolic process	BP	-0.522671357	0.739758227	-0.391833734	5.350293084	18.78443454	0.00030294	0.002167353	PREDICTED: probable 2-oxoglutarate dehydrogenase E1 component DHKTD1 homolog, mitochondrial
RS010115	GO:0006568	tryptophan metabolic process	BP	-0.130017178	1.261118944	-0.650626648	1.761465135	17.35059423	0.000598577	0.004053643	PREDICTED: tryptophan 5-hydroxylase 1
RS015505	GO:0006568	tryptophan metabolic process	BP	-0.333963264	-1.254068775	-0.333900304	7.203178713	16.32816273	0.000971162	0.006353837	PREDICTED: probable methylmalonate-semialdehyde dehydrogenase [acylating], mitochondrial
RS008573	GO:0006568	tryptophan metabolic process	BP	-0.877847198	-1.285642597	-0.317627515	5.276427543	15.89720024	0.001190362	0.007667666	PREDICTED: probable aspartate aminotransferase, cytoplasmic
RS007469	GO:0006568	tryptophan metabolic process	BP	0.109921824	-1.512321655	-1.260766781	10.37063405	14.46509492	0.002335814	0.014022266	PREDICTED: retinal dehydrogenase 1
RS002083	GO:0006568	tryptophan metabolic process	BP	0.177250401	-0.761079147	-0.577437436	8.133614126	10.062607	0.018041381	0.084660246	PREDICTED: aspartate aminotransferase, mitochondrial
RS008575	GO:0006568	tryptophan metabolic process	BP	0.285937384	-0.731842967	-0.265423735	6.102447533	9.775911583	0.020570235	0.094235294	PREDICTED: probable aspartate aminotransferase, cytoplasmic
RS001672	GO:0006568	tryptophan metabolic process	BP	0.307357992	0.966128919	-0.03567067	4.711156529	8.400416794	0.038422093	0.159922993	PREDICTED: catalase
RS007471	GO:0006568	tryptophan metabolic process	BP	0.488223208	0.356479238	-0.43697813	8.831136905	7.56070493	0.056019028	0.215925459	PREDICTED: lambda-crystallin homolog
RS011854	GO:0006568	tryptophan metabolic process	BP	-0.744270551	0.041529434	-0.702768939	7.795747502	7.164883594	0.066823987	0.248104681	PREDICTED: aldehyde dehydrogenase, dimeric NADP-preferring
RS006400	GO:0006568	tryptophan metabolic process	BP	0.791751698	-0.199311153	0.789475208	3.864467767	6.803637183	0.07842698	0.281327411	PREDICTED: leucine-rich repeat-containing protein 47-like
RS013013	GO:0006568	tryptophan metabolic process	BP	0.208074223	1.342259058	-0.041659798	4.182064207	6.279402018	0.09878034	0.33526706	PREDICTED: peptidoglycan-recognition protein LB-like
RS000758	GO:0006568	tryptophan metabolic process	BP	0.33512919	-0.05711749	-0.463250935	7.63864689	5.751926409	0.124323472	0.394805661	PREDICTED: kynurenine--oxoglutarate transaminase 3
RS011781	GO:0006568	tryptophan metabolic process	BP	0.194868998	-0.290152413	-0.647996521	9.541772745	5.013408504	0.170817937	0.49469854	PREDICTED: enolase
RS010080	GO:0006568	tryptophan metabolic process	BP	-0.624995317	-0.751441981	-0.311360855	8.760336944	4.839771278	0.183912955	0.520034833	PREDICTED: 2-oxoglutarate dehydrogenase, mitochondrial
RS013397	GO:0006568	tryptophan metabolic process	BP	0.276762068	-0.133208457	-0.437714221	7.582877367	4.164631585	0.244226876	0.623342031	PREDICTED: delta(3,5)-Delta(2,4)-dienoyl-CoA isomerase, mitochondrial
RS014514	GO:0006568	tryptophan metabolic process	BP	0.454293395	0.189819706	-0.159566019	8.374000108	3.535332448	0.316208451	0.719511677	PREDICTED: trifunctional enzyme subunit alpha, mitochondrial
RS015233	GO:0006568	tryptophan metabolic process	BP	0.540850916	0.558601533	0.185240719	3.554345825	3.3904938	0.335244879	0.740735907	PREDICTED: kynurenine formamidase
RS011393	GO:0006568	tryptophan metabolic process	BP	0.02917404	-0.508183169	0.050743806	7.352702277	3.024628885	0.387843452	0.796886111	PREDICTED: hydroxyacyl-coenzyme A dehydrogenase, mitochondrial-like
RS013885	GO:0006568	tryptophan metabolic process	BP	-1.296946996	-0.308203642	-1.037010877	0.265185356	2.784418498	0.426071337	0.832842713	PREDICTED: tyrosine decarboxylase
RS002090	GO:0006568	tryptophan metabolic process	BP	-0.035193593	0.360328133	-0.131582113	4.860304023	2.500878624	0.475132317	0.871772722	PREDICTED: mitochondrial ribosome-associated GTPase 2
RS002642	GO:0006568	tryptophan metabolic process	BP	0.327968475	-0.152878617	-0.267497897	5.475828266	2.41164637	0.491470337	0.881592142	PREDICTED: acetyl-CoA acetyltransferase, cytosolic
RS002932	GO:0006568	tryptophan metabolic process	BP	0.285330244	-0.183360297	-0.196925118	7.264005384	2.38943308	0.495604683	0.88489721	PREDICTED: aldehyde dehydrogenase, mitochondrial
RS011797	GO:0006568	tryptophan metabolic process	BP	0.384815831	0.110590536	0.031157322	6.971559534	2.046333709	0.562845874	0.920617663	PREDICTED: tyrosine--tRNA ligase, cytoplasmic
RS008554	GO:0006568	tryptophan metabolic process	BP	0.26061457	-0.150564831	0.073116667	6.805535643	2.011998083	0.569920196	0.923921867	PREDICTED: probable phenylalanine--tRNA ligase beta subunit
RS014702	GO:0006568	tryptophan metabolic process	BP	0.053889903	0.000945402	-0.337929036	6.872299671	1.971861167	0.578267518	0.929137767	PREDICTED: glutaryl-CoA dehydrogenase, mitochondrial
RS001412	GO:0006568	tryptophan metabolic process	BP	-0.432560841	0.07421703	-0.330687573	10.62227785	1.773660119	0.620683494	0.951636173	catalase
RS002269	GO:0006568	tryptophan metabolic process	BP	-0.115899934	-0.045489339	-0.35700385	6.156428651	1.746693481	0.626603143	0.953618312	PREDICTED: succinate-semialdehyde dehydrogenase, mitochondrial
RS012407	GO:0006568	tryptophan metabolic process	BP	-0.056883468	0.282034432	0.214431303	6.135655265	1.676615982	0.64214505	0.961518328	PREDICTED: probable phenylalanine--tRNA ligase, mitochondrial
RS013884	GO:0006568	tryptophan metabolic process	BP	-0.632046343	-0.62122136	-0.1334654	1.884245628	1.381585071	0.709856913	0.989330549	PREDICTED: aromatic-L-amino-acid decarboxylase-like
RS001454	GO:0006568	tryptophan metabolic process	BP	0.068681475	-0.060934606	-0.164757009	5.079370103	0.528175367	0.912659209	0.998581907	PREDICTED: tryptophan--tRNA ligase, cytoplasmic
RS004573	GO:0006568	tryptophan metabolic process	BP	0.017752395	0.123015371	-0.190111273	5.974861078	0.679282781	0.878065689	0.998581907	PREDICTED: tryptophan--tRNA ligase, mitochondrial
RS009535	GO:0006568	tryptophan metabolic process	BP	-0.056065203	0.092504311	-0.098751469	6.062674479	0.266403412	0.966218072	0.998581907	PREDICTED: delta-1-pyrroline-5-carboxylate dehydrogenase, mitochondrial
RS011502	GO:0006568	tryptophan metabolic process	BP	0.178723895	0.074408536	-0.154428329	7.266745855	1.03873978	0.791879214	0.998581907	PREDICTED: 3-hydroxyacyl-CoA dehydrogenase type-2
RS003577	GO:0006568	tryptophan metabolic process	BP	0.046547345	-0.14594425	0.038124755	5.480941987	0.544458042	0.90902107	0.998581907	PREDICTED: probable phenylalanine--tRNA ligase alpha subunit
RS010099	GO:0006568	tryptophan metabolic process	BP	0.100814249	0.052396139	-0.166198377	8.020158747	0.756965839	0.859730062	0.998581907	PREDICTED: protein transport protein Sec24A
RS012839	GO:0006568	tryptophan metabolic process	BP	0.292242717	0.226216567	0.09849742	4.671305028	0.96001386	0.810926117	0.998581907	PREDICTED: tyrosine--tRNA ligase, mitochondrial
RS013008	GO:0006568	tryptophan metabolic process	BP	0.250876024	0.120317607	0.240583891	4.769299517	0.556791171	0.906248768	0.998581907	PREDICTED: probable phenylalanine--tRNA ligase alpha subunit
RS009744	GO:0009308	amine metabolic process	BP	3.201394793	9.259149545	2.881573793	2.526912099	116.4189494	4.55598E-25	1.68285E-23	PREDICTED: sphingomyelin phosphodiesterase-like
RS014580	GO:0009308	amine metabolic process	BP	-2.238409419	-4.554196395	-0.83576737	3.778928597	98.73572107	2.90617E-21	8.73744E-20	PREDICTED: kynurenine formamidase-like
RS003967	GO:0009308	amine metabolic process	BP	-0.834456324	4.291752911	4.483141964	7.868795768	96.08447484	1.07954E-20	3.14331E-19	ebony
RS006640	GO:0009308	amine metabolic process	BP	0	8.362754097	5.318907063	1.840319552	94.18289464	2.76637E-20	7.86014E-19	PREDICTED: aromatic-L-amino-acid decarboxylase-like
RS001654	GO:0009308	amine metabolic process	BP	1.733073604	-4.46574722	-0.501137925	5.066069368	73.06021491	9.4359E-16	2.10655E-14	PREDICTED: catalase
RS009746	GO:0009308	amine metabolic process	BP	-2.291159813	-2.901001572	-0.149472189	5.118614926	71.34595412	2.19792E-15	4.71181E-14	PREDICTED: sphingomyelin phosphodiesterase-like
RS010135	GO:0009308	amine metabolic process	BP	1.840019098	-2.32625182	0.732787728	7.759250511	67.76710296	1.28317E-14	2.58392E-13	PREDICTED: protein henna
RS007624	GO:0009308	amine metabolic process	BP	-1.038705777	-2.417981438	1.4929059	2.541304599	63.78671286	9.11699E-14	1.74098E-12	PREDICTED: tyrosine aminotransferase
RS011394	GO:0009308	amine metabolic process	BP	-0.808232837	-2.973798175	-1.001114062	5.701841087	63.34834224	1.13133E-13	2.15086E-12	PREDICTED: hydroxyacyl-coenzyme A dehydrogenase, mitochondrial-like
RS100005	GO:0009308	amine metabolic process	BP	-2.512831781	-3.07482716	-0.793132985	4.249756653	57.0339543	2.52735E-12	4.2935E-11	beta-glucosidase
RS011853	GO:0009308	amine metabolic process	BP	-0.198391061	-2.402376009	-1.315737207	5.883199768	42.14749002	3.73315E-09	5.06955E-08	PREDICTED: aldehyde dehydrogenase, dimeric NADP-preferring
RS005123	GO:0009308	amine metabolic process	BP	2.214245853	4.384368384	1.931581004	5.434815601	37.74744679	3.19676E-08	3.88783E-07	PREDICTED: kynurenine formamidase-like
RS009745	GO:0009308	amine metabolic process	BP	1.717491152	-2.500586916	-0.975453266	8.905614638	26.48198539	7.55933E-06	6.97054E-05	PREDICTED: sphingomyelin phosphodiesterase-like
RS003648	GO:0009308	amine metabolic process	BP	1.268807283	1.776217158	0.41179627	5.528161964	23.03387344	3.97318E-05	0.000329264	PREDICTED: protein singed
RS007468	GO:0009308	amine metabolic process	BP	1.554961358	-0.015902902	0.482470573	9.656522243	22.44534397	5.26932E-05	0.0004279	PREDICTED: retinal dehydrogenase 1
RS006642	GO:0009308	amine metabolic process	BP	-0.637747527	1.174042561	-0.357044754	3.154543638	21.5719212	8.00738E-05	0.000631986	PREDICTED: aromatic-L-amino-acid decarboxylase
RS009998	GO:0009308	amine metabolic process	BP	-0.09363052	0.506668162	1.222395436	5.448013038	20.12058269	0.00016025	0.001201688	PREDICTED: tryptophan 2,3-dioxygenase
RS006985	GO:0009308	amine metabolic process	BP	-0.522671357	0.739758227	-0.391833734	5.350293084	18.78443454	0.00030294	0.002167353	PREDICTED: probable 2-oxoglutarate dehydrogenase E1 component DHKTD1 homolog, mitochondrial
RS010115	GO:0009308	amine metabolic process	BP	-0.130017178	1.261118944	-0.650626648	1.761465135	17.35059423	0.000598577	0.004053643	PREDICTED: tryptophan 5-hydroxylase 1
RS015505	GO:0009308	amine metabolic process	BP	-0.333963264	-1.254068775	-0.333900304	7.203178713	16.32816273	0.000971162	0.006353837	PREDICTED: probable methylmalonate-semialdehyde dehydrogenase [acylating], mitochondrial
RS008573	GO:0009308	amine metabolic process	BP	-0.877847198	-1.285642597	-0.317627515	5.276427543	15.89720024	0.001190362	0.007667666	PREDICTED: probable aspartate aminotransferase, cytoplasmic
RS007469	GO:0009308	amine metabolic process	BP	0.109921824	-1.512321655	-1.260766781	10.37063405	14.46509492	0.002335814	0.014022266	PREDICTED: retinal dehydrogenase 1
RS010097	GO:0009308	amine metabolic process	BP	0.17435877	-0.952852764	-0.044428493	4.263987481	10.44397577	0.015145798	0.072762866	PREDICTED: spermine synthase
RS002083	GO:0009308	amine metabolic process	BP	0.177250401	-0.761079147	-0.577437436	8.133614126	10.062607	0.018041381	0.084660246	PREDICTED: aspartate aminotransferase, mitochondrial
RS008575	GO:0009308	amine metabolic process	BP	0.285937384	-0.731842967	-0.265423735	6.102447533	9.775911583	0.020570235	0.094235294	PREDICTED: probable aspartate aminotransferase, cytoplasmic
RS001672	GO:0009308	amine metabolic process	BP	0.307357992	0.966128919	-0.03567067	4.711156529	8.400416794	0.038422093	0.159922993	PREDICTED: catalase
RS007471	GO:0009308	amine metabolic process	BP	0.488223208	0.356479238	-0.43697813	8.831136905	7.56070493	0.056019028	0.215925459	PREDICTED: lambda-crystallin homolog
RS005623	GO:0009308	amine metabolic process	BP	-0.207148985	0.861315813	0.148164528	5.401930947	7.439227213	0.059140699	0.225072403	PREDICTED: sphingomyelin phosphodiesterase
RS011854	GO:0009308	amine metabolic process	BP	-0.744270551	0.041529434	-0.702768939	7.795747502	7.164883594	0.066823987	0.248104681	PREDICTED: aldehyde dehydrogenase, dimeric NADP-preferring
RS006400	GO:0009308	amine metabolic process	BP	0.791751698	-0.199311153	0.789475208	3.864467767	6.803637183	0.07842698	0.281327411	PREDICTED: leucine-rich repeat-containing protein 47-like
RS001701	GO:0009308	amine metabolic process	BP	0.123419788	-1.195769507	-0.558171808	9.403198181	6.717198573	0.08147924	0.289544699	PREDICTED: ornithine decarboxylase 1-like
RS009874	GO:0009308	amine metabolic process	BP	0.672394618	0.650048825	0.385379854	5.350943263	6.344436273	0.096003635	0.327649153	PREDICTED: spermidine synthase
RS013013	GO:0009308	amine metabolic process	BP	0.208074223	1.342259058	-0.041659798	4.182064207	6.279402018	0.09878034	0.33526706	PREDICTED: peptidoglycan-recognition protein LB-like
RS000758	GO:0009308	amine metabolic process	BP	0.33512919	-0.05711749	-0.463250935	7.63864689	5.751926409	0.124323472	0.394805661	PREDICTED: kynurenine--oxoglutarate transaminase 3
RS011781	GO:0009308	amine metabolic process	BP	0.194868998	-0.290152413	-0.647996521	9.541772745	5.013408504	0.170817937	0.49469854	PREDICTED: enolase
RS010080	GO:0009308	amine metabolic process	BP	-0.624995317	-0.751441981	-0.311360855	8.760336944	4.839771278	0.183912955	0.520034833	PREDICTED: 2-oxoglutarate dehydrogenase, mitochondrial
RS000725	GO:0009308	amine metabolic process	BP	-0.160887996	-0.686070869	-0.437727278	8.133751905	4.569821729	0.206146263	0.558946915	PREDICTED: neuropathy target esterase sws
RS013397	GO:0009308	amine metabolic process	BP	0.276762068	-0.133208457	-0.437714221	7.582877367	4.164631585	0.244226876	0.623342031	PREDICTED: delta(3,5)-Delta(2,4)-dienoyl-CoA isomerase, mitochondrial
RS009609	GO:0009308	amine metabolic process	BP	0.210078244	-0.06842499	-0.397693417	4.048414359	3.700886628	0.295627068	0.69337205	PREDICTED: S-methyl-5'-thioadenosine phosphorylase
RS014514	GO:0009308	amine metabolic process	BP	0.454293395	0.189819706	-0.159566019	8.374000108	3.535332448	0.316208451	0.719511677	PREDICTED: trifunctional enzyme subunit alpha, mitochondrial
RS015233	GO:0009308	amine metabolic process	BP	0.540850916	0.558601533	0.185240719	3.554345825	3.3904938	0.335244879	0.740735907	PREDICTED: kynurenine formamidase
RS013491	GO:0009308	amine metabolic process	BP	-0.181773747	-0.876367231	-0.869756564	4.885158359	3.065746684	0.381598996	0.790214932	PREDICTED: amine oxidase [flavin-containing] B
RS011393	GO:0009308	amine metabolic process	BP	0.02917404	-0.508183169	0.050743806	7.352702277	3.024628885	0.387843452	0.796886111	PREDICTED: hydroxyacyl-coenzyme A dehydrogenase, mitochondrial-like
RS001973	GO:0009308	amine metabolic process	BP	0.055182938	0.849890155	0.027680752	1.268346164	2.932735676	0.402112703	0.809489057	PREDICTED: zinc finger protein 177-like
RS013885	GO:0009308	amine metabolic process	BP	-1.296946996	-0.308203642	-1.037010877	0.265185356	2.784418498	0.426071337	0.832842713	PREDICTED: tyrosine decarboxylase
RS002090	GO:0009308	amine metabolic process	BP	-0.035193593	0.360328133	-0.131582113	4.860304023	2.500878624	0.475132317	0.871772722	PREDICTED: mitochondrial ribosome-associated GTPase 2
RS002642	GO:0009308	amine metabolic process	BP	0.327968475	-0.152878617	-0.267497897	5.475828266	2.41164637	0.491470337	0.881592142	PREDICTED: acetyl-CoA acetyltransferase, cytosolic
RS002932	GO:0009308	amine metabolic process	BP	0.285330244	-0.183360297	-0.196925118	7.264005384	2.38943308	0.495604683	0.88489721	PREDICTED: aldehyde dehydrogenase, mitochondrial
RS003701	GO:0009308	amine metabolic process	BP	0.050229794	0.499103503	0.080945241	6.654471113	2.36698837	0.499809308	0.885993958	PREDICTED: probable deoxyhypusine synthase
RS011797	GO:0009308	amine metabolic process	BP	0.384815831	0.110590536	0.031157322	6.971559534	2.046333709	0.562845874	0.920617663	PREDICTED: tyrosine--tRNA ligase, cytoplasmic
RS008554	GO:0009308	amine metabolic process	BP	0.26061457	-0.150564831	0.073116667	6.805535643	2.011998083	0.569920196	0.923921867	PREDICTED: probable phenylalanine--tRNA ligase beta subunit
RS014702	GO:0009308	amine metabolic process	BP	0.053889903	0.000945402	-0.337929036	6.872299671	1.971861167	0.578267518	0.929137767	PREDICTED: glutaryl-CoA dehydrogenase, mitochondrial
RS001412	GO:0009308	amine metabolic process	BP	-0.432560841	0.07421703	-0.330687573	10.62227785	1.773660119	0.620683494	0.951636173	catalase
RS002269	GO:0009308	amine metabolic process	BP	-0.115899934	-0.045489339	-0.35700385	6.156428651	1.746693481	0.626603143	0.953618312	PREDICTED: succinate-semialdehyde dehydrogenase, mitochondrial
RS000318	GO:0009308	amine metabolic process	BP	-0.088415405	0.316315043	-0.022734089	7.458187859	1.720429115	0.632401528	0.95623074	PREDICTED: alpha-aminoadipic semialdehyde dehydrogenase
RS012407	GO:0009308	amine metabolic process	BP	-0.056883468	0.282034432	0.214431303	6.135655265	1.676615982	0.64214505	0.961518328	PREDICTED: probable phenylalanine--tRNA ligase, mitochondrial
RS007076	GO:0009308	amine metabolic process	BP	-0.307781838	-0.12916029	-0.445208222	6.08361277	1.518426518	0.678024076	0.976798812	PREDICTED: serine palmitoyltransferase 1
RS013884	GO:0009308	amine metabolic process	BP	-0.632046343	-0.62122136	-0.1334654	1.884245628	1.381585071	0.709856913	0.989330549	PREDICTED: aromatic-L-amino-acid decarboxylase-like
RS013476	GO:0009308	amine metabolic process	BP	0.198784997	0.042497253	-0.109203051	4.200710783	0.77622549	0.855144718	0.998581907	PREDICTED: diamine acetyltransferase 2-like
RS001454	GO:0009308	amine metabolic process	BP	0.068681475	-0.060934606	-0.164757009	5.079370103	0.528175367	0.912659209	0.998581907	PREDICTED: tryptophan--tRNA ligase, cytoplasmic

RS004573	GO:0009308	amine metabolic process	BP	0.017752395	0.123015371	-0.190111273	5.974861078	0.679282781	0.878065689	0.998581907	PREDICTED: tryptophan--tRNA ligase, mitochondrial
RS009535	GO:0009308	amine metabolic process	BP	-0.056065203	0.092504311	-0.098751469	6.062674479	0.266403412	0.966218072	0.998581907	PREDICTED: delta-1-pyrroline-5-carboxylate dehydrogenase, mitochondrial
RS011502	GO:0009308	amine metabolic process	BP	0.178723895	0.074408536	-0.154428329	7.266745855	1.03873978	0.791879214	0.998581907	PREDICTED: 3-hydroxyacyl-CoA dehydrogenase type-2
RS002780	GO:0009308	amine metabolic process	BP	0.045465929	-0.189873187	-0.126863793	4.210389331	0.543847218	0.909158008	0.998581907	PREDICTED: S-adenosylmethionine decarboxylase proenzyme
RS003577	GO:0009308	amine metabolic process	BP	0.046547345	-0.14594425	0.038124755	5.480941987	0.544458042	0.90902107	0.998581907	PREDICTED: probable phenylalanine--tRNA ligase alpha subunit
RS010099	GO:0009308	amine metabolic process	BP	0.100814249	0.052396139	-0.166198377	8.020158747	0.756965839	0.859730062	0.998581907	PREDICTED: protein transport protein Sec24A
RS012839	GO:0009308	amine metabolic process	BP	0.292242717	0.226216567	0.09849742	4.671305028	0.96001386	0.810926117	0.998581907	PREDICTED: tyrosine--tRNA ligase, mitochondrial
RS013008	GO:0009308	amine metabolic process	BP	0.250876024	0.120317607	0.240583891	4.769299517	0.556791171	0.906248768	0.998581907	PREDICTED: probable phenylalanine--tRNA ligase alpha subunit
RS009744	GO:0044106	cellular amine metabolic process	BP	3.201394793	9.259149545	2.881573793	2.526912099	116.4189494	4.55598E-25	1.68285E-23	PREDICTED: sphingomyelin phosphodiesterase-like
RS014580	GO:0044106	cellular amine metabolic process	BP	-2.238409419	-4.554196395	-0.83576737	3.778928597	98.73572107	2.90617E-21	8.73744E-20	PREDICTED: kynurenine formamidase-like
RS003967	GO:0044106	cellular amine metabolic process	BP	-0.834456324	4.291752911	4.483141964	7.868795768	96.08447484	1.07954E-20	3.14331E-19	ebony
RS006640	GO:0044106	cellular amine metabolic process	BP	0	8.362754097	5.318907063	1.840319552	94.18289464	2.76637E-20	7.86014E-19	PREDICTED: aromatic-L-amino-acid decarboxylase-like
RS001654	GO:0044106	cellular amine metabolic process	BP	1.733073604	-4.46574722	-0.501137925	5.066069368	73.06021491	9.4359E-16	2.10655E-14	PREDICTED: catalase
RS009746	GO:0044106	cellular amine metabolic process	BP	-2.291159813	-2.901001572	-0.149472189	5.118614926	71.34595412	2.19792E-15	4.71181E-14	PREDICTED: sphingomyelin phosphodiesterase-like
RS010135	GO:0044106	cellular amine metabolic process	BP	1.840019098	-2.32625182	0.732787728	7.759250511	67.76710296	1.28317E-14	2.58392E-13	PREDICTED: protein henna
RS007624	GO:0044106	cellular amine metabolic process	BP	-1.038705777	-2.417981438	1.4929059	2.541304599	63.78671286	9.11699E-14	1.74098E-12	PREDICTED: tyrosine aminotransferase
RS011394	GO:0044106	cellular amine metabolic process	BP	-0.808232837	-2.973798175	-1.001114062	5.701841087	63.34834224	1.13133E-13	2.15086E-12	PREDICTED: hydroxyacyl-coenzyme A dehydrogenase, mitochondrial-like
RS100005	GO:0044106	cellular amine metabolic process	BP	-2.512831781	-3.07482716	-0.793132985	4.249756653	57.0339543	2.52735E-12	4.2935E-11	beta-glucosidase
RS011853	GO:0044106	cellular amine metabolic process	BP	-0.198391061	-2.402376009	-1.315737207	5.883199768	42.14749002	3.73315E-09	5.06955E-08	PREDICTED: aldehyde dehydrogenase, dimeric NADP-preferring
RS005123	GO:0044106	cellular amine metabolic process	BP	2.214245853	4.384368384	1.931581004	5.434815601	37.74744679	3.19676E-08	3.88783E-07	PREDICTED: kynurenine formamidase-like
RS009745	GO:0044106	cellular amine metabolic process	BP	1.717491152	-2.500586916	-0.975453266	8.905614638	26.48198539	7.55933E-06	6.97054E-05	PREDICTED: sphingomyelin phosphodiesterase-like
RS003648	GO:0044106	cellular amine metabolic process	BP	1.268807283	1.776217158	0.41179627	5.528161964	23.03387344	3.97318E-05	0.000329264	PREDICTED: protein singed
RS007468	GO:0044106	cellular amine metabolic process	BP	1.554961358	-0.015902902	0.482470573	9.656522243	22.44534397	5.26932E-05	0.0004279	PREDICTED: retinal dehydrogenase 1
RS006642	GO:0044106	cellular amine metabolic process	BP	-0.637747527	1.174042561	-0.357044754	3.154543638	21.5719212	8.00738E-05	0.000631986	PREDICTED: aromatic-L-amino-acid decarboxylase
RS009998	GO:0044106	cellular amine metabolic process	BP	-0.09363052	0.506668162	1.222395436	5.448013038	20.12058269	0.00016025	0.001201688	PREDICTED: tryptophan 2,3-dioxygenase
RS006985	GO:0044106	cellular amine metabolic process	BP	-0.522671357	0.739758227	-0.391833734	5.350293084	18.78443454	0.00030294	0.002167353	PREDICTED: probable 2-oxoglutarate dehydrogenase E1 component DHKTD1 homolog, mitochondrial
RS010115	GO:0044106	cellular amine metabolic process	BP	-0.130017178	1.261118944	-0.650626648	1.761465135	17.35059423	0.000598577	0.004053643	PREDICTED: tryptophan 5-hydroxylase 1
RS015505	GO:0044106	cellular amine metabolic process	BP	-0.333963264	-1.254068775	-0.333900304	7.203178713	16.32816273	0.000971162	0.006353837	PREDICTED: probable methylmalonate-semialdehyde dehydrogenase [acylating], mitochondrial
RS008573	GO:0044106	cellular amine metabolic process	BP	-0.877847198	-1.285642597	-0.317627515	5.276427543	15.89720024	0.001190362	0.007667666	PREDICTED: probable aspartate aminotransferase, cytoplasmic
RS007469	GO:0044106	cellular amine metabolic process	BP	0.109921824	-1.512321655	-1.260766781	10.37063405	14.46509492	0.002335814	0.014022266	PREDICTED: retinal dehydrogenase 1
RS010097	GO:0044106	cellular amine metabolic process	BP	0.17435877	-0.952852764	-0.044428493	4.263987481	10.44397577	0.015145798	0.072762866	PREDICTED: spermine synthase
RS002083	GO:0044106	cellular amine metabolic process	BP	0.177250401	-0.761079147	-0.577437436	8.133614126	10.062607	0.018041381	0.084660246	PREDICTED: aspartate aminotransferase, mitochondrial
RS008575	GO:0044106	cellular amine metabolic process	BP	0.285937384	-0.731842967	-0.265423735	6.102447533	9.775911583	0.020570235	0.094235294	PREDICTED: probable aspartate aminotransferase, cytoplasmic
RS001672	GO:0044106	cellular amine metabolic process	BP	0.307357992	0.966128919	-0.03567067	4.711156529	8.400416794	0.038422093	0.159922993	PREDICTED: catalase
RS007471	GO:0044106	cellular amine metabolic process	BP	0.488223208	0.356479238	-0.43697813	8.831136905	7.56070493	0.056019028	0.215925459	PREDICTED: lambda-crystallin homolog
RS005623	GO:0044106	cellular amine metabolic process	BP	-0.207148985	0.861315813	0.148164528	5.401930947	7.439227213	0.059140699	0.225072403	PREDICTED: sphingomyelin phosphodiesterase
RS011854	GO:0044106	cellular amine metabolic process	BP	-0.744270551	0.041529434	-0.702768939	7.795747502	7.164883594	0.066823987	0.248104681	PREDICTED: aldehyde dehydrogenase, dimeric NADP-preferring
RS006400	GO:0044106	cellular amine metabolic process	BP	0.791751698	-0.199311153	0.789475208	3.864467767	6.803637183	0.07842698	0.281327411	PREDICTED: leucine-rich repeat-containing protein 47-like
RS001701	GO:0044106	cellular amine metabolic process	BP	0.123419788	-1.195769507	-0.558171808	9.403198181	6.717198573	0.08147924	0.289544699	PREDICTED: ornithine decarboxylase 1-like
RS009874	GO:0044106	cellular amine metabolic process	BP	0.672394618	0.650048825	0.385379854	5.350943263	6.344436273	0.096003635	0.327649153	PREDICTED: spermidine synthase
RS013013	GO:0044106	cellular amine metabolic process	BP	0.208074223	1.342259058	-0.041659798	4.182064207	6.279402018	0.09878034	0.33526706	PREDICTED: peptidoglycan-recognition protein LB-like
RS000758	GO:0044106	cellular amine metabolic process	BP	0.33512919	-0.05711749	-0.463250935	7.63864689	5.751926409	0.124323472	0.394805661	PREDICTED: kynurenine--oxoglutarate transaminase 3
RS011781	GO:0044106	cellular amine metabolic process	BP	0.194868998	-0.290152413	-0.647996521	9.541772745	5.013408504	0.170817937	0.49469854	PREDICTED: enolase
RS010080	GO:0044106	cellular amine metabolic process	BP	-0.624995317	-0.751441981	-0.311360855	8.760336944	4.839771278	0.183912955	0.520034833	PREDICTED: 2-oxoglutarate dehydrogenase, mitochondrial
RS000725	GO:0044106	cellular amine metabolic process	BP	-0.160887996	-0.686070869	-0.437727278	8.133751905	4.569821729	0.206146263	0.558946915	PREDICTED: neuropathy target esterase sws
RS013397	GO:0044106	cellular amine metabolic process	BP	0.276762068	-0.133208457	-0.437714221	7.582877367	4.164631585	0.244226876	0.623342031	PREDICTED: delta(3,5)-Delta(2,4)-dienoyl-CoA isomerase, mitochondrial
RS009609	GO:0044106	cellular amine metabolic process	BP	0.210078244	-0.06842499	-0.397693417	4.048414359	3.700886628	0.295627068	0.69337205	PREDICTED: S-methyl-5'-thioadenosine phosphorylase
RS014514	GO:0044106	cellular amine metabolic process	BP	0.454293395	0.189819706	-0.159566019	8.374000108	3.535332448	0.316208451	0.719511677	PREDICTED: trifunctional enzyme subunit alpha, mitochondrial
RS015233	GO:0044106	cellular amine metabolic process	BP	0.540850916	0.558601533	0.185240719	3.554345825	3.3904938	0.335244879	0.740735907	PREDICTED: kynurenine formamidase
RS013491	GO:0044106	cellular amine metabolic process	BP	-0.181773747	-0.876367231	-0.869756564	4.885158359	3.065746684	0.381598996	0.790214932	PREDICTED: amine oxidase [flavin-containing] B
RS011393	GO:0044106	cellular amine metabolic process	BP	0.02917404	-0.508183169	0.050743806	7.352702277	3.024628885	0.387843452	0.796886111	PREDICTED: hydroxyacyl-coenzyme A dehydrogenase, mitochondrial-like
RS001973	GO:0044106	cellular amine metabolic process	BP	0.055182938	0.849890155	0.027680752	1.268346164	2.932735676	0.402112703	0.809489057	PREDICTED: zinc finger protein 177-like
RS013885	GO:0044106	cellular amine metabolic process	BP	-1.296946996	-0.308203642	-1.037010877	0.265185356	2.784418498	0.426071337	0.832842713	PREDICTED: tyrosine decarboxylase
RS002090	GO:0044106	cellular amine metabolic process	BP	-0.035193593	0.360328133	-0.131582113	4.860304023	2.500878624	0.475132317	0.871772722	PREDICTED: mitochondrial ribosome-associated GTPase 2
RS002642	GO:0044106	cellular amine metabolic process	BP	0.327968475	-0.152878617	-0.267497897	5.475828266	2.41164637	0.491470337	0.881592142	PREDICTED: acetyl-CoA acetyltransferase, cytosolic
RS002932	GO:0044106	cellular amine metabolic process	BP	0.285330244	-0.183360297	-0.196925118	7.264005384	2.38943308	0.495604683	0.88489721	PREDICTED: aldehyde dehydrogenase, mitochondrial
RS003701	GO:0044106	cellular amine metabolic process	BP	0.050229794	0.499103503	0.080945241	6.654471113	2.36698837	0.499809308	0.885993958	PREDICTED: probable deoxyhypusine synthase
RS011797	GO:0044106	cellular amine metabolic process	BP	0.384815831	0.110590536	0.031157322	6.971559534	2.046333709	0.562845874	0.920617663	PREDICTED: tyrosine--tRNA ligase, cytoplasmic
RS008554	GO:0044106	cellular amine metabolic process	BP	0.26061457	-0.150564831	0.073116667	6.805535643	2.011998083	0.569920196	0.923921867	PREDICTED: probable phenylalanine--tRNA ligase beta subunit
RS014702	GO:0044106	cellular amine metabolic process	BP	0.053889903	0.000945402	-0.337929036	6.872299671	1.971861167	0.578267518	0.929137767	PREDICTED: glutaryl-CoA dehydrogenase, mitochondrial
RS001412	GO:0044106	cellular amine metabolic process	BP	-0.432560841	0.07421703	-0.330687573	10.62227785	1.773660119	0.620683494	0.951636173	catalase
RS002269	GO:0044106	cellular amine metabolic process	BP	-0.115899934	-0.045489339	-0.35700385	6.156428651	1.746693481	0.626603143	0.953618312	PREDICTED: succinate-semialdehyde dehydrogenase, mitochondrial
RS000318	GO:0044106	cellular amine metabolic process	BP	-0.088415405	0.316315043	-0.022734089	7.458187859	1.720429115	0.632401528	0.95623074	PREDICTED: alpha-aminoadipic semialdehyde dehydrogenase
RS012407	GO:0044106	cellular amine metabolic process	BP	-0.056883468	0.282034432	0.214431303	6.135655265	1.676615982	0.64214505	0.961518328	PREDICTED: probable phenylalanine--tRNA ligase, mitochondrial
RS007076	GO:0044106	cellular amine metabolic process	BP	-0.307781838	-0.12916029	-0.445208222	6.08361277	1.518426518	0.678024076	0.976798812	PREDICTED: serine palmitoyltransferase 1
RS013884	GO:0044106	cellular amine metabolic process	BP	-0.632046343	-0.62122136	-0.1334654	1.884245628	1.381585071	0.709856913	0.989330549	PREDICTED: aromatic-L-amino-acid decarboxylase-like
RS013476	GO:0044106	cellular amine metabolic process	BP	0.198784997	0.042497253	-0.109203051	4.200710783	0.77622549	0.855144718	0.998581907	PREDICTED: diamine acetyltransferase 2-like
RS001454	GO:0044106	cellular amine metabolic process	BP	0.068681475	-0.060934606	-0.164757009	5.079370103	0.528175367	0.912659209	0.998581907	PREDICTED: tryptophan--tRNA ligase, cytoplasmic
RS004573	GO:0044106	cellular amine metabolic process	BP	0.017752395	0.123015371	-0.190111273	5.974861078	0.679282781	0.878065689	0.998581907	PREDICTED: tryptophan--tRNA ligase, mitochondrial
RS009535	GO:0044106	cellular amine metabolic process	BP	-0.056065203	0.092504311	-0.098751469	6.062674479	0.266403412	0.966218072	0.998581907	PREDICTED: delta-1-pyrroline-5-carboxylate dehydrogenase, mitochondrial
RS011502	GO:0044106	cellular amine metabolic process	BP	0.178723895	0.074408536	-0.154428329	7.266745855	1.03873978	0.791879214	0.998581907	PREDICTED: 3-hydroxyacyl-CoA dehydrogenase type-2
RS003577	GO:0044106	cellular amine metabolic process	BP	0.046547345	-0.14594425	0.038124755	5.480941987	0.544458042	0.90902107	0.998581907	PREDICTED: probable phenylalanine--tRNA ligase alpha subunit
RS010099	GO:0044106	cellular amine metabolic process	BP	0.100814249	0.052396139	-0.166198377	8.020158747	0.756965839	0.859730062	0.998581907	PREDICTED: protein transport protein Sec24A
RS012839	GO:0044106	cellular amine metabolic process	BP	0.292242717	0.226216567	0.09849742	4.671305028	0.96001386	0.810926117	0.998581907	PREDICTED: tyrosine--tRNA ligase, mitochondrial
RS013008	GO:0044106	cellular amine metabolic process	BP	0.250876024	0.120317607	0.240583891	4.769299517	0.556791171	0.906248768	0.998581907	PREDICTED: probable phenylalanine--tRNA ligase alpha subunit
RS002780	GO:0044106	cellular amine metabolic process	BP	0.045465929	-0.189873187	-0.126863793	4.210389331	0.543847218	0.909158008	0.998581907	PREDICTED: S-adenosylmethionine decarboxylase proenzyme
RS009744	GO:0006576	cellular biogenic amine metabolic process	BP	3.201394793	9.259149545	2.881573793	2.526912099	116.4189494	4.55598E-25	1.68285E-23	PREDICTED: sphingomyelin phosphodiesterase-like
RS014580	GO:0006576	cellular biogenic amine metabolic process	BP	-2.238409419	-4.554196395	-0.83576737	3.778928597	98.73572107	2.90617E-21	8.73744E-20	PREDICTED: kynurenine formamidase-like
RS003967	GO:0006576	cellular biogenic amine metabolic process	BP	-0.834456324	4.291752911	4.483141964	7.868795768	96.08447484	1.07954E-20	3.14331E-19	ebony
RS006640	GO:0006576	cellular biogenic amine metabolic process	BP	0	8.362754097	5.318907063	1.840319552	94.18289464	2.76637E-20	7.86014E-19	PREDICTED: aromatic-L-amino-acid decarboxylase-like
RS001654	GO:0006576	cellular biogenic amine metabolic process	BP	1.733073604	-4.46574722	-0.501137925	5.066069368	73.06021491	9.4359E-16	2.10655E-14	PREDICTED: catalase
RS009746	GO:0006576	cellular biogenic amine metabolic process	BP	-2.291159813	-2.901001572	-0.149472189	5.118614926	71.34595412	2.19792E-15	4.71181E-14	PREDICTED: sphingomyelin phosphodiesterase-like
RS010135	GO:0006576	cellular biogenic amine metabolic process	BP	1.840019098	-2.32625182	0.732787728	7.759250511	67.76710296	1.28317E-14	2.58392E-13	PREDICTED: protein henna
RS007624	GO:0006576	cellular biogenic amine metabolic process	BP	-1.038705777	-2.417981438	1.4929059	2.541304599	63.78671286	9.11699E-14	1.74098E-12	PREDICTED: tyrosine aminotransferase
RS011394	GO:0006576	cellular biogenic amine metabolic process	BP	-0.808232837	-2.973798175	-1.001114062	5.701841087	63.34834224	1.13133E-13	2.15086E-12	PREDICTED: hydroxyacyl-coenzyme A dehydrogenase, mitochondrial-like
RS100005	GO:0006576	cellular biogenic amine metabolic process	BP	-2.512831781	-3.07482716	-0.793132985	4.249756653	57.0339543	2.52735E-12	4.2935E-11	beta-glucosidase
RS011853	GO:0006576	cellular biogenic amine metabolic process	BP	-0.198391061	-2.402376009	-1.315737207	5.883199768	42.14749002	3.73315E-09	5.06955E-08	PREDICTED: aldehyde dehydrogenase, dimeric NADP-preferring
RS005123	GO:0006576	cellular biogenic amine metabolic process	BP	2.214245853	4.384368384	1.931581004	5.434815601	37.74744679	3.19676E-08	3.88783E-07	PREDICTED: kynurenine formamidase-like
RS009745	GO:0006576	cellular biogenic amine metabolic process	BP	1.717491152	-2.500586916	-0.975453266	8.905614638	26.48198539	7.55933E-06	6.97054E-05	PREDICTED: sphingomyelin phosphodiesterase-like
RS003648	GO:0006576	cellular biogenic amine metabolic process	BP	1.268807283	1.776217158	0.41179627	5.528161964	23.03387344	3.97318E-05	0.000329264	PREDICTED: protein singed
RS007468	GO:0006576	cellular biogenic amine metabolic process	BP	1.554961358	-0.015902902	0.482470573	9.656522243	22.44534397	5.26932E-05	0.0004279	PREDICTED: retinal dehydrogenase 1
RS006642	GO:0006576	cellular biogenic amine metabolic process	BP	-0.637747527	1.174042561	-0.357044754	3.154543638	21.5719212	8.00738E-05	0.000631986	PREDICTED: aromatic-L-amino-acid decarboxylase
RS009998	GO:0006576	cellular biogenic amine metabolic process	BP	-0.09363052	0.506668162	1.222395436	5.448013038	20.12058269	0.00016025	0.001201688	PREDICTED: tryptophan 2,3-dioxygenase
RS006985	GO:0006576	cellular biogenic amine metabolic process	BP	-0.522671357	0.739758227	-0.391833734	5.350293084	18.78443454	0.00030294	0.002167353	PREDICTED: probable 2-oxoglutarate dehydrogenase E1 component DHKTD1 homolog, mitochondrial
RS010115	GO:0006576	cellular biogenic amine metabolic process	BP	-0.130017178	1.261118944	-0.650626648	1.761465135	17.35059423	0.000598577	0.004053643	PREDICTED: tryptophan 5-hydroxylase 1
RS015505	GO:0006576	cellular biogenic amine metabolic process	BP	-0.333963264	-1.254068775	-0.333900304	7.203178713	16.32816273	0.000971162	0.006353837	PREDICTED: probable methylmalonate-semialdehyde dehydrogenase [acylating], mitochondrial
RS008573	GO:0006576	cellular biogenic amine metabolic process	BP	-0.877847198	-1.285642597	-0.317627515	5.276427543	15.89720024	0.001190362	0.007667666	PREDICTED: probable aspartate aminotransferase, cytoplasmic
RS007469	GO:0006576	cellular biogenic amine metabolic process	BP	0.109921824	-1.512321655	-1.260766781	10.37063405	14.46509492	0.002335814	0.014022266	PREDICTED: retinal dehydrogenase 1
RS010097	GO:0006576	cellular biogenic amine metabolic process	BP	0.17435877	-0.952852764	-0.044428493	4.263987481	10.44397577	0.015145798	0.072762866	PREDICTED: spermine synthase
RS002083	GO:0006576	cellular biogenic amine metabolic process	BP	0.177250401	-0.761079147	-0.577437436	8.133614126	10.062607	0.018041381	0.084660246	PREDICTED: aspartate aminotransferase, mitochondrial
RS008575	GO:0006576	cellular biogenic amine metabolic process	BP	0.285937384	-0.731842967	-0.265423735	6.102447533	9.775911583	0.020570235	0.094235294	PREDICTED: probable aspartate aminotransferase, cytoplasmic
RS001672	GO:0006576	cellular biogenic amine metabolic process	BP	0.307357992	0.966128919	-0.03567067	4.711156529	8.400416794	0.038422093	0.159922993	PREDICTED: catalase
RS007471	GO:0006576	cellular biogenic amine metabolic process	BP	0.488223208	0.356479238	-0.43697813	8.831136905	7.56070493	0.056019028	0.215925459	PREDICTED: lambda-crystallin homolog
RS005623	GO:0006576	cellular biogenic amine metabolic process	BP	-0.207148985	0.861315813	0.148164528	5.401930947	7.439227213	0.059140699	0.225072403	PREDICTED: sphingomyelin phosphodiesterase
RS011854	GO:0006576	cellular biogenic amine metabolic process	BP	-0.744270551	0.041529434	-0.702768939	7.795747502	7.164883594	0.066823987	0.248104681	PREDICTED: aldehyde dehydrogenase, dimeric NADP-preferring
RS006400	GO:0006576	cellular biogenic amine metabolic process	BP	0.791751698	-0.199311153	0.789475208	3.864467767	6.803637183	0.07842698	0.281327411	PREDICTED: leucine-rich repeat-containing protein 47-like
RS001701	GO:0006576	cellular biogenic amine metabolic process	BP	0.123419788	-1.195769507	-0.558171808	9.403198181	6.717198573	0.08147924	0.289544699	PREDICTED: ornithine decarboxylase 1-like
RS009874	GO:0006576	cellular biogenic amine metabolic process	BP	0.672394618	0.650048825	0.385379854	5.350943263	6.344436273	0.096003635	0.327649153	PREDICTED: spermidine synthase
RS013013	GO:0006576	cellular biogenic amine metabolic process	BP	0.208074223	1.342259058	-0.041659798	4.182064207	6.279402018	0.09878034	0.33526706	PREDICTED: peptidoglycan-recognition protein LB-like
RS000758	GO:0006576	cellular biogenic amine metabolic process	BP	0.33512919	-0.05711749	-0.463250935	7.63864689	5.751926409	0.124323472	0.394805661	PREDICTED: kynurenine--oxoglutarate transaminase 3
RS011781	GO:0006576	cellular biogenic amine metabolic process	BP	0.194868998	-0.290152413	-0.647996521	9.541772745	5.013408504	0.170817937	0.49469854	PREDICTED: enolase

RS010080	GO:0006576	cellular biogenic amine metabolic process	BP	-0.624995317	-0.751441981	-0.311360855	8.760336944	4.839771278	0.183912955	0.520034833 PREDICTED: 2-oxoglutarate dehydrogenase, mitochondrial
RS000725	GO:0006576	cellular biogenic amine metabolic process	BP	-0.160887996	-0.686070869	-0.437727278	8.133751905	4.569821729	0.206146263	0.558946915 PREDICTED: neuropathy target esterase sws
RS013397	GO:0006576	cellular biogenic amine metabolic process	BP	0.276762068	-0.133208457	-0.437714221	7.582877367	4.164631585	0.244226876	0.623342031 PREDICTED: delta(3,5)-Delta(2,4)-dienoyl-CoA isomerase, mitochondrial
RS009609	GO:0006576	cellular biogenic amine metabolic process	BP	0.210078244	-0.06842499	-0.397693417	4.048414359	3.700886628	0.295627068	0.69337205 PREDICTED: S-methyl-5'-thioadenosine phosphorylase
RS014514	GO:0006576	cellular biogenic amine metabolic process	BP	0.454293395	0.189819706	-0.159566019	8.374000108	3.535332448	0.316208451	0.719511677 PREDICTED: trifunctional enzyme subunit alpha, mitochondrial
RS015233	GO:0006576	cellular biogenic amine metabolic process	BP	0.540850916	0.558601533	0.185240719	3.554345825	3.3904938	0.335244879	0.740735907 PREDICTED: kynurenine formamidase
RS013491	GO:0006576	cellular biogenic amine metabolic process	BP	-0.181773747	-0.876367231	-0.869756564	4.885158359	3.065746684	0.381598996	0.790214932 PREDICTED: amine oxidase [flavin-containing] B
RS011393	GO:0006576	cellular biogenic amine metabolic process	BP	0.02917404	-0.508183169	0.050743806	7.352702277	3.024628885	0.387843452	0.796886111 PREDICTED: hydroxyacyl-coenzyme A dehydrogenase, mitochondrial-like
RS001973	GO:0006576	cellular biogenic amine metabolic process	BP	0.055182938	0.849890155	0.027680752	1.268346164	2.932735676	0.402112703	0.809489057 PREDICTED: zinc finger protein 177-like
RS013885	GO:0006576	cellular biogenic amine metabolic process	BP	-1.296946996	-0.308203642	-1.037010877	0.265185356	2.784418498	0.426071337	0.832842713 PREDICTED: tyrosine decarboxylase
RS002090	GO:0006576	cellular biogenic amine metabolic process	BP	-0.035193593	0.360328133	-0.131582113	4.860304023	2.500878624	0.475132317	0.871772722 PREDICTED: mitochondrial ribosome-associated GTPase 2
RS002642	GO:0006576	cellular biogenic amine metabolic process	BP	0.327968475	-0.152878617	-0.267497897	5.475828266	2.41164637	0.491470337	0.881592142 PREDICTED: acetyl-CoA acetyltransferase, cytosolic
RS002932	GO:0006576	cellular biogenic amine metabolic process	BP	0.285330244	-0.183360297	-0.196925118	7.264005384	2.38943308	0.495604683	0.88489721 PREDICTED: aldehyde dehydrogenase, mitochondrial
RS003701	GO:0006576	cellular biogenic amine metabolic process	BP	0.050229794	0.499103503	0.080945241	6.654471113	2.36698837	0.499809308	0.885993958 PREDICTED: probable deoxyhypusine synthase
RS011797	GO:0006576	cellular biogenic amine metabolic process	BP	0.384815831	0.110590536	0.031157322	6.971559534	2.046333709	0.562845874	0.920617663 PREDICTED: tyrosine--tRNA ligase, cytoplasmic
RS008554	GO:0006576	cellular biogenic amine metabolic process	BP	0.26061457	-0.150564831	0.073116667	6.805535643	2.011998083	0.569920196	0.923921867 PREDICTED: probable phenylalanine--tRNA ligase beta subunit
RS014702	GO:0006576	cellular biogenic amine metabolic process	BP	0.053889903	0.000945402	-0.337929036	6.872299671	1.971861167	0.578267518	0.929137767 PREDICTED: glutaryl-CoA dehydrogenase, mitochondrial
RS001412	GO:0006576	cellular biogenic amine metabolic process	BP	-0.432560841	0.07421703	-0.330687573	10.62227785	1.773660119	0.620683494	0.951636173 catalase
RS002269	GO:0006576	cellular biogenic amine metabolic process	BP	-0.115899934	-0.045489339	-0.35700385	6.156428651	1.746693481	0.626603143	0.953618312 PREDICTED: succinate-semialdehyde dehydrogenase, mitochondrial
RS000318	GO:0006576	cellular biogenic amine metabolic process	BP	-0.088415405	0.316315043	-0.022734089	7.458187859	1.720429115	0.632401528	0.95623074 PREDICTED: alpha-aminoadipic semialdehyde dehydrogenase
RS012407	GO:0006576	cellular biogenic amine metabolic process	BP	-0.056883468	0.282034432	0.214431303	6.135655265	1.676615982	0.64214505	0.961518328 PREDICTED: probable phenylalanine--tRNA ligase, mitochondrial
RS007076	GO:0006576	cellular biogenic amine metabolic process	BP	-0.307781838	-0.12916029	-0.445208222	6.08361277	1.518426518	0.678024076	0.976798812 PREDICTED: serine palmitoyltransferase 1
RS013884	GO:0006576	cellular biogenic amine metabolic process	BP	-0.632046343	-0.62122136	-0.1334654	1.884245628	1.381585071	0.709856913	0.989330549 PREDICTED: aromatic-L-amino-acid decarboxylase-like
RS001454	GO:0006576	cellular biogenic amine metabolic process	BP	0.068681475	-0.060934606	-0.164757009	5.079370103	0.528175367	0.912659209	0.998581907 PREDICTED: tryptophan--tRNA ligase, cytoplasmic
RS004573	GO:0006576	cellular biogenic amine metabolic process	BP	0.017752395	0.123015371	-0.190111273	5.974861078	0.679282781	0.878065689	0.998581907 PREDICTED: tryptophan--tRNA ligase, mitochondrial
RS009535	GO:0006576	cellular biogenic amine metabolic process	BP	-0.056065203	0.092504311	-0.098751469	6.062674479	0.266403412	0.966218072	0.998581907 PREDICTED: delta-1-pyrroline-5-carboxylate dehydrogenase, mitochondrial
RS011502	GO:0006576	cellular biogenic amine metabolic process	BP	0.178723895	0.074408536	-0.154428329	7.266745855	1.03873978	0.791879214	0.998581907 PREDICTED: 3-hydroxyacyl-CoA dehydrogenase type-2
RS003577	GO:0006576	cellular biogenic amine metabolic process	BP	0.046547345	-0.14594425	0.038124755	5.480941987	0.544458042	0.90902107	0.998581907 PREDICTED: probable phenylalanine--tRNA ligase alpha subunit
RS010099	GO:0006576	cellular biogenic amine metabolic process	BP	0.100814249	0.052396139	-0.166198377	8.020158747	0.756965839	0.859730062	0.998581907 PREDICTED: protein transport protein Sec24A
RS012839	GO:0006576	cellular biogenic amine metabolic process	BP	0.292242717	0.226216567	0.09849742	4.671305028	0.96001386	0.810926117	0.998581907 PREDICTED: tyrosine--tRNA ligase, mitochondrial
RS013008	GO:0006576	cellular biogenic amine metabolic process	BP	0.250876024	0.120317607	0.240583891	4.769299517	0.556791171	0.906248768	0.998581907 PREDICTED: probable phenylalanine--tRNA ligase alpha subunit
RS013476	GO:0006576	cellular biogenic amine metabolic process	BP	0.198784997	0.042497253	-0.109203051	4.200710783	0.77622549	0.855144718	0.998581907 PREDICTED: diamine acetyltransferase 2-like
RS002780	GO:0006576	cellular biogenic amine metabolic process	BP	0.045465929	-0.189873187	-0.126863793	4.210389331	0.543847218	0.909158008	0.998581907 PREDICTED: S-adenosylmethionine decarboxylase proenzyme
RS010208	GO:0046394	carboxylic acid biosynthetic process	BP	2.631335902	7.346050423	-0.754450343	6.457203767	336.2297923	1.42946E-72	3.48681E-70 PREDICTED: acyl-CoA Delta(11) desaturase-like
RS005642	GO:0046394	carboxylic acid biosynthetic process	BP	-0.634739278	5.138380698	0.16781642	8.170113934	236.0930111	6.6583E-51	6.01948E-49 PREDICTED: elongation of very long chain fatty acids protein 4-like
RS006873	GO:0046394	carboxylic acid biosynthetic process	BP	5.089410523	5.334189165	-2.08043589	4.84259895	170.0556289	1.23782E-36	6.75211E-35 PREDICTED: acyl-CoA Delta(11) desaturase-like
RS006872	GO:0046394	carboxylic acid biosynthetic process	BP	-0.33466745	7.238803945	0.909787823	2.73390939	153.642158	4.31543E-33	2.18784E-31 PREDICTED: acyl-CoA desaturase-like
RS006279	GO:0046394	carboxylic acid biosynthetic process	BP	-0.750412564	2.952200618	2.15034714	5.909933988	146.8352109	1.26889E-31	6.12095E-30 PREDICTED: fatty acid synthase-like
RS001262	GO:0046394	carboxylic acid biosynthetic process	BP	0.334308699	4.247574702	0.34765291	5.028809362	130.6997196	3.8219E-28	1.60421E-26 PREDICTED: elongation of very long chain fatty acids protein AAEL008004
RS001805	GO:0046394	carboxylic acid biosynthetic process	BP	-0.870588518	2.833390077	1.14899972	4.47590462	111.9265088	4.2238E-24	1.50428E-22 PREDICTED: elongation of very long chain fatty acids protein AAEL008004-like
RS000681	GO:0046394	carboxylic acid biosynthetic process	BP	-1.191628428	3.200519867	1.555766858	5.258400593	106.9126587	5.06608E-23	1.69236E-21 PREDICTED: elongation of very long chain fatty acids protein AAEL008004-like
RS013198	GO:0046394	carboxylic acid biosynthetic process	BP	1.398067761	-1.345458381	-0.627931524	6.033691931	101.6972579	6.70695E-22	2.08934E-20 PREDICTED: fatty acid hydroxylase domain-containing protein 2
RS013686	GO:0046394	carboxylic acid biosynthetic process	BP	-0.717818102	-3.908909796	-1.614926839	7.193517866	94.76250858	2.0766E-20	5.96585E-19 PREDICTED: acyl-CoA Delta(11) desaturase-like
RS002678	GO:0046394	carboxylic acid biosynthetic process	BP	1.88850699	2.464921217	-0.538157218	6.037232379	81.46777569	1.4865E-17	3.65352E-16 PREDICTED: fatty acid synthase-like
RS006876	GO:0046394	carboxylic acid biosynthetic process	BP	-1.10740967	-4.875531905	-1.927422906	9.200285447	80.41642171	2.49869E-17	6.0606E-16 PREDICTED: acyl-CoA Delta(11) desaturase-like
RS010135	GO:0046394	carboxylic acid biosynthetic process	BP	1.840019098	-2.32625182	0.732787728	7.759250511	67.76710296	1.28317E-14	2.58392E-13 PREDICTED: protein henna
RS007624	GO:0046394	carboxylic acid biosynthetic process	BP	-1.038705777	-2.417981438	1.4929059	2.541304599	63.78671286	9.11699E-14	1.74098E-12 PREDICTED: tyrosine aminotransferase
RS011394	GO:0046394	carboxylic acid biosynthetic process	BP	-0.808232837	-2.973798175	-1.001114062	5.701841087	63.34834224	1.13133E-13	2.15086E-12 PREDICTED: hydroxyacyl-coenzyme A dehydrogenase, mitochondrial-like
RS013687	GO:0046394	carboxylic acid biosynthetic process	BP	1.493196769	-2.49034267	0.605103464	4.035957267	63.33591434	1.13828E-13	2.16089E-12 PREDICTED: acyl-CoA Delta(11) desaturase-like
RS001358	GO:0046394	carboxylic acid biosynthetic process	BP	1.04922649	-6.683100048	-1.661386259	7.235051984	48.39535779	1.75442E-10	2.64163E-09 putative C-5 sterol desaturase
RS006871	GO:0046394	carboxylic acid biosynthetic process	BP	-0.242054109	1.84490496	-1.948230684	5.642008932	46.08729724	5.4342E-10	7.86711E-09 PREDICTED: acyl-CoA Delta(11) desaturase-like
RS011853	GO:0046394	carboxylic acid biosynthetic process	BP	-0.198391061	-2.402376009	-1.315737207	5.883199768	42.14749002	3.73315E-09	5.06955E-08 PREDICTED: aldehyde dehydrogenase, dimeric NADP-preferring
RS001544	GO:0046394	carboxylic acid biosynthetic process	BP	0.274963904	-1.09360695	-1.805361323	6.749172802	41.74350775	4.54779E-09	6.11798E-08 PREDICTED: alkaline phosphatase 4-like
RS013244	GO:0046394	carboxylic acid biosynthetic process	BP	-2.118886964	-5.134511847	-2.304467706	2.520197622	40.7543233	7.37271E-09	9.7359E-08 PREDICTED: acyl-CoA Delta(11) desaturase-like
RS002190	GO:0046394	carboxylic acid biosynthetic process	BP	-1.374803006	-2.081968712	0.437978506	4.918669629	35.63124542	8.96069E-08	1.03896E-06 PREDICTED: fatty acid 2-hydroxylase
RS009089	GO:0046394	carboxylic acid biosynthetic process	BP	0.780156718	-1.142779879	-0.944341962	6.520545706	35.56337079	9.26164E-08	1.07193E-06 PREDICTED: probable phosphoserine aminotransferase
RS010978	GO:0046394	carboxylic acid biosynthetic process	BP	-0.105950757	-2.718350483	-0.164657054	9.167446834	32.70854985	3.71036E-07	3.99729E-06 PREDICTED: fatty acid synthase
RS005544	GO:0046394	carboxylic acid biosynthetic process	BP	0.405463151	1.50203441	-1.817605524	9.291358907	27.83750659	3.92874E-06	3.76786E-05 PREDICTED: beta-ureidopropionase
RS002535	GO:0046394	carboxylic acid biosynthetic process	BP	-0.730643229	-1.637072136	0.172680139	8.113462827	26.98303681	5.93576E-06	5.54863E-05 PREDICTED: acetyl-CoA carboxylase
RS001261	GO:0046394	carboxylic acid biosynthetic process	BP	-2.235645034	0.224656739	-1.086038105	2.162252071	26.93058301	6.08797E-06	5.68269E-05 PREDICTED: elongation of very long chain fatty acids protein 7
RS003917	GO:0046394	carboxylic acid biosynthetic process	BP	1.262100569	0.159623921	-0.096731621	5.707277733	25.44668354	1.24519E-05	0.000111558 PREDICTED: asparagine synthetase [glutamine-hydrolyzing]
RS005087	GO:0046394	carboxylic acid biosynthetic process	BP	-0.35126078	1.741640675	2.124824608	1.745993058	24.29506603	2.16759E-05	0.000187316 PREDICTED: GTP cyclohydrolase 1
RS002240	GO:0046394	carboxylic acid biosynthetic process	BP	-0.632574541	0.882911763	0.780104643	5.192425356	23.60200145	3.02453E-05	0.000256065 PREDICTED: probable pyruvate dehydrogenase E1 component subunit alpha, mitochondrial
RS003648	GO:0046394	carboxylic acid biosynthetic process	BP	1.268807283	1.776217158	0.41179627	5.528161964	23.03387344	3.97318E-05	0.000329264 PREDICTED: protein singed
RS007468	GO:0046394	carboxylic acid biosynthetic process	BP	1.554961358	-0.015902902	0.482470573	9.656522243	22.44534397	5.26932E-05	0.0004279 PREDICTED: retinal dehydrogenase 1
RS004115	GO:0046394	carboxylic acid biosynthetic process	BP	-3.338103077	-2.132121097	-3.540885575	2.658495116	22.39384183	5.40105E-05	0.000438323 PREDICTED: pyrroline-5-carboxylate reductase 2
RS006877	GO:0046394	carboxylic acid biosynthetic process	BP	0.265818642	-1.05943473	-2.08149481	3.30161852	22.30893928	5.62543E-05	0.000455388 PREDICTED: acyl-CoA Delta(11) desaturase
RS000146	GO:0046394	carboxylic acid biosynthetic process	BP	0.439480055	-1.070680667	-0.239174518	6.074478122	21.95270329	6.67261E-05	0.000533478 PREDICTED: serine hydroxymethyltransferase, mitochondrial
RS010210	GO:0046394	carboxylic acid biosynthetic process	BP	-1.577649228	0.880954578	-0.51929089	3.453369802	21.20123124	9.56162E-05	0.000742418 PREDICTED: acyl-CoA Delta(11) desaturase-like
RS011319	GO:0046394	carboxylic acid biosynthetic process	BP	-1.721607065	-0.37953699	0.32859135	5.346304498	21.05802428	0.000102395	0.000789835 PREDICTED: elongation of very long chain fatty acids protein AAEL008004-like
RS012880	GO:0046394	carboxylic acid biosynthetic process	BP	-0.807344323	-1.895186526	-0.086915908	8.413207767	20.19903013	0.00015436	0.001160887 PREDICTED: C-1-tetrahydrofolate synthase, cytoplasmic
RS013389	GO:0046394	carboxylic acid biosynthetic process	BP	-0.662472987	-1.005914654	0.744834151	7.265622757	19.41054869	0.000224837	0.001636653 PREDICTED: propionyl-CoA carboxylase alpha chain, mitochondrial
RS013246	GO:0046394	carboxylic acid biosynthetic process	BP	0.026801121	-2.623174489	-1.205156148	1.300160489	18.64961266	0.000323008	0.002299477 PREDICTED: acyl-CoA Delta(11) desaturase-like
RS008510	GO:0046394	carboxylic acid biosynthetic process	BP	-0.007861455	1.657826497	1.802497318	2.194239545	18.48937541	0.000348586	0.002469326 PREDICTED: GTP cyclohydrolase 1
RS000267	GO:0046394	carboxylic acid biosynthetic process	BP	-0.550819695	-1.165273853	0.191627701	5.343147996	18.17657843	0.000404462	0.002829484 PREDICTED: argininosuccinate lyase
RS010737	GO:0046394	carboxylic acid biosynthetic process	BP	0.07086237	1.016941323	1.268374972	5.272102935	16.67839044	0.000822948	0.005444763 PREDICTED: elongation of very long chain fatty acids protein AAEL008004
RS013655	GO:0046394	carboxylic acid biosynthetic process	BP	-0.256056345	1.603293117	0.591268857	9.642915994	16.658626	0.000830678	0.0054931 PREDICTED: transforming growth factor-beta-induced protein ig-h3-like
RS015505	GO:0046394	carboxylic acid biosynthetic process	BP	-0.333963264	-1.254068775	-0.333900304	7.203178713	16.32816273	0.000971162	0.006353837 PREDICTED: probable methylmalonate-semialdehyde dehydrogenase [acylating], mitochondrial
RS008573	GO:0046394	carboxylic acid biosynthetic process	BP	-0.877847198	-1.285642597	-0.317627515	5.276427543	15.89720024	0.001190362	0.007667666 PREDICTED: probable aspartate aminotransferase, cytoplasmic
RS007469	GO:0046394	carboxylic acid biosynthetic process	BP	0.109921824	-1.512321655	-1.260766781	10.37063405	14.46509492	0.002335814	0.014022266 PREDICTED: retinal dehydrogenase 1
RS013245	GO:0046394	carboxylic acid biosynthetic process	BP	0.205086194	-2.137451995	-0.303300922	1.873173886	13.92971456	0.003002401	0.017603194 PREDICTED: acyl-CoA Delta(11) desaturase-like
RS013640	GO:0046394	carboxylic acid biosynthetic process	BP	-0.507605075	0.88378812	-0.370589145	3.007371753	13.90545704	0.003036708	0.017788199 PREDICTED: alkaline phosphatase-like
RS010105	GO:0046394	carboxylic acid biosynthetic process	BP	0.005457692	-0.665472681	-1.12936068	7.211526424	13.47669162	0.003711352	0.021249053 PREDICTED: elongation of very long chain fatty acids protein 6
RS015477	GO:0046394	carboxylic acid biosynthetic process	BP	-0.897264721	-1.291332363	-0.434228581	5.338765823	13.37548035	0.003891121	0.022209456 PREDICTED: pyruvate carboxylase, mitochondrial
RS011903	GO:0046394	carboxylic acid biosynthetic process	BP	0.785032756	-0.185002642	0.61952285	5.789786927	13.31924568	0.003994703	0.022750447 PREDICTED: CAD protein
RS007097	GO:0046394	carboxylic acid biosynthetic process	BP	0.795617782	-0.143458305	-0.302194899	4.974094685	13.15347812	0.004316187	0.024302988 PREDICTED: methylcrotonoyl-CoA carboxylase subunit alpha, mitochondrial
RS010159	GO:0046394	carboxylic acid biosynthetic process	BP	-0.646502633	0.639073484	-0.488386035	5.47525374	12.90472858	0.004847231	0.02695269 PREDICTED: delta-1-pyrroline-5-carboxylate synthase
RS001674	GO:0046394	carboxylic acid biosynthetic process	BP	-0.383074682	-0.838511587	0.254025522	6.390380362	12.56965972	0.005665996	0.031011855 PREDICTED: cystathionine beta-synthase
RS006613	GO:0046394	carboxylic acid biosynthetic process	BP	0.452094938	-0.623868558	0.040645578	5.891109809	12.3268201	0.006343489	0.034284545 PREDICTED: enolase-phosphatase E1
RS005142	GO:0046394	carboxylic acid biosynthetic process	BP	-0.386591935	-1.296698312	-1.484278386	1.705974468	10.73484454	0.013249577	0.065055273 PREDICTED: methylenetetrahydrofolate reductase
RS007239	GO:0046394	carboxylic acid biosynthetic process	BP	-0.80039341	-0.711870169	-2.286672983	9.552046577	10.33732368	0.015906021	0.075795442 PREDICTED: D-3-phosphoglycerate dehydrogenase
RS002083	GO:0046394	carboxylic acid biosynthetic process	BP	0.177250401	-0.761079147	-0.577437436	8.133614126	10.062607	0.018041381	0.084660246 PREDICTED: aspartate aminotransferase, mitochondrial
RS011651	GO:0046394	carboxylic acid biosynthetic process	BP	0.27609398	-1.005784534	0.026713349	8.399697414	9.976153219	0.018769933	0.087538852 PREDICTED: fatty acid synthase-like
RS008575	GO:0046394	carboxylic acid biosynthetic process	BP	0.285937384	-0.731842967	-0.265423735	6.102447533	9.775911583	0.020570235	0.094235294 PREDICTED: probable aspartate aminotransferase, cytoplasmic
RS003105	GO:0046394	carboxylic acid biosynthetic process	BP	0.093511576	0.720219714	0.022101776	5.289399073	8.772772376	0.032469645	0.138903896 PREDICTED: alkylglycerol monooxygenase-like
RS010012	GO:0046394	carboxylic acid biosynthetic process	BP	0.014321587	0.739129238	0.71793314	5.662732627	8.6765526	0.033915232	0.143944884 PREDICTED: probable low-specificity L-threonine aldolase 2
RS010957	GO:0046394	carboxylic acid biosynthetic process	BP	-0.597747337	-0.61322871	-0.993570939	7.669505911	8.638947236	0.034497148	0.146126846 PREDICTED: ornithine aminotransferase, mitochondrial
RS015101	GO:0046394	carboxylic acid biosynthetic process	BP	0.185501806	-0.365075161	-0.63038581	6.561629341	8.335808276	0.039558189	0.163703031 PREDICTED: 2-aminoethylphosphonate--pyruvate transaminase-like
RS005894	GO:0046394	carboxylic acid biosynthetic process	BP	-0.450956232	-0.957793122	-0.804806328	8.60102836	8.306166461	0.040090329	0.16548141 PREDICTED: pantothenate kinase 3
RS007596	GO:0046394	carboxylic acid biosynthetic process	BP	-0.809534745	-0.666982264	0.578146648	4.874681874	7.980578839	0.046414792	0.186004475 PREDICTED: fatty acid synthase-like
RS007471	GO:0046394	carboxylic acid biosynthetic process	BP	0.488223208	0.356479238	-0.43697813	8.831136905	7.56070493	0.056019028	0.215925459 PREDICTED: lambda-crystallin homolog
RS010209	GO:0046394	carboxylic acid biosynthetic process	BP	-0.767235758	0.184554649	-0.438670204	6.018691533	7.178862894	0.066410121	0.247135878 PREDICTED: acyl-CoA Delta(11) desaturase
RS011854	GO:0046394	carboxylic acid biosynthetic process	BP	-0.744270551	0.041529434	-0.702768939	7.795747502	7.164883594	0.066823987	0.248104681 PREDICTED: aldehyde dehydrogenase, dimeric NADP-preferring
RS007595	GO:0046394	carboxylic acid biosynthetic process	BP	-1.222953796	-0.412402431	-0.073293638	6.073896472	6.930709003	0.074139413	0.269207268 PREDICTED: fatty acid synthase-like
RS006400	GO:0046394	carboxylic acid biosynthetic process	BP	0.791751698	-0.199311153	0.789475208	3.864467767	6.803637183	0.07842698	0.281327411 PREDICTED: leucine-rich repeat-containing protein 47-like
RS001514	GO:0046394	carboxylic acid biosynthetic process	BP	0.259150932	-0.508483967	-0.206376495	6.407252152	6.444096885	0.091893449	0.316041105 PREDICTED: pantothenate kinase 4
RS006504	GO:0046394	carboxylic acid biosynthetic process	BP	0.429662448	0.076826048	-0.459250569	4.576152449	5.755047279	0.124155283	0.394562316 PREDICTED: 5-formyltetrahydrofolate cyclo-ligase
RS012801	GO:0046394	carboxylic acid biosynthetic process	BP	-0.305373229	-0.483960269	-0.704209767	4.980361046	5.699688733	0.127171057	0.401776007 PREDICTED: trimethyllysine dioxygenase, mitochondrial
RS014856	GO:0046394	carboxylic acid biosynthetic process	BP	0.143481668	-0.166733568	0.485678581	6.941672661	5.624645258	0.131370639	0.410823283 PREDICTED: lysine--tRNA ligase
RS003393	GO:0046394	carboxylic acid biosynthetic process	BP	0.293569646	-0.648014171	-0.531549171	8.391513924	5.38529422	0.145662549	0.443610233 PREDICTED: leukotriene A-4 hydrolase
RS000178	GO:0046394	carboxylic acid biosynthetic process	BP	0.187222857	-0.580152531	-0.650904567	5.507795536	5.118447643	0.163326473	0.479848848 PREDICTED: argininosuccinate synthase

RS012653	GO:0046394	carboxylic acid biosynthetic process	BP	0.457094882	-0.098825212	-0.22264278	4.112007138	5.014152731	0.17076374	0.494652394 PREDICTED: 3-oxoacyl-[acyl-carrier-protein] synthase, mitochondrial
RS011781	GO:0046394	carboxylic acid biosynthetic process	BP	0.194868998	-0.290152413	-0.647996521	9.541772745	5.013408504	0.170817937	0.49469854 PREDICTED: enolase
RS014512	GO:0046394	carboxylic acid biosynthetic process	BP	-0.141977713	-0.560717408	-0.429830427	6.661293348	4.739753613	0.191876097	0.533343557 PREDICTED: cysteine desulfurase, mitochondrial
RS014553	GO:0046394	carboxylic acid biosynthetic process	BP	-0.211371951	-1.732733164	0.093753139	0.949304848	4.739747226	0.191876615	0.533343557 PREDICTED: fatty acid synthase-like
RS007594	GO:0046394	carboxylic acid biosynthetic process	BP	-0.717242258	-0.207577159	0.27013743	8.519802645	4.633705425	0.200669656	0.549838123 PREDICTED: fatty acid synthase
RS007875	GO:0046394	carboxylic acid biosynthetic process	BP	0.142712287	-0.392317307	-0.251606633	6.099847719	4.332732172	0.227704312	0.595300577 PREDICTED: splicing factor 3B subunit 6-like protein
RS012259	GO:0046394	carboxylic acid biosynthetic process	BP	-0.651022963	-0.413329834	-0.034985405	8.655623119	4.197617943	0.240900483	0.618788285 PREDICTED: putative glutamate synthase [NADPH]
RS013197	GO:0046394	carboxylic acid biosynthetic process	BP	-0.368534608	-0.921242977	-0.538013398	7.366413675	4.1646868	0.244221273	0.623342031 PREDICTED: fatty acid hydroxylase domain-containing protein 2
RS013397	GO:0046394	carboxylic acid biosynthetic process	BP	0.276762068	-0.133208457	-0.437714221	7.582877367	4.164631585	0.244226876	0.623342031 PREDICTED: delta(3,5)-Delta(2,4)-dienoyl-CoA isomerase, mitochondrial
RS006104	GO:0046394	carboxylic acid biosynthetic process	BP	0.050741232	0.067982723	-0.679870588	9.220750703	3.978415949	0.26380427	0.654473536 PREDICTED: glutamine synthetase 2 cytoplasmic
RS001242	GO:0046394	carboxylic acid biosynthetic process	BP	-0.066255895	-0.531998073	-0.515824902	6.37443736	3.732497257	0.291836068	0.68973614 PREDICTED: 1,2-dihydroxy-3-keto-5-methylthiopentene dioxygenase
RS009609	GO:0046394	carboxylic acid biosynthetic process	BP	0.210078244	-0.06842499	-0.397693417	4.048414359	3.700886628	0.295627068	0.69337205 PREDICTED: S-methyl-5'-thioadenosine phosphorylase
RS001808	GO:0046394	carboxylic acid biosynthetic process	BP	-0.058607321	-0.547033169	0.506368301	2.661379544	3.681395157	0.297986539	0.696883137 PREDICTED: elongation of very long chain fatty acids protein AAEL008004-like
RS013812	GO:0046394	carboxylic acid biosynthetic process	BP	-0.236673306	-0.669275541	-0.156642409	5.411761702	3.625031862	0.304904278	0.707049777 PREDICTED: propionyl-CoA carboxylase beta chain, mitochondrial
RS014514	GO:0046394	carboxylic acid biosynthetic process	BP	0.454293395	0.189819706	-0.159566019	8.374000108	3.535332448	0.316208451	0.719511677 PREDICTED: trifunctional enzyme subunit alpha, mitochondrial
RS000797	GO:0046394	carboxylic acid biosynthetic process	BP	-0.378894012	-0.143187194	0.419208563	4.992333982	3.492017406	0.321798913	0.726929294 stripe
RS013622	GO:0046394	carboxylic acid biosynthetic process	BP	1.304002696	-0.790491216	0.213979312	6.381917919	3.432062346	0.329680888	0.734589515 PREDICTED: membrane-bound alkaline phosphatase-like
RS010695	GO:0046394	carboxylic acid biosynthetic process	BP	0.167669466	-0.139565623	-0.427342962	4.043742135	3.369399197	0.338099779	0.74419257 PREDICTED: dihydrofolate reductase
RS008584	GO:0046394	carboxylic acid biosynthetic process	BP	0.292833023	-0.220059953	-0.331841648	2.908313798	3.364840859	0.338719484	0.745047978 PREDICTED: thymidylate synthase
RS009254	GO:0046394	carboxylic acid biosynthetic process	BP	0.39225852	0.2856945	-0.18015587	6.987646529	3.296181412	0.348174475	0.75656684 PREDICTED: histidine triad nucleotide-binding protein 1
RS010718	GO:0046394	carboxylic acid biosynthetic process	BP	0.477981617	0.174790255	-0.000903128	4.437079375	3.242803222	0.355683034	0.764213108 PREDICTED: pyrroline-5-carboxylate reductase 2
RS003843	GO:0046394	carboxylic acid biosynthetic process	BP	0.523865093	0.896702368	0.950902499	1.712239692	3.188197528	0.36350902	0.771665781 Homeobox protein ceh-9
RS013983	GO:0046394	carboxylic acid biosynthetic process	BP	0.163996819	-0.412664616	-0.172942215	4.146360298	3.170825747	0.366029671	0.775234533 PREDICTED: valine--tRNA ligase-like
RS011393	GO:0046394	carboxylic acid biosynthetic process	BP	0.02917404	-0.508183169	0.050743806	7.352702277	3.024628885	0.387843452	0.796886111 PREDICTED: hydroxyacyl-coenzyme A dehydrogenase, mitochondrial-like
RS002533	GO:0046394	carboxylic acid biosynthetic process	BP	0.009324912	-0.457888597	-0.251888724	5.442114645	2.939900614	0.400984439	0.808724935 PREDICTED: sepiapterin reductase-like
RS006771	GO:0046394	carboxylic acid biosynthetic process	BP	0.501095992	0.373222122	0.613510743	5.629228769	2.925902508	0.403191208	0.81102473 PREDICTED: C-terminal-binding protein
RS007071	GO:0046394	carboxylic acid biosynthetic process	BP	0.467608724	-0.301366132	-0.260466132	7.337430779	2.897512578	0.407698127	0.815522417 PREDICTED: dihydropyrimidine dehydrogenase [NADP(+)]
RS005332	GO:0046394	carboxylic acid biosynthetic process	BP	0.252504321	-0.254767833	0.253581312	5.674282755	2.884779986	0.409733095	0.818012746 PREDICTED: proline dehydrogenase 1, mitochondrial
RS006022	GO:0046394	carboxylic acid biosynthetic process	BP	-0.558832073	0.180125688	-0.312287278	3.868357371	2.817386095	0.420645848	0.827686496 PREDICTED: beta-ureidopropionase
RS002807	GO:0046394	carboxylic acid biosynthetic process	BP	0.314604033	-0.27626321	0.073564947	4.559683139	2.773114399	0.42794491	0.834208655 PREDICTED: bifunctional coenzyme A synthase
RS008955	GO:0046394	carboxylic acid biosynthetic process	BP	0.09488584	0.430414285	-0.039546417	5.805257816	2.750823103	0.431659417	0.838415407 PREDICTED: phosphopantothenate--cysteine ligase
RS009061	GO:0046394	carboxylic acid biosynthetic process	BP	0.094799519	-0.293006841	0.438370152	4.816522105	2.727064709	0.435647507	0.842238818 PREDICTED: elongation of very long chain fatty acids protein AAEL008004
RS012946	GO:0046394	carboxylic acid biosynthetic process	BP	0.016653937	-0.311667272	-0.341290684	4.595276815	2.59124215	0.45902698	0.858969108 PREDICTED: dephospho-CoA kinase domain-containing protein
RS005230	GO:0046394	carboxylic acid biosynthetic process	BP	0.315236538	-0.240475439	-0.124610322	6.721541958	2.533750866	0.469222413	0.866586765 PREDICTED: probable pyruvate dehydrogenase E1 component subunit alpha, mitochondrial
RS002090	GO:0046394	carboxylic acid biosynthetic process	BP	-0.035193593	0.360328133	-0.131582113	4.860304023	2.500878624	0.475132317	0.871772722 PREDICTED: mitochondrial ribosome-associated GTPase 2
RS002642	GO:0046394	carboxylic acid biosynthetic process	BP	0.327968475	-0.152878617	-0.267497897	5.475828266	2.41164637	0.491470337	0.881592142 PREDICTED: acetyl-CoA acetyltransferase, cytosolic
RS002932	GO:0046394	carboxylic acid biosynthetic process	BP	0.285330244	-0.183360297	-0.196925118	7.264005384	2.38943308	0.495604683	0.88489721 PREDICTED: aldehyde dehydrogenase, mitochondrial
RS009283	GO:0046394	carboxylic acid biosynthetic process	BP	-0.253801091	-0.561371746	-0.432226856	3.757124054	2.376080437	0.498102773	0.885168836 PREDICTED: dephospho-CoA kinase domain-containing protein
RS003701	GO:0046394	carboxylic acid biosynthetic process	BP	0.050229794	0.499103503	0.080945241	6.654471113	2.36698837	0.499809308	0.885993958 PREDICTED: probable deoxyhypusine synthase
RS008466	GO:0046394	carboxylic acid biosynthetic process	BP	-0.036817151	0.287541151	-0.132779006	6.018678883	2.3009665	0.512335776	0.893493528 PREDICTED: protein deglycase DJ-1-like
RS005671	GO:0046394	carboxylic acid biosynthetic process	BP	-0.480675607	-0.708308755	-0.470560703	3.09032693	2.292163205	0.514023891	0.894026754 PREDICTED: branched-chain-amino-acid aminotransferase, cytosolic
RS009359	GO:0046394	carboxylic acid biosynthetic process	BP	0.431271915	0.013846908	0.123224034	4.779215464	2.127519011	0.546365573	0.911798733 PREDICTED: probable malonyl-CoA-acyl carrier protein transacylase, mitochondrial
RS012759	GO:0046394	carboxylic acid biosynthetic process	BP	0.437886801	0.165851875	0.124048717	5.098367147	2.12034101	0.547808612	0.911989536 PREDICTED: probable methylthioribulose-1-phosphate dehydratase
RS010234	GO:0046394	carboxylic acid biosynthetic process	BP	0.397862328	0.082893355	0.188460041	6.583182307	2.068323395	0.55834769	0.917857428 PREDICTED: pyruvate dehydrogenase E1 component subunit beta, mitochondrial
RS011797	GO:0046394	carboxylic acid biosynthetic process	BP	0.384815831	0.110590536	0.031157322	6.971559534	2.046333709	0.562845874	0.920617663 PREDICTED: tyrosine--tRNA ligase, cytoplasmic
RS010471	GO:0046394	carboxylic acid biosynthetic process	BP	0.265576454	0.4004117	0.242777713	6.717703663	2.034793268	0.565216727	0.921627726 PREDICTED: valine--tRNA ligase
RS008554	GO:0046394	carboxylic acid biosynthetic process	BP	0.26061457	-0.150564831	0.073116667	6.805535643	2.011998083	0.569920196	0.923921867 PREDICTED: probable phenylalanine--tRNA ligase beta subunit
RS000973	GO:0046394	carboxylic acid biosynthetic process	BP	0.263732587	0.354708067	-0.040869662	4.212093581	2.001565175	0.57208191	0.925266014 PREDICTED: 6-pyruvoyl tetrahydrobiopterin synthase
RS004350	GO:0046394	carboxylic acid biosynthetic process	BP	0.021061662	-0.16406786	-0.333571588	6.82816194	1.740909041	0.627877403	0.953618312 PREDICTED: folylpolyglutamate synthase, mitochondrial
RS002269	GO:0046394	carboxylic acid biosynthetic process	BP	-0.115899934	-0.045489339	-0.35700385	6.156428651	1.746693481	0.626603143	0.953618312 PREDICTED: succinate-semialdehyde dehydrogenase, mitochondrial
RS000318	GO:0046394	carboxylic acid biosynthetic process	BP	-0.088415405	0.316315043	-0.022734089	7.458187859	1.720429115	0.632401528	0.95623074 PREDICTED: alpha-aminoadipic semialdehyde dehydrogenase
RS012407	GO:0046394	carboxylic acid biosynthetic process	BP	-0.056883468	0.282034432	0.214431303	6.135655265	1.676615982	0.64214505	0.961518328 PREDICTED: probable phenylalanine--tRNA ligase, mitochondrial
RS002341	GO:0046394	carboxylic acid biosynthetic process	BP	0.188480527	0.412959004	0.124638329	7.534002694	1.650331217	0.648032154	0.964512973 PREDICTED: leucine--tRNA ligase, cytoplasmic
RS000435	GO:0046394	carboxylic acid biosynthetic process	BP	0.097794964	0.272631441	-0.105500604	4.943259538	1.627478009	0.653175539	0.966947133 PREDICTED: 39S ribosomal protein L13, mitochondrial
RS004258	GO:0046394	carboxylic acid biosynthetic process	BP	0.076139199	-0.213937947	-0.355275678	5.510581231	1.539067955	0.67328326	0.976156591 PREDICTED: methylthioribose-1-phosphate isomerase
RS005932	GO:0046394	carboxylic acid biosynthetic process	BP	0.312931303	0.436376779	0.655332807	1.360297128	1.523170661	0.676932972	0.976280926 sex peptide receptor
RS002759	GO:0046394	carboxylic acid biosynthetic process	BP	-0.185668843	0.215710017	0.144115499	4.697523831	1.413098998	0.702467146	0.985874136 PREDICTED: bifunctional methylenetetrahydrofolate dehydrogenase/cyclohydrolase, mitochondrial
RS006755	GO:0046394	carboxylic acid biosynthetic process	BP	0.02517928	0.327701814	0.097792094	5.301143253	1.404785963	0.704413259	0.986819333 PREDICTED: Parkinson disease 7 domain-containing protein 1
RS010651	GO:0046394	carboxylic acid biosynthetic process	BP	-0.516006269	-0.442663257	-0.581308717	1.16069732	1.312541931	0.726157047	0.994807952 PREDICTED: L-aminoadipate-semialdehyde dehydrogenase-phosphopantetheinyl transferase
RS005010	GO:0046394	carboxylic acid biosynthetic process	BP	-0.276298645	-0.291724048	-0.400047835	3.211566343	1.199006	0.753242724	0.997734248 PREDICTED: asparagine synthetase domain-containing protein 1
RS003577	GO:0046394	carboxylic acid biosynthetic process	BP	0.046547345	-0.14594425	0.038124755	5.480941987	0.544458042	0.90902107	0.998581907 PREDICTED: probable phenylalanine--tRNA ligase alpha subunit
RS010099	GO:0046394	carboxylic acid biosynthetic process	BP	0.100814249	0.052396139	-0.166198377	8.020158747	0.756965839	0.859730062	0.998581907 PREDICTED: protein transport protein Sec24A
RS012839	GO:0046394	carboxylic acid biosynthetic process	BP	0.292242717	0.226216567	0.09849742	4.671305028	0.96001386	0.810926117	0.998581907 PREDICTED: tyrosine--tRNA ligase, mitochondrial
RS013008	GO:0046394	carboxylic acid biosynthetic process	BP	0.250876024	0.120317607	0.240583891	4.769299517	0.556791171	0.906248768	0.998581907 PREDICTED: probable phenylalanine--tRNA ligase alpha subunit
RS001213	GO:0046394	carboxylic acid biosynthetic process	BP	-0.319817665	-0.271724632	-0.064878391	6.781700265	0.965949527	0.809490365	0.998581907 PREDICTED: probable leucine--tRNA ligase, mitochondrial
RS002959	GO:0046394	carboxylic acid biosynthetic process	BP	-0.03933261	-0.133607381	0.003883204	3.797582146	0.2051226	0.976758129	0.998581907 PREDICTED: 2-oxoisovalerate dehydrogenase subunit beta, mitochondrial
RS006565	GO:0046394	carboxylic acid biosynthetic process	BP	-0.201859081	0.012056294	0.116666653	5.874627529	1.096921685	0.777817253	0.998581907 PREDICTED: isoleucine--tRNA ligase, mitochondrial
RS007611	GO:0046394	carboxylic acid biosynthetic process	BP	0.261975759	0.051822498	0.008480477	5.96011995	0.853024059	0.836746769	0.998581907 Cytochrome b-c1 complex subunit 2, mitochondrial
RS010829	GO:0046394	carboxylic acid biosynthetic process	BP	0.079194703	-0.196131284	0.13745607	5.854681831	0.889043398	0.828071052	0.998581907 PREDICTED: isoleucine--tRNA ligase, cytoplasmic
RS013385	GO:0046394	carboxylic acid biosynthetic process	BP	-0.033304702	0.01202318	-0.211466025	6.570590345	0.700461348	0.87309543	0.998581907 PREDICTED: translation initiation factor eIF-2B subunit beta
RS002397	GO:0046394	carboxylic acid biosynthetic process	BP	0.424459441	0.210287477	0.214472503	5.318744702	1.153194401	0.764250694	0.998581907 PREDICTED: phosphoserine phosphatase
RS009535	GO:0046394	carboxylic acid biosynthetic process	BP	-0.056065203	0.092504311	-0.098751469	6.062674479	0.266403412	0.966218072	0.998581907 PREDICTED: delta-1-pyrroline-5-carboxylate dehydrogenase, mitochondrial
RS010395	GO:0046394	carboxylic acid biosynthetic process	BP	-0.341120196	-0.109016823	-0.088861773	2.332771442	0.616668735	0.892606069	0.998581907 PREDICTED: beta-1-syntrophin
RS001719	GO:0046394	carboxylic acid biosynthetic process	BP	0.097459661	-0.142723959	-0.19721835	8.066839194	0.957896501	0.811438234	0.998581907 PREDICTED: trifunctional enzyme subunit beta, mitochondrial
RS003278	GO:0046394	carboxylic acid biosynthetic process	BP	-0.310870402	-0.028931291	-0.174379076	4.536612129	1.015091246	0.797600386	0.998581907 PREDICTED: malonyl-CoA decarboxylase, mitochondrial-like
RS006548	GO:0046394	carboxylic acid biosynthetic process	BP	0.182887474	0.228802524	0.101581	7.103315126	0.61752435	0.892409116	0.998581907 PREDICTED: acyl carrier protein, mitochondrial
RS011502	GO:0046394	carboxylic acid biosynthetic process	BP	0.178723895	0.074408536	-0.154428329	7.266745855	1.03873978	0.791879214	0.998581907 PREDICTED: 3-hydroxyacyl-CoA dehydrogenase type-2
RS013199	GO:0046394	carboxylic acid biosynthetic process	BP	0.203269509	-0.047918732	0.0993171	3.772217525	0.467665815	0.925935449	0.998581907 PREDICTED: fatty acid hydroxylase domain-containing protein 2
RS002778	GO:0046394	carboxylic acid biosynthetic process	BP	0.217731258	0.316763309	0.035803473	4.91794969	0.930889778	0.817967714	0.998581907 PREDICTED: N-alpha-acetyltransferase 40
RS003723	GO:0046394	carboxylic acid biosynthetic process	BP	-0.120315549	0.28197622	0.125986987	0.826875698	0.275982045	0.964480449	0.998581907 PREDICTED: growth arrest-specific protein 8
RS009901	GO:0046394	carboxylic acid biosynthetic process	BP	-0.140951982	-0.133601127	0.017831827	4.766869113	0.357680713	0.948838986	0.998581907 PREDICTED: dephospho-CoA kinase domain-containing protein
RS013452	GO:0046394	carboxylic acid biosynthetic process	BP	0.000662677	-0.194572268	-0.259470401	2.666680125	0.558379763	0.905890672	0.998581907 PREDICTED: putative lipoyltransferase 2, mitochondrial
RS012825	GO:0046394	carboxylic acid biosynthetic process	BP	-0.004348558	-0.066993864	0.038526307	5.093195487	0.100359128	0.991794292	0.999283281 PREDICTED: lipoyl synthase, mitochondrial
RS010208	GO:0016053	organic acid biosynthetic process	BP	2.631335902	7.346050423	-0.754450343	6.457203767	336.2297923	1.42946E-72	3.48681E-70 PREDICTED: acyl-CoA Delta(11) desaturase-like
RS005642	GO:0016053	organic acid biosynthetic process	BP	-0.634739278	5.138380698	0.16781642	8.170113934	236.0930111	6.6583E-51	6.01948E-49 PREDICTED: elongation of very long chain fatty acids protein 4-like
RS006873	GO:0016053	organic acid biosynthetic process	BP	5.089410523	5.334189165	-2.08043589	4.84259895	170.0556289	1.23782E-36	6.75211E-35 PREDICTED: acyl-CoA Delta(11) desaturase-like
RS006872	GO:0016053	organic acid biosynthetic process	BP	-0.33466745	7.238803945	0.909787823	2.73390939	153.642158	4.31543E-33	2.18784E-31 PREDICTED: acyl-CoA desaturase-like
RS006279	GO:0016053	organic acid biosynthetic process	BP	-0.750412564	2.952200618	2.15034714	5.909933988	146.8352109	1.26889E-31	6.12095E-30 PREDICTED: fatty acid synthase-like
RS001262	GO:0016053	organic acid biosynthetic process	BP	0.334308699	4.247574702	0.34765291	5.028809362	130.6997196	3.8219E-28	1.60421E-26 PREDICTED: elongation of very long chain fatty acids protein AAEL008004
RS001805	GO:0016053	organic acid biosynthetic process	BP	-0.870588518	2.833390077	1.14899972	4.47590462	111.9265088	4.2238E-24	1.50428E-22 PREDICTED: elongation of very long chain fatty acids protein AAEL008004-like
RS000681	GO:0016053	organic acid biosynthetic process	BP	-1.191628428	3.200519867	1.555766858	5.258400593	106.9126587	5.06608E-23	1.69236E-21 PREDICTED: elongation of very long chain fatty acids protein AAEL008004-like
RS013198	GO:0016053	organic acid biosynthetic process	BP	1.398067761	-1.345458381	-0.627931524	6.033691931	101.6972579	6.70695E-22	2.08934E-20 PREDICTED: fatty acid hydroxylase domain-containing protein 2
RS013686	GO:0016053	organic acid biosynthetic process	BP	-0.717818102	-3.908909796	-1.614926839	7.193517866	94.76250858	2.0766E-20	5.96585E-19 PREDICTED: acyl-CoA Delta(11) desaturase-like
RS002678	GO:0016053	organic acid biosynthetic process	BP	1.88850699	2.464921217	-0.538157218	6.037232379	81.46777569	1.4865E-17	3.65352E-16 PREDICTED: fatty acid synthase-like
RS006876	GO:0016053	organic acid biosynthetic process	BP	-1.10740967	-4.875531905	-1.927422906	9.200285447	80.41642171	2.49869E-17	6.0606E-16 PREDICTED: acyl-CoA Delta(11) desaturase-like
RS010135	GO:0016053	organic acid biosynthetic process	BP	1.840019098	-2.32625182	0.732787728	7.759250511	67.76710296	1.28317E-14	2.58392E-13 PREDICTED: protein henna
RS007624	GO:0016053	organic acid biosynthetic process	BP	-1.038705777	-2.417981438	1.4929059	2.541304599	63.78671286	9.11699E-14	1.74098E-12 PREDICTED: tyrosine aminotransferase
RS011394	GO:0016053	organic acid biosynthetic process	BP	-0.808232837	-2.973798175	-1.001114062	5.701841087	63.34834224	1.13133E-13	2.15086E-12 PREDICTED: hydroxyacyl-coenzyme A dehydrogenase, mitochondrial-like
RS013687	GO:0016053	organic acid biosynthetic process	BP	1.493196769	-2.49034267	0.605103464	4.035957267	63.33591434	1.13828E-13	2.16089E-12 PREDICTED: acyl-CoA Delta(11) desaturase-like
RS001358	GO:0016053	organic acid biosynthetic process	BP	1.04922649	-6.683100048	-1.661386259	7.235051984	48.39535779	1.75442E-10	2.64163E-09 putative C-5 sterol desaturase
RS006871	GO:0016053	organic acid biosynthetic process	BP	-0.242054109	1.84490496	-1.948230684	5.642008932	46.08729724	5.4342E-10	7.86711E-09 PREDICTED: acyl-CoA Delta(11) desaturase-like
RS011853	GO:0016053	organic acid biosynthetic process	BP	-0.198391061	-2.402376009	-1.315737207	5.883199768	42.14749002	3.73315E-09	5.06955E-08 PREDICTED: aldehyde dehydrogenase, dimeric NADP-preferring
RS001544	GO:0016053	organic acid biosynthetic process	BP	0.274963904	-1.09360695	-1.805361323	6.749172802	41.74350775	4.54779E-09	6.11798E-08 PREDICTED: alkaline phosphatase 4-like
RS013244	GO:0016053	organic acid biosynthetic process	BP	-2.118886964	-5.134511847	-2.304467706	2.520197622	40.7543233	7.37271E-09	9.7359E-08 PREDICTED: acyl-CoA Delta(11) desaturase-like
RS002190	GO:0016053	organic acid biosynthetic process	BP	-1.374803006	-2.081968712	0.437978506	4.918669629	35.63124542	8.96069E-08	1.03896E-06 PREDICTED: fatty acid 2-hydroxylase
RS009089	GO:0016053	organic acid biosynthetic process	BP	0.780156718	-1.142779879	-0.944341962	6.520545706	35.56337079	9.26164E-08	1.07193E-06 PREDICTED: probable phosphoserine aminotransferase
RS010978	GO:0016053	organic acid biosynthetic process	BP	-0.105950757	-2.718350483	-0.164657054	9.167446834	32.70854985	3.71036E-07	3.99729E-06 PREDICTED: fatty acid synthase
RS005544	GO:0016053	organic acid biosynthetic process	BP	0.405463151	1.50203441	-1.817605524	9.291358907	27.83750659	3.92874E-06	3.76786E-05 PREDICTED: beta-ureidopropionase
RS002535	GO:0016053	organic acid biosynthetic process	BP	-0.730643229	-1.637072136	0.172680139	8.113462827	26.98303681	5.93576E-06	5.54863E-05 PREDICTED: acetyl-CoA carboxylase
RS001261	GO:0016053	organic acid biosynthetic process	BP	-2.235645034	0.224656739	-1.086038105	2.162252071	26.93058301	6.08797E-06	5.68269E-05 PREDICTED: elongation of very long chain fatty acids protein 7
RS003917	GO:0016053	organic acid biosynthetic process	BP	1.262100569	0.159623921	-0.096731621	5.707277733	25.44668354	1.24519E-05	0.000111558 PREDICTED: asparagine synthetase [glutamine-hydrolyzing]

RS005087	GO:0016053	organic acid biosynthetic process	BP	-0.35126078	1.741640675	2.124824608	1.745993058	24.29506603	2.16759E-05	0.000187316	PREDICTED: GTP cyclohydrolase 1
RS002240	GO:0016053	organic acid biosynthetic process	BP	-0.632574541	0.882911763	0.780104643	5.192425356	23.60200145	3.02453E-05	0.000256065	PREDICTED: probable pyruvate dehydrogenase E1 component subunit alpha, mitochondrial
RS003648	GO:0016053	organic acid biosynthetic process	BP	1.268807283	1.776217158	0.41179627	5.528161964	23.03387344	3.97318E-05	0.000329264	PREDICTED: protein singed
RS007468	GO:0016053	organic acid biosynthetic process	BP	1.554961358	-0.015902902	0.482470573	9.656522243	22.44534397	5.26932E-05	0.0004279	PREDICTED: retinal dehydrogenase 1
RS004115	GO:0016053	organic acid biosynthetic process	BP	-3.338103077	-2.132121097	-3.540885575	2.658495116	22.39384183	5.40105E-05	0.000438323	PREDICTED: pyrroline-5-carboxylate reductase 2
RS006877	GO:0016053	organic acid biosynthetic process	BP	0.265818642	-1.05943473	-2.08149481	3.30161852	22.30893928	5.62543E-05	0.000455388	PREDICTED: acyl-CoA Delta(11) desaturase
RS000146	GO:0016053	organic acid biosynthetic process	BP	0.439480055	-1.070680667	-0.239174518	6.074478122	21.95270329	6.67261E-05	0.000533478	PREDICTED: serine hydroxymethyltransferase, mitochondrial
RS010210	GO:0016053	organic acid biosynthetic process	BP	-1.577649228	0.880954578	-0.51929089	3.453369802	21.20123124	9.56162E-05	0.000742418	PREDICTED: acyl-CoA Delta(11) desaturase-like
RS011319	GO:0016053	organic acid biosynthetic process	BP	-1.721607065	-0.37953699	0.32859135	5.346304498	21.05802428	0.000102395	0.000789835	PREDICTED: elongation of very long chain fatty acids protein AAEL008004-like
RS012880	GO:0016053	organic acid biosynthetic process	BP	-0.807344323	-1.895186526	-0.086915908	8.413207767	20.19903013	0.00015436	0.001160887	PREDICTED: C-1-tetrahydrofolate synthase, cytoplasmic
RS013389	GO:0016053	organic acid biosynthetic process	BP	-0.662472987	-1.005914654	0.744834151	7.265622757	19.41054869	0.000224837	0.001636653	PREDICTED: propionyl-CoA carboxylase alpha chain, mitochondrial
RS013246	GO:0016053	organic acid biosynthetic process	BP	0.026801121	-2.623174489	-1.205156148	1.300160489	18.64961266	0.000323008	0.002299477	PREDICTED: acyl-CoA Delta(11) desaturase-like
RS008510	GO:0016053	organic acid biosynthetic process	BP	-0.007861455	1.657826497	1.802497318	2.194239545	18.48937541	0.000348586	0.002469326	PREDICTED: GTP cyclohydrolase 1
RS000267	GO:0016053	organic acid biosynthetic process	BP	-0.550819695	-1.165273853	0.191627701	5.343147996	18.17657843	0.000404462	0.002829484	PREDICTED: argininosuccinate lyase
RS010737	GO:0016053	organic acid biosynthetic process	BP	0.07086237	1.016941323	1.268374972	5.272102935	16.67839044	0.000822948	0.005444763	PREDICTED: elongation of very long chain fatty acids protein AAEL008004
RS013655	GO:0016053	organic acid biosynthetic process	BP	-0.256056345	1.603293117	0.591268857	9.642915994	16.658626	0.000830678	0.0054931	PREDICTED: transforming growth factor-beta-induced protein ig-h3-like
RS015505	GO:0016053	organic acid biosynthetic process	BP	-0.333963264	-1.254068775	-0.333900304	7.203178713	16.32816273	0.000971162	0.006353837	PREDICTED: probable methylmalonate-semialdehyde dehydrogenase [acylating], mitochondrial
RS008573	GO:0016053	organic acid biosynthetic process	BP	-0.877847198	-1.285642597	-0.317627515	5.276427543	15.89720024	0.001190362	0.007667666	PREDICTED: probable aspartate aminotransferase, cytoplasmic
RS007469	GO:0016053	organic acid biosynthetic process	BP	0.109921824	-1.512321655	-1.260766781	10.37063405	14.46509492	0.002335814	0.014022266	PREDICTED: retinal dehydrogenase 1
RS013245	GO:0016053	organic acid biosynthetic process	BP	0.205086194	-2.137451995	-0.303300922	1.873173886	13.92971456	0.003002401	0.017603194	PREDICTED: acyl-CoA Delta(11) desaturase-like
RS013640	GO:0016053	organic acid biosynthetic process	BP	-0.507605075	0.88378812	-0.370589145	3.007371753	13.90545704	0.003036708	0.017788199	PREDICTED: alkaline phosphatase-like
RS010105	GO:0016053	organic acid biosynthetic process	BP	0.005457692	-0.665472681	-1.12936068	7.211526424	13.47669162	0.003711352	0.021249053	PREDICTED: elongation of very long chain fatty acids protein 6
RS015477	GO:0016053	organic acid biosynthetic process	BP	-0.897264721	-1.291332363	-0.434228581	5.338765823	13.37548035	0.003891121	0.022209456	PREDICTED: pyruvate carboxylase, mitochondrial
RS011903	GO:0016053	organic acid biosynthetic process	BP	0.785032756	-0.185002642	0.61952285	5.789786927	13.31924568	0.003994703	0.022750447	PREDICTED: CAD protein
RS007097	GO:0016053	organic acid biosynthetic process	BP	0.795617782	-0.143458305	-0.302194899	4.974094685	13.15347812	0.004316187	0.024302988	PREDICTED: methylcrotonoyl-CoA carboxylase subunit alpha, mitochondrial
RS010159	GO:0016053	organic acid biosynthetic process	BP	-0.646502633	0.639073484	-0.488386035	5.47525374	12.90472858	0.004847231	0.02695269	PREDICTED: delta-1-pyrroline-5-carboxylate synthase
RS001674	GO:0016053	organic acid biosynthetic process	BP	-0.383074682	-0.838511587	0.254025522	6.390380362	12.56965972	0.005665996	0.031011855	PREDICTED: cystathionine beta-synthase
RS006613	GO:0016053	organic acid biosynthetic process	BP	0.452094938	-0.623868558	0.040645578	5.891109809	12.3268201	0.006343489	0.034284545	PREDICTED: enolase-phosphatase E1
RS005142	GO:0016053	organic acid biosynthetic process	BP	-0.386591935	-1.296698312	-1.484278386	1.705974468	10.73484454	0.013249577	0.065055273	PREDICTED: methylenetetrahydrofolate reductase
RS007239	GO:0016053	organic acid biosynthetic process	BP	-0.80039341	-0.711870169	-2.286672983	9.552046577	10.33732368	0.015906021	0.075795442	PREDICTED: D-3-phosphoglycerate dehydrogenase
RS002083	GO:0016053	organic acid biosynthetic process	BP	0.177250401	-0.761079147	-0.577437436	8.133614126	10.062607	0.018041381	0.084660246	PREDICTED: aspartate aminotransferase, mitochondrial
RS011651	GO:0016053	organic acid biosynthetic process	BP	0.27609398	-1.005784534	0.026713349	8.399697414	9.976153219	0.018769933	0.087538852	PREDICTED: fatty acid synthase-like
RS008575	GO:0016053	organic acid biosynthetic process	BP	0.285937384	-0.731842967	-0.265423735	6.102447533	9.775911583	0.020570235	0.094235294	PREDICTED: probable aspartate aminotransferase, cytoplasmic
RS003105	GO:0016053	organic acid biosynthetic process	BP	0.093511576	0.720219714	0.022101776	5.289399073	8.772772376	0.032469645	0.138903896	PREDICTED: alkylglycerol monooxygenase-like
RS010012	GO:0016053	organic acid biosynthetic process	BP	0.014321587	0.739129238	0.71793314	5.662732627	8.6765526	0.033915232	0.143944884	PREDICTED: probable low-specificity L-threonine aldolase 2
RS010957	GO:0016053	organic acid biosynthetic process	BP	-0.597747337	-0.61322871	-0.993570939	7.669505911	8.638947236	0.034497148	0.146126846	PREDICTED: ornithine aminotransferase, mitochondrial
RS015101	GO:0016053	organic acid biosynthetic process	BP	0.185501806	-0.365075161	-0.63038581	6.561629341	8.335808276	0.039558189	0.163703031	PREDICTED: 2-aminoethylphosphonate--pyruvate transaminase-like
RS005894	GO:0016053	organic acid biosynthetic process	BP	-0.450956232	-0.957793122	-0.804806328	8.60102836	8.306166461	0.040090329	0.16548141	PREDICTED: pantothenate kinase 3
RS007596	GO:0016053	organic acid biosynthetic process	BP	-0.809534745	-0.666982264	0.578146648	4.874681874	7.980578839	0.046414792	0.186004475	PREDICTED: fatty acid synthase-like
RS007471	GO:0016053	organic acid biosynthetic process	BP	0.488223208	0.356479238	-0.43697813	8.831136905	7.56070493	0.056019028	0.215925459	PREDICTED: lambda-crystallin homolog
RS010209	GO:0016053	organic acid biosynthetic process	BP	-0.767235758	0.184554649	-0.438670204	6.018691533	7.178862894	0.066410121	0.247135878	PREDICTED: acyl-CoA Delta(11) desaturase
RS011854	GO:0016053	organic acid biosynthetic process	BP	-0.744270551	0.041529434	-0.702768939	7.795747502	7.164883594	0.066823987	0.248104681	PREDICTED: aldehyde dehydrogenase, dimeric NADP-preferring
RS007595	GO:0016053	organic acid biosynthetic process	BP	-1.222953796	-0.412402431	-0.073293638	6.073896472	6.930709003	0.074139413	0.269207268	PREDICTED: fatty acid synthase-like
RS006400	GO:0016053	organic acid biosynthetic process	BP	0.791751698	-0.199311153	0.789475208	3.864467767	6.803637183	0.07842698	0.281327411	PREDICTED: leucine-rich repeat-containing protein 47-like
RS001514	GO:0016053	organic acid biosynthetic process	BP	0.259150932	-0.508483967	-0.206376495	6.407252152	6.444096885	0.091893449	0.316041105	PREDICTED: pantothenate kinase 4
RS006504	GO:0016053	organic acid biosynthetic process	BP	0.429662448	0.076826048	-0.459250569	4.576152449	5.755047279	0.124155283	0.394562316	PREDICTED: 5-formyltetrahydrofolate cyclo-ligase
RS012801	GO:0016053	organic acid biosynthetic process	BP	-0.305373229	-0.483960269	-0.704209767	4.980361046	5.699688733	0.127171057	0.401776007	PREDICTED: trimethyllysine dioxygenase, mitochondrial
RS014856	GO:0016053	organic acid biosynthetic process	BP	0.143481668	-0.166733568	0.485678581	6.941672661	5.624645258	0.131370639	0.410823283	PREDICTED: lysine--tRNA ligase
RS003393	GO:0016053	organic acid biosynthetic process	BP	0.293569646	-0.648014171	-0.531549171	8.391513924	5.38529422	0.145662549	0.443610233	PREDICTED: leukotriene A-4 hydrolase
RS000178	GO:0016053	organic acid biosynthetic process	BP	0.187222857	-0.580152531	-0.650904567	5.507795536	5.118447643	0.163326473	0.479848848	PREDICTED: argininosuccinate synthase
RS012653	GO:0016053	organic acid biosynthetic process	BP	0.457094882	-0.098825212	-0.22264278	4.112007138	5.014152731	0.17076374	0.494652394	PREDICTED: 3-oxoacyl-[acyl-carrier-protein] synthase, mitochondrial
RS011781	GO:0016053	organic acid biosynthetic process	BP	0.194868998	-0.290152413	-0.647996521	9.541772745	5.013408504	0.170817937	0.49469854	PREDICTED: enolase
RS014553	GO:0016053	organic acid biosynthetic process	BP	-0.211371951	-1.732733164	0.093753139	0.949304848	4.739747226	0.191876615	0.533343557	PREDICTED: fatty acid synthase-like
RS014512	GO:0016053	organic acid biosynthetic process	BP	-0.141977713	-0.560717408	-0.429830427	6.661293348	4.739753613	0.191876097	0.533343557	PREDICTED: cysteine desulfurase, mitochondrial
RS007594	GO:0016053	organic acid biosynthetic process	BP	-0.717242258	-0.207577159	0.27013743	8.519802645	4.633705425	0.200669656	0.549838123	PREDICTED: fatty acid synthase
RS007875	GO:0016053	organic acid biosynthetic process	BP	0.142712287	-0.392317307	-0.251606633	6.099847719	4.332732172	0.227704312	0.595300577	PREDICTED: splicing factor 3B subunit 6-like protein
RS012259	GO:0016053	organic acid biosynthetic process	BP	-0.651022963	-0.413329834	-0.034985405	8.655623119	4.197617943	0.240900483	0.618788285	PREDICTED: putative glutamate synthase [NADPH]
RS013197	GO:0016053	organic acid biosynthetic process	BP	-0.368534608	-0.921242977	-0.538013398	7.366413675	4.1646868	0.244221273	0.623342031	PREDICTED: fatty acid hydroxylase domain-containing protein 2
RS013397	GO:0016053	organic acid biosynthetic process	BP	0.276762068	-0.133208457	-0.437714221	7.582877367	4.164631585	0.244226876	0.623342031	PREDICTED: delta(3,5)-Delta(2,4)-dienoyl-CoA isomerase, mitochondrial
RS006104	GO:0016053	organic acid biosynthetic process	BP	0.050741232	0.067982723	-0.679870588	9.220750703	3.978415949	0.26380427	0.654473536	PREDICTED: glutamine synthetase 2 cytoplasmic
RS001242	GO:0016053	organic acid biosynthetic process	BP	-0.066255895	-0.531998073	-0.515824902	6.37443736	3.732497257	0.291836068	0.68973614	PREDICTED: 1,2-dihydroxy-3-keto-5-methylthiopentene dioxygenase
RS009609	GO:0016053	organic acid biosynthetic process	BP	0.210078244	-0.06842499	-0.397693417	4.048414359	3.700886628	0.295627068	0.69337205	PREDICTED: S-methyl-5'-thioadenosine phosphorylase
RS001808	GO:0016053	organic acid biosynthetic process	BP	-0.058607321	-0.547033169	0.506368301	2.661379544	3.681395157	0.297986539	0.696883137	PREDICTED: elongation of very long chain fatty acids protein AAEL008004-like
RS013812	GO:0016053	organic acid biosynthetic process	BP	-0.236673306	-0.669275541	-0.156642409	5.411761702	3.625031862	0.304904278	0.707049777	PREDICTED: propionyl-CoA carboxylase beta chain, mitochondrial
RS014514	GO:0016053	organic acid biosynthetic process	BP	0.454293395	0.189819706	-0.159566019	8.374000108	3.535332448	0.316208451	0.719511677	PREDICTED: trifunctional enzyme subunit alpha, mitochondrial
RS000797	GO:0016053	organic acid biosynthetic process	BP	-0.378894012	-0.143187194	0.419208563	4.992333982	3.492017406	0.321798913	0.726929294	stripe
RS013622	GO:0016053	organic acid biosynthetic process	BP	1.304002696	-0.790491216	0.213979312	6.381917919	3.432062346	0.329680888	0.734589515	PREDICTED: membrane-bound alkaline phosphatase-like
RS010695	GO:0016053	organic acid biosynthetic process	BP	0.167669466	-0.139565623	-0.427342962	4.043742135	3.369399197	0.338099779	0.74419257	PREDICTED: dihydrofolate reductase
RS008584	GO:0016053	organic acid biosynthetic process	BP	0.292833023	-0.220059953	-0.331841648	2.908313798	3.364840859	0.338719484	0.745047978	PREDICTED: thymidylate synthase
RS009254	GO:0016053	organic acid biosynthetic process	BP	0.39225852	0.2856945	-0.18015587	6.987646529	3.296181412	0.348174475	0.75656684	PREDICTED: histidine triad nucleotide-binding protein 1
RS010718	GO:0016053	organic acid biosynthetic process	BP	0.477981617	0.174790255	-0.000903128	4.437079375	3.242803222	0.355683034	0.764213108	PREDICTED: pyrroline-5-carboxylate reductase 2
RS003843	GO:0016053	organic acid biosynthetic process	BP	0.523865093	0.896702368	0.950902499	1.712239692	3.188197528	0.36350902	0.771665781	Homeobox protein ceh-9
RS013983	GO:0016053	organic acid biosynthetic process	BP	0.163996819	-0.412664616	-0.172942215	4.146360298	3.170825747	0.366029671	0.775234533	PREDICTED: valine--tRNA ligase-like
RS011393	GO:0016053	organic acid biosynthetic process	BP	0.02917404	-0.508183169	0.050743806	7.352702277	3.024628885	0.387843452	0.796886111	PREDICTED: hydroxyacyl-coenzyme A dehydrogenase, mitochondrial-like
RS002533	GO:0016053	organic acid biosynthetic process	BP	0.009324912	-0.457888597	-0.251888724	5.442114645	2.939900614	0.400984439	0.808724935	PREDICTED: sepiapterin reductase-like
RS006771	GO:0016053	organic acid biosynthetic process	BP	0.501095992	0.373222122	0.613510743	5.629228769	2.925902508	0.403191208	0.81102473	PREDICTED: C-terminal-binding protein
RS007071	GO:0016053	organic acid biosynthetic process	BP	0.467608724	-0.301366132	-0.260466132	7.337430779	2.897512578	0.407698127	0.815522417	PREDICTED: dihydropyrimidine dehydrogenase [NADP(+)]
RS005332	GO:0016053	organic acid biosynthetic process	BP	0.252504321	-0.254767833	0.253581312	5.674282755	2.884779986	0.409733095	0.818012746	PREDICTED: proline dehydrogenase 1, mitochondrial
RS006022	GO:0016053	organic acid biosynthetic process	BP	-0.558832073	0.180125688	-0.312287278	3.868357371	2.817386095	0.420645848	0.827686496	PREDICTED: beta-ureidopropionase
RS002807	GO:0016053	organic acid biosynthetic process	BP	0.314604033	-0.27626321	0.073564947	4.559683139	2.773114399	0.42794491	0.834208655	PREDICTED: bifunctional coenzyme A synthase
RS008955	GO:0016053	organic acid biosynthetic process	BP	0.09488584	0.430414285	-0.039546417	5.805257816	2.750823103	0.431659417	0.838415407	PREDICTED: phosphopantothenate--cysteine ligase
RS009061	GO:0016053	organic acid biosynthetic process	BP	0.094799519	-0.293006841	0.438370152	4.816522105	2.727064709	0.435647507	0.842238818	PREDICTED: elongation of very long chain fatty acids protein AAEL008004
RS012946	GO:0016053	organic acid biosynthetic process	BP	0.016653937	-0.311667272	-0.341290684	4.595276815	2.59124215	0.45902698	0.858969108	PREDICTED: dephospho-CoA kinase domain-containing protein
RS005230	GO:0016053	organic acid biosynthetic process	BP	0.315236538	-0.240475439	-0.124610322	6.721541958	2.533750866	0.469222413	0.866586765	PREDICTED: probable pyruvate dehydrogenase E1 component subunit alpha, mitochondrial
RS002090	GO:0016053	organic acid biosynthetic process	BP	-0.035193593	0.360328133	-0.131582113	4.860304023	2.500878624	0.475132317	0.871772722	PREDICTED: mitochondrial ribosome-associated GTPase 2
RS002642	GO:0016053	organic acid biosynthetic process	BP	0.327968475	-0.152878617	-0.267497897	5.475828266	2.41164637	0.491470337	0.881592142	PREDICTED: acetyl-CoA acetyltransferase, cytosolic
RS002932	GO:0016053	organic acid biosynthetic process	BP	0.285330244	-0.183360297	-0.196925118	7.264005384	2.38943308	0.495604683	0.88489721	PREDICTED: aldehyde dehydrogenase, mitochondrial
RS009283	GO:0016053	organic acid biosynthetic process	BP	-0.253801091	-0.561371746	-0.432226856	3.757124054	2.376080437	0.498102773	0.885168836	PREDICTED: dephospho-CoA kinase domain-containing protein
RS003701	GO:0016053	organic acid biosynthetic process	BP	0.050229794	0.499103503	0.080945241	6.654471113	2.36698837	0.499809308	0.885993958	PREDICTED: probable deoxyhypusine synthase
RS008466	GO:0016053	organic acid biosynthetic process	BP	-0.036817151	0.287541151	-0.132779006	6.018678883	2.3009665	0.512335776	0.893493528	PREDICTED: protein deglycase DJ-1-like
RS005671	GO:0016053	organic acid biosynthetic process	BP	-0.480675607	-0.708308755	-0.470560703	3.09032693	2.292163205	0.514023891	0.894026754	PREDICTED: branched-chain-amino-acid aminotransferase, cytosolic
RS009359	GO:0016053	organic acid biosynthetic process	BP	0.431271915	0.013846908	0.123224034	4.779215464	2.127519011	0.546365573	0.911798733	PREDICTED: probable malonyl-CoA-acyl carrier protein transacylase, mitochondrial
RS012759	GO:0016053	organic acid biosynthetic process	BP	0.437886801	0.165851875	0.124048717	5.098367147	2.12034101	0.547808612	0.911989536	PREDICTED: probable methylthioribulose-1-phosphate dehydratase
RS010234	GO:0016053	organic acid biosynthetic process	BP	0.397862328	0.082893355	0.188460041	6.583182307	2.068323395	0.55834769	0.917857428	PREDICTED: pyruvate dehydrogenase E1 component subunit beta, mitochondrial
RS011797	GO:0016053	organic acid biosynthetic process	BP	0.384815831	0.110590536	0.031157322	6.971559534	2.046333709	0.562845874	0.920617663	PREDICTED: tyrosine--tRNA ligase, cytoplasmic
RS010471	GO:0016053	organic acid biosynthetic process	BP	0.265576454	0.4004117	0.242777713	6.717703663	2.034793268	0.565216727	0.921627726	PREDICTED: valine--tRNA ligase
RS008554	GO:0016053	organic acid biosynthetic process	BP	0.26061457	-0.150564831	0.073116667	6.805535643	2.011998083	0.569920196	0.923921867	PREDICTED: probable phenylalanine--tRNA ligase beta subunit
RS000973	GO:0016053	organic acid biosynthetic process	BP	0.263732587	0.354708067	-0.040869662	4.212093581	2.001565175	0.57208191	0.925266014	PREDICTED: 6-pyruvoyl tetrahydrobiopterin synthase
RS004350	GO:0016053	organic acid biosynthetic process	BP	0.021061662	-0.16406786	-0.333571588	6.82816194	1.740909041	0.627877403	0.953618312	PREDICTED: folylpolyglutamate synthase, mitochondrial
RS002269	GO:0016053	organic acid biosynthetic process	BP	-0.115899934	-0.045489339	-0.35700385	6.156428651	1.746693481	0.626603143	0.953618312	PREDICTED: succinate-semialdehyde dehydrogenase, mitochondrial
RS000318	GO:0016053	organic acid biosynthetic process	BP	-0.088415405	0.316315043	-0.022734089	7.458187859	1.720429115	0.632401528	0.95623074	PREDICTED: alpha-aminoadipic semialdehyde dehydrogenase
RS012407	GO:0016053	organic acid biosynthetic process	BP	-0.056883468	0.282034432	0.214431303	6.135655265	1.676615982	0.64214505	0.961518328	PREDICTED: probable phenylalanine--tRNA ligase, mitochondrial
RS002341	GO:0016053	organic acid biosynthetic process	BP	0.188480527	0.412959004	0.124638329	7.534002694	1.650331217	0.648032154	0.964512973	PREDICTED: leucine--tRNA ligase, cytoplasmic
RS000435	GO:0016053	organic acid biosynthetic process	BP	0.097794964	0.272631441	-0.105500604	4.943259538	1.627478009	0.653175539	0.966947133	PREDICTED: 39S ribosomal protein L13, mitochondrial
RS004258	GO:0016053	organic acid biosynthetic process	BP	0.076139199	-0.213937947	-0.355275678	5.510581231	1.539067955	0.67328326	0.976156591	PREDICTED: methylthioribose-1-phosphate isomerase
RS005932	GO:0016053	organic acid biosynthetic process	BP	0.312931303	0.436376779	0.655332807	1.360297128	1.523170661	0.676932972	0.976280926	sex peptide receptor
RS002759	GO:0016053	organic acid biosynthetic process	BP	-0.185668843	0.215710017	0.144115499	4.697523831	1.413098998	0.702467146	0.985874136	PREDICTED: bifunctional methylenetetrahydrofolate dehydrogenase/cyclohydrolase, mitochondrial
RS006755	GO:0016053	organic acid biosynthetic process	BP	0.02517928	0.327701814	0.097792094	5.301143253	1.404785963	0.704413259	0.986819333	PREDICTED: Parkinson disease 7 domain-containing protein 1
RS010651	GO:0016053	organic acid biosynthetic process	BP	-0.516006269	-0.442663257	-0.581308717	1.16069732	1.312541931	0.726157047	0.994807952	PREDICTED: L-aminoadipate-semialdehyde dehydrogenase-phosphopantetheinyl transferase
RS005010	GO:0016053	organic acid biosynthetic process	BP	-0.276298645	-0.291724048	-0.400047835	3.211566343	1.199006	0.753242724	0.997734248	PREDICTED: asparagine synthetase domain-containing protein 1
RS009535	GO:0016053	organic acid biosynthetic process	BP	-0.056065203	0.092504311	-0.098751469	6.062674479	0.266403412	0.966218072	0.998581907	PREDICTED: delta-1-pyrroline-5-carboxylate dehydrogenase, mitochondrial

RS010395	GO:0016053	organic acid biosynthetic process	BP	-0.341120196	-0.109016823	-0.088861773	2.332771442	0.616668735	0.892606069	0.998581907	PREDICTED: beta-1-syntrophin
RS001719	GO:0016053	organic acid biosynthetic process	BP	0.097459661	-0.142723959	-0.19721835	8.066839194	0.957896501	0.811438234	0.998581907	PREDICTED: trifunctional enzyme subunit beta, mitochondrial
RS003278	GO:0016053	organic acid biosynthetic process	BP	-0.310870402	-0.028931291	-0.174379076	4.536612129	1.015091246	0.797600386	0.998581907	PREDICTED: malonyl-CoA decarboxylase, mitochondrial-like
RS006548	GO:0016053	organic acid biosynthetic process	BP	0.182887474	0.228802524	0.101581	7.103315126	0.61752435	0.892409116	0.998581907	PREDICTED: acyl carrier protein, mitochondrial
RS011502	GO:0016053	organic acid biosynthetic process	BP	0.178723895	0.074408536	-0.154428329	7.266745855	1.03873978	0.791879214	0.998581907	PREDICTED: 3-hydroxyacyl-CoA dehydrogenase type-2
RS013199	GO:0016053	organic acid biosynthetic process	BP	0.203269509	-0.047918732	0.0993171	3.772217525	0.467665815	0.925935449	0.998581907	PREDICTED: fatty acid hydroxylase domain-containing protein 2
RS002778	GO:0016053	organic acid biosynthetic process	BP	0.217731258	0.316763309	0.035803473	4.91794969	0.930889778	0.817967714	0.998581907	PREDICTED: N-alpha-acetyltransferase 40
RS003723	GO:0016053	organic acid biosynthetic process	BP	-0.120315549	0.28197622	0.125986987	0.826875698	0.275982045	0.964480449	0.998581907	PREDICTED: growth arrest-specific protein 8
RS009901	GO:0016053	organic acid biosynthetic process	BP	-0.140951982	-0.133601127	0.017831827	4.766869113	0.357680713	0.948838986	0.998581907	PREDICTED: dephospho-CoA kinase domain-containing protein
RS003577	GO:0016053	organic acid biosynthetic process	BP	0.046547345	-0.14594425	0.038124755	5.480941987	0.544458042	0.90902107	0.998581907	PREDICTED: probable phenylalanine--tRNA ligase alpha subunit
RS010099	GO:0016053	organic acid biosynthetic process	BP	0.100814249	0.052396139	-0.166198377	8.020158747	0.756965839	0.859730062	0.998581907	PREDICTED: protein transport protein Sec24A
RS012839	GO:0016053	organic acid biosynthetic process	BP	0.292242717	0.226216567	0.09849742	4.671305028	0.96001386	0.810926117	0.998581907	PREDICTED: tyrosine--tRNA ligase, mitochondrial
RS013008	GO:0016053	organic acid biosynthetic process	BP	0.250876024	0.120317607	0.240583891	4.769299517	0.556791171	0.906248768	0.998581907	PREDICTED: probable phenylalanine--tRNA ligase alpha subunit
RS001213	GO:0016053	organic acid biosynthetic process	BP	-0.319817665	-0.271724632	-0.064878391	6.781700265	0.965949527	0.809490365	0.998581907	PREDICTED: probable leucine--tRNA ligase, mitochondrial
RS002959	GO:0016053	organic acid biosynthetic process	BP	-0.03933261	-0.133607381	0.003883204	3.797582146	0.2051226	0.976758129	0.998581907	PREDICTED: 2-oxoisovalerate dehydrogenase subunit beta, mitochondrial
RS006565	GO:0016053	organic acid biosynthetic process	BP	-0.201859081	0.012056294	0.116666653	5.874627529	1.096921685	0.777817253	0.998581907	PREDICTED: isoleucine--tRNA ligase, mitochondrial
RS007611	GO:0016053	organic acid biosynthetic process	BP	0.261975759	0.051822498	0.008480477	5.96011995	0.853024059	0.836746769	0.998581907	Cytochrome b-c1 complex subunit 2, mitochondrial
RS010829	GO:0016053	organic acid biosynthetic process	BP	0.079194703	-0.196131284	0.13745607	5.854681831	0.889043398	0.828071052	0.998581907	PREDICTED: isoleucine--tRNA ligase, cytoplasmic
RS013452	GO:0016053	organic acid biosynthetic process	BP	0.000662677	-0.194572268	-0.259470401	2.666680125	0.558379763	0.905890672	0.998581907	PREDICTED: putative lipoyltransferase 2, mitochondrial
RS013385	GO:0016053	organic acid biosynthetic process	BP	-0.033304702	0.01202318	-0.211466025	6.570590345	0.700461348	0.87309543	0.998581907	PREDICTED: translation initiation factor eIF-2B subunit beta
RS002397	GO:0016053	organic acid biosynthetic process	BP	0.424459441	0.210287477	0.214472503	5.318744702	1.153194401	0.764250694	0.998581907	PREDICTED: phosphoserine phosphatase
RS012825	GO:0016053	organic acid biosynthetic process	BP	-0.004348558	-0.066993864	0.038526307	5.093195487	0.100359128	0.991794292	0.999283281	PREDICTED: lipoyl synthase, mitochondrial
RS010208	GO:0032787	monocarboxylic acid metabolic process	BP	2.631335902	7.346050423	-0.754450343	6.457203767	336.2297923	1.42946E-72	3.48681E-70	PREDICTED: acyl-CoA Delta(11) desaturase-like
RS005642	GO:0032787	monocarboxylic acid metabolic process	BP	-0.634739278	5.138380698	0.16781642	8.170113934	236.0930111	6.6583E-51	6.01948E-49	PREDICTED: elongation of very long chain fatty acids protein 4-like
RS011942	GO:0032787	monocarboxylic acid metabolic process	BP	-0.29966522	5.562248468	1.292757929	6.487064844	232.701417	3.60349E-50	3.04483E-48	PREDICTED: 2-hydroxyacylsphingosine 1-beta-galactosyltransferase
RS006873	GO:0032787	monocarboxylic acid metabolic process	BP	5.089410523	5.334189165	-2.08043589	4.84259895	170.0556289	1.23782E-36	6.75211E-35	PREDICTED: acyl-CoA Delta(11) desaturase-like
RS006872	GO:0032787	monocarboxylic acid metabolic process	BP	-0.33466745	7.238803945	0.909787823	2.73390939	153.642158	4.31543E-33	2.18784E-31	PREDICTED: acyl-CoA desaturase-like
RS006279	GO:0032787	monocarboxylic acid metabolic process	BP	-0.750412564	2.952200618	2.15034714	5.909933988	146.8352109	1.26889E-31	6.12095E-30	PREDICTED: fatty acid synthase-like
RS001262	GO:0032787	monocarboxylic acid metabolic process	BP	0.334308699	4.247574702	0.34765291	5.028809362	130.6997196	3.8219E-28	1.60421E-26	PREDICTED: elongation of very long chain fatty acids protein AAEL008004
RS001805	GO:0032787	monocarboxylic acid metabolic process	BP	-0.870588518	2.833390077	1.14899972	4.47590462	111.9265088	4.2238E-24	1.50428E-22	PREDICTED: elongation of very long chain fatty acids protein AAEL008004-like
RS000681	GO:0032787	monocarboxylic acid metabolic process	BP	-1.191628428	3.200519867	1.555766858	5.258400593	106.9126587	5.06608E-23	1.69236E-21	PREDICTED: elongation of very long chain fatty acids protein AAEL008004-like
RS013198	GO:0032787	monocarboxylic acid metabolic process	BP	1.398067761	-1.345458381	-0.627931524	6.033691931	101.6972579	6.70695E-22	2.08934E-20	PREDICTED: fatty acid hydroxylase domain-containing protein 2
RS014580	GO:0032787	monocarboxylic acid metabolic process	BP	-2.238409419	-4.554196395	-0.83576737	3.778928597	98.73572107	2.90617E-21	8.73744E-20	PREDICTED: kynurenine formamidase-like
RS013686	GO:0032787	monocarboxylic acid metabolic process	BP	-0.717818102	-3.908909796	-1.614926839	7.193517866	94.76250858	2.0766E-20	5.96585E-19	PREDICTED: acyl-CoA Delta(11) desaturase-like
RS002678	GO:0032787	monocarboxylic acid metabolic process	BP	1.88850699	2.464921217	-0.538157218	6.037232379	81.46777569	1.4865E-17	3.65352E-16	PREDICTED: fatty acid synthase-like
RS006876	GO:0032787	monocarboxylic acid metabolic process	BP	-1.10740967	-4.875531905	-1.927422906	9.200285447	80.41642171	2.49869E-17	6.0606E-16	PREDICTED: acyl-CoA Delta(11) desaturase-like
RS011394	GO:0032787	monocarboxylic acid metabolic process	BP	-0.808232837	-2.973798175	-1.001114062	5.701841087	63.34834224	1.13133E-13	2.15086E-12	PREDICTED: hydroxyacyl-coenzyme A dehydrogenase, mitochondrial-like
RS013687	GO:0032787	monocarboxylic acid metabolic process	BP	1.493196769	-2.49034267	0.605103464	4.035957267	63.33591434	1.13828E-13	2.16089E-12	PREDICTED: acyl-CoA Delta(11) desaturase-like
RS007724	GO:0032787	monocarboxylic acid metabolic process	BP	-1.01941508	-2.888896578	0.282945189	5.96399049	58.43902276	1.26668E-12	2.22194E-11	PREDICTED: histidine ammonia-lyase
RS001358	GO:0032787	monocarboxylic acid metabolic process	BP	1.04922649	-6.683100048	-1.661386259	7.235051984	48.39535779	1.75442E-10	2.64163E-09	putative C-5 sterol desaturase
RS006871	GO:0032787	monocarboxylic acid metabolic process	BP	-0.242054109	1.84490496	-1.948230684	5.642008932	46.08729724	5.4342E-10	7.86711E-09	PREDICTED: acyl-CoA Delta(11) desaturase-like
RS015462	GO:0032787	monocarboxylic acid metabolic process	BP	-1.651648964	-0.319791888	1.03492915	7.1197948	43.87721617	1.60259E-09	2.23017E-08	PREDICTED: probable citrate synthase 2, mitochondrial
RS011853	GO:0032787	monocarboxylic acid metabolic process	BP	-0.198391061	-2.402376009	-1.315737207	5.883199768	42.14749002	3.73315E-09	5.06955E-08	PREDICTED: aldehyde dehydrogenase, dimeric NADP-preferring
RS013244	GO:0032787	monocarboxylic acid metabolic process	BP	-2.118886964	-5.134511847	-2.304467706	2.520197622	40.7543233	7.37271E-09	9.7359E-08	PREDICTED: acyl-CoA Delta(11) desaturase-like
RS005123	GO:0032787	monocarboxylic acid metabolic process	BP	2.214245853	4.384368384	1.931581004	5.434815601	37.74744679	3.19676E-08	3.88783E-07	PREDICTED: kynurenine formamidase-like
RS011602	GO:0032787	monocarboxylic acid metabolic process	BP	-0.603761397	-2.67449238	0.263382649	7.873774299	36.97611314	4.65524E-08	5.59321E-07	PREDICTED: acetyl-coenzyme A synthetase
RS002190	GO:0032787	monocarboxylic acid metabolic process	BP	-1.374803006	-2.081968712	0.437978506	4.918669629	35.63124542	8.96069E-08	1.03896E-06	PREDICTED: fatty acid 2-hydroxylase
RS010978	GO:0032787	monocarboxylic acid metabolic process	BP	-0.105950757	-2.718350483	-0.164657054	9.167446834	32.70854985	3.71036E-07	3.99729E-06	PREDICTED: fatty acid synthase
RS009864	GO:0032787	monocarboxylic acid metabolic process	BP	-0.959104172	-2.339035894	-1.594321797	5.998879226	30.22964854	1.23477E-06	1.25201E-05	PREDICTED: acyl-CoA synthetase family member 2, mitochondrial-like
RS007610	GO:0032787	monocarboxylic acid metabolic process	BP	-0.545113401	-1.570572207	-0.099561652	6.521035254	28.76411148	2.51014E-06	2.45842E-05	PREDICTED: short-chain specific acyl-CoA dehydrogenase, mitochondrial
RS005544	GO:0032787	monocarboxylic acid metabolic process	BP	0.405463151	1.50203441	-1.817605524	9.291358907	27.83750659	3.92874E-06	3.76786E-05	PREDICTED: beta-ureidopropionase
RS002535	GO:0032787	monocarboxylic acid metabolic process	BP	-0.730643229	-1.637072136	0.172680139	8.113462827	26.98303681	5.93576E-06	5.54863E-05	PREDICTED: acetyl-CoA carboxylase
RS001261	GO:0032787	monocarboxylic acid metabolic process	BP	-2.235645034	0.224656739	-1.086038105	2.162252071	26.93058301	6.08797E-06	5.68269E-05	PREDICTED: elongation of very long chain fatty acids protein 7
RS009917	GO:0032787	monocarboxylic acid metabolic process	BP	-2.368390697	-3.021829018	-2.11122478	5.72104937	26.62797216	7.04523E-06	6.51042E-05	PREDICTED: phosphoenolpyruvate carboxykinase [GTP]-like
RS002240	GO:0032787	monocarboxylic acid metabolic process	BP	-0.632574541	0.882911763	0.780104643	5.192425356	23.60200145	3.02453E-05	0.000256065	PREDICTED: probable pyruvate dehydrogenase E1 component subunit alpha, mitochondrial
RS003648	GO:0032787	monocarboxylic acid metabolic process	BP	1.268807283	1.776217158	0.41179627	5.528161964	23.03387344	3.97318E-05	0.000329264	PREDICTED: protein singed
RS007468	GO:0032787	monocarboxylic acid metabolic process	BP	1.554961358	-0.015902902	0.482470573	9.656522243	22.44534397	5.26932E-05	0.0004279	PREDICTED: retinal dehydrogenase 1
RS006877	GO:0032787	monocarboxylic acid metabolic process	BP	0.265818642	-1.05943473	-2.08149481	3.30161852	22.30893928	5.62543E-05	0.000455388	PREDICTED: acyl-CoA Delta(11) desaturase
RS010210	GO:0032787	monocarboxylic acid metabolic process	BP	-1.577649228	0.880954578	-0.51929089	3.453369802	21.20123124	9.56162E-05	0.000742418	PREDICTED: acyl-CoA Delta(11) desaturase-like
RS011319	GO:0032787	monocarboxylic acid metabolic process	BP	-1.721607065	-0.37953699	0.32859135	5.346304498	21.05802428	0.000102395	0.000789835	PREDICTED: elongation of very long chain fatty acids protein AAEL008004-like
RS013213	GO:0032787	monocarboxylic acid metabolic process	BP	-0.363489728	-0.566451634	0.868323084	6.648835729	20.32055339	0.000145658	0.001097362	PREDICTED: 4-hydroxybutyrate coenzyme A transferase
RS012880	GO:0032787	monocarboxylic acid metabolic process	BP	-0.807344323	-1.895186526	-0.086915908	8.413207767	20.19903013	0.00015436	0.001160887	PREDICTED: C-1-tetrahydrofolate synthase, cytoplasmic
RS013389	GO:0032787	monocarboxylic acid metabolic process	BP	-0.662472987	-1.005914654	0.744834151	7.265622757	19.41054869	0.000224837	0.001636653	PREDICTED: propionyl-CoA carboxylase alpha chain, mitochondrial
RS005437	GO:0032787	monocarboxylic acid metabolic process	BP	-1.890048432	-1.506694681	-1.067338185	4.309701597	19.07886591	0.000263323	0.001902872	PREDICTED: acylphosphatase-2
RS013246	GO:0032787	monocarboxylic acid metabolic process	BP	0.026801121	-2.623174489	-1.205156148	1.300160489	18.64961266	0.000323008	0.002299477	PREDICTED: acyl-CoA Delta(11) desaturase-like
RS015561	GO:0032787	monocarboxylic acid metabolic process	BP	0.869672605	0.201330391	-0.563462618	6.23335556	17.7167479	0.000503153	0.003452636	malate dehydrogenase
RS008160	GO:0032787	monocarboxylic acid metabolic process	BP	-2.41543084	1.144946968	-0.139456084	1.085418032	16.94702776	0.000724694	0.00484178	PREDICTED: UDP-glucuronosyltransferase 2C1-like
RS010737	GO:0032787	monocarboxylic acid metabolic process	BP	0.07086237	1.016941323	1.268374972	5.272102935	16.67839044	0.000822948	0.005444763	PREDICTED: elongation of very long chain fatty acids protein AAEL008004
[truncated: 1,425,202 more chars]
